# Effects of Alanyl-Glutamine Treatment on the Peritoneal Dialysis Effluent Proteome Reveal Pathomechanism-Associated Molecular Signatures*[Fn FN2]

**DOI:** 10.1074/mcp.RA117.000186

**Published:** 2017-12-04

**Authors:** Rebecca Herzog, Michael Boehm, Markus Unterwurzacher, Anja Wagner, Katja Parapatics, Peter Májek, André C. Mueller, Anton Lichtenauer, Keiryn L. Bennett, Seth L. Alper, Andreas Vychytil, Christoph Aufricht, Klaus Kratochwill

**Affiliations:** From the ‡Division of Pediatric Nephrology and Gastroenterology, Department of Pediatrics and Adolescent Medicine, Medical University of Vienna, Vienna, Austria;; §Christian Doppler Laboratory for Molecular Stress Research in Peritoneal Dialysis, Department of Pediatrics and Adolescent Medicine, Medical University of Vienna, Vienna, Austria;; ¶CeMM Research Center for Molecular Medicine of the Austrian Academy of Sciences, Vienna, Austria;; ‖Division of Nephrology, Beth Israel Deaconess Medical Center, Boston, Massachusetts;; **Department of Medicine, Harvard Medical School, Boston, Massachusetts;; ‡‡Department of Medicine III, Division of Nephrology and Dialysis, Medical University of Vienna, Vienna, Austria

## Abstract

Peritoneal dialysis (PD) is a modality of renal replacement therapy in which the high volumes of available PD effluent (PDE) represents a rich source of biomarkers for monitoring disease and therapy. Although this information could help guide the management of PD patients, little is known about the potential of PDE to define pathomechanism-associated molecular signatures in PD.

We therefore subjected PDE to a high-performance multiplex proteomic analysis after depletion of highly-abundant plasma proteins and enrichment of low-abundance proteins. A combination of label-free and isobaric labeling strategies was applied to PDE samples from PD patients (*n* = 20) treated in an open-label, randomized, two-period, cross-over clinical trial with standard PD fluid or with a novel PD fluid supplemented with alanyl-glutamine (AlaGln).

With this workflow we identified 2506 unique proteins in the PDE proteome, greatly increasing coverage beyond the 171 previously-reported proteins. The proteins identified range from high abundance plasma proteins to low abundance cellular proteins, and are linked to larger numbers of biological processes and pathways, some of which are novel for PDE. Interestingly, proteins linked to membrane remodeling and fibrosis are overrepresented in PDE compared with plasma, whereas the proteins underrepresented in PDE suggest decreases in host defense, immune-competence and response to stress. Treatment with AlaGln-supplemented PD fluid is associated with reduced activity of membrane injury-associated mechanisms and with restoration of biological processes involved in stress responses and host defense.

Our study represents the first application of the PDE proteome in a randomized controlled prospective clinical trial of PD. This novel proteomic workflow allowed detection of low abundance biomarkers to define pathomechanism-associated molecular signatures in PD and their alterations by a novel therapeutic intervention.

More than two million patients world-wide rely on dialysis as life-saving renal replacement therapy. Peritoneal dialysis (PD)^1^, as a home-based therapy equivalent in efficacy and safety to hemodialysis, offers an attractive alternative to hemodialysis with respect to quality of life and independence from center-based care (1, 2). PD introduces a hyper-osmolar, mostly glucose-based PD fluid into the peritoneal cavity via a permanent percutaneous catheter. Following an equilibration time, the peritoneal dialysis effluent (PDE) is drained from the peritoneum, thereby removing net solutes and water from the uremic patient. During PD treatment, however, the physicochemical properties of the fluid harm the peritoneal membrane. This leads to detachment and transdifferentiation of cells, sterile inflammation, increased risk of infections/peritonitis and eventual failure of ultrafiltration capacity ultimately resulting in clinical complications in most PD patients (3–5). Biomarkers are needed to guide the clinical course of PD patients, facilitating complication-free survival, identification of high-risk patients, stratification of patient groups for therapeutic interventions and evaluation of novel therapeutic approaches (6).

PDE contains small molecules, proteins and free-floating cells that can be sampled to provide noninvasive liquid biopsy specimens available in volumes up to 20 liters per day. Nevertheless, previous proteomic efforts to develop novel PDE biomarkers have been hindered by the PDE's plasma-like dynamic range of protein abundances, by the limited performance of separation and detection techniques applied to date, and by the design of the clinical trials from which the PDE samples were obtained. Thirteen published proteomic analyses of soluble PDE proteins have to date identified 171 unique proteins (7–19).

To minimize masking of low abundance candidate biomarker proteins by the background of high abundance proteins in PDE, we recently developed a depletion and enrichment strategy that was assessed in artificial PDE (20). Spiked-in aliquots of low abundance cellular proteins could be detected in PDE by two-dimensional difference gel electrophoresis (2D-DIGE) only by application of the combinatorial peptide ligand library (CPLL) technique. Because only few accepted PDE biomarkers are in routine clinical use, we propose that our CPLL method may facilitate development of clinically useful PDE biomarkers based on pathomechanism-associated molecular signatures (PAMS) originating from open -omics approaches (6).

Alanyl-glutamine (AlaGln) has been shown *in vitro* and *in vivo* to improve cellular stress responses and survival of multiple peritoneal cell types and thus to protect from peritoneal membrane deterioration (21–23). In experimental systems, our open -omic approach has revealed decreased cytoprotective stress responses during repeated or chronic exposure to PD fluid or in the presence of sterile inflammation (24, 25) that could be restored by AlaGln (26). Our recently reported first-in-man, cross-over, randomized controlled trial demonstrated that restoration of stress responses and improved cellular host-defenses in peritoneal cells by AlaGln supplementation of PD fluid can be translated into the clinical setting (27). That prospective clinical trial allowed collection of clinical PDE samples for delineation of cellular mechanisms relevant in PD and modified by AlaGln.

Here, we combined a CPLL-based depletion and enrichment approach with multiplex LC-MS analyses to explore the proteome of clinical PDE. Our study links proteins specifically enriched in the PDE proteome to PD pathomechanisms such as membrane fibrosis and decreased host defense. Further, we show that AlaGln supplementation of PD fluid interferes with these pathomechanism-associated signatures in PDE.

## EXPERIMENTAL PROCEDURES

Chemicals were purchased from Sigma-Aldrich (St. Louis, MO) if not specified otherwise.

### 

#### 

##### Patients and Samples

The PDE samples were obtained during a prospective randomized, open-label, two-period, cross-over phase I/II study conducted at the Department of Nephrology at the Medical University of Vienna Austria. The study protocol was approved by the local ethics committee of the Medical University of Vienna (EK 867/2010, EK 1167/2013, EK 2035/2015), registered in www.clinicaltrials.gov (NCT01353638), and performed in accord with the Declaration of Helsinki. The study design, clinical methods, eligibility criteria, randomization, patient characteristics and adverse events have been previously described (27). All patients provided written informed consent before trial participation.

PDE samples were obtained from 20 stable PD patients treated per protocol (from a total of 21 enrolled, ≥19 years old, m/f: 13/7, free from peritonitis for at least 2 months). The protocol included for each PD patient two PDE samples designated for protein expression analysis collected from 4 h peritoneal equilibration tests (PET), one with commercially available PD fluid (Dianeal, 3.86% glucose, Baxter, Deerfield, IL) and another with the same PD fluid supplemented with AlaGln (8 mm, Dipeptiven, Fresenius, Bad Homburg, Germany) in a randomized order. PETs were separated by a wash-out period (28–35 days).

##### Depletion of High Abundance Plasma Proteins (and Enrichment of Low Abundance Proteins) in PDE (CPLL)

**Fig. 1.**
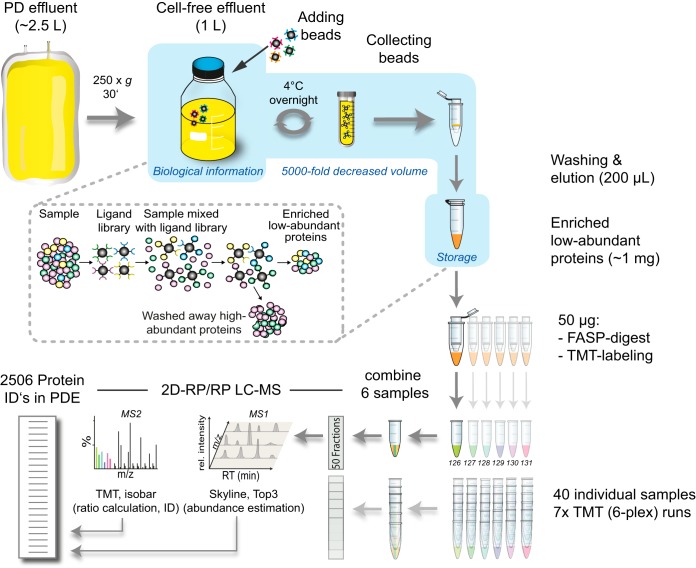
**Schematic workflow representation.** Beads with a combinatorial peptide ligand library (CPLL) are used to deplete high abundance plasma proteins and enrich low abundance proteins in cell-free PD effluent (PDE) samples. Protein samples are digested (filter-aided sample preparation, FASP), labeled with isobaric tandem mass tags (TMT) and 6 differentially labeled samples (TMT 6-plex) are mixed and analyzed by 2 dimensional reverse phase liquid chromatography mass spectrometry (2D RP/RP LC-MS) followed by computational abundance estimation (Skyline and Top3) and ratio calculation (Isobar).

The complete volume of PDE after the 4 h dwell of the PET was collected from each of the 40 PETs of the 20 patients receiving both treatments. The PDE was processed immediately following the end of the dwell and aliquoted samples were stored at −80 °C until further analysis. The protocol was performed as previously reported (20) with minor adaptations for clinical samples. CPLL equalizer beads (ProteoMiner, BioRad, Hercules, CA) were prepared according to the manufacturer's protocol. Following centrifugation (250 × *g*, 30 min, 600 ml aliquots), 1 L of cell-free PDE was mixed with 250 μl CPLL bead solution (50 μl bead bed volume) and incubated on a roller mixer (overnight, 4 °C). The beads were allowed to sediment for 30 min, 900 ml effluent was carefully aspirated and the beads were recovered and centrifuged (100 × *g*, 10 min). The supernatant was removed and beads recovered in 1 ml remaining effluent were transferred to Mini Bio-Spin chromatography columns (BioRad). Following four washing steps with washing buffer (5 min, 150 μl, 150 mm NaCl, 10 mm NaH_2_PO_4_, pH 7.4) and once with 200 μl deionized water, proteins were eluted by four sequential elutions (15 min, 50 μl, 8 m urea, 2% CHAPS in 5% acetic acid) yielding 200 μl protein solution (Fig. 1). Protein samples were precipitated (100% acetone, overnight, −20 °C), washed with ethanol and resuspended in 150 μl buffer (30 mm Tris, pH 8.5, 7 m urea, 2 m thiourea, 4% 3-[(3-cholamidopropyl) dimethylammonio]-1-propanesulfonate (CHAPS), 1 mm EDTA, 1 tablet of Complete Protease Inhibitor (Roche, Basel; Switzerland) and 1 tablet of phosphatase inhibitor (PhosSTOP, Roche) per 100 ml). Protein concentration was determined using the 2D Quant kit (GE Healthcare, Uppsala, Sweden) per manufacturer's instructions.

##### Filter-Aided Sample Preparation (FASP)

FASP was performed using a 30 kDa molecular weight cut-off filter (VIVACON 500; Sartorius Stedim Biotech, Goettingen, Germany) as described previously (28). In brief, 50 μg of each sample was mixed with 180 μl UA buffer (8 m urea in 100 mm Tris-HCl, pH 8.5) in the filter unit and centrifuged (14,000 × *g*, 15 min, 20 °C). Following a second washing step with UA (200 μl), the proteins were alkylated (100 μl 50 mm iodoacetamide, 30 min, RT). Following 3 UA washes (100 μl) and 3 more washes with TEAB buffer (50 mm, 100 μl), proteins were digested with trypsin (overnight, 37 °C). Peptides were recovered using 40 μl 50 mm TEAB buffer, followed by 50 μl 0.5 m NaCl. Acidified tryptic peptides were concentrated and desalted using C18 spin columns (The Nest Group, Southborough, MA, USA).

##### Tandem Mass Tag (TMT) Derivatization

TMT labeling was performed according to the instructions provided by the manufacturer for the 6-plex isobaric labeling reagent (ThermoFisher Scientific, Waltham, MA, USA). Pooled samples were concentrated and desalted with C18 microspin columns (5–60 μg, The Nest Group). Eluates were lyophilized in a vacuum concentrator and reconstituted in 20 mm ammonia formate buffer, pH 10 before fractionation at basic pH.

##### 2D-RP/RP Liquid Chromatography Mass Spectrometry

Two-dimensional liquid chromatography was performed by reverse-phase chromatography at high and low pH. In the first dimension, peptides were separated on a Gemini-NX C18 (150 × 2 mm, 3 μm, 110 Å, Phenomenex, Torrance, CA) in 20 mm ammonia formate buffer, pH 10, and eluted over 45 min by a 5–70% acetonitrile gradient at 100 μl/min using an Agilent 1200 HPLC system (Agilent Biotechnologies, Palo Alto, CA). Seventy-two time-based fractions were collected and pooled into 50 HPLC vials based on the UV-trace at 214 nm. Samples were acidified by the addition of 5 μl of 5% formic acid, organic solvent was removed in a vacuum concentrator, and samples were reconstituted in 5% formic acid. Every fraction was analyzed with a single injection. Mass spectrometry was performed on a hybrid linear trap quadrupole (LTQ) Orbitrap Velos mass spectrometer (ThermoFisher Scientific) using the Xcalibur version 2.1.0 coupled to an Agilent 1200 HPLC nanoflow system (dual pump system with one trap-column and one analytical column) via a nano-electrospray ion source using a liquid junction (Proxeon, Odense, Denmark). Technical details of the nanoHPLC and MS conditions used in this study are described in detail elsewhere (29). Analyses were performed in a data-dependent acquisition mode using a top-10 higher-energy collision-induced dissociation (HCD) method for peptide identification plus relative quantitation of TMT reporter ions. Dynamic exclusion for selected ions was 60 s. A single lock mass at *m*/*z* 445.120024 was employed (30). The maximal ion accumulation time allowed for MS mode in the Orbitrap was 500 ms, and the accumulation time for HCD was 200 ms. Automatic gain control (AGC) was used to prevent overfilling of the ion traps with AGC targets and set to 10E6 and 10E5 ions for MS^1^ and MS^2^, respectively. A resolution of 30,000 and 7500 (at *m*/*z* 400) was chosen for MS^1^ and MS^2^, respectively. The threshold for triggering MS^2^ scans was set to 5000 counts.

##### LC-MS Data Analysis

The acquired raw MS data files were processed with msconvert (ProteoWizard Library v2.1.2708) and converted into Mascot generic format (mgf) files. The resultant peak lists were searched against the human SwissProt database version v2016.11 (37,398 sequences, including isoforms as obtained from varsplic.pl) with the search engines Mascot (v2.3.02, MatrixScience, London, U.K.) and Phenyx (v2.5.14, GeneBio, Geneva, Switzerland) (31). Submission to the search engines was done via a Perl script that performs an initial search with relatively broad mass tolerances (Mascot only) on both the precursor and fragment ions (±10 ppm and ±0.6 Da, respectively). High-confidence peptide identifications were used to recalibrate all precursor and fragment ion masses before a second search with narrower mass tolerances (±4 ppm and ±0.025 Da). One missed tryptic cleavage site was allowed. Carbamidomethyl cysteine, N-terminal, and lysine-modified TMT 6-plex were set as fixed modifications, and oxidized methionine was set as a variable modification. To validate the proteins, Mascot and Phenyx output files were processed by internally developed parsers. Proteins with ≥2 unique peptides above a score T1 or with a single peptide above a score T2 were selected as unambiguous identifications. Additional peptides for these validated proteins with score >T3 were also accepted. T1, T2, and T3 values for Mascot were 16, 40, and 10, and for Phenyx were 5.5, 9.5, 3.5, respectively (*p* value <10^−3^). Following the selection criteria, proteins were grouped based on shared peptides, and only the group reporters are considered in the final output of identified proteins. Spectral conflicts between Mascot and Phenyx peptide identifications were discarded. The whole procedure was repeated against a reversed database to assess the protein group false discovery rate (FDR). Peptide and protein group identifications were <0.1 and <1% FDR, respectively. Cytokeratins are markers of mesothelial cells lining the peritoneal wall, thus identified keratins were excluded from the results only if containing the terms “hair” or “cuticular” (*n* = 18).

Because of the lack of differentiation of protein isoforms in gene ontology and pathway analysis, protein isoforms were merged at the level of UniProt entry names.

##### Protein Abundance Estimation

Proteins abundance was quantified by the Top3 method (32) using Skyline (version 3.1) (33) for peptide area calculation. For some peptides with multiple PSMs, Skyline integration incorrectly considers only a local subpeak around a single PSM, possibly underestimating true peptide abundance. Therefore, for each peptide with at least 2 PSMs, on top of the peptide area calculated by Skyline *A_S_* we additionally calculate *A_0f_* as area under the chromatogram curve from time *T_0_* to time *T_f_*, where *T_0_* and *T_f_* are retention times of the earliest and the latest PSM for a given peptide. We consider the larger of *A_S_* and *A_0f_* as an estimate of peptide area for the Top3 algorithm.

##### TMT Semiquantitative Analysis

The R software package Isobar (34) was used to calculate protein ratios. Isobar is publically available under the LGPL license from the CeMM Web site (http://bioinformatics.cemm.oeaw.ac.at) and through Bioconductor (http://bioconductor.org/packages/release/bioc/html/isobar.html). Isobar implements a signal intensity noise model to account for heteroscedasticity in TMT reporter ion ratios, and calculates *p* values for each quantitated protein, estimating technical and biological variability. The statistical framework of Isobar was described previously (34). TMT reporter ions were extracted at the expected *m*/*z* ± 0.005 *m*/*z*, and intensities were corrected for isotopic impurities, as described by the manufacturer. All channels were normalized to the equal sum of the intensities. Protein identifications were grouped based on peptide matches from both samples. Protein ratios were calculated based on unique peptides. Proteins were denoted as significantly regulated when the distribution of protein (effect) log ratios from individual patients was significantly different from 1 (*p* value <0.05, one-sided *t* test, numbers of observations from 3 to 20 ratios were considered).

##### Experimental Design and Statistical Rationale

The total number of samples analyzed was 40 that were obtained from 20 patients. For the analysis on general PDE protein abundance, each of the 7 TMT runs can be regarded as a biological replicate of 6 pooled samples from three patients. For the paired analysis of the effect of AlaGln in each patient, the same patient treated without the additive in the cross-over design trial acted as the control. Following an observation period, patients were randomized immediately before start of treatment to one of the two sequences. Random allocation of treatments with AlaGln or without AlaGln in a ratio 1:1 was performed using RandomizerR (www.randomizer.at) and stratified by sex, age (< or ≥ 60 years), time on PD (< or ≥ 1 year) and peritonitis history (yes or no) (27).

Sample size calculation for the clinical trial was based on the primary outcome parameter “total heat shock protein (HSP) expression” in cells from PDE, expecting at least 30% percent increase after treatment with AlaGln compared with a control group, and a standard deviation of within-subject period differences of less than 50 percentage points, estimated from previous results in the *ex vivo* setting and from the literature (27).

Cut-offs were chosen following an extremes-of-phenotype approach, so that the number of candidate molecules was sufficient for subsequent bioinformatic steps. For this reason, and because of the relatively high interindividual differences and small effect size (especially for high abundance proteins), protein candidates were chosen without correction for multiple testing but further discussed only on fulfillment of the second criterion of Bonferroni-correction and pathway enrichment. Individual protein candidates not fulfilling at least two criteria were regarded as false-positives and not further discussed.

Graphical representations of the results were computed with R (http://www.r-project.org/), Excel (Microsoft, Office 2016) and Venn Diagram Plotter (version 1.5.5228.29250). Cellular component and biological process mapping and statistical overrepresentation tests of candidate proteins were performed using the Panther Classification System and implemented Gene Ontology (GO) database (version 11.1). All results were Bonferroni-corrected for multiple testing (*p* < 0.05). ReviGO (http://revigo.irb.hr/) (35) and Cytoscape (version 3.5.1) (36) were used to summarize and visualize GO enrichment results. Ingenuity Pathway Analysis (IPA 7.0, Qiagen, http//www.ingenuity.com) was used to identify pathways and interaction networks significantly affected by differentially expressed genes, calculating a *p* value for each functional pathway using a one-tailed Fisher exact test. Pathways with *p* values <0.05 were considered significantly affected. For each network, IPA calculates a score derived from the *p* value of the one-tailed Fisher exact test (score = -log(*p* value)) and indicates the likelihood of focus genes appearing together in the network because of random chance.

##### Data Availability

All mass spectrometry data, corresponding proteins, protein coverage and raw data files have been deposited into the ProteomeXchange Consortium (http://proteomecentral.proteomexchange.org) via the PRIDE partner repository (37) with the data set identifier PXD006863.

##### Literature Search on PDE and Consensus Plasma Protein Concentrations

A literature search in NCBI Pubmed utilizing the query string (proteomics AND peritoneal dialysis) OR (proteome AND peritoneal dialysis) OR (proteomics AND peritoneal fluid) OR (proteome AND peritoneal fluid) OR (proteomics AND peritoneal effluent) OR (proteome AND peritoneal effluent) on Oct 26, 2017 resulted in 83 hits. These were restricted to clinical peritoneal dialysis effluent from humans, to return only 15 studies (7–19, 38, 39) of which two were excluded because they focused solely on the exosomal fraction (38, 39) and one did not provide any protein accessions (19), resulting in 12 studies and 171 unique proteins for analysis (supplemental Table S1) (7–18). Manual query of protein names presented by Zavvos *et al.* (19) yielded 19 protein names not contained in the 171, of which 11 were unreviewed and 8 were reviewed entries in UniProt. Of those 8, not included in our analysis, 3 were immunoglobulin kappa variable region proteins and the remaining 5 were CD44 antigen, Dermatopontin, Nuclear fragile X mental retardation-interacting protein 2, Zinc finger protein 527 and Kelch-like protein 3.

The human plasma proteome database (HPPDB) (40) was downloaded on May 15, 2017 from www.plasmaproteomedatabase.org. Mean concentrations for individual proteins computed from all reported studies were used as an approximation of a consensus plasma protein proteome.

## RESULTS

Forty PDEs obtained from a prospective randomized controlled cross-over trial in 20 PD patients treated with standard or AlaGln-supplemented PD fluids were depleted and enriched using the CPLL approach previously developed in artificial PDE (20). Clinical results of a trial of this novel therapy were recently reported (27).

Pre-processing by the CPLL depletion and enrichment strategy was combined with TMT-labeling and FASP-LC-MS (Fig. 1). The 40 samples were arranged in seven TMT 6-plex runs (see supplemental Table S2 for experimental design), ensuring that both the treatment and control sample of each of three patients were grouped in one individual run. The last of the seven analyses contained only two patients. The two remaining TMT labels included two replicates of samples that were included in two previous analyses as functioned as an internal quality control.

**Fig. 2.**
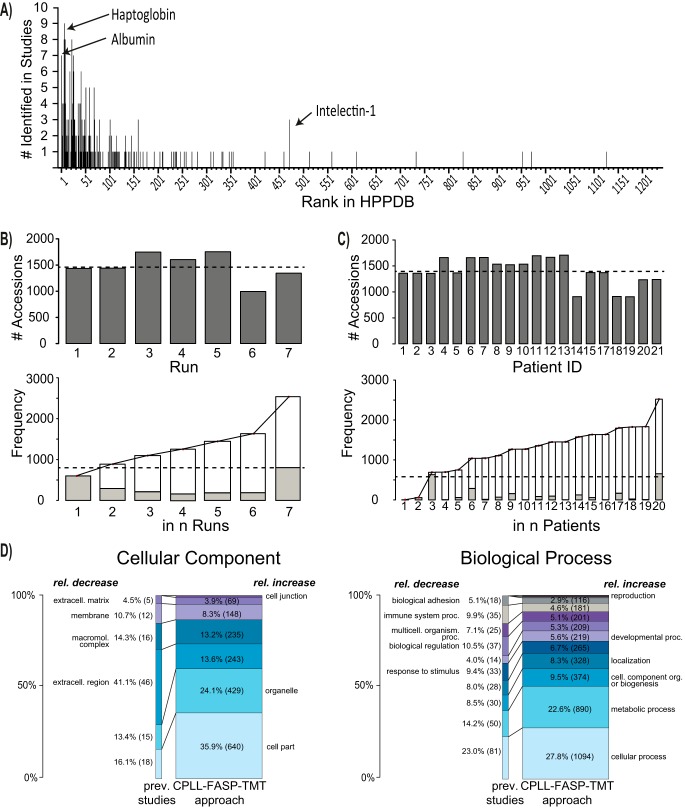
**PD effluent protein identification.** *A*, Frequency of protein identification in existing PD effluent (PDE) studies (12 studies with 171 individual proteins, supplemental Table S1) according to the respective abundance rank in the human plasma proteome database (HPPDB). Examples are labeled with arrows and protein name. *B*, Numbers of protein identifications per TMT 6-plex run (upper panel) and cumulative frequency of identifications (stacked bars) ranked by number of occurrences in runs (lower panel). *C*, Numbers of protein identifications per patient (upper panel) and cumulative frequency of identifications (stacked bars) ranked by number of occurrences in patients (lower panel). Dashed line represents the average number of identifications. D, Distribution of gene ontology (GO) cellular component (left) and biological process (right) associations to protein identifications in previously reported PDE studies (left) and in the presented approach (CPLL: combinatorial peptide ligand library, FASP: filter aided sample preparation, TMT: isobaric tandem mass tags). PDE proteins identified in seven TMT 6-plex runs (right). Only unambiguous protein identifications from the LC-MS analysis were considered. Areas of the bars correspond to the total numbers of identifications (171 *versus* 2506). Total numbers per cellular component or biological process are in parentheses. (The full list including GO identifiers is presented in supplemental Table S5.)

A literature search identified 12 studies investigating the soluble proteome of human clinical PDE. Per-study and cumulative summation of PDE proteins previously identified in the literature (*n* = 171) plus mapping of these to the HPPDB abundance ranking of 1237 proteins with reported plasma concentrations are presented in supplemental Table S1. Previously identified soluble proteins in PDE are almost entirely clustered at the high end of the plasma protein abundance range (Fig. 2*A* and supplemental Table S1). Using the CPLL-FASP-TMT (CFT) LC-MS approach (supplemental Table S3 and supplemental Table S4) we identified 2506 PDE proteins, representing a 15-fold increase in coverage compared with all previous reports.

Each TMT 6-plex experiment yielded on average 11,812 peptides, ranging from 7874 to 14,389. 23,956 unique peptides were identified in total, associated with 1422 unambiguously identified proteins (ranging from 958 to 1688). In addition, on average 1448 protein groups (ranging from 977 to 1723) were identified. A comparison of identified proteins in the 7 TMT 6-plex experiments is shown in Fig. 2*B*.

From each of the 20 sample pairs representing an individual patient in the cross-over trial, on average, 11,581 peptides (ranging from 7574 to 14,234 with a total of 23,520 unique peptides) were identified. These were associated to 1289 unambiguously identified proteins (ranging from 820 to 1555) and 1322 protein groups on average (ranging from 846 to 1600). A comparison of the identified proteins is shown in Fig. 2*C*. Supplemental Table S4 provides details on numbers of identified peptides and proteins.

Using the CFT-LC-MS approach, the distribution of identified proteins was markedly shifted toward proteins of cellular origin. GO annotations of identified proteins in our data set compared with previous literature showed an increased proportion of the cellular component categories “cell part” and “organelle” and biological process categories “metabolic process” and “cellular process” (Fig. 2*D* and supplemental Table S5).

Based on MS1 information from the identified peptides, estimated abundances of PDE proteins were calculated using Skyline and the Top3 method (see supplemental Table S3). Even after depletion and enrichment with the CPLL technique, plasma proteins remained the most abundant proteins in PDE. Indeed, the CPLL method should only compress the dynamic range of the protein sample (equalizing effect) without significantly altering the protein abundance rank, in contrast to (*e.g.* multiple affinity) removal techniques.

**Fig. 3.**
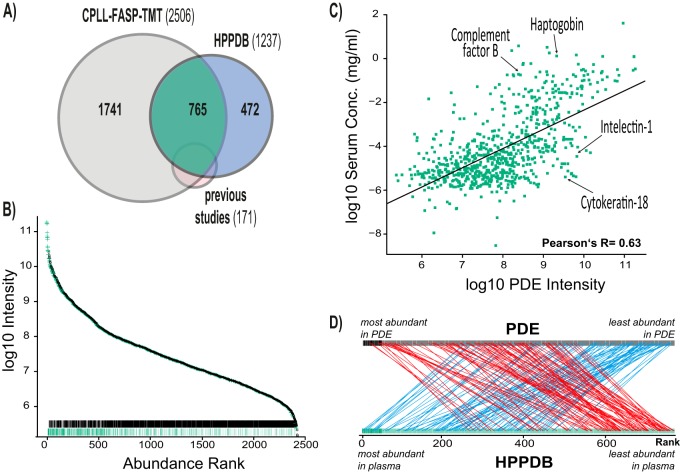
**Comparison of protein composition in PD effluent and plasma.** *A*, Venn diagram illustrating the overlap among identified proteins in the presented approach (CPLL: combinatorial peptide ligand library, FASP: filter aided sample preparation, TMT: isobaric tandem mass tags) and in the human plasma proteome database (HPPDB) and previous studies. Proteins identified in previous studies but not in the CPLL-FASP-TMT approach (*n* = 30) were all reported in only one study. *B*, Illustration of abundance (mean Top3 intensity) of typical plasma proteins and potential cellular proteins and low abundance proteins in PD effluent (PDE). Rag plots at the bottom represent the distributions. Black: typical nominally cellular and low abundance proteins; green: plasma proteins. *C*, Correlation of PDE concentrations (global Top3 intensities) and estimated consensus plasma concentration (Person's *r* = 0.63). Plasma concentrations are mean concentrations from the HPPDB. Individual examples of proteins are indicated. *D*, Illustration of top-differentially ranked proteins between PDE and plasma. Red: 100 most extreme proteins with higher abundance in PDE *versus* plasma, blue: 100 most extreme proteins with lower abundance in PDE *versus* plasma.

Further systematic investigation required definition of a set of “typical plasma proteins.” Proteins identified in PDE were therefore mapped to 1237 proteins with reported plasma concentrations from the HPPDB. Computed mean plasma concentrations are summarized in supplemental Table S6 (40). The 765 proteins present in both the HPPDB and our data set (Fig. 3*A*) were termed “typical plasma proteins”. In our experiments, a set of 472 plasma proteins with reported concentrations in the HPPDB were not identified in PDE.

The ranking of PDE proteins by observed abundance and discrimination by protein type confirms that typical plasma proteins constitute the top region of the distribution, and that these proteins are significantly more abundant than nonplasma proteins even in CPLL-treated PDE (Fig. 3B), but to a much lesser degree than in crude PDE or plasma (20).

**Fig. 4.**
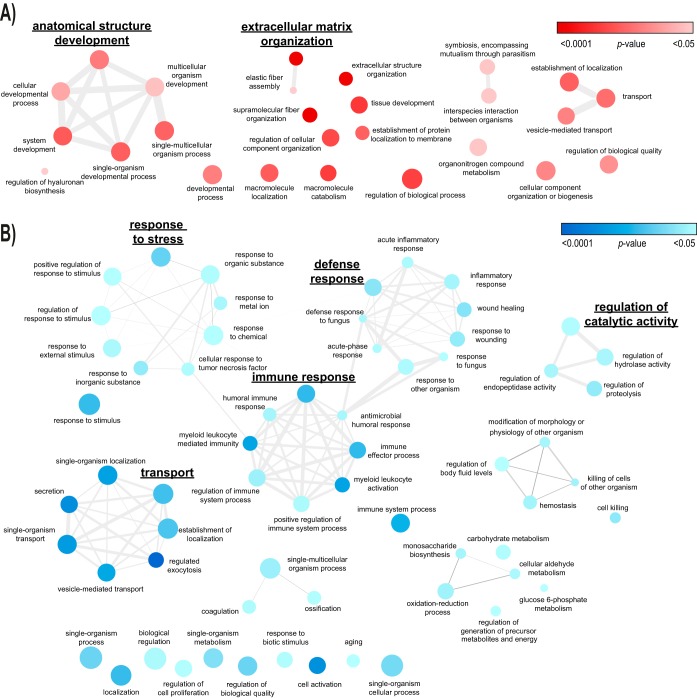
**Biological process enrichment maps of top-differentially ranked proteins in PD effluent *versus* plasma.** *A*, clusters of biological processes enriched in 100 most extreme proteins with higher abundance in PD effluent (PDE) than in the human plasma protein database (HPPDB). *B*, clusters of biological processes enriched in 100 most extreme proteins with lower abundance in PDE than in the HPPDB. node color intensity: *p* value overrepresentation test (Panther database; Bonferroni-corrected *p* < 0.05), node size: proportional to prevalence of the term in the GO database (homo sapiens - all genes, ReviGO), edge width: degree of similarity (ReviGO) (Full list see supplemental Table S7).

The observed protein abundances in PDE correlate with reference concentrations in the HPPDB (Pearson's *r* = 0.63, Fig. 3C). Certain proteins, however, clearly deviate from this general trend. We were particularly interested in the biological roles of proteins that most differed in abundance ranking in PDE as compared with normal plasma (Fig. 3*D*). To investigate their biological roles, two groups of 100 proteins were selected for enrichment analysis of biological processes: the 100 least abundant proteins in PDE relative to plasma concentrations (lower ranked, based on their relative ranks), and the 100 most abundant proteins in PDE relative to plasma concentrations (higher ranked). Using the Panther database, among the higher ranked proteins we identified 30 significantly enriched processes with 13 specific subclasses, including “supramolecular fiber organization” and “tissue development” (Fig. 4*A*). Among the lower ranked proteins, we identified 86 significantly enriched processes with 21 specific subclasses including “neutrophil degranulation” (Table I, Fig. 4*B* and supplemental Table S7). Varying the input number of proteins as a means of robustness analysis showed that the general motifs of IPA canonical pathways are not sensitive to this input number within a reasonable range (not shown).

**Table I:** PDE vs HPPDB top 100 higher and Top 100 lower ranked significantly enriched specific biological processes

Enriched biological processes from proteins in PDE (vs. HPPDB)^a^	Homo sapiens (20972)	Observed	Expected	Fold enrichment	*p-Value*^b^
***Top 100 higher ranked***					
Supramolecular fiber organization (GO:0097435)	374	12	1.78	6.73	2.16E-03
Tissue development (GO:0009888)	1643	24	7.83	3.06	5.27E-03
Macromolecule catabolic process (GO:0009057)	962	18	4.59	3.92	5.63E-03
Regulation of cellular component organization (GO:0051128)	2321	29	11.07	2.62	6.78E-03
System development (GO:0048731)	4156	41	19.82	2.07	8.56E-03
Protein targeting to membrane (GO:0006612)	162	8	0.77	10.36	1.07E-02
Vesicle-mediated transport (GO:0016192)	1748	24	8.33	2.88	1.58E-02
Regulation of biological quality (GO:0065008)	3487	36	16.63	2.17	1.99E-02
Cellular developmental process (GO:0048869)	3534	36	16.85	2.14	2.74E-02
Symbiosis, encompassing mutualism through parasitism (GO:0044403)	789	15	3.76	3.99	4.44E-02
Organonitrogen compound metabolic process (GO:1901564)	5458	47	26.03	1.81	4.44E-02
Elastic fiber assembly (GO:0048251)	7	3	0.03	89.88	4.98E-02
Regulation of hyaluronan biosynthetic process (GO:1900125)	7	3	0.03	89.88	4.98E-02
***Top 100 lower ranked***					
Neutrophil degranulation (GO:0043312)	483	24	2.3	10.42	6.16E-14
Platelet degranulation (GO:0002576)	127	15	0.61	24.77	7.16E-13
Oxidation-reduction process (GO:0055114)	951	19	4.53	4.19	9.64E-04
Killing of cells of other organism (GO:0031640)	48	6	0.23	26.21	1.21E-03
Negative regulation of endopeptidase activity (GO:0010951)	239	10	1.14	8.77	2.14E-03
Monosaccharide biosynthetic process (GO:0046364)	54	6	0.26	23.3	2.40E-03
Antimicrobial humoral response (GO:0019730)	95	7	0.45	15.45	3.68E-03
Response to metal ion (GO:0010038)	321	11	1.53	7.19	3.72E-03
Defence response to fungus (GO:0050832)	33	5	0.16	31.78	5.45E-03
Cellular aldehyde metabolic process (GO:0006081)	65	6	0.31	19.36	6.99E-03
Positive regulation of immune system process (GO:0002684)	1005	18	4.79	3.76	1.05E-02
Blood coagulation (GO:0007596)	294	10	1.4	7.13	1.37E-02
Acute-phase response (GO:0006953)	40	5	0.19	26.21	1.39E-02
Cellular response to tumor necrosis factor (GO:0071356)	235	9	1.12	8.03	1.80E-02
Regulation of cell proliferation (GO:0042127)	1558	22	7.43	2.96	2.98E-02
Ossification (GO:0001503)	251	9	1.2	7.52	3.05E-02
Positive regulation of response to stimulus (GO:0048584)	2088	26	9.96	2.61	3.21E-02
Regulation of generation of precursor metabolites and energy (GO:0043467)	89	6	0.42	14.14	4.20E-02
Aging (GO:0007568)	262	9	1.25	7.2	4.30E-02
Glucose 6-phosphate metabolic process (GO:0051156)	23	4	0.11	36.47	4.43E-02
Organonitrogen compound metabolic process (GO:1901564)	5458	47	26.03	1.81	4.44E-02

^a^most specific significantly enriched biological processes from Gene Ontology (full hierarchical list is given in supplemental Table S7).

^b^Bonferroni corrected for multiple testing.

The paired character of the clinical cross-over study design and the ability to directly compare channels allowed study of the effect of AlaGln supplementation of PD fluid on PDE protein composition. The isobar algorithm was used to generate protein ratios for individual patients (supplemental Table S8 and supplemental Table S9). Numbers of isobar ratios per patient and run were marginally lower than Top3 quantitations because of the additional restriction of sufficient reporter ion signals from both samples of each patient.

**Fig. 5.**
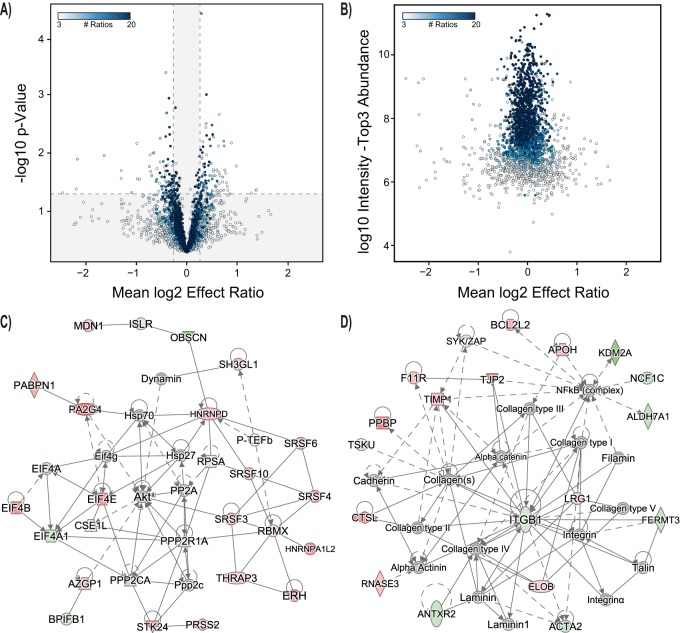
**Effect of Alanyl-glutamine on PD effluent proteins.** *A*, Volcano plot for effect ratios and *p* value for all proteins identified in PD effluent (PDE) in both samples of an individual patient for which at least 3 ratios were available. Outer segments (white) represent statistical cut-offs for process and pathway analysis (FC>1.2; *p* < 0.05) (*n* = 164). Color gradient visualizes the number of available effect ratios (= number of patients for whom identifications were available from both treatments). One data point of a significantly altered protein (LRRC15) with a log expression ratio of −3.6 was removed from the graph for better scaling. *B*, Abundance *versus* effect ratios plot for effect ratios, and *p* value for all proteins identified in PDE in both samples of an individual patient for which at least 3 ratios were available. Color gradient visualizes the number of available effect ratios. *C*, *D*, Interaction networks generated from differentially expressed genes. Each node represents a gene, and edges represent interactions among genes. The node color indicates up-regulation (red) or down-regulation (green) in the presence of added AlaGln.

We then assessed the distribution of ratios for a given protein to indicate significant up- or down-regulation. The differential abundance analysis was performed for all proteins for which a protein ratio could be obtained in at least 3 patients from the TMT data. One hundred sixty-four proteins exhibited significant differential abundance (85 increased, 79 decreased, Fig. 5*A*). Overrepresentation analysis of biological processes for this differential abundance group yielded 60 processes associated with 16 specific subclasses mainly linked to immune regulation (*i.e.* “neutrophil degranulation,” “viral processes,” “antigen processing and presentation of exogenous peptide antigen”) and RNA regulation (*i.e.* “mRNA metabolic process,” “RNA splicing,” “regulation of mRNA splicing, via spliceosome”) (Table II). The propensity of proteins for significant differential abundance was associated with the number of observations and the sample abundance. Thus, high abundance proteins were more often detected in all patients, but also showed less difference on AlaGln treatment (Fig. 5*B*). We also performed the analysis for stably abundant proteins (putative high abundance proteins), for which a protein ratio could be obtained in at least 17 of 20 patients from the TMT data. As expected among those high abundance proteins, fewer (*n* = 50) were found with significantly differential abundance (21 increased, 29 decreased, supplemental Fig. S1). This group showed enrichment in only 15 processes (with 3 specific subclasses) (“platelet degranulation,” “plasma lipoprotein particle organization,” and “lipid transport”) (supplemental Table S10).

**Table II:** Specific biological processes significantly enriched in PD fluid with added AlaGln vs. control

Enriched biological processes PD fluid *vs.* PD fluid with AlaGln^a^	H.sapiens (20972)	Observed	Expected	Fold enrichment	*p* value^b^
**neutrophil degranulation (GO:0043312)**	483	19	3.64	5.22	4.82E-05
neutrophil mediated immunity (GO:0002446)	497	20	3.74	5.34	1.27E-05
myeloid leukocyte mediated immunity (GO:0002444)	518	20	3.9	5.12	2.53E-05
leukocyte mediated immunity (GO:0002443)	725	20	5.46	3.66	5.63E-03
leukocyte degranulation (GO:0043299)	505	19	3.8	4.99	9.76E-05
regulated exocytosis (GO:0045055)	686	26	5.17	5.03	1.25E-07
exocytosis (GO:0006887)	774	27	5.83	4.63	3.09E-07
secretion by cell (GO:0032940)	963	27	7.26	3.72	3.47E-05
single-organism cellular process (GO:0044763)	9841	105	74.14	1.42	4.95E-03
single-organism process (GO:0044699)	12686	123	95.57	1.29	2.33E-02
secretion (GO:0046903)	1068	28	8.05	3.48	7.30E-05
single-organism transport (GO:0044765)	2521	43	18.99	2.26	1.51E-03
single-organism localization (GO:1902578)	2646	45	19.93	2.26	7.61E-04
localization (GO:0051179)	5375	75	40.49	1.85	2.45E-05
transport (GO:0006810)	4368	70	32.91	2.13	2.50E-07
establishment of localization (GO:0051234)	4499	71	33.89	2.09	3.36E-07
vesicle-mediated transport (GO:0016192)	1748	44	13.17	3.34	4.90E-09
neutrophil activation involved in immune response (GO:0002283)	484	19	3.65	5.21	4.98E-05
myeloid cell activation involved in immune response (GO:0002275)	514	19	3.87	4.91	1.29E-04
leukocyte activation involved in immune response (GO:0002366)	606	19	4.57	4.16	1.64E-03
cell activation (GO:0001775)	1026	23	7.73	2.98	2.62E-02
cell activation involved in immune response (GO:0002263)	610	19	4.6	4.13	1.81E-03
myeloid leukocyte activation (GO:0002274)	565	19	4.26	4.46	5.62E-04
neutrophil activation (GO:0042119)	490	19	3.69	5.15	6.06E-05
granulocyte activation (GO:0036230)	494	19	3.72	5.11	6.89E-05
**macromolecule localization (GO:0033036)**	2214	41	16.68	2.46	3.47E-04
**single-organism metabolic process (GO:0044710)**	3572	54	26.91	2.01	1.22E-03
**mRNA metabolic process (GO:0016071)**	663	20	4.99	4	1.40E-03
**viral process (GO:0016032)**	610	19	4.6	4.13	1.81E-03
symbiosis, encompassing mutualism through parasitism (GO:0044403)	789	24	5.94	4.04	6.09E-05
interspecies interaction between organisms (GO:0044419)	791	24	5.96	4.03	6.39E-05
multi-organism process (GO:0051704)	2398	43	18.07	2.38	3.66E-04
**RNA splicing (GO:0008380)**	393	15	2.96	5.07	3.10E-03
RNA processing (GO:0006396)	870	21	6.55	3.2	2.38E-02
**antigen processing and presentation of exogenous peptide antigen (GO:0002478)**	169	10	1.27	7.85	6.63E-03
antigen processing and presentation of exogenous antigen (GO:0019884)	176	10	1.33	7.54	9.52E-03
antigen processing and presentation (GO:0019882)	218	11	1.64	6.7	8.62E-03
antigen processing and presentation of peptide antigen (GO:0048002)	179	10	1.35	7.42	1.11E-02
**organic substance transport (GO:0071702)**	2048	36	15.43	2.33	1.01E-02
**regulation of mRNA splicing, via spliceosome (GO:0048024)**	72	7	0.54	12.9	1.29E-02
regulation of mRNA processing (GO:0050684)	104	8	0.78	10.21	1.29E-02
regulation of mRNA metabolic process (GO:1903311)	135	9	1.02	8.85	9.13E-03
**protein-lipid complex subunit organization (GO:0071825)**	46	6	0.35	17.31	1.40E-02
macromolecular complex subunit organization (GO:0043933)	1689	34	12.72	2.67	9.76E-04
cellular component organization (GO:0016043)	5242	72	39.49	1.82	1.38E-04
cellular process (GO:0009987)	14882	140	112.12	1.25	7.86E-04
cellular component organization or biogenesis (GO:0071840)	5457	74	41.11	1.8	1.28E-04
**negative regulation of protein metabolic process (GO:0051248)**	1087	24	8.19	2.93	2.02E-02
negative regulation of biological process (GO:0048519)	4778	64	36	1.78	4.22E-03
negative regulation of macromolecule metabolic process (GO:0010605)	2444	39	18.41	2.12	3.47E-02
**negative regulation of catalytic activity (GO:0043086)**	866	21	6.52	3.22	2.22E-02
**positive regulation of RNA splicing (GO:0033120)**	28	5	0.21	23.7	2.34E-02
regulation of RNA splicing (GO:0043484)	110	8	0.83	9.65	1.95E-02
biological regulation (GO:0065007)	11745	118	88.49	1.33	7.51E-03
positive regulation of biological process (GO:0048518)	5400	72	40.68	1.77	5.06E-04
**macromolecular complex assembly (GO:0065003)**	1448	28	10.91	2.57	3.39E-02
cellular component assembly (GO:0022607)	2254	42	16.98	2.47	1.88E-04
cellular component biogenesis (GO:0044085)	2508	44	18.89	2.33	4.56E-04
**response to chemical (GO:0042221)**	3897	53	29.36	1.81	4.65E-02
**multicellular organismal process (GO:0032501)**	6633	77	49.97	1.54	4.77E-02

^a^significantly enriched most specific (boldface) biological processes with significantly enriched parent processes from Gene Ontology, sorted according to *p* values of the most specific biological processes.

^b^Bonferroni-corrected for multiple testing.

The PDE proteins that changed in abundance following AlaGln treatment were subjected to IPA network analysis and analysis of upstream regulators. The three largest networks were centered on “Akt and Hsp” (Network 1, Fig. 5*C*), “lipoproteins and the proteasome system” (Network 2, supplemental Fig. S2) and “integrin B1” (Network 3, Fig. 5*D*). The analysis considered only those upstream regulatory elements with *p* values for overlap less than 0.05 and activation z-scores above 1.5 or below −1.5 (Table III). supplemental Table S11 lists all networks including associated molecules.

**Table III:** Upstream regulators

Upstream Regulator	Molecule Type	Predicted Activation State	Expr Log Ratio^a^	Activation z-score^b^	*p* value of overlap^c^	Target molecules in dataset
IFNG	Cytokine	Inhibited	−	−3.107	6.36E-04	ACTA2, ALDH7A1, AZGP1, BCL2L2, CD163L1, CSE1L, F11R, GNB4, HLA-B, HLA-DRA, HMGB1, HSP90AB1, ITGAM, ITGB1, ITGB2, KCTD12, PRDM1, PSMA2, PSMA6, TIMP1, TIMP4
KLF4	transcription regulator	Inhibited	−	−2.42	6.62E-04	ACTA2, ACTC1, ALB, EFEMP1, ITGAM, PPP2CA, PRDM1, TF
EDN1	Cytokine	Inhibited	−	−2.372	1.28E-04	ACTA2, CDC42, ITGAM, ITGB1, NCF1, RPSA, TIMP1, TUBB1
F2	Peptidase	Inhibited	−0.037	−2.186	4.52E-02	ACTA2, EIF4A1, ITGB1, NCF1, NCF1C
EGR2	transcription regulator	Inhibited	−	−2	5.82E-03	GNB4, NSF, PPP2CA, PRDM1, SLC16A1
VCAN	Other	Inhibited	−0.009	−1.982	1.71E-02	CD59, ITGB1, LOXL4, TIMP1
IL27	Cytokine	Inhibited	−	−1.972	1.63E-02	HLA-B, HLA-DRA, ITGAM, PRDM1
Vegf	Group	Inhibited	−	−1.941	1.35E-02	ACTA2, CDC42, CRYAB, CSE1L, CSGALNACT1, ITGB1, MAP2K3, PABPN1, TIMP1
RICTOR	Other	Inhibited	−	−1.633	1.48E-02	PSMA2, PSMA6, PSMB6, PSMD4, RPL21, RPSA
TGFB1	growth factor	Inhibited	−0.423	−1.509	1.95E-04	ACTA2, ACTC1, ACTG2, ALB, APOB, CD59, CPQ, DYNLL1, GNB4, ITGAM, ITGB1, ITGB2, MAP2K3, NCF1, PA2G4, PDLIM7, PNP, PPP2CA, PRDM1, RPN2, SERPINF1, SNRNP70, SRSF3, SRSF4, SRSF6, TIMP1, TJP2
SMAD3	transcription regulator	Inhibited	−	−1.506	3.85E-04	ACTA2, ALB, APOB, CD59, HP, ITGB1, TF, TIMP1
IL4	Cytokine	Inhibited	−	−1.505	1.02E-03	ACTA2, APRT, CBX3, CD163L1, EIF4A1, EPHX1, ITGB1, ITGB2, LIPG, PKP3, PNP, PRDM1, PSMA2, PSMA6, TIMP1
EGFR	Kinase	Activated	−	1.546	8.81E-03	ACTA2, CRYAB, EIF4E, HNRNPD, HP, NSF, PA2G4, PSMB6
NOS2	Enzyme	Activated	−	2	5.16E-02	ACTC1, AZGP1, KRT4, TIMP1
FSH	Complex	Activated	−	2.153	1.05E-02	ACTA2, ACTG2, BCL2L2, MAP2K3, PPP2R1A, STK24, TF, TIMP1

^a^Expression log ratio for those proteins that were actually identified in PDE; “-“ for indirectly identified upstream regulators.

^b^z-score: ≥ 1.5, upstream regulator is activated; ≤− 1.5: upstream regulator is inhibited.

^c^*p* value: calculated by Fisher's Exact Test, indicating statistical significance of downstream genes in the data set.

## DISCUSSION

The choice of PD as an initial dialysis modality can be associated with improved outcomes for patients and lower costs to health care systems (1, 41). However, the molecular mechanisms underlying the most common reasons for discontinuation of PD, including infectious peritonitis, chronic sterile inflammation, membrane and ultrafiltration failure, remain incompletely understood. In contrast to some other clinical conditions, up to 20 liters per day of PD biomaterial (peritoneal dialysis effluent, PDE) containing small molecules, proteins and cells is available every few hours at no additional burden to the patient.

PDE thus represents not only a rich pool of potential biomarkers for monitoring PD therapy but also an ideal liquid biopsy for systems biology analyses, including multi-omics techniques. For the cells contained in PDE highly sensitive transcriptomic and (to a lesser degree) proteomic studies have been informative (27, 42). Analysis of the soluble protein repertoire of cell-free PDE will likely also provide important insight into the peritoneal transport and membrane status of the patient, and into mechanisms involving secretory pathways and cell injury, *i.e.* proteins released on purpose or by disintegration of distinct peritoneal cell populations.

Despite these obvious investigational benefits of PDE, current strategies of proteomic analysis of soluble PDE proteins in cell-free PDE have typically been compromised by the high dynamic range of protein concentrations, resembling a plasma-like composition with several high abundance proteins. Because of the employed analytical techniques, these challenges have limited previously reported proteomic analyses of PDE to identification of only 171 unique proteins, most of which are high-abundance plasma proteins (7–19).

To overcome the problem of these proteins masking low abundance biomarker candidates in PDE, we developed a novel two-component workflow. The first component (recently assessed in artificial PDE) is based on incubation of PDE with a bead-coupled, combinatorial peptide ligand library (CPLL) followed by elution of the enriched proteome in a small volume of buffer compatible with downstream analysis (20). Only the application of the CPLL technique to the analysis of artificial PDE permitted 2D-DIGE detection of low abundance cellular proteins spiked-in during sample preparation. The beads harbor a limited number of semispecific binding sites for each protein component of the PDE sample. Whereas high abundance proteins quickly saturate the binding sites (and the excess is washed away), low abundance proteins can be completely captured by the beads and so are relatively enriched. Therefore, the proteome is relatively equalized, such that ratios of high abundance proteins in the bead-treated samples will be compressed in quantitative analysis. Although the precise mechanism of binding has been disputed (43), we have shown for low abundance proteins that the CPLL procedure influences quantitation to a lesser extent (20). It should be noted, that a suboptimal bead-to-PDE ratio, by which high abundance proteins are insufficiently removed by the CPLL beads, or the use of insensitive downstream methods still results in loss of low abundance proteins (8, 19). The second workflow component is therefore the application of highly accurate methods of separation, detection and quantitation, including use of sensitive fluorescent dyes in gels and isobaric tags in LC-MS for efficient and quantitative comparison of PDE samples.

The interindividual variation among PD patients, combined with typically small cohort sizes, together constitute a major challenge in obtaining significant results from -omic studies because of massive undersampling. Proteomic studies have traditionally responded to this challenge by pooling clinical samples in a single multiplex run, with subsequent validation by alternate methods, such as ELISA, immunoblot, or targeted LC-MS. This strategy, however, can lead to false-positive and false-negative results through loss of the information inherent to individual variation. In contrast, our approach integrates biological variation through independent analyses of each of the forty PDE samples collected from a recent clinical PD trial of AlaGln supplementation of PD fluid (27). This approach optimally exploited the information content of the biomaterial (6, 20) by increasing the number of detected PDE proteins well beyond that previously reported, yielding a more balanced distribution of proteins over cellular components and processes (including informative cellular processes).

The mechanisms that determine the composition of the PDE proteome remain unknown. Plasma proteins have been hypothesized to undergo transport into the peritoneal cavity in proportion to their apparent molecular weight in their quaternary structure, with a linear correlation among the PDE to plasma concentration ratio and that molecular mass (44). Other sources of PDE proteins include free-floating leukocytes that migrate into the peritoneal cavity and detached or resident mesothelial cells of the peritoneal wall (45). Each of these cell types has been shown to secrete proteins (including danger signals) and to contribute to the secretome through cellular disintegration, thereby regulating peritoneal sterile inflammation (25).

We hypothesized that the differential compositions of PDE and plasma can be interpreted in terms of the biological processes associated with over- and underrepresented proteins. By comparing the abundance ranking of proteins in the PDE proteome to a consensus plasma proteome, we discriminated soluble factors that were most prominently over- or underrepresented in PDE compared with plasma. Among the 100 proteins of highest relative abundance in PDE, we found enrichment of processes related to fiber formation and extracellular matrix formation. The peritoneum of long-term PD patients undergoes progressive remodeling with the accumulation of extracellular matrix leading to fibrosis (46). In our study, the pattern of enrichment of proteins involved in these processes is consistent with recently published cell-derived signatures of mesothelial-mesenchymal transition (MMT) (47, 48). Among the proteins of lowest relative abundance in PDE compared with plasma, we found enrichment of processes related to host defense, including neutrophil and platelet degranulation, antimicrobial killing, inflammation signaling, regulation of stress responses, and oxidative stress. These findings derived from our novel workflow boost the potential of the PDE proteome to provide deeper insights into biological processes underlying the pathological changes of the peritoneum that typically accompany PD therapy (3, 4).

Peritonitis still represents the most prominent serious complication of chronic PD, likely reflecting decreased immune competence in the peritoneum (6, 42). Our results are also a first step toward defining the molecular mechanisms for peritonitis risk, and toward developing the ability to identify the subgroup of PD patients at highest risk for peritonitis. The findings do not, however, determine whether the changes in soluble PDE proteins cause or are the result of peritoneal pathomechanisms such as membrane fibrosis and impaired host defense. The secreted proteins could produce sterile inflammation, in turn releasing factors that stimulate deposition of excessive extracellular matrix leading to fibrosis (49, 50). In addition, the pathophysiology of the peritoneum reflects both PD fluid-induced local alterations and systemic effects such as the chronic inflammation and oxidative stress present in uremia and other clinical settings. One approach to further investigation will be to interfere with the PDE proteome and analyze resulting functional changes.

We therefore hypothesized in the second part of this study that the PDE proteome can be exploited to study the molecular mechanisms of action of AlaGln supplementation of PD fluids in clinical PD. AlaGln supplementation modulated heat shock protein (HSP) expression in experimental systems, and increased abundance of peritoneal cell HSP as the primary outcome parameter in the clinical trial that served as source of our current PDE samples (27). However, in view of very low PDE cell number and the tedious nature of PDE cell harvest procedures, demonstration that detectable changes in soluble PDE protein abundance might reflect molecular changes in peritoneal cellular processes would be of great interest.

TMT quantitation of PDE proteomes of patients treated with AlaGln revealed significant enrichment of cellular processes linked to stress responses, immune defense and fibrosis. To elucidate, if this enrichment represents activation or inactivation, we further characterized involved players. Unbiased analysis of our data set highlighted the Akt pathway as a signaling hub of the peritoneal stress response. This is particularly interesting in the context of increased HSP expression in peritoneal cells harvested from AlaGln-supplemented PDE samples in the same clinical trial (27), and suggests that AlaGln addition to PD fluid might improve the cellular stress response by modulating Akt-dependent pathways. We further speculate that regulation of stress kinases during PD fluid exposure may be required to regulate HSP induction in peritoneal effluent cells (27). Down-regulation in our networks of AMP-activated protein kinase (AMPK) has also been associated with HSP regulation (51). As both kinases phosphorylate heat shock factor 1 (HSF1), future studies in PD might profitably focus on this key transcription factor controlling HSP expression.

In the original analysis of the clinical trial from which our PDE samples were obtained, AlaGln supplementation of PD fluid modulated basal peritoneal inflammation, potentially leading to lower chronic inflammation, while at the same time increasing responsiveness of immune cells to specific stimuli, such as toll-like receptor (TLR) ligands (27). This latter finding was confirmed in a small follow-up pilot trial, in which the transcriptome of peritoneal effluent cells exposed to AlaGln-supplemented PD fluid revealed lower basal tumor necrosis factor alpha (TNFα) levels whereas responsiveness to LPS was increased (42).

The current study has complemented these findings by analyzing upstream regulatory elements based on proteins differentially abundant in PDE from patients treated in random sequence with and without AlaGln. The most strongly inhibited regulators included interferon gamma (IFNγ), transforming growth factor beta 1 (TGFβ1) and vascular endothelial growth factor (VEGF), indicating down-regulation of pro-fibrotic and pro-angiogenic processes (47, 48). The association of basal IFNγ levels with increased inflammation might explain the clinical observation of smoldering sterile inflammation in the dialyzed peritoneal cavity. Future AlaGln studies with longer treatment phases should therefore evaluate inhibition of this pathway as a strategy to reduce peritonitis risk. Increased basal IFNγ levels might additionally increase the risk of longer-term complications such as encapsulating peritoneal sclerosis (EPS) (52).

With respect to the peritoneal membrane remodeling observed in PD, VEGF, TGFβ1, and SMAD3 (found in the same cluster) are prototypical inducers of membrane transformation (49, 53). The observed effects of AlaGln are particularly interesting, in light of the recently described rodent model of chronic PD in which *in vivo* AlaGln supplementation of PD fluid decreased membrane deterioration and fibrosis, associated with attenuation of inflammatory pathways with interleukin 17 (IL-17) as key driver (23). Our results also revealed AlaGln-associated inhibition of PRDM1 and VCAN, both previously described as drivers of endometriosis (54, 55). Integrins constitute another key hub in the networks around the inhibitory regulators. The observed biological processes around extracellular matrix, elastic fiber formation and fibrosis are also supported by an interaction network centered around down-regulated integrin beta-1 (ITGB1) also associated with early epithelial-mesenchymal transition (EMT) (56, 57). Thus, the observed effects on regulatory elements could contribute to a molecular mechanistic explanation for suppression of membrane deterioration in PD.

Analysis of sample variation and clustering confirmed that changes associated with AlaGln treatment were smaller than the range of variation among individual patients, consistent with our previous studies investigating the stress proteome of peritoneal cells in response to PD fluid exposure. We therefore decided to use accurate relative quantitation based on TMT labeling for the paired samples in the cross-over trial within patients. A potential limitation of this approach resides in having obtained the data on overall PDE composition from 7 groups of 3 patients, each under standard and AlaGln treatment, based on label-free quantitation algorithms. Although, the paired cross-over design is ideal to compare within patient effects of treatment with and without AlaGln, the analysis of differences between patients or subgroups requires much larger numbers because of interindividual variation, as well as matching among TMT runs *e.g.,* with internal standards. The option of using an internal standard to compare PDE composition among patients based on TMT data would imply that only 2 patients can be compared within a 6-plex. Future studies using this approach will therefore either rely on more highly multiplexed tags or on more sophisticated normalization techniques, currently under development, to more effectively meet both goals (interindividual and intra-individual precision). The effort and cost required to overcome the inter- and intraindividual variation even in larger cohorts represent limitations inherent to system-wide discovery. These will also be addressed with follow-up studies exploiting candidate markers in a targeted approach without prior depletion of high-abundance proteins.

In principle, this technique should be applicable to all biological fluids available in high volumes but compromised by a background of high abundance proteins, such as ascites or nondialysis related peritoneal fluid. However, these applications remain to be validated.

In conclusion, this study demonstrates that a novel workflow integrating a depletion and enrichment approach with FASP digestion and TMT-labeling of all individual samples from a complete clinical trial enables identification and quantification in PDE of 15-fold higher numbers of individual proteins than the combined previous literature on PDE. This proteome thus encompasses not only greater numbers of processes, pathways and cellular components, but also provides a better balance among typical extracellular proteins (including high abundance plasma proteins) and proteins of cellular origin. Lastly, this workflow allowed analysis of the differential effects of AlaGln addition in PDE samples from a randomized, controlled, cross-over clinical trial, providing relevant molecular mechanistic insights on interference with these pathomechanisms. Future implementation of this proteomic workflow may help define novel biomarkers for disease staging and therapeutic monitoring, and help evaluate additional novel cytoprotective interventions for PD.

## DATA AVAILABILITY

All mass spectrometry data, corresponding proteins, protein coverage and raw data files have been deposited into the ProteomeXchange Consortium (http://proteomecentral.proteomexchange.org) with the data set identifier PXD006863.

## Supplementary Material

Supplemental Data

## Footnotes

^1^ The abbreviations used are:

PD: peritoneal dialysis
2D-DIGE: two dimensional difference gel electrophoresis
2D-RP/RP: two dimensional reversed phase
AGC: automatic gain control
AlaGln: alanyl-glutamine
AMPK: AMP-activated protein kinase
CHAPS: 3-[(3-cholamidopropyl) dimethylammonio]-1-propanesulfonate
CPLL: combinatorial peptide ligand library
CFT: CPLL-FASP-TMT
EMT: epithelial-mesenchymal transition
EPS: encapsulating peritoneal sclerosis
FASP: filter-aided sample preparation
FDR: false discovery rate
GO: gene ontology
HCD: higher-energy collision-induced dissociation
HPLC: high-performance liquid chromatography
HPPDB: human plasma proteome data base
HSF: heat shock factor
HSP: heat shock protein
IL: interleukin
IFN: interferon
IPA: Ingenuity Pathway Analysis
ITG: integrin
LTQ: linear trap quadrupole
MMT: mesothelial-mesenchymal transition
PAMS: pathomechanism-associated molecular signatures
PDE: peritoneal dialysis effluent
PET: peritoneal equilibration test
RCT: randomized controlled trial
TEAB: triethyl-ammonium bicarbonate
TGF: transforming growth factor
TLR: toll-like receptor
TMT: tandem mass tag
VEGF: vascular endothelial growth factor.
