# Supplementary material for: Effects of Alanyl-Glutamine Treatment on the Peritoneal Dialysis Effluent Proteome Reveal Pathomechanism-Associated Molecular Signatures
Source: Mol Cell Proteomics. 2017 Dec 4;17(3):516–32. doi: 10.1074/mcp.RA117.000186 (PMC5836375; doi:10.1074/mcp.RA117.000186)
Supplement: Supplemental Data [file supp_17_3_516__index.html]

Effects of alanyl-glutamine treatment on the peritoneal dialysis effluent proteome reveal pathomechanism-associated molecular signatures — Exploration of the peritoneal dialysis effluent proteome — Effects of Alanyl-Glutamine Treatment on the Peritoneal Dialysis Effluent Proteome Reveal Pathomechanism-Associated Molecular Signatures — Exploration of the Peritoneal Dialysis Effluent Proteome — Supplemental Data 

# Effects of Alanyl-Glutamine Treatment on the Peritoneal Dialysis Effluent Proteome Reveal Pathomechanism-Associated Molecular Signatures

## Supplemental Data

- Supplemental Figures - Supplemental Figure S1 and S2
- Supplemetal Table S1 - PDE literature
- Supplemental Table S2 - experimental design
- Supplemental Table S3 - MS IDs and Top3 abundance
- Supplemental Table S4 - TMT run data
- Supplemental Table S5 - GO CC an BP full list
- Supplemental Table S6 - HPPDB vs PDE
- Supplemental Table S7 - top 100 up and down ranked proteins - GO processes
- Supplemental Table S8 - MS IDs and Isobar-Ratios
- Supplemental Table S9 - Isobar Reports
- Supplemental Table S10 - AlaGln effect on high abundance proteins - GO processes
- Supplemental Table S11 - AlaGln effect IPA networks
