# Supplementary material for: Effects of Alanyl-Glutamine Treatment on the Peritoneal Dialysis Effluent Proteome Reveal Pathomechanism-Associated Molecular Signatures
Source: Mol Cell Proteomics. 2017 Dec 4;17(3):516–32. doi: 10.1074/mcp.RA117.000186 (PMC5836375; doi:10.1074/mcp.RA117.000186)

Supplemental Figures:

Effects of alanyl-glutamine treatment on the peritoneal dialysis effluent proteome reveal pathomechanism-associated molecular signatures

Rebecca Herzog[1,2], Michael Böhm[1], Markus Unterwurzacher[1,2], Anja Wagner[1,2], Katja Parapatics[3], Peter Májek[3], André C. Mueller[3], Anton Lichtenauer[1], Keiryn L. Bennett[3], Seth L. Alper[4,5], Andreas Vychytil[6], Christoph Aufricht[1], Klaus Kratochwill[1,2*]

[1] Division of Pediatric Nephrology and Gastroenterology, Department of Pediatrics and Adolescent Medicine, Medical University of Vienna, Vienna, Vienna, Austria

[2] Christian Doppler Laboratory for Molecular Stress Research in Peritoneal Dialysis, Department of Pediatrics and Adolescent Medicine, Medical University of Vienna, Vienna, Austria

[3] CeMM Research Center for Molecular Medicine of the Austrian Academy of Sciences, Vienna, Austria

[4] Division of Nephrology, Beth Israel Deaconess Medical Center, Boston, MA, USA

[5] Department of Medicine, Harvard Medical School, Boston, MA, USA

[6] Medical University of Vienna, Department of Medicine III, Division of Nephrology and Dialysis, Vienna, Austria

Running title: Exploration of the peritoneal dialysis effluent proteome

***Corresponding author:**
Priv.Doz. DI Dr. Klaus Kratochwill
Division of Pediatric Nephrology and Gastroenterology, Department of Pediatrics and Adolescent Medicine, Medical University of Vienna
Währinger Gürtel 18-20, AT-1090 Vienna
Phone: +43/1/40400-73747
Fax: +43/1/40400-73598
E-mail: klaus.kratochwill@meduniwien.ac.at

Supplemental Figures

Supplemental Figure S1: Effect of Alanyl-glutamine on PD effluent proteins. (A) Volcano plot for effect ratios and p-values for all proteins identified in PD effluent (PDE) in both samples of an individual patient for which at least 17 ratios were available. Outer segments (white) represent statistical cut-offs for process and pathway analyses (FC>1.2; p<0.05) (n=50). Color gradient visualizes the number of available effect ratios (=number of patients for whom identifications were available from both treatments). (B) Plot of abundance versus effect ratios and p-values for all proteins identified in PDE in both samples of an individual patient for which at least 17 ratios were available. Color gradient visualizes the number of available effect ratios.

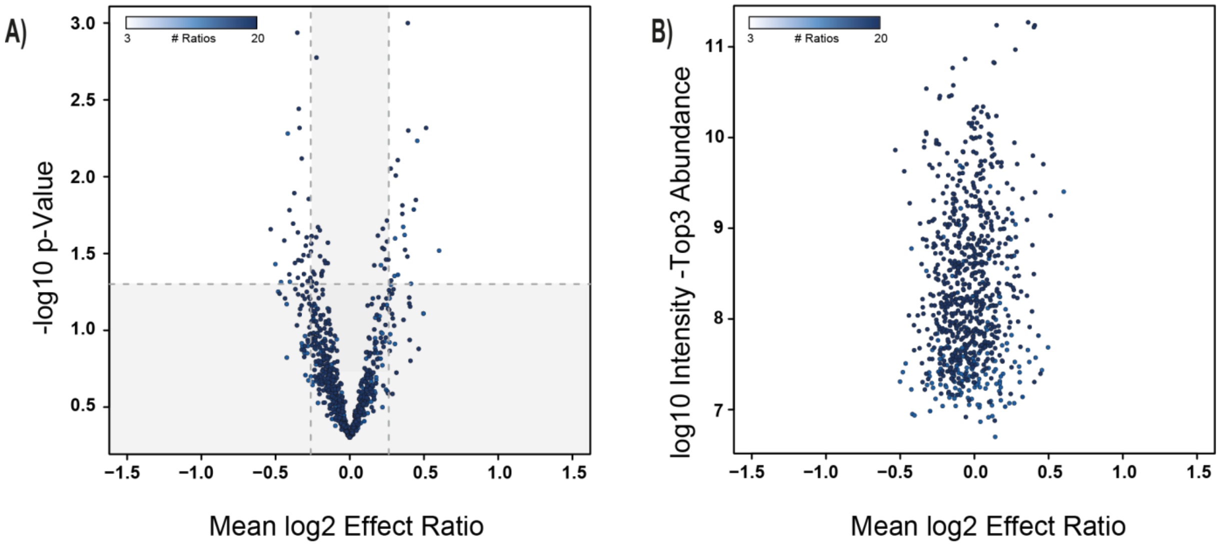

Supplemental Figure S2: Effect of Alanyl-glutamine on PD effluent proteins. Interaction networks generated from differentially expressed genes. Each node represents a gene and each edge an interaction between genes. The node colour indicates up-regulation (red) or down-regulation (green) in the presence of added AlaGln.

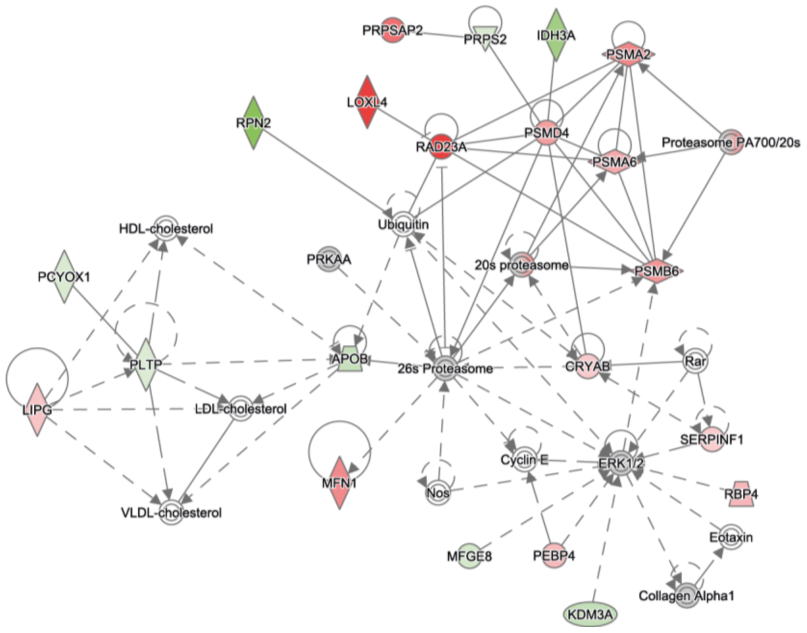

Supplement: Supplemental Data [file supp_RA117.000186_4744_1_supp_24988_rzj4bv.pdf]
