# Supplementary material for: Effects of Alanyl-Glutamine Treatment on the Peritoneal Dialysis Effluent Proteome Reveal Pathomechanism-Associated Molecular Signatures
Source: Mol Cell Proteomics. 2017 Dec 4;17(3):516–32. doi: 10.1074/mcp.RA117.000186 (PMC5836375; doi:10.1074/mcp.RA117.000186)
Supplement: Supplemental Data [file supp_RA117.000186_4744_1_supp_5319_rv4l0l.pdf]

Isobaric Tag Quantification Report
P6653_Run 1 *

isobar R package v1.18.0

February 6, 2017

Channel	Class
126	*P5-ctrl*
127	*P5-treat*
128	*P15-ctrl*
129	*P15-treat*
130	*P17-ctrl*
131	*P17-treat*

Column Descriptions

protein	Protein name, accession code, and description.
group	Protein group size, only displayed if it is not '1/1'. First number: Number of group reporters; second number: group size. See Protein Group Details for details.
peptides	Number of peptides for protein.
spectra	Number of idenfitied spectra for protein.
quant	Number of spectra with quantitative information for *ch2* versus *ch1*.
ratio	Ratio of protein in channel *ch2* relative to *ch1*.
*	Significance of protein ratio in sample.

1 Protein Relative Quantitation

Number of quantified proteins: P15-treat/P15-ctrl: 1312; P17-treat/P17-ctrl: 1310; P5-treat/P5-ctrl: 1305

1.1 Quantified Proteins

#	protein	group	peptides	spectra	ch1	ch2	quant	ratio
**1**	136429		7	93	126	127	82	**6.19**
					128	129	87	**2.43**
					130	131	91	**3.26**
**2**	162648		28	283	126	127	37	**5.49**
					128	129	30	**1.76**
					130	131	39	**2.48**
**3**	A0A0A0MS15		1	2	126	127	2	**0.64**
					128	129	2	**1.99**
					130	131	2	**0.97**

*This report was generated using the `isobar` R package version 1.18.0 [built using R 3.3.2; ; 2017-02-05 14:44:42 UTC; unix]. If you use it in published work, please cite 'Breitwieser FP *et al.*: General statistical modeling of data from protein relative expression isobaric tags, *Journal of Proteome Research* 2011' and 'Breitwieser FP and Colinge J: isobarPTM: A software tool for the quantitative analysis of post-translationally modified proteins,*Journal of Proteomics* 2013'

#	protein	group	peptides	spectra	ch1	ch2	quant	ratio
4	A0A0B4J1V0		2	3	126	127	3	**0.89**
					128	129	3	**2.08**
					130	131	3	**1.08**
5	A0A0C4DH25		3	10	126	127	1	**0.58**
					128	129	1	**2.35**
					130	131	1	**1.02**
6	A0A0C4DH33		3	21	126	127	12	**1.21**
					128	129	8	**1.24**
					130	131	8	**1.17**
7	A1BG P04217$_1$: Alpha-1B-glycoprotein	1/2	10	14	126	127	10	**1.11**
					128	129	12	**2.21**
					130	131	13	**1.02**
8	A2M P01023: Alpha-2-macroglobulin		43	90	126	127	75	**1.35**
					128	129	74	**2.00**
					130	131	71	**0.92**
9	AARS P49588$_{1,2}$: Alanine--tRNA ligase, cytoplasmic	2/2	9	11	126	127	10	**0.30**
					128	129	10	**0.28**
					130	131	11	**0.98**
10	ABHD14B Q96IU4$_1$: Protein ABHD14B		2	2	126	127	2	**0.43**
					128	129	2	**0.21**
					130	131	2	**1.01**
11	ABI3BP Q7Z7G0$_1$: Target of Nesh-SH3		3	4	126	127	3	**0.17**
					128	129	4	**0.19**
					130	131	3	**0.82**
12	ACLY P53396$_{1,2}$: ATP-citrate synthase	2/3	7	9	126	127	9	**0.79**
					128	129	9	**0.44**
					130	131	8	**1.02**
13	ACTA2 P62736, ACTC1 P68032: Actin, aortic smooth muscle, Actin, alpha cardiac muscle 1	2/5	16	239	126	127	62	**0.49**
					128	129	63	**0.41**
					130	131	59	**1.07**
14	ACTB P60709, ACTG1 P63261: Actin, cytoplasmic 1, Actin, cytoplasmic 2	2/3	19	260	126	127	61	**0.54**
					128	129	60	**0.38**
					130	131	61	**1.00**
15	ACTBL2 Q562R1: Beta-actin-like protein 2		7	123	126	127	1	**1.16**
					128	129	1	**1.74**
					130	131	1	**1.00**

#	protein	group	peptides	spectra	ch1	ch2	quant	ratio
**16**	ACTN1 P12814$_{1-4}$: Alpha-actinin-1	4/4	14	17	126	127	3	**0.96**
					128	129	3	**1.08**
					130	131	3	**1.17**
**18**	ACTN3 Q08043: Alpha-actinin-3		7	8	126	127	4	**3.61**
					128	129	4	**1.78**
					130	131	4	**1.34**
**19**	ACTN4 O43707$_1$: Alpha-actinin-4	1/3	20	29	126	127	14	**0.92**
					128	129	16	**0.72**
					130	131	14	**1.06**
**20**	ACTR1A P61163: Alpha-centractin		3	21	126	127	2	**2.00**
					128	129	2	**0.72**
					130	131	2	**1.20**
**21**	ACTR2 P61160$_1$: Actin-related protein 2		4	24	126	127	5	**1.49**
					128	129	5	**0.72**
					130	131	4	**0.92**
**22**	ACTR3 P61158: Actin-related protein 3		3	3	126	127	3	**1.51**
					128	129	3	**0.45**
					130	131	3	**0.79**
**23**	ADAM10 O14672$_1$: Disintegrin and metalloproteinase domain-containing protein 10		5	12	126	127	10	**0.34**
					128	129	7	**1.24**
					130	131	11	**1.01**
**24**	ADAM15 Q13444$_{1-10,12}$: Disintegrin and metalloproteinase domain-containing protein 15	11/13	4	5	126	127	5	**1.14**
					128	129	4	**1.34**
					130	131	5	**1.05**
**25**	ADAMDEC1 O15204$_{1,2}$: ADAM DEC1	2/2	3	3	126	127	3	**0.55**
					128	129	3	**1.37**
					130	131	3	**0.85**
**26**	ADAMTS1 Q9UHI8: A disintegrin and metalloproteinase with thrombospondin motifs 1		1	2	126	127	2	**0.19**
					128	129	2	**1.19**
					130	131	2	**0.93**
**27**	ADAMTS15 Q8TE58: A disintegrin and metalloproteinase with thrombospondin motifs 15		5	5	126	127	4	**0.65**
					128	129	4	**1.42**
					130	131	3	**0.90**
**28**	ADAMTS2 O95450$_{1,2}$: A disintegrin and metalloproteinase with thrombospondin motifs 2	2/2	5	8	126	127	7	**0.83**
					128	129	7	**2.20**
					130	131	7	**0.93**

#	protein	group	peptides	spectra	ch1	ch2	quant	ratio
**29**	ADAMTS5 Q9UNA0: A disintegrin and metalloproteinase with thrombospondin motifs 5		5	6	126	127	4	**0.49**
					128	129	5	**1.80**
					130	131	4	**0.99**
**30**	ADAMTS8 Q9UP79: A disintegrin and metalloproteinase with thrombospondin motifs 8		2	3	126	127	3	**0.84**
					128	129	3	**1.25**
					130	131	3	**0.95**
**31**	ADAMTSL1 Q8N6G6$_{3}$: ADAMTS-like protein 1	1/3	5	5	126	127	5	**0.53**
					128	129	5	**0.99**
					130	131	5	**0.97**
**32**	ADAMTSL2 Q86TH1: ADAMTS-like protein 2		2	2	126	127	2	**0.40**
					128	129	2	**1.93**
					130	131	2	**0.95**
**33**	ADAMTSL4 Q6UY14$_{1,3}$: ADAMTS-like protein 4	2/3	14	18	126	127	15	**0.85**
					128	129	16	**1.33**
					130	131	17	**0.97**
**34**	ADH5 P11766: Alcohol dehydrogenase class-3		8	9	126	127	9	**0.67**
					128	129	6	**0.44**
					130	131	9	**1.11**
**35**	ADIPOQ Q15848: Adiponectin		3	5	126	127	4	**0.72**
					128	129	4	**1.30**
					130	131	5	**1.15**
**36**	AEBP1 Q8IUX7$_{1}$: Adipocyte enhancer-binding protein 1	1/2	33	79	126	127	73	**0.47**
					128	129	76	**0.78**
					130	131	76	**1.01**
**37**	AFM P43652: Afamin		27	83	126	127	79	**0.59**
					128	129	80	**1.35**
					130	131	77	**0.93**
**38**	AGRN O00468$_{1-7}$: Agrin [Cleaved into: Agrin N-terminal 110 kDa subunit; Agrin C-terminal 110 ...	7/7	9	12	126	127	9	**0.63**
					128	129	10	**0.42**
					130	131	11	**0.97**
**39**	AGT P01019: Angiotensinogen		8	16	126	127	15	**0.38**
					128	129	14	**0.24**
					130	131	15	**0.86**
**40**	AHNAK Q09666$_{1}$: Neuroblast differentiation-associated protein AHNAK		31	36	126	127	31	**5.12**
					128	129	32	**1.35**
					130	131	30	**0.86**

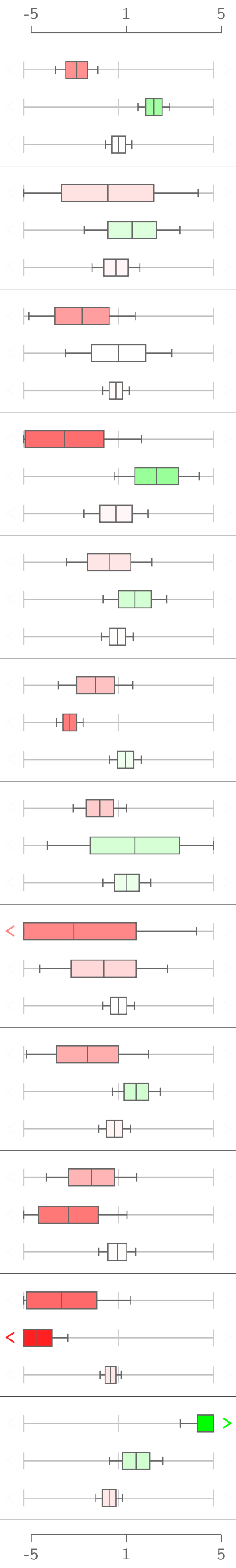

#	protein	group	peptides	spectra	ch1	ch2	quant	ratio
**41**	AHSA1 O95433$_1$: Activator of 90 kDa heat shock protein ATPase homolog 1	1/2	4	5	126	127	5	**0.96**
					128	129	5	**0.37**
					130	131	3	**0.87**
**42**	AHSG P02765: Alpha-2-HS-glycoprotein		13	417	126	127	376	**0.60**
					128	129	414	**1.28**
					130	131	404	**0.95**
**43**	AK1 P00568: Adenylate kinase isoenzyme 1		4	4	126	127	4	**1.06**
					128	129	4	**0.45**
					130	131	3	**1.12**
**44**	AKNA Q7Z591$_{1,2,4,5,7,8}$: AT-hook-containing transcription factor	6/6	1	5	126	127	4	**0.82**
					128	129	5	**2.25**
					130	131	3	**1.08**
**45**	ALB P02768$_1$: Serum albumin	1/3	60	2995	126	127	2506	**0.98**
					128	129	2592	**1.51**
					130	131	2515	**0.98**
**46**	ALDH1A2 O94788$_1$: Retinal dehydrogenase 2	1/2	5	5	126	127	4	**0.89**
					128	129	3	**0.52**
					130	131	4	**0.98**
**47**	ALDH1A3 P47895: Aldehyde dehydrogenase family 1 member A3		8	8	126	127	7	**1.46**
					128	129	6	**0.55**
					130	131	7	**0.98**
**48**	ALDH7A1 P49419$_{1,2,4}$: Alpha-aminoadipic semialdehyde dehydrogenase	3/3	2	2	126	127	2	**0.38**
					128	129	2	**0.21**
					130	131	2	**1.04**
**49**	ALDH9A1 P49189$_1$: 4-trimethylaminobutyraldehyde dehydrogenase	1/2	5	6	126	127	6	**0.96**
					128	129	6	**0.65**
					130	131	5	**1.17**
**50**	ALDOA P04075$_{1,2}$: Fructose-bisphosphate aldolase A	2/3	7	7	126	127	7	**4.03**
					128	129	6	**0.67**
					130	131	7	**0.98**
**51**	ALOX15 P16050$_{1,2}$: Arachidonate 15-lipoxygenase	2/2	2	2	126	127	2	**0.95**
					128	129	2	**0.52**
					130	131	2	**1.15**
**52**	ALYREF Q86V81: THO complex subunit 4		2	3	126	127	3	**2.70**
					128	129	3	**1.30**
					130	131	3	**1.03**

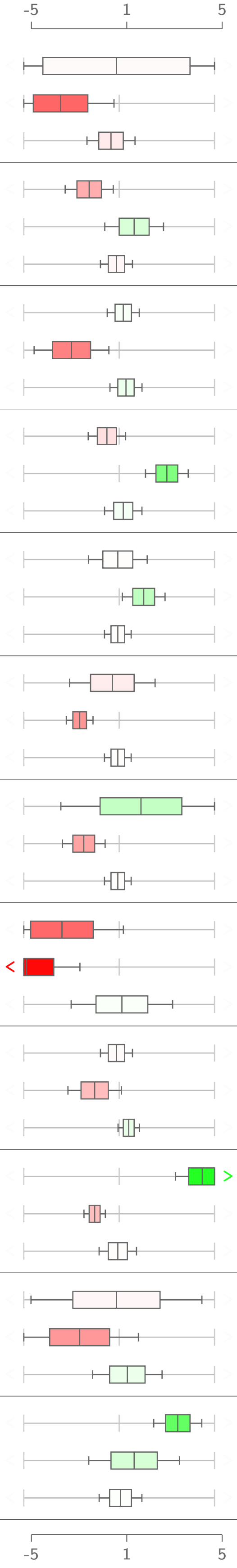

#	protein	group	peptides	spectra	ch1	ch2	quant	ratio
**53**	AMBP P02760: Protein AMBP [Cleaved into: Alpha-1-microglobulin		15	182	126	127	130	**0.53**
					128	129	151	**0.75**
					130	131	145	**1.06**
**54**	ANG P03950: Angiogenin		12	172	126	127	159	**0.94**
					128	129	167	**2.05**
					130	131	161	**1.08**
**55**	ANGPTL1 O95841: Angiopoietin-related protein 1		3	3	126	127	2	**0.70**
					128	129	2	**1.07**
					130	131	2	**0.90**
**56**	ANGPTL2 Q9UKU9$_1$: Angiopoietin-related protein 2	1/2	9	14	126	127	13	**1.70**
					128	129	12	**1.05**
					130	131	12	**0.90**
**57**	ANGPTL4 Q9BY76$_1$: Angiopoietin-related protein 4	1/3	7	11	126	127	11	**0.83**
					128	129	11	**1.34**
					130	131	11	**1.11**
**58**	ANGPTL8 Q6UXH0: Angiopoietin-like protein 8		2	2	126	127	2	**2.25**
					128	129	2	**1.30**
					130	131	2	**0.42**
**59**	ANK1 P16157$_{1,3,5,8,10,12,14,16,21}$: Ankyrin-1	9/9	3	4	126	127	3	**1.46**
					128	129	3	**1.49**
					130	131	3	**0.96**
**60**	ANTXR1 Q9H6X2$_1$: Anthrax toxin receptor 1	1/6	10	12	126	127	9	**0.69**
					128	129	11	**0.99**
					130	131	11	**0.87**
**61**	ANTXR2 P58335$_{1,2,4}$: Anthrax toxin receptor 2	3/4	7	7	126	127	5	**0.15**
					128	129	6	**0.21**
					130	131	6	**0.90**
**62**	ANXA1 P04083: Annexin A1		9	14	126	127	12	**3.71**
					128	129	13	**1.61**
					130	131	12	**0.81**
**63**	ANXA11 P50995$_{1,2}$: Annexin A11	2/2	3	3	126	127	3	**4.13**
					128	129	3	**1.01**
					130	131	3	**0.90**
**64**	ANXA2 P07355$_{1,2}$: Annexin A2	2/3	15	33	126	127	30	**11.01**
					128	129	32	**2.40**
					130	131	30	**0.72**

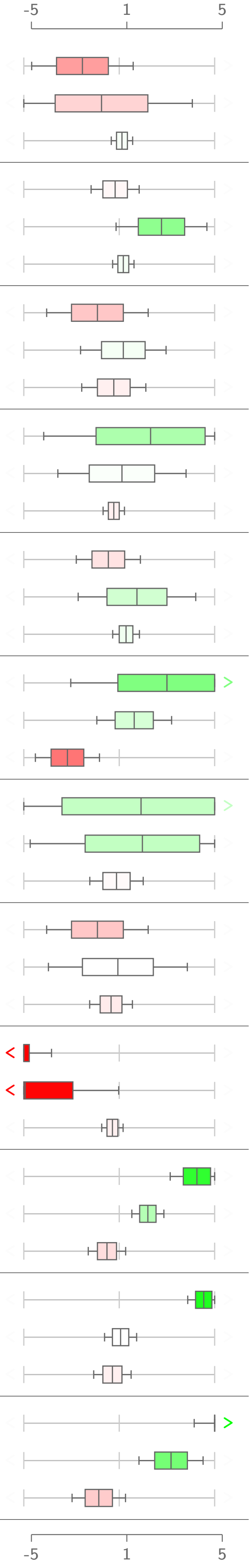

#	protein	group	peptides	spectra	ch1	ch2	quant	ratio
65	ANXA4 P09525$_1$: Annexin A4	1/2	3	4	126	127	1	**15.54**
					128	129	1	**3.50**
					130	131	1	**0.80**
66	ANXA5 P08758: Annexin A5		8	9	126	127	5	**7.40**
					128	129	4	**1.77**
					130	131	4	**0.72**
67	ANXA6 P08133$_{1,2}$: Annexin A6	2/2	12	13	126	127	12	**2.84**
					128	129	13	**0.87**
					130	131	13	**0.83**
68	AP1B1 Q10567$_{1-4}$: AP-1 complex subunit beta-1	4/4	5	5	126	127	1	**0.06**
					128	129	1	**0.10**
					130	131	1	**0.97**
69	AP1G1 O43747$_{1,2}$: AP-1 complex subunit gamma-1	2/2	2	2	126	127	2	**0.23**
					128	129	2	**0.25**
					130	131	2	**1.01**
70	AP1M1 Q9BXS5$_{1,2}$: AP-1 complex subunit mu-1	2/2	2	2	126	127	2	**0.47**
					128	129	2	**0.36**
					130	131	2	**0.98**
71	AP2A1 O95782$_{1,2}$, AP2A2 O94973$_{1-3}$: AP-2 complex subunit alpha-1, AP-2 complex subunit alpha-2	5/5	3	3	126	127	3	**0.57**
					128	129	3	**0.51**
					130	131	3	**1.01**
72	AP2B1 P63010$_{1,2}$: AP-2 complex subunit beta	2/3	6	6	126	127	2	**0.99**
					128	129	2	**0.37**
					130	131	2	**0.87**
73	AP2M1 Q96CW1$_{1,2}$: AP-2 complex subunit mu	2/2	2	2	126	127	2	**1.00**
					128	129	2	**0.58**
					130	131	2	**0.90**
74	AP3D1 O14617$_5$: AP-3 complex subunit delta-1	1/5	5	7	126	127	7	**0.75**
					128	129	6	**1.12**
					130	131	6	**0.98**
75	APCS P02743: Serum amyloid P-component		9	254	126	127	245	**0.98**
					128	129	240	**0.84**
					130	131	209	**1.05**
76	APEH P13798: Acylamino-acid-releasing enzyme		1	1	126	127	1	**0.80**
					128	129	1	**1.15**
					130	131	1	**1.13**

#	protein	group	peptides	spectra	ch1	ch2	quant	ratio
**77**	API5 Q9BZZ5$_{1,2,4-6}$: Apoptosis inhibitor 5	5/6	3	3	126	127	3	**0.54**
					128	129	3	**0.87**
					130	131	3	**1.00**
**78**	APLP2 Q06481$_{1,3,6}$: Amyloid-like protein 2	3/6	5	16	126	127	15	**2.29**
					128	129	13	**1.66**
					130	131	12	**1.37**
**79**	APMAP Q9HDC9$_{1}$: Adipocyte plasma membrane-associated protein	1/2	7	7	126	127	6	**0.79**
					128	129	6	**0.36**
					130	131	7	**0.95**
**80**	APOA1 P02647: Apolipoprotein A-I		34	2431	126	127	2104	**0.75**
					128	129	2124	**1.82**
					130	131	2058	**1.10**
**81**	APOA2 P02652: Apolipoprotein A-II		5	366	126	127	346	**0.58**
					128	129	347	**0.93**
					130	131	357	**0.98**
**82**	APOA4 P06727: Apolipoprotein A-IV		40	2577	126	127	2167	**0.98**
					128	129	2304	**2.60**
					130	131	2225	**1.05**
**83**	APOA5 Q6Q788: Apolipoprotein A-V		4	4	126	127	3	**2.30**
					128	129	3	**1.05**
					130	131	3	**1.10**
**84**	APOB P04114: Apolipoprotein B-100		215	768	126	127	718	**0.67**
					128	129	699	**0.10**
					130	131	731	**0.87**
**85**	APOBR Q0VD83$_{1,3,4}$: Apolipoprotein B receptor	3/4	6	6	126	127	6	**0.75**
					128	129	6	**0.56**
					130	131	5	**1.18**
**86**	APOC1 P02654: Apolipoprotein C-I		6	113	126	127	110	**1.81**
					128	129	112	**2.49**
					130	131	99	**0.43**
**87**	APOC2 P02655: Apolipoprotein C-II		4	60	126	127	54	**1.05**
					128	129	50	**0.91**
					130	131	55	**0.33**
**88**	APOC3 P02656: Apolipoprotein C-III		5	271	126	127	225	**0.78**
					128	129	225	**0.89**
					130	131	235	**0.96**

#	protein	group	peptides	spectra	ch1	ch2	quant	ratio
89	APOC4 P55056: Apolipoprotein C-IV		6	7	126	127	7	**0.64**
					128	129	7	**0.18**
					130	131	7	**0.93**
90	APOD P05090: Apolipoprotein D		8	345	126	127	331	**0.60**
					128	129	333	**0.84**
					130	131	320	**1.26**
91	APOE P02649: Apolipoprotein E		27	366	126	127	346	**0.67**
					128	129	351	**0.45**
					130	131	331	**1.01**
92	APOF Q13790: Apolipoprotein F		5	13	126	127	9	**1.38**
					128	129	12	**0.48**
					130	131	13	**1.07**
93	APOH P02749: Beta-2-glycoprotein 1		12	28	126	127	28	**1.38**
					128	129	25	**2.66**
					130	131	26	**0.95**
94	APOL1 O14791$_{1-3}$: Apolipoprotein L1	3/3	15	115	126	127	94	**0.24**
					128	129	88	**0.08**
					130	131	102	**0.79**
95	APOL2 Q9BQE5: Apolipoprotein L2		4	7	126	127	7	**0.69**
					128	129	7	**0.52**
					130	131	7	**0.90**
96	APOL3 O95236$_{1-3}$: Apolipoprotein L3	3/3	2	11	126	127	1	**0.93**
					128	129	1	**1.11**
					130	131	1	**0.87**
97	APOM O95445$_{1}$: Apolipoprotein M	1/2	10	25	126	127	23	**0.80**
					128	129	23	**0.56**
					130	131	23	**1.26**
98	APP P05067$_{1,7-9,11}$: Amyloid beta A4 protein	5/11	7	7	126	127	6	**0.37**
					128	129	6	**1.20**
					130	131	7	**1.30**
99	APRT P07741$_{1,2}$: Adenine phosphoribosyltransferase	2/2	3	3	126	127	3	**0.30**
					128	129	3	**0.48**
					130	131	3	**1.04**
100	ARCN1 P48444$_{1,2}$: Coatomer subunit delta	2/2	4	4	126	127	4	**0.85**
					128	129	3	**0.44**
					130	131	4	**0.89**

#	protein	group	peptides	spectra	ch1	ch2	quant	ratio
**101**	ARHGAP1 Q07960: Rho GTPase-activating protein 1		4	4	126	127	4	**0.42**
					128	129	4	**0.34**
					130	131	4	**0.93**
**102**	ARHGAP17 Q68EM7$_{1,2,5,6}$: Rho GTPase-activating protein 17	4/4	2	4	126	127	4	**0.41**
					128	129	4	**0.22**
					130	131	4	**0.82**
**103**	ARHGDIA P52565$_{1}$: Rho GDP-dissociation inhibitor 1	1/2	7	12	126	127	12	**0.74**
					128	129	12	**0.35**
					130	131	12	**0.83**
**104**	ARHGDIB P52566: Rho GDP-dissociation inhibitor 2		5	7	126	127	7	**1.21**
					128	129	6	**0.36**
					130	131	7	**0.84**
**105**	ARHGEF1 Q92888$_{1-4}$: Rho guanine nucleotide exchange factor 1	4/4	3	3	126	127	3	**5.43**
					128	129	3	**0.72**
					130	131	3	**0.81**
**106**	ARPC5 O15511$_{1,2}$: Actin-related protein 2/3 complex subunit 5	2/2	2	2	126	127	2	**3.62**
					128	129	2	**0.87**
					130	131	2	**0.78**
**107**	ASGR2 P07307$_{1-3}$: Asialoglycoprotein receptor 2	3/3	4	6	126	127	4	**0.79**
					128	129	6	**2.26**
					130	131	6	**1.02**
**108**	ASPH Q12797$_{1,10}$: Aspartyl/asparaginyl beta-hydroxylase	2/2	6	7	126	127	7	**0.68**
					128	129	6	**0.73**
					130	131	7	**1.04**
**109**	ASPSCR1 Q9BZE9$_{1-4}$: Tether containing UBX domain for GLUT4	4/4	1	1	126	127	1	**0.50**
					128	129	1	**0.46**
					130	131	1	**0.92**
**110**	ATIC P31939$_{1,2}$: Bifunctional purine biosynthesis protein PURH [Cleaved into: Bifunctional pu . . .	2/2	4	4	126	127	3	**0.49**
					128	129	3	**0.38**
					130	131	4	**0.98**
**111**	ATL3 Q6DD88: Atlastin-3		4	4	126	127	4	**1.04**
					128	129	4	**0.34**
					130	131	4	**1.24**
**112**	ATP5A1 P25705$_{1,2}$: ATP synthase subunit alpha, mitochondrial	2/3	14	16	126	127	15	**7.25**
					128	129	15	**0.81**
					130	131	15	**0.76**

#	protein	group	peptides	spectra	ch1	ch2	quant	ratio
**113**	ATP5D P30049: ATP synthase subunit delta, mitochondrial		2	2	126	127	2	**4.07**
					128	129	2	**1.75**
					130	131	2	**0.89**
**114**	ATP5H $O75947_{1,2}$: ATP synthase subunit d, mitochondrial	2/2	2	2	126	127	2	**8.67**
					128	129	2	**1.73**
					130	131	2	**0.82**
**115**	ATP5O P48047: ATP synthase subunit O, mitochondrial		2	2	126	127	2	**9.07**
					128	129	2	**1.47**
					130	131	2	**0.72**
**116**	ATP6AP1 Q15904: V-type proton ATPase subunit S1		3	4	126	127	4	**0.71**
					128	129	4	**0.89**
					130	131	4	**1.24**
**117**	ATP6AP2 $O75787_{1,2}$: Renin receptor	2/2	5	8	126	127	7	**0.37**
					128	129	7	**0.27**
					130	131	8	**1.06**
**118**	ATP6V1B1 P15313, ATP6V1B2 P21281: V-type proton ATPase subunit B, kidney isoform, V-type proton ATPase subunit B, brain isoform	2/2	3	5	126	127	5	**1.15**
					128	129	5	**0.40**
					130	131	5	**1.14**
**119**	ATP6V1E1 $P36543_{1,3}$, ATP6V1E2 Q96A05: V-type proton ATPase subunit E 1, V-type proton ATPase subunit E 2	3/3	3	4	126	127	4	**0.93**
					128	129	4	**0.52**
					130	131	4	**0.99**
**120**	ATP6V1G1 O75348: V-type proton ATPase subunit G 1		2	3	126	127	3	**4.04**
					128	129	3	**1.51**
					130	131	3	**0.83**
**121**	ATRX $P46100_{1,4}$: Transcriptional regulator ATRX	2/5	12	50	126	127	1	**0.21**
					128	129	1	**0.40**
					130	131	1	**1.31**
**122**	ATRX $P46100_{6}$: Transcriptional regulator ATRX		11	42	126	127	1	**0.54**
					128	129	1	**1.58**
					130	131	1	**1.41**
**123**	AXL $P30530_{1,2}$: Tyrosine-protein kinase receptor UFO	2/2	2	2	126	127	2	**0.88**
					128	129	2	**1.06**
					130	131	2	**1.06**
**124**	AZGP1 P25311: Zinc-alpha-2-glycoprotein		6	10	126	127	9	**5.87**
					128	129	10	**3.08**
					130	131	10	**0.98**

#	protein	group	peptides	spectra	ch1	ch2	quant	ratio
125	AZU1 P20160: Azurocidin		2	2	126	127	2	**2.61**
					128	129	2	**0.45**
					130	131	2	**1.11**
126	B2M P61769: Beta-2-microglobulin [Cleaved into: Beta-2-microglobulin form pI 5.3]		5	17	126	127	15	**1.88**
					128	129	17	**4.17**
					130	131	14	**1.01**
127	B3GAT3 O94766$_{1,2}$: Galactosylgalactosylxylosylprotein 3-beta-glucuronosyltransferase 3	2/2	4	5	126	127	4	**0.62**
					128	129	5	**0.61**
					130	131	4	**0.77**
128	B3GNT9 Q6UX72: UDP-GlcNAc:betaGal beta-1,3-N-acetylglucosaminyltransferase 9		1	1	126	127	1	**0.13**
					128	129	1	**0.46**
					130	131	1	**0.75**
129	B4GALT1 P15291$_{1,2}$: Beta-1,4-galactosyltransferase 1	2/2	5	11	126	127	8	**0.23**
					128	129	11	**0.28**
					130	131	11	**0.65**
130	B4GALT4 O60513: Beta-1,4-galactosyltransferase 4		3	4	126	127	3	**0.39**
					128	129	4	**0.33**
					130	131	4	**0.70**
131	B4GAT1 O43505: Beta-1,4-glucuronyltransferase 1		3	4	126	127	4	**0.78**
					128	129	4	**1.78**
					130	131	4	**0.90**
132	BAX Q07812$_{1,2,8}$: Apoptosis regulator BAX	3/6	3	3	126	127	3	**5.28**
					128	129	3	**2.83**
					130	131	3	**0.67**
133	BCAP31 P51572$_{1,2}$: B-cell receptor-associated protein 31	2/2	1	1	126	127	1	**1.69**
					128	129	1	**0.23**
					130	131	1	**0.97**
134	BCHE P06276: Cholinesterase		6	11	126	127	9	**1.22**
					128	129	11	**1.36**
					130	131	8	**0.87**
135	BCL2L2 Q92843$_{2}$, PABPN1 Q86U42$_{1,2}$: Bcl-2-like protein 2, Polyadenylate-binding protein 2	3/3	2	2	126	127	2	**1.09**
					128	129	2	**0.99**
					130	131	2	**1.01**
136	BDH2 Q9BUT1$_{1}$: 3-hydroxybutyrate dehydrogenase type 2	1/2	2	2	126	127	2	**0.64**
					128	129	2	**0.45**
					130	131	2	**0.99**

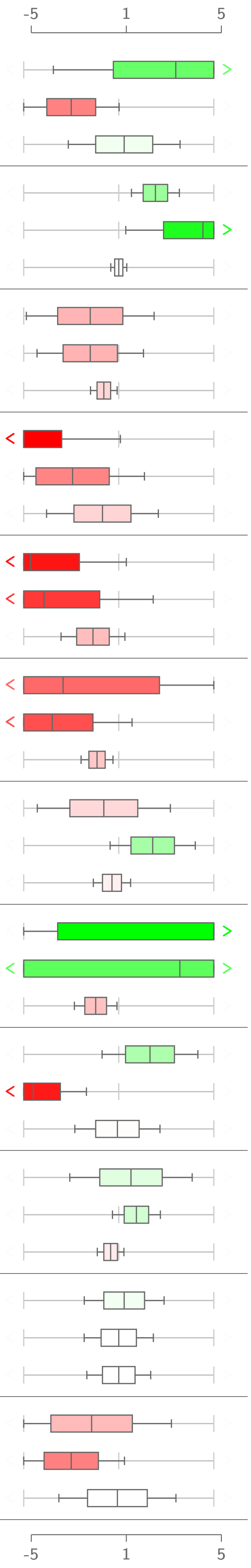

#	protein	group	peptides	spectra	ch1	ch2	quant	ratio
**137**	BGN P21810: Biglycan		5	10	126	127	3	**0.46**
					128	129	3	**0.61**
					130	131	4	**1.02**
**138**	BID P55957$_{1,2}$: BH3-interacting domain death agonist	2/2	1	1	126	127		
					128	129	1	**0.03**
					130	131	1	**0.63**
**139**	BLVRA P53004: Biliverdin reductase A		3	3	126	127	3	**0.72**
					128	129	3	**0.34**
					130	131	3	**0.96**
**140**	BLVRB P30043: Flavin reductase		5	6	126	127	5	**1.11**
					128	129	6	**0.75**
					130	131	6	**1.16**
**141**	BMP1 P13497$_1$: Bone morphogenetic protein 1	1/7	22	31	126	127	5	**1.95**
					128	129	6	**1.93**
					130	131	6	**0.90**
**142**	BMP1 P13497$_2$: Bone morphogenetic protein 1		18	25	126	127	1	**1.63**
					128	129	1	**1.37**
					130	131	1	**0.91**
**143**	BMP3 P12645: Bone morphogenetic protein 3		2	2	126	127	2	**0.37**
					128	129	2	**0.79**
					130	131	2	**0.79**
**144**	BOLA2 Q9H3K6$_{1,2}$: BolA-like protein 2	2/2	1	1	126	127	1	**2.59**
					128	129	1	**1.17**
					130	131	1	**0.92**
**145**	BPIFB1 Q8TDL5$_1$: BPI fold-containing family B member 1		4	4	126	127	3	**0.54**
					128	129	4	**0.15**
					130	131	3	**1.29**
**146**	BPTF Q12830$_{1,2,4}$: Nucleosome-remodeling factor subunit BPTF	3/3	5	16	126	127	10	**1.91**
					128	129	11	**1.41**
					130	131	13	**1.34**
**147**	BTD P43251$_{1-4}$: Biotinidase	4/4	3	9	126	127	8	**0.70**
					128	129	9	**1.34**
					130	131	9	**1.11**
**148**	C16orf89 Q6UX73$_{1,2}$: UPF0764 protein C16orf89	2/2	3	3	126	127	2	**0.19**
					128	129	2	**0.48**
					130	131	2	**0.98**

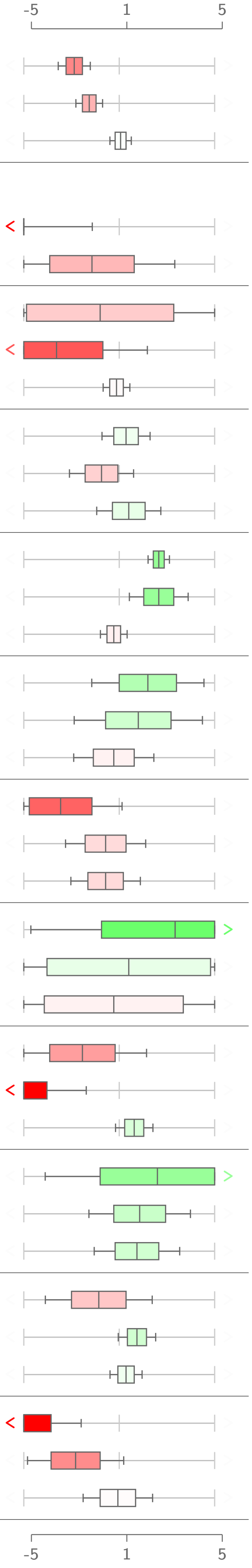

#	protein	group	peptides	spectra	ch1	ch2	quant	ratio
**149**	C1QA P02745: Complement C1q subcomponent subunit A		4	13	126	127	13	**0.42**
					128	129	13	**0.67**
					130	131	10	**0.98**
**150**	C1QB P02746: Complement C1q subcomponent subunit B		5	42	126	127	38	**1.01**
					128	129	41	**1.02**
					130	131	36	**1.06**
**151**	C1QC P02747: Complement C1q subcomponent subunit C		3	12	126	127	12	**0.57**
					128	129	12	**1.19**
					130	131	11	**1.02**
**152**	C1QTNF1 Q9BXJ1$_{1,2}$: Complement C1q tumor necrosis factor-related protein 1	2/2	2	3	126	127	3	**0.94**
					128	129	3	**1.27**
					130	131	3	**1.08**
**153**	C1R P00736: Complement C1r subcomponent		32	122	126	127	105	**0.70**
					128	129	103	**1.28**
					130	131	100	**1.01**
**154**	C1RL Q9NZP8: Complement C1r subcomponent-like protein		11	27	126	127	13	**0.42**
					128	129	14	**0.36**
					130	131	14	**0.99**
**155**	C1S P09871: Complement C1s subcomponent		26	197	126	127	134	**0.43**
					128	129	133	**1.20**
					130	131	134	**0.96**
**156**	C2 P06681$_1$: Complement C2	1/3	23	30	126	127	29	**0.48**
					128	129	30	**0.51**
					130	131	27	**0.86**
**157**	C2orf40 Q9H1Z8: Augurin		3	9	126	127	9	**1.26**
					128	129	7	**1.21**
					130	131	9	**0.84**
**158**	C3 P01024: Complement C3		116	2086	126	127	1889	**0.36**
					128	129	1953	**0.53**
					130	131	1946	**0.97**
**159**	C4A P0C0L4$_1$: Complement C4-A	1/2	86	1640	126	127	6	**2.01**
					128	129	6	**0.35**
					130	131	6	**1.19**
**160**	C4B P0C0L5: Complement C4-B		90	1768	126	127	130	**0.32**
					128	129	130	**0.43**
					130	131	128	**1.03**

#	protein	group	peptides	spectra	ch1	ch2	quant	ratio
**161**	C4BPA P04003: C4b-binding protein alpha chain		33	388	126	127	364	**0.42**
					128	129	382	**0.46**
					130	131	377	**1.15**
**162**	C4BPB P20851$_{1,2}$: C4b-binding protein beta chain	2/2	11	23	126	127	21	**0.47**
					128	129	22	**0.43**
					130	131	22	**1.19**
**163**	C5 P01031: Complement C5		55	142	126	127	133	**0.58**
					128	129	129	**0.66**
					130	131	138	**1.11**
**164**	C6 P13671: Complement component C6		40	91	126	127	71	**1.08**
					128	129	77	**0.97**
					130	131	83	**1.08**
**165**	C7 P10643: Complement component C7		32	106	126	127	95	**0.60**
					128	129	87	**1.01**
					130	131	97	**1.07**
**166**	C8A P07357: Complement component C8 alpha chain		21	56	126	127	49	**0.44**
					128	129	48	**1.38**
					130	131	52	**0.96**
**167**	C8B P07358: Complement component C8 beta chain		24	78	126	127	76	**0.44**
					128	129	77	**0.57**
					130	131	76	**0.90**
**168**	C8G P07360: Complement component C8 gamma chain		9	20	126	127	17	**0.57**
					128	129	20	**0.91**
					130	131	20	**1.01**
**169**	C9 P02748: Complement component C9 [Cleaved into: Complement component C9a; Complement ...		31	816	126	127	663	**0.22**
					128	129	758	**0.16**
					130	131	725	**0.78**
**170**	CACNA1D Q01668$_{2}$: Voltage-dependent L-type calcium channel subunit alpha-1D	1/4	4	84	126	127	70	**2.79**
					128	129	64	**1.71**
					130	131	73	**0.87**
**171**	CACYBP Q9HB71$_{1}$: Calcyclin-binding protein	1/2	3	169	126	127	2	**0.94**
					128	129	2	**0.76**
					130	131	2	**0.75**
**172**	CALM1 P62158: Calmodulin		8	27	126	127	20	**0.36**
					128	129	18	**1.05**
					130	131	14	**0.92**

#	protein	group	peptides	spectra	ch1	ch2	quant	ratio
173	CALML3 P27482: Calmodulin-like protein 3		3	8	126	127	1	**2.57**
					128	129	1	**1.70**
					130	131	1	**1.09**
174	CALR P27797: Calreticulin		18	45	126	127	38	**0.91**
					128	129	43	**0.78**
					130	131	41	**1.20**
175	CALU O43852$_{1,3}$: Calumenin	2/15	7	9	126	127	7	**0.73**
					128	129	7	**1.44**
					130	131	6	**1.17**
176	CAND1 Q86VP6$_{1}$: Cullin-associated NEDD8-dissociated protein 1	1/3	10	12	126	127	9	**0.28**
					128	129	8	**0.16**
					130	131	11	**1.04**
177	CANX P27824$_{1,2}$: Calnexin	2/3	12	14	126	127	11	**1.04**
					128	129	13	**0.61**
					130	131	13	**1.03**
178	CAP1 Q01518$_{1,2}$: Adenylyl cyclase-associated protein 1	2/2	11	15	126	127	15	**0.84**
					128	129	14	**0.32**
					130	131	15	**0.82**
179	CAPG P40121$_{1,2}$: Macrophage-capping protein	2/2	6	11	126	127	11	**0.44**
					128	129	11	**0.34**
					130	131	11	**0.84**
180	CAPN1 P07384: Calpain-1 catalytic subunit		2	2	126	127	2	**0.59**
					128	129	2	**0.41**
					130	131	2	**0.97**
181	CAPN2 P17655$_{1}$: Calpain-2 catalytic subunit	1/2	6	8	126	127	8	**2.22**
					128	129	8	**0.89**
					130	131	8	**1.21**
182	CAPNS1 P04632: Calpain small subunit 1		4	4	126	127	4	**0.45**
					128	129	3	**0.22**
					130	131	4	**1.07**
183	CAPRIN1 Q14444$_{1,2}$: Caprin-1	2/2	2	2	126	127	2	**0.74**
					128	129	2	**0.59**
					130	131	2	**1.00**
184	CAPZA1 P52907: F-actin-capping protein subunit alpha-1		5	7	126	127	5	**2.23**
					128	129	6	**1.10**
					130	131	6	**0.83**

#	protein	group	peptides	spectra	ch1	ch2	quant	ratio
**185**	CAPZA2 P47755$_1$: F-actin-capping protein subunit alpha-2	1/2	3	3	126	127	2	**1.07**
					128	129	2	**0.96**
					130	131	2	**1.09**
**186**	CAPZB P47756$_2$: F-actin-capping protein subunit beta	1/2	6	7	126	127	7	**1.23**
					128	129	6	**0.64**
					130	131	6	**1.01**
**187**	CARD11 Q9BXL7: Caspase recruitment domain-containing protein 11		3	28	126	127	24	**0.73**
					128	129	25	**0.68**
					130	131	28	**1.06**
**188**	CAST P20810$_{5-7,9,10}$: Calpastatin	5/10	5	5	126	127	5	**3.14**
					128	129	5	**0.89**
					130	131	5	**0.96**
**189**	CBR1 P16152$_1$: Carbonyl reductase [NADPH] 1		4	4	126	127	4	**0.50**
					128	129	4	**0.24**
					130	131	4	**1.03**
**190**	CCDC144A A2RUR9$_1$: Coiled-coil domain-containing protein 144A	1/2	6	6	126	127	3	**1.25**
					128	129	5	**1.65**
					130	131	6	**0.99**
**191**	CCDC80 Q76M96$_1$: Coiled-coil domain-containing protein 80	1/2	22	44	126	127	42	**0.74**
					128	129	42	**0.80**
					130	131	37	**0.94**
**192**	CCL14 Q16627$_1$: C-C motif chemokine 14	1/2	4	4	126	127	4	**2.17**
					128	129	4	**3.07**
					130	131	4	**1.06**
**193**	CCL18 P55774: C-C motif chemokine 18		4	6	126	127	5	**2.07**
					128	129	6	**9.36**
					130	131	6	**0.43**
**194**	CCS O14618: Copper chaperone for superoxide dismutase		2	2	126	127	2	**1.22**
					128	129	2	**1.29**
					130	131	2	**1.16**
**195**	CCT2 P78371$_1$: T-complex protein 1 subunit beta	1/2	11	12	126	127	12	**1.16**
					128	129	11	**0.36**
					130	131	12	**1.03**
**196**	CCT3 P49368$_1$: T-complex protein 1 subunit gamma	1/2	10	10	126	127	10	**0.67**
					128	129	9	**0.20**
					130	131	10	**1.00**

#	protein	group	peptides	spectra	ch1	ch2	quant	ratio
**197**	CCT4 P50991$_1$: T-complex protein 1 subunit delta	1/2	4	4	126	127	4	**0.96**
					128	129	4	**0.32**
					130	131	3	**0.71**
**198**	CCT5 P48643$_{1,2}$: T-complex protein 1 subunit epsilon	2/2	7	9	126	127	9	**2.17**
					128	129	9	**0.90**
					130	131	9	**0.98**
**199**	CCT6A P40227$_{1,2}$: T-complex protein 1 subunit zeta	2/5	7	7	126	127	7	**0.91**
					128	129	6	**0.33**
					130	131	7	**1.08**
**200**	CCT7 Q99832$_3$: T-complex protein 1 subunit eta	1/4	8	9	126	127	9	**1.15**
					128	129	7	**0.31**
					130	131	9	**1.03**
**201**	CCT8 P50990$_{1,2}$: T-complex protein 1 subunit theta	2/3	7	7	126	127	5	**0.86**
					128	129	7	**0.38**
					130	131	6	**1.05**
**202**	CD109 Q6YHK3$_{1,2,4}$: CD109 antigen	3/3	5	6	126	127	6	**0.58**
					128	129	6	**1.07**
					130	131	6	**0.88**
**203**	CD14 P08571: Monocyte differentiation antigen CD14		14	120	126	127	115	**0.52**
					128	129	118	**0.33**
					130	131	116	**0.76**
**204**	CD163 Q86VB7$_{1-3}$: Scavenger receptor cysteine-rich type 1 protein M130	3/4	10	10	126	127	9	**1.57**
					128	129	9	**2.82**
					130	131	10	**0.96**
**205**	CD163L1 Q9NR16$_{1-4}$: Scavenger receptor cysteine-rich type 1 protein M160	4/4	2	2	126	127	2	**1.14**
					128	129	2	**1.39**
					130	131	2	**1.04**
**206**	CD248 Q9HCU0$_1$: Endosialin		8	11	126	127	11	**0.72**
					128	129	11	**1.35**
					130	131	9	**1.14**
**207**	CD5L O43866: CD5 antigen-like		17	41	126	127	36	**0.76**
					128	129	38	**1.08**
					130	131	39	**1.06**
**208**	CD63 P08962$_{1-3}$: CD63 antigen	3/3	2	3	126	127	3	**1.04**
					128	129	3	**0.21**
					130	131	3	**0.83**

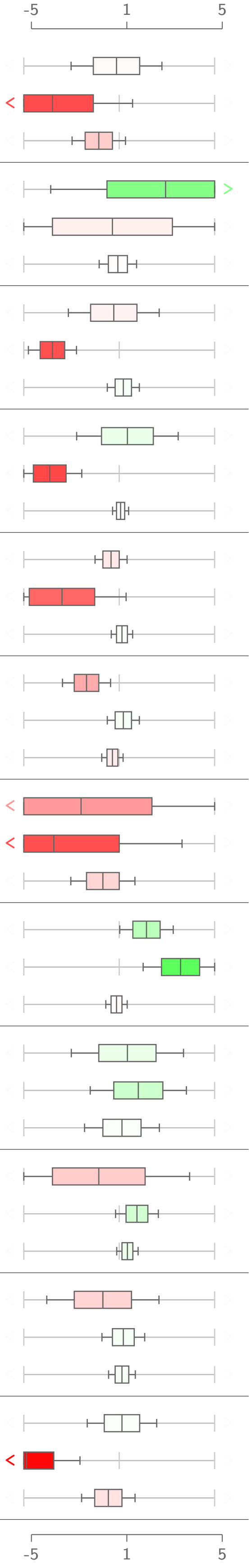

#	protein	group	peptides	spectra	ch1	ch2	quant	ratio
209	CD97 P48960$_{1-3}$: CD97 antigen	3/3	4	4	126	127	4	0.93
					128	129	3	0.81
					130	131	3	0.98
210	CD99 P14209$_{1,3}$: CD99 antigen	2/2	2	2	126	127	2	0.81
					128	129	2	0.23
					130	131	2	1.12
211	CDC37 Q16543: Hsp90 co-chaperone Cdc37		4	4	126	127	4	0.54
					128	129	4	1.11
					130	131	3	1.00
212	CDC42 P60953$_{2}$: Cell division control protein 42 homolog	1/2	3	6	126	127	5	0.84
					128	129	5	0.43
					130	131	4	1.05
213	CDH1 P12830$_{1,2}$: Cadherin-1	2/2	3	7	126	127	6	1.31
					128	129	7	1.07
					130	131	7	0.82
214	CDH2 P19022$_{1}$: Cadherin-2	1/2	2	4	126	127	3	0.81
					128	129	4	1.35
					130	131	3	0.97
215	CDK12 Q9NYV4$_{1-3}$, PHF20L1 A8MW92$_{1,2,4}$: Cyclin-dependent kinase 12, PHD finger protein 20-like protein 1	6/6	1	1	126	127	1	1.59
					128	129	1	2.83
					130	131	1	0.39
216	CELF1 Q92879$_{1-6}$, CELF2 O95319$_{1-5}$: CUGBP Elav-like family member 1, CUGBP Elav-like family member 2	11/11	2	2	126	127	2	0.84
					128	129	2	0.30
					130	131	2	0.96
217	CETP P11597$_{1}$: Cholesteryl ester transfer protein	1/2	12	25	126	127	20	0.18
					128	129	25	0.20
					130	131	23	1.09
218	CFB P00751$_{1}$: Complement factor B	1/2	22	31	126	127	29	1.05
					128	129	25	1.58
					130	131	30	0.93
219	CFD P00746: Complement factor D		15	89	126	127	87	1.06
					128	129	88	0.71
					130	131	88	1.01
220	CFH P08603$_{1}$: Complement factor H	1/2	65	747	126	127	552	0.86
					128	129	557	1.56
					130	131	535	1.06

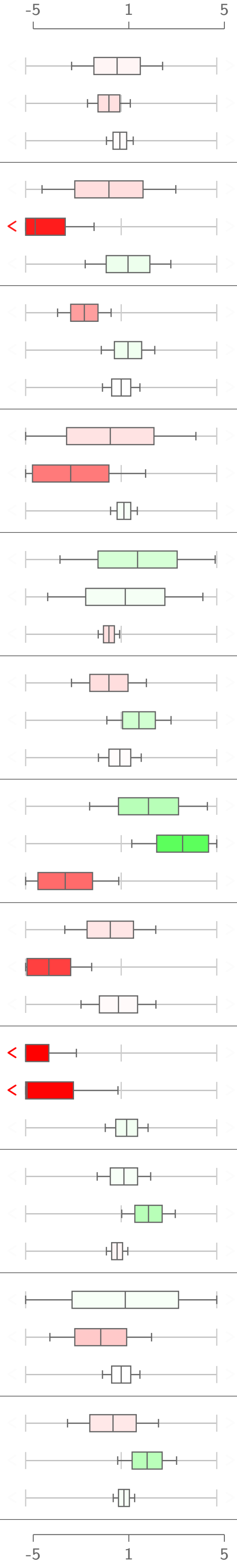

#	protein	group	peptides	spectra	ch1	ch2	quant	ratio
**221**	CFHR1 Q03591: Complement factor H-related protein 1		12	286	126	127	36	**0.74**
					128	129	36	**1.35**
					130	131	36	**1.15**
**222**	CFHR2 P36980$_1$: Complement factor H-related protein 2	1/2	12	86	126	127	12	**1.52**
					128	129	16	**2.97**
					130	131	16	**1.03**
**223**	CFHR3 Q02985$_{1,2}$: Complement factor H-related protein 3	2/2	10	60	126	127	26	**1.08**
					128	129	27	**1.84**
					130	131	25	**1.00**
**224**	CFHR4 Q92496$_{1,2}$: Complement factor H-related protein 4	2/3	10	36	126	127	7	**1.47**
					128	129	7	**0.96**
					130	131	7	**1.10**
**225**	CFHR5 Q9BXR6: Complement factor H-related protein 5		22	91	126	127	54	**0.66**
					128	129	53	**0.79**
					130	131	54	**1.00**
**226**	CFI P05156: Complement factor I		20	61	126	127	52	**1.32**
					128	129	57	**2.95**
					130	131	42	**0.94**
**227**	CFL1 P23528: Cofilin-1		6	7	126	127	6	**3.09**
					128	129	7	**1.17**
					130	131	7	**0.88**
**228**	CFP P27918: Properdin		14	38	126	127	32	**1.28**
					128	129	33	**1.00**
					130	131	32	**1.18**
**229**	CGREF1 Q99674$_{1,4,5}$: Cell growth regulator with EF hand domain protein 1	3/3	8	16	126	127	13	**0.39**
					128	129	15	**2.73**
					130	131	15	**0.96**
**230**	CHD3 Q12873$_{1-3}$: Chromodomain-helicase-DNA-binding protein 3	3/3	3	7	126	127	5	**1.49**
					128	129	6	**1.42**
					130	131	6	**1.01**
**231**	CHGA P10645: Chromogranin-A		26	117	126	127	111	**0.73**
					128	129	109	**1.54**
					130	131	112	**1.03**
**232**	CHGB P05060: Secretogranin-1		24	61	126	127	47	**0.39**
					128	129	50	**1.79**
					130	131	57	**1.04**

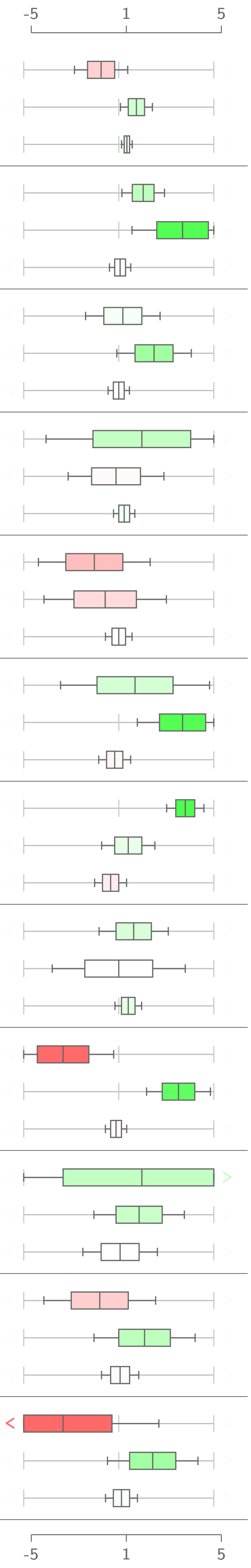

#	protein	group	peptides	spectra	ch1	ch2	quant	ratio
**233**	CHMP4A Q9BY43$_{1,2}$: Charged multivesicular body protein 4a	2/2	2	3	126	127	1	**0.22**
					128	129	1	**0.48**
					130	131	1	**1.22**
**234**	CHMP4B Q9H444: Charged multivesicular body protein 4b		3	13	126	127	13	**1.73**
					128	129	13	**1.34**
					130	131	13	**0.97**
**235**	CHRD Q9H2X0$_{1,5}$: Chordin	2/3	5	6	126	127	6	**1.78**
					128	129	6	**2.28**
					130	131	6	**0.98**
**236**	CHRDL1 Q9BU40$_{2,4}$: Chordin-like protein 1	2/6	16	35	126	127	32	**0.60**
					128	129	35	**0.91**
					130	131	35	**0.99**
**237**	CHRDL2 Q6WN34$_{1}$: Chordin-like protein 2		8	13	126	127	2	**0.38**
					128	129	2	**0.51**
					130	131	2	**0.66**
**238**	CHRDL2 Q6WN34$_{2}$: Chordin-like protein 2		8	13	126	127	2	**0.92**
					128	129	2	**0.93**
					130	131	2	**0.76**
**239**	CHST12 Q9NRB3: Carbohydrate sulfotransferase 12		1	1	126	127	1	**1.26**
					128	129	1	**1.02**
					130	131	1	**1.38**
**240**	CHST14 Q8NCH0: Carbohydrate sulfotransferase 14		5	7	126	127	7	**0.43**
					128	129	7	**0.55**
					130	131	6	**1.00**
**241**	CILP O75339: Cartilage intermediate layer protein 1		49	155	126	127	146	**0.99**
					128	129	145	**1.62**
					130	131	143	**0.89**
**242**	CILP2 Q8IUL8: Cartilage intermediate layer protein 2		6	8	126	127	5	**0.89**
					128	129	4	**0.94**
					130	131	5	**0.87**
**243**	CKAP4 Q07065: Cytoskeleton-associated protein 4		6	7	126	127	4	**4.71**
					128	129	4	**0.71**
					130	131	5	**0.80**
**244**	CKB P12277: Creatine kinase B-type		6	6	126	127	6	**1.16**
					128	129	5	**0.85**
					130	131	6	**1.22**

#	protein	group	peptides	spectra	ch1	ch2	quant	ratio
**245**	CLC Q05315: Galectin-10		3	4	126	127	4	**0.50**
					128	129	4	**0.50**
					130	131	4	**0.73**
**246**	CLEC11A Q9Y240: C-type lectin domain family 11 member A		15	36	126	127	34	**0.49**
					128	129	36	**0.63**
					130	131	35	**1.21**
**247**	CLEC3B P05452: Tetranectin		10	17	126	127	17	**0.71**
					128	129	15	**2.25**
					130	131	15	**0.94**
**248**	CLIC1 O00299: Chloride intracellular channel protein 1		6	8	126	127	8	**1.01**
					128	129	8	**0.76**
					130	131	6	**1.03**
**249**	CLIC4 Q9Y696: Chloride intracellular channel protein 4		5	6	126	127	6	**1.96**
					128	129	6	**0.73**
					130	131	5	**1.01**
**250**	CLSTN1 O94985$_1$: Calsyntenin-1	1/2	15	18	126	127	17	**0.48**
					128	129	17	**2.49**
					130	131	13	**1.03**
**251**	CLTA P09496$_{1-4}$: Clathrin light chain A	4/4	2	2	126	127	2	**1.77**
					128	129	2	**1.30**
					130	131	2	**1.02**
**252**	CLTB P09497$_{1,2}$: Clathrin light chain B	2/2	2	2	126	127	2	**1.86**
					128	129	2	**1.81**
					130	131	2	**1.24**
**253**	CLTC Q00610$_{1,2}$: Clathrin heavy chain 1	2/3	18	20	126	127	14	**0.84**
					128	129	15	**0.33**
					130	131	12	**1.09**
**254**	CLTCL1 P53675$_1$: Clathrin heavy chain 2		5	6	126	127	1	**8.21**
					128	129	1	**1.88**
					130	131	1	**0.50**
**255**	CLU P10909$_4$: Clusterin	1/5	27	722	126	127	644	**0.53**
					128	129	684	**0.54**
					130	131	669	**1.17**
**256**	CNBP P62633$_{1-3,7}$: Cellular nucleic acid-binding protein	4/6	4	6	126	127	1	**1.23**
					128	129	1	**1.29**
					130	131	2	**1.33**

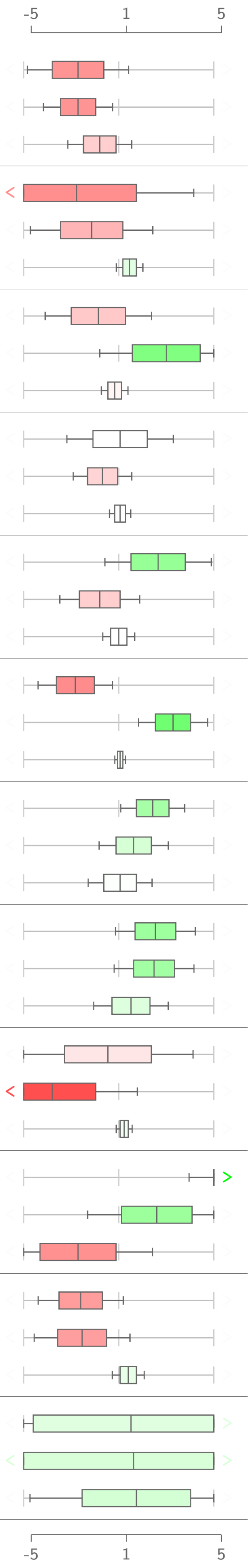

#	protein	group	peptides	spectra	ch1	ch2	quant	ratio
**257**	CNBP P62633$_{5,6}$: Cellular nucleic acid-binding protein	2/2	4	9	126	127	3	**2.31**
					128	129	3	**2.72**
					130	131	2	**1.01**
**258**	CNDP1 Q96KN2: Beta-Ala-His dipeptidase		3	3	126	127	3	**0.69**
					128	129	3	**1.15**
					130	131	3	**0.97**
**259**	CNN3 Q15417$_{1,3}$: Calponin-3	2/3	5	6	126	127	6	**1.04**
					128	129	6	**0.48**
					130	131	6	**0.87**
**260**	CNPY2 Q9Y2B0$_{1}$: Protein canopy homolog 2		3	3	126	127	3	**0.88**
					128	129	3	**2.21**
					130	131	3	**1.06**
**261**	COL12A1 Q99715$_{1}$: Collagen alpha-1(XII) chain	1/3	44	61	126	127	56	**0.31**
					128	129	57	**0.40**
					130	131	53	**0.92**
**262**	COL14A1 Q05707$_{1,2}$: Collagen alpha-1(XIV) chain	2/3	40	56	126	127	54	**0.41**
					128	129	51	**0.46**
					130	131	54	**0.96**
**263**	COL15A1 P39059: Collagen alpha-1(XV) chain [Cleaved into: Restin		2	2	126	127	2	**0.74**
					128	129	2	**1.81**
					130	131	2	**1.01**
**264**	COL16A1 Q07092$_{1,2}$: Collagen alpha-1(XVI) chain	2/2	5	6	126	127	5	**0.55**
					128	129	6	**1.10**
					130	131	6	**0.96**
**265**	COL18A1 P39060$_{1-3}$: Collagen alpha-1(XVIII) chain [Cleaved into: Endostatin]	3/3	8	15	126	127	12	**0.78**
					128	129	14	**1.47**
					130	131	13	**0.97**
**266**	COL1A1 P02452: Collagen alpha-1(I) chain		18	87	126	127	79	**0.35**
					128	129	77	**3.44**
					130	131	84	**1.05**
**267**	COL1A2 P08123: Collagen alpha-2(I) chain		15	27	126	127	24	**1.02**
					128	129	23	**2.78**
					130	131	26	**0.88**
**268**	COL3A1 P02461$_{1}$: Collagen alpha-1(III) chain	1/2	16	36	126	127	32	**0.51**
					128	129	31	**0.75**
					130	131	32	**0.92**

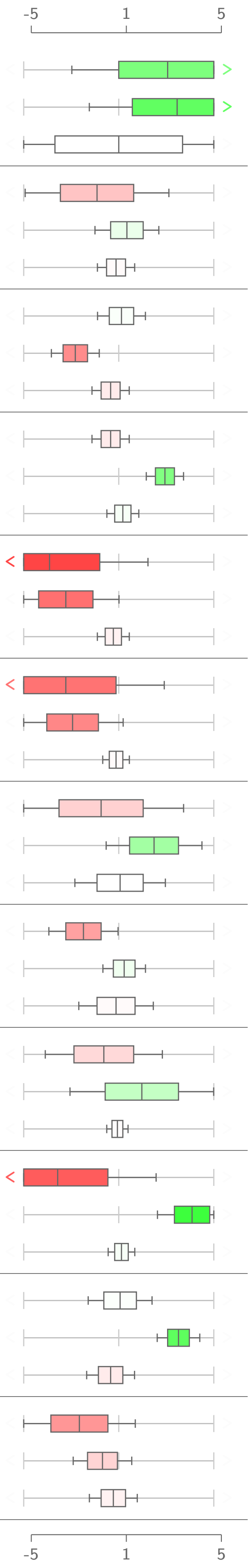

#	protein	group	peptides	spectra	ch1	ch2	quant	ratio
**269**	COL4A1 P02462$_{1,2}$: Collagen alpha-1(IV) chain [Cleaved into: Arresten]	2/2	1	1	126	127	1	**0.56**
					128	129	1	**1.08**
					130	131	1	**0.80**
**270**	COL4A2 P08572: Collagen alpha-2(IV) chain [Cleaved into: Canstatin]		2	2	126	127	2	**1.77**
					128	129	2	**1.24**
					130	131	2	**0.87**
**271**	COL5A1 P20908: Collagen alpha-1(V) chain		9	19	126	127	18	**0.42**
					128	129	16	**2.05**
					130	131	18	**1.12**
**272**	COL5A2 P05997: Collagen alpha-2(V) chain		15	26	126	127	22	**0.40**
					128	129	22	**1.63**
					130	131	21	**1.03**
**273**	COL6A1 P12109: Collagen alpha-1(VI) chain		22	49	126	127	42	**0.55**
					128	129	47	**0.36**
					130	131	45	**0.91**
**274**	COL6A2 P12110$_{1}$: Collagen alpha-2(VI) chain	1/3	20	160	126	127	137	**0.61**
					128	129	128	**1.31**
					130	131	147	**0.91**
**275**	COL6A3 P12111$_{1}$: Collagen alpha-3(VI) chain	1/4	98	252	126	127	12	**0.19**
					128	129	16	**0.21**
					130	131	16	**0.97**
**276**	COL6A3 P12111$_{4}$: Collagen alpha-3(VI) chain		88	246	126	127	9	**0.16**
					128	129	8	**0.08**
					130	131	10	**0.67**
**277**	COL8A1 P27658: Collagen alpha-1(VIII) chain		3	3	126	127	2	**0.73**
					128	129	2	**0.71**
					130	131	2	**1.04**
**278**	COLEC10 Q9Y6Z7: Collectin-10		3	3	126	127	3	**0.85**
					128	129	3	**1.04**
					130	131	3	**1.08**
**279**	COLEC11 Q9BWP8$_{1-10}$: Collectin-11	10/10	2	2	126	127	2	**0.75**
					128	129	2	**0.73**
					130	131	2	**1.19**
**280**	COLGALT1 Q8NBJ5: Procollagen galactosyltransferase 1		3	3	126	127	3	**1.15**
					128	129	3	**0.93**
					130	131	3	**0.83**

#	protein	group	peptides	spectra	ch1	ch2	quant	ratio
**281**	COMP P49747$_1$: Cartilage oligomeric matrix protein	1/2	24	80	126	127	67	**0.40**
					128	129	72	**0.97**
					130	131	71	**0.91**
**282**	COPA P53621$_{1,2}$: Coatomer subunit alpha	2/2	6	7	126	127	5	**0.70**
					128	129	6	**0.58**
					130	131	7	**1.11**
**283**	COPB1 P53618: Coatomer subunit beta		9	10	126	127	6	**0.44**
					128	129	10	**0.21**
					130	131	9	**1.05**
**284**	COPG1 Q9Y678: Coatomer subunit gamma-1		6	6	126	127	5	**0.27**
					128	129	5	**0.21**
					130	131	4	**1.04**
**285**	CORO1A P31146: Coronin-1A		12	17	126	127	17	**1.17**
					128	129	17	**0.49**
					130	131	17	**0.93**
**286**	CORO1B Q9BR76: Coronin-1B		3	7	126	127	6	**0.81**
					128	129	6	**0.69**
					130	131	7	**0.83**
**287**	CORO1C Q9ULV4$_{1-3}$: Coronin-1C	3/3	4	5	126	127	3	**0.84**
					128	129	5	**0.73**
					130	131	5	**0.85**
**288**	COTL1 Q14019: Coactosin-like protein		3	4	126	127	4	**2.33**
					128	129	4	**0.72**
					130	131	4	**0.91**
**289**	CP P00450: Ceruloplasmin		29	72	126	127	71	**0.54**
					128	129	67	**2.97**
					130	131	64	**1.16**
**290**	CPB2 Q96IY4$_1$: Carboxypeptidase B2	1/2	7	9	126	127	9	**0.61**
					128	129	9	**0.49**
					130	131	8	**1.06**
**291**	CPE P16870$_{1,2}$: Carboxypeptidase E	2/2	19	47	126	127	42	**0.26**
					128	129	42	**0.79**
					130	131	47	**0.93**
**292**	CPN1 P15169: Carboxypeptidase N catalytic chain		11	21	126	127	19	**1.25**
					128	129	19	**2.19**
					130	131	21	**0.95**

#	protein	group	peptides	spectra	ch1	ch2	quant	ratio
**293**	CPN2 P22792: Carboxypeptidase N subunit 2		12	28	126	127	28	**0.48**
					128	129	27	**0.35**
					130	131	28	**1.20**
**294**	CPQ Q9Y646: Carboxypeptidase Q		3	3	126	127	3	**0.26**
					128	129	3	**0.20**
					130	131	3	**0.88**
**295**	CPVL Q9H3G5: Probable serine carboxypeptidase CPVL		3	3	126	127	3	**0.69**
					128	129	3	**0.47**
					130	131	3	**0.90**
**296**	CPXM1 Q96SM3: Probable carboxypeptidase X1		9	13	126	127	12	**0.68**
					128	129	11	**0.85**
					130	131	11	**0.96**
**297**	CPXM2 Q8N436: Inactive carboxypeptidase-like protein X2		13	16	126	127	14	**1.09**
					128	129	15	**1.13**
					130	131	14	**1.01**
**298**	CPZ Q66K79$_{1-3}$: Carboxypeptidase Z	3/3	6	7	126	127	6	**0.87**
					128	129	6	**0.77**
					130	131	6	**1.20**
**299**	CRABP2 P29373: Cellular retinoic acid-binding protein 2		4	6	126	127	6	**0.78**
					128	129	6	**0.63**
					130	131	5	**0.97**
**300**	CRHBP P24387: Corticotropin-releasing factor-binding protein		2	2	126	127	2	**0.82**
					128	129	2	**1.13**
					130	131	2	**0.99**
**301**	CRIP1 P50238: Cysteine-rich protein 1		2	2	126	127	1	**3.67**
					128	129	1	**3.83**
					130	131	1	**1.83**
**302**	CRIP2 P52943$_{1,2}$: Cysteine-rich protein 2	2/2	2	2	126	127	1	**1.09**
					128	129	1	**1.28**
					130	131	1	**0.69**
**303**	CRISPLD2 Q9H0B8$_{1,2}$: Cysteine-rich secretory protein LCCL domain-containing 2	2/2	2	2	126	127	2	**1.38**
					128	129	2	**1.91**
					130	131	2	**0.94**
**304**	CRP P02741$_1$: C-reactive protein [Cleaved into: C-reactive protein(1-205)]	1/2	8	61	126	127	60	**0.33**
					128	129	59	**1.19**
					130	131	58	**0.74**

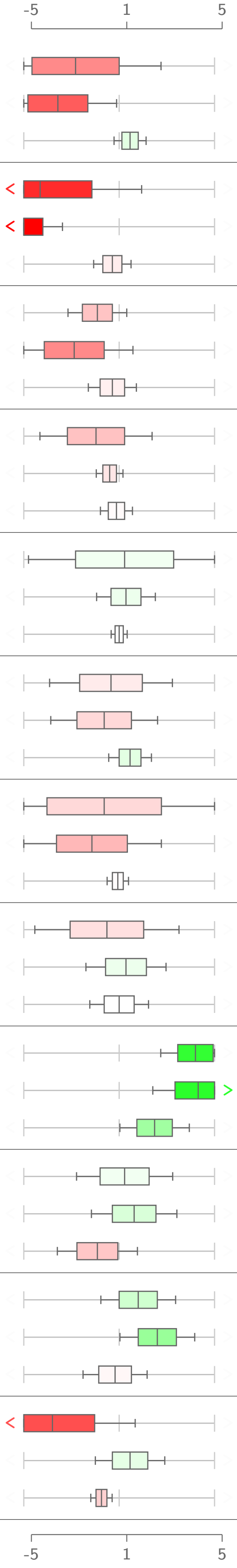

#	protein	group	peptides	spectra	ch1	ch2	quant	ratio
**305**	CRTAC1 Q9NQ79$_{1-3}$: Cartilage acidic protein 1	3/3	4	4	126	127	4	**0.90**
					128	129	4	**0.99**
					130	131	4	**1.07**
**306**	CRTAP O75718: Cartilage-associated protein		3	3	126	127	3	**1.24**
					128	129	3	**0.61**
					130	131	3	**1.12**
**307**	CRYAB P02511: Alpha-crystallin B chain		3	5	126	127	4	**2.64**
					128	129	5	**1.64**
					130	131	4	**1.07**
**308**	CRYL1 Q9Y2S2$_{1,2}$: Lambda-crystallin homolog	2/2	1	1	126	127	1	**0.63**
					128	129	1	**0.63**
					130	131	1	**0.91**
**309**	CSE1L P55060$_{1,3}$: Exportin-2	2/3	4	6	126	127	4	**0.35**
					128	129	3	**0.22**
					130	131	5	**0.99**
**310**	CSPG4 Q6UVK1: Chondroitin sulfate proteoglycan 4		5	5	126	127	4	**0.68**
					128	129	5	**0.76**
					130	131	3	**0.84**
**311**	CST3 P01034: Cystatin-C		6	40	126	127	36	**0.98**
					128	129	35	**1.96**
					130	131	36	**1.00**
**312**	CST6 Q15828: Cystatin-M		5	6	126	127	6	**1.16**
					128	129	5	**1.80**
					130	131	6	**1.10**
**313**	CSTB P04080: Cystatin-B		2	2	126	127	2	**1.76**
					128	129	2	**0.60**
					130	131	2	**1.14**
**314**	CTGF P29279$_{1,2}$: Connective tissue growth factor	2/2	9	17	126	127	16	**1.83**
					128	129	16	**1.55**
					130	131	15	**0.97**
**315**	CTHRC1 Q96CG8$_{1-3}$: Collagen triple helix repeat-containing protein 1	3/3	2	2	126	127	2	**0.39**
					128	129	2	**0.97**
					130	131	2	**0.75**
**316**	CTNNA1 P35221$_{1,2}$: Catenin alpha-1	2/9	6	6	126	127	5	**1.42**
					128	129	6	**0.67**
					130	131	6	**1.06**

#	protein	group	peptides	spectra	ch1	ch2	quant	ratio
317	CTSB P07858: Cathepsin B		5	12	126	127	10	**0.62**
					128	129	11	**1.11**
					130	131	12	**0.96**
318	CTSD P07339: Cathepsin D		5	8	126	127	8	**1.06**
					128	129	8	**0.92**
					130	131	8	**0.84**
319	CTSF Q9UBX1: Cathepsin F		7	7	126	127	7	**0.44**
					128	129	7	**0.69**
					130	131	7	**0.94**
320	CTSH P09668: Pro-cathepsin H [Cleaved into: Cathepsin H mini chain; Cathepsin H		2	2	126	127	2	**1.21**
					128	129	2	**0.90**
					130	131	2	**1.11**
321	CTSK P43235: Cathepsin K		3	4	126	127	4	**1.16**
					128	129	4	**1.70**
					130	131	3	**1.01**
322	CTSS P25774$_{1,2}$: Cathepsin S	2/2	1	1	126	127	1	**1.56**
					128	129	1	**0.79**
					130	131	1	**0.86**
323	CTSZ Q9UBR2: Cathepsin Z		3	3	126	127	3	**1.57**
					128	129	3	**1.30**
					130	131	3	**0.91**
324	CXCL10 P02778: C-X-C motif chemokine 10		2	2	126	127	2	**2.57**
					128	129	2	**2.09**
					130	131	2	**1.03**
325	CXCL12 P48061$_{1-6}$: Stromal cell-derived factor 1	6/6	5	34	126	127	34	**1.43**
					128	129	29	**1.34**
					130	131	33	**0.64**
326	CYB5B O43169: Cytochrome b5 type B		1	2	126	127	2	**1.94**
					128	129	2	**0.59**
					130	131	2	**0.84**
327	CYFIP1 Q7L576$_{1}$: Cytoplasmic FMR1-interacting protein 1	1/3	2	3	126	127	1	**0.42**
					128	129	1	**0.37**
					130	131	3	**0.89**
328	CYTL1 Q9NRR1: Cytokine-like protein 1		3	4	126	127	4	**1.50**
					128	129	4	**2.78**
					130	131	4	**0.94**

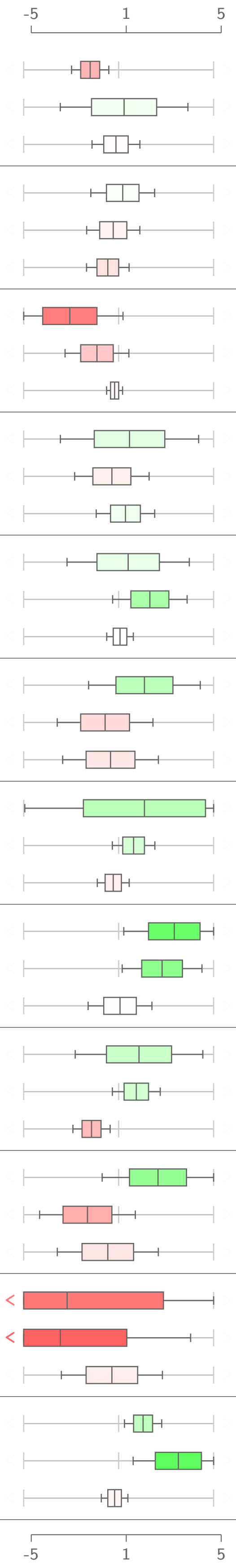

#	protein	group	peptides	spectra	ch1	ch2	quant	ratio
**329**	DAG1 Q14118: Dystroglycan		5	6	126	127	6	**0.80**
					128	129	5	**1.80**
					130	131	5	**1.08**
**330**	DARS $P14868_1$: Aspartate--tRNA ligase, cytoplasmic	1/2	4	4	126	127	4	**0.92**
					128	129	3	**0.64**
					130	131	4	**0.96**
**331**	DBNL $Q9UJU6_{1,5}$: Drebrin-like protein	2/5	4	7	126	127	7	**0.89**
					128	129	7	**0.70**
					130	131	7	**0.80**
**332**	DCD $P81605_1$: Dermcidin	1/2	6	13	126	127	11	**3.39**
					128	129	13	**1.33**
					130	131	11	**2.65**
**333**	DCN $P07585_1$: Decorin	1/5	12	29	126	127	23	**0.36**
					128	129	22	**0.59**
					130	131	23	**1.02**
**334**	DCTN1 $Q14203_{1,3,4,6}$: Dynactin subunit 1	4/4	3	3	126	127	2	**0.57**
					128	129	2	**0.47**
					130	131	2	**0.90**
**335**	DCTN3 $O75935_{1,2}$: Dynactin subunit 3	2/2	2	3	126	127	3	**1.07**
					128	129	3	**0.97**
					130	131	3	**0.73**
**336**	DDX1 $Q92499_1$: ATP-dependent RNA helicase DDX1	1/3	5	10	126	127	3	**0.83**
					128	129	3	**0.29**
					130	131	4	**1.07**
**337**	DDX5 $P17844_{1,2}$: Probable ATP-dependent RNA helicase DDX5	2/2	6	6	126	127	6	**1.02**
					128	129	6	**0.37**
					130	131	6	**1.05**
**338**	DEFA1 P59665: Neutrophil defensin 1		6	10	126	127	3	**4.03**
					128	129	3	**1.19**
					130	131	3	**0.68**
**339**	DEFA3 P59666: Neutrophil defensin 3		7	15	126	127	7	**1.54**
					128	129	7	**1.20**
					130	131	7	**0.92**
**340**	DEFA4 P12838: Neutrophil defensin 4		1	1	126	127	1	**1.28**
					128	129	1	**4.44**
					130	131	1	**0.80**

#	protein	group	peptides	spectra	ch1	ch2	quant	ratio
341	DEK P35659$_1$: Protein DEK		2	11	126	127	2	**0.50**
					128	129	3	**0.45**
					130	131	2	**1.39**
342	DES P17661: Desmin		14	35	126	127	10	**1.81**
					128	129	10	**1.20**
					130	131	10	**0.99**
343	DFNA5 O60443$_1$: Non-syndromic hearing impairment protein 5	1/2	5	5	126	127	5	**0.47**
					128	129	4	**0.31**
					130	131	5	**1.38**
344	DGKI O75912: Diacylglycerol kinase iota		2	3	126	127		
					128	129	1	**0.86**
					130	131		
345	DHX9 Q08211$_1$: ATP-dependent RNA helicase A		4	4	126	127	4	**0.91**
					128	129	3	**0.28**
					130	131	3	**1.05**
346	DIAPH1 O60610$_{1-3}$: Protein diaphanous homolog 1	3/3	4	4	126	127	3	**0.21**
					128	129	3	**0.25**
					130	131	3	**0.91**
347	DKK3 Q9UBP4: Dickkopf-related protein 3		10	75	126	127	60	**0.56**
					128	129	60	**0.96**
					130	131	69	**1.20**
348	DLD P09622$_1$: Dihydrolipoyl dehydrogenase, mitochondrial	1/2	3	3	126	127	3	**9.33**
					128	129	3	**1.53**
					130	131	3	**0.85**
349	DMKN Q6E0U4$_{2,5,6,16}$: Dermokine	4/8	7	9	126	127	7	**0.56**
					128	129	9	**1.74**
					130	131	9	**0.92**
350	DNAJA2 O60884: DnaJ homolog subfamily A member 2		2	2	126	127	2	**1.44**
					128	129	2	**0.67**
					130	131	2	**1.26**
351	DNAJB1 P25685$_1$: DnaJ homolog subfamily B member 1		2	2	126	127	2	**1.38**
					128	129	2	**0.90**
					130	131	2	**1.03**
352	DNAJB11 Q9UBS4: DnaJ homolog subfamily B member 11		6	8	126	127	7	**1.48**
					128	129	8	**0.83**
					130	131	8	**0.91**

#	protein	group	peptides	spectra	ch1	ch2	quant	ratio
**353**	DNAJC3 Q13217: DnaJ homolog subfamily C member 3		13	15	126	127	13	**0.82**
					128	129	14	**0.92**
					130	131	12	**0.89**
**354**	DNM2 $P50570_{1-5}$: Dynamin-2	5/14	8	8	126	127	8	**1.06**
					128	129	8	**0.41**
					130	131	8	**0.90**
**355**	DOCK11 Q5JSL3: Dedicator of cytokinesis protein 11		3	3	126	127	3	**0.68**
					128	129	3	**0.23**
					130	131	3	**0.98**
**356**	DOK2 O60496: Docking protein 2		1	1	126	127	1	**1.24**
					128	129	1	**0.52**
					130	131	1	**1.03**
**357**	DPP4 P27487: Dipeptidyl peptidase 4		2	2	126	127	2	**2.20**
					128	129	2	**1.05**
					130	131	2	**0.95**
**358**	DPT Q07507: Dermatopontin		10	221	126	127	179	**0.42**
					128	129	182	**0.70**
					130	131	199	**1.13**
**359**	DPYSL2 $Q16555_{1,2}$: Dihydropyrimidinase-related protein 2	2/2	3	3	126	127	2	**1.67**
					128	129	2	**0.92**
					130	131	2	**1.00**
**360**	DPYSL3 $Q14195_{2}$: Dihydropyrimidinase-related protein 3	1/2	8	10	126	127	8	**0.73**
					128	129	8	**0.59**
					130	131	8	**1.22**
**361**	DSC3 $Q14574_{1,2}$: Desmocollin-3	2/2	3	3	126	127	3	**1.58**
					128	129	3	**3.21**
					130	131	3	**0.96**
**362**	DSP $P15924_{1,3}$: Desmoplakin	2/3	12	12	126	127	11	**3.98**
					128	129	11	**1.08**
					130	131	12	**2.76**
**363**	DSTN $P60981_{1,2}$: Destrin	2/2	4	5	126	127	5	**1.59**
					128	129	5	**1.79**
					130	131	5	**1.01**
**364**	DUSP23 Q9BVJ7: Dual specificity protein phosphatase 23		2	2	126	127	2	**1.15**
					128	129	2	**1.00**
					130	131	2	**1.10**

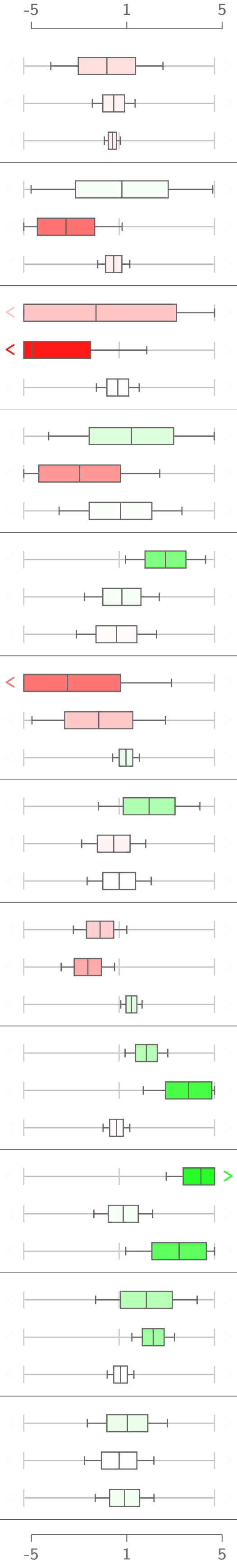

#	protein	group	peptides	spectra	ch1	ch2	quant	ratio
**365**	DYNC1H1 Q14204: Cytoplasmic dynein 1 heavy chain 1		17	19	126	127	16	**0.54**
					128	129	16	**0.39**
					130	131	17	**0.98**
**366**	DYNC1I2 Q13409$_{1-3,5-7}$: Cytoplasmic dynein 1 intermediate chain 2	6/6	1	1	126	127	1	**0.68**
					128	129	1	**0.76**
					130	131	1	**1.08**
**367**	ECM1 Q16610$_{1,4}$: Extracellular matrix protein 1	2/4	21	31	126	127	28	**2.42**
					128	129	27	**1.28**
					130	131	28	**1.04**
**368**	ECM2 O94769$_1$: Extracellular matrix protein 2		16	30	126	127	2	**0.26**
					128	129	3	**0.50**
					130	131	3	**1.04**
**369**	ECM2 O94769$_2$: Extracellular matrix protein 2		14	28	126	127	1	**1.26**
					128	129	1	**2.05**
					130	131	1	**0.90**
**370**	EEF1A1 P68104$_1$, EEF1A1P5 Q5VTE0: Elongation factor 1-alpha 1, Putative elongation factor 1-alpha-like 3	2/4	15	40	126	127	39	**0.75**
					128	129	37	**0.44**
					130	131	36	**0.95**
**371**	EEF1B2 P24534: Elongation factor 1-beta		4	4	126	127	3	**1.00**
					128	129	3	**0.93**
					130	131	3	**1.20**
**372**	EEF1D P29692$_{1-3}$: Elongation factor 1-delta	3/4	4	5	126	127	4	**1.14**
					128	129	3	**0.99**
					130	131	3	**1.18**
**373**	EEF1G P26641$_{1,2}$: Elongation factor 1-gamma	2/2	11	12	126	127	11	**0.63**
					128	129	11	**0.42**
					130	131	12	**1.14**
**374**	EEF2 P13639: Elongation factor 2		22	28	126	127	26	**0.63**
					128	129	28	**0.33**
					130	131	26	**0.99**
**375**	EFEMP1 Q12805$_{1,3,4}$: EGF-containing fibulin-like extracellular matrix protein 1	3/4	27	675	126	127	204	**0.41**
					128	129	215	**0.49**
					130	131	225	**0.88**
**376**	EFEMP1 Q12805$_5$: EGF-containing fibulin-like extracellular matrix protein 1		24	433	126	127	12	**0.39**
					128	129	12	**0.51**
					130	131	12	**0.89**

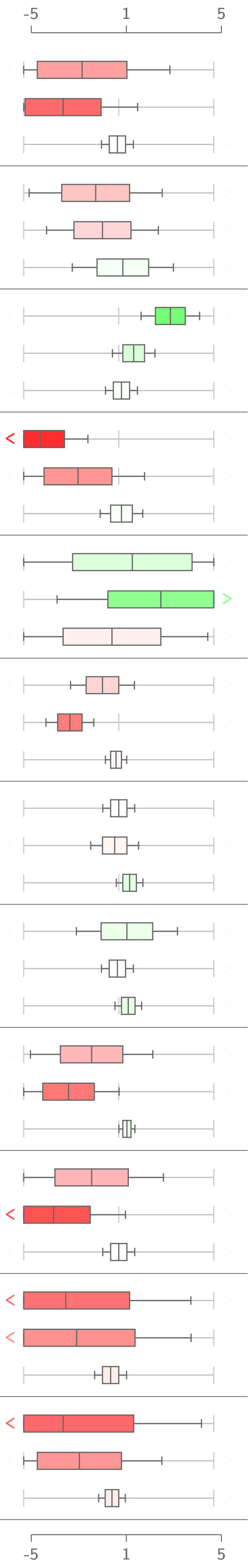

#	protein	group	peptides	spectra	ch1	ch2	quant	ratio
377	EFEMP2 O95967: EGF-containing fibulin-like extracellular matrix protein 2		17	95	126	127	77	**0.51**
					128	129	75	**0.68**
					130	131	81	**0.90**
378	EHD1 Q9H4M9: EH domain-containing protein 1	1/3	3	4	126	127	2	**0.60**
					128	129	2	**0.32**
					130	131	2	**1.25**
379	EHD2 Q9NZN4$_1$: EH domain-containing protein 2	1/2	2	3	126	127	1	**0.61**
					128	129	1	**0.68**
					130	131	1	**1.21**
380	EHD4 Q9H223: EH domain-containing protein 4		1	1	126	127	1	**0.99**
					128	129	1	**0.51**
					130	131	1	**0.98**
381	EIF2S1 P05198: Eukaryotic translation initiation factor 2 subunit 1		3	4	126	127	4	**0.68**
					128	129	4	**0.59**
					130	131	4	**0.99**
382	EIF3A Q14152$_{1,2}$: Eukaryotic translation initiation factor 3 subunit A	2/2	7	8	126	127	6	**0.90**
					128	129	7	**0.71**
					130	131	6	**1.12**
383	EIF3B P55884$_{1,2}$: Eukaryotic translation initiation factor 3 subunit B	2/2	4	4	126	127	3	**0.67**
					128	129	3	**0.72**
					130	131	3	**1.02**
384	EIF3C Q99613$_{1,2}$: Eukaryotic translation initiation factor 3 subunit C	2/3	3	3	126	127	3	**0.75**
					128	129	3	**0.60**
					130	131	2	**0.97**
385	EIF3L Q9Y262$_{1,2}$: Eukaryotic translation initiation factor 3 subunit L	2/2	2	2	126	127	2	**0.72**
					128	129	2	**0.51**
					130	131	2	**0.99**
386	EIF4A1 P60842$_{1,2}$: Eukaryotic initiation factor 4A-I	2/2	5	6	126	127	3	**0.26**
					128	129	4	**0.50**
					130	131	4	**0.98**
387	EIF4A2 Q14240$_{1,2}$: Eukaryotic initiation factor 4A-II	2/2	3	3	126	127	1	**2.11**
					128	129	1	**1.17**
					130	131	1	**1.20**
388	EIF4A3 P38919: Eukaryotic initiation factor 4A-III		1	1	126	127	1	**0.46**
					128	129	1	**0.40**
					130	131	1	**0.92**

#	protein	group	peptides	spectra	ch1	ch2	quant	ratio
389	EIF4G1 Q04637$_{1,3-9}$: Eukaryotic translation initiation factor 4 gamma 1	8/8	6	8	126	127	5	**0.34**
					128	129	6	**0.22**
					130	131	7	**1.19**
390	EIF4G2 P78344$_{1,2}$: Eukaryotic translation initiation factor 4 gamma 2	2/2	4	4	126	127	3	**0.47**
					128	129	3	**0.33**
					130	131	3	**0.94**
391	EIF4H Q15056$_{1,2}$: Eukaryotic translation initiation factor 4H	2/2	3	3	126	127	3	**2.08**
					128	129	3	**1.48**
					130	131	3	**1.03**
392	EIF5A P63241$_{1,2}$: Eukaryotic translation initiation factor 5A-1	2/2	3	5	126	127	5	**0.94**
					128	129	5	**1.12**
					130	131	4	**1.09**
393	ELANE P08246: Neutrophil elastase		2	2	126	127	2	**1.97**
					128	129	2	**0.77**
					130	131	2	**0.77**
394	ELL2 O00472$_{1,2}$: RNA polymerase II elongation factor ELL2	2/2	1	2	126	127	2	**0.72**
					128	129	2	**0.85**
					130	131	2	**0.97**
395	ELN P15502$_{1,2,4,5}$: Elastin	4/4	1	2	126	127	2	**1.74**
					128	129	2	**4.20**
					130	131	2	**0.19**
396	EMILIN1 Q9Y6C2$_{1}$: EMILIN-1	1/2	11	13	126	127	12	**0.94**
					128	129	13	**0.90**
					130	131	12	**1.01**
397	EMILIN2 Q9BXX0: EMILIN-2		12	13	126	127	13	**1.01**
					128	129	12	**0.88**
					130	131	13	**1.02**
398	ENO1 P06733$_{1}$: Alpha-enolase	1/7	16	23	126	127	21	**1.16**
					128	129	20	**0.12**
					130	131	23	**1.06**
399	EPB41L2 O43491$_{1,3,4}$: Band 4.1-like protein 2	3/3	2	2	126	127	2	**0.96**
					128	129	2	**0.59**
					130	131	2	**1.20**
400	EPHX1 P07099: Epoxide hydrolase 1		2	2	126	127	2	**1.46**
					128	129	2	**0.61**
					130	131	2	**0.86**

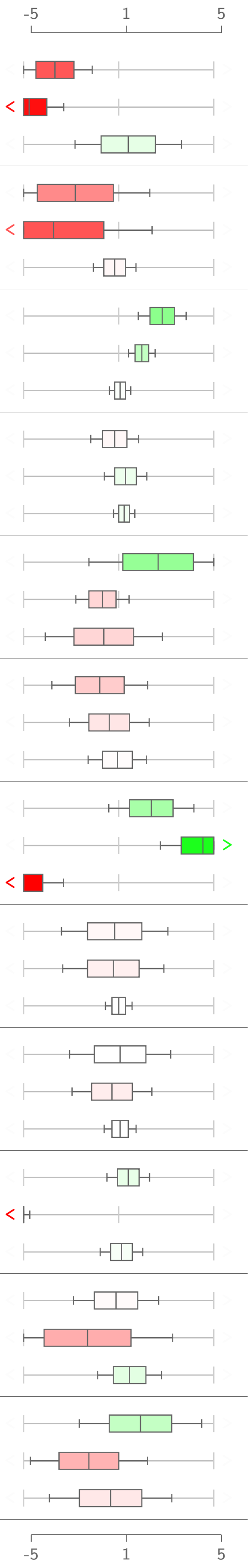

#	protein	group	peptides	spectra	ch1	ch2	quant	ratio
**401**	EPPK1 P58107: Epiplakin		7	10	126	127	3	**2.27**
					128	129	5	**2.42**
					130	131	3	**1.26**
**402**	EPX P11678: Eosinophil peroxidase		6	7	126	127	3	**3.26**
					128	129	4	**0.48**
					130	131	4	**0.42**
**403**	ERH P84090: Enhancer of rudimentary homolog		2	2	126	127	2	**4.38**
					128	129	2	**1.89**
					130	131	2	**1.01**
**404**	ERP44 Q9BS26: Endoplasmic reticulum resident protein 44		7	12	126	127	12	**1.58**
					128	129	12	**1.21**
					130	131	11	**1.01**
**405**	ESD P10768: S-formylglutathione hydrolase		2	2	126	127	2	**0.88**
					128	129	2	**0.51**
					130	131	2	**1.02**
**406**	ETF1 $P62495_1$: Eukaryotic peptide chain release factor subunit 1	1/2	4	5	126	127	3	**0.30**
					128	129	3	**0.37**
					130	131	5	**1.03**
**407**	EWSR1 $Q01844_{1-3,5,6}$: RNA-binding protein EWS	5/5	2	2	126	127	2	**2.08**
					128	129	2	**0.69**
					130	131	2	**0.90**
**408**	EZR P15311: Ezrin		10	17	126	127	16	**3.14**
					128	129	15	**1.45**
					130	131	16	**0.99**
**409**	F10 P00742: Coagulation factor X		12	51	126	127	46	**0.44**
					128	129	46	**0.53**
					130	131	45	**1.26**
**410**	F11 $P03951_1$: Coagulation factor XI	1/2	11	18	126	127	16	**0.86**
					128	129	16	**1.53**
					130	131	15	**1.05**
**411**	F12 P00748: Coagulation factor XII		18	57	126	127	55	**0.78**
					128	129	53	**1.17**
					130	131	57	**1.06**
**412**	F13A1 P00488: Coagulation factor XIII A chain		23	52	126	127	47	**1.43**
					128	129	51	**1.07**
					130	131	50	**0.88**

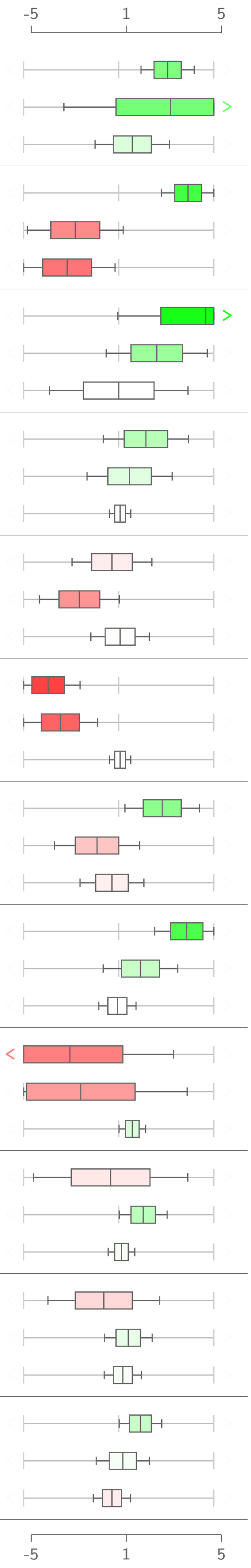

#	protein	group	peptides	spectra	ch1	ch2	quant	ratio
**413**	F13B P05160: Coagulation factor XIII B chain		7	8	126	127	8	**1.26**
					128	129	7	**2.18**
					130	131	8	**1.05**
**414**	F2 P00734: Prothrombin		39	718	126	127	698	**0.43**
					128	129	670	**0.71**
					130	131	650	**1.26**
**415**	F5 P12259: Coagulation factor V		20	23	126	127	23	**0.55**
					128	129	23	**0.38**
					130	131	22	**1.06**
**416**	F7 P08709$_{1,2}$: Coagulation factor VII	2/2	2	3	126	127	3	**0.36**
					128	129	3	**0.36**
					130	131	3	**1.12**
**417**	F9 P00740$_{1}$: Coagulation factor IX	1/2	15	37	126	127	36	**0.43**
					128	129	35	**0.40**
					130	131	36	**1.08**
**418**	FABP3 P05413: Fatty acid-binding protein, heart		2	3	126	127	3	**0.71**
					128	129	3	**0.83**
					130	131	3	**1.05**
**419**	FABP5 Q01469: Fatty acid-binding protein, epidermal	1/2	3	3	126	127	3	**5.58**
					128	129	3	**1.59**
					130	131	3	**1.10**
**420**	FAM114A1 Q8IWE2$_{1,2}$: Protein NOXP20	2/2	3	3	126	127	3	**0.33**
					128	129	3	**0.40**
					130	131	3	**0.94**
**421**	FAM129A Q9BZQ8: Protein Niban		2	2	126	127	2	**1.26**
					128	129	2	**0.63**
					130	131	2	**0.87**
**422**	FAM180A Q6UWF9: Protein FAM180A		3	3	126	127	3	**0.69**
					128	129	3	**1.39**
					130	131	3	**0.64**
**423**	FAM184B Q9ULE4: Protein FAM184B		4	12	126	127	5	**0.52**
					128	129	7	**0.96**
					130	131	5	**0.92**
**424**	FAM20A Q96MK3: Pseudokinase FAM20A		3	3	126	127	3	**0.64**
					128	129	3	**0.95**
					130	131	3	**0.94**

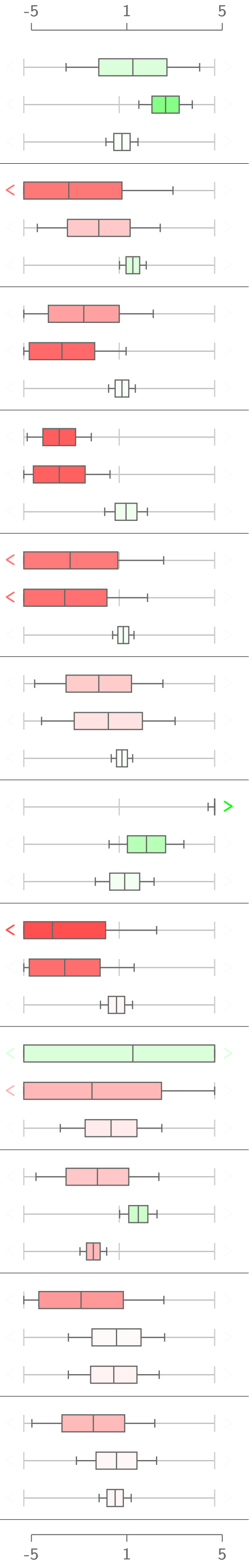

#	protein	group	peptides	spectra	ch1	ch2	quant	ratio
**425**	FAM49B Q9NUQ9$_1$: Protein FAM49B		3	3	126	127	3	**1.33**
					128	129	3	**0.57**
					130	131	3	**0.88**
**426**	FBLN1 P23142$_1$: Fibulin-1	1/2	31	1129	126	127	181	**0.36**
					128	129	177	**0.48**
					130	131	176	**0.97**
**427**	FBLN1 P23142$_3$: Fibulin-1		23	950	126	127	2	**5.83**
					128	129	2	**2.82**
					130	131	2	**0.86**
**428**	FBLN1 P23142$_4$: Fibulin-1		27	1041	126	127	78	**0.44**
					128	129	71	**0.88**
					130	131	84	**0.94**
**429**	FBLN2 P98095$_{1,2}$: Fibulin-2	2/2	24	68	126	127	68	**0.79**
					128	129	66	**0.71**
					130	131	67	**0.98**
**430**	FBLN5 Q9UBX5: Fibulin-5		14	247	126	127	222	**0.56**
					128	129	224	**0.63**
					130	131	235	**0.92**
**431**	FBLN7 Q53RD9$_{1-3}$: Fibulin-7	3/4	6	8	126	127	7	**0.69**
					128	129	7	**0.75**
					130	131	7	**1.05**
**432**	FBN1 P35555: Fibrillin-1 [Cleaved into: Asprosin]		84	197	126	127	146	**0.72**
					128	129	151	**1.33**
					130	131	154	**1.00**
**433**	FBN2 P35556$_1$: Fibrillin-2 [Cleaved into: Fibrillin-2 C-terminal peptide]	1/2	23	52	126	127	27	**2.55**
					128	129	24	**1.54**
					130	131	23	**1.00**
**434**	FBXO2 Q9UK22: F-box only protein 2		2	2	126	127	2	**0.71**
					128	129	2	**1.08**
					130	131	2	**1.29**
**435**	FCGBP Q9Y6R7: IgGFc-binding protein		17	18	126	127	18	**0.99**
					128	129	17	**1.03**
					130	131	18	**1.03**
**436**	FCN1 O00602: Ficolin-1		6	9	126	127	6	**1.68**
					128	129	7	**0.42**
					130	131	5	**0.91**

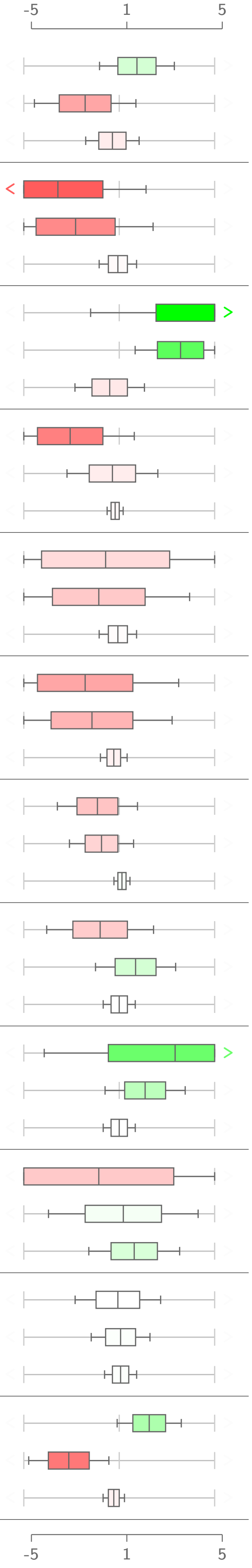

#	protein	group	peptides	spectra	ch1	ch2	quant	ratio
437	FCN2 Q15485$_{1,2}$: Ficolin-2	2/2	3	4	126	127	2	**0.63**
					128	129	2	**1.27**
					130	131	2	**1.07**
438	FCN3 O75636$_{1}$: Ficolin-3		10	22	126	127	2	**0.46**
					128	129	2	**0.75**
					130	131	2	**1.02**
440	FERMT3 Q86UX7$_{1,2}$: Fermitin family homolog 3	2/2	4	4	126	127	3	**0.61**
					128	129	3	**0.24**
					130	131	4	**1.07**
441	FETUB Q9UGM5$_{1}$: Fetuin-B	1/2	14	32	126	127	28	**0.22**
					128	129	32	**0.32**
					130	131	32	**0.73**
442	FGA P02671$_{1}$: Fibrinogen alpha chain [Cleaved into: Fibrinopeptide A; Fibrinogen alpha chain]	1/2	64	4713	126	127	4494	**7.99**
					128	129	4602	**2.78**
					130	131	4445	**0.86**
443	FGB P02675: Fibrinogen beta chain [Cleaved into: Fibrinopeptide B; Fibrinogen beta chain]		46	3869	126	127	3590	**5.87**
					128	129	3656	**1.97**
					130	131	3442	**0.93**
444	FGD6 Q6ZV73$_{1,2}$: FYVE, RhoGEF and PH domain-containing protein 6	2/2	2	2	126	127	1	**3.74**
					128	129	1	**3.41**
					130	131		
445	FGG P02679$_{1}$: Fibrinogen gamma chain	1/2	33	3187	126	127	3057	**5.76**
					128	129	2888	**1.95**
					130	131	2902	**0.92**
446	FIBIN Q8TAL6: Fin bud initiation factor homolog		2	2	126	127	2	**0.61**
					128	129	2	**0.74**
					130	131	2	**1.06**
447	FKBP1A P62942: Peptidyl-prolyl cis-trans isomerase FKBP1A		1	1	126	127	1	**0.94**
					128	129	1	**0.82**
					130	131	1	**1.03**
448	FKBP9 O95302$_{1,3}$: Peptidyl-prolyl cis-trans isomerase FKBP9	2/2	3	3	126	127	3	**2.02**
					128	129	3	**0.97**
					130	131	3	**1.05**
449	FLG P20930: Filaggrin		3	3	126	127	3	**3.13**
					128	129	3	**1.90**
					130	131	3	**3.26**

#	protein	group	peptides	spectra	ch1	ch2	quant	ratio
**450**	FLII Q13045$_{1-3}$: Protein flightless-1 homolog	3/3	2	3	126	127	3	**0.47**
					128	129	3	**0.49**
					130	131	3	**0.88**
**451**	FLNA P21333$_{1,2}$: Filamin-A	2/2	61	82	126	127	62	**1.13**
					128	129	65	**0.39**
					130	131	65	**0.97**
**452**	FLNB O75369$_{1-3,6,8,9}$: Filamin-B	6/9	12	14	126	127	5	**1.88**
					128	129	5	**0.57**
					130	131	5	**0.93**
**453**	FLNC Q14315$_{1,2}$: Filamin-C	2/2	43	52	126	127	33	**0.90**
					128	129	34	**0.31**
					130	131	32	**0.96**
**454**	FMOD Q06828: Fibromodulin		9	33	126	127	14	**1.31**
					128	129	16	**0.86**
					130	131	16	**0.96**
**455**	FN1 P02751$_{1,3,17}$: Fibronectin	3/17	87	1059	126	127	990	**0.70**
					128	129	1005	**0.63**
					130	131	994	**1.06**
**456**	FNDC1 Q4ZHG4$_{1,2}$: Fibronectin type III domain-containing protein 1	2/2	11	12	126	127	9	**0.77**
					128	129	11	**1.12**
					130	131	11	**0.98**
**457**	FRAS1 Q86XX4$_{1,2}$: Extracellular matrix protein FRAS1	2/5	9	12	126	127	6	**0.52**
					128	129	7	**0.97**
					130	131	8	**0.79**
**458**	FRZB Q92765: Secreted frizzled-related protein 3		1	1	126	127	1	**0.23**
					128	129	1	**3.42**
					130	131	1	**0.84**
**459**	FSCN1 Q16658: Fascin		3	5	126	127	4	**1.48**
					128	129	5	**0.91**
					130	131	5	**1.02**
**460**	FST P19883$_{1,2}$: Follistatin	2/2	11	11	126	127	7	**0.23**
					128	129	10	**0.91**
					130	131	11	**1.05**
**461**	FSTL1 Q12841$_{1}$: Follistatin-related protein 1	1/2	13	39	126	127	34	**0.72**
					128	129	33	**3.77**
					130	131	39	**0.92**

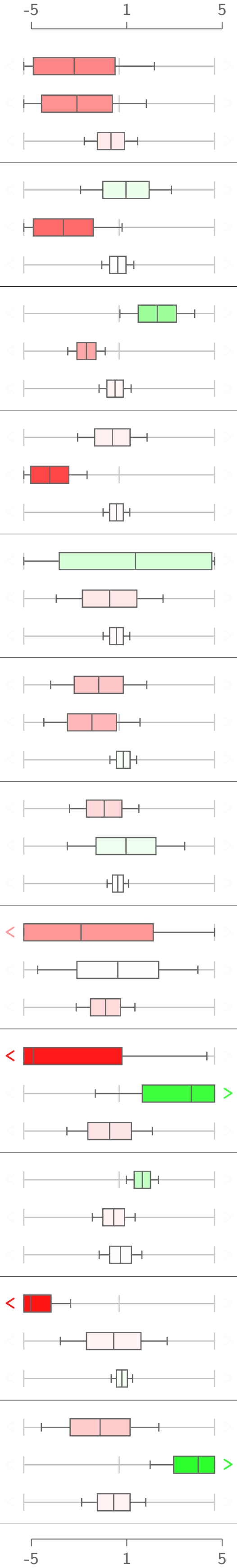

#	protein	group	peptides	spectra	ch1	ch2	quant	ratio
**462**	FSTL3 O95633$_{1,2}$: Follistatin-related protein 3	2/2	3	3	126	127	3	**0.84**
					128	129	3	**6.84**
					130	131	3	**0.79**
**463**	FTL P02792: Ferritin light chain		5	5	126	127	5	**2.06**
					128	129	5	**1.62**
					130	131	4	**0.86**
**464**	FUBP1 Q96AE4$_{1,2}$: Far upstream element-binding protein 1	2/2	4	4	126	127	3	**2.26**
					128	129	3	**0.89**
					130	131	3	**0.96**
**465**	FUS P35637$_{1,2}$: RNA-binding protein FUS	2/2	4	10	126	127	8	**1.71**
					128	129	6	**1.45**
					130	131	8	**0.86**
**466**	FXR1 P51114$_{1-3}$: Fragile X mental retardation syndrome-related protein 1	3/3	1	1	126	127	1	**1.09**
					128	129	1	**0.44**
					130	131	1	**1.06**
**467**	G3BP1 Q13283$_{1}$: Ras GTPase-activating protein-binding protein 1		2	4	126	127	3	**1.03**
					128	129	2	**0.40**
					130	131	3	**1.03**
**468**	G6PD P11413$_{1-3}$: Glucose-6-phosphate 1-dehydrogenase	3/3	2	2	126	127	2	**0.83**
					128	129	2	**0.64**
					130	131	2	**0.76**
**469**	GAL3ST4 Q96RP7$_{1,2}$: Galactose-3-O-sulfotransferase 4	2/2	1	2	126	127	2	**0.56**
					128	129	2	**2.06**
					130	131	2	**0.52**
**470**	GALK1 P51570$_{1,2}$: Galactokinase	2/2	2	3	126	127	3	**1.00**
					128	129	3	**0.60**
					130	131	3	**1.07**
**471**	GANAB Q14697$_{1}$: Neutral alpha-glucosidase AB		19	32	126	127	2	**1.41**
					128	129	2	**0.33**
					130	131	2	**0.90**
**472**	GANAB Q14697$_{2}$: Neutral alpha-glucosidase AB		19	31	126	127	1	**1.03**
					128	129	1	**0.72**
					130	131	1	**0.88**
**473**	GANAB Q14697$_{3}$: Neutral alpha-glucosidase AB		2	7	126	127		
					128	129		
					130	131	1	**0.53**

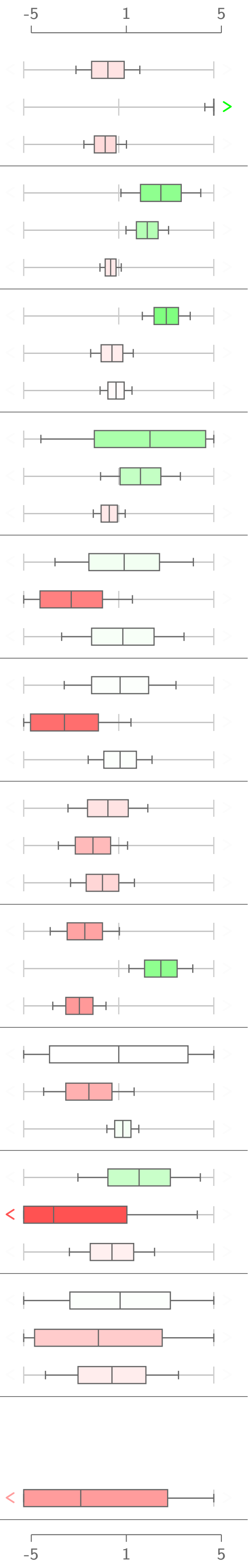

#	protein	group	peptides	spectra	ch1	ch2	quant	ratio
**474**	GAPDH P04406$_1$: Glyceraldehyde-3-phosphate dehydrogenase	1/2	15	58	126	127	57	**1.13**
					128	129	58	**0.27**
					130	131	57	**1.29**
**475**	GARS P41250: Glycine--tRNA ligase		6	10	126	127	6	**0.93**
					128	129	6	**0.71**
					130	131	6	**1.04**
**476**	GAS1 P54826: Growth arrest-specific protein 1		8	11	126	127	11	**0.57**
					128	129	10	**1.01**
					130	131	11	**1.08**
**477**	GAS6 Q14393$_{1,2}$: Growth arrest-specific protein 6	2/5	14	23	126	127	21	**0.50**
					128	129	22	**0.73**
					130	131	23	**1.00**
**478**	GBA P04062$_{1,2,4,5}$: Glucosylceramidase	4/5	3	4	126	127	4	**0.39**
					128	129	4	**0.62**
					130	131	4	**0.96**
**479**	GC P02774$_{1,3}$: Vitamin D-binding protein	2/3	27	340	126	127	273	**0.85**
					128	129	295	**1.89**
					130	131	300	**1.05**
**481**	GCG P01275: Glucagon [Cleaved into: Glicentin; Glicentin-related polypeptide		3	5	126	127	4	**0.52**
					128	129	5	**1.80**
					130	131	5	**0.88**
**482**	GDF15 Q99988: Growth/differentiation factor 15		5	7	126	127	7	**1.56**
					128	129	7	**1.34**
					130	131	6	**1.03**
**483**	GDF6 Q6KF10: Growth/differentiation factor 6		2	2	126	127	2	**0.99**
					128	129	2	**0.78**
					130	131	2	**0.85**
**484**	GFRA2 O00451$_1$: GDNF family receptor alpha-2		2	2	126	127	2	**1.50**
					128	129	2	**3.89**
					130	131	2	**1.21**
**485**	GHRL Q9UBU3$_{1-5}$: Appetite-regulating hormone	5/5	1	1	126	127	1	**1.54**
					128	129	1	**1.00**
					130	131	1	**0.68**
**486**	GIMAP4 Q9NUV9: GTPase IMAP family member 4		3	3	126	127	2	**0.43**
					128	129	2	**0.14**
					130	131	3	**1.17**

#	protein	group	peptides	spectra	ch1	ch2	quant	ratio
**487**	GIP P09681: Gastric inhibitory polypeptide		3	6	126	127	6	**0.68**
					128	129	6	**0.98**
					130	131	6	**0.63**
**488**	GIPC1 O14908$_{1,2}$: PDZ domain-containing protein GIPC1	2/2	1	2	126	127	2	**0.42**
					128	129	2	**0.61**
					130	131	2	**0.93**
**489**	GKN1 Q9NS71: Gastrokine-1		6	12	126	127	12	**0.46**
					128	129	12	**1.37**
					130	131	12	**0.87**
**490**	GKN2 Q86XP6: Gastrokine-2		1	1	126	127	1	**0.69**
					128	129	1	**0.61**
					130	131	1	**0.74**
**491**	GLG1 Q92896$_{1-3}$: Golgi apparatus protein 1	3/3	2	2	126	127	2	**2.28**
					128	129	2	**1.35**
					130	131	2	**1.02**
**492**	GLIPR1L2 Q4G1C9$_{1,5,6}$: GLIPR1-like protein 2	3/3	3	35	126	127	15	**3.02**
					128	129	17	**1.52**
					130	131	21	**1.11**
**493**	GLRX3 O76003: Glutaredoxin-3		2	2	126	127	2	**0.82**
					128	129	2	**0.84**
					130	131	2	**1.14**
**494**	GM2A P17900: Ganglioside GM2 activator		7	9	126	127	8	**0.40**
					128	129	9	**0.27**
					130	131	9	**0.85**
**495**	GNAI2 P04899$_{1-6}$: Guanine nucleotide-binding protein G(i) subunit alpha-2	6/6	2	2	126	127	2	**0.44**
					128	129	2	**0.22**
					130	131	2	**1.07**
**496**	GNB1 P62873$_{1,2}$: Guanine nucleotide-binding protein G(I)/G(S)/G(T) subunit beta-1	2/5	4	5	126	127	2	**1.83**
					128	129	2	**0.81**
					130	131	2	**1.27**
**497**	GNB4 Q9HAV0: Guanine nucleotide-binding protein subunit beta-4	1/2	4	5	126	127	2	**0.91**
					128	129	2	**0.40**
					130	131	2	**0.86**
**498**	GNPTG Q9UJJ9: N-acetylglucosamine-1-phosphotransferase subunit gamma		1	1	126	127	1	**0.42**
					128	129	1	**1.56**
					130	131	1	**1.03**

#	protein	group	peptides	spectra	ch1	ch2	quant	ratio
**499**	GOLIM4 O00461: Golgi integral membrane protein 4		10	16	126	127	13	**0.38**
					128	129	14	**0.78**
					130	131	14	**1.04**
**500**	GOLM1 Q8NBJ4$_{1,2}$: Golgi membrane protein 1	2/2	9	11	126	127	8	**0.65**
					128	129	6	**1.30**
					130	131	8	**1.10**
**501**	GPLD1 P80108$_{1}$: Phosphatidylinositol-glycan-specific phospholipase D		9	10	126	127	10	**0.64**
					128	129	9	**0.95**
					130	131	8	**1.12**
**502**	GPS1 Q13098$_{4-7}$: COP9 signalosome complex subunit 1	4/4	2	2	126	127	2	**0.32**
					128	129	2	**0.25**
					130	131	2	**1.00**
**503**	GPX3 P22352: Glutathione peroxidase 3		8	13	126	127	13	**0.61**
					128	129	13	**1.37**
					130	131	12	**0.87**
**504**	GREM1 O60565$_{1}$: Gremlin-1	1/2	2	3	126	127	2	**0.29**
					128	129	2	**0.55**
					130	131	3	**0.98**
**505**	GREM2 Q9H772: Gremlin-2		1	1	126	127	1	**0.24**
					128	129	1	**0.35**
					130	131	1	**1.03**
**507**	GRN P28799$_{1}$: Granulins	1/3	8	9	126	127	6	**1.14**
					128	129	9	**1.47**
					130	131	9	**1.08**
**508**	GSDMD P57764: Gasdermin-D		2	2	126	127	2	**0.32**
					128	129	2	**0.28**
					130	131	2	**1.36**
**509**	GSN P06396$_{1}$: Gelsolin	1/3	36	520	126	127	24	**0.24**
					128	129	24	**0.38**
					130	131	22	**0.78**
**510**	GSN P06396$_{2}$: Gelsolin		36	498	126	127	2	**0.64**
					128	129	2	**0.79**
					130	131	2	**0.98**
**511**	GSTM3 P21266: Glutathione S-transferase Mu 3		3	3	126	127	3	**0.19**
					128	129	3	**0.35**
					130	131	3	**0.96**

#	protein	group	peptides	spectra	ch1	ch2	quant	ratio
**512**	GSTP1 P09211: Glutathione S-transferase P		2	2	126	127	2	**0.41**
					128	129	2	**0.40**
					130	131	2	**1.15**
**513**	GUCA2A Q02747: Guanylin		2	2	126	127	2	**1.11**
					128	129	2	**4.65**
					130	131	2	**0.86**
**514**	H1F0 P07305$_{1,2}$: Histone H1.0	2/2	3	3	126	127	2	**3.54**
					128	129	2	**1.07**
					130	131	2	**1.13**
**515**	H1FX Q92522: Histone H1x		4	5	126	127	5	**3.66**
					128	129	5	**0.81**
					130	131	5	**0.84**
**516**	H2AFV Q71UI9$_{1,2}$, H2AFZ P0C0S5: Histone H2A.V, Histone H2A.Z	3/5	3	10	126	127	1	**4.79**
					128	129	1	**0.13**
					130	131	1	**0.41**
**517**	H2AFX P16104: Histone H2AX		7	25	126	127	4	**6.30**
					128	129	5	**1.14**
					130	131	4	**0.57**
**518**	H3F3A P84243: Histone H3.3		8	26	126	127	1	**10.58**
					128	129	1	**0.94**
					130	131	1	**0.76**
**519**	HABP2 Q14520$_{1,2}$: Hyaluronan-binding protein 2	2/2	16	34	126	127	30	**0.21**
					128	129	34	**0.45**
					130	131	34	**1.36**
**520**	HADHA P40939$_1$: Trifunctional enzyme subunit alpha, mitochondrial		3	5	126	127	4	**6.07**
					128	129	5	**0.57**
					130	131	4	**0.69**
**521**	HADHB P55084$_1$: Trifunctional enzyme subunit beta, mitochondrial	1/2	3	4	126	127	4	**6.40**
					128	129	3	**1.04**
					130	131	4	**0.78**
**522**	HAMP P81172: Hepcidin		1	3	126	127	3	**2.49**
					128	129	3	**3.24**
					130	131	3	**0.89**
**523**	HBA1; P69905: Hemoglobin subunit alpha		4	14	126	127	14	**9.24**
					128	129	14	**1.08**
					130	131	13	**0.79**

#	protein	group	peptides	spectra	ch1	ch2	quant	ratio
**524**	HBB P68871: Hemoglobin subunit beta	1/2	8	15	126	127	14	**6.28**
					128	129	13	**0.83**
					130	131	15	**0.96**
**525**	HCK P08631$_{1-4}$: Tyrosine-protein kinase HCK	4/6	4	6	126	127	4	**0.92**
					128	129	3	**0.37**
					130	131	3	**0.94**
**526**	HCLS1 P14317$_{1}$: Hematopoietic lineage cell-specific protein		3	3	126	127	3	**1.08**
					128	129	3	**0.89**
					130	131	3	**1.02**
**527**	HDGF P51858$_{1-3}$: Hepatoma-derived growth factor	3/3	3	5	126	127	5	**1.75**
					128	129	5	**1.47**
					130	131	3	**1.07**
**528**	HDLBP Q00341$_{1,2}$: Vigilin	2/2	7	7	126	127	7	**0.72**
					128	129	7	**0.52**
					130	131	5	**1.01**
**529**	HERC2 O95714: E3 ubiquitin-protein ligase HERC2		4	30	126	127	10	**1.62**
					128	129	13	**1.49**
					130	131	14	**0.95**
**530**	HEXA P06865$_{1}$: Beta-hexosaminidase subunit alpha		4	6	126	127	5	**0.42**
					128	129	6	**1.18**
					130	131	6	**0.92**
**531**	HEXB P07686: Beta-hexosaminidase subunit beta		5	5	126	127	4	**0.76**
					128	129	3	**1.28**
					130	131	3	**0.92**
**532**	HINT1 P49773: Histidine triad nucleotide-binding protein 1		2	2	126	127	2	**1.13**
					128	129	2	**1.28**
					130	131	2	**1.14**
**533**	HIST1H1B P16401: Histone H1.5		13	37	126	127	11	**4.57**
					128	129	10	**0.41**
					130	131	12	**0.64**
**534**	HIST1H1C P16403: Histone H1.2		16	44	126	127	7	**5.12**
					128	129	7	**0.56**
					130	131	7	**0.63**
**535**	HIST1H1D P16402: Histone H1.3	1/3	15	39	126	127	9	**3.67**
					128	129	10	**0.80**
					130	131	10	**0.74**

#	protein	group	peptides	spectra	ch1	ch2	quant	ratio
536	HIST1H1E P10412: Histone H1.4		13	43	126	127	6	**4.61**
					128	129	4	**0.69**
					130	131	6	**0.71**
537	HIST1H2AB P04908, HIST1H2AD P20671, HIST3H2A Q7L7L0: Histone H2A type 1-B/E, Histone H2A type 1-D, . . .	3/3	6	23	126	127	1	**4.79**
					128	129	1	**0.23**
					130	131	1	**0.67**
542	HIST1H2BM Q99879: Histone H2B type 1-M		11	65	126	127	1	**2.10**
					128	129	1	**0.63**
					130	131	1	**0.66**
543	HIST1H3A P68431, HIST2H3A; Q71DI3, HIST3H3 Q16695: Histone H3.1, Histone H3.2, . . .	3/4	8	28	126	127	3	**2.87**
					128	129	3	**0.35**
					130	131	3	**0.49**
544	HIST1H4A P62805: Histone H4		6	36	126	127	32	**3.94**
					128	129	36	**0.54**
					130	131	36	**0.90**
545	HIST2H2AB Q8IUE6: Histone H2A type 2-B		5	19	126	127	2	**5.72**
					128	129	2	**0.51**
					130	131	2	**0.60**
546	HIST2H2AC Q16777: Histone H2A type 2-C	1/4	6	23	126	127	1	**8.07**
					128	129	1	**0.93**
					130	131	1	**0.56**
550	HLA-A P10316: HLA class I histocompatibility antigen, A-69 alpha chain	1/2	7	9	126	127	1	**1.02**
					128	129	1	**1.10**
					130	131	1	**1.20**
553	HLA-A P30447: HLA class I histocompatibility antigen, A-23 alpha chain	1/2	6	10	126	127	1	**2.73**
					128	129	1	**2.26**
					130	131	1	**0.92**
560	HLA-C P10321: HLA class I histocompatibility antigen, Cw-7 alpha chain	1/2	8	8	126	127	1	**0.63**
					128	129	1	**2.00**
					130	131	1	**0.81**
562	HLA-DRB1 P01911, HLA-DRB1 P01912, HLA-DRB1 P04229, . . . : HLA class II histocompatibility antigen, DRB1-15 beta chain, HLA class II histocompatibility antigen, DRB1-3 chain, . . .	11/11	2	2	126	127	2	**3.69**
					128	129	2	**1.21**
					130	131	2	**0.70**
563	HMCN1 Q96RW7$_{1,2}$: Hemicentin-1	2/2	24	28	126	127	19	**0.66**
					128	129	20	**0.47**
					130	131	20	**0.94**

#	protein	group	peptides	spectra	ch1	ch2	quant	ratio
564	HMCN2 Q8NDA2$_1$: Hemicentin-2	1/3	21	21	126	127	18	**0.62**
					128	129	20	**0.54**
					130	131	18	**0.94**
565	HMGN1 P05114: Non-histone chromosomal protein HMG-14		2	2	126	127	2	**6.85**
					128	129	2	**0.89**
					130	131	2	**0.66**
566	HMGN2 P05204: Non-histone chromosomal protein HMG-17		4	4	126	127	3	**10.64**
					128	129	4	**0.84**
					130	131	4	**0.55**
567	HMGN4 O00479: High mobility group nucleosome-binding domain-containing protein 4		3	5	126	127	5	**9.15**
					128	129	5	**1.23**
					130	131	4	**0.70**
568	HMHA1 Q92619$_{1,2}$: Minor histocompatibility protein HA-1 [Cleaved into: Minor histocompatibilit ...	2/2	3	4	126	127	3	**0.98**
					128	129	4	**0.36**
					130	131	3	**0.96**
569	HNMT P50135$_1$: Histamine N-methyltransferase		3	3	126	127	2	**0.07**
					128	129	3	**0.27**
					130	131	3	**0.89**
570	HNRNPA0 Q13151: Heterogeneous nuclear ribonucleoprotein A0		2	2	126	127	1	**1.97**
					128	129	1	**1.31**
					130	131	1	**1.01**
571	HNRNPA1 P09651$_{1-3}$, HNRNPA1L2 Q32P51: Heterogeneous nuclear ribonucleoprotein A1, Heterogeneous nuclear ribonucleoprotein A1-like 2	4/4	4	7	126	127	3	**3.27**
					128	129	3	**1.42**
					130	131	3	**0.98**
572	HNRNPA2B1 P22626$_1$: Heterogeneous nuclear ribonucleoproteins A2/B1	1/2	10	14	126	127	8	**4.29**
					128	129	10	**1.30**
					130	131	10	**0.84**
573	HNRNPA3 P51991$_1$: Heterogeneous nuclear ribonucleoprotein A3	1/2	5	9	126	127	7	**1.67**
					128	129	8	**1.24**
					130	131	7	**1.21**
574	HNRNPAB Q99729$_{1-4}$: Heterogeneous nuclear ribonucleoprotein A/B	4/4	4	4	126	127	3	**2.22**
					128	129	3	**1.24**
					130	131	3	**0.81**
575	HNRNPC P07910$_{1,2}$: Heterogeneous nuclear ribonucleoproteins C1/C2	2/4	9	10	126	127	6	**1.36**
					128	129	6	**0.70**
					130	131	6	**1.40**

#	protein	group	peptides	spectra	ch1	ch2	quant	ratio
**576**	HNRNPCL1 O60812, HNRNPCL2 B2RXH8: Heterogeneous nuclear ribonucleoprotein C-like 1, Heterogeneous nuclear ribonucleoprotein C-like 2	2/4	5	7	126	127	3	**0.18**
					128	129	3	**1.50**
					130	131	3	**0.95**
**577**	HNRNPD Q14103$_{1,3}$: Heterogeneous nuclear ribonucleoprotein D0	2/2	5	6	126	127	3	**3.07**
					128	129	3	**1.21**
					130	131	3	**0.93**
**578**	HNRNPD Q14103$_{2,4}$: Heterogeneous nuclear ribonucleoprotein D0	2/2	4	4	126	127	1	**2.55**
					128	129	1	**2.63**
					130	131	1	**0.88**
**579**	HNRNPDL O14979$_{1-3}$: Heterogeneous nuclear ribonucleoprotein D-like	3/3	2	2	126	127	1	**1.41**
					128	129	1	**1.37**
					130	131	1	**1.14**
**580**	HNRNPF P52597: Heterogeneous nuclear ribonucleoprotein F		4	5	126	127	2	**1.05**
					128	129	2	**0.99**
					130	131	2	**0.90**
**581**	HNRNPH1 P31943: Heterogeneous nuclear ribonucleoprotein H	1/2	5	7	126	127	4	**1.45**
					128	129	4	**0.93**
					130	131	3	**1.18**
**582**	HNRNPH3 P31942$_{1-3}$: Heterogeneous nuclear ribonucleoprotein H3	3/6	3	3	126	127	3	**1.13**
					128	129	3	**1.34**
					130	131	3	**1.13**
**583**	HNRNPK P61978$_{1-3}$: Heterogeneous nuclear ribonucleoprotein K	3/3	13	26	126	127	22	**1.13**
					128	129	20	**0.54**
					130	131	23	**1.21**
**584**	HNRNPL P14866$_{1}$: Heterogeneous nuclear ribonucleoprotein L	1/2	9	11	126	127	9	**1.21**
					128	129	10	**0.72**
					130	131	10	**1.00**
**585**	HNRNPM P52272$_{1,2}$: Heterogeneous nuclear ribonucleoprotein M	2/2	9	10	126	127	8	**1.37**
					128	129	9	**0.52**
					130	131	9	**0.96**
**586**	HNRNPU Q00839$_{1}$: Heterogeneous nuclear ribonucleoprotein U		13	16	126	127	2	**1.37**
					128	129	2	**0.28**
					130	131	2	**0.99**
**587**	HNRNPU Q00839$_{2}$: Heterogeneous nuclear ribonucleoprotein U		12	16	126	127	2	**0.78**
					128	129	2	**0.54**
					130	131	2	**1.00**

#	protein	group	peptides	spectra	ch1	ch2	quant	ratio
588	HNRNPUL2 Q1KMD3: Heterogeneous nuclear ribonucleoprotein U-like protein 2		11	18	126	127	14	**0.87**
					128	129	17	**0.95**
					130	131	17	**0.98**
589	HP P00738$_1$: Haptoglobin	1/2	21	46	126	127	17	**3.41**
					128	129	16	**2.46**
					130	131	17	**1.05**
590	HP1BP3 Q5SSJ5$_{1,2}$: Heterochromatin protein 1-binding protein 3	2/2	3	3	126	127	2	**4.21**
					128	129	2	**0.62**
					130	131	2	**0.96**
591	HPR P00739$_{1,2}$: Haptoglobin-related protein	2/2	18	48	126	127	18	**1.46**
					128	129	18	**0.30**
					130	131	19	**1.11**
592	HPX P02790: Hemopexin		12	27	126	127	24	**3.04**
					128	129	27	**3.25**
					130	131	24	**1.06**
593	HRC P23327: Sarcoplasmic reticulum histidine-rich calcium-binding protein		4	9	126	127	8	**5.20**
					128	129	8	**3.03**
					130	131	8	**1.16**
594	HRG P04196: Histidine-rich glycoprotein		20	250	126	127	170	**0.25**
					128	129	218	**0.56**
					130	131	230	**2.45**
595	HRNR Q86YZ3: Hornerin		4	4	126	127	3	**1.51**
					128	129	3	**1.50**
					130	131	3	**2.70**
596	HSP90AA1 P07900$_{1,2}$: Heat shock protein HSP 90-alpha	2/6	26	76	126	127	23	**0.54**
					128	129	24	**0.43**
					130	131	23	**1.08**
597	HSP90AA5P Q58FG0: Putative heat shock protein HSP 90-alpha A5		4	22	126	127	5	**4.14**
					128	129	5	**1.68**
					130	131	5	**1.09**
598	HSP90AB1 P08238: Heat shock protein HSP 90-beta	1/2	29	72	126	127	13	**0.24**
					128	129	13	**0.27**
					130	131	13	**1.00**
599	HSP90AB3P Q58FF7: Putative heat shock protein HSP 90-beta-3		12	43	126	127		
					128	129	1	**1.34**
					130	131		

#	protein	group	peptides	spectra	ch1	ch2	quant	ratio
**600**	HSP90AB4P Q58FF6: Putative heat shock protein HSP 90-beta 4		5	29	126	127	1	**1.30**
					128	129	1	**1.38**
					130	131	1	**1.15**
**601**	HSP90B1 P14625: Endoplasmin	1/2	32	74	126	127	54	**1.02**
					128	129	69	**0.40**
					130	131	64	**1.07**
**602**	HSPA1A P0DMV8$_{1,2}$, HSPA1B P0DMV9: Heat shock 70 kDa protein 1A, Heat shock 70 kDa protein 1B	3/4	15	25	126	127	5	**0.97**
					128	129	4	**0.47**
					130	131	5	**1.07**
**604**	HSPA2 P54652: Heat shock-related 70 kDa protein 2		10	17	126	127	3	**1.37**
					128	129	3	**0.60**
					130	131	3	**1.19**
**605**	HSPA5 P11021: 78 kDa glucose-regulated protein		26	38	126	127	30	**2.33**
					128	129	28	**1.26**
					130	131	29	**0.99**
**606**	HSPA6 P17066: Heat shock 70 kDa protein 6		6	8	126	127	2	**0.22**
					128	129	2	**0.61**
					130	131	2	**0.96**
**607**	HSPA8 P11142$_1$: Heat shock cognate 71 kDa protein	1/2	18	36	126	127	19	**1.50**
					128	129	19	**0.55**
					130	131	19	**0.98**
**608**	HSPA9 P38646: Stress-70 protein, mitochondrial		5	5	126	127	4	**5.87**
					128	129	3	**0.78**
					130	131	5	**0.76**
**609**	HSPB1 P04792: Heat shock protein beta-1		7	10	126	127	9	**1.82**
					128	129	9	**2.19**
					130	131	10	**1.05**
**610**	HSPB6 O14558: Heat shock protein beta-6		2	2	126	127	2	**1.04**
					128	129	2	**1.33**
					130	131	2	**1.21**
**611**	HSPD1 P10809$_1$: 60 kDa heat shock protein, mitochondrial		5	5	126	127	4	**6.59**
					128	129	3	**1.48**
					130	131	5	**0.88**
**612**	HSPE1 P61604: 10 kDa heat shock protein, mitochondrial		1	1	126	127	1	**32.00**
					128	129	1	**2.59**
					130	131	1	**0.99**

#	protein	group	peptides	spectra	ch1	ch2	quant	ratio
613	HSPG2 P98160: Basement membrane-specific heparan sulfate proteoglycan core protein		53	73	126	127	70	**0.47**
					128	129	71	**0.77**
					130	131	70	**0.98**
614	HTATSF1 O43719: HIV Tat-specific factor 1		11	25	126	127	19	**3.27**
					128	129	20	**1.80**
					130	131	20	**0.94**
615	HTRA1 Q92743: Serine protease HTRA1		14	44	126	127	41	**0.62**
					128	129	43	**0.57**
					130	131	43	**0.82**
616	HTRA3 P83110$_{1,2}$: Serine protease HTRA3	2/2	3	3	126	127	3	**0.51**
					128	129	3	**0.94**
					130	131	3	**1.01**
617	HUWE1 Q7Z6Z7$_{1-3}$: E3 ubiquitin-protein ligase HUWE1	3/3	4	6	126	127	4	**0.17**
					128	129	4	**0.12**
					130	131	5	**0.62**
618	HYDIN Q4G0P3$_{1}$: Hydrocephalus-inducing protein homolog		6	11	126	127	3	**0.57**
					128	129	4	**1.45**
					130	131	3	**0.70**
619	HYOU1 Q9Y4L1$_{1}$: Hypoxia up-regulated protein 1	1/2	11	12	126	127	9	**0.76**
					128	129	12	**1.00**
					130	131	12	**0.84**
620	IFI30 P13284: Gamma-interferon-inducible lysosomal thiol reductase		6	30	126	127	29	**3.76**
					128	129	29	**1.37**
					130	131	26	**0.97**
621	IGF1 P05019$_{1}$: Insulin-like growth factor I	1/4	6	9	126	127	9	**1.37**
					128	129	8	**1.42**
					130	131	8	**0.76**
622	IGF2 P01344$_{1-3}$: Insulin-like growth factor II	3/3	4	6	126	127	6	**1.48**
					128	129	6	**1.68**
					130	131	6	**0.94**
623	IGF2R P11717: Cation-independent mannose-6-phosphate receptor		5	7	126	127	6	**0.67**
					128	129	7	**1.04**
					130	131	7	**1.13**
624	IGFALS P35858$_{1,2}$: Insulin-like growth factor-binding protein complex acid labile subunit	2/2	7	7	126	127	7	**0.81**
					128	129	7	**0.90**
					130	131	6	**1.03**

#	protein	group	peptides	spectra	ch1	ch2	quant	ratio
**625**	IGFBP1 P08833: Insulin-like growth factor-binding protein 1		8	18	126	127	17	**0.85**
					128	129	14	**1.21**
					130	131	16	**0.87**
**626**	IGFBP2 P18065: Insulin-like growth factor-binding protein 2		12	28	126	127	27	**1.17**
					128	129	28	**3.54**
					130	131	26	**0.84**
**627**	IGFBP3 $P17936_{1,2}$: Insulin-like growth factor-binding protein 3	2/2	17	33	126	127	30	**1.41**
					128	129	30	**2.05**
					130	131	31	**1.06**
**628**	IGFBP4 $P22692_{1}$: Insulin-like growth factor-binding protein 4	1/2	8	16	126	127	13	**1.34**
					128	129	14	**2.31**
					130	131	14	**0.98**
**629**	IGFBP5 P24593: Insulin-like growth factor-binding protein 5		13	38	126	127	31	**1.03**
					128	129	31	**1.56**
					130	131	36	**1.05**
**630**	IGFBP6 P24592: Insulin-like growth factor-binding protein 6		6	10	126	127	9	**1.62**
					128	129	9	**3.44**
					130	131	9	**0.90**
**631**	IGFBP7 $Q16270_{1,2}$: Insulin-like growth factor-binding protein 7	2/2	10	15	126	127	15	**1.32**
					128	129	15	**1.98**
					130	131	14	**0.89**
**632**	IGHA1 P01876: Ig alpha-1 chain C region		13	139	126	127	47	**0.80**
					128	129	45	**1.84**
					130	131	40	**1.05**
**633**	IGHA2 P01877: Ig alpha-2 chain C region		9	95	126	127	4	**1.62**
					128	129	3	**3.51**
					130	131	4	**1.08**
**634**	IGHG1 P01857: Ig gamma-1 chain C region		14	358	126	127	162	**0.58**
					128	129	149	**1.93**
					130	131	166	**0.96**
**635**	IGHG2 P01859: Ig gamma-2 chain C region		10	171	126	127	30	**0.79**
					128	129	33	**2.14**
					130	131	31	**1.11**
**636**	IGHG3 P01860: Ig gamma-3 chain C region		12	220	126	127	32	**0.57**
					128	129	31	**1.58**
					130	131	31	**1.01**

#	protein	group	peptides	spectra	ch1	ch2	quant	ratio
**637**	IGHG4 P01861: Ig gamma-4 chain C region		9	110	126	127	20	**0.64**
					128	129	18	**1.81**
					130	131	17	**1.06**
**638**	IGHM P01871$_{1,2}$: Ig mu chain C region	2/2	15	115	126	127	43	**0.77**
					128	129	42	**1.14**
					130	131	38	**1.01**
**639**	IGHV1-2 P23083, A0A0C4DH31: Immunoglobulin heavy variable 1-2	2/2	2	14	126	127	4	**1.39**
					128	129	5	**2.68**
					130	131	5	**1.01**
**641**	IGHV1-69 P01742: Immunoglobulin heavy variable 1-69		4	14	126	127	5	**0.42**
					128	129	4	**1.79**
					130	131	5	**0.99**
**642**	IGHV3-13 P01766: Immunoglobulin heavy variable 3-13		1	1	126	127	1	**1.13**
					128	129	1	**2.06**
					130	131	1	**0.96**
**643**	IGHV3-30 P01768: Immunoglobulin heavy variable 3-30	1/2	2	3	126	127	2	**0.78**
					128	129	2	**1.46**
					130	131	2	**0.94**
**645**	IGHV3-7 P01780: Immunoglobulin heavy variable 3-7	1/5	2	3	126	127	1	**0.98**
					128	129	1	**0.95**
					130	131	1	**1.02**
**646**	IGHV3-9 P01782: Immunoglobulin heavy variable 3-9		1	2	126	127	2	**0.71**
					128	129	2	**1.91**
					130	131	2	**1.01**
**647**	IGHV4-34 P06331, IGHV4-39 P01824, IGHV4-59 P01825: Immunoglobulin heavy variable 4-34, Immunoglobulin heavy variable 4-39, ...	3/3	3	12	126	127	10	**0.45**
					128	129	12	**1.28**
					130	131	12	**1.00**
**648**	IGKC P01834: Ig kappa chain C region		5	110	126	127	99	**0.83**
					128	129	100	**1.95**
					130	131	99	**1.01**
**649**	IGKV1-17 P01599: Immunoglobulin kappa variable 1-17		3	9	126	127	2	**0.87**
					128	129	2	**2.04**
					130	131	2	**0.86**
**650**	IGKV1D-33 P01593, IGKV1-33 P01594: Immunoglobulin kappa variable 1D-33, Immunoglobulin kappa variable 1-33	2/2	1	1	126	127	1	**0.98**
					128	129	1	**3.29**
					130	131	1	**1.08**

#	protein	group	peptides	spectra	ch1	ch2	quant	ratio
**651**	IGKV1D-12 P01611, IGKV1D-39 P04432, IGKV1-39 P01597, . . . : Immunoglobulin kappa variable 1D-12, Immunoglobulin kappa variable 1D-39, . . .	4/4	2	8	126	127	1	**2.43**
					128	129	1	**1.39**
					130	131	1	**1.05**
**652**	IGKV1-5 P01602: Immunoglobulin kappa variable 1-5		2	10	126	127	3	**0.60**
					128	129	3	**1.35**
					130	131	3	**1.14**
**653**	IGKV2-30 P06310: Immunoglobulin kappa variable 2-30	1/2	3	15	126	127	7	**1.96**
					128	129	7	**2.31**
					130	131	7	**0.99**
**654**	IGKV2D-28 P01615, IGKV2D-40 P01614, A0A075B6P5: Immunoglobulin kappa variable 2D-28, Immunoglobulin kappa variable 2D-40	3/3	2	13	126	127	4	**0.77**
					128	129	4	**2.34**
					130	131	3	**1.03**
**655**	IGKV3-11 P04433: Immunoglobulin kappa variable 3-11		4	32	126	127	5	**0.74**
					128	129	6	**1.46**
					130	131	6	**1.12**
**656**	IGKV3-15 P01624: Immunoglobulin kappa variable 3-15		3	28	126	127	2	**0.64**
					128	129	2	**1.51**
					130	131	2	**1.16**
**657**	IGKV3-20 P01619: Immunoglobulin kappa variable 3-20		4	25	126	127	1	**1.03**
					128	129	1	**2.06**
					130	131	1	**1.32**
**658**	IGKV4-1 P06312: Immunoglobulin kappa variable 4-1		4	17	126	127	17	**1.11**
					128	129	13	**2.10**
					130	131	17	**1.02**
**659**	IGLC1 P0CG04, IGLL5 $B9A064_1$: Ig lambda-1 chain C regions, Immunoglobulin lambda-like polypeptide 5	2/2	6	190	126	127	29	**0.74**
					128	129	30	**2.23**
					130	131	30	**0.90**
**660**	IGLC2 P0CG05: Ig lambda-2 chain C regions	1/2	7	219	126	127	81	**0.75**
					128	129	83	**2.25**
					130	131	84	**0.95**
**662**	IGLC7 A0M8Q6: Ig lambda-7 chain C region		5	108	126	127	1	**0.87**
					128	129	1	**1.97**
					130	131	1	**0.84**
**663**	IGLV1-40 P01703: Immunoglobulin lambda variable 1-40		1	1	126	127	1	**1.02**
					128	129	1	**1.65**
					130	131	1	**1.17**

#	protein	group	peptides	spectra	ch1	ch2	quant	ratio
**664**	IGLV1-47 P01700: Immunoglobulin lambda variable 1-47	1/2	2	3	126	127	3	**1.03**
					128	129	3	**1.71**
					130	131	3	**1.14**
**665**	IGLV2-23 P01705: Immunoglobulin lambda variable 2-23		2	5	126	127	5	**0.88**
					128	129	5	**1.98**
					130	131	4	**0.90**
**666**	IGLV2-8 P01709: Immunoglobulin lambda variable 2-8		2	9	126	127	9	**1.03**
					128	129	8	**2.07**
					130	131	9	**0.90**
**667**	IGLV3-21 P80748: Immunoglobulin lambda variable 3-21		2	5	126	127	4	**0.57**
					128	129	5	**2.81**
					130	131	5	**1.18**
**668**	IGLV3-25 P01717: Immunoglobulin lambda variable 3-25		2	5	126	127	2	**0.87**
					128	129	2	**1.94**
					130	131	2	**0.93**
**669**	IGLV3-27 P01718: Immunoglobulin lambda variable 3-27		2	4	126	127	1	**1.29**
					128	129	1	**1.57**
					130	131	1	**0.97**
**670**	IGLV7-43 P04211: Immunoglobulin lambda variable 7-43		2	2	126	127	2	**0.80**
					128	129	2	**1.90**
					130	131	2	**1.12**
**671**	ILF3 Q12906$_{1-7}$: Interleukin enhancer-binding factor 3	7/7	2	2	126	127	2	**0.70**
					128	129	2	**0.42**
					130	131	2	**1.01**
**672**	IMMT Q16891$_{1,2,4}$: MICOS complex subunit MIC60	3/4	5	5	126	127	5	**7.41**
					128	129	4	**1.29**
					130	131	5	**0.73**
**673**	INHBA P08476: Inhibin beta A chain		2	2	126	127	2	**0.49**
					128	129	2	**1.13**
					130	131	2	**0.92**
**674**	INHBB P09529: Inhibin beta B chain		4	5	126	127	3	**1.24**
					128	129	5	**0.82**
					130	131	4	**1.03**
**675**	INHBC P55103: Inhibin beta C chain		9	20	126	127	16	**0.78**
					128	129	16	**0.84**
					130	131	17	**1.03**

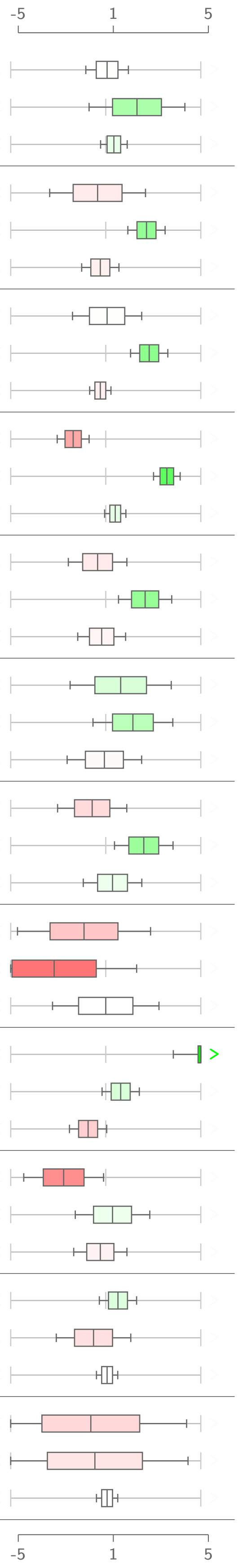

#	protein	group	peptides	spectra	ch1	ch2	quant	ratio
676	INHBE P58166: Inhibin beta E chain		5	9	126	127	9	0.97
					128	129	9	1.20
					130	131	9	1.06
677	IPO5 O00410$_{1,3}$: Importin-5	2/3	4	7	126	127	3	0.13
					128	129	3	0.16
					130	131	3	1.05
678	IQGAP1 P46940: Ras GTPase-activating-like protein IQGAP1	1/2	21	25	126	127	25	0.94
					128	129	23	0.49
					130	131	25	1.04
679	ISLR O14498: Immunoglobulin superfamily containing leucine-rich repeat protein		5	10	126	127	8	0.19
					128	129	10	0.85
					130	131	10	1.00
680	ISM1 B1AKI9: Isthmin-1		2	3	126	127	3	0.49
					128	129	3	0.87
					130	131	3	0.96
681	ITGB1 P05556$_{1}$: Integrin beta-1	1/5	8	8	126	127	7	0.75
					128	129	4	0.46
					130	131	6	0.88
682	ITGB2 P05107: Integrin beta-2		6	6	126	127	5	0.76
					128	129	6	0.74
					130	131	6	0.81
683	ITGBL1 O95965$_{1}$: Integrin beta-like protein 1	1/2	12	27	126	127	6	1.15
					128	129	4	1.62
					130	131	6	0.99
685	ITIH1 P19827$_{1}$: Inter-alpha-trypsin inhibitor heavy chain H1	1/3	17	78	126	127	68	0.28
					128	129	73	0.26
					130	131	68	1.07
686	ITIH2 P19823: Inter-alpha-trypsin inhibitor heavy chain H2		30	271	126	127	248	0.41
					128	129	261	0.23
					130	131	249	1.15
687	ITIH3 Q06033$_{1,2}$: Inter-alpha-trypsin inhibitor heavy chain H3	2/2	32	96	126	127	90	0.53
					128	129	96	0.24
					130	131	93	0.96
688	ITIH4 Q14624$_{1}$: Inter-alpha-trypsin inhibitor heavy chain H4		33	159	126	127	2	0.59
					128	129	2	0.76
					130	131	2	1.15

#	protein	group	peptides	spectra	ch1	ch2	quant	ratio
**689**	ITIH4 Q14624$_{2,3}$: Inter-alpha-trypsin inhibitor heavy chain H4	2/3	33	159	126	127	1	**0.14**
					128	129	2	**0.25**
					130	131	2	**0.97**
**690**	ITIH5 Q86UX2$_{1}$: Inter-alpha-trypsin inhibitor heavy chain H5	1/4	12	16	126	127	16	**0.84**
					128	129	16	**0.72**
					130	131	14	**0.82**
**691**	ITLN1 Q8WWA0: Intelectin-1		13	101	126	127	56	**0.73**
					128	129	55	**2.03**
					130	131	55	**1.02**
**692**	ITLN2 Q8WWU7$_{1,2}$: Intelectin-2	2/2	8	44	126	127	1	**3.61**
					128	129	1	**1.09**
					130	131	1	**0.60**
**693**	ITM2B Q9Y287$_{1,2}$: Integral membrane protein 2B	2/2	2	5	126	127	5	**0.62**
					128	129	4	**1.10**
					130	131	3	**1.42**
**694**	JCHAIN P01591: Immunoglobulin J chain		3	20	126	127	18	**0.66**
					128	129	20	**1.82**
					130	131	20	**1.10**
**695**	KAZALD1 Q96I82$_{1,2}$: Kazal-type serine protease inhibitor domain-containing protein 1	2/2	1	1	126	127	1	**0.48**
					128	129	1	**0.73**
					130	131	1	**1.08**
**696**	KCTD12 Q96CX2: BTB/POZ domain-containing protein KCTD12	1/2	7	9	126	127	5	**0.38**
					128	129	9	**0.29**
					130	131	7	**0.96**
**697**	KHDRBS1 Q07666$_{1,2}$: KH domain-containing, RNA-binding, signal transduction-associated protein 1	2/3	4	4	126	127	3	**1.59**
					128	129	4	**0.53**
					130	131	4	**0.96**
**698**	KHSRP Q92945: Far upstream element-binding protein 2		4	4	126	127	3	**1.24**
					128	129	3	**0.74**
					130	131	3	**0.93**
**699**	KIAA1107 Q9UPP5$_{1,2}$: Uncharacterized protein KIAA1107	2/2	3	10	126	127	7	**1.01**
					128	129	9	**3.76**
					130	131	8	**1.36**
**700**	KIF5B P33176: Kinesin-1 heavy chain		8	8	126	127	6	**0.50**
					128	129	8	**0.32**
					130	131	6	**0.89**

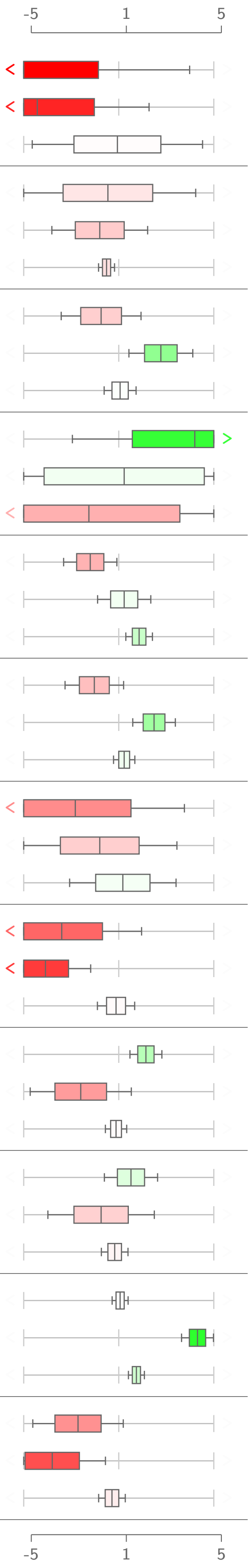

#	protein	group	peptides	spectra	ch1	ch2	quant	ratio	
701	KLK10 O43240: Kallikrein-10		1	1	126	127	1	**1.96**	
					128	129	1	**1.56**	
					130	131	1	**0.99**	
702	KLK14 Q9P0G3: Kallikrein-14		2	2	126	127	2	**0.87**	
					128	129	2	**0.96**	
					130	131	2	**0.96**	
703	KLKB1 P03952: Plasma kallikrein		30	61	126	127	59	**0.75**	
					128	129	51	**1.36**	
					130	131	58	**0.96**	
704	KMT2A $Q03164_{1-3}$: Histone-lysine N-methyltransferase 2A	3/3	7	44	126	127	36	**1.40**	
					128	129	38	**1.82**	
					130	131	41	**1.07**	
705	KNG1 $P01042_{1}$: Kininogen-1		30	181	126	127	6	**0.66**	
					128	129	8	**0.82**	
					130	131	8	**2.08**	
706	KNG1 $P01042_{2}$: Kininogen-1	1/2	26	193	126	127	21	**0.70**	
					128	129	20	**0.83**	
					130	131	21	**0.84**	
707	KPNB1 $Q14974_{1}$: Importin subunit beta-1	1/2	11	12	126	127	11	**0.72**	
					128	129	11	**0.39**	<
					130	131	11	**1.01**	
708	KPRP Q5T749: Keratinocyte proline-rich protein		3	3	126	127	3	**2.32**	
					128	129	3	**1.34**	
					130	131	3	**1.70**	
709	KRT1 P04264: Keratin, type II cytoskeletal 1	1/2	37	132	126	127	92	**3.78**	
					128	129	88	**1.76**	
					130	131	93	**4.79**	>
710	KRT10 P13645: Keratin, type I cytoskeletal 10	1/4	34	119	126	127	75	**4.42**	>
					128	129	78	**2.02**	
					130	131	79	**4.78**	>
711	KRT13 $P13646_{1}$: Keratin, type I cytoskeletal 13	1/3	16	34	126	127	5	**1.56**	
					128	129	5	**1.44**	
					130	131	6	**1.42**	
712	KRT14 P02533: Keratin, type I cytoskeletal 14		24	46	126	127	6	**5.02**	>
					128	129	6	**1.69**	
					130	131	5	**3.56**	

#	protein	group	peptides	spectra	ch1	ch2	quant	ratio
713	KRT15 P19012$_1$: Keratin, type I cytoskeletal 15	1/2	13	33	126	127	3	**0.55**
					128	129	3	**1.65**
					130	131	3	**0.97**
714	KRT16 P08779: Keratin, type I cytoskeletal 16		21	39	126	127	8	**4.94**
					128	129	10	**0.69**
					130	131	10	**2.06**
716	KRT18 P05783: Keratin, type I cytoskeletal 18		28	76	126	127	61	**4.58**
					128	129	62	**0.44**
					130	131	62	**1.01**
717	KRT19 P08727: Keratin, type I cytoskeletal 19	1/4	33	100	126	127	61	**4.72**
					128	129	63	**0.45**
					130	131	59	**1.04**
718	KRT2 P35908: Keratin, type II cytoskeletal 2 epidermal		34	78	126	127	37	**2.77**
					128	129	36	**2.21**
					130	131	42	**4.24**
720	KRT24 Q2M2I5: Keratin, type I cytoskeletal 24		5	18	126	127	1	**1.57**
					128	129	1	**1.41**
					130	131	1	**1.07**
721	KRT28 Q7Z3Y7: Keratin, type I cytoskeletal 28		7	32	126	127	1	**1.07**
					128	129	1	**0.80**
					130	131	1	**1.72**
722	KRT3 P12035: Keratin, type II cytoskeletal 3		8	26	126	127	1	**1.39**
					128	129	1	**0.83**
					130	131	1	**0.95**
723	KRT35 Q92764: Keratin, type I cuticular Ha5		3	22	126	127	4	**1.94**
					128	129	5	**1.41**
					130	131	7	**0.76**
725	KRT5 P13647: Keratin, type II cytoskeletal 5	1/6	27	69	126	127	23	**4.12**
					128	129	23	**0.95**
					130	131	23	**1.78**
728	KRT7 P08729: Keratin, type II cytoskeletal 7		28	80	126	127	44	**5.22**
					128	129	46	**0.32**
					130	131	46	**1.11**
731	KRT78 Q8N1N4$_1$: Keratin, type II cytoskeletal 78	1/2	5	16	126	127	2	**3.33**
					128	129	2	**1.96**
					130	131	2	**3.19**

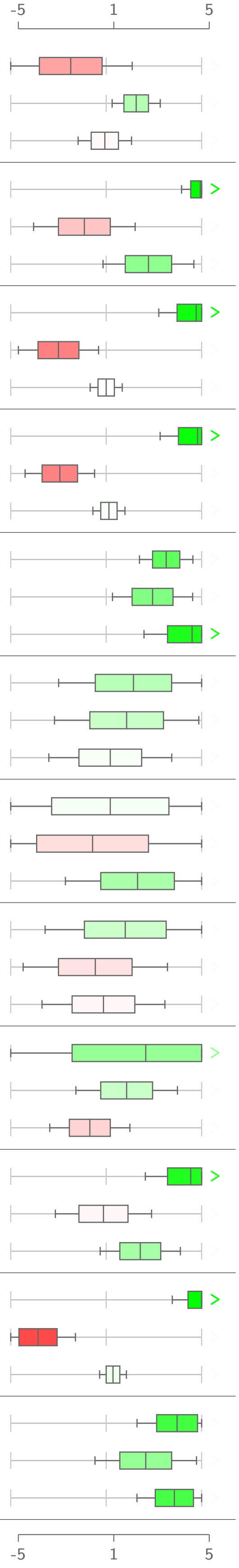

#	protein	group	peptides	spectra	ch1	ch2	quant	ratio
733	KRT8 P05787$_{1,2}$: Keratin, type II cytoskeletal 8	2/2	37	125	126	127	78	**4.94**
					128	129	73	**0.57**
					130	131	74	**1.07**
734	KRT9 P35527: Keratin, type I cytoskeletal 9		29	93	126	127	90	**2.73**
					128	129	89	**1.46**
					130	131	90	**3.97**
735	LAMA2 P24043: Laminin subunit alpha-2		34	57	126	127	49	**0.94**
					128	129	50	**1.47**
					130	131	42	**0.97**
736	LAMA3 Q16787$_{2,3}$: Laminin subunit alpha-3	2/2	3	6	126	127		
					128	129	2	**1.16**
					130	131	1	**2.12**
737	LAMA4 Q16363$_{1,2}$: Laminin subunit alpha-4	2/2	25	32	126	127	31	**0.54**
					128	129	31	**0.56**
					130	131	30	**0.96**
738	LAMA5 O15230$_{1}$: Laminin subunit alpha-5		13	18	126	127	13	**1.45**
					128	129	11	**1.23**
					130	131	13	**1.08**
739	LAMB1 P07942: Laminin subunit beta-1		31	42	126	127	40	**0.51**
					128	129	39	**0.73**
					130	131	39	**1.02**
740	LAMB2 P55268: Laminin subunit beta-2		16	17	126	127	16	**0.66**
					128	129	15	**0.85**
					130	131	16	**1.04**
741	LAMC1 P11047: Laminin subunit gamma-1		35	55	126	127	47	**0.76**
					128	129	51	**0.88**
					130	131	48	**1.03**
742	LASP1 Q14847$_{1}$: LIM and SH3 domain protein 1		3	6	126	127	4	**1.54**
					128	129	4	**1.95**
					130	131	3	**1.03**
743	LBP P18428: Lipopolysaccharide-binding protein		10	89	126	127	79	**0.23**
					128	129	89	**0.27**
					130	131	85	**1.02**
744	LCAT P04180: Phosphatidylcholine-sterol acyltransferase	1/2	10	26	126	127	22	**0.22**
					128	129	24	**0.41**
					130	131	26	**1.09**

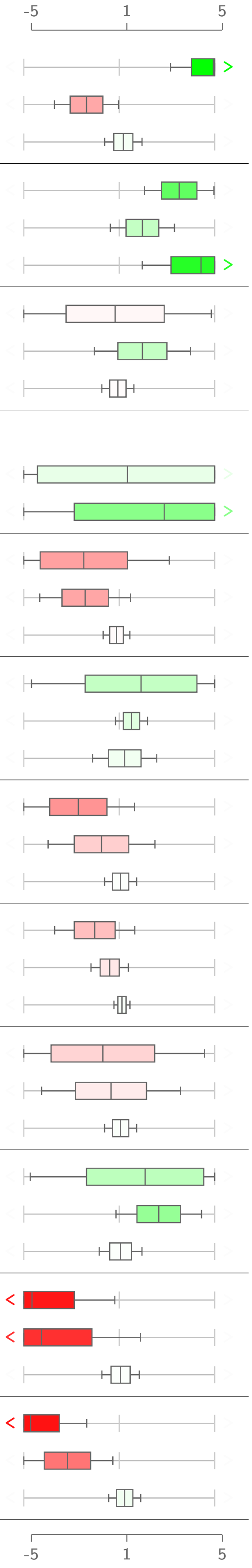

#	protein	group	peptides	spectra	ch1	ch2	quant	ratio
**745**	LCN1 P31025: Lipocalin-1		2	2	126	127	2	**0.86**
					128	129	2	**3.91**
					130	131	2	**1.22**
**746**	LCP1 $P13796_{1}$: Plastin-2	1/3	26	66	126	127	38	**0.30**
					128	129	42	**0.15**
					130	131	45	**0.88**
**747**	LDHA $P00338_{1,3}$: L-lactate dehydrogenase A chain	2/8	4	5	126	127	5	**4.17**
					128	129	5	**0.50**
					130	131	4	**1.49**
**748**	LDLR $P01130_{1,5}$: Low-density lipoprotein receptor	2/5	6	11	126	127	5	**0.87**
					128	129	5	**1.46**
					130	131	6	**1.06**
**749**	LECT2 O14960: Leukocyte cell-derived chemotaxin-2		4	6	126	127	5	**0.77**
					128	129	6	**2.74**
					130	131	6	**1.03**
**750**	LEFTY1 O75610, LEFTY2 $O00292_{1}$: Left-right determination factor 1, Left-right determination factor 2	2/3	3	4	126	127	4	**0.76**
					128	129	4	**0.88**
					130	131	4	**1.03**
**751**	LEP P41159: Leptin		3	8	126	127	7	**1.34**
					128	129	7	**1.16**
					130	131	7	**1.24**
**752**	LFNG $Q8NES3_{4}$: Beta-1,3-N-acetylglucosaminyltransferase lunatic fringe	1/4	4	5	126	127	4	**0.88**
					128	129	3	**0.80**
					130	131	3	**0.90**
**753**	LGALS1 P09382: Galectin-1		8	28	126	127	24	**1.14**
					128	129	27	**1.71**
					130	131	27	**0.93**
**754**	LGALS3 P17931: Galectin-3		4	4	126	127	4	**1.18**
					128	129	4	**1.05**
					130	131	3	**1.09**
**755**	LGALS3BP Q08380: Galectin-3-binding protein		14	45	126	127	44	**0.56**
					128	129	45	**0.69**
					130	131	44	**1.23**
**756**	LGALSL Q3ZCW2: Galectin-related protein		1	1	126	127	1	**0.99**
					128	129	1	**0.88**
					130	131	1	**0.88**

#	protein	group	peptides	spectra	ch1	ch2	quant	ratio
757	LGI2 Q8N0V4: Leucine-rich repeat LGI family member 2		2	3	126	127	3	**8.76**
					128	129	3	**1.29**
					130	131	3	**0.80**
758	LIPG Q9Y5X9$_{1,2}$: Endothelial lipase	2/2	2	2	126	127	2	**1.49**
					128	129	2	**0.88**
					130	131	2	**1.48**
759	LMAN1 P49257: Protein ERGIC-53		9	10	126	127	9	**1.08**
					128	129	10	**1.27**
					130	131	9	**0.91**
760	LMAN2 Q12907: Vesicular integral-membrane protein VIP36		3	3	126	127	3	**0.73**
					128	129	3	**0.49**
					130	131	3	**0.93**
761	LMNA P02545$_{1-3,6}$: Prelamin-A/C [Cleaved into: Lamin-A/C	4/6	19	28	126	127	27	**4.45**
					128	129	25	**2.03**
					130	131	24	**1.08**
762	LMNB1 P20700: Lamin-B1		8	8	126	127	8	**1.89**
					128	129	8	**0.63**
					130	131	7	**0.76**
763	LNPEP Q9UIQ6$_{1-3}$: Leucyl-cystinyl aminopeptidase	3/3	1	1	126	127		
					128	129	1	**0.67**
					130	131	1	**0.92**
764	LOX P28300: Protein-lysine 6-oxidase		9	27	126	127	18	**1.15**
					128	129	18	**1.37**
					130	131	20	**0.97**
765	LOXL1 Q08397: Lysyl oxidase homolog 1		17	48	126	127	38	**0.60**
					128	129	37	**0.88**
					130	131	39	**1.00**
766	LOXL2 Q9Y4K0: Lysyl oxidase homolog 2		15	23	126	127	16	**0.52**
					128	129	16	**0.93**
					130	131	16	**0.97**
767	LPA P08519: Apolipoprotein(a)		23	43	126	127	38	**0.86**
					128	129	42	**0.28**
					130	131	39	**0.98**
768	LPAR3 Q9UBY5: Lysophosphatidic acid receptor 3		5	218	126	127	137	**2.04**
					128	129	152	**2.02**
					130	131	128	**1.08**

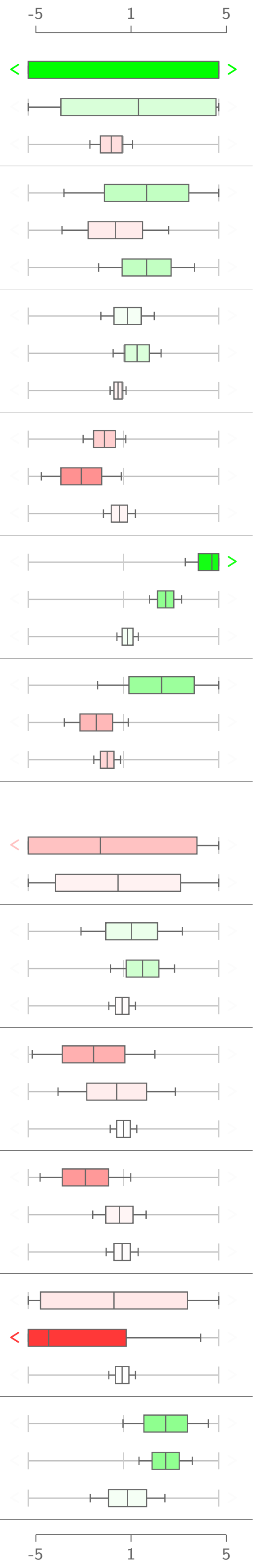

#	protein	group	peptides	spectra	ch1	ch2	quant	ratio
**769**	LPL P06858: Lipoprotein lipase		9	11	126	127	11	**0.86**
					128	129	9	**0.64**
					130	131	10	**0.91**
**770**	LRG1 P02750: Leucine-rich alpha-2-glycoprotein		3	4	126	127	3	**2.25**
					128	129	3	**1.92**
					130	131	3	**0.89**
**771**	LRP1 Q07954$_1$: Prolow-density lipoprotein receptor-related protein 1		52	129	126	127	107	**1.39**
					128	129	105	**1.27**
					130	131	107	**1.03**
**772**	LRP1B Q9NZR2: Low-density lipoprotein receptor-related protein 1B		18	155	126	127	41	**2.55**
					128	129	45	**1.77**
					130	131	46	**0.97**
**773**	LRP8 Q14114$_{1,3}$: Low-density lipoprotein receptor-related protein 8	2/4	9	25	126	127	20	**1.88**
					128	129	22	**1.30**
					130	131	21	**0.98**
**774**	LRRC17 Q8N6Y2$_1$: Leucine-rich repeat-containing protein 17		4	5	126	127	4	**0.55**
					128	129	5	**1.38**
					130	131	5	**1.17**
**775**	LRRC57 Q8N9N7: Leucine-rich repeat-containing protein 57		2	2	126	127	2	**0.33**
					128	129	2	**0.17**
					130	131	2	**0.85**
**776**	LRRFIP1 Q32MZ4$_{1-3}$: Leucine-rich repeat flightless-interacting protein 1	3/3	3	3	126	127	2	**2.50**
					128	129	2	**1.90**
					130	131	2	**1.43**
**777**	LRRFIP1 Q32MZ4$_4$: Leucine-rich repeat flightless-interacting protein 1		2	2	126	127	1	**1.75**
					128	129	1	**1.52**
					130	131	1	**0.98**
**778**	LSP1 P33241$_{1,3}$: Lymphocyte-specific protein 1	2/3	3	4	126	127	3	**0.51**
					128	129	4	**0.84**
					130	131	4	**0.88**
**779**	LSR Q86X29$_{1,4}$: Lipolysis-stimulated lipoprotein receptor	2/6	4	9	126	127	9	**1.43**
					128	129	8	**1.40**
					130	131	9	**1.04**
**780**	LTBP1 Q14766$_{1,4}$: Latent-transforming growth factor beta-binding protein 1	2/4	34	81	126	127	10	**0.56**
					128	129	11	**1.15**
					130	131	10	**0.89**

#	protein	group	peptides	spectra	ch1	ch2	quant	ratio
**781**	LTBP1 Q14766$_{3}$: Latent-transforming growth factor beta-binding protein 1		29	69	126	127	2	**0.45**
					128	129	2	**1.19**
					130	131	2	**0.90**
**782**	LTBP2 Q14767: Latent-transforming growth factor beta-binding protein 2		38	90	126	127	74	**1.21**
					128	129	78	**1.33**
					130	131	82	**1.00**
**783**	LTBP3 Q9NS15$_{1}$: Latent-transforming growth factor beta-binding protein 3	1/2	10	12	126	127	11	**0.73**
					128	129	11	**0.69**
					130	131	10	**0.92**
**784**	LTBP4 Q8N2S1$_{2}$: Latent-transforming growth factor beta-binding protein 4	1/4	37	61	126	127	57	**1.41**
					128	129	59	**1.29**
					130	131	60	**1.04**
**785**	LTF P02788$_{1,2}$: Lactotransferrin	2/2	30	44	126	127	38	**1.53**
					128	129	44	**0.60**
					130	131	41	**0.94**
**786**	LUM P51884: Lumican		14	160	126	127	153	**0.47**
					128	129	150	**1.64**
					130	131	149	**1.08**
**787**	LXN Q9BS40: Latexin		2	3	126	127	3	**2.10**
					128	129	3	**1.40**
					130	131	3	**0.82**
**788**	LYPLA1 O75608$_{1,2}$: Acyl-protein thioesterase 1	2/2	3	4	126	127	2	**0.60**
					128	129	3	**0.39**
					130	131	2	**1.05**
**789**	LYPLA2 O95372: Acyl-protein thioesterase 2		2	2	126	127	2	**0.37**
					128	129	2	**0.27**
					130	131	2	**1.02**
**790**	LYZ P61626: Lysozyme C		12	399	126	127	392	**1.42**
					128	129	385	**3.09**
					130	131	359	**0.91**
**791**	M6PR P20645: Cation-dependent mannose-6-phosphate receptor		1	1	126	127	1	**2.14**
					128	129	1	**0.32**
					130	131	1	**0.66**
**792**	MACF1 Q9UPN3$_{1}$: Microtubule-actin cross-linking factor 1, isoforms 1/2/3/5	1/5	8	18	126	127	16	**1.60**
					128	129	14	**2.38**
					130	131	9	**1.13**

#	protein	group	peptides	spectra	ch1	ch2	quant	ratio
**793**	MAN1A1 P33908$_1$: Mannosyl-oligosaccharide 1,2-alpha-mannosidase IA	1/2	17	22	126	127	18	**0.38**
					128	129	20	**0.43**
					130	131	20	**0.88**
**794**	MAP1B P46821: Microtubule-associated protein 1B		5	5	126	127	3	**0.43**
					128	129	3	**0.31**
					130	131	5	**0.72**
**795**	MAP7 Q14244$_{1,2,4,6,7}$: Ensconsin	5/10	3	3	126	127	2	**2.47**
					128	129	2	**1.38**
					130	131	2	**1.25**
**796**	MAP7D3 Q8IWC1$_{1-4}$: MAP7 domain-containing protein 3	4/4	2	2	126	127	1	**18.03**
					128	129	1	**2.20**
					130	131	1	**0.52**
**797**	MAPRE1 Q15691: Microtubule-associated protein RP/EB family member 1		2	2	126	127	2	**0.63**
					128	129	2	**0.56**
					130	131	2	**1.05**
**798**	MARCKS P29966: Myristoylated alanine-rich C-kinase substrate		3	5	126	127	4	**3.21**
					128	129	4	**2.34**
					130	131	5	**0.80**
**799**	MARCO Q9UEW3$_{1,2}$: Macrophage receptor MARCO	2/2	4	4	126	127	4	**3.88**
					128	129	3	**1.24**
					130	131	3	**1.13**
**800**	MASP1 P48740$_1$: Mannan-binding lectin serine protease 1	1/2	12	73	126	127	13	**0.74**
					128	129	13	**0.68**
					130	131	11	**1.39**
**801**	MASP1 P48740$_{2,4}$: Mannan-binding lectin serine protease 1	2/2	10	62	126	127	2	**0.30**
					128	129	2	**0.64**
					130	131	2	**1.11**
**802**	MASP2 O00187$_1$: Mannan-binding lectin serine protease 2		8	14	126	127	2	**0.69**
					128	129	2	**0.66**
					130	131	2	**1.26**
**803**	MASP2 O00187$_2$: Mannan-binding lectin serine protease 2		7	13	126	127	1	**1.32**
					128	129	1	**1.44**
					130	131	1	**1.03**
**804**	MATN2 O00339$_{1-3}$: Matrilin-2	3/4	5	6	126	127	5	**1.62**
					128	129	3	**0.62**
					130	131	5	**0.92**

#	protein	group	peptides	spectra	ch1	ch2	quant	ratio
**805**	MATR3 P43243$_1$: Matrin-3	1/2	8	10	126	127	9	**0.94**
					128	129	8	**0.38**
					130	131	10	**1.03**
**806**	MB P02144: Myoglobin		6	10	126	127	10	**1.59**
					128	129	10	< **0.35**
					130	131	8	**1.40**
**807**	MDK P21741$_1$: Midkine		3	3	126	127	3	**0.96**
					128	129	3	**1.21**
					130	131	3	**1.26**
**808**	MDN1 Q9NU22: Midasin		3	17	126	127	14	**1.30**
					128	129	13	**1.46**
					130	131	16	**1.02**
**809**	MEGF6 O75095$_1$: Multiple epidermal growth factor-like domains protein 6	1/2	8	13	126	127	11	**1.50**
					128	129	11	**1.74**
					130	131	11	**1.01**
**810**	METRNL Q641Q3$_1$: Meteor-in-like protein	1/2	6	6	126	127	5	**0.51**
					128	129	6	**0.89**
					130	131	6	**1.09**
**811**	MFAP2 P55001$_{1-3}$: Microfibrillar-associated protein 2	3/3	3	3	126	127	3	**0.50**
					128	129	3	**0.77**
					130	131	3	**1.10**
**812**	MFAP4 P55083$_{1,2}$: Microfibril-associated glycoprotein 4	2/2	7	124	126	127	119	**0.41**
					128	129	105	< **0.39**
					130	131	118	**0.89**
**813**	MFAP5 Q13361$_{1,2}$: Microfibrillar-associated protein 5	2/2	6	14	126	127	11	**0.48**
					128	129	11	**0.95**
					130	131	11	**1.19**
**814**	MFN1 Q8IWA4$_{1,3}$: Mitofusin-1	2/2	2	3	126	127	3	**1.93**
					128	129	3	**1.49**
					130	131	3	**1.36**
**815**	MGAT1 P26572: Alpha-1,3-mannosyl-glycoprotein 2-beta-N-acetylglucosaminyltransferase		10	12	126	127	11	**0.51**
					128	129	12	< **0.41**
					130	131	12	**1.02**
**816**	MGP P08493$_{1,2}$: Matrix Gla protein	2/2	5	251	126	127	232	**1.03**
					128	129	231	**1.51**
					130	131	228	**0.83**

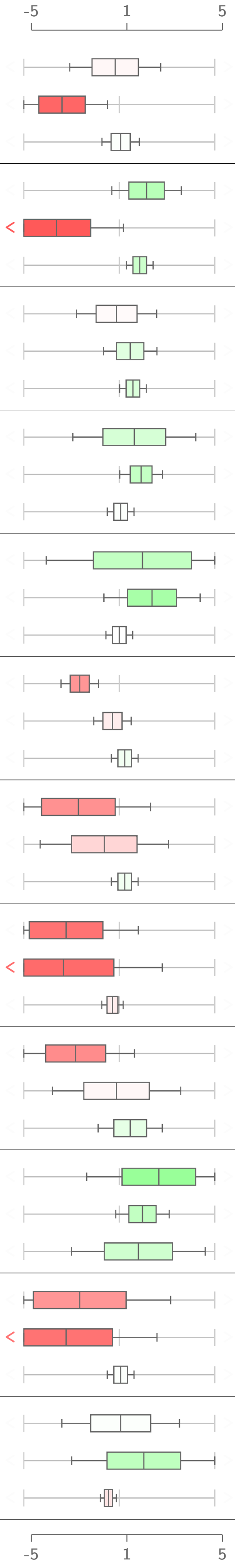

#	protein	group	peptides	spectra	ch1	ch2	quant	ratio
**817**	MIA3 Q5JRA6$_{1,2}$: Melanoma inhibitory activity protein 3	2/2	2	2	126	127	2	**0.68**
					128	129	2	**1.13**
					130	131	2	**1.10**
**818**	MINPP1 Q9UNW1$_{1}$: Multiple inositol polyphosphate phosphatase 1	1/4	5	6	126	127	5	**0.88**
					128	129	5	**0.38**
					130	131	5	**0.99**
**819**	MLEC Q14165: Malectin		4	4	126	127	3	**0.68**
					128	129	4	**0.83**
					130	131	4	**1.08**
**820**	MMP14 P50281: Matrix metalloproteinase-14		5	10	126	127	10	**2.39**
					128	129	10	**1.88**
					130	131	9	**0.96**
**821**	MMP2 P08253$_{1}$: 72 kDa type IV collagenase	1/3	18	30	126	127	29	**0.46**
					128	129	29	**2.09**
					130	131	28	**0.93**
**822**	MMP23A O75900$_{1-3}$: Matrix metalloproteinase-23	3/3	2	4	126	127	4	**0.66**
					128	129	4	**0.84**
					130	131	4	**1.04**
**823**	MMRN2 Q9H8L6: Multimerin-2		4	5	126	127	5	**1.17**
					128	129	5	**1.66**
					130	131	4	**1.08**
**824**	MNDA P41218: Myeloid cell nuclear differentiation antigen		3	3	126	127	3	**0.82**
					128	129	3	**0.38**
					130	131	3	**0.88**
**825**	MPO P05164$_{1-3}$: Myeloperoxidase	3/3	9	14	126	127	11	**1.68**
					128	129	11	**0.50**
					130	131	10	**1.17**
**826**	MPST P25325$_{1,2}$: 3-mercaptopyruvate sulfurtransferase	2/2	2	2	126	127	2	**0.62**
					128	129	2	**0.60**
					130	131	2	**0.96**
**827**	MRC2 Q9UBG0: C-type mannose receptor 2		3	3	126	127	3	**0.74**
					128	129	3	**1.41**
					130	131	3	**0.86**
**828**	MROH1 Q8NDA8$_{1,2}$: Maestro heat-like repeat-containing protein family member 1	2/2	2	2	126	127	2	**0.70**
					128	129	2	**0.70**
					130	131	2	**0.92**

#	protein	group	peptides	spectra	ch1	ch2	quant	ratio
829	MSLN Q13421$_{1-4}$: Mesothelin	4/4	13	32	126	127	29	**0.93**
					128	129	28	**1.35**
					130	131	30	**1.05**
830	MSN P26038: Moesin	1/3	12	18	126	127	17	**2.56**
					128	129	17	**0.67**
					130	131	17	**0.81**
831	MSR1 P21757$_1$: Macrophage scavenger receptor types I and II	1/3	7	24	126	127	17	**1.42**
					128	129	18	**0.89**
					130	131	15	**1.08**
832	MST1 P26927: Hepatocyte growth factor-like protein	1/3	9	11	126	127	11	**0.70**
					128	129	8	**1.49**
					130	131	10	**1.00**
833	MTHFD1 P11586: C-1-tetrahydrofolate synthase, cytoplasmic		7	8	126	127	8	**0.80**
					128	129	8	**0.46**
					130	131	7	**1.04**
834	MUC2 Q02817: Mucin-2		7	71	126	127	67	**1.29**
					128	129	61	**2.78**
					130	131	67	**1.14**
835	MVP Q14764: Major vault protein		5	5	126	127	5	**1.89**
					128	129	3	**0.51**
					130	131	4	**0.95**
836	MXRA5 Q9NR99: Matrix-remodeling-associated protein 5		54	80	126	127	75	**0.39**
					128	129	77	**0.71**
					130	131	76	**0.95**
837	MXRA7 P84157$_1$: Matrix-remodeling-associated protein 7	1/2	3	4	126	127		
					128	129	1	**0.72**
					130	131	1	**0.96**
838	MXRA7 P84157$_2$: Matrix-remodeling-associated protein 7		3	4	126	127	1	**0.04**
					128	129	1	**1.75**
					130	131	1	**1.34**
839	MYH1 P12882: Myosin-1	1/4	5	5	126	127	4	**1.87**
					128	129	4	**1.72**
					130	131	3	**0.88**
840	MYH10 P35580$_{4,5}$: Myosin-10	2/5	31	40	126	127	16	**0.54**
					128	129	17	**0.24**
					130	131	17	**1.05**

#	protein	group	peptides	spectra	ch1	ch2	quant	ratio
841	MYH11 P35749$_{1-4}$: Myosin-11	4/4	17	26	126	127	1	**7.33**
					128	129	1	**1.47**
					130	131	1	**0.70**
842	MYH14 Q7Z406$_{1,2,6}$: Myosin-14	3/5	8	12	126	127	2	**0.57**
					128	129	2	**0.35**
					130	131	2	**0.88**
844	MYH7 P12883: Myosin-7		6	6	126	127	1	**0.54**
					128	129	1	**0.99**
					130	131	1	**0.95**
845	MYH9 P35579$_{1}$: Myosin-9	1/2	68	122	126	127	91	**0.53**
					128	129	94	**0.25**
					130	131	92	**0.93**
846	MYL1 P05976$_{1}$: Myosin light chain 1/3, skeletal muscle isoform	1/2	10	17	126	127	17	**1.26**
					128	129	16	**1.16**
					130	131	15	**1.34**
847	MYL12A P19105, MYL12B O14950: Myosin regulatory light chain 12A, Myosin regulatory light chain 12B	2/2	6	13	126	127	4	**1.21**
					128	129	5	**0.64**
					130	131	5	**1.04**
848	MYL2 P10916: Myosin regulatory light chain 2, ventricular/cardiac muscle isoform	1/3	8	21	126	127	20	**1.33**
					128	129	18	**0.94**
					130	131	20	**1.38**
849	MYL3 P08590: Myosin light chain 3		7	10	126	127	10	**0.84**
					128	129	10	**0.61**
					130	131	8	**1.60**
850	MYL6 P60660$_{1,2}$: Myosin light polypeptide 6	2/3	7	13	126	127	13	**1.07**
					128	129	12	**0.63**
					130	131	12	**1.05**
851	MYL9 P24844$_{1}$: Myosin regulatory light polypeptide 9	1/2	5	11	126	127	3	**0.30**
					128	129	3	**0.91**
					130	131	3	**1.25**
852	MYLK Q15746$_{1-7,11}$: Myosin light chain kinase, smooth muscle	8/11	6	9	126	127	7	**0.35**
					128	129	7	**0.88**
					130	131	9	**1.06**
853	MYLPF Q96A32: Myosin regulatory light chain 2, skeletal muscle isoform		12	23	126	127	22	**1.25**
					128	129	23	**0.87**
					130	131	21	**1.48**

#	protein	group	peptides	spectra	ch1	ch2	quant	ratio
854	MYOC Q99972: Myocilin		12	15	126	127	15	**0.60**
					128	129	14	**1.33**
					130	131	14	**0.92**
855	NAMPT P43490: Nicotinamide phosphoribosyltransferase		3	3	126	127	3	**0.79**
					128	129	3	**0.17**
					130	131	3	**0.88**
856	NANS Q9NR45: Sialic acid synthase		3	5	126	127	4	**0.58**
					128	129	3	**0.51**
					130	131	3	**1.09**
857	NAP1L1 P55209$_{1-3}$: Nucleosome assembly protein 1-like 1	3/5	2	3	126	127	3	**0.47**
					128	129	3	**0.57**
					130	131	3	**1.15**
858	NAPA P54920: Alpha-soluble NSF attachment protein		4	6	126	127	4	**0.67**
					128	129	5	**0.53**
					130	131	6	**0.88**
859	NASP P49321$_{1,3,4}$: Nuclear autoantigenic sperm protein	3/3	2	3	126	127	3	**0.73**
					128	129	3	**0.92**
					130	131	3	**0.90**
860	NBL1 P41271$_{1,2}$: Neuroblastoma suppressor of tumorigenicity 1	2/2	2	4	126	127	4	**1.22**
					128	129	4	**5.89**
					130	131	4	**1.12**
861	NCF1 P14598$_{1}$, NCF1B A6NI72, NCF1C A8MVU1: Neutrophil cytosol factor 1, Putative neutrophil cytosol factor 1B, ...	3/3	2	2	126	127	2	**0.50**
					128	129	2	**0.41**
					130	131	2	**0.74**
862	NCL P19338: Nucleolin		13	14	126	127	12	**1.62**
					128	129	14	**0.81**
					130	131	14	**1.09**
863	NDRG1 Q92597$_{1-3}$: Protein NDRG1	3/3	2	2	126	127	2	**0.46**
					128	129	2	**0.54**
					130	131	2	**1.05**
864	NECAP2 Q9NVZ3$_{1,2}$: Adaptin ear-binding coat-associated protein 2	2/4	5	6	126	127	3	**0.32**
					128	129	6	**0.37**
					130	131	6	**0.79**
865	NECTIN2 Q92692$_{1,2}$: Nectin-2	2/2	2	2	126	127	2	**0.26**
					128	129	2	**0.42**
					130	131	2	**0.69**

#	protein	group	peptides	spectra	ch1	ch2	quant	ratio
**866**	NEFH P12036$_{1,2}$: Neurofilament heavy polypeptide	2/2	4	9	126	127	1	**0.35**
					128	129	1	**1.78**
					130	131	1	**0.70**
**867**	NES P48681: Nestin		6	10	126	127	8	**1.70**
					128	129	8	**1.66**
					130	131	9	**1.07**
**868**	NEXN Q0ZGT2$_{1-4}$: Nexilin	4/4	2	2	126	127	2	**1.77**
					128	129	2	**1.60**
					130	131	2	**1.03**
**869**	NFKB1 P19838$_{1-3}$: Nuclear factor NF-kappa-B p105 subunit	3/3	3	3	126	127	3	**0.83**
					128	129	3	**0.57**
					130	131	3	**0.92**
**870**	NID1 P14543$_{1}$: Nidogen-1	1/2	16	30	126	127	30	**1.21**
					128	129	30	**1.25**
					130	131	30	**1.11**
**871**	NID2 Q14112$_{1,2}$: Nidogen-2	2/2	11	13	126	127	9	**0.76**
					128	129	10	**1.14**
					130	131	9	**1.04**
**872**	NKTR P30414: NK-tumor recognition protein		2	3	126	127	3	**7.81**
					128	129	3	**3.62**
					130	131	3	**1.04**
**873**	NNMT P40261: Nicotinamide N-methyltransferase		1	1	126	127	1	**0.14**
					128	129	1	**0.16**
					130	131	1	**1.21**
**874**	NONO Q15233$_{1,2}$: Non-POU domain-containing octamer-binding protein	2/2	4	4	126	127	2	**1.59**
					128	129	2	**0.85**
					130	131	2	**1.16**
**875**	NOTCH3 Q9UM47: Neurogenic locus notch homolog protein 3		6	8	126	127	6	**0.82**
					128	129	5	**0.50**
					130	131	6	**0.83**
**876**	NPM1 P06748$_{1,3}$: Nucleophosmin	2/3	8	11	126	127	11	**2.25**
					128	129	11	**1.01**
					130	131	10	**1.19**
**877**	NPPA P01160: Natriuretic peptides A		4	5	126	127	4	**0.94**
					128	129	4	**1.70**
					130	131	5	**0.87**

#	protein	group	peptides	spectra	ch1	ch2	quant	ratio
**878**	NRP2 O60462$_{4,5}$: Neuropilin-2	2/6	13	14	126	127	13	**0.41**
					128	129	13	**0.60**
					130	131	13	**1.09**
**879**	NSF P46459$_{1,2}$: Vesicle-fusing ATPase	2/2	3	3	126	127	3	**0.71**
					128	129	3	**0.36**
					130	131	3	**1.10**
**880**	NUCB1 Q02818: Nucleobindin-1		31	112	126	127	102	**0.53**
					128	129	100	**1.07**
					130	131	106	**0.96**
**881**	NUCB2 P80303$_{1,2}$: Nucleobindin-2	2/2	16	30	126	127	25	**2.35**
					128	129	26	**1.40**
					130	131	26	**0.92**
**882**	NUCKS1 Q9H1E3$_{1}$: Nuclear ubiquitous casein and cyclin-dependent kinase substrate 1		6	10	126	127	8	**1.20**
					128	129	9	**2.34**
					130	131	8	**1.21**
**883**	NUDC Q9Y266: Nuclear migration protein nudC		2	57	126	127	44	**3.19**
					128	129	42	**1.80**
					130	131	39	**0.82**
**884**	NUMA1 Q14980$_{1-5}$: Nuclear mitotic apparatus protein 1	5/5	2	140	126	127	1	**0.82**
					128	129	1	**0.81**
					130	131	1	**0.79**
**885**	OAF Q86UD1: Out at first protein homolog		6	14	126	127	14	**0.51**
					128	129	14	**0.66**
					130	131	13	**1.03**
**886**	OGN P20774: Mimecan		8	32	126	127	31	**0.39**
					128	129	28	**0.74**
					130	131	31	**0.82**
**887**	OIT3 Q8WWZ8$_{1}$: Oncoprotein-induced transcript 3 protein		3	3	126	127	3	**0.63**
					128	129	3	**0.52**
					130	131	3	**1.03**
**888**	OLFML1 Q6UWY5: Olfactomedin-like protein 1		8	14	126	127	12	**0.46**
					128	129	14	**0.80**
					130	131	13	**0.81**
**889**	OLFML2B Q68BL8$_{1}$: Olfactomedin-like protein 2B		3	4	126	127	3	**1.27**
					128	129	4	**0.59**
					130	131	4	**0.86**

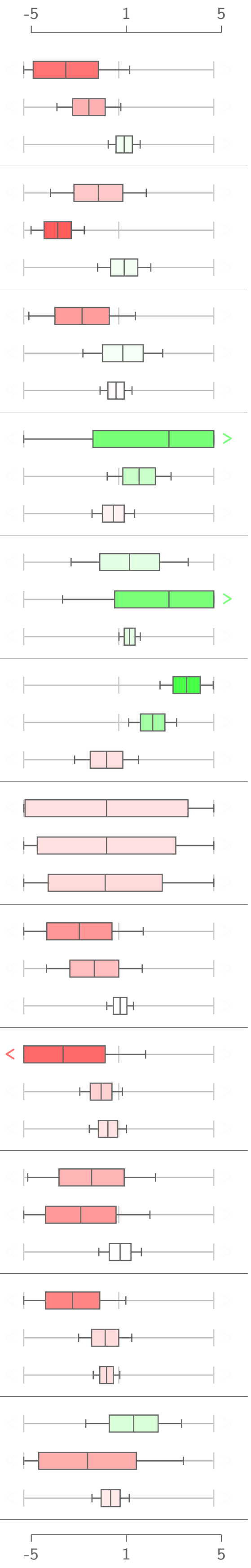

#	protein	group	peptides	spectra	ch1	ch2	quant	ratio
**890**	OLFML3 Q9NRN5$_1$: Olfactomedin-like protein 3	1/3	17	64	126	127	62	**0.51**
					128	129	59	**0.80**
					130	131	61	**0.84**
**891**	OMD Q99983: Osteomodulin		3	6	126	127	6	**0.33**
					128	129	6	**0.40**
					130	131	6	**1.16**
**893**	ORM1 P02763: Alpha-1-acid glycoprotein 1		9	19	126	127	4	**13.28**
					128	129	4	**7.09**
					130	131	4	**1.57**
**894**	ORM2 P19652: Alpha-1-acid glycoprotein 2		9	19	126	127	4	**3.16**
					128	129	3	**0.85**
					130	131	5	**1.09**
**895**	P04220: Ig mu heavy chain disease protein		11	84	126	127	12	**0.64**
					128	129	12	**0.91**
					130	131	12	**0.80**
**896**	P3H1 Q32P28$_{1,3,4}$: Prolyl 3-hydroxylase 1	3/3	7	7	126	127	6	**1.27**
					128	129	5	**0.78**
					130	131	6	**1.00**
**897**	P4HA1 P13674$_{1-3}$: Prolyl 4-hydroxylase subunit alpha-1	3/3	2	3	126	127	3	**1.43**
					128	129	3	**0.94**
					130	131	3	**0.95**
**898**	P4HA2 O15460$_{1,2}$: Prolyl 4-hydroxylase subunit alpha-2	2/2	3	3	126	127	3	**0.83**
					128	129	3	**0.97**
					130	131	3	**1.02**
**899**	P4HB P07237: Protein disulfide-isomerase		24	35	126	127	29	**1.72**
					128	129	30	**0.92**
					130	131	31	**1.02**
**900**	PABPC1 P11940$_{1,2}$: Polyadenylate-binding protein 1	2/3	5	8	126	127	8	**3.17**
					128	129	8	**0.84**
					130	131	8	**1.11**
**901**	PACSIN2 Q9UNF0$_1$: Protein kinase C and casein kinase substrate in neurons protein 2	1/2	6	8	126	127	6	**0.62**
					128	129	6	**0.99**
					130	131	7	**1.03**
**902**	PAK2 Q13177: Serine/threonine-protein kinase PAK 2		2	2	126	127	2	**0.27**
					128	129	2	**0.17**
					130	131	2	**0.89**

#	protein	group	peptides	spectra	ch1	ch2	quant	ratio
**903**	PAM P19021$_{1,3,5}$: Peptidyl-glycine alpha-amidating monooxygenase	3/6	15	19	126	127	16	**0.85**
					128	129	17	**1.05**
					130	131	18	**0.94**
**904**	PAMR1 Q6UXH9$_{1}$: Inactive serine protease PAMR1	1/2	12	21	126	127	3	**0.56**
					128	129	3	**1.29**
					130	131	3	**0.77**
**905**	PAMR1 Q6UXH9$_{3}$: Inactive serine protease PAMR1		12	23	126	127	4	**0.70**
					128	129	3	**2.05**
					130	131	3	**0.57**
**906**	PAPLN O95428$_{1,5,6}$: Papilin	3/6	28	50	126	127	46	**0.84**
					128	129	45	**0.86**
					130	131	42	**0.98**
**907**	PATE3 B3GLJ2: Prostate and testis expressed protein 3		2	29	126	127	13	**1.56**
					128	129	13	**0.84**
					130	131	15	**1.19**
**908**	PBXIP1 Q96AQ6$_{1,2}$: Pre-B-cell leukemia transcription factor-interacting protein 1	2/2	3	4	126	127	4	**1.33**
					128	129	4	**1.72**
					130	131	3	**0.92**
**909**	PCBP1 Q15365: Poly(rC)-binding protein 1	1/6	8	10	126	127	4	**0.76**
					128	129	4	**0.51**
					130	131	4	**0.98**
**910**	PCBP2 Q15366$_{1-8}$: Poly(rC)-binding protein 2	8/8	5	7	126	127	1	**0.81**
					128	129	1	**0.42**
					130	131	1	**0.95**
**911**	PCMT1 P22061$_{1,2}$: Protein-L-isoaspartate(D-aspartate) O-methyltransferase	2/2	3	3	126	127	3	**0.63**
					128	129	3	**0.33**
					130	131	3	**0.84**
**912**	PCOLCE Q15113: Procollagen C-endopeptidase enhancer 1		19	105	126	127	97	**0.79**
					128	129	102	**1.58**
					130	131	100	**0.98**
**913**	PCOLCE2 Q9UKZ9: Procollagen C-endopeptidase enhancer 2		10	17	126	127	17	**1.59**
					128	129	17	**0.82**
					130	131	14	**1.02**
**914**	PCSK1N Q9UHG2: ProSAAS		5	26	126	127	23	**0.48**
					128	129	24	**1.81**
					130	131	23	**1.04**

#	protein	group	peptides	spectra	ch1	ch2	quant	ratio
**915**	PCSK5 Q92824$_1$: Proprotein convertase subtilisin/kexin type 5	1/2	27	158	126	127	117	**3.24**
					128	129	112	**1.64**
					130	131	117	**0.95**
**916**	PCSK7 Q16549: Proprotein convertase subtilisin/kexin type 7		1	1	126	127	1	**1.07**
					128	129	1	**2.35**
					130	131	1	**0.87**
**917**	PCSK9 Q8NBP7$_1$: Proprotein convertase subtilisin/kexin type 9	1/2	11	20	126	127	20	**0.53**
					128	129	18	**0.66**
					130	131	20	**1.09**
**918**	PCYOX1 Q9UHG3$_1$: Prenylcysteine oxidase 1	1/2	5	6	126	127	6	**0.58**
					128	129	6	**0.38**
					130	131	6	**1.13**
**919**	PDCD6IP Q8WUM4$_{1,2}$: Programmed cell death 6-interacting protein	2/3	19	23	126	127	20	**0.47**
					128	129	21	**0.27**
					130	131	23	**0.91**
**920**	PDGFB P01127$_{1,2}$: Platelet-derived growth factor subunit B	2/2	2	2	126	127	2	**0.47**
					128	129	2	**2.60**
					130	131	2	**0.93**
**921**	PDGFC Q9NRA1$_1$: Platelet-derived growth factor C	1/3	8	14	126	127	7	**0.39**
					128	129	6	**0.49**
					130	131	6	**1.19**
**922**	PDGFD Q9GZP0$_{1,2}$: Platelet-derived growth factor D	2/2	7	7	126	127	7	**0.68**
					128	129	6	**0.80**
					130	131	7	**1.18**
**923**	PDGFRL Q15198: Platelet-derived growth factor receptor-like protein		6	7	126	127	3	**0.52**
					128	129	5	**1.32**
					130	131	6	**0.82**
**924**	PDIA3 P30101: Protein disulfide-isomerase A3		12	14	126	127	12	**7.22**
					128	129	14	**1.51**
					130	131	13	**0.95**
**925**	PDIA4 P13667: Protein disulfide-isomerase A4		9	12	126	127	9	**1.69**
					128	129	10	**0.88**
					130	131	10	**1.11**
**926**	PDIA6 Q15084$_{1-5}$: Protein disulfide-isomerase A6	5/5	9	12	126	127	11	**1.50**
					128	129	12	**0.53**
					130	131	9	**0.91**

#	protein	group	peptides	spectra	ch1	ch2	quant	ratio
**927**	PDLIM7 Q9NR12$_{1,2}$: PDZ and LIM domain protein 7	2/2	3	4	126	127	3	**1.17**
					128	129	3	**1.12**
					130	131	3	**1.09**
**928**	PDXDC1 Q6P996$_{1,3-5}$: Pyridoxal-dependent decarboxylase domain-containing protein 1	4/4	1	1	126	127	1	**1.61**
					128	129	1	**0.51**
					130	131	1	**0.66**
**929**	PENK P01210: Proenkephalin-A [Cleaved into: Synenkephalin; Met-enkephalin		10	14	126	127	13	**0.95**
					128	129	12	**0.94**
					130	131	13	**0.91**
**930**	PFKL P17858$_{1,2}$: ATP-dependent 6-phosphofructokinase, liver type	2/2	7	10	126	127	7	**0.42**
					128	129	7	**0.83**
					130	131	7	**1.22**
**931**	PFKM P08237$_{1,3}$: ATP-dependent 6-phosphofructokinase, muscle type	2/2	3	5	126	127	4	**0.81**
					128	129	4	**0.79**
					130	131	4	**1.01**
**932**	PFN1 P07737: Profilin-1		4	10	126	127	9	**1.17**
					128	129	8	**0.29**
					130	131	9	**0.92**
**933**	PGAM1 P18669: Phosphoglycerate mutase 1		2	4	126	127	4	**2.07**
					128	129	4	**0.50**
					130	131	4	**0.95**
**934**	PGLYRP2 Q96PD5$_{1,2}$: N-acetylmuramoyl-L-alanine amidase	2/2	10	37	126	127	36	**0.64**
					128	129	37	**0.29**
					130	131	36	**1.10**
**935**	PGM1 P36871$_{1}$: Phosphoglucomutase-1		2	2	126	127	2	**0.62**
					128	129	2	**1.31**
					130	131	2	**1.08**
**936**	PGM3 O95394$_{1,3,4}$: Phosphoacetylglucosamine mutase	3/3	3	3	126	127	3	**0.58**
					128	129	3	**0.33**
					130	131	3	**1.01**
**937**	PGRMC1 O00264$_{1,2}$: Membrane-associated progesterone receptor component 1	2/2	2	2	126	127	2	**0.59**
					128	129	2	**1.16**
					130	131	2	**1.02**
**938**	PHB P35232$_{1}$: Prohibitin		1	1	126	127	1	**12.36**
					128	129	1	**1.60**
					130	131	1	**0.58**

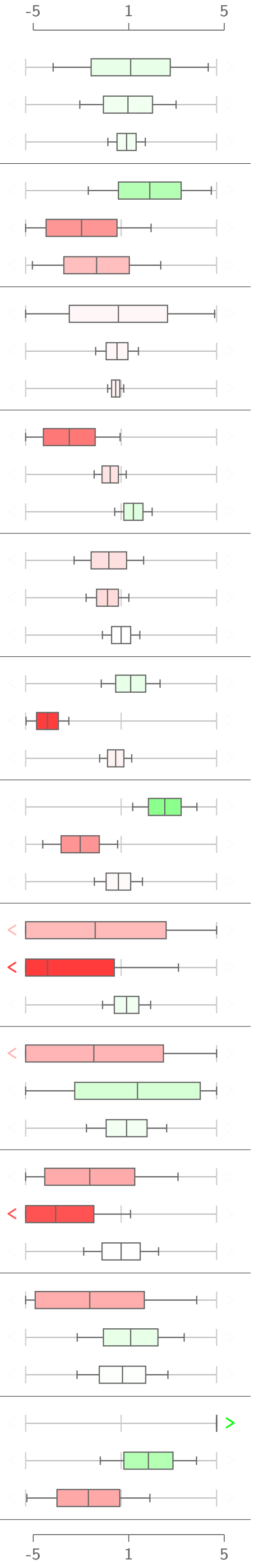

#	protein	group	peptides	spectra	ch1	ch2	quant	ratio
**939**	PHB2 Q99623$_1$: Prohibitin-2	1/2	3	4	126	127	4	**13.31**
					128	129	4	**1.60**
					130	131	4	**0.67**
**940**	PHGDH O43175: D-3-phosphoglycerate dehydrogenase		9	12	126	127	12	**0.36**
					128	129	11	**0.29**
					130	131	12	**1.20**
**941**	PHPT1 Q9NRX4$_{1,2}$: 14 kDa phosphohistidine phosphatase	2/2	1	1	126	127	1	**1.21**
					128	129	1	**0.61**
					130	131	1	**1.08**
**942**	PICALM Q13492$_{1-5}$: Phosphatidylinositol-binding clathrin assembly protein	5/5	2	3	126	127	3	**0.33**
					128	129	3	**0.24**
					130	131	3	**0.93**
**943**	PIGR P01833: Polymeric immunoglobulin receptor		9	9	126	127	7	**0.95**
					128	129	8	**0.86**
					130	131	8	**0.97**
**944**	PITPNA Q00169: Phosphatidylinositol transfer protein alpha isoform		3	6	126	127	4	**0.57**
					128	129	4	**0.49**
					130	131	3	**0.92**
**945**	PITPNB P48739$_{1-3}$: Phosphatidylinositol transfer protein beta isoform	3/3	2	3	126	127	1	**0.27**
					128	129	1	**0.35**
					130	131	1	**0.88**
**946**	PKDCC Q504Y2: Extracellular tyrosine-protein kinase PKDCC		4	4	126	127	4	**0.55**
					128	129	3	**0.81**
					130	131	3	**0.99**
**947**	PKM P14618$_2$: Pyruvate kinase PKM	1/3	26	46	126	127	42	**0.59**
					128	129	45	**0.32**
					130	131	44	**0.91**
**948**	PLA2G2A P14555: Phospholipase A2, membrane associated		11	33	126	127	30	**0.61**
					128	129	27	**1.26**
					130	131	32	**0.82**
**949**	PLAT P00750$_1$: Tissue-type plasminogen activator	1/3	15	22	126	127	7	**1.59**
					128	129	7	**1.24**
					130	131	6	**0.94**
**950**	PLAT P00750$_3$: Tissue-type plasminogen activator		12	16	126	127	1	**0.42**
					128	129	1	**0.99**
					130	131	1	**0.87**

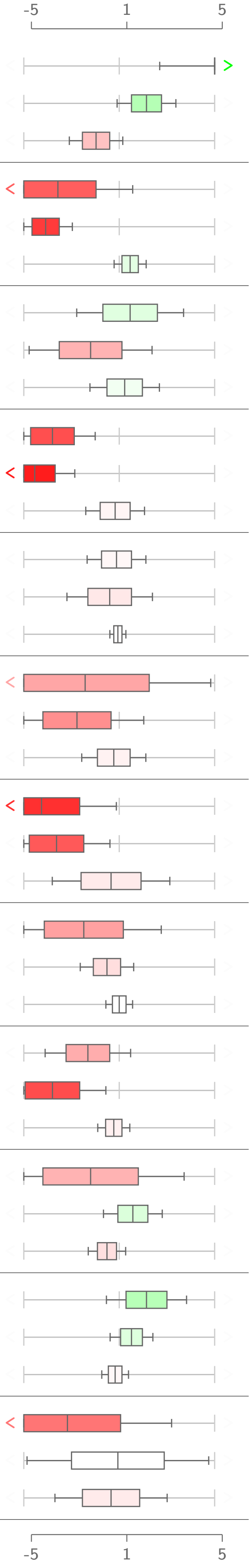

#	protein	group	peptides	spectra	ch1	ch2	quant	ratio
**952**	PLEC Q15149$_6$: Plectin	1/8	95	107	126	127	1	**4.25**
					128	129	1	**1.66**
					130	131	1	**0.84**
**953**	PLEKHO2 Q8TD55$_2$: Pleckstrin homology domain-containing family O member 2	1/2	4	5	126	127	3	**0.99**
					128	129	5	**0.47**
					130	131	5	**0.84**
**954**	PLG P00747: Plasminogen	1/2	52	578	126	127	484	**0.82**
					128	129	503	**0.87**
					130	131	489	**1.02**
**955**	PLGLB1 Q02325: Plasminogen-like protein B		5	36	126	127	1	**2.79**
					128	129	1	**1.03**
					130	131	1	**1.19**
**956**	PLIN3 O60664$_1$: Perilipin-3	1/4	9	10	126	127	10	**1.19**
					128	129	9	**0.64**
					130	131	8	**0.89**
**957**	PLOD1 Q02809$_{1,2}$: Procollagen-lysine,2-oxoglutarate 5-dioxygenase 1	2/2	4	4	126	127	3	**0.77**
					128	129	3	**0.99**
					130	131	4	**0.80**
**958**	PLOD3 O60568: Procollagen-lysine,2-oxoglutarate 5-dioxygenase 3		4	4	126	127	4	**0.55**
					128	129	4	**0.58**
					130	131	4	**0.92**
**959**	PLS3 P13797$_1$: Plastin-3	1/3	17	30	126	127	10	**1.01**
					128	129	9	**0.24**
					130	131	10	**0.89**
**960**	PLTP P55058$_1$: Phospholipid transfer protein	1/4	18	320	126	127	243	**0.24**
					128	129	301	**0.36**
					130	131	294	**0.87**
**961**	PLXDC2 Q6UX71$_{1,2}$: Plexin domain-containing protein 2	2/2	3	3	126	127	3	**0.61**
					128	129	3	**0.72**
					130	131	3	**1.00**
**962**	PLXNB2 O15031: Plexin-B2		3	3	126	127	2	**1.76**
					128	129	2	**1.39**
					130	131	2	**0.85**
**963**	PODN Q7Z5L7$_{1-3}$: Podocan	3/4	10	11	126	127	11	**1.49**
					128	129	10	**0.75**
					130	131	9	**0.86**

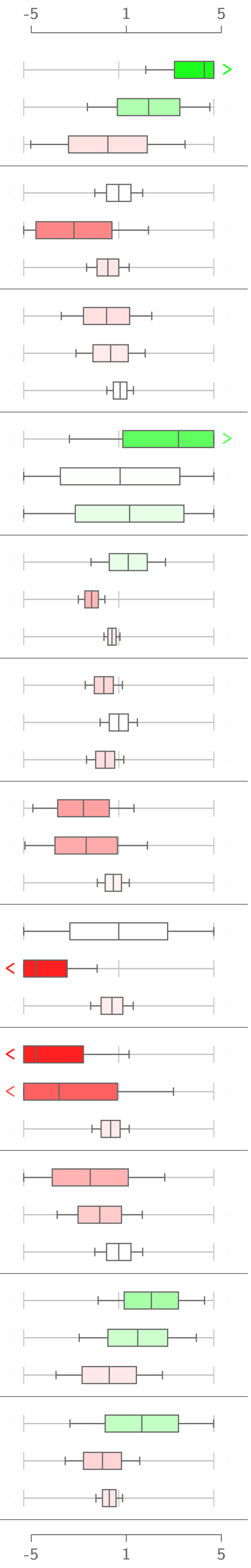

#	protein	group	peptides	spectra	ch1	ch2	quant	ratio
**964**	PON1 P27169: Serum paraoxonase/arylesterase 1		13	66	126	127	56	**0.45**
					128	129	60	**0.41**
					130	131	59	**1.04**
**965**	PON3 Q15166: Serum paraoxonase/lactonase 3		8	13	126	127	7	**0.78**
					128	129	6	**0.43**
					130	131	7	**1.14**
**966**	POSTN Q15063$_1$: Periostin		42	221	126	127	3	**0.20**
					128	129	3	**0.32**
					130	131	3	**1.26**
**973**	POTEA Q6S8J7$_{1,2}$: POTE ankyrin domain family member A	2/2	3	11	126	127	3	**0.90**
					128	129	4	**1.27**
					130	131	4	**1.38**
**974**	POTEE Q6S8J3$_1$: POTE ankyrin domain family member E		15	151	126	127	29	**1.29**
					128	129	38	**1.41**
					130	131	36	**1.00**
**975**	POTEF A5A3E0: POTE ankyrin domain family member F		12	96	126	127	3	**1.18**
					128	129	3	**2.16**
					130	131	4	**0.82**
**977**	PPIA P62937$_1$: Peptidyl-prolyl cis-trans isomerase A	1/8	5	8	126	127	7	**3.11**
					128	129	8	**0.96**
					130	131	6	**1.26**
**978**	PPIB P23284: Peptidyl-prolyl cis-trans isomerase B		3	3	126	127	2	**5.90**
					128	129	3	**1.51**
					130	131	3	**1.22**
**979**	PPL O60437: Periplakin		7	7	126	127	5	**2.03**
					128	129	5	**1.07**
					130	131	6	**0.96**
**980**	PPP1CB P62140: Serine/threonine-protein phosphatase PP1-beta catalytic subunit		2	2	126	127	2	**0.89**
					128	129	2	**0.81**
					130	131	2	**1.08**
**981**	PPP1R7 Q15435$_{1,3}$: Protein phosphatase 1 regulatory subunit 7	2/5	3	4	126	127	2	**0.38**
					128	129	3	**1.10**
					130	131	3	**0.95**
**982**	PPP2CA P67775$_{1,2}$, PPP2CB P62714: Serine/threonine-protein phosphatase 2A catalytic subunit alpha isoform, Serine/threonine-protein phosphatase 2A catalytic subunit beta isoform	3/3	1	1	126	127	1	**0.55**
					128	129	1	**0.60**
					130	131	1	**1.03**

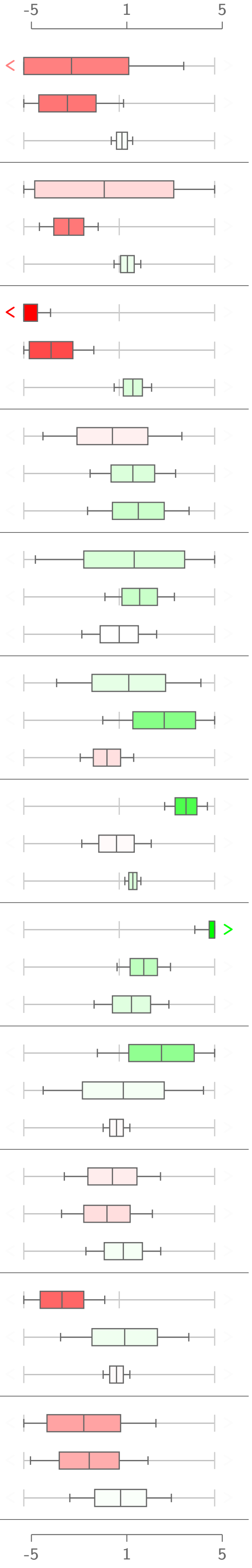

#	protein	group	peptides	spectra	ch1	ch2	quant	ratio
**983**	PPP2R1A P30153: Serine/threonine-protein phosphatase 2A 65 kDa regulatory subunit A alpha is . . .		7	7	126	127	6	**0.75**
					128	129	7	**0.27**
					130	131	5	**1.05**
**984**	PPP2R2A $P63151_{1,2}$: Serine/threonine-protein phosphatase 2A 55 kDa regulatory subunit B alpha is . . .	2/2	3	3	126	127	3	**0.99**
					128	129	3	**1.32**
					130	131	3	**1.07**
**985**	PRAP1 $Q96NZ9_{1,4}$: Proline-rich acidic protein 1	2/3	4	6	126	127	2	**0.65**
					128	129	2	**2.38**
					130	131	2	**0.98**
**986**	PRAP1 $Q96NZ9_{3}$: Proline-rich acidic protein 1		4	8	126	127	4	**2.50**
					128	129	4	**2.07**
					130	131	3	**0.87**
**987**	PRDX1 Q06830: Peroxiredoxin-1	1/2	6	7	126	127	5	**1.85**
					128	129	6	**0.66**
					130	131	6	**1.01**
**988**	PRDX2 $P32119_{1}$: Peroxiredoxin-2	1/2	4	4	126	127	3	**1.68**
					128	129	3	**0.44**
					130	131	3	**1.08**
**989**	PRDX6 P30041: Peroxiredoxin-6		9	16	126	127	16	**0.46**
					128	129	16	**0.27**
					130	131	16	**0.96**
**990**	PRELP P51888: Prolargin		6	7	126	127	6	**0.67**
					128	129	7	**0.80**
					130	131	7	**0.92**
**991**	PRG2 $P13727_{1,2}$: Bone marrow proteoglycan	2/2	1	1	126	127	1	**1.95**
					128	129	1	**1.37**
					130	131	1	**0.97**
**992**	PRG3 Q9Y2Y8: Proteoglycan 3		1	1	126	127	1	**0.47**
					128	129	1	**0.59**
					130	131	1	**0.78**
**993**	PRG4 $Q92954_{1,6}$: Proteoglycan 4	2/4	42	164	126	127	3	**0.25**
					128	129	3	**0.66**
					130	131	3	**0.83**
**994**	PRG4 $Q92954_{3}$: Proteoglycan 4	1/2	42	167	126	127	3	**1.88**
					128	129	2	**1.70**
					130	131	3	**1.09**

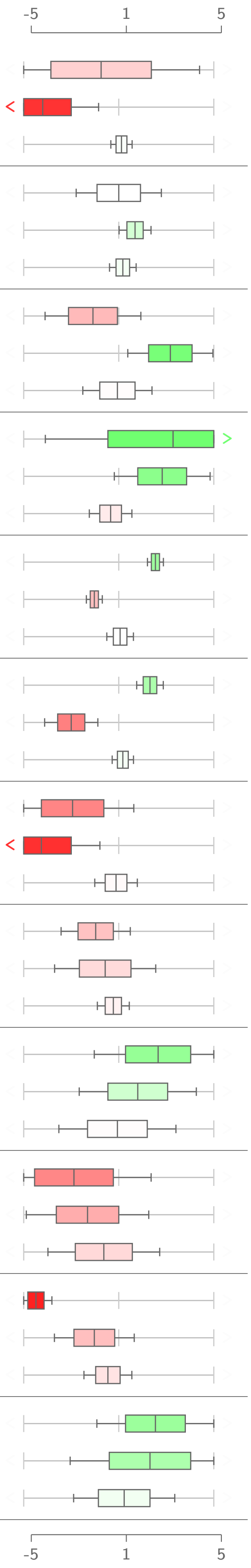

#	protein	group	peptides	spectra	ch1	ch2	quant	ratio
995	PRKAA1 Q13131$_{1,2}$: 5'-AMP-activated protein kinase catalytic subunit alpha-1	2/2	2	2	126	127	1	**0.13**
					128	129	1	**0.37**
					130	131	1	**1.00**
996	PRKAR1A P10644$_{1}$: cAMP-dependent protein kinase type I-alpha regulatory subunit		2	2	126	127	2	**0.25**
					128	129	2	**0.33**
					130	131	2	**0.86**
997	PRKAR2A P13861$_{1}$: cAMP-dependent protein kinase type II-alpha regulatory subunit	1/2	7	9	126	127	9	**0.74**
					128	129	8	**0.62**
					130	131	7	**0.97**
998	PRKCSH P14314$_{1,2}$: Glucosidase 2 subunit beta	2/2	9	14	126	127	14	**1.53**
					128	129	14	**0.82**
					130	131	14	**1.31**
999	PRKX P51817: cAMP-dependent protein kinase catalytic subunit PRKX		3	3	126	127	2	**0.99**
					128	129	2	**1.38**
					130	131	2	**0.87**
1000	PRNP P04156$_{1,2}$: Major prion protein	2/2	4	7	126	127	7	**0.81**
					128	129	7	**1.82**
					130	131	7	**0.94**
1001	PROC P04070$_{1,2}$: Vitamin K-dependent protein C	2/2	12	27	126	127	26	**0.45**
					128	129	26	**0.78**
					130	131	24	**1.34**
1002	PROS1 P07225: Vitamin K-dependent protein S		23	66	126	127	52	**0.24**
					128	129	59	**0.33**
					130	131	58	**1.17**
1003	PROZ P22891$_{1,2}$: Vitamin K-dependent protein Z	2/2	3	3	126	127	3	**0.27**
					128	129	3	**0.50**
					130	131	3	**1.04**
1005	PRPS1 P60891$_{1,2}$, PRPS2 P11908$_{1,2}$: Ribose-phosphate pyrophosphokinase 1, Ribose-phosphate pyrophosphokinase 2	4/4	2	2	126	127	2	**0.57**
					128	129	2	**1.12**
					130	131	2	**1.04**
1006	PRSS2 P07478: Trypsin-2	1/3	4	13	126	127	11	**3.74**
					128	129	9	**2.18**
					130	131	13	**2.00**
1007	PRSS23 O95084$_{1}$: Serine protease 23		1	1	126	127	1	**1.43**
					128	129	1	**1.63**
					130	131	1	**0.93**

#	protein	group	peptides	spectra	ch1	ch2	quant	ratio
**1008**	PSAP P07602$_{1-3}$: Prosaposin	3/3	18	56	126	127	54	**0.98**
					128	129	55	**1.32**
					130	131	51	**0.86**
**1009**	PSAPL1 Q6NUJ1: Proactivator polypeptide-like 1 [Cleaved into: Saposin A-like; Saposin B-Val . . .		2	2	126	127	2	**2.51**
					128	129	2	**1.81**
					130	131	2	**2.17**
**1010**	PSMA1 P25786$_{1,2}$: Proteasome subunit alpha type-1	2/2	2	3	126	127	3	**1.17**
					128	129	3	**0.94**
					130	131	3	**1.16**
**1011**	PSMA3 P25788$_{1,2}$: Proteasome subunit alpha type-3	2/2	3	4	126	127	4	**1.25**
					128	129	4	**0.79**
					130	131	4	**1.21**
**1012**	PSMA5 P28066$_{1}$: Proteasome subunit alpha type-5	1/2	2	2	126	127	2	**1.12**
					128	129	2	**1.12**
					130	131	2	**1.29**
**1013**	PSMA6 P60900$_{1}$: Proteasome subunit alpha type-6	1/2	3	3	126	127	3	**1.86**
					128	129	3	**1.13**
					130	131	3	**1.25**
**1014**	PSMA7 O14818$_{1,2}$: Proteasome subunit alpha type-7	2/2	3	4	126	127	3	**1.38**
					128	129	4	**1.24**
					130	131	4	**1.03**
**1015**	PSMB1 P20618: Proteasome subunit beta type-1		2	2	126	127	2	**1.10**
					128	129	2	**0.85**
					130	131	2	**0.96**
**1016**	PSMB7 Q99436$_{1,2}$: Proteasome subunit beta type-7	2/2	1	2	126	127	2	**1.02**
					128	129	2	**1.69**
					130	131	2	**1.27**
**1017**	PSMD1 Q99460$_{1,2}$: 26S proteasome non-ATPase regulatory subunit 1	2/2	3	4	126	127	4	**0.81**
					128	129	4	**0.37**
					130	131	4	**0.96**
**1018**	PSMD13 Q9UNM6$_{1,2}$: 26S proteasome non-ATPase regulatory subunit 13	2/2	1	2	126	127	2	**0.45**
					128	129	2	**0.23**
					130	131	2	**0.92**
**1019**	PSMD2 Q13200$_{1,3}$: 26S proteasome non-ATPase regulatory subunit 2	2/2	3	3	126	127	3	**0.67**
					128	129	3	**0.40**
					130	131	3	**1.01**

#	protein	group	peptides	spectra	ch1	ch2	quant	ratio
**1020**	PSMD3 O43242$_1$: 26S proteasome non-ATPase regulatory subunit 3		1	2	126	127	2	**0.55**
					128	129	2	**0.71**
					130	131	2	**0.81**
**1021**	PSME1 Q06323$_{1-3}$: Proteasome activator complex subunit 1	3/3	5	7	126	127	7	**0.72**
					128	129	7	**0.33**
					130	131	7	**0.78**
**1022**	PSME2 Q9UL46: Proteasome activator complex subunit 2		7	9	126	127	8	**2.09**
					128	129	9	**0.78**
					130	131	9	**0.81**
**1023**	PSMG1 O95456$_{1,2}$: Proteasome assembly chaperone 1	2/2	1	1	126	127	1	**0.57**
					128	129	1	**0.50**
					130	131	1	**1.05**
**1024**	PTBP1 P26599$_{1-3}$: Polypyrimidine tract-binding protein 1	3/10	3	7	126	127	7	**1.41**
					128	129	6	**0.32**
					130	131	7	**0.82**
**1025**	PTGDS P41222: Prostaglandin-H2 D-isomerase		3	4	126	127	4	**1.44**
					128	129	3	**2.25**
					130	131	4	**1.08**
**1026**	PTGIS Q16647: Prostacyclin synthase		3	3	126	127	3	**1.56**
					128	129	3	**0.51**
					130	131	3	**0.90**
**1027**	PTGR1 Q14914$_{1,2}$: Prostaglandin reductase 1	2/2	2	2	126	127	2	**0.15**
					128	129	2	**0.13**
					130	131	2	**0.81**
**1028**	PTMA P06454$_{1,2}$: Prothymosin alpha [Cleaved into: Prothymosin alpha, N-terminally processed; . . .	2/2	2	2	126	127	2	**1.89**
					128	129	2	**0.48**
					130	131	2	**1.32**
**1029**	PTMS P20962: Parathymosin		3	6	126	127	5	**0.67**
					128	129	6	**0.65**
					130	131	5	**1.36**
**1030**	PTN P21246: Pleiotrophin		2	2	126	127	2	**0.67**
					128	129	2	**1.40**
					130	131	2	**1.03**
**1031**	PTPRF P10586$_{1,2}$: Receptor-type tyrosine-protein phosphatase F	2/2	2	2	126	127	2	**0.57**
					128	129	2	**0.58**
					130	131	2	**0.90**

#	protein	group	peptides	spectra	ch1	ch2	quant	ratio
**1032**	PTRF Q6NZI2$_1$: Polymerase I and transcript release factor	1/3	12	12	126	127	12	**2.80**
					128	129	12	**1.62**
					130	131	12	**1.05**
**1033**	PTX3 P26022: Pentraxin-related protein PTX3		2	2	126	127	2	**2.00**
					128	129	2	**0.36**
					130	131	2	**1.32**
**1034**	PXDN Q92626$_1$: Peroxidasin homolog	1/2	30	42	126	127	41	**0.93**
					128	129	39	**0.94**
					130	131	40	**0.86**
**1035**	PXN P49023$_{1-4}$: Paxillin	4/4	3	4	126	127	4	**2.36**
					128	129	4	**1.11**
					130	131	4	**0.94**
**1036**	PZP P20742$_1$: Pregnancy zone protein	1/2	17	22	126	127	9	**0.50**
					128	129	8	**0.60**
					130	131	9	**1.08**
**1037**	QARS P47897$_{1,2}$: Glutamine--tRNA ligase	2/2	1	1	126	127	1	**0.97**
					128	129	1	**0.45**
					130	131	1	**0.92**
**1038**	QPCT Q16769$_1$: Glutaminyl-peptide cyclotransferase	1/2	6	12	126	127	12	**0.63**
					128	129	11	**0.98**
					130	131	12	**1.03**
**1039**	QSOX1 O00391$_1$: Sulfhydryl oxidase 1	1/2	33	107	126	127	100	**0.27**
					128	129	103	**0.32**
					130	131	102	**0.97**
**1040**	QTRT2 Q9H974$_{1-4}$: Queuine tRNA-ribosyltransferase accessory subunit 2	4/4	3	63	126	127	25	**3.32**
					128	129	23	**1.36**
					130	131	25	**1.31**
**1041**	RAB10 P61026: Ras-related protein Rab-10		2	5	126	127	3	**1.11**
					128	129	4	**0.72**
					130	131	4	**2.94**
**1042**	RAB11A P62491$_{1,2}$, RAB11B Q15907$_{1,2}$: Ras-related protein Rab-11A, Ras-related protein Rab-11B	4/4	3	3	126	127	3	**1.03**
					128	129	3	**0.44**
					130	131	3	**0.90**
**1043**	RAB1A P62820$_{1,2}$: Ras-related protein Rab-1A	2/2	3	3	126	127	2	**0.41**
					128	129	2	**0.20**
					130	131	2	**0.79**

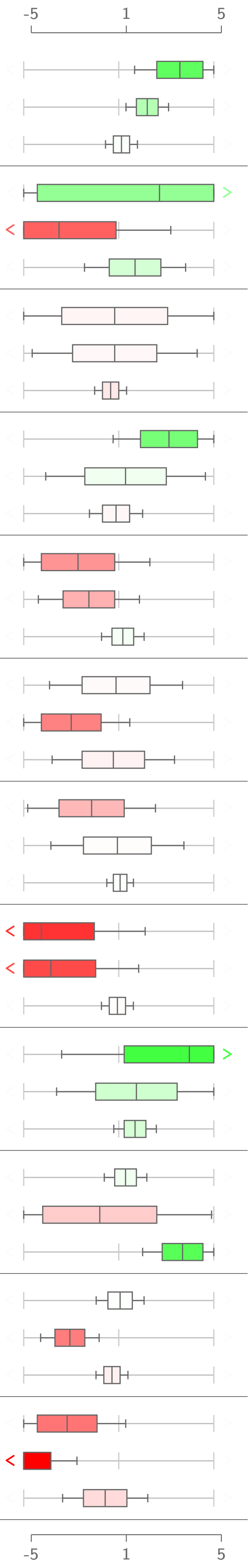

#	protein	group	peptides	spectra	ch1	ch2	quant	ratio
**1044**	RAB1B Q9H0U4: Ras-related protein Rab-1B		2	2	126	127	1	**0.33**
					128	129	1	**0.18**
					130	131	1	**0.76**
**1045**	RAB5C P51148$_{1,2}$: Ras-related protein Rab-5C	2/6	4	4	126	127	4	**1.26**
					128	129	4	**0.73**
					130	131	3	**0.86**
**1046**	RAB7A P51149: Ras-related protein Rab-7a		2	2	126	127	2	**0.75**
					128	129	2	**0.63**
					130	131	2	**1.01**
**1047**	RABEP1 Q15276$_1$: Rab GTPase-binding effector protein 1	1/2	3	4	126	127	2	**2.90**
					128	129	2	**1.01**
					130	131	2	**0.71**
**1048**	RAC1 P63000$_{1,2}$, RAC2 P15153: Ras-related C3 botulinum toxin substrate 1, Ras-related C3 botulinum toxin substrate 2	3/8	4	5	126	127	4	**0.62**
					128	129	3	**0.35**
					130	131	4	**1.13**
**1049**	RACK1 P63244: Receptor of activated protein C kinase 1		6	6	126	127	5	**0.87**
					128	129	6	**0.58**
					130	131	6	**1.54**
**1050**	RAD23B P54727$_{1,2}$: UV excision repair protein RAD23 homolog B	2/2	2	2	126	127	2	**1.19**
					128	129	2	**1.41**
					130	131	2	**1.05**
**1051**	RALY Q9UKM9$_{1,2}$: RNA-binding protein Raly	2/2	3	3	126	127	3	**1.51**
					128	129	3	**0.66**
					130	131	3	**0.96**
**1052**	RAN P62826: GTP-binding nuclear protein Ran		4	4	126	127	3	**0.91**
					128	129	4	**0.53**
					130	131	4	**1.09**
**1053**	RAP1A P62834, RAP1B P61224$_{1-3}$, A6NIZ1: Ras-related protein Rap-1A, Ras-related protein Rap-1b, . . .	5/5	1	1	126	127	1	**0.54**
					128	129	1	**0.14**
					130	131	1	**1.04**
**1054**	RARRES1 P49788$_{1,2}$: Retinoic acid receptor responder protein 1	2/2	7	8	126	127	6	**0.66**
					128	129	7	**0.79**
					130	131	8	**1.16**
**1055**	RARRES2 Q99969: Retinoic acid receptor responder protein 2		8	78	126	127	75	**0.86**
					128	129	74	**1.69**
					130	131	68	**0.88**

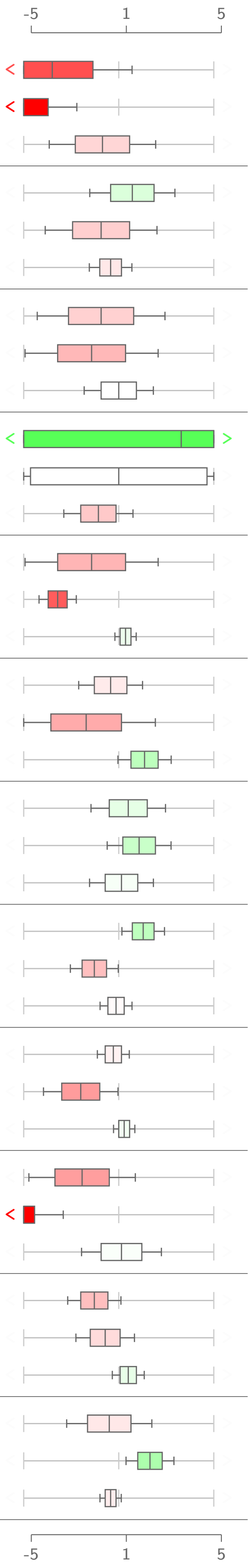

#	protein	group	peptides	spectra	ch1	ch2	quant	ratio
**1056**	RARS P54136$_{1,2}$: Arginine--tRNA ligase, cytoplasmic	2/2	1	1	126	127	1	**1.51**
					128	129	1	**0.55**
					130	131	1	**0.91**
**1057**	RBBP4 Q09028$_{1,3}$: Histone-binding protein RBBP4	2/6	3	5	126	127	4	**0.55**
					128	129	5	**0.62**
					130	131	5	**1.08**
**1058**	RBM6 P78332$_{3}$: RNA-binding protein 6	1/2	3	11	126	127	6	**0.92**
					128	129	10	**0.75**
					130	131	11	**0.88**
**1059**	RBMX P38159$_{1,2}$: RNA-binding motif protein, X chromosome	2/3	9	10	126	127	8	**4.06**
					128	129	10	**1.00**
					130	131	9	**0.79**
**1060**	RBP4 P02753: Retinol-binding protein 4		9	101	126	127	91	**1.25**
					128	129	93	**4.34**
					130	131	92	**0.96**
**1061**	RCN1 Q15293$_{1,2}$: Reticulocalbin-1	2/2	2	2	126	127	2	**1.59**
					128	129	2	**1.81**
					130	131	2	**0.82**
**1062**	RCN3 Q96D15: Reticulocalbin-3		5	7	126	127	7	**1.74**
					128	129	7	**2.69**
					130	131	6	**1.01**
**1063**	RECK O95980: Reversion-inducing cysteine-rich protein with Kazal motifs		2	3	126	127	3	**1.32**
					128	129	3	**1.88**
					130	131	3	**0.84**
**1064**	REG3A Q06141: Regenerating islet-derived protein 3-alpha		3	3	126	127	3	**1.54**
					128	129	3	**5.78**
					130	131	3	**0.93**
**1065**	RHOA P61586, RHOC P08134: Transforming protein RhoA, Rho-related GTP-binding protein RhoC	2/2	2	3	126	127	3	**0.71**
					128	129	3	**0.35**
					130	131	3	**1.15**
**1066**	RIC8A Q9NPQ8$_{1-4}$: Synembryn-A	4/4	2	4	126	127	1	**0.05**
					128	129	1	**0.21**
					130	131	1	**1.27**
**1067**	RLF Q13129: Zinc finger protein Rlf		4	11	126	127	10	**0.90**
					128	129	10	**1.80**
					130	131	10	**1.08**

#	protein	group	peptides	spectra	ch1	ch2	quant	ratio
**1068**	RNASE1 P07998: Ribonuclease pancreatic		6	20	126	127	20	**0.71**
					128	129	19	**2.92**
					130	131	20	**1.11**
**1069**	RNASE3 P12724: Eosinophil cationic protein		5	5	126	127	5	**2.02**
					128	129	5	**0.87**
					130	131	5	**0.75**
**1070**	RNASE4 P34096: Ribonuclease 4		11	259	126	127	246	**0.95**
					128	129	230	**1.66**
					130	131	233	**1.04**
**1071**	RNASE6 Q93091: Ribonuclease K6		2	2	126	127	2	**1.63**
					128	129	2	**2.04**
					130	131	2	**1.13**
**1072**	RNH1 P13489: Ribonuclease inhibitor		11	14	126	127	14	**0.89**
					128	129	14	**0.28**
					130	131	13	**1.37**
**1073**	RPA1 P27694: Replication protein A 70 kDa DNA-binding subunit		5	6	126	127	3	**0.82**
					128	129	4	**0.53**
					130	131	4	**0.90**
**1074**	RPL10A P62906: 60S ribosomal protein L10a		2	4	126	127	4	**2.01**
					128	129	4	**1.32**
					130	131	4	**0.99**
**1075**	RPL12 P30050$_{1}$: 60S ribosomal protein L12		3	4	126	127	4	**2.24**
					128	129	4	**0.41**
					130	131	4	**0.96**
**1076**	RPL13 P26373$_{1,2}$: 60S ribosomal protein L13	2/2	3	3	126	127	2	**7.76**
					128	129	2	**1.52**
					130	131	2	**0.79**
**1077**	RPL14 P50914: 60S ribosomal protein L14		5	5	126	127	4	**6.20**
					128	129	5	**1.28**
					130	131	4	**0.86**
**1078**	RPL18 Q07020$_{1,2}$: 60S ribosomal protein L18	2/2	1	1	126	127	1	**1.47**
					128	129	1	**0.77**
					130	131	1	**1.19**
**1079**	RPL19 P84098: 60S ribosomal protein L19		2	2	126	127	1	**1.12**
					128	129	1	**0.69**
					130	131	1	**1.18**

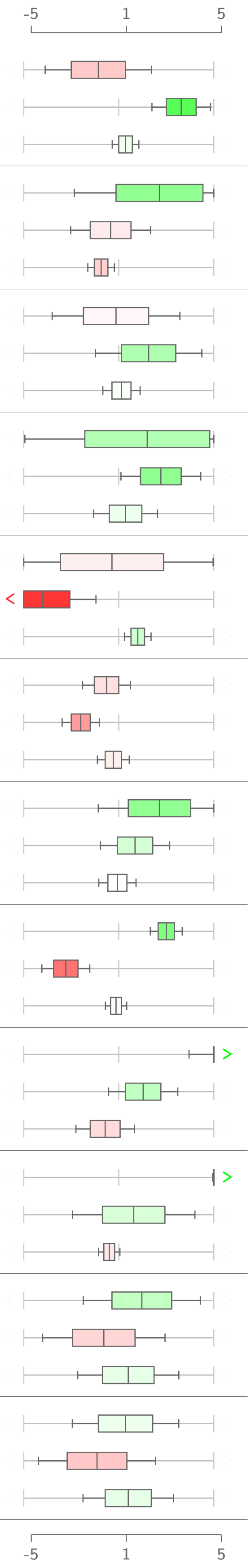

#	protein	group	peptides	spectra	ch1	ch2	quant	ratio
**1080**	RPL21 P46778: 60S ribosomal protein L21		2	2	126	127	2	**2.81**
					128	129	2	**0.79**
					130	131	2	**1.34**
**1081**	RPL22 P35268: 60S ribosomal protein L22		4	5	126	127	4	**1.56**
					128	129	3	**0.84**
					130	131	4	**0.99**
**1082**	RPL23A P62750: 60S ribosomal protein L23a		4	4	126	127	4	**3.27**
					128	129	4	**0.83**
					130	131	4	**1.20**
**1083**	RPL24 P83731: 60S ribosomal protein L24		2	2	126	127	2	**3.31**
					128	129	2	**0.96**
					130	131	2	**0.93**
**1084**	RPL26 P61254: 60S ribosomal protein L26	1/2	3	3	126	127	3	**2.51**
					128	129	3	**0.93**
					130	131	3	**1.23**
**1085**	RPL27 P61353: 60S ribosomal protein L27		4	4	126	127	3	**3.47**
					128	129	4	**0.98**
					130	131	3	**1.19**
**1086**	RPL28 $P46779_1$: 60S ribosomal protein L28	1/5	4	4	126	127	4	**3.36**
					128	129	4	**0.88**
					130	131	4	**1.09**
**1087**	RPL30 P62888: 60S ribosomal protein L30		3	7	126	127	7	**2.59**
					128	129	6	**1.17**
					130	131	6	**1.00**
**1088**	RPL31 $P62899_{1-3}$: 60S ribosomal protein L31	3/3	3	5	126	127	5	**2.70**
					128	129	4	**0.68**
					130	131	5	**0.95**
**1089**	RPL32 P62910: 60S ribosomal protein L32		2	2	126	127	1	**2.45**
					128	129	1	**0.86**
					130	131	1	**0.88**
**1090**	RPL34 P49207: 60S ribosomal protein L34		3	3	126	127	3	**2.97**
					128	129	3	**1.05**
					130	131	3	**1.31**
**1091**	RPL35 P42766: 60S ribosomal protein L35		3	4	126	127	3	**2.30**
					128	129	3	**1.18**
					130	131	3	**1.12**

#	protein	group	peptides	spectra	ch1	ch2	quant	ratio
**1092**	RPL37A P61513, RPL37AP8 A6NKH3: 60S ribosomal protein L37a, Putative 60S ribosomal protein L37a-like protein	2/2	1	1	126	127	1	**4.10**
					128	129	1	**1.33**
					130	131	1	**0.95**
**1093**	RPL4 P36578: 60S ribosomal protein L4		3	3	126	127	3	**2.88**
					128	129	3	**0.86**
					130	131	3	**0.81**
**1094**	RPL5 P46777: 60S ribosomal protein L5		5	8	126	127	6	**1.00**
					128	129	7	**0.73**
					130	131	7	**1.05**
**1095**	RPL6 Q02878: 60S ribosomal protein L6		3	3	126	127	3	**1.41**
					128	129	3	**0.70**
					130	131	3	**1.05**
**1096**	RPL7 P18124: 60S ribosomal protein L7		3	4	126	127	3	**1.31**
					128	129	3	**0.87**
					130	131	4	**0.86**
**1097**	RPL7A P62424: 60S ribosomal protein L7a		4	4	126	127	3	**5.43**
					128	129	3	**1.10**
					130	131	4	**1.00**
**1098**	RPL8 P62917: 60S ribosomal protein L8		5	5	126	127	5	**2.27**
					128	129	5	**0.62**
					130	131	5	**1.03**
**1099**	RPL9 P32969: 60S ribosomal protein L9		3	3	126	127	3	**0.85**
					128	129	3	**0.80**
					130	131	3	**1.43**
**1100**	RPLP0 P05388[1,2], RPLP0P6 Q8NHW5: 60S acidic ribosomal protein P0, 60S acidic ribosomal protein P0-like	3/3	5	7	126	127	7	**0.42**
					128	129	7	**0.18**
					130	131	7	**1.12**
**1101**	RPLP2 P05387: 60S acidic ribosomal protein P2		4	4	126	127	3	**0.77**
					128	129	4	**0.67**
					130	131	3	**1.42**
**1102**	RPN1 P04843: Dolichyl-diphosphooligosaccharide--protein glycosyltransferase subunit 1		5	5	126	127	5	**2.77**
					128	129	4	**0.47**
					130	131	5	**0.71**
**1103**	RPS10 P46783: 40S ribosomal protein S10	1/2	7	7	126	127	6	**2.11**
					128	129	4	**1.12**
					130	131	6	**0.84**

#	protein	group	peptides	spectra	ch1	ch2	quant	ratio
1104	RPS11 P62280: 40S ribosomal protein S11		2	2	126	127	2	**2.65**
					128	129	2	**0.86**
					130	131	2	**1.01**
1105	RPS13 P62277: 40S ribosomal protein S13		4	7	126	127	7	**4.23**
					128	129	7	**1.56**
					130	131	7	**1.05**
1106	RPS14 P62263: 40S ribosomal protein S14		4	5	126	127	4	**2.62**
					128	129	4	**0.50**
					130	131	4	**0.98**
1107	RPS16 P62249: 40S ribosomal protein S16		2	2	126	127	2	**2.48**
					128	129	2	**0.84**
					130	131	2	**0.98**
1108	RPS18 P62269: 40S ribosomal protein S18		3	4	126	127	4	**4.12**
					128	129	4	**1.51**
					130	131	4	**1.07**
1109	RPS19 P39019: 40S ribosomal protein S19		3	6	126	127	6	**4.13**
					128	129	6	**0.88**
					130	131	6	**0.98**
1110	RPS20 P60866$_{1,2}$: 40S ribosomal protein S20	2/2	3	5	126	127	4	**2.12**
					128	129	4	**0.40**
					130	131	4	**0.99**
1111	RPS21 P63220: 40S ribosomal protein S21		2	2	126	127	2	**4.85**
					128	129	2	**1.11**
					130	131	2	**1.00**
1112	RPS27A P62979: Ubiquitin-40S ribosomal protein S27a	1/3	6	10	126	127	1	**0.94**
					128	129	1	**0.72**
					130	131	1	**1.10**
1113	RPS3 P23396$_{1,2}$: 40S ribosomal protein S3	2/2	3	3	126	127	3	**1.67**
					128	129	3	**0.79**
					130	131	3	**1.19**
1114	RPS3A P61247: 40S ribosomal protein S3a		6	9	126	127	8	**2.28**
					128	129	9	**0.98**
					130	131	9	**0.83**
1115	RPS4X P62701: 40S ribosomal protein S4, X isoform	1/3	4	6	126	127	5	**1.72**
					128	129	6	**0.66**
					130	131	5	**1.03**

#	protein	group	peptides	spectra	ch1	ch2	quant	ratio
1116	RPS6 P62753: 40S ribosomal protein S6		7	9	126	127	9	**1.57**
					128	129	9	**0.74**
					130	131	8	**1.03**
1117	RPS7 P62081: 40S ribosomal protein S7		2	4	126	127	4	**2.06**
					128	129	4	**0.60**
					130	131	3	**1.10**
1118	RPS8 P62241: 40S ribosomal protein S8		2	2	126	127	2	**1.28**
					128	129	2	**0.69**
					130	131	2	**0.91**
1119	RPS9 P46781: 40S ribosomal protein S9		3	3	126	127	3	**0.95**
					128	129	3	**0.48**
					130	131	3	**1.12**
1120	RPSA P08865: 40S ribosomal protein SA		6	7	126	127	7	**0.47**
					128	129	6	**0.45**
					130	131	5	**1.22**
1121	RRBP1 Q9P2E9$_{1,2}$: Ribosome-binding protein 1	2/3	7	8	126	127	8	**4.95**
					128	129	7	**0.92**
					130	131	8	**0.75**
1122	RTCB Q9Y3I0: tRNA-splicing ligase RtcB homolog		2	2	126	127	2	**1.83**
					128	129	2	**0.83**
					130	131	2	**1.24**
1123	RTN4 Q9NQC3$_{1,2,5}$: Reticulon-4	3/3	1	1	126	127	1	**2.02**
					128	129	1	**1.00**
					130	131	1	**0.91**
1124	RYR3 Q15413$_{1,2}$: Ryanodine receptor 3	2/2	4	10	126	127	3	**0.37**
					128	129	6	**0.56**
					130	131	8	**0.88**
1125	S100A10 P60903: Protein S100-A10		6	19	126	127	16	**4.18**
					128	129	15	**1.79**
					130	131	15	**0.87**
1126	S100A11 P31949: Protein S100-A11		3	3	126	127	3	**7.36**
					128	129	3	**2.18**
					130	131	3	**0.65**
1127	S100A4 P26447: Protein S100-A4		3	4	126	127	3	**1.31**
					128	129	3	**1.11**
					130	131	4	**0.84**

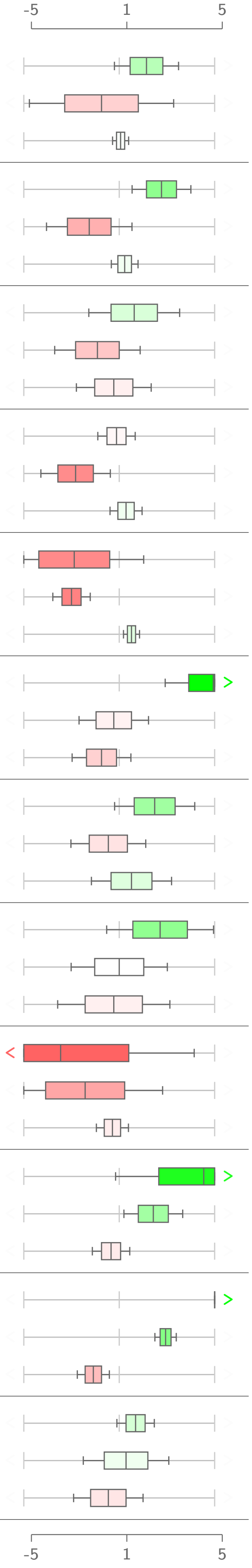

#	protein	group	peptides	spectra	ch1	ch2	quant	ratio
**1128**	S100A6 P06703: Protein S100-A6		4	8	126	127	8	**6.46**
					128	129	8	**1.58**
					130	131	8	**0.83**
**1129**	S100A8 P05109: Protein S100-A8		8	25	126	127	23	**4.82**
					128	129	25	**0.51**
					130	131	21	**0.94**
**1130**	S100A9 P06702: Protein S100-A9		5	7	126	127	7	**5.38**
					128	129	7	**0.81**
					130	131	7	**1.05**
**1131**	SAA1 P0DJI8: Serum amyloid A-1 protein	1/2	8	65	126	127	16	**2.01**
					128	129	17	**2.22**
					130	131	15	**0.69**
**1132**	SAA2 P0DJI9$_1$: Serum amyloid A-2 protein		8	55	126	127	6	**1.24**
					128	129	7	**2.82**
					130	131	7	**0.83**
**1133**	SAA4 P35542: Serum amyloid A-4 protein		7	130	126	127	122	**0.63**
					128	129	118	**0.52**
					130	131	125	**0.94**
**1134**	SAFB Q15424$_{1-4}$: Scaffold attachment factor B1	4/4	6	7	126	127	7	**0.73**
					128	129	7	**0.50**
					130	131	7	**1.12**
**1135**	SAMHD1 Q9Y3Z3$_1$: Deoxynucleoside triphosphate triphosphohydrolase SAMHD1	1/4	8	8	126	127	8	**0.57**
					128	129	8	**0.49**
					130	131	7	**0.98**
**1136**	SARS P49591: Serine--tRNA ligase, cytoplasmic		8	10	126	127	8	**0.81**
					128	129	8	**0.68**
					130	131	9	**1.10**
**1137**	SBSN Q6UWP8$_1$: Suprabasin		4	4	126	127	4	**1.24**
					128	129	4	**1.50**
					130	131	4	**0.88**
**1138**	SCG2 P13521: Secretogranin-2		30	46	126	127	43	**0.58**
					128	129	44	**1.08**
					130	131	41	**1.11**
**1139**	SCG3 Q8WXD2$_1$: Secretogranin-3	1/2	8	8	126	127	8	**0.65**
					128	129	7	**1.67**
					130	131	8	**1.04**

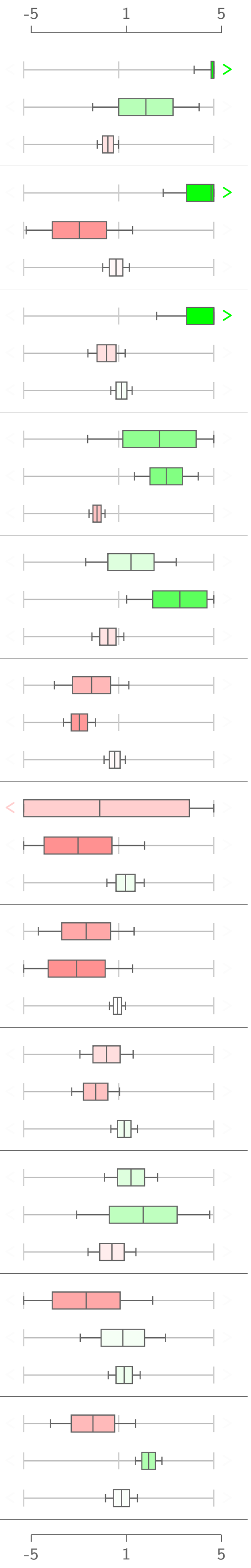

#	protein	group	peptides	spectra	ch1	ch2	quant	ratio
1140	SCG5 P05408$_1$: Neuroendocrine protein 7B2		2	11	126	127	2	**0.43**
					128	129	3	**1.41**
					130	131	3	**1.16**
1141	SCG5 P05408$_2$: Neuroendocrine protein 7B2		2	8	126	127	2	**0.30**
					128	129	2	**0.50**
					130	131	2	**1.03**
1142	SCGB1A1 P11684: Uteroglobin		3	5	126	127	5	**1.21**
					128	129	5	**1.98**
					130	131	5	**0.65**
1143	SCPEP1 Q9HB40$_1$: Retinoid-inducible serine carboxypeptidase		3	3	126	127	3	**0.58**
					128	129	3	**0.70**
					130	131	3	**0.85**
1144	SCRN1 Q12765$_{1,2}$: Secernin-1	2/3	3	4	126	127	3	**0.37**
					128	129	4	**1.35**
					130	131	4	**1.17**
1145	SDF4 Q9BRK5$_1$: 45 kDa calcium-binding protein	1/6	11	22	126	127	22	**0.69**
					128	129	22	**1.43**
					130	131	19	**0.95**
1146	SDPR O95810: Serum deprivation-response protein		3	4	126	127	4	**3.44**
					128	129	3	**1.04**
					130	131	3	**1.08**
1147	SEC23IP Q9Y6Y8$_1$: SEC23-interacting protein	1/2	4	5	126	127	3	**0.55**
					128	129	4	**0.42**
					130	131	3	**0.98**
1148	SEC31A O94979$_7$: Protein transport protein Sec31A	1/9	4	4	126	127	4	**0.77**
					128	129	4	**0.38**
					130	131	4	**1.03**
1149	SEMA3C Q99985$_1$: Semaphorin-3C		2	3	126	127	3	**1.53**
					128	129	3	**1.34**
					130	131	2	**1.10**
1150	SEMA3F Q13275$_{1,2}$: Semaphorin-3F	2/2	3	3	126	127	3	**0.86**
					128	129	3	**1.87**
					130	131	3	**0.89**
1151	SEMG1 P04279$_1$: Semenogelin-1	1/2	16	20	126	127	14	**0.33**
					128	129	12	**1.82**
					130	131	16	**2.46**

#	protein	group	peptides	spectra	ch1	ch2	quant	ratio
1152	SEMG2 Q02383: Semenogelin-2		9	10	126	127	3	**0.21**
					128	129	6	**1.84**
					130	131	6	**2.62**
1153	SEPP1 P49908: Selenoprotein P		6	10	126	127	7	**0.47**
					128	129	9	**1.61**
					130	131	9	**1.30**
1154	SEPT2 Q15019$_{1,2}$: Septin-2	2/3	3	3	126	127	3	**1.26**
					128	129	3	**0.72**
					130	131	3	**1.06**
1155	SEPT7 Q16181$_{1,2}$: Septin-7	2/2	6	53	126	127	34	**1.95**
					128	129	32	**0.69**
					130	131	40	**0.96**
1156	SEPT9 Q9UHD8$_{1,2,5,7}$: Septin-9	4/8	4	5	126	127	4	**2.57**
					128	129	5	**0.53**
					130	131	3	**0.80**
1157	SERBP1 Q8NC51$_{1-4}$: Plasminogen activator inhibitor 1 RNA-binding protein	4/4	1	1	126	127	1	**6.12**
					128	129	1	**1.36**
					130	131	1	**0.83**
1158	SERPINA1 P01009$_{1}$: Alpha-1-antitrypsin	1/3	25	205	126	127	196	**1.64**
					128	129	189	**1.88**
					130	131	193	**0.99**
1159	SERPINA10 Q9UK55: Protein Z-dependent protease inhibitor		13	28	126	127	28	**0.30**
					128	129	28	**0.26**
					130	131	27	**0.94**
1160	SERPINA11 Q86U17: Serpin A11		1	1	126	127	1	**0.19**
					128	129	1	**0.21**
					130	131	1	**0.96**
1161	SERPINA3 P01011$_{1}$: Alpha-1-antichymotrypsin	1/2	13	27	126	127	22	**0.82**
					128	129	24	**1.14**
					130	131	25	**0.96**
1162	SERPINA4 P29622: Kallistatin		11	16	126	127	14	**0.47**
					128	129	16	**0.29**
					130	131	14	**1.13**
1163	SERPINA5 P05154: Plasma serine protease inhibitor		10	16	126	127	15	**0.49**
					128	129	14	**0.52**
					130	131	15	**1.12**

#	protein	group	peptides	spectra	ch1	ch2	quant	ratio
**1164**	SERPINA6 P08185: Corticosteroid-binding globulin		2	2	126	127	2	**0.37**
					128	129	2	**0.45**
					130	131	2	**0.74**
**1165**	SERPINA7 P05543: Thyroxine-binding globulin		8	10	126	127	10	**0.63**
					128	129	10	**0.35**
					130	131	10	**0.97**
**1166**	SERPINB1 $P30740_{1,2}$: Leukocyte elastase inhibitor	2/2	2	2	126	127	2	**0.81**
					128	129	2	**0.62**
					130	131	2	**0.85**
**1167**	SERPINB2 P05120: Plasminogen activator inhibitor 2		2	3	126	127	3	**0.31**
					128	129	3	**0.14**
					130	131	3	**0.99**
**1168**	SERPINC1 P01008: Antithrombin-III		21	84	126	127	82	**0.56**
					128	129	84	**0.80**
					130	131	84	**1.02**
**1169**	SERPIND1 P05546: Heparin cofactor 2		20	148	126	127	133	**0.30**
					128	129	144	**0.18**
					130	131	143	**0.81**
**1170**	SERPINE1 $P05121_{1,2}$: Plasminogen activator inhibitor 1	2/2	6	9	126	127	9	**1.20**
					128	129	8	**0.96**
					130	131	9	**0.77**
**1171**	SERPINE2 $P07093_{1-3}$: Glia-derived nexin	3/3	4	4	126	127	4	**0.23**
					128	129	4	**0.32**
					130	131	3	**1.29**
**1172**	SERPINF1 P36955: Pigment epithelium-derived factor		15	34	126	127	33	**1.49**
					128	129	31	**4.03**
					130	131	32	**0.92**
**1173**	SERPINF2 $P08697_{1}$: Alpha-2-antiplasmin	1/2	14	89	126	127	89	**0.72**
					128	129	88	**0.63**
					130	131	89	**0.88**
**1174**	SERPING1 $P05155_{1-3}$: Plasma protease C1 inhibitor	3/3	6	9	126	127	7	**0.48**
					128	129	9	**0.47**
					130	131	8	**1.06**
**1175**	SERPINH1 P50454: Serpin H1		5	6	126	127	6	**4.35**
					128	129	6	**1.35**
					130	131	6	**0.91**

#	protein	group	peptides	spectra	ch1	ch2	quant	ratio
**1176**	SFN P31947$_{1,2}$: 14-3-3 protein sigma	2/2	7	13	126	127	3	**1.72**
					128	129	3	**1.69**
					130	131	3	**1.06**
**1177**	SFPQ P23246$_{1}$: Splicing factor, proline- and glutamine-rich	1/2	8	9	126	127	7	**3.06**
					128	129	6	**1.04**
					130	131	7	**0.86**
**1178**	SFRP1 Q8N474: Secreted frizzled-related protein 1		2	4	126	127	3	**2.07**
					128	129	4	**1.67**
					130	131	3	**0.99**
**1179**	SFRP2 Q96HF1: Secreted frizzled-related protein 2		11	35	126	127	33	**0.47**
					128	129	29	**1.37**
					130	131	33	**0.86**
**1180**	SFRP4 Q6FHJ7: Secreted frizzled-related protein 4		6	14	126	127	7	**0.78**
					128	129	6	**1.68**
					130	131	6	**0.95**
**1181**	SGTA O43765: Small glutamine-rich tetratricopeptide repeat-containing protein alpha		4	5	126	127	4	**0.76**
					128	129	5	**1.01**
					130	131	3	**1.02**
**1182**	SH3BGRL3 Q9H299: SH3 domain-binding glutamic acid-rich-like protein 3		2	4	126	127	4	**3.72**
					128	129	3	**0.94**
					130	131	4	**0.80**
**1183**	SH3GL1 Q99961$_{1-3}$: Endophilin-A2	3/3	2	2	126	127	2	**2.19**
					128	129	2	**1.73**
					130	131	2	**1.14**
**1184**	SHBG P04278$_{1-4}$: Sex hormone-binding globulin	4/5	10	20	126	127	18	**0.30**
					128	129	19	**0.29**
					130	131	17	**0.84**
**1185**	SKP1 P63208$_{1}$: S-phase kinase-associated protein 1	1/2	6	8	126	127	6	**0.52**
					128	129	7	**1.02**
					130	131	6	**1.17**
**1186**	SLC16A1 P53985$_{1}$: Monocarboxylate transporter 1	1/2	2	2	126	127	2	**1.15**
					128	129	2	**0.59**
					130	131	2	**0.83**
**1187**	SLC25A3 Q00325$_{1,2}$: Phosphate carrier protein, mitochondrial	2/2	1	1	126	127	1	**3.45**
					128	129	1	**0.37**
					130	131	1	**0.63**

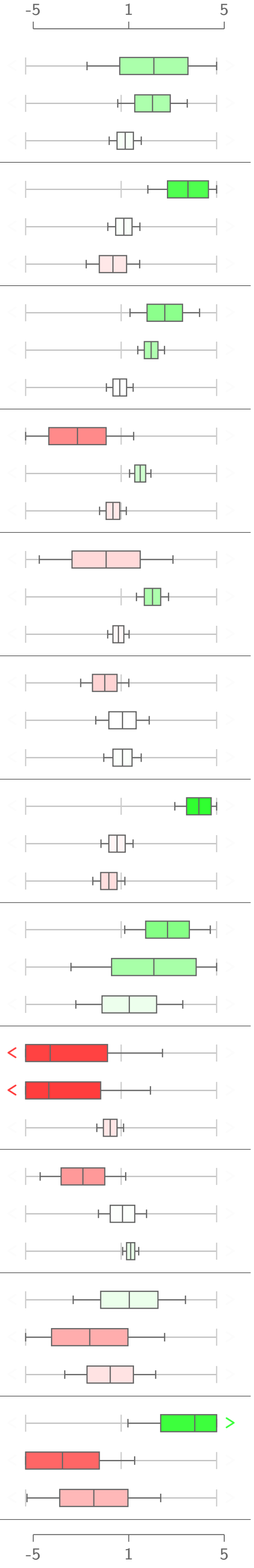

#	protein	group	peptides	spectra	ch1	ch2	quant	ratio
1188	SLC25A4 P12235, SLC25A5 P05141, SLC25A6 P12236: ADP/ATP translocase 1, ADP/ATP translocase 2, ...	3/3	2	2	126	127	2	**3.19**
					128	129	2	**0.79**
					130	131	2	**1.10**
1189	SLC9A3R1 O14745$_1$: Na(+)/H(+) exchange regulatory cofactor NHE-RF1	1/2	4	4	126	127	3	**2.82**
					128	129	3	**1.39**
					130	131	3	**0.70**
1190	SLIT3 O75094$_{1,2,4}$: Slit homolog 3 protein	3/4	6	11	126	127	10	**1.21**
					128	129	10	**0.76**
					130	131	6	**1.14**
1191	SLK Q9H2G2$_{1,2}$: STE20-like serine/threonine-protein kinase	2/2	3	3	126	127	3	**0.43**
					128	129	3	**0.40**
					130	131	3	**0.87**
1192	SLPI P03973: Antileukoproteinase		14	100	126	127	94	**0.97**
					128	129	92	**2.20**
					130	131	86	**0.93**
1193	SLURP1 P55000: Secreted Ly-6/uPAR-related protein 1		2	3	126	127	3	**1.90**
					128	129	3	**2.61**
					130	131	3	**0.70**
1194	SMARCC2 Q8TAQ2$_{1-3}$: SWI/SNF complex subunit SMARCC2	3/3	1	1	126	127	1	**2.21**
					128	129	1	**0.33**
					130	131	1	**0.84**
1195	SMOC2 Q9H3U7$_{1,2}$: SPARC-related modular calcium-binding protein 2	2/2	2	2	126	127	2	**1.01**
					128	129	2	**2.95**
					130	131	2	**0.91**
1196	SND1 Q7KZF4: Staphylococcal nuclease domain-containing protein 1		5	7	126	127	7	**0.96**
					128	129	7	**0.33**
					130	131	7	**1.15**
1197	SNED1 Q8TER0$_{1,3-5}$: Sushi, nidogen and EGF-like domain-containing protein 1	4/4	8	10	126	127	9	**0.38**
					128	129	6	**0.77**
					130	131	9	**0.86**
1198	SNRNP70 P08621$_{1,2}$: U1 small nuclear ribonucleoprotein 70 kDa	2/2	2	2	126	127	2	**1.53**
					128	129	2	**0.96**
					130	131	2	**0.96**
1199	SNRPD2 P62316$_1$: Small nuclear ribonucleoprotein Sm D2		2	2	126	127	2	**4.10**
					128	129	2	**0.93**
					130	131	2	**1.12**

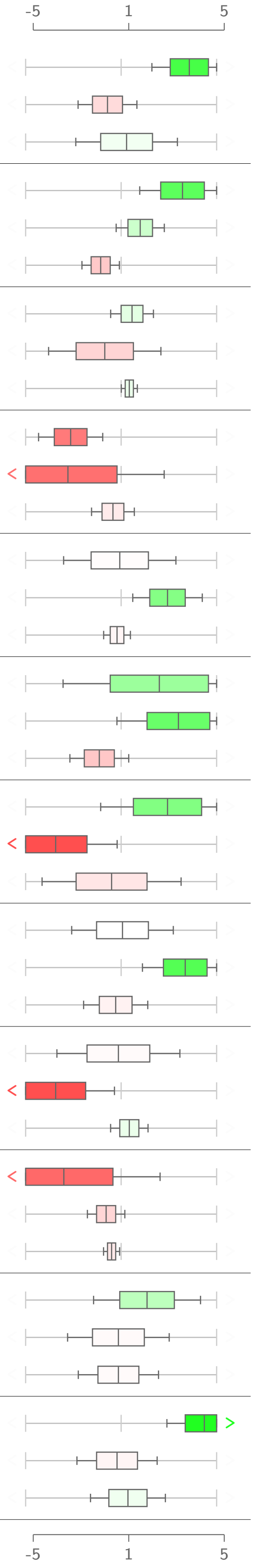

#	protein	group	peptides	spectra	ch1	ch2	quant	ratio
1200	SNRPF P62306: Small nuclear ribonucleoprotein F		1	2	126	127	2	**0.70**
					128	129	2	**0.45**
					130	131	2	**0.89**
1201	SNX3 O60493$_{1,2,4}$: Sorting nexin-3	3/3	2	3	126	127	3	**0.84**
					128	129	3	**0.32**
					130	131	3	**0.67**
1202	SNX5 Q9Y5X3$_{1}$: Sorting nexin-5		2	3	126	127	3	**0.75**
					128	129	3	**0.82**
					130	131	3	**0.81**
1203	SNX6 Q9UNH7$_{1}$: Sorting nexin-6	1/2	4	5	126	127	4	**0.78**
					128	129	5	**0.85**
					130	131	4	**0.85**
1204	SOCS7 O14512$_{1,2}$: Suppressor of cytokine signaling 7	2/2	3	141	126	127	90	**2.76**
					128	129	108	**2.26**
					130	131	71	**0.97**
1205	SOD2 P04179$_{1,2,4}$: Superoxide dismutase [Mn], mitochondrial	3/3	2	2	126	127	2	**11.49**
					128	129	2	**2.92**
					130	131	2	**1.16**
1206	SOD3 P08294: Extracellular superoxide dismutase [Cu-Zn]		10	67	126	127	62	**1.50**
					128	129	58	**2.03**
					130	131	66	**1.08**
1207	SORT1 Q99523$_{1}$: Sortilin		1	1	126	127	1	**0.47**
					128	129	1	**0.29**
					130	131	1	**0.97**
1208	SOS1 Q07889$_{1}$: Son of sevenless homolog 1		2	2	126	127	2	**3.19**
					128	129	2	**1.37**
					130	131	2	**0.76**
1209	SPARC P09486: SPARC		15	50	126	127	48	**0.28**
					128	129	47	**3.80**
					130	131	49	**1.13**
1210	SPARCL1 Q14515$_{1}$: SPARC-like protein 1	1/2	33	82	126	127	70	**0.49**
					128	129	72	**1.45**
					130	131	80	**1.18**
1211	SPCS2 Q15005: Signal peptidase complex subunit 2		2	2	126	127	2	**1.39**
					128	129	2	**0.82**
					130	131	2	**0.72**

#	protein	group	peptides	spectra	ch1	ch2	quant	ratio
1212	SPON1 Q9HCB6: Spondin-1		23	50	126	127	46	**0.80**
					128	129	40	**2.38**
					130	131	45	**1.03**
1213	SPP1 P10451$_{1-5}$: Osteopontin	5/5	3	3	126	127	3	**0.16**
					128	129	3	**2.94**
					130	131	3	**1.10**
1214	SPP2 Q13103: Secreted phosphoprotein 24		9	18	126	127	17	**0.83**
					128	129	16	**0.58**
					130	131	17	**1.22**
1215	SPTAN1 Q13813$_{1-3}$: Spectrin alpha chain, non-erythrocytic 1	3/3	10	51	126	127	47	**3.94**
					128	129	42	**2.25**
					130	131	41	**0.93**
1216	SPTBN1 Q01082$_{1-3}$: Spectrin beta chain, non-erythrocytic 1	3/3	12	23	126	127	19	**1.69**
					128	129	18	**0.70**
					130	131	21	**0.93**
1217	SRI P30626$_{1-3}$: Sorcin	3/3	3	3	126	127	3	**1.22**
					128	129	3	**1.12**
					130	131	3	**0.86**
1218	SRPX P78539$_{1,4}$: Sushi repeat-containing protein SRPX	2/5	11	17	126	127	14	**0.21**
					128	129	17	**0.23**
					130	131	17	**1.00**
1219	SRRM1 Q8IYB3$_{1,2}$: Serine/arginine repetitive matrix protein 1	2/2	2	6	126	127	6	**0.44**
					128	129	4	**2.49**
					130	131	6	**1.10**
1220	SRSF1 Q07955$_{1}$: Serine/arginine-rich splicing factor 1	1/3	7	12	126	127	11	**2.11**
					128	129	11	**1.07**
					130	131	11	**1.04**
1221	SRSF3 P84103$_{1,2}$: Serine/arginine-rich splicing factor 3	2/2	5	54	126	127	48	**6.57**
					128	129	44	**2.09**
					130	131	53	**0.92**
1222	SRSF7 Q16629$_{1-4}$: Serine/arginine-rich splicing factor 7	4/4	4	6	126	127	3	**1.29**
					128	129	3	**0.93**
					130	131	3	**0.89**
1223	SSC5D A1L4H1$_{1,2}$: Soluble scavenger receptor cysteine-rich domain-containing protein SSC5D	2/2	6	7	126	127	5	**0.92**
					128	129	5	**1.11**
					130	131	6	**0.99**

-5 1 5

#	protein	group	peptides	spectra	ch1	ch2	quant	ratio
1224	ST13 P50502: Hsc70-interacting protein	1/2	9	13	126	127	7	1.49
					128	129	6	1.42
					130	131	7	0.99
1225	ST13P4 Q8IZP2: Putative protein FAM10A4		6	7	126	127	1	0.79
					128	129	1	0.92
					130	131	1	1.17
1226	ST6GAL1 $P15907_1$: Beta-galactoside alpha-2,6-sialyltransferase 1		1	1	126	127	1	0.59
					128	129	1	0.81
					130	131	1	0.87
1227	STAB1 $Q9NY15_1$: Stabilin-1	1/2	27	54	126	127	47	1.30
					128	129	50	0.85
					130	131	43	0.95
1228	STAT5A $P42229_{1,2}$, STAT5B P51692: Signal transducer and activator of transcription 5A, Signal transducer and activator of transcription 5B	3/3	2	2	126	127	2	< 0.26
					128	129	2	< 0.20
					130	131	2	0.83
1229	STIP1 $P31948_{1-3}$: Stress-induced-phosphoprotein 1	3/3	3	5	126	127	5	2.11
					128	129	4	1.60
					130	131	5	0.70
1230	STXBP2 $Q15833_{1-3}$: Syntaxin-binding protein 2	3/3	2	2	126	127	2	0.42
					128	129	2	< 0.31
					130	131	2	0.90
1231	SUB1 P53999: Activated RNA polymerase II transcriptional coactivator p15		6	8	126	127	8	2.24
					128	129	8	1.19
					130	131	8	1.01
1232	SULF1 Q8IWU6: Extracellular sulfatase Sulf-1		11	13	126	127	12	1.02
					128	129	13	1.20
					130	131	13	0.91
1233	SULT1A1 $P50225_{1,2}$, SULT1A3 $P0DMM9_{1,3}$, SULT1A4 P0DMN0: Sulfotransferase 1A1, Sulfotransferase 1A3, ...	5/6	2	2	126	127	2	0.56
					128	129	2	0.37
					130	131	2	1.12
1234	SUSD5 O60279: Sushi domain-containing protein 5		4	7	126	127	7	0.53
					128	129	7	1.10
					130	131	7	1.20
1235	SVEP1 $Q4LDE5_1$: Sushi, von Willebrand factor type A, EGF and pentraxin domain-containing pro ...	1/4	57	89	126	127	85	0.53
					128	129	89	0.88
					130	131	83	0.92

#	protein	group	peptides	spectra	ch1	ch2	quant	ratio
**1236**	SYNCRIP O60506$_{3,4}$: Heterogeneous nuclear ribonucleoprotein Q	2/9	7	8	126	127	5	**1.32**
					128	129	7	**0.67**
					130	131	7	**0.90**
**1237**	TAF15 Q92804$_{1,2}$: TATA-binding protein-associated factor 2N	2/2	5	5	126	127	3	**5.94**
					128	129	3	**3.01**
					130	131	3	**1.49**
**1238**	TAGLN Q01995: Transgelin		8	13	126	127	12	**0.48**
					128	129	11	**0.28**
					130	131	12	**0.94**
**1239**	TAGLN2 P37802$_{1,2}$: Transgelin-2	2/2	12	17	126	127	17	**1.31**
					128	129	17	**0.64**
					130	131	17	**0.91**
**1240**	TARDBP Q13148$_{1}$: TAR DNA-binding protein 43	1/2	3	4	126	127	4	**1.06**
					128	129	4	**0.64**
					130	131	3	**1.10**
**1241**	TBCB Q99426$_{1,2}$: Tubulin-folding cofactor B	2/2	2	2	126	127	2	**0.56**
					128	129	2	**0.58**
					130	131	2	**1.08**
**1242**	TCN2 P20062$_{1}$: Transcobalamin-2	1/2	6	7	126	127	6	**0.46**
					128	129	7	**0.41**
					130	131	6	**0.76**
**1243**	TCP1 P17987: T-complex protein 1 subunit alpha		2	2	126	127	2	**0.76**
					128	129	2	**0.25**
					130	131	2	**1.12**
**1244**	TDRD15 B5MCY1: Tudor domain-containing protein 15		6	68	126	127	55	**2.38**
					128	129	54	**1.76**
					130	131	51	**1.00**
**1245**	TF P02787: Serotransferrin		35	58	126	127	54	**4.46**
					128	129	57	**2.51**
					130	131	56	**1.13**
**1246**	TFG Q92734$_{1-4}$: Protein TFG	4/4	4	4	126	127	4	**1.03**
					128	129	3	**0.74**
					130	131	4	**1.19**
**1247**	TFPI P10646$_{1}$: Tissue factor pathway inhibitor		4	4	126	127	4	**1.52**
					128	129	4	**1.13**
					130	131	3	**1.14**

#	protein	group	peptides	spectra	ch1	ch2	quant	ratio
**1248**	TFPI2 P48307$_{1,2}$: Tissue factor pathway inhibitor 2	2/2	6	7	126	127	6	**1.61**
					128	129	6	**1.14**
					130	131	5	**0.99**
**1249**	TGFBI Q15582: Transforming growth factor-beta-induced protein ig-h3		25	48	126	127	43	**0.29**
					128	129	46	**0.58**
					130	131	46	**0.81**
**1250**	TGFBR3 Q03167$_{1,2}$: Transforming growth factor beta receptor type 3	2/2	9	12	126	127	10	**0.28**
					128	129	12	**0.56**
					130	131	10	**0.91**
**1251**	TGM2 P21980$_{1}$: Protein-glutamine gamma-glutamyltransferase 2	1/2	8	9	126	127	7	**2.00**
					128	129	7	**2.03**
					130	131	7	**1.09**
**1252**	TGM4 P49221: Protein-glutamine gamma-glutamyltransferase 4		3	5	126	127	3	**0.97**
					128	129	3	**1.49**
					130	131	3	**1.02**
**1253**	THBS1 P07996$_{1}$: Thrombospondin-1	1/2	40	88	126	127	75	**0.49**
					128	129	78	**0.57**
					130	131	76	**0.93**
**1254**	THBS2 P35442: Thrombospondin-2		37	81	126	127	71	**0.52**
					128	129	67	**0.98**
					130	131	70	**0.95**
**1255**	THBS3 P49746$_{1}$: Thrombospondin-3	1/2	9	10	126	127	9	**0.86**
					128	129	9	**0.84**
					130	131	9	**0.98**
**1256**	THBS4 P35443: Thrombospondin-4		22	54	126	127	33	**1.10**
					128	129	44	**0.20**
					130	131	45	**0.93**
**1257**	THSD4 Q6ZMP0$_{1}$: Thrombospondin type-1 domain-containing protein 4	1/2	11	17	126	127	14	**1.03**
					128	129	14	**1.39**
					130	131	12	**0.97**
**1258**	TIMP1 P01033: Metalloproteinase inhibitor 1		5	7	126	127	7	**2.37**
					128	129	7	**4.14**
					130	131	7	**0.95**
**1259**	TIMP2 P16035: Metalloproteinase inhibitor 2		6	15	126	127	9	**0.82**
					128	129	10	**4.27**
					130	131	9	**0.83**

#	protein	group	peptides	spectra	ch1	ch2	quant	ratio
**1260**	TIMP4 Q99727: Metalloproteinase inhibitor 4		2	2	126	127	2	**1.53**
					128	129	2	**3.21**
					130	131	2	**0.86**
**1261**	TINAGL1 Q9GZM7$_{1,3}$: Tubulointerstitial nephritis antigen-like	2/2	3	20	126	127	12	**1.58**
					128	129	11	**1.25**
					130	131	15	**0.96**
**1262**	TIPRL O75663$_{1}$: TIP41-like protein	1/2	3	3	126	127	3	**0.49**
					128	129	3	**0.69**
					130	131	3	**0.87**
**1264**	TLL1 O43897$_{1}$: Tolloid-like protein 1		6	7	126	127	2	**0.77**
					128	129	2	**1.70**
					130	131	2	**0.51**
**1265**	TLN1 Q9Y490: Talin-1		53	76	126	127	59	**0.63**
					128	129	62	**0.28**
					130	131	62	**0.90**
**1266**	TLN2 Q9Y4G6: Talin-2		11	14	126	127	3	**0.29**
					128	129	3	**0.64**
					130	131	3	**1.16**
**1267**	TMED2 Q15363: Transmembrane emp24 domain-containing protein 2		4	4	126	127	4	**1.15**
					128	129	4	**0.74**
					130	131	3	**0.91**
**1268**	TMEM43 Q9BTV4: Transmembrane protein 43		1	1	126	127	1	**1.25**
					128	129	1	**0.35**
					130	131	1	**0.79**
**1269**	TMEM59 Q9BXS4: Transmembrane protein 59		2	4	126	127	1	**0.37**
					128	129	1	**0.72**
					130	131	1	**1.07**
**1270**	TMOD3 Q9NYL9: Tropomodulin-3		1	1	126	127	1	**2.22**
					128	129	1	**1.35**
					130	131	1	**0.93**
**1271**	TMSB4X P62328: Thymosin beta-4		2	2	126	127	2	**3.31**
					128	129	2	**0.91**
					130	131	2	**1.04**
**1272**	TNC P24821$_{1,4}$: Tenascin	2/5	37	58	126	127	1	**0.23**
					128	129	1	**0.47**
					130	131	1	**0.89**

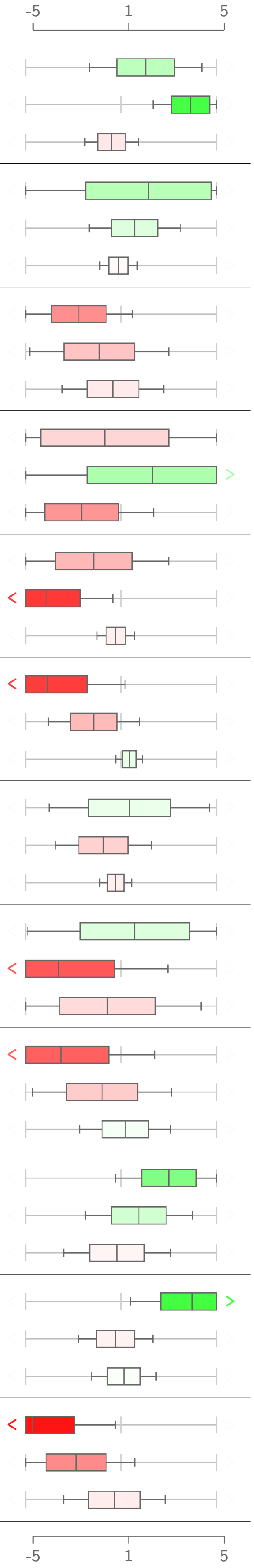

#	protein	group	peptides	spectra	ch1	ch2	quant	ratio
**1273**	TNC P24821$_6$: Tenascin		37	58	126	127	1	**0.71**
					128	129	1	**0.66**
					130	131	1	**1.40**
**1274**	TNFAIP6 P98066: Tumor necrosis factor-inducible gene 6 protein		4	5	126	127	5	**0.37**
					128	129	5	**1.15**
					130	131	5	**1.16**
**1275**	TNNT1 P13805$_{1-3}$: Troponin T, slow skeletal muscle	3/11	5	16	126	127	10	**0.65**
					128	129	8	**0.85**
					130	131	11	**1.79**
**1276**	TNNT2 P45379$_{3,4}$: Troponin T, cardiac muscle	2/2	2	5	126	127	1	**2.58**
					128	129	1	**0.84**
					130	131	1	**0.94**
**1277**	TNNT3 P45378$_{1-7}$: Troponin T, fast skeletal muscle	7/7	2	2	126	127	1	**0.64**
					128	129	1	**0.41**
					130	131	1	**1.34**
**1278**	TNPO1 Q92973$_{1-3}$: Transport-in-1	3/3	4	5	126	127	4	**0.95**
					128	129	4	**0.59**
					130	131	3	**1.19**
**1279**	TNPO2 O14787$_2$: Transportin-2	1/2	4	4	126	127	2	**0.46**
					128	129	2	**0.29**
					130	131	3	**0.99**
**1280**	TNXB P22105$_{1,3,4}$: Tenascin-X	3/5	66	93	126	127	81	**0.67**
					128	129	86	**1.04**
					130	131	81	**1.02**
**1281**	TPI1 P60174$_{1,3,4}$: Triosephosphate isomerase	3/3	1	1	126	127	1	**8.74**
					128	129	1	**0.97**
					130	131	1	**0.99**
**1284**	TPM1 P09493$_5$: Tropomyosin alpha-1 chain		17	33	126	127	1	**1.04**
					128	129	1	**1.78**
					130	131	1	**1.13**
**1288**	TPM2 P07951$_1$: Tropomyosin beta chain		23	43	126	127	4	**0.50**
					128	129	4	**1.22**
					130	131	4	**1.47**
**1289**	TPM2 P07951$_2$: Tropomyosin beta chain	1/2	24	50	126	127	13	**0.69**
					128	129	13	**1.37**
					130	131	13	**1.20**

#	protein	group	peptides	spectra	ch1	ch2	quant	ratio
1290	TPM3 P06753$_1$: Tropomyosin alpha-3 chain		20	36	126	127	1	**0.33**
					128	129	1	**1.87**
					130	131	1	**1.49**
1291	TPM3 P06753$_2$: Tropomyosin alpha-3 chain	1/3	21	39	126	127	4	**0.90**
					128	129	4	**0.77**
					130	131	3	**0.93**
1293	TPM4 P67936$_1$: Tropomyosin alpha-4 chain		22	41	126	127	7	**0.82**
					128	129	7	**1.13**
					130	131	7	**1.11**
1295	TPP1 O14773$_{1,2}$: Tripeptidyl-peptidase 1	2/2	3	4	126	127	4	**1.05**
					128	129	4	**1.15**
					130	131	3	**1.07**
1296	TRH P20396: Pro-thyrotropin-releasing hormone		3	5	126	127	3	**2.29**
					128	129	3	**0.80**
					130	131	5	**1.32**
1297	TSKU Q8WUA8: Tsukushin		6	9	126	127	9	**0.36**
					128	129	9	**0.23**
					130	131	9	**0.78**
1298	TTN Q8WZ42$_{12}$: Titin	1/12	35	74	126	127	45	**5.11**
					128	129	47	**2.08**
					130	131	49	**1.26**
1299	TTN Q8WZ42$_6$: Titin		4	4	126	127	1	**0.48**
					128	129	1	**0.79**
					130	131	1	**0.89**
1300	TTR P02766: Transthyretin		8	78	126	127	73	**0.91**
					128	129	68	**1.59**
					130	131	72	**1.11**
1302	TUBA1C Q9BQE3: Tubulin alpha-1C chain		16	38	126	127	1	**2.27**
					128	129	1	**0.86**
					130	131	1	**1.08**
1303	TUBA4A P68366$_{1,2}$: Tubulin alpha-4A chain	2/6	16	34	126	127	2	**0.84**
					128	129	2	**0.40**
					130	131	2	**1.15**
1304	TUBB P07437: Tubulin beta chain	1/5	14	48	126	127	5	**0.29**
					128	129	4	**0.26**
					130	131	5	**1.13**

#	protein	group	peptides	spectra	ch1	ch2	quant	ratio
**1305**	TUBB3 Q13509$_1$: Tubulin beta-3 chain		10	23	126	127	1	**7.19**
					128	129	1	**0.24**
					130	131	1	**0.83**
**1306**	TUBB4A P04350: Tubulin beta-4A chain	1/2	11	31	126	127	1	**0.81**
					128	129	1	**0.48**
					130	131	1	**1.24**
**1307**	TUBB4B P68371: Tubulin beta-4B chain	1/3	14	45	126	127	2	**2.10**
					128	129	2	**0.92**
					130	131	2	**1.27**
**1308**	TWSG1 Q9GZX9$_1$: Twisted gastrulation protein homolog 1	1/2	4	5	126	127	2	**0.92**
					128	129	2	**4.77**
					130	131	2	**0.99**
**1309**	TXNDC5 Q8NBS9$_{1,2}$: Thioredoxin domain-containing protein 5	2/2	5	6	126	127	4	**2.15**
					128	129	4	**2.31**
					130	131	4	**1.03**
**1310**	TXNL1 O43396: Thioredoxin-like protein 1		1	1	126	127	1	**0.55**
					128	129	1	**0.75**
					130	131	1	**1.09**
**1311**	TYMP P19971$_1$: Thymidine phosphorylase	1/2	5	7	126	127	6	**0.43**
					128	129	7	**0.53**
					130	131	7	**0.97**
**1312**	UBA1 P22314$_{1,2}$: Ubiquitin-like modifier-activating enzyme 1	2/2	12	14	126	127	14	**0.76**
					128	129	14	**0.35**
					130	131	9	**1.08**
**1313**	UBA52 P62987: Ubiquitin-60S ribosomal protein L40		6	10	126	127	1	**2.66**
					128	129	1	**1.12**
					130	131	1	**1.02**
**1314**	UBA6 A0AVT1$_1$: Ubiquitin-like modifier-activating enzyme 6		4	4	126	127	4	**0.96**
					128	129	4	**1.04**
					130	131	4	**1.03**
**1315**	UBE2K P61086$_{1,3}$: Ubiquitin-conjugating enzyme E2 K	2/2	1	1	126	127	1	**0.33**
					128	129	1	**0.15**
					130	131	1	**0.44**
**1316**	UBE2N P61088, UBE2NL Q5JXB2: Ubiquitin-conjugating enzyme E2 N, Putative ubiquitin-conjugating enzyme E2 N-like	2/2	3	3	126	127	3	**0.53**
					128	129	3	**0.21**
					130	131	3	**0.98**

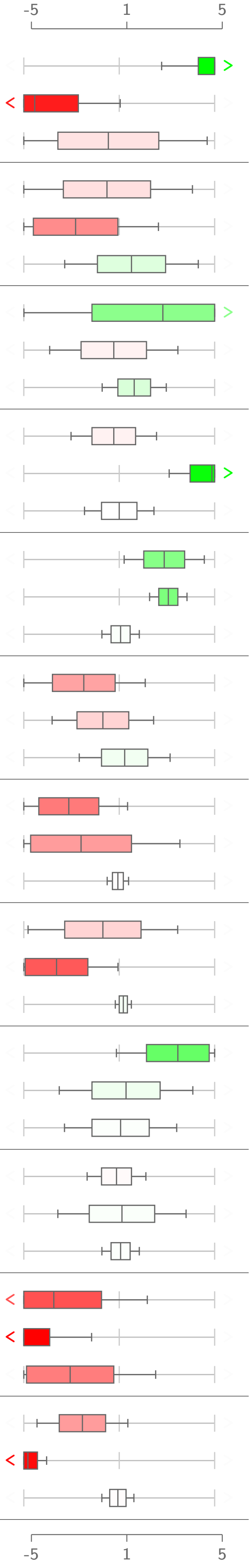

#	protein	group	peptides	spectra	ch1	ch2	quant	ratio
**1317**	UBE2V1 Q13404$_{1,2,4,6,7}$: Ubiquitin-conjugating enzyme E2 variant 1	5/5	2	2	126	127	2	**0.41**
					128	129	2	**0.22**
					130	131	2	**0.82**
**1318**	UBR4 Q5T4S7$_{1-4}$: E3 ubiquitin-protein ligase UBR4	4/4	7	17	126	127	11	**0.90**
					128	129	16	**1.94**
					130	131	16	**1.48**
**1319**	UGDH O60701$_{1}$: UDP-glucose 6-dehydrogenase	1/3	8	8	126	127	8	**0.46**
					128	129	8	**0.45**
					130	131	6	**1.13**
**1320**	UGP2 Q16851$_{2}$: UTP--glucose-1-phosphate uridylyltransferase	1/2	7	9	126	127	9	**1.01**
					128	129	9	**0.51**
					130	131	9	**0.92**
**1321**	UQCRB P14927$_{1,2}$: Cytochrome b-c1 complex subunit 7	2/2	1	1	126	127		
					128	129	1	**2.65**
					130	131	1	**0.38**
**1322**	UQCRC1 P31930: Cytochrome b-c1 complex subunit 1, mitochondrial		1	1	126	127	1	**5.81**
					128	129	1	**1.11**
					130	131	1	**0.94**
**1323**	UQCRC2 P22695: Cytochrome b-c1 complex subunit 2, mitochondrial		2	4	126	127	3	**0.08**
					128	129	4	**0.14**
					130	131	4	**0.89**
**1324**	UQCRH P07919: Cytochrome b-c1 complex subunit 6, mitochondrial	1/2	3	3	126	127	3	**4.00**
					128	129	3	**1.73**
					130	131	3	**0.83**
**1325**	USO1 O60763$_{1,2}$: General vesicular transport factor p115	2/2	6	7	126	127	7	**0.26**
					128	129	6	**0.23**
					130	131	6	**1.04**
**1326**	USP5 P45974$_{1,2}$: Ubiquitin carboxyl-terminal hydrolase 5	2/2	4	4	126	127	4	**0.77**
					128	129	4	**0.73**
					130	131	3	**1.20**
**1327**	VAPA Q9P0L0$_{1,2}$: Vesicle-associated membrane protein-associated protein A	2/2	1	1	126	127	1	**1.44**
					128	129	1	**0.26**
					130	131	1	**0.87**
**1328**	VASP P50552: Vasodilator-stimulated phosphoprotein		2	2	126	127	2	**1.36**
					128	129	2	**0.72**
					130	131	2	**0.92**

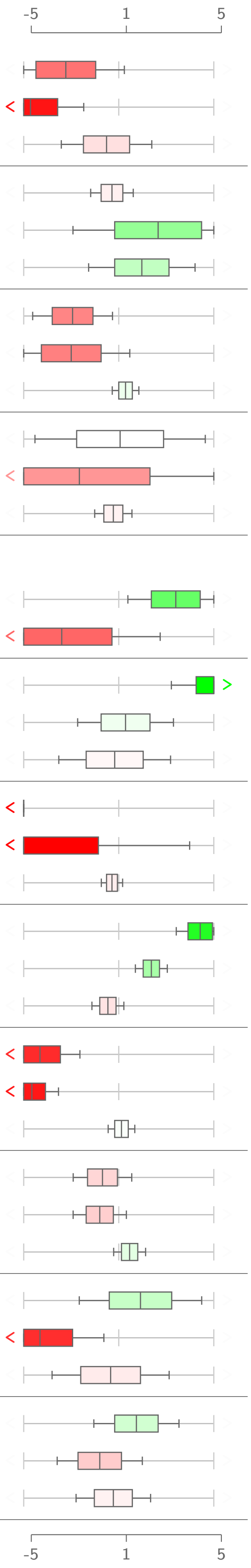

#	protein	group	peptides	spectra	ch1	ch2	quant	ratio
**1329**	VAT1 Q99536$_1$: Synaptic vesicle membrane protein VAT-1 homolog	1/3	5	8	126	127	7	**0.53**
					128	129	7	**0.33**
					130	131	7	**1.13**
**1330**	VCAM1 P19320$_{1,3}$: Vascular cell adhesion protein 1	2/3	10	13	126	127	12	**0.61**
					128	129	11	**0.62**
					130	131	13	**0.92**
**1331**	VCAN P13611$_{1-5}$: Versican core protein	5/5	5	6	126	127	3	**0.91**
					128	129	5	**0.79**
					130	131	5	**1.05**
**1332**	VCL P18206$_{1,2}$: Vinculin	2/3	32	41	126	127	41	**0.63**
					128	129	40	**0.21**
					130	131	41	**0.91**
**1333**	VCP P55072: Transitional endoplasmic reticulum ATPase		11	12	126	127	10	**1.45**
					128	129	11	**0.86**
					130	131	12	**1.14**
**1334**	VDAC1 P21796: Voltage-dependent anion-selective channel protein 1		2	2	126	127	2	**11.92**
					128	129	2	**1.19**
					130	131	2	**0.71**
**1335**	VEGFC P49767: Vascular endothelial growth factor C		15	26	126	127	24	**0.68**
					128	129	23	**1.34**
					130	131	24	**0.91**
**1336**	VGF O15240: Neurosecretory protein VGF [Cleaved into: Neuroendocrine regulatory peptide-1		7	15	126	127	7	**0.22**
					128	129	12	**1.65**
					130	131	12	**1.25**
**1337**	VIM P08670: Vimentin	1/5	37	183	126	127	113	**1.99**
					128	129	130	**0.64**
					130	131	120	**0.99**
**1338**	VIPAS39 Q9H9C1$_{1,2}$: Spermatogenesis-defective protein 39 homolog	2/2	1	1	126	127	1	**0.19**
					128	129	1	**0.66**
					130	131	1	**0.83**
**1339**	VLDLR P98155$_{1,2}$: Very low-density lipoprotein receptor	2/2	9	19	126	127	14	**1.69**
					128	129	14	**1.64**
					130	131	11	**1.00**
**1340**	VPS29 Q9UBQ0$_{1,2}$: Vacuolar protein sorting-associated protein 29	2/2	1	1	126	127	1	**0.57**
					128	129	1	**0.36**
					130	131	1	**1.01**

#	protein	group	peptides	spectra	ch1	ch2	quant	ratio
**1341**	VPS35 Q96QK1: Vacuolar protein sorting-associated protein 35		6	7	126	127	7	**1.54**
					128	129	5	**0.37**
					130	131	6	**1.07**
**1342**	VSIG4 $Q9Y279_{1,2}$: V-set and immunoglobulin domain-containing protein 4	2/2	3	3	126	127	3	**0.86**
					128	129	3	**0.84**
					130	131	3	**1.32**
**1343**	VTN P04004: Vitronectin		18	1395	126	127	1200	**0.27**
					128	129	1288	**0.51**
					130	131	1345	**1.19**
**1344**	VWF $P04275_{1}$: von Willebrand factor	1/2	59	84	126	127	76	**0.64**
					128	129	76	**0.29**
					130	131	79	**1.00**
**1345**	WARS $P23381_{1,2}$: Tryptophan--tRNA ligase, cytoplasmic	2/2	3	3	126	127	3	**0.51**
					128	129	3	**0.44**
					130	131	3	**1.00**
**1346**	WDFY3 $Q8IZQ1_{1,2}$: WD repeat and FYVE domain-containing protein 3	2/2	2	15	126	127	14	**1.33**
					128	129	12	**1.25**
					130	131	13	**1.00**
**1347**	WFDC1 Q9HC57: WAP four-disulfide core domain protein 1		3	4	126	127	3	**1.38**
					128	129	4	**1.52**
					130	131	4	**0.80**
**1348**	WISP2 $O76076_{1}$: WNT1-inducible-signaling pathway protein 2	1/2	9	47	126	127	42	**0.97**
					128	129	44	**1.40**
					130	131	44	**0.99**
**1349**	WWC3 $Q9ULE0_{1,2}$: Protein WWC3	2/2	2	4	126	127	2	**1.00**
					128	129	2	**3.46**
					130	131	2	**0.52**
**1350**	XRCC5 P13010: X-ray repair cross-complementing protein 5		2	5	126	127	5	**0.51**
					128	129	3	**0.40**
					130	131	4	**1.01**
**1351**	XRCC6 $P12956_{1,2}$: X-ray repair cross-complementing protein 6	2/2	5	5	126	127	3	**1.02**
					128	129	5	**0.49**
					130	131	5	**1.07**
**1352**	YARS P54577: Tyrosine--tRNA ligase, cytoplasmic		2	2	126	127	2	**0.39**
					128	129	2	**0.32**
					130	131	2	**0.83**

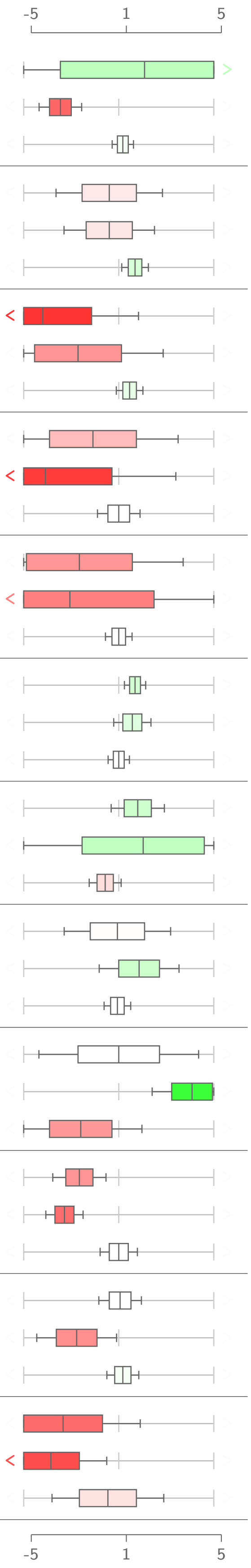

#	protein	group	peptides	spectra	ch1	ch2	quant	ratio
**1353**	YBX1 P67809: Nuclease-sensitive element-binding protein 1		2	3	126	127	3	**2.25**
					128	129	3	**0.99**
					130	131	3	**1.01**
**1354**	YKT6 O15498$_1$: Synaptobrevin homolog YKT6	1/2	3	4	126	127	4	**0.46**
					128	129	3	**0.27**
					130	131	3	**0.73**
**1355**	YWHAB P31946$_{1,2}$: 14-3-3 protein beta/alpha	2/2	12	33	126	127	3	**0.46**
					128	129	4	**0.54**
					130	131	4	**1.03**
**1356**	YWHAE P62258$_1$: 14-3-3 protein epsilon	1/2	15	24	126	127	16	**0.35**
					128	129	15	**0.94**
					130	131	16	**1.12**
**1357**	YWHAG P61981: 14-3-3 protein gamma		15	39	126	127	19	**0.33**
					128	129	18	**0.85**
					130	131	18	**1.12**
**1358**	YWHAH Q04917: 14-3-3 protein eta		12	29	126	127	15	**0.26**
					128	129	15	**0.52**
					130	131	17	**1.07**
**1359**	YWHAQ P27348: 14-3-3 protein theta		16	39	126	127	18	**0.49**
					128	129	17	**0.65**
					130	131	16	**1.14**
**1360**	YWHAZ P63104$_1$: 14-3-3 protein zeta/delta	1/2	18	51	126	127	28	**0.47**
					128	129	29	**0.74**
					130	131	28	**1.09**
**1361**	ZNF236 Q9UL36$_{1,2}$: Zinc finger protein 236	2/2	3	10	126	127	7	**1.79**
					128	129	8	**1.89**
					130	131	7	**0.82**
**1362**	ZNF320 A2RRD8: Zinc finger protein 320	1/3	3	17	126	127	10	**1.66**
					128	129	11	**1.23**
					130	131	10	**0.85**
**1363**	ZNF428 Q96B54: Zinc finger protein 428		1	1	126	127	1	**0.21**
					128	129	1	**2.40**
					130	131	1	**1.05**
**1364**	ZNF85 Q03923$_{1,2}$: Zinc finger protein 85	2/2	3	40	126	127	23	**0.91**
					128	129	22	**1.12**
					130	131	30	**1.07**

#	protein	group	peptides	spectra	ch1	ch2	quant	ratio
**1365**	ZYX Q15942$_{1,2}$: Zyxin	2/2	3	3	126	127	3	**1.62**
					128	129	3	**0.99**
					130	131	3	**1.01**
**1366**	ZZEF1 O43149$_{1}$: Zinc finger ZZ-type and EF-hand domain-containing protein 1	1/2	6	28	126	127	8	**1.09**
					128	129	11	**1.14**
					130	131	11	**1.15**

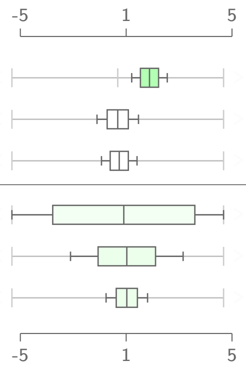

1.2 Not Quantified Proteins

Number of identified but not quantified proteins: P15-treat/P15-ctrl: 54; P17-treat/P17-ctrl: 56; P5-treat/P5-ctrl: 61

#	protein	group	peptides	spectra
640	IGHV1-46 P01743, A0A0C4DH29: Immunoglobulin heavy variable 1-46	2/2	2	18
540	HIST1H2BC P62807, HIST1H2BD P58876, HIST1H2BH Q93079, . . . : Histone H2B type 1-C/E/F/G/I, Histone H2B type 1-D, . . .	7/8	11	72
439	FCN3 O75636$_{2}$: Ficolin-3		10	21
729	KRT75 O95678: Keratin, type II cytoskeletal 75		14	45
684	ITGBL1 O95965$_{2}$: Integrin beta-like protein 1		10	14
644	IGHV3-33 P01772: Immunoglobulin heavy variable 3-33		2	3
548	HLA-A P01891: HLA class I histocompatibility antigen, A-68 alpha chain		6	8
726	KRT6B P04259: Keratin, type II cytoskeletal 6B		23	53
549	HLA-A P04439, HLA-A P13746$_{1,2}$: HLA class I histocompatibility antigen, A-3 alpha chain, HLA class I histocompatibility antigen, A-11 alpha chain	3/5	5	7
1292	TPM3 P06753$_{5}$: Tropomyosin alpha-3 chain	1/3	23	42
539	HIST1H2BB P33778, HIST1H2BJ P06899, HIST1H2BO P23527, . . . : Histone H2B type 1-B, Histone H2B type 1-J, . . .	4/6	11	70
1282	TPM1 P09493$_{2}$: Tropomyosin alpha-1 chain		15	29
1283	TPM1 P09493$_{3}$: Tropomyosin alpha-1 chain	1/3	20	35
1285	TPM1 P09493$_{7}$: Tropomyosin alpha-1 chain	1/2	20	38
1286	TPM1 P09493$_{8}$: Tropomyosin alpha-1 chain		19	35
1287	TPM1 P09493$_{9}$: Tropomyosin alpha-1 chain	1/2	21	38
538	HIST1H2AC Q93077, HIST1H2AG P0C0S8, HIST1H2AH Q96KK5, . . . : Histone H2A type 1-C, Histone H2A type 1, . . .	5/5	6	23
661	IGLC6 P0CF74: Ig lambda-6 chain C region		4	92
976	POTEI P0CG38, POTEJ P0CG39: POTE ankyrin domain family member I, POTE ankyrin domain family member J	2/2	11	97
843	MYH6 P13533: Myosin-6		6	6
551	HLA-A P16188: HLA class I histocompatibility antigen, A-30 alpha chain		6	10
552	HLA-A P16190: HLA class I histocompatibility antigen, A-33 alpha chain	1/5	6	10
724	KRT4 P19013: Keratin, type II cytoskeletal 4		5	14
555	HLA-B P30460, HLA-B P30480: HLA class I histocompatibility antigen, B-8 alpha chain, HLA class I histocompatibility antigen, B-42 alpha chain	2/26	8	13
556	HLA-B P30464, HLA-B P30484: HLA class I histocompatibility antigen, B-15 alpha chain, HLA class I histocompatibility antigen, B-46 alpha chain	2/2	6	11
557	HLA-B P30492: HLA class I histocompatibility antigen, B-54 alpha chain		7	11

#	protein	group	peptides	spectra
558	HLA-B P30493, HLA-B Q29940: HLA class I histocompatibility antigen, B-55 alpha chain, HLA class I histocompatibility antigen, B-59 alpha chain	2/5	7	12
561	HLA-C P30508, HLA-C Q29963: HLA class I histocompatibility antigen, Cw-12 alpha chain, HLA class I histocompatibility antigen, Cw-6 alpha chain	2/16	9	12
554	HLA-A P30512: HLA class I histocompatibility antigen, A-29 alpha chain	1/6	6	9
603	HSPA1L P34931: Heat shock 70 kDa protein 1-like		10	16
17	ACTN2 P35609$_2$: Alpha-actinin-2	1/2	5	6
719	KRT20 P35900: Keratin, type I cytoskeletal 20		4	12
1004	PRPH P41219$_{1,2}$: Peripherin	2/2	5	38
727	KRT6C P48668: Keratin, type II cytoskeletal 6C	1/2	23	53
1294	TPM4 P67936$_2$: Tropomyosin alpha-4 chain		19	38
1301	TUBA1B P68363$_1$: Tubulin alpha-1B chain	1/7	17	39
730	KRT76 Q01546: Keratin, type II cytoskeletal 2 oral		8	32
715	KRT17 Q04695: Keratin, type I cytoskeletal 17		13	28
967	POSTN Q15063$_2$: Periostin		38	180
968	POSTN Q15063$_3$: Periostin		41	211
969	POSTN Q15063$_4$: Periostin		38	170
970	POSTN Q15063$_5$: Periostin		41	221
971	POSTN Q15063$_6$: Periostin		36	167
972	POSTN Q15063$_7$: Periostin		36	157
951	PLEC Q15149$_1$: Plectin		95	107
559	HLA-B Q31612: HLA class I histocompatibility antigen, B-73 alpha chain		7	10
1263	TKFC Q3LXA3$_{1,2}$: Triokinase/FMN cyclase	2/2	1	1
732	KRT79 Q5XKE5: Keratin, type II cytoskeletal 79		8	22
547	HIST3H2BB Q8N257: Histone H2B type 3-B		10	56
480	GCDH Q92947$_{1,2}$: Glutaryl-CoA dehydrogenase, mitochondrial	2/2	2	2
892	OPTN Q96CV9$_{1-3}$: Optineurin	3/3	2	51
541	HIST1H2BL Q99880: Histone H2B type 1-L	1/2	11	65
506	GRID1 Q9ULK0$_1$: Glutamate receptor ionotropic, delta-1		2	9

2 Protein Group Details

Protein groups are created by grouping according to mass spectrometry evidence:

1. Proteins which are detected with the exact same set of peptides are clustered - they are indistinguishable based on available MS data. Very often these are splice variants. In the following text, proteins are clustered proteins (cluster size one if they have specific peptides).
2. Proteins, which have unique peptides (*i. e.* peptides not present in other detected proteins) are promoted to *reporters*.
3. Remaining proteins are grouped to those reporters which have a superset of the peptides they have. These proteins are termed *group members*

Peptides are characterized based on their specificity to one or more proteins, and one or more protein groups:

reporter specific rs — Number of peptides specific to reporter.
group specific gs — Number of peptides specific to the group (including rs).
unspecific us — Number of peptides shared with any protein.

1 136429, NA, NA 7/0/0

2 162648, NA, NA 22/0/6

3		A0A0A0MS15, NA, NA	1/0/0
4		A0A0B4J1V0, NA, NA	2/0/0
5		A0A0C4DH25, NA, NA	1/0/2
6		A0A0C4DH33, NA, NA	2/0/1
7	(1)	P04217-1, A1BG, Alpha-1B-glycoprotein	3/0/0
	(2)	P04217-2, A1BG, Alpha-1B-glycoprotein	
8		P01023, A2M, Alpha-2-macroglobulin	35/0/8
9		P49588-1,2, AARS, Alanine--tRNA ligase, cytoplasmic	9/0/0
10		Q96IU4-1, ABHD14B, Protein ABHD14B	2/0/0
11		Q7Z7G0-1, ABI3BP, Target of Nesh-SH3	3/0/0
12	(1)	P53396-1,2, ACLY, ATP-citrate synthase	1/0/0
	(2)	P53396-3, ACLY, ATP-citrate synthase	
13	(1)	P62736, ACTA2, Actin, aortic smooth muscle	0/5/10
	·	P68032, ACTC1, Actin, alpha cardiac muscle 1	
	(2)	P68133, ACTA1, Actin, alpha skeletal muscle	
	(3)	P63267-1, ACTG2, Actin, gamma-enteric smooth muscle	
	(4)	P63267-2, ACTG2, Actin, gamma-enteric smooth muscle	
14	(1)	P60709, ACTB, Actin, cytoplasmic 1	6/0/13
	·	P63261, ACTG1, Actin, cytoplasmic 2	
	(2)	Q9BYX7, POTEKP, Putative beta-actin-like protein 3	
15		Q562R1, ACTBL2, Beta-actin-like protein 2	1/0/6
16		P12814-1-4, ACTN1, Alpha-actinin-1	3/0/11
17	(1)	P35609-2, ACTN2, Alpha-actinin-2	0/0/5
	(2)	P35609-1, ACTN2, Alpha-actinin-2	
18		Q08043, ACTN3, Alpha-actinin-3	3/0/4
19	(1)	O43707-1, ACTN4, Alpha-actinin-4	0/8/9
	(2)	O43707-2, ACTN4, Alpha-actinin-4	
	(3)	O43707-3, ACTN4, Alpha-actinin-4	
20		P61163, ACTR1A, Alpha-centractin	2/0/1
21		P61160-1, ACTR2, Actin-related protein 2	3/0/1
22		P61158, ACTR3, Actin-related protein 3	3/0/0
23		O14672-1, ADAM10, Disintegrin and metalloproteinase domain-containing protein 10	5/0/0

24	(1)	Q13444-1-10,12, ADAM15, Disintegrin and metalloproteinase domain-containing protein 15	0/0/0
	(2)	Q13444-11, ADAM15, Disintegrin and metalloproteinase domain-containing protein 15	
	(3)	Q13444-13, ADAM15, Disintegrin and metalloproteinase domain-containing protein 15	
25		O15204-1,2, ADAMDEC1, ADAM DEC1	3/0/0
26		Q9UHI8, ADAMTS1, A disintegrin and metalloproteinase with thrombospondin motifs 1	1/0/0
27		Q8TE58, ADAMTS15, A disintegrin and metalloproteinase with thrombospondin motifs 15	5/0/0
28		O95450-1,2, ADAMTS2, A disintegrin and metalloproteinase with thrombospondin motifs 2	5/0/0
29		Q9UNA0, ADAMTS5, A disintegrin and metalloproteinase with thrombospondin motifs 5	5/0/0
30		Q9UP79, ADAMTS8, A disintegrin and metalloproteinase with thrombospondin motifs 8	2/0/0
31	(1)	Q8N6G6-3, ADAMTSL1, ADAMTS-like protein 1	1/0/0
	(2)	Q8N6G6-4, ADAMTSL1, ADAMTS-like protein 1	
	(3)	Q8N6G6-6, ADAMTSL1, ADAMTS-like protein 1	
32		Q86TH1, ADAMTSL2, ADAMTS-like protein 2	2/0/0
33	(1)	Q6UY14-1,3, ADAMTSL4, ADAMTS-like protein 4	5/0/0
	(2)	Q6UY14-2, ADAMTSL4, ADAMTS-like protein 4	
34		P11766, ADH5, Alcohol dehydrogenase class-3	8/0/0
35		Q15848, ADIPOQ, Adiponectin	3/0/0
36	(1)	Q8IUX7-1, AEBP1, Adipocyte enhancer-binding protein 1	16/0/0
	(2)	Q8IUX7-2, AEBP1, Adipocyte enhancer-binding protein 1	
37		P43652, AFM, Afamin	27/0/0
38		O00468-1-7, AGRN, Agrin [Cleaved into: Agrin N-terminal 110 kDa subunit; Agrin C-terminal 110 kDa subunit; Agrin C-terminal 90 kDa fragment	9/0/0
39		P01019, AGT, Angiotensinogen	8/0/0
40		Q09666-1, AHNAK, Neuroblast differentiation-associated protein AHNAK	30/0/1
41	(1)	O95433-1, AHSA1, Activator of 90 kDa heat shock protein ATPase homolog 1	1/0/0
	(2)	O95433-2, AHSA1, Activator of 90 kDa heat shock protein ATPase homolog 1	
42		P02765, AHSG, Alpha-2-HS-glycoprotein	13/0/0

43 P00568, AK1, Adenylate kinase isoenzyme 1 — 4/0/0

44 Q7Z591-1,2,4,5,7,8, AKNA, AT-hook-containing transcription factor — 1/0/0

45 — 5/23/6
(1) P02768-1, ALB, Serum albumin
(2) P02768-2, ALB, Serum albumin
(3) P02768-3, ALB, Serum albumin

46 — 0/0/1
(1) O94788-1, ALDH1A2, Retinal dehydrogenase 2
(2) O94788-2, ALDH1A2, Retinal dehydrogenase 2

47 P47895, ALDH1A3, Aldehyde dehydrogenase family 1 member A3 — 7/0/1

48 P49419-1,2,4, ALDH7A1, Alpha-aminoadipic semialdehyde dehydrogenase — 2/0/0

49 — 2/0/0
(1) P49189-1, ALDH9A1, 4-trimethylaminobutyraldehyde dehydrogenase
(2) P49189-2, ALDH9A1, 4-trimethylaminobutyraldehyde dehydrogenase

50 — 5/0/0
(1) P04075-1,2, ALDOA, Fructose-bisphosphate aldolase A
(2) P09972, ALDOC, Fructose-bisphosphate aldolase C

51 P16050-1,2, ALOX15, Arachidonate 15-lipoxygenase — 2/0/0

52 Q86V81, ALYREF, THO complex subunit 4 — 2/0/0

53 P02760, AMBP, Protein AMBP [Cleaved into: Alpha-1-microglobulin — 15/0/0

54 P03950, ANG, Angiogenin — 12/0/0

55 O95841, ANGPTL1, Angiopoietin-related protein 1 — 2/0/1

56 — 4/0/1
(1) Q9UKU9-1, ANGPTL2, Angiopoietin-related protein 2
(2) Q9UKU9-2, ANGPTL2, Angiopoietin-related protein 2

57 — 0/3/0
(1) Q9BY76-1, ANGPTL4, Angiopoietin-related protein 4
(2) Q9BY76-2, ANGPTL4, Angiopoietin-related protein 4
(3) Q9BY76-3, ANGPTL4, Angiopoietin-related protein 4

58 Q6UXH0, ANGPTL8, Angiopoietin-like protein 8 — 2/0/0

59 P16157-1,3,5,8,10,12,14,16,21, ANK1, Ankyrin-1 — 3/0/0

60 — 2/4/1
(1) Q9H6X2-1, ANTXR1, Anthrax toxin receptor 1
(2) Q9H6X2-5, ANTXR1, Anthrax toxin receptor 1
(3) Q9H6X2-2-4,6, ANTXR1, Anthrax toxin receptor 1

61 — 1/0/1
(1) P58335-1,2,4, ANTXR2, Anthrax toxin receptor 2

	(2)	P58335-3, ANTXR2, Anthrax toxin receptor 2	
62		P04083, ANXA1, Annexin A1	9/0/0
63		P50995-1,2, ANXA11, Annexin A11	3/0/0
64	(1)	P07355-1,2, ANXA2, Annexin A2	5/0/0
	(2)	A6NMY6, ANXA2P2, Putative annexin A2-like protein	
65	(1)	P09525-1, ANXA4, Annexin A4	0/0/2
	(2)	P09525-2, ANXA4, Annexin A4	
66		P08758, ANXA5, Annexin A5	6/0/2
67		P08133-1,2, ANXA6, Annexin A6	12/0/0
68		Q10567-1-4, AP1B1, AP-1 complex subunit beta-1	1/0/4
69		O43747-1,2, AP1G1, AP-1 complex subunit gamma-1	2/0/0
70		Q9BXS5-1,2, AP1M1, AP-1 complex subunit mu-1	2/0/0
71		O94973-1-3, AP2A2, AP-2 complex subunit alpha-2<br>O95782-1,2, AP2A1, AP-2 complex subunit alpha-1	3/0/0
72	(1)	P63010-1,2, AP2B1, AP-2 complex subunit beta	1/0/4
	(2)	P63010-3, AP2B1, AP-2 complex subunit beta	
73		Q96CW1-1,2, AP2M1, AP-2 complex subunit mu	2/0/0
74	(1)	O14617-5, AP3D1, AP-3 complex subunit delta-1	1/0/0
	(2)	O14617-1-4, AP3D1, AP-3 complex subunit delta-1	
75		P02743, APCS, Serum amyloid P-component	9/0/0
76		P13798, APEH, Acylamino-acid-releasing enzyme	1/0/0
77	(1)	Q9BZZ5-1,2,4-6, API5, Apoptosis inhibitor 5	1/0/0
	(2)	Q9BZZ5-3, API5, Apoptosis inhibitor 5	
78	(1)	Q06481-1,3,6, APLP2, Amyloid-like protein 2	3/0/0
	(2)	Q06481-2,4,5, APLP2, Amyloid-like protein 2	
79	(1)	Q9HDC9-1, APMAP, Adipocyte plasma membrane-associated protein	1/0/0
	(2)	Q9HDC9-2, APMAP, Adipocyte plasma membrane-associated protein	
80		P02647, APOA1, Apolipoprotein A-I	33/0/1
81		P02652, APOA2, Apolipoprotein A-II	5/0/0
82		P06727, APOA4, Apolipoprotein A-IV	38/0/2
83		Q6Q788, APOA5, Apolipoprotein A-V	4/0/0
84		P04114, APOB, Apolipoprotein B-100	213/0/2

85	(1)	Q0VD83-1,3,4, APOBR, Apolipoprotein B receptor	3/0/0
	(2)	Q0VD83-2, APOBR, Apolipoprotein B receptor	
86		P02654, APOC1, Apolipoprotein C-I	6/0/0
87		P02655, APOC2, Apolipoprotein C-II	4/0/0
88		P02656, APOC3, Apolipoprotein C-III	5/0/0
89		P55056, APOC4, Apolipoprotein C-IV	6/0/0
90		P05090, APOD, Apolipoprotein D	8/0/0
91		P02649, APOE, Apolipoprotein E	27/0/0
92		Q13790, APOF, Apolipoprotein F	5/0/0
93		P02749, APOH, Beta-2-glycoprotein 1	12/0/0
94		O14791-1-3, APOL1, Apolipoprotein L1	14/0/1
95		Q9BQE5, APOL2, Apolipoprotein L2	4/0/0
96		O95236-1-3, APOL3, Apolipoprotein L3	1/0/1
97	(1)	O95445-1, APOM, Apolipoprotein M	1/0/0
	(2)	O95445-2, APOM, Apolipoprotein M	
98	(1)	P05067-1,7-9,11, APP, Amyloid beta A4 protein	1/6/0
	(2)	P05067-3-6, APP, Amyloid beta A4 protein	
	(3)	P05067-2, APP, Amyloid beta A4 protein	
	(4)	P05067-10, APP, Amyloid beta A4 protein	
99		P07741-1,2, APRT, Adenine phosphoribosyltransferase	3/0/0
100		P48444-1,2, ARCN1, Coatomer subunit delta	4/0/0
101		Q07960, ARHGAP1, Rho GTPase-activating protein 1	4/0/0
102		Q68EM7-1,2,5,6, ARHGAP17, Rho GTPase-activating protein 17	2/0/0
103	(1)	P52565-1, ARHGDIA, Rho GDP-dissociation inhibitor 1	2/0/0
	(2)	P52565-2, ARHGDIA, Rho GDP-dissociation inhibitor 1	
104		P52566, ARHGDIB, Rho GDP-dissociation inhibitor 2	5/0/0
105		Q92888-1-4, ARHGEF1, Rho guanine nucleotide exchange factor 1	3/0/0
106		O15511-1,2, ARPC5, Actin-related protein 2/3 complex subunit 5	2/0/0
107		P07307-1-3, ASGR2, Asialoglycoprotein receptor 2	4/0/0
108		Q12797-1,10, ASPH, Aspartyl/asparaginyl beta-hydroxylase	6/0/0

109		Q9BZE9-1-4, ASPSCR1, Tether containing UBX domain for GLUT4	1/0/0
110		P31939-1,2, ATIC, Bifunctional purine biosynthesis protein PURH [Cleaved into: Bifunctional purine biosynthesis protein PURH, N-terminally processed] [Includes: Phosphoribosylaminoimidazolecarboxamide formyltransferase	4/0/0
111		Q6DD88, ATL3, Atlastin-3	4/0/0
112	(1)	P25705-1,2, ATP5A1, ATP synthase subunit alpha, mitochondrial	1/0/0
	(2)	P25705-3, ATP5A1, ATP synthase subunit alpha, mitochondrial	
113		P30049, ATP5D, ATP synthase subunit delta, mitochondrial	2/0/0
114		O75947-1,2, ATP5H, ATP synthase subunit d, mitochondrial	2/0/0
115		P48047, ATP5O, ATP synthase subunit O, mitochondrial	2/0/0
116		Q15904, ATP6AP1, V-type proton ATPase subunit S1	3/0/0
117		O75787-1,2, ATP6AP2, Renin receptor	5/0/0
118		P15313, ATP6V1B1, V-type proton ATPase subunit B, kidney isoform	3/0/0
		P21281, ATP6V1B2, V-type proton ATPase subunit B, brain isoform	
119		P36543-1,3, ATP6V1E1, V-type proton ATPase subunit E 1	3/0/0
		Q96A05, ATP6V1E2, V-type proton ATPase subunit E 2	
120		O75348, ATP6V1G1, V-type proton ATPase subunit G 1	2/0/0
121	(1)	P46100-1,4, ATRX, Transcriptional regulator ATRX	0/2/10
	(2)	P46100-2, ATRX, Transcriptional regulator ATRX	
	(3)	P46100-3,5, ATRX, Transcriptional regulator ATRX	
122		P46100-6, ATRX, Transcriptional regulator ATRX	1/0/10
123		P30530-1,2, AXL, Tyrosine-protein kinase receptor UFO	2/0/0
124		P25311, AZGP1, Zinc-alpha-2-glycoprotein	6/0/0
125		P20160, AZU1, Azurocidin	2/0/0
126		P61769, B2M, Beta-2-microglobulin [Cleaved into: Beta-2-microglobulin form pI 5.3]	5/0/0
127		O94766-1,2, B3GAT3, Galactosylgalactosylxylosylprotein 3-beta-glucuronosyltransferase 3	4/0/0
128		Q6UX72, B3GNT9, UDP-GlcNAc:betaGal beta-1,3-N-acetylglucosaminyltransferase 9	1/0/0
129		P15291-1,2, B4GALT1, Beta-1,4-galactosyltransferase 1	5/0/0
130		O60513, B4GALT4, Beta-1,4-galactosyltransferase 4	3/0/0
131		O43505, B4GAT1, Beta-1,4-glucuronyltransferase 1	3/0/0

			0/3/0
132	(1)	Q07812-1,2,8, BAX, Apoptosis regulator BAX	
	(2)	Q07812-4, BAX, Apoptosis regulator BAX	
	(3)	Q07812-5, BAX, Apoptosis regulator BAX	
	(4)	Q07812-7, BAX, Apoptosis regulator BAX	
133		P51572-1,2, BCAP31, B-cell receptor-associated protein 31	1/0/0
134		P06276, BCHE, Cholinesterase	6/0/0
135		Q86U42-1,2, PABPN1, Polyadenylate-binding protein 2	2/0/0
		Q92843-2, BCL2L2, Bcl-2-like protein 2	
			1/0/0
136	(1)	Q9BUT1-1, BDH2, 3-hydroxybutyrate dehydrogenase type 2	
	(2)	Q9BUT1-2, BDH2, 3-hydroxybutyrate dehydrogenase type 2	
137		P21810, BGN, Biglycan	4/0/1
138		P55957-1,2, BID, BH3-interacting domain death agonist	1/0/0
139		P53004, BLVRA, Biliverdin reductase A	3/0/0
140		P30043, BLVRB, Flavin reductase	5/0/0
141	(1)	P13497-1, BMP1, Bone morphogenetic protein 1	4/0/18
	(2)	P13497-5,6, BMP1, Bone morphogenetic protein 1	
	(3)	Q9Y6L7, TLL2, Tolloid-like protein 2	
	(4)	O43897-2, TLL1, Tolloid-like protein 1	
	(5)	P13497-3, BMP1, Bone morphogenetic protein 1	
	(6)	P13497-4, BMP1, Bone morphogenetic protein 1	
142		P13497-2, BMP1, Bone morphogenetic protein 1	1/0/17
143		P12645, BMP3, Bone morphogenetic protein 3	2/0/0
144		Q9H3K6-1,2, BOLA2, BolA-like protein 2	1/0/0
145		Q8TDL5-1, BPIFB1, BPI fold-containing family B member 1	4/0/0
146		Q12830-1,2,4, BPTF, Nucleosome-remodeling factor subunit BPTF	4/0/1
147		P43251-1-4, BTD, Biotinidase	3/0/0
148		Q6UX73-1,2, C16orf89, UPF0764 protein C16orf89	3/0/0
149		P02745, C1QA, Complement C1q subcomponent subunit A	4/0/0
150		P02746, C1QB, Complement C1q subcomponent subunit B	5/0/0
151		P02747, C1QC, Complement C1q subcomponent subunit C	3/0/0
152		Q9BXJ1-1,2, C1QTNF1, Complement C1q tumor necrosis factor-related protein 1	2/0/0
153		P00736, C1R, Complement C1r subcomponent	30/0/2

154		Q9NZP8, C1RL, Complement C1r subcomponent-like protein	9/0/2
155		P09871, C1S, Complement C1s subcomponent	25/0/1
			2/20/0
156	(1)	P06681-1, C2, Complement C2	
	(2)	P06681-2, C2, Complement C2	
	(3)	P06681-3, C2, Complement C2	
157		Q9H1Z8, C2orf40, Augurin	3/0/0
158		P01024, C3, Complement C3	116/0/0
			0/0/85
159	(1)	P0C0L4-1, C4A, Complement C4-A	
	(2)	P0C0L4-2, C4A, Complement C4-A	
160		P0C0L5, C4B, Complement C4-B	5/0/85
161		P04003, C4BPA, C4b-binding protein alpha chain	33/0/0
162		P20851-1,2, C4BPB, C4b-binding protein beta chain	11/0/0
163		P01031, C5, Complement C5	55/0/0
164		P13671, C6, Complement component C6	40/0/0
165		P10643, C7, Complement component C7	32/0/0
166		P07357, C8A, Complement component C8 alpha chain	21/0/0
167		P07358, C8B, Complement component C8 beta chain	24/0/0
168		P07360, C8G, Complement component C8 gamma chain	9/0/0
169		P02748, C9, Complement component C9 [Cleaved into: Complement component C9a; Complement component C9b]	30/0/1
			1/0/0
170	(1)	Q01668-2, CACNA1D, Voltage-dependent L-type calcium channel subunit alpha-1D	
	(2)	Q01668-1,3,4, CACNA1D, Voltage-dependent L-type calcium channel subunit alpha-1D	
			1/0/1
171	(1)	Q9HB71-1, CACYBP, Calcyclin-binding protein	
	(2)	Q9HB71-2, CACYBP, Calcyclin-binding protein	
172		P62158, CALM1, Calmodulin	6/0/2
173		P27482, CALML3, Calmodulin-like protein 3	1/0/2
174		P27797, CALR, Calreticulin	18/0/0

0/6/1

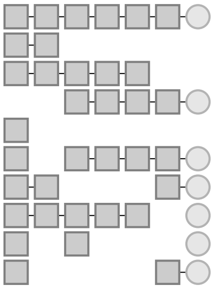

175 (1) O43852-1,3, CALU, Calumenin
(2) O43852-11,13,14, CALU, Calumenin
(3) O43852-10, CALU, Calumenin
(4) O43852-15, CALU, Calumenin
(5) O43852-7,12, CALU, Calumenin
(6) O43852-2,4, CALU, Calumenin
(7) O43852-5, CALU, Calumenin
(8) O43852-6, CALU, Calumenin
(9) O43852-8, CALU, Calumenin
(10) O43852-9, CALU, Calumenin

1/1/0

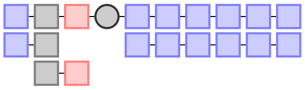

176 (1) Q86VP6-1, CAND1, Cullin-associated NEDD8-dissociated protein 1
(2) Q86VP6-2, CAND1, Cullin-associated NEDD8-dissociated protein 1
(3) Q86VP6-3, CAND1, Cullin-associated NEDD8-dissociated protein 1

2/0/0

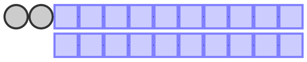

177 (1) P27824-1,2, CANX, Calnexin
(2) P27824-3, CANX, Calnexin

178 Q01518-1,2, CAP1, Adenylyl cyclase-associated protein 1

11/0/0

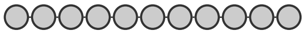

179 P40121-1,2, CAPG, Macrophage-capping protein

6/0/0

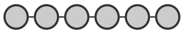

180 P07384, CAPN1, Calpain-1 catalytic subunit

2/0/0

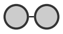

4/0/0

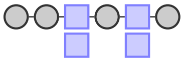

181 (1) P17655-1, CAPN2, Calpain-2 catalytic subunit
(2) P17655-2, CAPN2, Calpain-2 catalytic subunit

182 P04632, CAPNS1, Calpain small subunit 1

4/0/0

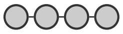

183 Q14444-1,2, CAPRIN1, Caprin-1

2/0/0

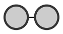

184 P52907, CAPZA1, F-actin-capping protein subunit alpha-1

4/0/1

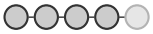

0/0/1

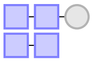

185 (1) P47755-1, CAPZA2, F-actin-capping protein subunit alpha-2
(2) P47755-2, CAPZA2, F-actin-capping protein subunit alpha-2

1/0/0

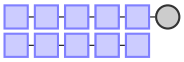

186 (1) P47756-2, CAPZB, F-actin-capping protein subunit beta
(2) P47756-1, CAPZB, F-actin-capping protein subunit beta

3/0/0

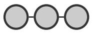

187 Q9BXL7, CARD11, Caspase recruitment domain-containing protein 11

2/2/0 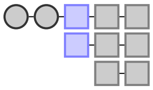

188 (1) P20810-5-7,9,10, CAST, Calpastatin
(2) P20810-1,2,4,8, CAST, Calpastatin
(3) P20810-3, CAST, Calpastatin

4/0/0 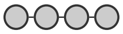

189 P16152-1, CBR1, Carbonyl reductase [NADPH] 1

1/0/0 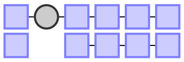

190 (1) A2RUR9-1, CCDC144A, Coiled-coil domain-containing protein 144A
(2) A2RUR9-3, CCDC144A, Coiled-coil domain-containing protein 144A

1/0/0 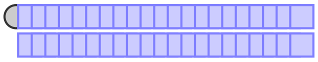

191 (1) Q76M96-1, CCDC80, Coiled-coil domain-containing protein 80
(2) Q76M96-2, CCDC80, Coiled-coil domain-containing protein 80

1/0/0 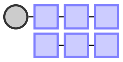

192 (1) Q16627-1, CCL14, C-C motif chemokine 14
(2) Q16627-2, CCL14, C-C motif chemokine 14

4/0/0 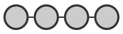

193 P55774, CCL18, C-C motif chemokine 18

2/0/0 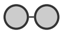

194 O14618, CCS, Copper chaperone for superoxide dismutase

1/0/0 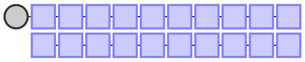

195 (1) P78371-1, CCT2, T-complex protein 1 subunit beta
(2) P78371-2, CCT2, T-complex protein 1 subunit beta

1/0/0 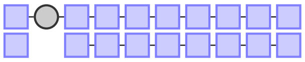

196 (1) P49368-1, CCT3, T-complex protein 1 subunit gamma
(2) P49368-2, CCT3, T-complex protein 1 subunit gamma

1/0/0 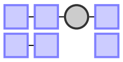

197 (1) P50991-1, CCT4, T-complex protein 1 subunit delta
(2) P50991-2, CCT4, T-complex protein 1 subunit delta

7/0/0 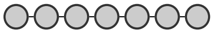

198 P48643-1,2, CCT5, T-complex protein 1 subunit epsilon

4/0/0 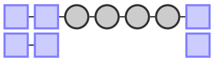

199 (1) P40227-1,2, CCT6A, T-complex protein 1 subunit zeta
(2) Q92526-1-3, CCT6B, T-complex protein 1 subunit zeta-2

1/4/0 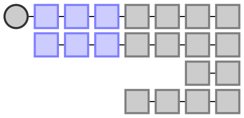

200 (1) Q99832-3, CCT7, T-complex protein 1 subunit eta
(2) Q99832-1, CCT7, T-complex protein 1 subunit eta
(3) Q99832-2, CCT7, T-complex protein 1 subunit eta
(4) Q99832-4, CCT7, T-complex protein 1 subunit eta

2/0/0 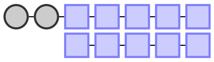

201 (1) P50990-1,2, CCT8, T-complex protein 1 subunit theta
(2) P50990-3, CCT8, T-complex protein 1 subunit theta

5/0/0 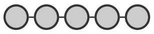

202 Q6YHK3-1,2,4, CD109, CD109 antigen

14/0/0 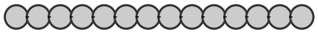

203 P08571, CD14, Monocyte differentiation antigen CD14

204 (1) Q86VB7-1-3, CD163, Scavenger receptor cysteine-rich type 1 protein M130 — 1/0/0

(2) Q86VB7-4, CD163, Scavenger receptor cysteine-rich type 1 protein M130

205 Q9NR16-1-4, CD163L1, Scavenger receptor cysteine-rich type 1 protein M160 — 2/0/0

206 Q9HCU0-1, CD248, Endosialin — 8/0/0

207 O43866, CD5L, CD5 antigen-like — 17/0/0

208 P08962-1-3, CD63, CD63 antigen — 2/0/0

209 P48960-1-3, CD97, CD97 antigen — 4/0/0

210 P14209-1,3, CD99, CD99 antigen — 2/0/0

211 Q16543, CDC37, Hsp90 co-chaperone Cdc37 — 4/0/0

212 (1) P60953-2, CDC42, Cell division control protein 42 homolog — 1/0/1

(2) P60953-1, CDC42, Cell division control protein 42 homolog

213 P12830-1,2, CDH1, Cadherin-1 — 3/0/0

214 (1) P19022-1, CDH2, Cadherin-2 — 1/0/0

(2) P19022-2, CDH2, Cadherin-2

215 A8MW92-1,2,4, PHF20L1, PHD finger protein 20-like protein 1 — 1/0/0

Q9NYV4-1-3, CDK12, Cyclin-dependent kinase 12

216 O95319-1-5, CELF2, CUGBP Elav-like family member 2 — 2/0/0

Q92879-1-6, CELF1, CUGBP Elav-like family member 1

217 (1) P11597-1, CETP, Cholesteryl ester transfer protein — 2/0/0

(2) P11597-2, CETP, Cholesteryl ester transfer protein

218 (1) P00751-1, CFB, Complement factor B — 12/0/0

(2) P00751-2, CFB, Complement factor B

219 P00746, CFD, Complement factor D — 15/0/0

220 (1) P08603-1, CFH, Complement factor H — 29/0/9

(2) P08603-2, CFH, Complement factor H

221 Q03591, CFHR1, Complement factor H-related protein 1 — 1/0/11

222 (1) P36980-1, CFHR2, Complement factor H-related protein 2 — 1/0/4

(2) P36980-2, CFHR2, Complement factor H-related protein 2

223 Q02985-1,2, CFHR3, Complement factor H-related protein 3 — 3/0/7

224 (1) Q92496-1,2, CFHR4, Complement factor H-related protein 4 — 3/0/6

(2) Q92496-3, CFHR4, Complement factor H-related protein 4

225		Q9BXR6, CFHR5, Complement factor H-related protein 5	20/0/2
226		P05156, CFI, Complement factor I	20/0/0
227		P23528, CFL1, Cofilin-1	6/0/0
228		P27918, CFP, Properdin	13/0/1
229		Q99674-1,4,5, CGREF1, Cell growth regulator with EF hand domain protein 1	8/0/0
230		Q12873-1-3, CHD3, Chromodomain-helicase-DNA-binding protein 3	3/0/0
231		P10645, CHGA, Chromogranin-A	26/0/0
232		P05060, CHGB, Secretogranin-1	23/0/1
233		Q9BY43-1,2, CHMP4A, Charged multivesicular body protein 4a	1/0/1
234		Q9H444, CHMP4B, Charged multivesicular body protein 4b	3/0/0
235	(1)	Q9H2X0-1,5, CHRD, Chordin	3/0/0
	(2)	Q9H2X0-4, CHRD, Chordin	
236	(1)	Q9BU40-2,4, CHRDL1, Chordin-like protein 1	1/15/0
	(2)	Q9BU40-1,6, CHRDL1, Chordin-like protein 1	
	(3)	Q9BU40-3, CHRDL1, Chordin-like protein 1	
	(4)	Q9BU40-5, CHRDL1, Chordin-like protein 1	
237		Q6WN34-1, CHRDL2, Chordin-like protein 2	2/0/6
238		Q6WN34-2, CHRDL2, Chordin-like protein 2	2/0/6
239		Q9NRB3, CHST12, Carbohydrate sulfotransferase 12	1/0/0
240		Q8NCH0, CHST14, Carbohydrate sulfotransferase 14	5/0/0
241		O75339, CILP, Cartilage intermediate layer protein 1	48/0/1
242		Q8IUL8, CILP2, Cartilage intermediate layer protein 2	5/0/1
243		Q07065, CKAP4, Cytoskeleton-associated protein 4	5/0/1
244		P12277, CKB, Creatine kinase B-type	6/0/0
245		Q05315, CLC, Galectin-10	3/0/0
246		Q9Y240, CLEC11A, C-type lectin domain family 11 member A	15/0/0
247		P05452, CLEC3B, Tetranectin	10/0/0
248		O00299, CLIC1, Chloride intracellular channel protein 1	6/0/0
249		Q9Y696, CLIC4, Chloride intracellular channel protein 4	5/0/0

250 (1) O94985-1, CLSTN1, Calsyntenin-1 — 1/0/0
(2) O94985-2, CLSTN1, Calsyntenin-1

251 P09496-1-4, CLTA, Clathrin light chain A — 2/0/0

252 P09497-1,2, CLTB, Clathrin light chain B — 2/0/0

253 (1) Q00610-1,2, CLTC, Clathrin heavy chain 1 — 14/0/4
(2) P53675-2, CLTCL1, Clathrin heavy chain 2

254 P53675-1, CLTCL1, Clathrin heavy chain 2 — 1/0/4

255 (1) P10909-4, CLU, Clusterin — 1/16/0
(2) P10909-3, CLU, Clusterin
(3) P10909-1,2,5, CLU, Clusterin

256 (1) P62633-1-3,7, CNBP, Cellular nucleic acid-binding protein — 1/0/3
(2) P62633-4,8, CNBP, Cellular nucleic acid-binding protein

257 P62633-5,6, CNBP, Cellular nucleic acid-binding protein — 1/0/3

258 Q96KN2, CNDP1, Beta-Ala-His dipeptidase — 3/0/0

259 (1) Q15417-1,3, CNN3, Calponin-3 — 2/0/0
(2) Q15417-2, CNN3, Calponin-3

260 Q9Y2B0-1, CNPY2, Protein canopy homolog 2 — 3/0/0

261 (1) Q99715-1, COL12A1, Collagen alpha-1(XII) chain — 0/16/1
(2) Q99715-2, COL12A1, Collagen alpha-1(XII) chain
(3) Q99715-4, COL12A1, Collagen alpha-1(XII) chain

262 (1) Q05707-1,2, COL14A1, Collagen alpha-1(XIV) chain — 3/0/0
(2) Q05707-3, COL14A1, Collagen alpha-1(XIV) chain

263 P39059, COL15A1, Collagen alpha-1(XV) chain [Cleaved into: Restin — 2/0/0

264 Q07092-1,2, COL16A1, Collagen alpha-1(XVI) chain — 5/0/0

265 P39060-1-3, COL18A1, Collagen alpha-1(XVIII) chain [Cleaved into: Endostatin] — 8/0/0

266 P02452, COL1A1, Collagen alpha-1(I) chain — 17/0/1

267 P08123, COL1A2, Collagen alpha-2(I) chain — 15/0/0

268 (1) P02461-1, COL3A1, Collagen alpha-1(III) chain — 4/0/3
(2) P02461-2, COL3A1, Collagen alpha-1(III) chain

269 P02462-1,2, COL4A1, Collagen alpha-1(IV) chain [Cleaved into: Arresten] — 1/0/0

270		P08572, COL4A2, Collagen alpha-2(IV) chain [Cleaved into: Canstatin]	2/0/0
271		P20908, COL5A1, Collagen alpha-1(V) chain	9/0/0
272		P05997, COL5A2, Collagen alpha-2(V) chain	13/0/2
273		P12109, COL6A1, Collagen alpha-1(VI) chain	22/0/0
274	(1)	P12110-1, COL6A2, Collagen alpha-2(VI) chain	6/0/1
	(2)	P12110-2,3, COL6A2, Collagen alpha-2(VI) chain	
275	(1)	P12111-1, COL6A3, Collagen alpha-3(VI) chain	3/9/86
	(2)	P12111-2, COL6A3, Collagen alpha-3(VI) chain	
	(3)	P12111-3, COL6A3, Collagen alpha-3(VI) chain	
	(4)	P12111-5, COL6A3, Collagen alpha-3(VI) chain	
276		P12111-4, COL6A3, Collagen alpha-3(VI) chain	2/0/86
277		P27658, COL8A1, Collagen alpha-1(VIII) chain	2/0/1
278		Q9Y6Z7, COLEC10, Collectin-10	3/0/0
279		Q9BWP8-1-10, COLEC11, Collectin-11	2/0/0
280		Q8NBJ5, COLGALT1, Procollagen galactosyltransferase 1	3/0/0
281	(1)	P49747-1, COMP, Cartilage oligomeric matrix protein	1/0/2
	(2)	P49747-2, COMP, Cartilage oligomeric matrix protein	
282		P53621-1,2, COPA, Coatomer subunit alpha	6/0/0
283		P53618, COPB1, Coatomer subunit beta	9/0/0
284		Q9Y678, COPG1, Coatomer subunit gamma-1	6/0/0
285		P31146, CORO1A, Coronin-1A	12/0/0
286		Q9BR76, CORO1B, Coronin-1B	3/0/0
287		Q9ULV4-1-3, CORO1C, Coronin-1C	4/0/0
288		Q14019, COTL1, Coactosin-like protein	3/0/0
289		P00450, CP, Ceruloplasmin	29/0/0
290	(1)	Q96IY4-1, CPB2, Carboxypeptidase B2	3/0/0
	(2)	Q96IY4-2, CPB2, Carboxypeptidase B2	
291		P16870-1,2, CPE, Carboxypeptidase E	19/0/0
292		P15169, CPN1, Carboxypeptidase N catalytic chain	11/0/0
293		P22792, CPN2, Carboxypeptidase N subunit 2	12/0/0
294		Q9Y646, CPQ, Carboxypeptidase Q	3/0/0

295		Q9H3G5, CPVL, Probable serine carboxypeptidase CPVL	3/0/0
296		Q96SM3, CPXM1, Probable carboxypeptidase X1	9/0/0
297		Q8N436, CPXM2, Inactive carboxypeptidase-like protein X2	13/0/0
298		Q66K79-1-3, CPZ, Carboxypeptidase Z	6/0/0
299		P29373, CRABP2, Cellular retinoic acid-binding protein 2	4/0/0
300		P24387, CRHBP, Corticotropin-releasing factor-binding protein	2/0/0
301		P50238, CRIP1, Cysteine-rich protein 1	1/0/1
302		P52943-1,2, CRIP2, Cysteine-rich protein 2	1/0/1
303		Q9H0B8-1,2, CRISPLD2, Cysteine-rich secretory protein LCCL domain-containing 2	2/0/0
304	(1)	P02741-1, CRP, C-reactive protein [Cleaved into: C-reactive protein(1-205)]	3/0/0
	(2)	P02741-2, CRP, C-reactive protein [Cleaved into: C-reactive protein(1-205)]	
305		Q9NQ79-1-3, CRTAC1, Cartilage acidic protein 1	4/0/0
306		O75718, CRTAP, Cartilage-associated protein	3/0/0
307		P02511, CRYAB, Alpha-crystallin B chain	3/0/0
308		Q9Y2S2-1,2, CRYL1, Lambda-crystallin homolog	1/0/0
309	(1)	P55060-1,3, CSE1L, Exportin-2	1/0/0
	(2)	P55060-4, CSE1L, Exportin-2	
310		Q6UVK1, CSPG4, Chondroitin sulfate proteoglycan 4	5/0/0
311		P01034, CST3, Cystatin-C	6/0/0
312		Q15828, CST6, Cystatin-M	5/0/0
313		P04080, CSTB, Cystatin-B	2/0/0
314		P29279-1,2, CTGF, Connective tissue growth factor	9/0/0
315		Q96CG8-1-3, CTHRC1, Collagen triple helix repeat-containing protein 1	2/0/0
316	(1)	P35221-1,2, CTNNA1, Catenin alpha-1	0/2/0
	(2)	P26232-1-3,5, CTNNA2, Catenin alpha-2	
	(3)	P35221-3, CTNNA1, Catenin alpha-1	
	(4)	P26232-4,6, CTNNA2, Catenin alpha-2	
317		P07858, CTSB, Cathepsin B	5/0/0
318		P07339, CTSD, Cathepsin D	5/0/0
319		Q9UBX1, CTSF, Cathepsin F	7/0/0

320		P09668, CTSH, Pro-cathepsin H [Cleaved into: Cathepsin H mini chain; Cathepsin H	2/0/0
321		P43235, CTSK, Cathepsin K	3/0/0
322		P25774-1,2, CTSS, Cathepsin S	1/0/0
323		Q9UBR2, CTSZ, Cathepsin Z	3/0/0
324		P02778, CXCL10, C-X-C motif chemokine 10	2/0/0
325		P48061-1-6, CXCL12, Stromal cell-derived factor 1	5/0/0
326		O43169, CYB5B, Cytochrome b5 type B	1/0/0
327	(1)	Q7L576-1, CYFIP1, Cytoplasmic FMR1-interacting protein 1	1/0/0
	(2)	Q7L576-2,3, CYFIP1, Cytoplasmic FMR1-interacting protein 1	
328		Q9NRR1, CYTL1, Cytokine-like protein 1	3/0/0
329		Q14118, DAG1, Dystroglycan	5/0/0
330	(1)	P14868-1, DARS, Aspartate--tRNA ligase, cytoplasmic	1/0/0
	(2)	P14868-2, DARS, Aspartate--tRNA ligase, cytoplasmic	
331	(1)	Q9UJU6-1,5, DBNL, Drebrin-like protein	1/0/0
	(2)	Q9UJU6-2-4, DBNL, Drebrin-like protein	
332	(1)	P81605-1, DCD, Dermcidin	1/0/0
	(2)	P81605-2, DCD, Dermcidin	
333	(1)	P07585-1, DCN, Decorin	4/2/1
	(2)	P07585-4, DCN, Decorin	
	(3)	P07585-5, DCN, Decorin	
	(4)	P07585-2,3, DCN, Decorin	
334		Q14203-1,3,4,6, DCTN1, Dynactin subunit 1	2/0/1
335		O75935-1,2, DCTN3, Dynactin subunit 3	2/0/0
336	(1)	Q92499-1, DDX1, ATP-dependent RNA helicase DDX1	0/1/1
	(2)	Q92499-2, DDX1, ATP-dependent RNA helicase DDX1	
	(3)	Q92499-3, DDX1, ATP-dependent RNA helicase DDX1	
337		P17844-1,2, DDX5, Probable ATP-dependent RNA helicase DDX5	6/0/0
338		P59665, DEFA1, Neutrophil defensin 1	2/0/4
339		P59666, DEFA3, Neutrophil defensin 3	3/0/4

340		P12838, DEFA4, Neutrophil defensin 4	1/0/0
341		P35659-1, DEK, Protein DEK	2/0/0
342		P17661, DES, Desmin	7/0/7
343	(1)	O60443-1, DFNA5, Non-syndromic hearing impairment protein 5	4/0/0
	(2)	O60443-3, DFNA5, Non-syndromic hearing impairment protein 5	
344		O75912, DGKI, Diacylglycerol kinase iota	2/0/0
345		Q08211-1, DHX9, ATP-dependent RNA helicase A	4/0/0
346		O60610-1-3, DIAPH1, Protein diaphanous homolog 1	4/0/0
347		Q9UBP4, DKK3, Dickkopf-related protein 3	10/0/0
348	(1)	P09622-1, DLD, Dihydrolipoyl dehydrogenase, mitochondrial	1/0/0
	(2)	P09622-2, DLD, Dihydrolipoyl dehydrogenase, mitochondrial	
349	(1)	Q6E0U4-2,5,6,16, DMKN, Dermokine	1/0/0
	(2)	Q6E0U4-1,3,4,7, DMKN, Dermokine	
350		O60884, DNAJA2, DnaJ homolog subfamily A member 2	2/0/0
351		P25685-1, DNAJB1, DnaJ homolog subfamily B member 1	2/0/0
352		Q9UBS4, DNAJB11, DnaJ homolog subfamily B member 11	6/0/0
353		Q13217, DNAJC3, DnaJ homolog subfamily C member 3	13/0/0
354	(1)	P50570-1-5, DNM2, Dynamin-2	3/3/0
	(2)	Q9UQ16-1-5, DNM3, Dynamin-3	
	(3)	Q05193-1-3,5, DNM1, Dynamin-1	
355		Q5JSL3, DOCK11, Dedicator of cytokinesis protein 11	3/0/0
356		O60496, DOK2, Docking protein 2	1/0/0
357		P27487, DPP4, Dipeptidyl peptidase 4	2/0/0
358		Q07507, DPT, Dermatopontin	10/0/0
359		Q16555-1,2, DPYSL2, Dihydropyrimidinase-related protein 2	2/0/1
360	(1)	Q14195-2, DPYSL3, Dihydropyrimidinase-related protein 3	1/0/1
	(2)	Q14195-1, DPYSL3, Dihydropyrimidinase-related protein 3	
361		Q14574-1,2, DSC3, Desmocollin-3	3/0/0
362	(1)	P15924-1,3, DSP, Desmoplakin	1/0/0
	(2)	P15924-2, DSP, Desmoplakin	
363		P60981-1,2, DSTN, Destrin	4/0/0
364		Q9BVJ7, DUSP23, Dual specificity protein phosphatase 23	2/0/0

365		Q14204, DYNC1H1, Cytoplasmic dynein 1 heavy chain 1	17/0/0
366		Q13409-1-3,5-7, DYNC1I2, Cytoplasmic dynein 1 intermediate chain 2	1/0/0
367	(1)	Q16610-1,4, ECM1, Extracellular matrix protein 1	8/5/0
	(2)	Q16610-2, ECM1, Extracellular matrix protein 1	
	(3)	Q16610-3, ECM1, Extracellular matrix protein 1	
368		O94769-1, ECM2, Extracellular matrix protein 2	3/0/13
369		O94769-2, ECM2, Extracellular matrix protein 2	1/0/13
370	(1)	P68104-1, EEF1A1, Elongation factor 1-alpha 1	1/5/0
	·	Q5VTE0, EEF1A1P5, Putative elongation factor 1-alpha-like 3	
	(2)	P68104-2, EEF1A1, Elongation factor 1-alpha 1	
	(3)	Q05639, EEF1A2, Elongation factor 1-alpha 2	
371		P24534, EEF1B2, Elongation factor 1-beta	3/0/1
372	(1)	P29692-1-3, EEF1D, Elongation factor 1-delta	1/0/1
	(2)	P29692-4, EEF1D, Elongation factor 1-delta	
373		P26641-1,2, EEF1G, Elongation factor 1-gamma	11/0/0
374		P13639, EEF2, Elongation factor 2	22/0/0
375	(1)	Q12805-1,3,4, EFEMP1, EGF-containing fibulin-like extracellular matrix protein 1	1/0/22
	(2)	Q12805-2, EFEMP1, EGF-containing fibulin-like extracellular matrix protein 1	
376		Q12805-5, EFEMP1, EGF-containing fibulin-like extracellular matrix protein 1	2/0/22
377		O95967, EFEMP2, EGF-containing fibulin-like extracellular matrix protein 2	17/0/0
378	(1)	Q9H4M9, EHD1, EH domain-containing protein 1	2/0/1
	(2)	Q9NZN3-1,2, EHD3, EH domain-containing protein 3	
379	(1)	Q9NZN4-1, EHD2, EH domain-containing protein 2	0/0/1
	(2)	Q9NZN4-2, EHD2, EH domain-containing protein 2	
380		Q9H223, EHD4, EH domain-containing protein 4	1/0/0
381		P05198, EIF2S1, Eukaryotic translation initiation factor 2 subunit 1	3/0/0
382		Q14152-1,2, EIF3A, Eukaryotic translation initiation factor 3 subunit A	7/0/0
383		P55884-1,2, EIF3B, Eukaryotic translation initiation factor 3 subunit B	4/0/0
384	(1)	Q99613-1,2, EIF3C, Eukaryotic translation initiation factor 3 subunit C	1/0/0

	(2)	B5ME19, EIF3CL, Eukaryotic translation initiation factor 3 subunit C-like protein	
385		Q9Y262-1,2, EIF3L, Eukaryotic translation initiation factor 3 subunit L	2/0/0
386		P60842-1,2, EIF4A1, Eukaryotic initiation factor 4A-I	3/0/2
387		Q14240-1,2, EIF4A2, Eukaryotic initiation factor 4A-II	1/0/2
388		P38919, EIF4A3, Eukaryotic initiation factor 4A-III	1/0/0
389		Q04637-1,3-9, EIF4G1, Eukaryotic translation initiation factor 4 gamma 1	6/0/0
390		P78344-1,2, EIF4G2, Eukaryotic translation initiation factor 4 gamma 2	4/0/0
391		Q15056-1,2, EIF4H, Eukaryotic translation initiation factor 4H	3/0/0
392		P63241-1,2, EIF5A, Eukaryotic translation initiation factor 5A-1	3/0/0
393		P08246, ELANE, Neutrophil elastase	2/0/0
394		O00472-1,2, ELL2, RNA polymerase II elongation factor ELL2	1/0/0
395		P15502-1,2,4,5, ELN, Elastin	1/0/0
396	(1)	Q9Y6C2-1, EMILIN1, EMILIN-1	8/0/0
	(2)	Q9Y6C2-2, EMILIN1, EMILIN-1	
397		Q9BXX0, EMILIN2, EMILIN-2	12/0/0
398	(1)	P06733-1, ENO1, Alpha-enolase	3/4/0
	(2)	P13929-1-3, ENO3, Beta-enolase	
	(3)	P09104-1,2, ENO2, Gamma-enolase	
	(4)	P06733-2, ENO1, Alpha-enolase	
399		O43491-1,3,4, EPB41L2, Band 4.1-like protein 2	2/0/0
400		P07099, EPHX1, Epoxide hydrolase 1	2/0/0
401		P58107, EPPK1, Epiplakin	4/0/3
402		P11678, EPX, Eosinophil peroxidase	4/0/2
403		P84090, ERH, Enhancer of rudimentary homolog	2/0/0
404		Q9BS26, ERP44, Endoplasmic reticulum resident protein 44	7/0/0
405		P10768, ESD, S-formylglutathione hydrolase	2/0/0
406	(1)	P62495-1, ETF1, Eukaryotic peptide chain release factor subunit 1	2/0/0
	(2)	P62495-2, ETF1, Eukaryotic peptide chain release factor subunit 1	
407		Q01844-1-3,5,6, EWSR1, RNA-binding protein EWS	2/0/0

408		P15311, EZR, Ezrin	9/0/1
409		P00742, F10, Coagulation factor X	12/0/0
410	(1)	P03951-1, F11, Coagulation factor XI	1/0/0
	(2)	P03951-2, F11, Coagulation factor XI	
411		P00748, F12, Coagulation factor XII	18/0/0
412		P00488, F13A1, Coagulation factor XIII A chain	23/0/0
413		P05160, F13B, Coagulation factor XIII B chain	7/0/0
414		P00734, F2, Prothrombin	39/0/0
415		P12259, F5, Coagulation factor V	20/0/0
416		P08709-1,2, F7, Coagulation factor VII	2/0/0
417	(1)	P00740-1, F9, Coagulation factor IX	1/0/0
	(2)	P00740-2, F9, Coagulation factor IX	
418		P05413, FABP3, Fatty acid-binding protein, heart	2/0/0
419	(1)	Q01469, FABP5, Fatty acid-binding protein, epidermal	1/0/0
	(2)	A8MUU1, FABP5P3, Putative fatty acid-binding protein 5-like protein 3	
420		Q8IWE2-1,2, FAM114A1, Protein NOXP20	3/0/0
421		Q9BZQ8, FAM129A, Protein Niban	2/0/0
422		Q6UWF9, FAM180A, Protein FAM180A	3/0/0
423		Q9ULE4, FAM184B, Protein FAM184B	3/0/1
424		Q96MK3, FAM20A, Pseudokinase FAM20A	3/0/0
425		Q9NUQ9-1, FAM49B, Protein FAM49B	3/0/0
426	(1)	P23142-1, FBLN1, Fibulin-1	9/0/22
	(2)	P23142-2, FBLN1, Fibulin-1	
427		P23142-3, FBLN1, Fibulin-1	1/0/22
428		P23142-4, FBLN1, Fibulin-1	5/0/22
429		P98095-1,2, FBLN2, Fibulin-2	24/0/0
430		Q9UBX5, FBLN5, Fibulin-5	14/0/0
431	(1)	Q53RD9-1-3, FBLN7, Fibulin-7	1/0/0
	(2)	Q53RD9-4, FBLN7, Fibulin-7	
432		P35555, FBN1, Fibrillin-1 [Cleaved into: Asprosin]	78/0/6
433	(1)	P35556-1, FBN2, Fibrillin-2 [Cleaved into: Fibrillin-2 C-terminal peptide]	6/0/7

	(2)	P35556-2, FBN2, Fibrillin-2 [Cleaved into: Fibrillin-2 C-terminal peptide]	
434		Q9UK22, FBXO2, F-box only protein 2	2/0/0
435		Q9Y6R7, FCGBP, IgGFc-binding protein	17/0/0
436		O00602, FCN1, Ficolin-1	5/0/1
437		Q15485-1,2, FCN2, Ficolin-2	2/0/1
438		O75636-1, FCN3, Ficolin-3	1/0/9
439		O75636-2, FCN3, Ficolin-3	0/0/10
440		Q86UX7-1,2, FERMT3, Fermitin family homolog 3	4/0/0
441	(1)	Q9UGM5-1, FETUB, Fetuin-B	1/0/0
	(2)	Q9UGM5-2, FETUB, Fetuin-B	
442	(1)	P02671-1, FGA, Fibrinogen alpha chain [Cleaved into: Fibrinopeptide A; Fibrinogen alpha chain]	4/0/0
	(2)	P02671-2, FGA, Fibrinogen alpha chain [Cleaved into: Fibrinopeptide A; Fibrinogen alpha chain]	
443		P02675, FGB, Fibrinogen beta chain [Cleaved into: Fibrinopeptide B; Fibrinogen beta chain]	46/0/0
444		Q6ZV73-1,2, FGD6, FYVE, RhoGEF and PH domain-containing protein 6	2/0/0
445	(1)	P02679-1, FGG, Fibrinogen gamma chain	1/0/0
	(2)	P02679-2, FGG, Fibrinogen gamma chain	
446		Q8TAL6, FIBIN, Fin bud initiation factor homolog	2/0/0
447		P62942, FKBP1A, Peptidyl-prolyl cis-trans isomerase FKBP1A	1/0/0
448		O95302-1,3, FKBP9, Peptidyl-prolyl cis-trans isomerase FKBP9	3/0/0
449		P20930, FLG, Filaggrin	3/0/0
450		Q13045-1-3, FLII, Protein flightless-1 homolog	2/0/0
451		P21333-1,2, FLNA, Filamin-A	49/0/12
452	(1)	O75369-1-3,6,8,9, FLNB, Filamin-B	0/4/7
	(2)	O75369-7, FLNB, Filamin-B	
	(3)	O75369-4,5, FLNB, Filamin-B	
453		Q14315-1,2, FLNC, Filamin-C	32/0/11
454		Q06828, FMOD, Fibromodulin	9/0/0

0/87/0

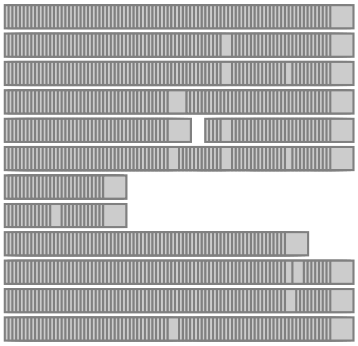

455 (1) P02751-1,3,17, FN1, Fibronectin
(2) P02751-8,9,14, FN1, Fibronectin
(3) P02751-10, FN1, Fibronectin
(4) P02751-11, FN1, Fibronectin
(5) P02751-12, FN1, Fibronectin
(6) P02751-13, FN1, Fibronectin
(7) P02751-16, FN1, Fibronectin
(8) P02751-2, FN1, Fibronectin
(9) P02751-4, FN1, Fibronectin
(10) P02751-5, FN1, Fibronectin
(11) P02751-6, FN1, Fibronectin
(12) P02751-7,15, FN1, Fibronectin

456 Q4ZHG4-1,2, FNDC1, Fibronectin type III domain-containing protein 1

11/0/0 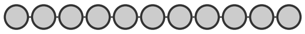

2/4/0 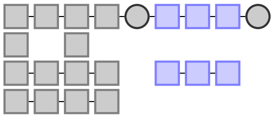

457 (1) Q86XX4-1,2, FRAS1, Extracellular matrix protein FRAS1
(2) Q86XX4-4, FRAS1, Extracellular matrix protein FRAS1
(3) Q86XX4-5, FRAS1, Extracellular matrix protein FRAS1
(4) Q86XX4-6, FRAS1, Extracellular matrix protein FRAS1

458 Q92765, FRZB, Secreted frizzled-related protein 3

1/0/0 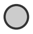

459 Q16658, FSCN1, Fascin

3/0/0 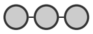

460 P19883-1,2, FST, Follistatin

11/0/0 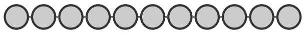

2/0/0 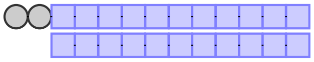

461 (1) Q12841-1, FSTL1, Follistatin-related protein 1
(2) Q12841-2, FSTL1, Follistatin-related protein 1

462 O95633-1,2, FSTL3, Follistatin-related protein 3

3/0/0 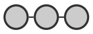

463 P02792, FTL, Ferritin light chain

5/0/0 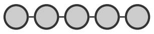

464 Q96AE4-1,2, FUBP1, Far upstream element-binding protein 1

3/0/1 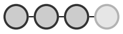

465 P35637-1,2, FUS, RNA-binding protein FUS

3/0/1 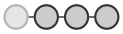

466 P51114-1-3, FXR1, Fragile X mental retardation syndrome-related protein 1

1/0/0 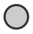

467 Q13283-1, G3BP1, Ras GTPase-activating protein-binding protein 1

2/0/0 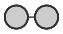

468 P11413-1-3, G6PD, Glucose-6-phosphate 1-dehydrogenase 2/0/0

469 Q96RP7-1,2, GAL3ST4, Galactose-3-O-sulfotransferase 4 1/0/0

470 P51570-1,2, GALK1, Galactokinase 2/0/0

471 Q14697-1, GANAB, Neutral alpha-glucosidase AB 1/0/18

472 Q14697-2, GANAB, Neutral alpha-glucosidase AB 1/0/18

473 Q14697-3, GANAB, Neutral alpha-glucosidase AB 1/0/1

474 (1) P04406-1, GAPDH, Glyceraldehyde-3-phosphate dehydrogenase 2/0/0

(2) P04406-2, GAPDH, Glyceraldehyde-3-phosphate dehydrogenase

475 P41250, GARS, Glycine--tRNA ligase 6/0/0

476 P54826, GAS1, Growth arrest-specific protein 1 8/0/0

477 (1) Q14393-1,2, GAS6, Growth arrest-specific protein 6 1/10/0

(2) Q14393-3, GAS6, Growth arrest-specific protein 6

(3) Q14393-4, GAS6, Growth arrest-specific protein 6

(4) Q14393-5, GAS6, Growth arrest-specific protein 6

478 (1) P04062-1,2,4,5, GBA, Glucosylceramidase 1/0/0

(2) P04062-3, GBA, Glucosylceramidase

479 (1) P02774-1,3, GC, Vitamin D-binding protein 9/0/0

(2) P02774-2, GC, Vitamin D-binding protein

480 Q92947-1,2, GCDH, Glutaryl-CoA dehydrogenase, mitochondrial 0/0/2

481 P01275, GCG, Glucagon [Cleaved into: Glicentin; Glicentin-related polypeptide 3/0/0

482 Q99988, GDF15, Growth/differentiation factor 15 5/0/0

483 Q6KF10, GDF6, Growth/differentiation factor 6 2/0/0

484 O00451-1, GFRA2, GDNF family receptor alpha-2 2/0/0

485 Q9UBU3-1-5, GHRL, Appetite-regulating hormone 1/0/0

486 Q9NUV9, GIMAP4, GTPase IMAP family member 4 3/0/0

487 P09681, GIP, Gastric inhibitory polypeptide 3/0/0

488 O14908-1,2, GIPC1, PDZ domain-containing protein GIPC1 1/0/0

489 Q9NS71, GKN1, Gastrokine-1 6/0/0

490 Q86XP6, GKN2, Gastrokine-2 1/0/0

491 Q92896-1-3, GLG1, Golgi apparatus protein 1 2/0/0

492 Q4G1C9-1,5,6, GLIPR1L2, GLIPR1-like protein 2 — 3/0/0

493 O76003, GLRX3, Glutaredoxin-3 — 2/0/0

494 P17900, GM2A, Ganglioside GM2 activator — 7/0/0

495 P04899-1-6, GNAI2, Guanine nucleotide-binding protein G(i) subunit alpha-2 — 2/0/0

496 (1) P62873-1,2, GNB1, Guanine nucleotide-binding protein G(I)/G(S)/G(T) subunit beta-1 — 1/0/2
(2) P16520-1,2, GNB3, Guanine nucleotide-binding protein G(I)/G(S)/G(T) subunit beta-3
(3) P62879-2, GNB2, Guanine nucleotide-binding protein G(I)/G(S)/G(T) subunit beta-2

497 (1) Q9HAV0, GNB4, Guanine nucleotide-binding protein subunit beta-4 — 1/0/2
(2) P62879-1, GNB2, Guanine nucleotide-binding protein G(I)/G(S)/G(T) subunit beta-2

498 Q9UJJ9, GNPTG, N-acetylglucosamine-1-phosphotransferase subunit gamma — 1/0/0

499 O00461, GOLIM4, Golgi integral membrane protein 4 — 10/0/0

500 Q8NBJ4-1,2, GOLM1, Golgi membrane protein 1 — 9/0/0

501 P80108-1, GPLD1, Phosphatidylinositol-glycan-specific phospholipase D — 9/0/0

502 Q13098-4-7, GPS1, COP9 signalosome complex subunit 1 — 2/0/0

503 P22352, GPX3, Glutathione peroxidase 3 — 8/0/0

504 (1) O60565-1, GREM1, Gremlin-1 — 1/0/0
(2) O60565-2, GREM1, Gremlin-1

505 Q9H772, GREM2, Gremlin-2 — 1/0/0

506 Q9ULK0-1, GRID1, Glutamate receptor ionotropic, delta-1 — 0/0/2

507 (1) P28799-1, GRN, Granulins — 0/4/0
(2) P28799-2, GRN, Granulins
(3) P28799-3, GRN, Granulins

508 P57764, GSDMD, Gasdermin-D — 2/0/0

509 (1) P06396-1, GSN, Gelsolin — 1/0/35
(2) P06396-3,4, GSN, Gelsolin

510 P06396-2, GSN, Gelsolin — 1/0/35

511 P21266, GSTM3, Glutathione S-transferase Mu 3 — 3/0/0

512 P09211, GSTP1, Glutathione S-transferase P — 2/0/0

513 Q02747, GUCA2A, Guanylin — 2/0/0

514		P07305-1,2, H1F0, Histone H1.0	3/0/0
515		Q92522, H1FX, Histone H1x	4/0/0
516	(1)	P0C0S5, H2AFZ, Histone H2A.Z	0/0/2
	·	Q71UI9-1,2, H2AFV, Histone H2A.V	
	(2)	Q71UI9-3,4, H2AFV, Histone H2A.V	
517		P16104, H2AFX, Histone H2AX	4/0/3
518		P84243, H3F3A, Histone H3.3	1/0/7
519		Q14520-1,2, HABP2, Hyaluronan-binding protein 2	16/0/0
520		P40939-1, HADHA, Trifunctional enzyme subunit alpha, mitochondrial	3/0/0
521	(1)	P55084-1, HADHB, Trifunctional enzyme subunit beta, mitochondrial	1/0/0
	(2)	P55084-2, HADHB, Trifunctional enzyme subunit beta, mitochondrial	
522		P81172, HAMP, Hepcidin	1/0/0
523		P69905, HBA1;, Hemoglobin subunit alpha	4/0/0
524	(1)	P68871, HBB, Hemoglobin subunit beta	4/0/0
	(2)	P02042, HBD, Hemoglobin subunit delta	
525	(1)	P08631-1-4, HCK, Tyrosine-protein kinase HCK	2/0/0
	(2)	P07948-1,2, LYN, Tyrosine-protein kinase Lyn	
526		P14317-1, HCLS1, Hematopoietic lineage cell-specific protein	3/0/0
527		P51858-1-3, HDGF, Hepatoma-derived growth factor	3/0/0
528		Q00341-1,2, HDLBP, Vigilin	7/0/0
529		O95714, HERC2, E3 ubiquitin-protein ligase HERC2	4/0/0
530		P06865-1, HEXA, Beta-hexosaminidase subunit alpha	4/0/0
531		P07686, HEXB, Beta-hexosaminidase subunit beta	5/0/0
532		P49773, HINT1, Histidine triad nucleotide-binding protein 1	2/0/0
533		P16401, HIST1H1B, Histone H1.5	8/0/5
534		P16403, HIST1H1C, Histone H1.2	7/0/9
535	(1)	P16402, HIST1H1D, Histone H1.3	7/0/8
	(2)	P22492, HIST1H1T, Histone H1t	
	(3)	Q02539, HIST1H1A, Histone H1.1	
536		P10412, HIST1H1E, Histone H1.4	3/0/10
537		P04908, HIST1H2AB, Histone H2A type 1-B/E	1/0/5
		P20671, HIST1H2AD, Histone H2A type 1-D	
		Q7L7L0, HIST3H2A, Histone H2A type 3	
538		P0C0S8, HIST1H2AG, Histone H2A type 1	0/0/6
		Q6FI13, HIST2H2AA3, Histone H2A type 2-A	

Q93077, HIST1H2AC, Histone H2A type 1-C
Q96KK5, HIST1H2AH, Histone H2A type 1-H
Q99878, HIST1H2AJ, Histone H2A type 1-J

539 (1) P06899, HIST1H2BJ, Histone H2B type 1-J
· P23527, HIST1H2BO, Histone H2B type 1-O
· P33778, HIST1H2BB, Histone H2B type 1-B — 0/0/11
· Q16778, HIST2H2BE, Histone H2B type 2-E
(2) Q6DN03, HIST2H2BC, Putative histone H2B type 2-C
· Q6DRA6, HIST2H2BD, Putative histone H2B type 2-D

540 (1) O60814, HIST1H2BK, Histone H2B type 1-K
· P58876, HIST1H2BD, Histone H2B type 1-D
· P62807, HIST1H2BC, Histone H2B type 1-C/E/F/G/I — 0/0/11
· Q5QNW6-1,2, HIST2H2BF, Histone H2B type 2-F
· Q93079, HIST1H2BH, Histone H2B type 1-H
· Q99877, HIST1H2BN, Histone H2B type 1-N
(2) P57053, H2BFS, Histone H2B type F-S

541 (1) Q99880, HIST1H2BL, Histone H2B type 1-L — 0/0/11
(2) Q96A08, HIST1H2BA, Histone H2B type 1-A

542 Q99879, HIST1H2BM, Histone H2B type 1-M — 1/0/10

543 (1) P68431, HIST1H3A, Histone H3.1
· Q16695, HIST3H3, Histone H3.1t — 1/0/7
· Q71DI3, HIST2H3A;, Histone H3.2
(2) Q6NXT2, H3F3C, Histone H3.3C

544 P62805, HIST1H4A, Histone H4 — 6/0/0

545 Q8IUE6, HIST2H2AB, Histone H2A type 2-B — 2/0/3

546 (1) Q16777, HIST2H2AC, Histone H2A type 2-C — 1/0/5
(2) Q96QV6, HIST1H2AA, Histone H2A type 1-A
· Q9BTM1-2, H2AFJ, Histone H2A.J
(3) Q9BTM1-1, H2AFJ, Histone H2A.J

547 Q8N257, HIST3H2BB, Histone H2B type 3-B — 0/0/10

548 P01891, HLA-A, HLA class I histocompatibility antigen, A-68 alpha chain — 0/0/6

549 (1) P04439, HLA-A, HLA class I histocompatibility antigen, A-3 alpha chain
· P13746-1,2, HLA-A, HLA class I histocompatibility antigen, A-11 alpha chain — 0/0/5
(2) P30443, HLA-A, HLA class I histocompatibility antigen, A-1 alpha chain
· P30455, HLA-A, HLA class I histocompatibility antigen, A-36 alpha chain

550 (1) P10316, HLA-A, HLA class I histocompatibility antigen, A-69 alpha chain — 0/0/6
(2) P01892, HLA-A, HLA class I histocompatibility antigen, A-2 alpha chain

551 P16188, HLA-A, HLA class I histocompatibility antigen, A-30 alpha chain — 0/0/6

552 (1) P16190, HLA-A, HLA class I histocompatibility antigen, A-33 alpha chain

0/0/6 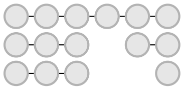

(2) P30453, HLA-A, HLA class I histocompatibility antigen, A-34 alpha chain

(3) P18462, HLA-A, HLA class I histocompatibility antigen, A-25 alpha chain

· P30450, HLA-A, HLA class I histocompatibility antigen, A-26 alpha chain

· P30457, HLA-A, HLA class I histocompatibility antigen, A-66 alpha chain

553 (1) P30447, HLA-A, HLA class I histocompatibility antigen, A-23 alpha chain

0/0/5 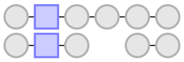

(2) P05534, HLA-A, HLA class I histocompatibility antigen, A-24 alpha chain

554 (1) P30512, HLA-A, HLA class I histocompatibility antigen, A-29 alpha chain

0/0/6 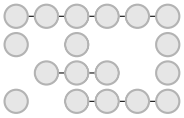

(2) P30456, HLA-A, HLA class I histocompatibility antigen, A-43 alpha chain

(3) Q09160, HLA-A, HLA class I histocompatibility antigen, A-80 alpha chain

(4) P10314, HLA-A, HLA class I histocompatibility antigen, A-32 alpha chain

· P16189, HLA-A, HLA class I histocompatibility antigen, A-31 alpha chain

· P30459, HLA-A, HLA class I histocompatibility antigen, A-74 alpha chain

555 (1) P30460, HLA-B, HLA class I histocompatibility antigen, B-8 alpha chain

· P30480, HLA-B, HLA class I histocompatibility antigen, B-42 alpha chain

(2) P03989, HLA-B, HLA class I histocompatibility antigen, B-27 alpha chain

· P18464, HLA-B, HLA class I histocompatibility antigen, B-51 alpha chain

· P30475, HLA-B, HLA class I histocompatibility antigen, B-39 alpha chain

· P30490, HLA-B, HLA class I histocompatibility antigen, B-52 alpha chain

0/0/8 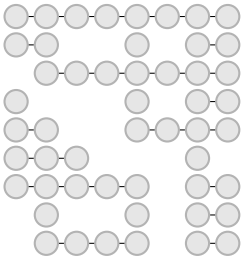

· P30491, HLA-B, HLA class I histocompatibility antigen, B-53 alpha chain

· P30498, HLA-B, HLA class I histocompatibility antigen, B-78 alpha chain

· P30685, HLA-B, HLA class I histocompatibility antigen, B-35 alpha chain

· Q29836, HLA-B, HLA class I histocompatibility antigen, B-67 alpha chain

· Q95365, HLA-B, HLA class I histocompatibility antigen, B-38 alpha chain

(3) P30479, HLA-B, HLA class I histocompatibility antigen, B-41 alpha chain

(4) P10319, HLA-B, HLA class I histocompatibility antigen, B-58 alpha chain

· P18465, HLA-B, HLA class I histocompatibility antigen, B-57 alpha chain

(5) P18463, HLA-B, HLA class I histocompatibility antigen, B-37 alpha chain

· P30462, HLA-B, HLA class I histocompatibility antigen, B-14 alpha chain

· P30466, HLA-B, HLA class I histocompatibility antigen, B-18 alpha chain

(6) P30486, HLA-B, HLA class I histocompatibility antigen, B-48 alpha chain

· Q31610, HLA-B, HLA class I histocompatibility antigen, B-81 alpha chain

(7) P01889, HLA-B, HLA class I histocompatibility antigen, B-7 alpha chain

(8) P30481, HLA-B, HLA class I histocompatibility antigen, B-44 alpha chain

· P30483, HLA-B, HLA class I histocompatibility antigen, B-45 alpha chain

· P30485, HLA-B, HLA class I histocompatibility antigen, B-47 alpha chain

· P30487, HLA-B, HLA class I histocompatibility antigen, B-49 alpha chain

· P30488, HLA-B, HLA class I histocompatibility antigen, B-50 alpha chain

(9) Q04826, HLA-B, HLA class I histocompatibility antigen, B-40 alpha chain

556 P30464, HLA-B, HLA class I histocompatibility antigen, B-15 alpha chain — 0/0/6 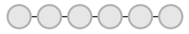

P30484, HLA-B, HLA class I histocompatibility antigen, B-46 alpha chain

557 P30492, HLA-B, HLA class I histocompatibility antigen, B-54 alpha chain — 0/0/7 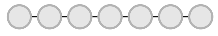

558 (1) P30493, HLA-B, HLA class I histocompatibility antigen, B-55 alpha chain — 0/0/7 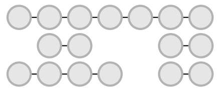

· Q29940, HLA-B, HLA class I histocompatibility antigen, B-59 alpha chain

(2) P30461, HLA-B, HLA class I histocompatibility antigen, B-13 alpha chain

(3) P30495, HLA-B, HLA class I histocompatibility antigen, B-56 alpha chain

· Q29718, HLA-B, HLA class I histocompatibility antigen, B-82 alpha chain

559 Q31612, HLA-B, HLA class I histocompatibility antigen, B-73 alpha chain — 0/0/7 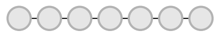

560 (1) P10321, HLA-C, HLA class I histocompatibility antigen, Cw-7 alpha chain — 0/0/7 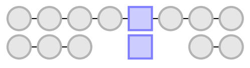

(2) Q95604, HLA-C, HLA class I histocompatibility antigen, Cw-17 alpha chain

561 (1) P30508, HLA-C, HLA class I histocompatibility antigen, Cw-12 alpha chain

· Q29963, HLA-C, HLA class I histocompatibility antigen, Cw-6 alpha chain — 0/0/9 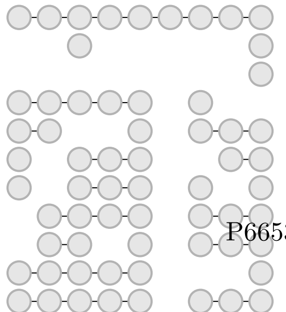

(2) P17693, HLA-G, HLA class I histocompatibility antigen, alpha chain G

(3) P13747, HLA-E, HLA class I histocompatibility antigen, alpha chain E

(4) P30499, HLA-C, HLA class I histocompatibility antigen, Cw-1 alpha chain

(5) Q29960-1,2, HLA-C, HLA class I histocompatibility antigen, Cw-16 alpha chain

(6) P30501, HLA-C, HLA class I histocompatibility antigen, Cw-2 alpha chain

(7) P30504, HLA-C, HLA class I histocompatibility antigen, Cw-4 alpha chain

(8) P30505, HLA-C, HLA class I histocompatibility antigen, Cw-8 alpha chain

(9) Q9TNN7, HLA-C, HLA class I histocompatibility antigen, Cw-5 alpha chain

(10) P04222, HLA-C, HLA class I histocompatibility antigen, Cw-3 alpha chain

(11) Q07000, HLA-C, HLA class I histocompatibility antigen, Cw-15 alpha chain

(12) P30510, HLA-C, HLA class I histocompatibility antigen, Cw-14 alpha chain

· Q29865, HLA-C, HLA class I histocompatibility antigen, Cw-18 alpha chain

(13) P01893, HLA-H, Putative HLA class I histocompatibility antigen, alpha chain H

562 P01911, HLA-DRB1, HLA class II histocompatibility antigen, DRB1-15 beta chain

P01912, HLA-DRB1, HLA class II histocompatibility antigen, DRB1-3 chain

P04229, HLA-DRB1, HLA class II histocompatibility antigen, DRB1-1 beta chain

2/0/0 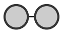

P13762, HLA-DRB4, HLA class II histocompatibility antigen, DR beta 4 chain

P20039, HLA-DRB1, HLA class II histocompatibility antigen, DRB1-11 beta chain

Q29974, HLA-DRB1, HLA class II histocompatibility antigen, DRB1-16 beta chain

Q30134, HLA-DRB1, HLA class II histocompatibility antigen, DRB1-8 beta chain

Q30167, HLA-DRB1, HLA class II histocompatibility antigen, DRB1-10 beta chain

Q5Y7A7, HLA-DRB1, HLA class II histocompatibility antigen, DRB1-13 beta chain

Q95IE3, HLA-DRB1, HLA class II histocompatibility antigen, DRB1-12 beta chain

Q9GIY3, HLA-DRB1, HLA class II histocompatibility antigen, DRB1-14 beta chain

563 Q96RW7-1,2, HMCN1, Hemicentin-1

23/0/1 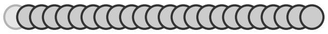

1/14/1 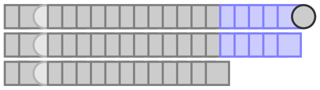

564 (1) Q8NDA2-1, HMCN2, Hemicentin-2

(2) Q8NDA2-2, HMCN2, Hemicentin-2

(3) Q8NDA2-4, HMCN2, Hemicentin-2

565 P05114, HMGN1, Non-histone chromosomal protein HMG-14 — 2/0/0

566 P05204, HMGN2, Non-histone chromosomal protein HMG-17 — 4/0/0

567 O00479, HMGN4, High mobility group nucleosome-binding domain-containing protein 4 — 3/0/0

568 Q92619-1,2, HMHA1, Minor histocompatibility protein HA-1 [Cleaved into: Minor histocompatibility antigen HA-1 — 3/0/0

569 P50135-1, HNMT, Histamine N-methyltransferase — 3/0/0

570 Q13151, HNRNPA0, Heterogeneous nuclear ribonucleoprotein A0 — 1/0/1

571 P09651-1-3, HNRNPA1, Heterogeneous nuclear ribonucleoprotein A1 — 2/0/2

Q32P51, HNRNPA1L2, Heterogeneous nuclear ribonucleoprotein A1-like 2

1/0/2

572 (1) P22626-1, HNRNPA2B1, Heterogeneous nuclear ribonucleoproteins A2/B1

(2) P22626-2, HNRNPA2B1, Heterogeneous nuclear ribonucleoproteins A2/B1

1/0/1

573 (1) P51991-1, HNRNPA3, Heterogeneous nuclear ribonucleoprotein A3

(2) P51991-2, HNRNPA3, Heterogeneous nuclear ribonucleoprotein A3

574 Q99729-1-4, HNRNPAB, Heterogeneous nuclear ribonucleoprotein A/B — 3/0/1

0/5/4

575 (1) P07910-1,2, HNRNPC, Heterogeneous nuclear ribonucleoproteins C1/C2

(2) P07910-3, HNRNPC, Heterogeneous nuclear ribonucleoproteins C1/C2

(3) P07910-4, HNRNPC, Heterogeneous nuclear ribonucleoproteins C1/C2

576 (1) B2RXH8, HNRNPCL2, Heterogeneous nuclear ribonucleoprotein C-like 2 — 1/0/4

· O60812, HNRNPCL1, Heterogeneous nuclear ribonucleoprotein C-like 1

(2) B7ZW38, HNRNPCL3, Heterogeneous nuclear ribonucleoprotein C-like 3

· P0DMR1, HNRNPCL4, Heterogeneous nuclear ribonucleoprotein C-like 4

577 Q14103-1,3, HNRNPD, Heterogeneous nuclear ribonucleoprotein D0 — 2/0/3

578 Q14103-2,4, HNRNPD, Heterogeneous nuclear ribonucleoprotein D0 — 1/0/3

579 O14979-1-3, HNRNPDL, Heterogeneous nuclear ribonucleoprotein D-like — 1/0/1

580 P52597, HNRNPF, Heterogeneous nuclear ribonucleoprotein F — 2/0/2

581 (1) P31943, HNRNPH1, Heterogeneous nuclear ribonucleoprotein H — 1/0/2
(2) P55795, HNRNPH2, Heterogeneous nuclear ribonucleoprotein H2

582 (1) P31942-1-3, HNRNPH3, Heterogeneous nuclear ribonucleoprotein H3 — 1/1/0
(2) P31942-4, HNRNPH3, Heterogeneous nuclear ribonucleoprotein H3
(3) P31942-5,6, HNRNPH3, Heterogeneous nuclear ribonucleoprotein H3

583 P61978-1-3, HNRNPK, Heterogeneous nuclear ribonucleoprotein K — 13/0/0

584 (1) P14866-1, HNRNPL, Heterogeneous nuclear ribonucleoprotein L — 4/0/0
(2) P14866-2, HNRNPL, Heterogeneous nuclear ribonucleoprotein L

585 P52272-1,2, HNRNPM, Heterogeneous nuclear ribonucleoprotein M — 9/0/0

586 Q00839-1, HNRNPU, Heterogeneous nuclear ribonucleoprotein U — 2/0/11

587 Q00839-2, HNRNPU, Heterogeneous nuclear ribonucleoprotein U — 1/0/11

588 Q1KMD3, HNRNPUL2, Heterogeneous nuclear ribonucleoprotein U-like protein 2 — 11/0/0

589 (1) P00738-1, HP, Haptoglobin — 0/0/11
(2) P00738-2, HP, Haptoglobin

590 Q5SSJ5-1,2, HP1BP3, Heterochromatin protein 1-binding protein 3 — 3/0/0

591 P00739-1,2, HPR, Haptoglobin-related protein — 7/0/11

592 P02790, HPX, Hemopexin — 12/0/0

593 P23327, HRC, Sarcoplasmic reticulum histidine-rich calcium-binding protein — 4/0/0

594 P04196, HRG, Histidine-rich glycoprotein — 20/0/0

595 Q86YZ3, HRNR, Hornerin — 4/0/0

596 (1) P07900-1,2, HSP90AA1, Heat shock protein HSP 90-alpha — 10/0/14
(2) Q12931-1,2, TRAP1, Heat shock protein 75 kDa, mitochondrial
(3) Q58FG1, HSP90AA4P, Putative heat shock protein HSP 90-alpha A4
(4) Q14568, HSP90AA2P, Heat shock protein HSP 90-alpha A2

597		Q58FG0, HSP90AA5P, Putative heat shock protein HSP 90-alpha A5	1/0/3
598	(1)	P08238, HSP90AB1, Heat shock protein HSP 90-beta	7/0/19
	(2)	Q58FF8, HSP90AB2P, Putative heat shock protein HSP 90-beta 2	
599		Q58FF7, HSP90AB3P, Putative heat shock protein HSP 90-beta-3	1/0/11
600		Q58FF6, HSP90AB4P, Putative heat shock protein HSP 90-beta 4	1/0/4
601	(1)	P14625, HSP90B1, Endoplasmin	23/0/1
	(2)	Q58FF3, HSP90B2P, Putative endoplasmin-like protein	
602	(1)	P0DMV8-1,2, HSPA1A, Heat shock 70 kDa protein 1A	3/0/12
	·	P0DMV9, HSPA1B, Heat shock 70 kDa protein 1B	
	(2)	P48741, HSPA7, Putative heat shock 70 kDa protein 7	
603		P34931, HSPA1L, Heat shock 70 kDa protein 1-like	0/0/10
604		P54652, HSPA2, Heat shock-related 70 kDa protein 2	3/0/7
605		P11021, HSPA5, 78 kDa glucose-regulated protein	23/0/3
606		P17066, HSPA6, Heat shock 70 kDa protein 6	1/0/5
607	(1)	P11142-1, HSPA8, Heat shock cognate 71 kDa protein	4/0/8
	(2)	P11142-2, HSPA8, Heat shock cognate 71 kDa protein	
608		P38646, HSPA9, Stress-70 protein, mitochondrial	5/0/0
609		P04792, HSPB1, Heat shock protein beta-1	7/0/0
610		O14558, HSPB6, Heat shock protein beta-6	2/0/0
611		P10809-1, HSPD1, 60 kDa heat shock protein, mitochondrial	5/0/0
612		P61604, HSPE1, 10 kDa heat shock protein, mitochondrial	1/0/0
613		P98160, HSPG2, Basement membrane-specific heparan sulfate proteoglycan core protein	53/0/0
614		O43719, HTATSF1, HIV Tat-specific factor 1	11/0/0
615		Q92743, HTRA1, Serine protease HTRA1	14/0/0
616		P83110-1,2, HTRA3, Serine protease HTRA3	3/0/0
617		Q7Z6Z7-1-3, HUWE1, E3 ubiquitin-protein ligase HUWE1	4/0/0
618		Q4G0P3-1, HYDIN, Hydrocephalus-inducing protein homolog	6/0/0
619	(1)	Q9Y4L1-1, HYOU1, Hypoxia up-regulated protein 1	5/0/0
	(2)	Q9Y4L1-2, HYOU1, Hypoxia up-regulated protein 1	
620		P13284, IFI30, Gamma-interferon-inducible lysosomal thiol reductase	6/0/0

621	(1)	P05019-1, IGF1, Insulin-like growth factor I	1/4/0
	(2)	P05019-2,3, IGF1, Insulin-like growth factor I	
	(3)	P05019-4, IGF1, Insulin-like growth factor I	
622		P01344-1-3, IGF2, Insulin-like growth factor II	4/0/0
623		P11717, IGF2R, Cation-independent mannose-6-phosphate receptor	5/0/0
624		P35858-1,2, IGFALS, Insulin-like growth factor-binding protein complex acid labile subunit	7/0/0
625		P08833, IGFBP1, Insulin-like growth factor-binding protein 1	8/0/0
626		P18065, IGFBP2, Insulin-like growth factor-binding protein 2	12/0/0
627		P17936-1,2, IGFBP3, Insulin-like growth factor-binding protein 3	16/0/1
628	(1)	P22692-1, IGFBP4, Insulin-like growth factor-binding protein 4	1/0/1
	(2)	P22692-2, IGFBP4, Insulin-like growth factor-binding protein 4	
629		P24593, IGFBP5, Insulin-like growth factor-binding protein 5	13/0/0
630		P24592, IGFBP6, Insulin-like growth factor-binding protein 6	6/0/0
631		Q16270-1,2, IGFBP7, Insulin-like growth factor-binding protein 7	10/0/0
632		P01876, IGHA1, Ig alpha-1 chain C region	5/0/8
633		P01877, IGHA2, Ig alpha-2 chain C region	1/0/8
634		P01857, IGHG1, Ig gamma-1 chain C region	6/0/8
635		P01859, IGHG2, Ig gamma-2 chain C region	3/0/7
636		P01860, IGHG3, Ig gamma-3 chain C region	3/0/9
637		P01861, IGHG4, Ig gamma-4 chain C region	4/0/5
638		P01871-1,2, IGHM, Ig mu chain C region	5/0/10
639		A0A0C4DH31, NA, NA	1/0/1
		P23083, IGHV1-2, Immunoglobulin heavy variable 1-2	
640		A0A0C4DH29, NA, NA	0/0/2
		P01743, IGHV1-46, Immunoglobulin heavy variable 1-46	
641		P01742, IGHV1-69, Immunoglobulin heavy variable 1-69	3/0/1
642		P01766, IGHV3-13, Immunoglobulin heavy variable 3-13	1/0/0
643	(1)	P01768, IGHV3-30, Immunoglobulin heavy variable 3-30	0/0/1
	(2)	P01764, IGHV3-23, Immunoglobulin heavy variable 3-23	

644		P01772, IGHV3-33, Immunoglobulin heavy variable 3-33	0/0/2
645	(1)	P01780, IGHV3-7, Immunoglobulin heavy variable 3-7	1/0/1
	(2)	A0A0B4J1V1, NA, NA	
	·	P01762, IGHV3-11, Immunoglobulin heavy variable 3-11	
	·	P01763, IGHV3-48, Immunoglobulin heavy variable 3-48	
	·	P01767, IGHV3-53, Immunoglobulin heavy variable 3-53	
646		P01782, IGHV3-9, Immunoglobulin heavy variable 3-9	1/0/0
647		P01824, IGHV4-39, Immunoglobulin heavy variable 4-39	3/0/0
		P01825, IGHV4-59, Immunoglobulin heavy variable 4-59	
		P06331, IGHV4-34, Immunoglobulin heavy variable 4-34	
648		P01834, IGKC, Ig kappa chain C region	5/0/0
649		P01599, IGKV1-17, Immunoglobulin kappa variable 1-17	2/0/1
650		P01593, IGKV1D-33, Immunoglobulin kappa variable 1D-33	1/0/0
		P01594, IGKV1-33, Immunoglobulin kappa variable 1-33	
651		A0A0C4DH73, NA, NA	1/0/1
		P01597, IGKV1-39, Immunoglobulin kappa variable 1-39	
		P01611, IGKV1D-12, Immunoglobulin kappa variable 1D-12	
		P04432, IGKV1D-39, Immunoglobulin kappa variable 1D-39	
652		P01602, IGKV1-5, Immunoglobulin kappa variable 1-5	1/0/1
653	(1)	P06310, IGKV2-30, Immunoglobulin kappa variable 2-30	2/0/1
	(2)	A0A075B6S6, NA, NA	
654		A0A075B6P5, NA, NA	1/0/1
		P01614, IGKV2D-40, Immunoglobulin kappa variable 2D-40	
		P01615, IGKV2D-28, Immunoglobulin kappa variable 2D-28	
655		P04433, IGKV3-11, Immunoglobulin kappa variable 3-11	2/0/2
656		P01624, IGKV3-15, Immunoglobulin kappa variable 3-15	1/0/2
657		P01619, IGKV3-20, Immunoglobulin kappa variable 3-20	1/0/3
658		P06312, IGKV4-1, Immunoglobulin kappa variable 4-1	4/0/0
659		B9A064-1, IGLL5, Immunoglobulin lambda-like polypeptide 5	2/0/4
		P0CG04, IGLC1, Ig lambda-1 chain C regions	
660	(1)	P0CG05, IGLC2, Ig lambda-2 chain C regions	1/0/4
	(2)	P0CG06, IGLC3, Ig lambda-3 chain C regions	
661		P0CF74, IGLC6, Ig lambda-6 chain C region	0/0/4
662		A0M8Q6, IGLC7, Ig lambda-7 chain C region	1/0/4
663		P01703, IGLV1-40, Immunoglobulin lambda variable 1-40	1/0/0
664	(1)	P01700, IGLV1-47, Immunoglobulin lambda variable 1-47	1/0/0
	(2)	P01699, IGLV1-44, Immunoglobulin lambda variable 1-44	
665		P01705, IGLV2-23, Immunoglobulin lambda variable 2-23	2/0/0
666		P01709, IGLV2-8, Immunoglobulin lambda variable 2-8	2/0/0

667		P80748, IGLV3-21, Immunoglobulin lambda variable 3-21	2/0/0
668		P01717, IGLV3-25, Immunoglobulin lambda variable 3-25	1/0/1
669		P01718, IGLV3-27, Immunoglobulin lambda variable 3-27	1/0/1
670		P04211, IGLV7-43, Immunoglobulin lambda variable 7-43	2/0/0
671		Q12906-1-7, ILF3, Interleukin enhancer-binding factor 3	2/0/0
672	(1)	Q16891-1,2,4, IMMT, MICOS complex subunit MIC60	1/0/0
	(2)	Q16891-3, IMMT, MICOS complex subunit MIC60	
673		P08476, INHBA, Inhibin beta A chain	2/0/0
674		P09529, INHBB, Inhibin beta B chain	4/0/0
675		P55103, INHBC, Inhibin beta C chain	9/0/0
676		P58166, INHBE, Inhibin beta E chain	5/0/0
677	(1)	O00410-1,3, IPO5, Importin-5	1/0/0
	(2)	O00410-2, IPO5, Importin-5	
678	(1)	P46940, IQGAP1, Ras GTPase-activating-like protein IQGAP1	19/0/0
	(2)	Q86VI3, IQGAP3, Ras GTPase-activating-like protein IQGAP3	
679		O14498, ISLR, Immunoglobulin superfamily containing leucine-rich repeat protein	5/0/0
680		B1AKI9, ISM1, Isthmin-1	2/0/0
681	(1)	P05556-1, ITGB1, Integrin beta-1	1/0/0
	(2)	P05556-2-5, ITGB1, Integrin beta-1	
682		P05107, ITGB2, Integrin beta-2	6/0/0
683	(1)	O95965-1, ITGBL1, Integrin beta-like protein 1	1/0/9
	(2)	O95965-3, ITGBL1, Integrin beta-like protein 1	
684		O95965-2, ITGBL1, Integrin beta-like protein 1	1/0/9
685	(1)	P19827-1, ITIH1, Inter-alpha-trypsin inhibitor heavy chain H1	7/6/1
	(2)	P19827-2, ITIH1, Inter-alpha-trypsin inhibitor heavy chain H1	
	(3)	P19827-3, ITIH1, Inter-alpha-trypsin inhibitor heavy chain H1	
686		P19823, ITIH2, Inter-alpha-trypsin inhibitor heavy chain H2	30/0/0
687		Q06033-1,2, ITIH3, Inter-alpha-trypsin inhibitor heavy chain H3	32/0/0

688		Q14624-1, ITIH4, Inter-alpha-trypsin inhibitor heavy chain H4	1/0/32
689	(1)	Q14624-2,3, ITIH4, Inter-alpha-trypsin inhibitor heavy chain H4	0/0/32
	(2)	Q14624-4, ITIH4, Inter-alpha-trypsin inhibitor heavy chain H4	
690	(1)	Q86UX2-1, ITIH5, Inter-alpha-trypsin inhibitor heavy chain H5	0/8/0
	(2)	Q86UX2-2, ITIH5, Inter-alpha-trypsin inhibitor heavy chain H5	
	(3)	Q86UX2-3, ITIH5, Inter-alpha-trypsin inhibitor heavy chain H5	
	(4)	Q86UX2-4, ITIH5, Inter-alpha-trypsin inhibitor heavy chain H5	
691		Q8WWA0, ITLN1, Intelectin-1	6/0/7
692		Q8WWU7-1,2, ITLN2, Intelectin-2	1/0/7
693		Q9Y287-1,2, ITM2B, Integral membrane protein 2B	2/0/0
694		P01591, JCHAIN, Immunoglobulin J chain	3/0/0
695		Q96I82-1,2, KAZALD1, Kazal-type serine protease inhibitor domain-containing protein 1	1/0/0
696	(1)	Q96CX2, KCTD12, BTB/POZ domain-containing protein KCTD12	6/0/0
	(2)	Q6ZWB6, KCTD8, BTB/POZ domain-containing protein KCTD8	
697	(1)	Q07666-1,2, KHDRBS1, KH domain-containing, RNA-binding, signal transduction-associated protein 1	2/0/0
	(2)	Q5VWX1, KHDRBS2, KH domain-containing, RNA-binding, signal transduction-associated protein 2	
698		Q92945, KHSRP, Far upstream element-binding protein 2	3/0/1
699		Q9UPP5-1,2, KIAA1107, Uncharacterized protein KIAA1107	3/0/0
700		P33176, KIF5B, Kinesin-1 heavy chain	8/0/0
701		O43240, KLK10, Kallikrein-10	1/0/0
702		Q9P0G3, KLK14, Kallikrein-14	2/0/0
703		P03952, KLKB1, Plasma kallikrein	30/0/0
704		Q03164-1-3, KMT2A, Histone-lysine N-methyltransferase 2A	7/0/0
705		P01042-1, KNG1, Kininogen-1	7/0/23
706	(1)	P01042-2, KNG1, Kininogen-1	0/0/23

	(2)	P01042-3, KNG1, Kininogen-1	
707	(1)	Q14974-1, KPNB1, Importin subunit beta-1	6/0/0
	(2)	Q14974-2, KPNB1, Importin subunit beta-1	
708		Q5T749, KPRP, Keratinocyte proline-rich protein	3/0/0
709	(1)	P04264, KRT1, Keratin, type II cytoskeletal 1	29/0/8
	(2)	Q7Z794, KRT77, Keratin, type II cytoskeletal 1b	
710	(1)	P13645, KRT10, Keratin, type I cytoskeletal 10	25/0/9
	(2)	Q7Z3Y8, KRT27, Keratin, type I cytoskeletal 27	
	(3)	Q7Z3Y9, KRT26, Keratin, type I cytoskeletal 26	
	(4)	Q7Z3Z0, KRT25, Keratin, type I cytoskeletal 25	
711	(1)	P13646-1, KRT13, Keratin, type I cytoskeletal 13	1/2/12
	(2)	P13646-2, KRT13, Keratin, type I cytoskeletal 13	
	(3)	P13646-3, KRT13, Keratin, type I cytoskeletal 13	
712		P02533, KRT14, Keratin, type I cytoskeletal 14	6/0/18
713	(1)	P19012-1, KRT15, Keratin, type I cytoskeletal 15	0/0/12
	(2)	P19012-2, KRT15, Keratin, type I cytoskeletal 15	
714		P08779, KRT16, Keratin, type I cytoskeletal 16	9/0/12
715		Q04695, KRT17, Keratin, type I cytoskeletal 17	0/0/13
716		P05783, KRT18, Keratin, type I cytoskeletal 18	25/0/3
717	(1)	P08727, KRT19, Keratin, type I cytoskeletal 19	19/0/12
	(2)	Q99456, KRT12, Keratin, type I cytoskeletal 12	
	(3)	Q8N1A0-1,2, KRT222, Keratin-like protein KRT222	
718		P35908, KRT2, Keratin, type II cytoskeletal 2 epidermal	25/0/9
719		P35900, KRT20, Keratin, type I cytoskeletal 20	0/0/4
720		Q2M2I5, KRT24, Keratin, type I cytoskeletal 24	1/0/4
721		Q7Z3Y7, KRT28, Keratin, type I cytoskeletal 28	1/0/6
722		P12035, KRT3, Keratin, type II cytoskeletal 3	1/0/7
723		Q92764, KRT35, Keratin, type I cuticular Ha5	1/0/2
724		P19013, KRT4, Keratin, type II cytoskeletal 4	0/0/5
725	(1)	P13647, KRT5, Keratin, type II cytoskeletal 5	11/0/16
	(2)	Q14CN4-1-3, KRT72, Keratin, type II cytoskeletal 72	
	(3)	Q7RTS7, KRT74, Keratin, type II cytoskeletal 74	
	·	Q86Y46-1, KRT73, Keratin, type II cytoskeletal 73	
726		P04259, KRT6B, Keratin, type II cytoskeletal 6B	0/0/23
727	(1)	P48668, KRT6C, Keratin, type II cytoskeletal 6C	0/0/23
	(2)	P02538, KRT6A, Keratin, type II cytoskeletal 6A	

728 P08729, KRT7, Keratin, type II cytoskeletal 7 — 20/0/8

729 O95678, KRT75, Keratin, type II cytoskeletal 75 — 0/0/14

730 Q01546, KRT76, Keratin, type II cytoskeletal 2 oral — 0/0/8

731 (1) Q8N1N4-1, KRT78, Keratin, type II cytoskeletal 78 — 1/0/3
(2) Q8N1N4-2, KRT78, Keratin, type II cytoskeletal 78

732 Q5XKE5, KRT79, Keratin, type II cytoskeletal 79 — 0/0/8

733 P05787-1,2, KRT8, Keratin, type II cytoskeletal 8 — 26/0/11

734 P35527, KRT9, Keratin, type I cytoskeletal 9 — 28/0/1

735 P24043, LAMA2, Laminin subunit alpha-2 — 33/0/1

736 Q16787-2,3, LAMA3, Laminin subunit alpha-3 — 1/0/2

737 Q16363-1,2, LAMA4, Laminin subunit alpha-4 — 25/0/0

738 O15230-1, LAMA5, Laminin subunit alpha-5 — 12/0/1

739 P07942, LAMB1, Laminin subunit beta-1 — 31/0/0

740 P55268, LAMB2, Laminin subunit beta-2 — 16/0/0

741 P11047, LAMC1, Laminin subunit gamma-1 — 35/0/0

742 Q14847-1, LASP1, LIM and SH3 domain protein 1 — 3/0/0

743 P18428, LBP, Lipopolysaccharide-binding protein — 10/0/0

744 (1) P04180, LCAT, Phosphatidylcholine-sterol acyltransferase — 8/0/0
(2) Q8NCC3-1, PLA2G15, Group XV phospholipase A2

745 P31025, LCN1, Lipocalin-1 — 2/0/0

746 (1) P13796-1, LCP1, Plastin-2 — 11/0/7
(2) Q14651, PLS1, Plastin-1
(3) P13796-2, LCP1, Plastin-2

747 (1) P00338-1,3, LDHA, L-lactate dehydrogenase A chain — 0/1/0
(2) P00338-2,5, LDHA, L-lactate dehydrogenase A chain
(3) P00338-4, LDHA, L-lactate dehydrogenase A chain
(4) P07195, LDHB, L-lactate dehydrogenase B chain
· P07864, LDHC, L-lactate dehydrogenase C chain
· Q6ZMR3, LDHAL6A, L-lactate dehydrogenase A-like 6A

748 (1) P01130-1,5, LDLR, Low-density lipoprotein receptor — 0/3/0
(2) P01130-4, LDLR, Low-density lipoprotein receptor
(3) P01130-2,3, LDLR, Low-density lipoprotein receptor

749 O14960, LECT2, Leukocyte cell-derived chemotaxin-2 — 4/0/0

750 (1) O00292-1, LEFTY2, Left-right determination factor 2 — 2/0/0
· O75610, LEFTY1, Left-right determination factor 1
(2) O00292-2, LEFTY2, Left-right determination factor 2

751		P41159, LEP, Leptin	3/0/0
752	(1)	Q8NES3-4, LFNG, Beta-1,3-N-acetylglucosaminyltransferase lunatic fringe	1/0/0
	(2)	Q8NES3-1-3, LFNG, Beta-1,3-N-acetylglucosaminyltransferase lunatic fringe	
753		P09382, LGALS1, Galectin-1	8/0/0
754		P17931, LGALS3, Galectin-3	4/0/0
755		Q08380, LGALS3BP, Galectin-3-binding protein	14/0/0
756		Q3ZCW2, LGALSL, Galectin-related protein	1/0/0
757		Q8N0V4, LGI2, Leucine-rich repeat LGI family member 2	2/0/0
758		Q9Y5X9-1,2, LIPG, Endothelial lipase	2/0/0
759		P49257, LMAN1, Protein ERGIC-53	9/0/0
760		Q12907, LMAN2, Vesicular integral-membrane protein VIP36	3/0/0
761	(1)	P02545-1-3,6, LMNA, Prelamin-A/C [Cleaved into: Lamin-A/C	7/0/0
	(2)	P02545-4,5, LMNA, Prelamin-A/C [Cleaved into: Lamin-A/C	
762		P20700, LMNB1, Lamin-B1	8/0/0
763		Q9UIQ6-1-3, LNPEP, Leucyl-cystinyl aminopeptidase	1/0/0
764		P28300, LOX, Protein-lysine 6-oxidase	8/0/1
765		Q08397, LOXL1, Lysyl oxidase homolog 1	16/0/1
766		Q9Y4K0, LOXL2, Lysyl oxidase homolog 2	14/0/1
767		P08519, LPA, Apolipoprotein(a)	23/0/0
768		Q9UBY5, LPAR3, Lysophosphatidic acid receptor 3	5/0/0
769		P06858, LPL, Lipoprotein lipase	9/0/0
770		P02750, LRG1, Leucine-rich alpha-2-glycoprotein	2/0/1
771		Q07954-1, LRP1, Prolow-density lipoprotein receptor-related protein 1	51/0/1
772		Q9NZR2, LRP1B, Low-density lipoprotein receptor-related protein 1B	17/0/1
773	(1)	Q14114-1,3, LRP8, Low-density lipoprotein receptor-related protein 8	2/0/1
	(2)	Q14114-2,4, LRP8, Low-density lipoprotein receptor-related protein 8	
774		Q8N6Y2-1, LRRC17, Leucine-rich repeat-containing protein 17	4/0/0

775 Q8N9N7, LRRC57, Leucine-rich repeat-containing protein 57 — 2/0/0 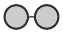

776 Q32MZ4-1-3, LRRFIP1, Leucine-rich repeat flightless-interacting protein 1 — 2/0/1 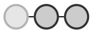

777 Q32MZ4-4, LRRFIP1, Leucine-rich repeat flightless-interacting protein 1 — 1/0/1 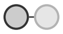

778 (1) P33241-1,3, LSP1, Lymphocyte-specific protein 1 — 2/0/0 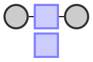
(2) P33241-2, LSP1, Lymphocyte-specific protein 1

779 (1) Q86X29-1,4, LSR, Lipolysis-stimulated lipoprotein receptor — 0/1/0 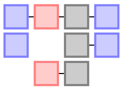
(2) Q86X29-2,3, LSR, Lipolysis-stimulated lipoprotein receptor
(3) Q86X29-5,6, LSR, Lipolysis-stimulated lipoprotein receptor

780 (1) Q14766-1,4, LTBP1, Latent-transforming growth factor beta-binding protein 1 — 2/0/28 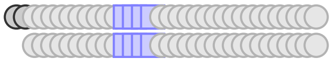
(2) Q14766-2,5, LTBP1, Latent-transforming growth factor beta-binding protein 1

781 Q14766-3, LTBP1, Latent-transforming growth factor beta-binding protein 1 — 1/0/28 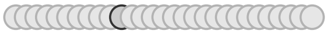

782 Q14767, LTBP2, Latent-transforming growth factor beta-binding protein 2 — 38/0/0 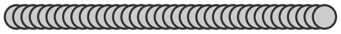

783 (1) Q9NS15-1, LTBP3, Latent-transforming growth factor beta-binding protein 3 — 1/0/1 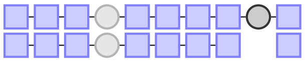
(2) Q9NS15-2, LTBP3, Latent-transforming growth factor beta-binding protein 3

784 (1) Q8N2S1-2, LTBP4, Latent-transforming growth factor beta-binding protein 4 — 2/11/0 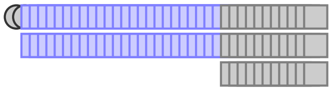
(2) Q8N2S1-1,3, LTBP4, Latent-transforming growth factor beta-binding protein 4
(3) Q8N2S1-4, LTBP4, Latent-transforming growth factor beta-binding protein 4

785 P02788-1,2, LTF, Lactotransferrin — 30/0/0 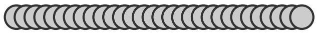

786 P51884, LUM, Lumican — 14/0/0 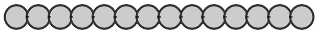

787 Q9BS40, LXN, Latexin — 2/0/0 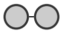

788 O75608-1,2, LYPLA1, Acyl-protein thioesterase 1 — 3/0/0 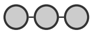

789 O95372, LYPLA2, Acyl-protein thioesterase 2 — 2/0/0 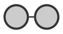

790 P61626, LYZ, Lysozyme C — 12/0/0 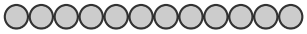

791 P20645, M6PR, Cation-dependent mannose-6-phosphate receptor — 1/0/0 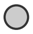

792 (1) Q9UPN3-1, MACF1, Microtubule-actin cross-linking factor 1, isoforms 1/2/3/5 — 0/4/1 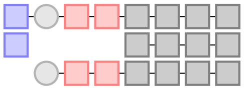

(2) Q9UPN3-2-4, MACF1, Microtubule-actin cross-linking factor 1, isoforms 1/2/3/5

(3) Q9UPN3-5, MACF1, Microtubule-actin cross-linking factor 1, isoforms 1/2/3/5

793 (1) P33908-1, MAN1A1, Mannosyl-oligosaccharide 1,2-alpha-mannosidase IA — 9/0/0

(2) P33908-2, MAN1A1, Mannosyl-oligosaccharide 1,2-alpha-mannosidase IA

794 P46821, MAP1B, Microtubule-associated protein 1B — 5/0/0

795 (1) Q14244-1,2,4,6,7, MAP7, Ensconsin — 0/0/1

(2) Q14244-3,5, MAP7, Ensconsin

(3) Q3KQU3-1,2,4, MAP7D1, MAP7 domain-containing protein 1

796 Q8IWC1-1-4, MAP7D3, MAP7 domain-containing protein 3 — 1/0/1

797 Q15691, MAPRE1, Microtubule-associated protein RP/EB family member 1 — 2/0/0

798 P29966, MARCKS, Myristoylated alanine-rich C-kinase substrate — 3/0/0

799 Q9UEW3-1,2, MARCO, Macrophage receptor MARCO — 4/0/0

800 (1) P48740-1, MASP1, Mannan-binding lectin serine protease 1 — 4/0/8

(2) P48740-3, MASP1, Mannan-binding lectin serine protease 1

801 P48740-2,4, MASP1, Mannan-binding lectin serine protease 1 — 2/0/8

802 O00187-1, MASP2, Mannan-binding lectin serine protease 2 — 2/0/6

803 O00187-2, MASP2, Mannan-binding lectin serine protease 2 — 1/0/6

804 (1) O00339-1-3, MATN2, Matrilin-2 — 2/0/0

(2) O00339-4, MATN2, Matrilin-2

805 (1) P43243-1, MATR3, Matrin-3 — 4/0/0

(2) P43243-2, MATR3, Matrin-3

806 P02144, MB, Myoglobin — 6/0/0

807 P21741-1, MDK, Midkine — 3/0/0

808 Q9NU22, MDN1, Midasin — 3/0/0

809 (1) O75095-1, MEGF6, Multiple epidermal growth factor-like domains protein 6 — 1/0/0

(2) O75095-2, MEGF6, Multiple epidermal growth factor-like domains protein 6

810 (1) Q641Q3-1, METRNL, Meteorin-like protein — 3/0/0

(2) Q641Q3-2, METRNL, Meteorin-like protein

811 P55001-1-3, MFAP2, Microfibrillar-associated protein 2 — 3/0/0

812 P55083-1,2, MFAP4, Microfibril-associated glycoprotein 4 — 7/0/0

813 Q13361-1,2, MFAP5, Microfibrillar-associated protein 5 — 5/0/1

814 Q8IWA4-1,3, MFN1, Mitofusin-1 — 2/0/0

815 P26572, MGAT1, Alpha-1,3-mannosyl-glycoprotein 2-beta-N-acetylglucosaminyltransferase — 10/0/0

816 P08493-1,2, MGP, Matrix Gla protein — 5/0/0

817 Q5JRA6-1,2, MIA3, Melanoma inhibitory activity protein 3 — 2/0/0

0/2/0

818 (1) Q9UNW1-1, MINPP1, Multiple inositol polyphosphate phosphatase 1
(2) Q9UNW1-2,3, MINPP1, Multiple inositol polyphosphate phosphatase 1
(3) Q9UNW1-4, MINPP1, Multiple inositol polyphosphate phosphatase 1

819 Q14165, MLEC, Malectin — 4/0/0

820 P50281, MMP14, Matrix metalloproteinase-14 — 5/0/0

2/15/0

821 (1) P08253-1, MMP2, 72 kDa type IV collagenase
(2) P08253-2, MMP2, 72 kDa type IV collagenase
(3) P08253-3, MMP2, 72 kDa type IV collagenase

822 O75900-1-3, MMP23A, Matrix metalloproteinase-23 — 2/0/0

823 Q9H8L6, MMRN2, Multimerin-2 — 4/0/0

824 P41218, MNDA, Myeloid cell nuclear differentiation antigen — 3/0/0

825 P05164-1-3, MPO, Myeloperoxidase — 7/0/2

826 P25325-1,2, MPST, 3-mercaptopyruvate sulfurtransferase — 2/0/0

827 Q9UBG0, MRC2, C-type mannose receptor 2 — 3/0/0

828 Q8NDA8-1,2, MROH1, Maestro heat-like repeat-containing protein family member 1 — 2/0/0

829 Q13421-1-4, MSLN, Mesothelin — 13/0/0

830 (1) P26038, MSN, Moesin — 10/0/1
(2) P35241-1,5, RDX, Radixin

1/0/0

831 (1) P21757-1, MSR1, Macrophage scavenger receptor types I and II
(2) P21757-2,3, MSR1, Macrophage scavenger receptor types I and II

832 (1) P26927, MST1, Hepatocyte growth factor-like protein — 4/4/0
(2) Q2TV78-1, MST1L, Putative macrophage stimulating 1-like protein
(3) Q2TV78-2, MST1L, Putative macrophage stimulating 1-like protein

833		P11586, MTHFD1, C-1-tetrahydrofolate synthase, cytoplasmic	7/0/0
834		Q02817, MUC2, Mucin-2	7/0/0
835		Q14764, MVP, Major vault protein	5/0/0
836		Q9NR99, MXRA5, Matrix-remodeling-associated protein 5	54/0/0
837	(1)	P84157-1, MXRA7, Matrix-remodeling-associated protein 7	1/0/2
	(2)	P84157-3, MXRA7, Matrix-remodeling-associated protein 7	
838		P84157-2, MXRA7, Matrix-remodeling-associated protein 7	1/0/2
839	(1)	P12882, MYH1, Myosin-1	1/1/1
	(2)	Q9UKX3, MYH13, Myosin-13	
	·	Q9Y623, MYH4, Myosin-4	
	(3)	Q9UKX2-1, MYH2, Myosin-2	
840	(1)	P35580-4,5, MYH10, Myosin-10	1/0/15
	(2)	P35580-1-3, MYH10, Myosin-10	
841		P35749-1-4, MYH11, Myosin-11	1/0/16
842	(1)	Q7Z406-1,2,6, MYH14, Myosin-14	0/0/6
	(2)	Q7Z406-4,5, MYH14, Myosin-14	
843		P13533, MYH6, Myosin-6	0/0/6
844		P12883, MYH7, Myosin-7	1/0/5
845	(1)	P35579-1, MYH9, Myosin-9	19/0/15
	(2)	P35579-2, MYH9, Myosin-9	
846	(1)	P05976-1, MYL1, Myosin light chain 1/3, skeletal muscle isoform	3/0/0
	(2)	P05976-2, MYL1, Myosin light chain 1/3, skeletal muscle isoform	
847		O14950, MYL12B, Myosin regulatory light chain 12B	2/0/4
		P19105, MYL12A, Myosin regulatory light chain 12A	
848	(1)	P10916, MYL2, Myosin regulatory light chain 2, ventricular/cardiac muscle isoform	5/0/1
	(2)	Q9BUA6, MYL10, Myosin regulatory light chain 10	
	(3)	Q02045-1, MYL5, Myosin light chain 5	
849		P08590, MYL3, Myosin light chain 3	7/0/0
850	(1)	P60660-1,2, MYL6, Myosin light polypeptide 6	4/0/0
	(2)	P14649, MYL6B, Myosin light chain 6B	
851	(1)	P24844-1, MYL9, Myosin regulatory light polypeptide 9	0/0/4
	(2)	P24844-2, MYL9, Myosin regulatory light polypeptide 9	

852	(1)	Q15746-1-7,11, MYLK, Myosin light chain kinase, smooth muscle	1/4/0
	(2)	Q15746-8,10, MYLK, Myosin light chain kinase, smooth muscle	
	(3)	Q15746-9, MYLK, Myosin light chain kinase, smooth muscle	
853		Q96A32, MYLPF, Myosin regulatory light chain 2, skeletal muscle isoform	12/0/0
854		Q99972, MYOC, Myocilin	12/0/0
855		P43490, NAMPT, Nicotinamide phosphoribosyltransferase	3/0/0
856		Q9NR45, NANS, Sialic acid synthase	3/0/0
857	(1)	P55209-1-3, NAP1L1, Nucleosome assembly protein 1-like 1	1/0/0
	(2)	Q99733-1,2, NAP1L4, Nucleosome assembly protein 1-like 4	
858		P54920, NAPA, Alpha-soluble NSF attachment protein	4/0/0
859		P49321-1,3,4, NASP, Nuclear autoantigenic sperm protein	2/0/0
860		P41271-1,2, NBL1, Neuroblastoma suppressor of tumorigenicity 1	2/0/0
861		A6NI72, NCF1B, Putative neutrophil cytosol factor 1B	
		A8MVU1, NCF1C, Putative neutrophil cytosol factor 1C	2/0/0
		P14598-1, NCF1, Neutrophil cytosol factor 1	
862		P19338, NCL, Nucleolin	13/0/0
863		Q92597-1-3, NDRG1, Protein NDRG1	2/0/0
864	(1)	Q9NVZ3-1,2, NECAP2, Adaptin ear-binding coat-associated protein 2	0/3/0
	(2)	Q9NVZ3-3, NECAP2, Adaptin ear-binding coat-associated protein 2	
	(3)	Q9NVZ3-4, NECAP2, Adaptin ear-binding coat-associated protein 2	
865		Q92692-1,2, NECTIN2, Nectin-2	2/0/0
866		P12036-1,2, NEFH, Neurofilament heavy polypeptide	1/0/3
867		P48681, NES, Nestin	6/0/0
868		Q0ZGT2-1-4, NEXN, Nexilin	2/0/0
869		P19838-1-3, NFKB1, Nuclear factor NF-kappa-B p105 subunit	3/0/0
870	(1)	P14543-1, NID1, Nidogen-1	1/0/0
	(2)	P14543-2, NID1, Nidogen-1	
871		Q14112-1,2, NID2, Nidogen-2	10/0/1
872		P30414, NKTR, NK-tumor recognition protein	2/0/0

873		P40261, NNMT, Nicotinamide N-methyltransferase	1/0/0
874		Q15233-1,2, NONO, Non-POU domain-containing octamer-binding protein	2/0/2
875		Q9UM47, NOTCH3, Neurogenic locus notch homolog protein 3	6/0/0
876	(1)	P06748-1,3, NPM1, Nucleophosmin	1/0/0
	(2)	P06748-2, NPM1, Nucleophosmin	
877		P01160, NPPA, Natriuretic peptides A	4/0/0
878	(1)	O60462-4,5, NRP2, Neuropilin-2	1/9/0
	(2)	O60462-1-3, NRP2, Neuropilin-2	
	(3)	O60462-6, NRP2, Neuropilin-2	
879		P46459-1,2, NSF, Vesicle-fusing ATPase	3/0/0
880		Q02818, NUCB1, Nucleobindin-1	30/0/1
881		P80303-1,2, NUCB2, Nucleobindin-2	15/0/1
882		Q9H1E3-1, NUCKS1, Nuclear ubiquitous casein and cyclin-dependent kinase substrate 1	6/0/0
883		Q9Y266, NUDC, Nuclear migration protein nudC	2/0/0
884		Q14980-1-5, NUMA1, Nuclear mitotic apparatus protein 1	1/0/1
885		Q86UD1, OAF, Out at first protein homolog	6/0/0
886		P20774, OGN, Mimecan	8/0/0
887		Q8WWZ8-1, OIT3, Oncoprotein-induced transcript 3 protein	3/0/0
888		Q6UWY5, OLFML1, Olfactomedin-like protein 1	8/0/0
889		Q68BL8-1, OLFML2B, Olfactomedin-like protein 2B	3/0/0
890	(1)	Q9NRN5-1, OLFML3, Olfactomedin-like protein 3	0/5/0
	(2)	Q9NRN5-2, OLFML3, Olfactomedin-like protein 3	
	(3)	Q9NRN5-3, OLFML3, Olfactomedin-like protein 3	
891		Q99983, OMD, Osteomodulin	3/0/0
892		Q96CV9-1-3, OPTN, Optineurin	0/0/2
893		P02763, ORM1, Alpha-1-acid glycoprotein 1	3/0/6
894		P19652, ORM2, Alpha-1-acid glycoprotein 2	3/0/6
895		P04220, NA, Ig mu heavy chain disease protein	1/0/10
896		Q32P28-1,3,4, P3H1, Prolyl 3-hydroxylase 1	6/0/1
897		P13674-1-3, P4HA1, Prolyl 4-hydroxylase subunit alpha-1	2/0/0

898 O15460-1,2, P4HA2, Prolyl 4-hydroxylase subunit alpha-2 — 3/0/0

899 P07237, P4HB, Protein disulfide-isomerase — 23/0/1

900 (1) P11940-1,2, PABPC1, Polyadenylate-binding protein 1 — 2/0/0
(2) Q9H361, PABPC3, Polyadenylate-binding protein 3

901 (1) Q9UNF0-1, PACSIN2, Protein kinase C and casein kinase substrate in neurons protein 2 — 2/0/0
(2) Q9UNF0-2, PACSIN2, Protein kinase C and casein kinase substrate in neurons protein 2

902 Q13177, PAK2, Serine/threonine-protein kinase PAK 2 — 2/0/0

903 (1) P19021-1,3,5, PAM, Peptidyl-glycine alpha-amidating monooxygenase — 0/11/0
(2) P19021-2, PAM, Peptidyl-glycine alpha-amidating monooxygenase
(3) P19021-4,6, PAM, Peptidyl-glycine alpha-amidating monooxygenase

904 (1) Q6UXH9-1, PAMR1, Inactive serine protease PAMR1 — 0/0/10
(2) Q6UXH9-2, PAMR1, Inactive serine protease PAMR1

905 Q6UXH9-3, PAMR1, Inactive serine protease PAMR1 — 2/0/10

906 (1) O95428-1,5,6, PAPLN, Papilin — 0/11/0
(2) O95428-2, PAPLN, Papilin
(3) O95428-3, PAPLN, Papilin
(4) O95428-4, PAPLN, Papilin

907 B3GLJ2, PATE3, Prostate and testis expressed protein 3 — 2/0/0

908 Q96AQ6-1,2, PBXIP1, Pre-B-cell leukemia transcription factor-interacting protein 1 — 3/0/0

909 (1) Q15365, PCBP1, Poly(rC)-binding protein 1 — 4/0/4
(2) P57721-1-5, PCBP3, Poly(rC)-binding protein 3

910 Q15366-1-8, PCBP2, Poly(rC)-binding protein 2 — 1/0/4

911 P22061-1,2, PCMT1, Protein-L-isoaspartate(D-aspartate) O-methyltransferase — 3/0/0

912 Q15113, PCOLCE, Procollagen C-endopeptidase enhancer 1 — 19/0/0

913 Q9UKZ9, PCOLCE2, Procollagen C-endopeptidase enhancer 2 — 10/0/0

914 Q9UHG2, PCSK1N, ProSAAS — 5/0/0

915 (1) Q92824-1, PCSK5, Proprotein convertase subtilisin/kexin type 5 — 11/0/0
(2) Q92824-2, PCSK5, Proprotein convertase subtilisin/kexin type 5

916 Q16549, PCSK7, Proprotein convertase subtilisin/kexin type 7 — 1/0/0

917 (1) Q8NBP7-1, PCSK9, Proprotein convertase subtilisin/kexin type 9 — 9/0/0
(2) Q8NBP7-2, PCSK9, Proprotein convertase subtilisin/kexin type 9

918 (1) Q9UHG3-1, PCYOX1, Prenylcysteine oxidase 1 — 1/0/0
(2) Q9UHG3-2, PCYOX1, Prenylcysteine oxidase 1

919 (1) Q8WUM4-1,2, PDCD6IP, Programmed cell death 6-interacting protein — 16/0/0
(2) Q8WUM4-3, PDCD6IP, Programmed cell death 6-interacting protein

920 P01127-1,2, PDGFB, Platelet-derived growth factor subunit B — 2/0/0

921 (1) Q9NRA1-1, PDGFC, Platelet-derived growth factor C — 2/5/0
(2) Q9NRA1-2, PDGFC, Platelet-derived growth factor C
(3) Q9NRA1-3, PDGFC, Platelet-derived growth factor C

922 Q9GZP0-1,2, PDGFD, Platelet-derived growth factor D — 7/0/0

923 Q15198, PDGFRL, Platelet-derived growth factor receptor-like protein — 6/0/0

924 P30101, PDIA3, Protein disulfide-isomerase A3 — 12/0/0

925 P13667, PDIA4, Protein disulfide-isomerase A4 — 9/0/0

926 Q15084-1-5, PDIA6, Protein disulfide-isomerase A6 — 9/0/0

927 Q9NR12-1,2, PDLIM7, PDZ and LIM domain protein 7 — 3/0/0

928 Q6P996-1,3-5, PDXDC1, Pyridoxal-dependent decarboxylase domain-containing protein 1 — 1/0/0

929 P01210, PENK, Proenkephalin-A [Cleaved into: Synenkephalin; Met-enkephalin — 10/0/0

930 P17858-1,2, PFKL, ATP-dependent 6-phosphofructokinase, liver type — 6/0/1

931 P08237-1,3, PFKM, ATP-dependent 6-phosphofructokinase, muscle type — 2/0/1

932 P07737, PFN1, Profilin-1 — 4/0/0

933 P18669, PGAM1, Phosphoglycerate mutase 1 — 2/0/0

934 Q96PD5-1,2, PGLYRP2, N-acetylmuramoyl-L-alanine amidase — 10/0/0

935 P36871-1, PGM1, Phosphoglucomutase-1 — 2/0/0

936 O95394-1,3,4, PGM3, Phosphoacetylglucosamine mutase — 3/0/0

937		O00264-1,2, PGRMC1, Membrane-associated progesterone receptor component 1	2/0/0
938		P35232-1, PHB, Prohibitin	1/0/0
939	(1)	Q99623-1, PHB2, Prohibitin-2	1/0/0
	(2)	Q99623-2, PHB2, Prohibitin-2	
940		O43175, PHGDH, D-3-phosphoglycerate dehydrogenase	9/0/0
941		Q9NRX4-1,2, PHPT1, 14 kDa phosphohistidine phosphatase	1/0/0
942		Q13492-1-5, PICALM, Phosphatidylinositol-binding clathrin assembly protein	2/0/0
943		P01833, PIGR, Polymeric immunoglobulin receptor	9/0/0
944		Q00169, PITPNA, Phosphatidylinositol transfer protein alpha isoform	2/0/1
945		P48739-1-3, PITPNB, Phosphatidylinositol transfer protein beta isoform	1/0/1
946		Q504Y2, PKDCC, Extracellular tyrosine-protein kinase PKDCC	4/0/0
947	(1)	P14618-2, PKM, Pyruvate kinase PKM	1/21/0
	(2)	P14618-1, PKM, Pyruvate kinase PKM	
	(3)	P14618-3, PKM, Pyruvate kinase PKM	
948		P14555, PLA2G2A, Phospholipase A2, membrane associated	11/0/0
949	(1)	P00750-1, PLAT, Tissue-type plasminogen activator	0/1/11
	(2)	P00750-2, PLAT, Tissue-type plasminogen activator	
	(3)	P00750-4, PLAT, Tissue-type plasminogen activator	
950		P00750-3, PLAT, Tissue-type plasminogen activator	1/0/11
951		Q15149-1, PLEC, Plectin	0/0/95
952	(1)	Q15149-6, PLEC, Plectin	1/0/94
	(2)	Q15149-3, PLEC, Plectin	
	(3)	Q15149-2,4,5,7-9, PLEC, Plectin	
953	(1)	Q8TD55-2, PLEKHO2, Pleckstrin homology domain-containing family O member 2	1/0/0
	(2)	Q8TD55-1, PLEKHO2, Pleckstrin homology domain-containing family O member 2	
954	(1)	P00747, PLG, Plasminogen	46/0/5
	(2)	Q15195, PLGLA, Plasminogen-like protein A	
955		Q02325, PLGLB1, Plasminogen-like protein B	1/0/4

956 (1) O60664-1, PLIN3, Perilipin-3 — 0/8/0
(2) O60664-2, PLIN3, Perilipin-3
(3) O60664-3, PLIN3, Perilipin-3
(4) O60664-4, PLIN3, Perilipin-3

957 Q02809-1,2, PLOD1, Procollagen-lysine,2-oxoglutarate 5-dioxygenase 1 — 4/0/0

958 O60568, PLOD3, Procollagen-lysine,2-oxoglutarate 5-dioxygenase 3 — 4/0/0

959 (1) P13797-1, PLS3, Plastin-3 — 1/0/7
(2) P13797-2,3, PLS3, Plastin-3

960 (1) P55058-1, PLTP, Phospholipid transfer protein — 0/13/0
(2) P55058-2,3, PLTP, Phospholipid transfer protein
(3) P55058-4, PLTP, Phospholipid transfer protein

961 Q6UX71-1,2, PLXDC2, Plexin domain-containing protein 2 — 3/0/0

962 O15031, PLXNB2, Plexin-B2 — 3/0/0

963 (1) Q7Z5L7-1-3, PODN, Podocan — 2/0/0
(2) Q7Z5L7-4, PODN, Podocan

964 P27169, PON1, Serum paraoxonase/arylesterase 1 — 11/0/2

965 Q15166, PON3, Serum paraoxonase/lactonase 3 — 6/0/2

966 Q15063-1, POSTN, Periostin — 2/0/40

967 Q15063-2, POSTN, Periostin — 0/0/38

968 Q15063-3, POSTN, Periostin — 0/0/41

969 Q15063-4, POSTN, Periostin — 0/0/38

970 Q15063-5, POSTN, Periostin — 0/0/41

971 Q15063-6, POSTN, Periostin — 0/0/36

972 Q15063-7, POSTN, Periostin — 0/0/36

973 Q6S8J7-1,2, POTEA, POTE ankyrin domain family member A — 3/0/0

974 Q6S8J3-1, POTEE, POTE ankyrin domain family member E — 3/0/12

975 A5A3E0, POTEF, POTE ankyrin domain family member F — 2/0/10

976 P0CG38, POTEI, POTE ankyrin domain family member I — 0/0/11
P0CG39, POTEJ, POTE ankyrin domain family member J

977 (1) P62937-1, PPIA, Peptidyl-prolyl cis-trans isomerase A — 1/2/0
(2) A0A075B759, NA, NA

	·	A0A0B4J2A2, NA, NA	
	·	F5H284, PPIAL4D, Peptidyl-prolyl cis-trans isomerase A-like 4D	
	·	P0DN26, PPIAL4F, Peptidyl-prolyl cis-trans isomerase A-like 4F	
	·	P0DN37, PPIAL4G, Peptidyl-prolyl cis-trans isomerase A-like 4G	
	·	Q9Y536, PPIAL4A, Peptidyl-prolyl cis-trans isomerase A-like 4A	
	(3)	P62937-2, PPIA, Peptidyl-prolyl cis-trans isomerase A	
978		P23284, PPIB, Peptidyl-prolyl cis-trans isomerase B	3/0/0
979		O60437, PPL, Periplakin	6/0/1
980		P62140, PPP1CB, Serine/threonine-protein phosphatase PP1-beta catalytic subunit	2/0/0
981	(1)	Q15435-1,3, PPP1R7, Protein phosphatase 1 regulatory subunit 7	1/0/0
	(2)	Q15435-2,4,5, PPP1R7, Protein phosphatase 1 regulatory subunit 7	
982		P62714, PPP2CB, Serine/threonine-protein phosphatase 2A catalytic subunit beta isoform	1/0/0
		P67775-1,2, PPP2CA, Serine/threonine-protein phosphatase 2A catalytic subunit alpha isoform	
983		P30153, PPP2R1A, Serine/threonine-protein phosphatase 2A 65 kDa regulatory subunit A alpha isoform	7/0/0
984		P63151-1,2, PPP2R2A, Serine/threonine-protein phosphatase 2A 55 kDa regulatory subunit B alpha isoform	3/0/0
985	(1)	Q96NZ9-1,4, PRAP1, Proline-rich acidic protein 1	0/0/3
	(2)	Q96NZ9-2, PRAP1, Proline-rich acidic protein 1	
986		Q96NZ9-3, PRAP1, Proline-rich acidic protein 1	1/0/3
987	(1)	Q06830, PRDX1, Peroxiredoxin-1	3/0/1
	(2)	Q13162, PRDX4, Peroxiredoxin-4	
988	(1)	P32119-1, PRDX2, Peroxiredoxin-2	2/0/1
	(2)	P32119-2, PRDX2, Peroxiredoxin-2	
989		P30041, PRDX6, Peroxiredoxin-6	9/0/0
990		P51888, PRELP, Prolargin	6/0/0
991		P13727-1,2, PRG2, Bone marrow proteoglycan	1/0/0
992		Q9Y2Y8, PRG3, Proteoglycan 3	1/0/0
993	(1)	Q92954-1,6, PRG4, Proteoglycan 4	0/2/40
	(2)	Q92954-2, PRG4, Proteoglycan 4	
	(3)	Q92954-5, PRG4, Proteoglycan 4	

0/0/40
994 (1) Q92954-3, PRG4, Proteoglycan 4
(2) Q92954-4, PRG4, Proteoglycan 4

2/0/0
995 Q13131-1,2, PRKAA1, 5'-AMP-activated protein kinase catalytic subunit alpha-1

2/0/0
996 P10644-1, PRKAR1A, cAMP-dependent protein kinase type I-alpha regulatory subunit

1/0/0
997 (1) P13861-1, PRKAR2A, cAMP-dependent protein kinase type II-alpha regulatory subunit
(2) P13861-2, PRKAR2A, cAMP-dependent protein kinase type II-alpha regulatory subunit

9/0/0
998 P14314-1,2, PRKCSH, Glucosidase 2 subunit beta

2/0/1
999 P51817, PRKX, cAMP-dependent protein kinase catalytic subunit PRKX

4/0/0
1000 P04156-1,2, PRNP, Major prion protein

12/0/0
1001 P04070-1,2, PROC, Vitamin K-dependent protein C

22/0/1
1002 P07225, PROS1, Vitamin K-dependent protein S

3/0/0
1003 P22891-1,2, PROZ, Vitamin K-dependent protein Z

0/0/5
1004 P41219-1,2, PRPH, Peripherin

1005 P11908-1,2, PRPS2, Ribose-phosphate pyrophosphokinase 2
2/0/0
P60891-1,2, PRPS1, Ribose-phosphate pyrophosphokinase 1

1006 (1) P07478, PRSS2, Trypsin-2
3/0/0
(2) P07477, PRSS1, Trypsin-1
· Q8NHM4, PRSS3P2, Putative trypsin-6

1/0/0
1007 O95084-1, PRSS23, Serine protease 23

18/0/0
1008 P07602-1-3, PSAP, Prosaposin

2/0/0
1009 Q6NUJ1, PSAPL1, Proactivator polypeptide-like 1 [Cleaved into: Saposin A-like; Saposin B-Val-like; Saposin B-like; Saposin C-like; Saposin D-like]

2/0/0
1010 P25786-1,2, PSMA1, Proteasome subunit alpha type-1

3/0/0
1011 P25788-1,2, PSMA3, Proteasome subunit alpha type-3

1/0/0
1012 (1) P28066-1, PSMA5, Proteasome subunit alpha type-5
(2) P28066-2, PSMA5, Proteasome subunit alpha type-5

1/0/0
1013 (1) P60900-1, PSMA6, Proteasome subunit alpha type-6
(2) P60900-2, PSMA6, Proteasome subunit alpha type-6

3/0/0
1014 O14818-1,2, PSMA7, Proteasome subunit alpha type-7

2/0/0
1015 P20618, PSMB1, Proteasome subunit beta type-1

1/0/0
1016 Q99436-1,2, PSMB7, Proteasome subunit beta type-7

1017		Q99460-1,2, PSMD1, 26S proteasome non-ATPase regulatory subunit 1	3/0/0
1018		Q9UNM6-1,2, PSMD13, 26S proteasome non-ATPase regulatory subunit 13	1/0/0
1019		Q13200-1,3, PSMD2, 26S proteasome non-ATPase regulatory subunit 2	3/0/0
1020		O43242-1, PSMD3, 26S proteasome non-ATPase regulatory subunit 3	1/0/0
1021		Q06323-1-3, PSME1, Proteasome activator complex subunit 1	5/0/0
1022		Q9UL46, PSME2, Proteasome activator complex subunit 2	7/0/0
1023		O95456-1,2, PSMG1, Proteasome assembly chaperone 1	1/0/0
1024	(1)	P26599-1-3, PTBP1, Polypyrimidine tract-binding protein 1	2/0/0
	(2)	O95758-1-7, PTBP3, Polypyrimidine tract-binding protein 3	
1025		P41222, PTGDS, Prostaglandin-H2 D-isomerase	3/0/0
1026		Q16647, PTGIS, Prostacyclin synthase	3/0/0
1027		Q14914-1,2, PTGR1, Prostaglandin reductase 1	2/0/0
1028		P06454-1,2, PTMA, Prothymosin alpha [Cleaved into: Prothymosin alpha, N-terminally processed; Thymosin alpha-1]	2/0/0
1029		P20962, PTMS, Parathymosin	3/0/0
1030		P21246, PTN, Pleiotrophin	2/0/0
1031		P10586-1,2, PTPRF, Receptor-type tyrosine-protein phosphatase F	2/0/0
1032	(1)	Q6NZI2-1, PTRF, Polymerase I and transcript release factor	5/6/0
	(2)	Q6NZI2-2, PTRF, Polymerase I and transcript release factor	
	(3)	Q6NZI2-3, PTRF, Polymerase I and transcript release factor	
1033		P26022, PTX3, Pentraxin-related protein PTX3	2/0/0
1034	(1)	Q92626-1, PXDN, Peroxidasin homolog	16/0/1
	(2)	Q92626-2, PXDN, Peroxidasin homolog	
1035		P49023-1-4, PXN, Paxillin	3/0/0
1036	(1)	P20742-1, PZP, Pregnancy zone protein	2/0/8
	(2)	P20742-2, PZP, Pregnancy zone protein	
1037		P47897-1,2, QARS, Glutamine--tRNA ligase	1/0/0
1038	(1)	Q16769-1, QPCT, Glutaminyl-peptide cyclotransferase	2/0/0
	(2)	Q16769-2, QPCT, Glutaminyl-peptide cyclotransferase	
1039	(1)	O00391-1, QSOX1, Sulfhydryl oxidase 1	2/0/0

	(2)	O00391-2, QSOX1, Sulfhydryl oxidase 1	
1040		Q9H974-1-4, QTRT2, Queuine tRNA-ribosyltransferase accessory subunit 2	3/0/0
1041		P61026, RAB10, Ras-related protein Rab-10	2/0/0
1042		P62491-1,2, RAB11A, Ras-related protein Rab-11A	3/0/0
		Q15907-1,2, RAB11B, Ras-related protein Rab-11B	
1043		P62820-1,2, RAB1A, Ras-related protein Rab-1A	2/0/1
1044		Q9H0U4, RAB1B, Ras-related protein Rab-1B	1/0/1
1045	(1)	P51148-1,2, RAB5C, Ras-related protein Rab-5C	3/0/0
	(2)	P20339-1,2, RAB5A, Ras-related protein Rab-5A	
	·	P61020-1,2, RAB5B, Ras-related protein Rab-5B	
1046		P51149, RAB7A, Ras-related protein Rab-7a	2/0/0
1047	(1)	Q15276-1, RABEP1, Rab GTPase-binding effector protein 1	1/0/1
	(2)	Q15276-2, RABEP1, Rab GTPase-binding effector protein 1	
1048	(1)	P15153, RAC2, Ras-related C3 botulinum toxin substrate 2	1/0/1
	·	P63000-1,2, RAC1, Ras-related C3 botulinum toxin substrate 1	
	(2)	P60763, RAC3, Ras-related C3 botulinum toxin substrate 3	
	(3)	P17081, RHOQ, Rho-related GTP-binding protein RhoQ	
	·	P84095, RHOG, Rho-related GTP-binding protein RhoG	
	·	Q9H4E5-1,2, RHOJ, Rho-related GTP-binding protein RhoJ	
1049		P63244, RACK1, Receptor of activated protein C kinase 1	6/0/0
1050		P54727-1,2, RAD23B, UV excision repair protein RAD23 homolog B	2/0/0
1051		Q9UKM9-1,2, RALY, RNA-binding protein Raly	3/0/0
1052		P62826, RAN, GTP-binding nuclear protein Ran	4/0/0
1053		A6NIZ1, NA, Ras-related protein Rap-1b-like protein	1/0/0
		P61224-1-3, RAP1B, Ras-related protein Rap-1b	
		P62834, RAP1A, Ras-related protein Rap-1A	
1054		P49788-1,2, RARRES1, Retinoic acid receptor responder protein 1	7/0/0
1055		Q99969, RARRES2, Retinoic acid receptor responder protein 2	8/0/0
1056		P54136-1,2, RARS, Arginine--tRNA ligase, cytoplasmic	1/0/0
1057	(1)	Q09028-1,3, RBBP4, Histone-binding protein RBBP4	1/0/0
	(2)	Q09028-2,4, RBBP4, Histone-binding protein RBBP4	
	·	Q16576-1,2, RBBP7, Histone-binding protein RBBP7	
1058	(1)	P78332-3, RBM6, RNA-binding protein 6	1/0/0
	(2)	P78332-1, RBM6, RNA-binding protein 6	
1059	(1)	P38159-1,2, RBMX, RNA-binding motif protein, X chromosome	3/0/0

	(2)	Q96E39, RBMXL1, RNA binding motif protein, X-linked-like-1	
1060		P02753, RBP4, Retinol-binding protein 4	9/0/0
1061		Q15293-1,2, RCN1, Reticulocalbin-1	2/0/0
1062		Q96D15, RCN3, Reticulocalbin-3	5/0/0
1063		O95980, RECK, Reversion-inducing cysteine-rich protein with Kazal motifs	2/0/0
1064		Q06141, REG3A, Regenerating islet-derived protein 3-alpha	3/0/0
1065		P08134, RHOC, Rho-related GTP-binding protein RhoC	2/0/0
		P61586, RHOA, Transforming protein RhoA	
1066		Q9NPQ8-1-4, RIC8A, Synembryn-A	1/0/1
1067		Q13129, RLF, Zinc finger protein Rlf	4/0/0
1068		P07998, RNASE1, Ribonuclease pancreatic	6/0/0
1069		P12724, RNASE3, Eosinophil cationic protein	5/0/0
1070		P34096, RNASE4, Ribonuclease 4	11/0/0
1071		Q93091, RNASE6, Ribonuclease K6	2/0/0
1072		P13489, RNH1, Ribonuclease inhibitor	11/0/0
1073		P27694, RPA1, Replication protein A 70 kDa DNA-binding subunit	5/0/0
1074		P62906, RPL10A, 60S ribosomal protein L10a	2/0/0
1075		P30050-1, RPL12, 60S ribosomal protein L12	3/0/0
1076		P26373-1,2, RPL13, 60S ribosomal protein L13	2/0/1
1077		P50914, RPL14, 60S ribosomal protein L14	5/0/0
1078		Q07020-1,2, RPL18, 60S ribosomal protein L18	1/0/0
1079		P84098, RPL19, 60S ribosomal protein L19	1/0/1
1080		P46778, RPL21, 60S ribosomal protein L21	2/0/0
1081		P35268, RPL22, 60S ribosomal protein L22	4/0/0
1082		P62750, RPL23A, 60S ribosomal protein L23a	4/0/0
1083		P83731, RPL24, 60S ribosomal protein L24	2/0/0
1084	(1)	P61254, RPL26, 60S ribosomal protein L26	1/0/0
	(2)	Q9UNX3, RPL26L1, 60S ribosomal protein L26-like 1	
1085		P61353, RPL27, 60S ribosomal protein L27	4/0/0
1086	(1)	P46779-1, RPL28, 60S ribosomal protein L28	1/1/0
	(2)	P46779-2,3, RPL28, 60S ribosomal protein L28	
	(3)	P46779-4,5, RPL28, 60S ribosomal protein L28	

1087		P62888, RPL30, 60S ribosomal protein L30	3/0/0
1088		P62899-1-3, RPL31, 60S ribosomal protein L31	3/0/0
1089		P62910, RPL32, 60S ribosomal protein L32	2/0/0
1090		P49207, RPL34, 60S ribosomal protein L34	3/0/0
1091		P42766, RPL35, 60S ribosomal protein L35	2/0/1
1092		A6NKH3, RPL37AP8, Putative 60S ribosomal protein L37a-like protein	1/0/0
		P61513, RPL37A, 60S ribosomal protein L37a	
1093		P36578, RPL4, 60S ribosomal protein L4	3/0/0
1094		P46777, RPL5, 60S ribosomal protein L5	5/0/0
1095		Q02878, RPL6, 60S ribosomal protein L6	3/0/0
1096		P18124, RPL7, 60S ribosomal protein L7	3/0/0
1097		P62424, RPL7A, 60S ribosomal protein L7a	4/0/0
1098		P62917, RPL8, 60S ribosomal protein L8	5/0/0
1099		P32969, RPL9, 60S ribosomal protein L9	3/0/0
1100		P05388-1,2, RPLP0, 60S acidic ribosomal protein P0	5/0/0
		Q8NHW5, RPLP0P6, 60S acidic ribosomal protein P0-like	
1101		P05387, RPLP2, 60S acidic ribosomal protein P2	4/0/0
1102		P04843, RPN1, Dolichyl-diphosphooligosaccharide--protein glycosyltransferase subunit 1	5/0/0
1103	(1)	P46783, RPS10, 40S ribosomal protein S10	3/0/1
	(2)	Q9NQ39, RPS10P5, Putative 40S ribosomal protein S10-like	
1104		P62280, RPS11, 40S ribosomal protein S11	2/0/0
1105		P62277, RPS13, 40S ribosomal protein S13	4/0/0
1106		P62263, RPS14, 40S ribosomal protein S14	4/0/0
1107		P62249, RPS16, 40S ribosomal protein S16	2/0/0
1108		P62269, RPS18, 40S ribosomal protein S18	3/0/0
1109		P39019, RPS19, 40S ribosomal protein S19	3/0/0
1110		P60866-1,2, RPS20, 40S ribosomal protein S20	3/0/0
1111		P63220, RPS21, 40S ribosomal protein S21	2/0/0
1112	(1)	P62979, RPS27A, Ubiquitin-40S ribosomal protein S27a	1/0/5
	(2)	P0CG47, UBB, Polyubiquitin-B [Cleaved into: Ubiquitin]	
	·	P0CG48, UBC, Polyubiquitin-C [Cleaved into: Ubiquitin]	
1113		P23396-1,2, RPS3, 40S ribosomal protein S3	3/0/0
1114		P61247, RPS3A, 40S ribosomal protein S3a	6/0/0
1115	(1)	P62701, RPS4X, 40S ribosomal protein S4, X isoform	1/0/0

	(2)	P22090, RPS4Y1, 40S ribosomal protein S4, Y isoform 1	
	·	Q8TD47, RPS4Y2, 40S ribosomal protein S4, Y isoform 2	
1116		P62753, RPS6, 40S ribosomal protein S6	7/0/0
1117		P62081, RPS7, 40S ribosomal protein S7	2/0/0
1118		P62241, RPS8, 40S ribosomal protein S8	2/0/0
1119		P46781, RPS9, 40S ribosomal protein S9	3/0/0
1120		P08865, RPSA, 40S ribosomal protein SA	6/0/0
1121	(1)	Q9P2E9-1,2, RRBP1, Ribosome-binding protein 1	2/0/0
	(2)	Q9P2E9-3, RRBP1, Ribosome-binding protein 1	
1122		Q9Y3I0, RTCB, tRNA-splicing ligase RtcB homolog	2/0/0
1123		Q9NQC3-1,2,5, RTN4, Reticulon-4	1/0/0
1124		Q15413-1,2, RYR3, Ryanodine receptor 3	4/0/0
1125		P60903, S100A10, Protein S100-A10	6/0/0
1126		P31949, S100A11, Protein S100-A11	3/0/0
1127		P26447, S100A4, Protein S100-A4	3/0/0
1128		P06703, S100A6, Protein S100-A6	4/0/0
1129		P05109, S100A8, Protein S100-A8	8/0/0
1130		P06702, S100A9, Protein S100-A9	5/0/0
1131	(1)	P0DJI8, SAA1, Serum amyloid A-1 protein	3/0/5
	(2)	P0DJI9-2, SAA2, Serum amyloid A-2 protein	
1132		P0DJI9-1, SAA2, Serum amyloid A-2 protein	3/0/5
1133		P35542, SAA4, Serum amyloid A-4 protein	7/0/0
1134		Q15424-1-4, SAFB, Scaffold attachment factor B1	6/0/0
1135	(1)	Q9Y3Z3-1, SAMHD1, Deoxynucleoside triphosphate triphosphohydrolase SAMHD1	0/8/0
	(2)	Q9Y3Z3-2, SAMHD1, Deoxynucleoside triphosphate triphosphohydrolase SAMHD1	
	(3)	Q9Y3Z3-3, SAMHD1, Deoxynucleoside triphosphate triphosphohydrolase SAMHD1	
	(4)	Q9Y3Z3-4, SAMHD1, Deoxynucleoside triphosphate triphosphohydrolase SAMHD1	
1136		P49591, SARS, Serine--tRNA ligase, cytoplasmic	8/0/0
1137		Q6UWP8-1, SBSN, Suprabasin	4/0/0
1138		P13521, SCG2, Secretogranin-2	29/0/1

1139	(1)	Q8WXD2-1, SCG3, Secretogranin-3	3/0/0
	(2)	Q8WXD2-2, SCG3, Secretogranin-3	
1140		P05408-1, SCG5, Neuroendocrine protein 7B2	1/0/1
1141		P05408-2, SCG5, Neuroendocrine protein 7B2	1/0/1
1142		P11684, SCGB1A1, Uteroglobin	3/0/0
1143		Q9HB40-1, SCPEP1, Retinoid-inducible serine carboxypeptidase	3/0/0
1144	(1)	Q12765-1,2, SCRN1, Secernin-1	1/0/0
	(2)	Q12765-3, SCRN1, Secernin-1	
1145	(1)	Q9BRK5-1, SDF4, 45 kDa calcium-binding protein	1/10/0
	(2)	Q9BRK5-2, SDF4, 45 kDa calcium-binding protein	
	(3)	Q9BRK5-5, SDF4, 45 kDa calcium-binding protein	
	(4)	Q9BRK5-6, SDF4, 45 kDa calcium-binding protein	
	(5)	Q9BRK5-3,4, SDF4, 45 kDa calcium-binding protein	
1146		O95810, SDPR, Serum deprivation-response protein	3/0/0
1147	(1)	Q9Y6Y8-1, SEC23IP, SEC23-interacting protein	1/0/0
	(2)	Q9Y6Y8-2, SEC23IP, SEC23-interacting protein	
1148	(1)	O94979-7, SEC31A, Protein transport protein Sec31A	1/0/0
	(2)	O94979-1-4,6,8-10, SEC31A, Protein transport protein Sec31A	
1149		Q99985-1, SEMA3C, Semaphorin-3C	2/0/0
1150		Q13275-1,2, SEMA3F, Semaphorin-3F	3/0/0
1151	(1)	P04279-1, SEMG1, Semenogelin-1	1/0/3
	(2)	P04279-2, SEMG1, Semenogelin-1	
1152		Q02383, SEMG2, Semenogelin-2	6/0/3
1153		P49908, SEPP1, Selenoprotein P	6/0/0
1154	(1)	Q15019-1,2, SEPT2, Septin-2	1/0/0
	(2)	Q15019-3, SEPT2, Septin-2	
1155		Q16181-1,2, SEPT7, Septin-7	6/0/0
1156	(1)	Q9UHD8-1,2,5,7, SEPT9, Septin-9	2/0/0
	(2)	Q9UHD8-3,4,8,9, SEPT9, Septin-9	
1157		Q8NC51-1-4, SERBP1, Plasminogen activator inhibitor 1 RNA-binding protein	1/0/0

3/18/0

1158 (1) P01009-1, SERPINA1, Alpha-1-antitrypsin

(2) P01009-2, SERPINA1, Alpha-1-antitrypsin

(3) P01009-3, SERPINA1, Alpha-1-antitrypsin

1159 Q9UK55, SERPINA10, Protein Z-dependent protease inhibitor 13/0/0

1160 Q86U17, SERPINA11, Serpin A11 1/0/0

6/0/0

1161 (1) P01011-1, SERPINA3, Alpha-1-antichymotrypsin

(2) P01011-2, SERPINA3, Alpha-1-antichymotrypsin

1162 P29622, SERPINA4, Kallistatin 11/0/0

1163 P05154, SERPINA5, Plasma serine protease inhibitor 10/0/0

1164 P08185, SERPINA6, Corticosteroid-binding globulin 2/0/0

1165 P05543, SERPINA7, Thyroxine-binding globulin 8/0/0

1166 P30740-1,2, SERPINB1, Leukocyte elastase inhibitor 2/0/0

1167 P05120, SERPINB2, Plasminogen activator inhibitor 2 2/0/0

1168 P01008, SERPINC1, Antithrombin-III 21/0/0

1169 P05546, SERPIND1, Heparin cofactor 2 20/0/0

1170 P05121-1,2, SERPINE1, Plasminogen activator inhibitor 1 6/0/0

1171 P07093-1-3, SERPINE2, Glia-derived nexin 4/0/0

1172 P36955, SERPINF1, Pigment epithelium-derived factor 15/0/0

3/0/0

1173 (1) P08697-1, SERPINF2, Alpha-2-antiplasmin

(2) P08697-2, SERPINF2, Alpha-2-antiplasmin

1174 P05155-1-3, SERPING1, Plasma protease C1 inhibitor 6/0/0

1175 P50454, SERPINH1, Serpin H1 5/0/0

1176 P31947-1,2, SFN, 14-3-3 protein sigma 3/0/4

2/0/2

1177 (1) P23246-1, SFPQ, Splicing factor, proline- and glutamine-rich

(2) P23246-2, SFPQ, Splicing factor, proline- and glutamine-rich

1178 Q8N474, SFRP1, Secreted frizzled-related protein 1 2/0/0

1179 Q96HF1, SFRP2, Secreted frizzled-related protein 2 11/0/0

1180 Q6FHJ7, SFRP4, Secreted frizzled-related protein 4 6/0/0

1181 O43765, SGTA, Small glutamine-rich tetratricopeptide repeat-containing protein alpha 4/0/0

1182 Q9H299, SH3BGRL3, SH3 domain-binding glutamic acid-rich-like protein 3 2/0/0

1183		Q99961-1-3, SH3GL1, Endophilin-A2	2/0/0
1184	(1)	P04278-1-4, SHBG, Sex hormone-binding globulin	2/0/0
	(2)	P04278-5, SHBG, Sex hormone-binding globulin	
1185	(1)	P63208-1, SKP1, S-phase kinase-associated protein 1	2/0/1
	(2)	P63208-2, SKP1, S-phase kinase-associated protein 1	
1186	(1)	P53985-1, SLC16A1, Monocarboxylate transporter 1	1/0/0
	(2)	P53985-2, SLC16A1, Monocarboxylate transporter 1	
1187		Q00325-1,2, SLC25A3, Phosphate carrier protein, mitochondrial	1/0/0
1188		P05141, SLC25A5, ADP/ATP translocase 2	2/0/0
		P12235, SLC25A4, ADP/ATP translocase 1	
		P12236, SLC25A6, ADP/ATP translocase 3	
1189	(1)	O14745-1, SLC9A3R1, Na(+)/H(+) exchange regulatory cofactor NHE-RF1	2/0/1
	(2)	O14745-2, SLC9A3R1, Na(+)/H(+) exchange regulatory cofactor NHE-RF1	
1190	(1)	O75094-1,2,4, SLIT3, Slit homolog 3 protein	2/0/0
	(2)	O75094-3, SLIT3, Slit homolog 3 protein	
1191		Q9H2G2-1,2, SLK, STE20-like serine/threonine-protein kinase	3/0/0
1192		P03973, SLPI, Antileukoproteinase	14/0/0
1193		P55000, SLURP1, Secreted Ly-6/uPAR-related protein 1	2/0/0
1194		Q8TAQ2-1-3, SMARCC2, SWI/SNF complex subunit SMARCC2	1/0/0
1195		Q9H3U7-1,2, SMOC2, SPARC-related modular calcium-binding protein 2	2/0/0
1196		Q7KZF4, SND1, Staphylococcal nuclease domain-containing protein 1	5/0/0
1197		Q8TER0-1,3-5, SNED1, Sushi, nidogen and EGF-like domain-containing protein 1	8/0/0
1198		P08621-1,2, SNRNP70, U1 small nuclear ribonucleoprotein 70 kDa	2/0/0
1199		P62316-1, SNRPD2, Small nuclear ribonucleoprotein Sm D2	2/0/0
1200		P62306, SNRPF, Small nuclear ribonucleoprotein F	1/0/0
1201		O60493-1,2,4, SNX3, Sorting nexin-3	2/0/0
1202		Q9Y5X3-1, SNX5, Sorting nexin-5	2/0/0
1203	(1)	Q9UNH7-1, SNX6, Sorting nexin-6	1/0/0
	(2)	Q9UNH7-2, SNX6, Sorting nexin-6	

1204 O14512-1,2, SOCS7, Suppressor of cytokine signaling 7 — 3/0/0

1205 P04179-1,2,4, SOD2, Superoxide dismutase [Mn], mitochondrial — 2/0/0

1206 P08294, SOD3, Extracellular superoxide dismutase [Cu-Zn] — 10/0/0

1207 Q99523-1, SORT1, Sortilin — 1/0/0

1208 Q07889-1, SOS1, Son of sevenless homolog 1 — 2/0/0

1209 P09486, SPARC, SPARC — 15/0/0

1210 — 6/0/0
(1) Q14515-1, SPARCL1, SPARC-like protein 1
(2) Q14515-2, SPARCL1, SPARC-like protein 1

1211 Q15005, SPCS2, Signal peptidase complex subunit 2 — 2/0/0

1212 Q9HCB6, SPON1, Spondin-1 — 23/0/0

1213 P10451-1-5, SPP1, Osteopontin — 3/0/0

1214 Q13103, SPP2, Secreted phosphoprotein 24 — 9/0/0

1215 Q13813-1-3, SPTAN1, Spectrin alpha chain, non-erythrocytic 1 — 10/0/0

1216 Q01082-1-3, SPTBN1, Spectrin beta chain, non-erythrocytic 1 — 12/0/0

1217 P30626-1-3, SRI, Sorcin — 3/0/0

1218 — 0/8/0
(1) P78539-1,4, SRPX, Sushi repeat-containing protein SRPX
(2) P78539-3, SRPX, Sushi repeat-containing protein SRPX
(3) P78539-2,5, SRPX, Sushi repeat-containing protein SRPX

1219 Q8IYB3-1,2, SRRM1, Serine/arginine repetitive matrix protein 1 — 2/0/0

1220 — 1/0/0
(1) Q07955-1, SRSF1, Serine/arginine-rich splicing factor 1
(2) Q07955-2,3, SRSF1, Serine/arginine-rich splicing factor 1

1221 P84103-1,2, SRSF3, Serine/arginine-rich splicing factor 3 — 4/0/1

1222 Q16629-1-4, SRSF7, Serine/arginine-rich splicing factor 7 — 3/0/1

1223 A1L4H1-1,2, SSC5D, Soluble scavenger receptor cysteine-rich domain-containing protein SSC5D — 6/0/0

1224 — 1/0/5
(1) P50502, ST13, Hsc70-interacting protein
(2) Q8NFI4, ST13P5, Putative protein FAM10A5

1225 Q8IZP2, ST13P4, Putative protein FAM10A4 — 1/0/5

1226 P15907-1, ST6GAL1, Beta-galactoside alpha-2,6-sialyltransferase 1 — 1/0/0

1227 — 10/0/0
(1) Q9NY15-1, STAB1, Stabilin-1
(2) Q9NY15-2, STAB1, Stabilin-1

1228 P42229-1,2, STAT5A, Signal transducer and activator of transcription 5A — 2/0/0
P51692, STAT5B, Signal transducer and activator of transcription 5B

1229 P31948-1-3, STIP1, Stress-induced-phosphoprotein 1 — 3/0/0

1230 Q15833-1-3, STXBP2, Syntaxin-binding protein 2 — 2/0/0

1231 P53999, SUB1, Activated RNA polymerase II transcriptional coactivator p15 — 6/0/0

1232 Q8IWU6, SULF1, Extracellular sulfatase Sulf-1 — 11/0/0

1233 (1) P0DMM9-1,3, SULT1A3, Sulfotransferase 1A3 — 1/0/0
· P0DMN0, SULT1A4, Sulfotransferase 1A4
· P50225-1,2, SULT1A1, Sulfotransferase 1A1
(2) P50226, SULT1A2, Sulfotransferase 1A2

1234 O60279, SUSD5, Sushi domain-containing protein 5 — 4/0/0

1235 (1) Q4LDE5-1, SVEP1, Sushi, von Willebrand factor type A, EGF and pentraxin domain-containing protein 1 — 7/24/0
(2) Q4LDE5-2, SVEP1, Sushi, von Willebrand factor type A, EGF and pentraxin domain-containing protein 1
(3) Q4LDE5-3, SVEP1, Sushi, von Willebrand factor type A, EGF and pentraxin domain-containing protein 1
(4) Q4LDE5-4, SVEP1, Sushi, von Willebrand factor type A, EGF and pentraxin domain-containing protein 1

1236 (1) O60506-3,4, SYNCRIP, Heterogeneous nuclear ribonucleoprotein Q — 0/5/0
(2) O60506-5, SYNCRIP, Heterogeneous nuclear ribonucleoprotein Q
(3) O60506-1,2, SYNCRIP, Heterogeneous nuclear ribonucleoprotein Q
(4) O43390-1-4, HNRNPR, Heterogeneous nuclear ribonucleoprotein R

1237 Q92804-1,2, TAF15, TATA-binding protein-associated factor 2N — 4/0/1

1238 Q01995, TAGLN, Transgelin — 8/0/0

1239 P37802-1,2, TAGLN2, Transgelin-2 — 12/0/0

1240 (1) Q13148-1, TARDBP, TAR DNA-binding protein 43 — 1/0/0
(2) Q13148-4, TARDBP, TAR DNA-binding protein 43

1241 Q99426-1,2, TBCB, Tubulin-folding cofactor B — 2/0/0

1242 (1) P20062-1, TCN2, Transcobalamin-2 — 1/0/0
(2) P20062-2, TCN2, Transcobalamin-2

1243 P17987, TCP1, T-complex protein 1 subunit alpha — 2/0/0

1244 B5MCY1, TDRD15, Tudor domain-containing protein 15 — 6/0/0

1245		P02787, TF, Serotransferrin	35/0/0
1246		Q92734-1-4, TFG, Protein TFG	4/0/0
1247		P10646-1, TFPI, Tissue factor pathway inhibitor	4/0/0
1248		P48307-1,2, TFPI2, Tissue factor pathway inhibitor 2	6/0/0
1249		Q15582, TGFBI, Transforming growth factor-beta-induced protein ig-h3	25/0/0
1250		Q03167-1,2, TGFBR3, Transforming growth factor beta receptor type 3	9/0/0
1251	(1)	P21980-1, TGM2, Protein-glutamine gamma-glutamyltransferase 2	3/0/1
	(2)	P21980-2, TGM2, Protein-glutamine gamma-glutamyltransferase 2	
1252		P49221, TGM4, Protein-glutamine gamma-glutamyltransferase 4	2/0/1
1253	(1)	P07996-1, THBS1, Thrombospondin-1	5/0/2
	(2)	P07996-2, THBS1, Thrombospondin-1	
1254		P35442, THBS2, Thrombospondin-2	35/0/2
1255	(1)	P49746-1, THBS3, Thrombospondin-3	2/0/1
	(2)	P49746-2, THBS3, Thrombospondin-3	
1256		P35443, THBS4, Thrombospondin-4	19/0/3
1257	(1)	Q6ZMP0-1, THSD4, Thrombospondin type-1 domain-containing protein 4	1/0/0
	(2)	Q6ZMP0-4, THSD4, Thrombospondin type-1 domain-containing protein 4	
1258		P01033, TIMP1, Metalloproteinase inhibitor 1	5/0/0
1259		P16035, TIMP2, Metalloproteinase inhibitor 2	6/0/0
1260		Q99727, TIMP4, Metalloproteinase inhibitor 4	2/0/0
1261		Q9GZM7-1,3, TINAGL1, Tubulointerstitial nephritis antigen-like	3/0/0
1262	(1)	O75663-1, TIPRL, TIP41-like protein	1/0/0
	(2)	O75663-2, TIPRL, TIP41-like protein	
1263		Q3LXA3-1,2, TKFC, Triokinase/FMN cyclase	1/0/0
1264		O43897-1, TLL1, Tolloid-like protein 1	2/0/4
1265		Q9Y490, TLN1, Talin-1	44/0/9
1266		Q9Y4G6, TLN2, Talin-2	2/0/9
1267		Q15363, TMED2, Transmembrane emp24 domain-containing protein 2	4/0/0

1268		Q9BTV4, TMEM43, Transmembrane protein 43	1/0/0
1269		Q9BXS4, TMEM59, Transmembrane protein 59	2/0/0
1270		Q9NYL9, TMOD3, Tropomodulin-3	1/0/0
1271		P62328, TMSB4X, Thymosin beta-4	2/0/0
1272	(1)	P24821-1,4, TNC, Tenascin	1/0/36
	(2)	P24821-2,3,5, TNC, Tenascin	
1273		P24821-6, TNC, Tenascin	1/0/36
1274		P98066, TNFAIP6, Tumor necrosis factor-inducible gene 6 protein	4/0/0
1275	(1)	P13805-1-3, TNNT1, Troponin T, slow skeletal muscle	3/0/2
	(2)	P45379-1,2,5-8,10,11, TNNT2, Troponin T, cardiac muscle	
1276		P45379-3,4, TNNT2, Troponin T, cardiac muscle	1/0/1
1277		P45378-1-7, TNNT3, Troponin T, fast skeletal muscle	1/0/1
1278		Q92973-1-3, TNPO1, Transportin-1	3/0/1
1279	(1)	O14787-2, TNPO2, Transportin-2	0/0/1
	(2)	O14787-1, TNPO2, Transportin-2	
1280	(1)	P22105-1,3,4, TNXB, Tenascin-X	59/4/0
	(2)	P22105-2, TNXB, Tenascin-X	
	(3)	Q16473, TNXA, Putative tenascin-XA	
1281		P60174-1,3,4, TPI1, Triosephosphate isomerase	1/0/0
1282		P09493-2, TPM1, Tropomyosin alpha-1 chain	0/0/15
1283	(1)	P09493-3, TPM1, Tropomyosin alpha-1 chain	0/0/20
	(2)	P09493-4,10, TPM1, Tropomyosin alpha-1 chain	
1284		P09493-5, TPM1, Tropomyosin alpha-1 chain	1/0/16
1285	(1)	P09493-7, TPM1, Tropomyosin alpha-1 chain	0/0/20
	(2)	P09493-6, TPM1, Tropomyosin alpha-1 chain	
1286		P09493-8, TPM1, Tropomyosin alpha-1 chain	0/0/19
1287	(1)	P09493-9, TPM1, Tropomyosin alpha-1 chain	0/0/21
	(2)	P09493-1, TPM1, Tropomyosin alpha-1 chain	
1288		P07951-1, TPM2, Tropomyosin beta chain	3/0/20
1289	(1)	P07951-2, TPM2, Tropomyosin beta chain	0/0/19
	(2)	P07951-3, TPM2, Tropomyosin beta chain	
1290		P06753-1, TPM3, Tropomyosin alpha-3 chain	1/0/19

0/0/19

1291 (1) P06753-2, TPM3, Tropomyosin alpha-3 chain

(2) P06753-3,6, TPM3, Tropomyosin alpha-3 chain

0/0/23

1292 (1) P06753-5, TPM3, Tropomyosin alpha-3 chain

(2) P06753-4, TPM3, Tropomyosin alpha-3 chain

(3) P06753-7, TPM3, Tropomyosin alpha-3 chain

5/0/17

1293 P67936-1, TPM4, Tropomyosin alpha-4 chain

0/0/19

1294 P67936-2, TPM4, Tropomyosin alpha-4 chain

3/0/0

1295 O14773-1,2, TPP1, Tripeptidyl-peptidase 1

3/0/0

1296 P20396, TRH, Pro-thyrotropin-releasing hormone

6/0/0

1297 Q8WUA8, TSKU, Tsukushin

2/27/6

1298 (1) Q8WZ42-12, TTN, Titin

(2) Q8WZ42-4,7,11, TTN, Titin

(3) Q8WZ42-1,2,8,13, TTN, Titin

(4) Q8WZ42-3,9,10, TTN, Titin

(5) Q8WZ42-5, TTN, Titin

1/0/3

1299 Q8WZ42-6, TTN, Titin

8/0/0

1300 P02766, TTR, Transthyretin

0/0/17

1301 (1) P68363-1, TUBA1B, Tubulin alpha-1B chain

(2) Q6PEY2, TUBA3E, Tubulin alpha-3E chain

(3) Q13748-1, TUBA3C, Tubulin alpha-3C/D chain

(4) Q13748-2, TUBA3C, Tubulin alpha-3C/D chain

(5) Q71U36-1,2, TUBA1A, Tubulin alpha-1A chain

(6) P68363-2, TUBA1B, Tubulin alpha-1B chain

1/0/15

1302 Q9BQE3, TUBA1C, Tubulin alpha-1C chain

3/0/13

1303 (1) P68366-1,2, TUBA4A, Tubulin alpha-4A chain

(2) A6NHL2-1,2, TUBAL3, Tubulin alpha chain-like 3

(3) Q9NY65-1,2, TUBA8, Tubulin alpha-8 chain

2/0/12

1304 (1) P07437, TUBB, Tubulin beta chain

(2) Q13885, TUBB2A, Tubulin beta-2A chain

· Q9BVA1, TUBB2B, Tubulin beta-2B chain

(3) A6NNZ2, NA, Tubulin beta-8 chain-like protein LOC260334

(4) Q9H4B7, TUBB1, Tubulin beta-1 chain

1/0/9

1305 Q13509-1, TUBB3, Tubulin beta-3 chain

0/0/10

1306 (1) P04350, TUBB4A, Tubulin beta-4A chain

(2) Q9BUF5, TUBB6, Tubulin beta-6 chain

1/0/13

1307 (1) P68371, TUBB4B, Tubulin beta-4B chain

(2) Q13509-2, TUBB3, Tubulin beta-3 chain

	(3)	Q3ZCM7, TUBB8, Tubulin beta-8 chain	
1308	(1)	Q9GZX9-1, TWSG1, Twisted gastrulation protein homolog 1	3/0/0
	(2)	Q9GZX9-2, TWSG1, Twisted gastrulation protein homolog 1	
1309		Q8NBS9-1,2, TXNDC5, Thioredoxin domain-containing protein 5	4/0/1
1310		O43396, TXNL1, Thioredoxin-like protein 1	1/0/0
1311	(1)	P19971-1, TYMP, Thymidine phosphorylase	1/0/0
	(2)	P19971-2, TYMP, Thymidine phosphorylase	
1312		P22314-1,2, UBA1, Ubiquitin-like modifier-activating enzyme 1	12/0/0
1313		P62987, UBA52, Ubiquitin-60S ribosomal protein L40	1/0/5
1314		A0AVT1-1, UBA6, Ubiquitin-like modifier-activating enzyme 6	4/0/0
1315		P61086-1,3, UBE2K, Ubiquitin-conjugating enzyme E2 K	1/0/0
1316		P61088, UBE2N, Ubiquitin-conjugating enzyme E2 N	3/0/0
		Q5JXB2, UBE2NL, Putative ubiquitin-conjugating enzyme E2 N-like	
1317		Q13404-1,2,4,6,7, UBE2V1, Ubiquitin-conjugating enzyme E2 variant 1	2/0/0
1318		Q5T4S7-1-4, UBR4, E3 ubiquitin-protein ligase UBR4	7/0/0
1319	(1)	O60701-1, UGDH, UDP-glucose 6-dehydrogenase	0/4/0
	(2)	O60701-2, UGDH, UDP-glucose 6-dehydrogenase	
	(3)	O60701-3, UGDH, UDP-glucose 6-dehydrogenase	
1320	(1)	Q16851-2, UGP2, UTP--glucose-1-phosphate uridylyltransferase	1/0/0
	(2)	Q16851-1, UGP2, UTP--glucose-1-phosphate uridylyltransferase	
1321		P14927-1,2, UQCRB, Cytochrome b-c1 complex subunit 7	1/0/0
1322		P31930, UQCRC1, Cytochrome b-c1 complex subunit 1, mitochondrial	1/0/0
1323		P22695, UQCRC2, Cytochrome b-c1 complex subunit 2, mitochondrial	2/0/0
1324	(1)	P07919, UQCRH, Cytochrome b-c1 complex subunit 6, mitochondrial	1/0/0
	(2)	A0A096LP55, NA, NA	
1325		O60763-1,2, USO1, General vesicular transport factor p115	6/0/0
1326		P45974-1,2, USP5, Ubiquitin carboxyl-terminal hydrolase 5	4/0/0

1327		Q9P0L0-1,2, VAPA, Vesicle-associated membrane protein-associated protein A	1/0/0
1328		P50552, VASP, Vasodilator-stimulated phosphoprotein	2/0/0
1329	(1)	Q99536-1, VAT1, Synaptic vesicle membrane protein VAT-1 homolog	1/2/0
	(2)	Q99536-2, VAT1, Synaptic vesicle membrane protein VAT-1 homolog	
	(3)	Q99536-3, VAT1, Synaptic vesicle membrane protein VAT-1 homolog	
1330	(1)	P19320-1,3, VCAM1, Vascular cell adhesion protein 1	1/0/0
	(2)	P19320-2, VCAM1, Vascular cell adhesion protein 1	
1331		P13611-1-5, VCAN, Versican core protein	5/0/0
1332	(1)	P18206-1,2, VCL, Vinculin	28/0/0
	(2)	P18206-3, VCL, Vinculin	
1333		P55072, VCP, Transitional endoplasmic reticulum ATPase	11/0/0
1334		P21796, VDAC1, Voltage-dependent anion-selective channel protein 1	2/0/0
1335		P49767, VEGFC, Vascular endothelial growth factor C	15/0/0
1336		O15240, VGF, Neurosecretory protein VGF [Cleaved into: Neuroendocrine regulatory peptide-1	7/0/0
1337	(1)	P08670, VIM, Vimentin	27/0/9
	(2)	Q9NSB2, KRT84, Keratin, type II cuticular Hb4	
	(3)	P14136-1-3, GFAP, Glial fibrillary acidic protein	
1338		Q9H9C1-1,2, VIPAS39, Spermatogenesis-defective protein 39 homolog	1/0/0
1339		P98155-1,2, VLDLR, Very low-density lipoprotein receptor	9/0/0
1340		Q9UBQ0-1,2, VPS29, Vacuolar protein sorting-associated protein 29	1/0/0
1341		Q96QK1, VPS35, Vacuolar protein sorting-associated protein 35	6/0/0
1342		Q9Y279-1,2, VSIG4, V-set and immunoglobulin domain-containing protein 4	3/0/0
1343		P04004, VTN, Vitronectin	18/0/0
1344	(1)	P04275-1, VWF, von Willebrand factor	57/0/0
	(2)	P04275-2, VWF, von Willebrand factor	
1345		P23381-1,2, WARS, Tryptophan--tRNA ligase, cytoplasmic	3/0/0
1346		Q8IZQ1-1,2, WDFY3, WD repeat and FYVE domain-containing protein 3	2/0/0

1347		Q9HC57, WFDC1, WAP four-disulfide core domain protein 1	3/0/0
1348	(1)	O76076-1, WISP2, WNT1-inducible-signaling pathway protein 2	5/0/1
	(2)	O76076-2, WISP2, WNT1-inducible-signaling pathway protein 2	
1349		Q9ULE0-1,2, WWC3, Protein WWC3	1/0/1
1350		P13010, XRCC5, X-ray repair cross-complementing protein 5	2/0/0
1351		P12956-1,2, XRCC6, X-ray repair cross-complementing protein 6	5/0/0
1352		P54577, YARS, Tyrosine--tRNA ligase, cytoplasmic	2/0/0
1353		P67809, YBX1, Nuclease-sensitive element-binding protein 1	2/0/0
1354	(1)	O15498-1, YKT6, Synaptobrevin homolog YKT6	1/0/0
	(2)	O15498-2, YKT6, Synaptobrevin homolog YKT6	
1355		P31946-1,2, YWHAB, 14-3-3 protein beta/alpha	3/0/9
1356	(1)	P62258-1, YWHAE, 14-3-3 protein epsilon	3/0/3
	(2)	P62258-2, YWHAE, 14-3-3 protein epsilon	
1357		P61981, YWHAG, 14-3-3 protein gamma	9/0/6
1358		Q04917, YWHAH, 14-3-3 protein eta	7/0/5
1359		P27348, YWHAQ, 14-3-3 protein theta	10/0/6
1360	(1)	P63104-1, YWHAZ, 14-3-3 protein zeta/delta	8/0/7
	(2)	P63104-2, YWHAZ, 14-3-3 protein zeta/delta	
1361		Q9UL36-1,2, ZNF236, Zinc finger protein 236	3/0/0
1362	(1)	A2RRD8, ZNF320, Zinc finger protein 320	1/0/1
	(2)	P17035-1,2, ZNF28, Zinc finger protein 28	
1363		Q96B54, ZNF428, Zinc finger protein 428	1/0/0
1364		Q03923-1,2, ZNF85, Zinc finger protein 85	2/0/1
1365		Q15942-1,2, ZYX, Zyxin	3/0/0
1366	(1)	O43149-1, ZZEF1, Zinc finger ZZ-type and EF-hand domain-containing protein 1	1/0/0
	(2)	O43149-2, ZZEF1, Zinc finger ZZ-type and EF-hand domain-containing protein 1	

Isobaric Tag Quantification Report
P7107_Run 2 *

isobar R package v1.18.0

February 6, 2017

Channel	Class
126	*P1-treat*
127	*P1-ctrl*
128	*P2-treat*
129	*P2-ctrl*
130	*P3-treat*
131	*P3-ctrl*

Column Descriptions	
protein	Protein name, accession code, and description.
group	Protein group size, only displayed if it is not '1/1'. First number: Number of group reporters; second number: group size. See Protein Group Details for details.
peptides	Number of peptides for protein.
spectra	Number of idenfitied spectra for protein.
quant	Number of spectra with quantitative information for *ch2* versus *ch1*.
ratio	Ratio of protein in channel *ch2* relative to *ch1*.
*	Significance of protein ratio in sample.

1 Protein Relative Quantitation

Number of quantified proteins: P1-treat/P1-ctrl: 1323; P2-treat/P2-ctrl: 1323; P3-treat/P3-ctrl: 1321

1.1 Quantified Proteins

#	protein	group	peptides	spectra	ch1	ch2	quant	ratio
					127	126	3	**0.04**
**1**	125116		21	94	129	128	2	**0.22**
					131	130	3	**1.07**
					127	126	107	**0.62**
**2**	136429		7	121	129	128	106	**1.33**
					131	130	115	**1.78**
					127	126	35	**0.62**
**3**	162648		23	218	129	128	34	**1.78**
					131	130	33	**1.29**

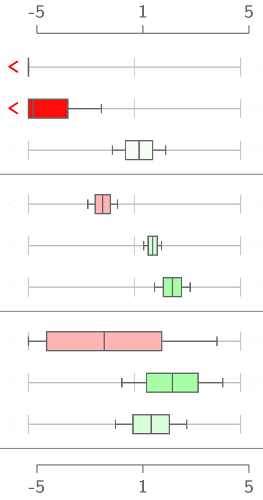

*This report was generated using the **isobar** R package version 1.18.0 [built using R 3.3.2; ; 2017-02-05 14:44:42 UTC; unix]. If you use it in published work, please cite 'Breitwieser FP *et al.*: General statistical modeling of data from protein relative expression isobaric tags, *Journal of Proteome Research* 2011' and 'Breitwieser FP and Colinge J: isobarPTM: A software tool for the quantitative analysis of post-translationally modified proteins,*Journal of Proteomics* 2013'

#	protein	group	peptides	spectra	ch1	ch2	quant	ratio
**4**	A0A075B6Q5		1	2	127	126	2	**0.65**
					129	128	2	**1.60**
					131	130	2	**1.29**
**5**	A0A0A0MS15		1	2	127	126	2	**0.70**
					129	128	2	**2.84**
					131	130	2	**1.40**
**6**	A0A0C4DH25		3	14	127	126	1	**0.81**
					129	128	1	**0.77**
					131	130	1	**1.37**
**7**	A0A0C4DH29	1/2	3	6	127	126	1	**0.84**
					129	128	1	**1.58**
					131	130	1	**2.04**
**8**	A0A0C4DH33		2	4	127	126	2	**0.87**
					129	128	2	**1.36**
					131	130	2	**0.60**
**9**	A1BG P04217$_1$: Alpha-1B-glycoprotein	1/2	12	19	127	126	16	**1.67**
					129	128	19	**1.90**
					131	130	17	**1.12**
**10**	A2M P01023: Alpha-2-macroglobulin		54	125	127	126	107	**1.27**
					129	128	105	**1.35**
					131	130	105	**1.04**
**11**	A4GALT Q9NPC4: Lactosylceramide 4-alpha-galactosyltransferase		2	3	127	126	3	**0.69**
					129	128	3	**1.11**
					131	130	3	**1.77**
**12**	AARS P49588$_{1,2}$: Alanine--tRNA ligase, cytoplasmic	2/2	8	9	127	126	9	**0.97**
					129	128	9	**0.60**
					131	130	9	**1.42**
**13**	ABHD14A Q9BUJ0: Protein ABHD14A		2	2	127	126	2	**0.52**
					129	128	2	**1.56**
					131	130	2	**1.75**
**14**	ABI3BP Q7Z7G0$_1$: Target of Nesh-SH3		2	3	127	126	3	**1.95**
					129	128	3	**0.37**
					131	130	3	**1.88**
**15**	ACAA2 P42765: 3-ketoacyl-CoA thiolase, mitochondrial		4	4	127	126	3	**0.49**
					129	128	4	**0.86**
					131	130	3	**0.06**

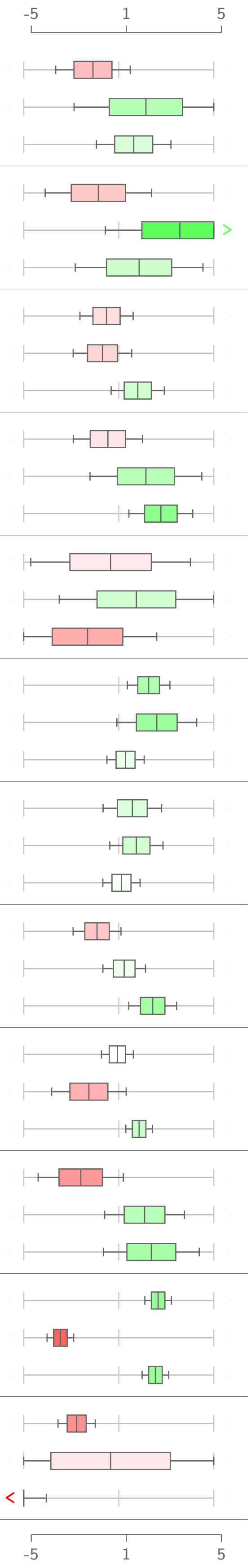

#	protein	group	peptides	spectra	ch1	ch2	quant	ratio
**16**	ACADVL P49748$_{1-3}$: Very long-chain specific acyl-CoA dehydrogenase, mitochondrial	3/3	4	5	127	126	4	**0.50**
					129	128	5	**0.41**
					131	130	4	**0.20**
**17**	ACLY P53396$_1$: ATP-citrate synthase	1/3	4	4	127	126	3	**1.05**
					129	128	4	**0.51**
					131	130	4	**0.69**
**18**	ACO2 Q99798: Aconitate hydratase, mitochondrial		5	8	127	126	7	**0.72**
					129	128	8	**1.37**
					131	130	8	**1.15**
**19**	ACTB P60709, ACTG1 P63261: Actin, cytoplasmic 1, Actin, cytoplasmic 2	2/2	20	206	127	126	50	**1.15**
					129	128	53	**0.50**
					131	130	49	**1.00**
**20**	ACTBL2 Q562R1: Beta-actin-like protein 2		9	85	127	126	4	**1.18**
					129	128	5	**1.33**
					131	130	3	**1.81**
**21**	ACTC1 P68032: Actin, alpha cardiac muscle 1	1/3	17	174	127	126	1	**0.64**
					129	128	1	**0.76**
					131	130	1	**1.31**
**22**	ACTG2 P63267$_1$: Actin, gamma-enteric smooth muscle	1/2	16	172	127	126	1	**0.82**
					129	128	1	**0.73**
					131	130	1	**0.69**
**23**	ACTN1 P12814$_{1,3}$: Alpha-actinin-1	2/4	30	37	127	126	16	**0.70**
					129	128	16	**0.32**
					131	130	15	**1.05**
**25**	ACTN3 Q08043: Alpha-actinin-3		8	10	127	126	2	**0.41**
					129	128	2	**0.64**
					131	130	2	**0.91**
**26**	ACTN4 O43707$_1$: Alpha-actinin-4	1/3	34	43	127	126	22	**0.56**
					129	128	26	**0.27**
					131	130	24	**1.02**
**27**	ACTR1A P61163: Alpha-centractin		4	20	127	126	5	**0.70**
					129	128	6	**0.33**
					131	130	5	**0.92**
**28**	ACTR2 P61160$_1$: Actin-related protein 2	1/2	6	19	127	126	9	**1.35**
					129	128	8	**0.44**
					131	130	9	**0.51**

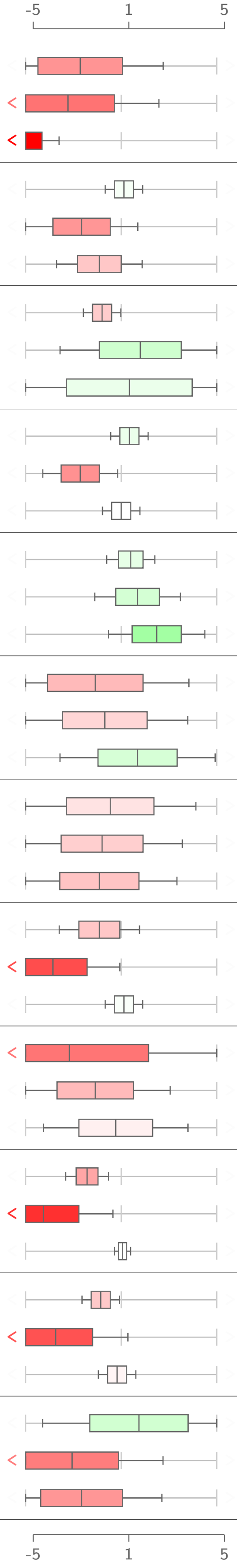

#	protein	group	peptides	spectra	ch1	ch2	quant	ratio
29	ACTR3 P61158: Actin-related protein 3		4	4	127	126	4	**1.23**
					129	128	4	**0.23**
					131	130	4	**0.67**
30	ADAM10 O14672$_{1}$: Disintegrin and metalloproteinase domain-containing protein 10		8	29	127	126	21	**0.87**
					129	128	18	**2.36**
					131	130	17	**1.33**
31	ADAM15 Q13444$_{1-10,12}$: Disintegrin and metalloproteinase domain-containing protein 15	11/13	6	6	127	126	6	**0.85**
					129	128	5	**1.14**
					131	130	6	**0.96**
32	ADAMDEC1 O15204$_{1,2}$: ADAM DEC1	2/2	6	7	127	126	6	**1.08**
					129	128	7	**1.30**
					131	130	7	**1.66**
33	ADAMTS13 Q76LX8$_{1-3}$: A disintegrin and metalloproteinase with thrombospondin motifs 13	3/3	4	4	127	126	3	**0.69**
					129	128	4	**1.66**
					131	130	3	**1.89**
34	ADAMTS15 Q8TE58: A disintegrin and metalloproteinase with thrombospondin motifs 15		2	3	127	126	3	**0.86**
					129	128	3	**1.53**
					131	130	3	**1.48**
35	ADAMTS2 O95450$_{1}$: A disintegrin and metalloproteinase with thrombospondin motifs 2	1/2	6	8	127	126	6	**0.68**
					129	128	7	**1.62**
					131	130	7	**1.48**
36	ADAMTS5 Q9UNA0: A disintegrin and metalloproteinase with thrombospondin motifs 5		2	2	127	126	2	**0.62**
					129	128	2	**2.08**
					131	130	2	**2.31**
37	ADAMTS8 Q9UP79: A disintegrin and metalloproteinase with thrombospondin motifs 8		5	11	127	126	9	**0.87**
					129	128	8	**1.64**
					131	130	8	**1.44**
38	ADAMTSL4 Q6UY14$_{1,3}$: ADAMTS-like protein 4	2/3	12	12	127	126	11	**0.59**
					129	128	11	**1.48**
					131	130	11	**1.39**
39	ADH1B P00325$_{1,2}$: Alcohol dehydrogenase 1B	2/2	3	3	127	126	1	**1.46**
					129	128	1	**1.57**
					131	130	1	**0.99**
40	ADH1C P00326: Alcohol dehydrogenase 1C	1/2	3	3	127	126	1	**0.69**
					129	128	1	**0.71**
					131	130	1	**1.81**

#	protein	group	peptides	spectra	ch1	ch2	quant	ratio
**41**	ADH5 P11766: Alcohol dehydrogenase class-3		9	10	127	126	10	**0.90**
					129	128	10	**1.06**
					131	130	10	**1.65**
**42**	ADIPOQ Q15848: Adiponectin		4	8	127	126	7	**0.42**
					129	128	7	**2.71**
					131	130	8	**0.91**
**43**	AEBP1 Q8IUX7$_{1}$: Adipocyte enhancer-binding protein 1	1/2	38	75	127	126	70	**0.41**
					129	128	69	**1.74**
					131	130	68	**1.79**
**44**	AFM P43652: Afamin		33	89	127	126	88	**0.69**
					129	128	86	**1.39**
					131	130	85	**1.09**
**45**	AGRN O00468$_{3,6}$: Agrin [Cleaved into: Agrin N-terminal 110 kDa subunit; Agrin C-terminal 110 . . .	2/7	10	11	127	126	9	**1.84**
					129	128	11	**0.55**
					131	130	10	**1.53**
**46**	AGT P01019: Angiotensinogen		12	27	127	126	26	**1.77**
					129	128	25	**0.48**
					131	130	26	**1.37**
**47**	AHCY P23526$_{1}$: Adenosylhomocysteinase	1/2	2	2	127	126	2	**0.71**
					129	128	2	**0.52**
					131	130	2	**1.00**
**48**	AHCYL1 O43865$_{1,2}$, AHCYL2 Q96HN2$_{1-4}$: Adenosylhomocysteinase 2, Adenosylhomocysteinase 3	6/6	2	2	127	126	2	**0.87**
					129	128	2	**0.63**
					131	130	2	**1.25**
**49**	AHNAK Q09666$_{1}$: Neuroblast differentiation-associated protein AHNAK	1/2	29	36	127	126	35	**0.86**
					129	128	35	**0.18**
					131	130	35	**0.51**
**50**	AHSA1 O95433$_{1}$: Activator of 90 kDa heat shock protein ATPase homolog 1	1/2	3	5	127	126	5	**1.45**
					129	128	4	**0.65**
					131	130	3	**1.45**
**51**	AHSG P02765: Alpha-2-HS-glycoprotein		12	316	127	126	304	**1.14**
					129	128	293	**1.29**
					131	130	283	**1.56**
**52**	AIFM1 O95831$_{1-3,5}$: Apoptosis-inducing factor 1, mitochondrial	4/4	1	2	127	126	1	**0.75**
					129	128	1	**0.41**
					131	130	1	**0.06**

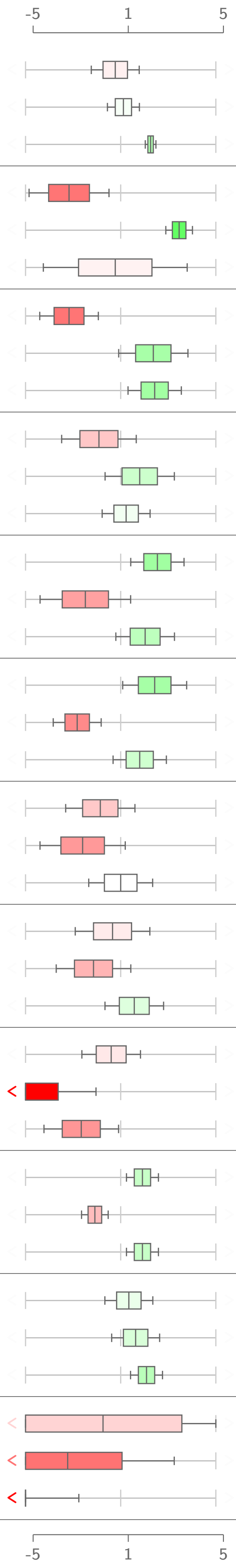

#	protein	group	peptides	spectra	ch1	ch2	quant	ratio
**53**	ALB P02768$_1$: Serum albumin	1/3	66	2631	127	126	2268	**1.48**
					129	128	2345	**1.69**
					131	130	2306	**1.63**
**54**	ALDH1A2 O94788$_1$: Retinal dehydrogenase 2	1/4	9	10	127	126	6	**1.14**
					129	128	7	**1.22**
					131	130	6	**1.49**
**55**	ALDH1A3 P47895: Aldehyde dehydrogenase family 1 member A3		8	8	127	126	4	**0.86**
					129	128	5	**0.67**
					131	130	4	**1.68**
**57**	ALDH2 P05091$_1$: Aldehyde dehydrogenase, mitochondrial	1/3	4	5	127	126	4	**1.20**
					129	128	4	**0.77**
					131	130	4	**1.11**
**58**	ALDH7A1 P49419$_{1,2}$: Alpha-aminoadipic semialdehyde dehydrogenase	2/2	2	2	127	126	2	**1.07**
					129	128	2	**0.53**
					131	130	2	**0.80**
**59**	ALDH9A1 P49189$_1$: 4-trimethylaminobutyraldehyde dehydrogenase	1/2	5	5	127	126	5	**0.89**
					129	128	3	**0.69**
					131	130	5	**0.85**
**60**	ALDOA P04075$_{1,2}$: Fructose-bisphosphate aldolase A	2/3	8	9	127	126	8	**0.95**
					129	128	9	**0.29**
					131	130	9	**0.67**
**61**	ALOX15 P16050$_{1,2}$: Arachidonate 15-lipoxygenase	2/2	3	3	127	126	3	**1.37**
					129	128	3	**0.94**
					131	130	3	**1.60**
**62**	AMBP P02760: Protein AMBP [Cleaved into: Alpha-1-microglobulin		15	189	127	126	148	**1.43**
					129	128	181	**0.82**
					131	130	141	**1.44**
**63**	ANG P03950: Angiogenin		13	79	127	126	75	**0.98**
					129	128	72	**2.38**
					131	130	73	**1.86**
**64**	ANGPTL2 Q9UKU9$_1$: Angiopoietin-related protein 2	1/2	13	17	127	126	15	**0.51**
					129	128	13	**1.31**
					131	130	16	**1.51**
**65**	ANGPTL3 Q9Y5C1: Angiopoietin-related protein 3		3	3	127	126	3	**0.85**
					129	128	3	**2.19**
					131	130	3	**1.42**

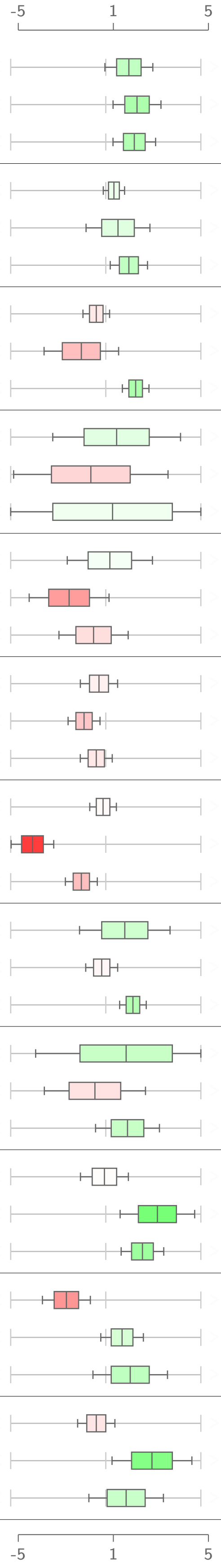

#	protein	group	peptides	spectra	ch1	ch2	quant	ratio
66	ANGPTL4 Q9BY76$_1$: Angiopoietin-related protein 4	1/3	7	13	127	126	13	**0.55**
					129	128	13	**1.85**
					131	130	13	**1.49**
67	ANTXR1 Q9H6X2$_{1,5}$: Anthrax toxin receptor 1	2/6	11	14	127	126	10	**1.06**
					129	128	11	**0.62**
					131	130	11	**1.61**
68	ANTXR2 P58335$_1$: Anthrax toxin receptor 2		12	20	127	126	3	**1.81**
					129	128	3	**0.76**
					131	130	3	**0.43**
69	ANTXR2 P58335$_4$: Anthrax toxin receptor 2	1/3	13	19	127	126	2	**1.36**
					129	128	2	**0.58**
					131	130	2	**1.09**
70	ANXA1 P04083: Annexin A1		17	29	127	126	23	**1.23**
					129	128	24	**0.08**
					131	130	26	**0.57**
71	ANXA11 P50995$_{1,2}$: Annexin A11	2/2	3	3	127	126	3	**0.87**
					129	128	3	**0.48**
					131	130	3	**0.30**
72	ANXA2 P07355$_{1,2}$: Annexin A2	2/3	21	39	127	126	33	**0.75**
					129	128	35	**0.22**
					131	130	36	**0.27**
73	ANXA4 P09525$_{1,2}$: Annexin A4	2/2	6	8	127	126	5	**0.65**
					129	128	5	**0.29**
					131	130	5	**0.25**
74	ANXA5 P08758: Annexin A5		8	11	127	126	7	**0.97**
					129	128	6	**0.53**
					131	130	8	**0.12**
75	ANXA6 P08133$_{1,2}$: Annexin A6	2/2	7	7	127	126	7	**0.79**
					129	128	6	**0.49**
					131	130	5	**0.15**
76	ANXA7 P20073$_{1,2}$: Annexin A7	2/2	2	2	127	126	2	**0.82**
					129	128	2	**0.54**
					131	130	2	**0.40**
77	AP1B1 Q10567$_{1-4}$: AP-1 complex subunit beta-1	4/7	5	6	127	126	6	**1.37**
					129	128	6	**0.49**
					131	130	5	**1.12**

#	protein	group	peptides	spectra	ch1	ch2	quant	ratio
78	AP1G1 O43747$_{1,2}$: AP-1 complex subunit gamma-1	2/2	1	1	127	126	1	**1.10**
					129	128	1	**0.34**
					131	130	1	**1.05**
79	AP2A1 O95782$_{1,2}$: AP-2 complex subunit alpha-1	2/5	4	5	127	126	4	**0.79**
					129	128	5	**0.46**
					131	130	3	**0.78**
80	APCS P02743: Serum amyloid P-component		10	219	127	126	217	**0.50**
					129	128	219	**4.17**
					131	130	218	**2.52**
81	APEH P13798: Acylamino-acid-releasing enzyme		3	3	127	126	3	**0.42**
					129	128	3	**0.26**
					131	130	3	**1.04**
82	APMAP Q9HDC9$_{1}$: Adipocyte plasma membrane-associated protein	1/2	8	8	127	126	7	**0.64**
					129	128	8	**1.17**
					131	130	7	**0.95**
83	APOA1 P02647: Apolipoprotein A-I		37	2322	127	126	2151	**0.24**
					129	128	2138	**2.03**
					131	130	2155	**1.74**
84	APOA2 P02652: Apolipoprotein A-II		6	274	127	126	253	**0.29**
					129	128	258	**1.69**
					131	130	261	**2.12**
85	APOA4 P06727: Apolipoprotein A-IV		43	1572	127	126	1392	**0.79**
					129	128	1461	**2.56**
					131	130	1437	**1.62**
86	APOA5 Q6Q788: Apolipoprotein A-V		2	2	127	126	2	**0.58**
					129	128	2	**1.07**
					131	130	2	**1.35**
87	APOB P04114: Apolipoprotein B-100		266	1596	127	126	1501	**0.68**
					129	128	1506	**0.32**
					131	130	1512	**1.42**
88	APOBR Q0VD83$_{1,3,4}$: Apolipoprotein B receptor	3/3	4	4	127	126	4	**0.83**
					129	128	3	**1.27**
					131	130	4	**1.31**
89	APOC1 P02654: Apolipoprotein C-I		6	42	127	126	37	**0.78**
					129	128	37	**1.88**
					131	130	39	**3.83**

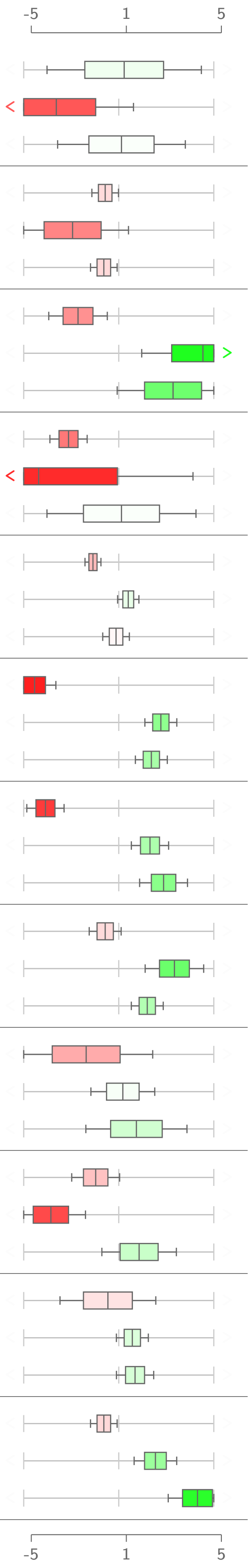

#	protein	group	peptides	spectra	ch1	ch2	quant	ratio
**90**	APOC2 P02655: Apolipoprotein C-II		4	11	127	126	10	**0.86**
					129	128	11	**2.63**
					131	130	11	**2.15**
**91**	APOC3 P02656: Apolipoprotein C-III		5	198	127	126	162	**0.77**
					129	128	178	**1.22**
					131	130	172	**1.19**
**92**	APOC4 P55056: Apolipoprotein C-IV		7	11	127	126	9	**0.66**
					129	128	9	**0.62**
					131	130	10	**1.19**
**93**	APOD P05090: Apolipoprotein D		11	191	127	126	186	**0.36**
					129	128	178	**2.42**
					131	130	181	**1.53**
**94**	APOE P02649: Apolipoprotein E		29	234	127	126	214	**0.43**
					129	128	212	**1.67**
					131	130	204	**1.56**
**95**	APOF Q13790: Apolipoprotein F		5	9	127	126	9	**0.97**
					129	128	9	**1.47**
					131	130	8	**1.37**
**96**	APOH P02749: Beta-2-glycoprotein 1		13	31	127	126	31	**1.38**
					129	128	31	**1.90**
					131	130	28	**1.18**
**97**	APOL1 $O14791_{1-3}$: Apolipoprotein L1	3/3	22	112	127	126	100	**1.58**
					129	128	106	**0.32**
					131	130	107	**1.57**
**98**	APOL2 Q9BQE5: Apolipoprotein L2		2	2	127	126	2	**1.53**
					129	128	2	**0.29**
					131	130	2	**1.74**
**99**	APOM $O95445_{1}$: Apolipoprotein M		12	40	127	126	2	**0.35**
					129	128	2	**0.71**
					131	130	2	**1.23**
**100**	APOM $O95445_{2}$: Apolipoprotein M		12	40	127	126	3	**1.05**
					129	128	3	**0.82**
					131	130	3	**2.14**
**101**	APP $P05067_{1,3-9,11}$: Amyloid beta A4 protein	9/11	9	9	127	126	8	**0.61**
					129	128	8	**1.91**
					131	130	6	**1.61**

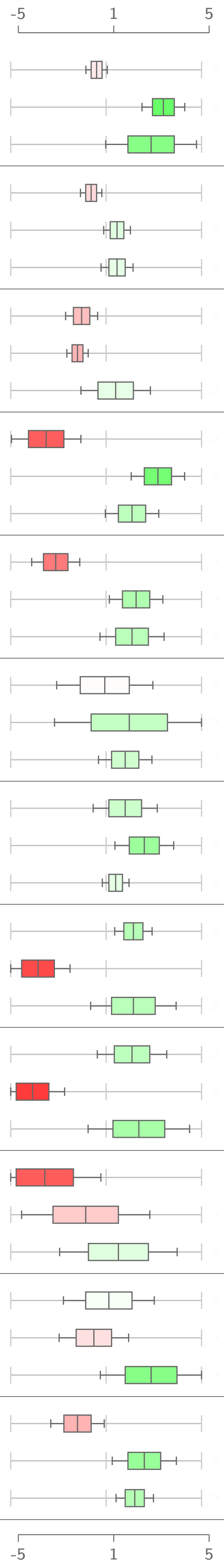

#	protein	group	peptides	spectra	ch1	ch2	quant	ratio
**102**	APRT P07741$_{1,2}$: Adenine phosphoribosyltransferase	2/2	3	3	127	126	3	**0.63**
					129	128	3	**0.86**
					131	130	3	**1.19**
**103**	ARCN1 P48444$_{1,2}$: Coatomer subunit delta	2/2	3	4	127	126	4	**1.12**
					129	128	4	**0.77**
					131	130	4	**1.16**
**104**	ARF1 P84077, ARF3 P61204$_{1,2}$: ADP-ribosylation factor 1, ADP-ribosylation factor 3	3/3	2	2	127	126	2	**1.16**
					129	128	2	**0.31**
					131	130	2	**1.50**
**105**	ARHGAP1 Q07960: Rho GTPase-activating protein 1		5	5	127	126	4	**1.85**
					129	128	5	**0.67**
					131	130	5	**1.68**
**106**	ARHGAP18 Q8N392$_{1}$: Rho GTPase-activating protein 18	1/2	4	4	127	126	3	**1.35**
					129	128	4	**0.79**
					131	130	3	**1.94**
**107**	ARHGAP35 Q9NRY4: Rho GTPase-activating protein 35		3	4	127	126	1	**0.75**
					129	128	1	**0.65**
					131	130	1	**0.67**
**108**	ARHGDIA P52565$_{1}$: Rho GDP-dissociation inhibitor 1	1/2	9	10	127	126	8	**1.24**
					129	128	8	**0.40**
					131	130	10	**1.74**
**109**	ARHGDIB P52566: Rho GDP-dissociation inhibitor 2		3	5	127	126	4	**1.27**
					129	128	5	**0.60**
					131	130	5	**0.80**
**110**	ARL3 P36405: ADP-ribosylation factor-like protein 3		2	2	127	126	2	**0.82**
					129	128	2	**0.75**
					131	130	2	**1.16**
**111**	ARPC5 O15511$_{1,2}$: Actin-related protein 2/3 complex subunit 5	2/2	2	2	127	126	2	**0.78**
					129	128	2	**0.24**
					131	130	2	**0.60**
**112**	ASAH1 Q13510$_{1-3}$: Acid ceramidase	3/3	3	3	127	126	3	**1.43**
					129	128	3	**0.83**
					131	130	3	**0.69**
**113**	ASGR2 P07307$_{1-3}$: Asialoglycoprotein receptor 2	3/3	5	5	127	126	5	**1.77**
					129	128	3	**1.72**
					131	130	4	**1.09**

#	protein	group	peptides	spectra	ch1	ch2	quant	ratio
**114**	ASMTL O95671$_{1-3}$: N-acetylserotonin O-methyltransferase-like protein	3/3	1	1	127	126	1	**1.04**
					129	128	1	**0.80**
					131	130	1	**1.14**
**115**	ASPH Q12797$_{1,10}$: Aspartyl/asparaginyl beta-hydroxylase	2/2	1	1	127	126	1	**0.81**
					129	128	1	**1.01**
					131	130	1	**0.71**
**116**	ATG3 Q9NT62$_{1,2}$: Ubiquitin-like-conjugating enzyme ATG3	2/2	2	2	127	126	2	**0.81**
					129	128	2	**0.90**
					131	130	2	**1.55**
**117**	ATIC P31939$_{1,2}$: Bifunctional purine biosynthesis protein PURH [Cleaved into: Bifunctional pu . . .	2/2	3	3	127	126	3	**1.23**
					129	128	3	**0.85**
					131	130	3	**1.28**
**118**	ATL3 Q6DD88: Atlastin-3		3	3	127	126	3	**1.15**
					129	128	3	**0.38**
					131	130	3	**0.63**
**119**	ATP2A1 O14983$_{1}$: Sarcoplasmic/endoplasmic reticulum calcium ATPase 1		3	3	127	126	1	**1.71**
					129	128	1	**0.81**
					131	130	1	**0.60**
**120**	ATP2A2 P16615$_{1,4}$: Sarcoplasmic/endoplasmic reticulum calcium ATPase 2	2/5	4	4	127	126	2	**1.33**
					129	128	2	**0.28**
					131	130	2	**0.36**
**121**	ATP2B3 Q16720$_{1-8}$: Plasma membrane calcium-transporting ATPase 3	8/8	2	2	127	126	1	**0.21**
					129	128	1	**0.99**
					131	130	1	**0.94**
**122**	ATP2B4 P23634$_{1,6-8}$: Plasma membrane calcium-transporting ATPase 4	4/4	2	2	127	126	1	**1.02**
					129	128	1	**0.39**
					131	130	1	**0.68**
**123**	ATP5A1 P25705$_{1}$: ATP synthase subunit alpha, mitochondrial	1/3	21	31	127	126	29	**0.70**
					129	128	31	**0.36**
					131	130	29	**0.15**
**124**	ATP5B P06576: ATP synthase subunit beta, mitochondrial		10	14	127	126	14	**1.04**
					129	128	13	**0.48**
					131	130	14	**0.09**
**125**	ATP5C1 P36542$_{1,2}$: ATP synthase subunit gamma, mitochondrial	2/2	6	8	127	126	7	**1.02**
					129	128	6	**0.38**
					131	130	6	**0.18**

#	protein	group	peptides	spectra	ch1	ch2	quant	ratio
126	ATP5H O75947$_{1,2}$: ATP synthase subunit d, mitochondrial	2/2	2	2	127	126	2	0.98
					129	128	2	0.44
					131	130	2	0.06
127	ATP5I P56385: ATP synthase subunit e, mitochondrial		2	2	127	126	2	0.68
					129	128	2	0.07
					131	130	2	0.05
128	ATP6AP1 Q15904: V-type proton ATPase subunit S1		2	2	127	126	2	0.62
					129	128	2	1.11
					131	130	2	1.18
129	ATP6AP2 O75787$_{1,2}$: Renin receptor	2/2	3	5	127	126	4	0.92
					129	128	4	1.06
					131	130	3	2.06
130	ATP6V1E1 P36543$_{1,3}$: V-type proton ATPase subunit E 1	2/4	3	3	127	126	3	0.61
					129	128	3	0.55
					131	130	3	0.76
131	AZGP1 P25311: Zinc-alpha-2-glycoprotein		7	9	127	126	7	1.29
					129	128	8	2.12
					131	130	9	0.85
132	B2M P61769: Beta-2-microglobulin [Cleaved into: Beta-2-microglobulin form pI 5.3]		5	15	127	126	15	1.37
					129	128	15	1.63
					131	130	14	0.96
133	B3GAT3 O94766$_{1,2}$: Galactosylgalactosylxylosylprotein 3-beta-glucuronosyltransferase 3	2/2	2	2	127	126	2	1.10
					129	128	2	0.78
					131	130	2	1.21
134	B3GNT2 Q9NY97$_{1,2}$: N-acetyllactosaminide beta-1,3-N-acetylglucosaminyltransferase 2	2/2	2	2	127	126	1	1.55
					129	128	1	0.62
					131	130	1	1.52
135	B4GALT1 P15291$_{1,2}$: Beta-1,4-galactosyltransferase 1	2/2	10	12	127	126	10	1.76
					129	128	12	0.73
					131	130	12	3.30
136	B4GALT4 O60513: Beta-1,4-galactosyltransferase 4		4	5	127	126	3	1.29
					129	128	5	0.68
					131	130	5	1.92
137	B4GAT1 O43505: Beta-1,4-glucuronyltransferase 1		4	4	127	126	4	1.35
					129	128	3	1.15
					131	130	3	1.54

#	protein	group	peptides	spectra	ch1	ch2	quant	ratio
**138**	BCAP31 P51572$_{1,2}$: B-cell receptor-associated protein 31	2/2	5	6	127	126	4	**0.60**
					129	128	4	**0.12**
					131	130	4	**0.23**
**139**	BCHE P06276: Cholinesterase		7	19	127	126	17	**0.69**
					129	128	16	**1.17**
					131	130	15	**1.07**
**140**	BEND3 Q5T5X7: BEN domain-containing protein 3		2	3	127	126	3	**0.61**
					129	128	3	**2.00**
					131	130	3	**1.62**
**141**	BGN P21810: Biglycan		7	12	127	126	6	**0.71**
					129	128	8	**0.46**
					131	130	7	**2.68**
**142**	BID P55957$_{1,2}$: BH3-interacting domain death agonist	2/2	1	2	127	126	1	**3.97**
					129	128	2	**0.37**
					131	130	1	**1.69**
**143**	BLVRB P30043: Flavin reductase		3	6	127	126	4	**0.76**
					129	128	5	**0.99**
					131	130	5	**1.43**
**144**	BMP1 P13497$_{1}$: Bone morphogenetic protein 1	1/7	18	23	127	126	18	**1.07**
					129	128	18	**1.87**
					131	130	16	**0.70**
**145**	BMP3 P12645: Bone morphogenetic protein 3		3	4	127	126	4	**0.93**
					129	128	4	**0.90**
					131	130	3	**0.98**
**146**	BOLA2 Q9H3K6$_{1,2}$: BolA-like protein 2	2/2	1	2	127	126	2	**1.25**
					129	128	2	**0.97**
					131	130	2	**0.45**
**147**	BPIFB1 Q8TDL5$_{1}$: BPI fold-containing family B member 1	1/2	6	6	127	126	6	**1.15**
					129	128	6	**0.73**
					131	130	5	**1.17**
**148**	BPTF Q12830$_{1,2,4}$: Nucleosome-remodeling factor subunit BPTF	3/3	7	14	127	126	6	**0.72**
					129	128	10	**0.90**
					131	130	8	**1.00**
**149**	BTD P43251$_{1-4}$: Biotinidase	4/4	4	6	127	126	6	**0.43**
					129	128	5	**2.75**
					131	130	6	**1.90**

#	protein	group	peptides	spectra	ch1	ch2	quant	ratio
**150**	BZW1 Q7L1Q6$_{1-4}$: Basic leucine zipper and W2 domain-containing protein 1	4/4	2	2	127	126	2	**1.10**
					129	128	2	**0.79**
					131	130	2	**1.24**
**151**	C16orf89 Q6UX73$_{1,2}$: UPF0764 protein C16orf89	2/2	4	4	127	126	3	**0.87**
					129	128	3	**0.74**
					131	130	3	**1.77**
**152**	C1QA P02745: Complement C1q subcomponent subunit A		2	13	127	126	12	**0.36**
					129	128	13	**2.66**
					131	130	13	**2.20**
**153**	C1QB P02746: Complement C1q subcomponent subunit B		8	85	127	126	85	**0.48**
					129	128	85	**2.32**
					131	130	84	**1.82**
**154**	C1QBP Q07021: Complement component 1 Q subcomponent-binding protein, mitochondrial		1	2	127	126	2	**0.51**
					129	128	2	**0.78**
					131	130	2	**0.18**
**155**	C1QC P02747: Complement C1q subcomponent subunit C		5	14	127	126	13	**0.40**
					129	128	14	**2.73**
					131	130	14	**2.41**
**156**	C1QTNF4 Q9BXJ3: Complement C1q tumor necrosis factor-related protein 4		3	3	127	126	3	**0.48**
					129	128	3	**1.36**
					131	130	3	**1.33**
**157**	C1QTNF5 Q9BXJ0: Complement C1q tumor necrosis factor-related protein 5		2	2	127	126	2	**0.26**
					129	128	2	**1.68**
					131	130	2	**1.57**
**158**	C1R P00736: Complement C1r subcomponent		34	123	127	126	104	**0.45**
					129	128	115	**2.10**
					131	130	109	**2.06**
**159**	C1RL Q9NZP8: Complement C1r subcomponent-like protein		11	17	127	126	11	**1.60**
					129	128	11	**0.76**
					131	130	11	**2.01**
**160**	C1S P09871: Complement C1s subcomponent		25	121	127	126	98	**0.48**
					129	128	104	**2.36**
					131	130	98	**1.86**
**161**	C2 P06681$_{1}$: Complement C2	1/3	29	41	127	126	35	**1.93**
					129	128	38	**0.97**
					131	130	37	**1.75**

#	protein	group	peptides	spectra	ch1	ch2	quant	ratio
162	C2orf40 Q9H1Z8: Augurin		1	1	127	126	1	**0.50**
					129	128	1	**1.95**
					131	130	1	**1.24**
163	C3 P01024: Complement C3		135	2783	127	126	2512	**1.65**
					129	128	2418	**0.96**
					131	130	2559	**1.38**
164	C4A P0C0L4$_1$: Complement C4-A	1/2	88	1839	127	126	6	**0.72**
					129	128	5	**1.87**
					131	130	6	**1.58**
165	C4B P0C0L5: Complement C4-B		90	1937	127	126	97	**0.54**
					129	128	96	**1.68**
					131	130	98	**1.89**
166	C4BPA P04003: C4b-binding protein alpha chain		34	551	127	126	528	**0.40**
					129	128	520	**1.96**
					131	130	525	**2.02**
167	C4BPB P20851$_{1,2}$: C4b-binding protein beta chain	2/2	12	73	127	126	65	**0.45**
					129	128	65	**1.62**
					131	130	65	**1.75**
168	C5 P01031: Complement C5		77	245	127	126	232	**1.82**
					129	128	222	**1.00**
					131	130	220	**1.36**
169	C6 P13671: Complement component C6		46	110	127	126	96	**2.03**
					129	128	96	**1.22**
					131	130	86	**0.86**
170	C7 P10643: Complement component C7		36	103	127	126	90	**1.18**
					129	128	95	**1.01**
					131	130	86	**1.21**
171	C8A P07357: Complement component C8 alpha chain		24	72	127	126	63	**1.76**
					129	128	67	**1.35**
					131	130	57	**1.65**
172	C8B P07358: Complement component C8 beta chain		24	112	127	126	106	**2.03**
					129	128	104	**1.01**
					131	130	107	**1.61**
173	C8G P07360: Complement component C8 gamma chain		12	30	127	126	29	**2.06**
					129	128	27	**1.25**
					131	130	29	**1.44**

#	protein	group	peptides	spectra	ch1	ch2	quant	ratio
**174**	C9 P02748: Complement component C9 [Cleaved into: Complement component C9a; Complement . . .		30	600	127	126	483	**3.96**
					129	128	490	**0.66**
					131	130	499	**1.40**
**175**	CACNA1D $Q01668_2$: Voltage-dependent L-type calcium channel subunit alpha-1D	1/4	6	122	127	126	93	**0.80**
					129	128	100	**0.88**
					131	130	95	**0.84**
**176**	CALD1 $Q05682_{1,2,6}$: Caldesmon	3/6	5	5	127	126	3	**0.38**
					129	128	4	**0.56**
					131	130	3	**1.01**
**177**	CALM1 P62158: Calmodulin		10	23	127	126	14	**0.45**
					129	128	16	**1.51**
					131	130	16	**1.32**
**178**	CALML3 P27482: Calmodulin-like protein 3		9	23	127	126	16	**0.47**
					129	128	16	**0.36**
					131	130	15	**1.31**
**179**	CALML5 Q9NZT1: Calmodulin-like protein 5		2	2	127	126	2	**0.40**
					129	128	2	**0.74**
					131	130	2	**1.50**
**180**	CALR P27797: Calreticulin		21	45	127	126	40	**0.52**
					129	128	42	**2.31**
					131	130	37	**0.92**
**181**	CALU $O43852_{1,3}$: Calumenin	2/15	12	12	127	126	9	**0.62**
					129	128	9	**1.45**
					131	130	9	**0.80**
**182**	CAMK2D $Q13557_{1,3-6,8-12}$: Calcium/calmodulin-dependent protein kinase type II subunit delta	10/10	2	2	127	126	1	**1.64**
					129	128	1	**0.62**
					131	130	1	**0.80**
**183**	CAMP P49913: Cathelicidin antimicrobial peptide		2	2	127	126	2	**0.52**
					129	128	2	**0.79**
					131	130	2	**1.22**
**184**	CAND1 $Q86VP6_1$: Cullin-associated NEDD8-dissociated protein 1	1/3	13	15	127	126	10	**1.19**
					129	128	10	**0.49**
					131	130	10	**1.27**
**185**	CANX $P27824_{1,2}$: Calnexin	2/3	15	21	127	126	17	**0.74**
					129	128	15	**0.81**
					131	130	16	**0.39**

#	protein	group	peptides	spectra	ch1	ch2	quant	ratio
186	CAP1 Q01518$_{1,2}$: Adenylyl cyclase-associated protein 1	2/3	10	12	127	126	12	**1.41**
					129	128	12	**0.35**
					131	130	10	**1.46**
187	CAPG P40121$_{1,2}$: Macrophage-capping protein	2/2	3	4	127	126	4	**2.45**
					129	128	4	**0.53**
					131	130	4	**1.53**
188	CAPN1 P07384: Calpain-1 catalytic subunit		4	6	127	126	3	**1.58**
					129	128	3	**0.39**
					131	130	3	**1.40**
189	CAPN2 P17655$_{1}$: Calpain-2 catalytic subunit	1/4	13	15	127	126	12	**0.87**
					129	128	12	**0.97**
					131	130	11	**1.16**
190	CAPNS1 P04632: Calpain small subunit 1		2	3	127	126	3	**0.89**
					129	128	3	**0.88**
					131	130	3	**1.29**
191	CAPZA1 P52907: F-actin-capping protein subunit alpha-1		6	9	127	126	3	**1.01**
					129	128	3	**0.49**
					131	130	4	**0.87**
192	CAPZA2 P47755$_{1}$: F-actin-capping protein subunit alpha-2	1/2	4	7	127	126	2	**0.51**
					129	128	2	**1.09**
					131	130	2	**1.31**
193	CAPZB P47756$_{2}$: F-actin-capping protein subunit beta	1/2	5	5	127	126	5	**0.73**
					129	128	5	**0.63**
					131	130	5	**1.26**
194	CASP14 P31944: Caspase-14		4	4	127	126	4	**0.38**
					129	128	4	**0.19**
					131	130	4	**0.67**
195	CAV1 Q03135$_{1,2}$: Caveolin-1	2/2	3	6	127	126	6	**1.19**
					129	128	6	**0.60**
					131	130	6	**0.12**
196	CBR1 P16152$_{1}$: Carbonyl reductase [NADPH] 1	1/3	2	2	127	126	2	**1.04**
					129	128	2	**0.60**
					131	130	2	**1.56**
197	CCDC146 Q8IYE0$_{1}$: Coiled-coil domain-containing protein 146		3	6	127	126		
					129	128	2	**0.64**
					131	130		

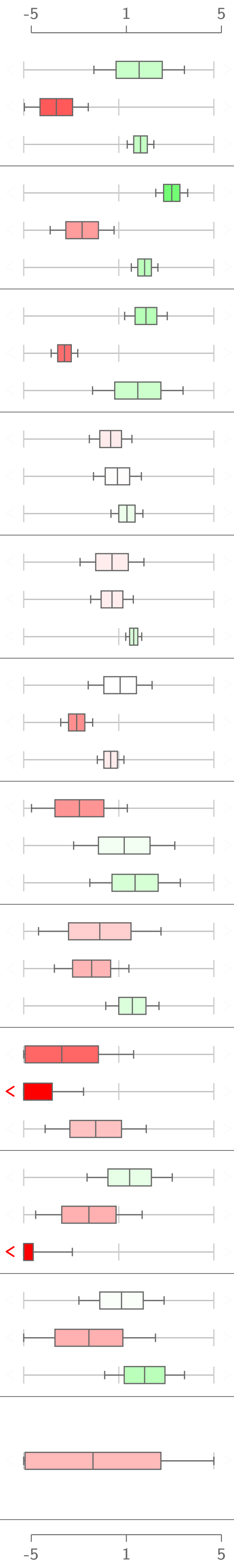

#	protein	group	peptides	spectra	ch1	ch2	quant	ratio
**198**	CCDC80 Q76M96$_{1,2}$: Coiled-coil domain-containing protein 80	2/2	19	30	127	126	30	**0.77**
					129	128	27	**1.37**
					131	130	28	**1.95**
**199**	CCL14 Q16627$_{1}$: C-C motif chemokine 14	1/2	4	4	127	126	4	**2.21**
					129	128	4	**1.51**
					131	130	4	**1.33**
**200**	CCL16 O15467: C-C motif chemokine 16		2	2	127	126	2	**1.43**
					129	128	2	**1.12**
					131	130	2	**1.22**
**201**	CCL18 P55774: C-C motif chemokine 18		2	2	127	126	2	**1.56**
					129	128	2	**3.29**
					131	130	2	**3.46**
**202**	CCS O14618: Copper chaperone for superoxide dismutase		1	1	127	126	1	**0.95**
					129	128	1	**0.67**
					131	130	1	**1.11**
**203**	CCT2 P78371$_{1,2}$: T-complex protein 1 subunit beta	2/2	6	6	127	126	5	**0.88**
					129	128	6	**0.52**
					131	130	6	**1.25**
**204**	CCT3 P49368$_{1,2}$: T-complex protein 1 subunit gamma	2/2	7	7	127	126	7	**0.86**
					129	128	7	**0.78**
					131	130	6	**1.08**
**205**	CCT4 P50991$_{1}$: T-complex protein 1 subunit delta	1/2	4	4	127	126	4	**0.66**
					129	128	4	**0.57**
					131	130	3	**1.22**
**206**	CCT5 P48643$_{1}$: T-complex protein 1 subunit epsilon	1/2	6	10	127	126	10	**0.99**
					129	128	10	**0.61**
					131	130	8	**1.02**
**207**	CCT6A P40227$_{1,2}$: T-complex protein 1 subunit zeta	2/2	3	3	127	126	3	**0.61**
					129	128	3	**0.55**
					131	130	3	**1.47**
**208**	CCT8 P50990$_{1,2}$: T-complex protein 1 subunit theta	2/3	8	8	127	126	7	**1.00**
					129	128	8	**0.54**
					131	130	7	**1.19**
**209**	CD109 Q6YHK3$_{1,4}$: CD109 antigen	2/4	12	14	127	126	13	**0.79**
					129	128	11	**0.63**
					131	130	12	**1.19**

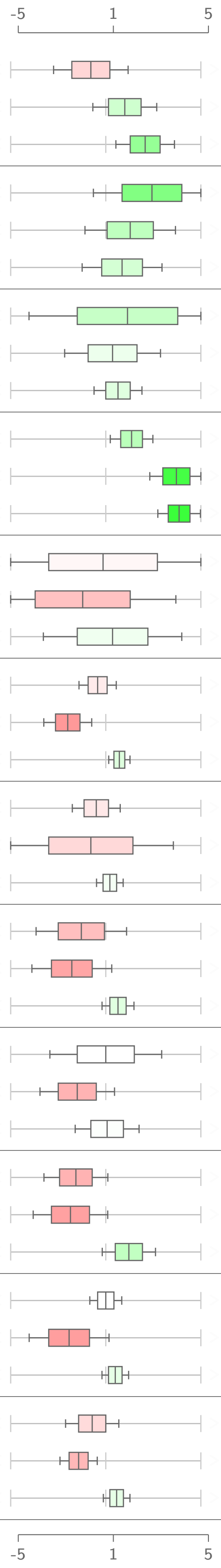

#	protein	group	peptides	spectra	ch1	ch2	quant	ratio
**210**	CD14 P08571: Monocyte differentiation antigen CD14		15	77	127	126	74	**1.78**
					129	128	73	**0.62**
					131	130	77	**1.70**
**211**	CD163 $Q86VB7_{1-4}$: Scavenger receptor cysteine-rich type 1 protein M130	4/4	5	5	127	126	5	**0.88**
					129	128	4	**1.49**
					131	130	5	**1.45**
**212**	CD248 $Q9HCU0_{1}$: Endosialin		9	12	127	126	12	**0.71**
					129	128	11	**0.98**
					131	130	12	**1.36**
**213**	CD59 P13987: CD59 glycoprotein		1	1	127	126	1	**0.69**
					129	128	1	**0.77**
					131	130	1	**0.62**
**214**	CD5L O43866: CD5 antigen-like		21	55	127	126	49	**0.47**
					129	128	51	**1.52**
					131	130	48	**1.38**
**215**	CD74 $P04233_{1,2}$: HLA class II histocompatibility antigen gamma chain	2/2	2	3	127	126	2	**1.03**
					129	128	2	**1.03**
					131	130	2	**0.67**
**216**	CD81 P60033: CD81 antigen		3	3	127	126	3	**1.76**
					129	128	3	**0.23**
					131	130	3	**0.30**
**217**	CD9 P21926: CD9 antigen		2	2	127	126	2	**1.08**
					129	128	2	**0.14**
					131	130	2	**0.28**
**218**	CD97 $P48960_{1,3}$: CD97 antigen	2/3	6	10	127	126	9	**1.15**
					129	128	9	**1.07**
					131	130	9	**1.24**
**219**	CD99 $P14209_{1,3}$: CD99 antigen	2/2	1	1	127	126	1	**0.74**
					129	128	1	**0.79**
					131	130	1	**1.43**
**220**	CDC37 Q16543: Hsp90 co-chaperone Cdc37		2	2	127	126	2	**0.87**
					129	128	2	**0.50**
					131	130	2	**0.91**
**221**	CDC42 $P60953_{1,2}$: Cell division control protein 42 homolog	2/6	4	5	127	126	4	**0.98**
					129	128	4	**0.43**
					131	130	3	**1.04**

#	protein	group	peptides	spectra	ch1	ch2	quant	ratio
**222**	CDC42BPA Q5VT25$_{1,2,4-6}$: Serine/threonine-protein kinase MRCK alpha	5/5	3	3	127	126	3	**1.04**
					129	128	3	**0.83**
					131	130	2	**1.06**
**223**	CDH1 P12830$_{1,2}$: Cadherin-1	2/2	2	5	127	126	5	**0.44**
					129	128	4	**2.66**
					131	130	5	**1.87**
**224**	CDH2 P19022$_{1}$: Cadherin-2	1/2	3	4	127	126	3	**1.32**
					129	128	4	**1.20**
					131	130	4	**1.38**
**225**	CDK17 Q00537$_{1}$: Cyclin-dependent kinase 17	1/2	4	16	127	126	12	**1.43**
					129	128	12	**0.92**
					131	130	12	**0.55**
**226**	CENPF P49454: Centromere protein F		2	2	127	126	1	**3.39**
					129	128	1	**0.72**
					131	130	1	**1.39**
**227**	CEP350 Q5VT06: Centrosome-associated protein 350		5	12	127	126	7	**0.36**
					129	128	7	**2.24**
					131	130	6	**2.37**
**228**	CES2 O00748$_{1,2}$: Cocaine esterase	2/2	2	2	127	126	2	**1.87**
					129	128	2	**0.76**
					131	130	2	**1.61**
**229**	CETP P11597$_{1}$: Cholesteryl ester transfer protein	1/2	15	59	127	126	58	**0.89**
					129	128	50	**0.73**
					131	130	59	**1.14**
**230**	CFB P00751$_{1}$: Complement factor B	1/2	25	37	127	126	35	**1.26**
					129	128	36	**1.65**
					131	130	34	**1.43**
**231**	CFD P00746: Complement factor D		16	58	127	126	57	**1.79**
					129	128	54	**0.71**
					131	130	58	**1.01**
**232**	CFH P08603$_{1}$: Complement factor H	1/2	67	570	127	126	432	**0.82**
					129	128	458	**2.74**
					131	130	443	**1.87**
**233**	CFHR1 Q03591: Complement factor H-related protein 1		13	166	127	126	7	**0.71**
					129	128	7	**4.42**
					131	130	7	**2.10**

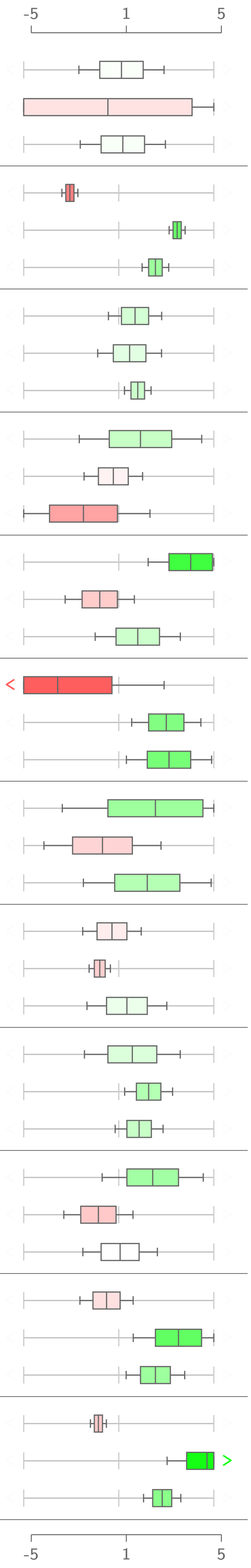

#	protein	group	peptides	spectra	ch1	ch2	quant	ratio
**234**	CFHR2 P36980$_1$: Complement factor H-related protein 2	1/2	10	70	127	126	12	**1.64**
					129	128	10	**2.22**
					131	130	10	**1.38**
**235**	CFHR3 Q02985$_{1,2}$: Complement factor H-related protein 3	2/2	8	51	127	126	10	**0.74**
					129	128	11	**1.71**
					131	130	11	**1.89**
**236**	CFHR4 Q92496$_{1,2}$: Complement factor H-related protein 4	2/3	8	34	127	126	8	**0.43**
					129	128	8	**2.63**
					131	130	8	**1.20**
**237**	CFHR5 Q9BXR6: Complement factor H-related protein 5		23	63	127	126	40	**0.52**
					129	128	46	**2.52**
					131	130	41	**1.70**
**238**	CFI P05156: Complement factor I		21	82	127	126	60	**1.13**
					129	128	61	**1.66**
					131	130	61	**1.18**
**239**	CFL1 P23528: Cofilin-1		7	8	127	126	7	**0.66**
					129	128	6	**0.47**
					131	130	7	**0.94**
**240**	CFP P27918: Properdin		13	21	127	126	17	**0.40**
					129	128	19	**2.26**
					131	130	18	**0.77**
**241**	CGREF1 Q99674$_{1,4,5}$: Cell growth regulator with EF hand domain protein 1	3/4	10	12	127	126	11	**0.89**
					129	128	10	**1.81**
					131	130	11	**1.23**
**242**	CHGA P10645: Chromogranin-A		25	104	127	126	101	**0.67**
					129	128	99	**2.62**
					131	130	95	**1.27**
**243**	CHGB P05060: Secretogranin-1		21	49	127	126	45	**0.81**
					129	128	41	**2.26**
					131	130	41	**1.18**
**244**	CHMP4A Q9BY43$_{1,2}$: Charged multivesicular body protein 4a	2/2	2	9	127	126	1	**0.91**
					129	128	1	**1.27**
					131	130	1	**1.44**
**245**	CHRD Q9H2X0$_{1,5}$: Chordin	2/3	6	6	127	126	5	**0.50**
					129	128	6	**1.98**
					131	130	5	**1.99**

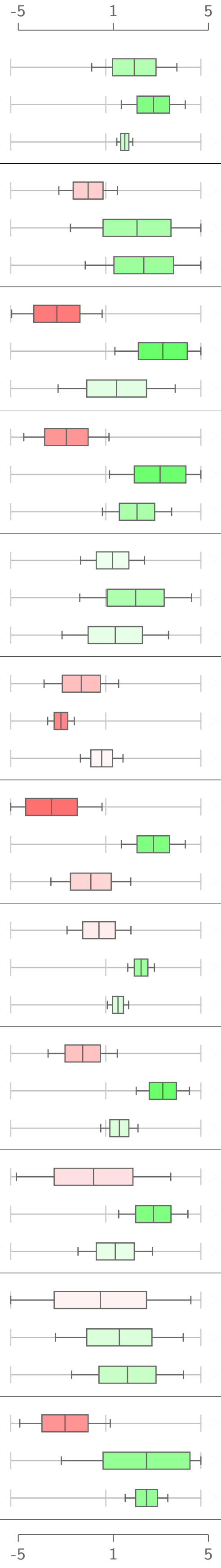

#	protein	group	peptides	spectra	ch1	ch2	quant	ratio
**246**	CHRDL1 Q9BU40$_{2,4}$: Chordin-like protein 1	2/6	16	25	127	126	24	**0.69**
					129	128	24	**1.77**
					131	130	23	**1.73**
**247**	CHRDL2 Q6WN34$_{1}$: Chordin-like protein 2	1/2	5	5	127	126	4	**0.56**
					129	128	4	**1.72**
					131	130	5	**1.61**
**248**	CHST14 Q8NCH0: Carbohydrate sulfotransferase 14		2	2	127	126	2	**0.99**
					129	128	2	**0.86**
					131	130	2	**2.21**
**249**	CILP O75339: Cartilage intermediate layer protein 1		47	94	127	126	92	**0.55**
					129	128	91	**2.34**
					131	130	93	**1.61**
**250**	CILP2 Q8IUL8: Cartilage intermediate layer protein 2		4	5	127	126	3	**1.11**
					129	128	4	**1.24**
					131	130	4	**0.94**
**251**	CKAP4 Q07065: Cytoskeleton-associated protein 4		15	16	127	126	13	**0.98**
					129	128	12	**0.29**
					131	130	11	**0.15**
**252**	CKB P12277: Creatine kinase B-type	1/2	5	5	127	126	5	**0.72**
					129	128	5	**1.28**
					131	130	5	**1.82**
**253**	CLC Q05315: Galectin-10		3	3	127	126	3	**0.83**
					129	128	3	**1.17**
					131	130	3	**2.40**
**254**	CLEC11A Q9Y240: C-type lectin domain family 11 member A		12	22	127	126	19	**0.43**
					129	128	22	**1.51**
					131	130	19	**1.51**
**255**	CLEC3B P05452: Tetranectin		9	11	127	126	11	**1.58**
					129	128	11	**1.14**
					131	130	10	**1.20**
**256**	CLGN O14967$_{1}$: Calmegin		2	2	127	126	1	**2.04**
					129	128	1	**1.06**
					131	130	1	**0.37**
**257**	CLIC1 O00299: Chloride intracellular channel protein 1		5	6	127	126	6	**0.62**
					129	128	5	**1.11**
					131	130	6	**1.04**

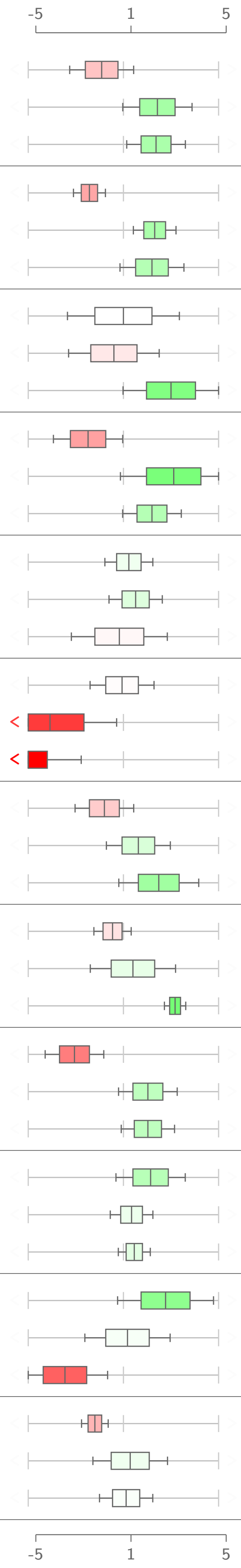

#	protein	group	peptides	spectra	ch1	ch2	quant	ratio
**258**	CLIC4 Q9Y696: Chloride intracellular channel protein 4		4	4	127	126	4	**1.02**
					129	128	3	**0.63**
					131	130	3	**0.81**
**259**	CLSTN1 O94985$_1$: Calsyntenin-1	1/2	14	14	127	126	13	**0.86**
					129	128	13	**1.45**
					131	130	14	**1.24**
**260**	CLTB P09497$_2$: Clathrin light chain B	1/2	5	5	127	126	5	**0.73**
					129	128	4	**0.60**
					131	130	5	**0.81**
**261**	CLTC Q00610$_{1,2}$: Clathrin heavy chain 1	2/2	26	28	127	126	18	**0.83**
					129	128	19	**0.35**
					131	130	13	**1.03**
**262**	CLTCL1 P53675$_{1,2}$: Clathrin heavy chain 2	2/2	9	10	127	126	1	**1.19**
					129	128	1	**1.40**
					131	130	1	**1.18**
**263**	CLU P10909$_4$: Clusterin	1/5	28	672	127	126	618	**0.53**
					129	128	623	**2.09**
					131	130	593	**1.70**
**264**	CNDP1 Q96KN2: Beta-Ala-His dipeptidase		6	8	127	126	8	**0.48**
					129	128	8	**0.94**
					131	130	6	**1.25**
**265**	CNN2 Q99439$_{1,2}$: Calponin-2	2/2	2	2	127	126	1	**0.71**
					129	128	1	**0.52**
					131	130	1	**2.01**
**266**	CNN3 Q15417$_{1,3}$: Calponin-3	2/5	8	8	127	126	6	**0.73**
					129	128	7	**0.75**
					131	130	7	**1.93**
**267**	CNOT7 Q9UIV1$_1$: CCR4-NOT transcription complex subunit 7		4	11	127	126	11	**0.86**
					129	128	11	**1.14**
					131	130	6	**1.06**
**268**	CNPY2 Q9Y2B0$_1$: Protein canopy homolog 2		2	3	127	126	3	**0.89**
					129	128	3	**1.24**
					131	130	3	**0.80**
**269**	COL12A1 Q99715$_1$: Collagen alpha-1(XII) chain	1/3	51	63	127	126	55	**1.83**
					129	128	56	**0.73**
					131	130	58	**1.90**

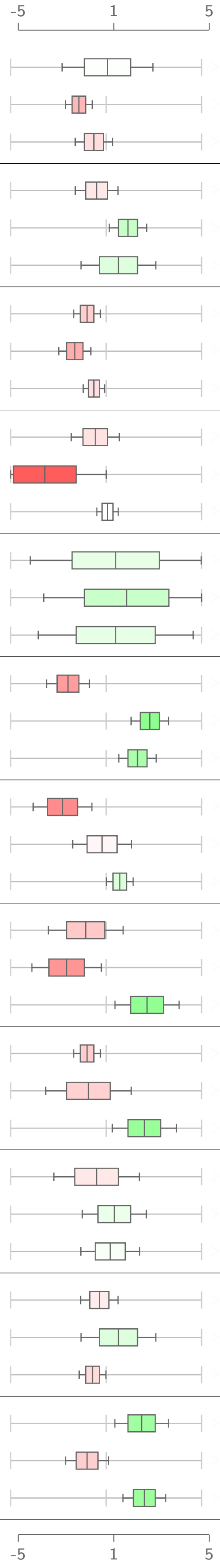

#	protein	group	peptides	spectra	ch1	ch2	quant	ratio
**270**	COL14A1 Q05707$_2$: Collagen alpha-1(XIV) chain	1/3	43	62	127	126	58	**0.73**
					129	128	55	**1.03**
					131	130	59	**1.98**
**271**	COL15A1 P39059: Collagen alpha-1(XV) chain [Cleaved into: Restin		3	3	127	126	3	**1.16**
					129	128	3	**0.92**
					131	130	3	**1.56**
**272**	COL18A1 P39060$_{1-3}$: Collagen alpha-1(XVIII) chain [Cleaved into: Endostatin]	3/3	15	25	127	126	22	**1.87**
					129	128	23	**1.89**
					131	130	23	**1.59**
**273**	COL1A1 P02452: Collagen alpha-1(I) chain		23	114	127	126	100	**1.10**
					129	128	103	**1.31**
					131	130	109	**1.22**
**274**	COL1A2 P08123: Collagen alpha-2(I) chain		21	30	127	126	30	**0.87**
					129	128	27	**1.64**
					131	130	27	**1.17**
**275**	COL3A1 P02461$_1$: Collagen alpha-1(III) chain	1/2	16	28	127	126	23	**1.67**
					129	128	19	**0.84**
					131	130	23	**1.88**
**276**	COL5A1 P20908: Collagen alpha-1(V) chain		12	22	127	126	22	**0.85**
					129	128	22	**1.34**
					131	130	22	**1.23**
**277**	COL5A2 P05997: Collagen alpha-2(V) chain		13	21	127	126	16	**0.74**
					129	128	18	**1.60**
					131	130	18	**1.48**
**278**	COL6A1 P12109: Collagen alpha-1(VI) chain		25	43	127	126	35	**2.76**
					129	128	37	**0.44**
					131	130	38	**1.11**
**279**	COL6A2 P12110$_1$: Collagen alpha-2(VI) chain	1/2	21	125	127	126	10	**1.65**
					129	128	12	**0.43**
					131	130	12	**0.85**
**280**	COL6A2 P12110$_2$: Collagen alpha-2(VI) chain		15	114	127	126	1	**1.04**
					129	128	1	**0.80**
					131	130	1	**1.45**
**281**	COL6A3 P12111$_1$: Collagen alpha-3(VI) chain	1/4	107	225	127	126	18	**1.75**
					129	128	18	**0.46**
					131	130	19	**1.49**

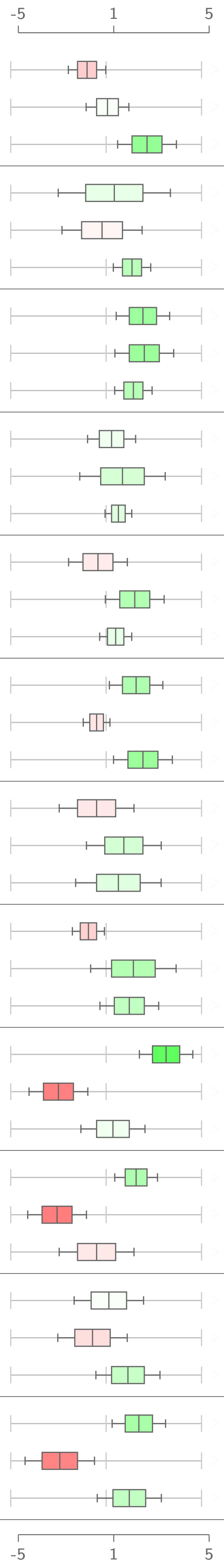

#	protein	group	peptides	spectra	ch1	ch2	quant	ratio
**282**	COL6A3 P12111$_4$: Collagen alpha-3(VI) chain		94	211	127	126	5	**3.11**
					129	128	3	**0.38**
					131	130	5	**1.92**
**283**	COLEC10 Q9Y6Z7: Collectin-10		2	3	127	126	2	**0.88**
					129	128	2	**1.72**
					131	130	3	**1.33**
**284**	COLEC11 Q9BWP8$_{1-10}$: Collectin-11	10/10	2	2	127	126	2	**0.60**
					129	128	2	**2.09**
					131	130	2	**1.41**
**285**	COMP P49747$_1$: Cartilage oligomeric matrix protein	1/2	30	57	127	126	44	**0.86**
					129	128	49	**1.53**
					131	130	42	**1.87**
**286**	COPA P53621$_{1,2}$: Coatomer subunit alpha	2/2	3	3	127	126	3	**0.70**
					129	128	3	**0.53**
					131	130	3	**1.02**
**287**	COPB1 P53618: Coatomer subunit beta		7	7	127	126	4	**1.10**
					129	128	6	**0.48**
					131	130	7	**1.29**
**288**	COPB2 P35606$_{1,2}$: Coatomer subunit beta'	2/2	9	10	127	126	8	**0.80**
					129	128	9	**0.66**
					131	130	10	**1.40**
**289**	COPE O14579$_{1,3}$: Coatomer subunit epsilon	2/3	2	2	127	126	2	**0.88**
					129	128	2	**0.92**
					131	130	2	**1.16**
**290**	COPG1 Q9Y678: Coatomer subunit gamma-1		6	6	127	126	6	**1.02**
					129	128	6	**0.76**
					131	130	6	**1.43**
**291**	CORO1A P31146: Coronin-1A	1/6	7	7	127	126	6	**0.85**
					129	128	6	**0.60**
					131	130	6	**0.84**
**292**	CORO1C Q9ULV4$_{1-3}$: Coronin-1C	3/3	5	5	127	126	3	**1.82**
					129	128	4	**0.61**
					131	130	4	**0.88**
**293**	CP P00450: Ceruloplasmin		39	129	127	126	126	**1.34**
					129	128	129	**1.63**
					131	130	121	**0.82**

-5 1 5

#	protein	group	peptides	spectra	ch1	ch2	quant	ratio
**294**	CPA4 Q9UI42$_{1,2}$: Carboxypeptidase A4	2/2	3	3	127	126	3	**0.81**
					129	128	3	**0.10**
					131	130	3	**0.97**
**295**	CPB2 Q96IY4$_1$: Carboxypeptidase B2	1/2	11	21	127	126	21	**0.95**
					129	128	20	**1.17**
					131	130	21	**1.92**
**296**	CPE P16870$_{1,2}$: Carboxypeptidase E	2/2	23	66	127	126	62	**0.84**
					129	128	58	**0.82**
					131	130	62	**1.65**
**297**	CPN1 P15169: Carboxypeptidase N catalytic chain		11	23	127	126	22	**0.77**
					129	128	23	**1.53**
					131	130	22	**1.04**
**298**	CPN2 P22792: Carboxypeptidase N subunit 2		13	36	127	126	34	**1.14**
					129	128	32	**0.91**
					131	130	34	**1.35**
**299**	CPQ Q9Y646: Carboxypeptidase Q		5	6	127	126	6	**0.80**
					129	128	6	**0.98**
					131	130	5	**1.42**
**300**	CPXM1 Q96SM3: Probable carboxypeptidase X1		4	4	127	126	4	**0.44**
					129	128	4	**1.44**
					131	130	3	**1.37**
**301**	CPXM2 Q8N436: Inactive carboxypeptidase-like protein X2		8	9	127	126	7	**0.44**
					129	128	8	**1.97**
					131	130	7	**1.76**
**302**	CPZ Q66K79$_{1-3}$: Carboxypeptidase Z	3/3	2	3	127	126	3	**0.70**
					129	128	3	**1.11**
					131	130	3	**1.39**
**303**	CRABP2 P29373: Cellular retinoic acid-binding protein 2		6	6	127	126	6	**0.99**
					129	128	6	**0.55**
					131	130	6	**2.20**
**304**	CRHBP P24387: Corticotropin-releasing factor-binding protein		2	2	127	126	2	**0.50**
					129	128	2	**1.32**
					131	130	2	**1.26**
**305**	CRISPLD2 Q9H0B8$_{1-3}$: Cysteine-rich secretory protein LCCL domain-containing 2	3/3	2	4	127	126	4	**0.69**
					129	128	4	**1.41**
					131	130	4	**0.61**

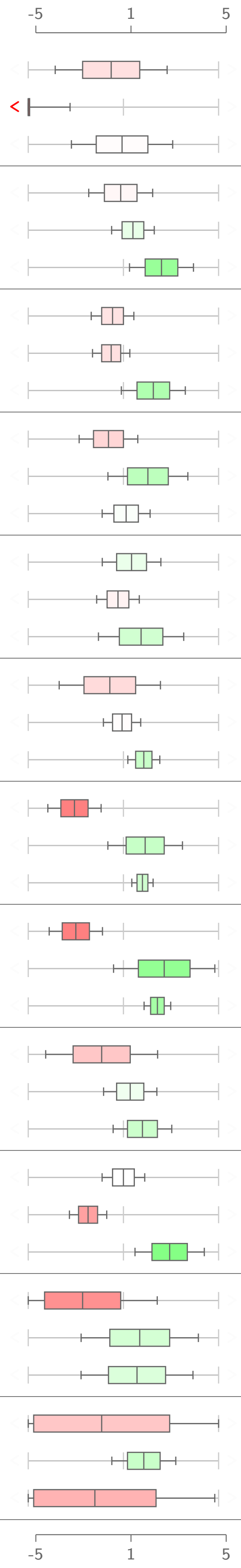

#	protein	group	peptides	spectra	ch1	ch2	quant	ratio
**306**	CRP P02741$_{1}$: C-reactive protein [Cleaved into: C-reactive protein(1-205)]	1/2	8	34	127	126	34	**0.71**
					129	128	34	**4.58**
					131	130	34	**1.75**
**307**	CRTAC1 Q9NQ79$_{1-3}$: Cartilage acidic protein 1	3/3	1	1	127	126	1	**0.98**
					129	128	1	**1.53**
					131	130	1	**1.30**
**308**	CRYAB P02511: Alpha-crystallin B chain		3	4	127	126	4	**0.61**
					129	128	4	**1.16**
					131	130	3	**0.71**
**309**	CSDE1 O75534$_{1-4}$: Cold shock domain-containing protein E1	4/4	1	1	127	126	1	**0.54**
					129	128	1	**0.53**
					131	130	1	**0.93**
**310**	CSE1L P55060$_{1,3,4}$: Exportin-2	3/3	1	1	127	126	1	**0.78**
					129	128	1	**0.26**
					131	130	1	**1.46**
**311**	CSPG4 Q6UVK1: Chondroitin sulfate proteoglycan 4		6	6	127	126	6	**0.92**
					129	128	5	**1.05**
					131	130	6	**1.59**
**312**	CST3 P01034: Cystatin-C		7	31	127	126	31	**1.29**
					129	128	29	**1.10**
					131	130	26	**1.63**
**313**	CST6 Q15828: Cystatin-M		8	13	127	126	13	**0.92**
					129	128	11	**0.94**
					131	130	13	**1.64**
**314**	CSTB P04080: Cystatin-B		3	3	127	126	3	**0.96**
					129	128	3	**0.49**
					131	130	3	**1.94**
**315**	CTGF P29279$_{1,2}$: Connective tissue growth factor	2/2	6	10	127	126	8	**0.63**
					129	128	9	**1.29**
					131	130	9	**1.52**
**316**	CTNNA1 P35221$_{1,2}$: Catenin alpha-1	2/7	6	6	127	126	4	**1.08**
					129	128	5	**0.37**
					131	130	6	**1.24**
**317**	CTNNB1 P35222$_{1}$: Catenin beta-1		9	11	127	126	6	**0.62**
					129	128	5	**0.20**
					131	130	5	**0.92**

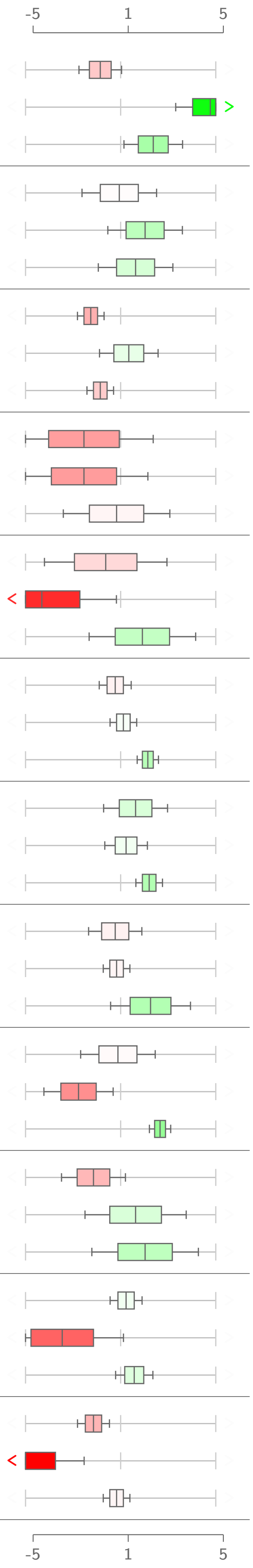

#	protein	group	peptides	spectra	ch1	ch2	quant	ratio
**318**	CTNND1 O60716$_{1-32}$: Catenin delta-1	32/32	1	1	127	126	1	**0.96**
					129	128	1	**0.51**
					131	130	1	**0.78**
**319**	CTSB P07858: Cathepsin B		4	8	127	126	8	**0.38**
					129	128	8	**1.59**
					131	130	8	**1.22**
**320**	CTSC P53634$_1$: Dipeptidyl peptidase 1		2	2	127	126	2	**0.73**
					129	128	2	**1.38**
					131	130	2	**1.24**
**321**	CTSD P07339: Cathepsin D		6	7	127	126	6	**0.83**
					129	128	7	**0.82**
					131	130	7	**0.82**
**322**	CTSF Q9UBX1: Cathepsin F		4	4	127	126	4	**1.19**
					129	128	3	**0.91**
					131	130	4	**1.75**
**323**	CTSV O60911: Cathepsin L2		2	2	127	126	2	**0.88**
					129	128	2	**0.19**
					131	130	2	**1.07**
**324**	CTTN Q14247$_{1,3}$: Src substrate cortactin	2/3	3	3	127	126	3	**0.58**
					129	128	3	**1.02**
					131	130	3	**1.51**
**325**	CUL3 Q13618$_{1-3}$: Cullin-3	3/3	5	6	127	126	6	**0.75**
					129	128	6	**1.05**
					131	130	6	**1.11**
**326**	CXCL12 P48061$_{1-6}$: Stromal cell-derived factor 1	6/6	4	5	127	126	5	**0.61**
					129	128	3	**1.33**
					131	130	5	**1.63**
**327**	CYB5A P00167$_{1-3}$: Cytochrome b5	3/3	1	1	127	126	1	**0.67**
					129	128	1	**0.40**
					131	130	1	**0.63**
**328**	CYFIP1 Q7L576$_1$: Cytoplasmic FMR1-interacting protein 1		5	5	127	126	4	**1.56**
					129	128	5	**0.47**
					131	130	4	**0.83**
**329**	CYTL1 Q9NRR1: Cytokine-like protein 1		5	5	127	126	5	**1.21**
					129	128	4	**1.30**
					131	130	4	**1.17**

#	protein	group	peptides	spectra	ch1	ch2	quant	ratio	-5 1 5
**330**	DAG1 Q14118: Dystroglycan		8	9	127	126	9	**1.49**	
					129	128	9	**1.28**	
					131	130	8	**1.37**	
**331**	DARS $P14868_{1,2}$: Aspartate--tRNA ligase, cytoplasmic	2/2	4	4	127	126	4	**1.04**	
					129	128	4	**0.43**	
					131	130	3	**0.87**	
**332**	DCD $P81605_{1}$: Dermcidin	1/2	8	15	127	126	15	**0.48**	
					129	128	15	**1.99**	
					131	130	15	**0.72**	
**333**	DCHS1 Q96JQ0: Protocadherin-16		4	4	127	126	4	**0.69**	
					129	128	3	**1.11**	
					131	130	4	**1.70**	
**334**	DCN $P07585_{1}$: Decorin	1/4	9	23	127	126	20	**1.09**	
					129	128	20	**0.61**	
					131	130	20	**2.58**	
**335**	DCTN1 $Q14203_{1,3,4,6}$: Dynactin subunit 1	4/6	7	7	127	126	4	**1.04**	
					129	128	6	**0.67**	
					131	130	6	**1.08**	
**336**	DDX1 $Q92499_{1,3}$: ATP-dependent RNA helicase DDX1	2/2	4	11	127	126	3	**1.24**	
					129	128	3	**0.33**	<
					131	130	3	**1.06**	
**337**	DDX39B $Q13838_{1,2}$: Spliceosome RNA helicase DDX39B	2/2	5	6	127	126	6	**1.22**	
					129	128	6	**0.35**	
					131	130	6	**1.06**	
**338**	DDX3X $O00571_{1,2}$: ATP-dependent RNA helicase DDX3X	2/4	3	4	127	126	4	**1.07**	
					129	128	4	**0.18**	<
					131	130	4	**1.14**	
**339**	DDX5 $P17844_{1,2}$: Probable ATP-dependent RNA helicase DDX5	2/6	4	4	127	126	3	**0.94**	
					129	128	4	**0.41**	
					131	130	4	**1.05**	
**340**	DEFA1 P59665: Neutrophil defensin 1		3	5	127	126	4	**1.07**	
					129	128	4	**1.58**	
					131	130	4	**1.42**	
**341**	DEFA3 P59666: Neutrophil defensin 3		4	20	127	126	13	**0.57**	
					129	128	15	**1.24**	
					131	130	12	**1.44**	

-5 1 5

#	protein	group	peptides	spectra	ch1	ch2	quant	ratio
**342**	DEFA4 P12838: Neutrophil defensin 4		2	2	127	126	2	**0.77**
					129	128	2	**2.24**
					131	130	2	**1.51**
**343**	DEFA5 Q01523: Defensin-5		1	1	127	126	1	**0.52**
					129	128	1	**4.36**
					131	130	1	**2.32**
**344**	DEFA6 Q01524: Defensin-6		2	2	127	126	1	**0.63**
					129	128	1	**2.97**
					131	130	1	**1.56**
**345**	DES P17661: Desmin		15	27	127	126	10	**0.81**
					129	128	9	**2.04**
					131	130	10	**1.03**
**346**	DFNA5 O60443$_1$: Non-syndromic hearing impairment protein 5	1/3	6	6	127	126	5	**1.30**
					129	128	5	**0.76**
					131	130	5	**1.90**
**347**	DHX9 Q08211$_1$: ATP-dependent RNA helicase A		4	5	127	126	5	**0.98**
					129	128	4	**0.51**
					131	130	4	**1.28**
**348**	DKK3 Q9UBP4: Dickkopf-related protein 3		8	36	127	126	26	**0.44**
					129	128	23	**1.68**
					131	130	25	**1.63**
**349**	DLD P09622$_{1-3}$: Dihydrolipoyl dehydrogenase, mitochondrial	3/3	2	2	127	126	2	**0.53**
					129	128	2	**0.56**
					131	130	2	**0.03**
**350**	DLST P36957$_1$: Dihydrolipoyllysine-residue succinyltransferase component of 2-oxoglutarate ...	1/2	3	4	127	126	3	**0.81**
					129	128	3	**0.30**
					131	130	3	**0.11**
**351**	DMKN Q6E0U4$_{1-7,16}$: Dermokine	8/8	6	7	127	126	6	**0.88**
					129	128	7	**1.82**
					131	130	6	**1.52**
**352**	DMP1 Q13316$_{1,2}$: Dentin matrix acidic phosphoprotein 1	2/2	3	3	127	126	3	**0.53**
					129	128	3	**1.14**
					131	130	3	**1.38**
**353**	DNAH1 Q9P2D7$_{1,2,4}$: Dynein heavy chain 1, axonemal	3/4	7	17	127	126	4	**0.62**
					129	128	4	**0.77**
					131	130	5	**0.99**

#	protein	group	peptides	spectra	ch1	ch2	quant	ratio
**354**	DNAH7 Q8WXX0$_1$: Dynein heavy chain 7, axonemal		7	17	127	126	14	**0.73**
					129	128	12	**1.07**
					131	130	14	**1.07**
**355**	DNAJB1 P25685$_1$: DnaJ homolog subfamily B member 1		2	2	127	126	2	**0.77**
					129	128	2	**0.31**
					131	130	2	**1.30**
**356**	DNAJC3 Q13217: DnaJ homolog subfamily C member 3		12	13	127	126	10	**0.91**
					129	128	11	**1.14**
					131	130	10	**1.41**
**357**	DNM2 P50570$_{1-5}$: Dynamin-2	5/14	7	7	127	126	6	**1.16**
					129	128	7	**0.60**
					131	130	5	**1.39**
**358**	DNPH1 O43598$_{1,2}$: 2'-deoxynucleoside 5'-phosphate N-hydrolase 1	2/2	2	2	127	126	2	**0.66**
					129	128	2	**1.13**
					131	130	2	**1.05**
**359**	DOCK10 Q96BY6$_{1-3}$: Dedicator of cytokinesis protein 10	3/3	3	44	127	126	1	**0.97**
					129	128	1	**0.57**
					131	130	1	**2.82**
**361**	DPP4 P27487: Dipeptidyl peptidase 4		2	2	127	126	2	**1.13**
					129	128	2	**0.97**
					131	130	2	**0.32**
**362**	DPT Q07507: Dermatopontin		9	118	127	126	85	**0.39**
					129	128	94	**2.15**
					131	130	91	**1.50**
**363**	DPYSL2 Q16555$_{1,2}$: Dihydropyrimidinase-related protein 2	2/2	7	8	127	126	3	**0.97**
					129	128	4	**0.88**
					131	130	4	**1.04**
**364**	DPYSL3 Q14195$_2$: Dihydropyrimidinase-related protein 3	1/2	10	11	127	126	7	**0.97**
					129	128	8	**1.06**
					131	130	7	**1.35**
**365**	DSC1 Q08554$_1$: Desmocollin-1	1/2	13	62	127	126	35	**0.66**
					129	128	35	**0.31**
					131	130	39	**0.45**
**366**	DSC3 Q14574$_{1,2}$: Desmocollin-3	2/4	10	12	127	126	11	**0.85**
					129	128	11	**0.67**
					131	130	9	**1.24**

#	protein	group	peptides	spectra	ch1	ch2	quant	ratio
**367**	DSG1 Q02413$_1$: Desmoglein-1	1/2	17	24	127	126	15	**0.60**
					129	128	16	**0.24**
					131	130	17	**0.85**
**369**	DSG4 Q86SJ6$_{1,2}$: Desmoglein-4	2/2	4	6	127	126	2	**0.16**
					129	128	2	**0.15**
					131	130	2	**0.95**
**370**	DSP P15924$_1$: Desmoplakin	1/3	133	190	127	126	181	**0.51**
					129	128	176	**0.08**
					131	130	167	**0.62**
**371**	DSTN P60981$_1$: Destrin	1/2	6	6	127	126	4	**0.70**
					129	128	5	**0.45**
					131	130	5	**1.18**
**372**	DYNC1H1 Q14204: Cytoplasmic dynein 1 heavy chain 1		20	24	127	126	20	**1.50**
					129	128	23	**0.35**
					131	130	20	**1.18**
**373**	DYNC1I2 Q13409$_{1-3,5-7}$: Cytoplasmic dynein 1 intermediate chain 2	6/6	2	2	127	126	2	**0.71**
					129	128	2	**0.67**
					131	130	2	**1.06**
**374**	DYNC1LI2 O43237$_1$: Cytoplasmic dynein 1 light intermediate chain 2		4	4	127	126	3	**1.00**
					129	128	4	**0.61**
					131	130	3	**1.17**
**375**	DYNC2H1 Q8NCM8$_{1,2}$: Cytoplasmic dynein 2 heavy chain 1	2/2	5	5	127	126	5	**1.54**
					129	128	5	**0.96**
					131	130	4	**1.29**
**376**	ECH1 Q13011: Delta(3,5)-Delta(2,4)-dienoyl-CoA isomerase, mitochondrial		3	3	127	126	3	**0.73**
					129	128	3	**0.64**
					131	130	3	**0.11**
**377**	ECHS1 P30084: Enoyl-CoA hydratase, mitochondrial		2	2	127	126	2	**0.55**
					129	128	2	**0.48**
					131	130	2	**0.11**
**378**	ECM1 Q16610$_{1,4}$: Extracellular matrix protein 1	2/4	25	42	127	126	40	**0.65**
					129	128	40	**0.75**
					131	130	42	**0.78**
**379**	ECM2 O94769$_1$: Extracellular matrix protein 2		17	26	127	126	1	**0.80**
					129	128	1	**1.56**
					131	130	1	**1.52**

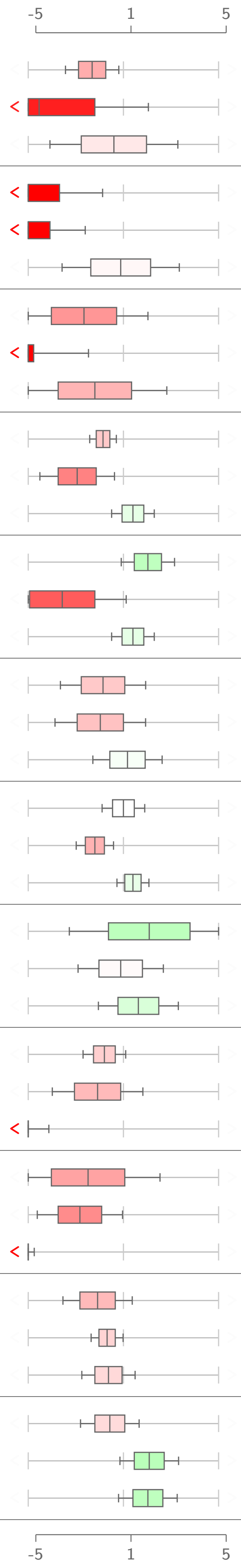

#	protein	group	peptides	spectra	ch1	ch2	quant	ratio
**380**	ECM2 O94769$_2$: Extracellular matrix protein 2		17	26	127	126	1	**0.71**
					129	128	1	**1.81**
					131	130	1	**1.75**
**381**	EEF1A1 P68104$_1$, EEF1A1P5 Q5VTE0: Elongation factor 1-alpha 1, Putative elongation factor 1-alpha-like 3	2/4	19	35	127	126	34	**0.89**
					129	128	35	**0.37**
					131	130	31	**1.47**
**382**	EEF1B2 P24534: Elongation factor 1-beta		3	3	127	126	3	**0.77**
					129	128	3	**0.61**
					131	130	3	**0.83**
**383**	EEF1D P29692$_{1-4}$: Elongation factor 1-delta	4/4	3	3	127	126	3	**0.60**
					129	128	3	**0.97**
					131	130	3	**1.09**
**384**	EEF1G P26641$_{1,2}$: Elongation factor 1-gamma	2/2	14	17	127	126	15	**1.07**
					129	128	17	**0.28**
					131	130	14	**1.24**
**385**	EEF2 P13639: Elongation factor 2		23	36	127	126	36	**1.70**
					129	128	34	**0.27**
					131	130	31	**1.12**
**386**	EFEMP1 Q12805$_{1-4}$: EGF-containing fibulin-like extracellular matrix protein 1	4/4	26	251	127	126	92	**1.00**
					129	128	100	**1.32**
					131	130	101	**1.72**
**387**	EFEMP1 Q12805$_5$: EGF-containing fibulin-like extracellular matrix protein 1		24	142	127	126	3	**0.92**
					129	128	3	**1.41**
					131	130	3	**2.04**
**388**	EFEMP2 O95967: EGF-containing fibulin-like extracellular matrix protein 2		16	40	127	126	38	**0.72**
					129	128	39	**1.48**
					131	130	39	**1.55**
**389**	EHD1 Q9H4M9: EH domain-containing protein 1	1/3	9	10	127	126	9	**1.48**
					129	128	8	**0.64**
					131	130	9	**1.35**
**390**	EHD2 Q9NZN4$_1$: EH domain-containing protein 2	1/2	9	11	127	126	8	**1.28**
					129	128	8	**1.33**
					131	130	7	**1.34**
**391**	EIF2S1 P05198: Eukaryotic translation initiation factor 2 subunit 1		3	3	127	126	3	**1.36**
					129	128	3	**0.55**
					131	130	3	**1.03**

#	protein	group	peptides	spectra	ch1	ch2	quant	ratio
**392**	EIF3A Q14152$_{1,2}$: Eukaryotic translation initiation factor 3 subunit A	2/2	3	3	127	126	1	**0.78**
					129	128	1	**0.43**
					131	130	1	**0.88**
**393**	EIF3B P55884$_{1,2}$: Eukaryotic translation initiation factor 3 subunit B	2/2	2	2	127	126	2	**0.67**
					129	128	2	**0.56**
					131	130	2	**1.23**
**394**	EIF3E P60228: Eukaryotic translation initiation factor 3 subunit E		3	3	127	126	3	**0.63**
					129	128	3	**0.30**
					131	130	3	**0.91**
**395**	EIF3H O15372: Eukaryotic translation initiation factor 3 subunit H		3	3	127	126	3	**1.00**
					129	128	3	**0.58**
					131	130	3	**1.10**
**396**	EIF3I Q13347: Eukaryotic translation initiation factor 3 subunit I		2	2	127	126	2	**0.52**
					129	128	2	**0.99**
					131	130	2	**1.23**
**397**	EIF4A1 P60842$_{1,2}$: Eukaryotic initiation factor 4A-I	2/4	8	10	127	126	7	**1.27**
					129	128	7	**0.41**
					131	130	7	**1.02**
**398**	EIF4A3 P38919: Eukaryotic initiation factor 4A-III		3	4	127	126	1	**1.11**
					129	128	1	**0.50**
					131	130	1	**0.89**
**399**	EIF4G1 Q04637$_{1,3,8,9}$: Eukaryotic translation initiation factor 4 gamma 1	4/8	6	6	127	126	6	**0.72**
					129	128	6	**0.52**
					131	130	6	**1.65**
**400**	EIF4H Q15056$_{1,2}$: Eukaryotic translation initiation factor 4H	2/2	2	2	127	126	2	**0.67**
					129	128	2	**0.44**
					131	130	2	**0.95**
**401**	EIF5A P63241$_{1,2}$: Eukaryotic translation initiation factor 5A-1	2/2	2	3	127	126	3	**1.06**
					129	128	3	**0.41**
					131	130	3	**0.88**
**402**	EMILIN1 Q9Y6C2$_{1}$: EMILIN-1		9	10	127	126	10	**0.56**
					129	128	10	**1.20**
					131	130	9	**1.52**
**403**	ENO1 P06733$_{1}$: Alpha-enolase	1/5	17	23	127	126	18	**1.47**
					129	128	21	**0.36**
					131	130	20	**1.19**

#	protein	group	peptides	spectra	ch1	ch2	quant	ratio
**404**	ENO2 P09104$_{1,2}$: Gamma-enolase	2/2	3	3	127	126	1	**1.32**
					129	128	1	**0.37**
					131	130	1	**1.29**
**405**	EPHX1 P07099: Epoxide hydrolase 1		2	2	127	126	1	**1.32**
					129	128	1	**0.40**
					131	130	2	**0.25**
**406**	EPPK1 P58107: Epiplakin		12	16	127	126	6	**1.04**
					129	128	7	**0.11**
					131	130	7	**0.73**
**407**	ERH P84090: Enhancer of rudimentary homolog		1	2	127	126	2	**0.72**
					129	128	2	**1.29**
					131	130	2	**1.07**
**408**	ERLIN2 O94905$_1$: Erlin-2		3	3	127	126	3	**0.75**
					129	128	3	**0.37**
					131	130	3	**0.13**
**409**	ERP29 P30040$_1$: Endoplasmic reticulum resident protein 29		1	1	127	126	1	**0.93**
					129	128	1	**0.47**
					131	130	1	**0.05**
**410**	ERP44 Q9BS26: Endoplasmic reticulum resident protein 44		6	11	127	126	11	**0.54**
					129	128	11	**1.18**
					131	130	9	**0.73**
**411**	ETF1 P62495$_{1,2}$: Eukaryotic peptide chain release factor subunit 1	2/2	1	1	127	126	1	**1.04**
					129	128	1	**0.50**
					131	130	1	**1.26**
**412**	EVPL Q92817: Envoplakin		5	5	127	126	5	**1.08**
					129	128	5	**0.41**
					131	130	4	**0.48**
**413**	EWSR1 Q01844$_{1-3,5,6}$: RNA-binding protein EWS	5/5	3	3	127	126	3	**0.69**
					129	128	3	**0.66**
					131	130	3	**1.11**
**414**	EZR P15311: Ezrin	1/2	17	24	127	126	17	**1.75**
					129	128	17	**0.32**
					131	130	17	**0.60**
**415**	F10 P00742: Coagulation factor X		16	46	127	126	34	**0.57**
					129	128	35	**1.25**
					131	130	37	**1.82**

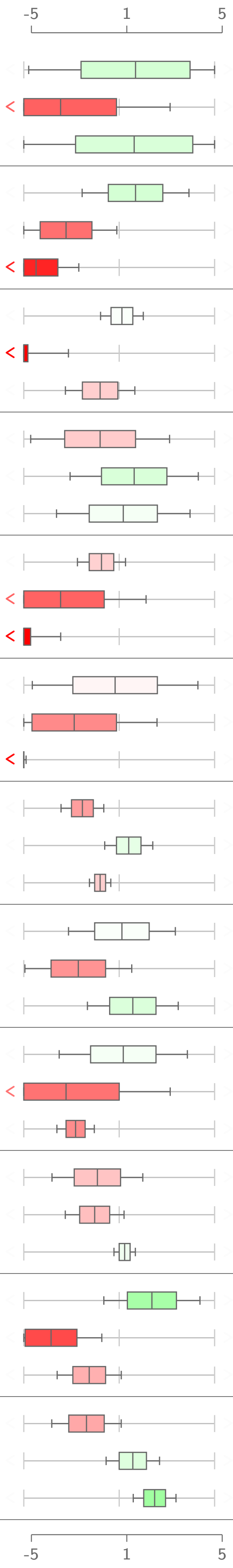

#	protein	group	peptides	spectra	ch1	ch2	quant	ratio
416	F11 P03951$_1$: Coagulation factor XI	1/2	24	33	127	126	32	**0.98**
					129	128	33	**1.65**
					131	130	31	**1.48**
417	F12 P00748: Coagulation factor XII		21	47	127	126	47	**1.47**
					129	128	46	**1.22**
					131	130	44	**1.43**
418	F13A1 P00488: Coagulation factor XIII A chain		26	77	127	126	71	**0.74**
					129	128	72	**1.09**
					131	130	72	**0.93**
419	F13B P05160: Coagulation factor XIII B chain		21	32	127	126	30	**0.97**
					129	128	31	**1.94**
					131	130	29	**1.33**
420	F2 P00734: Prothrombin		39	640	127	126	619	**1.12**
					129	128	600	**1.42**
					131	130	621	**1.47**
421	F5 P12259: Coagulation factor V		38	61	127	126	59	**0.67**
					129	128	59	**2.48**
					131	130	56	**1.89**
422	F7 P08709$_{1,2}$: Coagulation factor VII	2/2	10	13	127	126	12	**1.38**
					129	128	13	**0.79**
					131	130	13	**1.48**
423	F8 P00451$_1$: Coagulation factor VIII		10	14	127	126	10	**0.76**
					129	128	8	**1.81**
					131	130	9	**1.07**
424	F9 P00740$_1$: Coagulation factor IX	1/2	18	47	127	126	39	**0.93**
					129	128	37	**1.41**
					131	130	39	**1.83**
425	FABP3 P05413: Fatty acid-binding protein, heart		3	3	127	126	3	**0.89**
					129	128	3	**0.83**
					131	130	3	**1.86**
426	FABP5 Q01469: Fatty acid-binding protein, epidermal	1/2	5	5	127	126	5	**0.57**
					129	128	4	**0.12**
					131	130	4	**1.16**
427	FABP9 Q0Z7S8: Fatty acid-binding protein 9		5	9	127	126	4	**0.71**
					129	128	7	**0.08**
					131	130	5	**1.00**

#	protein	group	peptides	spectra	ch1	ch2	quant	ratio
**428**	FAM114A1 Q8IWE2$_{1,2}$: Protein NOXP20	2/2	2	2	127	126	2	**0.72**
					129	128	1	**0.82**
					131	130	2	**1.92**
**429**	FAM129B Q96TA1$_{2}$: Niban-like protein 1	1/2	8	16	127	126	13	**1.23**
					129	128	13	**0.47**
					131	130	13	**1.29**
**430**	FAM49B Q9NUQ9$_{1}$: Protein FAM49B	1/2	4	4	127	126	4	**1.19**
					129	128	3	**0.74**
					131	130	3	**0.98**
**431**	FARSB Q9NSD9$_{1}$: Phenylalanine--tRNA ligase beta subunit	1/2	3	3	127	126	3	**1.47**
					129	128	3	**0.39**
					131	130	3	**1.40**
**432**	FBLN1 P23142$_{1}$: Fibulin-1	1/2	29	472	127	126	95	**0.62**
					129	128	97	**1.24**
					131	130	102	**1.70**
**433**	FBLN1 P23142$_{3}$: Fibulin-1		22	373	127	126	6	**1.70**
					129	128	6	**0.95**
					131	130	6	**0.40**
**434**	FBLN1 P23142$_{4}$: Fibulin-1		28	389	127	126	20	**0.39**
					129	128	21	**2.25**
					131	130	22	**2.02**
**435**	FBLN2 P98095$_{1,2}$: Fibulin-2	2/2	29	49	127	126	46	**0.75**
					129	128	46	**1.15**
					131	130	47	**1.74**
**436**	FBLN5 Q9UBX5: Fibulin-5		15	114	127	126	110	**0.42**
					129	128	108	**2.05**
					131	130	105	**1.57**
**437**	FBLN7 Q53RD9$_{1-4}$: Fibulin-7	4/4	4	4	127	126	4	**0.75**
					129	128	4	**1.19**
					131	130	4	**1.84**
**438**	FBN1 P35555: Fibrillin-1 [Cleaved into: Asprosin]		83	163	127	126	114	**0.52**
					129	128	123	**2.07**
					131	130	112	**1.81**
**439**	FBN2 P35556$_{1}$: Fibrillin-2 [Cleaved into: Fibrillin-2 C-terminal peptide]	1/2	23	60	127	126	24	**0.52**
					129	128	28	**1.43**
					131	130	30	**1.11**

#	protein	group	peptides	spectra	ch1	ch2	quant	ratio
**440**	FBP1 P09467: Fructose-1,6-bisphosphatase 1		2	3	127	126	3	**0.92**
					129	128	3	**0.12**
					131	130	3	**0.64**
**441**	FBXO2 Q9UK22: F-box only protein 2		2	2	127	126	2	**0.70**
					129	128	2	**1.21**
					131	130	2	**1.14**
**442**	FCGBP Q9Y6R7: IgGFc-binding protein		26	32	127	126	31	**0.39**
					129	128	29	**1.56**
					131	130	31	**1.80**
**443**	FCGRT P55899: IgG receptor FcRn large subunit p51		2	4	127	126	4	**1.05**
					129	128	4	**0.93**
					131	130	3	**0.21**
**444**	FCN1 O00602: Ficolin-1		2	4	127	126	1	**0.25**
					129	128	1	**3.34**
					131	130	1	**0.53**
**445**	FCN2 Q15485$_1$: Ficolin-2	1/2	7	15	127	126	12	**0.25**
					129	128	11	**1.61**
					131	130	12	**1.41**
**446**	FCN3 O75636$_1$: Ficolin-3		12	50	127	126	3	**0.23**
					129	128	3	**5.74**
					131	130	3	**3.09**
**448**	FER1L4 A9Z1Z3$_1$: Fer-1-like protein 4	1/2	5	16	127	126	3	**1.02**
					129	128	3	**1.59**
					131	130	3	**1.10**
**449**	FERMT3 Q86UX7$_{1,2}$: Fermitin family homolog 3	2/2	3	3	127	126	2	**0.76**
					129	128	2	**0.93**
					131	130	3	**1.36**
**450**	FETUB Q9UGM5$_1$: Fetuin-B	1/2	16	39	127	126	37	**1.66**
					129	128	35	**0.57**
					131	130	38	**2.60**
**451**	FGA P02671$_1$: Fibrinogen alpha chain [Cleaved into: Fibrinopeptide A; Fibrinogen alpha chain]	1/2	68	4785	127	126	4522	**3.03**
					129	128	4531	**1.14**
					131	130	4394	**0.22**
**452**	FGB P02675: Fibrinogen beta chain [Cleaved into: Fibrinopeptide B; Fibrinogen beta chain]		48	4892	127	126	4618	**2.97**
					129	128	4484	**0.88**
					131	130	4647	**0.25**

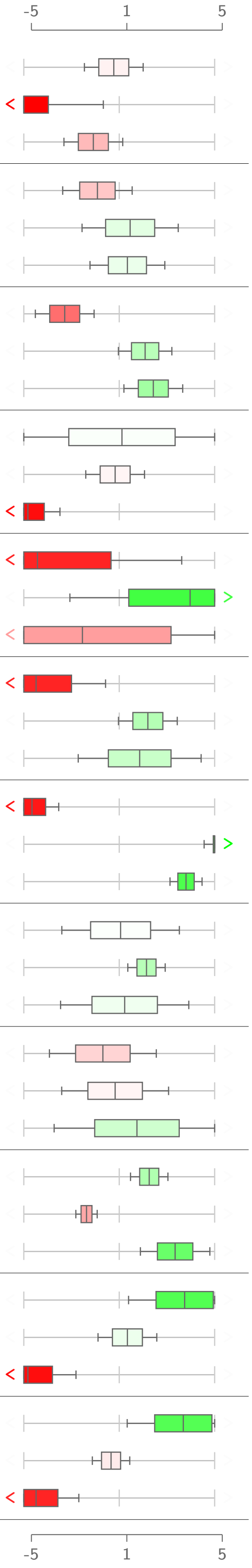

#	protein	group	peptides	spectra	ch1	ch2	quant	ratio
**453**	FGD6 Q6ZV73$_{1,2}$: FYVE, RhoGEF and PH domain-containing protein 6	2/2	4	9	127	126	7	**1.87**
					129	128	7	**1.65**
					131	130	8	**0.39**
**454**	FGG P02679$_{1,2}$: Fibrinogen gamma chain	2/2	37	2858	127	126	2704	**3.07**
					129	128	2627	**0.88**
					131	130	2664	**0.24**
**455**	FH P07954$_{1}$: Fumarate hydratase, mitochondrial	1/2	3	3	127	126	3	**0.80**
					129	128	3	**1.21**
					131	130	3	**1.34**
**456**	FHL2 Q14192$_{1}$: Four and a half LIM domains protein 2		11	24	127	126	16	**0.93**
					129	128	14	**1.20**
					131	130	15	**1.09**
**457**	FKBP1A P62942: Peptidyl-prolyl cis-trans isomerase FKBP1A		1	2	127	126	2	**1.01**
					129	128	2	**0.45**
					131	130	2	**1.57**
**458**	FKBP9 O95302$_{3}$: Peptidyl-prolyl cis-trans isomerase FKBP9	1/3	5	7	127	126	7	**0.65**
					129	128	7	**0.80**
					131	130	7	**0.76**
**459**	FLG P20930: Filaggrin		8	9	127	126	9	**0.43**
					129	128	9	**0.24**
					131	130	9	**0.95**
**460**	FLG2 Q5D862: Filaggrin-2		6	7	127	126	6	**0.20**
					129	128	5	**0.16**
					131	130	6	**0.60**
**461**	FLII Q13045$_{1-3}$: Protein flightless-1 homolog	3/3	2	2	127	126	2	**0.94**
					129	128	2	**0.35**
					131	130	2	**1.16**
**462**	FLNA P21333$_{1,2}$: Filamin-A	2/2	55	68	127	126	54	**1.43**
					129	128	56	**0.43**
					131	130	50	**1.03**
**463**	FLNB O75369$_{1,2,8,9}$: Filamin-B	4/9	28	30	127	126	20	**0.92**
					129	128	22	**0.24**
					131	130	20	**0.67**
**464**	FLNC Q14315$_{1,2}$: Filamin-C	2/2	41	45	127	126	36	**1.43**
					129	128	34	**0.77**
					131	130	34	**1.18**

#	protein	group	peptides	spectra	ch1	ch2	quant	ratio
**465**	FMOD Q06828: Fibromodulin		9	22	127	126	15	**0.58**
					129	128	14	**1.71**
					131	130	12	**1.83**
**466**	FN1 P02751$_{1,3}$: Fibronectin	2/17	95	1292	127	126	1228	**0.61**
					129	128	1204	**1.97**
					131	130	1220	**1.49**
**467**	FNDC1 Q4ZHG4$_{1,2}$: Fibronectin type III domain-containing protein 1	2/2	4	4	127	126	4	**0.91**
					129	128	4	**1.36**
					131	130	4	**1.28**
**468**	FRA10AC1 Q70Z53$_{3}$: Protein FRA10AC1	1/5	6	46	127	126	41	**1.29**
					129	128	34	**1.00**
					131	130	39	**1.32**
**469**	FRZB Q92765: Secreted frizzled-related protein 3		1	1	127	126	1	**0.61**
					129	128	1	**3.98**
					131	130	1	**2.06**
**470**	FSCN1 Q16658: Fascin		2	2	127	126	2	**0.59**
					129	128	2	**0.56**
					131	130	2	**1.12**
**471**	FST P19883$_{1,2}$: Follistatin	2/2	8	14	127	126	11	**0.88**
					129	128	9	**1.78**
					131	130	13	**1.70**
**472**	FSTL1 Q12841$_{1}$: Follistatin-related protein 1	1/2	17	40	127	126	36	**1.38**
					129	128	37	**1.20**
					131	130	37	**0.98**
**473**	FTL P02792: Ferritin light chain		2	2	127	126	2	**0.52**
					129	128	2	**1.43**
					131	130	2	**1.73**
**474**	FUS P35637$_{1,2}$: RNA-binding protein FUS	2/2	2	4	127	126	4	**0.63**
					129	128	3	**0.61**
					131	130	4	**1.01**
**475**	G3BP1 Q13283$_{1}$: Ras GTPase-activating protein-binding protein 1		2	2	127	126	2	**0.89**
					129	128	2	**0.83**
					131	130	2	**1.17**
**476**	GALK1 P51570$_{1,2}$: Galactokinase	2/2	2	2	127	126	2	**0.77**
					129	128	2	**1.03**
					131	130	2	**1.43**

#	protein	group	peptides	spectra	ch1	ch2	quant	ratio
**477**	GALNT2 Q10471$_{1,2}$: Polypeptide N-acetylgalactosaminyltransferase 2	2/2	1	1	127	126	1	**1.10**
					129	128	1	**0.81**
					131	130	1	**0.74**
**478**	GANAB Q14697$_{1}$: Neutral alpha-glucosidase AB	1/2	17	24	127	126	2	**0.51**
					129	128	2	**0.55**
					131	130	2	**0.60**
**479**	GANAB Q14697$_{2}$: Neutral alpha-glucosidase AB		16	23	127	126	1	**0.59**
					129	128	1	**1.51**
					131	130	1	**0.56**
**480**	GAPDH P04406$_{1}$: Glyceraldehyde-3-phosphate dehydrogenase	1/2	16	37	127	126	34	**0.86**
					129	128	32	**0.84**
					131	130	36	**1.44**
**481**	GAS1 P54826: Growth arrest-specific protein 1		9	15	127	126	15	**0.55**
					129	128	14	**1.60**
					131	130	14	**1.31**
**482**	GAS6 Q14393$_{1,2}$: Growth arrest-specific protein 6	2/5	11	18	127	126	15	**0.93**
					129	128	15	**0.91**
					131	130	12	**1.57**
**483**	GC P02774$_{1,3}$: Vitamin D-binding protein	2/3	28	305	127	126	244	**1.02**
					129	128	269	**1.64**
					131	130	250	**1.05**
**484**	GDF15 Q99988: Growth/differentiation factor 15		3	3	127	126	3	**0.90**
					129	128	3	**1.42**
					131	130	3	**0.69**
**486**	GHRL Q9UBU3$_{1-4}$: Appetite-regulating hormone	4/5	2	2	127	126	2	**1.09**
					129	128	2	**1.55**
					131	130	2	**1.24**
**487**	GIP P09681: Gastric inhibitory polypeptide		2	2	127	126	2	**2.46**
					129	128	2	**1.07**
					131	130	2	**1.29**
**488**	GIPC1 O14908$_{1,2}$: PDZ domain-containing protein GIPC1	2/2	1	1	127	126	1	**0.95**
					129	128	1	**1.40**
					131	130	1	**1.37**
**489**	GJA1 P17302: Gap junction alpha-1 protein		2	19	127	126	19	**1.65**
					129	128	15	**1.00**
					131	130	19	**0.32**

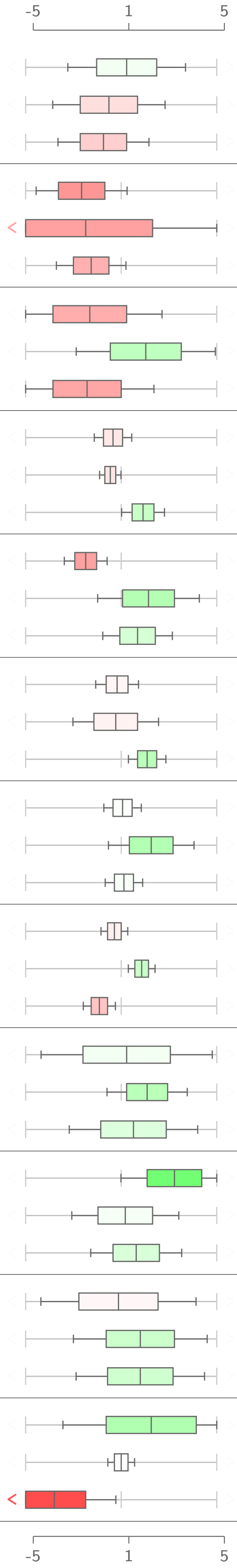

#	protein	group	peptides	spectra	ch1	ch2	quant	ratio
**490**	GKN1 Q9NS71: Gastrokine-1		6	17	127	126	14	**1.25**
					129	128	16	**1.23**
					131	130	15	**1.21**
**491**	GKN2 Q86XP6: Gastrokine-2		1	1	127	126	1	**0.64**
					129	128	1	**0.99**
					131	130	1	**1.55**
**492**	GLB1 $P16278_{1-3}$: Beta-galactosidase	3/3	1	1	127	126	1	**1.30**
					129	128	1	**0.94**
					131	130	1	**0.52**
**493**	GLG1 $Q92896_{1-3}$: Golgi apparatus protein 1	3/3	6	8	127	126	5	**0.71**
					129	128	7	**0.65**
					131	130	7	**0.36**
**494**	GLS $O94925_{1,3}$: Glutaminase kidney isoform, mitochondrial	2/2	2	2	127	126	2	**0.64**
					129	128	2	**0.64**
					131	130	2	**0.23**
**495**	GLUD1 $P00367_{1}$: Glutamate dehydrogenase 1, mitochondrial	1/3	3	3	127	126	3	**0.66**
					129	128	3	**0.41**
					131	130	3	**0.08**
**496**	GLUL P15104: Glutamine synthetase		4	4	127	126	4	**0.45**
					129	128	4	**0.93**
					131	130	3	**1.17**
**497**	GM2A P17900: Ganglioside GM2 activator		5	12	127	126	11	**1.12**
					129	128	12	**0.72**
					131	130	12	**2.57**
**498**	GMFG O60234: Glia maturation factor gamma		2	2	127	126	2	**0.70**
					129	128	2	**0.69**
					131	130	2	**0.93**
**499**	GNAI2 $P04899_{1-6}$: Guanine nucleotide-binding protein G(i) subunit alpha-2	6/6	3	3	127	126	3	**1.08**
					129	128	3	**0.23**
					131	130	3	**0.62**
**500**	GNB1 $P62873_{1,2}$: Guanine nucleotide-binding protein G(I)/G(S)/G(T) subunit beta-1	2/4	3	3	127	126	1	**0.72**
					129	128	1	**0.85**
					131	130	1	**0.80**
**502**	GNPTG Q9UJJ9: N-acetylglucosamine-1-phosphotransferase subunit gamma		3	3	127	126	3	**1.00**
					129	128	3	**1.46**
					131	130	3	**2.13**

#	protein	group	peptides	spectra	ch1	ch2	quant	ratio
**503**	GOLGA6L22 H0YM25: Golgin subfamily A member 6-like protein 22		13	105	127	126	60	**0.83**
					129	128	59	**1.66**
					131	130	60	**0.77**
**504**	GOLIM4 O00461: Golgi integral membrane protein 4		11	15	127	126	9	**0.58**
					129	128	11	**2.36**
					131	130	11	**1.43**
**505**	GOLM1 Q8NBJ4$_1$: Golgi membrane protein 1	1/2	9	16	127	126	10	**0.63**
					129	128	9	**0.87**
					131	130	8	**1.14**
**506**	GOT2 P00505$_1$: Aspartate aminotransferase, mitochondrial		1	1	127	126	1	**0.56**
					129	128	1	**0.37**
					131	130	1	**0.07**
**507**	GPATCH8 Q9UKJ3$_1$: G patch domain-containing protein 8	1/2	4	6	127	126	4	**0.50**
					129	128	5	**1.87**
					131	130	4	**1.39**
**508**	GPLD1 P80108$_1$: Phosphatidylinositol-glycan-specific phospholipase D		15	22	127	126	22	**0.77**
					129	128	22	**1.71**
					131	130	20	**1.81**
**509**	GPS1 Q13098$_{4-7}$: COP9 signalosome complex subunit 1	4/4	2	2	127	126	2	**1.37**
					129	128	2	**0.47**
					131	130	2	**1.35**
**510**	GPX3 P22352: Glutathione peroxidase 3		9	22	127	126	21	**0.64**
					129	128	21	**1.46**
					131	130	19	**1.52**
**512**	GRN P28799$_1$: Granulins	1/3	6	6	127	126	5	**0.92**
					129	128	3	**1.43**
					131	130	5	**1.23**
**513**	GSDMA Q96QA5: Gasdermin-A		5	6	127	126	6	**0.99**
					129	128	6	**0.23**
					131	130	6	**0.70**
**514**	GSN P06396$_1$: Gelsolin	1/3	44	700	127	126	20	**3.31**
					129	128	20	**0.50**
					131	130	20	**2.72**
**515**	GSN P06396$_2$: Gelsolin		43	681	127	126	2	**1.76**
					129	128	2	**0.42**
					131	130	2	**1.31**

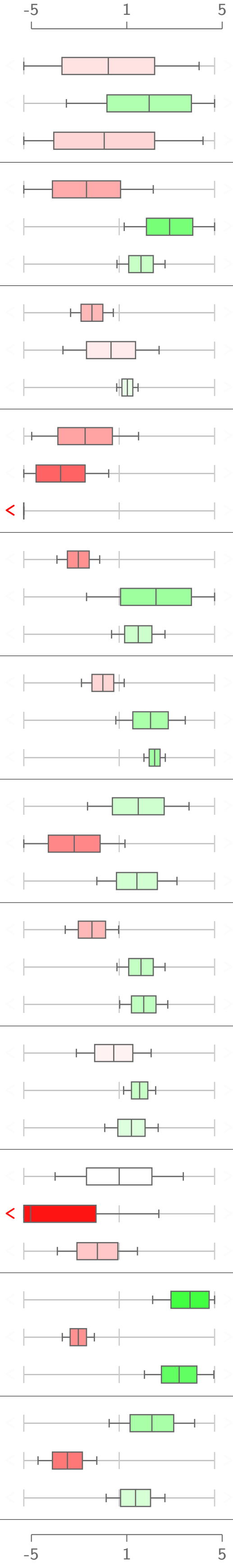

#	protein	group	peptides	spectra	ch1	ch2	quant	ratio
**516**	GSTM3 P21266: Glutathione S-transferase Mu 3		3	3	127	126	3	**0.97**
					129	128	3	**0.99**
					131	130	3	**2.03**
**517**	GSTO1 $P78417_{1,3}$: Glutathione S-transferase omega-1	2/2	2	2	127	126	2	**1.03**
					129	128	2	**0.72**
					131	130	2	**0.98**
**518**	GSTP1 P09211: Glutathione S-transferase P		3	4	127	126	3	**1.68**
					129	128	3	**0.75**
					131	130	4	**1.02**
**519**	GUCA2A Q02747: Guanylin		3	6	127	126	5	**1.97**
					129	128	6	**2.06**
					131	130	6	**0.83**
**520**	H1F0 $P07305_{1,2}$: Histone H1.0	2/2	2	2	127	126	2	**0.28**
					129	128	2	**0.14**
					131	130	2	**0.38**
**521**	H1FX Q92522: Histone H1x		2	3	127	126	3	**0.51**
					129	128	3	**0.49**
					131	130	3	**0.42**
**522**	H2AFV $Q71UI9_{1,2}$, H2AFZ P0C0S5: Histone H2A.V, Histone H2A.Z	3/5	3	6	127	126	1	**0.39**
					129	128	1	**0.34**
					131	130	1	**0.19**
**523**	H2AFX P16104: Histone H2AX		6	15	127	126	3	**0.39**
					129	128	3	**0.38**
					131	130	3	**0.21**
**524**	H3F3A P84243: Histone H3.3		8	25	127	126	1	**0.72**
					129	128	1	**0.28**
					131	130	1	**0.16**
**525**	HABP2 $Q14520_{1,2}$: Hyaluro-nan-binding protein 2	2/2	21	35	127	126	32	**0.76**
					129	128	28	**2.10**
					131	130	33	**1.95**
**526**	HACD2 Q6Y1H2: Very-long-chain		1	1	127	126	1	**1.61**
					129	128	1	**0.17**
					131	130	1	**0.09**
**527**	HADHA $P40939_{1}$: Trifunctional enzyme subunit alpha, mitochondrial	1/2	8	9	127	126	7	**0.82**
					129	128	9	**0.45**
					131	130	9	**0.24**

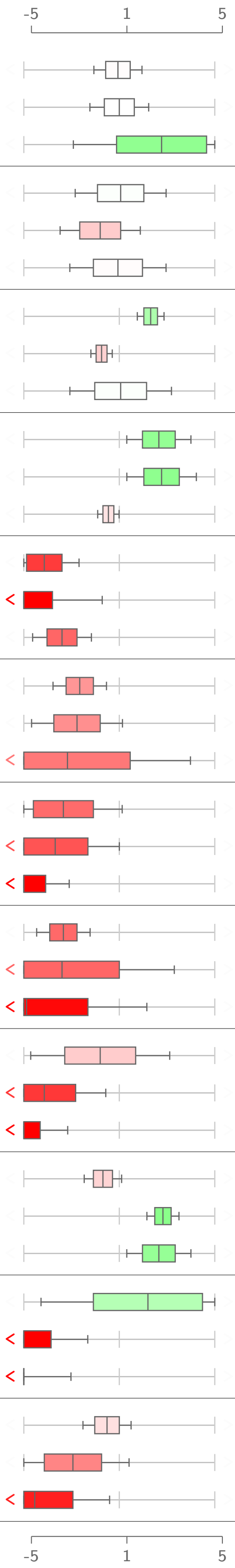

#	protein	group	peptides	spectra	ch1	ch2	quant	ratio
**528**	HADHB P55084$_1$: Trifunctional enzyme subunit beta, mitochondrial	1/2	4	4	127	126	3	**0.87**
					129	128	4	**0.46**
					131	130	4	**0.17**
**529**	HBA1; P69905: Hemoglobin subunit alpha		3	7	127	126	7	**0.74**
					129	128	6	**0.70**
					131	130	7	**0.27**
**530**	HBB P68871: Hemoglobin subunit beta	1/2	5	8	127	126	8	**0.73**
					129	128	8	**0.60**
					131	130	8	**0.12**
**531**	HDGF P51858$_{1-3}$: Hepatoma-derived growth factor	3/3	5	6	127	126	4	**1.22**
					129	128	3	**1.04**
					131	130	5	**0.75**
**532**	HDLBP Q00341$_{1,2}$: Vigilin	2/2	2	3	127	126	3	**1.41**
					129	128	3	**0.74**
					131	130	3	**1.87**
**533**	HERC2 O95714: E3 ubiquitin-protein ligase HERC2		7	41	127	126	20	**1.86**
					129	128	22	**1.07**
					131	130	22	**0.39**
**534**	HEXA P06865$_1$: Beta-hexosaminidase subunit alpha		4	7	127	126	6	**0.53**
					129	128	7	**1.51**
					131	130	7	**1.32**
**535**	HEXB P07686: Beta-hexosaminidase subunit beta		2	3	127	126	3	**0.47**
					129	128	3	**1.11**
					131	130	3	**0.99**
**537**	HIST1H1B P16401: Histone H1.5		10	15	127	126	5	**0.62**
					129	128	4	**0.15**
					131	130	6	**0.28**
**538**	HIST1H1C P16403: Histone H1.2	1/2	17	24	127	126	6	**1.01**
					129	128	6	**0.31**
					131	130	7	**0.27**
**539**	HIST1H1D P16402: Histone H1.3		11	15	127	126	2	**0.70**
					129	128	2	**0.19**
					131	130	2	**0.22**
**540**	HIST1H1E P10412: Histone H1.4		15	22	127	126	4	**0.54**
					129	128	5	**0.31**
					131	130	5	**0.21**

#	protein	group	peptides	spectra	ch1	ch2	quant	ratio
541	HIST1H2AB P04908, HIST1H2AD P20671, HIST3H2A Q7L7L0: Histone H2A type 1-B/E, Histone H2A type 1-D, . . .	3/3	5	14	127	126	1	**0.52**
					129	128	1	**0.63**
					131	130	1	**0.26**
545	HIST1H3A P68431, HIST2H3A; Q71DI3, HIST3H3 Q16695: Histone H3.1, Histone H3.2, . . .	3/4	8	26	127	126	2	**0.65**
					129	128	2	**0.18**
					131	130	2	**0.22**
546	HIST1H4A P62805: Histone H4		6	17	127	126	17	**0.41**
					129	128	14	**0.28**
					131	130	13	**0.20**
547	HIST2H2AC Q16777: Histone H2A type 2-C	1/10	7	18	127	126	4	**0.28**
					129	128	4	**0.18**
					131	130	5	**0.13**
551	HLA-A P10316: HLA class I histocompatibility antigen, A-69 alpha chain	1/2	6	7	127	126	1	**0.93**
					129	128	1	**1.10**
					131	130	1	**0.79**
556	HLA-B P03989: HLA class I histocompatibility antigen, B-27 alpha chain	1/15	7	9	127	126	1	**0.75**
					129	128	1	**0.76**
					131	130	1	**0.99**
558	HLA-B P30481: HLA class I histocompatibility antigen, B-44 alpha chain	1/2	6	8	127	126	1	**1.29**
					129	128	1	**1.21**
					131	130	1	**1.00**
563	HLA-C P10321: HLA class I histocompatibility antigen, Cw-7 alpha chain		11	13	127	126	2	**0.77**
					129	128	2	**2.54**
					131	130	2	**1.57**
566	HLA-C Q07000: HLA class I histocompatibility antigen, Cw-15 alpha chain	1/8	10	14	127	126	2	**0.44**
					129	128	2	**2.72**
					131	130	2	**0.92**
568	HLA-DRA P01903: HLA class II histocompatibility antigen, DR alpha chain	1/2	2	2	127	126	2	**0.53**
					129	128	2	**0.51**
					131	130	2	**0.28**
569	HMCN1 Q96RW7$_2$: Hemicentin-1	1/2	21	24	127	126	17	**1.70**
					129	128	17	**0.47**
					131	130	17	**1.86**
570	HMCN2 Q8NDA2$_1$: Hemicentin-2	1/3	17	19	127	126	15	**1.06**
					129	128	18	**0.97**
					131	130	17	**1.92**

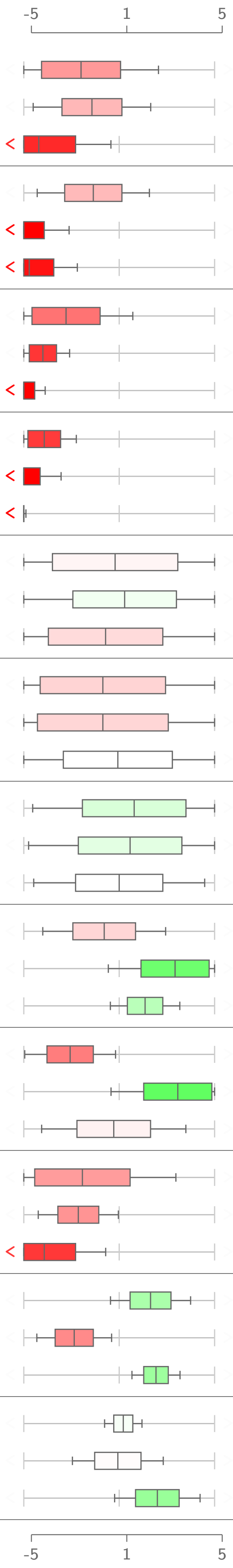

#	protein	group	peptides	spectra	ch1	ch2	quant	ratio	-5 1 5
**571**	HMGA1 P17096$_{1-3}$: High mobility group protein HMG-I/HMG-Y	3/3	1	1	127	126	1	**0.64**	
					129	128	1	**1.01**	
					131	130	1	**0.67**	
**572**	HMGN2 P05204: Non-histone chromosomal protein HMG-17		2	2	127	126	2	**0.73**	
					129	128	2	**0.51**	
					131	130	2	**0.47**	
**573**	HNRNPA0 Q13151: Heterogeneous nuclear ribonucleoprotein A0		2	2	127	126	2	**0.93**	
					129	128	2	**0.77**	
					131	130	2	**0.87**	
**574**	HNRNPA1 P09651$_{1-3}$: Heterogeneous nuclear ribonucleoprotein A1	3/4	5	7	127	126	2	**0.76**	
					129	128	2	**0.45**	
					131	130	2	**0.95**	
**575**	HNRNPA2B1 P22626$_{1,2}$: Heterogeneous nuclear ribonucleoproteins A2/B1	2/2	7	11	127	126	8	**0.99**	
					129	128	9	**0.55**	
					131	130	7	**0.82**	
**576**	HNRNPA3 P51991$_{1}$: Heterogeneous nuclear ribonucleoprotein A3	1/2	8	10	127	126	7	**0.61**	
					129	128	7	**0.76**	
					131	130	5	**1.04**	
**577**	HNRNPAB Q99729$_{1-4}$: Heterogeneous nuclear ribonucleoprotein A/B	4/4	2	2	127	126	1	**0.70**	
					129	128	1	**0.44**	
					131	130	1	**0.95**	
**578**	HNRNPC P07910$_{1,2,4}$: Heterogeneous nuclear ribonucleoproteins C1/C2	3/5	3	3	127	126	2	**0.45**	
					129	128	2	**0.39**	
					131	130	2	**1.02**	
**579**	HNRNPCL1 O60812, HNRNPCL2 B2RXH8: Heterogeneous nuclear ribonucleoprotein C-like 1, Heterogeneous nuclear ribonucleoprotein C-like 2	2/2	2	4	127	126	3	**0.86**	
					129	128	3	**1.13**	
					131	130	3	**1.57**	
**580**	HNRNPD Q14103$_{1,3}$: Heterogeneous nuclear ribonucleoprotein D0	2/2	4	5	127	126	1	**1.50**	
					129	128	1	**0.80**	
					131	130	1	**0.92**	
**581**	HNRNPD Q14103$_{2,4}$: Heterogeneous nuclear ribonucleoprotein D0	2/2	3	4	127	126	1	**0.96**	
					129	128	1	**0.77**	
					131	130	1	**1.10**	
**582**	HNRNPF P52597: Heterogeneous nuclear ribonucleoprotein F		5	7	127	126	5	**1.35**	
					129	128	5	**0.78**	
					131	130	5	**0.50**	
									-5 1 5

#	protein	group	peptides	spectra	ch1	ch2	quant	ratio
**583**	HNRNPH1 P31943: Heterogeneous nuclear ribonucleoprotein H	1/2	4	5	127	126	3	**0.69**
					129	128	3	**0.68**
					131	130	3	**1.08**
**584**	HNRNPK P61978$_3$: Heterogeneous nuclear ribonucleoprotein K	1/3	17	25	127	126	23	**1.03**
					129	128	24	**0.72**
					131	130	23	**1.24**
**585**	HNRNPL P14866$_1$: Heterogeneous nuclear ribonucleoprotein L	1/2	5	5	127	126	5	**0.92**
					129	128	4	**0.57**
					131	130	5	**1.33**
**586**	HNRNPM P52272$_{1,2}$: Heterogeneous nuclear ribonucleoprotein M	2/2	3	3	127	126	3	**0.87**
					129	128	3	**0.28**
					131	130	3	**1.09**
**587**	HNRNPR O43390$_{1-3}$: Heterogeneous nuclear ribonucleoprotein R	3/4	6	6	127	126	3	**0.81**
					129	128	3	**1.01**
					131	130	3	**1.49**
**588**	HNRNPU Q00839$_2$: Heterogeneous nuclear ribonucleoprotein U	1/2	9	10	127	126	9	**1.00**
					129	128	10	**0.41**
					131	130	10	**1.28**
**589**	HNRNPUL2 Q1KMD3: Heterogeneous nuclear ribonucleoprotein U-like protein 2		9	20	127	126	20	**0.72**
					129	128	20	**0.87**
					131	130	20	**1.10**
**590**	HP P00738$_1$: Haptoglobin	1/2	27	82	127	126	43	**1.72**
					129	128	43	**1.91**
					131	130	43	**1.24**
**591**	HPR P00739$_{1,2}$: Haptoglobin-related protein	2/2	23	66	127	126	27	**0.75**
					129	128	27	**0.76**
					131	130	26	**1.61**
**592**	HPX P02790: Hemopexin		11	21	127	126	21	**1.24**
					129	128	19	**1.92**
					131	130	18	**1.33**
**593**	HRC P23327: Sarcoplasmic reticulum histidine-rich calcium-binding protein		4	5	127	126	4	**0.89**
					129	128	5	**2.13**
					131	130	5	**1.34**
**594**	HRG P04196: Histidine-rich glycoprotein		22	192	127	126	181	**1.42**
					129	128	166	**0.88**
					131	130	172	**1.87**

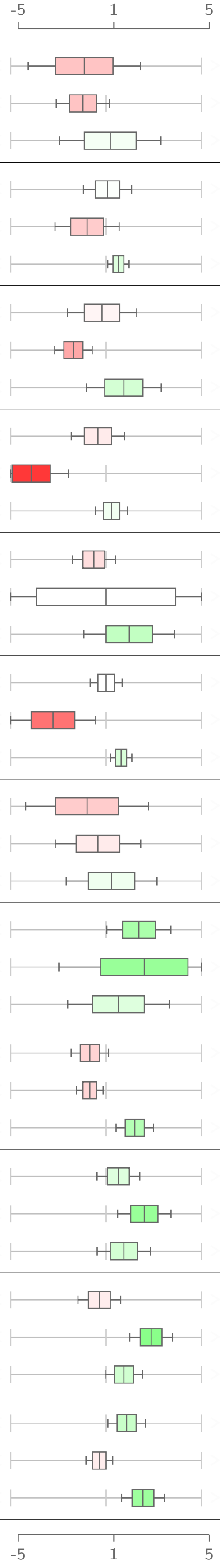

#	protein	group	peptides	spectra	ch1	ch2	quant	ratio
**595**	HRNR Q86YZ3: Hornerin		4	5	127	126	3	**0.46**
					129	128	3	**1.06**
					131	130	4	**0.64**
**596**	HSP90AA1 P07900$_{1,2}$: Heat shock protein HSP 90-alpha	2/7	29	68	127	126	24	**1.09**
					129	128	23	**0.77**
					131	130	24	**1.18**
**597**	HSP90AB1 P08238: Heat shock protein HSP 90-beta	1/3	33	70	127	126	23	**1.19**
					129	128	22	**0.60**
					131	130	24	**1.13**
**598**	HSP90AB4P Q58FF6: Putative heat shock protein HSP 90-beta 4		5	17	127	126	1	**2.68**
					129	128	1	**0.94**
					131	130	1	**0.26**
**599**	HSP90B1 P14625: Endoplasmin	1/2	30	51	127	126	48	**1.07**
					129	128	44	**0.92**
					131	130	49	**0.59**
**600**	HSPA1A P0DMV8$_{1}$, HSPA1B P0DMV9: Heat shock 70 kDa protein 1A, Heat shock 70 kDa protein 1B	2/4	21	41	127	126	9	**0.90**
					129	128	11	**0.43**
					131	130	9	**1.09**
**601**	HSPA1L P34931: Heat shock 70 kDa protein 1-like		13	28	127	126	1	**1.06**
					129	128	1	**1.03**
					131	130	1	**0.66**
**602**	HSPA2 P54652: Heat shock-related 70 kDa protein 2		13	22	127	126	4	**0.99**
					129	128	4	**0.46**
					131	130	4	**1.52**
**603**	HSPA4 P34932$_{1}$: Heat shock 70 kDa protein 4		7	12	127	126	12	**1.93**
					129	128	9	**0.75**
					131	130	12	**0.55**
**604**	HSPA5 P11021: 78 kDa glucose-regulated protein		34	52	127	126	40	**0.92**
					129	128	40	**0.93**
					131	130	37	**0.41**
**606**	HSPA8 P11142$_{1}$: Heat shock cognate 71 kDa protein	1/2	18	30	127	126	9	**0.90**
					129	128	9	**0.40**
					131	130	9	**0.88**
**607**	HSPA9 P38646: Stress-70 protein, mitochondrial		8	13	127	126	9	**0.89**
					129	128	11	**0.29**
					131	130	8	**0.16**

#	protein	group	peptides	spectra	ch1	ch2	quant	ratio
**608**	HSPB1 P04792: Heat shock protein beta-1		9	11	127	126	11	**0.83**
					129	128	11	**0.50**
					131	130	11	**0.92**
**609**	HSPB6 O14558: Heat shock protein beta-6		2	3	127	126	3	**0.85**
					129	128	3	**1.52**
					131	130	3	**1.57**
**610**	HSPD1 P10809$_1$: 60 kDa heat shock protein, mitochondrial	1/2	10	13	127	126	11	**0.78**
					129	128	11	**0.87**
					131	130	13	**0.30**
**611**	HSPE1 P61604: 10 kDa heat shock protein, mitochondrial		2	3	127	126	3	**0.73**
					129	128	3	**0.66**
					131	130	3	**0.05**
**612**	HSPG2 P98160: Basement membrane-specific heparan sulfate proteoglycan core protein		65	81	127	126	75	**1.27**
					129	128	78	**1.05**
					131	130	76	**1.69**
**613**	HTATSF1 O43719: HIV Tat-specific factor 1		15	37	127	126	35	**1.08**
					129	128	33	**0.93**
					131	130	35	**0.63**
**614**	HTRA1 Q92743: Serine protease HTRA1		17	45	127	126	33	**1.22**
					129	128	32	**1.11**
					131	130	32	**1.40**
**615**	HYOU1 Q9Y4L1$_1$: Hypoxia up-regulated protein 1	1/2	10	10	127	126	10	**1.60**
					129	128	10	**1.00**
					131	130	10	**0.68**
**616**	IDH3A P50213$_{1,2}$: Isocitrate dehydrogenase [NAD] subunit alpha, mitochondrial	2/2	2	2	127	126	2	**0.71**
					129	128	2	**0.41**
					131	130	2	**0.45**
**617**	IFI30 P13284: Gamma-interferon-inducible lysosomal thiol reductase		4	18	127	126	14	**0.84**
					129	128	18	**1.14**
					131	130	16	**0.46**
**618**	IFIT1B Q5T764: Interferon-induced protein with tetratricopeptide repeats 1B		4	4	127	126	3	**0.82**
					129	128	3	**1.32**
					131	130	3	**1.62**
**619**	IGF1 P05019$_1$: Insulin-like growth factor I	1/4	5	5	127	126	4	**1.15**
					129	128	4	**1.70**
					131	130	4	**0.94**

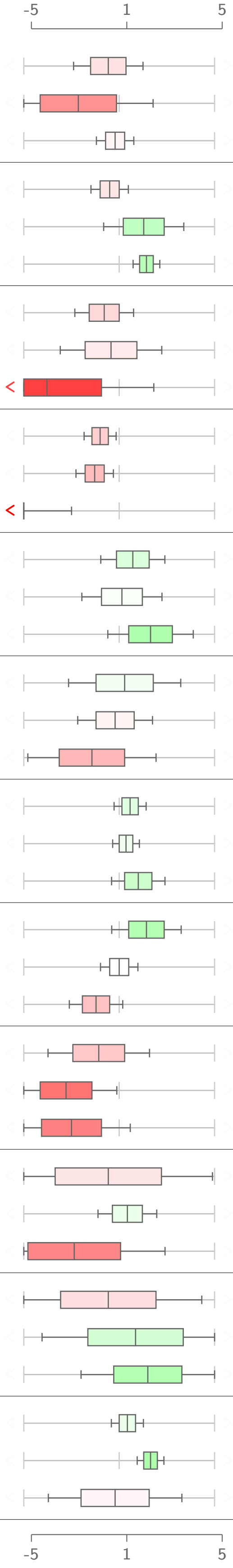

#	protein	group	peptides	spectra	ch1	ch2	quant	ratio
**620**	IGF2 P01344$_{1-3}$: Insulin-like growth factor II	3/3	5	5	127	126	4	**0.59**
					129	128	4	**1.92**
					131	130	4	**1.36**
**621**	IGF2R P11717: Cation-independent mannose-6-phosphate receptor		4	4	127	126	3	**1.01**
					129	128	3	**1.11**
					131	130	3	**0.86**
**622**	IGFALS P35858$_{1,2}$: Insulin-like growth factor-binding protein complex acid labile subunit	2/2	19	24	127	126	23	**1.26**
					129	128	23	**0.85**
					131	130	24	**1.24**
**623**	IGFBP1 P08833: Insulin-like growth factor-binding protein 1		10	15	127	126	13	**0.84**
					129	128	11	**1.24**
					131	130	11	**0.85**
**624**	IGFBP2 P18065: Insulin-like growth factor-binding protein 2		16	32	127	126	32	**1.80**
					129	128	32	**1.87**
					131	130	31	**1.32**
**625**	IGFBP3 P17936$_{1,2}$: Insulin-like growth factor-binding protein 3	2/2	17	34	127	126	26	**0.73**
					129	128	29	**2.03**
					131	130	30	**1.54**
**626**	IGFBP4 P22692$_{1}$: Insulin-like growth factor-binding protein 4	1/2	11	12	127	126	10	**1.26**
					129	128	10	**2.51**
					131	130	11	**1.36**
**627**	IGFBP5 P24593: Insulin-like growth factor-binding protein 5		12	28	127	126	24	**0.82**
					129	128	25	**2.31**
					131	130	24	**1.43**
**628**	IGFBP6 P24592: Insulin-like growth factor-binding protein 6		7	12	127	126	11	**1.31**
					129	128	11	**1.44**
					131	130	10	**0.96**
**629**	IGFBP7 Q16270$_{1,2}$: Insulin-like growth factor-binding protein 7	2/2	8	11	127	126	11	**1.59**
					129	128	11	**1.62**
					131	130	11	**1.06**
**630**	IGHA1 P01876: Ig alpha-1 chain C region		13	74	127	126	17	**0.91**
					129	128	13	**1.33**
					131	130	14	**1.38**
**631**	IGHA2 P01877: Ig alpha-2 chain C region		9	59	127	126	2	**0.98**
					129	128	2	**1.37**
					131	130	2	**0.71**

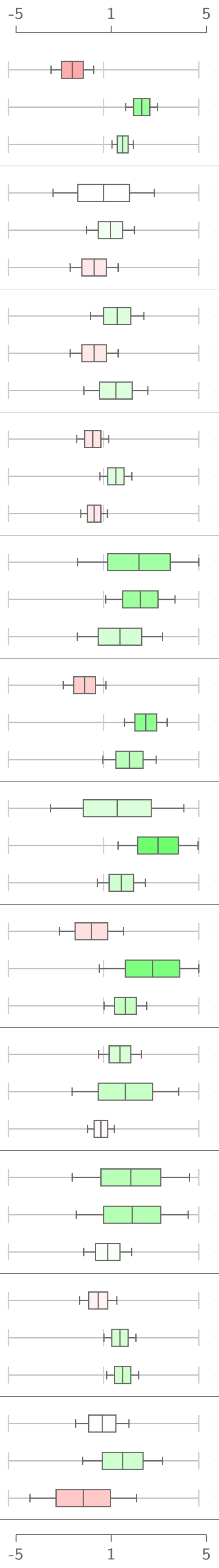

#	protein	group	peptides	spectra	ch1	ch2	quant	ratio
**632**	IGHD P01880$_2$: Ig delta chain C region	1/2	3	5	127	126	4	**0.78**
					129	128	4	**1.04**
					131	130	5	**1.59**
**633**	IGHG1 P01857: Ig gamma-1 chain C region		18	326	127	126	148	**0.90**
					129	128	156	**1.42**
					131	130	156	**1.58**
**634**	IGHG2 P01859: Ig gamma-2 chain C region		13	139	127	126	18	**0.73**
					129	128	19	**1.55**
					131	130	18	**1.43**
**635**	IGHG3 P01860: Ig gamma-3 chain C region		17	230	127	126	77	**0.78**
					129	128	78	**1.62**
					131	130	78	**2.16**
**636**	IGHG4 P01861: Ig gamma-4 chain C region		13	123	127	126	15	**0.96**
					129	128	15	**0.72**
					131	130	15	**1.20**
**637**	IGHM P01871$_1$: Ig mu chain C region	1/2	23	180	127	126	76	**0.36**
					129	128	80	**1.46**
					131	130	75	**1.35**
**638**	IGHV1-2 P23083, A0A0C4DH31: Immunoglobulin heavy variable 1-2	2/2	2	6	127	126	4	**0.93**
					129	128	4	**1.99**
					131	130	3	**1.68**
**639**	IGHV1-69 P01742: Immunoglobulin heavy variable 1-69		3	8	127	126	4	**1.13**
					129	128	5	**1.15**
					131	130	5	**1.87**
**640**	IGHV3-13 P01766: Immunoglobulin heavy variable 3-13		1	1	127	126	1	**0.90**
					129	128	1	**2.29**
					131	130	1	**1.35**
**641**	IGHV3-30 P01768: Immunoglobulin heavy variable 3-30	1/2	2	5	127	126	1	**0.60**
					129	128	1	**1.72**
					131	130	1	**1.62**
**643**	IGHV3-7 P01780: Immunoglobulin heavy variable 3-7	1/5	3	8	127	126	4	**0.65**
					129	128	5	**1.23**
					131	130	4	**1.21**
**644**	IGHV3-9 P01782: Immunoglobulin heavy variable 3-9		1	1	127	126	1	**0.85**
					129	128	1	**1.06**
					131	130	1	**1.18**

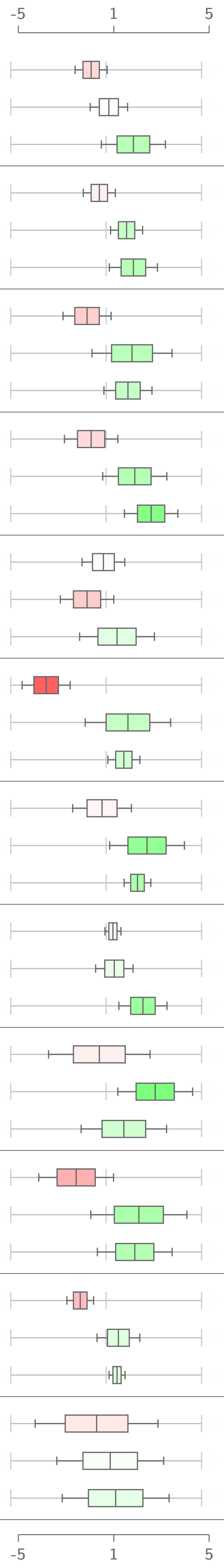

#	protein	group	peptides	spectra	ch1	ch2	quant	ratio
**645**	IGHV4-34 P06331, IGHV4-39 P01824, IGHV4-59 P01825: Immunoglobulin heavy variable 4-34, Immunoglobulin heavy variable 4-39, ...	3/3	3	13	127	126	13	**0.78**
					129	128	11	**1.61**
					131	130	11	**1.61**
**646**	IGKC P01834: Ig kappa chain C region		6	112	127	126	105	**0.83**
					129	128	103	**1.28**
					131	130	107	**1.32**
**647**	IGKV1-17 P01599: Immunoglobulin kappa variable 1-17		3	5	127	126	2	**0.80**
					129	128	2	**2.10**
					131	130	2	**1.66**
**648**	IGKV1D-33 P01593, IGKV1-33 P01594: Immunoglobulin kappa variable 1D-33, Immunoglobulin kappa variable 1-33	2/2	1	3	127	126	3	**1.37**
					129	128	3	**1.26**
					131	130	3	**0.87**
**649**	IGKV1D-12 P01611, IGKV1D-39 P04432, IGKV1-39 P01597, ...: Immunoglobulin kappa variable 1D-12, Immunoglobulin kappa variable 1D-39, ...	4/4	2	4	127	126	1	**0.96**
					129	128	1	**1.28**
					131	130	1	**1.26**
**650**	IGKV1-5 P01602: Immunoglobulin kappa variable 1-5		2	5	127	126	2	**0.63**
					129	128	2	**1.48**
					131	130	2	**1.61**
**651**	IGKV2-30 P06310: Immunoglobulin kappa variable 2-30		2	5	127	126	3	**0.67**
					129	128	3	**1.30**
					131	130	3	**1.43**
**652**	IGKV2D-40 P01614: Immunoglobulin kappa variable 2D-40	1/4	3	16	127	126	14	**0.44**
					129	128	13	**0.68**
					131	130	12	**1.52**
**653**	IGKV3-11 P04433: Immunoglobulin kappa variable 3-11		3	13	127	126	2	**0.46**
					129	128	2	**1.34**
					131	130	2	**1.42**
**654**	IGKV3-15 P01624: Immunoglobulin kappa variable 3-15		4	13	127	126	2	**0.62**
					129	128	2	**2.01**
					131	130	2	**1.70**
**655**	IGKV3-20 P01619: Immunoglobulin kappa variable 3-20		4	24	127	126	2	**0.68**
					129	128	2	**1.71**
					131	130	2	**1.65**
**656**	IGKV4-1 P06312: Immunoglobulin kappa variable 4-1		5	10	127	126	10	**0.71**
					129	128	10	**1.16**
					131	130	10	**0.98**

#	protein	group	peptides	spectra	ch1	ch2	quant	ratio
**657**	IGLC2 P0CG05: Ig lambda-2 chain C regions	1/2	6	220	127	126	45	**0.68**
					129	128	48	**1.50**
					131	130	44	**1.72**
**659**	IGLC7 A0M8Q6: Ig lambda-7 chain C region		5	127	127	126	2	**0.72**
					129	128	2	**1.77**
					131	130	2	**1.66**
**660**	IGLL1 $P15814_1$: Immunoglobulin lambda-like polypeptide 1		2	2	127	126	1	**0.54**
					129	128	1	**1.41**
					131	130	1	**1.19**
**661**	IGLL5 $B9A064_1$: Immunoglobulin lambda-like polypeptide 5	1/2	7	172	127	126	29	**0.63**
					129	128	31	**1.55**
					131	130	31	**1.92**
**662**	IGLV1-47 P01700: Immunoglobulin lambda variable 1-47		1	1	127	126	1	**0.90**
					129	128	1	**1.18**
					131	130	1	**1.49**
**663**	IGLV2-11 P01706: Immunoglobulin lambda variable 2-11		2	6	127	126	5	**0.61**
					129	128	6	**1.61**
					131	130	6	**2.13**
**664**	IGLV2-14 P01704: Immunoglobulin lambda variable 2-14		1	3	127	126	3	**0.92**
					129	128	3	**0.98**
					131	130	3	**0.86**
**665**	IGLV3-21 P80748: Immunoglobulin lambda variable 3-21		2	4	127	126	4	**0.93**
					129	128	3	**3.38**
					131	130	3	**1.63**
**666**	IGLV3-25 P01717: Immunoglobulin lambda variable 3-25		2	4	127	126	4	**0.40**
					129	128	4	**1.80**
					131	130	4	**1.32**
**667**	IGLV7-43 P04211: Immunoglobulin lambda variable 7-43		2	3	127	126	3	**0.65**
					129	128	3	**1.64**
					131	130	3	**1.55**
**668**	ILF3 $Q12906_{1-7}$: Interleukin enhancer-binding factor 3	7/7	5	8	127	126	7	**0.72**
					129	128	7	**0.81**
					131	130	7	**1.22**
**669**	ILK $Q13418_{1-3}$: Integrin-linked protein kinase	3/3	1	1	127	126	1	**1.03**
					129	128	1	**0.81**
					131	130	1	**1.32**

#	protein	group	peptides	spectra	ch1	ch2	quant	ratio
**670**	IMMT Q16891$_{1,2,4}$: MICOS complex subunit MIC60	3/4	7	9	127	126	7	**0.72**
					129	128	9	**0.49**
					131	130	9	**0.09**
**671**	INHBA P08476: Inhibin beta A chain		2	2	127	126	2	**0.67**
					129	128	2	**1.34**
					131	130	2	**1.76**
**672**	INHBB P09529: Inhibin beta B chain		2	2	127	126	2	**1.40**
					129	128	2	**0.75**
					131	130	2	**1.63**
**673**	INHBC P55103: Inhibin beta C chain		9	18	127	126	16	**0.98**
					129	128	17	**1.21**
					131	130	17	**1.51**
**674**	INHBE P58166: Inhibin beta E chain		4	6	127	126	6	**0.49**
					129	128	5	**0.91**
					131	130	5	**1.80**
**675**	IPO5 O00410$_{1,3}$: Importin-5	2/3	4	4	127	126	4	**1.02**
					129	128	4	**0.85**
					131	130	4	**1.20**
**676**	IQGAP1 P46940: Ras GTPase-activating-like protein IQGAP1		23	31	127	126	31	**0.90**
					129	128	27	**0.35**
					131	130	25	**0.97**
**677**	ISLR O14498: Immunoglobulin superfamily containing leucine-rich repeat protein		4	7	127	126	6	**1.15**
					129	128	7	**0.79**
					131	130	7	**0.94**
**678**	ITGA11 Q9UKX5$_{1,2}$: Integrin alpha-11	2/2	3	6	127	126	5	**0.82**
					129	128	6	**1.25**
					131	130	6	**1.97**
**679**	ITGB1 P05556$_{1}$: Integrin beta-1	1/5	9	9	127	126	7	**1.05**
					129	128	7	**0.43**
					131	130	8	**0.67**
**680**	ITGBL1 O95965$_{1}$: Integrin beta-like protein 1	1/3	16	23	127	126	13	**0.50**
					129	128	14	**1.69**
					131	130	14	**1.50**
**681**	ITIH1 P19827$_{1}$: Inter-alpha-trypsin inhibitor heavy chain H1	1/3	28	102	127	126	96	**2.69**
					129	128	94	**0.56**
					131	130	94	**1.67**

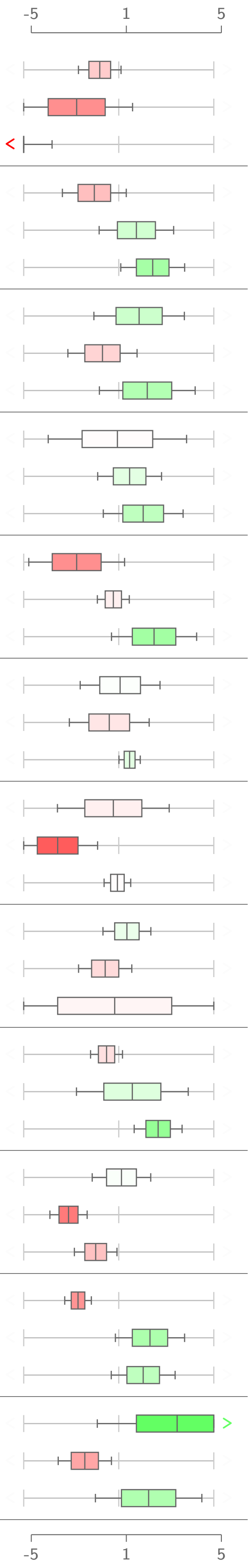

#	protein	group	peptides	spectra	ch1	ch2	quant	ratio
**682**	ITIH2 P19823: Inter-alpha-trypsin inhibitor heavy chain H2		32	273	127	126	265	**2.32**
					129	128	262	**0.67**
					131	130	272	**1.68**
**683**	ITIH3 Q06033$_{1,2}$: Inter-alpha-trypsin inhibitor heavy chain H3	2/2	36	100	127	126	98	**1.88**
					129	128	94	**0.96**
					131	130	99	**1.25**
**684**	ITIH4 Q14624$_{1}$: Inter-alpha-trypsin inhibitor heavy chain H4		38	265	127	126	14	**1.16**
					129	128	15	**2.46**
					131	130	16	**1.44**
**685**	ITIH4 Q14624$_{2,3}$: Inter-alpha-trypsin inhibitor heavy chain H4	2/3	35	251	127	126	2	**2.05**
					129	128	2	**0.74**
					131	130	2	**1.37**
**686**	ITIH5 Q86UX2$_{1}$: Inter-alpha-trypsin inhibitor heavy chain H5	1/4	12	14	127	126	14	**1.17**
					129	128	12	**1.00**
					131	130	14	**1.67**
**687**	ITLN1 Q8WWA0: Intelectin-1	1/3	16	173	127	126	162	**0.23**
					129	128	170	**1.30**
					131	130	162	**1.21**
**688**	ITM2B Q9Y287$_{1,2}$: Integral membrane protein 2B	2/2	2	5	127	126	4	**0.55**
					129	128	5	**1.42**
					131	130	5	**1.40**
**689**	IVL P07476: Involucrin		13	13	127	126	11	**0.76**
					129	128	11	**0.14**
					131	130	11	**1.04**
**690**	JCHAIN P01591: Immunoglobulin J chain		5	23	127	126	21	**0.44**
					129	128	17	**1.85**
					131	130	21	**1.47**
**691**	JUP P14923: Junction plakoglobin	1/2	25	37	127	126	30	**0.43**
					129	128	31	**0.16**
					131	130	28	**0.84**
**692**	KCTD12 Q96CX2: BTB/POZ domain-containing protein KCTD12		6	6	127	126	4	**1.99**
					129	128	6	**0.47**
					131	130	6	**1.00**
**693**	KDM2A Q9Y2K7$_{1}$: Lysine-specific demethylase 2A		4	8	127	126	4	**0.39**
					129	128	4	**0.69**
					131	130	4	**0.52**

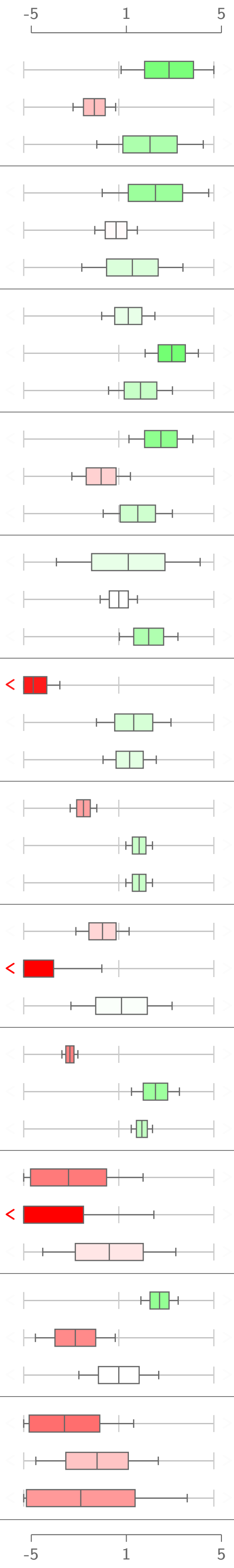

#	protein	group	peptides	spectra	ch1	ch2	quant	ratio
**694**	KHDRBS1 Q07666$_{1-3}$: KH domain-containing, RNA-binding, signal transduction-associated protein 1	3/3	3	3	127	126	3	**0.98**
					129	128	3	**0.47**
					131	130	3	**1.17**
**695**	KIAA1324 Q6UXG2$_3$: UPF0577 protein KIAA1324		2	3	127	126	1	**0.44**
					129	128	1	**2.01**
					131	130	1	**1.20**
**696**	KIF5B P33176: Kinesin-1 heavy chain		10	11	127	126	7	**1.00**
					129	128	7	**0.37**
					131	130	6	**1.31**
**697**	KIF5C O60282$_1$: Kinesin heavy chain isoform 5C		5	6	127	126	2	**0.25**
					129	128	2	**0.18**
					131	130	2	**0.15**
**699**	KLK10 O43240: Kallikrein-10		1	1	127	126	1	**0.78**
					129	128	1	**0.90**
					131	130	1	**1.14**
**700**	KLK7 P49862$_{1,2}$: Kallikrein-7	2/2	3	3	127	126	3	**0.84**
					129	128	3	**0.36**
					131	130	3	**1.11**
**701**	KLKB1 P03952: Plasma kallikrein	1/2	38	86	127	126	80	**0.98**
					129	128	84	**1.53**
					131	130	81	**1.70**
**702**	KMT2A Q03164$_{1-3}$: Histone-lysine N-methyltransferase 2A	3/3	4	32	127	126	11	**0.65**
					129	128	10	**1.14**
					131	130	16	**0.62**
**703**	KNG1 P01042$_1$: Kininogen-1		34	196	127	126	15	**1.35**
					129	128	15	**1.28**
					131	130	15	**1.63**
**704**	KNG1 P01042$_2$: Kininogen-1	1/2	28	201	127	126	20	**1.68**
					129	128	18	**0.98**
					131	130	21	**1.46**
**705**	KNTC1 P50748$_1$: Kinetochore-associated protein 1		4	25	127	126	10	**1.01**
					129	128	10	**1.05**
					131	130	10	**1.14**
**706**	KPNB1 Q14974$_1$: Importin subunit beta-1	1/2	8	10	127	126	10	**0.65**
					129	128	10	**0.59**
					131	130	9	**1.27**

#	protein	group	peptides	spectra	ch1	ch2	quant	ratio
707	KPRP Q5T749: Keratinocyte proline-rich protein		4	4	127	126	3	**0.35**
					129	128	3	**0.19**
					131	130	3	**0.36**
708	KRT1 P04264: Keratin, type II cytoskeletal 1		47	163	127	126	125	**0.30**
					129	128	122	**0.26**
					131	130	126	**0.50**
709	KRT10 P13645: Keratin, type I cytoskeletal 10		35	212	127	126	99	**0.31**
					129	128	99	**0.14**
					131	130	95	**0.45**
710	KRT13 P13646$_1$: Keratin, type I cytoskeletal 13	1/3	16	63	127	126	2	**1.80**
					129	128	3	**1.75**
					131	130	3	**0.97**
711	KRT14 P02533: Keratin, type I cytoskeletal 14		35	201	127	126	23	**0.65**
					129	128	25	**0.11**
					131	130	21	**1.06**
712	KRT15 P19012$_1$: Keratin, type I cytoskeletal 15	1/2	20	92	127	126	8	**0.67**
					129	128	9	**0.12**
					131	130	8	**0.87**
713	KRT16 P08779: Keratin, type I cytoskeletal 16		37	163	127	126	46	**0.64**
					129	128	50	**0.14**
					131	130	44	**0.87**
714	KRT17 Q04695: Keratin, type I cytoskeletal 17		35	184	127	126	54	**0.65**
					129	128	57	**0.11**
					131	130	49	**1.05**
715	KRT18 P05783: Keratin, type I cytoskeletal 18		31	86	127	126	65	**0.69**
					129	128	68	**1.19**
					131	130	66	**1.00**
716	KRT19 P08727: Keratin, type I cytoskeletal 19	1/4	35	157	127	126	70	**0.59**
					129	128	72	**1.49**
					131	130	69	**1.02**
717	KRT2 P35908: Keratin, type II cytoskeletal 2 epidermal		34	110	127	126	40	**0.30**
					129	128	40	**0.45**
					131	130	39	**0.65**
719	KRT23 Q9C075$_1$: Keratin, type I cytoskeletal 23	1/2	3	12	127	126	1	**0.49**
					129	128	1	**0.05**
					131	130	1	**0.07**

#	protein	group	peptides	spectra	ch1	ch2	quant	ratio
**720**	KRT24 Q2M2I5: Keratin, type I cytoskeletal 24		6	30	127	126	1	**0.86**
					129	128	1	**1.34**
					131	130	1	**0.61**
**721**	KRT25 Q7Z3Z0: Keratin, type I cytoskeletal 25		26	153	127	126	44	**0.81**
					129	128	48	**0.07**
					131	130	31	**1.10**
**722**	KRT26 Q7Z3Y9: Keratin, type I cytoskeletal 26		9	42	127	126	7	**0.72**
					129	128	7	**0.02**
					131	130	5	**0.72**
**723**	KRT27 Q7Z3Y8: Keratin, type I cytoskeletal 27		25	170	127	126	46	**0.78**
					129	128	46	**0.08**
					131	130	39	**0.83**
**724**	KRT28 Q7Z3Y7: Keratin, type I cytoskeletal 28		19	122	127	126	12	**0.56**
					129	128	12	**0.05**
					131	130	9	**0.79**
**725**	KRT3 P12035: Keratin, type II cytoskeletal 3		13	77	127	126	1	**0.80**
					129	128	1	**0.09**
					131	130	1	**0.46**
**726**	KRT31 Q15323: Keratin, type I cuticular Ha1	1/2	26	101	127	126	14	**0.05**
					129	128	12	**0.35**
					131	130	15	**1.22**
**727**	KRT32 Q14532: Keratin, type I cuticular Ha2		8	39	127	126	2	**0.03**
					129	128	1	**0.26**
					131	130	2	**0.97**
**728**	KRT33A O76009: Keratin, type I cuticular Ha3-I		28	95	127	126	5	**0.14**
					129	128	4	**0.70**
					131	130	5	**0.82**
**729**	KRT33B Q14525: Keratin, type I cuticular Ha3-II		20	76	127	126	8	**0.05**
					129	128	7	**0.75**
					131	130	8	**0.77**
**730**	KRT34 O76011: Keratin, type I cuticular Ha4	1/2	25	59	127	126	15	**0.04**
					129	128	8	**0.56**
					131	130	13	**1.19**
**731**	KRT35 Q92764: Keratin, type I cuticular Ha5		19	41	127	126	9	**0.03**
					129	128	5	**0.57**
					131	130	9	**0.96**

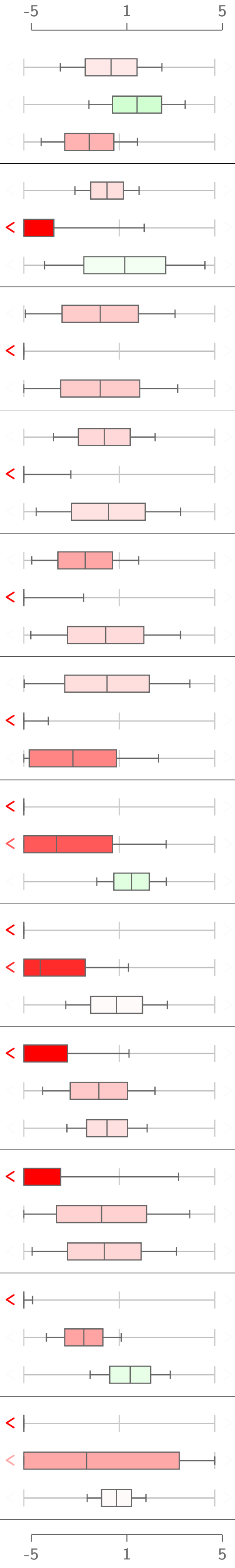

#	protein	group	peptides	spectra	ch1	ch2	quant	ratio
**733**	KRT38 O76015: Keratin, type I cuticular Ha8		3	13	127	126	1	**0.72**
					129	128	1	**0.82**
					131	130	1	**0.39**
**734**	KRT39 Q6A163: Keratin, type I cytoskeletal 39		5	7	127	126	4	**0.05**
					129	128	1	**0.20**
					131	130	2	**1.41**
**736**	KRT5 P13647: Keratin, type II cytoskeletal 5		44	197	127	126	69	**0.69**
					129	128	69	**0.07**
					131	130	72	**0.66**
**737**	KRT6A P02538: Keratin, type II cytoskeletal 6A		45	201	127	126	3	**0.51**
					129	128	4	**0.11**
					131	130	4	**0.70**
**738**	KRT6B P04259: Keratin, type II cytoskeletal 6B		44	198	127	126	2	**0.63**
					129	128	2	**0.10**
					131	130	1	**0.90**
**740**	KRT7 P08729: Keratin, type II cytoskeletal 7		32	113	127	126	49	**0.71**
					129	128	48	**1.46**
					131	130	43	**1.14**
**741**	KRT71 Q3SY84: Keratin, type II cytoskeletal 71		41	236	127	126	97	**0.78**
					129	128	100	**0.09**
					131	130	85	**1.08**
**742**	KRT72 Q14CN4$_1$: Keratin, type II cytoskeletal 72	1/3	17	89	127	126	2	**0.94**
					129	128	2	**0.15**
					131	130	2	**0.58**
**743**	KRT73 Q86Y46$_1$: Keratin, type II cytoskeletal 73	1/2	25	123	127	126	3	**1.16**
					129	128	3	**0.05**
					131	130	3	**0.22**
**744**	KRT74 Q7RTS7: Keratin, type II cytoskeletal 74		34	162	127	126	31	**0.88**
					129	128	31	**0.08**
					131	130	27	**0.98**
**745**	KRT75 O95678: Keratin, type II cytoskeletal 75		37	148	127	126	28	**0.76**
					129	128	25	**0.04**
					131	130	25	**0.96**
**748**	KRT78 Q8N1N4$_1$: Keratin, type II cytoskeletal 78	1/2	6	39	127	126	2	**0.54**
					129	128	2	**0.42**
					131	130	2	**0.71**

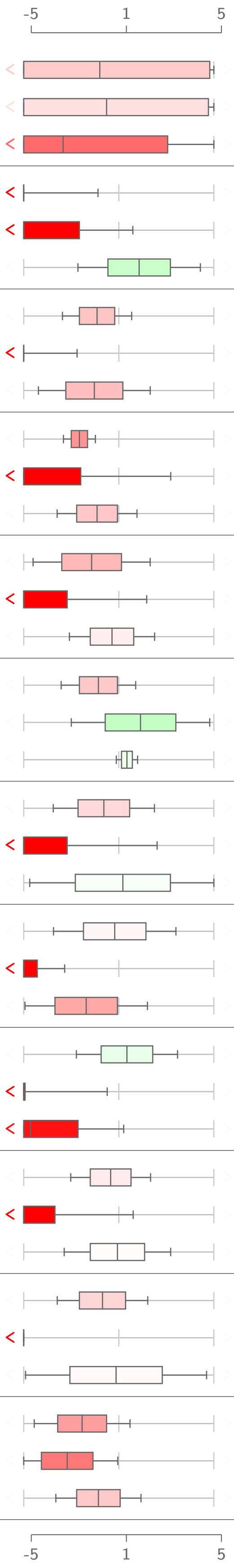

#	protein	group	peptides	spectra	ch1	ch2	quant	ratio
749	KRT79 Q5XKE5: Keratin, type II cytoskeletal 79		19	70	127	126	9	0.96
					129	128	10	0.14
					131	130	10	0.58
750	KRT8 P05787$_{1,2}$: Keratin, type II cytoskeletal 8	2/3	39	158	127	126	74	0.64
					129	128	75	1.70
					131	130	75	1.04
751	KRT80 Q6KB66$_{1,2}$: Keratin, type II cytoskeletal 80	2/3	5	7	127	126	4	0.74
					129	128	4	0.12
					131	130	4	1.71
752	KRT81 Q14533: Keratin, type II cuticular Hb1		35	142	127	126	1	0.04
					129	128		
					131	130	1	0.57
753	KRT82 Q9NSB4: Keratin, type II cuticular Hb2		6	25	127	126	1	0.10
					129	128	1	0.19
					131	130	1	0.36
754	KRT83 P78385: Keratin, type II cuticular Hb3	1/2	36	143	127	126	3	0.09
					129	128	2	0.71
					131	130	3	0.96
755	KRT85 P78386: Keratin, type II cuticular Hb5		40	147	127	126	26	0.05
					129	128	14	0.64
					131	130	21	1.04
756	KRT86 O43790: Keratin, type II cuticular Hb6		42	166	127	126	12	0.10
					129	128	11	0.98
					131	130	12	0.99
757	KRT9 P35527: Keratin, type I cytoskeletal 9		27	94	127	126	63	0.28
					129	128	63	0.57
					131	130	60	0.47
758	KRTAP11-1 Q8IUC1: Keratin-associated protein 11-1		2	3	127	126	3	0.22
					129	128	1	0.41
					131	130	2	1.01
759	KRTAP2-1 Q9BYU5, KRTAP2-2 Q9BYT5, KRTAP2-3 P0C7H8, . . . : Keratin-associated protein 2-1, Keratin-associated protein 2-2, . . .	4/4	2	5	127	126	4	0.02
					129	128	4	0.57
					131	130	4	0.93
761	KRTAP4-3 Q9BYR4: Keratin-associated protein 4-3		1	2	127	126	2	0.01
					129	128		
					131	130	1	1.06

#	protein	group	peptides	spectra	ch1	ch2	quant	ratio
**762**	KRTAP9-2 Q9BYQ4, KRTAP9-8 Q9BYQ0, KRTAP9-9 Q9BYP9$_{1-3}$: Keratin-associated protein 9-2, Keratin-associated protein 9-8, ...	5/9	2	3	127	126	2	**0.75**
					129	128	2	**1.53**
					131	130	2	**1.70**
**763**	LAMA2 P24043: Laminin subunit alpha-2		29	53	127	126	40	**0.59**
					129	128	41	**1.32**
					131	130	44	**1.20**
**764**	LAMA4 Q16363$_{1,2}$: Laminin subunit alpha-4	2/2	14	15	127	126	15	**1.23**
					129	128	15	**1.14**
					131	130	14	**1.72**
**765**	LAMA5 O15230$_1$: Laminin subunit alpha-5		7	12	127	126	10	**0.56**
					129	128	9	**1.67**
					131	130	10	**1.45**
**766**	LAMB1 P07942: Laminin subunit beta-1		24	26	127	126	20	**0.75**
					129	128	22	**1.45**
					131	130	21	**1.55**
**767**	LAMB2 P55268: Laminin subunit beta-2		17	19	127	126	16	**0.78**
					129	128	18	**1.22**
					131	130	18	**1.40**
**768**	LAMC1 P11047: Laminin subunit gamma-1		29	38	127	126	34	**0.76**
					129	128	34	**1.68**
					131	130	34	**1.63**
**769**	LAMP1 P11279$_1$: Lysosome-associated membrane glycoprotein 1		2	2	127	126	2	**0.32**
					129	128	2	**0.19**
					131	130	2	**0.26**
**770**	LASP1 Q14847$_1$: LIM and SH3 domain protein 1	1/3	5	7	127	126	6	**1.20**
					129	128	7	**0.75**
					131	130	5	**0.82**
**771**	LBP P18428: Lipopolysaccharide-binding protein		15	191	127	126	170	**1.24**
					129	128	173	**1.08**
					131	130	176	**2.13**
**772**	LCAT P04180: Phosphatidylcholine-sterol acyltransferase	1/2	11	35	127	126	30	**0.62**
					129	128	28	**1.13**
					131	130	31	**1.72**
**773**	LCP1 P13796$_1$: Plastin-2	1/2	26	59	127	126	44	**2.31**
					129	128	48	**0.33**
					131	130	49	**1.29**

#	protein	group	peptides	spectra	ch1	ch2	quant	ratio
**774**	LDHA P00338$_{1,3}$: L-lactate dehydrogenase A chain	2/8	8	9	127	126	7	**0.86**
					129	128	9	**0.10**
					131	130	9	**0.61**
**775**	LDLR P01130$_{1,5}$: Low-density lipoprotein receptor	2/5	10	26	127	126	16	**0.94**
					129	128	17	**0.97**
					131	130	17	**0.93**
**776**	LECT2 O14960: Leukocyte cell-derived chemotaxin-2		3	3	127	126	3	**0.52**
					129	128	3	**4.69**
					131	130	3	**2.16**
**777**	LEFTY1 O75610, LEFTY2 O00292$_{1}$: Left-right determination factor 1, Left-right determination factor 2	2/2	2	3	127	126	3	**0.81**
					129	128	3	**0.94**
					131	130	3	**1.36**
**778**	LEP P41159: Leptin		3	5	127	126	4	**0.78**
					129	128	3	**0.76**
					131	130	5	**1.91**
**779**	LFNG Q8NES3$_{4}$: Beta-1,3-N-acetylglucosaminyltransferase lunatic fringe	1/4	5	7	127	126	7	**1.08**
					129	128	7	**0.94**
					131	130	6	**1.18**
**780**	LGALS1 P09382: Galectin-1		8	18	127	126	17	**0.74**
					129	128	18	**1.11**
					131	130	17	**1.41**
**781**	LGALS3 P17931: Galectin-3		4	5	127	126	5	**0.62**
					129	128	4	**0.34**
					131	130	4	**1.29**
**782**	LGALS3BP Q08380: Galectin-3-binding protein		16	34	127	126	34	**0.27**
					129	128	32	**1.95**
					131	130	33	**1.58**
**783**	LGALS7 P47929: Galectin-7		4	6	127	126	5	**0.41**
					129	128	6	**0.24**
					131	130	5	**1.04**
**784**	LMAN1 P49257: Protein ERGIC-53		7	11	127	126	11	**0.87**
					129	128	11	**1.03**
					131	130	11	**0.63**
**785**	LMAN2 Q12907: Vesicular integral-membrane protein VIP36		2	3	127	126	3	**0.80**
					129	128	3	**0.64**
					131	130	3	**0.84**

#	protein	group	peptides	spectra	ch1	ch2	quant	ratio
**786**	LMNA P02545$_{1-3,6}$: Prelamin-A/C [Cleaved into: Lamin-A/C	4/6	28	44	127	126	37	**0.76**
					129	128	40	**0.26**
					131	130	36	**0.59**
**787**	LMNB1 P20700: Lamin-B1		7	8	127	126	5	**0.50**
					129	128	5	**0.27**
					131	130	5	**0.56**
**788**	LMNB2 Q03252: Lamin-B2		5	6	127	126	2	**0.70**
					129	128	2	**0.71**
					131	130	2	**0.85**
**789**	LOX P28300: Protein-lysine 6-oxidase		11	19	127	126	14	**0.66**
					129	128	15	**1.30**
					131	130	15	**1.05**
**790**	LOXL1 Q08397: Lysyl oxidase homolog 1		12	16	127	126	11	**0.36**
					129	128	12	**2.73**
					131	130	12	**1.68**
**791**	LOXL2 Q9Y4K0: Lysyl oxidase homolog 2		12	18	127	126	10	**0.55**
					129	128	11	**1.49**
					131	130	12	**1.54**
**792**	LOXL3 P58215$_{1,3}$: Lysyl oxidase homolog 3	2/2	4	7	127	126	3	**0.53**
					129	128	3	**1.62**
					131	130	3	**1.77**
**793**	LPA P08519: Apolipoprotein(a)		20	34	127	126	33	**1.08**
					129	128	30	**0.23**
					131	130	34	**2.32**
**794**	LPAR3 Q9UBY5: Lysophosphatidic acid receptor 3		5	161	127	126	85	**1.61**
					129	128	99	**0.86**
					131	130	108	**0.37**
**795**	LPL P06858: Lipoprotein lipase		3	3	127	126	3	**0.87**
					129	128	3	**1.06**
					131	130	3	**1.41**
**796**	LRG1 P02750: Leucine-rich alpha-2-glycoprotein		6	6	127	126	5	**1.10**
					129	128	5	**2.46**
					131	130	4	**0.92**
**797**	LRP1 Q07954$_{1}$: Prolow-density lipoprotein receptor-related protein 1		50	106	127	126	68	**0.46**
					129	128	74	**1.75**
					131	130	69	**1.05**

#	protein	group	peptides	spectra	ch1	ch2	quant	ratio
**798**	LRP1B Q9NZR2: Low-density lipoprotein receptor-related protein 1B		23	96	127	126	37	**1.15**
					129	128	46	**0.99**
					131	130	48	**0.81**
**799**	LRP2 P98164: Low-density lipoprotein receptor-related protein 2		21	101	127	126	55	**0.98**
					129	128	60	**1.12**
					131	130	64	**0.86**
**800**	LRRC15 Q8TF66$_{1,2}$: Leucine-rich repeat-containing protein 15	2/2	1	1	127	126	1	**0.13**
					129	128	1	**0.02**
					131	130		
**801**	LRRN4 Q8WUT4: Leucine-rich repeat neuronal protein 4		3	3	127	126	3	**2.05**
					129	128	3	**0.62**
					131	130	3	**0.92**
**802**	LSR Q86X29$_{1,4}$: Lipolysis-stimulated lipoprotein receptor	2/6	5	19	127	126	15	**0.90**
					129	128	16	**1.46**
					131	130	17	**1.25**
**803**	LTBP1 Q14766$_{1,4}$: Latent-transforming growth factor beta-binding protein 1	2/4	26	48	127	126	10	**0.80**
					129	128	8	**1.49**
					131	130	8	**1.58**
**804**	LTBP1 Q14766$_3$: Latent-transforming growth factor beta-binding protein 1		22	39	127	126	1	**0.63**
					129	128	1	**1.83**
					131	130	1	**1.90**
**805**	LTBP2 Q14767: Latent-transforming growth factor beta-binding protein 2		27	45	127	126	38	**0.73**
					129	128	43	**1.37**
					131	130	39	**1.54**
**806**	LTBP3 Q9NS15$_1$: Latent-transforming growth factor beta-binding protein 3	1/2	8	8	127	126	5	**0.58**
					129	128	7	**1.79**
					131	130	6	**1.57**
**807**	LTBP4 Q8N2S1$_2$: Latent-transforming growth factor beta-binding protein 4	1/4	28	36	127	126	32	**0.54**
					129	128	35	**1.97**
					131	130	33	**1.39**
**808**	LTF P02788$_1$: Lactotransferrin	1/2	10	15	127	126	15	**0.80**
					129	128	14	**1.69**
					131	130	15	**1.43**
**809**	LUM P51884: Lumican		14	125	127	126	119	**0.97**
					129	128	125	**1.44**
					131	130	119	**1.64**

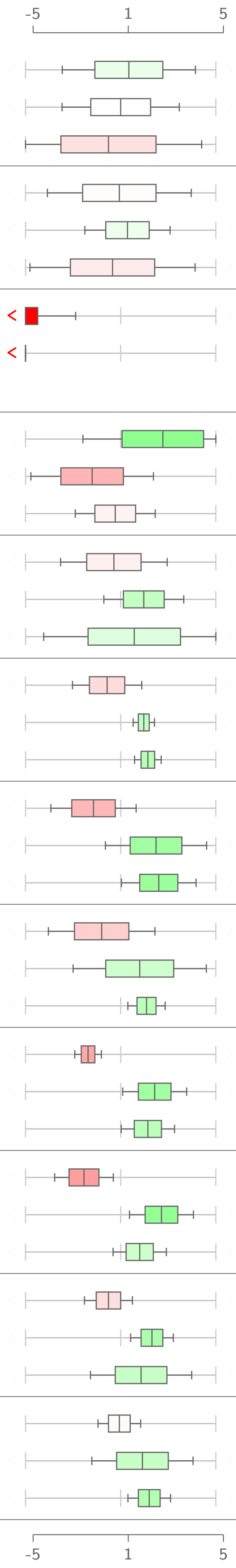

#	protein	group	peptides	spectra	ch1	ch2	quant	ratio
**810**	LYZ P61626: Lysozyme C		13	274	127	126	256	**0.87**
					129	128	250	**1.59**
					131	130	270	**1.52**
**812**	M6PR P20645: Cation-dependent mannose-6-phosphate receptor		2	2	127	126	2	**0.79**
					129	128	2	**0.49**
					131	130	2	**0.91**
**813**	MACF1 Q9UPN3$_1$: Microtubule-actin cross-linking factor 1, isoforms 1/2/3/5		9	21	127	126	13	**1.21**
					129	128	13	**0.86**
					131	130	12	**0.78**
**814**	MALRD1 Q5VYJ5: MAM and LDL-receptor class A domain-containing protein 1		12	85	127	126	73	**1.85**
					129	128	73	**0.90**
					131	130	73	**0.44**
**815**	MAMDC2 Q7Z304$_1$: MAM domain-containing protein 2		1	1	127	126	1	**0.86**
					129	128	1	**0.59**
					131	130	1	**1.13**
**816**	MAN1A1 P33908$_1$: Mannosyl-oligosaccharide 1,2-alpha-mannosidase IA	1/2	12	17	127	126	16	**1.03**
					129	128	14	**0.71**
					131	130	17	**1.23**
**817**	MAP1B P46821: Microtubule-associated protein 1B		6	12	127	126	5	**1.76**
					129	128	6	**0.68**
					131	130	6	**1.07**
**819**	MARCO Q9UEW3$_{1,2}$: Macrophage receptor MARCO	2/2	3	3	127	126	3	**1.29**
					129	128	3	**0.51**
					131	130	3	**1.22**
**820**	MASP1 P48740$_1$: Mannan-binding lectin serine protease 1		16	43	127	126	9	**0.30**
					129	128	7	**2.80**
					131	130	9	**1.35**
**821**	MASP1 P48740$_2$: Mannan-binding lectin serine protease 1	1/2	17	41	127	126	6	**0.31**
					129	128	6	**2.61**
					131	130	6	**2.57**
**822**	MASP1 P48740$_3$: Mannan-binding lectin serine protease 1		11	33	127	126	1	**0.35**
					129	128	1	**2.09**
					131	130	1	**1.32**
**823**	MASP2 O00187$_1$: Mannan-binding lectin serine protease 2		9	19	127	126	3	**0.25**
					129	128	3	**3.01**
					131	130	3	**2.22**

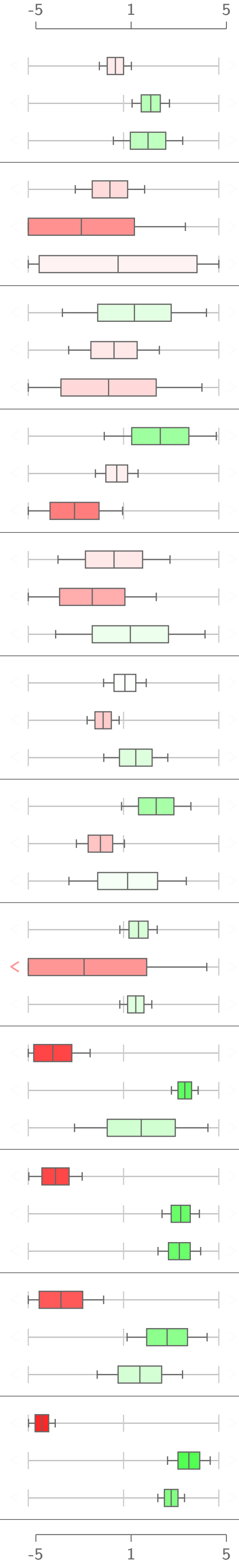

#	protein	group	peptides	spectra	ch1	ch2	quant	ratio
**824**	MASP2 O00187$_2$: Mannan-binding lectin serine protease 2		7	19	127	126	3	**1.12**
					129	128	3	**1.24**
					131	130	3	**0.86**
**825**	MAT2A P31153$_1$: S-adenosylmethionine synthase isoform type-2	1/3	4	5	127	126	5	**0.90**
					129	128	5	**0.35**
					131	130	4	**1.01**
**826**	MATN2 O00339$_{1-3}$: Matrilin-2	3/4	4	5	127	126	5	**0.80**
					129	128	5	**1.07**
					131	130	5	**1.44**
**827**	MATR3 P43243$_1$: Matrin-3	1/2	7	10	127	126	6	**0.99**
					129	128	6	**0.68**
					131	130	6	**1.23**
**828**	MB P02144: Myoglobin		7	8	127	126	8	**0.59**
					129	128	7	**2.73**
					131	130	8	**1.72**
**829**	MBL2 P11226: Mannose-binding protein C		4	4	127	126	4	**1.05**
					129	128	3	**1.29**
					131	130	4	**1.23**
**830**	MCCC2 Q9HCC0$_{1,2}$: Methylcrotonoyl-CoA carboxylase beta chain, mitochondrial	2/2	1	1	127	126	1	**0.51**
					129	128	1	**0.58**
					131	130	1	**0.44**
**831**	MDH1 P40925$_{1-3}$: Malate dehydrogenase, cytoplasmic	3/3	2	3	127	126	3	**1.02**
					129	128	3	**0.74**
					131	130	3	**1.51**
**832**	MDH2 P40926$_1$: Malate dehydrogenase, mitochondrial	1/2	7	7	127	126	7	**0.74**
					129	128	7	**0.71**
					131	130	7	**0.21**
**833**	MDK P21741$_1$: Midkine		3	3	127	126	3	**0.46**
					129	128	3	**2.09**
					131	130	3	**1.32**
**834**	MDN1 Q9NU22: Midasin		11	39	127	126	32	**1.27**
					129	128	31	**1.22**
					131	130	30	**1.16**
**835**	MEGF6 O75095$_{1,2}$: Multiple epidermal growth factor-like domains protein 6	2/2	4	5	127	126	4	**0.64**
					129	128	4	**1.35**
					131	130	4	**1.44**

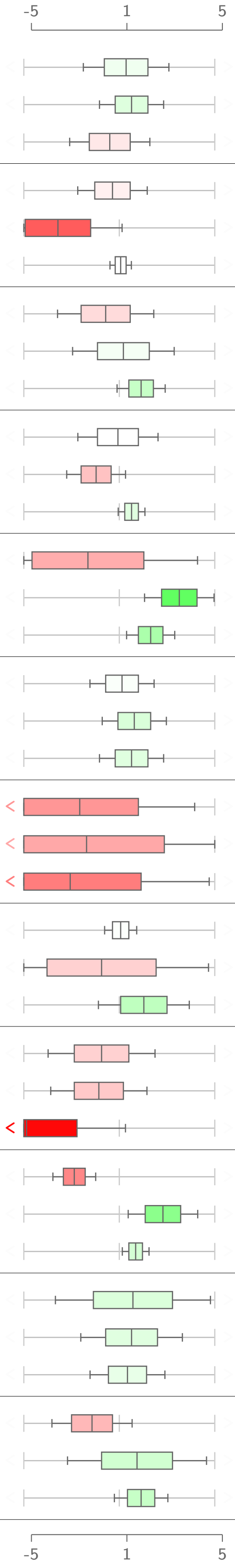

#	protein	group	peptides	spectra	ch1	ch2	quant	ratio
836	METRNL Q641Q3$_{1,2}$: Meteor-in-like protein	2/2	2	2	127	126	2	**1.33**
					129	128	2	**0.76**
					131	130	2	**0.79**
837	MFAP4 P55083$_{1,2}$: Microfibril-associated glycoprotein 4	2/2	6	30	127	126	30	**0.21**
					129	128	27	**2.56**
					131	130	29	**1.72**
838	MFAP5 Q13361$_{1}$: Microfibrillar-associated protein 5	1/2	7	11	127	126	8	**0.67**
					129	128	6	**1.00**
					131	130	9	**1.22**
839	MFGE8 Q08431$_{1,3}$: Lactadherin	2/2	2	2	127	126	2	**0.97**
					129	128	2	**0.81**
					131	130	2	**0.53**
840	MGAT1 P26572: Alpha-1,3-mannosyl-glycoprotein 2-beta-N-acetylglucosaminyltransferase		8	10	127	126	8	**1.63**
					129	128	10	**0.50**
					131	130	10	**1.51**
841	MGP P08493$_{1,2}$: Matrix Gla protein	2/2	5	85	127	126	84	**0.97**
					129	128	81	**1.84**
					131	130	77	**1.59**
842	MINPP1 Q9UNW1$_{1}$: Multiple inositol polyphosphate phosphatase 1	1/4	10	13	127	126	13	**1.61**
					129	128	12	**0.52**
					131	130	11	**1.55**
843	MLEC Q14165: Malectin		6	6	127	126	6	**0.70**
					129	128	5	**0.66**
					131	130	5	**0.48**
844	MMP14 P50281: Matrix metalloproteinase-14		3	3	127	126	3	**1.07**
					129	128	3	**1.68**
					131	130	3	**2.08**
845	MMP19 Q99542$_{1,3}$: Matrix metalloproteinase-19	2/2	3	3	127	126	3	**0.77**
					129	128	3	**1.43**
					131	130	3	**1.49**
846	MMP2 P08253$_{1}$: 72 kDa type IV collagenase	1/3	18	29	127	126	26	**1.03**
					129	128	25	**1.27**
					131	130	22	**1.25**
847	MMRN2 Q9H8L6: Multimerin-2		4	4	127	126	4	**0.59**
					129	128	4	**1.27**
					131	130	4	**1.87**

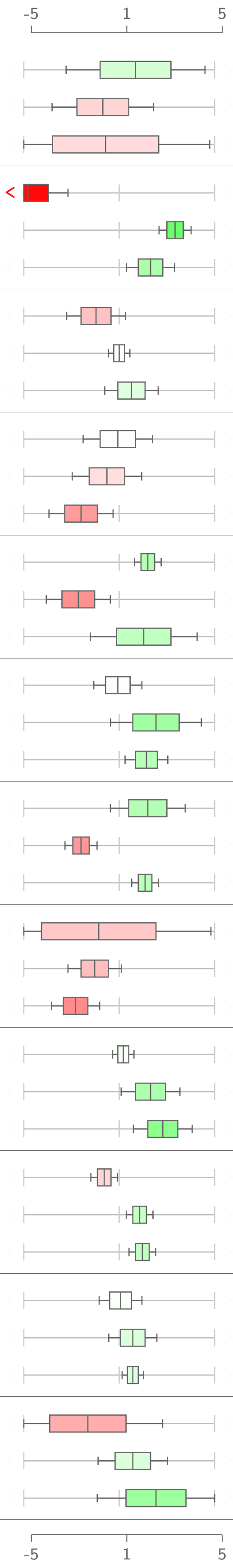

#	protein	group	peptides	spectra	ch1	ch2	quant	ratio
**848**	MPO P05164$_{1-3}$: Myeloperoxidase	3/3	7	8	127	126	7	**0.71**
					129	128	8	**0.52**
					131	130	8	**1.16**
**849**	MPST P25325$_{1,2}$: 3-mercaptopyruvate sulfurtransferase	2/2	2	2	127	126	2	**1.00**
					129	128	2	**0.87**
					131	130	2	**1.17**
**850**	MRC2 Q9UBG0: C-type mannose receptor 2		7	7	127	126	7	**0.92**
					129	128	7	**0.89**
					131	130	7	**0.89**
**851**	MSLN Q13421$_{1-4}$: Mesothelin	4/4	17	30	127	126	30	**0.80**
					129	128	29	**0.92**
					131	130	27	**1.22**
**852**	MSN P26038: Moesin	1/3	17	20	127	126	12	**1.44**
					129	128	12	**0.40**
					131	130	13	**0.47**
**853**	MST1 P26927: Hepatocyte growth factor-like protein	1/3	17	19	127	126	18	**1.34**
					129	128	18	**1.87**
					131	130	17	**1.50**
**854**	MVP Q14764: Major vault protein		9	11	127	126	11	**0.97**
					129	128	11	**0.70**
					131	130	11	**0.98**
**855**	MXRA5 Q9NR99: Matrix-remodeling-associated protein 5		35	60	127	126	59	**0.67**
					129	128	59	**1.23**
					131	130	56	**2.08**
**856**	MXRA7 P84157$_{2}$: Matrix-remodeling-associated protein 7		2	2	127	126	2	**1.19**
					129	128	2	**0.82**
					131	130	2	**0.94**
**857**	MYH10 P35580$_{1,3}$: Myosin-10	2/5	55	66	127	126	34	**1.16**
					129	128	32	**0.55**
					131	130	34	**0.58**
**858**	MYH11 P35749$_{1-4}$: Myosin-11	4/4	21	33	127	126	2	**2.65**
					129	128	2	**1.09**
					131	130	2	**0.30**
**859**	MYH14 Q7Z406$_{1,2,6}$: Myosin-14	3/5	20	24	127	126	9	**0.52**
					129	128	11	**0.34**
					131	130	10	**1.85**

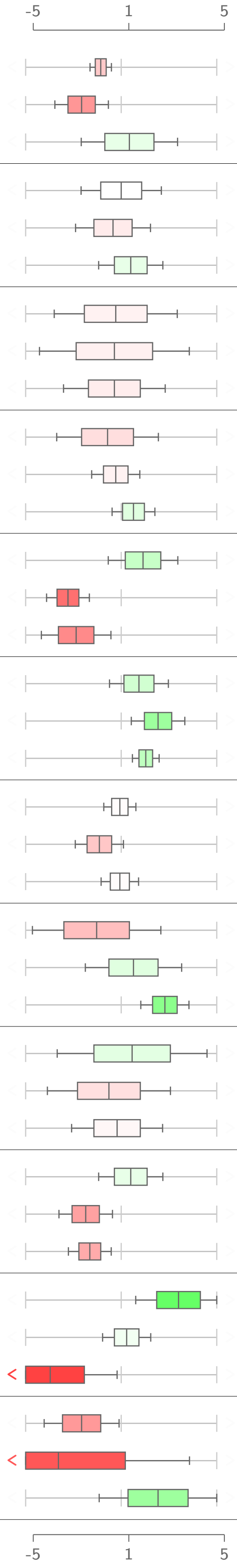

#	protein	group	peptides	spectra	ch1	ch2	quant	ratio
860	MYH3 P11055: Myosin-3	1/7	3	3	127	126	2	**2.09**
					129	128	2	**2.20**
					131	130	2	**1.18**
861	MYH6 P13533: Myosin-6		3	4	127	126	1	**1.33**
					129	128	1	**0.46**
					131	130	1	**0.33**
862	MYH9 P35579$_1$: Myosin-9	1/2	96	157	127	126	117	**1.40**
					129	128	120	**0.30**
					131	130	109	**0.81**
863	MYL1 P05976$_1$: Myosin light chain 1/3, skeletal muscle isoform	1/2	6	7	127	126	7	**0.95**
					129	128	7	**2.75**
					131	130	7	**0.86**
864	MYL12A P19105, MYL12B O14950: Myosin regulatory light chain 12A, Myosin regulatory light chain 12B	2/2	8	17	127	126	8	**0.76**
					129	128	7	**0.65**
					131	130	8	**0.64**
865	MYL2 P10916: Myosin regulatory light chain 2, ventricular/cardiac muscle isoform		7	15	127	126	14	**1.05**
					129	128	15	**3.88**
					131	130	15	**1.20**
866	MYL3 P08590: Myosin light chain 3		6	7	127	126	7	**1.21**
					129	128	7	**4.29**
					131	130	5	**0.97**
867	MYL6 P60660$_{1,2}$: Myosin light polypeptide 6	2/3	6	14	127	126	14	**0.77**
					129	128	14	**0.78**
					131	130	14	**0.76**
868	MYL9 P24844$_1$: Myosin regulatory light polypeptide 9	1/2	6	12	127	126	3	**0.56**
					129	128	3	**2.05**
					131	130	3	**1.18**
869	MYLK Q15746$_{1-6}$: Myosin light chain kinase, smooth muscle	6/11	7	11	127	126	10	**0.74**
					129	128	11	**1.64**
					131	130	11	**2.10**
870	MYLPF Q96A32: Myosin regulatory light chain 2, skeletal muscle isoform		6	6	127	126	4	**1.02**
					129	128	5	**3.44**
					131	130	6	**0.97**
871	MYO1C O00159$_{1-3}$: Unconventional myosin-Ic	3/3	3	3	127	126	3	**1.50**
					129	128	3	**0.42**
					131	130	3	**0.65**

#	protein	group	peptides	spectra	ch1	ch2	quant	ratio
**872**	MYOC Q99972: Myocilin		11	11	127	126	10	**0.90**
					129	128	11	**1.37**
					131	130	10	**1.84**
**873**	NAGK $Q9UJ70_{1,2}$: N-acetyl-D-glucosamine kinase	2/2	1	1	127	126	1	**1.50**
					129	128	1	**0.50**
					131	130	1	**0.75**
**874**	NAGLU P54802: Alpha-N-acetylglucosaminidase		1	1	127	126	1	**0.78**
					129	128	1	**0.77**
					131	130	1	**1.16**
**875**	NAP1L1 $P55209_{1-3}$, NAP1L4 $Q99733_{1,2}$: Nucleosome assembly protein 1-like 1, Nucleosome assembly protein 1-like 4	5/5	1	1	127	126	1	**0.52**
					129	128	1	**0.80**
					131	130	1	**1.27**
**876**	NAPA P54920: Alpha-soluble NSF attachment protein		3	3	127	126	3	**0.52**
					129	128	3	**0.50**
					131	130	3	**1.67**
**877**	NARS $O43776_{1,2}$: Asparagine--tRNA ligase, cytoplasmic	2/2	2	2	127	126	2	**1.29**
					129	128	2	**1.49**
					131	130	2	**1.85**
**878**	NBL1 $P41271_{1,2}$: Neuroblastoma suppressor of tumorigenicity 1	2/2	3	5	127	126	4	**1.63**
					129	128	5	**1.52**
					131	130	5	**1.11**
**879**	NCAM1 $P13591_{1,2}$: Neural cell adhesion molecule 1	2/2	3	21	127	126	10	**1.50**
					129	128	11	**1.32**
					131	130	12	**0.99**
**880**	NCL P19338: Nucleolin		14	14	127	126	13	**0.79**
					129	128	13	**0.52**
					131	130	14	**1.13**
**881**	NDRG1 $Q92597_{1-3}$: Protein NDRG1	3/3	1	1	127	126	1	**0.55**
					129	128	1	**2.13**
					131	130	1	**2.65**
**882**	NDUFS1 $P28331_{1-5}$: NADH-ubiquinone oxidoreductase 75 kDa subunit, mitochondrial	5/5	2	2	127	126	2	**1.25**
					129	128	2	**0.78**
					131	130	2	**0.31**
**883**	NDUFS3 $O75489_{1}$: NADH dehydrogenase [ubiquinone] iron-sulfur protein 3, mitochondrial	1/2	4	4	127	126	4	**0.91**
					129	128	4	**1.17**
					131	130	4	**0.45**

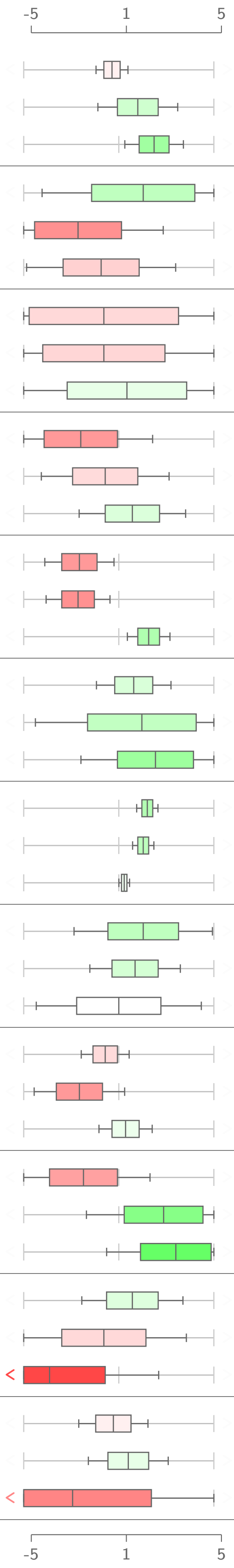

#	protein	group	peptides	spectra	ch1	ch2	quant	ratio
**884**	NECAP2 Q9NVZ3$_{1,2,4}$: Adaptin ear-binding coat-associated protein 2	3/3	2	2	127	126	2	**0.86**
					129	128	2	**0.65**
					131	130	2	**2.17**
**886**	NID1 P14543$_{1}$: Nidogen-1	1/2	21	25	127	126	22	**0.53**
					129	128	24	**1.84**
					131	130	22	**1.91**
**887**	NID2 Q14112$_{1,2}$: Nidogen-2	2/2	10	12	127	126	7	**0.45**
					129	128	7	**2.49**
					131	130	8	**1.76**
**888**	NNMT P40261: Nicotinamide N-methyltransferase		1	1	127	126	1	**1.03**
					129	128	1	**1.14**
					131	130	1	**2.34**
**889**	NONO Q15233$_{1}$: Non-POU domain-containing octamer-binding protein	1/2	6	6	127	126	4	**1.10**
					129	128	4	**0.61**
					131	130	4	**0.76**
**890**	NOTCH3 Q9UM47: Neurogenic locus notch homolog protein 3		9	13	127	126	11	**0.60**
					129	128	12	**1.18**
					131	130	9	**1.85**
**891**	NPM1 P06748$_{1}$: Nucleophosmin	1/3	10	13	127	126	12	**0.49**
					129	128	13	**1.31**
					131	130	12	**1.08**
**892**	NPPA P01160: Natriuretic peptides A		4	5	127	126	5	**1.89**
					129	128	4	**2.20**
					131	130	5	**1.47**
**893**	NQO1 P15559$_{1-3}$: NAD(P)H dehydrogenase [quinone] 1	3/3	3	3	127	126	3	**0.67**
					129	128	3	**0.98**
					131	130	3	**2.38**
**894**	NSF P46459$_{1,2}$: Vesicle-fusing ATPase	2/2	2	2	127	126	2	**0.63**
					129	128	2	**0.54**
					131	130	2	**1.01**
**895**	NUCB1 Q02818: Nucleobindin-1		29	73	127	126	67	**0.35**
					129	128	66	**2.19**
					131	130	66	**1.20**
**896**	NUCB2 P80303$_{1,2}$: Nucleobindin-2	2/2	17	28	127	126	22	**0.38**
					129	128	24	**1.73**
					131	130	19	**0.95**

#	protein	group	peptides	spectra	ch1	ch2	quant	ratio
897	NUCKS1 Q9H1E3$_1$: Nuclear ubiquitous casein and cyclin-dependent kinase substrate 1	1/2	3	3	127	126	3	**0.57**
					129	128	3	**0.72**
					131	130	3	**0.86**
898	NUMA1 Q14980$_{1-4}$: Nuclear mitotic apparatus protein 1	4/4	2	65	127	126	1	**0.93**
					129	128	1	**0.17**
					131	130	1	**2.04**
899	OAF Q86UD1: Out at first protein homolog		8	18	127	126	17	**0.70**
					129	128	17	**1.33**
					131	130	17	**1.73**
900	OBSCN Q5VST9$_2$: Obscurin		3	8	127	126	1	**0.11**
					129	128	1	**0.32**
					131	130	1	**0.62**
901	OGDH Q02218$_{1,2}$: 2-oxoglutarate dehydrogenase, mitochondrial	2/2	3	3	127	126	3	**0.29**
					129	128	3	**0.80**
					131	130	3	**0.52**
902	OGN P20774: Mimecan		11	29	127	126	28	**0.93**
					129	128	27	**1.12**
					131	130	29	**1.44**
903	OIT3 Q8WWZ8$_1$: Oncoprotein-induced transcript 3 protein	1/2	3	4	127	126	4	**0.59**
					129	128	4	**1.57**
					131	130	4	**1.04**
904	OLFML1 Q6UWY5: Olfactomedin-like protein 1		8	9	127	126	7	**0.46**
					129	128	9	**2.08**
					131	130	7	**1.84**
905	OLFML2B Q68BL8$_1$: Olfactomedin-like protein 2B		3	5	127	126	5	**0.99**
					129	128	5	**1.74**
					131	130	5	**1.16**
906	OLFML3 Q9NRN5$_1$: Olfactomedin-like protein 3	1/3	17	42	127	126	38	**0.57**
					129	128	37	**2.23**
					131	130	39	**1.98**
907	OMD Q99983: Osteomodulin		3	4	127	126	3	**0.54**
					129	128	4	**2.00**
					131	130	4	**2.02**
909	ORM1 P02763: Alpha-1-acid glycoprotein 1		11	25	127	126	9	**1.57**
					129	128	9	**1.71**
					131	130	10	**0.58**

#	protein	group	peptides	spectra	ch1	ch2	quant	ratio
**910**	ORM2 P19652: Alpha-1-acid glycoprotein 2		9	21	127	126	5	**1.11**
					129	128	5	**1.44**
					131	130	4	**0.95**
**911**	OTUB1 Q96FW1$_{1}$: Ubiquitin thioesterase OTUB1		3	3	127	126	3	**1.28**
					129	128	3	**0.76**
					131	130	3	**0.85**
**912**	P04220: Ig mu heavy chain disease protein		17	110	127	126	11	**0.37**
					129	128	12	**1.69**
					131	130	12	**1.71**
**913**	P3H1 Q32P28$_{1,3,4}$: Prolyl 3-hydroxylase 1	3/3	2	2	127	126	2	**0.46**
					129	128	2	**1.44**
					131	130	2	**0.71**
**914**	P4HA1 P13674$_{1-3}$: Prolyl 4-hydroxylase subunit alpha-1	3/3	1	1	127	126	1	**0.59**
					129	128	1	**0.90**
					131	130	1	**0.45**
**915**	P4HB P07237: Protein disulfide-isomerase		28	38	127	126	36	**0.67**
					129	128	35	**1.03**
					131	130	34	**0.47**
**916**	PABPC1 P11940$_{1,2}$: Polyadenylate-binding protein 1	2/3	4	6	127	126	6	**0.90**
					129	128	6	**0.50**
					131	130	6	**0.95**
**917**	PACSIN2 Q9UNF0$_{1}$: Protein kinase C and casein kinase substrate in neurons protein 2	1/2	4	7	127	126	5	**0.66**
					129	128	5	**0.95**
					131	130	6	**1.21**
**918**	PAFAH1B2 P68402$_{1}$: Platelet-activating factor acetylhydrolase IB subunit beta		2	2	127	126	2	**1.24**
					129	128	2	**0.77**
					131	130	2	**1.46**
**919**	PAM P19021$_{1,3,5}$: Peptidyl-glycine alpha-amidating monooxygenase	3/6	18	22	127	126	20	**1.12**
					129	128	21	**1.30**
					131	130	21	**1.51**
**921**	PAMR1 Q6UXH9$_{2}$: Inactive serine protease PAMR1		12	28	127	126	1	**0.69**
					129	128	1	**1.73**
					131	130	1	**1.75**
**923**	PAPLN O95428$_{1,5,6}$: Papilin	3/6	15	18	127	126	16	**0.61**
					129	128	16	**1.85**
					131	130	16	**1.78**

#	protein	group	peptides	spectra	ch1	ch2	quant	ratio
**924**	PARK2 O60260$_{1,2,4,6-8}$: E3 ubiquitin-protein ligase parkin	6/6	6	46	127	126	5	**1.01**
					129	128	7	**1.44**
					131	130	5	**1.06**
**925**	PARK7 Q99497: Protein deglycase DJ-1		2	3	127	126	3	**0.70**
					129	128	3	**1.08**
					131	130	3	**1.15**
**926**	PBXIP1 Q96AQ6$_{1,2}$: Pre-B-cell leukemia transcription factor-interacting protein 1	2/2	3	3	127	126	3	**0.63**
					129	128	3	**1.83**
					131	130	3	**1.22**
**927**	PCBP1 Q15365: Poly(rC)-binding protein 1	1/6	8	10	127	126	3	**0.96**
					129	128	4	**0.54**
					131	130	4	**1.46**
**928**	PCBP2 Q15366$_{1-8}$: Poly(rC)-binding protein 2	8/8	7	9	127	126	3	**0.98**
					129	128	3	**0.49**
					131	130	3	**1.25**
**929**	PCMT1 P22061$_{1,2}$: Protein-L-isoaspartate(D-aspartate) O-methyltransferase	2/2	2	2	127	126	2	**0.94**
					129	128	2	**0.53**
					131	130	2	**1.23**
**930**	PCOLCE Q15113: Procollagen C-endopeptidase enhancer 1		20	72	127	126	70	**0.77**
					129	128	71	**2.66**
					131	130	71	**2.07**
**931**	PCOLCE2 Q9UKZ9: Procollagen C-endopeptidase enhancer 2		8	10	127	126	10	**0.85**
					129	128	10	**1.32**
					131	130	10	**1.47**
**932**	PCSK1N Q9UHG2: ProSAAS		5	13	127	126	11	**0.63**
					129	128	12	**1.69**
					131	130	9	**1.23**
**933**	PCSK5 Q92824$_{1}$: Proprotein convertase subtilisin/kexin type 5	1/2	21	92	127	126	58	**0.64**
					129	128	58	**1.42**
					131	130	63	**1.35**
**934**	PCSK9 Q8NBP7$_{1}$: Proprotein convertase subtilisin/kexin type 9	1/2	10	14	127	126	14	**0.59**
					129	128	14	**1.69**
					131	130	13	**1.71**
**935**	PCYOX1 Q9UHG3$_{1}$: Prenylcysteine oxidase 1	1/2	12	14	127	126	13	**0.50**
					129	128	14	**0.71**
					131	130	13	**0.89**

#	protein	group	peptides	spectra	ch1	ch2	quant	ratio
**936**	PDCD6IP Q8WUM4$_{1,2}$: Programmed cell death 6-interacting protein	2/3	13	14	127	126	12	**1.40**
					129	128	12	**0.34**
					131	130	13	**1.45**
**937**	PDGFB P01127$_{1,2}$: Platelet-derived growth factor subunit B	2/2	2	3	127	126	3	**0.77**
					129	128	3	**1.44**
					131	130	3	**1.46**
**938**	PDGFC Q9NRA1$_{1}$: Platelet-derived growth factor C	1/3	4	6	127	126	2	**0.42**
					129	128	2	**1.13**
					131	130	2	**1.25**
**939**	PDGFD Q9GZP0$_{1,2}$: Platelet-derived growth factor D	2/2	3	3	127	126	3	**0.85**
					129	128	3	**2.34**
					131	130	3	**1.84**
**940**	PDGFRL Q15198: Platelet-derived growth factor receptor-like protein		7	7	127	126	6	**0.86**
					129	128	5	**1.94**
					131	130	6	**1.77**
**941**	PDIA3 P30101: Protein disulfide-isomerase A3		14	16	127	126	16	**0.80**
					129	128	16	**0.80**
					131	130	16	**0.24**
**942**	PDIA4 P13667: Protein disulfide-isomerase A4		17	27	127	126	19	**0.62**
					129	128	18	**1.75**
					131	130	16	**0.56**
**943**	PDIA6 Q15084$_{1-5}$: Protein disulfide-isomerase A6	5/5	9	11	127	126	11	**1.34**
					129	128	11	**0.65**
					131	130	11	**0.46**
**944**	PDLIM5 Q96HC4$_{1}$: PDZ and LIM domain protein 5	1/6	7	7	127	126	7	**1.09**
					129	128	7	**0.50**
					131	130	7	**1.04**
**945**	PEA15 Q15121$_{1,2}$: Astrocytic phosphoprotein PEA-15	2/2	3	3	127	126	3	**0.48**
					129	128	3	**1.39**
					131	130	3	**1.10**
**946**	PENK P01210: Proenkephalin-A [Cleaved into: Synenkephalin; Met-enkephalin		8	10	127	126	10	**0.63**
					129	128	7	**1.54**
					131	130	9	**0.87**
**947**	PFN1 P07737: Profilin-1		6	8	127	126	8	**1.00**
					129	128	8	**0.51**
					131	130	8	**1.68**

#	protein	group	peptides	spectra	ch1	ch2	quant	ratio
948	PGA3 P0DJD8, PGA4 P0DJD7, PGA5 P0DJD9: Pepsin A-3, Pepsin A-4, ...	3/3	3	3	127	126	3	**1.09**
					129	128	3	**1.25**
					131	130	3	**0.95**
949	PGAM1 P18669, PGAM2 P15259: Phosphoglycerate mutase 1, Phosphoglycerate mutase 2	2/3	3	3	127	126	3	**0.91**
					129	128	3	**0.27**
					131	130	3	**1.22**
950	PGD $P52209_{1,2}$: 6-phosphogluconate dehydrogenase, decarboxylating	2/2	4	4	127	126	4	**1.09**
					129	128	4	**0.38**
					131	130	3	**1.13**
951	PGK1 $P00558_{1,2}$: Phosphoglycerate kinase 1	2/3	7	9	127	126	9	**0.65**
					129	128	9	**0.37**
					131	130	7	**0.72**
952	PGLYRP2 $Q96PD5_{1,2}$: N-acetylmuramoyl-L-alanine amidase	2/2	10	31	127	126	30	**2.05**
					129	128	26	**0.54**
					131	130	31	**1.76**
953	PHB $P35232_{1}$: Prohibitin		8	8	127	126	8	**0.81**
					129	128	7	**0.36**
					131	130	6	**0.11**
954	PHB2 $Q99623_{1}$: Prohibitin-2	1/2	7	8	127	126	8	**0.78**
					129	128	8	**0.29**
					131	130	8	**0.15**
955	PHGDH O43175: D-3-phosphoglycerate dehydrogenase		7	10	127	126	9	**1.17**
					129	128	8	**0.84**
					131	130	10	**1.67**
957	PICALM $Q13492_{1-5}$: Phosphatidylinositol-binding clathrin assembly protein	5/5	3	3	127	126	3	**1.31**
					129	128	3	**0.60**
					131	130	3	**1.49**
958	PIGR P01833: Polymeric immunoglobulin receptor		8	10	127	126	10	**0.54**
					129	128	9	**1.19**
					131	130	9	**1.29**
959	PKM $P14618_{1}$: Pyruvate kinase PKM	1/5	23	35	127	126	35	**1.28**
					129	128	35	**0.56**
					131	130	29	**1.28**
960	PKP1 $Q13835_{2}$: Plakophilin-1	1/2	22	37	127	126	37	**0.68**
					129	128	36	**0.22**
					131	130	35	**0.68**

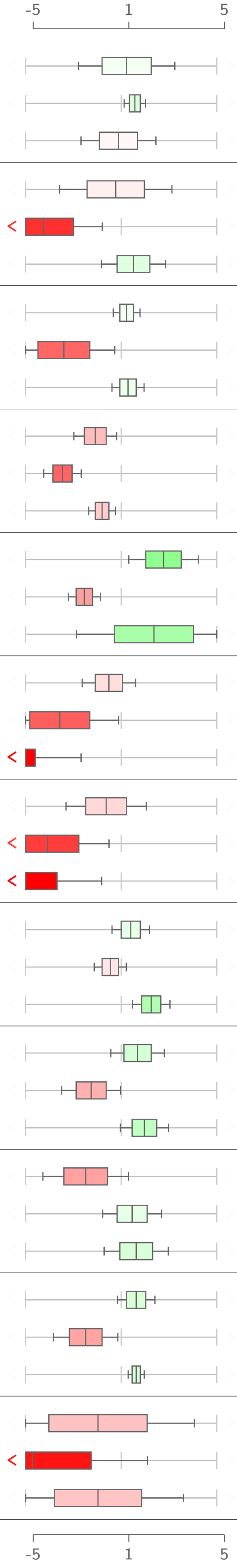

#	protein	group	peptides	spectra	ch1	ch2	quant	ratio
**961**	PKP3 Q9Y446$_{1,2}$: Plakophilin-3	2/2	5	5	127	126	5	**0.86**
					129	128	5	**0.64**
					131	130	5	**0.87**
**962**	PLA2G2A P14555: Phospholipase A2, membrane associated		8	36	127	126	23	**0.50**
					129	128	24	**2.05**
					131	130	24	**1.28**
**963**	PLA2G7 Q13093: Platelet-activating factor acetylhydrolase		2	2	127	126	2	**0.42**
					129	128	2	**0.85**
					131	130	2	**1.58**
**964**	PLAT P00750$_{1}$: Tissue-type plasminogen activator	1/4	13	19	127	126	17	**0.89**
					129	128	19	**1.39**
					131	130	19	**0.71**
**965**	PLEC Q15149$_{5}$: Plectin	1/9	137	166	127	126	151	**1.22**
					129	128	151	**0.31**
					131	130	153	**0.86**
**966**	PLG P00747: Plasminogen	1/2	59	768	127	126	654	**1.38**
					129	128	645	**0.88**
					131	130	679	**1.14**
**967**	PLGLB1 Q02325: Plasminogen-like protein B		6	45	127	126	1	**1.17**
					129	128	1	**0.60**
					131	130	1	**0.72**
**968**	PLIN3 O60664$_{1,3}$: Perilipin-3	2/3	2	2	127	126	2	**1.48**
					129	128	2	**0.71**
					131	130	2	**1.62**
**969**	PLIN4 Q96Q06$_{1,2}$: Perilipin-4	2/2	6	7	127	126	7	**0.86**
					129	128	7	**0.77**
					131	130	7	**3.74**
**970**	PLOD1 Q02809$_{1,2}$: Procollagen-lysine,2-oxoglutarate 5-dioxygenase 1	2/2	4	4	127	126	4	**0.66**
					129	128	4	**1.30**
					131	130	4	**1.03**
**971**	PLOD3 O60568: Procollagen-lysine,2-oxoglutarate 5-dioxygenase 3		3	3	127	126	3	**0.62**
					129	128	3	**1.44**
					131	130	3	**1.08**
**972**	PLS3 P13797$_{1-3}$: Plastin-3	3/3	12	16	127	126	8	**1.81**
					129	128	7	**0.33**
					131	130	8	**0.98**

#	protein	group	peptides	spectra	ch1	ch2	quant	ratio
**973**	PLTP P55058$_1$: Phospholipid transfer protein	1/4	18	170	127	126	167	**1.20**
					129	128	160	**0.70**
					131	130	168	**1.57**
**974**	PODN Q7Z5L7$_{3,4}$: Podocan	2/4	9	14	127	126	13	**1.12**
					129	128	11	**1.04**
					131	130	14	**1.21**
**975**	POF1B Q8WVV4$_{1,2}$: Protein POF1B	2/2	4	4	127	126	4	**0.32**
					129	128	3	**0.05**
					131	130	4	**0.71**
**976**	PON1 P27169: Serum paraoxonase/arylesterase 1	1/4	15	88	127	126	81	**0.43**
					129	128	75	**1.32**
					131	130	84	**1.54**
**977**	PON3 Q15166: Serum paraoxonase/lactonase 3		5	9	127	126	6	**0.50**
					129	128	5	**1.21**
					131	130	5	**1.27**
**978**	POSTN Q15063$_1$: Periostin		48	194	127	126	4	**2.17**
					129	128	4	**0.63**
					131	130	3	**2.19**
**985**	POTEA Q6S8J7$_{1,2}$: POTE ankyrin domain family member A	2/2	4	55	127	126	44	**2.56**
					129	128	41	**0.92**
					131	130	43	**0.33**
**986**	POTEB Q6S5H4$_{1,3}$, POTEB3 A0JP26, POTEC B2RU33: POTE ankyrin domain family member B, POTE ankyrin domain family member B3, ...	4/4	6	20	127	126	2	**0.10**
					129	128	3	**0.75**
					131	130	3	**1.25**
**987**	POTEE Q6S8J3$_1$: POTE ankyrin domain family member E		18	144	127	126	29	**1.03**
					129	128	31	**1.15**
					131	130	37	**0.74**
**988**	POTEF A5A3E0: POTE ankyrin domain family member F	1/3	15	107	127	126	3	**1.09**
					129	128	6	**1.30**
					131	130	6	**1.18**
**989**	POTEKP Q9BYX7: Putative beta-actin-like protein 3		6	31	127	126	1	**1.15**
					129	128	1	**0.99**
					131	130	1	**0.71**
**990**	PPA1 Q15181: Inorganic pyrophosphatase		4	4	127	126	4	**0.65**
					129	128	3	**0.40**
					131	130	3	**1.36**

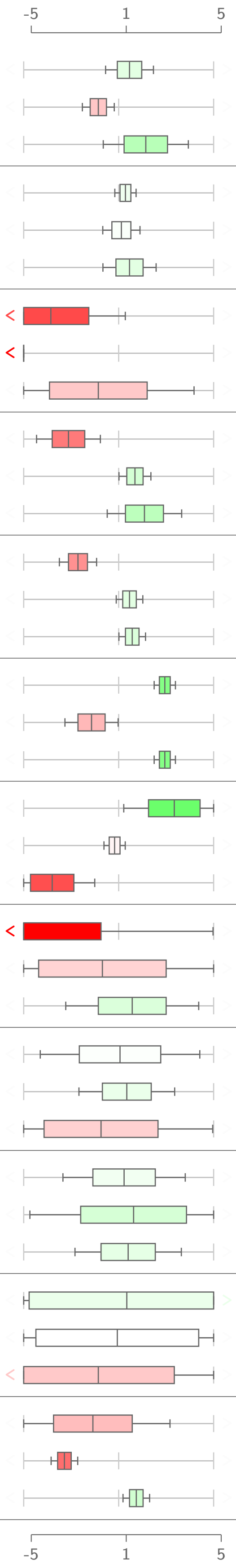

#	protein	group	peptides	spectra	ch1	ch2	quant	ratio
**991**	PPBP P02775: Platelet basic protein		1	1	127	126	1	**2.29**
					129	128	1	**2.19**
					131	130	1	**0.85**
**992**	PPIA P62937$_1$: Peptidyl-prolyl cis-trans isomerase A	1/8	10	15	127	126	13	**0.52**
					129	128	12	**0.48**
					131	130	13	**0.95**
**993**	PPIB P23284: Peptidyl-prolyl cis-trans isomerase B		5	6	127	126	4	**0.86**
					129	128	3	**0.49**
					131	130	3	**0.08**
**994**	PPIC P45877: Peptidyl-prolyl cis-trans isomerase C		2	3	127	126	1	**1.20**
					129	128	1	**0.77**
					131	130	1	**1.11**
**995**	PPL O60437: Periplakin		25	27	127	126	25	**0.78**
					129	128	24	**0.14**
					131	130	24	**0.81**
**996**	PPP1CA P62136$_{1-3}$, PPP1CB P62140, PPP1CC P36873$_{1,2}$: Serine/threonine-protein phosphatase PP1-alpha catalytic subunit, Serine/threonine-protein phosphatase PP1-beta catalytic subunit, . . .	6/6	5	5	127	126	5	**0.67**
					129	128	5	**0.42**
					131	130	4	**1.04**
**997**	PPP1R7 Q15435$_{1-5}$: Protein phosphatase 1 regulatory subunit 7	5/5	1	1	127	126	1	**0.56**
					129	128	1	**1.70**
					131	130	1	**1.61**
**998**	PPP2CA P67775$_{1,2}$: Serine/threonine-protein phosphatase 2A catalytic subunit alpha isoform	2/3	3	3	127	126	3	**0.67**
					129	128	3	**0.49**
					131	130	3	**1.01**
**999**	PPP2R1A P30153: Serine/threonine-protein phosphatase 2A 65 kDa regulatory subunit A alpha is . . .		7	7	127	126	7	**0.69**
					129	128	7	**0.33**
					131	130	7	**1.26**
**1000**	PPP4R3B Q5MIZ7$_{1-3,5}$: Serine/threonine-protein phosphatase 4 regulatory subunit 3B	4/4	2	54	127	126	8	**0.74**
					129	128	12	**1.14**
					131	130	10	**1.01**
**1001**	PRAP1 Q96NZ9$_1$: Proline-rich acidic protein 1	1/3	6	19	127	126	1	**1.36**
					129	128	1	**0.64**
					131	130	1	**1.05**
**1002**	PRAP1 Q96NZ9$_3$: Proline-rich acidic protein 1		6	19	127	126	1	**1.75**
					129	128	1	**1.48**
					131	130	1	**1.24**

#	protein	group	peptides	spectra	ch1	ch2	quant	ratio
**1003**	PRDM1 O75626$_1$: PR domain zinc finger protein 1		3	9	127	126	1	**1.42**
					129	128	1	**0.48**
					131	130	1	**0.34**
**1004**	PRDM1 O75626$_2$: PR domain zinc finger protein 1		4	21	127	126	2	**0.33**
					129	128	2	**1.10**
					131	130	2	**0.94**
**1005**	PRDX1 Q06830: Peroxiredoxin-1	1/2	7	8	127	126	7	**1.40**
					129	128	6	**0.25**
					131	130	7	**1.01**
**1006**	PRDX2 P32119$_1$: Peroxiredoxin-2		6	8	127	126	7	**1.31**
					129	128	6	**0.12**
					131	130	7	**1.12**
**1007**	PRDX5 P30044$_{1,2}$: Peroxiredoxin-5, mitochondrial	2/4	2	2	127	126	2	**1.17**
					129	128	2	**0.75**
					131	130	2	**0.76**
**1008**	PRDX6 P30041: Peroxiredoxin-6		11	17	127	126	16	**1.11**
					129	128	16	**1.06**
					131	130	17	**2.35**
**1009**	PRELP P51888: Prolargin		8	10	127	126	9	**1.03**
					129	128	10	**0.93**
					131	130	7	**1.11**
**1010**	PRG3 Q9Y2Y8: Proteoglycan 3		1	1	127	126	1	**0.88**
					129	128	1	**1.80**
					131	130	1	**1.57**
**1011**	PRG4 Q92954$_{1,6}$: Proteoglycan 4	2/4	47	202	127	126	6	**1.23**
					129	128	6	**0.32**
					131	130	6	**0.56**
**1012**	PRG4 Q92954$_3$: Proteoglycan 4	1/2	47	205	127	126	3	**0.40**
					129	128	4	**1.09**
					131	130	4	**1.85**
**1013**	PRKAR2A P13861$_{1,2}$: cAMP-dependent protein kinase type II-alpha regulatory subunit	2/2	2	2	127	126	2	**0.83**
					129	128	2	**0.58**
					131	130	2	**1.26**
**1014**	PRKCSH P14314$_{1,2}$: Glucosidase 2 subunit beta	2/2	10	16	127	126	14	**0.51**
					129	128	15	**1.74**
					131	130	16	**0.65**

#	protein	group	peptides	spectra	ch1	ch2	quant	ratio
**1015**	PRKDC P78527$_{1,2}$: DNA-dependent protein kinase catalytic subunit	2/2	2	2	127	126	2	**0.94**
					129	128	2	**0.33**
					131	130	2	**1.42**
**1016**	PRNP P04156$_{1,2}$: Major prion protein	2/2	3	3	127	126	3	**1.45**
					129	128	3	**1.37**
					131	130	3	**1.36**
**1017**	PROC P04070$_{1,2}$: Vitamin K-dependent protein C	2/2	11	21	127	126	21	**0.72**
					129	128	18	**2.42**
					131	130	20	**2.04**
**1018**	PROS1 P07225: Vitamin K-dependent protein S		27	81	127	126	70	**0.53**
					129	128	76	**1.82**
					131	130	69	**2.47**
**1019**	PROZ P22891$_{1,2}$: Vitamin K-dependent protein Z	2/2	3	4	127	126	3	**0.94**
					129	128	3	**1.43**
					131	130	3	**1.81**
**1021**	PRSS1 P07477: Trypsin-1		4	22	127	126	2	**2.97**
					129	128	2	**2.94**
					131	130	2	**1.48**
**1022**	PRSS2 P07478: Trypsin-2	1/7	5	25	127	126	5	**1.58**
					129	128	6	**2.23**
					131	130	6	**1.52**
**1023**	PRSS23 O95084$_{1}$: Serine protease 23		2	2	127	126	1	**0.81**
					129	128	1	**1.52**
					131	130	1	**2.15**
**1024**	PSAP P07602$_{1-3}$: Prosaposin	3/3	13	22	127	126	19	**0.37**
					129	128	22	**1.27**
					131	130	20	**1.11**
**1025**	PSAPL1 Q6NUJ1: Proactivator polypeptide-like 1 [Cleaved into: Saposin A-like; Saposin B-Val . . .		2	2	127	126	2	**0.59**
					129	128	2	**1.61**
					131	130	2	**2.29**
**1026**	PSMA1 P25786$_{1,2}$: Proteasome subunit alpha type-1	2/2	2	2	127	126	2	**0.61**
					129	128	2	**0.84**
					131	130	2	**1.08**
**1027**	PSMA3 P25788$_{1,2}$: Proteasome subunit alpha type-3	2/2	2	2	127	126	2	**0.48**
					129	128	2	**0.58**
					131	130	2	**1.07**

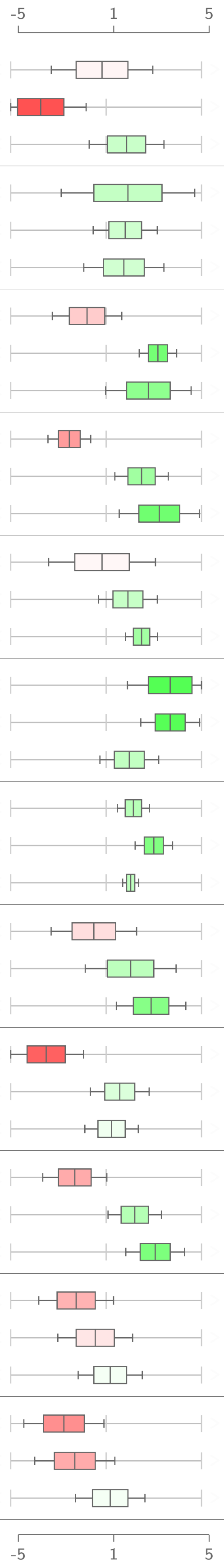

#	protein	group	peptides	spectra	ch1	ch2	quant	ratio
**1028**	PSMA4 P25789$_1$: Proteasome subunit alpha type-4	1/2	5	5	127	126	3	**0.55**
					129	128	5	**0.72**
					131	130	3	**1.33**
**1029**	PSMA7 O14818$_1$: Proteasome subunit alpha type-7	1/5	4	4	127	126	3	**0.50**
					129	128	4	**0.47**
					131	130	4	**0.96**
**1030**	PSMB1 P20618: Proteasome subunit beta type-1		1	1	127	126	1	**0.78**
					129	128	1	**0.82**
					131	130	1	**0.95**
**1031**	PSMB2 P49721: Proteasome subunit beta type-2		3	3	127	126	2	**0.75**
					129	128	2	**0.49**
					131	130	2	**1.05**
**1032**	PSMB5 P28074$_{1,2}$: Proteasome subunit beta type-5	2/2	2	2	127	126	2	**0.95**
					129	128	2	**0.41**
					131	130	2	**0.86**
**1033**	PSMB7 Q99436$_1$: Proteasome subunit beta type-7	1/2	2	2	127	126	2	**0.47**
					129	128	2	**0.42**
					131	130	2	**1.04**
**1034**	PSMC1 P62191$_1$: 26S protease regulatory subunit 4		4	4	127	126	2	**0.68**
					129	128	2	**0.29**
					131	130	2	**1.07**
**1035**	PSMC1 P62191$_2$: 26S protease regulatory subunit 4		3	3	127	126	1	**0.79**
					129	128	1	**0.28**
					131	130		
**1036**	PSMC3 P17980: 26S protease regulatory subunit 6A		4	4	127	126	4	**0.82**
					129	128	3	**0.54**
					131	130	4	**1.09**
**1037**	PSMC6 P62333: 26S protease regulatory subunit 10B		3	3	127	126	3	**0.97**
					129	128	3	**0.33**
					131	130	3	**1.11**
**1038**	PSMD1 Q99460$_{1,2}$: 26S proteasome non-ATPase regulatory subunit 1	2/2	5	5	127	126	4	**1.07**
					129	128	5	**0.37**
					131	130	4	**1.01**
**1039**	PSMD3 O43242$_1$: 26S proteasome non-ATPase regulatory subunit 3		3	3	127	126	3	**0.83**
					129	128	3	**1.25**
					131	130	3	**1.39**

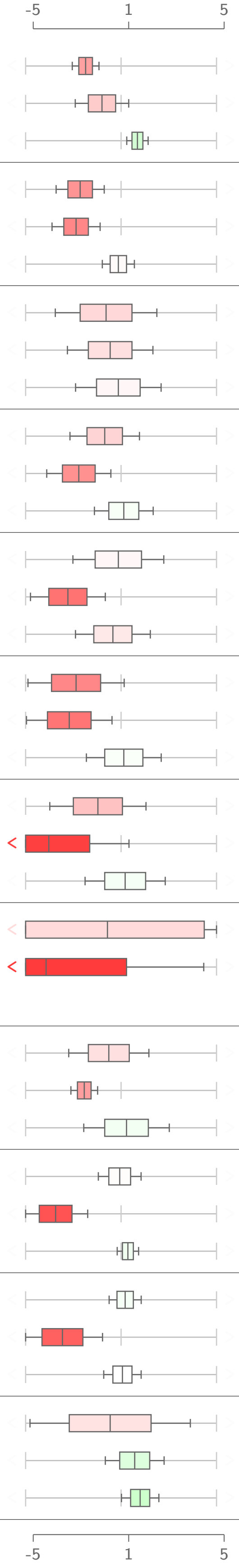

#	protein	group	peptides	spectra	ch1	ch2	quant	ratio
**1040**	PSMD6 Q15008$_{1-4}$: 26S proteasome non-ATPase regulatory subunit 6	4/4	1	1	127	126	1	**1.28**
					129	128	1	**0.18**
					131	130	1	**1.09**
**1041**	PSME1 Q06323$_{1-3}$: Proteasome activator complex subunit 1	3/3	5	5	127	126	5	**1.65**
					129	128	4	**0.73**
					131	130	4	**1.62**
**1042**	PSME2 Q9UL46: Proteasome activator complex subunit 2		6	9	127	126	7	**1.38**
					129	128	6	**0.60**
					131	130	6	**1.36**
**1043**	PTBP1 P26599$_{1-3}$: Polypyrimidine tract-binding protein 1	3/10	3	4	127	126	4	**0.87**
					129	128	4	**0.72**
					131	130	3	**1.48**
**1044**	PTGDS P41222: Prostaglandin-H2 D-isomerase		4	6	127	126	5	**0.69**
					129	128	5	**2.19**
					131	130	5	**1.55**
**1045**	PTGIS Q16647: Prostacyclin synthase		2	2	127	126	2	**1.43**
					129	128	2	**0.92**
					131	130	2	**0.32**
**1046**	PTMA P06454$_{1,2}$: Prothymosin alpha [Cleaved into: Prothymosin alpha, N-terminally processed; . . .	2/2	1	1	127	126	1	**0.67**
					129	128	1	**0.40**
					131	130	1	**0.83**
**1047**	PTMS P20962: Parathymosin		2	3	127	126	3	**0.25**
					129	128	3	**1.00**
					131	130	3	**1.10**
**1048**	PTPRF P10586$_{1,2}$: Receptor-type tyrosine-protein phosphatase F	2/2	2	2	127	126	2	**0.69**
					129	128	2	**0.98**
					131	130	2	**1.59**
**1049**	PTRF Q6NZI2$_{1}$: Polymerase I and transcript release factor	1/3	9	10	127	126	9	**0.71**
					129	128	10	**1.35**
					131	130	10	**0.72**
**1050**	PURA Q00577: Transcriptional activator protein Pur-alpha		2	2	127	126	2	**0.75**
					129	128	2	**0.51**
					131	130	2	**1.10**
**1051**	PXDN Q92626$_{1}$: Peroxidasin homolog	1/2	27	35	127	126	31	**0.78**
					129	128	31	**1.36**
					131	130	30	**1.62**

#	protein	group	peptides	spectra	ch1	ch2	quant	ratio
**1052**	PZP P20742$_1$: Pregnancy zone protein	1/2	31	42	127	126	26	**0.74**
					129	128	26	**1.55**
					131	130	25	**1.14**
**1053**	QPCT Q16769$_1$: Glutaminyl-peptide cyclotransferase	1/2	3	4	127	126	3	**0.57**
					129	128	3	**2.10**
					131	130	3	**1.86**
**1054**	QSOX1 O00391$_1$: Sulfhydryl oxidase 1	1/2	35	82	127	126	75	**1.37**
					129	128	62	**0.81**
					131	130	80	**1.75**
**1055**	RAB10 P61026: Ras-related protein Rab-10		4	9	127	126	7	**0.78**
					129	128	8	**0.64**
					131	130	7	**0.71**
**1056**	RAB11B Q15907$_1$: Ras-related protein Rab-11B	1/4	3	4	127	126	4	**1.01**
					129	128	4	**0.38**
					131	130	3	**0.96**
**1057**	RAB14 P61106: Ras-related protein Rab-14		3	3	127	126	3	**0.73**
					129	128	3	**0.21**
					131	130	3	**0.83**
**1058**	RAB1B Q9H0U4: Ras-related protein Rab-1B	1/2	5	7	127	126	7	**0.96**
					129	128	7	**0.28**
					131	130	7	**0.72**
**1060**	RAB7A P51149: Ras-related protein Rab-7a		4	4	127	126	3	**0.85**
					129	128	4	**0.39**
					131	130	3	**1.01**
**1061**	RAB8B Q92930: Ras-related protein Rab-8B		2	2	127	126	1	**1.13**
					129	128	1	**0.34**
					131	130	1	**0.86**
**1062**	RABEP1 Q15276$_{1,2}$: Rab GTPase-binding effector protein 1	2/2	5	32	127	126	20	**0.62**
					129	128	21	**1.38**
					131	130	22	**1.11**
**1063**	RAC1 P63000$_{1,2}$: Ras-related C3 botulinum toxin substrate 1	2/4	4	4	127	126	3	**1.04**
					129	128	3	**0.37**
					131	130	3	**0.97**
**1064**	RACK1 P63244: Receptor of activated protein C kinase 1		9	10	127	126	10	**0.75**
					129	128	9	**0.33**
					131	130	9	**1.07**

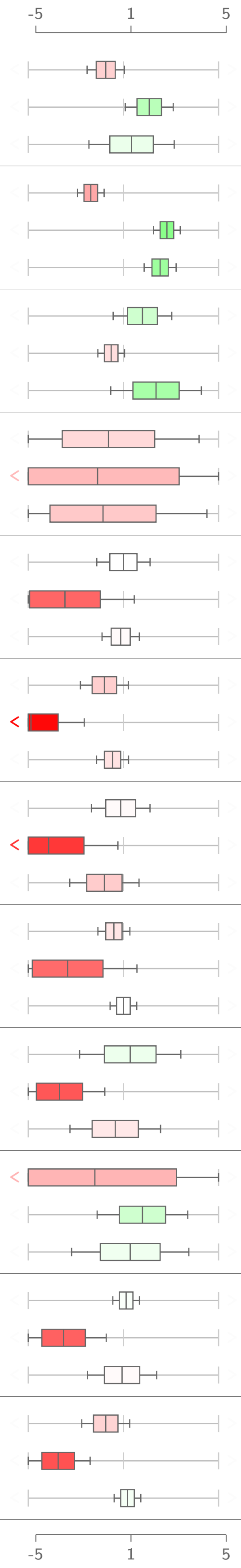

#	protein	group	peptides	spectra	ch1	ch2	quant	ratio
**1065**	RAD23B P54727$_1$: UV excision repair protein RAD23 homolog B		3	3	127	126	3	**0.91**
					129	128	3	**0.54**
					131	130	3	**0.82**
**1066**	RALY Q9UKM9$_{1,2}$: RNA-binding protein Raly	2/2	2	2	127	126	2	**0.60**
					129	128	2	**0.64**
					131	130	2	**0.90**
**1067**	RAN P62826: GTP-binding nuclear protein Ran		5	5	127	126	3	**1.12**
					129	128	5	**0.72**
					131	130	5	**0.98**
**1068**	RAP1A P62834, RAP1B P61224$_{1-3}$, A6NIZ1: Ras-related protein Rap-1A, Ras-related protein Rap-1b, ...	5/5	2	3	127	126	2	**0.46**
					129	128	2	**0.84**
					131	130	2	**0.93**
**1069**	RARRES1 P49788$_1$: Retinoic acid receptor responder protein 1	1/2	4	7	127	126	6	**0.97**
					129	128	5	**0.97**
					131	130	6	**1.22**
**1070**	RARRES2 Q99969: Retinoic acid receptor responder protein 2		8	65	127	126	51	**1.68**
					129	128	52	**3.07**
					131	130	58	**1.82**
**1071**	RARS P54136$_{1,2}$: Arginine--tRNA ligase, cytoplasmic	2/2	3	3	127	126	3	**1.00**
					129	128	3	**0.24**
					131	130	3	**1.12**
**1072**	RBBP4 Q09028$_{1-3}$, RBBP7 Q16576$_{1,2}$: Histone-binding protein RBBP4, Histone-binding protein RBBP7	5/5	2	2	127	126	2	**0.73**
					129	128	2	**1.12**
					131	130	2	**1.12**
**1073**	RBP4 P02753: Retinol-binding protein 4		10	119	127	126	104	**1.50**
					129	128	102	**1.87**
					131	130	104	**1.29**
**1074**	RCN1 Q15293$_{1,2}$: Reticulocalbin-1	2/2	2	2	127	126	2	**0.83**
					129	128	2	**2.43**
					131	130	2	**0.54**
**1075**	RCN3 Q96D15: Reticulocalbin-3		4	7	127	126	4	**0.78**
					129	128	5	**1.51**
					131	130	6	**0.66**
**1076**	REG3A Q06141: Regenerating islet-derived protein 3-alpha		4	4	127	126	4	**1.11**
					129	128	3	**1.89**
					131	130	3	**1.27**

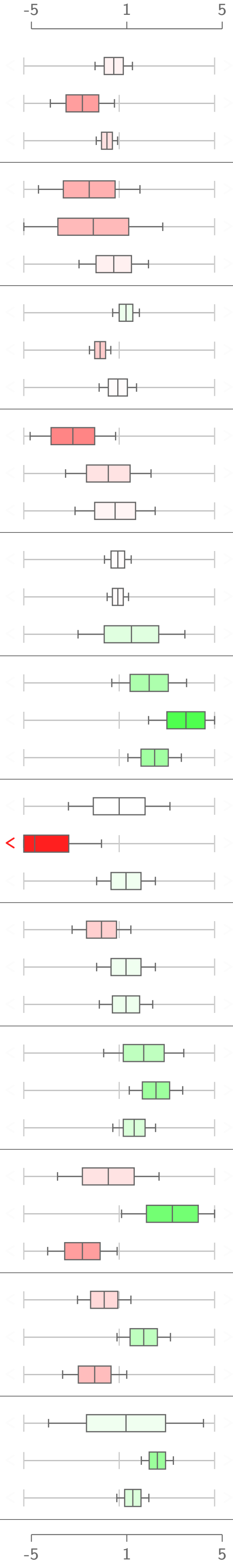

#	protein	group	peptides	spectra	ch1	ch2	quant	ratio
**1077**	RHOA P61586: Transforming protein RhoA	1/2	5	5	127	126	3	**0.96**
					129	128	3	**0.41**
					131	130	4	**1.05**
**1078**	RHOB P62745: Rho-related GTP-binding protein RhoB		2	2	127	126	1	**1.21**
					129	128	1	**0.47**
					131	130	1	**0.55**
**1079**	RIC8A Q9NPQ8$_{1-4}$: Synembryn-A	4/4	2	3	127	126	1	**1.02**
					129	128	1	**0.84**
					131	130	1	**2.84**
**1080**	RLF Q13129: Zinc finger protein Rlf		6	24	127	126	22	**1.69**
					129	128	23	**1.32**
					131	130	23	**0.54**
**1081**	RNASE1 P07998: Ribonuclease pancreatic		7	22	127	126	21	**1.08**
					129	128	21	**1.84**
					131	130	19	**1.37**
**1082**	RNASE4 P34096: Ribonuclease 4		13	89	127	126	83	**0.56**
					129	128	82	**2.51**
					131	130	83	**1.49**
**1083**	RNASE6 Q93091: Ribonuclease K6		2	2	127	126	2	**0.91**
					129	128	2	**1.20**
					131	130	2	**0.87**
**1084**	RNF19B Q6ZMZ0$_{1-4}$: E3 ubiquitin-protein ligase RNF19B	4/4	3	12	127	126	5	**0.68**
					129	128	5	**1.24**
					131	130	6	**0.79**
**1085**	RNH1 P13489: Ribonuclease inhibitor		8	11	127	126	8	**1.20**
					129	128	10	**0.56**
					131	130	8	**1.25**
**1086**	RPA2 P15927$_{1-3}$: Replication protein A 32 kDa subunit	3/3	2	2	127	126	2	**1.23**
					129	128	2	**0.77**
					131	130	2	**1.18**
**1087**	RPL10A P62906: 60S ribosomal protein L10a		2	2	127	126	2	**1.09**
					129	128	2	**0.43**
					131	130	2	**1.13**
**1088**	RPL12 P30050$_{1}$: 60S ribosomal protein L12		4	4	127	126	4	**0.91**
					129	128	3	**0.45**
					131	130	4	**1.12**

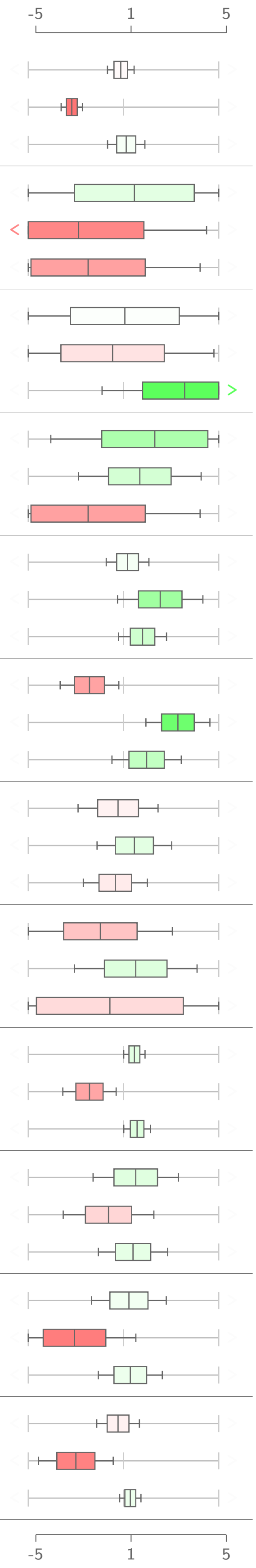

#	protein	group	peptides	spectra	ch1	ch2	quant	ratio
**1089**	RPL13 P26373$_{1,2}$: 60S ribosomal protein L13	2/2	2	3	127	126	2	**0.88**
					129	128	2	**0.11**
					131	130	2	**0.26**
**1090**	RPL13A P40429: 60S ribosomal protein L13a		2	2	127	126	2	**1.27**
					129	128	2	**0.46**
					131	130	2	**0.69**
**1091**	RPL14 P50914: 60S ribosomal protein L14		3	3	127	126	3	**0.73**
					129	128	3	**0.26**
					131	130	3	**0.75**
**1092**	RPL15 P61313$_{2}$: 60S ribosomal protein L15		3	4	127	126	3	**0.78**
					129	128	3	**0.20**
					131	130	4	**0.86**
**1093**	RPL17 P18621$_{1,3}$: 60S ribosomal protein L17	2/3	4	4	127	126	2	**0.75**
					129	128	2	**0.17**
					131	130	2	**0.61**
**1094**	RPL22 P35268: 60S ribosomal protein L22		2	2	127	126	2	**0.85**
					129	128	2	**0.64**
					131	130	2	**0.82**
**1095**	RPL23A P62750: 60S ribosomal protein L23a		4	4	127	126	4	**0.67**
					129	128	4	**0.46**
					131	130	4	**0.82**
**1096**	RPL24 P83731: 60S ribosomal protein L24		2	2	127	126	2	**0.69**
					129	128	2	**0.20**
					131	130	2	**0.53**
**1097**	RPL26 P61254, RPL26L1 Q9UNX3: 60S ribosomal protein L26, 60S ribosomal protein L26-like 1	2/2	2	2	127	126	2	**0.59**
					129	128	2	**0.33**
					131	130	2	**0.69**
**1098**	RPL28 P46779$_{1-3}$: 60S ribosomal protein L28	3/5	4	4	127	126	4	**1.49**
					129	128	4	**0.46**
					131	130	4	**0.46**
**1099**	RPL3 P39023: 60S ribosomal protein L3		4	7	127	126	7	**0.86**
					129	128	7	**0.38**
					131	130	7	**0.90**
**1100**	RPL30 P62888: 60S ribosomal protein L30		2	2	127	126	2	**0.59**
					129	128	2	**0.56**
					131	130	2	**0.88**

#	protein	group	peptides	spectra	ch1	ch2	quant	ratio
**1101**	RPL31 P62899$_{1-3}$: 60S ribosomal protein L31	3/3	1	2	127	126	2	**0.66**
					129	128	2	**0.23**
					131	130	2	**0.90**
**1102**	RPL34 P49207: 60S ribosomal protein L34		2	3	127	126	3	**0.76**
					129	128	3	**0.19**
					131	130	3	**0.79**
**1103**	RPL35 P42766: 60S ribosomal protein L35		3	4	127	126	4	**0.88**
					129	128	4	**0.25**
					131	130	3	**0.83**
**1104**	RPL35A P18077: 60S ribosomal protein L35a		2	2	127	126	2	**0.76**
					129	128	2	**0.19**
					131	130	2	**0.66**
**1105**	RPL37A P61513, RPL37AP8 A6NKH3: 60S ribosomal protein L37a, Putative 60S ribosomal protein L37a-like protein	2/2	1	2	127	126	2	**0.92**
					129	128	2	**0.14**
					131	130	2	**0.52**
**1106**	RPL5 P46777: 60S ribosomal protein L5		8	8	127	126	7	**0.95**
					129	128	7	**0.32**
					131	130	6	**0.84**
**1107**	RPL6 Q02878: 60S ribosomal protein L6		1	1	127	126	1	**0.71**
					129	128	1	**0.13**
					131	130	1	**0.92**
**1108**	RPL7 P18124: 60S ribosomal protein L7		1	1	127	126	1	**0.31**
					129	128	1	**0.63**
					131	130	1	**1.22**
**1109**	RPL7A P62424: 60S ribosomal protein L7a		3	5	127	126	5	**0.62**
					129	128	5	**0.26**
					131	130	5	**1.43**
**1110**	RPL8 P62917: 60S ribosomal protein L8		2	3	127	126	3	**0.83**
					129	128	3	**0.24**
					131	130	3	**0.71**
**1111**	RPL9 P32969: 60S ribosomal protein L9		3	3	127	126	3	**1.08**
					129	128	3	**0.32**
					131	130	3	**1.09**
**1112**	RPLP0 P05388$_{1,2}$: 60S acidic ribosomal protein P0	2/3	7	9	127	126	8	**1.00**
					129	128	8	**0.50**
					131	130	8	**1.41**

#	protein	group	peptides	spectra	ch1	ch2	quant	ratio
**1113**	RPLP2 P05387: 60S acidic ribosomal protein P2		2	3	127	126	3	**0.53**
					129	128	3	**0.67**
					131	130	3	**0.97**
**1114**	RPN1 P04843: Dolichyl-diphosphooligosaccharide--protein glycosyltransferase subunit 1		10	12	127	126	10	**0.78**
					129	128	10	< **0.30**
					131	130	12	< **0.17**
**1115**	RPN2 $P04844_{1,2}$: Dolichyl-diphosphooligosaccharide--protein glycosyltransferase subunit 2	2/2	3	3	127	126	3	**0.59**
					129	128	3	< **0.34**
					131	130	3	< **0.33**
**1116**	RPS10 P46783: 40S ribosomal protein S10		2	2	127	126	2	**0.74**
					129	128	2	< **0.28**
					131	130	2	**0.79**
**1117**	RPS12 P25398: 40S ribosomal protein S12		3	3	127	126	3	**0.90**
					129	128	3	< **0.36**
					131	130	3	**0.89**
**1118**	RPS13 P62277: 40S ribosomal protein S13		1	1	127	126	1	**1.15**
					129	128	1	< **0.14**
					131	130	1	**0.53**
**1119**	RPS14 P62263: 40S ribosomal protein S14		4	4	127	126	3	**1.05**
					129	128	4	**0.31**
					131	130	4	**0.71**
**1120**	RPS16 P62249: 40S ribosomal protein S16		3	3	127	126	3	**0.77**
					129	128	3	< **0.25**
					131	130	3	**1.17**
**1121**	RPS18 P62269: 40S ribosomal protein S18		3	4	127	126	3	**1.06**
					129	128	4	< **0.17**
					131	130	4	**0.51**
**1122**	RPS19 P39019: 40S ribosomal protein S19		3	3	127	126	3	**0.89**
					129	128	3	**0.47**
					131	130	3	**0.52**
**1123**	RPS2 P15880: 40S ribosomal protein S2		3	3	127	126	3	**0.98**
					129	128	3	< **0.14**
					131	130	3	**0.49**
**1124**	RPS20 $P60866_{1,2}$: 40S ribosomal protein S20	2/2	2	2	127	126	2	**0.87**
					129	128	2	**0.44**
					131	130	2	**0.75**

#	protein	group	peptides	spectra	ch1	ch2	quant	ratio	
1125	RPS23 P62266: 40S ribosomal protein S23		2	2	127	126	2	**1.37**	
					129	128	2	**0.58**	
					131	130	2	**0.45**	
1126	RPS27A P62979: Ubiquitin-40S ribosomal protein S27a	1/4	6	14	127	126	12	**0.56**	
					129	128	11	**0.31**	
					131	130	11	**0.56**	
1127	RPS3 P23396$_{1,2}$: 40S ribosomal protein S3	2/2	8	8	127	126	8	**0.82**	
					129	128	8	**0.23**	<
					131	130	8	**0.82**	
1128	RPS3A P61247: 40S ribosomal protein S3a		2	3	127	126	3	**1.12**	
					129	128	3	**0.18**	<
					131	130	3	**0.61**	
1129	RPS4X P62701: 40S ribosomal protein S4, X isoform	1/2	6	6	127	126	4	**1.04**	
					129	128	5	**0.25**	<
					131	130	5	**0.64**	
1130	RPS4Y2 Q8TD47: 40S ribosomal protein S4, Y isoform 2		2	2	127	126	1	**1.73**	
					129	128	1	**1.27**	
					131	130	1	**0.65**	
1131	RPS5 P46782: 40S ribosomal protein S5 [Cleaved into: 40S ribosomal protein S5, N-terminal . . .		2	2	127	126	2	**1.00**	
					129	128	2	**0.33**	
					131	130	2	**0.98**	
1132	RPS6 P62753: 40S ribosomal protein S6		5	7	127	126	6	**0.73**	
					129	128	6	**0.33**	
					131	130	7	**0.85**	
1133	RPS7 P62081: 40S ribosomal protein S7		2	2	127	126	2	**1.22**	
					129	128	2	**0.54**	
					131	130	2	**0.60**	
1134	RPS9 P46781: 40S ribosomal protein S9		2	2	127	126	2	**1.25**	
					129	128	2	**0.21**	<
					131	130	2	**0.61**	
1135	RPSA P08865: 40S ribosomal protein SA		9	13	127	126	10	**1.07**	
					129	128	13	**0.63**	
					131	130	9	**1.05**	
1136	RRBP1 Q9P2E9$_{1,2}$: Ribosome-binding protein 1	2/3	11	12	127	126	10	**1.17**	
					129	128	11	**0.55**	<
					131	130	11	**0.64**	

#	protein	group	peptides	spectra	ch1	ch2	quant	ratio
**1137**	RTN1 Q16799$_{1-3}$, RTN3 O95197$_{1-7}$: Reticulon-1, Reticulon-3	10/10	1	1	127	126	1	**0.53**
					129	128	1	**0.07**
					131	130	1	**0.27**
**1138**	RXFP2 Q8WXD0$_{1,2}$: Relaxin receptor 2	2/2	2	10	127	126	10	**1.33**
					129	128	10	**1.06**
					131	130	10	**0.47**
**1139**	S100A10 P60903: Protein S100-A10		7	19	127	126	16	**0.83**
					129	128	16	**0.63**
					131	130	14	**0.54**
**1140**	S100A11 P31949: Protein S100-A11		3	3	127	126	3	**0.79**
					129	128	3	**0.05**
					131	130	3	**0.42**
**1141**	S100A16 Q96FQ6: Protein S100-A16		2	2	127	126	2	**0.72**
					129	128	2	**0.15**
					131	130	2	**0.64**
**1142**	S100A2 P29034: Protein S100-A2		4	5	127	126	4	**0.59**
					129	128	4	**0.03**
					131	130	5	**0.60**
**1143**	S100A6 P06703: Protein S100-A6		3	6	127	126	6	**0.97**
					129	128	5	**0.40**
					131	130	6	**1.46**
**1144**	S100A8 P05109: Protein S100-A8		6	9	127	126	8	**0.55**
					129	128	9	**0.32**
					131	130	6	**1.02**
**1145**	S100A9 P06702: Protein S100-A9		4	4	127	126	3	**0.51**
					129	128	3	**0.49**
					131	130	4	**1.35**
**1146**	SAA1 P0DJI8: Serum amyloid A-1 protein	1/2	10	34	127	126	10	**0.61**
					129	128	10	**1.13**
					131	130	10	**0.97**
**1147**	SAA2 P0DJI9$_{1}$: Serum amyloid A-2 protein		9	26	127	126	2	**0.61**
					129	128	2	**1.20**
					131	130	2	**0.64**
**1148**	SAA4 P35542: Serum amyloid A-4 protein		7	179	127	126	171	**0.39**
					129	128	164	**0.78**
					131	130	176	**1.62**

#	protein	group	peptides	spectra	ch1	ch2	quant	ratio
**1149**	SARS P49591: Serine--tRNA ligase, cytoplasmic		5	7	127	126	7	**1.24**
					129	128	7	**0.43**
					131	130	6	**0.41**
**1150**	SBSN Q6UWP8$_1$: Suprabasin	1/2	8	10	127	126	9	**0.82**
					129	128	10	**0.65**
					131	130	9	**1.14**
**1151**	SCARF2 Q96GP6$_{1,2}$: Scavenger receptor class F member 2	2/2	5	15	127	126	9	**0.67**
					129	128	9	**1.30**
					131	130	7	**1.23**
**1152**	SCG2 P13521: Secretogranin-2		23	27	127	126	24	**0.59**
					129	128	26	**1.48**
					131	130	25	**1.34**
**1153**	SCG3 Q8WXD2$_1$: Secretogranin-3	1/2	6	6	127	126	6	**0.43**
					129	128	6	**1.86**
					131	130	6	**1.53**
**1154**	SCG5 P05408$_2$: Neuroendocrine protein 7B2	1/2	4	5	127	126	5	**0.36**
					129	128	5	**1.44**
					131	130	5	**1.28**
**1155**	SCGB1A1 P11684: Uteroglobin		3	5	127	126	5	**1.92**
					129	128	5	**1.75**
					131	130	4	**2.31**
**1156**	SCRN1 Q12765$_{1-3}$: Secernin-1	3/3	2	2	127	126	2	**0.55**
					129	128	2	**1.37**
					131	130	2	**1.01**
**1157**	SCUBE2 Q9NQ36$_{1,3}$: Signal peptide, CUB and EGF-like domain-containing protein 2	2/3	5	5	127	126	3	**0.99**
					129	128	5	**1.09**
					131	130	4	**2.32**
**1158**	SDCBP O00560$_{1-3}$: Syntenin-1	3/3	4	4	127	126	4	**0.91**
					129	128	4	**0.98**
					131	130	4	**1.25**
**1159**	SDF4 Q9BRK5$_1$: 45 kDa calcium-binding protein	1/6	9	16	127	126	16	**0.63**
					129	128	16	**1.88**
					131	130	15	**1.25**
**1160**	SDPR O95810: Serum deprivation-response protein		6	7	127	126	6	**0.59**
					129	128	7	**1.29**
					131	130	6	**0.97**

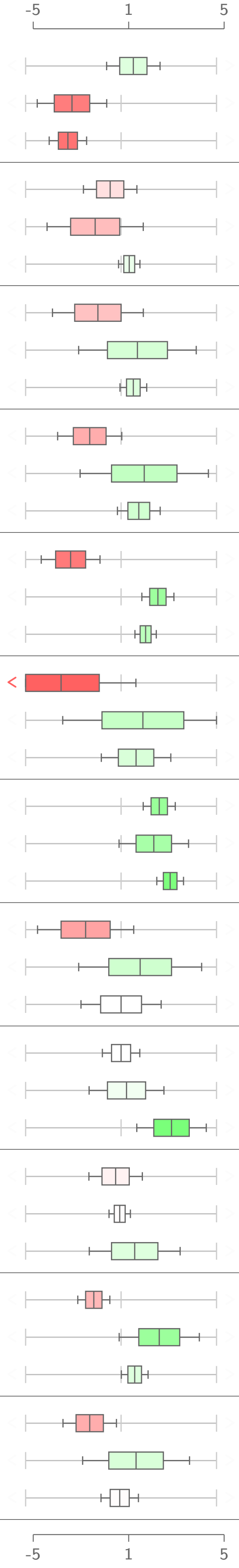

#	protein	group	peptides	spectra	ch1	ch2	quant	ratio
**1161**	SEC23A Q15436$_{1,2}$: Protein transport protein Sec23A	2/2	3	4	127	126	3	**1.03**
					129	128	4	**0.77**
					131	130	4	**1.05**
**1162**	SEC23IP Q9Y6Y8$_{1,2}$: SEC23-interacting protein	2/2	2	2	127	126	2	**0.75**
					129	128	2	**0.36**
					131	130	2	**1.41**
**1163**	SEC61A1 P61619$_{1,3}$: Protein transport protein Sec61 subunit alpha isoform 1	2/2	1	2	127	126	2	**0.75**
					129	128	2	**0.10**
					131	130	2	**0.19**
**1164**	SEL1L2 Q5TEA6$_{1,2}$: Protein sel-1 homolog 2	2/2	2	12	127	126	2	**4.01**
					129	128	7	**0.64**
					131	130	2	**1.43**
**1165**	SEMA3B Q13214$_{1,2}$: Semaphorin-3B	2/2	2	2	127	126	2	**0.60**
					129	128	2	**1.51**
					131	130	2	**1.51**
**1166**	SEMA3F Q13275$_{1,2}$: Semaphorin-3F	2/2	4	4	127	126	4	**0.74**
					129	128	4	**1.42**
					131	130	4	**1.62**
**1167**	SEPP1 P49908: Selenoprotein P		8	15	127	126	12	**1.04**
					129	128	13	**1.65**
					131	130	14	**1.37**
**1168**	SEPT2 Q15019$_{1,2}$: Septin-2	2/3	4	4	127	126	4	**1.14**
					129	128	4	**0.52**
					131	130	4	**0.99**
**1169**	SEPT7 Q16181$_{1,2}$: Septin-7	2/2	8	100	127	126	28	**1.16**
					129	128	37	**0.50**
					131	130	42	**0.92**
**1170**	SEPT9 Q9UHD8$_{1,2,5,7}$: Septin-9	4/4	3	3	127	126	3	**1.15**
					129	128	3	**0.55**
					131	130	3	**0.94**
**1171**	SERBP1 Q8NC51$_{1-4}$: Plasminogen activator inhibitor 1 RNA-binding protein	4/4	2	2	127	126	2	**0.64**
					129	128	2	**0.51**
					131	130	2	**0.79**
**1172**	SERPINA1 P01009$_1$: Alpha-1-antitrypsin	1/3	29	212	127	126	190	**1.95**
					129	128	192	**1.47**
					131	130	196	**0.90**

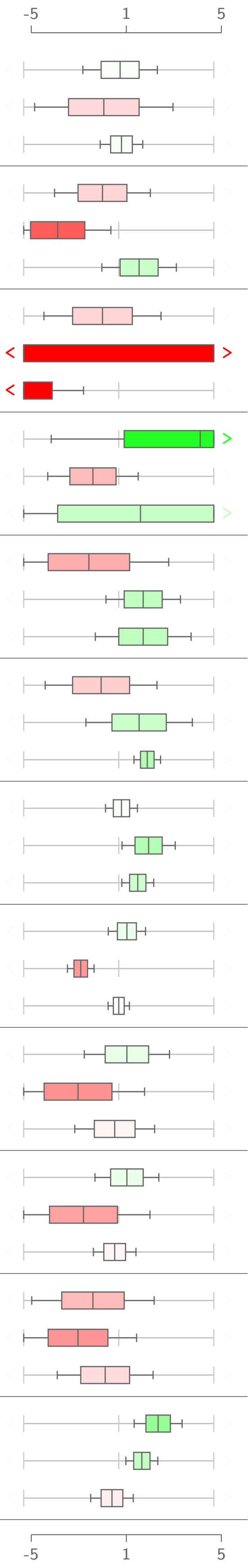

#	protein	group	peptides	spectra	ch1	ch2	quant	ratio
**1173**	SERPINA10 Q9UK55: Protein Z-dependent protease inhibitor		20	83	127	126	47	**1.77**
					129	128	39	**1.05**
					131	130	43	**1.36**
**1174**	SERPINA11 Q86U17: Serpin A11		1	1	127	126	1	**1.55**
					129	128	1	**0.48**
					131	130	1	**1.43**
**1175**	SERPINA3 P01011$_1$: Alpha-1-antichymotrypsin	1/2	14	35	127	126	33	**2.03**
					129	128	34	**1.29**
					131	130	35	**0.91**
**1176**	SERPINA4 P29622: Kallistatin		18	35	127	126	34	**1.82**
					129	128	33	**0.55**
					131	130	33	**1.13**
**1177**	SERPINA5 P05154: Plasma serine protease inhibitor		12	22	127	126	21	**1.04**
					129	128	19	**1.13**
					131	130	22	**1.70**
**1178**	SERPINA6 P08185: Corticosteroid-binding globulin		3	5	127	126	5	**1.37**
					129	128	5	**1.17**
					131	130	5	**2.12**
**1179**	SERPINA7 P05543: Thyroxine-binding globulin		8	10	127	126	10	**2.19**
					129	128	9	**0.62**
					131	130	9	**1.61**
**1180**	SERPINB3 P29508$_1$: Serpin B3	1/2	6	6	127	126	2	**0.62**
					129	128	2	**0.13**
					131	130	2	**0.90**
**1181**	SERPINB4 P48594: Serpin B4		7	7	127	126	3	**0.61**
					129	128	3	**0.11**
					131	130	3	**0.84**
**1182**	SERPINB5 P36952$_1$: Serpin B5	1/2	4	5	127	126	5	**0.56**
					129	128	3	**0.07**
					131	130	4	**1.35**
**1183**	SERPINC1 P01008: Antithrombin-III		26	148	127	126	146	**0.38**
					129	128	146	**1.63**
					131	130	148	**2.49**
**1184**	SERPIND1 P05546: Heparin cofactor 2		27	233	127	126	231	**2.13**
					129	128	218	**0.56**
					131	130	230	**1.78**

#	protein	group	peptides	spectra	ch1	ch2	quant	ratio
1185	SERPINE1 P05121$_{1,2}$: Plasminogen activator inhibitor 1	2/2	11	14	127	126	13	**1.18**
					129	128	14	**1.06**
					131	130	11	**0.85**
1186	SERPINE2 P07093$_{1-3}$: Glia-derived nexin	3/3	2	2	127	126	2	**1.04**
					129	128	2	**0.86**
					131	130	2	**2.11**
1187	SERPINF1 P36955: Pigment epithelium-derived factor		18	58	127	126	57	**2.32**
					129	128	54	**2.10**
					131	130	53	**1.45**
1188	SERPINF2 P08697$_{1}$: Alpha-2-antiplasmin	1/2	17	115	127	126	91	**1.32**
					129	128	93	**0.82**
					131	130	97	**1.25**
1189	SERPING1 P05155$_{1-3}$: Plasma protease C1 inhibitor	3/3	9	14	127	126	11	**1.20**
					129	128	14	**1.19**
					131	130	11	**1.98**
1190	SERPINH1 P50454: Serpin H1		9	12	127	126	12	**1.09**
					129	128	12	**0.99**
					131	130	12	**0.22**
1191	SFN P31947$_{1}$: 14-3-3 protein sigma	1/2	11	18	127	126	8	**0.30**
					129	128	6	**0.13**
					131	130	8	**1.29**
1192	SFPQ P23246$_{1,2}$: Splicing factor, proline- and glutamine-rich	2/2	10	15	127	126	10	**0.56**
					129	128	11	**0.61**
					131	130	9	**1.03**
1193	SFRP1 Q8N474: Secreted frizzled-related protein 1		2	2	127	126	2	**0.41**
					129	128	2	**1.55**
					131	130	2	**1.07**
1194	SFRP2 Q96HF1: Secreted frizzled-related protein 2		12	62	127	126	59	**1.00**
					129	128	60	**1.43**
					131	130	60	**1.43**
1195	SFRP4 Q6FHJ7: Secreted frizzled-related protein 4		2	3	127	126	2	**0.71**
					129	128	2	**2.12**
					131	130	2	**1.91**
1196	SH3BGRL3 Q9H299: SH3 domain-binding glutamic acid-rich-like protein 3		3	3	127	126	3	**0.90**
					129	128	3	**0.92**
					131	130	3	**0.88**

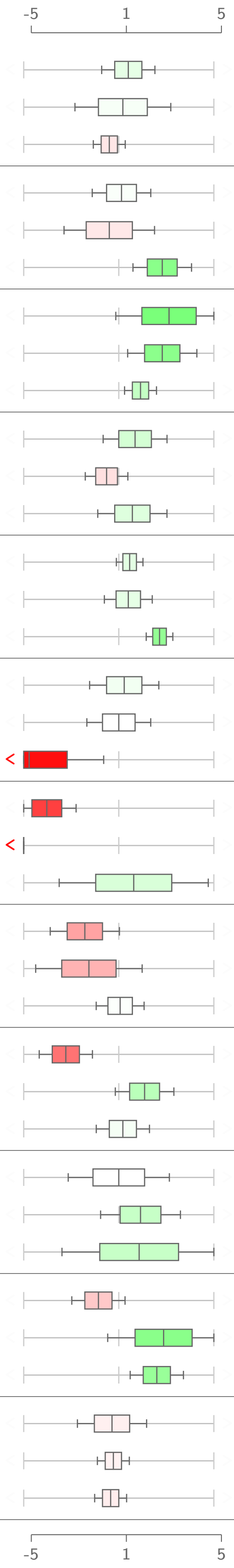

#	protein	group	peptides	spectra	ch1	ch2	quant	ratio
**1197**	SHBG P04278$_1$: Sex hormone-binding globulin	1/5	14	33	127	126	31	**2.64**
					129	128	29	**0.37**
					131	130	31	**0.90**
**1198**	SHROOM4 Q9ULL8$_1$: Protein Shroom4		5	29	127	126	9	**0.77**
					129	128	9	**1.96**
					131	130	7	**1.19**
**1199**	SIL1 Q9H173: Nucleotide exchange factor SIL1		2	2	127	126	2	**1.17**
					129	128	2	**1.02**
					131	130	2	**1.40**
**1200**	SKP1 P63208$_{1,2}$: S-phase kinase-associated protein 1	2/2	4	6	127	126	3	**1.10**
					129	128	3	**1.20**
					131	130	3	**1.11**
**1201**	SLC16A1 P53985$_1$: Monocarboxylate transporter 1		2	2	127	126	2	**0.85**
					129	128	2	**0.72**
					131	130	2	**0.50**
**1202**	SLC25A3 Q00325$_{1,2}$: Phosphate carrier protein, mitochondrial	2/2	2	5	127	126	4	**0.74**
					129	128	5	**0.21**
					131	130	5	**0.23**
**1203**	SLC25A6 P12236: ADP/ATP translocase 3	1/4	7	8	127	126	7	**0.58**
					129	128	6	**0.10**
					131	130	7	**0.08**
**1204**	SLC28A3 Q9HAS3$_2$: Solute carrier family 28 member 3		3	10	127	126	7	**0.97**
					129	128	6	**1.78**
					131	130	7	**2.11**
**1205**	SLC4A1AP Q9BWU0: Kanadaptin		5	8	127	126	7	**1.45**
					129	128	4	**1.07**
					131	130	6	**1.80**
**1206**	SLC9A3R1 O14745$_1$: Na(+)/H(+) exchange regulatory cofactor NHE-RF1		3	3	127	126	3	**1.05**
					129	128	3	**0.79**
					131	130	3	**0.93**
**1207**	SLIT3 O75094$_{1,2,4}$: Slit homolog 3 protein	3/3	6	13	127	126	13	**0.46**
					129	128	13	**2.30**
					131	130	9	**1.26**
**1208**	SLPI P03973: Antileukoproteinase		15	65	127	126	57	**0.51**
					129	128	58	**2.06**
					131	130	52	**1.69**

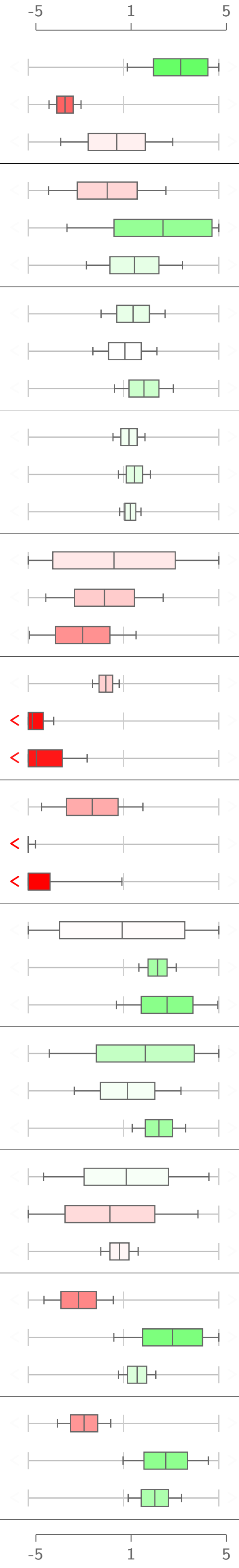

#	protein	group	peptides	spectra	ch1	ch2	quant	ratio
1209	SLURP1 P55000: Secreted Ly-6/uPAR-related protein 1		2	3	127	126	3	**1.92**
					129	128	3	**2.26**
					131	130	3	**1.68**
1210	SMS P52788$_{1}$: Spermine synthase		2	2	127	126	2	**0.75**
					129	128	2	**1.09**
					131	130	2	**1.36**
1211	SND1 Q7KZF4: Staphylococcal nuclease domain-containing protein 1		3	3	127	126	3	**0.96**
					129	128	3	**0.40**
					131	130	3	**1.19**
1212	SNED1 Q8TER0$_{1,3-5}$: Sushi, nidogen and EGF-like domain-containing protein 1	4/4	9	11	127	126	10	**0.93**
					129	128	9	**1.46**
					131	130	10	**1.38**
1213	SNRPD3 P62318$_{1,2}$: Small nuclear ribonucleoprotein Sm D3	2/2	2	2	127	126	2	**0.47**
					129	128	2	**0.51**
					131	130	2	**0.87**
1214	SNX12 Q9UMY4$_{1-3}$: Sorting nexin-12	3/3	1	2	127	126	2	**1.31**
					129	128	2	**0.72**
					131	130	2	**1.30**
1215	SNX6 Q9UNH7$_{1}$: Sorting nexin-6	1/2	5	6	127	126	5	**0.92**
					129	128	5	**0.84**
					131	130	5	**1.30**
1216	SOD2 P04179$_{1,4}$: Superoxide dismutase [Mn], mitochondrial	2/4	5	6	127	126	6	**0.58**
					129	128	6	**0.80**
					131	130	6	**0.17**
1217	SOD3 P08294: Extracellular superoxide dismutase [Cu-Zn]		6	32	127	126	31	**0.54**
					129	128	29	**2.10**
					131	130	30	**1.82**
1218	SOS1 Q07889$_{1}$: Son of sevenless homolog 1		3	6	127	126	5	**1.78**
					129	128	4	**1.00**
					131	130	6	**0.67**
1219	SP100 P23497$_{1}$: Nuclear autoantigen Sp-100		4	11	127	126	1	**0.56**
					129	128	1	**0.63**
					131	130	1	**1.18**
1220	SPARC P09486: SPARC		17	54	127	126	50	**1.37**
					129	128	49	**1.17**
					131	130	52	**1.15**

#	protein	group	peptides	spectra	ch1	ch2	quant	ratio
**1221**	SPARCL1 Q14515$_1$: SPARC-like protein 1	1/2	31	77	127	126	75	**0.48**
					129	128	75	**2.09**
					131	130	76	**1.57**
**1222**	SPEF2 Q9C093$_{1,2}$: Sperm flagellar protein 2	2/2	3	4	127	126	2	**0.98**
					129	128	2	**0.35**
					131	130	2	**1.15**
**1223**	SPINK5 Q9NQ38$_{1-3}$: Serine protease inhibitor Kazal-type 5	3/3	7	70	127	126	35	**1.88**
					129	128	40	**0.98**
					131	130	45	**0.34**
**1224**	SPON1 Q9HCB6: Spondin-1		18	35	127	126	30	**1.02**
					129	128	34	**2.42**
					131	130	34	**1.95**
**1225**	SPON2 Q9BUD6: Spondin-2		2	3	127	126	3	**1.16**
					129	128	3	**0.89**
					131	130	3	**1.45**
**1226**	SPP1 P10451$_{1-5}$: Osteopontin	5/5	5	7	127	126	6	**1.63**
					129	128	7	**2.00**
					131	130	7	**1.35**
**1227**	SPP2 Q13103: Secreted phosphoprotein 24		4	5	127	126	4	**0.46**
					129	128	5	**1.37**
					131	130	5	**0.79**
**1228**	SPRED1 Q7Z699: Sprouty-related, EVH1 domain-containing protein 1		4	17	127	126	8	**1.21**
					129	128	8	**1.47**
					131	130	6	**1.90**
**1229**	SPRR1A P35321, SPRR1B P22528: Cornifin-A, Cornifin-B	2/2	3	7	127	126	7	**0.92**
					129	128	6	**0.11**
					131	130	7	**0.82**
**1230**	SPRR2D P22532: Small proline-rich protein 2D		2	3	127	126	3	**0.54**
					129	128	3	**0.03**
					131	130	2	**0.27**
**1231**	SPRR4 Q96PI1: Small proline-rich protein 4		9	13	127	126	11	**0.94**
					129	128	13	**0.13**
					131	130	12	**0.88**
**1232**	SPTAN1 Q13813$_{1-3}$: Spectrin alpha chain, non-erythrocytic 1	3/3	20	77	127	126	57	**1.32**
					129	128	59	**0.52**
					131	130	65	**0.62**

#	protein	group	peptides	spectra	ch1	ch2	quant	ratio
1233	SPTBN1 Q01082$_{1}$: Spectrin beta chain, non-erythrocytic 1	1/2	20	58	127	126	1	**1.57**
					129	128	1	**0.53**
					131	130	1	**0.67**
1234	SPTBN1 Q01082$_{3}$: Spectrin beta chain, non-erythrocytic 1		20	58	127	126	1	**1.16**
					129	128	1	**0.19**
					131	130	1	**0.94**
1235	SPTBN2 O15020$_{1}$: Spectrin beta chain, non-erythrocytic 2	1/2	8	49	127	126	26	**0.65**
					129	128	28	**1.09**
					131	130	28	**0.83**
1236	SPTBN4 Q9H254$_{1}$: Spectrin beta chain, non-erythrocytic 4	1/3	4	4	127	126	2	**1.14**
					129	128	2	**1.37**
					131	130	2	**0.88**
1237	SQRDL Q9Y6N5: Sulfide:quinone oxidoreductase, mitochondrial		4	5	127	126	5	**0.87**
					129	128	4	**0.17**
					131	130	5	**0.09**
1238	SRPX P78539$_{1,4}$: Sushi repeat-containing protein SRPX	2/5	9	12	127	126	12	**1.20**
					129	128	12	**0.84**
					131	130	11	**2.44**
1239	SRRM1 Q8IYB3$_{1,2}$: Serine/arginine repetitive matrix protein 1	2/2	2	2	127	126	1	**0.54**
					129	128	1	**1.57**
					131	130	1	**1.99**
1240	SRSF1 Q07955$_{1-3}$: Serine/arginine-rich splicing factor 1	3/3	6	7	127	126	7	**0.74**
					129	128	7	**1.01**
					131	130	7	**1.03**
1241	SRSF10 O75494$_{1-6}$: Serine/arginine-rich splicing factor 10	6/6	2	2	127	126	2	**0.74**
					129	128	2	**0.61**
					131	130	2	**1.05**
1242	SRSF2 Q01130$_{1}$: Serine/arginine-rich splicing factor 2		2	2	127	126	1	**0.53**
					129	128	1	**0.87**
					131	130	1	**1.03**
1243	SRSF3 P84103$_{1,2}$: Serine/arginine-rich splicing factor 3	2/2	4	22	127	126	19	**3.94**
					129	128	19	**1.14**
					131	130	19	**0.21**
1244	SRSF7 Q16629$_{1-4}$: Serine/arginine-rich splicing factor 7	4/4	4	4	127	126	2	**0.52**
					129	128	3	**0.54**
					131	130	3	**1.13**

#	protein	group	peptides	spectra	ch1	ch2	quant	ratio
**1245**	SSC5D A1L4H1$_{1,2}$: Soluble scavenger receptor cysteine-rich domain-containing protein SSC5D	2/2	3	5	127	126	3	**0.84**
					129	128	5	**1.32**
					131	130	5	**1.46**
**1246**	SSR3 Q9UNL2$_{1,2}$: Translocon-associated protein subunit gamma	2/2	2	2	127	126	2	**0.42**
					129	128	2	**0.09**
					131	130	2	**0.28**
**1247**	ST13 P50502: Hsc70-interacting protein	1/3	8	10	127	126	10	**0.69**
					129	128	7	**1.70**
					131	130	6	**1.15**
**1248**	STAB1 Q9NY15$_{1}$: Stabilin-1	1/2	13	26	127	126	17	**0.83**
					129	128	20	**1.10**
					131	130	23	**1.22**
**1249**	STIP1 P31948$_{1-3}$: Stress-induced-phosphoprotein 1	3/3	4	4	127	126	3	**1.06**
					129	128	4	**0.80**
					131	130	3	**1.06**
**1250**	SUB1 P53999: Activated RNA polymerase II transcriptional coactivator p15		3	3	127	126	3	**0.77**
					129	128	3	**0.60**
					131	130	3	**0.82**
**1251**	SULT1A3 P0DMM9$_{1,3}$, SULT1A4 P0DMN0: Sulfotransferase 1A3, Sulfotransferase 1A4	3/6	5	6	127	126	5	**1.23**
					129	128	6	**0.65**
					131	130	6	**1.13**
**1252**	SUSD5 O60279: Sushi domain-containing protein 5		4	5	127	126	4	**0.73**
					129	128	5	**1.42**
					131	130	5	**1.17**
**1253**	SVEP1 Q4LDE5$_{1}$: Sushi, von Willebrand factor type A, EGF and pentraxin domain-containing pro ...	1/4	52	75	127	126	62	**0.93**
					129	128	67	**1.36**
					131	130	54	**1.76**
**1254**	SYNCRIP O60506$_{1-4}$: Heterogeneous nuclear ribonucleoprotein Q	4/5	7	8	127	126	4	**0.67**
					129	128	4	**0.78**
					131	130	5	**0.86**
**1255**	TAF15 Q92804$_{1,2}$: TATA-binding protein-associated factor 2N	2/2	4	4	127	126	4	**0.69**
					129	128	3	**1.11**
					131	130	3	**1.03**
**1256**	TAGLN Q01995: Transgelin		11	13	127	126	12	**1.23**
					129	128	12	**0.92**
					131	130	11	**2.43**

#	protein	group	peptides	spectra	ch1	ch2	quant	ratio
**1257**	TAGLN2 P37802$_{1,2}$: Transgelin-2	2/2	8	13	127	126	11	**0.70**
					129	128	13	**0.83**
					131	130	13	**1.70**
**1258**	TALDO1 P37837: Transaldolase		2	2	127	126	2	**0.57**
					129	128	2	**0.25**
					131	130	2	**0.94**
**1259**	TBCB Q99426$_{1,2}$: Tubulin-folding cofactor B	2/2	3	3	127	126	3	**1.37**
					129	128	3	**1.12**
					131	130	3	**1.23**
**1260**	TCHH Q07283: Trichohyalin		118	164	127	126	124	**0.76**
					129	128	133	**0.06**
					131	130	108	**0.96**
**1261**	TCN2 P20062$_{1,2}$: Transcobalamin-2	2/2	4	4	127	126	4	**1.56**
					129	128	4	**0.69**
					131	130	3	**2.01**
**1262**	TCP1 P17987: T-complex protein 1 subunit alpha		3	4	127	126	3	**0.94**
					129	128	3	**0.49**
					131	130	4	**0.91**
**1264**	TF P02787: Serotransferrin		37	67	127	126	66	**1.96**
					129	128	65	**2.17**
					131	130	62	**0.83**
**1265**	TFAM Q00059$_{1,2}$: Transcription factor A, mitochondrial	2/2	3	3	127	126	3	**0.72**
					129	128	3	**2.05**
					131	130	3	**0.87**
**1266**	TFG Q92734$_{1-4}$: Protein TFG	4/4	3	4	127	126	2	**0.87**
					129	128	2	**0.56**
					131	130	2	**1.03**
**1267**	TFPI P10646$_{1}$: Tissue factor pathway inhibitor		3	3	127	126	1	**0.69**
					129	128	1	**2.07**
					131	130	1	**1.18**
**1268**	TFPI P10646$_{2}$: Tissue factor pathway inhibitor		3	4	127	126	2	**0.59**
					129	128	2	**0.67**
					131	130	2	**1.23**
**1269**	TGFBI Q15582: Transforming growth factor-beta-induced protein ig-h3		19	33	127	126	32	**1.19**
					129	128	32	**1.00**
					131	130	32	**1.53**

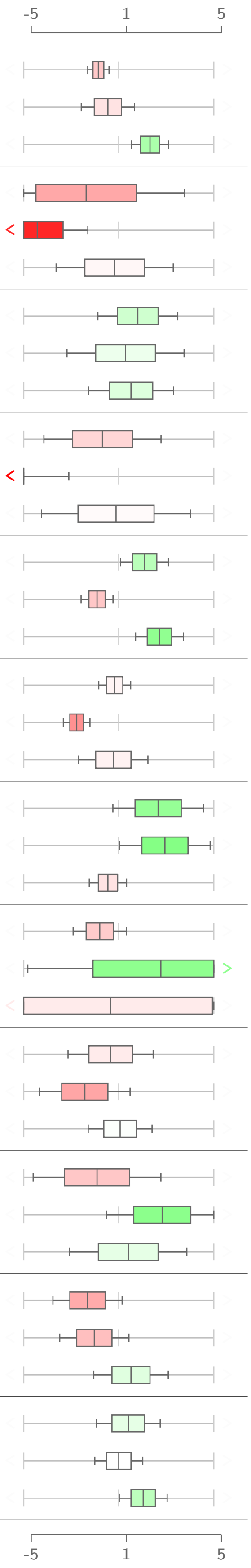

#	protein	group	peptides	spectra	ch1	ch2	quant	ratio
**1270**	TGFBR3 Q03167$_{1,2}$: Transforming growth factor beta receptor type 3	2/2	12	15	127	126	14	**1.42**
					129	128	15	**0.57**
					131	130	14	**1.56**
**1271**	TGM1 P22735$_{1}$: Protein-glutamine gamma-glutamyltransferase K	1/2	5	5	127	126	3	**0.71**
					129	128	5	**0.14**
					131	130	5	**0.70**
**1272**	TGM2 P21980$_{1}$: Protein-glutamine gamma-glutamyltransferase 2	1/2	11	15	127	126	11	**0.83**
					129	128	12	**0.93**
					131	130	12	**0.54**
**1273**	TGM3 Q08188: Protein-glutamine gamma-glutamyltransferase E		4	4	127	126	4	**0.11**
					129	128	3	**0.18**
					131	130	4	**0.86**
**1274**	THBS1 P07996$_{1}$: Thrombospondin-1	1/2	35	67	127	126	51	**1.07**
					129	128	49	**1.00**
					131	130	50	**1.65**
**1275**	THBS2 P35442: Thrombospondin-2		28	44	127	126	34	**0.50**
					129	128	37	**1.69**
					131	130	33	**1.63**
**1276**	THBS3 P49746$_{1}$: Thrombospondin-3	1/2	6	7	127	126	6	**0.91**
					129	128	6	**0.88**
					131	130	6	**1.57**
**1277**	THBS4 P35443: Thrombospondin-4		23	51	127	126	41	**1.91**
					129	128	41	**0.55**
					131	130	39	**2.14**
**1278**	TIA1 P31483$_{3}$: Nucleolysin TIA-1 isoform p40	1/3	3	4	127	126	4	**0.71**
					129	128	4	**0.82**
					131	130	3	**1.61**
**1279**	TICAM2 Q86XR7$_{2}$, TMED7 Q9Y3B3$_{1,2}$: TIR domain-containing adapter molecule 2, Transmembrane emp24 domain-containing protein 7	3/3	1	1	127	126	1	**1.45**
					129	128	1	**0.62**
					131	130	1	**0.52**
**1280**	TIE1 P35590$_{1}$: Tyrosine-protein kinase receptor Tie-1		2	2	127	126	2	**1.49**
					129	128	2	**0.71**
					131	130	2	**1.63**
**1281**	TIMP1 P01033: Metalloproteinase inhibitor 1		3	7	127	126	6	**1.70**
					129	128	7	**1.38**
					131	130	5	**1.15**

#	protein	group	peptides	spectra	ch1	ch2	quant	ratio
**1282**	TIMP2 P16035: Metalloproteinase inhibitor 2		9	39	127	126	21	**1.11**
					129	128	21	**1.31**
					131	130	22	**1.14**
**1283**	TINAGL1 Q9GZM7$_1$: Tubulointerstitial nephritis antigen-like	1/2	4	12	127	126	4	**0.66**
					129	128	5	**1.33**
					131	130	5	**1.59**
**1284**	TLL1 O43897$_1$: Tolloid-like protein 1		4	4	127	126	1	**1.29**
					129	128	1	**1.05**
					131	130	1	**0.36**
**1285**	TLN1 Q9Y490: Talin-1		51	54	127	126	44	**1.35**
					129	128	44	**0.61**
					131	130	42	**1.52**
**1286**	TLN2 Q9Y4G6: Talin-2		9	9	127	126	1	**0.56**
					129	128	1	**1.03**
					131	130	1	**0.96**
**1287**	TMED10 P49755: Transmembrane emp24 domain-containing protein 10		5	6	127	126	6	**1.02**
					129	128	4	**0.18**
					131	130	4	**0.11**
**1288**	TMED2 Q15363: Transmembrane emp24 domain-containing protein 2		3	3	127	126	3	**0.91**
					129	128	3	**0.36**
					131	130	3	**0.35**
**1289**	TMED9 Q9BVK6: Transmembrane emp24 domain-containing protein 9		2	2	127	126	2	**1.05**
					129	128	2	**0.36**
					131	130	2	**0.42**
**1290**	TMEM43 Q9BTV4: Transmembrane protein 43		2	2	127	126	2	**0.73**
					129	128	2	**0.16**
					131	130	2	**0.17**
**1291**	TMEM59 Q9BXS4: Transmembrane protein 59		2	6	127	126	3	**0.47**
					129	128	3	**1.15**
					131	130	3	**0.75**
**1292**	TMF1 P82094$_{1,2}$: TATA element modulatory factor	2/2	2	7	127	126	7	**0.26**
					129	128	6	**1.81**
					131	130	7	**1.86**
**1293**	TNC P24821$_{1,4}$: Tenascin	2/5	38	61	127	126	2	**0.51**
					129	128	2	**1.35**
					131	130	2	**2.32**

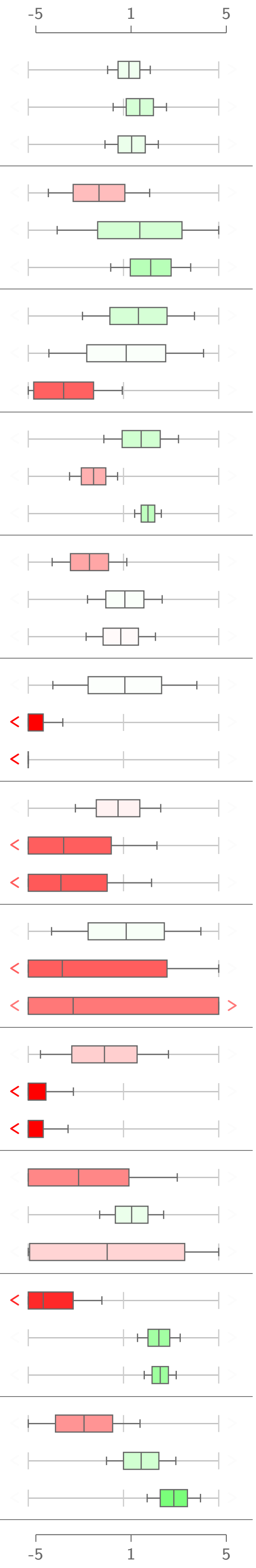

#	protein	group	peptides	spectra	ch1	ch2	quant	ratio
**1294**	TNC P24821$_6$: Tenascin		37	60	127	126	1	**0.87**
					129	128	1	**1.30**
					131	130	1	**1.51**
**1295**	TNFAIP6 P98066: Tumor necrosis factor-inducible gene 6 protein		4	5	127	126	5	**0.92**
					129	128	5	**1.21**
					131	130	5	**1.23**
**1296**	TNFRSF11B O00300: Tumor necrosis factor receptor superfamily member 11B		2	2	127	126	2	**0.49**
					129	128	2	**1.93**
					131	130	2	**1.49**
**1297**	TNNT1 P13805$_{1-3}$: Troponin T, slow skeletal muscle	3/3	4	7	127	126	7	**1.51**
					129	128	7	**3.56**
					131	130	6	**1.09**
**1298**	TNPO2 O14787$_{1,2}$: Transportin-2	2/2	3	3	127	126	3	**1.02**
					129	128	3	**0.58**
					131	130	3	**1.05**
**1299**	TNXB P22105$_{1,3}$: Tenascin-X	2/5	70	96	127	126	90	**0.78**
					129	128	91	**1.08**
					131	130	85	**1.60**
**1300**	TPI1 P60174$_{1,3,4}$: Triosephosphate isomerase	3/3	2	2	127	126	2	**0.53**
					129	128	2	**0.34**
					131	130	2	**0.70**
**1304**	TPM1 P09493$_5$: Tropomyosin alpha-1 chain		16	24	127	126	1	**0.64**
					129	128	1	**1.51**
					131	130	1	**0.92**
**1309**	TPM2 P07951$_1$: Tropomyosin beta chain		25	39	127	126	6	**0.83**
					129	128	5	**3.57**
					131	130	6	**0.96**
**1310**	TPM2 P07951$_2$: Tropomyosin beta chain	1/2	24	41	127	126	9	**0.58**
					129	128	9	**2.93**
					131	130	7	**1.17**
**1312**	TPM3 P06753$_2$: Tropomyosin alpha-3 chain	1/3	21	36	127	126	6	**0.65**
					129	128	6	**0.53**
					131	130	4	**0.83**
**1314**	TPM4 P67936$_1$: Tropomyosin alpha-4 chain		19	30	127	126	4	**0.23**
					129	128	4	**0.56**
					131	130	3	**0.84**

#	protein	group	peptides	spectra	ch1	ch2	quant	ratio
**1315**	TPM4 P67936$_2$: Tropomyosin alpha-4 chain		18	29	127	126	1	**0.58**
					129	128	1	**0.16**
					131	130	1	**1.07**
**1316**	TPP1 O14773$_1$: Tripeptidyl-peptidase 1		2	3	127	126	3	**0.59**
					129	128	3	**1.62**
					131	130	3	**0.92**
**1317**	TRANK1 O15050: TPR and ankyrin repeat-containing protein 1		6	11	127	126	3	**0.59**
					129	128	4	**0.69**
					131	130	3	**0.72**
**1318**	TRIM29 Q14134$_{1,2}$: Tripartite motif-containing protein 29	2/2	10	11	127	126	9	**0.38**
					129	128	10	**0.14**
					131	130	10	**0.67**
**1319**	TSKU Q8WUA8: Tsukushin		9	19	127	126	18	**0.86**
					129	128	15	**1.02**
					131	130	19	**2.23**
**1320**	TTN Q8WZ42$_{12}$: Titin	1/12	41	64	127	126	38	**1.03**
					129	128	36	**0.70**
					131	130	39	**0.56**
**1321**	TTR P02766: Transthyretin		10	131	127	126	125	**1.42**
					129	128	121	**1.02**
					131	130	128	**1.03**
**1324**	TUBA1C Q9BQE3: Tubulin alpha-1C chain		16	37	127	126		
					129	128	1	**0.77**
					131	130	1	**1.59**
**1326**	TUBA3E Q6PEY2: Tubulin alpha-3E chain		13	23	127	126	1	**1.47**
					129	128	1	**0.82**
					131	130	1	**0.46**
**1327**	TUBA4A P68366$_{1,2}$: Tubulin alpha-4A chain	2/2	16	38	127	126	3	**0.97**
					129	128	3	**0.47**
					131	130	3	**0.80**
**1328**	TUBA8 Q9NY65$_{1,2}$: Tubulin alpha-8 chain	2/2	11	24	127	126	1	**0.64**
					129	128	1	**0.45**
					131	130	1	**1.63**
**1329**	TUBAL3 A6NHL2$_{1,2}$: Tubulin alpha chain-like 3	2/2	4	11	127	126	1	**0.82**
					129	128	1	**0.84**
					131	130	1	**0.70**

#	protein	group	peptides	spectra	ch1	ch2	quant	ratio
					127	126	31	**0.87**
**1330**	TUBB P07437: Tubulin beta chain	1/10	14	45	129	128	32	**0.63**
					131	130	30	**1.09**
					127	126	1	**0.68**
**1331**	TUBB6 Q9BUF5: Tubulin beta-6 chain		6	14	129	128	1	**1.14**
					131	130	1	**0.99**
					127	126	3	**0.69**
**1332**	TUFM P49411: Elongation factor Tu, mitochondrial		5	5	129	128	4	**0.25**
					131	130	3	**0.17**
					127	126	5	**1.31**
**1333**	TWSG1 Q9GZX9$_1$: Twisted gastrulation protein homolog 1	1/2	5	10	129	128	6	**1.58**
					131	130	7	**1.09**
					127	126	7	**1.33**
**1334**	TXNDC5 Q8NBS9$_1$: Thioredoxin domain-containing protein 5	1/2	7	9	129	128	8	**0.81**
					131	130	8	**0.52**
					127	126	5	**0.70**
**1335**	TYMP P19971$_1$: Thymidine phosphorylase	1/2	6	6	129	128	5	**0.77**
					131	130	6	**1.13**
					127	126	12	**1.10**
**1336**	UBA1 P22314$_{1,2}$: Ubiquitin-like modifier-activating enzyme 1	2/2	10	12	129	128	12	**0.50**
					131	130	11	**1.17**
					127	126	1	**0.73**
**1337**	UBE2L3 P68036$_1$: Ubiquitin-conjugating enzyme E2 L3		2	65	129	128	1	**0.32**
					131	130	1	**1.08**
					127	126	5	**1.78**
**1338**	UBE2N P61088, UBE2NL Q5JXB2: Ubiquitin-conjugating enzyme E2 N, Putative ubiquitin-conjugating enzyme E2 N-like	2/2	4	5	129	128	4	**0.39**
					131	130	5	**1.52**
					127	126	7	**0.65**
**1339**	UBR4 Q5T4S7$_{1-4}$: E3 ubiquitin-protein ligase UBR4	4/4	5	10	129	128	6	**1.80**
					131	130	6	**1.33**
					127	126	3	**0.64**
**1340**	UCHL3 P15374: Ubiquitin carboxyl-terminal hydrolase isozyme L3		2	3	129	128	3	**0.55**
					131	130	3	**1.19**
					127	126	8	**1.36**
**1341**	UGDH O60701$_1$: UDP-glucose 6-dehydrogenase	1/3	7	9	129	128	8	**1.27**
					131	130	9	**1.94**

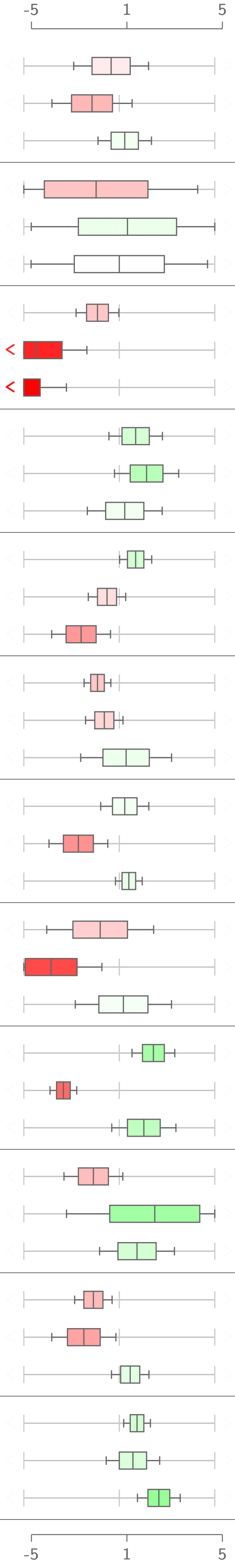

#	protein	group	peptides	spectra	ch1	ch2	quant	ratio
**1342**	UGGT1 Q9NYU2$_{1,2}$: UDP-glucose:glycoprotein glucosyltransferase 1	2/2	2	2	127	126	2	**0.82**
					129	128	2	**0.57**
					131	130	2	**0.57**
**1343**	UGP2 Q16851$_{1,2}$: UTP--glucose-1-phosphate uridylyltransferase	2/2	5	5	127	126	5	**3.00**
					129	128	5	**0.57**
					131	130	4	**1.39**
**1344**	UPF1 Q92900$_{1,2}$: Regulator of nonsense transcripts 1	2/2	2	2	127	126	2	**1.11**
					129	128	2	**0.53**
					131	130	2	**1.11**
**1345**	UQCRB P14927$_{1,2}$: Cytochrome b-c1 complex subunit 7	2/2	2	2	127	126	1	**0.90**
					129	128	1	**0.38**
					131	130	1	**0.23**
**1346**	UQCRC1 P31930: Cytochrome b-c1 complex subunit 1, mitochondrial		2	2	127	126	2	**0.90**
					129	128	2	**0.71**
					131	130	2	**0.24**
**1347**	UQCRC2 P22695: Cytochrome b-c1 complex subunit 2, mitochondrial		4	10	127	126	10	**3.53**
					129	128	7	**0.56**
					131	130	9	**1.41**
**1348**	UQCRH P07919: Cytochrome b-c1 complex subunit 6, mitochondrial		2	2	127	126	2	**0.75**
					129	128	2	**1.48**
					131	130	2	**0.15**
**1349**	USO1 O60763$_{1,2}$: General vesicular transport factor p115	2/2	6	7	127	126	6	**0.97**
					129	128	7	**0.62**
					131	130	7	**1.58**
**1350**	USP14 P54578$_{1-3}$: Ubiquitin carboxyl-terminal hydrolase 14	3/3	2	2	127	126	2	**1.55**
					129	128	2	**0.40**
					131	130	2	**1.10**
**1351**	USP5 P45974$_{1,2}$: Ubiquitin carboxyl-terminal hydrolase 5	2/2	4	4	127	126	4	**0.97**
					129	128	4	**0.78**
					131	130	3	**1.13**
**1352**	USP9X Q93008$_{1,3}$: Probable ubiquitin carboxyl-terminal hydrolase FAF-X	2/2	8	58	127	126	9	**0.92**
					129	128	10	**1.55**
					131	130	8	**1.22**
**1353**	USP9Y O00507$_{1}$: Probable ubiquitin carboxyl-terminal hydrolase FAF-Y	1/2	11	56	127	126	5	**0.49**
					129	128	5	**1.00**
					131	130	6	**0.87**

#	protein	group	peptides	spectra	ch1	ch2	quant	ratio
1354	UTP20 O75691: Small subunit processome component 20 homolog		2	3	127	126	3	**2.03**
					129	128	3	**0.59**
					131	130	3	**0.54**
1355	VARS P26640$_1$: Valine--tRNA ligase		2	2	127	126	2	**0.86**
					129	128	2	**0.84**
					131	130	2	**1.09**
1356	VAT1 Q99536$_1$: Synaptic vesicle membrane protein VAT-1 homolog	1/3	7	8	127	126	8	**0.85**
					129	128	8	**0.59**
					131	130	8	**1.35**
1357	VCAM1 P19320$_{1,3}$: Vascular cell adhesion protein 1	2/3	9	11	127	126	10	**1.33**
					129	128	11	**0.82**
					131	130	11	**1.40**
1358	VCL P18206$_{1,2}$: Vinculin	2/3	29	39	127	126	38	**2.28**
					129	128	35	**0.34**
					131	130	37	**1.21**
1359	VCP P55072: Transitional endoplasmic reticulum ATPase		11	13	127	126	12	**0.52**
					129	128	11	**0.38**
					131	130	12	**1.01**
1360	VDAC1 P21796: Voltage-dependent anion-selective channel protein 1		2	2	127	126	2	**0.99**
					129	128	2	**0.30**
					131	130	2	**0.05**
1361	VEGFC P49767: Vascular endothelial growth factor C		17	26	127	126	21	**0.61**
					129	128	22	**2.04**
					131	130	21	**1.39**
1362	VGF O15240: Neurosecretory protein VGF [Cleaved into: Neuroendocrine regulatory peptide-1		10	21	127	126	15	**0.79**
					129	128	18	**1.43**
					131	130	16	**1.30**
1363	VIM P08670: Vimentin		38	110	127	126	64	**0.68**
					129	128	67	**1.35**
					131	130	60	**0.68**
1364	VLDLR P98155$_{1,2}$: Very low-density lipoprotein receptor	2/2	5	14	127	126	8	**0.59**
					129	128	9	**1.60**
					131	130	9	**1.16**
1365	VPS35 Q96QK1: Vacuolar protein sorting-associated protein 35		2	2	127	126	2	**1.10**
					129	128	2	**0.52**
					131	130	2	**1.22**

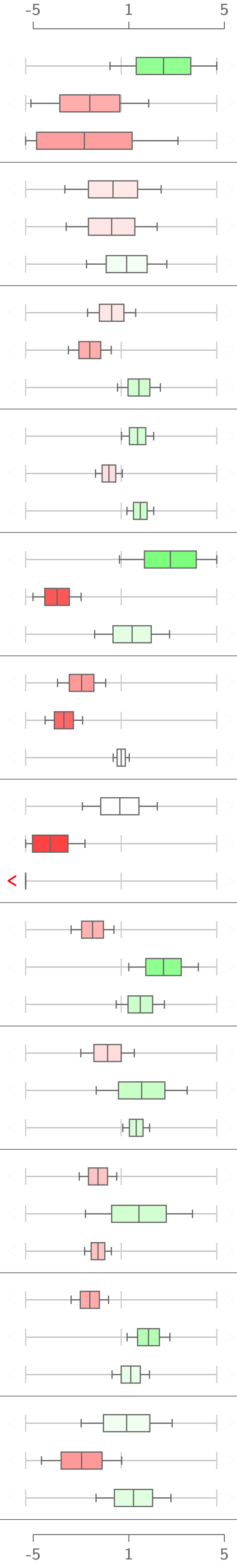

#	protein	group	peptides	spectra	ch1	ch2	quant	ratio
					127	126	760	**0.53**
**1366**	VTN P04004: Vitronectin		20	802	129	128	753	**3.51**
					131	130	750	**1.72**
					127	126	138	**1.47**
**1367**	VWF $P04275_1$: von Willebrand factor	1/2	83	142	129	128	139	**0.45**
					131	130	131	**1.43**
					127	126	3	**1.50**
**1368**	WARS $P23381_1$: Tryptophan--tRNA ligase, cytoplasmic	1/2	4	4	129	128	3	**0.61**
					131	130	4	**1.95**
					127	126	6	**2.89**
**1369**	WDR87 $Q6ZQQ6_{1,2}$: WD repeat-containing protein 87	2/2	5	12	129	128	8	**0.69**
					131	130	7	**0.27**
					127	126	2	**0.96**
**1370**	WFDC1 Q9HC57: WAP four-disulfide core domain protein 1		2	2	129	128	2	**2.96**
					131	130	2	**1.63**
					127	126	18	**0.38**
**1371**	WISP2 $O76076_1$: WNT1-inducible-signaling pathway protein 2	1/2	10	21	129	128	20	**2.03**
					131	130	17	**1.27**
					127	126	3	**2.08**
**1372**	XAB2 Q9HCS7: Pre-mRNA-splicing factor SYF1		3	6	129	128	5	**1.13**
					131	130	5	**0.45**
					127	126	2	**0.83**
**1373**	XPNPEP1 $Q9NQW7_{1,3}$: Xaa-Pro aminopeptidase 1	2/2	2	2	129	128	2	**0.53**
					131	130	2	**1.14**
					127	126	6	**3.51**
**1374**	XPO1 O14980: Exportin-1		5	7	129	128	6	**0.95**
					131	130	6	**0.28**
					127	126	8	**1.11**
**1375**	XRCC5 P13010: X-ray repair cross-complementing protein 5		5	8	129	128	8	**0.44**
					131	130	8	**1.23**
					127	126	10	**1.27**
**1376**	XRCC6 $P12956_{1,2}$: X-ray repair cross-complementing protein 6	2/2	11	13	129	128	11	**0.49**
					131	130	12	**1.39**
					127	126	5	**1.23**
**1377**	YKT6 $O15498_{1,2}$: Synaptobrevin homolog YKT6	2/2	4	5	129	128	4	**0.61**
					131	130	5	**1.18**

#	protein	group	peptides	spectra	ch1	ch2	quant	ratio
**1378**	YWHAB P31946$_{1,2}$: 14-3-3 protein beta/alpha	2/2	9	22	127	126	1	**0.97**
					129	128	1	**0.72**
					131	130	1	**0.90**
**1379**	YWHAE P62258$_{1}$: 14-3-3 protein epsilon	1/2	12	27	127	126	17	**0.57**
					129	128	21	**1.07**
					131	130	20	**1.35**
**1380**	YWHAG P61981: 14-3-3 protein gamma		16	29	127	126	12	**0.67**
					129	128	11	**1.59**
					131	130	10	**1.22**
**1381**	YWHAH Q04917: 14-3-3 protein eta		15	28	127	126	16	**0.70**
					129	128	16	**1.19**
					131	130	15	**1.27**
**1382**	YWHAQ P27348: 14-3-3 protein theta		15	30	127	126	16	**0.71**
					129	128	14	**0.46**
					131	130	13	**1.05**
**1383**	YWHAZ P63104$_{1}$: 14-3-3 protein zeta/delta	1/2	17	37	127	126	23	**0.78**
					129	128	22	**0.63**
					131	130	19	**1.28**
**1384**	ZFX P17010$_{1,3}$: Zinc finger X-chromosomal protein	2/2	9	48	127	126	1	**0.54**
					129	128	1	**0.24**
					131	130		
**1385**	ZFX P17010$_{2}$: Zinc finger X-chromosomal protein		9	79	127	126	23	**1.36**
					129	128	25	**0.49**
					131	130	28	**0.42**
**1386**	ZNF106 Q9H2Y7$_{1}$: Zinc finger protein 106		6	41	127	126	33	**1.27**
					129	128	37	**0.82**
					131	130	36	**0.70**
**1387**	ZNF318 Q5VUA4$_{1,2}$: Zinc finger protein 318	2/2	3	3	127	126	1	**1.98**
					129	128	1	**0.80**
					131	130	1	**0.29**
**1388**	ZNF33B Q06732: Zinc finger protein 33B		5	13	127	126	5	**0.96**
					129	128	11	**1.27**
					131	130	9	**1.06**
**1389**	ZNF827 Q17R98$_{1-3}$: Zinc finger protein 827	3/3	5	16	127	126	7	**0.53**
					129	128	9	**1.34**
					131	130	8	**1.49**

#	protein	group	peptides	spectra	ch1	ch2	quant	ratio
**1390**	ZYX Q15942$_{1,2}$: Zyxin	2/2	2	2	127	126	2	**1.13**
					129	128	2	**0.67**
					131	130	2	**0.93**
**1391**	ZZEF1 O43149$_1$: Zinc finger ZZ-type and EF-hand domain-containing protein 1	1/2	10	44	127	126	25	**1.42**
					129	128	22	**1.04**
					131	130	26	**0.85**

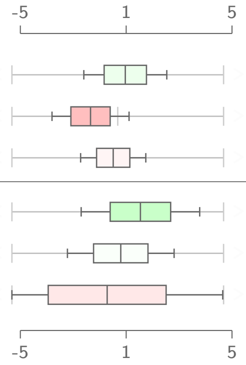

1.2 Not Quantified Proteins

Number of identified but not quantified proteins: P1-treat/P1-ctrl: 68; P2-treat/P2-ctrl: 68; P3-treat/P3-ctrl: 70

#	protein	group	peptides	spectra
543	HIST1H2BC P62807, HIST1H2BD P58876, HIST1H2BH Q93079, . . .: Histone H2B type 1-C/E/F/G/I, Histone H2B type 1-D, . . .	7/9	12	49
447	FCN3 O75636$_2$: Ficolin-3		11	49
732	KRT36 O76013$_{1,2}$: Keratin, type I cuticular Ha6	2/2	7	21
1059	RAB3D O95716: Ras-related protein Rab-3D		2	2
642	IGHV3-33 P01772: Immunoglobulin heavy variable 3-33		2	7
555	HLA-B P01889: HLA class I histocompatibility antigen, B-7 alpha chain		8	11
549	HLA-A P01891: HLA class I histocompatibility antigen, A-68 alpha chain		5	6
550	HLA-A P04439, HLA-A P13746$_{1,2}$: HLA class I histocompatibility antigen, A-3 alpha chain, HLA class I histocompatibility antigen, A-11 alpha chain	3/5	5	7
1311	TPM3 P06753$_1$: Tropomyosin alpha-3 chain		16	25
1313	TPM3 P06753$_5$: Tropomyosin alpha-3 chain	1/3	22	35
542	HIST1H2BB P33778, HIST1H2BJ P06899, HIST1H2BO P23527, . . .: Histone H2B type 1-B, Histone H2B type 1-J, . . .	4/6	11	45
1301	TPM1 P09493$_1$: Tropomyosin alpha-1 chain		22	31
1302	TPM1 P09493$_{4,10}$: Tropomyosin alpha-1 chain	2/2	23	32
1303	TPM1 P09493$_3$: Tropomyosin alpha-1 chain	1/2	23	33
1305	TPM1 P09493$_6$: Tropomyosin alpha-1 chain		21	29
1306	TPM1 P09493$_7$: Tropomyosin alpha-1 chain		21	30
1307	TPM1 P09493$_8$: Tropomyosin alpha-1 chain		22	31
1308	TPM1 P09493$_9$: Tropomyosin alpha-1 chain		22	32
658	IGLC6 P0CF74: Ig lambda-6 chain C region		4	129
885	NEFH P12036$_{1,2}$: Neurofilament heavy polypeptide	2/2	4	25
485	GFAP P14136$_{1-3}$: Glial fibrillary acidic protein	3/3	3	16
552	HLA-A P16188: HLA class I histocompatibility antigen, A-30 alpha chain	1/3	6	9
553	HLA-A P16190: HLA class I histocompatibility antigen, A-33 alpha chain	1/5	6	8
605	HSPA6 P17066: Heat shock 70 kDa protein 6		6	13
735	KRT4 P19013: Keratin, type II cytoskeletal 4		11	65
557	HLA-B P30460, HLA-B P30480: HLA class I histocompatibility antigen, B-8 alpha chain, HLA class I histocompatibility antigen, B-42 alpha chain	2/12	10	13
559	HLA-B P30486, HLA-B Q31610: HLA class I histocompatibility antigen, B-48 alpha chain, HLA class I histocompatibility antigen, B-81 alpha chain	2/2	6	6
560	HLA-B P30492: HLA class I histocompatibility antigen, B-54 alpha chain		6	9
564	HLA-C P30501: HLA class I histocompatibility antigen, Cw-2 alpha chain		8	11

#	protein	group	peptides	spectra
565	HLA-C P30508, HLA-C Q29963: HLA class I histocompatibility antigen, Cw-12 alpha chain, HLA class I histocompatibility antigen, Cw-6 alpha chain	2/7	10	14
554	HLA-A P30512: HLA class I histocompatibility antigen, A-29 alpha chain	1/6	6	8
56	ALDH1B1 P30837: Aldehyde dehydrogenase X, mitochondrial		3	3
368	DSG3 P32926: Desmoglein-3		3	4
24	ACTN2 $P35609_{2}$: Alpha-actinin-2	1/2	12	13
718	KRT20 P35900: Keratin, type I cytoskeletal 20		5	20
1020	PRPH $P41219_{1,2}$: Peripherin	2/2	5	17
739	KRT6C P48668: Keratin, type II cytoskeletal 6C		43	196
501	GNB2 $P62879_{1}$, GNB4 Q9HAV0: Guanine nucleotide-binding protein G(I)/G(S)/G(T) subunit beta-2, Guanine nucleotide-binding protein subunit beta-4	2/3	3	3
1323	TUBA1B $P68363_{1}$: Tubulin alpha-1B chain	1/2	17	43
746	KRT76 Q01546: Keratin, type II cytoskeletal 2 oral		12	88
536	HIST1H1A Q02539: Histone H1.1		5	8
561	HLA-B Q04826: HLA class I histocompatibility antigen, B-40 alpha chain		8	11
1325	TUBA3C $Q13748_{1}$: Tubulin alpha-3C/D chain	1/2	15	31
979	POSTN $Q15063_{2}$: Periostin		43	164
980	POSTN $Q15063_{3}$: Periostin		45	193
981	POSTN $Q15063_{4}$: Periostin		42	161
982	POSTN $Q15063_{5}$: Periostin		46	196
983	POSTN $Q15063_{6}$: Periostin		40	156
984	POSTN $Q15063_{7}$: Periostin		39	153
698	KIF7 Q2M1P5: Kinesin-like protein KIF7		2	3
562	HLA-B Q31612: HLA class I histocompatibility antigen, B-73 alpha chain		8	10
956	PHLDB3 $Q6NSJ2_{1}$: Pleckstrin homology-like domain family B member 3		2	2
920	PAMR1 $Q6UXH9_{1}$: Inactive serine protease PAMR1		12	28
922	PAMR1 $Q6UXH9_{3}$: Inactive serine protease PAMR1		12	29
1322	TUBA1A $Q71U36_{1,2}$: Tubulin alpha-1A chain	2/2	17	40
747	KRT77 Q7Z794: Keratin, type II cytoskeletal 1b		5	44
548	HIST3H2BB Q8N257: Histone H2B type 3-B		10	39
1263	TET1 Q8NFU7: Methylcytosine dioxygenase TET1		2	2
567	HLA-C Q95604: HLA class I histocompatibility antigen, Cw-17 alpha chain		7	8
908	OPTN $Q96CV9_{1-3}$: Optineurin	3/3	2	10
818	MAP7D2 $Q96T17_{1,2,4,5}$: MAP7 domain-containing protein 2	4/4	2	2
544	HIST1H2BL Q99880: Histone H2B type 1-L	1/2	12	46
811	LZTS2 Q9BRK4: Leucine zipper putative tumor suppressor 2		2	2
760	KRTAP3-3 Q9BYR6: Keratin-associated protein 3-3		1	1
511	GRID1 $Q9ULK0_{1}$: Glutamate receptor ionotropic, delta-1		2	19
360	DOPEY2 $Q9Y3R5_{1,2}$: Protein dopey-2	2/2	2	3

2 Protein Group Details

Protein groups are created by grouping according to mass spectrometry evidence:

1. Proteins which are detected with the exact same set of peptides are clustered - they are indistinguishable based on available MS data. Very often these are splice variants. In the following text, proteins are clustered proteins (cluster size one if they have specific peptides).
2. Proteins, which have unique peptides (*i. e.* peptides not present in other detected proteins) are promoted to *reporters*.
3. Remaining proteins are grouped to those reporters which have a superset of the peptides they have. These proteins are termed *group members*

Peptides are characterized based on their specificity to one or more proteins, and one or more protein groups:

reporter specific rs	●	Number of peptides specific to reporter.
group specific gs	■	Number of peptides specific to the group (including rs).
unspecific us	○	Number of peptides shared with any protein.

1		125116, NA, NA	1/0/20
2		136429, NA, NA	6/0/1
3		162648, NA, NA	17/0/6
4		A0A075B6Q5, NA, NA	1/0/0
5		A0A0A0MS15, NA, NA	1/0/0
6		A0A0C4DH25, NA, NA	1/0/2
7	(1)	A0A0C4DH29, NA, NA	1/0/2
	(2)	P01743, IGHV1-46, Immunoglobulin heavy variable 1-46	
8		A0A0C4DH33, NA, NA	1/0/1
9	(1)	P04217-1, A1BG, Alpha-1B-glycoprotein	4/0/0
	(2)	P04217-2, A1BG, Alpha-1B-glycoprotein	
10		P01023, A2M, Alpha-2-macroglobulin	46/0/8
11		Q9NPC4, A4GALT, Lactosylceramide 4-alpha-galactosyltransferase	2/0/0
12		P49588-1,2, AARS, Alanine--tRNA ligase, cytoplasmic	8/0/0
13		Q9BUJ0, ABHD14A, Protein ABHD14A	2/0/0
14		Q7Z7G0-1, ABI3BP, Target of Nesh-SH3	2/0/0
15		P42765, ACAA2, 3-ketoacyl-CoA thiolase, mitochondrial	4/0/0
16		P49748-1-3, ACADVL, Very long-chain specific acyl-CoA dehydrogenase, mitochondrial	4/0/0
17	(1)	P53396-1, ACLY, ATP-citrate synthase	2/0/0
	(2)	P53396-2,3, ACLY, ATP-citrate synthase	
18		Q99798, ACO2, Aconitate hydratase, mitochondrial	5/0/0
19		P60709, ACTB, Actin, cytoplasmic 1	5/0/15
		P63261, ACTG1, Actin, cytoplasmic 2	
20		Q562R1, ACTBL2, Beta-actin-like protein 2	3/0/6

21 (1) P68032, ACTC1, Actin, alpha cardiac muscle 1
(2) P68133, ACTA1, Actin, alpha skeletal muscle
(3) P62736, ACTA2, Actin, aortic smooth muscle

0/1/16 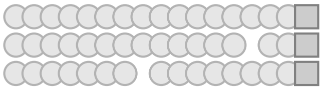

22 (1) P63267-1, ACTG2, Actin, gamma-enteric smooth muscle
(2) P63267-2, ACTG2, Actin, gamma-enteric smooth muscle

0/0/15 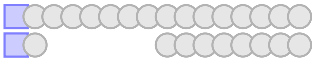

23 (1) P12814-1,3, ACTN1, Alpha-actinin-1
(2) P12814-2,4, ACTN1, Alpha-actinin-1

1/0/17 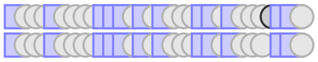

24 (1) P35609-2, ACTN2, Alpha-actinin-2
(2) P35609-1, ACTN2, Alpha-actinin-2

0/0/12 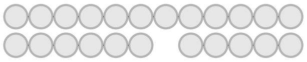

25 Q08043, ACTN3, Alpha-actinin-3

1/0/7 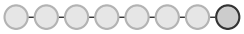

26 (1) O43707-1, ACTN4, Alpha-actinin-4
(2) O43707-2, ACTN4, Alpha-actinin-4
(3) O43707-3, ACTN4, Alpha-actinin-4

1/11/14 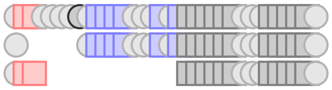

27 P61163, ACTR1A, Alpha-centractin

3/0/1 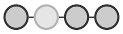

28 (1) P61160-1, ACTR2, Actin-related protein 2
(2) P61160-2, ACTR2, Actin-related protein 2

1/0/1 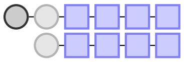

29 P61158, ACTR3, Actin-related protein 3

4/0/0 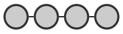

30 O14672-1, ADAM10, Disintegrin and metalloproteinase domain-containing protein 10

8/0/0 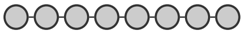

31 (1) Q13444-1-10,12, ADAM15, Disintegrin and metalloproteinase domain-containing protein 15
(2) Q13444-11, ADAM15, Disintegrin and metalloproteinase domain-containing protein 15
(3) Q13444-13, ADAM15, Disintegrin and metalloproteinase domain-containing protein 15

0/0/0 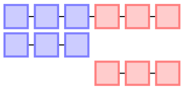

32 O15204-1,2, ADAMDEC1, ADAM DEC1

6/0/0 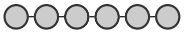

33 Q76LX8-1-3, ADAMTS13, A disintegrin and metalloproteinase with thrombospondin motifs 13

4/0/0 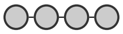

34 Q8TE58, ADAMTS15, A disintegrin and metalloproteinase with thrombospondin motifs 15

2/0/0 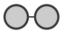

35 (1) O95450-1, ADAMTS2, A disintegrin and metalloproteinase with thrombospondin motifs 2
(2) O95450-2, ADAMTS2, A disintegrin and metalloproteinase with thrombospondin motifs 2

1/0/0 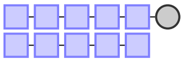

36 Q9UNA0, ADAMTS5, A disintegrin and metalloproteinase with thrombospondin motifs 5

2/0/0 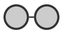

37 Q9UP79, ADAMTS8, A disintegrin and metalloproteinase with thrombospondin motifs 8

5/0/0 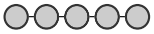

3/0/0

38 (1) Q6UY14-1,3, ADAMTSL4, ADAMTS-like protein 4

(2) Q6UY14-2, ADAMTSL4, ADAMTS-like protein 4

1/0/2

39 P00325-1,2, ADH1B, Alcohol dehydrogenase 1B

0/0/2

40 (1) P00326, ADH1C, Alcohol dehydrogenase 1C

(2) P07327, ADH1A, Alcohol dehydrogenase 1A

9/0/0

41 P11766, ADH5, Alcohol dehydrogenase class-3

4/0/0

42 Q15848, ADIPOQ, Adiponectin

18/0/1

43 (1) Q8IUX7-1, AEBP1, Adipocyte enhancer-binding protein 1

(2) Q8IUX7-2, AEBP1, Adipocyte enhancer-binding protein 1

33/0/0

44 P43652, AFM, Afamin

2/0/0

45 (1) O00468-3,6, AGRN, Agrin [Cleaved into: Agrin N-terminal 110 kDa subunit; Agrin C-terminal 110 kDa subunit; Agrin C-terminal 90 kDa fragment

(2) O00468-1,2,4,5,7, AGRN, Agrin [Cleaved into: Agrin N-terminal 110 kDa subunit; Agrin C-terminal 110 kDa subunit; Agrin C-terminal 90 kDa fragment

12/0/0

46 P01019, AGT, Angiotensinogen

1/0/0

47 (1) P23526-1, AHCY, Adenosylhomocysteinase

(2) P23526-2, AHCY, Adenosylhomocysteinase

48 O43865-1,2, AHCYL1, Adenosylhomocysteinase 2

2/0/0

Q96HN2-1-4, AHCYL2, Adenosylhomocysteinase 3

26/0/1

49 (1) Q09666-1, AHNAK, Neuroblast differentiation-associated protein AHNAK

(2) Q09666-2, AHNAK, Neuroblast differentiation-associated protein AHNAK

1/0/0

50 (1) O95433-1, AHSA1, Activator of 90 kDa heat shock protein ATPase homolog 1

(2) O95433-2, AHSA1, Activator of 90 kDa heat shock protein ATPase homolog 1

12/0/0

51 P02765, AHSG, Alpha-2-HS-glycoprotein

1/0/0

52 O95831-1-3,5, AIFM1, Apoptosis-inducing factor 1, mitochondrial

9/24/6

53 (1) P02768-1, ALB, Serum albumin

(2) P02768-2, ALB, Serum albumin

(3) P02768-3, ALB, Serum albumin

0/5/3

54 (1) O94788-1, ALDH1A2, Retinal dehydrogenase 2

(2) O94788-2, ALDH1A2, Retinal dehydrogenase 2
(3) O94788-3, ALDH1A2, Retinal dehydrogenase 2
(4) O94788-4, ALDH1A2, Retinal dehydrogenase 2

55 P47895, ALDH1A3, Aldehyde dehydrogenase family 1 member A3 — 5/0/3

56 P30837, ALDH1B1, Aldehyde dehydrogenase X, mitochondrial — 0/0/3

57 (1) P05091-1, ALDH2, Aldehyde dehydrogenase, mitochondrial — 0/0/1
(2) P05091-2, ALDH2, Aldehyde dehydrogenase, mitochondrial
(3) P00352, ALDH1A1, Retinal dehydrogenase 1

58 P49419-1,2, ALDH7A1, Alpha-aminoadipic semialdehyde dehydrogenase — 2/0/0

1/0/0

59 (1) P49189-1, ALDH9A1, 4-trimethylaminobutyraldehyde dehydrogenase
(2) P49189-2, ALDH9A1, 4-trimethylaminobutyraldehyde dehydrogenase

60 (1) P04075-1,2, ALDOA, Fructose-bisphosphate aldolase A — 6/0/0
(2) P09972, ALDOC, Fructose-bisphosphate aldolase C

61 P16050-1,2, ALOX15, Arachidonate 15-lipoxygenase — 3/0/0

62 P02760, AMBP, Protein AMBP [Cleaved into: Alpha-1-microglobulin — 15/0/0

63 P03950, ANG, Angiogenin — 13/0/0

7/0/0

64 (1) Q9UKU9-1, ANGPTL2, Angiopoietin-related protein 2
(2) Q9UKU9-2, ANGPTL2, Angiopoietin-related protein 2

65 Q9Y5C1, ANGPTL3, Angiopoietin-related protein 3 — 3/0/0

0/3/0

66 (1) Q9BY76-1, ANGPTL4, Angiopoietin-related protein 4
(2) Q9BY76-2, ANGPTL4, Angiopoietin-related protein 4
(3) Q9BY76-3, ANGPTL4, Angiopoietin-related protein 4

4/0/2

67 (1) Q9H6X2-1,5, ANTXR1, Anthrax toxin receptor 1
(2) Q9H6X2-2-4,6, ANTXR1, Anthrax toxin receptor 1

68 P58335-1, ANTXR2, Anthrax toxin receptor 2 — 1/0/11

2/0/11

69 (1) P58335-4, ANTXR2, Anthrax toxin receptor 2
(2) P58335-2, ANTXR2, Anthrax toxin receptor 2
(3) P58335-3, ANTXR2, Anthrax toxin receptor 2

70 P04083, ANXA1, Annexin A1 — 17/0/0

71 P50995-1,2, ANXA11, Annexin A11 — 3/0/0

72 (1) P07355-1,2, ANXA2, Annexin A2 — 5/0/0
(2) A6NMY6, ANXA2P2, Putative annexin A2-like protein

73		P09525-1,2, ANXA4, Annexin A4	5/0/1
74		P08758, ANXA5, Annexin A5	7/0/1
75		P08133-1,2, ANXA6, Annexin A6	7/0/0
76		P20073-1,2, ANXA7, Annexin A7	2/0/0
77	(1)	Q10567-1-4, AP1B1, AP-1 complex subunit beta-1	1/3/0
	(2)	P63010-1,2, AP2B1, AP-2 complex subunit beta	
	(3)	P63010-3, AP2B1, AP-2 complex subunit beta	
78		O43747-1,2, AP1G1, AP-1 complex subunit gamma-1	1/0/0
79	(1)	O95782-1,2, AP2A1, AP-2 complex subunit alpha-1	2/0/0
	(2)	O94973-1-3, AP2A2, AP-2 complex subunit alpha-2	
80		P02743, APCS, Serum amyloid P-component	10/0/0
81		P13798, APEH, Acylamino-acid-releasing enzyme	3/0/0
82	(1)	Q9HDC9-1, APMAP, Adipocyte plasma membrane-associated protein	2/0/0
	(2)	Q9HDC9-2, APMAP, Adipocyte plasma membrane-associated protein	
83		P02647, APOA1, Apolipoprotein A-I	37/0/0
84		P02652, APOA2, Apolipoprotein A-II	6/0/0
85		P06727, APOA4, Apolipoprotein A-IV	41/0/2
86		Q6Q788, APOA5, Apolipoprotein A-V	2/0/0
87		P04114, APOB, Apolipoprotein B-100	264/0/2
88		Q0VD83-1,3,4, APOBR, Apolipoprotein B receptor	4/0/0
89		P02654, APOC1, Apolipoprotein C-I	6/0/0
90		P02655, APOC2, Apolipoprotein C-II	4/0/0
91		P02656, APOC3, Apolipoprotein C-III	5/0/0
92		P55056, APOC4, Apolipoprotein C-IV	7/0/0
93		P05090, APOD, Apolipoprotein D	11/0/0
94		P02649, APOE, Apolipoprotein E	29/0/0
95		Q13790, APOF, Apolipoprotein F	5/0/0
96		P02749, APOH, Beta-2-glycoprotein 1	13/0/0
97		O14791-1-3, APOL1, Apolipoprotein L1	22/0/0
98		Q9BQE5, APOL2, Apolipoprotein L2	2/0/0
99		O95445-1, APOM, Apolipoprotein M	2/0/10
100		O95445-2, APOM, Apolipoprotein M	2/0/10

0/3/0

101 (1) P05067-1,3-9,11, APP, Amyloid beta A4 protein

(2) P05067-2, APP, Amyloid beta A4 protein

(3) P05067-10, APP, Amyloid beta A4 protein

3/0/0

102 P07741-1,2, APRT, Adenine phosphoribosyltransferase

3/0/0

103 P48444-1,2, ARCN1, Coatomer subunit delta

2/0/0

104 P61204-1,2, ARF3, ADP-ribosylation factor 3

P84077, ARF1, ADP-ribosylation factor 1

5/0/0

105 Q07960, ARHGAP1, Rho GTPase-activating protein 1

1/0/0

106 (1) Q8N392-1, ARHGAP18, Rho GTPase-activating protein 18

(2) Q8N392-2, ARHGAP18, Rho GTPase-activating protein 18

3/0/0

107 Q9NRY4, ARHGAP35, Rho GTPase-activating protein 35

2/0/0

108 (1) P52565-1, ARHGDIA, Rho GDP-dissociation inhibitor 1

(2) P52565-2, ARHGDIA, Rho GDP-dissociation inhibitor 1

3/0/0

109 P52566, ARHGDIB, Rho GDP-dissociation inhibitor 2

2/0/0

110 P36405, ARL3, ADP-ribosylation factor-like protein 3

2/0/0

111 O15511-1,2, ARPC5, Actin-related protein 2/3 complex subunit 5

3/0/0

112 Q13510-1-3, ASAH1, Acid ceramidase

5/0/0

113 P07307-1-3, ASGR2, Asialoglycoprotein receptor 2

1/0/0

114 O95671-1-3, ASMTL, N-acetylserotonin O-methyltransferase-like protein

1/0/0

115 Q12797-1,10, ASPH, Aspartyl/asparaginyl beta-hydroxylase

2/0/0

116 Q9NT62-1,2, ATG3, Ubiquitin-like-conjugating enzyme ATG3

3/0/0

117 P31939-1,2, ATIC, Bifunctional purine biosynthesis protein PURH [Cleaved into: Bifunctional purine biosynthesis protein PURH, N-terminally processed] [Includes: Phosphoribosylaminoimidazolecarboxamide formyltransferase

3/0/0

118 Q6DD88, ATL3, Atlastin-3

1/0/2

119 O14983-1, ATP2A1, Sarcoplasmic/endoplasmic reticulum calcium ATPase 1

1/0/2

120 (1) P16615-1,4, ATP2A2, Sarcoplasmic/endoplasmic reticulum calcium ATPase 2

(2) P16615-2,3,5, ATP2A2, Sarcoplasmic/endoplasmic reticulum calcium ATPase 2

1/0/1

121 Q16720-1-8, ATP2B3, Plasma membrane calcium-transporting ATPase 3

122		P23634-1,6-8, ATP2B4, Plasma membrane calcium-transporting ATPase 4	1/0/1
			0/18/0
123	(1)	P25705-1, ATP5A1, ATP synthase subunit alpha, mitochondrial	
	(2)	P25705-2, ATP5A1, ATP synthase subunit alpha, mitochondrial	
	(3)	P25705-3, ATP5A1, ATP synthase subunit alpha, mitochondrial	
124		P06576, ATP5B, ATP synthase subunit beta, mitochondrial	10/0/0
125		P36542-1,2, ATP5C1, ATP synthase subunit gamma, mitochondrial	5/0/1
126		O75947-1,2, ATP5H, ATP synthase subunit d, mitochondrial	2/0/0
127		P56385, ATP5I, ATP synthase subunit e, mitochondrial	2/0/0
128		Q15904, ATP6AP1, V-type proton ATPase subunit S1	2/0/0
129		O75787-1,2, ATP6AP2, Renin receptor	3/0/0
130	(1)	P36543-1,3, ATP6V1E1, V-type proton ATPase subunit E 1	0/1/0
	(2)	Q96A05, ATP6V1E2, V-type proton ATPase subunit E 2	
	(3)	P36543-2, ATP6V1E1, V-type proton ATPase subunit E 1	
131		P25311, AZGP1, Zinc-alpha-2-glycoprotein	7/0/0
132		P61769, B2M, Beta-2-microglobulin [Cleaved into: Beta-2-microglobulin form pI 5.3]	5/0/0
133		O94766-1,2, B3GAT3, Galactosylgalactosylxylosylprotein 3-beta-glucuronosyltransferase 3	2/0/0
134		Q9NY97-1,2, B3GNT2, N-acetyllactosaminide beta-1,3-N-acetylglucosaminyltransferase 2	1/0/1
135		P15291-1,2, B4GALT1, Beta-1,4-galactosyltransferase 1	10/0/0
136		O60513, B4GALT4, Beta-1,4-galactosyltransferase 4	4/0/0
137		O43505, B4GAT1, Beta-1,4-glucuronyltransferase 1	4/0/0
138		P51572-1,2, BCAP31, B-cell receptor-associated protein 31	4/0/1
139		P06276, BCHE, Cholinesterase	7/0/0
140		Q5T5X7, BEND3, BEN domain-containing protein 3	2/0/0
141		P21810, BGN, Biglycan	6/0/1
142		P55957-1,2, BID, BH3-interacting domain death agonist	1/0/0
143		P30043, BLVRB, Flavin reductase	3/0/0
144	(1)	P13497-1, BMP1, Bone morphogenetic protein 1	5/8/3
	(2)	P13497-2,5,6, BMP1, Bone morphogenetic protein 1	
	(3)	Q9Y6L7, TLL2, Tolloid-like protein 2	
	(4)	P13497-3, BMP1, Bone morphogenetic protein 1	
	(5)	P13497-4, BMP1, Bone morphogenetic protein 1	

145		P12645, BMP3, Bone morphogenetic protein 3	3/0/0
146		Q9H3K6-1,2, BOLA2, BolA-like protein 2	1/0/0
147	(1)	Q8TDL5-1, BPIFB1, BPI fold-containing family B member 1	4/0/0
	(2)	Q8TDL5-2, BPIFB1, BPI fold-containing family B member 1	
148		Q12830-1,2,4, BPTF, Nucleosome-remodeling factor subunit BPTF	7/0/0
149		P43251-1-4, BTD, Biotinidase	4/0/0
150		Q7L1Q6-1-4, BZW1, Basic leucine zipper and W2 domain-containing protein 1	2/0/0
151		Q6UX73-1,2, C16orf89, UPF0764 protein C16orf89	3/0/1
152		P02745, C1QA, Complement C1q subcomponent subunit A	2/0/0
153		P02746, C1QB, Complement C1q subcomponent subunit B	8/0/0
154		Q07021, C1QBP, Complement component 1 Q subcomponent-binding protein, mitochondrial	1/0/0
155		P02747, C1QC, Complement C1q subcomponent subunit C	5/0/0
156		Q9BXJ3, C1QTNF4, Complement C1q tumor necrosis factor-related protein 4	3/0/0
157		Q9BXJ0, C1QTNF5, Complement C1q tumor necrosis factor-related protein 5	2/0/0
158		P00736, C1R, Complement C1r subcomponent	33/0/1
159		Q9NZP8, C1RL, Complement C1r subcomponent-like protein	10/0/1
160		P09871, C1S, Complement C1s subcomponent	24/0/1
161	(1)	P06681-1, C2, Complement C2	2/24/0
	(2)	P06681-2, C2, Complement C2	
	(3)	P06681-3, C2, Complement C2	
162		Q9H1Z8, C2orf40, Augurin	1/0/0
163		P01024, C3, Complement C3	135/0/0
164	(1)	P0C0L4-1, C4A, Complement C4-A	0/0/85
	(2)	P0C0L4-2, C4A, Complement C4-A	
165		P0C0L5, C4B, Complement C4-B	5/0/85
166		P04003, C4BPA, C4b-binding protein alpha chain	34/0/0
167		P20851-1,2, C4BPB, C4b-binding protein beta chain	12/0/0
168		P01031, C5, Complement C5	77/0/0

169		P13671, C6, Complement component C6	46/0/0
170		P10643, C7, Complement component C7	36/0/0
171		P07357, C8A, Complement component C8 alpha chain	24/0/0
172		P07358, C8B, Complement component C8 beta chain	24/0/0
173		P07360, C8G, Complement component C8 gamma chain	12/0/0
174		P02748, C9, Complement component C9 [Cleaved into: Complement component C9a; Complement component C9b]	27/0/3
175	(1)	Q01668-2, CACNA1D, Voltage-dependent L-type calcium channel subunit alpha-1D	1/0/0
	(2)	Q01668-1,3,4, CACNA1D, Voltage-dependent L-type calcium channel subunit alpha-1D	0/2/1
176	(1)	Q05682-1,2,6, CALD1, Caldesmon	
	(2)	Q05682-3, CALD1, Caldesmon	
	(3)	Q05682-4, CALD1, Caldesmon	
	(4)	Q05682-5, CALD1, Caldesmon	
177		P62158, CALM1, Calmodulin	8/0/2
178		P27482, CALML3, Calmodulin-like protein 3	7/0/2
179		Q9NZT1, CALML5, Calmodulin-like protein 5	2/0/0
180		P27797, CALR, Calreticulin	21/0/0
181	(1)	O43852-1,3, CALU, Calumenin	0/11/1
	(2)	O43852-10, CALU, Calumenin	
	(3)	O43852-11, CALU, Calumenin	
	(4)	O43852-15, CALU, Calumenin	
	(5)	O43852-7,12, CALU, Calumenin	
	(6)	O43852-2,4, CALU, Calumenin	
	(7)	O43852-5, CALU, Calumenin	
	(8)	O43852-6, CALU, Calumenin	
	(9)	O43852-8, CALU, Calumenin	
	(10)	O43852-9, CALU, Calumenin	
	(11)	O43852-13,14, CALU, Calumenin	
182		Q13557-1,3-6,8-12, CAMK2D, Calcium/calmodulin-dependent protein kinase type II subunit delta	1/0/1

183		P49913, CAMP, Cathelicidin antimicrobial peptide	2/0/0
184	(1)	Q86VP6-1, CAND1, Cullin-associated NEDD8-dissociated protein 1	2/3/0
	(2)	Q86VP6-2, CAND1, Cullin-associated NEDD8-dissociated protein 1	
	(3)	Q86VP6-3, CAND1, Cullin-associated NEDD8-dissociated protein 1	
185	(1)	P27824-1,2, CANX, Calnexin	4/0/2
	(2)	P27824-3, CANX, Calnexin	
186	(1)	Q01518-1,2, CAP1, Adenylyl cyclase-associated protein 1	8/0/0
	(2)	P40123-1, CAP2, Adenylyl cyclase-associated protein 2	
187		P40121-1,2, CAPG, Macrophage-capping protein	3/0/0
188		P07384, CAPN1, Calpain-1 catalytic subunit	2/0/2
189	(1)	P17655-1, CAPN2, Calpain-2 catalytic subunit	3/0/2
	(2)	O14815-1,2, CAPN9, Calpain-9	
	(3)	P17655-2, CAPN2, Calpain-2 catalytic subunit	
190		P04632, CAPNS1, Calpain small subunit 1	2/0/0
191		P52907, CAPZA1, F-actin-capping protein subunit alpha-1	4/0/2
192	(1)	P47755-1, CAPZA2, F-actin-capping protein subunit alpha-2	0/0/2
	(2)	P47755-2, CAPZA2, F-actin-capping protein subunit alpha-2	
193	(1)	P47756-2, CAPZB, F-actin-capping protein subunit beta	1/0/0
	(2)	P47756-1, CAPZB, F-actin-capping protein subunit beta	
194		P31944, CASP14, Caspase-14	4/0/0
195		Q03135-1,2, CAV1, Caveolin-1	3/0/0
196	(1)	P16152-1, CBR1, Carbonyl reductase [NADPH] 1	1/0/0
	(2)	O75828, CBR3, Carbonyl reductase [NADPH] 3	
	·	P16152-2, CBR1, Carbonyl reductase [NADPH] 1	
197		Q8IYE0-1, CCDC146, Coiled-coil domain-containing protein 146	1/0/2
198		Q76M96-1,2, CCDC80, Coiled-coil domain-containing protein 80	19/0/0
199	(1)	Q16627-1, CCL14, C-C motif chemokine 14	2/0/0
	(2)	Q16627-2, CCL14, C-C motif chemokine 14	
200		O15467, CCL16, C-C motif chemokine 16	2/0/0
201		P55774, CCL18, C-C motif chemokine 18	2/0/0
202		O14618, CCS, Copper chaperone for superoxide dismutase	1/0/0
203		P78371-1,2, CCT2, T-complex protein 1 subunit beta	6/0/0

204		P49368-1,2, CCT3, T-complex protein 1 subunit gamma	7/0/0
205	(1)	P50991-1, CCT4, T-complex protein 1 subunit delta	1/0/0
	(2)	P50991-2, CCT4, T-complex protein 1 subunit delta	
206	(1)	P48643-1, CCT5, T-complex protein 1 subunit epsilon	2/0/0
	(2)	P48643-2, CCT5, T-complex protein 1 subunit epsilon	
207		P40227-1,2, CCT6A, T-complex protein 1 subunit zeta	3/0/0
208	(1)	P50990-1,2, CCT8, T-complex protein 1 subunit theta	2/0/0
	(2)	P50990-3, CCT8, T-complex protein 1 subunit theta	
209	(1)	Q6YHK3-1,4, CD109, CD109 antigen	0/5/0
	(2)	Q6YHK3-2, CD109, CD109 antigen	
	(3)	Q6YHK3-3, CD109, CD109 antigen	
210		P08571, CD14, Monocyte differentiation antigen CD14	15/0/0
211		Q86VB7-1-4, CD163, Scavenger receptor cysteine-rich type 1 protein M130	5/0/0
212		Q9HCU0-1, CD248, Endosialin	9/0/0
213		P13987, CD59, CD59 glycoprotein	1/0/0
214		O43866, CD5L, CD5 antigen-like	21/0/0
215		P04233-1,2, CD74, HLA class II histocompatibility antigen gamma chain	2/0/0
216		P60033, CD81, CD81 antigen	3/0/0
217		P21926, CD9, CD9 antigen	2/0/0
218	(1)	P48960-1,3, CD97, CD97 antigen	2/0/0
	(2)	P48960-2, CD97, CD97 antigen	
219		P14209-1,3, CD99, CD99 antigen	1/0/0
220		Q16543, CDC37, Hsp90 co-chaperone Cdc37	2/0/0
221	(1)	P60953-1,2, CDC42, Cell division control protein 42 homolog	3/0/1
	(2)	P17081, RHOQ, Rho-related GTP-binding protein RhoQ	
	·	P84095, RHOG, Rho-related GTP-binding protein RhoG	
	·	Q9H4E5-1,2, RHOJ, Rho-related GTP-binding protein RhoJ	
222		Q5VT25-1,2,4-6, CDC42BPA, Serine/threonine-protein kinase MRCK alpha	3/0/0
223		P12830-1,2, CDH1, Cadherin-1	2/0/0
224	(1)	P19022-1, CDH2, Cadherin-2	1/0/0
	(2)	P19022-2, CDH2, Cadherin-2	

225 (1) Q00537-1, CDK17, Cyclin-dependent kinase 17 — 1/0/1
(2) Q00537-2, CDK17, Cyclin-dependent kinase 17

226 P49454, CENPF, Centromere protein F — 1/0/1

227 Q5VT06, CEP350, Centrosome-associated protein 350 — 4/0/1

228 O00748-1,2, CES2, Cocaine esterase — 2/0/0

229 (1) P11597-1, CETP, Cholesteryl ester transfer protein — 5/0/0
(2) P11597-2, CETP, Cholesteryl ester transfer protein

230 (1) P00751-1, CFB, Complement factor B — 10/0/0
(2) P00751-2, CFB, Complement factor B

231 P00746, CFD, Complement factor D — 16/0/0

232 (1) P08603-1, CFH, Complement factor H — 30/0/9
(2) P08603-2, CFH, Complement factor H

233 Q03591, CFHR1, Complement factor H-related protein 1 — 1/0/12

234 (1) P36980-1, CFHR2, Complement factor H-related protein 2 — 1/0/4
(2) P36980-2, CFHR2, Complement factor H-related protein 2

235 Q02985-1,2, CFHR3, Complement factor H-related protein 3 — 3/0/5

236 (1) Q92496-1,2, CFHR4, Complement factor H-related protein 4 — 2/0/5
(2) Q92496-3, CFHR4, Complement factor H-related protein 4

237 Q9BXR6, CFHR5, Complement factor H-related protein 5 — 21/0/2

238 P05156, CFI, Complement factor I — 21/0/0

239 P23528, CFL1, Cofilin-1 — 6/0/1

240 P27918, CFP, Properdin — 12/0/1

241 (1) Q99674-1,4,5, CGREF1, Cell growth regulator with EF hand domain protein 1 — 9/0/0
(2) Q99674-2, CGREF1, Cell growth regulator with EF hand domain protein 1

242 P10645, CHGA, Chromogranin-A — 25/0/0

243 P05060, CHGB, Secretogranin-1 — 20/0/1

244 Q9BY43-1,2, CHMP4A, Charged multivesicular body protein 4a — 1/0/1

245 (1) Q9H2X0-1,5, CHRD, Chordin — 4/0/0
(2) Q9H2X0-4, CHRD, Chordin

			1/15/0
246	(1)	Q9BU40-2,4, CHRDL1, Chordin-like protein 1	
	(2)	Q9BU40-1,6, CHRDL1, Chordin-like protein 1	
	(3)	Q9BU40-3, CHRDL1, Chordin-like protein 1	
	(4)	Q9BU40-5, CHRDL1, Chordin-like protein 1	
			1/0/0
247	(1)	Q6WN34-1, CHRDL2, Chordin-like protein 2	
	(2)	Q6WN34-2, CHRDL2, Chordin-like protein 2	
248		Q8NCH0, CHST14, Carbohydrate sulfotransferase 14	2/0/0
249		O75339, CILP, Cartilage intermediate layer protein 1	46/0/1
250		Q8IUL8, CILP2, Cartilage intermediate layer protein 2	3/0/1
251		Q07065, CKAP4, Cytoskeleton-associated protein 4	14/0/1
252	(1)	P12277, CKB, Creatine kinase B-type	3/0/0
	(2)	P06732, CKM, Creatine kinase M-type	
253		Q05315, CLC, Galectin-10	3/0/0
254		Q9Y240, CLEC11A, C-type lectin domain family 11 member A	12/0/0
255		P05452, CLEC3B, Tetranectin	9/0/0
256		O14967-1, CLGN, Calmegin	1/0/1
257		O00299, CLIC1, Chloride intracellular channel protein 1	5/0/0
258		Q9Y696, CLIC4, Chloride intracellular channel protein 4	4/0/0
			1/0/0
259	(1)	O94985-1, CLSTN1, Calsyntenin-1	
	(2)	O94985-2, CLSTN1, Calsyntenin-1	
			1/0/0
260	(1)	P09497-2, CLTB, Clathrin light chain B	
	(2)	P09497-1, CLTB, Clathrin light chain B	
261		Q00610-1,2, CLTC, Clathrin heavy chain 1	18/0/8
262		P53675-1,2, CLTCL1, Clathrin heavy chain 2	1/0/8
			1/16/0
263	(1)	P10909-4, CLU, Clusterin	
	(2)	P10909-3, CLU, Clusterin	
	(3)	P10909-1,2,5, CLU, Clusterin	
264		Q96KN2, CNDP1, Beta-Ala-His dipeptidase	6/0/0
265		Q99439-1,2, CNN2, Calponin-2	1/0/1
266	(1)	Q15417-1,3, CNN3, Calponin-3	1/2/1
	(2)	Q15417-2, CNN3, Calponin-3	
	(3)	P51911-1,2, CNN1, Calponin-1	

267		Q9UIV1-1, CNOT7, CCR4-NOT transcription complex subunit 7	4/0/0
268		Q9Y2B0-1, CNPY2, Protein canopy homolog 2	2/0/0
			0/25/1
269	(1)	Q99715-1, COL12A1, Collagen alpha-1(XII) chain	
	(2)	Q99715-2, COL12A1, Collagen alpha-1(XII) chain	
	(3)	Q99715-4, COL12A1, Collagen alpha-1(XII) chain	
			1/35/1
270	(1)	Q05707-2, COL14A1, Collagen alpha-1(XIV) chain	
	(2)	Q05707-1, COL14A1, Collagen alpha-1(XIV) chain	
	(3)	Q05707-3, COL14A1, Collagen alpha-1(XIV) chain	
271		P39059, COL15A1, Collagen alpha-1(XV) chain [Cleaved into: Restin	3/0/0
272		P39060-1-3, COL18A1, Collagen alpha-1(XVIII) chain [Cleaved into: Endostatin]	15/0/0
273		P02452, COL1A1, Collagen alpha-1(I) chain	22/0/1
274		P08123, COL1A2, Collagen alpha-2(I) chain	21/0/0
			3/0/3
275	(1)	P02461-1, COL3A1, Collagen alpha-1(III) chain	
	(2)	P02461-2, COL3A1, Collagen alpha-1(III) chain	
276		P20908, COL5A1, Collagen alpha-1(V) chain	12/0/0
277		P05997, COL5A2, Collagen alpha-2(V) chain	11/0/2
278		P12109, COL6A1, Collagen alpha-1(VI) chain	25/0/0
			7/0/14
279	(1)	P12110-1, COL6A2, Collagen alpha-2(VI) chain	
	(2)	P12110-3, COL6A2, Collagen alpha-2(VI) chain	
280		P12110-2, COL6A2, Collagen alpha-2(VI) chain	1/0/14
			4/11/92
281	(1)	P12111-1, COL6A3, Collagen alpha-3(VI) chain	
	(2)	P12111-2, COL6A3, Collagen alpha-3(VI) chain	
	(3)	P12111-3, COL6A3, Collagen alpha-3(VI) chain	
	(4)	P12111-5, COL6A3, Collagen alpha-3(VI) chain	
282		P12111-4, COL6A3, Collagen alpha-3(VI) chain	2/0/92
283		Q9Y6Z7, COLEC10, Collectin-10	2/0/0
284		Q9BWP8-1-10, COLEC11, Collectin-11	2/0/0
			2/0/2
285	(1)	P49747-1, COMP, Cartilage oligomeric matrix protein	
	(2)	P49747-2, COMP, Cartilage oligomeric matrix protein	
286		P53621-1,2, COPA, Coatomer subunit alpha	3/0/0

287		P53618, COPB1, Coatomer subunit beta	7/0/0
288		P35606-1,2, COPB2, Coatomer subunit beta'	9/0/0
289	(1)	O14579-1,3, COPE, Coatomer subunit epsilon	1/0/0
	(2)	O14579-2, COPE, Coatomer subunit epsilon	
290		Q9Y678, COPG1, Coatomer subunit gamma-1	6/0/0
291	(1)	P31146, CORO1A, Coronin-1A	5/0/1
	(2)	Q6QEF8-1-5, CORO6, Coronin-6	
292		Q9ULV4-1-3, CORO1C, Coronin-1C	4/0/1
293		P00450, CP, Ceruloplasmin	39/0/0
294		Q9UI42-1,2, CPA4, Carboxypeptidase A4	3/0/0
295	(1)	Q96IY4-1, CPB2, Carboxypeptidase B2	4/0/0
	(2)	Q96IY4-2, CPB2, Carboxypeptidase B2	
296		P16870-1,2, CPE, Carboxypeptidase E	23/0/0
297		P15169, CPN1, Carboxypeptidase N catalytic chain	11/0/0
298		P22792, CPN2, Carboxypeptidase N subunit 2	13/0/0
299		Q9Y646, CPQ, Carboxypeptidase Q	5/0/0
300		Q96SM3, CPXM1, Probable carboxypeptidase X1	4/0/0
301		Q8N436, CPXM2, Inactive carboxypeptidase-like protein X2	7/0/1
302		Q66K79-1-3, CPZ, Carboxypeptidase Z	2/0/0
303		P29373, CRABP2, Cellular retinoic acid-binding protein 2	6/0/0
304		P24387, CRHBP, Corticotropin-releasing factor-binding protein	2/0/0
305		Q9H0B8-1-3, CRISPLD2, Cysteine-rich secretory protein LCCL domain-containing 2	2/0/0
306	(1)	P02741-1, CRP, C-reactive protein [Cleaved into: C-reactive protein(1-205)]	3/0/0
	(2)	P02741-2, CRP, C-reactive protein [Cleaved into: C-reactive protein(1-205)]	
307		Q9NQ79-1-3, CRTAC1, Cartilage acidic protein 1	1/0/0
308		P02511, CRYAB, Alpha-crystallin B chain	3/0/0
309		O75534-1-4, CSDE1, Cold shock domain-containing protein E1	1/0/0
310		P55060-1,3,4, CSE1L, Exportin-2	1/0/0
311		Q6UVK1, CSPG4, Chondroitin sulfate proteoglycan 4	6/0/0
312		P01034, CST3, Cystatin-C	7/0/0

313		Q15828, CST6, Cystatin-M	8/0/0
314		P04080, CSTB, Cystatin-B	3/0/0
315		P29279-1,2, CTGF, Connective tissue growth factor	6/0/0
316	(1)	P35221-1,2, CTNNA1, Catenin alpha-1	1/1/0
	(2)	P26232-1-3,5, CTNNA2, Catenin alpha-2	
	(3)	P35221-3, CTNNA1, Catenin alpha-1	
317		P35222-1, CTNNB1, Catenin beta-1	5/0/4
318		O60716-1-32, CTNND1, Catenin delta-1	1/0/0
319		P07858, CTSB, Cathepsin B	4/0/0
320		P53634-1, CTSC, Dipeptidyl peptidase 1	2/0/0
321		P07339, CTSD, Cathepsin D	6/0/0
322		Q9UBX1, CTSF, Cathepsin F	4/0/0
323		O60911, CTSV, Cathepsin L2	2/0/0
324	(1)	Q14247-1,3, CTTN, Src substrate cortactin	1/0/0
	(2)	Q14247-2, CTTN, Src substrate cortactin	
325		Q13618-1-3, CUL3, Cullin-3	5/0/0
326		P48061-1-6, CXCL12, Stromal cell-derived factor 1	4/0/0
327		P00167-1-3, CYB5A, Cytochrome b5	1/0/0
328		Q7L576-1, CYFIP1, Cytoplasmic FMR1-interacting protein 1	5/0/0
329		Q9NRR1, CYTL1, Cytokine-like protein 1	5/0/0
330		Q14118, DAG1, Dystroglycan	8/0/0
331		P14868-1,2, DARS, Aspartate--tRNA ligase, cytoplasmic	4/0/0
332	(1)	P81605-1, DCD, Dermcidin	2/0/0
	(2)	P81605-2, DCD, Dermcidin	
333		Q96JQ0, DCHS1, Protocadherin-16	4/0/0
334	(1)	P07585-1, DCN, Decorin	2/1/1
	(2)	P07585-4, DCN, Decorin	
	(3)	P07585-2,3, DCN, Decorin	
335	(1)	Q14203-1,3,4,6, DCTN1, Dynactin subunit 1	0/0/1
	(2)	Q14203-2,5, DCTN1, Dynactin subunit 1	
336		Q92499-1,3, DDX1, ATP-dependent RNA helicase DDX1	3/0/1
337		Q13838-1,2, DDX39B, Spliceosome RNA helicase DDX39B	5/0/0

338	(1)	O00571-1,2, DDX3X, ATP-dependent RNA helicase DDX3X	1/0/0
	(2)	O15523-1,2, DDX3Y, ATP-dependent RNA helicase DDX3Y	
339	(1)	P17844-1,2, DDX5, Probable ATP-dependent RNA helicase DDX5	2/0/0
	(2)	Q92841-1-4, DDX17, Probable ATP-dependent RNA helicase DDX17	
340		P59665, DEFA1, Neutrophil defensin 1	2/0/1
341		P59666, DEFA3, Neutrophil defensin 3	3/0/1
342		P12838, DEFA4, Neutrophil defensin 4	2/0/0
343		Q01523, DEFA5, Defensin-5	1/0/0
344		Q01524, DEFA6, Defensin-6	2/0/0
345		P17661, DES, Desmin	8/0/7
			4/1/0
346	(1)	O60443-1, DFNA5, Non-syndromic hearing impairment protein 5	
	(2)	O60443-2, DFNA5, Non-syndromic hearing impairment protein 5	
	(3)	O60443-3, DFNA5, Non-syndromic hearing impairment protein 5	
347		Q08211-1, DHX9, ATP-dependent RNA helicase A	4/0/0
348		Q9UBP4, DKK3, Dickkopf-related protein 3	8/0/0
349		P09622-1-3, DLD, Dihydrolipoyl dehydrogenase, mitochondrial	2/0/0
			1/0/0
350	(1)	P36957-1, DLST, Dihydrolipoyllysine-residue succinyltransferase component of 2-oxoglutarate dehydrogenase complex, mitochondrial	
	(2)	P36957-2, DLST, Dihydrolipoyllysine-residue succinyltransferase component of 2-oxoglutarate dehydrogenase complex, mitochondrial	
351		Q6E0U4-1-7,16, DMKN, Dermokine	6/0/0
352		Q13316-1,2, DMP1, Dentin matrix acidic phosphoprotein 1	3/0/0
			3/0/0
353	(1)	Q9P2D7-1,2,4, DNAH1, Dynein heavy chain 1, axonemal	
	(2)	Q9P2D7-5, DNAH1, Dynein heavy chain 1, axonemal	
354		Q8WXX0-1, DNAH7, Dynein heavy chain 7, axonemal	7/0/0
355		P25685-1, DNAJB1, DnaJ homolog subfamily B member 1	2/0/0
356		Q13217, DNAJC3, DnaJ homolog subfamily C member 3	11/0/1
357	(1)	P50570-1-5, DNM2, Dynamin-2	3/2/0
	(2)	Q9UQ16-1-5, DNM3, Dynamin-3	
	(3)	Q05193-1-3,5, DNM1, Dynamin-1	

358		O43598-1,2, DNPH1, 2'-deoxynucleoside 5'-phosphate N-hydrolase 1	2/0/0
359		Q96BY6-1-3, DOCK10, Dedicator of cytokinesis protein 10	1/0/2
360		Q9Y3R5-1,2, DOPEY2, Protein dopey-2	1/0/1
361		P27487, DPP4, Dipeptidyl peptidase 4	2/0/0
362		Q07507, DPT, Dermatopontin	9/0/0
363		Q16555-1,2, DPYSL2, Dihydropyrimidinase-related protein 2	5/0/2
364	(1)	Q14195-2, DPYSL3, Dihydropyrimidinase-related protein 3	1/0/2
	(2)	Q14195-1, DPYSL3, Dihydropyrimidinase-related protein 3	
365	(1)	Q08554-1, DSC1, Desmocollin-1	2/0/0
	(2)	Q08554-2, DSC1, Desmocollin-1	
366	(1)	Q14574-1,2, DSC3, Desmocollin-3	9/0/0
	(2)	Q02487-1,2, DSC2, Desmocollin-2	
367	(1)	Q02413-1, DSG1, Desmoglein-1	12/0/3
	(2)	Q02413-2, DSG1, Desmoglein-1	
368		P32926, DSG3, Desmoglein-3	1/0/2
369		Q86SJ6-1,2, DSG4, Desmoglein-4	1/0/3
370	(1)	P15924-1, DSP, Desmoplakin	27/95/2
	(2)	P15924-2, DSP, Desmoplakin	
	(3)	P15924-3, DSP, Desmoplakin	
371	(1)	P60981-1, DSTN, Destrin	1/0/1
	(2)	P60981-2, DSTN, Destrin	
372		Q14204, DYNC1H1, Cytoplasmic dynein 1 heavy chain 1	20/0/0
373		Q13409-1-3,5-7, DYNC1I2, Cytoplasmic dynein 1 intermediate chain 2	2/0/0
374		O43237-1, DYNC1LI2, Cytoplasmic dynein 1 light intermediate chain 2	4/0/0
375		Q8NCM8-1,2, DYNC2H1, Cytoplasmic dynein 2 heavy chain 1	5/0/0
376		Q13011, ECH1, Delta(3,5)-Delta(2,4)-dienoyl-CoA isomerase, mitochondrial	3/0/0
377		P30084, ECHS1, Enoyl-CoA hydratase, mitochondrial	2/0/0
378	(1)	Q16610-1,4, ECM1, Extracellular matrix protein 1	9/6/0
	(2)	Q16610-2, ECM1, Extracellular matrix protein 1	
	(3)	Q16610-3, ECM1, Extracellular matrix protein 1	

379		O94769-1, ECM2, Extracellular matrix protein 2	1/0/16
380		O94769-2, ECM2, Extracellular matrix protein 2	1/0/16
381	(1)	P68104-1, EEF1A1, Elongation factor 1-alpha 1	1/6/0
	·	Q5VTE0, EEF1A1P5, Putative elongation factor 1-alpha-like 3	
	(2)	P68104-2, EEF1A1, Elongation factor 1-alpha 1	
	(3)	Q05639, EEF1A2, Elongation factor 1-alpha 2	
382		P24534, EEF1B2, Elongation factor 1-beta	3/0/0
383		P29692-1-4, EEF1D, Elongation factor 1-delta	3/0/0
384		P26641-1,2, EEF1G, Elongation factor 1-gamma	14/0/0
385		P13639, EEF2, Elongation factor 2	23/0/0
386		Q12805-1-4, EFEMP1, EGF-containing fibulin-like extracellular matrix protein 1	4/0/22
387		Q12805-5, EFEMP1, EGF-containing fibulin-like extracellular matrix protein 1	2/0/22
388		O95967, EFEMP2, EGF-containing fibulin-like extracellular matrix protein 2	16/0/0
389	(1)	Q9H4M9, EHD1, EH domain-containing protein 1	5/0/1
	(2)	Q9NZN3-1, EHD3, EH domain-containing protein 3	
	(3)	Q9NZN3-2, EHD3, EH domain-containing protein 3	
390	(1)	Q9NZN4-1, EHD2, EH domain-containing protein 2	2/0/1
	(2)	Q9NZN4-2, EHD2, EH domain-containing protein 2	
391		P05198, EIF2S1, Eukaryotic translation initiation factor 2 subunit 1	3/0/0
392		Q14152-1,2, EIF3A, Eukaryotic translation initiation factor 3 subunit A	2/0/1
393		P55884-1,2, EIF3B, Eukaryotic translation initiation factor 3 subunit B	2/0/0
394		P60228, EIF3E, Eukaryotic translation initiation factor 3 subunit E	3/0/0
395		O15372, EIF3H, Eukaryotic translation initiation factor 3 subunit H	3/0/0
396		Q13347, EIF3I, Eukaryotic translation initiation factor 3 subunit I	2/0/0
397	(1)	P60842-1,2, EIF4A1, Eukaryotic initiation factor 4A-I	5/0/2
	(2)	Q14240-1,2, EIF4A2, Eukaryotic initiation factor 4A-II	
398		P38919, EIF4A3, Eukaryotic initiation factor 4A-III	1/0/2
399	(1)	Q04637-1,3,8,9, EIF4G1, Eukaryotic translation initiation factor 4 gamma 1	1/0/0
	(2)	Q04637-4-7, EIF4G1, Eukaryotic translation initiation factor 4 gamma 1	

400		Q15056-1,2, EIF4H, Eukaryotic translation initiation factor 4H	2/0/0
401		P63241-1,2, EIF5A, Eukaryotic translation initiation factor 5A-1	2/0/0
402		Q9Y6C2-1, EMILIN1, EMILIN-1	9/0/0
403	(1)	P06733-1, ENO1, Alpha-enolase	5/1/2
	(2)	P13929-1-3, ENO3, Beta-enolase	
	(3)	P06733-2, ENO1, Alpha-enolase	
404		P09104-1,2, ENO2, Gamma-enolase	1/0/2
405		P07099, EPHX1, Epoxide hydrolase 1	2/0/0
406		P58107, EPPK1, Epiplakin	8/0/4
407		P84090, ERH, Enhancer of rudimentary homolog	1/0/0
408		O94905-1, ERLIN2, Erlin-2	3/0/0
409		P30040-1, ERP29, Endoplasmic reticulum resident protein 29	1/0/0
410		Q9BS26, ERP44, Endoplasmic reticulum resident protein 44	6/0/0
411		P62495-1,2, ETF1, Eukaryotic peptide chain release factor subunit 1	1/0/0
412		Q92817, EVPL, Envoplakin	5/0/0
413		Q01844-1-3,5,6, EWSR1, RNA-binding protein EWS	3/0/0
414	(1)	P15311, EZR, Ezrin	12/0/5
	(2)	P35241-4, RDX, Radixin	
415		P00742, F10, Coagulation factor X	15/0/1
416	(1)	P03951-1, F11, Coagulation factor XI	4/0/0
	(2)	P03951-2, F11, Coagulation factor XI	
417		P00748, F12, Coagulation factor XII	21/0/0
418		P00488, F13A1, Coagulation factor XIII A chain	26/0/0
419		P05160, F13B, Coagulation factor XIII B chain	21/0/0
420		P00734, F2, Prothrombin	39/0/0
421		P12259, F5, Coagulation factor V	38/0/0
422		P08709-1,2, F7, Coagulation factor VII	10/0/0
423		P00451-1, F8, Coagulation factor VIII	10/0/0
424	(1)	P00740-1, F9, Coagulation factor IX	2/0/1
	(2)	P00740-2, F9, Coagulation factor IX	
425		P05413, FABP3, Fatty acid-binding protein, heart	3/0/0
426	(1)	Q01469, FABP5, Fatty acid-binding protein, epidermal	3/0/0

	(2)	A8MUU1, FABP5P3, Putative fatty acid-binding protein 5-like protein 3	
427		Q0Z7S8, FABP9, Fatty acid-binding protein 9	5/0/0
428		Q8IWE2-1,2, FAM114A1, Protein NOXP20	2/0/0
429	(1)	Q96TA1-2, FAM129B, Niban-like protein 1	1/0/0
	(2)	Q96TA1-1, FAM129B, Niban-like protein 1	
430	(1)	Q9NUQ9-1, FAM49B, Protein FAM49B	2/0/0
	(2)	Q9NUQ9-2, FAM49B, Protein FAM49B	
431	(1)	Q9NSD9-1, FARSB, Phenylalanine--tRNA ligase beta subunit	1/0/0
	(2)	Q9NSD9-2, FARSB, Phenylalanine--tRNA ligase beta subunit	
432	(1)	P23142-1, FBLN1, Fibulin-1	8/0/21
	(2)	P23142-2, FBLN1, Fibulin-1	
433		P23142-3, FBLN1, Fibulin-1	1/0/21
434		P23142-4, FBLN1, Fibulin-1	7/0/21
435		P98095-1,2, FBLN2, Fibulin-2	29/0/0
436		Q9UBX5, FBLN5, Fibulin-5	15/0/0
437		Q53RD9-1-4, FBLN7, Fibulin-7	4/0/0
438		P35555, FBN1, Fibrillin-1 [Cleaved into: Asprosin]	78/0/5
439	(1)	P35556-1, FBN2, Fibrillin-2 [Cleaved into: Fibrillin-2 C-terminal peptide]	9/0/6
	(2)	P35556-2, FBN2, Fibrillin-2 [Cleaved into: Fibrillin-2 C-terminal peptide]	
440		P09467, FBP1, Fructose-1,6-bisphosphatase 1	2/0/0
441		Q9UK22, FBXO2, F-box only protein 2	2/0/0
442		Q9Y6R7, FCGBP, IgGFc-binding protein	26/0/0
443		P55899, FCGRT, IgG receptor FcRn large subunit p51	2/0/0
444		O00602, FCN1, Ficolin-1	1/0/1
445	(1)	Q15485-1, FCN2, Ficolin-2	1/0/1
	(2)	Q15485-2, FCN2, Ficolin-2	
446		O75636-1, FCN3, Ficolin-3	2/0/10
447		O75636-2, FCN3, Ficolin-3	0/0/11
448	(1)	A9Z1Z3-1, FER1L4, Fer-1-like protein 4	1/0/0
	(2)	A9Z1Z3-3, FER1L4, Fer-1-like protein 4	

449		Q86UX7-1,2, FERMT3, Fermitin family homolog 3	3/0/0
450	(1)	Q9UGM5-1, FETUB, Fetuin-B	3/0/0
	(2)	Q9UGM5-2, FETUB, Fetuin-B	
451	(1)	P02671-1, FGA, Fibrinogen alpha chain [Cleaved into: Fibrinopeptide A; Fibrinogen alpha chain]	5/0/0
	(2)	P02671-2, FGA, Fibrinogen alpha chain [Cleaved into: Fibrinopeptide A; Fibrinogen alpha chain]	
452		P02675, FGB, Fibrinogen beta chain [Cleaved into: Fibrinopeptide B; Fibrinogen beta chain]	48/0/0
453		Q6ZV73-1,2, FGD6, FYVE, RhoGEF and PH domain-containing protein 6	4/0/0
454		P02679-1,2, FGG, Fibrinogen gamma chain	37/0/0
455	(1)	P07954-1, FH, Fumarate hydratase, mitochondrial	1/0/0
	(2)	P07954-2, FH, Fumarate hydratase, mitochondrial	
456		Q14192-1, FHL2, Four and a half LIM domains protein 2	11/0/0
457		P62942, FKBP1A, Peptidyl-prolyl cis-trans isomerase FKBP1A	1/0/0
458	(1)	O95302-3, FKBP9, Peptidyl-prolyl cis-trans isomerase FKBP9	1/0/0
	(2)	O95302-1,2, FKBP9, Peptidyl-prolyl cis-trans isomerase FKBP9	
459		P20930, FLG, Filaggrin	8/0/0
460		Q5D862, FLG2, Filaggrin-2	6/0/0
461		Q13045-1-3, FLII, Protein flightless-1 homolog	2/0/0
462		P21333-1,2, FLNA, Filamin-A	46/0/9
463	(1)	O75369-1,2,8,9, FLNB, Filamin-B	0/21/7
	(2)	O75369-7, FLNB, Filamin-B	
	(3)	O75369-3,6, FLNB, Filamin-B	
	(4)	O75369-4,5, FLNB, Filamin-B	
464		Q14315-1,2, FLNC, Filamin-C	33/0/8
465		Q06828, FMOD, Fibromodulin	9/0/0

0/95/0

466 (1) P02751-1,3, FN1, Fibronectin
(2) P02751-8,14, FN1, Fibronectin
(3) P02751-10, FN1, Fibronectin
(4) P02751-11, FN1, Fibronectin
(5) P02751-12, FN1, Fibronectin
(6) P02751-13, FN1, Fibronectin
(7) P02751-16, FN1, Fibronectin
(8) P02751-17, FN1, Fibronectin
(9) P02751-2, FN1, Fibronectin
(10) P02751-4, FN1, Fibronectin
(11) P02751-5, FN1, Fibronectin
(12) P02751-6, FN1, Fibronectin
(13) P02751-9, FN1, Fibronectin
(14) P02751-7,15, FN1, Fibronectin

467 Q4ZHG4-1,2, FNDC1, Fibronectin type III domain-containing protein 1 — 4/0/0

468 (1) Q70Z53-3, FRA10AC1, Protein FRA10AC1 — 1/0/0
(2) Q70Z53-1,2,4,5, FRA10AC1, Protein FRA10AC1

469 Q92765, FRZB, Secreted frizzled-related protein 3 — 1/0/0

470 Q16658, FSCN1, Fascin — 2/0/0

471 P19883-1,2, FST, Follistatin — 8/0/0

472 (1) Q12841-1, FSTL1, Follistatin-related protein 1 — 2/0/0
(2) Q12841-2, FSTL1, Follistatin-related protein 1

473 P02792, FTL, Ferritin light chain — 2/0/0

474 P35637-1,2, FUS, RNA-binding protein FUS — 2/0/0

475 Q13283-1, G3BP1, Ras GTPase-activating protein-binding protein 1 — 2/0/0

476 P51570-1,2, GALK1, Galactokinase — 2/0/0

477 Q10471-1,2, GALNT2, Polypeptide N-acetylgalactosaminyltransferase 2 — 1/0/0

478 (1) Q14697-1, GANAB, Neutral alpha-glucosidase AB — 2/0/15
(2) Q14697-3, GANAB, Neutral alpha-glucosidase AB

479		Q14697-2, GANAB, Neutral alpha-glucosidase AB	1/0/15
480	(1)	P04406-1, GAPDH, Glyceraldehyde-3-phosphate dehydrogenase	2/0/0
	(2)	P04406-2, GAPDH, Glyceraldehyde-3-phosphate dehydrogenase	
481		P54826, GAS1, Growth arrest-specific protein 1	9/0/0
482	(1)	Q14393-1,2, GAS6, Growth arrest-specific protein 6	2/6/0
	(2)	Q14393-4,5, GAS6, Growth arrest-specific protein 6	
	(3)	Q14393-3, GAS6, Growth arrest-specific protein 6	
483	(1)	P02774-1,3, GC, Vitamin D-binding protein	9/0/0
	(2)	P02774-2, GC, Vitamin D-binding protein	
484		Q99988, GDF15, Growth/differentiation factor 15	3/0/0
485		P14136-1-3, GFAP, Glial fibrillary acidic protein	0/0/3
486	(1)	Q9UBU3-1-4, GHRL, Appetite-regulating hormone	1/0/0
	(2)	Q9UBU3-5, GHRL, Appetite-regulating hormone	
487		P09681, GIP, Gastric inhibitory polypeptide	2/0/0
488		O14908-1,2, GIPC1, PDZ domain-containing protein GIPC1	1/0/0
489		P17302, GJA1, Gap junction alpha-1 protein	2/0/0
490		Q9NS71, GKN1, Gastrokine-1	6/0/0
491		Q86XP6, GKN2, Gastrokine-2	1/0/0
492		P16278-1-3, GLB1, Beta-galactosidase	1/0/0
493		Q92896-1-3, GLG1, Golgi apparatus protein 1	6/0/0
494		O94925-1,3, GLS, Glutaminase kidney isoform, mitochondrial	2/0/0
495	(1)	P00367-1, GLUD1, Glutamate dehydrogenase 1, mitochondrial	1/0/0
	(2)	P00367-2,3, GLUD1, Glutamate dehydrogenase 1, mitochondrial	
496		P15104, GLUL, Glutamine synthetase	4/0/0
497		P17900, GM2A, Ganglioside GM2 activator	5/0/0
498		O60234, GMFG, Glia maturation factor gamma	2/0/0
499		P04899-1-6, GNAI2, Guanine nucleotide-binding protein G(i) subunit alpha-2	3/0/0
500	(1)	P62873-1,2, GNB1, Guanine nucleotide-binding protein G(I)/G(S)/G(T) subunit beta-1	0/0/2

	(2)	P16520-1,2, GNB3, Guanine nucleotide-binding protein G(I)/G(S)/G(T) subunit beta-3	
501	(1)	P62879-1, GNB2, Guanine nucleotide-binding protein G(I)/G(S)/G(T) subunit beta-2	0/0/3
	·	Q9HAV0, GNB4, Guanine nucleotide-binding protein subunit beta-4	
	(2)	P62879-2, GNB2, Guanine nucleotide-binding protein G(I)/G(S)/G(T) subunit beta-2	
502		Q9UJJ9, GNPTG, N-acetylglucosamine-1-phosphotransferase subunit gamma	3/0/0
503		H0YM25, GOLGA6L22, Golgin subfamily A member 6-like protein 22	11/0/2
504		O00461, GOLIM4, Golgi integral membrane protein 4	11/0/0
505	(1)	Q8NBJ4-1, GOLM1, Golgi membrane protein 1	1/0/0
	(2)	Q8NBJ4-2, GOLM1, Golgi membrane protein 1	
506		P00505-1, GOT2, Aspartate aminotransferase, mitochondrial	1/0/0
507	(1)	Q9UKJ3-1, GPATCH8, G patch domain-containing protein 8	1/0/0
	(2)	Q9UKJ3-2, GPATCH8, G patch domain-containing protein 8	
508		P80108-1, GPLD1, Phosphatidylinositol-glycan-specific phospholipase D	15/0/0
509		Q13098-4-7, GPS1, COP9 signalosome complex subunit 1	2/0/0
510		P22352, GPX3, Glutathione peroxidase 3	9/0/0
511		Q9ULK0-1, GRID1, Glutamate receptor ionotropic, delta-1	0/0/2
512	(1)	P28799-1, GRN, Granulins	0/4/0
	(2)	P28799-2, GRN, Granulins	
	(3)	P28799-3, GRN, Granulins	
513		Q96QA5, GSDMA, Gasdermin-A	5/0/0
514	(1)	P06396-1, GSN, Gelsolin	2/0/42
	(2)	P06396-3,4, GSN, Gelsolin	
515		P06396-2, GSN, Gelsolin	1/0/42
516		P21266, GSTM3, Glutathione S-transferase Mu 3	3/0/0
517		P78417-1,3, GSTO1, Glutathione S-transferase omega-1	2/0/0
518		P09211, GSTP1, Glutathione S-transferase P	3/0/0
519		Q02747, GUCA2A, Guanylin	3/0/0
520		P07305-1,2, H1F0, Histone H1.0	2/0/0
521		Q92522, H1FX, Histone H1x	2/0/0

522	(1)	P0C0S5, H2AFZ, Histone H2A.Z	0/0/2
	·	Q71UI9-1,2, H2AFV, Histone H2A.V	
	(2)	Q71UI9-3,4, H2AFV, Histone H2A.V	
523		P16104, H2AFX, Histone H2AX	3/0/3
524		P84243, H3F3A, Histone H3.3	1/0/7
525		Q14520-1,2, HABP2, Hyaluronan-binding protein 2	21/0/0
526		Q6Y1H2, HACD2, Very-long-chain	1/0/0
527	(1)	P40939-1, HADHA, Trifunctional enzyme subunit alpha, mitochondrial	6/0/0
	(2)	P40939-2, HADHA, Trifunctional enzyme subunit alpha, mitochondrial	
528	(1)	P55084-1, HADHB, Trifunctional enzyme subunit beta, mitochondrial	1/0/0
	(2)	P55084-2, HADHB, Trifunctional enzyme subunit beta, mitochondrial	
529		P69905, HBA1;, Hemoglobin subunit alpha	3/0/0
530	(1)	P68871, HBB, Hemoglobin subunit beta	2/0/0
	(2)	P02042, HBD, Hemoglobin subunit delta	
531		P51858-1-3, HDGF, Hepatoma-derived growth factor	5/0/0
532		Q00341-1,2, HDLBP, Vigilin	2/0/0
533		O95714, HERC2, E3 ubiquitin-protein ligase HERC2	7/0/0
534		P06865-1, HEXA, Beta-hexosaminidase subunit alpha	4/0/0
535		P07686, HEXB, Beta-hexosaminidase subunit beta	2/0/0
536		Q02539, HIST1H1A, Histone H1.1	1/0/4
537		P16401, HIST1H1B, Histone H1.5	4/0/6
538	(1)	P16403, HIST1H1C, Histone H1.2	6/0/11
	(2)	P22492, HIST1H1T, Histone H1t	
539		P16402, HIST1H1D, Histone H1.3	2/0/9
540		P10412, HIST1H1E, Histone H1.4	3/0/12
541		P04908, HIST1H2AB, Histone H2A type 1-B/E	1/0/4
		P20671, HIST1H2AD, Histone H2A type 1-D	
		Q7L7L0, HIST3H2A, Histone H2A type 3	
542	(1)	P06899, HIST1H2BJ, Histone H2B type 1-J	0/0/11
	·	P23527, HIST1H2BO, Histone H2B type 1-O	
	·	P33778, HIST1H2BB, Histone H2B type 1-B	
	·	Q16778, HIST2H2BE, Histone H2B type 2-E	
	(2)	Q6DN03, HIST2H2BC, Putative histone H2B type 2-C	
	·	Q6DRA6, HIST2H2BD, Putative histone H2B type 2-D	
543	(1)	O60814, HIST1H2BK, Histone H2B type 1-K	0/0/12

· P58876, HIST1H2BD, Histone H2B type 1-D
· P62807, HIST1H2BC, Histone H2B type 1-C/E/F/G/I
· Q5QNW6-1,2, HIST2H2BF, Histone H2B type 2-F
· Q93079, HIST1H2BH, Histone H2B type 1-H
· Q99877, HIST1H2BN, Histone H2B type 1-N
(2) Q99879, HIST1H2BM, Histone H2B type 1-M
(3) P57053, H2BFS, Histone H2B type F-S

544 (1) Q99880, HIST1H2BL, Histone H2B type 1-L — 0/0/12 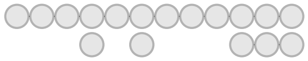
(2) Q96A08, HIST1H2BA, Histone H2B type 1-A

545 (1) P68431, HIST1H3A, Histone H3.1
· Q16695, HIST3H3, Histone H3.1t — 1/0/7 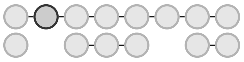
· Q71DI3, HIST2H3A;, Histone H3.2
(2) Q6NXT2, H3F3C, Histone H3.3C

546 P62805, HIST1H4A, Histone H4 — 6/0/0 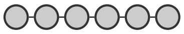

547 (1) Q16777, HIST2H2AC, Histone H2A type 2-C
(2) Q8IUE6, HIST2H2AB, Histone H2A type 2-B
(3) Q96QV6, HIST1H2AA, Histone H2A type 1-A
· Q9BTM1-2, H2AFJ, Histone H2A.J — 2/1/4 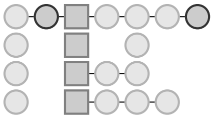
(4) P0C0S8, HIST1H2AG, Histone H2A type 1
· Q6FI13, HIST2H2AA3, Histone H2A type 2-A
· Q93077, HIST1H2AC, Histone H2A type 1-C
· Q96KK5, HIST1H2AH, Histone H2A type 1-H
· Q99878, HIST1H2AJ, Histone H2A type 1-J
· Q9BTM1-1, H2AFJ, Histone H2A.J

548 Q8N257, HIST3H2BB, Histone H2B type 3-B — 0/0/10 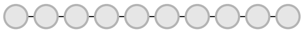

549 P01891, HLA-A, HLA class I histocompatibility antigen, A-68 alpha chain — 0/0/5 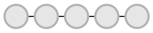

550 (1) P04439, HLA-A, HLA class I histocompatibility antigen, A-3 alpha chain — 0/0/5 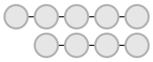
· P13746-1,2, HLA-A, HLA class I histocompatibility antigen, A-11 alpha chain
(2) P30443, HLA-A, HLA class I histocompatibility antigen, A-1 alpha chain
· P30455, HLA-A, HLA class I histocompatibility antigen, A-36 alpha chain

551 (1) P10316, HLA-A, HLA class I histocompatibility antigen, A-69 alpha chain — 0/0/5 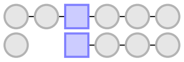
(2) P01892, HLA-A, HLA class I histocompatibility antigen, A-2 alpha chain

552 (1) P16188, HLA-A, HLA class I histocompatibility antigen, A-30 alpha chain — 0/0/6 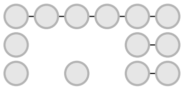
(2) P05534, HLA-A, HLA class I histocompatibility antigen, A-24 alpha chain
(3) P30447, HLA-A, HLA class I histocompatibility antigen, A-23 alpha chain

553 (1) P16190, HLA-A, HLA class I histocompatibility antigen, A-33 alpha chain
(2) P30453, HLA-A, HLA class I histocompatibility antigen, A-34 alpha chain — 0/0/6 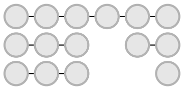

(3) P18462, HLA-A, HLA class I histocompatibility antigen, A-25 alpha chain

· P30450, HLA-A, HLA class I histocompatibility antigen, A-26 alpha chain

· P30457, HLA-A, HLA class I histocompatibility antigen, A-66 alpha chain

554 (1) P30512, HLA-A, HLA class I histocompatibility antigen, A-29 alpha chain

0/0/6

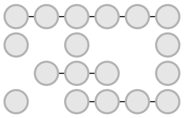

(2) P30456, HLA-A, HLA class I histocompatibility antigen, A-43 alpha chain

(3) Q09160, HLA-A, HLA class I histocompatibility antigen, A-80 alpha chain

(4) P10314, HLA-A, HLA class I histocompatibility antigen, A-32 alpha chain

· P16189, HLA-A, HLA class I histocompatibility antigen, A-31 alpha chain

· P30459, HLA-A, HLA class I histocompatibility antigen, A-74 alpha chain

555 P01889, HLA-B, HLA class I histocompatibility antigen, B-7 alpha chain

0/0/8

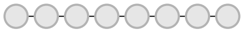

556 (1) P03989, HLA-B, HLA class I histocompatibility antigen, B-27 alpha chain

(2) P30461, HLA-B, HLA class I histocompatibility antigen, B-13 alpha chain

(3) P30487, HLA-B, HLA class I histocompatibility antigen, B-49 alpha chain

· P30488, HLA-B, HLA class I histocompatibility antigen, B-50 alpha chain

1/0/6

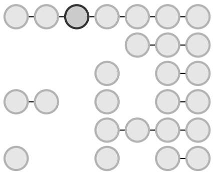

(4) P30464, HLA-B, HLA class I histocompatibility antigen, B-15 alpha chain

· P30475, HLA-B, HLA class I histocompatibility antigen, B-39 alpha chain

· P30484, HLA-B, HLA class I histocompatibility antigen, B-46 alpha chain

· P30491, HLA-B, HLA class I histocompatibility antigen, B-53 alpha chain

· P30495, HLA-B, HLA class I histocompatibility antigen, B-56 alpha chain

· P30685, HLA-B, HLA class I histocompatibility antigen, B-35 alpha chain

· Q29836, HLA-B, HLA class I histocompatibility antigen, B-67 alpha chain

· Q95365, HLA-B, HLA class I histocompatibility antigen, B-38 alpha chain

(5) P30485, HLA-B, HLA class I histocompatibility antigen, B-47 alpha chain

(6) P10319, HLA-B, HLA class I histocompatibility antigen, B-58 alpha chain

· P18465, HLA-B, HLA class I histocompatibility antigen, B-57 alpha chain

557 (1) P30460, HLA-B, HLA class I histocompatibility antigen, B-8 alpha chain

· P30480, HLA-B, HLA class I histocompatibility antigen, B-42 alpha chain

0/0/10

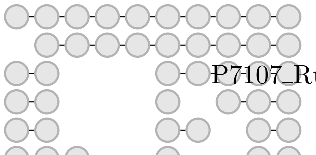

(2) P30479, HLA-B, HLA class I histocompatibility antigen, B-41 alpha chain

(3) P18463, HLA-B, HLA class I histocompatibility antigen, B-37 alpha chain

(4) P30462, HLA-B, HLA class I histocompatibility antigen, B-14 alpha chain

· P30466, HLA-B, HLA class I histocompatibility antigen, B-18 alpha chain

· P30493, HLA-B, HLA class I histocompatibility antigen, B-55 alpha chain

· Q29940, HLA-B, HLA class I histocompatibility antigen, B-59 alpha chain

(5) Q29718, HLA-B, HLA class I histocompatibility antigen, B-82 alpha chain

(6) P18464, HLA-B, HLA class I histocompatibility antigen, B-51 alpha chain

· P30490, HLA-B, HLA class I histocompatibility antigen, B-52 alpha chain

· P30498, HLA-B, HLA class I histocompatibility antigen, B-78 alpha chain

558 (1) P30481, HLA-B, HLA class I histocompatibility antigen, B-44 alpha chain — 1/0/5 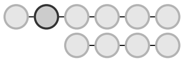

(2) P30483, HLA-B, HLA class I histocompatibility antigen, B-45 alpha chain

559 P30486, HLA-B, HLA class I histocompatibility antigen, B-48 alpha chain — 0/0/6 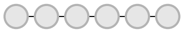

Q31610, HLA-B, HLA class I histocompatibility antigen, B-81 alpha chain

560 P30492, HLA-B, HLA class I histocompatibility antigen, B-54 alpha chain — 0/0/6 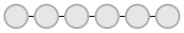

561 Q04826, HLA-B, HLA class I histocompatibility antigen, B-40 alpha chain — 0/0/8 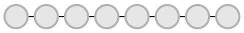

562 Q31612, HLA-B, HLA class I histocompatibility antigen, B-73 alpha chain — 0/0/8 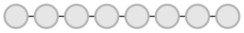

563 P10321, HLA-C, HLA class I histocompatibility antigen, Cw-7 alpha chain — 2/0/9 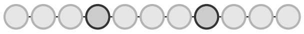

564 P30501, HLA-C, HLA class I histocompatibility antigen, Cw-2 alpha chain — 0/0/8 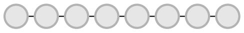

565 (1) P30508, HLA-C, HLA class I histocompatibility antigen, Cw-12 alpha chain — 0/0/10 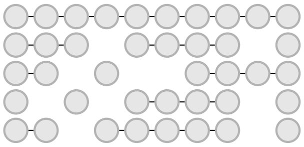

· Q29963, HLA-C, HLA class I histocompatibility antigen, Cw-6 alpha chain

(2) Q29865, HLA-C, HLA class I histocompatibility antigen, Cw-18 alpha chain

(3) Q29960-1,2, HLA-C, HLA class I histocompatibility antigen, Cw-16 alpha chain

(4) P30504, HLA-C, HLA class I histocompatibility antigen, Cw-4 alpha chain

(5) P30510, HLA-C, HLA class I histocompatibility antigen, Cw-14 alpha chain

566 (1) Q07000, HLA-C, HLA class I histocompatibility antigen, Cw-15 alpha chain — 2/0/8 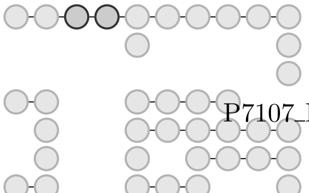

(2) P17693, HLA-G, HLA class I histocompatibility antigen, alpha chain G

(3) P13747, HLA-E, HLA class I histocompatibility antigen, alpha chain E

(4) P30499, HLA-C, HLA class I histocompatibility antigen, Cw-1 alpha chain

(5) P30505, HLA-C, HLA class I histocompatibility antigen, Cw-8 alpha chain

(6) Q9TNN7, HLA-C, HLA class I histocompatibility antigen, Cw-5 alpha chain

(7) P04222, HLA-C, HLA class I histocompatibility antigen, Cw-3 alpha chain

(8) P01893, HLA-H, Putative HLA class I histocompatibility antigen, alpha chain H

567 Q95604, HLA-C, HLA class I histocompatibility antigen, Cw-17 alpha chain — 0/0/7

568 (1) P01903, HLA-DRA, HLA class II histocompatibility antigen, DR alpha chain — 1/0/0

(2) P01906, HLA-DQA2, HLA class II histocompatibility antigen, DQ alpha 2 chain

1/0/0

569 (1) Q96RW7-2, HMCN1, Hemicentin-1

(2) Q96RW7-1, HMCN1, Hemicentin-1

5/9/0

570 (1) Q8NDA2-1, HMCN2, Hemicentin-2

(2) Q8NDA2-2, HMCN2, Hemicentin-2

(3) Q8NDA2-4, HMCN2, Hemicentin-2

571 P17096-1-3, HMGA1, High mobility group protein HMG-I/HMG-Y — 1/0/0

572 P05204, HMGN2, Non-histone chromosomal protein HMG-17 — 2/0/0

573 Q13151, HNRNPA0, Heterogeneous nuclear ribonucleoprotein A0 — 2/0/0

574 (1) P09651-1-3, HNRNPA1, Heterogeneous nuclear ribonucleoprotein A1 — 1/0/3

(2) Q32P51, HNRNPA1L2, Heterogeneous nuclear ribonucleoprotein A1-like 2

575 P22626-1,2, HNRNPA2B1, Heterogeneous nuclear ribonucleoproteins A2/B1 — 6/0/1

2/0/2

576 (1) P51991-1, HNRNPA3, Heterogeneous nuclear ribonucleoprotein A3

(2) P51991-2, HNRNPA3, Heterogeneous nuclear ribonucleoprotein A3

577 Q99729-1-4, HNRNPAB, Heterogeneous nuclear ribonucleoprotein A/B — 1/0/1

578 (1) P07910-1,2,4, HNRNPC, Heterogeneous nuclear ribonucleoproteins C1/C2 — 2/0/1

(2) B7ZW38, HNRNPCL3, Heterogeneous nuclear ribonucleoprotein C-like 3

	·	P0DMR1, HNRNPCL4, Heterogeneous nuclear ribonucleoprotein C-like 4	
579		B2RXH8, HNRNPCL2, Heterogeneous nuclear ribonucleoprotein C-like 2	1/0/1
		O60812, HNRNPCL1, Heterogeneous nuclear ribonucleoprotein C-like 1	
580		Q14103-1,3, HNRNPD, Heterogeneous nuclear ribonucleoprotein D0	1/0/3
581		Q14103-2,4, HNRNPD, Heterogeneous nuclear ribonucleoprotein D0	1/0/2
582		P52597, HNRNPF, Heterogeneous nuclear ribonucleoprotein F	4/0/1
583	(1)	P31943, HNRNPH1, Heterogeneous nuclear ribonucleoprotein H	1/0/1
	(2)	P55795, HNRNPH2, Heterogeneous nuclear ribonucleoprotein H2	
584	(1)	P61978-3, HNRNPK, Heterogeneous nuclear ribonucleoprotein K	1/15/0
	(2)	P61978-1, HNRNPK, Heterogeneous nuclear ribonucleoprotein K	
	(3)	P61978-2, HNRNPK, Heterogeneous nuclear ribonucleoprotein K	
585	(1)	P14866-1, HNRNPL, Heterogeneous nuclear ribonucleoprotein L	1/0/0
	(2)	P14866-2, HNRNPL, Heterogeneous nuclear ribonucleoprotein L	
586		P52272-1,2, HNRNPM, Heterogeneous nuclear ribonucleoprotein M	3/0/0
587	(1)	O43390-1-3, HNRNPR, Heterogeneous nuclear ribonucleoprotein R	1/0/3
	(2)	O43390-4, HNRNPR, Heterogeneous nuclear ribonucleoprotein R	
588	(1)	Q00839-2, HNRNPU, Heterogeneous nuclear ribonucleoprotein U	1/0/0
	(2)	Q00839-1, HNRNPU, Heterogeneous nuclear ribonucleoprotein U	
589		Q1KMD3, HNRNPUL2, Heterogeneous nuclear ribonucleoprotein U-like protein 2	9/0/0
590	(1)	P00738-1, HP, Haptoglobin	1/0/14
	(2)	P00738-2, HP, Haptoglobin	
591		P00739-1,2, HPR, Haptoglobin-related protein	9/0/14
592		P02790, HPX, Hemopexin	11/0/0
593		P23327, HRC, Sarcoplasmic reticulum histidine-rich calcium-binding protein	4/0/0

594		P04196, HRG, Histidine-rich glycoprotein	22/0/0
595		Q86YZ3, HRNR, Hornerin	4/0/0
596	(1)	P07900-1,2, HSP90AA1, Heat shock protein HSP 90-alpha	12/0/14
	(2)	Q12931-1,2, TRAP1, Heat shock protein 75 kDa, mitochondrial	
	(3)	Q58FG0, HSP90AA5P, Putative heat shock protein HSP 90-alpha A5	
	(4)	Q58FG1, HSP90AA4P, Putative heat shock protein HSP 90-alpha A4	
	(5)	Q14568, HSP90AA2P, Heat shock protein HSP 90-alpha A2	
597	(1)	P08238, HSP90AB1, Heat shock protein HSP 90-beta	10/1/14
	(2)	Q58FF7, HSP90AB3P, Putative heat shock protein HSP 90-beta-3	
	(3)	Q58FF8, HSP90AB2P, Putative heat shock protein HSP 90-beta 2	
598		Q58FF6, HSP90AB4P, Putative heat shock protein HSP 90-beta 4	1/0/4
599	(1)	P14625, HSP90B1, Endoplasmin	22/0/0
	(2)	Q58FF3, HSP90B2P, Putative endoplasmin-like protein	
600	(1)	P0DMV8-1, HSPA1A, Heat shock 70 kDa protein 1A	1/0/14
	·	P0DMV9, HSPA1B, Heat shock 70 kDa protein 1B	
	(2)	P0DMV8-2, HSPA1A, Heat shock 70 kDa protein 1A	
	(3)	P48741, HSPA7, Putative heat shock 70 kDa protein 7	
601		P34931, HSPA1L, Heat shock 70 kDa protein 1-like	1/0/12
602		P54652, HSPA2, Heat shock-related 70 kDa protein 2	4/0/9
603		P34932-1, HSPA4, Heat shock 70 kDa protein 4	7/0/0
604		P11021, HSPA5, 78 kDa glucose-regulated protein	31/0/3
605		P17066, HSPA6, Heat shock 70 kDa protein 6	0/0/6
606	(1)	P11142-1, HSPA8, Heat shock cognate 71 kDa protein	5/0/9
	(2)	P11142-2, HSPA8, Heat shock cognate 71 kDa protein	
607		P38646, HSPA9, Stress-70 protein, mitochondrial	7/0/1
608		P04792, HSPB1, Heat shock protein beta-1	9/0/0
609		O14558, HSPB6, Heat shock protein beta-6	2/0/0
610	(1)	P10809-1, HSPD1, 60 kDa heat shock protein, mitochondrial	7/0/0
	(2)	P10809-2, HSPD1, 60 kDa heat shock protein, mitochondrial	
611		P61604, HSPE1, 10 kDa heat shock protein, mitochondrial	2/0/0
612		P98160, HSPG2, Basement membrane-specific heparan sulfate proteoglycan core protein	65/0/0
613		O43719, HTATSF1, HIV Tat-specific factor 1	15/0/0
614		Q92743, HTRA1, Serine protease HTRA1	16/0/1

615	(1)	Q9Y4L1-1, HYOU1, Hypoxia up-regulated protein 1	3/0/0
	(2)	Q9Y4L1-2, HYOU1, Hypoxia up-regulated protein 1	
616		P50213-1,2, IDH3A, Isocitrate dehydrogenase [NAD] subunit alpha, mitochondrial	2/0/0
617		P13284, IFI30, Gamma-interferon-inducible lysosomal thiol reductase	4/0/0
618		Q5T764, IFIT1B, Interferon-induced protein with tetratricopeptide repeats 1B	4/0/0
619	(1)	P05019-1, IGF1, Insulin-like growth factor I	1/3/0
	(2)	P05019-2, IGF1, Insulin-like growth factor I	
	(3)	P05019-3, IGF1, Insulin-like growth factor I	
	(4)	P05019-4, IGF1, Insulin-like growth factor I	
620		P01344-1-3, IGF2, Insulin-like growth factor II	5/0/0
621		P11717, IGF2R, Cation-independent mannose-6-phosphate receptor	4/0/0
622		P35858-1,2, IGFALS, Insulin-like growth factor-binding protein complex acid labile subunit	19/0/0
623		P08833, IGFBP1, Insulin-like growth factor-binding protein 1	10/0/0
624		P18065, IGFBP2, Insulin-like growth factor-binding protein 2	16/0/0
625		P17936-1,2, IGFBP3, Insulin-like growth factor-binding protein 3	16/0/1
626	(1)	P22692-1, IGFBP4, Insulin-like growth factor-binding protein 4	2/0/1
	(2)	P22692-2, IGFBP4, Insulin-like growth factor-binding protein 4	
627		P24593, IGFBP5, Insulin-like growth factor-binding protein 5	12/0/0
628		P24592, IGFBP6, Insulin-like growth factor-binding protein 6	7/0/0
629		Q16270-1,2, IGFBP7, Insulin-like growth factor-binding protein 7	8/0/0
630		P01876, IGHA1, Ig alpha-1 chain C region	5/0/8
631		P01877, IGHA2, Ig alpha-2 chain C region	1/0/8
632	(1)	P01880-2, IGHD, Ig delta chain C region	1/0/0
	(2)	P01880-1, IGHD, Ig delta chain C region	
633		P01857, IGHG1, Ig gamma-1 chain C region	7/0/11
634		P01859, IGHG2, Ig gamma-2 chain C region	3/0/10

635		P01860, IGHG3, Ig gamma-3 chain C region	6/0/11
636		P01861, IGHG4, Ig gamma-4 chain C region	5/0/8
637	(1)	P01871-1, IGHM, Ig mu chain C region	0/0/15
	(2)	P01871-2, IGHM, Ig mu chain C region	
638		A0A0C4DH31, NA, NA	1/0/1
		P23083, IGHV1-2, Immunoglobulin heavy variable 1-2	
639		P01742, IGHV1-69, Immunoglobulin heavy variable 1-69	2/0/1
640		P01766, IGHV3-13, Immunoglobulin heavy variable 3-13	1/0/0
641	(1)	P01768, IGHV3-30, Immunoglobulin heavy variable 3-30	0/0/1
	(2)	P01764, IGHV3-23, Immunoglobulin heavy variable 3-23	
642		P01772, IGHV3-33, Immunoglobulin heavy variable 3-33	0/0/2
643	(1)	P01780, IGHV3-7, Immunoglobulin heavy variable 3-7	2/0/1
	(2)	A0A0B4J1V1, NA, NA	
	·	P01762, IGHV3-11, Immunoglobulin heavy variable 3-11	
	·	P01763, IGHV3-48, Immunoglobulin heavy variable 3-48	
	·	P01767, IGHV3-53, Immunoglobulin heavy variable 3-53	
644		P01782, IGHV3-9, Immunoglobulin heavy variable 3-9	1/0/0
645		P01824, IGHV4-39, Immunoglobulin heavy variable 4-39	3/0/0
		P01825, IGHV4-59, Immunoglobulin heavy variable 4-59	
		P06331, IGHV4-34, Immunoglobulin heavy variable 4-34	
646		P01834, IGKC, Ig kappa chain C region	6/0/0
647		P01599, IGKV1-17, Immunoglobulin kappa variable 1-17	2/0/1
648		P01593, IGKV1D-33, Immunoglobulin kappa variable 1D-33	1/0/0
		P01594, IGKV1-33, Immunoglobulin kappa variable 1-33	
649		A0A0C4DH73, NA, NA	1/0/1
		P01597, IGKV1-39, Immunoglobulin kappa variable 1-39	
		P01611, IGKV1D-12, Immunoglobulin kappa variable 1D-12	
		P04432, IGKV1D-39, Immunoglobulin kappa variable 1D-39	
650		P01602, IGKV1-5, Immunoglobulin kappa variable 1-5	1/0/1
651		P06310, IGKV2-30, Immunoglobulin kappa variable 2-30	1/0/1
652	(1)	P01614, IGKV2D-40, Immunoglobulin kappa variable 2D-40	1/0/1
	(2)	A0A075B6P5, NA, NA	
	·	P01615, IGKV2D-28, Immunoglobulin kappa variable 2D-28	
	(3)	A0A075B6S6, NA, NA	
653		P04433, IGKV3-11, Immunoglobulin kappa variable 3-11	1/0/2
654		P01624, IGKV3-15, Immunoglobulin kappa variable 3-15	2/0/2
655		P01619, IGKV3-20, Immunoglobulin kappa variable 3-20	1/0/3
656		P06312, IGKV4-1, Immunoglobulin kappa variable 4-1	5/0/0

657	(1)	P0CG05, IGLC2, Ig lambda-2 chain C regions	1/0/4
	(2)	P0CG06, IGLC3, Ig lambda-3 chain C regions	
658		P0CF74, IGLC6, Ig lambda-6 chain C region	0/0/4
659		A0M8Q6, IGLC7, Ig lambda-7 chain C region	1/0/4
660		P15814-1, IGLL1, Immunoglobulin lambda-like polypeptide 1	1/0/1
661	(1)	B9A064-1, IGLL5, Immunoglobulin lambda-like polypeptide 5	1/0/4
	(2)	P0CG04, IGLC1, Ig lambda-1 chain C regions	
662		P01700, IGLV1-47, Immunoglobulin lambda variable 1-47	1/0/0
663		P01706, IGLV2-11, Immunoglobulin lambda variable 2-11	2/0/0
664		P01704, IGLV2-14, Immunoglobulin lambda variable 2-14	1/0/0
665		P80748, IGLV3-21, Immunoglobulin lambda variable 3-21	2/0/0
666		P01717, IGLV3-25, Immunoglobulin lambda variable 3-25	2/0/0
667		P04211, IGLV7-43, Immunoglobulin lambda variable 7-43	2/0/0
668		Q12906-1-7, ILF3, Interleukin enhancer-binding factor 3	5/0/0
669		Q13418-1-3, ILK, Integrin-linked protein kinase	1/0/0
670	(1)	Q16891-1,2,4, IMMT, MICOS complex subunit MIC60	1/0/0
	(2)	Q16891-3, IMMT, MICOS complex subunit MIC60	
671		P08476, INHBA, Inhibin beta A chain	2/0/0
672		P09529, INHBB, Inhibin beta B chain	2/0/0
673		P55103, INHBC, Inhibin beta C chain	9/0/0
674		P58166, INHBE, Inhibin beta E chain	4/0/0
675	(1)	O00410-1,3, IPO5, Importin-5	1/0/0
	(2)	O00410-2, IPO5, Importin-5	
676		P46940, IQGAP1, Ras GTPase-activating-like protein IQGAP1	23/0/0
677		O14498, ISLR, Immunoglobulin superfamily containing leucine-rich repeat protein	4/0/0
678		Q9UKX5-1,2, ITGA11, Integrin alpha-11	3/0/0
679	(1)	P05556-1, ITGB1, Integrin beta-1	1/0/0
	(2)	P05556-2-5, ITGB1, Integrin beta-1	
680	(1)	O95965-1, ITGBL1, Integrin beta-like protein 1	1/12/0
	(2)	O95965-2, ITGBL1, Integrin beta-like protein 1	
	(3)	O95965-3, ITGBL1, Integrin beta-like protein 1	

681	(1)	P19827-1, ITIH1, Inter-alpha-trypsin inhibitor heavy chain H1	9/12/1
	(2)	P19827-2, ITIH1, Inter-alpha-trypsin inhibitor heavy chain H1	
	(3)	P19827-3, ITIH1, Inter-alpha-trypsin inhibitor heavy chain H1	
682		P19823, ITIH2, Inter-alpha-trypsin inhibitor heavy chain H2	32/0/0
683		Q06033-1,2, ITIH3, Inter-alpha-trypsin inhibitor heavy chain H3	36/0/0
684		Q14624-1, ITIH4, Inter-alpha-trypsin inhibitor heavy chain H4	4/0/34
685	(1)	Q14624-2,3, ITIH4, Inter-alpha-trypsin inhibitor heavy chain H4	0/0/34
	(2)	Q14624-4, ITIH4, Inter-alpha-trypsin inhibitor heavy chain H4	
686	(1)	Q86UX2-1, ITIH5, Inter-alpha-trypsin inhibitor heavy chain H5	0/5/0
	(2)	Q86UX2-2, ITIH5, Inter-alpha-trypsin inhibitor heavy chain H5	
	(3)	Q86UX2-3, ITIH5, Inter-alpha-trypsin inhibitor heavy chain H5	
	(4)	Q86UX2-4, ITIH5, Inter-alpha-trypsin inhibitor heavy chain H5	
687	(1)	Q8WWA0, ITLN1, Intelectin-1	9/0/0
	(2)	Q8WWU7-1,2, ITLN2, Intelectin-2	
688		Q9Y287-1,2, ITM2B, Integral membrane protein 2B	2/0/0
689		P07476, IVL, Involucrin	12/0/1
690		P01591, JCHAIN, Immunoglobulin J chain	5/0/0
691	(1)	P14923, JUP, Junction plakoglobin	21/0/4
	(2)	P35222-2, CTNNB1, Catenin beta-1	
692		Q96CX2, KCTD12, BTB/POZ domain-containing protein KCTD12	6/0/0
693		Q9Y2K7-1, KDM2A, Lysine-specific demethylase 2A	3/0/1
694		Q07666-1-3, KHDRBS1, KH domain-containing, RNA-binding, signal transduction-associated protein 1	3/0/0
695		Q6UXG2-3, KIAA1324, UPF0577 protein KIAA1324	2/0/0
696		P33176, KIF5B, Kinesin-1 heavy chain	7/0/3
697		O60282-1, KIF5C, Kinesin heavy chain isoform 5C	2/0/3
698		Q2M1P5, KIF7, Kinesin-like protein KIF7	0/0/2

699		O43240, KLK10, Kallikrein-10	1/0/0
700		P49862-1,2, KLK7, Kallikrein-7	3/0/0
701	(1)	P03952, KLKB1, Plasma kallikrein	37/0/0
	(2)	P20718-1, GZMH, Granzyme H	
702		Q03164-1-3, KMT2A, Histone-lysine N-methyltransferase 2A	4/0/0
703		P01042-1, KNG1, Kininogen-1	9/0/25
704	(1)	P01042-2, KNG1, Kininogen-1	0/0/25
	(2)	P01042-3, KNG1, Kininogen-1	
705		P50748-1, KNTC1, Kinetochore-associated protein 1	4/0/0
706	(1)	Q14974-1, KPNB1, Importin subunit beta-1	5/0/0
	(2)	Q14974-2, KPNB1, Importin subunit beta-1	
707		Q5T749, KPRP, Keratinocyte proline-rich protein	4/0/0
708		P04264, KRT1, Keratin, type II cytoskeletal 1	38/0/9
709		P13645, KRT10, Keratin, type I cytoskeletal 10	26/0/9
710	(1)	P13646-1, KRT13, Keratin, type I cytoskeletal 13	1/0/14
	(2)	P13646-2, KRT13, Keratin, type I cytoskeletal 13	
	(3)	P13646-3, KRT13, Keratin, type I cytoskeletal 13	
711		P02533, KRT14, Keratin, type I cytoskeletal 14	9/0/26
712	(1)	P19012-1, KRT15, Keratin, type I cytoskeletal 15	3/0/13
	(2)	P19012-2, KRT15, Keratin, type I cytoskeletal 15	
713		P08779, KRT16, Keratin, type I cytoskeletal 16	20/0/17
714		Q04695, KRT17, Keratin, type I cytoskeletal 17	17/0/18
715		P05783, KRT18, Keratin, type I cytoskeletal 18	28/0/3
716	(1)	P08727, KRT19, Keratin, type I cytoskeletal 19	20/0/13
	(2)	Q99456, KRT12, Keratin, type I cytoskeletal 12	
	(3)	Q8N1A0-1,2, KRT222, Keratin-like protein KRT222	
717		P35908, KRT2, Keratin, type II cytoskeletal 2 epidermal	23/0/11
718		P35900, KRT20, Keratin, type I cytoskeletal 20	0/0/5
719	(1)	Q9C075-1, KRT23, Keratin, type I cytoskeletal 23	0/0/2
	(2)	Q9C075-2, KRT23, Keratin, type I cytoskeletal 23	
720		Q2M2I5, KRT24, Keratin, type I cytoskeletal 24	1/0/5
721		Q7Z3Z0, KRT25, Keratin, type I cytoskeletal 25	13/0/13
722		Q7Z3Y9, KRT26, Keratin, type I cytoskeletal 26	5/0/4

723		Q7Z3Y8, KRT27, Keratin, type I cytoskeletal 27	11/0/14
724		Q7Z3Y7, KRT28, Keratin, type I cytoskeletal 28	7/0/12
725		P12035, KRT3, Keratin, type II cytoskeletal 3	1/0/12
726	(1)	Q15323, KRT31, Keratin, type I cuticular Ha1	6/0/20
	(2)	O76014, KRT37, Keratin, type I cuticular Ha7	
727		Q14532, KRT32, Keratin, type I cuticular Ha2	1/0/7
728		O76009, KRT33A, Keratin, type I cuticular Ha3-I	5/0/23
729		Q14525, KRT33B, Keratin, type I cuticular Ha3-II	3/0/17
730	(1)	O76011, KRT34, Keratin, type I cuticular Ha4	11/0/14
	(2)	Q6A162, KRT40, Keratin, type I cytoskeletal 40	
731		Q92764, KRT35, Keratin, type I cuticular Ha5	10/0/9
732		O76013-1,2, KRT36, Keratin, type I cuticular Ha6	0/0/7
733		O76015, KRT38, Keratin, type I cuticular Ha8	1/0/2
734		Q6A163, KRT39, Keratin, type I cytoskeletal 39	3/0/2
735		P19013, KRT4, Keratin, type II cytoskeletal 4	0/0/11
736		P13647, KRT5, Keratin, type II cytoskeletal 5	23/0/21
737		P02538, KRT6A, Keratin, type II cytoskeletal 6A	2/0/43
738		P04259, KRT6B, Keratin, type II cytoskeletal 6B	2/0/42
739		P48668, KRT6C, Keratin, type II cytoskeletal 6C	0/0/43
740		P08729, KRT7, Keratin, type II cytoskeletal 7	22/0/10
741		Q3SY84, KRT71, Keratin, type II cytoskeletal 71	18/0/23
742	(1)	Q14CN4-1, KRT72, Keratin, type II cytoskeletal 72	1/0/15
	(2)	Q14CN4-2, KRT72, Keratin, type II cytoskeletal 72	
	(3)	Q14CN4-3, KRT72, Keratin, type II cytoskeletal 72	
743	(1)	Q86Y46-1, KRT73, Keratin, type II cytoskeletal 73	2/0/22
	(2)	Q86Y46-2, KRT73, Keratin, type II cytoskeletal 73	
744		Q7RTS7, KRT74, Keratin, type II cytoskeletal 74	13/0/21
745		O95678, KRT75, Keratin, type II cytoskeletal 75	18/0/19
746		Q01546, KRT76, Keratin, type II cytoskeletal 2 oral	0/0/12
747		Q7Z794, KRT77, Keratin, type II cytoskeletal 1b	0/0/5
748	(1)	Q8N1N4-1, KRT78, Keratin, type II cytoskeletal 78	2/0/4
	(2)	Q8N1N4-2, KRT78, Keratin, type II cytoskeletal 78	
749		Q5XKE5, KRT79, Keratin, type II cytoskeletal 79	9/0/10

750	(1)	P05787-1,2, KRT8, Keratin, type II cytoskeletal 8	26/0/13
	(2)	Q9NSB2, KRT84, Keratin, type II cuticular Hb4	
			1/0/1
751	(1)	Q6KB66-1,2, KRT80, Keratin, type II cytoskeletal 80	
	(2)	Q6KB66-3, KRT80, Keratin, type II cytoskeletal 80	
752		Q14533, KRT81, Keratin, type II cuticular Hb1	1/0/34
753		Q9NSB4, KRT82, Keratin, type II cuticular Hb2	1/0/5
754	(1)	P78385, KRT83, Keratin, type II cuticular Hb3	2/0/33
	(2)	A6NCN2, KRT87P, Putative keratin-87 protein	
755		P78386, KRT85, Keratin, type II cuticular Hb5	13/0/27
756		O43790, KRT86, Keratin, type II cuticular Hb6	5/0/37
757		P35527, KRT9, Keratin, type I cytoskeletal 9	26/0/1
758		Q8IUC1, KRTAP11-1, Keratin-associated protein 11-1	2/0/0
759		P0C7H8, KRTAP2-3, Keratin-associated protein 2-3	
		Q9BYR9, KRTAP2-4, Keratin-associated protein 2-4	2/0/0
		Q9BYT5, KRTAP2-2, Keratin-associated protein 2-2	
		Q9BYU5, KRTAP2-1, Keratin-associated protein 2-1	
760		Q9BYR6, KRTAP3-3, Keratin-associated protein 3-3	1/0/0
761		Q9BYR4, KRTAP4-3, Keratin-associated protein 4-3	1/0/0
762	(1)	Q9BYP9-1-3, KRTAP9-9, Keratin-associated protein 9-9	
	·	Q9BYQ0, KRTAP9-8, Keratin-associated protein 9-8	
	·	Q9BYQ4, KRTAP9-2, Keratin-associated protein 9-2	1/0/0
	(2)	A8MTY7, KRTAP9-7, Keratin-associated protein 9-7	
	·	A8MXZ3, KRTAP9-1, Keratin-associated protein 9-1	
	·	Q9BYQ2, KRTAP9-4, Keratin-associated protein 9-4	
	·	Q9BYQ3, KRTAP9-3, Keratin-associated protein 9-3	
763		P24043, LAMA2, Laminin subunit alpha-2	29/0/0
764		Q16363-1,2, LAMA4, Laminin subunit alpha-4	14/0/0
765		O15230-1, LAMA5, Laminin subunit alpha-5	7/0/0
766		P07942, LAMB1, Laminin subunit beta-1	24/0/0
767		P55268, LAMB2, Laminin subunit beta-2	17/0/0
768		P11047, LAMC1, Laminin subunit gamma-1	29/0/0
769		P11279-1, LAMP1, Lysosome-associated membrane glycoprotein 1	2/0/0
			1/1/0
770	(1)	Q14847-1, LASP1, LIM and SH3 domain protein 1	
	(2)	Q14847-2, LASP1, LIM and SH3 domain protein 1	
	(3)	Q14847-3, LASP1, LIM and SH3 domain protein 1	
771		P18428, LBP, Lipopolysaccharide-binding protein	14/0/1

8/0/1 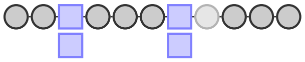

772 (1) P04180, LCAT, Phosphatidylcholine-sterol acyltransferase
(2) Q8NCC3-1, PLA2G15, Group XV phospholipase A2

16/0/4 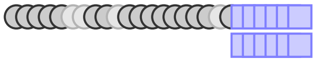

773 (1) P13796-1, LCP1, Plastin-2
(2) P13796-2, LCP1, Plastin-2

0/4/0 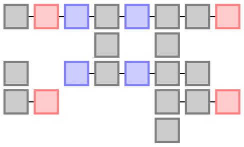

774 (1) P00338-1,3, LDHA, L-lactate dehydrogenase A chain
(2) P07195, LDHB, L-lactate dehydrogenase B chain
(3) P00338-2,5, LDHA, L-lactate dehydrogenase A chain
(4) P00338-4, LDHA, L-lactate dehydrogenase A chain
(5) P07864, LDHC, L-lactate dehydrogenase C chain
· Q6ZMR3, LDHAL6A, L-lactate dehydrogenase A-like 6A

0/6/0 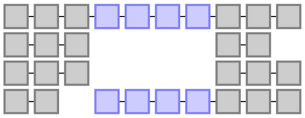

775 (1) P01130-1,5, LDLR, Low-density lipoprotein receptor
(2) P01130-2, LDLR, Low-density lipoprotein receptor
(3) P01130-3, LDLR, Low-density lipoprotein receptor
(4) P01130-4, LDLR, Low-density lipoprotein receptor

3/0/0 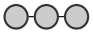

776 O14960, LECT2, Leukocyte cell-derived chemotaxin-2

2/0/0 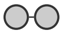

777 O00292-1, LEFTY2, Left-right determination factor 2
O75610, LEFTY1, Left-right determination factor 1

3/0/0 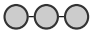

778 P41159, LEP, Leptin

1/0/0 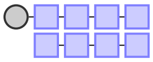

779 (1) Q8NES3-4, LFNG, Beta-1,3-N-acetylglucosaminyltransferase lunatic fringe
(2) Q8NES3-1-3, LFNG, Beta-1,3-N-acetylglucosaminyltransferase lunatic fringe

8/0/0 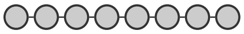

780 P09382, LGALS1, Galectin-1

4/0/0 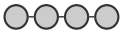

781 P17931, LGALS3, Galectin-3

16/0/0 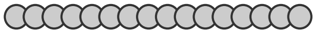

782 Q08380, LGALS3BP, Galectin-3-binding protein

4/0/0 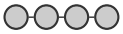

783 P47929, LGALS7, Galectin-7

7/0/0 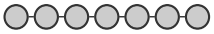

784 P49257, LMAN1, Protein ERGIC-53

2/0/0 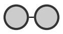

785 Q12907, LMAN2, Vesicular integral-membrane protein VIP36

8/0/2 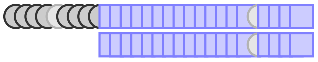

786 (1) P02545-1-3,6, LMNA, Prelamin-A/C [Cleaved into: Lamin-A/C
(2) P02545-4,5, LMNA, Prelamin-A/C [Cleaved into: Lamin-A/C

5/0/2 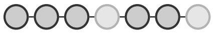

787 P20700, LMNB1, Lamin-B1

2/0/3 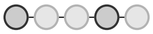

788 Q03252, LMNB2, Lamin-B2

10/0/1 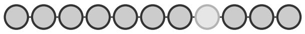

789 P28300, LOX, Protein-lysine 6-oxidase

11/0/1 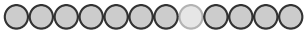

790 Q08397, LOXL1, Lysyl oxidase homolog 1

791 Q9Y4K0, LOXL2, Lysyl oxidase homolog 2 — 11/0/1

792 P58215-1,3, LOXL3, Lysyl oxidase homolog 3 — 3/0/1

793 P08519, LPA, Apolipoprotein(a) — 20/0/0

794 Q9UBY5, LPAR3, Lysophosphatidic acid receptor 3 — 5/0/0

795 P06858, LPL, Lipoprotein lipase — 3/0/0

796 P02750, LRG1, Leucine-rich alpha-2-glycoprotein — 5/0/1

797 Q07954-1, LRP1, Prolow-density lipoprotein receptor-related protein 1 — 49/0/1

798 Q9NZR2, LRP1B, Low-density lipoprotein receptor-related protein 1B — 22/0/1

799 P98164, LRP2, Low-density lipoprotein receptor-related protein 2 — 21/0/0

800 Q8TF66-1,2, LRRC15, Leucine-rich repeat-containing protein 15 — 1/0/0

801 Q8WUT4, LRRN4, Leucine-rich repeat neuronal protein 4 — 3/0/0

802 — 0/2/0
(1) Q86X29-1,4, LSR, Lipolysis-stimulated lipoprotein receptor
(2) Q86X29-2,3, LSR, Lipolysis-stimulated lipoprotein receptor
(3) Q86X29-5,6, LSR, Lipolysis-stimulated lipoprotein receptor

803 — 2/0/21
(1) Q14766-1,4, LTBP1, Latent-transforming growth factor beta-binding protein 1
(2) Q14766-2,5, LTBP1, Latent-transforming growth factor beta-binding protein 1

804 Q14766-3, LTBP1, Latent-transforming growth factor beta-binding protein 1 — 1/0/21

805 Q14767, LTBP2, Latent-transforming growth factor beta-binding protein 2 — 26/0/1

806 — 1/0/1
(1) Q9NS15-1, LTBP3, Latent-transforming growth factor beta-binding protein 3
(2) Q9NS15-2, LTBP3, Latent-transforming growth factor beta-binding protein 3

807 — 2/7/1
(1) Q8N2S1-2, LTBP4, Latent-transforming growth factor beta-binding protein 4
(2) Q8N2S1-1,3, LTBP4, Latent-transforming growth factor beta-binding protein 4
(3) Q8N2S1-4, LTBP4, Latent-transforming growth factor beta-binding protein 4

808 — 1/0/0
(1) P02788-1, LTF, Lactotransferrin
(2) P02788-2, LTF, Lactotransferrin

809 P51884, LUM, Lumican — 14/0/0

810		P61626, LYZ, Lysozyme C	13/0/0
811		Q9BRK4, LZTS2, Leucine zipper putative tumor suppressor 2	1/0/1
812		P20645, M6PR, Cation-dependent mannose-6-phosphate receptor	2/0/0
813		Q9UPN3-1, MACF1, Microtubule-actin cross-linking factor 1, isoforms 1/2/3/5	8/0/1
814		Q5VYJ5, MALRD1, MAM and LDL-receptor class A domain-containing protein 1	12/0/0
815		Q7Z304-1, MAMDC2, MAM domain-containing protein 2	1/0/0
816	(1)	P33908-1, MAN1A1, Mannosyl-oligosaccharide 1,2-alpha-mannosidase IA	5/0/0
	(2)	P33908-2, MAN1A1, Mannosyl-oligosaccharide 1,2-alpha-mannosidase IA	
817		P46821, MAP1B, Microtubule-associated protein 1B	6/0/0
818		Q96T17-1,2,4,5, MAP7D2, MAP7 domain-containing protein 2	0/0/2
819		Q9UEW3-1,2, MARCO, Macrophage receptor MARCO	3/0/0
820		P48740-1, MASP1, Mannan-binding lectin serine protease 1	5/0/11
821	(1)	P48740-2, MASP1, Mannan-binding lectin serine protease 1	0/0/11
	(2)	P48740-4, MASP1, Mannan-binding lectin serine protease 1	
822		P48740-3, MASP1, Mannan-binding lectin serine protease 1	1/0/10
823		O00187-1, MASP2, Mannan-binding lectin serine protease 2	3/0/6
824		O00187-2, MASP2, Mannan-binding lectin serine protease 2	1/0/6
825	(1)	P31153-1, MAT2A, S-adenosylmethionine synthase isoform type-2	1/2/0
	(2)	P31153-2, MAT2A, S-adenosylmethionine synthase isoform type-2	
	(3)	Q00266, MAT1A, S-adenosylmethionine synthase isoform type-1	
826	(1)	O00339-1-3, MATN2, Matrilin-2	1/0/0
	(2)	O00339-4, MATN2, Matrilin-2	
827	(1)	P43243-1, MATR3, Matrin-3	2/0/0
	(2)	P43243-2, MATR3, Matrin-3	
828		P02144, MB, Myoglobin	7/0/0
829		P11226, MBL2, Mannose-binding protein C	4/0/0
830		Q9HCC0-1,2, MCCC2, Methylcrotonoyl-CoA carboxylase beta chain, mitochondrial	1/0/0
831		P40925-1-3, MDH1, Malate dehydrogenase, cytoplasmic	2/0/0

2/0/0 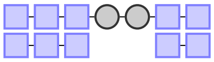

832 (1) P40926-1, MDH2, Malate dehydrogenase, mitochondrial

(2) P40926-2, MDH2, Malate dehydrogenase, mitochondrial

3/0/0 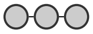

833 P21741-1, MDK, Midkine

11/0/0 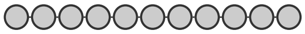

834 Q9NU22, MDN1, Midasin

4/0/0 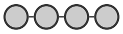

835 O75095-1,2, MEGF6, Multiple epidermal growth factor-like domains protein 6

2/0/0 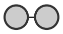

836 Q641Q3-1,2, METRNL, Meteorin-like protein

6/0/0 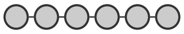

837 P55083-1,2, MFAP4, Microfibril-associated glycoprotein 4

2/0/1 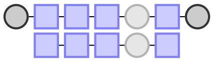

838 (1) Q13361-1, MFAP5, Microfibrillar-associated protein 5

(2) Q13361-2, MFAP5, Microfibrillar-associated protein 5

2/0/0 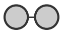

839 Q08431-1,3, MFGE8, Lactadherin

8/0/0 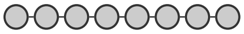

840 P26572, MGAT1, Alpha-1,3-mannosyl-glycoprotein 2-beta-N-acetylglucosaminyltransferase

5/0/0 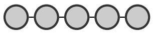

841 P08493-1,2, MGP, Matrix Gla protein

0/2/0 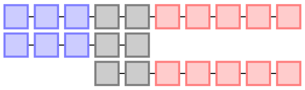

842 (1) Q9UNW1-1, MINPP1, Multiple inositol polyphosphate phosphatase 1

(2) Q9UNW1-2,3, MINPP1, Multiple inositol polyphosphate phosphatase 1

(3) Q9UNW1-4, MINPP1, Multiple inositol polyphosphate phosphatase 1

6/0/0 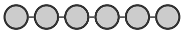

843 Q14165, MLEC, Malectin

3/0/0 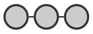

844 P50281, MMP14, Matrix metalloproteinase-14

3/0/0 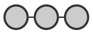

845 Q99542-1,3, MMP19, Matrix metalloproteinase-19

1/16/0 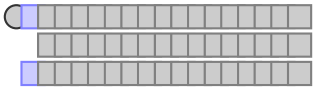

846 (1) P08253-1, MMP2, 72 kDa type IV collagenase

(2) P08253-2, MMP2, 72 kDa type IV collagenase

(3) P08253-3, MMP2, 72 kDa type IV collagenase

4/0/0 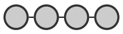

847 Q9H8L6, MMRN2, Multimerin-2

7/0/0 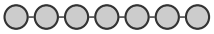

848 P05164-1-3, MPO, Myeloperoxidase

2/0/0 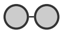

849 P25325-1,2, MPST, 3-mercaptopyruvate sulfurtransferase

7/0/0 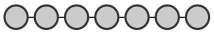

850 Q9UBG0, MRC2, C-type mannose receptor 2

17/0/0 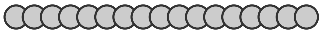

851 Q13421-1-4, MSLN, Mesothelin

11/0/5 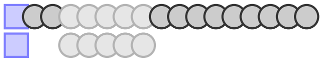

852 (1) P26038, MSN, Moesin

(2) P35241-1,5, RDX, Radixin

11/0/0 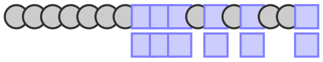

853 (1) P26927, MST1, Hepatocyte growth factor-like protein

(2) Q2TV78-1,2, MST1L, Putative macrophage stimulating 1-like protein

854		Q14764, MVP, Major vault protein	9/0/0
855		Q9NR99, MXRA5, Matrix-remodeling-associated protein 5	35/0/0
856		P84157-2, MXRA7, Matrix-remodeling-associated protein 7	2/0/0
857	(1)	P35580-1,3, MYH10, Myosin-10	1/0/19
	(2)	P35580-2,4,5, MYH10, Myosin-10	
858		P35749-1-4, MYH11, Myosin-11	1/0/20
			1/4/9
859	(1)	Q7Z406-1,2,6, MYH14, Myosin-14	
	(2)	Q7Z406-4, MYH14, Myosin-14	
	(3)	Q7Z406-5, MYH14, Myosin-14	
860	(1)	P11055, MYH3, Myosin-3	
	(2)	P12883, MYH7, Myosin-7	
	(3)	P12882, MYH1, Myosin-1	0/0/1
	·	P13535, MYH8, Myosin-8	
	·	Q9UKX2-1, MYH2, Myosin-2	
	·	Q9UKX3, MYH13, Myosin-13	
	·	Q9Y623, MYH4, Myosin-4	
861		P13533, MYH6, Myosin-6	1/0/2
			31/0/22
862	(1)	P35579-1, MYH9, Myosin-9	
	(2)	P35579-2, MYH9, Myosin-9	
			2/0/0
863	(1)	P05976-1, MYL1, Myosin light chain 1/3, skeletal muscle isoform	
	(2)	P05976-2, MYL1, Myosin light chain 1/3, skeletal muscle isoform	
864		O14950, MYL12B, Myosin regulatory light chain 12B	
		P19105, MYL12A, Myosin regulatory light chain 12A	3/0/5
865		P10916, MYL2, Myosin regulatory light chain 2, ventricular/cardiac muscle isoform	7/0/0
866		P08590, MYL3, Myosin light chain 3	6/0/0
867	(1)	P60660-1,2, MYL6, Myosin light polypeptide 6	3/0/0
	(2)	P14649, MYL6B, Myosin light chain 6B	
			0/0/5
868	(1)	P24844-1, MYL9, Myosin regulatory light polypeptide 9	
	(2)	P24844-2, MYL9, Myosin regulatory light polypeptide 9	
			2/3/0
869	(1)	Q15746-1-6, MYLK, Myosin light chain kinase, smooth muscle	
	(2)	Q15746-7,11, MYLK, Myosin light chain kinase, smooth muscle	

	(3)	Q15746-8,10, MYLK, Myosin light chain kinase, smooth muscle	
	(4)	Q15746-9, MYLK, Myosin light chain kinase, smooth muscle	
870		Q96A32, MYLPF, Myosin regulatory light chain 2, skeletal muscle isoform	6/0/0
871		O00159-1-3, MYO1C, Unconventional myosin-Ic	3/0/0
872		Q99972, MYOC, Myocilin	11/0/0
873		Q9UJ70-1,2, NAGK, N-acetyl-D-glucosamine kinase	1/0/0
874		P54802, NAGLU, Alpha-N-acetylglucosaminidase	1/0/0
875		P55209-1-3, NAP1L1, Nucleosome assembly protein 1-like 1<br>Q99733-1,2, NAP1L4, Nucleosome assembly protein 1-like 4	1/0/0
876		P54920, NAPA, Alpha-soluble NSF attachment protein	3/0/0
877		O43776-1,2, NARS, Asparagine--tRNA ligase, cytoplasmic	2/0/0
878		P41271-1,2, NBL1, Neuroblastoma suppressor of tumorigenicity 1	3/0/0
879		P13591-1,2, NCAM1, Neural cell adhesion molecule 1	3/0/0
880		P19338, NCL, Nucleolin	14/0/0
881		Q92597-1-3, NDRG1, Protein NDRG1	1/0/0
882		P28331-1-5, NDUFS1, NADH-ubiquinone oxidoreductase 75 kDa subunit, mitochondrial	2/0/0
			2/0/0
883	(1)	O75489-1, NDUFS3, NADH dehydrogenase [ubiquinone] iron-sulfur protein 3, mitochondrial	
	(2)	O75489-2, NDUFS3, NADH dehydrogenase [ubiquinone] iron-sulfur protein 3, mitochondrial	
884		Q9NVZ3-1,2,4, NECAP2, Adaptin ear-binding coat-associated protein 2	2/0/0
885		P12036-1,2, NEFH, Neurofilament heavy polypeptide	1/0/3
			1/0/0
886	(1)	P14543-1, NID1, Nidogen-1	
	(2)	P14543-2, NID1, Nidogen-1	
887		Q14112-1,2, NID2, Nidogen-2	10/0/0
888		P40261, NNMT, Nicotinamide N-methyltransferase	1/0/0
			1/0/2
889	(1)	Q15233-1, NONO, Non-POU domain-containing octamer-binding protein	
	(2)	Q15233-2, NONO, Non-POU domain-containing octamer-binding protein	
890		Q9UM47, NOTCH3, Neurogenic locus notch homolog protein 3	9/0/0
			0/6/0
891	(1)	P06748-1, NPM1, Nucleophosmin	

	(2)	P06748-2, NPM1, Nucleophosmin	
	(3)	P06748-3, NPM1, Nucleophosmin	
892		P01160, NPPA, Natriuretic peptides A	4/0/0
893		P15559-1-3, NQO1, NAD(P)H dehydrogenase [quinone] 1	3/0/0
894		P46459-1,2, NSF, Vesicle-fusing ATPase	2/0/0
895		Q02818, NUCB1, Nucleobindin-1	28/0/1
896		P80303-1,2, NUCB2, Nucleobindin-2	16/0/1
897	(1)	Q9H1E3-1, NUCKS1, Nuclear ubiquitous casein and cyclin-dependent kinase substrate 1	1/0/0
	(2)	Q9H1E3-2, NUCKS1, Nuclear ubiquitous casein and cyclin-dependent kinase substrate 1	
898		Q14980-1-4, NUMA1, Nuclear mitotic apparatus protein 1	1/0/1
899		Q86UD1, OAF, Out at first protein homolog	8/0/0
900		Q5VST9-2, OBSCN, Obscurin	2/0/1
901		Q02218-1,2, OGDH, 2-oxoglutarate dehydrogenase, mitochondrial	3/0/0
902		P20774, OGN, Mimecan	11/0/0
903	(1)	Q8WWZ8-1, OIT3, Oncoprotein-induced transcript 3 protein	2/0/0
	(2)	Q8WWZ8-2, OIT3, Oncoprotein-induced transcript 3 protein	
904		Q6UWY5, OLFML1, Olfactomedin-like protein 1	8/0/0
905		Q68BL8-1, OLFML2B, Olfactomedin-like protein 2B	3/0/0
906	(1)	Q9NRN5-1, OLFML3, Olfactomedin-like protein 3	0/5/0
	(2)	Q9NRN5-2, OLFML3, Olfactomedin-like protein 3	
	(3)	Q9NRN5-3, OLFML3, Olfactomedin-like protein 3	
907		Q99983, OMD, Osteomodulin	3/0/0
908		Q96CV9-1-3, OPTN, Optineurin	0/0/2
909		P02763, ORM1, Alpha-1-acid glycoprotein 1	5/0/6
910		P19652, ORM2, Alpha-1-acid glycoprotein 2	3/0/6
911		Q96FW1-1, OTUB1, Ubiquitin thioesterase OTUB1	3/0/0
912		P04220, NA, Ig mu heavy chain disease protein	2/0/15
913		Q32P28-1,3,4, P3H1, Prolyl 3-hydroxylase 1	2/0/0
914		P13674-1-3, P4HA1, Prolyl 4-hydroxylase subunit alpha-1	1/0/0
915		P07237, P4HB, Protein disulfide-isomerase	27/0/1
916	(1)	P11940-1,2, PABPC1, Polyadenylate-binding protein 1	2/0/0

(2) Q9H361, PABPC3, Polyadenylate-binding protein 3

917 (1) Q9UNF0-1, PACSIN2, Protein kinase C and casein kinase substrate in neurons protein 2 — 1/0/0
(2) Q9UNF0-2, PACSIN2, Protein kinase C and casein kinase substrate in neurons protein 2

918 P68402-1, PAFAH1B2, Platelet-activating factor acetylhydrolase IB subunit beta — 2/0/0

919 (1) P19021-1,3,5, PAM, Peptidyl-glycine alpha-amidating monooxygenase — 0/11/0
(2) P19021-2, PAM, Peptidyl-glycine alpha-amidating monooxygenase
(3) P19021-4,6, PAM, Peptidyl-glycine alpha-amidating monooxygenase

920 Q6UXH9-1, PAMR1, Inactive serine protease PAMR1 — 0/0/12

921 Q6UXH9-2, PAMR1, Inactive serine protease PAMR1 — 1/0/11

922 Q6UXH9-3, PAMR1, Inactive serine protease PAMR1 — 1/0/11

923 (1) O95428-1,5,6, PAPLN, Papilin — 0/6/0
(2) O95428-2, PAPLN, Papilin
(3) O95428-3, PAPLN, Papilin
(4) O95428-4, PAPLN, Papilin

924 O60260-1,2,4,6-8, PARK2, E3 ubiquitin-protein ligase parkin — 6/0/0

925 Q99497, PARK7, Protein deglycase DJ-1 — 2/0/0

926 Q96AQ6-1,2, PBXIP1, Pre-B-cell leukemia transcription factor-interacting protein 1 — 3/0/0

927 (1) Q15365, PCBP1, Poly(rC)-binding protein 1 — 3/0/5
(2) P57721-1-5, PCBP3, Poly(rC)-binding protein 3

928 Q15366-1-8, PCBP2, Poly(rC)-binding protein 2 — 2/0/5

929 P22061-1,2, PCMT1, Protein-L-isoaspartate(D-aspartate) O-methyltransferase — 2/0/0

930 Q15113, PCOLCE, Procollagen C-endopeptidase enhancer 1 — 20/0/0

931 Q9UKZ9, PCOLCE2, Procollagen C-endopeptidase enhancer 2 — 8/0/0

932 Q9UHG2, PCSK1N, ProSAAS — 5/0/0

933 (1) Q92824-1, PCSK5, Proprotein convertase subtilisin/kexin type 5 — 14/0/0
(2) Q92824-2, PCSK5, Proprotein convertase subtilisin/kexin type 5

7/0/0 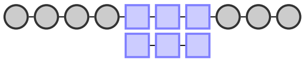

934 (1) Q8NBP7-1, PCSK9, Proprotein convertase subtilisin/kexin type 9

(2) Q8NBP7-2, PCSK9, Proprotein convertase subtilisin/kexin type 9

3/0/0 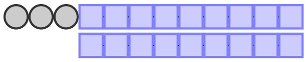

935 (1) Q9UHG3-1, PCYOX1, Prenylcysteine oxidase 1

(2) Q9UHG3-2, PCYOX1, Prenylcysteine oxidase 1

11/0/0 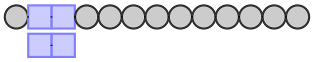

936 (1) Q8WUM4-1,2, PDCD6IP, Programmed cell death 6-interacting protein

(2) Q8WUM4-3, PDCD6IP, Programmed cell death 6-interacting protein

2/0/0 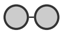

937 P01127-1,2, PDGFB, Platelet-derived growth factor subunit B

2/0/0 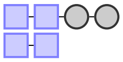

938 (1) Q9NRA1-1, PDGFC, Platelet-derived growth factor C

(2) Q9NRA1-2,3, PDGFC, Platelet-derived growth factor C

3/0/0 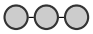

939 Q9GZP0-1,2, PDGFD, Platelet-derived growth factor D

7/0/0 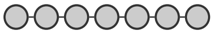

940 Q15198, PDGFRL, Platelet-derived growth factor receptor-like protein

14/0/0 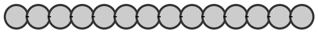

941 P30101, PDIA3, Protein disulfide-isomerase A3

17/0/0 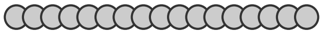

942 P13667, PDIA4, Protein disulfide-isomerase A4

9/0/0 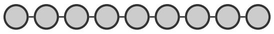

943 Q15084-1-5, PDIA6, Protein disulfide-isomerase A6

1/5/0 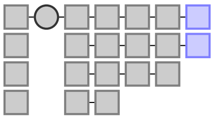

944 (1) Q96HC4-1, PDLIM5, PDZ and LIM domain protein 5

(2) Q96HC4-4,6, PDLIM5, PDZ and LIM domain protein 5

(3) Q96HC4-7, PDLIM5, PDZ and LIM domain protein 5

(4) Q96HC4-2,3, PDLIM5, PDZ and LIM domain protein 5

3/0/0 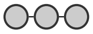

945 Q15121-1,2, PEA15, Astrocytic phosphoprotein PEA-15

8/0/0 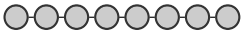

946 P01210, PENK, Proenkephalin-A [Cleaved into: Synenkephalin; Met-enkephalin

6/0/0 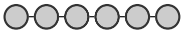

947 P07737, PFN1, Profilin-1

948 P0DJD7, PGA4, Pepsin A-4

3/0/0 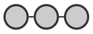

P0DJD8, PGA3, Pepsin A-3

P0DJD9, PGA5, Pepsin A-5

949 (1) P15259, PGAM2, Phosphoglycerate mutase 2

1/0/0 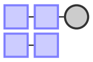

· P18669, PGAM1, Phosphoglycerate mutase 1

(2) Q8N0Y7, PGAM4, Probable phosphoglycerate mutase 4

4/0/0 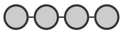

950 P52209-1,2, PGD, 6-phosphogluconate dehydrogenase, decarboxylating

4/0/0 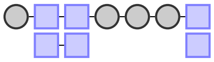

951 (1) P00558-1,2, PGK1, Phosphoglycerate kinase 1

(2) P07205, PGK2, Phosphoglycerate kinase 2

952		Q96PD5-1,2, PGLYRP2, N-acetylmuramoyl-L-alanine amidase	10/0/0
953		P35232-1, PHB, Prohibitin	8/0/0
			2/0/0
954	(1)	Q99623-1, PHB2, Prohibitin-2	
	(2)	Q99623-2, PHB2, Prohibitin-2	
955		O43175, PHGDH, D-3-phosphoglycerate dehydrogenase	7/0/0
956		Q6NSJ2-1, PHLDB3, Pleckstrin homology-like domain family B member 3	0/0/2
957		Q13492-1-5, PICALM, Phosphatidylinositol-binding clathrin assembly protein	3/0/0
958		P01833, PIGR, Polymeric immunoglobulin receptor	8/0/0
959	(1)	P14618-1, PKM, Pyruvate kinase PKM	0/18/0
	(2)	P14618-2, PKM, Pyruvate kinase PKM	
	(3)	P14618-3, PKM, Pyruvate kinase PKM	
	(4)	P30613-1,2, PKLR, Pyruvate kinase PKLR	
			1/0/0
960	(1)	Q13835-2, PKP1, Plakophilin-1	
	(2)	Q13835-1, PKP1, Plakophilin-1	
961		Q9Y446-1,2, PKP3, Plakophilin-3	5/0/0
962		P14555, PLA2G2A, Phospholipase A2, membrane associated	8/0/0
963		Q13093, PLA2G7, Platelet-activating factor acetylhydrolase	2/0/0
			0/10/0
964	(1)	P00750-1, PLAT, Tissue-type plasminogen activator	
	(2)	P00750-2, PLAT, Tissue-type plasminogen activator	
	(3)	P00750-3, PLAT, Tissue-type plasminogen activator	
	(4)	P00750-4, PLAT, Tissue-type plasminogen activator	
			1/129/6
965	(1)	Q15149-5, PLEC, Plectin	
	(2)	Q15149-3, PLEC, Plectin	
	(3)	Q15149-1,2,4,6-9, PLEC, Plectin	
966	(1)	P00747, PLG, Plasminogen	51/0/6
	(2)	Q15195, PLGLA, Plasminogen-like protein A	
967		Q02325, PLGLB1, Plasminogen-like protein B	1/0/5
			1/0/0
968	(1)	O60664-1,3, PLIN3, Perilipin-3	
	(2)	O60664-4, PLIN3, Perilipin-3	
969		Q96Q06-1,2, PLIN4, Perilipin-4	6/0/0
970		Q02809-1,2, PLOD1, Procollagen-lysine,2-oxoglutarate 5-dioxygenase 1	4/0/0

971		O60568, PLOD3, Procollagen-lysine,2-oxoglutarate 5-dioxygenase 3	3/0/0
972		P13797-1-3, PLS3, Plastin-3	8/0/4
			0/13/0
973	(1)	P55058-1, PLTP, Phospholipid transfer protein	
	(2)	P55058-2,3, PLTP, Phospholipid transfer protein	
	(3)	P55058-4, PLTP, Phospholipid transfer protein	
			1/0/0
974	(1)	Q7Z5L7-3,4, PODN, Podocan	
	(2)	Q7Z5L7-1,2, PODN, Podocan	
975		Q8WVV4-1,2, POF1B, Protein POF1B	4/0/0
976	(1)	P27169, PON1, Serum paraoxonase/arylesterase 1	13/0/1
	(2)	Q15165-1-3, PON2, Serum paraoxonase/arylesterase 2	
977		Q15166, PON3, Serum paraoxonase/lactonase 3	4/0/1
978		Q15063-1, POSTN, Periostin	3/0/45
979		Q15063-2, POSTN, Periostin	0/0/43
980		Q15063-3, POSTN, Periostin	0/0/45
981		Q15063-4, POSTN, Periostin	0/0/42
982		Q15063-5, POSTN, Periostin	0/0/46
983		Q15063-6, POSTN, Periostin	0/0/40
984		Q15063-7, POSTN, Periostin	0/0/39
985		Q6S8J7-1,2, POTEA, POTE ankyrin domain family member A	3/0/1
986		A0JP26, POTEB3, POTE ankyrin domain family member B3	2/0/4
		B2RU33, POTEC, POTE ankyrin domain family member C	
		Q6S5H4-1,3, POTEB, POTE ankyrin domain family member B	
987		Q6S8J3-1, POTEE, POTE ankyrin domain family member E	5/0/13
988	(1)	A5A3E0, POTEF, POTE ankyrin domain family member F	3/0/11
	(2)	P0CG38, POTEI, POTE ankyrin domain family member I	
	·	P0CG39, POTEJ, POTE ankyrin domain family member J	
989		Q9BYX7, POTEKP, Putative beta-actin-like protein 3	1/0/5
990		Q15181, PPA1, Inorganic pyrophosphatase	4/0/0
991		P02775, PPBP, Platelet basic protein	1/0/0
992	(1)	P62937-1, PPIA, Peptidyl-prolyl cis-trans isomerase A	
	(2)	Q9Y536, PPIAL4A, Peptidyl-prolyl cis-trans isomerase A-like 4A	2/3/1
	(3)	A0A075B759, NA, NA	

	·	A0A0B4J2A2, NA, NA	
	·	F5H284, PPIAL4D, Peptidyl-prolyl cis-trans isomerase A-like 4D	
	·	P0DN26, PPIAL4F, Peptidyl-prolyl cis-trans isomerase A-like 4F	
	·	P0DN37, PPIAL4G, Peptidyl-prolyl cis-trans isomerase A-like 4G	
	(4)	P62937-2, PPIA, Peptidyl-prolyl cis-trans isomerase A	
993		P23284, PPIB, Peptidyl-prolyl cis-trans isomerase B	4/0/1
994		P45877, PPIC, Peptidyl-prolyl cis-trans isomerase C	1/0/1
995		O60437, PPL, Periplakin	23/0/2
996		P36873-1,2, PPP1CC, Serine/threonine-protein phosphatase PP1-gamma catalytic subunit	5/0/0
		P62136-1-3, PPP1CA, Serine/threonine-protein phosphatase PP1-alpha catalytic subunit	
		P62140, PPP1CB, Serine/threonine-protein phosphatase PP1-beta catalytic subunit	
997		Q15435-1-5, PPP1R7, Protein phosphatase 1 regulatory subunit 7	1/0/0
998	(1)	P67775-1,2, PPP2CA, Serine/threonine-protein phosphatase 2A catalytic subunit alpha isoform	1/0/0
	(2)	P62714, PPP2CB, Serine/threonine-protein phosphatase 2A catalytic subunit beta isoform	
999		P30153, PPP2R1A, Serine/threonine-protein phosphatase 2A 65 kDa regulatory subunit A alpha isoform	7/0/0
1000		Q5MIZ7-1-3,5, PPP4R3B, Serine/threonine-protein phosphatase 4 regulatory subunit 3B	2/0/0
1001	(1)	Q96NZ9-1, PRAP1, Proline-rich acidic protein 1	0/1/5
	(2)	Q96NZ9-2, PRAP1, Proline-rich acidic protein 1	
	(3)	Q96NZ9-4, PRAP1, Proline-rich acidic protein 1	
1002		Q96NZ9-3, PRAP1, Proline-rich acidic protein 1	1/0/5
1003		O75626-1, PRDM1, PR domain zinc finger protein 1	1/0/2
1004		O75626-2, PRDM1, PR domain zinc finger protein 1	2/0/2
1005	(1)	Q06830, PRDX1, Peroxiredoxin-1	4/0/1
	(2)	Q13162, PRDX4, Peroxiredoxin-4	
1006		P32119-1, PRDX2, Peroxiredoxin-2	5/0/1
1007	(1)	P30044-1,2, PRDX5, Peroxiredoxin-5, mitochondrial	1/0/0
	(2)	P30044-3,4, PRDX5, Peroxiredoxin-5, mitochondrial	
1008		P30041, PRDX6, Peroxiredoxin-6	11/0/0
1009		P51888, PRELP, Prolargin	8/0/0
1010		Q9Y2Y8, PRG3, Proteoglycan 3	1/0/0

1011	(1)	Q92954-1,6, PRG4, Proteoglycan 4	0/3/44
	(2)	Q92954-2, PRG4, Proteoglycan 4	
	(3)	Q92954-5, PRG4, Proteoglycan 4	
1012	(1)	Q92954-3, PRG4, Proteoglycan 4	0/0/44
	(2)	Q92954-4, PRG4, Proteoglycan 4	
1013		P13861-1,2, PRKAR2A, cAMP-dependent protein kinase type II-alpha regulatory subunit	2/0/0
1014		P14314-1,2, PRKCSH, Glucosidase 2 subunit beta	10/0/0
1015		P78527-1,2, PRKDC, DNA-dependent protein kinase catalytic subunit	2/0/0
1016		P04156-1,2, PRNP, Major prion protein	3/0/0
1017		P04070-1,2, PROC, Vitamin K-dependent protein C	11/0/0
1018		P07225, PROS1, Vitamin K-dependent protein S	26/0/1
1019		P22891-1,2, PROZ, Vitamin K-dependent protein Z	3/0/0
1020		P41219-1,2, PRPH, Peripherin	0/0/5
1021		P07477, PRSS1, Trypsin-1	2/0/2
1022	(1)	P07478, PRSS2, Trypsin-2	1/2/1
	(2)	Q8NHM4, PRSS3P2, Putative trypsin-6	
	(3)	P35030-1-5, PRSS3, Trypsin-3	
1023		O95084-1, PRSS23, Serine protease 23	2/0/0
1024		P07602-1-3, PSAP, Prosaposin	13/0/0
1025		Q6NUJ1, PSAPL1, Proactivator polypeptide-like 1 [Cleaved into: Saposin A-like; Saposin B-Val-like; Saposin B-like; Saposin C-like; Saposin D-like]	2/0/0
1026		P25786-1,2, PSMA1, Proteasome subunit alpha type-1	2/0/0
1027		P25788-1,2, PSMA3, Proteasome subunit alpha type-3	2/0/0
1028	(1)	P25789-1, PSMA4, Proteasome subunit alpha type-4	1/0/0
	(2)	P25789-2, PSMA4, Proteasome subunit alpha type-4	
1029	(1)	O14818-1, PSMA7, Proteasome subunit alpha type-7	1/1/0
	(2)	O14818-2, PSMA7, Proteasome subunit alpha type-7	
	(3)	Q8TAA3-1,2,5, PSMA8, Proteasome subunit alpha type-7-like	
1030		P20618, PSMB1, Proteasome subunit beta type-1	1/0/0
1031		P49721, PSMB2, Proteasome subunit beta type-2	2/0/1
1032		P28074-1,2, PSMB5, Proteasome subunit beta type-5	2/0/0
1033	(1)	Q99436-1, PSMB7, Proteasome subunit beta type-7	1/0/0

	(2)	Q99436-2, PSMB7, Proteasome subunit beta type-7	
1034		P62191-1, PSMC1, 26S protease regulatory subunit 4	2/0/2
1035		P62191-2, PSMC1, 26S protease regulatory subunit 4	1/0/2
1036		P17980, PSMC3, 26S protease regulatory subunit 6A	4/0/0
1037		P62333, PSMC6, 26S protease regulatory subunit 10B	3/0/0
1038		Q99460-1,2, PSMD1, 26S proteasome non-ATPase regulatory subunit 1	5/0/0
1039		O43242-1, PSMD3, 26S proteasome non-ATPase regulatory subunit 3	3/0/0
1040		Q15008-1-4, PSMD6, 26S proteasome non-ATPase regulatory subunit 6	1/0/0
1041		Q06323-1-3, PSME1, Proteasome activator complex subunit 1	5/0/0
1042		Q9UL46, PSME2, Proteasome activator complex subunit 2	6/0/0
1043	(1)	P26599-1-3, PTBP1, Polypyrimidine tract-binding protein 1	2/0/0
	(2)	O95758-1-7, PTBP3, Polypyrimidine tract-binding protein 3	
1044		P41222, PTGDS, Prostaglandin-H2 D-isomerase	4/0/0
1045		Q16647, PTGIS, Prostacyclin synthase	2/0/0
1046		P06454-1,2, PTMA, Prothymosin alpha [Cleaved into: Prothymosin alpha, N-terminally processed; Thymosin alpha-1]	1/0/0
1047		P20962, PTMS, Parathymosin	2/0/0
1048		P10586-1,2, PTPRF, Receptor-type tyrosine-protein phosphatase F	2/0/0
1049	(1)	Q6NZI2-1, PTRF, Polymerase I and transcript release factor	5/3/0
	(2)	Q6NZI2-2, PTRF, Polymerase I and transcript release factor	
	(3)	Q6NZI2-3, PTRF, Polymerase I and transcript release factor	
1050		Q00577, PURA, Transcriptional activator protein Pur-alpha	2/0/0
1051	(1)	Q92626-1, PXDN, Peroxidasin homolog	13/0/1
	(2)	Q92626-2, PXDN, Peroxidasin homolog	
1052	(1)	P20742-1, PZP, Pregnancy zone protein	8/0/9
	(2)	P20742-2, PZP, Pregnancy zone protein	
1053	(1)	Q16769-1, QPCT, Glutaminyl-peptide cyclotransferase	1/0/0
	(2)	Q16769-2, QPCT, Glutaminyl-peptide cyclotransferase	
1054	(1)	O00391-1, QSOX1, Sulfhydryl oxidase 1	3/0/0
	(2)	O00391-2, QSOX1, Sulfhydryl oxidase 1	
1055		P61026, RAB10, Ras-related protein Rab-10	4/0/0

1056	(1)	Q15907-1, RAB11B, Ras-related protein Rab-11B	1/0/0
	(2)	P62491-1,2, RAB11A, Ras-related protein Rab-11A	
	·	Q15907-2, RAB11B, Ras-related protein Rab-11B	
1057		P61106, RAB14, Ras-related protein Rab-14	3/0/0
1058	(1)	Q9H0U4, RAB1B, Ras-related protein Rab-1B	1/0/0
	(2)	P62820-1, RAB1A, Ras-related protein Rab-1A	
1059		O95716, RAB3D, Ras-related protein Rab-3D	0/0/2
1060		P51149, RAB7A, Ras-related protein Rab-7a	4/0/0
1061		Q92930, RAB8B, Ras-related protein Rab-8B	1/0/1
1062		Q15276-1,2, RABEP1, Rab GTPase-binding effector protein 1	4/0/1
1063	(1)	P63000-1,2, RAC1, Ras-related C3 botulinum toxin substrate 1	2/0/1
	(2)	P15153, RAC2, Ras-related C3 botulinum toxin substrate 2	
	·	P60763, RAC3, Ras-related C3 botulinum toxin substrate 3	
1064		P63244, RACK1, Receptor of activated protein C kinase 1	9/0/0
1065		P54727-1, RAD23B, UV excision repair protein RAD23 homolog B	3/0/0
1066		Q9UKM9-1,2, RALY, RNA-binding protein Raly	2/0/0
1067		P62826, RAN, GTP-binding nuclear protein Ran	5/0/0
1068		A6NIZ1, NA, Ras-related protein Rap-1b-like protein	1/0/1
		P61224-1-3, RAP1B, Ras-related protein Rap-1b	
		P62834, RAP1A, Ras-related protein Rap-1A	
1069	(1)	P49788-1, RARRES1, Retinoic acid receptor responder protein 1	1/0/0
	(2)	P49788-2, RARRES1, Retinoic acid receptor responder protein 1	
1070		Q99969, RARRES2, Retinoic acid receptor responder protein 2	8/0/0
1071		P54136-1,2, RARS, Arginine--tRNA ligase, cytoplasmic	3/0/0
1072		Q09028-1-3, RBBP4, Histone-binding protein RBBP4	2/0/0
		Q16576-1,2, RBBP7, Histone-binding protein RBBP7	
1073		P02753, RBP4, Retinol-binding protein 4	10/0/0
1074		Q15293-1,2, RCN1, Reticulocalbin-1	2/0/0
1075		Q96D15, RCN3, Reticulocalbin-3	4/0/0
1076		Q06141, REG3A, Regenerating islet-derived protein 3-alpha	4/0/0
1077	(1)	P61586, RHOA, Transforming protein RhoA	1/0/1
	(2)	P08134, RHOC, Rho-related GTP-binding protein RhoC	
1078		P62745, RHOB, Rho-related GTP-binding protein RhoB	1/0/1

1079		Q9NPQ8-1-4, RIC8A, Synembryn-A	1/0/1	
1080		Q13129, RLF, Zinc finger protein Rlf	6/0/0	
1081		P07998, RNASE1, Ribonuclease pancreatic	7/0/0	
1082		P34096, RNASE4, Ribonuclease 4	13/0/0	
1083		Q93091, RNASE6, Ribonuclease K6	2/0/0	
1084		Q6ZMZ0-1-4, RNF19B, E3 ubiquitin-protein ligase RNF19B	3/0/0	
1085		P13489, RNH1, Ribonuclease inhibitor	7/0/1	
1086		P15927-1-3, RPA2, Replication protein A 32 kDa subunit	2/0/0	
1087		P62906, RPL10A, 60S ribosomal protein L10a	2/0/0	
1088		P30050-1, RPL12, 60S ribosomal protein L12	4/0/0	
1089		P26373-1,2, RPL13, 60S ribosomal protein L13	1/0/1	
1090		P40429, RPL13A, 60S ribosomal protein L13a	2/0/0	
1091		P50914, RPL14, 60S ribosomal protein L14	3/0/0	
1092		P61313-2, RPL15, 60S ribosomal protein L15	3/0/0	
1093	(1)	P18621-1,3, RPL17, 60S ribosomal protein L17	0/0/2	
	(2)	P18621-2, RPL17, 60S ribosomal protein L17		
1094		P35268, RPL22, 60S ribosomal protein L22	2/0/0	
1095		P62750, RPL23A, 60S ribosomal protein L23a	4/0/0	
1096		P83731, RPL24, 60S ribosomal protein L24	2/0/0	
1097		P61254, RPL26, 60S ribosomal protein L26<br>Q9UNX3, RPL26L1, 60S ribosomal protein L26-like 1	2/0/0	
1098	(1)	P46779-1-3, RPL28, 60S ribosomal protein L28	2/0/0	
	(2)	P46779-4,5, RPL28, 60S ribosomal protein L28		
1099		P39023, RPL3, 60S ribosomal protein L3	4/0/0	
1100		P62888, RPL30, 60S ribosomal protein L30	2/0/0	
1101		P62899-1-3, RPL31, 60S ribosomal protein L31	1/0/0	
1102		P49207, RPL34, 60S ribosomal protein L34	2/0/0	
1103		P42766, RPL35, 60S ribosomal protein L35	3/0/0	
1104		P18077, RPL35A, 60S ribosomal protein L35a	2/0/0	
1105		A6NKH3, RPL37AP8, Putative 60S ribosomal protein L37a-like protein<br>P61513, RPL37A, 60S ribosomal protein L37a	1/0/0	
1106		P46777, RPL5, 60S ribosomal protein L5	8/0/0	

1107		Q02878, RPL6, 60S ribosomal protein L6	1/0/0
1108		P18124, RPL7, 60S ribosomal protein L7	1/0/0
1109		P62424, RPL7A, 60S ribosomal protein L7a	3/0/0
1110		P62917, RPL8, 60S ribosomal protein L8	2/0/0
1111		P32969, RPL9, 60S ribosomal protein L9	3/0/0
1112	(1)	P05388-1,2, RPLP0, 60S acidic ribosomal protein P0	1/0/0
	(2)	Q8NHW5, RPLP0P6, 60S acidic ribosomal protein P0-like	
1113		P05387, RPLP2, 60S acidic ribosomal protein P2	2/0/0
1114		P04843, RPN1, Dolichyl-diphosphooligosaccharide--protein glycosyltransferase subunit 1	10/0/0
1115		P04844-1,2, RPN2, Dolichyl-diphosphooligosaccharide--protein glycosyltransferase subunit 2	3/0/0
1116		P46783, RPS10, 40S ribosomal protein S10	2/0/0
1117		P25398, RPS12, 40S ribosomal protein S12	3/0/0
1118		P62277, RPS13, 40S ribosomal protein S13	1/0/0
1119		P62263, RPS14, 40S ribosomal protein S14	4/0/0
1120		P62249, RPS16, 40S ribosomal protein S16	3/0/0
1121		P62269, RPS18, 40S ribosomal protein S18	3/0/0
1122		P39019, RPS19, 40S ribosomal protein S19	3/0/0
1123		P15880, RPS2, 40S ribosomal protein S2	3/0/0
1124		P60866-1,2, RPS20, 40S ribosomal protein S20	2/0/0
1125		P62266, RPS23, 40S ribosomal protein S23	2/0/0
1126	(1)	P62979, RPS27A, Ubiquitin-40S ribosomal protein S27a	
	(2)	P0CG47, UBB, Polyubiquitin-B [Cleaved into: Ubiquitin]	2/0/0
	·	P0CG48, UBC, Polyubiquitin-C [Cleaved into: Ubiquitin]	
	·	P62987, UBA52, Ubiquitin-60S ribosomal protein L40	
1127		P23396-1,2, RPS3, 40S ribosomal protein S3	8/0/0
1128		P61247, RPS3A, 40S ribosomal protein S3a	2/0/0
1129	(1)	P62701, RPS4X, 40S ribosomal protein S4, X isoform	4/0/1
	(2)	P22090, RPS4Y1, 40S ribosomal protein S4, Y isoform 1	
1130		Q8TD47, RPS4Y2, 40S ribosomal protein S4, Y isoform 2	1/0/1
1131		P46782, RPS5, 40S ribosomal protein S5 [Cleaved into: 40S ribosomal protein S5, N-terminally processed]	2/0/0
1132		P62753, RPS6, 40S ribosomal protein S6	5/0/0
1133		P62081, RPS7, 40S ribosomal protein S7	2/0/0
1134		P46781, RPS9, 40S ribosomal protein S9	2/0/0

1135		P08865, RPSA, 40S ribosomal protein SA	9/0/0
1136	(1)	Q9P2E9-1,2, RRBP1, Ribosome-binding protein 1	5/0/0
	(2)	Q9P2E9-3, RRBP1, Ribosome-binding protein 1	
1137		O95197-1-7, RTN3, Reticulon-3	1/0/0
		Q16799-1-3, RTN1, Reticulon-1	
1138		Q8WXD0-1,2, RXFP2, Relaxin receptor 2	2/0/0
1139		P60903, S100A10, Protein S100-A10	7/0/0
1140		P31949, S100A11, Protein S100-A11	3/0/0
1141		Q96FQ6, S100A16, Protein S100-A16	2/0/0
1142		P29034, S100A2, Protein S100-A2	4/0/0
1143		P06703, S100A6, Protein S100-A6	3/0/0
1144		P05109, S100A8, Protein S100-A8	6/0/0
1145		P06702, S100A9, Protein S100-A9	4/0/0
1146	(1)	P0DJI8, SAA1, Serum amyloid A-1 protein	3/0/7
	(2)	P0DJI9-2, SAA2, Serum amyloid A-2 protein	
1147		P0DJI9-1, SAA2, Serum amyloid A-2 protein	2/0/7
1148		P35542, SAA4, Serum amyloid A-4 protein	7/0/0
1149		P49591, SARS, Serine--tRNA ligase, cytoplasmic	5/0/0
1150	(1)	Q6UWP8-1, SBSN, Suprabasin	4/0/0
	(2)	Q6UWP8-2, SBSN, Suprabasin	
1151		Q96GP6-1,2, SCARF2, Scavenger receptor class F member 2	5/0/0
1152		P13521, SCG2, Secretogranin-2	23/0/0
1153	(1)	Q8WXD2-1, SCG3, Secretogranin-3	2/0/0
	(2)	Q8WXD2-2, SCG3, Secretogranin-3	
1154	(1)	P05408-2, SCG5, Neuroendocrine protein 7B2	1/0/0
	(2)	P05408-1, SCG5, Neuroendocrine protein 7B2	
1155		P11684, SCGB1A1, Uteroglobin	3/0/0
1156		Q12765-1-3, SCRN1, Secernin-1	2/0/0
1157	(1)	Q9NQ36-1,3, SCUBE2, Signal peptide, CUB and EGF-like domain-containing protein 2	2/0/0
	(2)	Q9NQ36-2, SCUBE2, Signal peptide, CUB and EGF-like domain-containing protein 2	
1158		O00560-1-3, SDCBP, Syntenin-1	4/0/0

0/9/0

1159 (1) Q9BRK5-1, SDF4, 45 kDa calcium-binding protein
(2) Q9BRK5-2, SDF4, 45 kDa calcium-binding protein
(3) Q9BRK5-5, SDF4, 45 kDa calcium-binding protein
(4) Q9BRK5-6, SDF4, 45 kDa calcium-binding protein
(5) Q9BRK5-3,4, SDF4, 45 kDa calcium-binding protein

1160 O95810, SDPR, Serum deprivation-response protein 6/0/0

1161 Q15436-1,2, SEC23A, Protein transport protein Sec23A 3/0/0

1162 Q9Y6Y8-1,2, SEC23IP, SEC23-interacting protein 2/0/0

1163 P61619-1,3, SEC61A1, Protein transport protein Sec61 subunit alpha isoform 1 1/0/0

1164 Q5TEA6-1,2, SEL1L2, Protein sel-1 homolog 2 2/0/0

1165 Q13214-1,2, SEMA3B, Semaphorin-3B 2/0/0

1166 Q13275-1,2, SEMA3F, Semaphorin-3F 4/0/0

1167 P49908, SEPP1, Selenoprotein P 8/0/0

1/0/0

1168 (1) Q15019-1,2, SEPT2, Septin-2
(2) Q15019-3, SEPT2, Septin-2

1169 Q16181-1,2, SEPT7, Septin-7 8/0/0

1170 Q9UHD8-1,2,5,7, SEPT9, Septin-9 3/0/0

1171 Q8NC51-1-4, SERBP1, Plasminogen activator inhibitor 1 RNA-binding protein 2/0/0

4/20/1

1172 (1) P01009-1, SERPINA1, Alpha-1-antitrypsin
(2) P01009-2, SERPINA1, Alpha-1-antitrypsin
(3) P01009-3, SERPINA1, Alpha-1-antitrypsin

1173 Q9UK55, SERPINA10, Protein Z-dependent protease inhibitor 20/0/0

1174 Q86U17, SERPINA11, Serpin A11 1/0/0

8/0/0

1175 (1) P01011-1, SERPINA3, Alpha-1-antichymotrypsin
(2) P01011-2, SERPINA3, Alpha-1-antichymotrypsin

1176 P29622, SERPINA4, Kallistatin 18/0/0

1177 P05154, SERPINA5, Plasma serine protease inhibitor 12/0/0

1178 P08185, SERPINA6, Corticosteroid-binding globulin 3/0/0

1179 P05543, SERPINA7, Thyroxine-binding globulin 8/0/0

0/0/4

1180 (1) P29508-1, SERPINB3, Serpin B3

	(2)	P29508-2, SERPINB3, Serpin B3	
1181		P48594, SERPINB4, Serpin B4	3/0/4
1182	(1)	P36952-1, SERPINB5, Serpin B5	3/0/0
	(2)	P36952-2, SERPINB5, Serpin B5	
1183		P01008, SERPINC1, Antithrombin-III	26/0/0
1184		P05546, SERPIND1, Heparin cofactor 2	27/0/0
1185		P05121-1,2, SERPINE1, Plasminogen activator inhibitor 1	11/0/0
1186		P07093-1-3, SERPINE2, Glia-derived nexin	2/0/0
1187		P36955, SERPINF1, Pigment epithelium-derived factor	18/0/0
1188	(1)	P08697-1, SERPINF2, Alpha-2-antiplasmin	3/0/0
	(2)	P08697-2, SERPINF2, Alpha-2-antiplasmin	
1189		P05155-1-3, SERPING1, Plasma protease C1 inhibitor	9/0/0
1190		P50454, SERPINH1, Serpin H1	9/0/0
1191	(1)	P31947-1, SFN, 14-3-3 protein sigma	1/0/5
	(2)	P31947-2, SFN, 14-3-3 protein sigma	
1192		P23246-1,2, SFPQ, Splicing factor, proline- and glutamine-rich	8/0/2
1193		Q8N474, SFRP1, Secreted frizzled-related protein 1	2/0/0
1194		Q96HF1, SFRP2, Secreted frizzled-related protein 2	11/0/1
1195		Q6FHJ7, SFRP4, Secreted frizzled-related protein 4	2/0/0
1196		Q9H299, SH3BGRL3, SH3 domain-binding glutamic acid-rich-like protein 3	3/0/0
1197	(1)	P04278-1, SHBG, Sex hormone-binding globulin	0/9/0
	(2)	P04278-2-4, SHBG, Sex hormone-binding globulin	
	(3)	P04278-5, SHBG, Sex hormone-binding globulin	
1198		Q9ULL8-1, SHROOM4, Protein Shroom4	5/0/0
1199		Q9H173, SIL1, Nucleotide exchange factor SIL1	2/0/0
1200		P63208-1,2, SKP1, S-phase kinase-associated protein 1	3/0/1
1201		P53985-1, SLC16A1, Monocarboxylate transporter 1	2/0/0
1202		Q00325-1,2, SLC25A3, Phosphate carrier protein, mitochondrial	2/0/0
1203	(1)	P12236, SLC25A6, ADP/ATP translocase 3	1/3/0
	(2)	Q9H0C2, SLC25A31, ADP/ATP translocase 4	
	(3)	P05141, SLC25A5, ADP/ATP translocase 2	
	·	P12235, SLC25A4, ADP/ATP translocase 1	

1204		Q9HAS3-2, SLC28A3, Solute carrier family 28 member 3	3/0/0
1205		Q9BWU0, SLC4A1AP, Kanadaptin	5/0/0
1206		O14745-1, SLC9A3R1, Na(+)/H(+) exchange regulatory cofactor NHE-RF1	3/0/0
1207		O75094-1,2,4, SLIT3, Slit homolog 3 protein	6/0/0
1208		P03973, SLPI, Antileukoproteinase	15/0/0
1209		P55000, SLURP1, Secreted Ly-6/uPAR-related protein 1	2/0/0
1210		P52788-1, SMS, Spermine synthase	2/0/0
1211		Q7KZF4, SND1, Staphylococcal nuclease domain-containing protein 1	3/0/0
1212		Q8TER0-1,3-5, SNED1, Sushi, nidogen and EGF-like domain-containing protein 1	9/0/0
1213		P62318-1,2, SNRPD3, Small nuclear ribonucleoprotein Sm D3	2/0/0
1214		Q9UMY4-1-3, SNX12, Sorting nexin-12	1/0/0
1215	(1)	Q9UNH7-1, SNX6, Sorting nexin-6	1/0/0
	(2)	Q9UNH7-2, SNX6, Sorting nexin-6	
1216	(1)	P04179-1,4, SOD2, Superoxide dismutase [Mn], mitochondrial	0/3/0
	(2)	P04179-2, SOD2, Superoxide dismutase [Mn], mitochondrial	
	(3)	P04179-3, SOD2, Superoxide dismutase [Mn], mitochondrial	
1217		P08294, SOD3, Extracellular superoxide dismutase [Cu-Zn]	6/0/0
1218		Q07889-1, SOS1, Son of sevenless homolog 1	3/0/0
1219		P23497-1, SP100, Nuclear autoantigen Sp-100	4/0/0
1220		P09486, SPARC, SPARC	17/0/0
1221	(1)	Q14515-1, SPARCL1, SPARC-like protein 1	3/0/0
	(2)	Q14515-2, SPARCL1, SPARC-like protein 1	
1222		Q9C093-1,2, SPEF2, Sperm flagellar protein 2	2/0/1
1223		Q9NQ38-1-3, SPINK5, Serine protease inhibitor Kazal-type 5	7/0/0
1224		Q9HCB6, SPON1, Spondin-1	18/0/0
1225		Q9BUD6, SPON2, Spondin-2	2/0/0
1226		P10451-1-5, SPP1, Osteopontin	5/0/0
1227		Q13103, SPP2, Secreted phosphoprotein 24	4/0/0
1228		Q7Z699, SPRED1, Sprouty-related, EVH1 domain-containing protein 1	4/0/0

1229		P22528, SPRR1B, Cornifin-B	3/0/0
		P35321, SPRR1A, Cornifin-A	
1230		P22532, SPRR2D, Small proline-rich protein 2D	2/0/0
1231		Q96PI1, SPRR4, Small proline-rich protein 4	9/0/0
1232		Q13813-1-3, SPTAN1, Spectrin alpha chain, non-erythrocytic 1	20/0/0
1233	(1)	Q01082-1, SPTBN1, Spectrin beta chain, non-erythrocytic 1	1/0/19
	(2)	Q01082-2, SPTBN1, Spectrin beta chain, non-erythrocytic 1	
1234		Q01082-3, SPTBN1, Spectrin beta chain, non-erythrocytic 1	1/0/19
1235	(1)	O15020-1, SPTBN2, Spectrin beta chain, non-erythrocytic 2	1/0/3
	(2)	O15020-2, SPTBN2, Spectrin beta chain, non-erythrocytic 2	
1236	(1)	Q9H254-1, SPTBN4, Spectrin beta chain, non-erythrocytic 4	2/0/2
	(2)	Q9H254-2,4, SPTBN4, Spectrin beta chain, non-erythrocytic 4	
1237		Q9Y6N5, SQRDL, Sulfide:quinone oxidoreductase, mitochondrial	4/0/0
1238	(1)	P78539-1,4, SRPX, Sushi repeat-containing protein SRPX	0/6/0
	(2)	P78539-3, SRPX, Sushi repeat-containing protein SRPX	
	(3)	P78539-2,5, SRPX, Sushi repeat-containing protein SRPX	
1239		Q8IYB3-1,2, SRRM1, Serine/arginine repetitive matrix protein 1	1/0/1
1240		Q07955-1-3, SRSF1, Serine/arginine-rich splicing factor 1	6/0/0
1241		O75494-1-6, SRSF10, Serine/arginine-rich splicing factor 10	2/0/0
1242		Q01130-1, SRSF2, Serine/arginine-rich splicing factor 2	1/0/1
1243		P84103-1,2, SRSF3, Serine/arginine-rich splicing factor 3	3/0/1
1244		Q16629-1-4, SRSF7, Serine/arginine-rich splicing factor 7	3/0/1
1245		A1L4H1-1,2, SSC5D, Soluble scavenger receptor cysteine-rich domain-containing protein SSC5D	3/0/0
1246		Q9UNL2-1,2, SSR3, Translocon-associated protein subunit gamma	2/0/0
1247	(1)	P50502, ST13, Hsc70-interacting protein	1/3/0
	(2)	Q8NFI4, ST13P5, Putative protein FAM10A5	
	(3)	Q8IZP2, ST13P4, Putative protein FAM10A4	
1248	(1)	Q9NY15-1, STAB1, Stabilin-1	5/0/0
	(2)	Q9NY15-2, STAB1, Stabilin-1	
1249		P31948-1-3, STIP1, Stress-induced-phosphoprotein 1	4/0/0

1250		P53999, SUB1, Activated RNA polymerase II transcriptional coactivator p15	3/0/0
1251	(1)	P0DMM9-1,3, SULT1A3, Sulfotransferase 1A3	2/3/0
	·	P0DMN0, SULT1A4, Sulfotransferase 1A4	
	(2)	P50226, SULT1A2, Sulfotransferase 1A2	
	(3)	P50225-1, SULT1A1, Sulfotransferase 1A1	
	(4)	P50225-2, SULT1A1, Sulfotransferase 1A1	
1252		O60279, SUSD5, Sushi domain-containing protein 5	4/0/0
1253	(1)	Q4LDE5-1, SVEP1, Sushi, von Willebrand factor type A, EGF and pentraxin domain-containing protein 1	9/22/0
	(2)	Q4LDE5-2, SVEP1, Sushi, von Willebrand factor type A, EGF and pentraxin domain-containing protein 1	
	(3)	Q4LDE5-3, SVEP1, Sushi, von Willebrand factor type A, EGF and pentraxin domain-containing protein 1	
	(4)	Q4LDE5-4, SVEP1, Sushi, von Willebrand factor type A, EGF and pentraxin domain-containing protein 1	
1254	(1)	O60506-1-4, SYNCRIP, Heterogeneous nuclear ribonucleoprotein Q	3/0/3
	(2)	O60506-5, SYNCRIP, Heterogeneous nuclear ribonucleoprotein Q	
1255		Q92804-1,2, TAF15, TATA-binding protein-associated factor 2N	4/0/0
1256		Q01995, TAGLN, Transgelin	11/0/0
1257		P37802-1,2, TAGLN2, Transgelin-2	8/0/0
1258		P37837, TALDO1, Transaldolase	2/0/0
1259		Q99426-1,2, TBCB, Tubulin-folding cofactor B	3/0/0
1260		Q07283, TCHH, Trichohyalin	112/0/6
1261		P20062-1,2, TCN2, Transcobalamin-2	4/0/0
1262		P17987, TCP1, T-complex protein 1 subunit alpha	3/0/0
1263		Q8NFU7, TET1, Methylcytosine dioxygenase TET1	2/0/0
1264		P02787, TF, Serotransferrin	37/0/0
1265		Q00059-1,2, TFAM, Transcription factor A, mitochondrial	3/0/0
1266		Q92734-1-4, TFG, Protein TFG	2/0/1
1267		P10646-1, TFPI, Tissue factor pathway inhibitor	1/0/2
1268		P10646-2, TFPI, Tissue factor pathway inhibitor	1/0/2
1269		Q15582, TGFBI, Transforming growth factor-beta-induced protein ig-h3	19/0/0

1270 Q03167-1,2, TGFBR3, Transforming growth factor beta receptor type 3 — 12/0/0

1271 (1) P22735-1, TGM1, Protein-glutamine gamma-glutamyltransferase K — 2/0/0
(2) P22735-2, TGM1, Protein-glutamine gamma-glutamyltransferase K

1272 (1) P21980-1, TGM2, Protein-glutamine gamma-glutamyltransferase 2 — 2/0/0
(2) P21980-2, TGM2, Protein-glutamine gamma-glutamyltransferase 2

1273 Q08188, TGM3, Protein-glutamine gamma-glutamyltransferase E — 4/0/0

1274 (1) P07996-1, THBS1, Thrombospondin-1 — 5/0/2
(2) P07996-2, THBS1, Thrombospondin-1

1275 P35442, THBS2, Thrombospondin-2 — 26/0/2

1276 (1) P49746-1, THBS3, Thrombospondin-3 — 2/0/1
(2) P49746-2, THBS3, Thrombospondin-3

1277 P35443, THBS4, Thrombospondin-4 — 20/0/3

1278 (1) P31483-3, TIA1, Nucleolysin TIA-1 isoform p40 — 1/0/0
(2) P31483-1,2, TIA1, Nucleolysin TIA-1 isoform p40

1279 Q86XR7-2, TICAM2, TIR domain-containing adapter molecule 2 — 1/0/0
Q9Y3B3-1,2, TMED7, Transmembrane emp24 domain-containing protein 7

1280 P35590-1, TIE1, Tyrosine-protein kinase receptor Tie-1 — 2/0/0

1281 P01033, TIMP1, Metalloproteinase inhibitor 1 — 3/0/0

1282 P16035, TIMP2, Metalloproteinase inhibitor 2 — 9/0/0

1283 (1) Q9GZM7-1, TINAGL1, Tubulointerstitial nephritis antigen-like — 1/0/0
(2) Q9GZM7-3, TINAGL1, Tubulointerstitial nephritis antigen-like

1284 O43897-1, TLL1, Tolloid-like protein 1 — 1/0/3

1285 Q9Y490, TLN1, Talin-1 — 43/0/8

1286 Q9Y4G6, TLN2, Talin-2 — 1/0/8

1287 P49755, TMED10, Transmembrane emp24 domain-containing protein 10 — 5/0/0

1288 Q15363, TMED2, Transmembrane emp24 domain-containing protein 2 — 3/0/0

1289		Q9BVK6, TMED9, Transmembrane emp24 domain-containing protein 9	2/0/0
1290		Q9BTV4, TMEM43, Transmembrane protein 43	2/0/0
1291		Q9BXS4, TMEM59, Transmembrane protein 59	2/0/0
1292		P82094-1,2, TMF1, TATA element modulatory factor	2/0/0
1293	(1)	P24821-1,4, TNC, Tenascin	2/0/36
	(2)	P24821-2,3,5, TNC, Tenascin	
1294		P24821-6, TNC, Tenascin	1/0/36
1295		P98066, TNFAIP6, Tumor necrosis factor-inducible gene 6 protein	4/0/0
1296		O00300, TNFRSF11B, Tumor necrosis factor receptor superfamily member 11B	2/0/0
1297		P13805-1-3, TNNT1, Troponin T, slow skeletal muscle	4/0/0
1298		O14787-1,2, TNPO2, Transportin-2	3/0/0
1299	(1)	P22105-1,3, TNXB, Tenascin-X	1/9/0
	(2)	P22105-2, TNXB, Tenascin-X	
	(3)	P22105-4, TNXB, Tenascin-X	
	(4)	Q16473, TNXA, Putative tenascin-XA	
1300		P60174-1,3,4, TPI1, Triosephosphate isomerase	2/0/0
1301		P09493-1, TPM1, Tropomyosin alpha-1 chain	0/0/22
1302		P09493-4,10, TPM1, Tropomyosin alpha-1 chain	0/0/23
1303	(1)	P09493-3, TPM1, Tropomyosin alpha-1 chain	0/0/23
	(2)	P09493-2, TPM1, Tropomyosin alpha-1 chain	
1304		P09493-5, TPM1, Tropomyosin alpha-1 chain	1/0/15
1305		P09493-6, TPM1, Tropomyosin alpha-1 chain	0/0/21
1306		P09493-7, TPM1, Tropomyosin alpha-1 chain	0/0/21
1307		P09493-8, TPM1, Tropomyosin alpha-1 chain	0/0/22
1308		P09493-9, TPM1, Tropomyosin alpha-1 chain	0/0/22
1309		P07951-1, TPM2, Tropomyosin beta chain	4/0/21
1310	(1)	P07951-2, TPM2, Tropomyosin beta chain	0/0/20
	(2)	P07951-3, TPM2, Tropomyosin beta chain	
1311		P06753-1, TPM3, Tropomyosin alpha-3 chain	0/0/16
1312	(1)	P06753-2, TPM3, Tropomyosin alpha-3 chain	0/0/18
	(2)	P06753-3,6, TPM3, Tropomyosin alpha-3 chain	

0/0/22

1313 (1) P06753-5, TPM3, Tropomyosin alpha-3 chain

(2) P06753-4, TPM3, Tropomyosin alpha-3 chain

(3) P06753-7, TPM3, Tropomyosin alpha-3 chain

1314 P67936-1, TPM4, Tropomyosin alpha-4 chain 4/0/15

1315 P67936-2, TPM4, Tropomyosin alpha-4 chain 1/0/17

1316 O14773-1, TPP1, Tripeptidyl-peptidase 1 2/0/0

1317 O15050, TRANK1, TPR and ankyrin repeat-containing protein 1 6/0/0

1318 Q14134-1,2, TRIM29, Tripartite motif-containing protein 29 10/0/0

1319 Q8WUA8, TSKU, Tsukushin 9/0/0

5/32/3

1320 (1) Q8WZ42-12, TTN, Titin

(2) Q8WZ42-4,7,11, TTN, Titin

(3) Q8WZ42-13, TTN, Titin

(4) Q8WZ42-3,9,10, TTN, Titin

(5) Q8WZ42-2, TTN, Titin

(6) Q8WZ42-5, TTN, Titin

(7) Q8WZ42-1,8, TTN, Titin

1321 P02766, TTR, Transthyretin 10/0/0

1322 Q71U36-1,2, TUBA1A, Tubulin alpha-1A chain 0/0/17

0/0/17

1323 (1) P68363-1, TUBA1B, Tubulin alpha-1B chain

(2) P68363-2, TUBA1B, Tubulin alpha-1B chain

1324 Q9BQE3, TUBA1C, Tubulin alpha-1C chain 1/0/15

0/0/15

1325 (1) Q13748-1, TUBA3C, Tubulin alpha-3C/D chain

(2) Q13748-2, TUBA3C, Tubulin alpha-3C/D chain

1326 Q6PEY2, TUBA3E, Tubulin alpha-3E chain 1/0/12

1327 P68366-1,2, TUBA4A, Tubulin alpha-4A chain 3/0/13

1328 Q9NY65-1,2, TUBA8, Tubulin alpha-8 chain 1/0/10

1329 A6NHL2-1,2, TUBAL3, Tubulin alpha chain-like 3 1/0/3

1330 (1) P07437, TUBB, Tubulin beta chain

(2) Q13885, TUBB2A, Tubulin beta-2A chain 1/8/5

· Q9BVA1, TUBB2B, Tubulin beta-2B chain

(3) A6NNZ2, NA, Tubulin beta-8 chain-like protein LOC260334

· Q3ZCM7, TUBB8, Tubulin beta-8 chain

	(4)	Q13509-1, TUBB3, Tubulin beta-3 chain	
	(5)	Q13509-2, TUBB3, Tubulin beta-3 chain	
	(6)	P04350, TUBB4A, Tubulin beta-4A chain	
	(7)	Q9H4B7, TUBB1, Tubulin beta-1 chain	
	(8)	P68371, TUBB4B, Tubulin beta-4B chain	
1331		Q9BUF5, TUBB6, Tubulin beta-6 chain	1/0/5
1332		P49411, TUFM, Elongation factor Tu, mitochondrial	5/0/0
1333	(1)	Q9GZX9-1, TWSG1, Twisted gastrulation protein homolog 1	4/0/0
	(2)	Q9GZX9-2, TWSG1, Twisted gastrulation protein homolog 1	
1334	(1)	Q8NBS9-1, TXNDC5, Thioredoxin domain-containing protein 5	1/0/1
	(2)	Q8NBS9-2, TXNDC5, Thioredoxin domain-containing protein 5	
1335	(1)	P19971-1, TYMP, Thymidine phosphorylase	1/0/0
	(2)	P19971-2, TYMP, Thymidine phosphorylase	
1336		P22314-1,2, UBA1, Ubiquitin-like modifier-activating enzyme 1	10/0/0
1337		P68036-1, UBE2L3, Ubiquitin-conjugating enzyme E2 L3	1/0/1
1338		P61088, UBE2N, Ubiquitin-conjugating enzyme E2 N<br>Q5JXB2, UBE2NL, Putative ubiquitin-conjugating enzyme E2 N-like	4/0/0
1339		Q5T4S7-1-4, UBR4, E3 ubiquitin-protein ligase UBR4	4/0/1
1340		P15374, UCHL3, Ubiquitin carboxyl-terminal hydrolase isozyme L3	2/0/0
1341	(1)	O60701-1, UGDH, UDP-glucose 6-dehydrogenase	0/5/0
	(2)	O60701-2, UGDH, UDP-glucose 6-dehydrogenase	
	(3)	O60701-3, UGDH, UDP-glucose 6-dehydrogenase	
1342		Q9NYU2-1,2, UGGT1, UDP-glucose:glycoprotein glucosyltransferase 1	2/0/0
1343		Q16851-1,2, UGP2, UTP--glucose-1-phosphate uridylyltransferase	5/0/0
1344		Q92900-1,2, UPF1, Regulator of nonsense transcripts 1	2/0/0
1345		P14927-1,2, UQCRB, Cytochrome b-c1 complex subunit 7	2/0/0
1346		P31930, UQCRC1, Cytochrome b-c1 complex subunit 1, mitochondrial	2/0/0
1347		P22695, UQCRC2, Cytochrome b-c1 complex subunit 2, mitochondrial	4/0/0
1348		P07919, UQCRH, Cytochrome b-c1 complex subunit 6, mitochondrial	2/0/0

1349 O60763-1,2, USO1, General vesicular transport factor p115 — 6/0/0

1350 P54578-1-3, USP14, Ubiquitin carboxyl-terminal hydrolase 14 — 2/0/0

1351 P45974-1,2, USP5, Ubiquitin carboxyl-terminal hydrolase 5 — 4/0/0

1352 Q93008-1,3, USP9X, Probable ubiquitin carboxyl-terminal hydrolase FAF-X — 2/0/6

1353 — 0/0/6
(1) O00507-1, USP9Y, Probable ubiquitin carboxyl-terminal hydrolase FAF-Y
(2) O00507-2, USP9Y, Probable ubiquitin carboxyl-terminal hydrolase FAF-Y

1354 O75691, UTP20, Small subunit processome component 20 homolog — 2/0/0

1355 P26640-1, VARS, Valine--tRNA ligase — 2/0/0

1356 — 2/3/0
(1) Q99536-1, VAT1, Synaptic vesicle membrane protein VAT-1 homolog
(2) Q99536-2, VAT1, Synaptic vesicle membrane protein VAT-1 homolog
(3) Q99536-3, VAT1, Synaptic vesicle membrane protein VAT-1 homolog

1357 — 1/0/0
(1) P19320-1,3, VCAM1, Vascular cell adhesion protein 1
(2) P19320-2, VCAM1, Vascular cell adhesion protein 1

1358 — 22/0/0
(1) P18206-1,2, VCL, Vinculin
(2) P18206-3, VCL, Vinculin

1359 P55072, VCP, Transitional endoplasmic reticulum ATPase — 11/0/0

1360 P21796, VDAC1, Voltage-dependent anion-selective channel protein 1 — 2/0/0

1361 P49767, VEGFC, Vascular endothelial growth factor C — 17/0/0

1362 O15240, VGF, Neurosecretory protein VGF [Cleaved into: Neuroendocrine regulatory peptide-1 — 10/0/0

1363 P08670, VIM, Vimentin — 28/0/10

1364 P98155-1,2, VLDLR, Very low-density lipoprotein receptor — 5/0/0

1365 Q96QK1, VPS35, Vacuolar protein sorting-associated protein 35 — 2/0/0

1366 P04004, VTN, Vitronectin — 20/0/0

1367 — 79/0/0
(1) P04275-1, VWF, von Willebrand factor
(2) P04275-2, VWF, von Willebrand factor

1368 — 2/0/0
(1) P23381-1, WARS, Tryptophan--tRNA ligase, cytoplasmic
(2) P23381-2, WARS, Tryptophan--tRNA ligase, cytoplasmic

1369		Q6ZQQ6-1,2, WDR87, WD repeat-containing protein 87	4/0/1
1370		Q9HC57, WFDC1, WAP four-disulfide core domain protein 1	2/0/0
1371	(1)	O76076-1, WISP2, WNT1-inducible-signaling pathway protein 2	6/0/1
	(2)	O76076-2, WISP2, WNT1-inducible-signaling pathway protein 2	
1372		Q9HCS7, XAB2, Pre-mRNA-splicing factor SYF1	3/0/0
1373		Q9NQW7-1,3, XPNPEP1, Xaa-Pro aminopeptidase 1	2/0/0
1374		O14980, XPO1, Exportin-1	5/0/0
1375		P13010, XRCC5, X-ray repair cross-complementing protein 5	5/0/0
1376		P12956-1,2, XRCC6, X-ray repair cross-complementing protein 6	11/0/0
1377		O15498-1,2, YKT6, Synaptobrevin homolog YKT6	4/0/0
1378		P31946-1,2, YWHAB, 14-3-3 protein beta/alpha	1/0/8
1379	(1)	P62258-1, YWHAE, 14-3-3 protein epsilon	1/0/3
	(2)	P62258-2, YWHAE, 14-3-3 protein epsilon	
1380		P61981, YWHAG, 14-3-3 protein gamma	9/0/7
1381		Q04917, YWHAH, 14-3-3 protein eta	9/0/6
1382		P27348, YWHAQ, 14-3-3 protein theta	10/0/5
1383	(1)	P63104-1, YWHAZ, 14-3-3 protein zeta/delta	7/0/7
	(2)	P63104-2, YWHAZ, 14-3-3 protein zeta/delta	
1384		P17010-1,3, ZFX, Zinc finger X-chromosomal protein	1/0/8
1385		P17010-2, ZFX, Zinc finger X-chromosomal protein	1/0/8
1386		Q9H2Y7-1, ZNF106, Zinc finger protein 106	6/0/0
1387		Q5VUA4-1,2, ZNF318, Zinc finger protein 318	1/0/2
1388		Q06732, ZNF33B, Zinc finger protein 33B	5/0/0
1389		Q17R98-1-3, ZNF827, Zinc finger protein 827	5/0/0
1390		Q15942-1,2, ZYX, Zyxin	2/0/0
1391	(1)	O43149-1, ZZEF1, Zinc finger ZZ-type and EF-hand domain-containing protein 1	4/0/0
	(2)	O43149-2, ZZEF1, Zinc finger ZZ-type and EF-hand domain-containing protein 1	

Isobaric Tag Quantification Report P7115_Run 3 [*]

isobar R package v1.18.0

February 6, 2017

Channel	Class
126	*P4-treat*
127	*P4-ctrl*
128	*P6-treat*
129	*P6-ctrl*
130	*P7-treat*
131	*P7-ctrl*

Column Descriptions	
protein	Protein name, accession code, and description.
group	Protein group size, only displayed if it is not '1/1'. First number: Number of group reporters; second number: group size. See Protein Group Details for details.
peptides	Number of peptides for protein.
spectra	Number of idenfitied spectra for protein.
quant	Number of spectra with quantitative information for *ch2* versus *ch1*.
ratio	Ratio of protein in channel *ch2* relative to *ch1*.
*	Significance of protein ratio in sample.

1 Protein Relative Quantitation

Number of quantified proteins: P4-treat/P4-ctrl: 1625; P6-treat/P6-ctrl: 1622; P7-treat/P7-ctrl: 1627

1.1 Quantified Proteins

#	protein	group	peptides	spectra	ch1	ch2	quant	ratio
					127	126	64	**0.05**
**1**	125116		36	433	129	128	55	**0.51**
					131	130	65	**1.12**
					127	126	132	**0.50**
**2**	136429		8	134	129	128	132	**0.59**
					131	130	112	**1.07**
					127	126	45	**0.67**
**3**	162648		27	357	129	128	45	**1.51**
					131	130	47	**0.97**

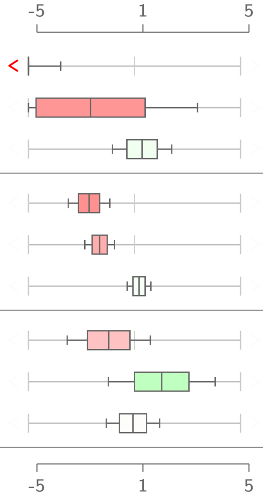

[*] This report was generated using the `isobar` R package version 1.18.0 [built using R 3.3.2; ; 2017-02-05 14:44:42 UTC; unix]. If you use it in published work, please cite 'Breitwieser FP *et al.*: General statistical modeling of data from protein relative expression isobaric tags, *Journal of Proteome Research* 2011' and 'Breitwieser FP and Colinge J: isobarPTM: A software tool for the quantitative analysis of post-translationally modified proteins,*Journal of Proteomics* 2013'

#	protein	group	peptides	spectra	ch1	ch2	quant	ratio
**4**	A0A075B6Q5		1	3	127	126	3	**0.87**
					129	128	3	**0.91**
					131	130	3	**0.99**
**5**	A0A0A0MS15		2	2	127	126	2	**0.87**
					129	128	2	**0.91**
					131	130	2	**1.02**
**6**	A0A0B4J1V0		3	5	127	126	4	**0.75**
					129	128	4	**0.89**
					131	130	4	**1.04**
**7**	A0A0C4DH25		3	10	127	126	2	**0.63**
					129	128	2	**0.72**
					131	130	2	**0.76**
**8**	A0A0C4DH29	1/2	3	5	127	126	1	**0.97**
					129	128	1	**0.88**
					131	130	1	**1.07**
**9**	A0A0C4DH33		2	3	127	126	2	**0.55**
					129	128	2	**0.97**
					131	130	2	**1.08**
**10**	A1BG $P04217_1$: Alpha-1B-glycoprotein	1/2	10	20	127	126	19	**1.11**
					129	128	18	**1.06**
					131	130	16	**0.98**
**11**	A2M P01023: Alpha-2-macroglobulin		49	132	127	126	101	**0.89**
					129	128	104	**0.95**
					131	130	106	**0.84**
**12**	A2ML1 $A8K2U0_1$: Alpha-2-macroglobulin-like protein 1	1/2	9	9	127	126	7	**10.48**
					129	128	9	**1.06**
					131	130	9	**1.00**
**13**	A4GALT Q9NPC4: Lactosylceramide 4-alpha-galactosyltransferase		3	3	127	126	3	**0.89**
					129	128	3	**0.86**
					131	130	3	**1.12**
**14**	AARS $P49588_1$: Alanine--tRNA ligase, cytoplasmic	1/2	16	18	127	126	17	**0.89**
					129	128	18	**1.88**
					131	130	17	**1.25**
**15**	ABI3BP $Q7Z7G0_1$: Target of Nesh-SH3	1/4	6	9	127	126	9	**1.38**
					129	128	9	**1.39**
					131	130	9	**0.82**

#	protein	group	peptides	spectra	ch1	ch2	quant	ratio
**16**	ACADVL P49748$_{1-3}$: Very long-chain specific acyl-CoA dehydrogenase, mitochondrial	3/3	3	3	127	126	3	**1.87**
					129	128	3	**1.45**
					131	130	3	**0.90**
**17**	ACLY P53396$_{1-3}$: ATP-citrate synthase	3/3	2	2	127	126	2	**1.17**
					129	128	2	**1.10**
					131	130	2	**1.08**
**18**	ACO1 P21399: Cytoplasmic aconitate hydratase		4	4	127	126	3	**1.77**
					129	128	4	**1.82**
					131	130	3	**0.90**
**19**	ACPP P15309$_{1,2}$: Prostatic acid phosphatase	2/2	3	3	127	126	3	**7.39**
					129	128	3	**1.02**
					131	130	3	**0.89**
**20**	ACTB P60709, ACTG1 P63261: Actin, cytoplasmic 1, Actin, cytoplasmic 2	2/3	21	250	127	126	72	**0.93**
					129	128	67	**1.72**
					131	130	73	**0.79**
**21**	ACTBL2 Q562R1: Beta-actin-like protein 2		8	102	127	126	2	**0.44**
					129	128	2	**0.77**
					131	130	2	**0.78**
**22**	ACTC1 P68032: Actin, alpha cardiac muscle 1	1/5	18	197	127	126	47	**0.84**
					129	128	53	**1.60**
					131	130	51	**0.81**
**23**	ACTN1 P12814$_{1,3}$: Alpha-actinin-1	2/4	25	29	127	126	10	**1.01**
					129	128	12	**2.05**
					131	130	11	**1.17**
**25**	ACTN3 Q08043: Alpha-actinin-3		5	5	127	126	2	**1.46**
					129	128	2	**1.69**
					131	130	2	**1.30**
**26**	ACTN4 O43707$_{1}$: Alpha-actinin-4	1/6	30	43	127	126	27	**1.74**
					129	128	28	**2.16**
					131	130	26	**1.32**
**27**	ACTR1A P61163: Alpha-centractin		4	33	127	126	5	**1.26**
					129	128	4	**1.93**
					131	130	5	**1.19**
**28**	ACTR2 P61160$_{1}$: Actin-related protein 2	1/2	6	34	127	126	6	**4.16**
					129	128	5	**2.25**
					131	130	6	**0.84**

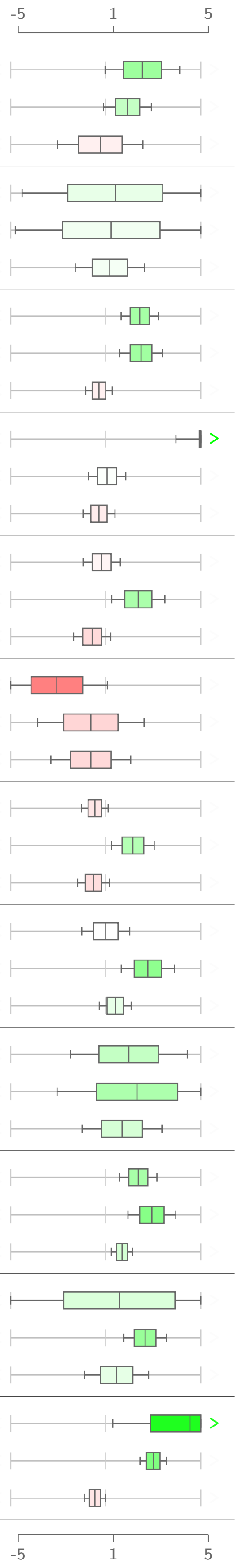

#	protein	group	peptides	spectra	ch1	ch2	quant	ratio
**29**	ACTR3 P61158: Actin-related protein 3		7	8	127	126	7	**5.17**
					129	128	7	**2.13**
					131	130	7	**0.96**
**30**	ADAM10 $O14672_1$: Disintegrin and metalloproteinase domain-containing protein 10		10	54	127	126	29	**0.52**
					129	128	21	**0.78**
					131	130	36	**0.89**
**31**	ADAM15 $Q13444_{1-10,12}$: Disintegrin and metalloproteinase domain-containing protein 15	11/13	5	9	127	126	8	**0.79**
					129	128	9	**1.01**
					131	130	8	**1.24**
**32**	ADAMDEC1 $O15204_1$: ADAM DEC1	1/2	9	9	127	126	9	**0.75**
					129	128	9	**1.11**
					131	130	9	**0.95**
**33**	ADAMTS1 Q9UHI8: A disintegrin and metalloproteinase with thrombospondin motifs 1		1	1	127	126	1	**0.61**
					129	128	1	**0.83**
					131	130	1	**0.94**
**34**	ADAMTS13 $Q76LX8_{1-3}$: A disintegrin and metalloproteinase with thrombospondin motifs 13	3/3	2	2	127	126	2	**0.96**
					129	128	2	**0.91**
					131	130	2	**0.89**
**35**	ADAMTS15 Q8TE58: A disintegrin and metalloproteinase with thrombospondin motifs 15		5	5	127	126	4	**0.58**
					129	128	5	**0.96**
					131	130	5	**0.83**
**36**	ADAMTS2 $O95450_{1,2}$: A disintegrin and metalloproteinase with thrombospondin motifs 2	2/2	6	9	127	126	5	**0.63**
					129	128	7	**0.97**
					131	130	7	**0.89**
**37**	ADAMTS5 Q9UNA0: A disintegrin and metalloproteinase with thrombospondin motifs 5		4	4	127	126	4	**0.58**
					129	128	4	**0.83**
					131	130	4	**0.94**
**38**	ADAMTS8 Q9UP79: A disintegrin and metalloproteinase with thrombospondin motifs 8		4	7	127	126	6	**0.67**
					129	128	5	**0.81**
					131	130	6	**0.88**
**39**	ADAMTS9 $Q9P2N4_{3,4}$: A disintegrin and metalloproteinase with thrombospondin motifs 9	2/4	7	9	127	126	8	**0.61**
					129	128	7	**0.82**
					131	130	9	**1.18**
**40**	ADAMTSL1 $Q8N6G6_3$: ADAMTS-like protein 1	1/2	6	8	127	126	4	**0.71**
					129	128	4	**1.07**
					131	130	5	**1.11**

#	protein	group	peptides	spectra	ch1	ch2	quant	ratio
**41**	ADAMTSL4 Q6UY14$_{1,3}$: ADAMTS-like protein 4	2/3	18	24	127	126	20	**0.68**
					129	128	14	**0.93**
					131	130	19	**0.87**
**42**	ADH1B P00325$_{1}$: Alcohol dehydrogenase 1B	1/4	7	7	127	126	6	**1.53**
					129	128	7	**2.42**
					131	130	7	**1.12**
**43**	ADH4 P08319$_{1,2}$: Alcohol dehydrogenase 4	2/2	4	4	127	126	4	**4.64**
					129	128	4	**0.98**
					131	130	4	**1.32**
**44**	ADH5 P11766: Alcohol dehydrogenase class-3		9	14	127	126	13	**1.32**
					129	128	13	**1.97**
					131	130	13	**1.74**
**45**	ADIPOQ Q15848: Adiponectin		5	10	127	126	10	**0.50**
					129	128	10	**0.82**
					131	130	9	**1.35**
**46**	AEBP1 Q8IUX7$_{1}$: Adipocyte enhancer-binding protein 1	1/2	38	80	127	126	70	**0.53**
					129	128	73	**0.80**
					131	130	73	**0.98**
**47**	AFM P43652: Afamin		30	89	127	126	87	**0.80**
					129	128	82	**0.99**
					131	130	88	**0.95**
**48**	AGRN O00468$_{1,3-7}$: Agrin [Cleaved into: Agrin N-terminal 110 kDa subunit; Agrin C-terminal 110 . . .	6/7	25	27	127	126	25	**1.39**
					129	128	25	**1.22**
					131	130	26	**0.70**
**49**	AGT P01019: Angiotensinogen		12	40	127	126	40	**1.86**
					129	128	38	**1.36**
					131	130	34	**0.44**
**50**	AHCY P23526$_{1}$: Adenosylhomocysteinase	1/2	5	5	127	126	5	**1.36**
					129	128	4	**2.10**
					131	130	5	**1.22**
**51**	AHCYL1 O43865$_{1}$: Adenosylhomocysteinase 2	1/2	4	4	127	126	4	**1.04**
					129	128	4	**2.48**
					131	130	4	**1.54**
**52**	AHNAK Q09666$_{1}$: Neuroblast differentiation-associated protein AHNAK		29	33	127	126	30	**2.85**
					129	128	28	**1.93**
					131	130	29	**1.33**

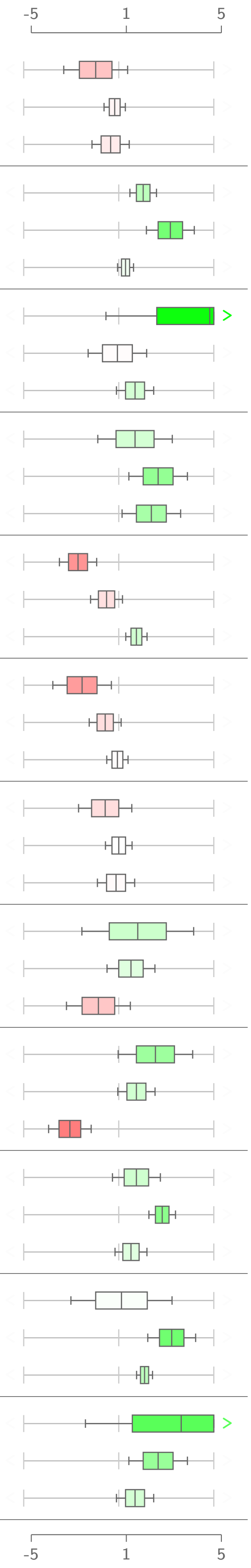

#	protein	group	peptides	spectra	ch1	ch2	quant	ratio
53	AHSA1 O95433$_1$: Activator of 90 kDa heat shock protein ATPase homolog 1	1/2	5	5	127	126	5	**1.64**
					129	128	5	**1.67**
					131	130	4	**2.06**
54	AHSG P02765: Alpha-2-HS-glycoprotein		13	386	127	126	367	**1.01**
					129	128	348	**1.24**
					131	130	350	**1.05**
55	AIFM1 O95831$_{1,3}$: Apoptosis-inducing factor 1, mitochondrial	2/4	2	2	127	126	2	**0.90**
					129	128	2	**1.22**
					131	130	2	**1.10**
56	AK1 P00568: Adenylate kinase isoenzyme 1		3	3	127	126	3	**1.74**
					129	128	3	**2.76**
					131	130	3	**0.84**
57	AKR1A1 P14550: Alcohol dehydrogenase [NADP(+)]		2	2	127	126	2	**2.21**
					129	128	2	**1.26**
					131	130	2	**1.07**
58	AKR7A2 O43488: Aflatoxin B1 aldehyde reductase member 2		5	7	127	126	7	**0.94**
					129	128	7	**1.51**
					131	130	6	**1.40**
59	ALB P02768$_1$: Serum albumin	1/3	71	3850	127	126	3231	**0.81**
					129	128	3190	**1.32**
					131	130	3342	**0.80**
60	ALDH1A1 P00352: Retinal dehydrogenase 1		4	6	127	126	3	**1.43**
					129	128	3	**1.36**
					131	130	3	**0.85**
61	ALDH1A2 O94788$_1$: Retinal dehydrogenase 2	1/5	10	13	127	126	8	**0.80**
					129	128	8	**2.38**
					131	130	8	**1.81**
62	ALDH1A3 P47895: Aldehyde dehydrogenase family 1 member A3		10	11	127	126	7	**1.38**
					129	128	6	**2.29**
					131	130	6	**1.59**
63	ALDH2 P05091$_{1,2}$: Aldehyde dehydrogenase, mitochondrial	2/2	4	5	127	126	2	**4.77**
					129	128	2	**1.49**
					131	130	2	**1.36**
64	ALDH3A1 P30838: Aldehyde dehydrogenase, dimeric NADP-preferring		3	3	127	126	3	**12.08**
					129	128	3	**1.81**
					131	130	3	**1.47**

#	protein	group	peptides	spectra	ch1	ch2	quant	ratio
65	ALDH9A1 P49189$_1$: 4-trimethylaminobutyraldehyde dehydrogenase	1/2	9	10	127	126	9	**1.28**
					129	128	7	**2.07**
					131	130	10	**1.60**
66	ALDOA P04075$_{1,2}$: Fructose-bisphosphate aldolase A	2/3	12	15	127	126	14	**6.16**
					129	128	15	**1.23**
					131	130	14	**1.20**
67	ALMS1 Q8TCU4$_{1-3}$: Alstrom syndrome protein 1	3/3	5	13	127	126	10	**0.69**
					129	128	10	**0.86**
					131	130	12	**0.80**
68	ALOX12B O75342: Arachidonate 12-lipoxygenase, 12R-type		4	4	127	126	4	**1.88**
					129	128	4	**0.78**
					131	130	4	**1.02**
69	ALOX15 P16050$_1$: Arachidonate 15-lipoxygenase	1/2	5	5	127	126	4	**1.14**
					129	128	4	**3.29**
					131	130	5	**1.45**
70	AMBP P02760: Protein AMBP [Cleaved into: Alpha-1-microglobulin		16	231	127	126	198	**1.42**
					129	128	190	**1.10**
					131	130	219	**0.75**
71	ANG P03950: Angiogenin		13	171	127	126	167	**0.46**
					129	128	165	**0.65**
					131	130	163	**0.91**
72	ANGPT2 O15123$_{1-3}$: Angiopoietin-2	3/3	1	2	127	126	2	**0.34**
					129	128	1	**1.20**
					131	130	2	**0.95**
73	ANGPTL1 O95841: Angiopoietin-related protein 1		5	5	127	126	4	**0.33**
					129	128	3	**1.04**
					131	130	5	**0.91**
74	ANGPTL2 Q9UKU9$_1$: Angiopoietin-related protein 2	1/2	14	20	127	126	20	**0.64**
					129	128	18	**0.90**
					131	130	19	**0.93**
75	ANGPTL3 Q9Y5C1: Angiopoietin-related protein 3		2	2	127	126	2	**0.48**
					129	128	2	**0.80**
					131	130	2	**1.38**
76	ANGPTL4 Q9BY76$_1$: Angiopoietin-related protein 4	1/3	8	12	127	126	12	**0.79**
					129	128	12	**0.89**
					131	130	9	**0.85**

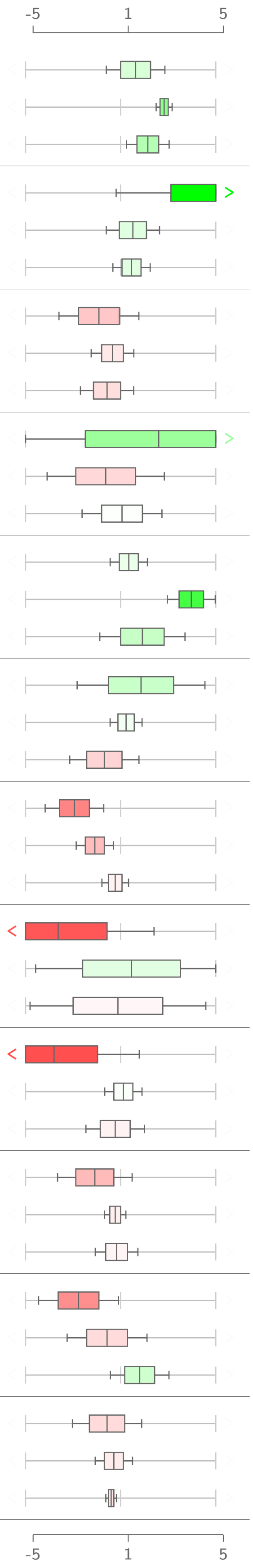

#	protein	group	peptides	spectra	ch1	ch2	quant	ratio
**77**	ANTXR1 Q9H6X2$_{1,5}$: Anthrax toxin receptor 1	2/6	9	13	127	126	10	**0.93**
					129	128	8	**1.01**
					131	130	10	**0.70**
**78**	ANTXR2 P58335$_{4}$: Anthrax toxin receptor 2	1/4	14	22	127	126	16	**1.54**
					129	128	16	**1.11**
					131	130	18	**0.61**
**79**	ANXA1 P04083: Annexin A1		14	20	127	126	20	**2.36**
					129	128	18	**2.00**
					131	130	19	**1.11**
**80**	ANXA11 P50995$_{1,2}$: Annexin A11	2/2	3	3	127	126	3	**1.76**
					129	128	3	**1.54**
					131	130	3	**1.50**
**81**	ANXA2 P07355$_{1,2}$: Annexin A2	2/3	16	26	127	126	23	**3.80**
					129	128	22	**1.74**
					131	130	20	**1.16**
**82**	ANXA3 P12429: Annexin A3		3	3	127	126	3	**1.47**
					129	128	3	**1.23**
					131	130	3	**1.22**
**83**	ANXA4 P09525$_{1}$: Annexin A4	1/2	5	6	127	126	3	**2.11**
					129	128	3	**1.21**
					131	130	3	**1.09**
**84**	ANXA5 P08758: Annexin A5		9	10	127	126	7	**2.21**
					129	128	7	**1.71**
					131	130	7	**1.15**
**85**	ANXA6 P08133$_{1,2}$: Annexin A6	2/2	4	5	127	126	4	**0.58**
					129	128	4	**1.02**
					131	130	5	**1.10**
**86**	ANXA7 P20073$_{1,2}$: Annexin A7	2/2	1	1	127	126	1	**1.13**
					129	128	1	**0.93**
					131	130	1	**0.99**
**87**	AP1B1 Q10567$_{3,4}$: AP-1 complex subunit beta-1	2/4	9	12	127	126	4	**1.51**
					129	128	4	**1.63**
					131	130	4	**1.09**
**88**	AP1G1 O43747$_{1,2}$: AP-1 complex subunit gamma-1	2/2	1	1	127	126	1	**1.67**
					129	128	1	**1.68**
					131	130	1	**0.86**

#	protein	group	peptides	spectra	ch1	ch2	quant	ratio
**89**	AP2A1 O95782$_{1,2}$: AP-2 complex subunit alpha-1	2/5	5	6	127	126	4	**1.13**
					129	128	4	**2.44**
					131	130	5	**1.04**
**90**	AP2B1 P63010$_{1,2}$: AP-2 complex subunit beta	2/3	11	13	127	126	4	**1.21**
					129	128	4	**3.24**
					131	130	5	**1.11**
**91**	AP2M1 Q96CW1$_{1,2}$: AP-2 complex subunit mu	2/2	4	4	127	126	4	**1.25**
					129	128	4	**2.00**
					131	130	4	**1.04**
**92**	APCS P02743: Serum amyloid P-component		9	248	127	126	224	**1.05**
					129	128	235	**0.92**
					131	130	245	**1.45**
**93**	API5 Q9BZZ5$_{1-6}$: Apoptosis inhibitor 5	6/6	2	2	127	126	2	**0.75**
					129	128	2	**1.24**
					131	130	2	**1.18**
**94**	APLP1 P51693$_{1,2}$: Amyloid-like protein 1	2/2	2	4	127	126	1	**0.50**
					129	128	1	**0.73**
					131	130	1	**1.00**
**95**	APLP2 Q06481$_{1,3,6}$: Amyloid-like protein 2	3/6	6	26	127	126	23	**1.10**
					129	128	23	**0.83**
					131	130	24	**1.25**
**96**	APMAP Q9HDC9$_{1}$: Adipocyte plasma membrane-associated protein	1/2	13	16	127	126	15	**1.36**
					129	128	16	**1.32**
					131	130	14	**0.88**
**97**	APOA1 P02647: Apolipoprotein A-I		35	2056	127	126	1799	**0.47**
					129	128	1793	**0.82**
					131	130	1777	**0.99**
**98**	APOA2 P02652: Apolipoprotein A-II		6	277	127	126	261	**0.60**
					129	128	253	**0.78**
					131	130	260	**0.66**
**99**	APOA4 P06727: Apolipoprotein A-IV		40	1695	127	126	1519	**0.35**
					129	128	1457	**0.84**
					131	130	1439	**0.80**
**100**	APOA5 Q6Q788: Apolipoprotein A-V		6	6	127	126	6	**1.58**
					129	128	6	**1.11**
					131	130	6	**1.47**

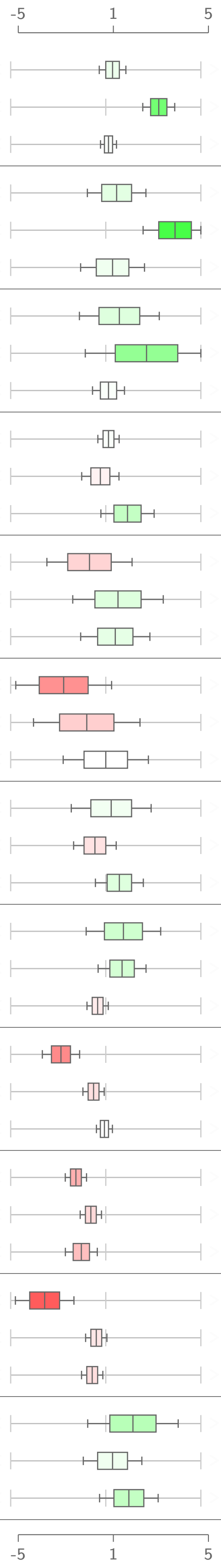

#	protein	group	peptides	spectra	ch1	ch2	quant	ratio
**101**	APOB P04114: Apolipoprotein B-100		272	2293	127	126	2205	**2.33**
					129	128	2152	**0.90**
					131	130	2213	**0.47**
**102**	APOBR Q0VD83$_{1,3,4}$: Apolipoprotein B receptor	3/3	4	4	127	126	3	**0.94**
					129	128	3	**0.84**
					131	130	4	**1.69**
**103**	APOC1 P02654: Apolipoprotein C-I		5	58	127	126	58	**0.43**
					129	128	53	**2.51**
					131	130	56	**0.69**
**104**	APOC2 P02655: Apolipoprotein C-II		3	30	127	126	27	**0.95**
					129	128	29	**1.87**
					131	130	27	**0.68**
**105**	APOC3 P02656: Apolipoprotein C-III		7	326	127	126	298	**0.60**
					129	128	283	**0.70**
					131	130	318	**0.52**
**106**	APOC4 P55056: Apolipoprotein C-IV		8	14	127	126	14	**1.64**
					129	128	10	**1.04**
					131	130	14	**0.81**
**107**	APOD P05090: Apolipoprotein D		10	260	127	126	250	**0.62**
					129	128	244	**0.70**
					131	130	235	**1.18**
**108**	APOE P02649: Apolipoprotein E		28	286	127	126	265	**0.70**
					129	128	257	**0.75**
					131	130	261	**1.10**
**109**	APOF Q13790: Apolipoprotein F		5	18	127	126	18	**1.02**
					129	128	17	**0.88**
					131	130	15	**0.93**
**110**	APOH P02749: Beta-2-glycoprotein 1		13	26	127	126	25	**0.92**
					129	128	25	**1.15**
					131	130	22	**1.26**
**111**	APOL1 O14791$_{1-3}$: Apolipoprotein L1	3/3	20	131	127	126	129	**2.72**
					129	128	124	**1.14**
					131	130	121	**0.71**
**112**	APOL2 Q9BQE5: Apolipoprotein L2		2	4	127	126	4	**2.11**
					129	128	3	**1.96**
					131	130	4	**0.92**

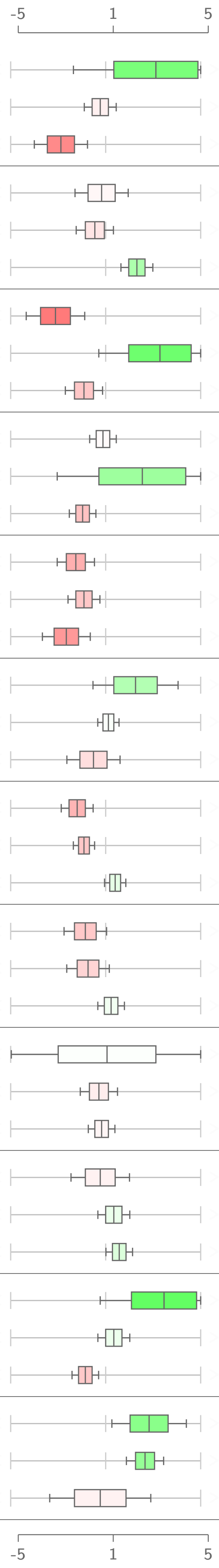

#	protein	group	peptides	spectra	ch1	ch2	quant	ratio
**113**	APOM O95445$_1$: Apolipoprotein M		12	39	127	126	2	**1.16**
					129	128	3	**1.19**
					131	130	4	**0.61**
**114**	APOM O95445$_2$: Apolipoprotein M		12	37	127	126	2	**1.05**
					129	128	2	**0.92**
					131	130	2	**0.86**
**115**	APP P05067$_{1,3-9,11}$: Amyloid beta A4 protein	9/11	8	13	127	126	9	**0.99**
					129	128	10	**0.73**
					131	130	6	**1.02**
**116**	APRT P07741$_{1,2}$: Adenine phosphoribosyltransferase	2/2	8	11	127	126	11	**1.19**
					129	128	10	**1.55**
					131	130	11	**1.33**
**117**	ARCN1 P48444$_{1,2}$: Coatomer subunit delta	2/2	9	12	127	126	12	**1.23**
					129	128	10	**2.44**
					131	130	11	**1.34**
**118**	ARF1 P84077, ARF3 P61204$_{1,2}$: ADP-ribosylation factor 1, ADP-ribosylation factor 3	3/3	2	5	127	126	3	**1.35**
					129	128	3	**1.79**
					131	130	3	**0.91**
**119**	ARF5 P84085: ADP-ribosylation factor 5		2	3	127	126	1	**1.13**
					129	128	1	**1.14**
					131	130	1	**0.91**
**120**	ARFGAP2 Q8N6H7$_{1,2}$: ADP-ribosylation factor GTPase-activating protein 2	2/2	3	3	127	126	3	**1.56**
					129	128	3	**1.32**
					131	130	3	**1.84**
**121**	ARG1 P05089$_1$: Arginase-1	1/3	9	10	127	126	10	**5.57**
					129	128	9	**0.99**
					131	130	10	**0.99**
**122**	ARHGAP1 Q07960: Rho GTPase-activating protein 1		3	3	127	126	3	**2.06**
					129	128	3	**2.47**
					131	130	3	**1.00**
**123**	ARHGAP18 Q8N392$_{1,2}$: Rho GTPase-activating protein 18	2/2	2	2	127	126	2	**1.06**
					129	128	2	**2.11**
					131	130	2	**1.31**
**124**	ARHGDIA P52565$_1$: Rho GDP-dissociation inhibitor 1	1/2	8	13	127	126	10	**2.33**
					129	128	11	**1.85**
					131	130	13	**0.95**

#	protein	group	peptides	spectra	ch1	ch2	quant	ratio
**125**	ARHGDIB P52566: Rho GDP-dissociation inhibitor 2		4	7	127	126	7	**1.85**
					129	128	7	**1.21**
					131	130	6	**1.07**
**126**	ARL3 P36405: ADP-ribosylation factor-like protein 3		2	2	127	126	2	**0.94**
					129	128	2	**2.07**
					131	130	2	**1.19**
**127**	ARPC2 O15144: Actin-related protein 2/3 complex subunit 2		6	6	127	126	3	**6.54**
					129	128	6	**2.12**
					131	130	6	**1.15**
**128**	ARPC4 $P59998_{1-3}$: Actin-related protein 2/3 complex subunit 4	3/3	2	2	127	126	2	**3.82**
					129	128	2	**1.63**
					131	130	2	**0.99**
**129**	ASAH1 $Q13510_{1-3}$: Acid ceramidase	3/3	3	3	127	126	3	**1.19**
					129	128	3	**1.54**
					131	130	3	**0.83**
**130**	ASGR2 $P07307_{1-3}$: Asialoglycoprotein receptor 2	3/3	3	7	127	126	7	**0.55**
					129	128	6	**0.82**
					131	130	6	**0.99**
**131**	ASPH $Q12797_{1,10}$: Aspartyl/asparaginyl beta-hydroxylase	2/2	9	10	127	126	9	**0.68**
					129	128	9	**1.11**
					131	130	9	**0.88**
**132**	ATG3 $Q9NT62_{1}$: Ubiquitin-like-conjugating enzyme ATG3	1/2	4	5	127	126	5	**0.79**
					129	128	4	**1.17**
					131	130	5	**1.29**
**133**	ATIC $P31939_{1,2}$: Bifunctional purine biosynthesis protein PURH [Cleaved into: Bifunctional pu ...	2/2	6	9	127	126	7	**1.52**
					129	128	8	**2.39**
					131	130	9	**1.47**
**134**	ATL3 Q6DD88: Atlastin-3		3	3	127	126	3	**3.11**
					129	128	3	**3.05**
					131	130	3	**1.53**
**135**	ATP2A2 $P16615_{1,4}$: Sarcoplasmic/endoplasmic reticulum calcium ATPase 2	2/8	6	6	127	126	5	**2.88**
					129	128	6	**1.36**
					131	130	6	**1.20**
**136**	ATP5A1 $P25705_{1,2}$: ATP synthase subunit alpha, mitochondrial	2/3	15	20	127	126	18	**2.11**
					129	128	18	**1.63**
					131	130	17	**0.96**

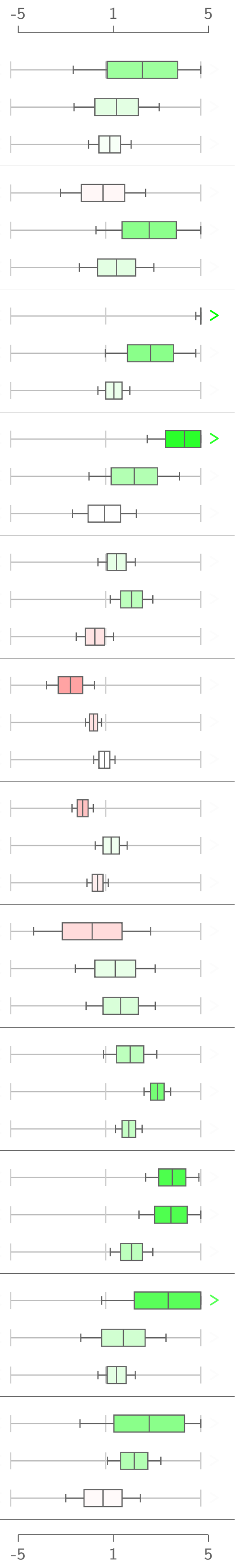

#	protein	group	peptides	spectra	ch1	ch2	quant	ratio
**137**	ATP5B P06576: ATP synthase subunit beta, mitochondrial		10	12	127	126	11	**2.37**
					129	128	11	**1.75**
					131	130	12	**1.22**
**138**	ATP6AP1 Q15904: V-type proton ATPase subunit S1		5	7	127	126	7	**0.78**
					129	128	6	**0.96**
					131	130	5	**1.02**
**139**	ATP6AP2 O75787$_{1,2}$: Renin receptor	2/2	10	13	127	126	11	**1.13**
					129	128	12	**1.18**
					131	130	12	**1.08**
**140**	ATP6V1A P38606$_{1,2}$: V-type proton ATPase catalytic subunit A	2/2	4	5	127	126	5	**1.62**
					129	128	3	**1.47**
					131	130	4	**0.97**
**141**	ATP6V1B2 P21281: V-type proton ATPase subunit B, brain isoform	1/2	3	3	127	126	2	**1.69**
					129	128	2	**1.69**
					131	130	2	**0.82**
**142**	ATP6V1E1 P36543$_{1,3}$: V-type proton ATPase subunit E 1	2/4	4	6	127	126	6	**0.95**
					129	128	4	**1.24**
					131	130	6	**1.07**
**143**	ATXN10 Q9UBB4$_{1,2}$: Ataxin-10	2/2	2	2	127	126	1	**1.15**
					129	128	2	**2.39**
					131	130	2	**1.24**
**144**	AZGP1 P25311: Zinc-alpha-2-glycoprotein		11	18	127	126	17	**1.81**
					129	128	15	**0.95**
					131	130	18	**1.00**
**145**	B2M P61769: Beta-2-microglobulin [Cleaved into: Beta-2-microglobulin form pI 5.3]		5	20	127	126	20	**1.01**
					129	128	20	**1.16**
					131	130	20	**0.79**
**146**	B3GAT3 O94766$_{1}$: Galactosylgalactosylxylosylprotein 3-beta-glucuronosyltransferase 3	1/2	7	7	127	126	6	**1.39**
					129	128	6	**1.39**
					131	130	5	**0.93**
**147**	B3GNT2 Q9NY97$_{1,2}$: N-acetyllactosaminide beta-1,3-N-acetylglucosaminyltransferase 2	2/2	1	1	127	126	1	**1.61**
					129	128	1	**1.21**
					131	130	1	**0.74**
**148**	B3GNT9 Q6UX72: UDP-GlcNAc:betaGal beta-1,3-N-acetylglucosaminyltransferase 9		2	2	127	126	2	**1.13**
					129	128	2	**1.11**
					131	130	2	**1.15**

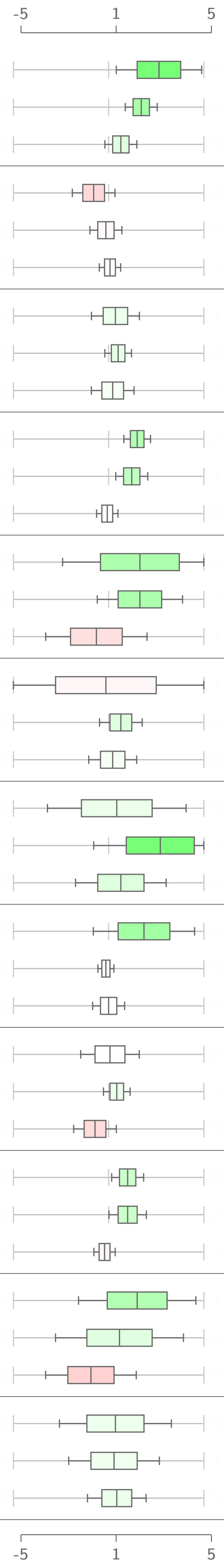

#	protein	group	peptides	spectra	ch1	ch2	quant	ratio
**149**	B4GALT1 P15291$_{1,2}$: Beta-1,4-galactosyltransferase 1	2/2	12	21	127	126	21	**1.68**
					129	128	19	**1.44**
					131	130	21	**0.81**
**150**	B4GALT4 O60513: Beta-1,4-galactosyltransferase 4		9	10	127	126	10	**1.47**
					129	128	10	**1.36**
					131	130	10	**0.82**
**151**	B4GAT1 O43505: Beta-1,4-glucuronyltransferase 1		6	8	127	126	8	**0.73**
					129	128	7	**0.98**
					131	130	8	**0.79**
**152**	BAX Q07812$_{1,2,4,8}$: Apoptosis regulator BAX	4/4	2	2	127	126	2	**1.66**
					129	128	2	**1.80**
					131	130	2	**0.85**
**153**	BCAP31 P51572$_{1,2}$: B-cell receptor-associated protein 31	2/2	3	4	127	126	2	**2.65**
					129	128	3	**1.53**
					131	130	4	**1.26**
**154**	BCHE P06276: Cholinesterase		2	2	127	126	2	**0.62**
					129	128	2	**1.10**
					131	130	2	**0.70**
**155**	BCL2L2 Q92843$_{2}$, PABPN1 Q86U42$_{1,2}$: Bcl-2-like protein 2, Polyadenylate-binding protein 2	3/3	3	3	127	126	3	**1.05**
					129	128	3	**2.37**
					131	130	3	**1.55**
**156**	BDH2 Q9BUT1$_{1}$: 3-hydroxybutyrate dehydrogenase type 2	1/2	3	3	127	126	3	**1.24**
					129	128	3	**1.95**
					131	130	3	**1.27**
**157**	BGN P21810: Biglycan		7	12	127	126	10	**1.63**
					129	128	9	**1.31**
					131	130	10	**0.86**
**158**	BLMH Q13867: Bleomycin hydrolase		9	11	127	126	9	**5.72**
					129	128	11	**1.08**
					131	130	11	**0.92**
**159**	BLVRA P53004: Biliverdin reductase A		3	3	127	126	3	**1.76**
					129	128	3	**2.26**
					131	130	3	**0.75**
**160**	BLVRB P30043: Flavin reductase		5	7	127	126	7	**1.19**
					129	128	7	**1.69**
					131	130	7	**0.83**

#	protein	group	peptides	spectra	ch1	ch2	quant	ratio
**161**	BMP1 P13497$_1$: Bone morphogenetic protein 1	1/6	17	22	127	126	14	**0.78**
					129	128	14	**0.89**
					131	130	14	**1.05**
**162**	BMP3 P12645: Bone morphogenetic protein 3		4	4	127	126	3	**1.00**
					129	128	4	**1.25**
					131	130	4	**1.45**
**163**	BOLA2 Q9H3K6$_{1,2}$: BolA-like protein 2	2/2	1	2	127	126	1	**0.69**
					129	128		
					131	130	2	**0.89**
**164**	BPIFB1 Q8TDL5$_1$: BPI fold-containing family B member 1	1/2	9	14	127	126	12	**1.45**
					129	128	11	**1.40**
					131	130	12	**1.13**
**165**	BPTF Q12830$_{1,2,4}$: Nucleosome-remodeling factor subunit BPTF	3/3	8	25	127	126	14	**1.00**
					129	128	15	**1.20**
					131	130	18	**0.85**
**166**	BTD P43251$_{1-4}$: Biotinidase	4/4	4	7	127	126	7	**0.71**
					129	128	7	**1.13**
					131	130	7	**0.81**
**167**	BTF3 P20290$_1$: Transcription factor BTF3	1/2	3	3	127	126	3	**3.58**
					129	128	3	**2.22**
					131	130	3	**1.57**
**168**	BZW1 Q7L1Q6$_{1-4}$: Basic leucine zipper and W2 domain-containing protein 1	4/4	4	4	127	126	3	**1.59**
					129	128	4	**1.79**
					131	130	4	**1.33**
**169**	C17orf58 Q2M2W7$_1$: UPF0450 protein C17orf58		2	2	127	126	2	**0.71**
					129	128	2	**1.16**
					131	130	2	**1.09**
**170**	C1QA P02745: Complement C1q subcomponent subunit A		3	17	127	126	11	**1.03**
					129	128	15	**0.83**
					131	130	16	**1.40**
**171**	C1QB P02746: Complement C1q subcomponent subunit B		7	61	127	126	58	**1.00**
					129	128	57	**0.81**
					131	130	60	**1.21**
**172**	C1QC P02747: Complement C1q subcomponent subunit C		5	14	127	126	14	**1.19**
					129	128	11	**0.88**
					131	130	14	**1.19**

#	protein	group	peptides	spectra	ch1	ch2	quant	ratio
**173**	C1QTNF4 Q9BXJ3: Complement C1q tumor necrosis factor-related protein 4		4	4	127	126	4	**0.49**
					129	128	4	**0.93**
					131	130	3	**1.11**
**174**	C1QTNF5 Q9BXJ0: Complement C1q tumor necrosis factor-related protein 5		3	3	127	126	3	**0.39**
					129	128	3	**0.86**
					131	130	3	**0.88**
**175**	C1R P00736: Complement C1r subcomponent		33	121	127	126	107	**0.56**
					129	128	107	**0.85**
					131	130	104	**0.98**
**176**	C1RL Q9NZP8: Complement C1r subcomponent-like protein		11	28	127	126	18	**1.56**
					129	128	16	**1.22**
					131	130	18	**0.81**
**177**	C1S P09871: Complement C1s subcomponent		27	146	127	126	119	**0.45**
					129	128	107	**0.83**
					131	130	116	**0.99**
**178**	C2 P06681$_1$: Complement C2	1/3	27	42	127	126	40	**1.70**
					129	128	41	**1.17**
					131	130	41	**0.76**
**179**	C2orf40 Q9H1Z8: Augurin		2	4	127	126	4	**0.60**
					129	128	3	**0.60**
					131	130	4	**0.74**
**180**	C3 P01024: Complement C3		130	3198	127	126	2864	**1.07**
					129	128	2775	**0.98**
					131	130	2880	**0.78**
**181**	C4A P0C0L4$_1$: Complement C4-A	1/2	87	1916	127	126	13	**0.87**
					129	128	14	**0.80**
					131	130	17	**0.94**
**182**	C4B P0C0L5: Complement C4-B		88	2027	127	126	117	**0.80**
					129	128	124	**0.88**
					131	130	122	**1.02**
**183**	C4BPA P04003: C4b-binding protein alpha chain		32	586	127	126	562	**0.89**
					129	128	565	**0.52**
					131	130	576	**1.52**
**184**	C4BPB P20851$_{1,2}$: C4b-binding protein beta chain	2/2	12	47	127	126	39	**0.86**
					129	128	43	**0.57**
					131	130	45	**1.35**

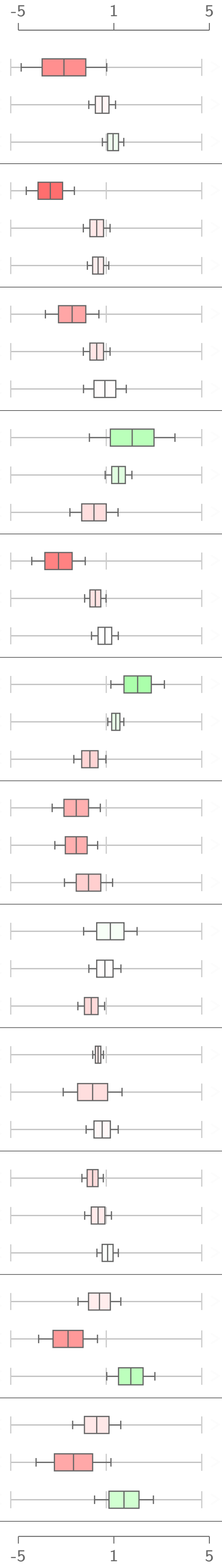

#	protein	group	peptides	spectra	ch1	ch2	quant	ratio
185	C5 P01031: Complement C5		73	300	127	126	274	**1.11**
					129	128	261	**0.85**
					131	130	281	**0.80**
186	C6 P13671: Complement component C6		45	103	127	126	90	**1.49**
					129	128	85	**1.19**
					131	130	78	**1.07**
187	C7 P10643: Complement component C7		42	118	127	126	111	**1.53**
					129	128	107	**1.24**
					131	130	111	**0.79**
188	C8A P07357: Complement component C8 alpha chain		23	88	127	126	76	**1.10**
					129	128	72	**1.19**
					131	130	81	**0.94**
189	C8B P07358: Complement component C8 beta chain		29	136	127	126	128	**1.69**
					129	128	129	**1.14**
					131	130	134	**0.68**
190	C8G P07360: Complement component C8 gamma chain		13	54	127	126	54	**1.52**
					129	128	54	**1.10**
					131	130	54	**0.83**
191	C9 P02748: Complement component C9 [Cleaved into: Complement component C9a; Complement . . .	1/4	33	899	127	126	770	**2.07**
					129	128	783	**1.39**
					131	130	792	**0.93**
192	CAB39 Q9Y376: Calcium-binding protein 39		2	3	127	126	3	**0.91**
					129	128	3	**1.72**
					131	130	3	**0.74**
193	CACNA1D $Q01668_2$: Voltage-dependent L-type calcium channel subunit alpha-1D	1/4	4	108	127	126	62	**0.92**
					129	128	78	**1.08**
					131	130	91	**0.86**
194	CACYBP $Q9HB71_{1,2}$: Calcyclin-binding protein	2/2	2	150	127	126	1	**1.50**
					129	128	1	**2.34**
					131	130	1	**0.90**
195	CAD P27708: CAD protein [Includes: Glutamine-dependent carbamoyl-phosphate synthase		2	2	127	126	2	**1.16**
					129	128	2	**2.24**
					131	130	2	**1.18**
196	CALD1 $Q05682_1$: Caldesmon	1/6	4	4	127	126	3	**0.67**
					129	128	2	**1.81**
					131	130	2	**1.27**

#	protein	group	peptides	spectra	ch1	ch2	quant	ratio
**197**	CALM1 P62158: Calmodulin		10	28	127	126	21	**0.64**
					129	128	22	**1.23**
					131	130	22	**0.94**
**198**	CALML3 P27482: Calmodulin-like protein 3		8	17	127	126	12	**3.35**
					129	128	12	**0.81**
					131	130	12	**1.12**
**199**	CALML5 Q9NZT1: Calmodulin-like protein 5		5	9	127	126	9	**12.51**
					129	128	8	**0.72**
					131	130	7	**1.09**
**200**	CALR P27797: Calreticulin		20	48	127	126	44	**0.79**
					129	128	45	**0.81**
					131	130	44	**1.33**
**201**	CALU $O43852_{1,3}$: Calumenin	2/8	6	6	127	126	1	**0.46**
					129	128	1	**0.69**
					131	130	1	**0.97**
**202**	CALU $O43852_{2,4}$: Calumenin	2/7	6	6	127	126	1	**0.64**
					129	128	1	**0.93**
					131	130	1	**1.00**
**203**	CAMK2D $Q13557_{1,3-6,8-12}$: Calcium/calmodulin-dependent protein kinase type II subunit delta	10/10	2	2	127	126	1	**1.28**
					129	128	1	**1.87**
					131	130	1	**1.35**
**204**	CAMP P49913: Cathelicidin antimicrobial peptide		3	3	127	126	3	**3.92**
					129	128	3	**1.20**
					131	130	3	**0.80**
**205**	CAND1 $Q86VP6_{1}$: Cullin-associated NEDD8-dissociated protein 1	1/3	19	25	127	126	23	**1.22**
					129	128	23	**1.84**
					131	130	21	**1.03**
**206**	CANX $P27824_{1,2}$: Calnexin	2/3	12	14	127	126	12	**1.38**
					129	128	11	**1.10**
					131	130	11	**1.48**
**207**	CAP1 $Q01518_{1,2}$: Adenylyl cyclase-associated protein 1	2/2	16	18	127	126	18	**3.05**
					129	128	17	**2.73**
					131	130	16	**1.36**
**208**	CAPG $P40121_{1,2}$: Macrophage-capping protein	2/2	5	13	127	126	11	**2.07**
					129	128	11	**1.93**
					131	130	13	**0.81**

#	protein	group	peptides	spectra	ch1	ch2	quant	ratio
**209**	CAPN1 P07384: Calpain-1 catalytic subunit		7	8	127	126	5	**1.80**
					129	128	5	**1.61**
					131	130	5	**1.14**
**210**	CAPN2 $P17655_1$: Calpain-2 catalytic subunit	1/2	10	14	127	126	10	**0.92**
					129	128	10	**1.73**
					131	130	12	**1.23**
**211**	CAPNS1 P04632: Calpain small subunit 1		6	11	127	126	10	**1.14**
					129	128	11	**1.79**
					131	130	11	**1.29**
**212**	CAPZA1 P52907: F-actin-capping protein subunit alpha-1		8	11	127	126	8	**1.44**
					129	128	8	**1.51**
					131	130	5	**1.19**
**213**	CAPZA2 $P47755_1$: F-actin-capping protein subunit alpha-2	1/2	5	6	127	126	3	**1.12**
					129	128	3	**1.44**
					131	130	3	**0.99**
**214**	CAPZB $P47756_2$: F-actin-capping protein subunit beta	1/2	7	9	127	126	9	**1.82**
					129	128	8	**2.37**
					131	130	6	**1.20**
**215**	CASP14 P31944: Caspase-14		11	16	127	126	16	**3.75**
					129	128	16	**0.92**
					131	130	15	**1.13**
**216**	CAST $P20810_{5,6,10}$: Calpastatin	3/10	8	8	127	126	7	**1.77**
					129	128	6	**2.01**
					131	130	7	**1.29**
**217**	CAT P04040: Catalase		2	2	127	126	2	**1.66**
					129	128	2	**1.13**
					131	130	2	**1.19**
**218**	CAV1 $Q03135_{1,2}$: Caveolin-1	2/2	2	3	127	126	3	**1.16**
					129	128	3	**1.60**
					131	130	3	**1.02**
**219**	CBR1 $P16152_1$: Carbonyl reductase [NADPH] 1	1/2	10	15	127	126	15	**2.03**
					129	128	15	**1.52**
					131	130	14	**0.82**
**220**	CBX3 Q13185: Chromobox protein homolog 3		1	1	127	126	1	**2.39**
					129	128	1	**2.75**
					131	130	1	**1.68**

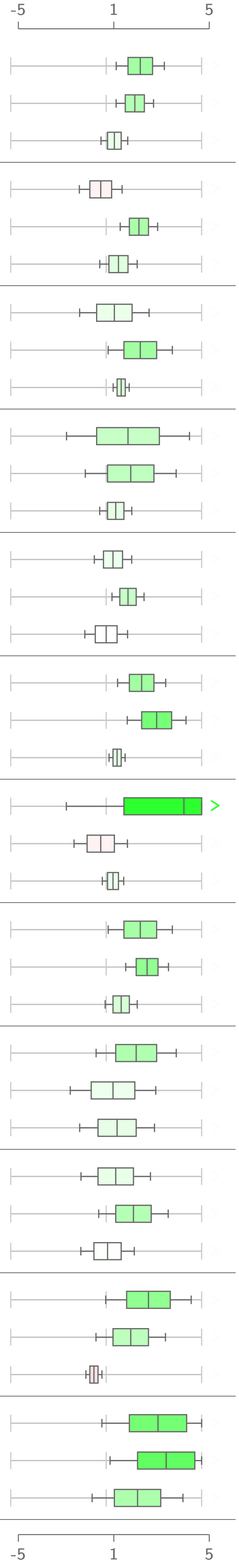

#	protein	group	peptides	spectra	ch1	ch2	quant	ratio
221	CCAR2 Q8N163$_{1,2}$: Cell cycle and apoptosis regulator protein 2	2/2	3	3	127	126	3	**1.95**
					129	128	3	**2.48**
					131	130	3	**2.57**
222	CCDC146 Q8IYE0$_{1}$: Coiled-coil domain-containing protein 146	1/2	8	8	127	126	4	**0.13**
					129	128	2	**0.91**
					131	130	3	**1.04**
223	CCDC57 Q2TAC2$_{1,2}$: Coiled-coil domain-containing protein 57	2/2	2	3	127	126	1	**0.74**
					129	128	1	**1.77**
					131	130	1	**1.68**
224	CCDC80 Q76M96$_{1,2}$: Coiled-coil domain-containing protein 80	2/2	24	43	127	126	40	**0.80**
					129	128	41	**0.95**
					131	130	39	**0.94**
225	CCER2 I3L3R5: Coiled-coil domain-containing glutamate-rich protein 2		1	1	127	126	1	**0.33**
					129	128	1	**0.62**
					131	130	1	**0.74**
226	CCL14 Q16627$_{1}$: C-C motif chemokine 14	1/2	3	3	127	126	3	**0.31**
					129	128	3	**0.73**
					131	130	3	**0.47**
227	CCL18 P55774: C-C motif chemokine 18		2	3	127	126	3	**0.20**
					129	128	3	**1.46**
					131	130	3	**0.72**
228	CCT2 P78371$_{1}$: T-complex protein 1 subunit beta	1/2	7	7	127	126	7	**0.76**
					129	128	6	**2.04**
					131	130	7	**1.15**
229	CCT3 P49368$_{1,2}$: T-complex protein 1 subunit gamma	2/2	10	10	127	126	10	**2.18**
					129	128	10	**1.53**
					131	130	10	**1.14**
230	CCT4 P50991$_{1,2}$: T-complex protein 1 subunit delta	2/2	6	6	127	126	5	**3.01**
					129	128	6	**2.05**
					131	130	6	**1.24**
231	CCT5 P48643$_{1}$: T-complex protein 1 subunit epsilon	1/2	10	11	127	126	10	**2.03**
					129	128	9	**2.03**
					131	130	10	**1.15**
232	CCT6A P40227$_{1,2}$: T-complex protein 1 subunit zeta	2/5	8	8	127	126	7	**1.92**
					129	128	8	**2.37**
					131	130	8	**1.15**

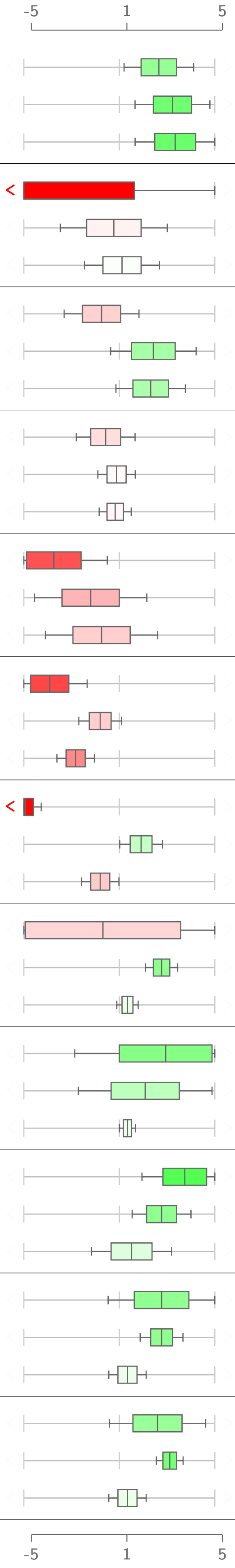

#	protein	group	peptides	spectra	ch1	ch2	quant	ratio
**233**	CCT7 Q99832$_1$: T-complex protein 1 subunit eta	1/4	9	9	127	126	8	**2.31**
					129	128	9	**2.11**
					131	130	9	**1.12**
**234**	CCT8 P50990$_{1,2}$: T-complex protein 1 subunit theta	2/3	14	15	127	126	15	**1.90**
					129	128	15	**1.99**
					131	130	15	**1.14**
**235**	CD109 Q6YHK3$_1$: CD109 antigen	1/4	13	15	127	126	14	**0.76**
					129	128	14	**1.16**
					131	130	14	**0.73**
**236**	CD14 P08571: Monocyte differentiation antigen CD14		15	229	127	126	201	**2.03**
					129	128	213	**1.37**
					131	130	207	**0.88**
**237**	CD163 Q86VB7$_{1-3}$: Scavenger receptor cysteine-rich type 1 protein M130	3/4	14	16	127	126	14	**0.55**
					129	128	14	**0.85**
					131	130	15	**0.89**
**238**	CD248 Q9HCU0$_1$: Endosialin		10	13	127	126	10	**0.77**
					129	128	10	**0.67**
					131	130	12	**1.18**
**239**	CD5L O43866: CD5 antigen-like		19	56	127	126	51	**0.86**
					129	128	54	**0.67**
					131	130	55	**1.07**
**240**	CD74 P04233$_{1,2}$: HLA class II histocompatibility antigen gamma chain	2/2	2	4	127	126	4	**0.76**
					129	128	3	**0.90**
					131	130	4	**1.08**
**241**	CD81 P60033: CD81 antigen		2	2	127	126	2	**1.64**
					129	128	2	**1.20**
					131	130	2	**0.59**
**242**	CD8A P01732$_{1-3}$: T-cell surface glycoprotein CD8 alpha chain	3/3	1	1	127	126	1	**1.37**
					129	128	1	**1.03**
					131	130	1	**0.72**
**243**	CD97 P48960$_{1-3}$: CD97 antigen	3/3	6	6	127	126	6	**0.72**
					129	128	5	**0.79**
					131	130	6	**0.94**
**244**	CD99 P14209$_{1,3}$: CD99 antigen	2/2	2	3	127	126	3	**1.16**
					129	128	3	**1.11**
					131	130	3	**1.08**

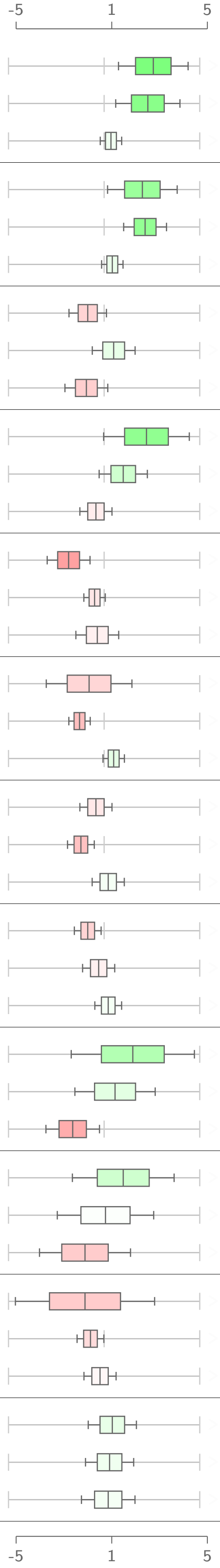

#	protein	group	peptides	spectra	ch1	ch2	quant	ratio
**245**	CDC37 Q16543: Hsp90 co-chaperone Cdc37		4	4	127	126	4	**0.95**
					129	128	4	**1.73**
					131	130	3	**1.02**
**246**	CDC42 P60953$_2$: Cell division control protein 42 homolog	1/2	4	7	127	126	6	**1.74**
					129	128	6	**1.50**
					131	130	5	**1.10**
**247**	CDH1 P12830$_{1,2}$: Cadherin-1	2/2	3	9	127	126	7	**0.65**
					129	128	8	**0.66**
					131	130	8	**1.13**
**248**	CDH2 P19022$_1$: Cadherin-2	1/2	6	7	127	126	7	**1.05**
					129	128	7	**1.17**
					131	130	7	**1.18**
**249**	CDSN Q15517: Corneodesmosin		1	1	127	126	1	**6.49**
					129	128	1	**0.60**
					131	130	1	**0.92**
**250**	CELF2 O95319$_{1-5}$: CUGBP Elav-like family member 2	5/11	3	3	127	126	3	**1.13**
					129	128	3	**2.85**
					131	130	3	**1.82**
**251**	CELSR2 Q9HCU4: Cadherin EGF LAG seven-pass G-type receptor 2		3	3	127	126	3	**0.52**
					129	128	3	**0.95**
					131	130	3	**0.91**
**252**	CENPF P49454: Centromere protein F		2	2	127	126	1	**1.30**
					129	128	1	**1.32**
					131	130	1	**1.02**
**253**	CENPK Q9BS16: Centromere protein K		3	26	127	126	15	**0.50**
					129	128	14	**0.87**
					131	130	18	**1.11**
**254**	CEP350 Q5VT06: Centrosome-associated protein 350		3	30	127	126	3	**0.72**
					129	128	3	**0.95**
					131	130	3	**1.17**
**255**	CETP P11597$_1$: Cholesteryl ester transfer protein	1/2	17	97	127	126	95	**1.49**
					129	128	88	**1.02**
					131	130	91	**1.95**
**256**	CFB P00751$_1$: Complement factor B	1/2	29	49	127	126	49	**1.22**
					129	128	47	**1.22**
					131	130	47	**0.98**

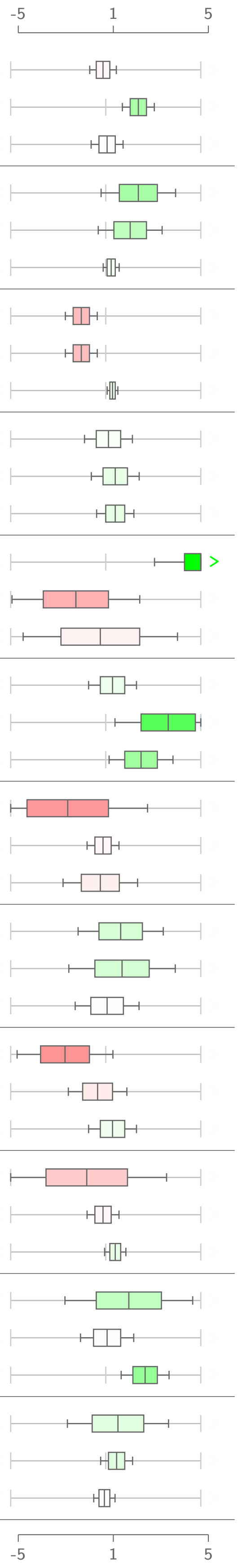

#	protein	group	peptides	spectra	ch1	ch2	quant	ratio
**257**	CFD P00746: Complement factor D		17	124	127	126	123	**1.89**
					129	128	120	**1.30**
					131	130	123	**0.79**
**258**	CFH P08603$_1$: Complement factor H	1/2	67	657	127	126	486	**0.64**
					129	128	486	**0.71**
					131	130	490	**1.23**
**259**	CFHR1 Q03591: Complement factor H-related protein 1		13	233	127	126	15	**1.03**
					129	128	14	**0.71**
					131	130	12	**1.32**
**260**	CFHR2 P36980$_1$: Complement factor H-related protein 2	1/2	12	81	127	126	11	**0.61**
					129	128	9	**0.90**
					131	130	11	**1.02**
**261**	CFHR3 Q02985$_{1,2}$: Complement factor H-related protein 3	2/2	9	46	127	126	6	**0.89**
					129	128	7	**1.12**
					131	130	5	**1.15**
**262**	CFHR4 Q92496$_{1,2}$: Complement factor H-related protein 4	2/3	10	29	127	126	8	**1.15**
					129	128	8	**0.65**
					131	130	7	**1.25**
**263**	CFHR5 Q9BXR6: Complement factor H-related protein 5		21	73	127	126	35	**0.96**
					129	128	36	**0.79**
					131	130	37	**1.38**
**264**	CFI P05156: Complement factor I		22	42	127	126	35	**0.73**
					129	128	34	**1.05**
					131	130	37	**0.99**
**265**	CFL1 P23528: Cofilin-1	1/3	9	12	127	126	11	**3.03**
					129	128	11	**1.40**
					131	130	11	**1.14**
**266**	CFP P27918: Properdin		14	21	127	126	19	**1.22**
					129	128	18	**0.69**
					131	130	18	**1.21**
**267**	CGREF1 Q99674$_{1,4,5}$: Cell growth regulator with EF hand domain protein 1	3/3	7	10	127	126	8	**0.40**
					129	128	8	**0.88**
					131	130	9	**0.84**
**268**	CHD9 Q3L8U1$_{1-3}$: Chromodomain-helicase-DNA-binding protein 9	3/3	2	2	127	126	1	**0.01**
					129	128		
					131	130	1	**1.37**

#	protein	group	peptides	spectra	ch1	ch2	quant	ratio
269	CHGA P10645: Chromogranin-A		30	167	127	126	165	**0.65**
					129	128	150	**0.82**
					131	130	156	**1.05**
270	CHGB P05060: Secretogranin-1		22	52	127	126	39	**0.45**
					129	128	35	**0.77**
					131	130	38	**0.90**
271	CHMP6 Q96FZ7: Charged multivesicular body protein 6		2	2	127	126	2	**1.14**
					129	128	2	**0.98**
					131	130	2	**0.49**
272	CHRD Q9H2X0$_{1,5}$: Chordin	2/5	11	11	127	126	10	**0.58**
					129	128	11	**0.78**
					131	130	7	**0.77**
273	CHRDL1 Q9BU40$_{2,4}$: Chordin-like protein 1	2/6	21	37	127	126	36	**0.59**
					129	128	31	**0.99**
					131	130	33	**1.06**
274	CHRDL2 Q6WN34$_{1}$: Chordin-like protein 2	1/2	7	7	127	126	7	**1.06**
					129	128	7	**0.94**
					131	130	7	**0.88**
275	CHST12 Q9NRB3: Carbohydrate sulfotransferase 12		1	1	127	126	1	**0.86**
					129	128	1	**0.89**
					131	130	1	**1.20**
276	CHST14 Q8NCH0: Carbohydrate sulfotransferase 14		3	5	127	126	5	**0.84**
					129	128	5	**1.17**
					131	130	5	**1.10**
277	CHST15 Q7LFX5$_{1}$: Carbohydrate sulfotransferase 15	1/2	6	8	127	126	8	**1.33**
					129	128	8	**1.37**
					131	130	7	**0.95**
278	CILP O75339: Cartilage intermediate layer protein 1		51	119	127	126	111	**0.56**
					129	128	111	**0.94**
					131	130	111	**0.94**
279	CILP2 Q8IUL8: Cartilage intermediate layer protein 2		4	6	127	126	4	**0.21**
					129	128	4	**0.81**
					131	130	4	**0.90**
280	CIRBP Q14011$_{1}$: Cold-inducible RNA-binding protein	1/2	4	4	127	126	4	**1.04**
					129	128	4	**1.77**
					131	130	4	**1.10**

#	protein	group	peptides	spectra	ch1	ch2	quant	ratio
281	CKAP4 Q07065: Cytoskeleton-associated protein 4		5	8	127	126	4	**1.17**
					129	128	3	**1.30**
					131	130	4	**0.95**
282	CKB P12277: Creatine kinase B-type		10	11	127	126	9	**0.88**
					129	128	9	**1.19**
					131	130	8	**1.26**
283	CKMT1A P12532$_{1,2}$: Creatine kinase U-type, mitochondrial	2/3	7	7	127	126	5	**0.96**
					129	128	4	**0.97**
					131	130	5	**0.72**
284	CLC Q05315: Galectin-10		3	3	127	126	3	**1.07**
					129	128	3	**1.41**
					131	130	3	**1.50**
285	CLEC11A Q9Y240: C-type lectin domain family 11 member A		14	28	127	126	26	**0.74**
					129	128	25	**0.71**
					131	130	23	**1.01**
286	CLEC3B P05452: Tetranectin		7	9	127	126	7	**0.59**
					129	128	8	**1.00**
					131	130	9	**1.01**
287	CLIC1 O00299: Chloride intracellular channel protein 1		9	14	127	126	11	**1.06**
					129	128	12	**1.33**
					131	130	10	**1.25**
288	CLIC3 O95833: Chloride intracellular channel protein 3		4	5	127	126	5	**1.50**
					129	128	4	**1.31**
					131	130	5	**1.24**
289	CLIC4 Q9Y696: Chloride intracellular channel protein 4		6	7	127	126	6	**1.03**
					129	128	4	**1.51**
					131	130	6	**1.26**
290	CLSTN1 O94985$_1$: Calsyntenin-1		16	17	127	126	1	**0.74**
					129	128	1	**1.08**
					131	130	1	**0.79**
291	CLSTN1 O94985$_2$: Calsyntenin-1		16	17	127	126	1	**0.48**
					129	128	1	**1.04**
					131	130	1	**0.94**
292	CLSTN3 Q9BQT9$_{1,2}$: Calsyntenin-3	2/2	5	6	127	126	6	**0.67**
					129	128	6	**0.99**
					131	130	6	**0.81**

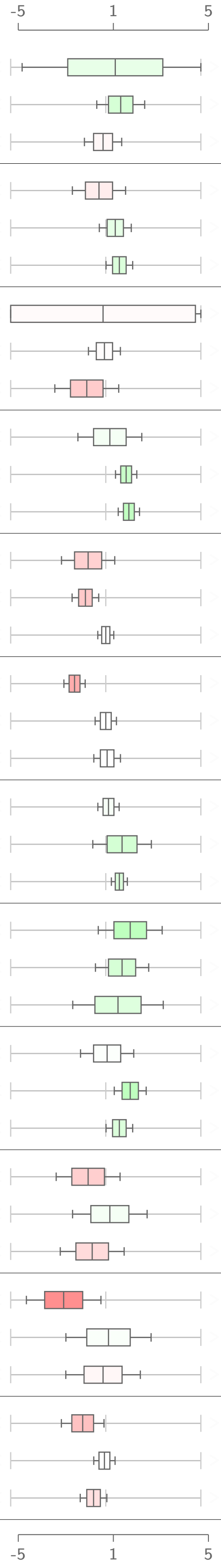

#	protein	group	peptides	spectra	ch1	ch2	quant	ratio
**293**	CLTA P09496$_{1-4}$: Clathrin light chain A	4/4	2	2	127	126	2	**0.69**
					129	128	2	**1.94**
					131	130	2	**1.30**
**294**	CLTB P09497$_{2}$: Clathrin light chain B	1/2	4	4	127	126	4	**1.42**
					129	128	4	**1.62**
					131	130	3	**1.26**
**295**	CLTC Q00610$_{1,2}$: Clathrin heavy chain 1	2/2	29	31	127	126	23	**1.72**
					129	128	23	**2.75**
					131	130	23	**1.15**
**296**	CLTCL1 P53675$_{1}$: Clathrin heavy chain 2	1/2	10	10	127	126	3	**0.77**
					129	128	3	**1.00**
					131	130	3	**1.30**
**297**	CLU P10909$_{4}$: Clusterin	1/5	29	781	127	126	719	**0.63**
					129	128	724	**0.75**
					131	130	745	**1.34**
**298**	CNDP1 Q96KN2: Beta-Ala-His dipeptidase		6	7	127	126	7	**0.47**
					129	128	5	**0.88**
					131	130	6	**0.95**
**299**	CNN2 Q99439$_{1}$: Calponin-2	1/2	4	4	127	126	3	**1.81**
					129	128	3	**1.68**
					131	130	3	**1.96**
**300**	CNN3 Q15417$_{1,3}$: Calponin-3	2/5	9	9	127	126	6	**0.96**
					129	128	7	**2.33**
					131	130	8	**2.43**
**301**	CNPY2 Q9Y2B0$_{1}$: Protein canopy homolog 2		1	1	127	126	1	**0.56**
					129	128	1	**0.60**
					131	130	1	**0.99**
**302**	COL12A1 Q99715$_{1}$: Collagen alpha-1(XII) chain	1/3	58	79	127	126	72	**1.01**
					129	128	77	**1.14**
					131	130	75	**0.70**
**303**	COL14A1 Q05707$_{1,2}$: Collagen alpha-1(XIV) chain	2/3	41	63	127	126	57	**0.85**
					129	128	56	**1.04**
					131	130	57	**0.64**
**304**	COL15A1 P39059: Collagen alpha-1(XV) chain [Cleaved into: Restin		5	5	127	126	5	**1.29**
					129	128	5	**1.25**
					131	130	5	**0.86**

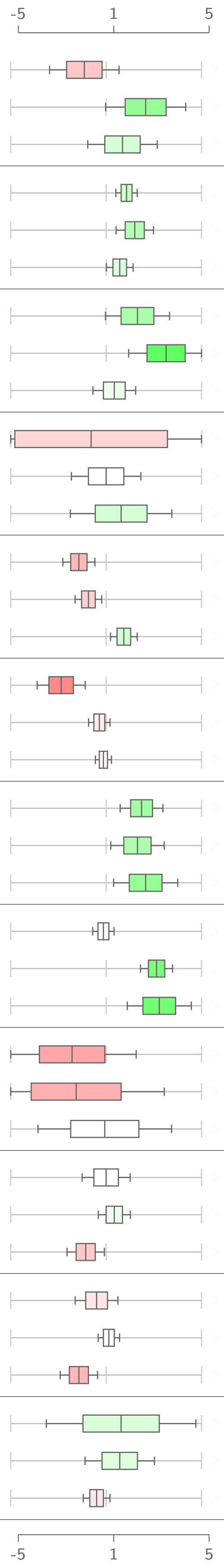

#	protein	group	peptides	spectra	ch1	ch2	quant	ratio
**305**	COL16A1 Q07092$_{1,2}$: Collagen alpha-1(XVI) chain	2/2	3	5	127	126	3	**1.02**
					129	128	3	**0.84**
					131	130	4	**0.90**
**306**	COL18A1 P39060$_{1-3}$: Collagen alpha-1(XVIII) chain [Cleaved into: Endostatin]	3/3	13	27	127	126	26	**0.67**
					129	128	26	**1.19**
					131	130	26	**0.86**
**307**	COL1A1 P02452: Collagen alpha-1(I) chain		25	86	127	126	59	**0.39**
					129	128	61	**0.86**
					131	130	58	**0.87**
**308**	COL1A2 P08123: Collagen alpha-2(I) chain		22	37	127	126	36	**0.51**
					129	128	32	**0.84**
					131	130	37	**0.82**
**309**	COL2A1 P02458$_2$: Collagen alpha-1(II) chain		2	25	127	126	1	**0.91**
					129	128	1	**1.14**
					131	130	1	**1.08**
**310**	COL3A1 P02461$_1$: Collagen alpha-1(III) chain	1/2	17	33	127	126	23	**0.84**
					129	128	26	**1.11**
					131	130	26	**0.85**
**311**	COL4A1 P02462$_{1,2}$: Collagen alpha-1(IV) chain [Cleaved into: Arresten]	2/2	1	1	127	126	1	**0.95**
					129	128	1	**1.00**
					131	130	1	**1.26**
**312**	COL4A3BP Q9Y5P4$_{1-3}$: Collagen type IV alpha-3-binding protein	3/3	2	2	127	126	2	**0.74**
					129	128	2	**1.54**
					131	130	2	**1.04**
**313**	COL5A1 P20908: Collagen alpha-1(V) chain		11	17	127	126	16	**0.59**
					129	128	17	**0.80**
					131	130	15	**1.01**
**314**	COL5A2 P05997: Collagen alpha-2(V) chain		15	25	127	126	18	**0.42**
					129	128	19	**0.70**
					131	130	16	**0.96**
**315**	COL6A1 P12109: Collagen alpha-1(VI) chain		30	79	127	126	74	**1.55**
					129	128	76	**1.44**
					131	130	76	**0.65**
**316**	COL6A2 P12110$_1$: Collagen alpha-2(VI) chain	1/2	24	141	127	126	11	**1.06**
					129	128	11	**1.10**
					131	130	11	**0.63**

#	protein	group	peptides	spectra	ch1	ch2	quant	ratio
**317**	COL6A2 P12110$_2$: Collagen alpha-2(VI) chain		18	130	127	126	1	**0.70**
					129	128	1	**1.10**
					131	130	1	**0.65**
**318**	COL6A3 P12111$_1$: Collagen alpha-3(VI) chain	1/4	122	307	127	126	22	**1.21**
					129	128	23	**1.28**
					131	130	23	**0.64**
**319**	COL6A3 P12111$_4$: Collagen alpha-3(VI) chain		106	292	127	126	9	**1.40**
					129	128	9	**1.49**
					131	130	9	**0.54**
**320**	COL6A6 A6NMZ7$_{1,2}$: Collagen alpha-6(VI) chain	2/2	3	3	127	126	3	**1.42**
					129	128	3	**1.48**
					131	130	3	**0.92**
**321**	COL8A1 P27658: Collagen alpha-1(VIII) chain		3	3	127	126	2	**0.62**
					129	128	2	**1.06**
					131	130	2	**0.77**
**322**	COLEC10 Q9Y6Z7: Collectin-10		1	1	127	126	1	**0.76**
					129	128	1	**0.95**
					131	130	1	**1.13**
**323**	COLEC11 Q9BWP8$_{1-10}$: Collectin-11	10/10	3	3	127	126	3	**0.58**
					129	128	3	**0.74**
					131	130	3	**1.08**
**324**	COLGALT1 Q8NBJ5: Procollagen galactosyltransferase 1		1	1	127	126	1	**1.19**
					129	128	1	**1.57**
					131	130	1	**0.62**
**325**	COMP P49747$_1$: Cartilage oligomeric matrix protein	1/2	26	50	127	126	40	**0.46**
					129	128	37	**0.86**
					131	130	44	**0.98**
**326**	COPA P53621$_{1,2}$: Coatomer subunit alpha	2/2	8	8	127	126	6	**1.88**
					129	128	8	**2.63**
					131	130	7	**1.40**
**327**	COPB1 P53618: Coatomer subunit beta		11	14	127	126	13	**1.38**
					129	128	13	**2.20**
					131	130	13	**1.31**
**328**	COPB2 P35606$_{1,2}$: Coatomer subunit beta’	2/2	2	2	127	126	2	**1.23**
					129	128	2	**1.92**
					131	130	2	**1.38**

#	protein	group	peptides	spectra	ch1	ch2	quant	ratio
**329**	COPE O14579$_{1,3}$: Coatomer subunit epsilon	2/3	2	2	127	126	2	**1.04**
					129	128	2	**2.69**
					131	130	2	**1.81**
**330**	COPG1 Q9Y678: Coatomer subunit gamma-1		9	9	127	126	7	**1.17**
					129	128	6	**2.39**
					131	130	7	**1.54**
**331**	COPG2 Q9UBF2$_{1,2}$: Coatomer subunit gamma-2	2/2	4	4	127	126	2	**0.45**
					129	128	2	**1.82**
					131	130	2	**1.18**
**332**	COPS2 P61201$_{1,2}$: COP9 signalosome complex subunit 2	2/2	1	1	127	126	1	**1.85**
					129	128	1	**2.56**
					131	130	1	**1.23**
**333**	CORO1A P31146: Coronin-1A		7	8	127	126	7	**1.03**
					129	128	7	**1.50**
					131	130	6	**0.93**
**334**	CORO1B Q9BR76: Coronin-1B		2	3	127	126	2	**1.25**
					129	128	2	**2.16**
					131	130	2	**0.70**
**335**	CORO1C Q9ULV4$_{1-3}$: Coronin-1C	3/3	5	6	127	126	5	**1.29**
					129	128	5	**2.36**
					131	130	3	**1.00**
**336**	COTL1 Q14019: Coactosin-like protein		3	3	127	126	3	**1.60**
					129	128	3	**1.42**
					131	130	3	**0.75**
**337**	CP P00450: Ceruloplasmin		34	101	127	126	97	**0.48**
					129	128	97	**0.81**
					131	130	96	**1.18**
**338**	CPB2 Q96IY4$_{1}$: Carboxypeptidase B2	1/2	12	29	127	126	29	**1.48**
					129	128	29	**0.68**
					131	130	29	**1.28**
**339**	CPE P16870$_{1,2}$: Carboxypeptidase E	2/2	24	120	127	126	113	**0.68**
					129	128	113	**1.11**
					131	130	114	**0.72**
**340**	CPN1 P15169: Carboxypeptidase N catalytic chain		12	32	127	126	30	**1.06**
					129	128	31	**0.83**
					131	130	31	**1.05**

#	protein	group	peptides	spectra	ch1	ch2	quant	ratio
341	CPN2 P22792: Carboxypeptidase N subunit 2		14	61	127	126	50	**1.32**
					129	128	52	**1.10**
					131	130	59	**0.78**
342	CPQ Q9Y646: Carboxypeptidase Q		8	9	127	126	8	**1.12**
					129	128	8	**1.19**
					131	130	9	**0.87**
343	CPSF6 Q16630$_{1-3}$: Cleavage and polyadenylation specificity factor subunit 6	3/3	1	1	127	126	1	**0.88**
					129	128	1	**1.74**
					131	130	1	**1.21**
344	CPVL Q9H3G5: Probable serine carboxypeptidase CPVL		2	3	127	126	3	**0.67**
					129	128	3	**1.12**
					131	130	3	**1.11**
345	CPXM1 Q96SM3: Probable carboxypeptidase X1		8	10	127	126	7	**0.59**
					129	128	9	**1.09**
					131	130	8	**1.07**
346	CPXM2 Q8N436: Inactive carboxypeptidase-like protein X2		10	12	127	126	9	**0.49**
					129	128	9	**0.89**
					131	130	11	**0.89**
347	CPZ Q66K79$_{1-3}$: Carboxypeptidase Z	3/3	4	7	127	126	6	**0.69**
					129	128	5	**1.02**
					131	130	5	**1.07**
348	CRABP2 P29373: Cellular retinoic acid-binding protein 2	1/2	6	10	127	126	8	**5.21**
					129	128	10	**1.83**
					131	130	10	**1.12**
349	CRCT1 Q9UGL9: Cysteine-rich C-terminal protein 1		1	1	127	126		
					129	128	1	**0.44**
					131	130		
350	CRHBP P24387: Corticotropin-releasing factor-binding protein		3	4	127	126	4	**0.91**
					129	128	4	**0.98**
					131	130	4	**1.00**
351	CRIP1 P50238: Cysteine-rich protein 1		2	2	127	126	2	**0.56**
					129	128	2	**0.90**
					131	130	2	**0.62**
352	CRP P02741$_{1}$: C-reactive protein [Cleaved into: C-reactive protein(1-205)]	1/2	8	40	127	126	38	**1.76**
					129	128	40	**0.64**
					131	130	38	**1.43**

#	protein	group	peptides	spectra	ch1	ch2	quant	ratio
**353**	CRTAC1 Q9NQ79$_{1,2}$: Cartilage acidic protein 1	2/3	6	6	127	126	6	**0.90**
					129	128	6	**1.02**
					131	130	6	**0.94**
**354**	CRYAB P02511: Alpha-crystallin B chain		5	5	127	126	5	**1.04**
					129	128	5	**1.61**
					131	130	4	**1.17**
**355**	CSE1L P55060$_{1,3}$: Exportin-2	2/4	5	7	127	126	7	**0.77**
					129	128	6	**2.29**
					131	130	7	**1.23**
**356**	CSPG4 Q6UVK1: Chondroitin sulfate proteoglycan 4		10	11	127	126	11	**0.76**
					129	128	10	**0.92**
					131	130	9	**0.66**
**357**	CST2 P09228, CST4 P01036: Cystatin-SA, Cystatin-S	2/2	1	1	127	126	1	**0.82**
					129	128	1	**1.95**
					131	130	1	**0.98**
**358**	CST3 P01034: Cystatin-C		6	31	127	126	29	**1.23**
					129	128	29	**1.51**
					131	130	30	**0.81**
**359**	CST6 Q15828: Cystatin-M		7	11	127	126	11	**1.17**
					129	128	11	**1.47**
					131	130	11	**0.81**
**360**	CSTA P01040: Cystatin-A		3	4	127	126	3	**6.85**
					129	128	4	**1.02**
					131	130	4	**1.35**
**361**	CSTB P04080: Cystatin-B		4	4	127	126	4	**3.99**
					129	128	3	**1.19**
					131	130	3	**0.69**
**362**	CTBS Q01459: Di-N-acetylchitobiase		1	1	127	126	1	**0.90**
					129	128	1	**0.89**
					131	130	1	**0.95**
**363**	CTGF P29279$_{1,2}$: Connective tissue growth factor	2/2	4	5	127	126	3	**0.68**
					129	128	3	**0.74**
					131	130	3	**0.89**
**364**	CTHRC1 Q96CG8$_{1,3}$: Collagen triple helix repeat-containing protein 1	2/3	4	4	127	126	3	**1.06**
					129	128	3	**1.10**
					131	130	4	**0.73**

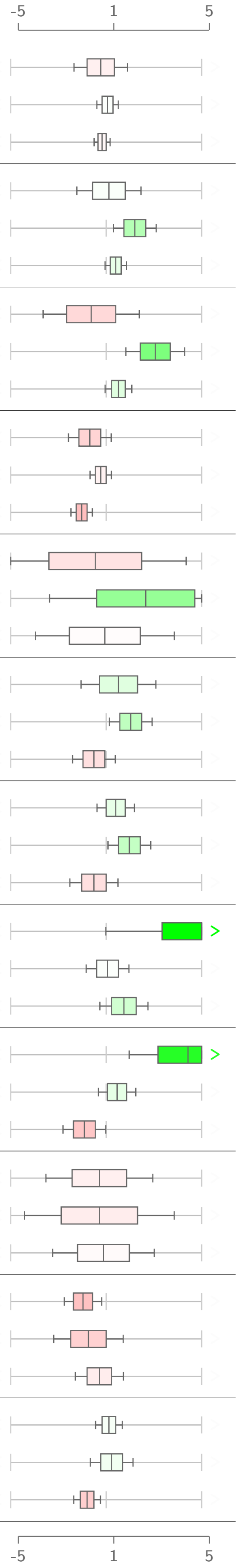

#	protein	group	peptides	spectra	ch1	ch2	quant	ratio
**365**	CTNNA1 P35221$_{1-3}$: Catenin alpha-1	3/9	8	10	127	126	9	**1.16**
					129	128	9	**1.21**
					131	130	10	**1.46**
**366**	CTNNB1 P35222$_{1}$: Catenin beta-1		6	6	127	126	3	**1.06**
					129	128	3	**1.02**
					131	130	3	**0.65**
**367**	CTNND1 O60716$_{1-32}$: Catenin delta-1	32/32	2	2	127	126	2	**2.25**
					129	128	2	**1.45**
					131	130	2	**1.14**
**368**	CTSA P10619$_{1,2}$: Lysosomal protective protein	2/2	6	7	127	126	7	**1.89**
					129	128	7	**1.10**
					131	130	7	**0.92**
**369**	CTSB P07858: Cathepsin B		5	14	127	126	14	**0.51**
					129	128	14	**0.66**
					131	130	14	**0.91**
**370**	CTSD P07339: Cathepsin D		11	14	127	126	14	**2.35**
					129	128	14	**1.09**
					131	130	10	**1.17**
**371**	CTSF Q9UBX1: Cathepsin F		13	15	127	126	14	**0.88**
					129	128	15	**1.23**
					131	130	13	**0.94**
**372**	CTSG P08311: Cathepsin G		2	2	127	126	2	**1.00**
					129	128	2	**1.35**
					131	130	2	**1.71**
**373**	CTSH P09668: Pro-cathepsin H [Cleaved into: Cathepsin H mini chain; Cathepsin H		2	2	127	126	2	**1.44**
					129	128	2	**1.48**
					131	130	2	**1.06**
**374**	CTSK P43235: Cathepsin K		3	3	127	126	3	**0.68**
					129	128	3	**1.00**
					131	130	3	**1.06**
**375**	CTSZ Q9UBR2: Cathepsin Z		2	2	127	126	2	**0.57**
					129	128	1	**0.73**
					131	130	1	**1.10**
**376**	CTTN Q14247$_{1,3}$: Src substrate cortactin	2/3	4	6	127	126	6	**1.28**
					129	128	6	**1.34**
					131	130	6	**0.94**

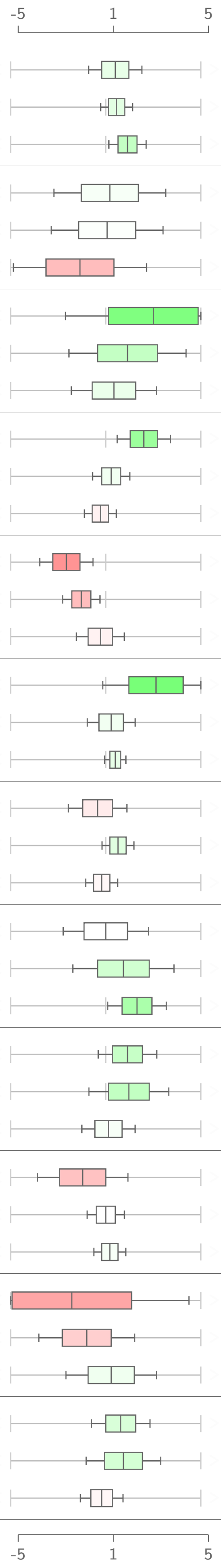

#	protein	group	peptides	spectra	ch1	ch2	quant	ratio
377	CUL3 Q13618$_{1-3}$: Cullin-3	3/3	3	3	127	126	3	**2.06**
					129	128	3	**2.29**
					131	130	3	**1.32**
378	CUTA O60888$_{1-3}$: Protein CutA	3/3	2	2	127	126	2	**1.20**
					129	128	2	**0.95**
					131	130	2	**1.05**
379	CXCL12 P48061$_{1-6}$: Stromal cell-derived factor 1	6/6	3	11	127	126	8	**0.39**
					129	128	11	**1.32**
					131	130	11	**0.70**
380	CXorf36 Q9H7Y0$_{1}$: Deleted in autism-related protein 1		4	4	127	126	4	**0.71**
					129	128	4	**1.00**
					131	130	3	**0.90**
381	CYB5A P00167$_{1-3}$: Cytochrome b5	3/3	1	1	127	126	1	**1.88**
					129	128	1	**1.42**
					131	130	1	**1.31**
382	CYB5R3 P00387$_{1-3}$: NADH-cytochrome b5 reductase 3	3/3	2	2	127	126	2	**2.04**
					129	128	2	**1.51**
					131	130	2	**1.23**
383	CYFIP1 Q7L576$_{1}$: Cytoplasmic FMR1-interacting protein 1	1/3	4	4	127	126	2	**1.24**
					129	128	2	**1.73**
					131	130	2	**0.88**
384	CYFIP2 Q96F07$_{1,2}$: Cytoplasmic FMR1-interacting protein 2	2/2	5	5	127	126	3	**1.21**
					129	128	3	**1.31**
					131	130	3	**0.45**
385	CYTL1 Q9NRR1: Cytokine-like protein 1		3	4	127	126	4	**0.64**
					129	128	4	**1.08**
					131	130	3	**0.90**
386	DAG1 Q14118: Dystroglycan		5	6	127	126	6	**0.73**
					129	128	6	**1.08**
					131	130	6	**1.13**
387	DARS P14868$_{1,2}$: Aspartate--tRNA ligase, cytoplasmic	2/2	5	6	127	126	5	**1.95**
					129	128	6	**2.02**
					131	130	6	**1.08**
388	DBI P07108$_{1-5}$: Acyl-CoA-binding protein	5/5	2	2	127	126	1	**7.02**
					129	128	2	**0.91**
					131	130	2	**0.97**

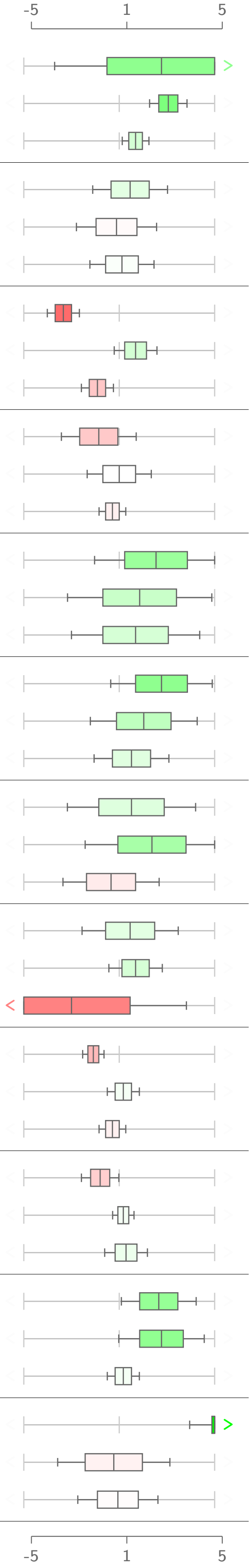

#	protein	group	peptides	spectra	ch1	ch2	quant	ratio
**389**	DBNL Q9UJU6$_{1,5}$: Drebrin-like protein	2/6	4	6	127	126	6	**1.02**
					129	128	5	**1.89**
					131	130	4	**1.09**
**390**	DCD P81605$_{1}$: Dermcidin	1/2	9	17	127	126	16	**0.48**
					129	128	16	**0.81**
					131	130	13	**2.58**
**391**	DCHS1 Q96JQ0: Protocadherin-16		2	2	127	126	2	**0.77**
					129	128	2	**1.17**
					131	130	2	**0.86**
**392**	DCN P07585$_{1}$: Decorin	1/5	14	40	127	126	37	**1.73**
					129	128	36	**1.52**
					131	130	38	**0.79**
**393**	DCPS Q96C86: m7GpppX diphosphatase		1	1	127	126	1	**1.66**
					129	128	1	**2.16**
					131	130	1	**0.77**
**394**	DCTN1 Q14203$_{1,3,4,6}$: Dynactin subunit 1	4/6	10	12	127	126	9	**1.58**
					129	128	9	**2.11**
					131	130	11	**1.03**
**395**	DCTN3 O75935$_{1,2}$: Dynactin subunit 3	2/2	1	1	127	126	1	**0.74**
					129	128	1	**1.24**
					131	130	1	**1.11**
**396**	DDB1 Q16531$_{1}$: DNA damage-binding protein 1	1/2	3	3	127	126	3	**0.81**
					129	128	3	**0.81**
					131	130	3	**1.78**
**397**	DDOST P39656$_{1,3}$: Dolichyl-diphosphooligosaccharide--protein glycosyltransferase 48 kDa subunit	2/2	2	2	127	126	2	**3.50**
					129	128	2	**1.48**
					131	130	2	**1.34**
**398**	DDX1 Q92499$_{1}$: ATP-dependent RNA helicase DDX1	1/3	7	42	127	126	8	**1.21**
					129	128	7	**1.50**
					131	130	8	**1.31**
**399**	DDX17 Q92841$_{1-4}$: Probable ATP-dependent RNA helicase DDX17	4/4	4	4	127	126	1	**1.90**
					129	128	1	**1.69**
					131	130	1	**1.36**
**400**	DDX19A Q9NUU7$_{1}$: ATP-dependent RNA helicase DDX19A	1/6	4	4	127	126	4	**0.85**
					129	128	4	**1.54**
					131	130	3	**1.22**

#	protein	group	peptides	spectra	ch1	ch2	quant	ratio
**401**	DDX39B Q13838$_{1,2}$: Spliceosome RNA helicase DDX39B	2/5	7	8	127	126	8	**1.53**
					129	128	8	**2.90**
					131	130	6	**1.51**
**402**	DDX3X O00571$_{1,2}$: ATP-dependent RNA helicase DDX3X	2/4	11	11	127	126	9	**1.76**
					129	128	7	**1.63**
					131	130	10	**1.22**
**403**	DDX5 P17844$_{1,2}$: Probable ATP-dependent RNA helicase DDX5	2/2	5	5	127	126	2	**2.36**
					129	128	2	**2.67**
					131	130	2	**1.56**
**404**	DEFA1 P59665: Neutrophil defensin 1		5	11	127	126	4	**0.71**
					129	128	4	**1.47**
					131	130	4	**0.53**
**405**	DEFA3 P59666: Neutrophil defensin 3		6	21	127	126	13	**0.56**
					129	128	13	**1.15**
					131	130	14	**0.74**
**406**	DEFA4 P12838: Neutrophil defensin 4		3	3	127	126	3	**0.65**
					129	128	3	**0.86**
					131	130	3	**0.82**
**407**	DEFA5 Q01523: Defensin-5		3	4	127	126	3	**0.61**
					129	128	4	**0.68**
					131	130	4	**0.46**
**408**	DEK P35659$_{1}$: Protein DEK	1/2	5	6	127	126	2	**0.61**
					129	128	3	**1.69**
					131	130	3	**1.42**
**409**	DES P17661: Desmin		17	30	127	126	11	**0.59**
					129	128	12	**2.63**
					131	130	12	**1.65**
**410**	DFNA5 O60443$_{1}$: Non-syndromic hearing impairment protein 5	1/3	9	9	127	126	9	**1.22**
					129	128	7	**1.85**
					131	130	7	**1.08**
**411**	DHX9 Q08211$_{1}$: ATP-dependent RNA helicase A		10	10	127	126	7	**1.29**
					129	128	9	**1.90**
					131	130	10	**1.56**
**412**	DIAPH1 O60610$_{1-3}$: Protein diaphanous homolog 1	3/3	4	4	127	126	4	**1.15**
					129	128	4	**1.49**
					131	130	3	**1.17**

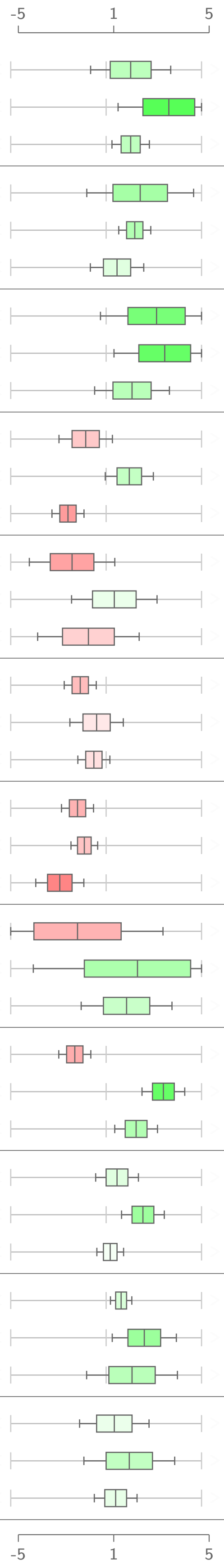

#	protein	group	peptides	spectra	ch1	ch2	quant	ratio
**413**	DIAPH2 O60879$_{1,2}$: Protein diaphanous homolog 2	2/2	6	7	127	126	5	**0.74**
					129	128	4	**1.37**
					131	130	6	**1.36**
**414**	DKK3 Q9UBP4: Dickkopf-related protein 3		8	42	127	126	37	**0.41**
					129	128	33	**0.81**
					131	130	38	**0.86**
**415**	DLD P09622$_{1,2}$: Dihydrolipoyl dehydrogenase, mitochondrial	2/3	2	2	127	126	2	**1.80**
					129	128	2	**1.31**
					131	130	2	**1.59**
**416**	DMKN Q6E0U4$_{1-7,16}$: Dermokine	8/8	6	7	127	126	6	**0.63**
					129	128	6	**0.81**
					131	130	6	**1.12**
**417**	DMP1 Q13316$_{1,2}$: Dentin matrix acidic phosphoprotein 1	2/2	3	3	127	126	3	**0.68**
					129	128	3	**0.63**
					131	130	3	**1.22**
**418**	DNAH12 Q6ZR08$_{1}$: Dynein heavy chain 12, axonemal		7	9	127	126	6	**0.57**
					129	128	6	**1.05**
					131	130	7	**0.87**
**419**	DNAJA2 O60884: DnaJ homolog subfamily A member 2		5	5	127	126	4	**1.35**
					129	128	4	**1.67**
					131	130	5	**1.33**
**420**	DNAJB1 P25685$_{1}$: DnaJ homolog subfamily B member 1		4	6	127	126	6	**1.94**
					129	128	6	**2.10**
					131	130	6	**1.68**
**421**	DNAJB11 Q9UBS4: DnaJ homolog subfamily B member 11		6	7	127	126	6	**1.00**
					129	128	6	**1.00**
					131	130	7	**0.88**
**422**	DNAJB9 Q9UBS3: DnaJ homolog subfamily B member 9		2	2	127	126	2	**0.50**
					129	128	2	**0.65**
					131	130	2	**0.90**
**423**	DNAJC3 Q13217: DnaJ homolog subfamily C member 3		19	21	127	126	20	**0.80**
					129	128	20	**1.02**
					131	130	19	**1.57**
**424**	DNM2 P50570$_{1-5}$: Dynamin-2	5/9	9	10	127	126	10	**1.62**
					129	128	10	**1.81**
					131	130	10	**1.21**

#	protein	group	peptides	spectra	ch1	ch2	quant	ratio
425	DNPH1 O43598$_1$: 2'-deoxynucleoside 5'-phosphate N-hydrolase 1	1/2	3	3	127	126	3	**0.87**
					129	128	3	**1.60**
					131	130	3	**0.95**
426	DOCK11 Q5JSL3: Dedicator of cytokinesis protein 11		2	2	127	126	1	**0.79**
					129	128	1	**1.12**
					131	130	1	**8.42**
427	DPP4 P27487: Dipeptidyl peptidase 4		2	2	127	126	2	**1.09**
					129	128	2	**1.35**
					131	130	2	**0.96**
428	DPT Q07507: Dermatopontin		9	143	127	126	118	**0.52**
					129	128	113	**0.72**
					131	130	117	**0.86**
429	DPYSL2 Q16555$_{1,2}$: Dihydropyrimidinase-related protein 2	2/4	6	6	127	126	4	**0.69**
					129	128	4	**1.47**
					131	130	4	**1.21**
430	DPYSL3 Q14195$_2$: Dihydropyrimidinase-related protein 3	1/2	6	7	127	126	3	**0.86**
					129	128	5	**1.61**
					131	130	5	**1.29**
431	DPYSL5 Q9BPU6: Dihydropyrimidinase-related protein 5		2	4	127	126	4	**0.86**
					129	128	4	**1.23**
					131	130	4	**1.44**
432	DSC1 Q08554$_1$: Desmocollin-1	1/2	8	20	127	126	12	**2.23**
					129	128	14	**0.69**
					131	130	19	**0.94**
433	DSC2 Q02487$_{1,2}$: Desmocollin-2	2/2	3	3	127	126	2	**2.87**
					129	128	2	**1.01**
					131	130	2	**0.93**
434	DSC3 Q14574$_1$: Desmocollin-3	1/2	8	9	127	126	8	**1.92**
					129	128	7	**0.92**
					131	130	6	**0.91**
435	DSG1 Q02413$_1$: Desmoglein-1	1/2	14	17	127	126	13	**3.04**
					129	128	13	**0.79**
					131	130	12	**1.09**
436	DSG4 Q86SJ6$_{1,2}$: Desmoglein-4	2/2	4	5	127	126	2	**0.04**
					129	128	2	**0.14**
					131	130	2	**0.81**

#	protein	group	peptides	spectra	ch1	ch2	quant	ratio
437	DSP P15924$_1$: Desmoplakin	1/3	126	165	127	126	142	**2.51**
					129	128	154	**0.86**
					131	130	160	**1.07**
439	DSTN P60981$_{1,2}$: Destrin	2/2	5	7	127	126	5	**1.65**
					129	128	6	**1.53**
					131	130	6	**1.20**
440	DUSP14 O95147: Dual specificity protein phosphatase 14		3	3	127	126	3	**0.09**
					129	128	2	**0.16**
					131	130	3	**1.31**
441	DYNC1H1 Q14204: Cytoplasmic dynein 1 heavy chain 1		38	47	127	126	46	**1.68**
					129	128	46	**2.05**
					131	130	43	**1.12**
442	DYNC1LI2 O43237$_1$: Cytoplasmic dynein 1 light intermediate chain 2		3	3	127	126	3	**1.24**
					129	128	3	**1.94**
					131	130	3	**1.18**
443	DYNLL1 P63167: Dynein light chain 1, cytoplasmic		2	5	127	126	3	**2.05**
					129	128	4	**1.77**
					131	130	3	**1.37**
444	ECM1 Q16610$_{1,4}$: Extracellular matrix protein 1	2/4	23	36	127	126	32	**0.67**
					129	128	36	**0.85**
					131	130	35	**1.21**
445	ECM2 O94769$_1$: Extracellular matrix protein 2		25	48	127	126	3	**0.75**
					129	128	3	**1.03**
					131	130	3	**0.77**
446	ECM2 O94769$_2$: Extracellular matrix protein 2		24	46	127	126	1	**0.55**
					129	128	1	**0.86**
					131	130	1	**1.01**
447	EEF1A1 P68104$_1$, EEF1A1P5 Q5VTE0: Elongation factor 1-alpha 1, Putative elongation factor 1-alpha-like 3	2/4	17	44	127	126	42	**1.85**
					129	128	40	**2.36**
					131	130	43	**1.57**
448	EEF1B2 P24534: Elongation factor 1-beta		6	7	127	126	6	**1.42**
					129	128	5	**2.09**
					131	130	6	**1.36**
449	EEF1D P29692$_{1-3}$: Elongation factor 1-delta	3/4	6	7	127	126	4	**1.11**
					129	128	6	**2.02**
					131	130	4	**1.38**

#	protein	group	peptides	spectra	ch1	ch2	quant	ratio
450	EEF1G P26641$_{1,2}$: Elongation factor 1-gamma	2/2	13	17	127	126	15	**1.91**
					129	128	17	**2.40**
					131	130	17	**1.49**
451	EEF2 P13639: Elongation factor 2		30	44	127	126	43	**2.73**
					129	128	41	**2.48**
					131	130	42	**1.12**
452	EFEMP1 Q12805$_{1-4}$: EGF-containing fibulin-like extracellular matrix protein 1	4/4	27	539	127	126	143	**0.92**
					129	128	135	**1.06**
					131	130	175	**0.66**
453	EFEMP1 Q12805$_5$: EGF-containing fibulin-like extracellular matrix protein 1		25	354	127	126	6	**0.80**
					129	128	6	**0.67**
					131	130	6	**0.53**
454	EFEMP2 O95967: EGF-containing fibulin-like extracellular matrix protein 2		17	106	127	126	91	**0.71**
					129	128	88	**1.07**
					131	130	97	**0.89**
455	EHD1 Q9H4M9: EH domain-containing protein 1	1/3	10	13	127	126	10	**1.36**
					129	128	11	**2.13**
					131	130	10	**1.22**
456	EHD2 Q9NZN4$_1$: EH domain-containing protein 2	1/2	15	20	127	126	18	**0.97**
					129	128	17	**2.36**
					131	130	16	**1.58**
457	EIF1AX P47813, EIF1AY O14602: Eukaryotic translation initiation factor 1A, X-chromosomal, Eukaryotic translation initiation factor 1A, Y-chromosomal	2/2	2	3	127	126	3	**2.03**
					129	128	3	**1.85**
					131	130	3	**1.14**
458	EIF2S1 P05198: Eukaryotic translation initiation factor 2 subunit 1		5	6	127	126	4	**1.16**
					129	128	5	**1.85**
					131	130	4	**1.26**
459	EIF2S2 P20042: Eukaryotic translation initiation factor 2 subunit 2		2	2	127	126	1	**1.05**
					129	128	1	**1.90**
					131	130	2	**1.29**
460	EIF2S3 P41091: Eukaryotic translation initiation factor 2 subunit 3	1/3	5	5	127	126	4	**1.88**
					129	128	3	**2.13**
					131	130	3	**1.32**
461	EIF3A Q14152$_{1,2}$: Eukaryotic translation initiation factor 3 subunit A	2/2	7	10	127	126	10	**1.31**
					129	128	10	**2.14**
					131	130	9	**1.26**

#	protein	group	peptides	spectra	ch1	ch2	quant	ratio
**462**	EIF3B P55884$_{1,2}$: Eukaryotic translation initiation factor 3 subunit B	2/2	6	7	127	126	7	**0.86**
					129	128	6	**1.24**
					131	130	7	**1.04**
**463**	EIF3C Q99613$_{1,2}$, EIF3CL B5ME19: Eukaryotic translation initiation factor 3 subunit C, Eukaryotic translation initiation factor 3 subunit C-like protein	3/3	4	5	127	126	5	**2.36**
					129	128	5	**2.18**
					131	130	5	**1.43**
**464**	EIF3E P60228: Eukaryotic translation initiation factor 3 subunit E		5	5	127	126	5	**1.37**
					129	128	5	**2.41**
					131	130	4	**1.35**
**465**	EIF3H O15372: Eukaryotic translation initiation factor 3 subunit H		4	4	127	126	3	**0.94**
					129	128	4	**1.68**
					131	130	4	**1.20**
**466**	EIF3I Q13347: Eukaryotic translation initiation factor 3 subunit I		2	2	127	126	2	**1.60**
					129	128	2	**1.61**
					131	130	2	**1.61**
**467**	EIF3J O75822$_{1}$: Eukaryotic translation initiation factor 3 subunit J	1/3	7	8	127	126	7	**0.96**
					129	128	7	**1.76**
					131	130	6	**1.28**
**468**	EIF3K Q9UBQ5$_{1,2}$: Eukaryotic translation initiation factor 3 subunit K	2/2	3	3	127	126	3	**1.40**
					129	128	3	**2.18**
					131	130	3	**1.29**
**469**	EIF4A1 P60842$_{1}$: Eukaryotic initiation factor 4A-I	1/2	10	15	127	126	6	**1.25**
					129	128	7	**1.92**
					131	130	7	**0.97**
**470**	EIF4A2 Q14240$_{1,2}$: Eukaryotic initiation factor 4A-II	2/2	6	9	127	126	1	**1.11**
					129	128	1	**1.82**
					131	130	1	**1.10**
**471**	EIF4A3 P38919: Eukaryotic initiation factor 4A-III		5	5	127	126	3	**3.31**
					129	128	3	**2.57**
					131	130	3	**1.47**
**472**	EIF4B P23588$_{1,2}$: Eukaryotic translation initiation factor 4B	2/2	3	3	127	126	3	**1.54**
					129	128	3	**1.65**
					131	130	3	**1.50**
**473**	EIF4E P06730$_{1-3}$: Eukaryotic translation initiation factor 4E	3/3	2	2	127	126	2	**1.23**
					129	128	2	**1.85**
					131	130	2	**1.46**

#	protein	group	peptides	spectra	ch1	ch2	quant	ratio
**474**	EIF4G1 Q04637$_{8}$: Eukaryotic translation initiation factor 4 gamma 1	1/8	11	11	127	126	8	**1.52**
					129	128	10	**1.66**
					131	130	10	**1.30**
**475**	EIF4G3 O43432$_{1,3,4}$: Eukaryotic translation initiation factor 4 gamma 3	3/3	5	8	127	126	3	**1.08**
					129	128	2	**1.78**
					131	130	4	**1.36**
**476**	EIF4H Q15056$_{1,2}$: Eukaryotic translation initiation factor 4H	2/2	4	4	127	126	4	**1.86**
					129	128	4	**1.76**
					131	130	4	**1.23**
**477**	EIF5A P63241$_{1,2}$: Eukaryotic translation initiation factor 5A-1	2/4	4	6	127	126	6	**1.77**
					129	128	6	**1.69**
					131	130	6	**1.23**
**478**	EIF6 P56537$_{1}$: Eukaryotic translation initiation factor 6		2	2	127	126	2	**0.94**
					129	128	2	**1.09**
					131	130	2	**0.81**
**479**	ELANE P08246: Neutrophil elastase		2	2	127	126	2	**1.01**
					129	128	2	**1.22**
					131	130	2	**1.45**
**480**	ELAVL1 Q15717$_{1,2}$: ELAV-like protein 1	2/2	1	1	127	126	1	**1.11**
					129	128	1	**1.24**
					131	130	1	**1.27**
**481**	ELN P15502$_{1,2,4,5}$: Elastin	4/4	2	2	127	126	2	**0.49**
					129	128	2	**1.69**
					131	130	2	**1.02**
**482**	EMILIN1 Q9Y6C2$_{1}$: EMILIN-1	1/2	13	16	127	126	15	**0.62**
					129	128	15	**0.80**
					131	130	15	**1.01**
**483**	EMILIN2 Q9BXX0: EMILIN-2		14	18	127	126	18	**0.52**
					129	128	16	**0.98**
					131	130	18	**0.89**
**484**	ENO1 P06733$_{1}$: Alpha-enolase	1/7	23	43	127	126	43	**3.74**
					129	128	43	**1.80**
					131	130	43	**1.04**
**485**	EPPK1 P58107: Epiplakin		24	33	127	126	21	**4.15**
					129	128	20	**1.35**
					131	130	18	**1.41**

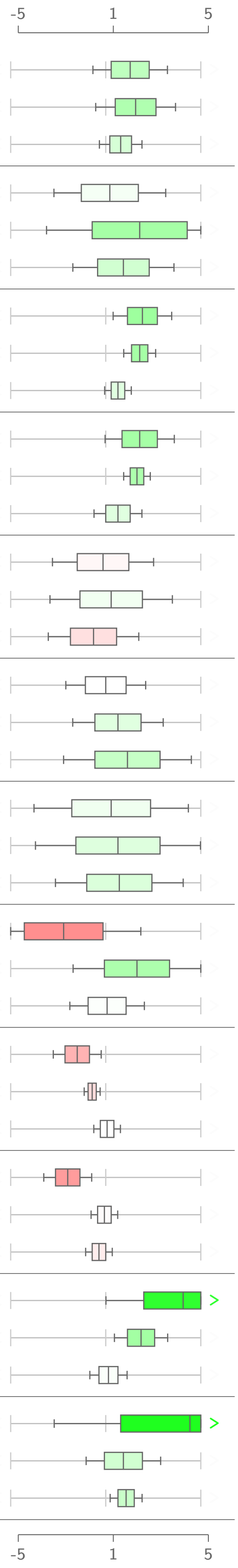

#	protein	group	peptides	spectra	ch1	ch2	quant	ratio
486	EPRS P07814: Bifunctional glutamate/proline--tRNA ligase		6	6	127	126	5	**0.92**
					129	128	5	**1.42**
					131	130	5	**1.01**
487	EPS8L1 Q8TE68$_{1,2,4}$: Epidermal growth factor receptor kinase substrate 8-like protein 1	3/3	3	3	127	126	3	**1.40**
					129	128	3	**0.99**
					131	130	3	**1.17**
488	EPS8L2 Q9H6S3$_{1,3}$: Epidermal growth factor receptor kinase substrate 8-like protein 2	2/2	4	4	127	126	4	**1.45**
					129	128	3	**1.34**
					131	130	3	**1.04**
489	ERH P84090: Enhancer of rudimentary homolog		2	3	127	126	3	**2.31**
					129	128	3	**1.34**
					131	130	3	**0.66**
490	ERLIN2 O94905$_{1}$: Erlin-2		2	2	127	126	2	**1.43**
					129	128	2	**1.44**
					131	130	2	**1.48**
491	ERP44 Q9BS26: Endoplasmic reticulum resident protein 44		10	15	127	126	15	**0.65**
					129	128	13	**1.03**
					131	130	13	**0.88**
492	ESD P10768: S-formylglutathione hydrolase		1	1	127	126	1	**1.03**
					129	128	1	**1.75**
					131	130	1	**1.37**
493	ETF1 P62495$_{1,2}$: Eukaryotic peptide chain release factor subunit 1	2/2	3	4	127	126	4	**1.12**
					129	128	4	**1.39**
					131	130	3	**1.01**
494	EVPL Q92817: Envoplakin		15	16	127	126	15	**3.10**
					129	128	14	**0.98**
					131	130	14	**0.91**
495	EWSR1 Q01844$_{1-3,5,6}$: RNA-binding protein EWS	5/5	1	1	127	126	1	**2.00**
					129	128	1	**2.82**
					131	130	1	**1.99**
496	EZR P15311: Ezrin	1/2	25	36	127	126	26	**1.89**
					129	128	24	**1.96**
					131	130	25	**1.01**
497	F10 P00742: Coagulation factor X		19	78	127	126	58	**0.86**
					129	128	59	**0.81**
					131	130	62	**1.60**

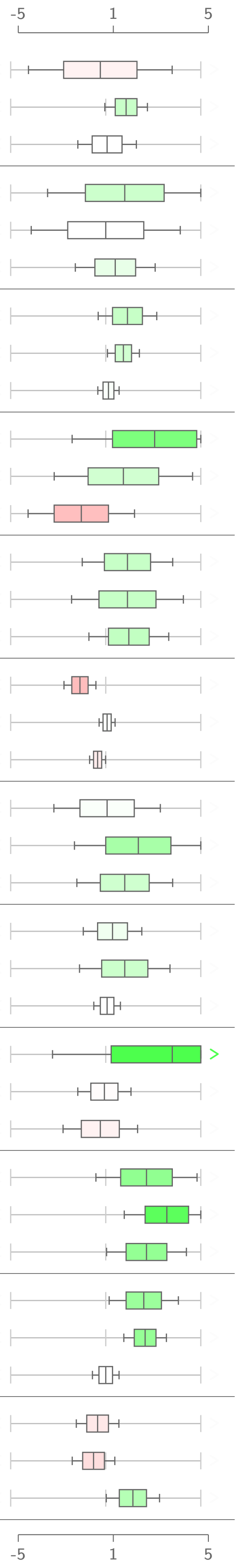

#	protein	group	peptides	spectra	ch1	ch2	quant	ratio
**498**	F11 P03951$_1$: Coagulation factor XI	1/2	22	33	127	126	32	**0.83**
					129	128	30	**0.71**
					131	130	29	**1.16**
**499**	F12 P00748: Coagulation factor XII		20	48	127	126	47	**0.97**
					129	128	46	**0.98**
					131	130	46	**1.00**
**500**	F13A1 P00488: Coagulation factor XIII A chain		25	65	127	126	62	**0.51**
					129	128	62	**0.82**
					131	130	63	**1.25**
**501**	F13B P05160: Coagulation factor XIII B chain		18	27	127	126	25	**0.67**
					129	128	23	**0.83**
					131	130	26	**1.23**
**502**	F2 P00734: Prothrombin		39	921	127	126	877	**0.77**
					129	128	875	**0.78**
					131	130	857	**1.05**
**503**	F5 P12259: Coagulation factor V		34	48	127	126	42	**1.29**
					129	128	45	**0.78**
					131	130	44	**1.33**
**504**	F7 P08709$_{1,2}$: Coagulation factor VII	2/2	9	17	127	126	17	**1.47**
					129	128	17	**1.13**
					131	130	17	**0.99**
**505**	F9 P00740$_1$: Coagulation factor IX	1/2	20	50	127	126	41	**1.10**
					129	128	39	**1.12**
					131	130	43	**1.64**
**506**	FABP1 P07148: Fatty acid-binding protein, liver		3	4	127	126	2	**0.90**
					129	128	2	**0.95**
					131	130	2	**0.86**
**507**	FABP3 P05413: Fatty acid-binding protein, heart		5	6	127	126	6	**1.70**
					129	128	6	**1.65**
					131	130	5	**0.68**
**508**	FABP4 P15090: Fatty acid-binding protein, adipocyte		2	2	127	126	2	**1.82**
					129	128	2	**1.82**
					131	130	2	**0.81**
**509**	FABP5 Q01469: Fatty acid-binding protein, epidermal	1/2	8	17	127	126	14	**10.27**
					129	128	15	**0.95**
					131	130	14	**0.98**

#	protein	group	peptides	spectra	ch1	ch2	quant	ratio
510	FABP9 Q0Z7S8: Fatty acid-binding protein 9		4	7	127	126	6	**0.51**
					129	128	7	**0.59**
					131	130	6	**1.01**
511	FAM114A1 Q8IWE2$_1$: Protein NOXP20	1/2	4	4	127	126	4	**0.88**
					129	128	4	**1.40**
					131	130	4	**1.35**
512	FAM129B Q96TA1$_{1,2}$: Niban-like protein 1	2/2	7	9	127	126	9	**1.60**
					129	128	9	**1.48**
					131	130	7	**0.89**
513	FAM198B Q6UWH4$_{1,2}$: Protein FAM198B	2/2	2	2	127	126	1	**0.94**
					129	128	1	**0.98**
					131	130	1	**0.91**
514	FAM20C Q8IXL6$_1$: Extracellular serine/threonine protein kinase FAM20C	1/2	4	4	127	126	4	**0.81**
					129	128	4	**0.91**
					131	130	4	**0.90**
515	FAM49B Q9NUQ9$_1$: Protein FAM49B		4	5	127	126	4	**1.35**
					129	128	5	**1.19**
					131	130	5	**0.81**
516	FARSA Q9Y285$_{1,2}$: Phenylalanine--tRNA ligase alpha subunit	2/2	3	3	127	126	3	**1.66**
					129	128	3	**2.48**
					131	130	3	**1.45**
517	FARSB Q9NSD9$_1$: Phenylalanine--tRNA ligase beta subunit	1/2	7	8	127	126	7	**1.91**
					129	128	7	**2.36**
					131	130	7	**1.71**
518	FASN P49327: Fatty acid synthase		7	7	127	126	7	**2.44**
					129	128	7	**1.66**
					131	130	5	**0.98**
519	FBLN1 P23142$_1$: Fibulin-1	1/2	31	791	127	126	109	**0.77**
					129	128	105	**1.06**
					131	130	108	**0.76**
520	FBLN1 P23142$_3$: Fibulin-1		22	675	127	126	2	**1.42**
					129	128	2	**0.91**
					131	130	2	**2.04**
521	FBLN1 P23142$_4$: Fibulin-1		27	715	127	126	39	**0.69**
					129	128	39	**0.89**
					131	130	36	**1.05**

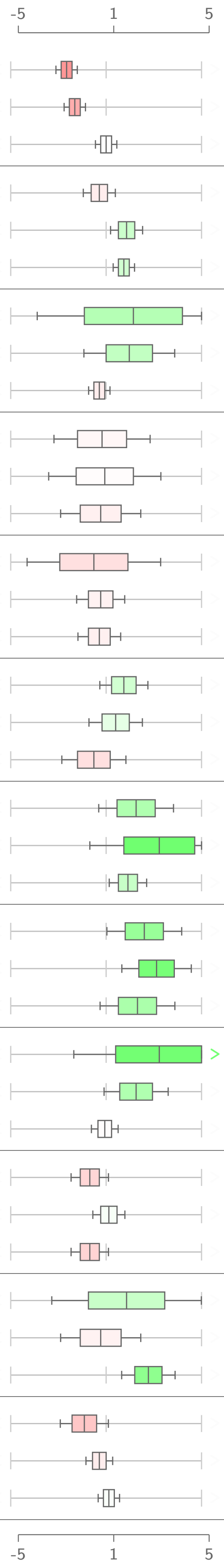

#	protein	group	peptides	spectra	ch1	ch2	quant	ratio
**522**	FBLN2 P98095$_2$: Fibulin-2	1/2	33	67	127	126	58	**0.78**
					129	128	66	**1.13**
					131	130	60	**0.85**
**523**	FBLN5 Q9UBX5: Fibulin-5		17	145	127	126	130	**0.59**
					129	128	134	**1.00**
					131	130	134	**0.93**
**524**	FBLN7 Q53RD9$_{1,2}$: Fibulin-7	2/4	6	9	127	126	9	**0.83**
					129	128	9	**1.06**
					131	130	9	**1.00**
**525**	FBN1 P35555: Fibrillin-1 [Cleaved into: Asprosin]		89	182	127	126	134	**0.60**
					129	128	128	**0.86**
					131	130	140	**0.98**
**526**	FBN2 P35556$_1$: Fibrillin-2 [Cleaved into: Fibrillin-2 C-terminal peptide]	1/2	25	73	127	126	26	**1.35**
					129	128	20	**1.08**
					131	130	33	**1.17**
**527**	FBXO2 Q9UK22: F-box only protein 2		2	2	127	126	2	**0.41**
					129	128	2	**1.20**
					131	130	2	**1.25**
**528**	FCGBP Q9Y6R7: IgGFc-binding protein		19	23	127	126	21	**0.57**
					129	128	20	**0.59**
					131	130	20	**1.01**
**529**	FCN1 O00602: Ficolin-1		5	7	127	126	4	**1.18**
					129	128	3	**1.30**
					131	130	4	**0.95**
**530**	FCN2 Q15485$_1$: Ficolin-2	1/2	6	13	127	126	9	**0.82**
					129	128	10	**1.01**
					131	130	10	**1.95**
**531**	FCN3 O75636$_1$: Ficolin-3		10	26	127	126	3	**1.15**
					129	128	3	**0.76**
					131	130	3	**1.61**
**533**	FCRL5 Q96RD9$_{1,3}$: Fc receptor-like protein 5	2/2	1	1	127	126	1	**0.58**
					129	128	1	**0.68**
					131	130	1	**0.82**
**534**	FERMT2 Q96AC1$_{1-3}$: Fermitin family homolog 2	3/3	5	6	127	126	4	**0.96**
					129	128	4	**2.01**
					131	130	4	**1.14**

#	protein	group	peptides	spectra	ch1	ch2	quant	ratio
**535**	FETUB Q9UGM5$_1$: Fetuin-B	1/2	15	43	127	126	41	**2.08**
					129	128	41	**1.33**
					131	130	39	**0.85**
**536**	FGA P02671$_1$: Fibrinogen alpha chain [Cleaved into: Fibrinopeptide A; Fibrinogen alpha chain]	1/2	65	2568	127	126	2479	**1.43**
					129	128	2349	**1.09**
					131	130	2451	**1.49**
**537**	FGB P02675: Fibrinogen beta chain [Cleaved into: Fibrinopeptide B; Fibrinogen beta chain]		42	2424	127	126	2340	**2.05**
					129	128	2144	**1.03**
					131	130	2342	**1.65**
**538**	FGG P02679$_{1,2}$: Fibrinogen gamma chain	2/2	33	1676	127	126	1611	**2.13**
					129	128	1554	**1.02**
					131	130	1577	**1.67**
**539**	FGL2 Q14314: Fibroleukin		3	4	127	126	2	**1.26**
					129	128	2	**1.41**
					131	130	2	**0.79**
**540**	FIBIN Q8TAL6: Fin bud initiation factor homolog		2	2	127	126	2	**0.49**
					129	128	2	**0.83**
					131	130	2	**0.89**
**541**	FKBP1A P62942: Peptidyl-prolyl cis-trans isomerase FKBP1A		2	3	127	126	3	**0.88**
					129	128	3	**1.12**
					131	130	3	**0.90**
**542**	FLG P20930: Filaggrin		38	48	127	126	39	**2.82**
					129	128	38	**0.90**
					131	130	42	**1.07**
**543**	FLG2 Q5D862: Filaggrin-2		12	14	127	126	11	**6.57**
					129	128	12	**0.72**
					131	130	14	**1.29**
**544**	FLII Q13045$_{1-3}$: Protein flightless-1 homolog	3/3	5	5	127	126	5	**1.46**
					129	128	4	**2.72**
					131	130	3	**1.07**
**545**	FLNA P21333$_{1,2}$: Filamin-A	2/2	69	96	127	126	77	**1.52**
					129	128	77	**2.50**
					131	130	81	**1.19**
**546**	FLNB O75369$_{1-3,6,8,9}$: Filamin-B	6/9	14	16	127	126	10	**2.62**
					129	128	10	**2.05**
					131	130	9	**1.23**

#	protein	group	peptides	spectra	ch1	ch2	quant	ratio
					127	126	41	**1.00**
**547**	FLNC Q14315$_{1,2}$: Filamin-C	2/2	48	57	129	128	43	**2.42**
					131	130	44	**1.31**
					127	126	20	**0.69**
**548**	FMOD Q06828: Fibromodulin		10	33	129	128	21	**0.95**
					131	130	21	**0.94**
					127	126	1007	**0.65**
**549**	FN1 P02751$_{1,3}$: Fibronectin	2/17	92	1082	129	128	996	**0.73**
					131	130	1011	**1.14**
					127	126	3	**0.62**
**550**	FNDC1 Q4ZHG4$_{1,2}$: Fibronectin type III domain-containing protein 1	2/2	3	3	129	128	3	**1.10**
					131	130	3	**1.04**
					127	126	2	**1.92**
**551**	FOXR1 Q6PIV2$_{1}$: Forkhead box protein R1		3	8	129	128	2	**1.06**
					131	130	2	**0.63**
					127	126	31	**1.16**
**552**	FRA10AC1 Q70Z53$_{3}$: Protein FRA10AC1	1/5	5	43	129	128	39	**1.09**
					131	130	38	**0.98**
					127	126	10	**0.87**
**553**	FRAS1 Q86XX4$_{2}$: Extracellular matrix protein FRAS1	1/3	11	16	129	128	10	**1.06**
					131	130	9	**0.87**
					127	126	2	**0.55**
**554**	FRZB Q92765: Secreted frizzled-related protein 3		2	2	129	128	2	**0.83**
					131	130	2	**1.07**
					127	126	7	**1.36**
**555**	FSCN1 Q16658: Fascin		6	7	129	128	7	**1.24**
					131	130	6	**0.92**
					127	126	12	**0.67**
**556**	FST P19883$_{1,2}$: Follistatin	2/2	12	14	129	128	13	**0.85**
					131	130	12	**0.89**
					127	126	36	**0.46**
**557**	FSTL1 Q12841$_{1}$: Follistatin-related protein 1	1/2	18	38	129	128	33	**1.02**
					131	130	36	**0.72**
					127	126	4	**0.62**
**558**	FSTL3 O95633$_{1,2}$: Follistatin-related protein 3	2/2	4	4	129	128	4	**0.85**
					131	130	4	**0.90**

#	protein	group	peptides	spectra	ch1	ch2	quant	ratio
**559**	FTL P02792: Ferritin light chain		3	3	127	126	3	**0.57**
					129	128	3	**1.46**
					131	130	3	**1.10**
**560**	FUBP1 Q96AE4$_{1,2}$: Far upstream element-binding protein 1	2/2	7	7	127	126	4	**1.04**
					129	128	3	**2.45**
					131	130	4	**1.57**
**561**	FUBP3 Q96I24$_{1}$: Far upstream element-binding protein 3		2	2	127	126	1	**1.02**
					129	128	1	**2.70**
					131	130	1	**1.41**
**562**	FUCA2 Q9BTY2$_{1}$: Plasma alpha-L-fucosidase		3	3	127	126	3	**0.87**
					129	128	3	**0.95**
					131	130	3	**0.85**
**563**	FUS P35637$_{1,2}$: RNA-binding protein FUS	2/2	7	9	127	126	8	**0.63**
					129	128	8	**1.41**
					131	130	8	**1.11**
**564**	G3BP1 Q13283$_{1}$: Ras GTPase-activating protein-binding protein 1		2	2	127	126	2	**0.90**
					129	128	2	**2.17**
					131	130	2	**2.29**
**565**	G6PD P11413$_{1-3}$: Glucose-6-phosphate 1-dehydrogenase	3/3	7	7	127	126	5	**1.44**
					129	128	7	**1.91**
					131	130	6	**1.16**
**566**	GALE Q14376$_{1,2}$: UDP-glucose 4-epimerase	2/2	2	2	127	126	2	**1.38**
					129	128	2	**2.23**
					131	130	2	**1.44**
**567**	GALK1 P51570$_{1,2}$: Galactokinase	2/2	3	3	127	126	3	**1.22**
					129	128	3	**1.85**
					131	130	3	**1.45**
**568**	GALNT2 Q10471$_{1,2}$: Polypeptide N-acetylgalactosaminyltransferase 2	2/2	1	1	127	126	1	**0.98**
					129	128	1	**1.61**
					131	130	1	**0.78**
**569**	GANAB Q14697$_{1}$: Neutral alpha-glucosidase AB		12	16	127	126	1	**0.79**
					129	128	1	**1.14**
					131	130	1	**0.76**
**570**	GANAB Q14697$_{2}$: Neutral alpha-glucosidase AB		12	16	127	126	1	**1.22**
					129	128	1	**1.00**
					131	130	1	**1.05**

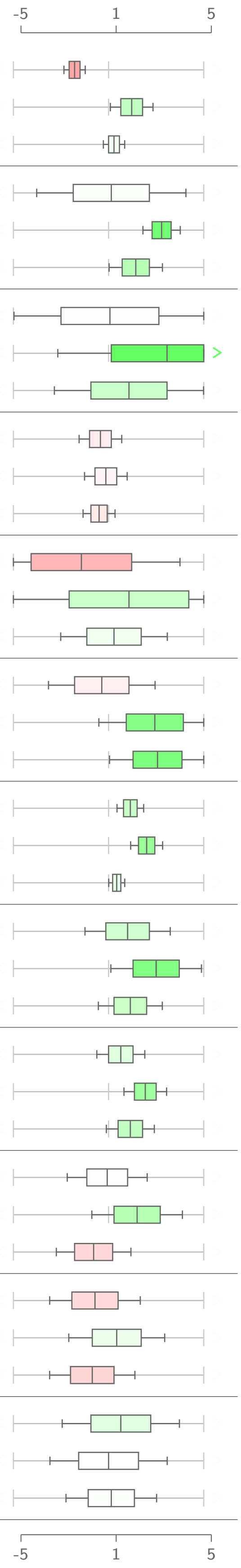

#	protein	group	peptides	spectra	ch1	ch2	quant	ratio
571	GANAB Q14697$_3$: Neutral alpha-glucosidase AB		2	7	127	126	1	**1.23**
					129	128		
					131	130		
572	GAPDH P04406$_1$: Glyceraldehyde-3-phosphate dehydrogenase	1/2	19	63	127	126	63	**1.97**
					129	128	63	**2.63**
					131	130	63	**2.08**
573	GARS P41250: Glycine--tRNA ligase		6	13	127	126	7	**1.72**
					129	128	6	**1.61**
					131	130	9	**1.09**
574	GAS1 P54826: Growth arrest-specific protein 1		9	20	127	126	19	**0.73**
					129	128	18	**0.75**
					131	130	19	**0.98**
575	GAS6 Q14393$_1$: Growth arrest-specific protein 6	1/5	18	29	127	126	29	**0.80**
					129	128	24	**1.06**
					131	130	27	**0.75**
576	GBA P04062$_{1,2,4,5}$: Glucosylceramidase	4/5	5	6	127	126	5	**1.57**
					129	128	6	**1.07**
					131	130	6	**0.92**
577	GBP1 P32455: Guanylate-binding protein 1		3	3	127	126	2	**1.73**
					129	128	2	**1.28**
					131	130	2	**0.72**
578	GBP2 P32456: Guanylate-binding protein 2		4	4	127	126	3	**1.37**
					129	128	3	**1.29**
					131	130	3	**1.27**
579	GC P02774$_{1,3}$: Vitamin D-binding protein	2/3	32	390	127	126	332	**0.58**
					129	128	313	**1.23**
					131	130	350	**0.95**
580	GCG P01275: Glucagon [Cleaved into: Glicentin; Glicentin-related polypeptide		5	6	127	126	5	**0.29**
					129	128	6	**0.92**
					131	130	6	**0.77**
581	GCN1 Q92616: eIF-2-alpha kinase activator GCN1		2	2	127	126	2	**1.31**
					129	128	2	**1.82**
					131	130	2	**0.96**
582	GDF15 Q99988: Growth/differentiation factor 15		2	3	127	126	3	**0.59**
					129	128	3	**0.77**
					131	130	3	**0.82**

#	protein	group	peptides	spectra	ch1	ch2	quant	ratio
**583**	GDF7 Q7Z4P5: Growth/differentiation factor 7		1	1	127	126	1	**0.91**
					129	128	1	**1.20**
					131	130	1	**1.04**
**584**	GDI1 P31150: Rab GDP dissociation inhibitor alpha		4	4	127	126	2	**1.61**
					129	128	2	**1.34**
					131	130	2	**0.92**
**585**	GDI2 P50395$_1$: Rab GDP dissociation inhibitor beta	1/2	7	8	127	126	6	**4.26**
					129	128	6	**1.41**
					131	130	6	**1.89**
**587**	GGCT O75223$_1$: Gamma-glutamylcyclotransferase	1/2	3	3	127	126	3	**30.94**
					129	128	3	**1.04**
					131	130	3	**1.02**
**588**	GGH Q92820: Gamma-glutamyl hydrolase		2	2	127	126	2	**2.84**
					129	128	2	**0.82**
					131	130	2	**0.83**
**589**	GHRL Q9UBU3$_{1-5}$: Appetite-regulating hormone	5/5	1	3	127	126	3	**2.48**
					129	128	3	**0.98**
					131	130	3	**0.55**
**590**	GIPC1 O14908$_{1,2}$: PDZ domain-containing protein GIPC1	2/2	1	2	127	126	2	**0.87**
					129	128	2	**0.90**
					131	130	2	**0.88**
**591**	GKN1 Q9NS71: Gastrokine-1		7	13	127	126	13	**1.04**
					129	128	12	**1.03**
					131	130	11	**0.90**
**592**	GLA P06280: Alpha-galactosidase A		9	27	127	126	14	**0.80**
					129	128	14	**1.13**
					131	130	14	**0.87**
**593**	GLIPR1L2 Q4G1C9$_{1,5,6}$: GLIPR1-like protein 2	3/3	4	42	127	126	17	**0.56**
					129	128	18	**0.96**
					131	130	24	**0.99**
**594**	GLTP Q9NZD2: Glycolipid transfer protein		1	1	127	126	1	**9.30**
					129	128	1	**1.19**
					131	130	1	**0.69**
**595**	GLUL P15104: Glutamine synthetase		4	6	127	126	5	**20.22**
					129	128	5	**1.39**
					131	130	6	**0.94**

#	protein	group	peptides	spectra	ch1	ch2	quant	ratio
**596**	GM2A P17900: Ganglioside GM2 activator		7	16	127	126	15	**1.42**
					129	128	16	**1.33**
					131	130	15	**1.11**
**597**	GMPPB Q9Y5P6$_{1,2}$: Mannose-1-phosphate guanyltransferase beta	2/2	3	3	127	126	3	**1.56**
					129	128	3	**2.42**
					131	130	3	**1.65**
**598**	GNAI2 P04899$_{6}$: Guanine nucleotide-binding protein G(i) subunit alpha-2	1/6	3	3	127	126	3	**0.39**
					129	128	2	**1.50**
					131	130	3	**1.02**
**599**	GNB1 P62873$_{1,2}$: Guanine nucleotide-binding protein G(I)/G(S)/G(T) subunit beta-1	2/2	3	3	127	126	2	**0.96**
					129	128	2	**1.48**
					131	130	2	**1.01**
**600**	GNB2 P62879$_{1}$, GNB4 Q9HAV0: Guanine nucleotide-binding protein G(I)/G(S)/G(T) subunit beta-2, Guanine nucleotide-binding protein subunit beta-4	2/3	3	3	127	126	2	**0.97**
					129	128	2	**0.99**
					131	130	2	**1.04**
**601**	GNG12 Q9UBI6: Guanine nucleotide-binding protein G(I)/G(S)/G(O) subunit gamma-12		2	2	127	126	2	**2.60**
					129	128	2	**1.61**
					131	130	2	**1.04**
**602**	GNL1 P36915$_{1,2}$: Guanine nucleotide-binding protein-like 1	2/2	1	1	127	126	1	**0.90**
					129	128	1	**1.50**
					131	130	1	**1.13**
**603**	GNPTG Q9UJJ9: N-acetylglucosamine-1-phosphotransferase subunit gamma		4	4	127	126	4	**0.78**
					129	128	3	**1.07**
					131	130	4	**0.85**
**604**	GOLGA4 Q13439$_{1,3-5}$: Golgin subfamily A member 4	4/4	2	5	127	126	4	**0.03**
					129	128	4	**0.34**
					131	130	4	**1.21**
**605**	GOLGA6L2 Q8N9W4$_{1}$: Golgin subfamily A member 6-like protein 2		6	35	127	126	9	**0.90**
					129	128	11	**0.78**
					131	130	15	**0.77**
**606**	GOLGA6L22 H0YM25: Golgin subfamily A member 6-like protein 22		9	93	127	126	6	**0.96**
					129	128	7	**1.00**
					131	130	10	**1.12**
**607**	GOLGA6L6 A8MZA4: Golgin subfamily A member 6-like protein 6	1/2	13	101	127	126	16	**0.80**
					129	128	9	**0.76**
					131	130	23	**1.00**

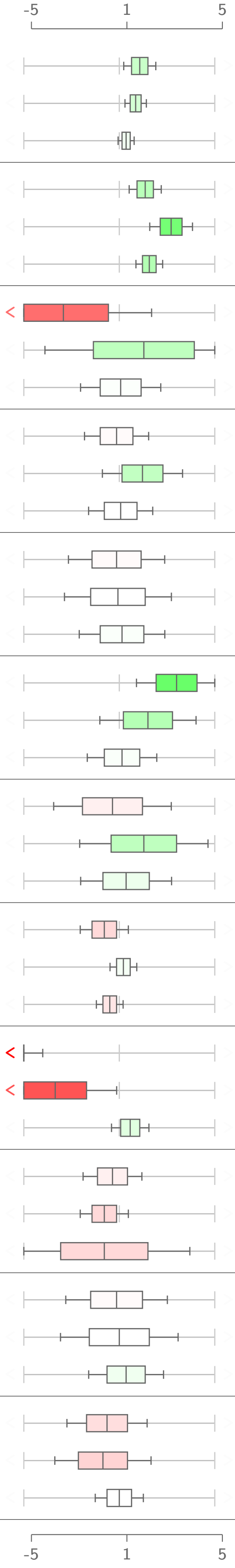

#	protein	group	peptides	spectra	ch1	ch2	quant	ratio
**608**	GOLGB1 Q14789$_{1-4}$: Golgin subfamily B member 1	4/4	2	2	127	126	1	**1.66**
					129	128	1	**1.22**
					131	130	1	**0.75**
**609**	GOLIM4 O00461: Golgi integral membrane protein 4		9	15	127	126	10	**0.65**
					129	128	10	**0.71**
					131	130	10	**1.08**
**610**	GOLM1 Q8NBJ4$_{1,2}$: Golgi membrane protein 1	2/2	6	8	127	126	7	**0.36**
					129	128	8	**0.69**
					131	130	6	**1.00**
**611**	GORASP2 Q9H8Y8$_{1-3}$: Golgi reassembly-stacking protein 2	3/3	2	2	127	126	2	**1.36**
					129	128	2	**2.10**
					131	130	2	**1.53**
**612**	GOT2 P00505$_{1}$: Aspartate aminotransferase, mitochondrial		1	1	127	126	1	**2.87**
					129	128	1	**0.85**
					131	130	1	**1.54**
**613**	GP5 P40197: Platelet glycoprotein V		1	1	127	126	1	**3.39**
					129	128	1	**1.14**
					131	130	1	**0.22**
**614**	GPC6 Q9Y625: Glypican-6 [Cleaved into: Secreted glypican-6]		3	24	127	126	18	**0.78**
					129	128	15	**0.95**
					131	130	19	**0.93**
**615**	GPI P06744$_{1,2}$: Glucose-6-phosphate isomerase	2/2	6	6	127	126	3	**6.57**
					129	128	5	**0.87**
					131	130	5	**1.32**
**616**	GPLD1 P80108$_{1}$: Phosphatidylinositol-glycan-specific phospholipase D		14	22	127	126	21	**0.56**
					129	128	21	**0.70**
					131	130	22	**1.04**
**617**	GPX3 P22352: Glutathione peroxidase 3		9	24	127	126	20	**0.69**
					129	128	23	**1.02**
					131	130	22	**1.11**
**618**	GRHPR Q9UBQ7$_{1}$: Glyoxylate reductase/hydroxypyruvate reductase		3	4	127	126	3	**2.07**
					129	128	4	**2.11**
					131	130	3	**1.72**
**620**	GRN P28799$_{1,3}$: Granulins	2/3	4	4	127	126	3	**1.01**
					129	128	3	**0.81**
					131	130	3	**0.94**

#	protein	group	peptides	spectra	ch1	ch2	quant	ratio
621	GSDMA Q96QA5: Gasdermin-A		7	9	127	126	9	**1.82**
					129	128	9	**1.10**
					131	130	9	**1.02**
622	GSDMD P57764: Gasdermin-D		3	5	127	126	5	**0.98**
					129	128	4	**1.64**
					131	130	3	**1.36**
623	GSN $P06396_1$: Gelsolin	1/3	45	1107	127	126	33	**1.21**
					129	128	33	**1.42**
					131	130	37	**0.66**
624	GSN $P06396_2$: Gelsolin		44	1070	127	126	2	**1.42**
					129	128	2	**1.27**
					131	130	2	**0.69**
627	GSTM3 P21266: Glutathione S-transferase Mu 3		10	17	127	126	15	**1.03**
					129	128	16	**1.65**
					131	130	15	**1.45**
628	GSTM5 P46439: Glutathione S-transferase Mu 5	1/4	4	4	127	126	1	**134.60**
					129	128	1	**1.06**
					131	130	1	**0.72**
629	GSTO1 $P78417_1$: Glutathione S-transferase omega-1	1/3	4	6	127	126	6	**1.11**
					129	128	6	**1.45**
					131	130	5	**0.96**
630	GSTP1 P09211: Glutathione S-transferase P		7	9	127	126	8	**3.16**
					129	128	9	**1.61**
					131	130	8	**0.87**
631	GUCA2A Q02747: Guanylin		2	5	127	126	5	**0.71**
					129	128	5	**1.28**
					131	130	4	**0.87**
632	H1FX Q92522: Histone H1x		2	2	127	126	2	**1.59**
					129	128	2	**2.07**
					131	130	2	**1.64**
633	H2AFJ $Q9BTM1_1$, HIST1H2AC Q93077, HIST1H2AG P0C0S8, . . . : Histone H2A.J, Histone H2A type 1-C, . . .	7/13	5	14	127	126	2	**0.79**
					129	128	2	**1.65**
					131	130	2	**1.39**
634	H2AFV $Q71UI9_{1,2}$, H2AFZ P0C0S5: Histone H2A.V, Histone H2A.Z	3/5	3	4	127	126	1	**1.45**
					129	128	1	**1.96**
					131	130	1	**1.67**

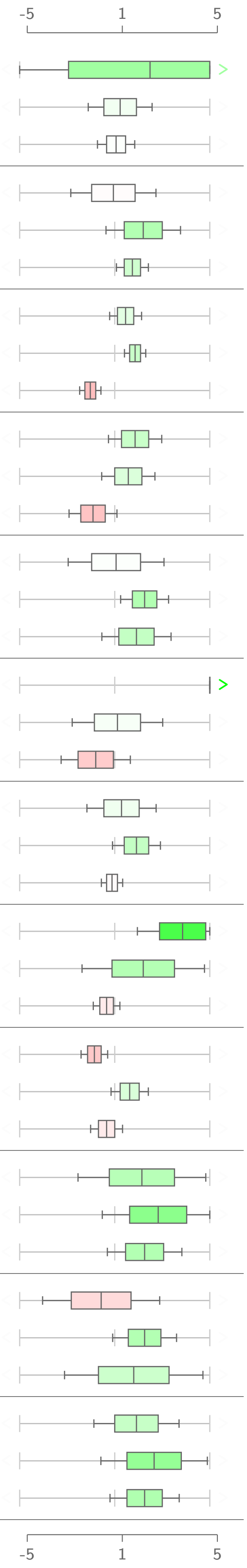

#	protein	group	peptides	spectra	ch1	ch2	quant	ratio	-5 1 5
635	H2AFX P16104: Histone H2AX		6	15	127	126	3	**0.84**	
					129	128	3	**1.20**	
					131	130	3	**1.43**	
636	H3F3A P84243: Histone H3.3		8	22	127	126	1	**1.30**	
					129	128	1	**2.66**	>
					131	130	1	**1.81**	
637	HABP2 Q14520$_{1,2}$: Hyaluronan-binding protein 2	2/2	19	66	127	126	61	**1.21**	
					129	128	57	**0.74**	
					131	130	56	**1.31**	
638	HACD2 Q6Y1H2: Very-long-chain		1	1	127	126	1	**9.02**	>
					129	128	1	**2.36**	
					131	130	1	**1.19**	
639	HADHA P40939$_1$: Trifunctional enzyme subunit alpha, mitochondrial		3	3	127	126	2	**3.32**	
					129	128	3	**1.46**	
					131	130	3	**1.25**	
640	HAL P42357$_{1-3}$: Histidine ammonia-lyase	3/3	2	2	127	126	2	**2.89**	>
					129	128	2	**0.94**	
					131	130	2	**1.02**	
641	HARS P12081$_{1-4}$: Histidine--tRNA ligase, cytoplasmic	4/4	2	2	127	126	2	**1.19**	
					129	128	2	**0.81**	
					131	130	2	**0.75**	
642	HBA1; P69905: Hemoglobin subunit alpha		5	28	127	126	22	**1.04**	
					129	128	26	**18.03**	>
					131	130	26	**1.30**	
643	HBB P68871: Hemoglobin subunit beta	1/2	9	22	127	126	20	**1.36**	
					129	128	18	**18.15**	>
					131	130	21	**1.17**	
644	HDGF P51858$_{1-3}$: Hepatoma-derived growth factor	3/3	8	8	127	126	6	**0.64**	
					129	128	7	**2.28**	
					131	130	6	**1.63**	
645	HDLBP Q00341$_{1,2}$: Vigilin	2/2	4	5	127	126	5	**1.26**	
					129	128	5	**1.67**	
					131	130	4	**1.79**	
646	HEBP1 Q9NRV9: Heme-binding protein 1		1	1	127	126	1	**1.50**	
					129	128	1	**2.53**	
					131	130	1	**0.72**	

-5 1 5

#	protein	group	peptides	spectra	ch1	ch2	quant	ratio
**647**	HERC2 O95714: E3 ubiquitin-protein ligase HERC2		4	14	127	126	4	**0.54**
					129	128	3	**0.63**
					131	130	6	**1.06**
**648**	HEXA $P06865_1$: Beta-hexosaminidase subunit alpha	1/2	8	10	127	126	9	**0.49**
					129	128	9	**0.94**
					131	130	10	**0.75**
**649**	HEXB P07686: Beta-hexosaminidase subunit beta		4	5	127	126	5	**0.61**
					129	128	5	**0.97**
					131	130	3	**0.83**
**650**	HINT1 P49773: Histidine triad nucleotide-binding protein 1		2	3	127	126	3	**0.88**
					129	128	3	**1.43**
					131	130	3	**1.14**
**651**	HIST1H1B P16401: Histone H1.5		9	18	127	126	4	**1.67**
					129	128	5	**2.72**
					131	130	5	**1.36**
**652**	HIST1H1C P16403: Histone H1.2		12	23	127	126	4	**4.59**
					129	128	4	**2.18**
					131	130	4	**1.49**
**653**	HIST1H1D P16402: Histone H1.3		12	23	127	126	4	**4.94**
					129	128	3	**2.07**
					131	130	4	**1.20**
**654**	HIST1H1E P10412: Histone H1.4	1/3	13	27	127	126	5	**1.09**
					129	128	6	**2.00**
					131	130	6	**1.66**
**658**	HIST1H3A P68431, HIST2H3A; Q71DI3, HIST3H3 Q16695: Histone H3.1, Histone H3.2, . . .	3/4	8	22	127	126	1	**1.89**
					129	128	1	**3.20**
					131	130	1	**1.64**
**659**	HIST1H4A P62805: Histone H4		6	16	127	126	13	**0.84**
					129	128	15	**1.55**
					131	130	16	**1.56**
**662**	HLA-A P04439: HLA class I histocompatibility antigen, A-3 alpha chain	1/8	7	10	127	126	2	**5.67**
					129	128	2	**0.52**
					131	130	2	**0.97**
**686**	HLA-B Q31612: HLA class I histocompatibility antigen, B-73 alpha chain		9	12	127	126	2	**0.66**
					129	128	2	**0.81**
					131	130	2	**0.74**

#	protein	group	peptides	spectra	ch1	ch2	quant	ratio
**688**	HLA-C P10321: HLA class I histocompatibility antigen, Cw-7 alpha chain		11	15	127	126	2	**0.07**
					129	128	2	**0.74**
					131	130	2	**0.85**
**690**	HLA-C P30501: HLA class I histocompatibility antigen, Cw-2 alpha chain		9	13	127	126	1	**0.69**
					129	128	1	**1.17**
					131	130	1	**0.70**
**697**	HMCN1 Q96RW7$_{1,2}$: Hemicentin-1	2/2	25	34	127	126	24	**1.12**
					129	128	25	**1.28**
					131	130	27	**0.61**
**698**	HMCN2 Q8NDA2$_{1}$: Hemicentin-2	1/3	16	19	127	126	18	**1.08**
					129	128	17	**1.15**
					131	130	15	**0.83**
**699**	HMGA1 P17096$_{1,2}$: High mobility group protein HMG-I/HMG-Y	2/3	3	3	127	126	3	**1.81**
					129	128	3	**2.06**
					131	130	3	**1.97**
**700**	HMGB1 P09429: High mobility group protein B1	1/2	3	3	127	126	2	**1.65**
					129	128	2	**2.25**
					131	130	2	**1.34**
**701**	HMGN4 O00479: High mobility group nucleosome-binding domain-containing protein 4		2	2	127	126	2	**0.78**
					129	128	2	**2.10**
					131	130	2	**1.43**
**702**	HNMT P50135$_{1}$: Histamine N-methyltransferase		5	5	127	126	4	**1.36**
					129	128	3	**1.66**
					131	130	4	**0.76**
**703**	HNRNPA0 Q13151: Heterogeneous nuclear ribonucleoprotein A0		3	4	127	126	4	**1.53**
					129	128	4	**2.40**
					131	130	3	**1.27**
**704**	HNRNPA1 P09651$_{1-3}$, HNRNPA1L2 Q32P51: Heterogeneous nuclear ribonucleoprotein A1, Heterogeneous nuclear ribonucleoprotein A1-like 2	4/4	4	6	127	126	2	**1.43**
					129	128	2	**2.22**
					131	130	2	**1.40**
**705**	HNRNPA2B1 P22626$_{1,2}$: Heterogeneous nuclear ribonucleoproteins A2/B1	2/2	10	14	127	126	12	**1.61**
					129	128	12	**3.02**
					131	130	12	**1.30**
**706**	HNRNPA3 P51991$_{1}$: Heterogeneous nuclear ribonucleoprotein A3	1/2	9	11	127	126	9	**1.09**
					129	128	9	**2.75**
					131	130	9	**1.55**

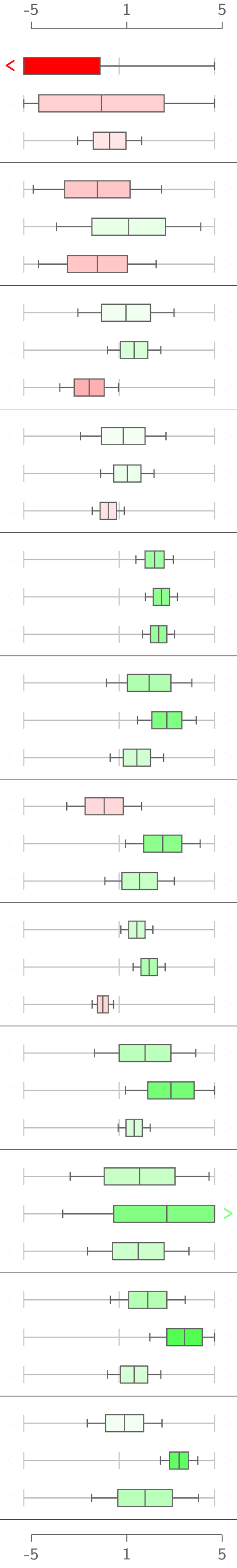

#	protein	group	peptides	spectra	ch1	ch2	quant	ratio
**707**	HNRNPAB Q99729$_{1-4}$: Heterogeneous nuclear ribonucleoprotein A/B	4/4	5	5	127	126	3	**1.73**
					129	128	3	**2.84**
					131	130	3	**1.88**
**708**	HNRNPC P07910$_{2}$: Heterogeneous nuclear ribonucleoproteins C1/C2	1/4	14	20	127	126	10	**3.49**
					129	128	10	**1.99**
					131	130	12	**1.55**
**709**	HNRNPCL1 O60812, HNRNPCL2 B2RXH8: Heterogeneous nuclear ribonucleoprotein C-like 1, Heterogeneous nuclear ribonucleoprotein C-like 2	2/2	6	12	127	126	4	**0.83**
					129	128	4	**1.05**
					131	130	4	**1.36**
**710**	HNRNPCL3 B7ZW38, HNRNPCL4 P0DMR1: Heterogeneous nuclear ribonucleoprotein C-like 3, Heterogeneous nuclear ribonucleoprotein C-like 4	2/2	6	10	127	126	2	**0.43**
					129	128	2	**0.90**
					131	130	2	**2.25**
**711**	HNRNPD Q14103$_{1,3}$: Heterogeneous nuclear ribonucleoprotein D0	2/4	7	10	127	126	7	**0.90**
					129	128	7	**2.43**
					131	130	7	**1.63**
**712**	HNRNPDL O14979$_{1-3}$: Heterogeneous nuclear ribonucleoprotein D-like	3/3	3	3	127	126	1	**0.38**
					129	128	1	**1.99**
					131	130	1	**1.74**
**713**	HNRNPF P52597: Heterogeneous nuclear ribonucleoprotein F		6	12	127	126	4	**1.41**
					129	128	3	**2.62**
					131	130	4	**1.30**
**714**	HNRNPH1 P31943: Heterogeneous nuclear ribonucleoprotein H	1/2	8	17	127	126	8	**1.23**
					129	128	7	**2.66**
					131	130	8	**1.70**
**715**	HNRNPH3 P31942$_{1,2}$: Heterogeneous nuclear ribonucleoprotein H3	2/6	3	3	127	126	2	**0.63**
					129	128	2	**2.50**
					131	130	2	**1.58**
**716**	HNRNPK P61978$_{3}$: Heterogeneous nuclear ribonucleoprotein K	1/3	22	40	127	126	38	**1.11**
					129	128	35	**2.75**
					131	130	38	**2.01**
**717**	HNRNPL P14866$_{1}$: Heterogeneous nuclear ribonucleoprotein L	1/2	7	10	127	126	8	**1.42**
					129	128	10	**2.43**
					131	130	10	**1.59**
**718**	HNRNPM P52272$_{1,2}$: Heterogeneous nuclear ribonucleoprotein M	2/2	11	12	127	126	12	**2.22**
					129	128	11	**2.34**
					131	130	12	**1.33**

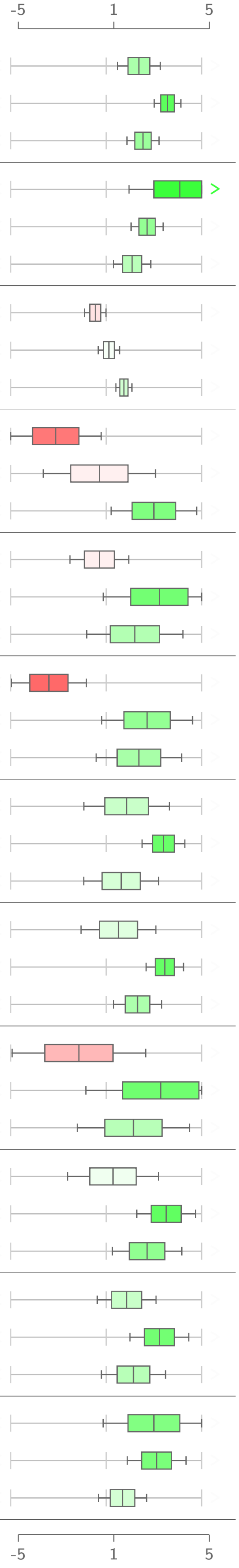

#	protein	group	peptides	spectra	ch1	ch2	quant	ratio
**719**	HNRNPR O43390$_{1-4}$: Heterogeneous nuclear ribonucleoprotein R	4/4	10	10	127	126	3	**1.13**
					129	128	3	**2.65**
					131	130	4	**1.31**
**720**	HNRNPU Q00839$_1$: Heterogeneous nuclear ribonucleoprotein U		15	19	127	126		
					129	128	1	**4.15**
					131	130	1	**3.41**
**721**	HNRNPU Q00839$_2$: Heterogeneous nuclear ribonucleoprotein U		15	20	127	126	2	**0.30**
					129	128	2	**2.92**
					131	130	2	**2.47**
**722**	HNRNPUL1 Q9BUJ2$_{1-4}$: Heterogeneous nuclear ribonucleoprotein U-like protein 1	4/4	2	2	127	126	2	**1.12**
					129	128	2	**2.39**
					131	130	2	**1.55**
**723**	HNRNPUL2 Q1KMD3: Heterogeneous nuclear ribonucleoprotein U-like protein 2		8	11	127	126	11	**0.86**
					129	128	11	**2.30**
					131	130	11	**1.34**
**724**	HP P00738$_1$: Haptoglobin	1/2	24	72	127	126	33	**1.27**
					129	128	28	**0.98**
					131	130	33	**1.26**
**725**	HP1BP3 Q5SSJ5$_{1,2}$: Heterochromatin protein 1-binding protein 3	2/2	2	2	127	126	2	**1.63**
					129	128	2	**1.52**
					131	130	2	**2.04**
**726**	HPR P00739$_{1,2}$: Haptoglobin-related protein	2/2	23	66	127	126	27	**1.35**
					129	128	27	**0.91**
					131	130	23	**0.79**
**727**	HPX P02790: Hemopexin		16	31	127	126	30	**1.05**
					129	128	29	**0.95**
					131	130	28	**0.94**
**728**	HRC P23327: Sarcoplasmic reticulum histidine-rich calcium-binding protein		5	11	127	126	9	**0.44**
					129	128	5	**0.87**
					131	130	9	**0.95**
**729**	HRG P04196: Histidine-rich glycoprotein		24	381	127	126	368	**0.52**
					129	128	341	**0.42**
					131	130	341	**0.96**
**730**	HRNR Q86YZ3: Hornerin		5	6	127	126	6	**0.44**
					129	128	5	**0.85**
					131	130	4	**1.29**

#	protein	group	peptides	spectra	ch1	ch2	quant	ratio
**731**	HSD17B4 P51659$_2$: Peroxisomal multifunctional enzyme type 2	1/3	2	2	127	126	2	**2.63**
					129	128	2	**1.13**
					131	130	2	**0.95**
**732**	HSP90AA1 P07900$_{1,2}$: Heat shock protein HSP 90-alpha	2/8	26	80	127	126	28	**1.22**
					129	128	29	**1.81**
					131	130	28	**1.18**
**733**	HSP90AB1 P08238: Heat shock protein HSP 90-beta	1/2	33	87	127	126	20	**1.37**
					129	128	23	**1.91**
					131	130	21	**1.08**
**734**	HSP90AB3P Q58FF7: Putative heat shock protein HSP 90-beta-3		13	43	127	126	2	**1.04**
					129	128	2	**0.85**
					131	130	2	**0.97**
**735**	HSP90B1 P14625: Endoplasmin	1/2	33	76	127	126	65	**1.10**
					129	128	67	**1.24**
					131	130	62	**1.22**
**736**	HSPA13 P48723: Heat shock 70 kDa protein 13		2	2	127	126	2	**1.21**
					129	128	2	**1.14**
					131	130	2	**0.63**
**737**	HSPA1A P0DMV8$_1$, HSPA1B P0DMV9: Heat shock 70 kDa protein 1A, Heat shock 70 kDa protein 1B	2/5	25	43	127	126	19	**2.53**
					129	128	18	**1.88**
					131	130	19	**1.70**
**739**	HSPA2 P54652: Heat shock-related 70 kDa protein 2		17	27	127	126	8	**0.84**
					129	128	7	**2.10**
					131	130	8	**2.26**
**740**	HSPA4 P34932$_1$: Heat shock 70 kDa protein 4		5	6	127	126	4	**2.33**
					129	128	6	**1.55**
					131	130	5	**1.43**
**741**	HSPA5 P11021: 78 kDa glucose-regulated protein		32	48	127	126	37	**1.35**
					129	128	38	**1.05**
					131	130	37	**1.25**
**742**	HSPA8 P11142$_1$: Heat shock cognate 71 kDa protein	1/2	24	44	127	126	20	**2.55**
					129	128	21	**1.74**
					131	130	22	**1.56**
**743**	HSPA9 P38646: Stress-70 protein, mitochondrial		9	10	127	126	8	**2.56**
					129	128	8	**1.65**
					131	130	7	**1.32**

#	protein	group	peptides	spectra	ch1	ch2	quant	ratio
744	HSPB1 P04792: Heat shock protein beta-1		9	15	127	126	14	**2.40**
					129	128	14	**1.60**
					131	130	15	**1.06**
745	HSPB6 O14558: Heat shock protein beta-6		3	3	127	126	3	**0.61**
					129	128	3	**1.18**
					131	130	3	**1.00**
746	HSPD1 P10809$_1$: 60 kDa heat shock protein, mitochondrial		5	5	127	126	4	**1.26**
					129	128	4	**1.98**
					131	130	4	**1.59**
747	HSPG2 P98160: Basement membrane-specific heparan sulfate proteoglycan core protein		81	123	127	126	115	**0.92**
					129	128	107	**1.29**
					131	130	114	**0.81**
748	HTRA1 Q92743: Serine protease HTRA1		19	58	127	126	47	**1.01**
					129	128	48	**1.13**
					131	130	47	**0.98**
749	HTRA3 P83110$_{1,2}$: Serine protease HTRA3	2/2	3	3	127	126	3	**1.21**
					129	128	3	**1.31**
					131	130	3	**1.15**
750	HUWE1 Q7Z6Z7$_{1-3}$: E3 ubiquitin-protein ligase HUWE1	3/3	3	3	127	126	3	**1.03**
					129	128	3	**1.87**
					131	130	3	**1.03**
751	HYAL1 Q12794$_{1-3}$: Hyaluronidase-1	3/4	5	11	127	126	5	**0.92**
					129	128	4	**0.87**
					131	130	6	**0.89**
752	HYOU1 Q9Y4L1$_1$: Hypoxia up-regulated protein 1	1/2	15	16	127	126	15	**1.23**
					129	128	15	**1.22**
					131	130	15	**1.02**
753	IDE P14735$_1$: Insulin-degrading enzyme	1/2	16	20	127	126	19	**4.80**
					129	128	18	**1.10**
					131	130	19	**0.97**
754	IFI30 P13284: Gamma-interferon-inducible lysosomal thiol reductase		4	8	127	126	7	**0.92**
					129	128	7	**0.64**
					131	130	8	**0.89**
755	IFT140 Q96RY7$_1$: Intraflagellar transport protein 140 homolog		4	30	127	126	26	**0.76**
					129	128	22	**1.19**
					131	130	26	**0.88**

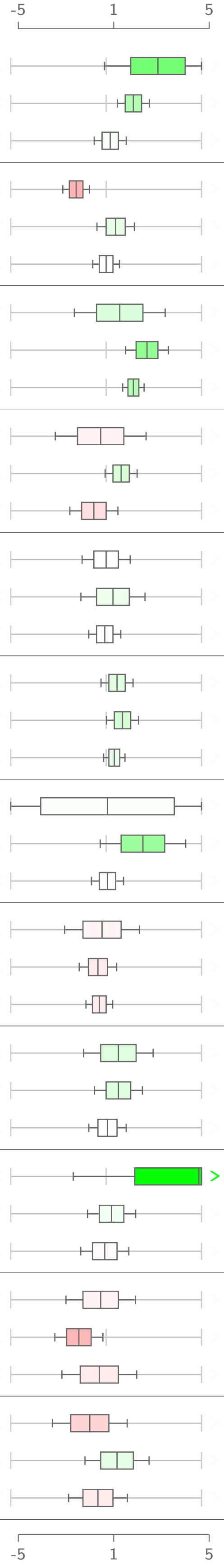

#	protein	group	peptides	spectra	ch1	ch2	quant	ratio
**756**	IGF1 P05019$_1$: Insulin-like growth factor I	1/4	6	10	127	126	10	**0.59**
					129	128	10	**0.85**
					131	130	9	**0.95**
**757**	IGF1R P08069: Insulin-like growth factor 1 receptor		6	10	127	126	6	**0.68**
					129	128	5	**1.23**
					131	130	6	**0.80**
**758**	IGF2 P01344$_{1-3}$: Insulin-like growth factor II	3/3	5	8	127	126	8	**0.51**
					129	128	7	**0.84**
					131	130	8	**0.79**
**759**	IGF2R P11717: Cation-independent mannose-6-phosphate receptor		10	10	127	126	8	**0.83**
					129	128	8	**1.04**
					131	130	8	**1.05**
**760**	IGFALS P35858$_{1,2}$: Insulin-like growth factor-binding protein complex acid labile subunit	2/2	15	26	127	126	24	**0.95**
					129	128	23	**0.96**
					131	130	25	**0.88**
**761**	IGFBP1 P08833: Insulin-like growth factor-binding protein 1		8	15	127	126	11	**0.63**
					129	128	15	**1.60**
					131	130	14	**1.15**
**762**	IGFBP2 P18065: Insulin-like growth factor-binding protein 2		10	18	127	126	16	**0.47**
					129	128	17	**1.00**
					131	130	18	**0.84**
**763**	IGFBP3 P17936$_{1,2}$: Insulin-like growth factor-binding protein 3	2/2	18	35	127	126	28	**0.67**
					129	128	32	**0.78**
					131	130	32	**1.03**
**764**	IGFBP4 P22692$_1$: Insulin-like growth factor-binding protein 4	1/2	11	15	127	126	14	**0.53**
					129	128	13	**0.68**
					131	130	14	**0.82**
**765**	IGFBP5 P24593: Insulin-like growth factor-binding protein 5		14	37	127	126	33	**0.54**
					129	128	35	**0.75**
					131	130	34	**0.81**
**766**	IGFBP6 P24592: Insulin-like growth factor-binding protein 6		7	14	127	126	12	**0.59**
					129	128	13	**1.24**
					131	130	13	**0.87**
**767**	IGFBP7 Q16270$_{1,2}$: Insulin-like growth factor-binding protein 7	2/2	11	17	127	126	17	**0.53**
					129	128	17	**1.00**
					131	130	17	**1.02**

#	protein	group	peptides	spectra	ch1	ch2	quant	ratio
768	IGHA1 P01876: Ig alpha-1 chain C region		13	131	127	126	43	**0.91**
					129	128	36	**0.99**
					131	130	43	**1.05**
769	IGHA2 P01877: Ig alpha-2 chain C region		9	90	127	126	2	**0.88**
					129	128	2	**1.22**
					131	130	2	**0.92**
770	IGHD $P01880_{1,2}$: Ig delta chain C region	2/2	9	14	127	126	13	**1.10**
					129	128	10	**0.98**
					131	130	12	**0.92**
771	IGHG1 P01857: Ig gamma-1 chain C region		16	472	127	126	192	**0.85**
					129	128	186	**0.94**
					131	130	191	**0.87**
772	IGHG2 P01859: Ig gamma-2 chain C region		14	210	127	126	30	**1.08**
					129	128	24	**0.98**
					131	130	26	**0.92**
773	IGHG3 P01860: Ig gamma-3 chain C region		20	383	127	126	114	**0.82**
					129	128	116	**0.99**
					131	130	117	**1.04**
774	IGHG4 P01861: Ig gamma-4 chain C region		11	155	127	126	17	**1.09**
					129	128	17	**1.16**
					131	130	16	**0.83**
775	IGHM $P01871_{1,2}$: Ig mu chain C region	2/2	21	150	127	126	61	**0.63**
					129	128	61	**0.68**
					131	130	58	**1.17**
776	IGHV1-2 P23083, A0A0C4DH31: Immunoglobulin heavy variable 1-2	2/2	2	6	127	126	5	**0.65**
					129	128	4	**0.88**
					131	130	4	**1.09**
777	IGHV1-69 P01742: Immunoglobulin heavy variable 1-69		3	5	127	126	2	**0.99**
					129	128	2	**1.27**
					131	130	2	**0.84**
778	IGHV3-13 P01766: Immunoglobulin heavy variable 3-13		1	1	127	126	1	**0.94**
					129	128	1	**0.80**
					131	130	1	**0.98**
779	IGHV3-30 P01768: Immunoglobulin heavy variable 3-30	1/2	2	9	127	126	4	**0.80**
					129	128	6	**0.67**
					131	130	6	**1.09**

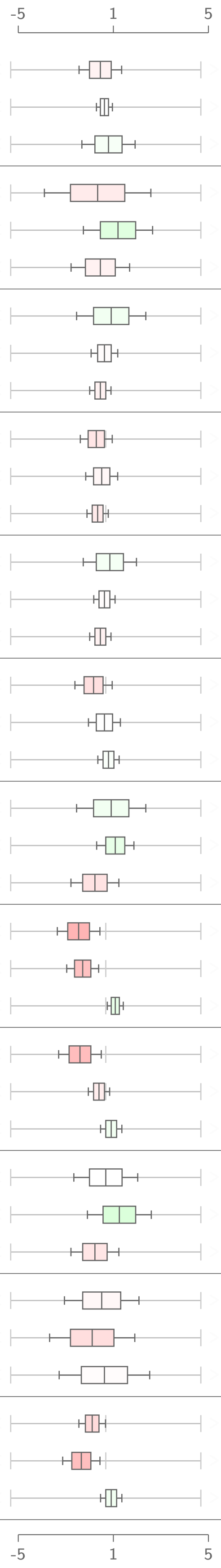

#	protein	group	peptides	spectra	ch1	ch2	quant	ratio
**781**	IGHV3-7 P01780: Immunoglobulin heavy variable 3-7	1/5	3	6	127	126	4	**0.78**
					129	128	3	**0.81**
					131	130	4	**1.01**
**782**	IGHV3-9 P01782: Immunoglobulin heavy variable 3-9		1	2	127	126	2	**0.93**
					129	128	2	**0.78**
					131	130	2	**1.12**
**783**	IGHV4-39 P01824: Immunoglobulin heavy variable 4-39	1/3	4	15	127	126	15	**1.18**
					129	128	15	**0.87**
					131	130	15	**0.85**
**784**	IGKC P01834: Ig kappa chain C region		6	167	127	126	162	**0.88**
					129	128	149	**1.02**
					131	130	161	**0.91**
**785**	IGKV1-17 P01599: Immunoglobulin kappa variable 1-17		3	13	127	126	3	**0.72**
					129	128	3	**0.83**
					131	130	3	**0.86**
**786**	IGKV1D-33 P01593, IGKV1-33 P01594: Immunoglobulin kappa variable 1D-33, Immunoglobulin kappa variable 1-33	2/2	1	1	127	126	1	**0.78**
					129	128	1	**1.00**
					131	130	1	**1.06**
**787**	IGKV1D-12 P01611, IGKV1D-39 P04432, IGKV1-39 P01597, . . . : Immunoglobulin kappa variable 1D-12, Immunoglobulin kappa variable 1D-39, . . .	4/4	2	11	127	126	1	**1.60**
					129	128	1	**1.58**
					131	130	1	**0.66**
**788**	IGKV1-5 P01602: Immunoglobulin kappa variable 1-5		2	12	127	126	2	**0.72**
					129	128	2	**0.83**
					131	130	2	**0.88**
**789**	IGKV2-30 P06310: Immunoglobulin kappa variable 2-30		2	24	127	126	12	**0.76**
					129	128	14	**0.93**
					131	130	13	**1.08**
**790**	IGKV2D-40 P01614: Immunoglobulin kappa variable 2D-40	1/4	3	29	127	126	20	**0.71**
					129	128	17	**0.94**
					131	130	18	**0.99**
**791**	IGKV3-11 P04433: Immunoglobulin kappa variable 3-11		3	23	127	126	2	**0.88**
					129	128	2	**1.14**
					131	130	2	**1.03**
**792**	IGKV3-15 P01624: Immunoglobulin kappa variable 3-15		4	25	127	126	3	**0.95**
					129	128	4	**0.96**
					131	130	4	**0.81**

#	protein	group	peptides	spectra	ch1	ch2	quant	ratio
**793**	IGKV3-20 P01619: Immunoglobulin kappa variable 3-20		4	25	127	126	1	**0.74**
					129	128	1	**0.88**
					131	130	1	**0.98**
**794**	IGKV4-1 P06312: Immunoglobulin kappa variable 4-1		5	29	127	126	25	**0.71**
					129	128	25	**0.81**
					131	130	29	**0.89**
**795**	IGLC2 P0CG05: Ig lambda-2 chain C regions	1/2	7	263	127	126	92	**0.70**
					129	128	89	**0.89**
					131	130	92	**0.92**
**797**	IGLC7 A0M8Q6: Ig lambda-7 chain C region		5	125	127	126	1	**0.95**
					129	128		
					131	130	1	**0.88**
**798**	IGLL1 P15814$_1$: Immunoglobulin lambda-like polypeptide 1		2	4	127	126	2	**0.36**
					129	128	2	**0.79**
					131	130	2	**1.44**
**799**	IGLL5 B9A064$_1$: Immunoglobulin lambda-like polypeptide 5	1/2	7	169	127	126	33	**0.75**
					129	128	35	**0.90**
					131	130	39	**0.86**
**800**	IGLV1-40 P01703: Immunoglobulin lambda variable 1-40		1	1	127	126	1	**1.54**
					129	128	1	**0.99**
					131	130	1	**1.29**
**801**	IGLV1-47 P01700: Immunoglobulin lambda variable 1-47	1/2	2	4	127	126	4	**1.16**
					129	128	4	**0.94**
					131	130	3	**0.83**
**802**	IGLV2-23 P01705: Immunoglobulin lambda variable 2-23		2	2	127	126	2	**0.78**
					129	128	2	**0.71**
					131	130	2	**0.78**
**803**	IGLV3-19 P01714: Immunoglobulin lambda variable 3-19		2	2	127	126	2	**0.79**
					129	128	2	**0.77**
					131	130	2	**0.74**
**804**	IGLV3-21 P80748: Immunoglobulin lambda variable 3-21		2	7	127	126	6	**0.70**
					129	128	6	**0.83**
					131	130	5	**1.08**
**805**	IGLV3-25 P01717: Immunoglobulin lambda variable 3-25		2	4	127	126	2	**0.74**
					129	128	2	**0.91**
					131	130	2	**0.87**

#	protein	group	peptides	spectra	ch1	ch2	quant	ratio
806	IGLV3-27 P01718: Immunoglobulin lambda variable 3-27		2	3	127	126	1	**0.52**
					129	128	1	**0.84**
					131	130	1	**0.98**
807	IGLV7-43 P04211: Immunoglobulin lambda variable 7-43		3	4	127	126	4	**0.95**
					129	128	4	**0.92**
					131	130	4	**0.84**
808	IGSF8 $Q969P0_{1,3}$: Immunoglobulin superfamily member 8	2/2	1	1	127	126	1	**0.59**
					129	128	1	**1.38**
					131	130	1	**4.41**
809	IKBIP $Q70UQ0_{4}$: Inhibitor of nuclear factor kappa-B kinase-interacting protein		2	24	127	126	14	**0.82**
					129	128	18	**0.83**
					131	130	18	**1.13**
810	IL36G $Q9NZH8_{1,2}$: Interleukin-36 gamma	2/2	3	3	127	126	2	**5.55**
					129	128	3	**0.75**
					131	130	3	**1.02**
811	ILF3 $Q12906_{4}$: Interleukin enhancer-binding factor 3	1/7	9	11	127	126	10	**1.41**
					129	128	9	**2.00**
					131	130	9	**1.10**
812	ILK $Q13418_{1-3}$: Integrin-linked protein kinase	3/3	1	1	127	126	1	**1.36**
					129	128	1	**2.34**
					131	130	1	**1.18**
813	IMPAD1 Q9NX62: Inositol monophosphatase 3		7	9	127	126	7	**1.82**
					129	128	9	**0.99**
					131	130	9	**0.95**
814	INHBA P08476: Inhibin beta A chain		4	4	127	126	4	**0.38**
					129	128	4	**0.84**
					131	130	3	**0.62**
815	INHBB P09529: Inhibin beta B chain		5	7	127	126	6	**0.96**
					129	128	6	**1.30**
					131	130	7	**0.83**
816	INHBC P55103: Inhibin beta C chain		8	16	127	126	16	**0.90**
					129	128	16	**1.00**
					131	130	16	**1.14**
817	INHBE P58166: Inhibin beta E chain		3	5	127	126	5	**0.99**
					129	128	4	**1.08**
					131	130	4	**0.92**

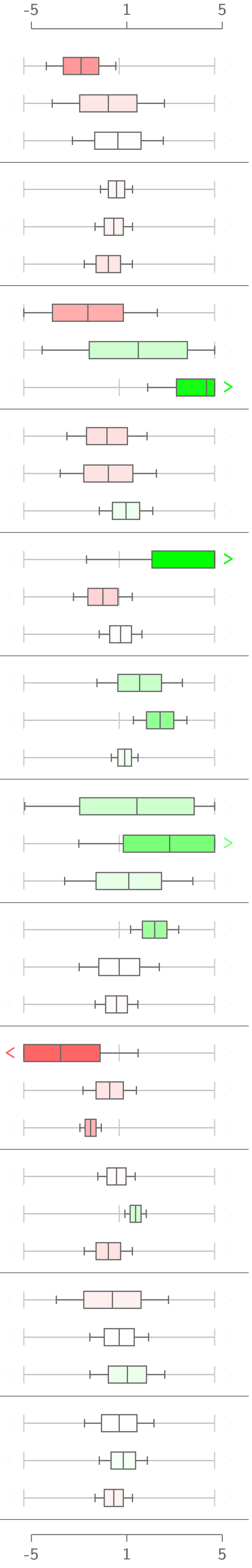

#	protein	group	peptides	spectra	ch1	ch2	quant	ratio
**818**	IPO5 O00410$_{1,3}$: Importin-5	2/3	7	7	127	126	7	**1.03**
					129	128	6	**2.26**
					131	130	6	**1.00**
**819**	IPO7 O95373: Importin-7		1	1	127	126	1	**1.08**
					129	128	1	**1.51**
					131	130	1	**1.21**
**820**	IQGAP1 P46940: Ras GTPase-activating-like protein IQGAP1		28	32	127	126	31	**1.13**
					129	128	31	**1.60**
					131	130	28	**0.91**
**821**	ISLR O14498: Immunoglobulin superfamily containing leucine-rich repeat protein		7	17	127	126	14	**0.68**
					129	128	17	**1.01**
					131	130	15	**0.55**
**822**	ITGAM P11215$_{1,2}$: Integrin alpha-M	2/2	3	3	127	126	3	**0.81**
					129	128	3	**1.18**
					131	130	3	**0.76**
**823**	ITGB1 P05556$_{1}$: Integrin beta-1	1/5	12	19	127	126	12	**1.18**
					129	128	9	**1.16**
					131	130	15	**0.71**
**824**	ITGB2 P05107: Integrin beta-2		3	3	127	126	3	**1.10**
					129	128	3	**1.37**
					131	130	3	**0.61**
**825**	ITGB8 P26012$_{1,2}$: Integrin beta-8	2/2	5	18	127	126	14	**0.78**
					129	128	15	**0.97**
					131	130	17	**1.14**
**826**	ITGBL1 O95965$_{1}$: Integrin beta-like protein 1	1/3	17	48	127	126	21	**0.63**
					129	128	17	**0.78**
					131	130	20	**1.12**
**827**	ITIH1 P19827$_{1}$: Inter-alpha-trypsin inhibitor heavy chain H1	1/3	27	197	127	126	188	**1.86**
					129	128	181	**1.08**
					131	130	193	**0.65**
**828**	ITIH2 P19823: Inter-alpha-trypsin inhibitor heavy chain H2		32	398	127	126	387	**2.11**
					129	128	372	**1.02**
					131	130	380	**0.72**
**829**	ITIH3 Q06033$_{1,2}$: Inter-alpha-trypsin inhibitor heavy chain H3	2/2	35	155	127	126	151	**2.73**
					129	128	144	**0.96**
					131	130	149	**0.88**

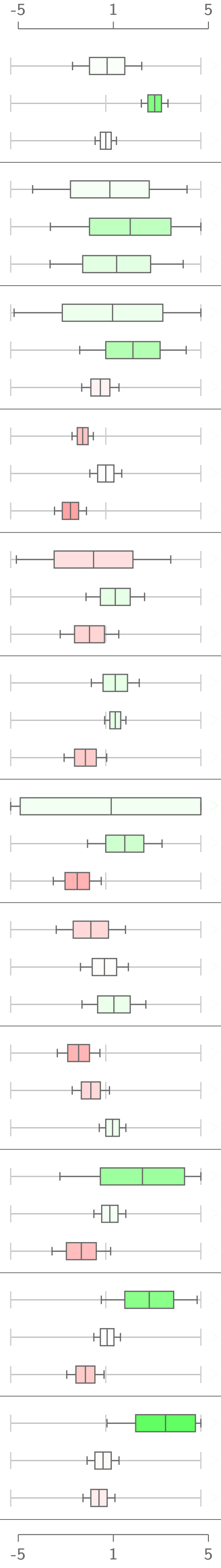

#	protein	group	peptides	spectra	ch1	ch2	quant	ratio
830	ITIH4 Q14624$_{1}$: Inter-alpha-trypsin inhibitor heavy chain H4		39	405	127	126	22	**0.91**
					129	128	25	**0.95**
					131	130	26	**1.10**
831	ITIH4 Q14624$_{2,3}$: Inter-alpha-trypsin inhibitor heavy chain H4	2/3	36	382	127	126	4	**1.48**
					129	128	4	**1.25**
					131	130	4	**0.60**
832	ITIH5 Q86UX2$_{1}$: Inter-alpha-trypsin inhibitor heavy chain H5	1/4	16	18	127	126	18	**0.91**
					129	128	18	**1.12**
					131	130	18	**0.78**
833	ITLN1 Q8WWA0: Intelectin-1	1/3	16	330	127	126	301	**0.36**
					129	128	320	**0.64**
					131	130	299	**0.84**
834	ITM2A O43736$_{1,2}$: Integral membrane protein 2A	2/2	2	2	127	126	2	**0.65**
					129	128	2	**0.88**
					131	130	2	**1.01**
835	ITM2B Q9Y287$_{1,2}$: Integral membrane protein 2B	2/2	2	4	127	126	4	**0.61**
					129	128	4	**0.86**
					131	130	4	**0.72**
836	ITPA Q9BY32$_{1}$: Inosine triphosphate pyrophosphatase		1	1	127	126	1	**0.84**
					129	128	1	**1.27**
					131	130	1	**1.04**
837	IVL P07476: Involucrin		13	14	127	126	12	**6.48**
					129	128	14	**1.05**
					131	130	13	**0.97**
838	JCHAIN P01591: Immunoglobulin J chain		5	12	127	126	12	**0.65**
					129	128	11	**0.76**
					131	130	10	**1.02**
839	JUP P14923: Junction plakoglobin		22	34	127	126	26	**1.10**
					129	128	27	**0.92**
					131	130	29	**0.89**
840	KAZALD1 Q96I82$_{1,2}$: Kazal-type serine protease inhibitor domain-containing protein 1	2/2	1	1	127	126	1	**0.43**
					129	128	1	**0.58**
					131	130	1	**0.26**
841	KCTD12 Q96CX2: BTB/POZ domain-containing protein KCTD12	1/2	10	12	127	126	11	**1.50**
					129	128	12	**2.10**
					131	130	11	**0.60**

#	protein	group	peptides	spectra	ch1	ch2	quant	ratio
**842**	KDM3A Q9Y4C1: Lysine-specific demethylase 3A		4	5	127	126	3	**0.63**
					129	128	3	**0.87**
					131	130	3	**0.58**
**843**	KHDRBS1 Q07666$_{1,2}$: KH domain-containing, RNA-binding, signal transduction-associated protein 1	2/3	3	4	127	126	4	**1.53**
					129	128	4	**3.01**
					131	130	4	**1.34**
**844**	KHSRP Q92945: Far upstream element-binding protein 2		5	5	127	126	2	**1.28**
					129	128	2	**3.01**
					131	130	2	**1.64**
**845**	KIAA0319L Q8IZA0$_{1,2}$: Dyslexia-associated protein KIAA0319-like protein	2/2	2	2	127	126	2	**0.54**
					129	128	2	**0.80**
					131	130	2	**1.02**
**846**	KIAA0754 O94854$_{1,2}$: Uncharacterized protein KIAA0754	2/2	2	24	127	126	11	**0.62**
					129	128	7	**1.22**
					131	130	12	**0.85**
**847**	KIF15 Q9NS87$_{1,2,4}$: Kinesin-like protein KIF15	3/3	2	2	127	126	1	**0.60**
					129	128	1	**0.83**
					131	130	1	**1.11**
**848**	KIF5B P33176: Kinesin-1 heavy chain		8	8	127	126	5	**1.43**
					129	128	5	**1.79**
					131	130	3	**1.12**
**849**	KIF5C O60282$_{1}$: Kinesin heavy chain isoform 5C		4	4	127	126	1	**1.13**
					129	128	1	**1.11**
					131	130	1	**1.43**
**850**	KLC1 Q07866$_{1-10}$: Kinesin light chain 1	10/10	3	3	127	126	3	**1.27**
					129	128	3	**2.07**
					131	130	3	**1.38**
**851**	KLK10 O43240: Kallikrein-10		4	4	127	126	4	**0.67**
					129	128	4	**1.11**
					131	130	4	**0.87**
**852**	KLK6 Q92876$_{1}$: Kallikrein-6	1/2	4	5	127	126	3	**2.69**
					129	128	5	**1.01**
					131	130	5	**0.99**
**853**	KLK7 P49862$_{1,2}$: Kallikrein-7	2/2	2	2	127	126	2	**2.55**
					129	128	2	**0.76**
					131	130	2	**1.01**

#	protein	group	peptides	spectra	ch1	ch2	quant	ratio
854	KLKB1 P03952: Plasma kallikrein		36	95	127	126	88	0.88
					129	128	87	0.99
					131	130	79	1.20
855	KMT2A $Q03164_{1-3}$: Histone-lysine N-methyltransferase 2A	3/3	7	37	127	126	12	0.82
					129	128	10	0.60
					131	130	20	1.15
856	KNG1 $P01042_1$: Kininogen-1		34	350	127	126	29	1.02
					129	128	30	0.71
					131	130	30	1.07
857	KNG1 $P01042_2$: Kininogen-1	1/2	27	350	127	126	29	0.92
					129	128	26	1.50
					131	130	30	0.92
858	KPNB1 $Q14974_1$: Importin subunit beta-1	1/2	10	12	127	126	10	1.27
					129	128	11	2.16
					131	130	10	1.12
859	KPRP Q5T749: Keratinocyte proline-rich protein		10	13	127	126	13	7.62
					129	128	12	0.89
					131	130	13	0.93
860	KRT1 P04264: Keratin, type II cytoskeletal 1		48	368	127	126	277	4.15
					129	128	296	0.56
					131	130	289	1.03
861	KRT10 P13645: Keratin, type I cytoskeletal 10		37	409	127	126	247	5.02
					129	128	235	0.55
					131	130	249	1.09
862	KRT13 $P13646_1$: Keratin, type I cytoskeletal 13	1/3	15	81	127	126	2	1.11
					129	128	2	1.28
					131	130	2	0.38
863	KRT14 P02533: Keratin, type I cytoskeletal 14		36	167	127	126	21	7.41
					129	128	24	0.41
					131	130	21	1.05
864	KRT15 $P19012_1$: Keratin, type I cytoskeletal 15	1/2	16	101	127	126	14	0.61
					129	128	13	1.22
					131	130	15	0.91
865	KRT16 P08779: Keratin, type I cytoskeletal 16	1/3	40	157	127	126	44	3.18
					129	128	50	0.23
					131	130	45	1.12

#	protein	group	peptides	spectra	ch1	ch2	quant	ratio
**866**	KRT17 Q04695: Keratin, type I cytoskeletal 17		35	131	127	126	25	**4.25**
					129	128	27	**0.47**
					131	130	29	**1.01**
**867**	KRT18 P05783: Keratin, type I cytoskeletal 18		27	91	127	126	53	**0.61**
					129	128	54	**3.20**
					131	130	55	**3.41**
**868**	KRT19 P08727: Keratin, type I cytoskeletal 19	1/2	37	135	127	126	57	**0.50**
					129	128	60	**3.08**
					131	130	61	**3.36**
**869**	KRT2 P35908: Keratin, type II cytoskeletal 2 epidermal		34	128	127	126	60	**0.68**
					129	128	60	**0.50**
					131	130	58	**0.98**
**871**	KRT23 Q9C075$_1$: Keratin, type I cytoskeletal 23	1/2	8	21	127	126	4	**5.62**
					129	128	4	**0.88**
					131	130	5	**1.05**
**872**	KRT24 Q2M2I5: Keratin, type I cytoskeletal 24		5	54	127	126	4	**0.47**
					129	128	4	**0.61**
					131	130	4	**1.13**
**873**	KRT25 Q7Z3Z0: Keratin, type I cytoskeletal 25	1/2	8	63	127	126	3	**0.49**
					129	128	3	**0.64**
					131	130	3	**1.08**
**874**	KRT27 Q7Z3Y8: Keratin, type I cytoskeletal 27		8	86	127	126	1	**0.54**
					129	128	1	**0.74**
					131	130	1	**0.90**
**875**	KRT28 Q7Z3Y7: Keratin, type I cytoskeletal 28		10	111	127	126	2	**0.65**
					129	128	2	**0.68**
					131	130	2	**1.36**
**877**	KRT31 Q15323: Keratin, type I cuticular Ha1		18	174	127	126	15	**0.08**
					129	128	17	**0.46**
					131	130	17	**0.49**
**878**	KRT32 Q14532: Keratin, type I cuticular Ha2		9	81	127	126	2	**0.02**
					129	128	1	**1.96**
					131	130	2	**1.11**
**879**	KRT33A O76009: Keratin, type I cuticular Ha3-I		16	131	127	126	1	**0.68**
					129	128	1	**1.63**
					131	130	1	**0.37**

#	protein	group	peptides	spectra	ch1	ch2	quant	ratio
**880**	KRT33B Q14525: Keratin, type I cuticular Ha3-II		11	124	127	126	5	**0.01**
					129	128	3	**0.74**
					131	130	5	**0.99**
**881**	KRT34 O76011: Keratin, type I cuticular Ha4		11	129	127	126	3	**0.45**
					129	128	3	**1.17**
					131	130	3	**1.06**
**882**	KRT35 Q92764: Keratin, type I cuticular Ha5		19	93	127	126	26	**0.02**
					129	128	17	**0.50**
					131	130	29	**1.14**
**883**	KRT36 $O76013_{1,2}$: Keratin, type I cuticular Ha6	2/2	12	112	127	126	19	**0.02**
					129	128	14	**0.66**
					131	130	23	**1.00**
**886**	KRT39 Q6A163: Keratin, type I cytoskeletal 39		2	3	127	126	1	**0.06**
					129	128	1	**0.41**
					131	130	1	**1.12**
**888**	KRT40 Q6A162: Keratin, type I cytoskeletal 40		5	43	127	126	1	**0.21**
					129	128	1	**0.57**
					131	130	1	**0.88**
**889**	KRT5 P13647: Keratin, type II cytoskeletal 5		43	198	127	126	75	**4.05**
					129	128	83	**0.74**
					131	130	82	**1.58**
**890**	KRT6A P02538: Keratin, type II cytoskeletal 6A		48	188	127	126	9	**9.65**
					129	128	11	**0.41**
					131	130	11	**0.97**
**891**	KRT6B P04259: Keratin, type II cytoskeletal 6B		46	184	127	126	2	**2.97**
					129	128	2	**1.09**
					131	130	2	**2.18**
**893**	KRT7 P08729: Keratin, type II cytoskeletal 7		33	102	127	126	47	**0.81**
					129	128	53	**2.63**
					131	130	54	**3.07**
**894**	KRT71 Q3SY84: Keratin, type II cytoskeletal 71	1/2	11	20	127	126	6	**0.50**
					129	128	6	**0.77**
					131	130	6	**1.13**
**899**	KRT77 Q7Z794: Keratin, type II cytoskeletal 1b		6	28	127	126	1	**2.76**
					129	128	1	**0.75**
					131	130	1	**1.21**

#	protein	group	peptides	spectra	ch1	ch2	quant	ratio
**900**	KRT78 Q8N1N4$_1$: Keratin, type II cytoskeletal 78	1/2	15	50	127	126	14	**2.81**
					129	128	12	**0.85**
					131	130	14	**1.17**
**901**	KRT79 Q5XKE5: Keratin, type II cytoskeletal 79		10	55	127	126	1	**5.36**
					129	128	1	**1.34**
					131	130	1	**2.05**
**902**	KRT8 P05787$_{1,2}$: Keratin, type II cytoskeletal 8	2/2	38	164	127	126	87	**0.51**
					129	128	89	**2.77**
					131	130	89	**2.82**
**903**	KRT80 Q6KB66$_{1,2}$: Keratin, type II cytoskeletal 80	2/3	9	9	127	126	8	**1.76**
					129	128	7	**0.86**
					131	130	8	**0.99**
**904**	KRT82 Q9NSB4: Keratin, type II cuticular Hb2		8	99	127	126	2	**0.07**
					129	128	2	**0.55**
					131	130	2	**0.92**
**906**	KRT84 Q9NSB2: Keratin, type II cuticular Hb4		5	28	127	126	1	**14.62**
					129	128		
					131	130	2	**1.25**
**907**	KRT85 P78386: Keratin, type II cuticular Hb5		28	338	127	126	16	**0.03**
					129	128	12	**0.49**
					131	130	15	**1.09**
**908**	KRT86 O43790: Keratin, type II cuticular Hb6	1/2	28	372	127	126	1	**0.05**
					129	128	1	**0.32**
					131	130	1	**1.09**
**909**	KRT9 P35527: Keratin, type I cytoskeletal 9		30	130	127	126	98	**0.21**
					129	128	99	**0.49**
					131	130	99	**0.98**
**910**	KRTAP11-1 Q8IUC1: Keratin-associated protein 11-1		1	2	127	126	2	**0.24**
					129	128	1	**1.01**
					131	130	2	**1.20**
**911**	KTN1 Q86UP2$_{1-4}$: Kinectin	4/4	2	2	127	126	2	**2.18**
					129	128	2	**1.65**
					131	130	2	**0.63**
**912**	LAMA2 P24043: Laminin subunit alpha-2		28	48	127	126	35	**0.51**
					129	128	34	**1.11**
					131	130	39	**1.00**

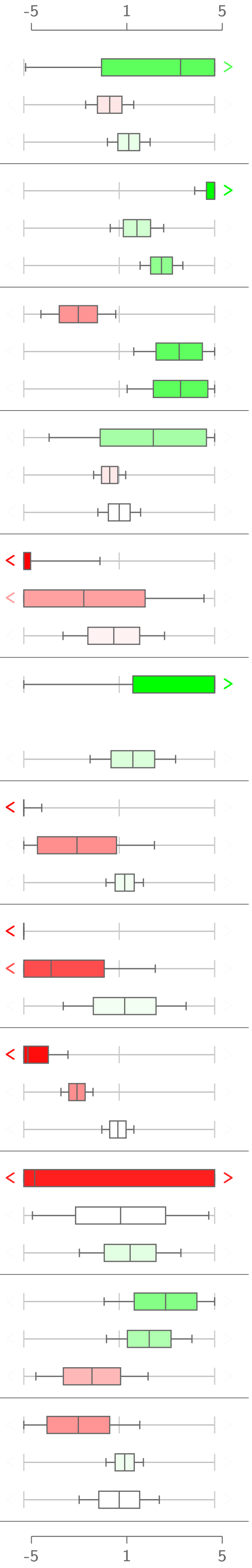

#	protein	group	peptides	spectra	ch1	ch2	quant	ratio
**913**	LAMA4 Q16363$_{1,2}$: Laminin subunit alpha-4	2/2	35	40	127	126	36	**0.98**
					129	128	32	**1.30**
					131	130	37	**0.82**
**914**	LAMA5 O15230$_{1}$: Laminin subunit alpha-5		10	17	127	126	11	**0.84**
					129	128	10	**1.15**
					131	130	13	**1.07**
**915**	LAMB1 P07942: Laminin subunit beta-1		34	41	127	126	34	**0.70**
					129	128	35	**1.15**
					131	130	37	**0.83**
**916**	LAMB2 P55268: Laminin subunit beta-2		28	34	127	126	30	**0.64**
					129	128	31	**1.20**
					131	130	29	**0.80**
**917**	LAMB3 Q13751: Laminin subunit beta-3		4	4	127	126	3	**0.51**
					129	128	3	**0.61**
					131	130	3	**0.97**
**918**	LAMC1 P11047: Laminin subunit gamma-1		42	61	127	126	47	**0.68**
					129	128	45	**1.17**
					131	130	53	**0.78**
**919**	LAMC2 Q13753$_{1,2}$: Laminin subunit gamma-2	2/2	7	9	127	126	9	**0.55**
					129	128	9	**0.98**
					131	130	6	**1.07**
**920**	LAMP1 P11279$_{1}$: Lysosome-associated membrane glycoprotein 1		2	2	127	126	2	**6.22**
					129	128	2	**1.14**
					131	130	2	**1.21**
**921**	LAP3 P28838$_{1,2}$: Cytosol aminopeptidase	2/2	3	3	127	126	3	**0.58**
					129	128	3	**1.00**
					131	130	3	**1.03**
**922**	LASP1 Q14847$_{1}$: LIM and SH3 domain protein 1	1/3	5	5	127	126	5	**0.70**
					129	128	5	**1.10**
					131	130	3	**0.92**
**923**	LBP P18428: Lipopolysaccharide-binding protein		15	347	127	126	335	**2.05**
					129	128	321	**1.32**
					131	130	335	**1.24**
**924**	LCAT P04180: Phosphatidylcholine-sterol acyltransferase	1/2	11	45	127	126	42	**0.99**
					129	128	43	**1.02**
					131	130	44	**1.01**

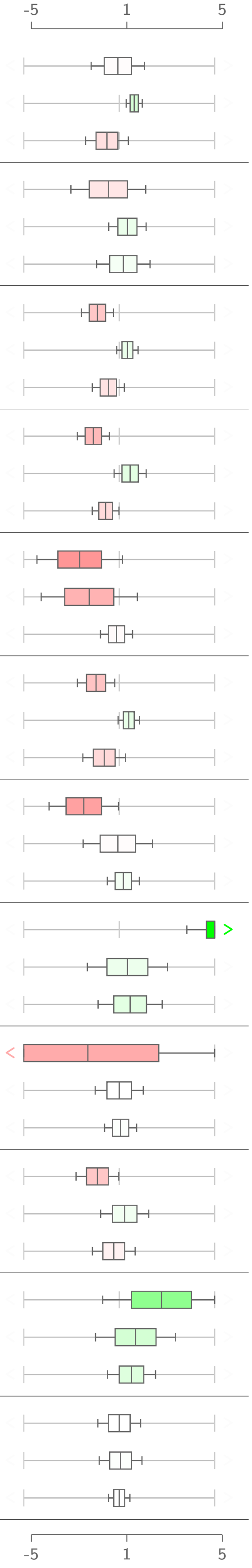

#	protein	group	peptides	spectra	ch1	ch2	quant	ratio
**925**	LCN1 P31025, LCN1P1 Q5VSP4: Lipocalin-1, Putative lipocalin 1-like protein 1	2/2	2	2	127	126	2	**0.87**
					129	128	2	**1.62**
					131	130	2	**0.72**
**926**	LCP1 $P13796_1$: Plastin-2	1/2	33	97	127	126	51	**1.43**
					129	128	43	**1.26**
					131	130	51	**0.57**
**927**	LCP1 $P13796_2$: Plastin-2		9	15	127	126	1	**0.51**
					129	128	1	**0.58**
					131	130	1	**1.20**
**928**	LDHA $P00338_{1,3}$: L-lactate dehydrogenase A chain	2/8	10	13	127	126	11	**7.56**
					129	128	12	**1.09**
					131	130	11	**1.09**
**929**	LDLR $P01130_{1,5}$: Low-density lipoprotein receptor	2/5	6	19	127	126	10	**0.57**
					129	128	12	**0.74**
					131	130	13	**1.07**
**930**	LECT2 O14960: Leukocyte cell-derived chemotaxin-2		6	9	127	126	8	**0.49**
					129	128	9	**0.65**
					131	130	7	**0.90**
**931**	LEP P41159: Leptin		6	9	127	126	9	**0.33**
					129	128	8	**1.62**
					131	130	9	**1.01**
**932**	LFNG $Q8NES3_{1-4}$: Beta-1,3-N-acetylglucosaminyltransferase lunatic fringe	4/4	4	5	127	126	4	**1.50**
					129	128	5	**1.13**
					131	130	5	**0.85**
**933**	LGALS1 P09382: Galectin-1		8	22	127	126	21	**0.65**
					129	128	20	**1.26**
					131	130	21	**1.11**
**934**	LGALS3 P17931: Galectin-3		9	14	127	126	14	**1.36**
					129	128	13	**1.71**
					131	130	14	**0.84**
**935**	LGALS3BP Q08380: Galectin-3-binding protein		18	42	127	126	37	**0.49**
					129	128	39	**0.77**
					131	130	40	**0.99**
**936**	LGALS7 P47929: Galectin-7		9	19	127	126	16	**8.91**
					129	128	19	**0.82**
					131	130	19	**1.13**

#	protein	group	peptides	spectra	ch1	ch2	quant	ratio
**937**	LGI2 Q8N0V4: Leucine-rich repeat LGI family member 2		3	3	127	126	3	**0.86**
					129	128	3	**1.16**
					131	130	3	**1.37**
**938**	LILRA1 O75019$_{1,2}$: Leukocyte immunoglobulin-like receptor subfamily A member 1	2/2	2	2	127	126	1	**1.37**
					129	128	1	**1.02**
					131	130	1	**0.90**
**939**	LIPC P11150: Hepatic triacylglycerol lipase		3	3	127	126	3	**1.29**
					129	128	3	**1.29**
					131	130	3	**0.93**
**940**	LIPG Q9Y5X9$_{1,2}$: Endothelial lipase	2/2	2	2	127	126	1	**1.17**
					129	128	1	**1.16**
					131	130	1	**1.44**
**941**	LMAN1 P49257: Protein ERGIC-53		7	9	127	126	7	**0.67**
					129	128	7	**1.02**
					131	130	8	**1.10**
**942**	LMAN2 Q12907: Vesicular integral-membrane protein VIP36		2	2	127	126	2	**2.01**
					129	128	2	**1.76**
					131	130	2	**1.02**
**943**	LMBRD2 Q68DH5: LMBR1 domain-containing protein 2		3	17	127	126	11	**0.75**
					129	128	8	**1.07**
					131	130	12	**1.42**
**944**	LMNA P02545$_{1-3,6}$: Prelamin-A/C [Cleaved into: Lamin-A/C	4/5	29	47	127	126	12	**3.31**
					129	128	12	**1.33**
					131	130	11	**1.29**
**945**	LMNA P02545$_{5}$: Prelamin-A/C [Cleaved into: Lamin-A/C		21	36	127	126	1	**0.68**
					129	128	1	**1.49**
					131	130	1	**0.99**
**946**	LMNB1 P20700: Lamin-B1		11	16	127	126	9	**1.35**
					129	128	8	**1.75**
					131	130	7	**1.42**
**947**	LMNB2 Q03252: Lamin-B2		4	9	127	126	1	**0.34**
					129	128	1	**1.05**
					131	130	1	**2.08**
**948**	LOX P28300: Protein-lysine 6-oxidase		11	20	127	126	17	**0.66**
					129	128	16	**0.88**
					131	130	16	**0.88**

#	protein	group	peptides	spectra	ch1	ch2	quant	ratio
**949**	LOXL1 Q08397: Lysyl oxidase homolog 1		19	32	127	126	28	**0.45**
					129	128	24	**0.77**
					131	130	28	**0.83**
**950**	LOXL2 Q9Y4K0: Lysyl oxidase homolog 2		14	18	127	126	15	**0.49**
					129	128	14	**0.73**
					131	130	15	**0.94**
**951**	LOXL3 $P58215_1$: Lysyl oxidase homolog 3	1/2	3	5	127	126	2	**0.43**
					129	128	2	**0.91**
					131	130	2	**1.02**
**952**	LOXL4 Q96JB6: Lysyl oxidase homolog 4		2	4	127	126	1	**1.67**
					129	128	1	**1.95**
					131	130	1	**4.38**
**953**	LPA P08519: Apolipoprotein(a)		29	65	127	126	59	**1.55**
					129	128	64	**0.92**
					131	130	64	**0.50**
**954**	LPAR3 Q9UBY5: Lysophosphatidic acid receptor 3		5	138	127	126	89	**1.27**
					129	128	61	**1.00**
					131	130	121	**2.14**
**955**	LPL P06858: Lipoprotein lipase		11	12	127	126	9	**0.63**
					129	128	11	**1.21**
					131	130	11	**1.08**
**956**	LPP Q93052: Lipoma-preferred partner		2	2	127	126	1	**0.51**
					129	128	1	**2.17**
					131	130	1	**1.30**
**957**	LRG1 P02750: Leucine-rich alpha-2-glycoprotein		4	4	127	126	4	**1.31**
					129	128	4	**1.29**
					131	130	3	**1.02**
**958**	LRP1 $Q07954_1$: Prolow-density lipoprotein receptor-related protein 1		46	127	127	126	92	**0.65**
					129	128	89	**0.90**
					131	130	100	**1.01**
**959**	LRP1B Q9NZR2: Low-density lipoprotein receptor-related protein 1B		28	177	127	126	46	**0.77**
					129	128	35	**0.98**
					131	130	53	**0.88**
**960**	LRP8 $Q14114_{1,3}$: Low-density lipoprotein receptor-related protein 8	2/4	11	49	127	126	21	**0.67**
					129	128	22	**0.85**
					131	130	29	**1.08**

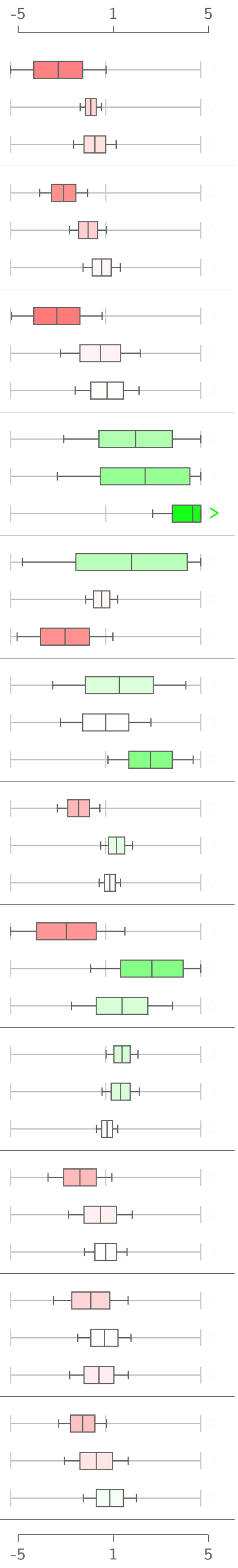

#	protein	group	peptides	spectra	ch1	ch2	quant	ratio
**961**	LRRC15 Q8TF66$_{1,2}$: Leucine-rich repeat-containing protein 15	2/2	1	1	127	126	1	**0.09**
					129	128	1	**0.02**
					131	130	1	**0.69**
**962**	LRRC17 Q8N6Y2$_{1}$: Leucine-rich repeat-containing protein 17	1/2	3	7	127	126	7	**0.63**
					129	128	7	**0.69**
					131	130	7	**1.11**
**963**	LRRC57 Q8N9N7: Leucine-rich repeat-containing protein 57		2	2	127	126	2	**1.26**
					129	128	2	**1.13**
					131	130	2	**1.29**
**964**	LRRFIP2 Q9Y608$_{1}$: Leucine-rich repeat flightless-interacting protein 2		2	2	127	126	2	**4.13**
					129	128	2	**2.91**
					131	130	2	**1.49**
**965**	LSP1 P33241$_{1,3}$: Lymphocyte-specific protein 1	2/2	1	2	127	126	2	**0.67**
					129	128	2	**0.92**
					131	130	2	**1.03**
**966**	LSR Q86X29$_{1,4}$: Lipolysis-stimulated lipoprotein receptor	2/5	9	16	127	126	3	**0.37**
					129	128	3	**0.79**
					131	130	4	**1.03**
**967**	LSR Q86X29$_{3}$: Lipolysis-stimulated lipoprotein receptor		8	13	127	126	1	**0.52**
					129	128	1	**0.95**
					131	130	1	**1.08**
**968**	LTBP1 Q14766$_{1,4}$: Latent-transforming growth factor beta-binding protein 1	2/4	35	60	127	126	10	**0.66**
					129	128	11	**0.96**
					131	130	11	**1.11**
**969**	LTBP1 Q14766$_{3}$: Latent-transforming growth factor beta-binding protein 1		31	50	127	126	1	**0.79**
					129	128	1	**1.02**
					131	130	1	**1.15**
**970**	LTBP2 Q14767: Latent-transforming growth factor beta-binding protein 2		34	64	127	126	50	**0.54**
					129	128	51	**0.92**
					131	130	53	**0.92**
**971**	LTBP3 Q9NS15$_{1}$: Latent-transforming growth factor beta-binding protein 3	1/2	16	17	127	126	13	**0.51**
					129	128	13	**0.93**
					131	130	13	**0.88**
**972**	LTBP4 Q8N2S1$_{2}$: Latent-transforming growth factor beta-binding protein 4	1/4	40	71	127	126	67	**0.52**
					129	128	61	**1.02**
					131	130	68	**0.91**

#	protein	group	peptides	spectra	ch1	ch2	quant	ratio
**973**	LTF $P02788_1$: Lactotransferrin	1/2	31	38	127	126	37	**0.78**
					129	128	37	**1.15**
					131	130	38	**1.84**
**974**	LUM P51884: Lumican		14	168	127	126	164	**0.51**
					129	128	158	**1.05**
					131	130	165	**0.82**
**975**	LY75 $O60449_{2,3}$: Lymphocyte antigen 75	2/2	4	7	127	126	6	**0.84**
					129	128	5	**1.02**
					131	130	6	**1.03**
**976**	LY96 $Q9Y6Y9_{1,2}$: Lymphocyte antigen 96	2/2	1	1	127	126	1	**1.30**
					129	128	1	**1.04**
					131	130	1	**0.78**
**977**	LYZ P61626: Lysozyme C		13	304	127	126	284	**0.54**
					129	128	282	**0.58**
					131	130	301	**0.69**
**978**	MACF1 $Q9UPN3_1$: Microtubule-actin cross-linking factor 1, isoforms 1/2/3/5	1/4	14	27	127	126	8	**1.31**
					129	128	6	**1.21**
					131	130	7	**1.07**
**979**	MACF1 $Q9UPN3_5$: Microtubule-actin cross-linking factor 1, isoforms 1/2/3/5		10	21	127	126	2	**1.13**
					129	128	2	**1.23**
					131	130	2	**0.56**
**980**	MAMDC2 $Q7Z304_1$: MAM domain-containing protein 2		2	2	127	126	2	**0.62**
					129	128	2	**0.86**
					131	130	2	**0.67**
**981**	MAN1A1 $P33908_1$: Mannosyl-oligosaccharide 1,2-alpha-mannosidase IA	1/2	21	32	127	126	29	**1.04**
					129	128	30	**1.43**
					131	130	31	**0.85**
**982**	MAN2A1 Q16706: Alpha-mannosidase 2		2	2	127	126	2	**0.90**
					129	128	2	**1.05**
					131	130	2	**1.03**
**983**	MAP1B P46821: Microtubule-associated protein 1B		4	5	127	126	3	**0.46**
					129	128	3	**0.86**
					131	130	3	**1.06**
**984**	MARCKS P29966: Myristoylated alanine-rich C-kinase substrate		3	3	127	126	3	**0.96**
					129	128	3	**1.25**
					131	130	3	**1.02**

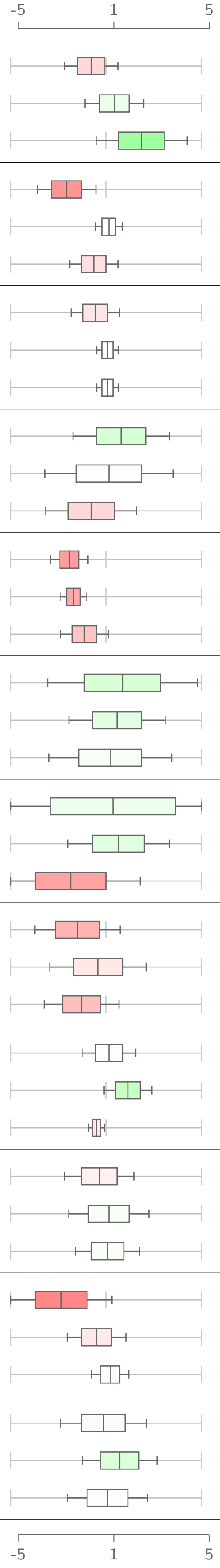

#	protein	group	peptides	spectra	ch1	ch2	quant	ratio
**985**	MASP1 P48740$_1$: Mannan-binding lectin serine protease 1	1/2	16	50	127	126	9	**0.66**
					129	128	8	**0.73**
					131	130	9	**1.55**
**986**	MASP1 P48740$_{2,4}$: Mannan-binding lectin serine protease 1	2/2	16	47	127	126	6	**0.75**
					129	128	6	**0.81**
					131	130	6	**1.20**
**987**	MASP2 O00187$_1$: Mannan-binding lectin serine protease 2		8	16	127	126	2	**0.77**
					129	128	2	**0.67**
					131	130	2	**1.41**
**988**	MASP2 O00187$_2$: Mannan-binding lectin serine protease 2		7	17	127	126	3	**0.63**
					129	128	3	**0.68**
					131	130	3	**1.25**
**989**	MAT2A P31153$_{1,2}$: S-adenosylmethionine synthase isoform type-2	2/2	2	2	127	126	2	**1.29**
					129	128	2	**3.23**
					131	130	2	**2.79**
**990**	MATN2 O00339$_2$: Matrilin-2	1/4	17	18	127	126	18	**0.96**
					129	128	18	**1.10**
					131	130	18	**0.72**
**991**	MATR3 P43243$_1$: Matrin-3	1/2	11	12	127	126	12	**1.37**
					129	128	12	**1.58**
					131	130	12	**1.49**
**992**	MatrixProt_SaCV_Frankf		2	2	127	126	2	**1.07**
					129	128	2	**0.74**
					131	130	2	**0.84**
**993**	MB P02144: Myoglobin		8	10	127	126	8	**0.84**
					129	128	9	**1.76**
					131	130	9	**1.11**
**994**	MCFD2 Q8NI22$_{1-3}$: Multiple coagulation factor deficiency protein 2	3/3	1	2	127	126	2	**0.61**
					129	128	2	**0.74**
					131	130	2	**0.99**
**995**	MDH1 P40925$_{1-3}$: Malate dehydrogenase, cytoplasmic	3/3	4	4	127	126	4	**1.59**
					129	128	4	**0.91**
					131	130	4	**1.10**
**996**	MDK P21741$_1$: Midkine	1/2	3	3	127	126	3	**0.42**
					129	128	3	**0.87**
					131	130	3	**0.93**

#	protein	group	peptides	spectra	ch1	ch2	quant	ratio
**997**	ME1 P48163$_1$: NADP-dependent malic enzyme		4	5	127	126	4	**1.33**
					129	128	4	**1.00**
					131	130	3	**1.13**
**998**	MED14 O60244: Mediator of RNA polymerase II transcription subunit 14		2	5	127	126	2	**0.76**
					129	128	1	**1.39**
					131	130	3	**1.50**
**999**	MEGF6 O75095$_{1,2}$: Multiple epidermal growth factor-like domains protein 6	2/2	7	8	127	126	8	**0.57**
					129	128	6	**0.81**
					131	130	8	**0.94**
**1000**	METRN Q9UJH8: Meteorin		4	4	127	126	4	**0.55**
					129	128	4	**0.79**
					131	130	3	**1.02**
**1001**	METRNL Q641Q3$_1$: Meteorin-like protein	1/2	6	6	127	126	5	**0.66**
					129	128	6	**0.90**
					131	130	5	**0.88**
**1002**	MFAP2 P55001$_{1-3}$: Microfibrillar-associated protein 2	3/3	3	3	127	126	3	**0.64**
					129	128	3	**0.94**
					131	130	3	**0.82**
**1003**	MFAP4 P55083$_{1,2}$: Microfibril-associated glycoprotein 4	2/2	7	85	127	126	84	**0.48**
					129	128	81	**0.76**
					131	130	85	**0.98**
**1004**	MFAP5 Q13361$_{1,2}$: Microfibrillar-associated protein 5	2/2	6	13	127	126	9	**1.01**
					129	128	8	**0.81**
					131	130	10	**0.91**
**1005**	MGAT1 P26572: Alpha-1,3-mannosyl-glycoprotein 2-beta-N-acetylglucosaminyltransferase		12	20	127	126	19	**1.19**
					129	128	20	**1.07**
					131	130	20	**0.84**
**1006**	MGAT4B Q9UQ53$_{1-3}$: Alpha-1,3-mannosyl-glycoprotein 4-beta-N-acetylglucosaminyltransferase B	3/3	4	5	127	126	4	**1.13**
					129	128	5	**1.18**
					131	130	4	**1.01**
**1007**	MGAT5 Q09328: Alpha-1,6-mannosylglycoprotein 6-beta-N-acetylglucosaminyltransferase A	1/2	8	8	127	126	8	**1.13**
					129	128	7	**1.14**
					131	130	7	**0.98**
**1008**	MGP P08493$_{1,2}$: Matrix Gla protein	2/2	5	108	127	126	108	**0.36**
					129	128	104	**1.02**
					131	130	106	**0.60**

#	protein	group	peptides	spectra	ch1	ch2	quant	ratio
**1009**	MIA3 Q5JRA6$_{1,2}$: Melanoma inhibitory activity protein 3	2/3	4	4	127	126	4	**0.63**
					129	128	4	**0.91**
					131	130	4	**1.01**
**1010**	MINPP1 Q9UNW1$_{1}$: Multiple inositol polyphosphate phosphatase 1	1/4	13	22	127	126	21	**1.20**
					129	128	19	**1.23**
					131	130	19	**0.84**
**1011**	MMP14 P50281: Matrix metalloproteinase-14		4	6	127	126	6	**0.57**
					129	128	6	**1.16**
					131	130	6	**0.92**
**1012**	MMP19 Q99542$_{1}$: Matrix metalloproteinase-19	1/3	5	5	127	126	5	**0.76**
					129	128	5	**0.84**
					131	130	4	**0.79**
**1013**	MMP2 P08253$_{1}$: 72 kDa type IV collagenase	1/3	22	29	127	126	28	**0.57**
					129	128	28	**1.24**
					131	130	28	**0.71**
**1014**	MMP28 Q9H239$_{1}$: Matrix metalloproteinase-28	1/2	3	3	127	126	3	**0.48**
					129	128	3	**1.05**
					131	130	3	**0.76**
**1015**	MMRN2 Q9H8L6: Multimerin-2		6	6	127	126	6	**0.59**
					129	128	5	**1.06**
					131	130	6	**0.90**
**1016**	MORC1 Q86VD1$_{1,2}$: MORC family CW-type zinc finger protein 1	2/2	2	2	127	126	2	**1.00**
					129	128	1	**0.70**
					131	130	2	**0.83**
**1017**	MPO P05164$_{1-3}$: Myeloperoxidase	3/4	7	9	127	126	9	**0.97**
					129	128	9	**1.37**
					131	130	9	**1.43**
**1018**	MPST P25325$_{1,2}$: 3-mercaptopyruvate sulfurtransferase	2/2	4	4	127	126	4	**0.99**
					129	128	4	**1.26**
					131	130	4	**1.08**
**1019**	MRC2 Q9UBG0: C-type mannose receptor 2		3	4	127	126	4	**0.66**
					129	128	4	**0.95**
					131	130	4	**0.95**
**1020**	MSLN Q13421$_{2-4}$: Mesothelin	3/4	22	53	127	126	51	**0.97**
					129	128	53	**1.11**
					131	130	52	**0.88**

#	protein	group	peptides	spectra	ch1	ch2	quant	ratio
**1021**	MSN P26038: Moesin	1/3	19	22	127	126	12	**2.82**
					129	128	13	**1.34**
					131	130	13	**1.25**
**1022**	MST1 P26927: Hepatocyte growth factor-like protein	1/3	13	17	127	126	13	**0.82**
					129	128	17	**0.90**
					131	130	13	**1.06**
**1023**	MT1A P04731: Metalloth-ionein-1A		4	21	127	126	6	**0.74**
					129	128	7	**1.06**
					131	130	9	**1.02**
**1024**	MTHFD1 P11586: C-1-tetrahydrofolate synthase, cytoplasmic		9	12	127	126	10	**1.77**
					129	128	11	**1.43**
					131	130	9	**1.40**
**1025**	MVP Q14764: Major vault protein		6	7	127	126	7	**1.32**
					129	128	6	**2.19**
					131	130	7	**1.17**
**1026**	MXRA5 Q9NR99: Matrix-remodeling-associated protein 5		41	66	127	126	62	**0.87**
					129	128	62	**0.95**
					131	130	64	**0.95**
**1027**	MXRA7 P84157$_1$: Matrix-remodeling-associated protein 7	1/2	4	6	127	126	4	**0.46**
					129	128	3	**0.99**
					131	130	3	**0.64**
**1028**	MXRA7 P84157$_2$: Matrix-remodeling-associated protein 7		3	3	127	126	1	**0.41**
					129	128	1	**0.94**
					131	130	1	**1.07**
**1029**	MYCBP2 O75592$_{1,2}$: E3 ubiquitin-protein ligase MYCBP2	2/2	8	27	127	126	25	**0.76**
					129	128	24	**0.86**
					131	130	27	**1.04**
**1030**	MYH10 P35580$_{4,5}$: Myosin-10	2/5	58	69	127	126	41	**1.05**
					129	128	39	**2.49**
					131	130	41	**1.68**
**1032**	MYH13 Q9UKX3: Myosin-13	1/7	3	4	127	126	2	**1.27**
					129	128	2	**1.57**
					131	130	2	**1.13**
**1033**	MYH14 Q7Z406$_{1,2,6}$: Myosin-14	3/5	12	13	127	126	2	**1.13**
					129	128	2	**0.93**
					131	130	2	**1.24**

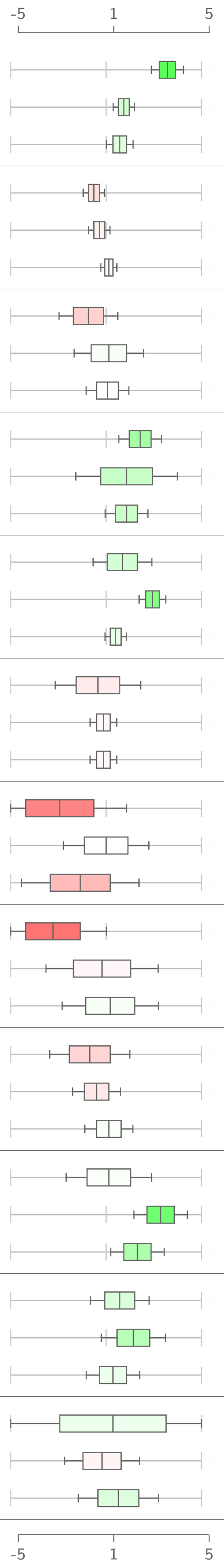

#	protein	group	peptides	spectra	ch1	ch2	quant	ratio
**1034**	MYH7 P12883: Myosin-7		2	2	127	126	1	**0.84**
					129	128	1	**0.89**
					131	130	1	**1.68**
**1035**	MYH9 $P35579_1$: Myosin-9	1/2	82	129	127	126	99	**2.16**
					129	128	94	**2.33**
					131	130	98	**1.31**
**1036**	MYL1 $P05976_1$: Myosin light chain 1/3, skeletal muscle isoform	1/2	8	18	127	126	15	**0.67**
					129	128	13	**1.13**
					131	130	15	**2.90**
**1037**	MYL10 Q9BUA6: Myosin regulatory light chain 10		3	5	127	126	2	**0.82**
					129	128	2	**0.86**
					131	130	2	**0.90**
**1038**	MYL12A P19105, MYL12B O14950: Myosin regulatory light chain 12A, Myosin regulatory light chain 12B	2/2	9	18	127	126	7	**1.25**
					129	128	7	**2.11**
					131	130	7	**1.32**
**1039**	MYL2 P10916: Myosin regulatory light chain 2, ventricular/cardiac muscle isoform	1/2	10	25	127	126	21	**0.46**
					129	128	21	**0.90**
					131	130	22	**0.84**
**1040**	MYL3 P08590: Myosin light chain 3		11	17	127	126	13	**0.46**
					129	128	12	**1.13**
					131	130	13	**0.88**
**1041**	MYL6 $P60660_{1,2}$: Myosin light polypeptide 6	2/3	8	17	127	126	13	**1.15**
					129	128	11	**1.87**
					131	130	14	**1.18**
**1042**	MYL9 $P24844_1$: Myosin regulatory light polypeptide 9	1/2	8	16	127	126	4	**0.47**
					129	128	5	**1.11**
					131	130	5	**0.99**
**1043**	MYLK $Q15746_{1-6}$: Myosin light chain kinase, smooth muscle	6/11	7	10	127	126	10	**0.68**
					129	128	8	**1.04**
					131	130	8	**0.91**
**1044**	MYLPF Q96A32: Myosin regulatory light chain 2, skeletal muscle isoform		6	9	127	126	9	**0.51**
					129	128	8	**1.05**
					131	130	9	**2.87**
**1045**	MYO18A $Q92614_{1-5}$: Unconventional myosin-XVIIIa	5/5	6	6	127	126	4	**1.12**
					129	128	4	**1.04**
					131	130	5	**1.02**

#	protein	group	peptides	spectra	ch1	ch2	quant	ratio
**1046**	MYO1C O00159$_{1-3}$: Unconventional myosin-Ic	3/3	4	7	127	126	5	**1.43**
					129	128	7	**1.97**
					131	130	7	**1.44**
**1047**	MYOC Q99972: Myocilin		16	22	127	126	22	**0.87**
					129	128	22	**1.31**
					131	130	22	**0.86**
**1048**	NACA E9PAV3$_{1,2}$, NACA Q13765$_1$: Nascent polypeptide-associated complex subunit alpha, muscle-specific form, Nascent polypeptide-associated complex subunit alpha	3/3	3	3	127	126	3	**2.04**
					129	128	3	**1.06**
					131	130	3	**0.84**
**1049**	NAGK Q9UJ70$_2$: N-acetyl-D-glucosamine kinase	1/2	4	4	127	126	3	**1.15**
					129	128	4	**1.16**
					131	130	4	**0.97**
**1050**	NAGLU P54802: Alpha-N-acetylglucosaminidase		1	1	127	126	1	**0.58**
					129	128	1	**0.92**
					131	130	1	**1.06**
**1051**	NAMPT P43490: Nicotinamide phosphoribosyltransferase		5	6	127	126	6	**4.29**
					129	128	6	**2.51**
					131	130	6	**0.93**
**1052**	NANS Q9NR45: Sialic acid synthase		5	5	127	126	4	**1.26**
					129	128	4	**2.59**
					131	130	4	**1.83**
**1053**	NAP1L1 P55209$_{1-3}$: Nucleosome assembly protein 1-like 1	3/3	2	2	127	126	1	**1.29**
					129	128	1	**1.72**
					131	130	1	**1.53**
**1054**	NAP1L4 Q99733$_{1,2}$: Nucleosome assembly protein 1-like 4	2/2	2	2	127	126	1	**2.62**
					129	128	1	**1.47**
					131	130	1	**1.70**
**1055**	NAPA P54920: Alpha-soluble NSF attachment protein		6	6	127	126	6	**1.14**
					129	128	6	**1.76**
					131	130	6	**1.20**
**1056**	NARS O43776$_1$: Asparagine--tRNA ligase, cytoplasmic	1/2	4	4	127	126	3	**3.49**
					129	128	3	**3.38**
					131	130	4	**0.99**
**1057**	NBL1 P41271$_{1,2}$: Neuroblastoma suppressor of tumorigenicity 1	2/2	3	8	127	126	7	**0.71**
					129	128	7	**0.91**
					131	130	8	**0.77**

#	protein	group	peptides	spectra	ch1	ch2	quant	ratio
**1058**	NCALD P61601: Neurocalcin-delta	1/3	5	6	127	126	5	**0.95**
					129	128	6	**1.52**
					131	130	6	**1.09**
**1059**	NCAM1 P13591$_{1-6}$: Neural cell adhesion molecule 1	6/6	3	5	127	126	3	**0.50**
					129	128	3	**0.81**
					131	130	5	**1.03**
**1060**	NCCRP1 Q6ZVX7: F-box only protein 50		4	6	127	126	3	**3.93**
					129	128	6	**1.12**
					131	130	6	**1.06**
**1061**	NCF1 P14598$_1$, NCF1B A6NI72, NCF1C A8MVU1: Neutrophil cytosol factor 1, Putative neutrophil cytosol factor 1B, ...	3/3	2	3	127	126	3	**0.54**
					129	128	3	**1.22**
					131	130	3	**1.02**
**1062**	NCL P19338: Nucleolin		24	25	127	126	25	**2.27**
					129	128	24	**1.96**
					131	130	25	**1.62**
**1063**	NCOA5 Q9HCD5: Nuclear receptor coactivator 5		6	8	127	126	6	**0.68**
					129	128	5	**1.58**
					131	130	8	**1.21**
**1064**	NDRG1 Q92597$_1$: Protein NDRG1	1/3	3	3	127	126	3	**0.95**
					129	128	3	**2.09**
					131	130	3	**1.48**
**1065**	NDRG2 Q9UN36$_{1,3}$: Protein NDRG2	2/2	2	2	127	126	2	**1.89**
					129	128	2	**0.99**
					131	130	2	**1.03**
**1066**	NECAP2 Q9NVZ3$_{1,2,4}$: Adaptin ear-binding coat-associated protein 2	3/4	4	5	127	126	5	**1.37**
					129	128	4	**1.59**
					131	130	5	**1.23**
**1067**	NECTIN2 Q92692$_{1,2}$: Nectin-2	2/2	2	2	127	126	2	**1.46**
					129	128	2	**1.46**
					131	130	2	**0.92**
**1068**	NEFH P12036$_{1,2}$: Neurofilament heavy polypeptide	2/2	5	21	127	126	1	**1.80**
					129	128	1	**0.80**
					131	130	2	**0.92**
**1069**	NES P48681: Nestin		4	4	127	126	2	**1.11**
					129	128	1	**0.89**
					131	130	1	**1.01**

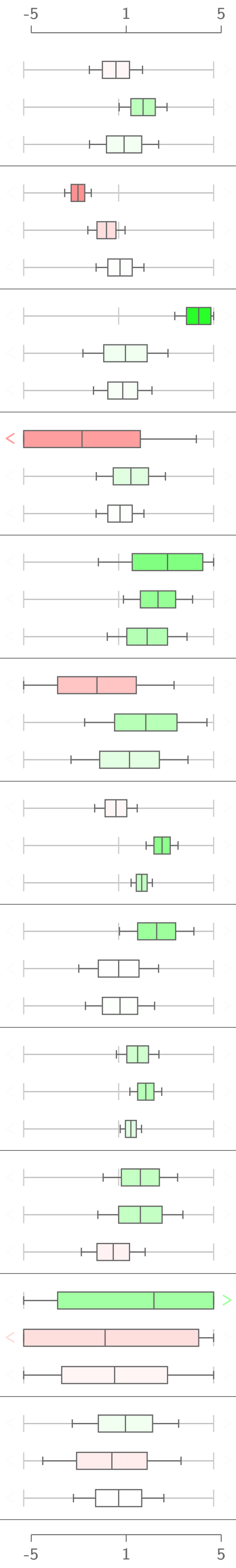

#	protein	group	peptides	spectra	ch1	ch2	quant	ratio
**1070**	NFKB1 P19838$_{1-3}$: Nuclear factor NF-kappa-B p105 subunit	3/3	2	2	127	126	2	**0.86**
					129	128	2	**2.33**
					131	130	2	**1.46**
**1071**	NID1 P14543$_{1}$: Nidogen-1	1/2	21	31	127	126	31	**0.67**
					129	128	29	**0.91**
					131	130	29	**0.87**
**1072**	NID2 Q14112$_{1}$: Nidogen-2	1/2	21	24	127	126	21	**0.67**
					129	128	20	**0.86**
					131	130	19	**0.85**
**1073**	NME2 P22392$_{1,2}$: Nucleoside diphosphate kinase B	2/5	3	3	127	126	3	**2.43**
					129	128	3	**1.89**
					131	130	3	**0.99**
**1074**	NNMT P40261: Nicotinamide N-methyltransferase		1	2	127	126	2	**1.50**
					129	128	2	**2.31**
					131	130	2	**1.48**
**1075**	NOL3 O60936$_{1-3}$: Nucleolar protein 3	3/3	2	3	127	126	3	**1.64**
					129	128	3	**1.19**
					131	130	3	**1.12**
**1076**	NONO Q15233$_{1}$: Non-POU domain-containing octamer-binding protein	1/4	11	13	127	126	10	**1.43**
					129	128	8	**2.56**
					131	130	9	**1.58**
**1077**	NOTCH2 Q04721: Neurogenic locus notch homolog protein 2		8	58	127	126	47	**0.63**
					129	128	49	**1.29**
					131	130	53	**1.06**
**1078**	NOTCH3 Q9UM47: Neurogenic locus notch homolog protein 3		10	13	127	126	12	**0.63**
					129	128	9	**0.86**
					131	130	11	**1.12**
**1079**	NPC2 P61916$_{1,2}$: Epididymal secretory protein E1	2/2	4	4	127	126	3	**2.19**
					129	128	4	**1.58**
					131	130	4	**0.79**
**1080**	NPEPPS P55786$_{1}$: Puromycin-sensitive aminopeptidase		2	2	127	126	2	**2.73**
					129	128	2	**1.63**
					131	130	2	**0.98**
**1081**	NPM1 P06748$_{1}$: Nucleophosmin	1/3	12	15	127	126	12	**1.99**
					129	128	14	**2.55**
					131	130	15	**1.98**

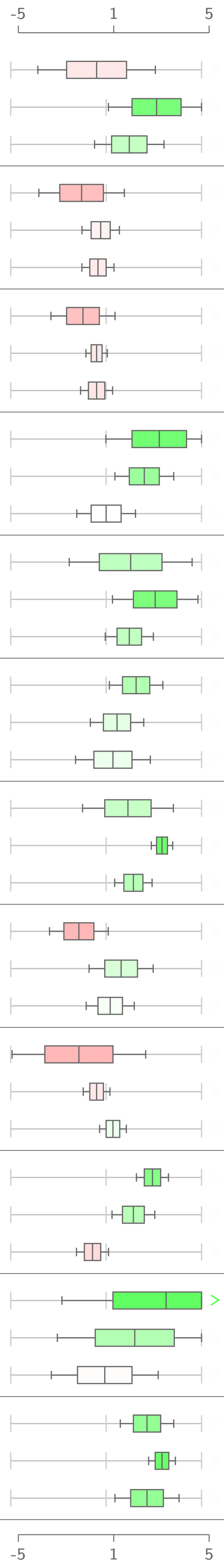

#	protein	group	peptides	spectra	ch1	ch2	quant	ratio
**1082**	NPPA P01160: Natriuretic peptides A		3	3	127	126	3	**0.52**
					129	128	3	**1.54**
					131	130	3	**0.54**
**1083**	NQO1 $P15559_{1-3}$: NAD(P)H dehydrogenase [quinone] 1	3/3	2	2	127	126	2	**0.67**
					129	128	2	**1.89**
					131	130	2	**2.02**
**1084**	NRP2 $O60462_{1-5}$: Neuropilin-2	5/6	5	5	127	126	4	**0.63**
					129	128	3	**0.67**
					131	130	5	**1.03**
**1085**	NSF $P46459_{1}$: Vesicle-fusing ATPase	1/2	4	4	127	126	4	**0.35**
					129	128	3	**1.43**
					131	130	4	**1.12**
**1086**	NSFL1C $Q9UNZ2_{1,4,5}$: NSFL1 cofactor p47	3/3	2	2	127	126	2	**1.31**
					129	128	2	**1.67**
					131	130	2	**1.07**
**1087**	NT5C3A $Q9H0P0_{1-4}$: Cytosolic 5'-nucleotidase 3A	4/4	3	3	127	126	3	**4.28**
					129	128	3	**1.38**
					131	130	3	**1.28**
**1088**	NUCB1 Q02818: Nucleobindin-1		32	74	127	126	63	**0.30**
					129	128	67	**0.89**
					131	130	68	**0.76**
**1089**	NUCB2 $P80303_{1,2}$: Nucleobindin-2	2/2	14	25	127	126	21	**0.40**
					129	128	19	**0.85**
					131	130	21	**0.85**
**1090**	NUCKS1 $Q9H1E3_{1}$: Nuclear ubiquitous casein and cyclin-dependent kinase substrate 1	1/2	8	11	127	126	10	**0.62**
					129	128	9	**1.62**
					131	130	11	**1.37**
**1091**	NUDC Q9Y266: Nuclear migration protein nudC		6	17	127	126	12	**0.70**
					129	128	10	**1.37**
					131	130	15	**0.91**
**1092**	NUDT3 O95989: Diphosphoinositol polyphosphate phosphohydrolase 1		1	1	127	126	1	**1.31**
					129	128	1	**1.30**
					131	130	1	**0.76**
**1093**	NUDT5 Q9UKK9: ADP-sugar pyrophosphatase		2	2	127	126	2	**0.69**
					129	128	2	**1.31**
					131	130	2	**0.93**

#	protein	group	peptides	spectra	ch1	ch2	quant	ratio
**1094**	NUMA1 Q14980$_{1-4}$: Nuclear mitotic apparatus protein 1	4/4	9	96	127	126	8	**0.49**
					129	128	8	**1.17**
					131	130	8	**1.75**
**1095**	NUTF2 P61970: Nuclear transport factor 2		2	3	127	126	3	**0.69**
					129	128	3	**1.28**
					131	130	3	**1.06**
**1096**	O00292$_1$	1/3	5	6	127	126	6	**0.81**
					129	128	6	**1.18**
					131	130	6	**0.91**
**1097**	OAF Q86UD1: Out at first protein homolog		7	19	127	126	15	**0.72**
					129	128	19	**0.98**
					131	130	16	**1.04**
**1098**	OGN P20774: Mimecan		13	70	127	126	68	**0.66**
					129	128	61	**1.14**
					131	130	70	**0.59**
**1099**	OIT3 Q8WWZ8$_1$: Oncoprotein-induced transcript 3 protein	1/2	3	3	127	126	3	**0.89**
					129	128	3	**0.91**
					131	130	3	**1.20**
**1100**	OLA1 Q9NTK5$_{1,2}$: Obg-like ATPase 1	2/2	4	5	127	126	5	**3.22**
					129	128	4	**2.72**
					131	130	4	**0.72**
**1101**	OLFML1 Q6UWY5: Olfactomedin-like protein 1		10	15	127	126	14	**0.53**
					129	128	15	**0.94**
					131	130	13	**0.88**
**1102**	OLFML2A Q68BL7$_{1-3}$: Olfactomedin-like protein 2A	3/3	1	1	127	126	1	**0.60**
					129	128	1	**1.14**
					131	130	1	**0.87**
**1103**	OLFML2B Q68BL8$_1$: Olfactomedin-like protein 2B		2	2	127	126	2	**0.50**
					129	128	2	**0.88**
					131	130	2	**1.43**
**1104**	OLFML3 Q9NRN5$_1$: Olfactomedin-like protein 3	1/3	17	59	127	126	58	**0.56**
					129	128	48	**0.88**
					131	130	58	**0.91**
**1105**	OMD Q99983: Osteomodulin		4	4	127	126	3	**0.96**
					129	128	3	**0.88**
					131	130	4	**0.96**

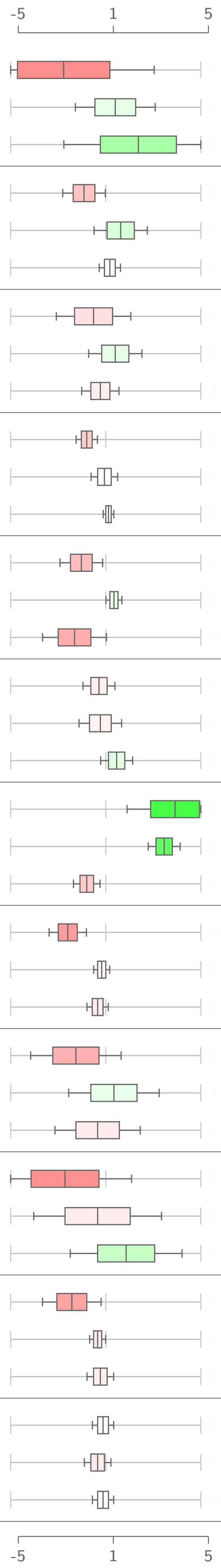

#	protein	group	peptides	spectra	ch1	ch2	quant	ratio
**1107**	ORM1 P02763: Alpha-1-acid glycoprotein 1		8	14	127	126	8	**1.65**
					129	128	8	**1.02**
					131	130	8	**1.10**
**1108**	ORM2 P19652: Alpha-1-acid glycoprotein 2		9	14	127	126	7	**1.56**
					129	128	6	**0.95**
					131	130	8	**1.06**
**1109**	OSBP P22059: Oxysterol-binding protein 1		3	5	127	126	5	**0.68**
					129	128	5	**0.97**
					131	130	5	**1.30**
**1110**	OVGP1 Q12889: Oviduct-specific glycoprotein		12	24	127	126	19	**1.25**
					129	128	20	**1.63**
					131	130	23	**0.70**
**1111**	P04220: Ig mu heavy chain disease protein		13	96	127	126	9	**0.62**
					129	128	8	**0.66**
					131	130	10	**1.15**
**1112**	P3H1 Q32P28$_{1,3,4}$: Prolyl 3-hydroxylase 1	3/3	5	5	127	126	3	**0.48**
					129	128	4	**0.99**
					131	130	4	**0.99**
**1113**	P4HA1 P13674$_{1-3}$: Prolyl 4-hydroxylase subunit alpha-1	3/3	1	1	127	126	1	**0.54**
					129	128	1	**1.25**
					131	130	1	**0.80**
**1114**	P4HB P07237: Protein disulfide-isomerase		27	51	127	126	46	**1.06**
					129	128	46	**1.07**
					131	130	47	**1.16**
**1115**	PA2G4 Q9UQ80$_{1,2}$: Proliferation-associated protein 2G4	2/2	5	7	127	126	5	**2.02**
					129	128	5	**1.94**
					131	130	4	**1.60**
**1116**	PABPC1 P11940$_{1}$: Polyadenylate-binding protein 1	1/5	12	13	127	126	11	**1.11**
					129	128	11	**1.70**
					131	130	13	**1.07**
**1117**	PAFAH1B2 P68402$_{1}$: Platelet-activating factor acetylhydrolase IB subunit beta		2	2	127	126	2	**1.53**
					129	128	2	**2.34**
					131	130	2	**1.02**
**1118**	PAIP1 Q9H074$_{1-3}$: Polyadenylate-binding protein-interacting protein 1	3/3	2	2	127	126	2	**0.98**
					129	128	2	**1.65**
					131	130	2	**1.12**

#	protein	group	peptides	spectra	ch1	ch2	quant	ratio
**1119**	PAK2 Q13177: Serine/threonine-protein kinase PAK 2		3	3	127	126	3	**1.53**
					129	128	3	**2.02**
					131	130	3	**1.04**
**1120**	PAM P19021$_{1,3,5}$: Peptidyl-glycine alpha-amidating monooxygenase	3/6	19	24	127	126	22	**0.64**
					129	128	22	**0.99**
					131	130	24	**0.83**
**1121**	PAMR1 Q6UXH9$_1$: Inactive serine protease PAMR1	1/2	16	31	127	126	2	**0.48**
					129	128	2	**0.89**
					131	130	2	**0.96**
**1122**	PAMR1 Q6UXH9$_2$: Inactive serine protease PAMR1		16	31	127	126	2	**0.50**
					129	128	2	**1.08**
					131	130	2	**0.78**
**1123**	PAPLN O95428$_{1,5,6}$: Papilin	3/6	22	28	127	126	23	**0.45**
					129	128	25	**0.90**
					131	130	27	**1.02**
**1124**	PAPSS2 O95340$_{1,2}$: Bifunctional 3'-phosphoadenosine 5'-phosphosulfate synthase 2	2/2	3	3	127	126	2	**0.98**
					129	128	2	**2.13**
					131	130	2	**1.09**
**1125**	PARK7 Q99497: Protein deglycase DJ-1		2	2	127	126	2	**1.84**
					129	128	2	**1.26**
					131	130	2	**1.37**
**1126**	PARVA Q9NVD7$_1$: Alpha-parvin		4	4	127	126	4	**0.93**
					129	128	4	**1.73**
					131	130	4	**1.09**
**1127**	PBXIP1 Q96AQ6$_{1,2}$: Pre-B-cell leukemia transcription factor-interacting protein 1	2/3	5	6	127	126	4	**0.43**
					129	128	5	**0.83**
					131	130	5	**0.83**
**1128**	PCBP1 Q15365: Poly(rC)-binding protein 1	1/6	11	14	127	126	6	**1.05**
					129	128	6	**2.01**
					131	130	6	**1.83**
**1129**	PCBP2 Q15366$_{1-8}$: Poly(rC)-binding protein 2	8/8	6	9	127	126	1	**2.74**
					129	128	1	**2.94**
					131	130	1	**1.09**
**1130**	PCDHGB6 Q9Y5F9$_{1,2}$: Protocadherin gamma-B6	2/2	2	3	127	126	3	**0.58**
					129	128	3	**0.81**
					131	130	3	**1.20**

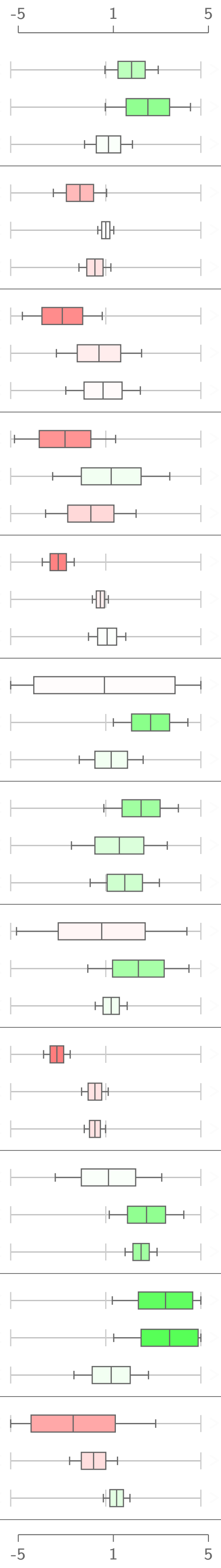

#	protein	group	peptides	spectra	ch1	ch2	quant	ratio
1131	PCMT1 P22061$_{1,2}$: Protein-L-isoaspartate(D-aspartate) O-methyltransferase	2/2	6	6	127	126	5	**1.61**
					129	128	6	**1.21**
					131	130	5	**0.99**
1132	PCNT O95613$_1$: Pericentrin		3	33	127	126	29	**1.22**
					129	128	30	**1.40**
					131	130	30	**1.90**
1133	PCOLCE Q15113: Procollagen C-endopeptidase enhancer 1		22	111	127	126	107	**0.50**
					129	128	110	**0.94**
					131	130	105	**1.08**
1134	PCOLCE2 Q9UKZ9: Procollagen C-endopeptidase enhancer 2		12	25	127	126	23	**0.81**
					129	128	24	**1.11**
					131	130	22	**0.98**
1135	PCSK1N Q9UHG2: ProSAAS		5	11	127	126	11	**0.59**
					129	128	11	**0.86**
					131	130	11	**0.82**
1136	PCSK5 Q92824$_1$: Proprotein convertase subtilisin/kexin type 5		32	133	127	126	70	**0.73**
					129	128	62	**0.88**
					131	130	89	**1.06**
1137	PCSK5 Q92824$_2$: Proprotein convertase subtilisin/kexin type 5		17	20	127	126	1	**0.58**
					129	128	1	**1.04**
					131	130	1	**0.81**
1138	PCSK9 Q8NBP7$_1$: Proprotein convertase subtilisin/kexin type 9	1/2	13	22	127	126	22	**0.76**
					129	128	22	**0.83**
					131	130	22	**1.08**
1139	PCYOX1 Q9UHG3$_1$: Prenylcysteine oxidase 1	1/2	13	20	127	126	17	**0.91**
					129	128	19	**0.97**
					131	130	19	**0.81**
1140	PDCD4 Q53EL6$_{1,2}$: Programmed cell death protein 4	2/2	2	3	127	126	3	**0.92**
					129	128	3	**2.57**
					131	130	3	**1.56**
1141	PDCD6IP Q8WUM4$_{1,2}$: Programmed cell death 6-interacting protein	2/3	17	20	127	126	18	**1.56**
					129	128	18	**2.07**
					131	130	19	**1.16**
1142	PDGFB P01127$_{1,2}$: Platelet-derived growth factor subunit B	2/2	2	4	127	126	4	**0.71**
					129	128	4	**0.83**
					131	130	4	**0.83**

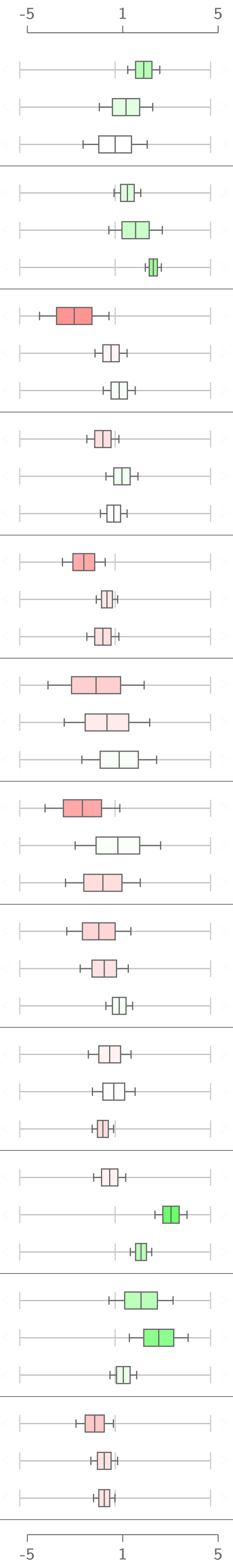

#	protein	group	peptides	spectra	ch1	ch2	quant	ratio
**1143**	PDGFC Q9NRA1$_1$: Platelet-derived growth factor C	1/4	9	10	127	126	8	**0.76**
					129	128	8	**0.87**
					131	130	7	**1.18**
**1144**	PDGFD Q9GZP0$_{1,2}$: Platelet-derived growth factor D	2/2	3	4	127	126	4	**0.58**
					129	128	4	**0.72**
					131	130	4	**1.17**
**1145**	PDGFRL Q15198: Platelet-derived growth factor receptor-like protein		8	10	127	126	8	**0.63**
					129	128	8	**1.03**
					131	130	8	**0.91**
**1146**	PDIA3 P30101: Protein disulfide-isomerase A3		9	13	127	126	12	**2.47**
					129	128	13	**1.18**
					131	130	13	**1.37**
**1147**	PDIA4 P13667: Protein disulfide-isomerase A4		15	16	127	126	14	**0.95**
					129	128	14	**1.06**
					131	130	13	**1.28**
**1148**	PDIA6 Q15084$_{1-5}$: Protein disulfide-isomerase A6	5/5	11	15	127	126	15	**1.75**
					129	128	14	**1.46**
					131	130	12	**1.14**
**1149**	PDLIM5 Q96HC4$_{1,4,6,7}$: PDZ and LIM domain protein 5	4/4	5	7	127	126	7	**1.02**
					129	128	7	**2.27**
					131	130	7	**1.47**
**1150**	PEA15 Q15121$_{1,2}$: Astrocytic phosphoprotein PEA-15	2/2	3	5	127	126	5	**0.99**
					129	128	5	**1.24**
					131	130	4	**0.93**
**1151**	PENK P01210: Proenkephalin-A [Cleaved into: Synenkephalin; Met-enkephalin		13	17	127	126	16	**0.57**
					129	128	16	**0.60**
					131	130	16	**0.96**
**1152**	PFKL P17858$_{1,2}$: ATP-dependent 6-phosphofructokinase, liver type	2/2	5	6	127	126	4	**1.47**
					129	128	3	**1.47**
					131	130	5	**0.89**
**1153**	PFKM P08237$_{1,3}$: ATP-dependent 6-phosphofructokinase, muscle type	2/3	4	4	127	126	3	**0.79**
					129	128	3	**1.70**
					131	130	3	**1.12**
**1154**	PFN1 P07737: Profilin-1		7	20	127	126	20	**1.91**
					129	128	18	**1.29**
					131	130	20	**0.89**

#	protein	group	peptides	spectra	ch1	ch2	quant	ratio
1155	PFN2 P35080$_{1,2}$: Profilin-2	2/2	1	1	127	126	1	**0.86**
					129	128	1	**1.58**
					131	130	1	**1.33**
1156	PGA3 P0DJD8, PGA4 P0DJD7, PGA5 P0DJD9: Pepsin A-3, Pepsin A-4, . . .	3/3	2	3	127	126	3	**2.50**
					129	128	3	**0.96**
					131	130	3	**0.96**
1157	PGAM1 P18669: Phosphoglycerate mutase 1	1/3	4	5	127	126	5	**3.59**
					129	128	4	**1.70**
					131	130	3	**0.83**
1158	PGD P52209$_{1,2}$: 6-phosphogluconate dehydrogenase, decarboxylating	2/2	10	12	127	126	12	**3.23**
					129	128	11	**1.55**
					131	130	11	**0.82**
1159	PGK1 P00558$_{1,2}$: Phosphoglycerate kinase 1	2/3	10	10	127	126	9	**3.69**
					129	128	9	**1.32**
					131	130	9	**1.19**
1160	PGLS O95336: 6-phosphogluconolactonase		5	5	127	126	4	**1.71**
					129	128	4	**1.97**
					131	130	5	**0.79**
1161	PGLYRP2 Q96PD5$_{1,2}$: N-acetylmuramoyl-L-alanine amidase	2/2	11	69	127	126	68	**1.74**
					129	128	67	**1.47**
					131	130	68	**0.87**
1162	PGM1 P36871$_{1}$: Phosphoglucomutase-1	1/3	9	10	127	126	10	**1.75**
					129	128	9	**1.96**
					131	130	10	**1.08**
1163	PGM2 Q96G03$_{1}$: Phosphoglucomutase-2	1/2	5	5	127	126	5	**1.56**
					129	128	4	**2.83**
					131	130	5	**0.97**
1164	PGM3 O95394$_{1,3,4}$: Phosphoacetylglucosamine mutase	3/3	5	5	127	126	5	**1.39**
					129	128	4	**2.03**
					131	130	3	**0.93**
1165	PGRMC1 O00264$_{1,2}$: Membrane-associated progesterone receptor component 1	2/2	3	4	127	126	4	**0.93**
					129	128	4	**1.34**
					131	130	4	**1.02**
1166	PHB P35232$_{1}$: Prohibitin		2	2	127	126	2	**3.16**
					129	128	2	**3.04**
					131	130	2	**1.46**

#	protein	group	peptides	spectra	ch1	ch2	quant	ratio
**1167**	PHB2 Q99623$_1$: Prohibitin-2		3	3	127	126	3	**1.34**
					129	128	3	**0.38**
					131	130	3	**0.97**
**1168**	PHGDH O43175: D-3-phosphoglycerate dehydrogenase		11	19	127	126	18	**1.33**
					129	128	19	**2.09**
					131	130	19	**2.07**
**1169**	PHPT1 Q9NRX4$_{1,2}$: 14 kDa phosphohistidine phosphatase	2/2	1	2	127	126	2	**0.87**
					129	128	2	**1.27**
					131	130	2	**1.12**
**1170**	PI3 P19957: Elafin		6	8	127	126	7	**10.68**
					129	128	7	**0.91**
					131	130	7	**0.92**
**1171**	PIGR P01833: Polymeric immunoglobulin receptor		5	5	127	126	4	**0.84**
					129	128	5	**0.85**
					131	130	4	**0.97**
**1172**	PIN1 Q13526: Peptidyl-prolyl cis-trans isomerase NIMA-interacting 1		1	1	127	126	1	**0.76**
					129	128	1	**1.62**
					131	130	1	**1.19**
**1173**	PITPNA Q00169: Phosphatidylinositol transfer protein alpha isoform		5	6	127	126	5	**1.55**
					129	128	6	**1.42**
					131	130	6	**0.74**
**1174**	PKDCC Q504Y2: Extracellular tyrosine-protein kinase PKDCC		6	7	127	126	7	**0.78**
					129	128	6	**1.09**
					131	130	7	**1.01**
**1175**	PKM P14618$_1$: Pyruvate kinase PKM	1/5	32	59	127	126	57	**1.82**
					129	128	56	**1.95**
					131	130	57	**1.28**
**1176**	PKP1 Q13835$_{1,2}$: Plakophilin-1	2/2	12	22	127	126	16	**2.34**
					129	128	20	**0.91**
					131	130	19	**0.99**
**1177**	PKP3 Q9Y446$_{1,2}$: Plakophilin-3	2/2	2	2	127	126	2	**0.72**
					129	128	2	**0.94**
					131	130	2	**0.97**
**1178**	PLA1A Q53H76$_{1,3}$: Phospholipase A1 member A	2/4	4	4	127	126	4	**1.07**
					129	128	3	**1.02**
					131	130	4	**1.07**

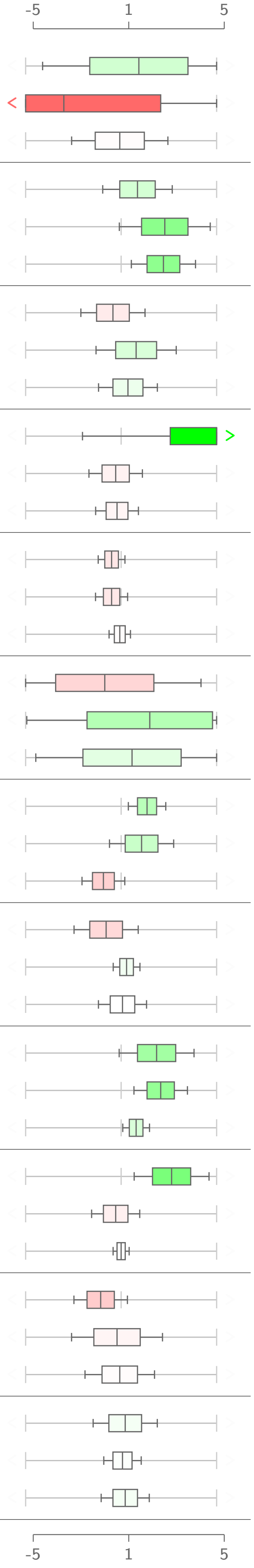

#	protein	group	peptides	spectra	ch1	ch2	quant	ratio
1179	PLA2G12A Q9BZM1: Group XIIA secretory phospholipase A2		4	62	127	126	42	**0.75**
					129	128	36	**0.97**
					131	130	60	**0.89**
1180	PLA2G2A P14555: Phospholipase A2, membrane associated		12	37	127	126	32	**0.83**
					129	128	33	**0.59**
					131	130	27	**0.89**
1181	PLAT $P00750_{1}$: Tissue-type plasminogen activator	1/3	17	22	127	126	5	**0.91**
					129	128	6	**0.95**
					131	130	6	**1.00**
1182	PLAT $P00750_{3}$: Tissue-type plasminogen activator		14	17	127	126	1	**0.75**
					129	128	1	**0.79**
					131	130	1	**0.88**
1183	PLCD1 $P51178_{1,2}$: 1-phosphatidylinositol 4,5-bisphosphate phosphodiesterase delta-1	2/2	2	2	127	126	2	**0.34**
					129	128	2	**1.33**
					131	130	2	**1.10**
1184	PLD4 Q96BZ4: Phospholipase D4		2	2	127	126	2	**0.87**
					129	128	2	**0.98**
					131	130	2	**0.90**
1185	PLEC $Q15149_{5}$: Plectin	1/9	110	125	127	126	109	**1.56**
					129	128	108	**2.15**
					131	130	110	**1.45**
1186	PLG P00747: Plasminogen	1/3	56	726	127	126	641	**1.22**
					129	128	629	**0.91**
					131	130	634	**1.12**
1187	PLIN3 $O60664_{1,3,4}$: Perilipin-3	3/4	8	8	127	126	7	**1.16**
					129	128	8	**1.51**
					131	130	8	**1.40**
1188	PLIN4 $Q96Q06_{1,2}$: Perilipin-4	2/2	6	6	127	126	4	**1.93**
					129	128	6	**1.06**
					131	130	6	**1.64**
1189	PLOD1 $Q02809_{1,2}$: Procollagen-lysine,2-oxoglutarate 5-dioxygenase 1	2/2	5	6	127	126	5	**0.58**
					129	128	6	**0.92**
					131	130	6	**0.90**
1190	PLOD3 O60568: Procollagen-lysine,2-oxoglutarate 5-dioxygenase 3		10	12	127	126	12	**0.50**
					129	128	11	**0.85**
					131	130	10	**0.90**

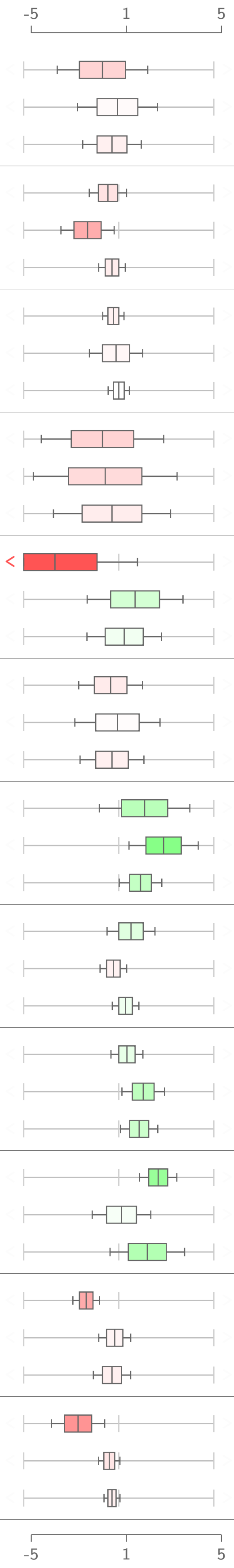

#	protein	group	peptides	spectra	ch1	ch2	quant	ratio
1191	PLS3 P13797$_{1,2}$: Plastin-3	2/3	21	52	127	126	18	**3.03**
					129	128	19	**2.16**
					131	130	18	**0.90**
1192	PLTP P55058$_{1}$: Phospholipid transfer protein	1/4	19	342	127	126	329	**1.52**
					129	128	314	**0.98**
					131	130	308	**1.02**
1193	PLXDC2 Q6UX71$_{1,2}$: Plexin domain-containing protein 2	2/2	2	2	127	126	2	**1.78**
					129	128	2	**1.04**
					131	130	2	**0.81**
1194	PLXNB2 O15031: Plexin-B2		3	5	127	126	4	**0.92**
					129	128	4	**1.09**
					131	130	4	**0.95**
1195	PMVK Q15126: Phosphomevalonate kinase		2	2	127	126	2	**1.31**
					129	128	2	**1.63**
					131	130	2	**1.20**
1196	PNP P00491: Purine nucleoside phosphorylase		2	10	127	126	8	**0.66**
					129	128	7	**0.86**
					131	130	8	**0.75**
1197	PNPLA8 Q9NP80$_{1-3}$: Calcium-independent phospholipase A2-gamma	3/3	2	4	127	126	1	**1.80**
					129	128	1	**0.60**
					131	130	1	**0.41**
1198	PODN Q7Z5L7$_{1-3}$: Podocan	3/4	20	28	127	126	27	**0.94**
					129	128	27	**1.27**
					131	130	23	**0.86**
1199	POF1B Q8WVV4$_{1,2}$: Protein POF1B	2/3	14	17	127	126	15	**5.90**
					129	128	14	**0.85**
					131	130	13	**0.95**
1200	POGZ Q7Z3K3$_{1-3,5-7}$: Pogo transposable element with ZNF domain	6/6	3	28	127	126	20	**0.88**
					129	128	23	**0.92**
					131	130	27	**1.25**
1201	POMGNT1 Q8WZA1$_{1,2}$: Protein O-linked-mannose beta-1,2-N-acetylglucosaminyltransferase 1	2/2	6	8	127	126	4	**1.09**
					129	128	5	**1.04**
					131	130	7	**0.67**
1202	PON1 P27169: Serum paraoxonase/arylesterase 1	1/4	14	149	127	126	129	**0.80**
					129	128	127	**0.81**
					131	130	133	**0.98**

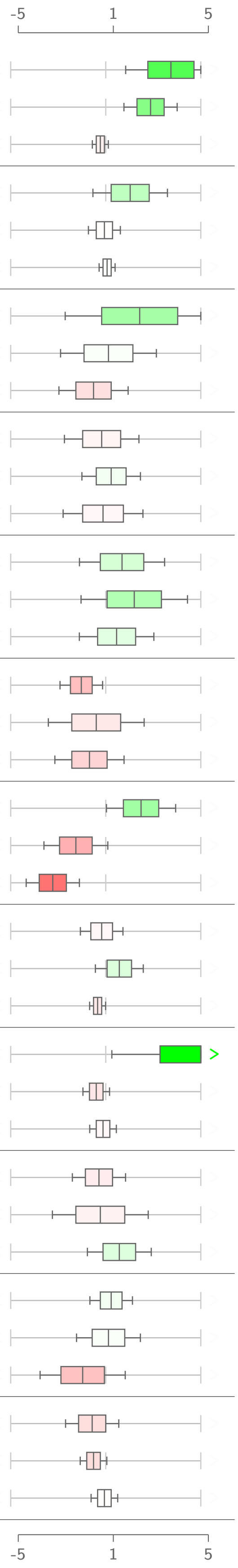

#	protein	group	peptides	spectra	ch1	ch2	quant	ratio
1203	PON3 Q15166: Serum paraoxonase/lactonase 3		8	19	127	126	6	**1.32**
					129	128	6	**0.87**
					131	130	5	**0.96**
1204	POSTN Q15063$_1$: Periostin		48	265	127	126	3	**1.56**
					129	128	3	**1.58**
					131	130	3	**1.04**
1211	POTEE Q6S8J3$_1$: POTE ankyrin domain family member E		14	168	127	126	37	**0.83**
					129	128	30	**1.34**
					131	130	37	**1.35**
1212	POTEF A5A3E0: POTE ankyrin domain family member F		12	120	127	126	3	**0.66**
					129	128	4	**1.12**
					131	130	4	**0.98**
1213	POTEH Q6S545$_{1,2}$: POTE ankyrin domain family member H	2/2	7	62	127	126	15	**0.54**
					129	128	14	**1.09**
					131	130	14	**0.96**
1215	PPA1 Q15181: Inorganic pyrophosphatase		3	4	127	126	4	**1.51**
					129	128	4	**1.73**
					131	130	3	**1.19**
1216	PPIA P62937$_1$: Peptidyl-prolyl cis-trans isomerase A	1/8	11	28	127	126	26	**1.48**
					129	128	25	**1.02**
					131	130	26	**1.13**
1217	PPIB P23284: Peptidyl-prolyl cis-trans isomerase B		6	7	127	126	6	**3.08**
					129	128	6	**1.37**
					131	130	5	**1.26**
1218	PPL O60437: Periplakin		44	47	127	126	41	**4.18**
					129	128	45	**1.47**
					131	130	45	**1.20**
1219	PPM1F P49593$_1$: Protein phosphatase 1F		3	4	127	126	2	**0.52**
					129	128	2	**0.74**
					131	130	2	**0.87**
1220	PPP1CA P62136$_{1-3}$, PPP1CB P62140, PPP1CC P36873$_{1,2}$: Serine/threonine-protein phosphatase PP1-alpha catalytic subunit, Serine/threonine-protein phosphatase PP1-beta catalytic subunit, [illegible]	6/6	3	3	127	126	3	**1.06**
					129	128	3	**2.14**
					131	130	3	**1.49**
1221	PPP1R7 Q15435$_1$: Protein phosphatase 1 regulatory subunit 7	1/5	4	4	127	126	3	**0.59**
					129	128	4	**1.60**
					131	130	4	**1.08**

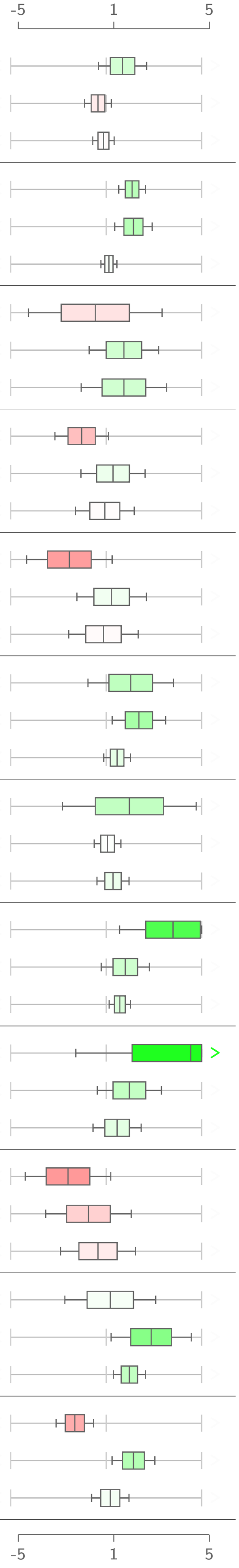

#	protein	group	peptides	spectra	ch1	ch2	quant	ratio
**1223**	PPP2CA P67775$_2$: Serine/threonine-protein phosphatase 2A catalytic subunit alpha isoform		4	5	127	126	1	**0.06**
					129	128	1	**0.57**
					131	130	1	**1.23**
**1224**	PPP2CB P62714: Serine/threonine-protein phosphatase 2A catalytic subunit beta isoform		4	5	127	126	1	**1.13**
					129	128	1	**1.41**
					131	130	1	**1.85**
**1225**	PPP2R1A P30153: Serine/threonine-protein phosphatase 2A 65 kDa regulatory subunit A alpha is . . .		10	13	127	126	11	**1.12**
					129	128	13	**2.16**
					131	130	11	**1.19**
**1226**	PPP3CA Q08209$_{1-3}$: Serine/threonine-protein phosphatase 2B catalytic subunit alpha isoform	3/3	2	2	127	126	2	**0.91**
					129	128	2	**1.66**
					131	130	2	**1.09**
**1227**	PRAP1 Q96NZ9$_1$: Proline-rich acidic protein 1	1/3	5	12	127	126	3	**0.84**
					129	128	3	**0.91**
					131	130	3	**0.84**
**1228**	PRAP1 Q96NZ9$_3$: Proline-rich acidic protein 1		5	12	127	126	3	**0.76**
					129	128	3	**0.93**
					131	130	3	**0.93**
**1229**	PRDM10 Q9NQV6$_6$: PR domain zinc finger protein 10		6	10	127	126	6	**0.72**
					129	128	5	**0.94**
					131	130	7	**0.93**
**1230**	PRDX1 Q06830: Peroxiredoxin-1		7	9	127	126	7	**3.93**
					129	128	6	**2.16**
					131	130	5	**0.96**
**1231**	PRDX2 P32119$_1$: Peroxiredoxin-2		7	10	127	126	8	**2.34**
					129	128	5	**4.66**
					131	130	7	**1.01**
**1232**	PRDX3 P30048$_{1,2}$: Thioredoxin-dependent peroxide reductase, mitochondrial	2/2	2	2	127	126	2	**1.59**
					129	128	2	**1.84**
					131	130	2	**1.42**
**1233**	PRDX5 P30044$_{1-3}$: Peroxiredoxin-5, mitochondrial	3/4	2	2	127	126	2	**1.55**
					129	128	2	**2.38**
					131	130	2	**1.28**
**1234**	PRDX6 P30041: Peroxiredoxin-6		11	23	127	126	21	**1.68**
					129	128	20	**2.32**
					131	130	23	**1.85**

#	protein	group	peptides	spectra	ch1	ch2	quant	ratio
**1235**	PRELP P51888: Prolargin		7	10	127	126	9	**0.97**
					129	128	10	**1.12**
					131	130	10	**0.83**
**1236**	PREP P48147: Prolyl endopeptidase		2	4	127	126	4	**0.86**
					129	128	3	**1.15**
					131	130	4	**1.57**
**1237**	PRG2 $P13727_{1,2}$: Bone marrow proteoglycan	2/2	2	2	127	126	2	**0.85**
					129	128	2	**1.21**
					131	130	2	**1.34**
**1238**	PRG4 $Q92954_{3}$: Proteoglycan 4	1/2	51	372	127	126	8	**0.61**
					129	128	6	**1.42**
					131	130	12	**1.07**
**1239**	PRG4 $Q92954_{6}$: Proteoglycan 4	1/4	53	366	127	126	6	**2.52**
					129	128	6	**1.53**
					131	130	7	**1.01**
**1240**	PRKAA1 $Q13131_{1,2}$: 5'-AMP-activated protein kinase catalytic subunit alpha-1	2/2	2	2	127	126	1	**0.71**
					129	128	1	**1.32**
					131	130	1	**1.20**
**1241**	PRKAR1A $P10644_{1}$: cAMP-dependent protein kinase type I-alpha regulatory subunit	1/2	4	4	127	126	4	**1.10**
					129	128	4	**1.73**
					131	130	4	**0.88**
**1242**	PRKAR2A $P13861_{1}$: cAMP-dependent protein kinase type II-alpha regulatory subunit	1/2	8	9	127	126	9	**0.90**
					129	128	8	**2.20**
					131	130	9	**1.40**
**1243**	PRKCSH $P14314_{1,2}$: Glucosidase 2 subunit beta	2/2	11	15	127	126	13	**1.17**
					129	128	15	**0.98**
					131	130	15	**1.31**
**1244**	PRKDC $P78527_{1,2}$: DNA-dependent protein kinase catalytic subunit	2/2	5	5	127	126	3	**0.87**
					129	128	5	**1.36**
					131	130	5	**1.15**
**1245**	PRMT1 $Q99873_{1-4}$: Protein arginine N-methyltransferase 1	4/4	4	4	127	126	4	**1.24**
					129	128	4	**2.10**
					131	130	4	**1.52**
**1246**	PRNP $P04156_{1,2}$: Major prion protein	2/2	4	6	127	126	6	**0.58**
					129	128	5	**0.90**
					131	130	6	**0.79**

#	protein	group	peptides	spectra	ch1	ch2	quant	ratio
**1247**	PROC P04070$_{1,2}$: Vitamin K-dependent protein C	2/2	12	25	127	126	24	**0.56**
					129	128	23	**0.73**
					131	130	23	**1.08**
**1248**	PROS1 P07225: Vitamin K-dependent protein S		28	95	127	126	86	**1.32**
					129	128	89	**0.81**
					131	130	91	**1.30**
**1249**	PROZ P22891$_{1,2}$: Vitamin K-dependent protein Z	2/2	8	9	127	126	8	**0.97**
					129	128	9	**0.82**
					131	130	8	**1.12**
**1250**	PRPF19 Q9UMS4: Pre-mRNA-processing factor 19		2	2	127	126	2	**2.01**
					129	128	2	**2.45**
					131	130	2	**1.57**
**1252**	PRPS1 P60891$_{1,2}$: Ribose-phosphate pyrophosphokinase 1	2/4	4	4	127	126	4	**1.53**
					129	128	3	**2.29**
					131	130	4	**1.39**
**1253**	PRPSAP2 O60256$_{1-4}$: Phosphoribosyl pyrophosphate synthase-associated protein 2	4/4	2	2	127	126	2	**2.07**
					129	128	2	**2.15**
					131	130	2	**1.23**
**1254**	PRSS1 P07477: Trypsin-1		2	27	127	126	1	**0.55**
					129	128	1	**0.85**
					131	130	1	**1.08**
**1255**	PRSS2 P07478: Trypsin-2	1/6	5	33	127	126	6	**0.58**
					129	128	6	**0.85**
					131	130	6	**0.99**
**1256**	PRSS23 O95084$_{1}$: Serine protease 23		4	5	127	126	3	**0.59**
					129	128	3	**1.00**
					131	130	3	**1.25**
**1257**	PRSS23 O95084$_{2}$: Serine protease 23		3	3	127	126	1	**1.64**
					129	128	1	**1.01**
					131	130	1	**0.86**
**1258**	PRSS3P2 Q8NHM4: Putative trypsin-6		3	28	127	126	1	**0.58**
					129	128	1	**0.85**
					131	130	1	**0.88**
**1259**	PSAP P07602$_{1-3}$: Prosaposin	3/3	19	42	127	126	40	**0.66**
					129	128	39	**0.78**
					131	130	39	**0.80**

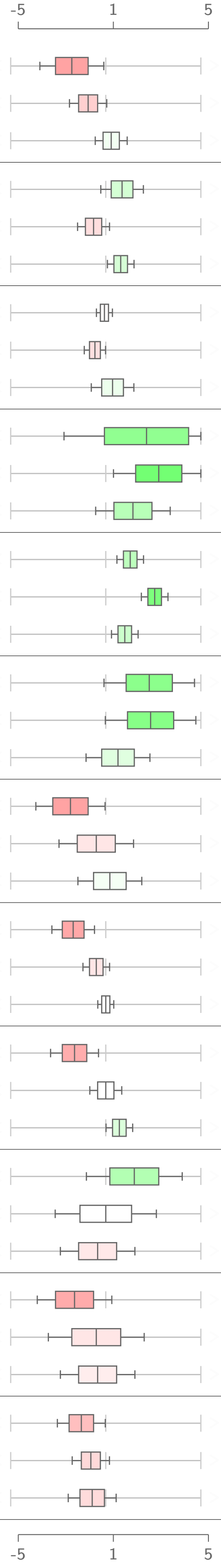

#	protein	group	peptides	spectra	ch1	ch2	quant	ratio
**1260**	PSMA1 P25786$_{1,2}$: Proteasome subunit alpha type-1	2/2	3	3	127	126	3	**1.78**
					129	128	3	**1.71**
					131	130	3	**1.08**
**1261**	PSMA2 P25787: Proteasome subunit alpha type-2		2	2	127	126	2	**2.04**
					129	128	2	**1.65**
					131	130	2	**1.22**
**1262**	PSMA3 P25788$_{1,2}$: Proteasome subunit alpha type-3	2/2	4	4	127	126	4	**1.72**
					129	128	4	**1.77**
					131	130	4	**1.25**
**1263**	PSMA4 P25789$_{1}$: Proteasome subunit alpha type-4	1/2	5	5	127	126	3	**2.15**
					129	128	5	**1.71**
					131	130	5	**1.21**
**1264**	PSMA6 P60900$_{1,2}$: Proteasome subunit alpha type-6	2/2	2	2	127	126	2	**1.86**
					129	128	2	**1.47**
					131	130	2	**1.34**
**1265**	PSMA7 O14818$_{1}$: Proteasome subunit alpha type-7	1/2	4	5	127	126	5	**1.62**
					129	128	5	**1.54**
					131	130	3	**1.52**
**1266**	PSMB1 P20618: Proteasome subunit beta type-1		2	3	127	126	3	**1.07**
					129	128	3	**1.10**
					131	130	3	**1.39**
**1267**	PSMB2 P49721: Proteasome subunit beta type-2		2	2	127	126	1	**2.38**
					129	128	1	**1.60**
					131	130	1	**1.10**
**1268**	PSMB7 Q99436$_{1}$: Proteasome subunit beta type-7	1/2	3	4	127	126	4	**1.92**
					129	128	4	**1.21**
					131	130	4	**1.31**
**1269**	PSMC1 P62191$_{1}$: 26S protease regulatory subunit 4	1/2	5	6	127	126	4	**2.30**
					129	128	5	**1.65**
					131	130	3	**1.13**
**1270**	PSMC2 P35998$_{1}$: 26S protease regulatory subunit 7	1/2	3	3	127	126	3	**1.72**
					129	128	3	**1.42**
					131	130	3	**0.98**
**1271**	PSMC5 P62195$_{1,2}$: 26S protease regulatory subunit 8	2/2	3	3	127	126	2	**1.99**
					129	128	2	**2.12**
					131	130	2	**0.80**

#	protein	group	peptides	spectra	ch1	ch2	quant	ratio
**1272**	PSMD1 Q99460$_{1,2}$: 26S proteasome non-ATPase regulatory subunit 1	2/2	5	5	127	126	3	**2.05**
					129	128	4	**2.20**
					131	130	4	**1.16**
**1273**	PSMD12 O00232$_{1}$: 26S proteasome non-ATPase regulatory subunit 12	1/2	3	3	127	126	3	**0.69**
					129	128	3	**1.78**
					131	130	3	**0.83**
**1274**	PSMD13 Q9UNM6$_{1,2}$: 26S proteasome non-ATPase regulatory subunit 13	2/2	3	3	127	126	3	**2.16**
					129	128	3	**1.35**
					131	130	3	**0.89**
**1275**	PSMD2 Q13200$_{1}$: 26S proteasome non-ATPase regulatory subunit 2	1/3	7	9	127	126	8	**1.75**
					129	128	9	**1.46**
					131	130	9	**1.10**
**1276**	PSMD3 O43242$_{1}$: 26S proteasome non-ATPase regulatory subunit 3		6	7	127	126	5	**0.88**
					129	128	6	**0.82**
					131	130	7	**0.89**
**1277**	PSMD5 Q16401$_{1,2}$: 26S proteasome non-ATPase regulatory subunit 5	2/2	1	1	127	126	1	**0.72**
					129	128	1	**1.26**
					131	130	1	**0.89**
**1278**	PSMD6 Q15008$_{1}$: 26S proteasome non-ATPase regulatory subunit 6	1/3	3	3	127	126	1	**2.00**
					129	128		
					131	130	1	**1.79**
**1279**	PSMD6 Q15008$_{2}$: 26S proteasome non-ATPase regulatory subunit 6		3	3	127	126	1	**0.64**
					129	128	1	**1.60**
					131	130	1	**0.94**
**1280**	PSME1 Q06323$_{1-3}$: Proteasome activator complex subunit 1	3/3	5	7	127	126	7	**1.95**
					129	128	7	**2.34**
					131	130	7	**1.45**
**1281**	PSME2 Q9UL46: Proteasome activator complex subunit 2		10	18	127	126	17	**1.63**
					129	128	16	**1.96**
					131	130	17	**1.39**
**1282**	PTBP1 P26599$_{1-3}$: Polypyrimidine tract-binding protein 1	3/10	4	5	127	126	5	**3.08**
					129	128	5	**3.47**
					131	130	5	**1.59**
**1283**	PTGDS P41222: Prostagland-in-H2 D-isomerase		5	8	127	126	7	**0.56**
					129	128	8	**0.96**
					131	130	8	**0.83**

#	protein	group	peptides	spectra	ch1	ch2	quant	ratio
**1284**	PTGES3 Q15185$_{1-4}$: Prostaglandin E synthase 3	4/4	2	2	127	126	2	**1.19**
					129	128	2	**1.04**
					131	130	2	**1.32**
**1285**	PTGFRN Q9P2B2: Prostaglandin F2 receptor negative regulator		1	1	127	126	1	**1.71**
					129	128	1	**1.37**
					131	130	1	**0.53**
**1286**	PTGIS Q16647: Prostacyclin synthase		2	2	127	126	2	**1.12**
					129	128	2	**1.75**
					131	130	2	**1.20**
**1287**	PTGR1 Q14914$_{1,2}$: Prostaglandin reductase 1	2/2	2	2	127	126	2	**4.49**
					129	128	2	**2.18**
					131	130	2	**0.87**
**1288**	PTMA P06454$_{1,2}$: Prothymosin alpha [Cleaved into: Prothymosin alpha, N-terminally processed; . . .	2/2	2	2	127	126	1	**4.87**
					129	128	2	**1.66**
					131	130	2	**3.28**
**1289**	PTMS P20962: Parathymosin		4	5	127	126	5	**1.97**
					129	128	4	**2.62**
					131	130	4	**1.64**
**1290**	PTN P21246: Pleiotrophin		2	2	127	126	2	**0.71**
					129	128	2	**0.70**
					131	130	2	**1.02**
**1291**	PTPN11 Q06124$_{2,3}$: Tyrosine-protein phosphatase non-receptor type 11	2/2	4	4	127	126	3	**1.06**
					129	128	4	**1.46**
					131	130	3	**1.20**
**1292**	PTPRF P10586$_{1,2}$: Receptor-type tyrosine-protein phosphatase F	2/2	3	3	127	126	3	**0.83**
					129	128	3	**1.13**
					131	130	3	**1.10**
**1293**	PTRF Q6NZI2$_{1}$: Polymerase I and transcript release factor	1/3	10	12	127	126	12	**0.69**
					129	128	11	**1.97**
					131	130	12	**1.79**
**1294**	PTX3 P26022: Pentraxin-related protein PTX3		2	2	127	126	2	**1.32**
					129	128	2	**1.10**
					131	130	2	**0.60**
**1295**	PURA Q00577: Transcriptional activator protein Pur-alpha		2	2	127	126	2	**1.15**
					129	128	2	**1.99**
					131	130	2	**1.30**

#	protein	group	peptides	spectra	ch1	ch2	quant	ratio
**1296**	PXDN Q92626$_1$: Peroxidasin homolog	1/2	30	42	127	126	33	**0.74**
					129	128	39	**1.10**
					131	130	39	**0.97**
**1297**	PYCARD Q9ULZ3$_{1-3}$: Apoptosis-associated speck-like protein containing a CARD	3/3	3	3	127	126	3	**5.98**
					129	128	3	**1.43**
					131	130	3	**1.03**
**1298**	PZP P20742$_1$: Pregnancy zone protein	1/2	39	80	127	126	54	**1.01**
					129	128	55	**1.07**
					131	130	57	**0.83**
**1299**	QPCT Q16769$_1$: Glutaminyl-peptide cyclotransferase	1/2	5	8	127	126	7	**0.75**
					129	128	8	**0.81**
					131	130	8	**0.96**
**1300**	QSOX1 O00391$_1$: Sulfhydryl oxidase 1	1/2	40	146	127	126	139	**1.24**
					129	128	142	**1.17**
					131	130	140	**0.71**
**1301**	QTRT2 Q9H974$_{1-4}$: Queuine tRNA-ribosyltransferase accessory subunit 2	4/4	3	39	127	126	17	**0.63**
					129	128	15	**0.80**
					131	130	28	**1.31**
**1302**	RAB10 P61026: Ras-related protein Rab-10		5	8	127	126	5	**2.62**
					129	128	5	**1.32**
					131	130	4	**0.78**
**1303**	RAB11B Q15907$_1$: Ras-related protein Rab-11B	1/4	6	7	127	126	7	**2.08**
					129	128	7	**1.63**
					131	130	7	**1.00**
**1304**	RAB14 P61106: Ras-related protein Rab-14		3	4	127	126	4	**2.38**
					129	128	4	**1.95**
					131	130	4	**0.97**
**1305**	RAB1A P62820$_1$: Ras-related protein Rab-1A	1/3	6	7	127	126	1	**4.14**
					129	128	1	**1.69**
					131	130	1	**0.72**
**1306**	RAB1B Q9H0U4: Ras-related protein Rab-1B	1/2	6	7	127	126	1	**2.42**
					129	128	1	**1.78**
					131	130	1	**0.97**
**1307**	RAB2A P61019$_{1,2}$: Ras-related protein Rab-2A	2/4	3	3	127	126	3	**1.47**
					129	128	3	**1.63**
					131	130	3	**0.83**

#	protein	group	peptides	spectra	ch1	ch2	quant	ratio
**1309**	RAB5A P20339$_{1,2}$: Ras-related protein Rab-5A	2/2	1	1	127	126	1	**5.34**
					129	128	1	**1.31**
					131	130	1	**1.07**
**1310**	RAB5C P51148$_{1,2}$: Ras-related protein Rab-5C	2/2	2	2	127	126	2	**1.78**
					129	128	2	**1.11**
					131	130	2	**0.86**
**1311**	RAB7A P51149: Ras-related protein Rab-7a		5	8	127	126	8	**1.80**
					129	128	7	**1.25**
					131	130	7	**1.09**
**1312**	RAB8B Q92930: Ras-related protein Rab-8B	1/4	4	5	127	126	3	**1.25**
					129	128	3	**1.27**
					131	130	3	**1.02**
**1313**	RABEP1 Q15276$_{1,2}$: Rab GTPase-binding effector protein 1	2/2	6	33	127	126	27	**0.83**
					129	128	25	**1.18**
					131	130	26	**1.23**
**1314**	RAC1 P63000$_{1,2}$: Ras-related C3 botulinum toxin substrate 1	2/4	6	6	127	126	5	**1.51**
					129	128	4	**1.48**
					131	130	5	**0.99**
**1315**	RACK1 P63244: Receptor of activated protein C kinase 1		12	12	127	126	10	**1.51**
					129	128	9	**2.35**
					131	130	12	**2.01**
**1316**	RAD23B P54727$_{1}$: UV excision repair protein RAD23 homolog B	1/4	4	4	127	126	4	**1.52**
					129	128	4	**2.04**
					131	130	3	**1.29**
**1317**	RALY Q9UKM9$_{1,2}$: RNA-binding protein Raly	2/2	3	3	127	126	3	**2.08**
					129	128	3	**2.17**
					131	130	3	**1.56**
**1318**	RAN P62826: GTP-binding nuclear protein Ran		4	7	127	126	7	**1.46**
					129	128	7	**2.09**
					131	130	7	**1.58**
**1319**	RAP1B P61224$_{3}$: Ras-related protein Rap-1b	1/5	3	3	127	126	3	**1.46**
					129	128	3	**1.32**
					131	130	3	**1.02**
**1320**	RARRES1 P49788$_{1,2}$: Retinoic acid receptor responder protein 1	2/2	7	12	127	126	11	**1.67**
					129	128	8	**1.07**
					131	130	12	**0.80**

#	protein	group	peptides	spectra	ch1	ch2	quant	ratio
**1321**	RARRES2 Q99969: Retinoic acid receptor responder protein 2		8	47	127	126	45	**1.02**
					129	128	47	**1.10**
					131	130	44	**0.97**
**1322**	RARS $P54136_{1,2}$: Arginine--tRNA ligase, cytoplasmic	2/2	3	3	127	126	3	**2.21**
					129	128	3	**2.27**
					131	130	3	**1.00**
**1323**	RBMX $P38159_{1,2}$: RNA-binding motif protein, X chromosome	2/3	9	12	127	126	11	**1.00**
					129	128	12	**1.80**
					131	130	12	**1.11**
**1324**	RBP4 P02753: Retinol-binding protein 4		10	130	127	126	120	**0.71**
					129	128	112	**1.08**
					131	130	116	**1.11**
**1325**	RCN1 $Q15293_{1,2}$: Reticulocalbin-1	2/2	3	3	127	126	3	**0.47**
					129	128	3	**0.95**
					131	130	3	**0.82**
**1326**	RCN3 Q96D15: Reticulocalbin-3		3	5	127	126	5	**0.41**
					129	128	5	**0.90**
					131	130	4	**0.79**
**1327**	RHOA P61586: Transforming protein RhoA		6	7	127	126	2	**3.38**
					129	128	2	**1.77**
					131	130	2	**1.06**
**1328**	RHOC P08134: Rho-related GTP-binding protein RhoC		5	6	127	126	1	**1.15**
					129	128	1	**2.07**
					131	130	1	**1.11**
**1329**	RHOJ $Q9H4E5_{1,2}$: Rho-related GTP-binding protein RhoJ	2/4	3	3	127	126	2	**0.73**
					129	128	2	**0.75**
					131	130	2	**0.95**
**1330**	RIC8A $Q9NPQ8_{1-4}$: Synembryn-A	4/4	3	4	127	126	2	**1.30**
					129	128	2	**1.83**
					131	130	2	**1.22**
**1331**	RNASE1 P07998: Ribonuclease pancreatic		6	25	127	126	24	**0.59**
					129	128	22	**0.61**
					131	130	24	**0.90**
**1332**	RNASE4 P34096: Ribonuclease 4		13	154	127	126	144	**0.58**
					129	128	134	**0.64**
					131	130	143	**0.86**

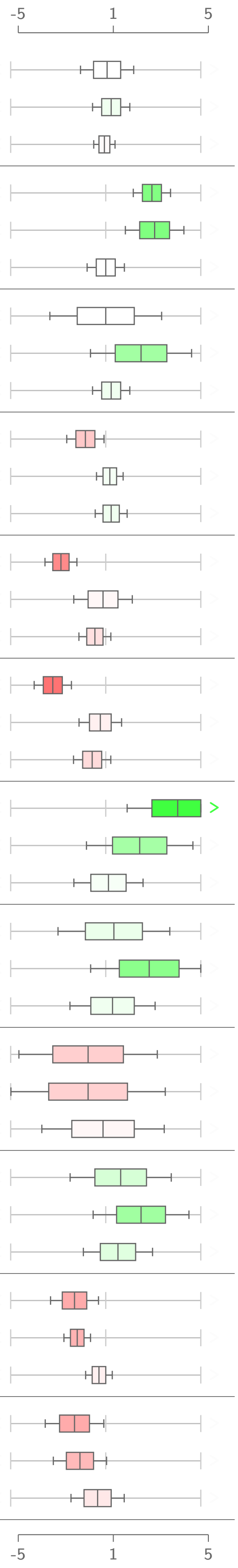

#	protein	group	peptides	spectra	ch1	ch2	quant	ratio
**1333**	RNF181 Q9P0P0: E3 ubiquitin-protein ligase RNF181		1	1	127	126	1	**0.80**
					129	128	1	**1.18**
					131	130	1	**1.51**
**1334**	RNH1 P13489: Ribonuclease inhibitor		12	18	127	126	18	**1.27**
					129	128	18	**1.47**
					131	130	16	**0.93**
**1335**	RPA2 $P15927_{1-3}$: Replication protein A 32 kDa subunit	3/3	2	2	127	126	2	**1.21**
					129	128	2	**2.45**
					131	130	2	**1.63**
**1336**	RPL10 P27635: 60S ribosomal protein L10		2	2	127	126	2	**2.27**
					129	128	2	**1.28**
					131	130	2	**1.06**
**1337**	RPL10A P62906: 60S ribosomal protein L10a		5	6	127	126	6	**1.39**
					129	128	5	**1.80**
					131	130	5	**0.96**
**1338**	RPL12 $P30050_{1}$: 60S ribosomal protein L12	1/2	8	8	127	126	7	**1.60**
					129	128	8	**2.02**
					131	130	8	**1.74**
**1339**	RPL13 $P26373_{1}$: 60S ribosomal protein L13	1/2	6	7	127	126	6	**1.53**
					129	128	6	**1.32**
					131	130	6	**1.13**
**1340**	RPL14 P50914: 60S ribosomal protein L14		8	8	127	126	8	**3.30**
					129	128	8	**1.21**
					131	130	8	**1.04**
**1341**	RPL15 $P61313_{1}$: 60S ribosomal protein L15	1/2	5	6	127	126	6	**1.65**
					129	128	6	**1.58**
					131	130	6	**1.21**
**1342**	RPL17 $P18621_{1-3}$: 60S ribosomal protein L17	3/3	3	3	127	126	2	**3.73**
					129	128	2	**1.94**
					131	130	2	**1.27**
**1343**	RPL18 $Q07020_{1,2}$: 60S ribosomal protein L18	2/2	2	2	127	126	2	**1.27**
					129	128	2	**1.49**
					131	130	2	**1.06**
**1344**	RPL19 P84098: 60S ribosomal protein L19		4	4	127	126	4	**1.73**
					129	128	4	**1.79**
					131	130	4	**1.04**

#	protein	group	peptides	spectra	ch1	ch2	quant	ratio
**1345**	RPL21 P46778: 60S ribosomal protein L21		2	3	127	126	3	**1.54**
					129	128	3	**1.87**
					131	130	3	**1.23**
**1346**	RPL22 P35268: 60S ribosomal protein L22		3	3	127	126	3	**1.54**
					129	128	3	**2.33**
					131	130	3	**1.13**
**1347**	RPL23A P62750: 60S ribosomal protein L23a		4	5	127	126	5	**3.10**
					129	128	5	**2.07**
					131	130	5	**1.11**
**1348**	RPL24 P83731: 60S ribosomal protein L24		4	5	127	126	3	**2.96**
					129	128	5	**1.72**
					131	130	4	**0.94**
**1349**	RPL26 P61254, RPL26L1 Q9UNX3: 60S ribosomal protein L26, 60S ribosomal protein L26-like 1	2/2	1	2	127	126	2	**3.51**
					129	128	2	**3.29**
					131	130	2	**0.93**
**1350**	RPL27 P61353: 60S ribosomal protein L27		5	6	127	126	6	**1.53**
					129	128	6	**1.33**
					131	130	4	**1.17**
**1351**	RPL28 P46779$_{1-3}$: 60S ribosomal protein L28	3/5	2	2	127	126	2	**2.92**
					129	128	2	**2.04**
					131	130	2	**1.27**
**1352**	RPL29 P47914: 60S ribosomal protein L29		3	3	127	126	3	**2.95**
					129	128	3	**1.62**
					131	130	3	**0.97**
**1353**	RPL3 P39023: 60S ribosomal protein L3		7	8	127	126	8	**1.76**
					129	128	7	**1.53**
					131	130	6	**1.25**
**1354**	RPL30 P62888: 60S ribosomal protein L30		4	5	127	126	5	**2.66**
					129	128	5	**1.54**
					131	130	5	**1.23**
**1355**	RPL31 P62899$_1$: 60S ribosomal protein L31	1/3	3	3	127	126	3	**1.56**
					129	128	3	**1.94**
					131	130	3	**0.92**
**1356**	RPL34 P49207: 60S ribosomal protein L34		3	4	127	126	4	**1.88**
					129	128	4	**1.39**
					131	130	4	**1.04**

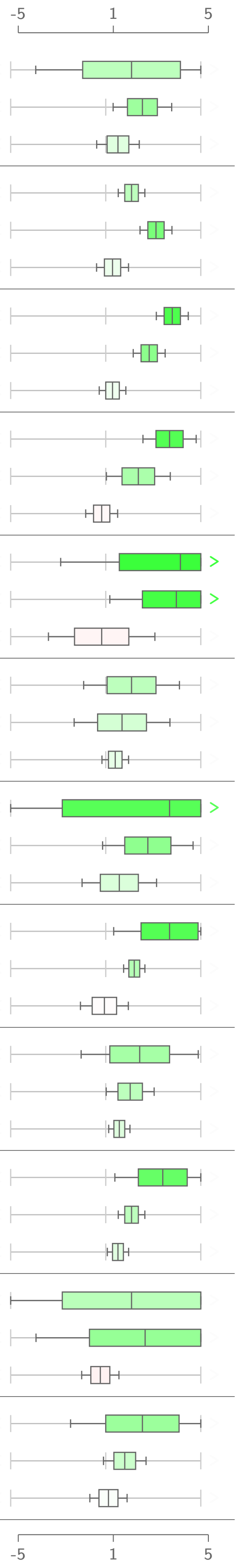

#	protein	group	peptides	spectra	ch1	ch2	quant	ratio	-5 1 5
1357	RPL35 P42766: 60S ribosomal protein L35		2	3	127	126	3	1.59	
					129	128	3	1.46	
					131	130	3	1.05	
1358	RPL35A P18077: 60S ribosomal protein L35a		4	4	127	126	4	1.66	
					129	128	4	1.59	
					131	130	3	1.10	
1359	RPL36 Q9Y3U8: 60S ribosomal protein L36		1	1	127	126	1	0.91	
					129	128	1	1.38	
					131	130	1	1.18	
1360	RPL37A P61513, RPL37AP8 A6NKH3: 60S ribosomal protein L37a, Putative 60S ribosomal protein L37a-like protein	2/2	1	1	127	126	1	2.63	
					129	128	1	1.42	
					131	130	1	0.86	
1361	RPL4 P36578: 60S ribosomal protein L4		5	5	127	126	4	2.09	
					129	128	4	1.37	
					131	130	4	1.09	
1362	RPL5 P46777: 60S ribosomal protein L5		8	12	127	126	10	2.83	
					129	128	7	2.05	
					131	130	11	1.19	
1363	RPL6 Q02878: 60S ribosomal protein L6		6	6	127	126	5	1.59	
					129	128	6	2.15	
					131	130	5	1.32	
1364	RPL7 P18124: 60S ribosomal protein L7		4	4	127	126	4	1.60	
					129	128	4	1.37	
					131	130	4	0.99	
1365	RPL7A P62424: 60S ribosomal protein L7a		4	4	127	126	4	2.08	
					129	128	4	1.63	
					131	130	4	1.20	
1366	RPL8 P62917: 60S ribosomal protein L8		7	7	127	126	6	1.29	
					129	128	6	1.68	
					131	130	7	0.84	
1367	RPL9 P32969: 60S ribosomal protein L9		6	6	127	126	6	1.54	
					129	128	5	1.72	
					131	130	4	0.99	
1368	RPLP0 P05388[1]: 60S acidic ribosomal protein P0	1/3	10	15	127	126	14	1.27	
					129	128	14	2.59	
					131	130	14	1.76	

-5 1 5

#	protein	group	peptides	spectra	ch1	ch2	quant	ratio
1369	RPLP2 P05387: 60S acidic ribosomal protein P2		2	5	127	126	3	**1.65**
					129	128	5	**2.30**
					131	130	4	**1.97**
1370	RPN1 P04843: Dolichyl-diphosphooligosaccharide--protein glycosyltransferase subunit 1		4	4	127	126	4	**3.49**
					129	128	4	**1.87**
					131	130	4	**1.41**
1371	RPS10 P46783: 40S ribosomal protein S10	1/2	8	11	127	126	11	**1.51**
					129	128	9	**1.76**
					131	130	10	**0.97**
1372	RPS11 P62280: 40S ribosomal protein S11		2	2	127	126	2	**1.52**
					129	128	2	**1.49**
					131	130	2	**1.00**
1373	RPS12 P25398: 40S ribosomal protein S12		3	3	127	126	3	**1.82**
					129	128	3	**1.85**
					131	130	3	**1.25**
1374	RPS13 P62277: 40S ribosomal protein S13		4	4	127	126	4	**1.77**
					129	128	4	**1.66**
					131	130	4	**0.98**
1375	RPS14 P62263: 40S ribosomal protein S14		4	7	127	126	6	**1.21**
					129	128	6	**2.01**
					131	130	7	**1.20**
1376	RPS15A P62244: 40S ribosomal protein S15a		2	2	127	126	2	**2.48**
					129	128	2	**1.95**
					131	130	2	**1.41**
1377	RPS16 P62249: 40S ribosomal protein S16		5	5	127	126	5	**1.31**
					129	128	4	**1.60**
					131	130	5	**1.11**
1378	RPS18 P62269: 40S ribosomal protein S18		4	4	127	126	4	**2.22**
					129	128	4	**1.72**
					131	130	4	**1.12**
1379	RPS19 P39019: 40S ribosomal protein S19		5	6	127	126	5	**2.26**
					129	128	6	**2.02**
					131	130	5	**0.98**
1380	RPS2 P15880: 40S ribosomal protein S2		8	8	127	126	6	**1.84**
					129	128	7	**1.77**
					131	130	7	**1.09**

#	protein	group	peptides	spectra	ch1	ch2	quant	ratio
1381	RPS20 P60866$_{1,2}$: 40S ribosomal protein S20	2/2	3	5	127	126	4	**2.13**
					129	128	5	**2.11**
					131	130	5	**1.58**
1382	RPS21 P63220: 40S ribosomal protein S21		2	2	127	126	2	**4.24**
					129	128	2	**1.35**
					131	130	2	**1.12**
1383	RPS23 P62266: 40S ribosomal protein S23		3	3	127	126	3	**2.35**
					129	128	3	**2.00**
					131	130	3	**1.15**
1384	RPS24 P62847$_{1-4}$: 40S ribosomal protein S24	4/4	3	5	127	126	3	**1.82**
					129	128	5	**0.98**
					131	130	4	**0.85**
1385	RPS27A P62979: Ubiquitin-40S ribosomal protein S27a	1/4	11	34	127	126	30	**1.81**
					129	128	31	**1.22**
					131	130	29	**1.28**
1386	RPS28 P62857: 40S ribosomal protein S28		2	3	127	126	3	**1.31**
					129	128	3	**0.47**
					131	130	3	**0.89**
1387	RPS3 P23396$_{1,2}$: 40S ribosomal protein S3	2/2	7	8	127	126	8	**1.54**
					129	128	7	**1.92**
					131	130	8	**1.28**
1388	RPS3A P61247: 40S ribosomal protein S3a		4	4	127	126	4	**1.40**
					129	128	3	**2.08**
					131	130	4	**0.96**
1389	RPS4X P62701: 40S ribosomal protein S4, X isoform	1/3	6	7	127	126	7	**1.37**
					129	128	6	**1.88**
					131	130	6	**1.14**
1390	RPS5 P46782: 40S ribosomal protein S5 [Cleaved into: 40S ribosomal protein S5, N-terminal ...		4	4	127	126	4	**1.79**
					129	128	4	**2.00**
					131	130	4	**1.38**
1391	RPS6 P62753: 40S ribosomal protein S6		6	7	127	126	7	**2.12**
					129	128	7	**1.65**
					131	130	7	**1.15**
1392	RPS6KA3 P51812: Ribosomal protein S6 kinase alpha-3		9	9	127	126	9	**1.13**
					129	128	9	**1.94**
					131	130	8	**1.20**

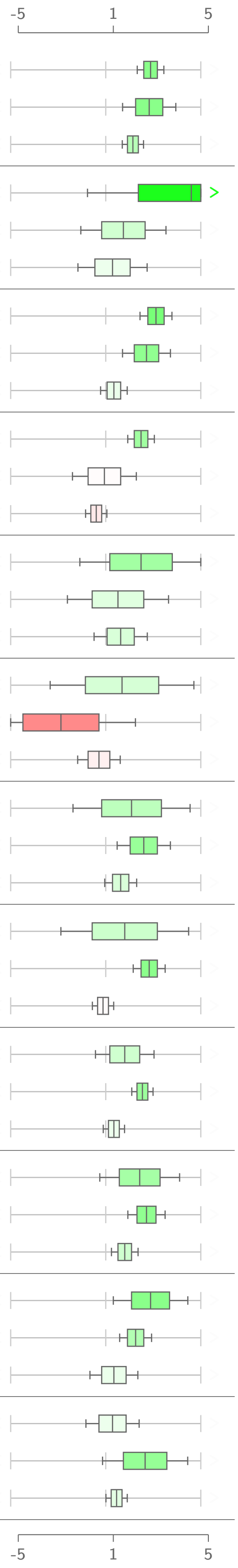

#	protein	group	peptides	spectra	ch1	ch2	quant	ratio
1393	RPS7 P62081: 40S ribosomal protein S7		3	3	127	126	3	**1.80**
					129	128	3	**2.08**
					131	130	3	**0.99**
1394	RPS8 P62241: 40S ribosomal protein S8		2	2	127	126	2	**1.55**
					129	128	2	**1.72**
					131	130	2	**1.49**
1395	RPS9 P46781: 40S ribosomal protein S9		7	9	127	126	7	**1.55**
					129	128	6	**1.42**
					131	130	9	**1.03**
1396	RPSA P08865: 40S ribosomal protein SA		6	10	127	126	10	**1.33**
					129	128	10	**1.68**
					131	130	9	**1.73**
1397	RRBP1 Q9P2E9$_{1,2}$: Ribosome-binding protein 1	2/3	5	7	127	126	7	**0.53**
					129	128	7	**1.44**
					131	130	7	**0.94**
1398	RSU1 Q15404$_1$: Ras suppressor protein 1		1	1	127	126	1	**1.18**
					129	128	1	**1.43**
					131	130	1	**0.63**
1399	RTCB Q9Y3I0: tRNA-splicing ligase RtcB homolog		3	3	127	126	3	**1.78**
					129	128	3	**2.52**
					131	130	3	**1.71**
1400	RTN1 Q16799$_{1-3}$, RTN3 O95197$_{1-7}$: Reticulon-1, Reticulon-3	10/10	1	1	127	126	1	**1.22**
					129	128	1	**1.87**
					131	130	1	**1.59**
1401	S100A10 P60903: Protein S100-A10		5	8	127	126	7	**1.01**
					129	128	7	**1.95**
					131	130	7	**1.13**
1402	S100A11 P31949: Protein S100-A11		5	7	127	126	5	**22.68**
					129	128	6	**1.24**
					131	130	7	**1.08**
1403	S100A14 Q9HCY8: Protein S100-A14		2	3	127	126	3	**3.78**
					129	128	3	**0.73**
					131	130	3	**0.94**
1404	S100A16 Q96FQ6: Protein S100-A16		2	3	127	126	3	**2.39**
					129	128	3	**1.07**
					131	130	3	**1.00**

#	protein	group	peptides	spectra	ch1	ch2	quant	ratio
1405	S100A2 P29034: Protein S100-A2		4	4	127	126	3	**0.77**
					129	128	4	**0.50**
					131	130	4	**1.09**
1406	S100A4 P26447: Protein S100-A4		3	3	127	126	3	**0.73**
					129	128	3	**1.36**
					131	130	3	**0.85**
1407	S100A6 P06703: Protein S100-A6		4	8	127	126	7	**0.83**
					129	128	6	**2.50**
					131	130	8	**0.80**
1408	S100A7 P31151: Protein S100-A7	1/2	7	15	127	126	10	**11.73**
					129	128	15	**0.80**
					131	130	15	**1.06**
1409	S100A8 P05109: Protein S100-A8		10	103	127	126	67	**12.91**
					129	128	94	**1.02**
					131	130	100	**1.21**
1410	S100A9 P06702: Protein S100-A9		7	65	127	126	64	**10.32**
					129	128	59	**0.92**
					131	130	65	**1.11**
1411	SAA1 P0DJI8: Serum amyloid A-1 protein	1/2	10	72	127	126	21	**1.91**
					129	128	21	**0.83**
					131	130	20	**0.84**
1412	SAA2 $P0DJI9_{1}$: Serum amyloid A-2 protein		9	55	127	126	4	**1.49**
					129	128	4	**1.05**
					131	130	4	**0.95**
1413	SAA4 P35542: Serum amyloid A-4 protein		7	162	127	126	156	**1.22**
					129	128	147	**0.93**
					131	130	151	**0.68**
1414	SAMHD1 $Q9Y3Z3_{1,3,4}$: Deoxynucleoside triphosphate triphosphohydrolase SAMHD1	3/4	3	4	127	126	3	**0.91**
					129	128	4	**1.90**
					131	130	4	**1.05**
1415	SARS P49591: Serine--tRNA ligase, cytoplasmic		7	10	127	126	10	**1.54**
					129	128	10	**1.83**
					131	130	9	**1.19**
1416	SART3 $Q15020_{1,4}$: Squamous cell carcinoma antigen recognized by T-cells 3	2/2	2	89	127	126		
					129	128		
					131	130	1	**1.98**

#	protein	group	peptides	spectra	ch1	ch2	quant	ratio
**1417**	SBSN Q6UWP8$_1$: Suprabasin	1/2	12	16	127	126	15	**2.37**
					129	128	15	**0.97**
					131	130	15	**0.95**
**1418**	SCARF2 Q96GP6$_{1,2}$: Scavenger receptor class F member 2	2/2	5	20	127	126	9	**0.60**
					129	128	8	**0.82**
					131	130	12	**0.84**
**1419**	SCG2 P13521: Secretogranin-2		21	31	127	126	28	**0.72**
					129	128	29	**0.76**
					131	130	27	**0.85**
**1420**	SCG3 Q8WXD2$_{1,2}$: Secretogranin-3	2/2	5	7	127	126	7	**0.56**
					129	128	7	**0.73**
					131	130	7	**0.81**
**1421**	SCG5 P05408$_1$: Neuroendocrine protein 7B2		2	3	127	126	2	**0.54**
					129	128	1	**0.67**
					131	130	2	**0.53**
**1422**	SCG5 P05408$_2$: Neuroendocrine protein 7B2		2	3	127	126	2	**0.62**
					129	128	2	**0.58**
					131	130	2	**0.99**
**1423**	SCGB1A1 P11684: Uteroglobin		3	3	127	126	3	**0.62**
					129	128	3	**1.20**
					131	130	3	**0.60**
**1424**	SCRN1 Q12765$_{1-3}$: Secernin-1	3/3	3	4	127	126	4	**0.55**
					129	128	4	**1.28**
					131	130	3	**1.21**
**1425**	SDCBP O00560$_{1-3}$: Syntenin-1	3/3	3	3	127	126	3	**1.15**
					129	128	3	**1.92**
					131	130	3	**1.04**
**1426**	SDF4 Q9BRK5$_1$: 45 kDa calcium-binding protein	1/6	12	24	127	126	22	**0.41**
					129	128	23	**0.93**
					131	130	24	**0.83**
**1427**	SDPR O95810: Serum deprivation-response protein		7	7	127	126	7	**0.68**
					129	128	7	**1.67**
					131	130	6	**2.10**
**1428**	SDR16C5 Q8N3Y7$_{1,2}$: Epidermal retinol dehydrogenase 2	2/2	1	1	127	126		
					129	128	1	**1.65**
					131	130	1	**1.98**

#	protein	group	peptides	spectra	ch1	ch2	quant	ratio
1429	SDR9C7 Q8NEX9: Short-chain dehydrogenase/reductase family 9C member 7		2	2	127	126	2	**3.34**
					129	128	2	**1.04**
					131	130	2	**0.85**
1430	SEC23A Q15436$_1$: Protein transport protein Sec23A	1/3	3	3	127	126	3	**1.12**
					129	128	3	**2.94**
					131	130	3	**1.25**
1431	SEC23IP Q9Y6Y8$_1$: SEC23-interacting protein	1/2	4	4	127	126	2	**1.28**
					129	128	2	**2.68**
					131	130	3	**2.27**
1432	SEC24C P53992$_1$: Protein transport protein Sec24C		4	4	127	126	3	**0.54**
					129	128	3	**2.01**
					131	130	3	**1.25**
1433	SEC31A O94979$_{1-4,6,8-10}$: Protein transport protein Sec31A	8/9	3	3	127	126	3	**0.70**
					129	128	3	**1.74**
					131	130	3	**1.08**
1434	SEL1L Q9UBV2$_{1,2}$: Protein sel-1 homolog 1	2/2	4	8	127	126	4	**0.87**
					129	128	4	**0.73**
					131	130	7	**1.16**
1435	SEMA3B Q13214$_{1,2}$: Semaphorin-3B	2/2	7	9	127	126	8	**0.67**
					129	128	6	**1.10**
					131	130	7	**0.94**
1436	SEMA3C Q99985$_1$: Semaphorin-3C	1/2	8	8	127	126	7	**1.21**
					129	128	7	**1.41**
					131	130	6	**1.01**
1437	SEMA3F Q13275$_{1,2}$: Semaphorin-3F	2/2	6	7	127	126	7	**0.87**
					129	128	6	**1.04**
					131	130	7	**1.00**
1438	SEMA4B Q9NPR2$_{1,2}$: Semaphorin-4B	2/2	3	3	127	126	3	**1.18**
					129	128	3	**1.24**
					131	130	3	**0.77**
1439	SEPP1 P49908: Selenoprotein P		10	24	127	126	24	**0.50**
					129	128	21	**0.81**
					131	130	18	**1.20**
1440	SEPT2 Q15019$_{1,2}$: Septin-2	2/3	6	6	127	126	6	**1.24**
					129	128	6	**2.08**
					131	130	6	**1.39**

#	protein	group	peptides	spectra	ch1	ch2	quant	ratio
1441	SEPT7 Q16181$_{1,2}$: Septin-7	2/2	7	68	127	126	48	**1.13**
					129	128	33	**1.63**
					131	130	54	**1.03**
1442	SEPT9 Q9UHD8$_{1,2,5,7}$: Septin-9	4/4	3	3	127	126	3	**1.67**
					129	128	3	**2.24**
					131	130	3	**1.24**
1443	SERBP1 Q8NC51$_{1-4}$: Plasminogen activator inhibitor 1 RNA-binding protein	4/4	2	2	127	126	2	**1.93**
					129	128	2	**1.90**
					131	130	2	**1.19**
1444	SERPINA1 P01009$_{1}$: Alpha-1-antitrypsin	1/3	26	263	127	126	244	**1.32**
					129	128	248	**1.12**
					131	130	240	**1.02**
1445	SERPINA10 Q9UK55: Protein Z-dependent protease inhibitor		17	52	127	126	49	**1.48**
					129	128	49	**1.06**
					131	130	49	**1.09**
1446	SERPINA11 Q86U17: Serpin A11		3	3	127	126	3	**1.46**
					129	128	3	**1.04**
					131	130	3	**0.81**
1447	SERPINA3 P01011$_{1}$: Alpha-1-antichymotrypsin	1/2	15	36	127	126	31	**1.43**
					129	128	35	**1.37**
					131	130	34	**1.07**
1448	SERPINA4 P29622: Kallistatin		20	51	127	126	50	**1.53**
					129	128	48	**1.19**
					131	130	51	**0.57**
1449	SERPINA5 P05154: Plasma serine protease inhibitor		12	22	127	126	20	**1.05**
					129	128	21	**0.92**
					131	130	21	**0.89**
1450	SERPINA6 P08185: Corticosteroid-binding globulin		5	8	127	126	8	**1.49**
					129	128	7	**0.86**
					131	130	8	**0.78**
1451	SERPINA7 P05543: Thyroxine-binding globulin		11	16	127	126	15	**1.45**
					129	128	16	**1.11**
					131	130	16	**0.76**
1452	SERPINB1 P30740$_{1,2}$: Leukocyte elastase inhibitor	2/2	2	2	127	126	2	**1.86**
					129	128	2	**1.07**
					131	130	2	**1.05**

#	protein	group	peptides	spectra	ch1	ch2	quant	ratio
1453	SERPINB12 Q96P63$_{1,2}$: Serpin B12	2/2	2	2	127	126	2	**1.79**
					129	128	2	**0.68**
					131	130	2	**1.09**
1454	SERPINB2 P05120: Plasminogen activator inhibitor 2		3	3	127	126	3	**4.87**
					129	128	3	**1.49**
					131	130	3	**0.59**
1455	SERPINB3 P29508$_{1}$: Serpin B3	1/2	21	38	127	126	14	**17.43**
					129	128	19	**1.03**
					131	130	18	**0.98**
1456	SERPINB4 P48594: Serpin B4		16	36	127	126	13	**6.75**
					129	128	18	**0.88**
					131	130	16	**0.83**
1457	SERPINB5 P36952$_{1}$: Serpin B5		4	4	127	126	3	**1.56**
					129	128	4	**0.98**
					131	130	3	**1.11**
1458	SERPINC1 P01008: Antithrombin-III		28	175	127	126	174	**1.09**
					129	128	163	**0.86**
					131	130	171	**1.35**
1459	SERPIND1 P05546: Heparin cofactor 2		27	402	127	126	395	**2.60**
					129	128	380	**1.18**
					131	130	382	**0.78**
1460	SERPINE1 P05121$_{1}$: Plasminogen activator inhibitor 1	1/2	9	13	127	126	11	**1.04**
					129	128	11	**1.05**
					131	130	11	**0.98**
1461	SERPINE2 P07093$_{1-3}$: Glia-derived nexin	3/3	4	4	127	126	3	**1.00**
					129	128	4	**1.14**
					131	130	4	**0.82**
1462	SERPINF1 P36955: Pigment epithelium-derived factor		18	60	127	126	57	**0.69**
					129	128	57	**1.16**
					131	130	58	**0.87**
1463	SERPINF2 P08697$_{1}$: Alpha-2-antiplasmin	1/2	18	131	127	126	122	**1.46**
					129	128	115	**1.18**
					131	130	124	**0.76**
1464	SERPING1 P05155$_{1,3}$: Plasma protease C1 inhibitor	2/3	14	29	127	126	29	**2.11**
					129	128	28	**1.56**
					131	130	27	**0.92**

#	protein	group	peptides	spectra	ch1	ch2	quant	ratio
					127	126	6	**1.13**
**1465**	SERPINH1 P50454: Serpin H1		5	6	129	128	6	**1.33**
					131	130	6	**1.09**
					127	126	3	**2.96**
**1466**	SET Q01105$_{1-4}$: Protein SET	4/5	3	3	129	128	3	**1.70**
					131	130	3	**2.02**
					127	126	28	**2.30**
**1467**	SFN P31947$_{1}$: 14-3-3 protein sigma	1/2	17	50	129	128	31	**0.85**
					131	130	32	**0.98**
					127	126	15	**1.31**
**1468**	SFPQ P23246$_{1}$: Splicing factor, proline- and glutamine-rich	1/2	12	18	129	128	15	**2.37**
					131	130	15	**1.63**
					127	126	5	**0.87**
**1469**	SFRP1 Q8N474: Secreted frizzled-related protein 1		5	5	129	128	5	**0.93**
					131	130	4	**0.67**
					127	126	41	**0.72**
**1470**	SFRP2 Q96HF1: Secreted frizzled-related protein 2		12	44	129	128	37	**0.93**
					131	130	42	**1.18**
					127	126	4	**0.42**
**1471**	SFRP4 Q6FHJ7: Secreted frizzled-related protein 4		4	5	129	128	4	**0.91**
					131	130	4	**1.01**
					127	126	2	**0.86**
**1472**	SGTA O43765: Small glutamine-rich tetratricopeptide repeat-containing protein alpha		2	2	129	128	2	**1.78**
					131	130	2	**1.36**
					127	126	3	**3.22**
**1473**	SH3BGRL3 Q9H299: SH3 domain-binding glutamic acid-rich-like protein 3		3	3	129	128	3	**0.90**
					131	130	3	**0.92**
					127	126	2	**1.17**
**1474**	SH3GL1 Q99961$_{1-3}$: Endophilin-A2	3/3	2	2	129	128	2	**2.00**
					131	130	2	**1.12**
					127	126	3	**0.67**
**1475**	SH3GLB1 Q9Y371$_{1,3}$: Endophilin-B1	2/2	3	3	129	128	3	**1.56**
					131	130	3	**1.60**
					127	126	87	**1.34**
**1476**	SHBG P04278$_{1}$: Sex hormone-binding globulin	1/5	15	91	129	128	84	**1.41**
					131	130	87	**0.66**

#	protein	group	peptides	spectra	ch1	ch2	quant	ratio
**1477**	SIL1 Q9H173: Nucleotide exchange factor SIL1		5	5	127	126	4	**0.98**
					129	128	5	**1.15**
					131	130	5	**1.05**
**1478**	SKIV2L Q15477: Helicase SKI2W		2	2	127	126	1	**2.01**
					129	128	1	**0.86**
					131	130	1	**0.97**
**1479**	SKIV2L2 P42285: Superkiller viralicidic activity 2-like 2		3	3	127	126	2	**0.62**
					129	128	2	**0.51**
					131	130	2	**0.62**
**1480**	SKP1 P63208$_{1,2}$: S-phase kinase-associated protein 1	2/2	4	6	127	126	4	**0.74**
					129	128	4	**1.52**
					131	130	3	**1.40**
**1481**	SLC25A3 Q00325$_{1,2}$: Phosphate carrier protein, mitochondrial	2/2	2	2	127	126	2	**5.28**
					129	128	2	**1.31**
					131	130	2	**0.62**
**1482**	SLC25A5 P05141: ADP/ATP translocase 2	1/4	6	7	127	126	7	**1.71**
					129	128	7	**1.28**
					131	130	6	**1.06**
**1483**	SLC9A3R1 O14745$_{1}$: Na(+)/H(+) exchange regulatory cofactor NHE-RF1	1/2	6	7	127	126	5	**1.36**
					129	128	3	**2.27**
					131	130	5	**1.55**
**1484**	SLF1 Q9BQI6$_{2}$: SMC5-SMC6 complex localization factor protein 1		2	3	127	126	2	**1.83**
					129	128	2	**1.52**
					131	130	2	**0.94**
**1485**	SLIT3 O75094$_{1,2,4}$: Slit homolog 3 protein	3/3	7	13	127	126	13	**0.62**
					129	128	12	**0.85**
					131	130	11	**0.85**
**1486**	SLK Q9H2G2$_{1,2}$: STE20-like serine/threonine-protein kinase	2/2	10	17	127	126	10	**1.28**
					129	128	9	**1.95**
					131	130	10	**1.14**
**1487**	SLPI P03973: Antileukoproteinase		15	85	127	126	68	**0.55**
					129	128	73	**0.52**
					131	130	68	**0.78**
**1488**	SLURP1 P55000: Secreted Ly-6/uPAR-related protein 1		2	3	127	126	3	**0.99**
					129	128	3	**1.14**
					131	130	3	**0.73**

#	protein	group	peptides	spectra	ch1	ch2	quant	ratio
**1490**	SNCA P37840$_{1,2}$: Alpha-synuclein	2/2	1	1	127	126	1	**0.81**
					129	128	1	**5.95**
					131	130	1	**1.18**
**1491**	SND1 Q7KZF4: Staphylococcal nuclease domain-containing protein 1		11	13	127	126	13	**1.31**
					129	128	13	**1.96**
					131	130	12	**1.64**
**1492**	SNED1 Q8TER0$_{1,3-5}$: Sushi, nidogen and EGF-like domain-containing protein 1	4/4	8	13	127	126	12	**0.84**
					129	128	9	**1.15**
					131	130	9	**0.77**
**1493**	SNRNP70 P08621$_{1-3}$: U1 small nuclear ribonucleoprotein 70 kDa	3/3	3	3	127	126	3	**1.33**
					129	128	3	**2.50**
					131	130	3	**1.47**
**1494**	SNRPD2 P62316$_{1}$: Small nuclear ribonucleoprotein Sm D2	1/2	3	3	127	126	3	**1.46**
					129	128	3	**1.66**
					131	130	3	**0.94**
**1495**	SNRPD3 P62318$_{1,2}$: Small nuclear ribonucleoprotein Sm D3	2/2	1	1	127	126	1	**1.09**
					129	128	1	**1.12**
					131	130	1	**1.27**
**1496**	SNX1 Q13596$_{1-3}$: Sorting nexin-1	3/3	4	4	127	126	3	**0.75**
					129	128	3	**1.37**
					131	130	4	**1.09**
**1497**	SNX2 O60749$_{1,2}$: Sorting nexin-2	2/2	2	2	127	126	2	**1.23**
					129	128	2	**2.21**
					131	130	2	**1.14**
**1498**	SNX3 O60493$_{1,2,4}$: Sorting nexin-3	3/3	2	3	127	126	3	**1.43**
					129	128	2	**3.30**
					131	130	3	**1.94**
**1499**	SNX5 Q9Y5X3$_{1}$: Sorting nexin-5		3	3	127	126	2	**1.17**
					129	128	2	**2.43**
					131	130	2	**1.41**
**1500**	SNX6 Q9UNH7$_{1,2}$: Sorting nexin-6	2/2	6	7	127	126	6	**0.81**
					129	128	7	**1.42**
					131	130	7	**1.10**
**1501**	SOD3 P08294: Extracellular superoxide dismutase [Cu-Zn]		8	55	127	126	55	**0.58**
					129	128	55	**0.89**
					131	130	55	**1.13**

#	protein	group	peptides	spectra	ch1	ch2	quant	ratio
**1502**	SP100 P23497$_1$: Nuclear autoantigen Sp-100		4	8	127	126	3	**0.59**
					129	128	4	**0.89**
					131	130	5	**1.24**
**1503**	SPARC P09486: SPARC		13	45	127	126	44	**0.48**
					129	128	42	**0.94**
					131	130	40	**0.96**
**1504**	SPARCL1 Q14515$_1$: SPARC-like protein 1	1/2	33	91	127	126	87	**0.47**
					129	128	87	**0.89**
					131	130	88	**0.91**
**1505**	SPECC1 Q5M775$_{1-5}$: Cytospin-B	5/5	2	2	127	126	1	**1.20**
					129	128	1	**2.00**
					131	130	1	**0.91**
**1506**	SPOCK2 Q92563$_1$: Testican-2		1	1	127	126	1	**0.56**
					129	128	1	**0.56**
					131	130	1	**0.70**
**1507**	SPON1 Q9HCB6: Spondin-1		21	35	127	126	33	**0.49**
					129	128	29	**0.97**
					131	130	33	**0.97**
**1508**	SPP1 P10451$_{1-5}$: Osteopontin	5/5	5	7	127	126	6	**0.37**
					129	128	7	**0.84**
					131	130	7	**1.26**
**1509**	SPP2 Q13103: Secreted phosphoprotein 24		8	12	127	126	12	**0.77**
					129	128	12	**0.83**
					131	130	12	**0.85**
**1510**	SPR P35270: Sepiapterin reductase		3	3	127	126	3	**0.97**
					129	128	3	**1.52**
					131	130	3	**1.30**
**1511**	SPRR1A P35321, SPRR1B P22528: Cornifin-A, Cornifin-B	2/2	5	9	127	126	7	**7.64**
					129	128	9	**1.62**
					131	130	9	**1.07**
**1512**	SPRR2D P22532: Small proline-rich protein 2D	1/2	5	11	127	126	3	**1.89**
					129	128	3	**1.07**
					131	130	4	**1.28**
**1513**	SPRR2E P22531: Small proline-rich protein 2E	1/4	4	8	127	126	1	**1.71**
					129	128	1	**0.96**
					131	130	1	**1.23**

#	protein	group	peptides	spectra	ch1	ch2	quant	ratio
**1514**	SPTAN1 Q13813$_{1-3}$: Spectrin alpha chain, non-erythrocytic 1	3/3	17	49	127	126	32	**1.49**
					129	128	32	**1.77**
					131	130	42	**1.49**
**1515**	SPTBN1 Q01082$_{1-3}$: Spectrin beta chain, non-erythrocytic 1	3/3	17	29	127	126	25	**0.95**
					129	128	23	**2.40**
					131	130	24	**1.49**
**1516**	SPTBN2 O15020$_{1}$: Spectrin beta chain, non-erythrocytic 2	1/2	6	15	127	126	12	**0.89**
					129	128	11	**1.04**
					131	130	12	**1.21**
**1517**	SRI P30626$_{1-3}$: Sorcin	3/3	5	6	127	126	5	**0.56**
					129	128	6	**1.47**
					131	130	6	**1.07**
**1518**	SRPX P78539$_{1}$: Sushi repeat-containing protein SRPX	1/4	18	36	127	126	7	**1.01**
					129	128	6	**0.90**
					131	130	7	**0.86**
**1519**	SRPX P78539$_{3}$: Sushi repeat-containing protein SRPX		15	29	127	126	1	**0.73**
					129	128	1	**0.56**
					131	130	1	**1.06**
**1520**	SRRM1 Q8IYB3$_{1,2}$: Serine/arginine repetitive matrix protein 1	2/2	2	3	127	126	2	**0.72**
					129	128	2	**0.86**
					131	130	2	**1.08**
**1521**	SRSF1 Q07955$_{1}$: Serine/arginine-rich splicing factor 1	1/3	11	13	127	126	13	**1.30**
					129	128	13	**2.26**
					131	130	13	**1.81**
**1522**	SRSF10 O75494$_{1-6}$: Serine/arginine-rich splicing factor 10	6/6	1	1	127	126	1	**1.26**
					129	128	1	**3.20**
					131	130	1	**2.06**
**1523**	SRSF3 P84103$_{1}$: Serine/arginine-rich splicing factor 3	1/2	5	37	127	126	32	**2.77**
					129	128	32	**0.76**
					131	130	29	**1.70**
**1524**	SRSF6 Q13247$_{1,3}$: Serine/arginine-rich splicing factor 6	2/2	2	2	127	126	2	**1.34**
					129	128	2	**2.10**
					131	130	2	**1.31**
**1525**	SRSF7 Q16629$_{1-4}$: Serine/arginine-rich splicing factor 7	4/4	6	9	127	126	6	**1.09**
					129	128	6	**1.80**
					131	130	6	**1.46**

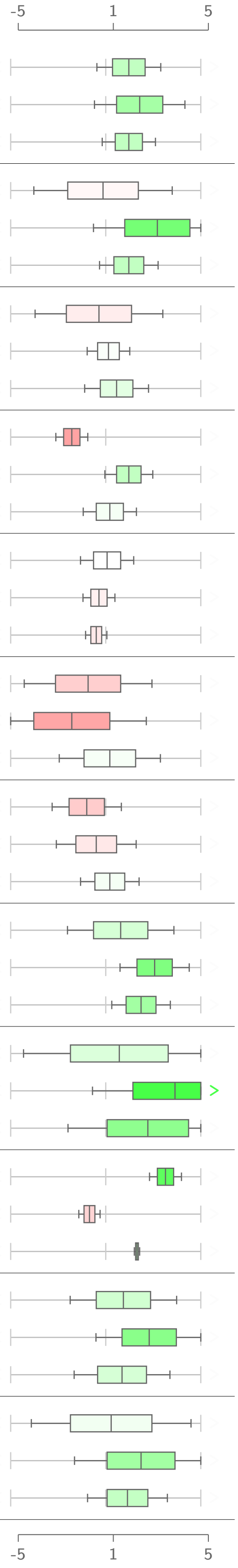

#	protein	group	peptides	spectra	ch1	ch2	quant	ratio
**1526**	SSC5D A1L4H1$_{1,2}$: Soluble scavenger receptor cysteine-rich domain-containing protein SSC5D	2/2	3	3	127	126	3	**0.57**
					129	128	2	**0.85**
					131	130	3	**0.82**
**1527**	ST13 P50502: Hsc70-interacting protein	1/3	12	19	127	126	17	**0.60**
					129	128	19	**1.41**
					131	130	17	**1.09**
**1528**	ST3GAL1 Q11201: CMP-N-acetylneuraminate-beta-galactosamide-alpha-2,3-sialyltransferase 1		5	6	127	126	6	**1.35**
					129	128	5	**1.11**
					131	130	5	**1.22**
**1529**	ST6GAL1 P15907$_1$: Beta-galactoside alpha-2,6-sialyltransferase 1		3	3	127	126	3	**0.80**
					129	128	3	**1.19**
					131	130	3	**1.02**
**1530**	STAB1 Q9NY15$_1$: Stabilin-1	1/2	19	27	127	126	22	**0.89**
					129	128	18	**1.07**
					131	130	24	**0.81**
**1531**	STAT3 P40763$_{1-3}$: Signal transducer and activator of transcription 3	3/3	5	5	127	126	4	**1.60**
					129	128	5	**2.11**
					131	130	4	**1.02**
**1532**	STAT5A P42229$_{1,2}$, STAT5B P51692: Signal transducer and activator of transcription 5A, Signal transducer and activator of transcription 5B	3/3	2	2	127	126	2	**1.33**
					129	128	2	**1.93**
					131	130	2	**0.99**
**1533**	STC2 O76061: Stanniocalcin-2		1	1	127	126	1	**0.93**
					129	128	1	**1.21**
					131	130	1	**0.97**
**1534**	STIP1 P31948$_{1-3}$: Stress-induced-phosphoprotein 1	3/3	2	2	127	126	2	**1.56**
					129	128	2	**2.69**
					131	130	2	**1.83**
**1535**	STK24 Q9Y6E0$_{1,2}$: Serine/threonine-protein kinase 24	2/2	2	2	127	126	2	**1.34**
					129	128	2	**1.98**
					131	130	2	**1.43**
**1536**	STRAP Q9Y3F4$_{1,2}$: Serine-threonine kinase receptor-associated protein	2/2	3	3	127	126	3	**0.96**
					129	128	3	**1.42**
					131	130	3	**1.21**
**1537**	SUB1 P53999: Activated RNA polymerase II transcriptional coactivator p15		6	8	127	126	7	**3.22**
					129	128	7	**2.17**
					131	130	6	**1.63**

#	protein	group	peptides	spectra	ch1	ch2	quant	ratio
**1538**	SUGT1 Q9Y2Z0$_{1,2}$: Protein SGT1 homolog	2/2	2	3	127	126	3	**0.95**
					129	128	3	**1.42**
					131	130	3	**1.35**
**1539**	SULF1 Q8IWU6: Extracellular sulfatase Sulf-1		6	6	127	126	6	**0.41**
					129	128	6	**0.67**
					131	130	5	**0.81**
**1540**	SULF2 Q8IWU5$_{1,2}$: Extracellular sulfatase Sulf-2	2/2	4	5	127	126	5	**0.60**
					129	128	4	**0.90**
					131	130	4	**0.92**
**1541**	SULT1A1 P50225$_{1}$: Sulfotransferase 1A1	1/3	7	7	127	126	3	**1.01**
					129	128	3	**1.43**
					131	130	3	**0.98**
**1542**	SULT1A3 P0DMM9$_{1,3}$, SULT1A4 P0DMN0: Sulfotransferase 1A3, Sulfotransferase 1A4	3/4	6	6	127	126	2	**0.96**
					129	128	2	**1.93**
					131	130	2	**1.02**
**1543**	SUSD5 O60279: Sushi domain-containing protein 5		7	9	127	126	9	**0.59**
					129	128	8	**0.80**
					131	130	8	**0.96**
**1544**	SVEP1 Q4LDE5$_{1}$: Sushi, von Willebrand factor type A, EGF and pentraxin domain-containing pro ...	1/4	65	96	127	126	90	**0.76**
					129	128	85	**1.18**
					131	130	90	**0.70**
**1545**	SWAP70 Q9UH65: Switch-associated protein 70		2	2	127	126	2	**1.07**
					129	128	2	**1.47**
					131	130	2	**0.93**
**1546**	SYNCRIP O60506$_{1}$: Heterogeneous nuclear ribonucleoprotein Q	1/5	13	13	127	126	6	**1.27**
					129	128	6	**2.08**
					131	130	6	**1.63**
**1547**	SYNE1 Q8NF91$_{1,4}$: Nesprin-1	2/2	4	9	127	126	8	**0.10**
					129	128	6	**0.71**
					131	130	8	**1.62**
**1548**	TAC3 Q9UHF0$_{1-3}$: Tachykinin-3	3/3	2	2	127	126	2	**0.72**
					129	128	2	**0.83**
					131	130	2	**0.88**
**1549**	TAF15 Q92804$_{1,2}$: TATA-binding protein-associated factor 2N	2/2	3	3	127	126	3	**1.13**
					129	128	3	**2.75**
					131	130	3	**1.42**

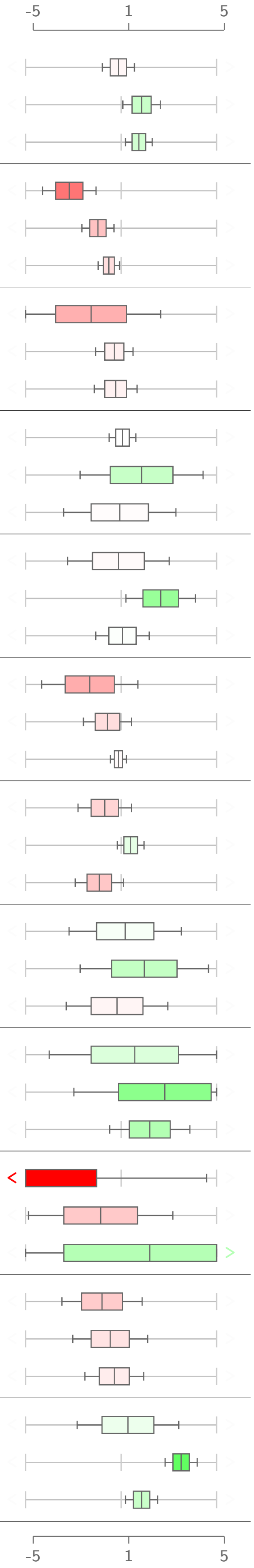

#	protein	group	peptides	spectra	ch1	ch2	quant	ratio
**1550**	TAGLN Q01995: Transgelin		9	13	127	126	11	**1.51**
					129	128	8	**2.15**
					131	130	8	**1.26**
**1551**	TAGLN2 P37802$_{1,2}$: Transgelin-2	2/2	11	20	127	126	17	**0.84**
					129	128	18	**1.59**
					131	130	18	**1.01**
**1553**	TALDO1 P37837: Transaldolase		6	6	127	126	6	**5.18**
					129	128	6	**1.12**
					131	130	6	**1.11**
**1554**	TARDBP Q13148$_{1}$: TAR DNA-binding protein 43	1/2	5	6	127	126	6	**1.51**
					129	128	3	**2.12**
					131	130	6	**1.73**
**1555**	TBCB Q99426$_{1,2}$: Tubulin-folding cofactor B	2/2	2	2	127	126	2	**1.20**
					129	128	2	**1.95**
					131	130	2	**1.03**
**1556**	TCEB2 Q15370$_{1,2}$: Transcription elongation factor B polypeptide 2	2/2	2	3	127	126	3	**1.30**
					129	128	3	**1.23**
					131	130	3	**1.16**
**1557**	TCHH Q07283: Trichohyalin		4	4	127	126	3	**0.48**
					129	128	3	**0.59**
					131	130	3	**1.10**
**1558**	TCN2 P20062$_{1}$: Transcobalamin-2	1/2	10	12	127	126	10	**2.22**
					129	128	9	**1.36**
					131	130	11	**0.87**
**1559**	TCP1 P17987: T-complex protein 1 subunit alpha		6	8	127	126	8	**1.15**
					129	128	8	**1.72**
					131	130	7	**1.19**
**1560**	TF P02787: Serotransferrin		38	75	127	126	75	**1.24**
					129	128	70	**0.90**
					131	130	69	**1.01**
**1561**	TFG Q92734$_{1-4}$: Protein TFG	4/4	3	5	127	126	4	**1.56**
					129	128	5	**2.67**
					131	130	3	**1.65**
**1562**	TFPI P10646$_{1}$: Tissue factor pathway inhibitor		1	1	127	126	1	**0.69**
					129	128	1	**0.81**
					131	130	1	**0.80**

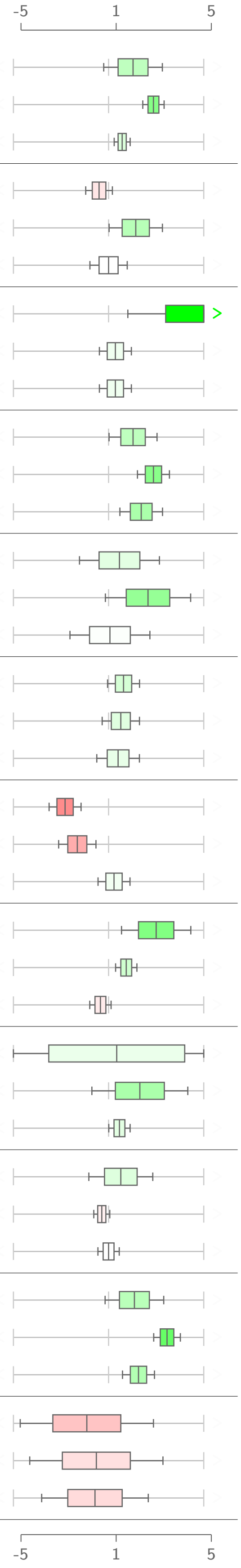

#	protein	group	peptides	spectra	ch1	ch2	quant	ratio
**1563**	TFPI2 P48307$_{1,2}$: Tissue factor pathway inhibitor 2	2/2	4	6	127	126	6	**0.75**
					129	128	5	**0.76**
					131	130	4	**0.88**
**1564**	TGFB1 P01137: Transforming growth factor beta-1		2	2	127	126	2	**0.35**
					129	128	2	**1.00**
					131	130	2	**0.91**
**1565**	TGFBI Q15582: Transforming growth factor-beta-induced protein ig-h3		25	47	127	126	44	**1.08**
					129	128	45	**1.36**
					131	130	44	**0.67**
**1566**	TGFBR3 Q03167$_{1,2}$: Transforming growth factor beta receptor type 3	2/2	14	20	127	126	18	**1.15**
					129	128	18	**1.18**
					131	130	19	**0.71**
**1567**	TGM1 P22735$_{1}$: Protein-glutamine gamma-glutamyltransferase K	1/2	11	12	127	126	11	**1.46**
					129	128	11	**1.21**
					131	130	10	**1.14**
**1568**	TGM2 P21980$_{1}$: Protein-glutamine gamma-glutamyltransferase 2	1/2	9	13	127	126	8	**0.68**
					129	128	9	**1.69**
					131	130	9	**1.09**
**1569**	TGM3 Q08188: Protein-glutamine gamma-glutamyltransferase E		11	22	127	126	19	**6.20**
					129	128	22	**1.01**
					131	130	21	**0.96**
**1570**	TGM4 P49221: Protein-glutamine gamma-glutamyltransferase 4		5	11	127	126	5	**0.77**
					129	128	5	**1.12**
					131	130	7	**1.10**
**1571**	THBS1 P07996$_{1}$: Thrombospondin-1	1/2	34	59	127	126	52	**1.02**
					129	128	51	**1.13**
					131	130	51	**0.76**
**1572**	THBS2 P35442: Thrombospondin-2		28	42	127	126	35	**0.52**
					129	128	34	**0.92**
					131	130	35	**0.83**
**1573**	THBS3 P49746$_{1}$: Thrombospondin-3	1/2	12	14	127	126	11	**1.37**
					129	128	11	**1.23**
					131	130	12	**0.68**
**1574**	THBS4 P35443: Thrombospondin-4		26	74	127	126	62	**1.56**
					129	128	58	**1.16**
					131	130	64	**0.72**

#	protein	group	peptides	spectra	ch1	ch2	quant	ratio
1575	THSD4 Q6ZMP0$_1$: Thrombospondin type-1 domain-containing protein 4	1/2	7	16	127	126	16	**0.58**
					129	128	12	**0.91**
					131	130	16	**0.94**
1576	TIE1 P35590$_1$: Tyrosine-protein kinase receptor Tie-1	1/3	10	12	127	126	11	**1.30**
					129	128	12	**1.20**
					131	130	12	**0.62**
1577	TIMP1 P01033: Metalloproteinase inhibitor 1		5	9	127	126	9	**0.75**
					129	128	7	**1.23**
					131	130	7	**0.99**
1578	TIMP2 P16035: Metalloproteinase inhibitor 2		9	33	127	126	23	**0.50**
					129	128	25	**1.16**
					131	130	27	**0.92**
1579	TIMP3 P35625: Metalloproteinase inhibitor 3		3	3	127	126	3	**0.84**
					129	128	3	**1.11**
					131	130	3	**1.10**
1580	TIMP4 Q99727: Metalloproteinase inhibitor 4		3	3	127	126	3	**0.50**
					129	128	3	**0.93**
					131	130	3	**0.91**
1581	TINAGL1 Q9GZM7$_1$: Tubulointerstitial nephritis antigen-like	1/2	4	13	127	126	6	**0.61**
					129	128	6	**0.87**
					131	130	7	**0.95**
1582	TIPRL O75663$_1$: TIP41-like protein		2	2	127	126	2	**1.43**
					129	128	2	**2.04**
					131	130	2	**0.75**
1583	TJP1 Q07157$_{1,2}$: Tight junction protein ZO-1	2/2	3	4	127	126	4	**1.00**
					129	128	4	**1.27**
					131	130	4	**1.28**
1584	TJP2 Q9UDY2$_{1-7}$: Tight junction protein ZO-2	7/7	3	3	127	126	3	**1.84**
					129	128	3	**2.14**
					131	130	3	**1.30**
1585	TKFC Q3LXA3$_1$: Triokinase/FMN cyclase	1/2	3	5	127	126	4	**1.06**
					129	128	5	**0.89**
					131	130	5	**0.93**
1586	TKT P29401$_{1,2}$: Transketolase	2/2	4	4	127	126	4	**3.69**
					129	128	4	**0.89**
					131	130	4	**1.09**

#	protein	group	peptides	spectra	ch1	ch2	quant	ratio
1587	TLL1 O43897$_1$: Tolloid-like protein 1	1/2	7	7	127	126	3	**0.80**
					129	128	3	**1.17**
					131	130	4	**1.82**
1589	TLN1 Q9Y490: Talin-1		61	78	127	126	63	**1.21**
					129	128	62	**2.28**
					131	130	64	**1.19**
1590	TLN2 Q9Y4G6: Talin-2		14	17	127	126	3	**0.87**
					129	128	3	**0.83**
					131	130	3	**0.90**
1591	TMOD3 Q9NYL9: Tropomodulin-3		2	2	127	126	2	**0.87**
					129	128	2	**1.64**
					131	130	2	**0.93**
1592	TNC P24821$_{1,4}$: Tenascin	2/6	36	53	127	126	47	**0.66**
					129	128	46	**0.76**
					131	130	49	**0.86**
1593	TNFAIP6 P98066: Tumor necrosis factor-inducible gene 6 protein		5	5	127	126	5	**0.65**
					129	128	5	**0.86**
					131	130	5	**0.83**
1594	TNFAIP8 O95379$_{1-4}$: Tumor necrosis factor alpha-induced protein 8	4/4	2	2	127	126	2	**1.19**
					129	128	2	**2.17**
					131	130	2	**1.03**
1595	TNKS1BP1 Q9C0C2$_{1,2}$: 182 kDa tankyrase-1-binding protein	2/2	3	4	127	126	4	**1.00**
					129	128	3	**1.84**
					131	130	4	**1.46**
1596	TNNT1 P13805$_{1-3}$: Troponin T, slow skeletal muscle	3/3	5	19	127	126	16	**0.55**
					129	128	17	**1.10**
					131	130	15	**1.01**
1597	TNPO1 Q92973$_{1-3}$: Transportin-1	3/3	6	6	127	126	5	**0.95**
					129	128	5	**0.78**
					131	130	5	**0.80**
1598	TNPO2 O14787$_2$: Transportin-2	1/2	3	4	127	126	3	**1.81**
					129	128	3	**3.16**
					131	130	3	**1.24**
1599	TNXB P22105$_{1,3}$: Tenascin-X	2/5	90	125	127	126	117	**0.71**
					129	128	114	**0.94**
					131	130	113	**0.87**

#	protein	group	peptides	spectra	ch1	ch2	quant	ratio
**1600**	TOR1A O14656$_{1}$: Torsin-1A		1	1	127	126	1	**0.54**
					129	128	1	**1.25**
					131	130	1	**0.84**
**1601**	TPD52L2 O43399$_{1-7}$: Tumor protein D54	7/7	2	2	127	126	2	**0.85**
					129	128	2	**1.28**
					131	130	2	**0.70**
**1602**	TPI1 P60174$_{1,3}$: Triosephosphate isomerase	2/3	5	5	127	126	5	**4.93**
					129	128	4	**1.03**
					131	130	5	**1.01**
**1610**	TPM2 P07951$_{1}$: Tropomyosin beta chain		21	45	127	126	7	**0.40**
					129	128	5	**0.90**
					131	130	7	**1.68**
**1611**	TPM2 P07951$_{2}$: Tropomyosin beta chain	1/2	21	45	127	126	7	**0.48**
					129	128	7	**1.67**
					131	130	8	**0.89**
**1612**	TPM3 P06753$_{1}$: Tropomyosin alpha-3 chain		18	39	127	126	1	**0.35**
					129	128	1	**1.54**
					131	130	1	**0.95**
**1613**	TPM3 P06753$_{2}$: Tropomyosin alpha-3 chain	1/3	21	38	127	126	3	**1.52**
					129	128	3	**1.31**
					131	130	3	**1.15**
**1615**	TPM4 P67936$_{1}$: Tropomyosin alpha-4 chain		19	40	127	126	5	**0.72**
					129	128	5	**1.33**
					131	130	6	**1.18**
**1617**	TPP1 O14773$_{1,2}$: Tripeptidyl-peptidase 1	2/2	4	5	127	126	4	**0.84**
					129	128	5	**1.03**
					131	130	3	**1.17**
**1618**	TPSAB1 Q15661$_{1}$, TPSB2 P20231: Tryptase alpha/beta-1, Tryptase beta-2	2/2	2	2	127	126	2	**0.67**
					129	128	2	**1.15**
					131	130	2	**0.94**
**1619**	TPT1 P13693$_{1}$: Translationally-controlled tumor protein	1/2	3	3	127	126	3	**1.24**
					129	128	3	**1.09**
					131	130	3	**1.03**
**1620**	TRIM29 Q14134$_{1,2}$: Tripartite motif-containing protein 29	2/2	13	14	127	126	11	**2.72**
					129	128	11	**0.73**
					131	130	11	**1.03**

#	protein	group	peptides	spectra	ch1	ch2	quant	ratio
1621	TRIP6 Q15654$_1$: Thyroid receptor-interacting protein 6		3	3	127	126	2	**0.78**
					129	128	2	**2.20**
					131	130	2	**1.55**
1622	TSKU Q8WUA8: Tsukushin		10	23	127	126	23	**1.14**
					129	128	22	**1.15**
					131	130	21	**1.10**
1623	TSTA3 Q13630: GDP-L-fucose synthase		3	10	127	126	8	**1.41**
					129	128	10	**1.67**
					131	130	10	**1.41**
1624	TTLL12 Q14166: Tubulin--tyrosine ligase-like protein 12		2	2	127	126	2	**1.93**
					129	128	2	**1.57**
					131	130	2	**1.11**
1625	TTN Q8WZ42$_{12}$: Titin	1/12	37	57	127	126	36	**0.89**
					129	128	29	**0.90**
					131	130	38	**0.89**
1626	TTN Q8WZ42$_6$: Titin		6	6	127	126	2	**1.18**
					129	128	2	**1.01**
					131	130	2	**1.03**
1627	TTR P02766: Transthyretin		11	166	127	126	149	**0.79**
					129	128	153	**0.89**
					131	130	162	**1.14**
1631	TUBA4A P68366$_{1,2}$: Tubulin alpha-4A chain	2/2	16	52	127	126	2	**3.70**
					129	128	2	**1.50**
					131	130	2	**1.33**
1632	TUBA8 Q9NY65$_1$: Tubulin alpha-8 chain	1/2	12	30	127	126	2	**1.60**
					129	128	2	**2.42**
					131	130	2	**2.16**
1633	TUBAL3 A6NHL2$_{1,2}$: Tubulin alpha chain-like 3	2/2	4	17	127	126	3	**1.17**
					129	128	3	**1.62**
					131	130	3	**1.25**
1634	TUBB P07437: Tubulin beta chain	1/2	17	66	127	126	6	**1.20**
					129	128	7	**2.04**
					131	130	7	**1.28**
1635	TUBB1 Q9H4B7: Tubulin beta-1 chain		4	13	127	126	1	**0.45**
					129	128	1	**0.90**
					131	130	1	**1.01**

#	protein	group	peptides	spectra	ch1	ch2	quant	ratio
**1636**	TUBB2A Q13885: Tubulin beta-2A chain	1/2	16	61	127	126	3	**0.87**
					129	128	3	**1.28**
					131	130	3	**1.12**
**1639**	TUBB4B P68371: Tubulin beta-4B chain	1/3	17	60	127	126	1	**1.81**
					129	128	1	**2.18**
					131	130	1	**1.38**
**1640**	TUBB6 Q9BUF5: Tubulin beta-6 chain		9	24	127	126	3	**0.98**
					129	128	3	**1.35**
					131	130	3	**1.17**
**1641**	TWF1 Q12792$_{2-4}$: Twinfilin-1	3/3	3	4	127	126	2	**2.71**
					129	128	2	**2.09**
					131	130	2	**1.13**
**1642**	TWF2 Q6IBS0: Twinfilin-2		3	4	127	126	2	**1.22**
					129	128	2	**1.73**
					131	130	2	**0.91**
**1643**	TWSG1 Q9GZX9$_{1}$: Twisted gastrulation protein homolog 1	1/2	5	8	127	126	2	**0.47**
					129	128	3	**1.02**
					131	130	5	**0.76**
**1644**	TXN P10599$_{1,2}$: Thioredoxin	2/2	2	3	127	126	3	**5.62**
					129	128	3	**0.84**
					131	130	3	**0.99**
**1645**	TXNDC5 Q8NBS9$_{1,2}$: Thioredoxin domain-containing protein 5	2/2	5	7	127	126	4	**0.83**
					129	128	4	**1.19**
					131	130	3	**1.11**
**1646**	TXNL1 O43396: Thioredoxin-like protein 1		5	5	127	126	3	**1.52**
					129	128	4	**1.71**
					131	130	5	**1.08**
**1647**	TYMP P19971$_{1}$: Thymidine phosphorylase	1/2	9	10	127	126	9	**2.42**
					129	128	8	**1.74**
					131	130	7	**0.76**
**1648**	UBA1 P22314$_{1,2}$: Ubiquitin-like modifier-activating enzyme 1	2/2	22	28	127	126	23	**1.28**
					129	128	24	**2.08**
					131	130	24	**1.08**
**1649**	UBA5 Q9GZZ9$_{1}$: Ubiquitin-like modifier-activating enzyme 5		3	3	127	126	3	**1.03**
					129	128	3	**1.30**
					131	130	3	**1.06**

-5 1 5

#	protein	group	peptides	spectra	ch1	ch2	quant	ratio
**1650**	UBA6 A0AVT1$_{1,2}$: Ubiquitin-like modifier-activating enzyme 6	2/2	1	1	127	126	1	**1.61**
					129	128	1	**2.53**
					131	130	1	**1.52**
**1651**	UBE2D2 P62837$_{1,2}$, UBE2D3 P61077$_{1-3}$: Ubiquitin-conjugating enzyme E2 D2, Ubiquitin-conjugating enzyme E2 D3	5/5	2	2	127	126	2	**2.05**
					129	128	2	**1.62**
					131	130	2	**0.60**
**1652**	UBE2F Q969M7$_{3}$: NEDD8-conjugating enzyme UBE2F	1/6	2	2	127	126	2	**2.00**
					129	128	2	**1.97**
					131	130	2	**1.56**
**1653**	UBE2K P61086$_{1,3}$: Ubiquitin-conjugating enzyme E2 K	2/2	4	5	127	126	4	**2.24**
					129	128	5	**1.72**
					131	130	4	**1.05**
**1654**	UBE2L3 P68036$_{1}$: Ubiquitin-conjugating enzyme E2 L3		2	89	127	126	1	**3.18**
					129	128	1	**2.20**
					131	130	1	**0.94**
**1655**	UBE2M P61081: NEDD8-conjugating enzyme Ubc12		1	1	127	126	1	**1.97**
					129	128	1	**3.46**
					131	130	1	**1.50**
**1656**	UBE2N P61088, UBE2NL Q5JXB2: Ubiquitin-conjugating enzyme E2 N, Putative ubiquitin-conjugating enzyme E2 N-like	2/2	3	3	127	126	3	**2.62**
					129	128	3	**2.36**
					131	130	3	**1.15**
**1657**	UBE2V2 Q15819: Ubiquitin-conjugating enzyme E2 variant 2	1/6	3	4	127	126	3	**2.20**
					129	128	4	**2.02**
					131	130	4	**0.86**
**1658**	UBR4 Q5T4S7$_{1-4}$: E3 ubiquitin-protein ligase UBR4	4/4	7	10	127	126	7	**0.83**
					129	128	7	**1.33**
					131	130	7	**1.02**
**1659**	UGDH O60701$_{1}$: UDP-glucose 6-dehydrogenase	1/3	13	13	127	126	13	**0.97**
					129	128	13	**1.96**
					131	130	13	**1.42**
**1660**	UGGT1 Q9NYU2$_{1,2}$: UDP-glucose:glycoprotein glucosyltransferase 1	2/2	4	4	127	126	3	**1.17**
					129	128	3	**1.17**
					131	130	4	**0.90**
**1661**	UGP2 Q16851$_{2}$: UTP--glucose-1-phosphate uridylyltransferase	1/2	12	14	127	126	14	**1.57**
					129	128	13	**2.73**
					131	130	13	**0.99**

-5 1 5

#	protein	group	peptides	spectra	ch1	ch2	quant	ratio
**1662**	UNC45A Q9H3U1$_{1,2}$: Protein unc-45 homolog A	2/2	3	3	127	126	3	**1.36**
					129	128	3	**1.64**
					131	130	3	**1.08**
**1663**	UNC80 Q8N2C7$_{1,2,4,7}$: Protein unc-80 homolog	4/4	2	3	127	126	3	**0.62**
					129	128	3	**0.99**
					131	130	3	**0.64**
**1664**	UPF1 Q92900$_{1,2}$: Regulator of nonsense transcripts 1	2/2	2	2	127	126	2	**1.29**
					129	128	2	**2.93**
					131	130	2	**1.73**
**1665**	UQCRH P07919: Cytochrome b-c1 complex subunit 6, mitochondrial		2	2	127	126	2	**0.57**
					129	128	2	**1.17**
					131	130	2	**0.99**
**1667**	USO1 O60763$_{1,2}$: General vesicular transport factor p115	2/2	7	7	127	126	7	**1.44**
					129	128	7	**2.23**
					131	130	6	**1.57**
**1668**	USP5 P45974$_{1,2}$: Ubiquitin carboxyl-terminal hydrolase 5	2/2	5	6	127	126	6	**1.25**
					129	128	6	**1.40**
					131	130	6	**1.14**
**1669**	USP9X Q93008$_{1,3}$: Probable ubiquitin carboxyl-terminal hydrolase FAF-X	2/2	9	45	127	126	7	**0.74**
					129	128	8	**0.88**
					131	130	8	**1.13**
**1670**	USP9Y O00507$_{1,2}$: Probable ubiquitin carboxyl-terminal hydrolase FAF-Y	2/2	9	60	127	126	1	**0.68**
					129	128	1	**0.71**
					131	130	2	**1.14**
**1671**	UTP3 Q9NQZ2: Something about silencing protein 10		2	6	127	126	3	**0.54**
					129	128	2	**0.89**
					131	130	3	**0.93**
**1672**	VAT1 Q99536$_{1}$: Synaptic vesicle membrane protein VAT-1 homolog	1/3	8	9	127	126	8	**1.14**
					129	128	9	**1.18**
					131	130	9	**0.71**
**1673**	VAT1L Q9HCJ6: Synaptic vesicle membrane protein VAT-1 homolog-like		1	1	127	126	1	**0.78**
					129	128	1	**2.46**
					131	130	1	**0.86**
**1674**	VCAM1 P19320$_{1,3}$: Vascular cell adhesion protein 1	2/3	15	18	127	126	16	**0.99**
					129	128	15	**1.11**
					131	130	16	**0.90**

#	protein	group	peptides	spectra	ch1	ch2	quant	ratio
**1675**	VCAN P13611$_{1-5}$: Versican core protein	5/5	3	7	127	126	7	**1.23**
					129	128	7	**0.92**
					131	130	7	**1.13**
**1676**	VCL P18206$_{1,2}$: Vinculin	2/3	43	57	127	126	55	**2.35**
					129	128	51	**2.18**
					131	130	52	**0.81**
**1677**	VCP P55072: Transitional endoplasmic reticulum ATPase		18	23	127	126	21	**1.85**
					129	128	20	**1.87**
					131	130	22	**1.36**
**1678**	VDAC1 P21796: Voltage-dependent anion-selective channel protein 1		2	2	127	126	2	**4.48**
					129	128	2	**1.63**
					131	130	2	**1.15**
**1679**	VEGFC P49767: Vascular endothelial growth factor C		17	33	127	126	32	**0.59**
					129	128	32	**0.83**
					131	130	30	**0.84**
**1680**	VEGFD O43915: Vascular endothelial growth factor D		2	2	127	126	2	**0.42**
					129	128	2	**1.02**
					131	130	2	**0.95**
**1681**	VGF O15240: Neurosecretory protein VGF [Cleaved into: Neuroendocrine regulatory peptide-1		11	24	127	126	14	**0.59**
					129	128	18	**0.85**
					131	130	16	**0.83**
**1682**	VIM P08670: Vimentin		32	99	127	126	56	**0.60**
					129	128	55	**2.29**
					131	130	57	**1.46**
**1683**	VLDLR P98155$_{1,2}$: Very low-density lipoprotein receptor	2/2	9	18	127	126	8	**0.61**
					129	128	7	**0.84**
					131	130	8	**1.10**
**1684**	VPS35 Q96QK1: Vacuolar protein sorting-associated protein 35		10	12	127	126	12	**1.05**
					129	128	12	**1.84**
					131	130	12	**1.18**
**1685**	VPS4A Q9UN37: Vacuolar protein sorting-associated protein 4A	1/4	3	3	127	126	3	**1.72**
					129	128	3	**1.80**
					131	130	3	**1.17**
**1686**	VTN P04004: Vitronectin		17	1197	127	126	1079	**0.72**
					129	128	1097	**0.63**
					131	130	1135	**1.28**

#	protein	group	peptides	spectra	ch1	ch2	quant	ratio
**1687**	VWA1 Q6PCB0$_1$: von Willebrand factor A domain-containing protein 1	1/2	4	5	127	126	4	**0.98**
					129	128	4	**1.49**
					131	130	5	**0.83**
**1688**	VWF P04275$_1$: von Willebrand factor	1/2	67	104	127	126	98	**1.26**
					129	128	97	**1.14**
					131	130	98	**0.69**
**1689**	WARS P23381$_{1,2}$: Tryptophan--tRNA ligase, cytoplasmic	2/2	4	4	127	126	4	**1.28**
					129	128	4	**1.58**
					131	130	4	**0.79**
**1690**	WDR1 O75083$_1$: WD repeat-containing protein 1	1/2	6	6	127	126	4	**1.54**
					129	128	6	**1.36**
					131	130	5	**1.06**
**1691**	WFDC12 Q8WWY7: WAP four-disulfide core domain protein 12		3	3	127	126	3	**6.40**
					129	128	2	**0.82**
					131	130	2	**0.94**
**1692**	WISP2 O76076$_1$: WNT1-inducible-signaling pathway protein 2	1/2	10	34	127	126	28	**0.40**
					129	128	29	**0.64**
					131	130	31	**0.62**
**1693**	XP32 Q5T750: Skin-specific protein 32		1	1	127	126	1	**26.43**
					129	128	1	**0.68**
					131	130	1	**1.24**
**1694**	XPNPEP1 Q9NQW7$_3$: Xaa-Pro aminopeptidase 1	1/2	2	2	127	126	2	**1.39**
					129	128	2	**2.02**
					131	130	2	**1.03**
**1695**	XPO1 O14980: Exportin-1		6	7	127	126	6	**1.11**
					129	128	6	**2.16**
					131	130	5	**0.96**
**1696**	XRCC5 P13010: X-ray repair cross-complementing protein 5		10	12	127	126	12	**1.87**
					129	128	12	**2.52**
					131	130	12	**1.85**
**1697**	XRCC6 P12956$_{1,2}$: X-ray repair cross-complementing protein 6	2/2	18	22	127	126	19	**2.64**
					129	128	20	**3.41**
					131	130	20	**2.36**
**1698**	XXYLT1 Q8NBI6$_1$: Xyloside xylosyltransferase 1	1/2	5	5	127	126	3	**1.26**
					129	128	3	**1.26**
					131	130	3	**0.84**

#	protein	group	peptides	spectra	ch1	ch2	quant	ratio
**1699**	XXYLT1 Q8NBI6$_3$: Xyloside xylosyltransferase 1		3	3	127	126	1	**1.43**
					129	128	1	**2.04**
					131	130	1	**0.80**
**1700**	YBX3 P16989$_{1-3}$: Y-box-binding protein 3	3/3	1	1	127	126	1	**0.91**
					129	128	1	**1.30**
					131	130	1	**0.98**
**1701**	YKT6 O15498$_1$: Synaptobrevin homolog YKT6	1/2	6	8	127	126	7	**1.71**
					129	128	7	**1.71**
					131	130	7	**1.02**
**1702**	YOD1 Q5VVQ6$_{1,2}$: Ubiquitin thioesterase OTU1	2/2	1	1	127	126	1	**2.76**
					129	128	1	**1.25**
					131	130	1	**0.88**
**1703**	YWHAB P31946$_{1,2}$: 14-3-3 protein beta/alpha	2/2	15	44	127	126	12	**0.76**
					129	128	13	**1.29**
					131	130	13	**0.93**
**1704**	YWHAE P62258$_1$: 14-3-3 protein epsilon	1/2	14	38	127	126	22	**0.61**
					129	128	25	**1.59**
					131	130	24	**1.06**
**1705**	YWHAG P61981: 14-3-3 protein gamma		16	45	127	126	21	**0.58**
					129	128	21	**1.23**
					131	130	21	**1.02**
**1706**	YWHAH Q04917: 14-3-3 protein eta		13	36	127	126	18	**0.60**
					129	128	18	**1.60**
					131	130	15	**1.03**
**1707**	YWHAQ P27348: 14-3-3 protein theta		18	51	127	126	25	**0.87**
					129	128	27	**1.67**
					131	130	27	**1.15**
**1708**	YWHAZ P63104$_1$: 14-3-3 protein zeta/delta	1/2	18	58	127	126	34	**1.05**
					129	128	34	**1.47**
					131	130	30	**1.03**
**1709**	ZBTB6 Q15916: Zinc finger and BTB domain-containing protein 6		2	24	127	126	18	**0.73**
					129	128	20	**1.42**
					131	130	20	**1.07**
**1710**	ZNF185 O15231$_{1,3-8}$: Zinc finger protein 185	7/7	2	2	127	126	2	**4.17**
					129	128	2	**1.42**
					131	130	2	**1.98**

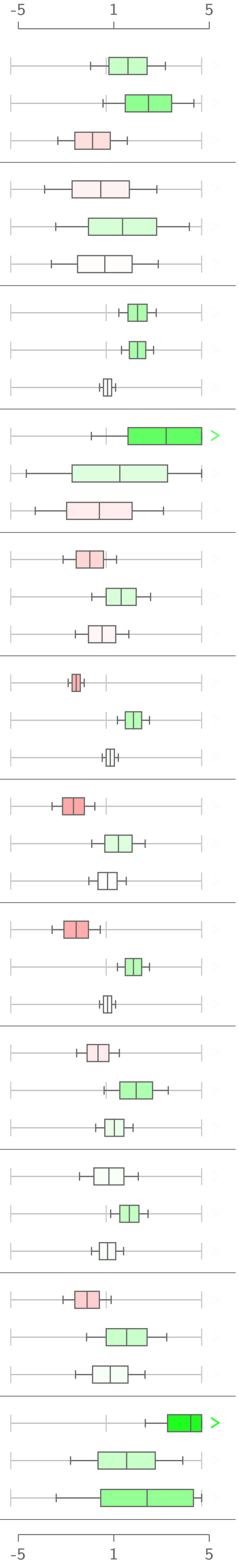

#	protein	group	peptides	spectra	ch1	ch2	quant	ratio
**1711**	ZNF318 Q5VUA4$_1$: Zinc finger protein 318		4	4	127	126	1	**0.56**
					129	128	1	**1.13**
					131	130	1	**1.48**
**1712**	ZNF462 Q96JM2$_{1,3}$: Zinc finger protein 462	2/2	8	22	127	126	14	**1.08**
					129	128	11	**1.09**
					131	130	19	**1.29**
**1713**	ZNF600 Q6ZNG1: Zinc finger protein 600		3	5	127	126	2	**1.76**
					129	128	2	**1.15**
					131	130	3	**0.91**
**1714**	ZNF85 Q03923$_{1,2}$: Zinc finger protein 85	2/2	5	61	127	126	40	**0.52**
					129	128	34	**0.78**
					131	130	49	**1.61**
**1715**	ZYX Q15942$_1$: Zyxin	1/2	3	3	127	126	3	**0.96**
					129	128	3	**1.78**
					131	130	3	**1.07**
**1716**	ZZEF1 O43149$_1$: Zinc finger ZZ-type and EF-hand domain-containing protein 1	1/2	9	49	127	126	19	**0.77**
					129	128	18	**1.14**
					131	130	23	**1.03**

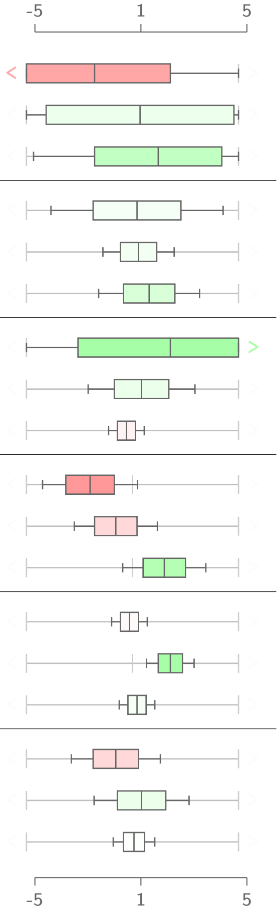

1.2 Not Quantified Proteins

Number of identified but not quantified proteins: P4-treat/P4-ctrl: 91; P6-treat/P6-ctrl: 94; P7-treat/P7-ctrl: 89

#	protein	group	peptides	spectra
656	HIST1H2BC P62807, HIST1H2BD P58876, HIST1H2BH Q93079, . . .: Histone H2B type 1-C/E/F/G/I, Histone H2B type 1-D, . . .	7/9	12	40
532	FCN3 O75636$_2$: Ficolin-3		9	24
884	KRT37 O76014: Keratin, type I cuticular Ha7		4	48
885	KRT38 O76015: Keratin, type I cuticular Ha8		5	55
1666	URI1 O94763$_1$: Unconventional prefoldin RPB5 interactor 1		2	20
897	KRT75 O95678: Keratin, type II cytoskeletal 75		19	97
780	IGHV3-33 P01772: Immunoglobulin heavy variable 3-33		2	5
668	HLA-B P01889: HLA class I histocompatibility antigen, B-7 alpha chain	1/2	8	12
661	HLA-A P01891: HLA class I histocompatibility antigen, A-68 alpha chain		6	6
696	HLA-H P01893: Putative HLA class I histocompatibility antigen, alpha chain H		4	6
687	HLA-C P04222: HLA class I histocompatibility antigen, Cw-3 alpha chain		8	12
1638	TUBB4A P04350: Tubulin beta-4A chain		14	46
663	HLA-A P05534, HLA-A P30447: HLA class I histocompatibility antigen, A-24 alpha chain, HLA class I histocompatibility antigen, A-23 alpha chain	2/2	4	6
1614	TPM3 P06753$_5$: Tropomyosin alpha-3 chain	1/3	22	42
655	HIST1H2BB P33778, HIST1H2BJ P06899, HIST1H2BO P23527, . . .: Histone H2B type 1-B, Histone H2B type 1-J, . . .	4/6	11	37

#	protein	group	peptides	spectra
625	GSTM1 P09488$_1$, GSTM4 Q03013$_1$: Glutathione S-transferase Mu 1, Glutathione S-transferase Mu 4	2/2	3	3
1603	TPM1 P09493$_1$: Tropomyosin alpha-1 chain		21	42
1604	TPM1 P09493$_{4,10}$: Tropomyosin alpha-1 chain	2/2	21	41
1605	TPM1 P09493$_3$: Tropomyosin alpha-1 chain	1/2	21	43
1606	TPM1 P09493$_6$: Tropomyosin alpha-1 chain		20	36
1607	TPM1 P09493$_7$: Tropomyosin alpha-1 chain		20	38
1608	TPM1 P09493$_8$: Tropomyosin alpha-1 chain		20	37
1609	TPM1 P09493$_9$: Tropomyosin alpha-1 chain	1/2	21	44
796	IGLC6 P0CF74: Ig lambda-6 chain C region		4	141
1214	POTEI P0CG38, POTEJ P0CG39: POTE ankyrin domain family member I, POTE ankyrin domain family member J	2/2	10	115
664	HLA-A P10316: HLA class I histocompatibility antigen, A-69 alpha chain	1/2	7	7
876	KRT3 P12035: Keratin, type II cytoskeletal 3		10	63
586	GFAP P14136$_{1-3}$: Glial fibrillary acidic protein	3/3	4	25
665	HLA-A P16188: HLA class I histocompatibility antigen, A-30 alpha chain		6	8
669	HLA-B P18463: HLA class I histocompatibility antigen, B-37 alpha chain		6	9
887	KRT4 P19013: Keratin, type II cytoskeletal 4		8	25
626	GSTM2 P28161$_1$: Glutathione S-transferase Mu 2		3	3
666	HLA-A P30453: HLA class I histocompatibility antigen, A-34 alpha chain	1/9	7	7
670	HLA-B P30460: HLA class I histocompatibility antigen, B-8 alpha chain	1/2	9	13
671	HLA-B P30462: HLA class I histocompatibility antigen, B-14 alpha chain		7	10
672	HLA-B P30464: HLA class I histocompatibility antigen, B-15 alpha chain		8	11
673	HLA-B P30466: HLA class I histocompatibility antigen, B-18 alpha chain		6	9
674	HLA-B P30475: HLA class I histocompatibility antigen, B-39 alpha chain	1/2	8	11
675	HLA-B P30479: HLA class I histocompatibility antigen, B-41 alpha chain	1/2	9	12
676	HLA-B P30480: HLA class I histocompatibility antigen, B-42 alpha chain	1/7	9	13
677	HLA-B P30483, HLA-B P30487, HLA-B P30488: HLA class I histocompatibility antigen, B-45 alpha chain, HLA class I histocompatibility antigen, B-49 alpha chain, . . .	3/3	5	7
678	HLA-B P30484: HLA class I histocompatibility antigen, B-46 alpha chain		8	11
679	HLA-B P30486: HLA class I histocompatibility antigen, B-48 alpha chain		7	8
680	HLA-B P30490: HLA class I histocompatibility antigen, B-52 alpha chain		6	9
681	HLA-B P30491, HLA-B P30685: HLA class I histocompatibility antigen, B-53 alpha chain, HLA class I histocompatibility antigen, B-35 alpha chain	2/3	5	8
682	HLA-B P30492: HLA class I histocompatibility antigen, B-54 alpha chain		6	9
689	HLA-C P30499: HLA class I histocompatibility antigen, Cw-1 alpha chain		7	9
691	HLA-C P30508, HLA-C Q29963: HLA class I histocompatibility antigen, Cw-12 alpha chain, HLA class I histocompatibility antigen, Cw-6 alpha chain	2/4	10	15
692	HLA-C P30510: HLA class I histocompatibility antigen, Cw-14 alpha chain		8	12
667	HLA-A P30512: HLA class I histocompatibility antigen, A-29 alpha chain		6	6
738	HSPA1L P34931: Heat shock 70 kDa protein 1-like		13	23
24	ACTN2 P35609$_2$: Alpha-actinin-2	1/2	8	9
1031	MYH11 P35749$_{1-4}$: Myosin-11	4/5	20	24
870	KRT20 P35900: Keratin, type I cytoskeletal 20		6	47
1251	PRPH P41219$_{1,2}$: Peripherin	2/2	4	9
892	KRT6C P48668: Keratin, type II cytoskeletal 6C		46	175
1222	PPP2CA P67775$_1$: Serine/threonine-protein phosphatase 2A catalytic subunit alpha isoform		4	5
1616	TPM4 P67936$_2$: Tropomyosin alpha-4 chain		17	36
1629	TUBA1B P68363$_1$: Tubulin alpha-1B chain	1/2	18	58

#	protein	group	peptides	spectra
905	KRT83 P78385: Keratin, type II cuticular Hb3	1/2	29	408
898	KRT76 Q01546: Keratin, type II cytoskeletal 2 oral		11	76
438	DST Q03001$_{7,8}$: Dystonin	2/2	2	11
683	HLA-B Q04826: HLA class I histocompatibility antigen, B-40 alpha chain	1/3	8	11
693	HLA-C Q07000: HLA class I histocompatibility antigen, Cw-15 alpha chain	1/3	8	12
1637	TUBB3 Q13509$_{1}$: Tubulin beta-3 chain		10	32
1630	TUBA3C Q13748$_{1}$: Tubulin alpha-3C/D chain	1/2	15	41
895	KRT72 Q14CN4$_{1-3}$: Keratin, type II cytoskeletal 72	3/3	6	25
1205	POSTN Q15063$_{2}$: Periostin		44	226
1206	POSTN Q15063$_{3}$: Periostin		44	260
1207	POSTN Q15063$_{4}$: Periostin		42	219
1208	POSTN Q15063$_{5}$: Periostin		46	267
1209	POSTN Q15063$_{6}$: Periostin		41	213
1210	POSTN Q15063$_{7}$: Periostin		39	206
1308	RAB35 Q15286$_{1,2}$: Ras-related protein Rab-35	2/2	2	7
684	HLA-B Q29836: HLA class I histocompatibility antigen, B-67 alpha chain		7	10
694	HLA-C Q29960$_{1,2}$: HLA class I histocompatibility antigen, Cw-16 alpha chain	2/2	7	10
685	HLA-B Q31610: HLA class I histocompatibility antigen, B-81 alpha chain		7	8
1489	SNAPC4 Q5SXM2: snRNA-activating protein complex subunit 4		2	3
1628	TUBA1A Q71U36$_{1,2}$: Tubulin alpha-1A chain	2/4	18	56
896	KRT73 Q86Y46$_{1}$, KRT74 Q7RTS7: Keratin, type II cytoskeletal 73, Keratin, type II cytoskeletal 74	2/2	8	28
660	HIST3H2BB Q8N257: Histone H2B type 3-B		10	30
695	HLA-C Q95604: HLA class I histocompatibility antigen, Cw-17 alpha chain		8	10
1106	OPTN Q96CV9$_{1-3}$: Optineurin	3/3	2	33
657	HIST1H2BL Q99880: Histone H2B type 1-L	1/2	12	37
1552	TAGLN3 Q9UI15: Transgelin-3		2	4
619	GRID1 Q9ULK0$_{1}$: Glutamate receptor ionotropic, delta-1		2	15
1588	TLL2 Q9Y6L7: Tolloid-like protein 2		7	7

2 Protein Group Details

Protein groups are created by grouping according to mass spectrometry evidence:

1. Proteins which are detected with the exact same set of peptides are clustered - they are indistinguishable based on available MS data. Very often these are splice variants. In the following text, proteins are clustered proteins (cluster size one if they have specific peptides).
2. Proteins, which have unique peptides (*i. e.* peptides not present in other detected proteins) are promoted to *reporters*.
3. Remaining proteins are grouped to those reporters which have a superset of the peptides they have. These proteins are termed *group members*

Peptides are characterized based on their specificity to one or more proteins, and one or more protein groups:

reporter specific rs	●	Number of peptides specific to reporter.
group specific gs	■	Number of peptides specific to the group (including rs).
unspecific us	○	Number of peptides shared with any protein.

1 125116, NA, NA 11/0/25 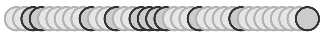

2 136429, NA, NA 8/0/0 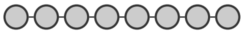

3		162648, NA, NA	20/0/7
4		A0A075B6Q5, NA, NA	1/0/0
5		A0A0A0MS15, NA, NA	2/0/0
6		A0A0B4J1V0, NA, NA	3/0/0
7		A0A0C4DH25, NA, NA	1/0/2
8	(1)	A0A0C4DH29, NA, NA	1/0/2
	(2)	P01743, IGHV1-46, Immunoglobulin heavy variable 1-46	
9		A0A0C4DH33, NA, NA	1/0/1
10	(1)	P04217-1, A1BG, Alpha-1B-glycoprotein	3/0/0
	(2)	P04217-2, A1BG, Alpha-1B-glycoprotein	
11		P01023, A2M, Alpha-2-macroglobulin	40/0/9
12	(1)	A8K2U0-1, A2ML1, Alpha-2-macroglobulin-like protein 1	1/0/0
	(2)	A8K2U0-2, A2ML1, Alpha-2-macroglobulin-like protein 1	
13		Q9NPC4, A4GALT, Lactosylceramide 4-alpha-galactosyltransferase	3/0/0
14	(1)	P49588-1, AARS, Alanine--tRNA ligase, cytoplasmic	2/0/0
	(2)	P49588-2, AARS, Alanine--tRNA ligase, cytoplasmic	
15	(1)	Q7Z7G0-1, ABI3BP, Target of Nesh-SH3	2/3/0
	(2)	Q7Z7G0-2, ABI3BP, Target of Nesh-SH3	
	(3)	Q7Z7G0-3,4, ABI3BP, Target of Nesh-SH3	
16		P49748-1-3, ACADVL, Very long-chain specific acyl-CoA dehydrogenase, mitochondrial	3/0/0
17		P53396-1-3, ACLY, ATP-citrate synthase	2/0/0
18		P21399, ACO1, Cytoplasmic aconitate hydratase	4/0/0
19		P15309-1,2, ACPP, Prostatic acid phosphatase	3/0/0
20	(1)	P60709, ACTB, Actin, cytoplasmic 1	5/0/16
	·	P63261, ACTG1, Actin, cytoplasmic 2	
	(2)	Q9BYX7, POTEKP, Putative beta-actin-like protein 3	
21		Q562R1, ACTBL2, Beta-actin-like protein 2	1/0/7
22	(1)	P68032, ACTC1, Actin, alpha cardiac muscle 1	0/6/12
	(2)	P62736, ACTA2, Actin, aortic smooth muscle	
	(3)	P68133, ACTA1, Actin, alpha skeletal muscle	
	(4)	P63267-1, ACTG2, Actin, gamma-enteric smooth muscle	
	(5)	P63267-2, ACTG2, Actin, gamma-enteric smooth muscle	
23	(1)	P12814-1,3, ACTN1, Alpha-actinin-1	1/0/13
	(2)	P12814-2,4, ACTN1, Alpha-actinin-1	

24	(1)	P35609-2, ACTN2, Alpha-actinin-2	0/0/8
	(2)	P35609-1, ACTN2, Alpha-actinin-2	
25		Q08043, ACTN3, Alpha-actinin-3	2/0/3
26	(1)	O43707-1, ACTN4, Alpha-actinin-4	1/11/11
	(2)	Q9H254-1,2,4, SPTBN4, Spectrin beta chain, non-erythrocytic 4	
	(3)	O43707-2, ACTN4, Alpha-actinin-4	
	(4)	O43707-3, ACTN4, Alpha-actinin-4	
27		P61163, ACTR1A, Alpha-centractin	3/0/1
28	(1)	P61160-1, ACTR2, Actin-related protein 2	1/0/1
	(2)	P61160-2, ACTR2, Actin-related protein 2	
29		P61158, ACTR3, Actin-related protein 3	7/0/0
30		O14672-1, ADAM10, Disintegrin and metalloproteinase domain-containing protein 10	10/0/0
31	(1)	Q13444-1-10,12, ADAM15, Disintegrin and metalloproteinase domain-containing protein 15	0/0/0
	(2)	Q13444-11, ADAM15, Disintegrin and metalloproteinase domain-containing protein 15	
	(3)	Q13444-13, ADAM15, Disintegrin and metalloproteinase domain-containing protein 15	
32	(1)	O15204-1, ADAMDEC1, ADAM DEC1	1/0/0
	(2)	O15204-2, ADAMDEC1, ADAM DEC1	
33		Q9UHI8, ADAMTS1, A disintegrin and metalloproteinase with thrombospondin motifs 1	1/0/0
34		Q76LX8-1-3, ADAMTS13, A disintegrin and metalloproteinase with thrombospondin motifs 13	2/0/0
35		Q8TE58, ADAMTS15, A disintegrin and metalloproteinase with thrombospondin motifs 15	5/0/0
36		O95450-1,2, ADAMTS2, A disintegrin and metalloproteinase with thrombospondin motifs 2	6/0/0
37		Q9UNA0, ADAMTS5, A disintegrin and metalloproteinase with thrombospondin motifs 5	4/0/0
38		Q9UP79, ADAMTS8, A disintegrin and metalloproteinase with thrombospondin motifs 8	4/0/0
39	(1)	Q9P2N4-3,4, ADAMTS9, A disintegrin and metalloproteinase with thrombospondin motifs 9	3/1/0
	(2)	Q9P2N4-1, ADAMTS9, A disintegrin and metalloproteinase with thrombospondin motifs 9	
	(3)	Q9P2N4-2, ADAMTS9, A disintegrin and metalloproteinase with thrombospondin motifs 9	

4/0/0 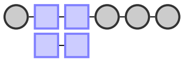

40 (1) Q8N6G6-3, ADAMTSL1, ADAMTS-like protein 1
(2) Q8N6G6-4, ADAMTSL1, ADAMTS-like protein 1

7/0/0 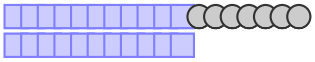

41 (1) Q6UY14-1,3, ADAMTSL4, ADAMTS-like protein 4
(2) Q6UY14-2, ADAMTSL4, ADAMTS-like protein 4

1/5/0 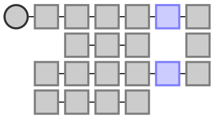

42 (1) P00325-1, ADH1B, Alcohol dehydrogenase 1B
(2) P07327, ADH1A, Alcohol dehydrogenase 1A
(3) P00325-2, ADH1B, Alcohol dehydrogenase 1B
(4) P00326, ADH1C, Alcohol dehydrogenase 1C

4/0/0 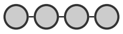

43 P08319-1,2, ADH4, Alcohol dehydrogenase 4

9/0/0 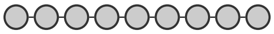

44 P11766, ADH5, Alcohol dehydrogenase class-3

5/0/0 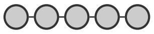

45 Q15848, ADIPOQ, Adiponectin

17/0/1 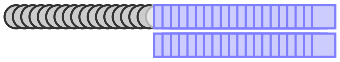

46 (1) Q8IUX7-1, AEBP1, Adipocyte enhancer-binding protein 1
(2) Q8IUX7-2, AEBP1, Adipocyte enhancer-binding protein 1

30/0/0 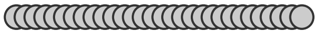

47 P43652, AFM, Afamin

1/0/1 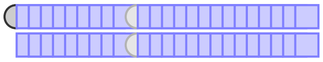

48 (1) O00468-1,3-7, AGRN, Agrin [Cleaved into: Agrin N-terminal 110 kDa subunit; Agrin C-terminal 110 kDa subunit; Agrin C-terminal 90 kDa fragment
(2) O00468-2, AGRN, Agrin [Cleaved into: Agrin N-terminal 110 kDa subunit; Agrin C-terminal 110 kDa subunit; Agrin C-terminal 90 kDa fragment

12/0/0 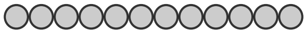

49 P01019, AGT, Angiotensinogen

1/0/0 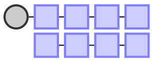

50 (1) P23526-1, AHCY, Adenosylhomocysteinase
(2) P23526-2, AHCY, Adenosylhomocysteinase

1/0/0 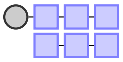

51 (1) O43865-1, AHCYL1, Adenosylhomocysteinase 2
(2) O43865-2, AHCYL1, Adenosylhomocysteinase 2

28/0/1 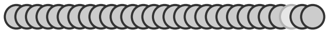

52 Q09666-1, AHNAK, Neuroblast differentiation-associated protein AHNAK

2/0/0 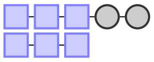

53 (1) O95433-1, AHSA1, Activator of 90 kDa heat shock protein ATPase homolog 1
(2) O95433-2, AHSA1, Activator of 90 kDa heat shock protein ATPase homolog 1

13/0/0 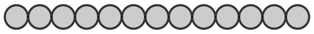

54 P02765, AHSG, Alpha-2-HS-glycoprotein

1/0/0 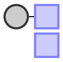

55 (1) O95831-1,3, AIFM1, Apoptosis-inducing factor 1, mitochondrial
(2) O95831-2,5, AIFM1, Apoptosis-inducing factor 1, mitochondrial

3/0/0 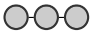

56 P00568, AK1, Adenylate kinase isoenzyme 1

2/0/0 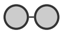

57 P14550, AKR1A1, Alcohol dehydrogenase [NADP(+)]

58		O43488, AKR7A2, Aflatoxin B1 aldehyde reductase member 2	5/0/0
59	(1)	P02768-1, ALB, Serum albumin	10/25/7
	(2)	P02768-2, ALB, Serum albumin	
	(3)	P02768-3, ALB, Serum albumin	
60		P00352, ALDH1A1, Retinal dehydrogenase 1	2/0/2
61	(1)	O94788-1, ALDH1A2, Retinal dehydrogenase 2	0/6/3
	(2)	P30837, ALDH1B1, Aldehyde dehydrogenase X, mitochondrial	
	(3)	O94788-2, ALDH1A2, Retinal dehydrogenase 2	
	(4)	O94788-3, ALDH1A2, Retinal dehydrogenase 2	
	(5)	O94788-4, ALDH1A2, Retinal dehydrogenase 2	
62		P47895, ALDH1A3, Aldehyde dehydrogenase family 1 member A3	7/0/3
63		P05091-1,2, ALDH2, Aldehyde dehydrogenase, mitochondrial	2/0/2
64		P30838, ALDH3A1, Aldehyde dehydrogenase, dimeric NADP-preferring	3/0/0
65	(1)	P49189-1, ALDH9A1, 4-trimethylaminobutyraldehyde dehydrogenase	2/0/0
	(2)	P49189-2, ALDH9A1, 4-trimethylaminobutyraldehyde dehydrogenase	
66	(1)	P04075-1,2, ALDOA, Fructose-bisphosphate aldolase A	10/0/0
	(2)	P09972, ALDOC, Fructose-bisphosphate aldolase C	
67		Q8TCU4-1-3, ALMS1, Alstrom syndrome protein 1	5/0/0
68		O75342, ALOX12B, Arachidonate 12-lipoxygenase, 12R-type	4/0/0
69	(1)	P16050-1, ALOX15, Arachidonate 15-lipoxygenase	1/0/0
	(2)	P16050-2, ALOX15, Arachidonate 15-lipoxygenase	
70		P02760, AMBP, Protein AMBP [Cleaved into: Alpha-1-microglobulin	16/0/0
71		P03950, ANG, Angiogenin	13/0/0
72		O15123-1-3, ANGPT2, Angiopoietin-2	1/0/0
73		O95841, ANGPTL1, Angiopoietin-related protein 1	5/0/0
74	(1)	Q9UKU9-1, ANGPTL2, Angiopoietin-related protein 2	8/0/0
	(2)	Q9UKU9-2, ANGPTL2, Angiopoietin-related protein 2	
75		Q9Y5C1, ANGPTL3, Angiopoietin-related protein 3	2/0/0
76	(1)	Q9BY76-1, ANGPTL4, Angiopoietin-related protein 4	0/3/0
	(2)	Q9BY76-2, ANGPTL4, Angiopoietin-related protein 4	

	(3)	Q9BY76-3, ANGPTL4, Angiopoietin-related protein 4	
77	(1)	Q9H6X2-1,5, ANTXR1, Anthrax toxin receptor 1	4/0/2
	(2)	Q9H6X2-2-4,6, ANTXR1, Anthrax toxin receptor 1	
78	(1)	P58335-4, ANTXR2, Anthrax toxin receptor 2	1/11/2
	(2)	P58335-1, ANTXR2, Anthrax toxin receptor 2	
	(3)	P58335-2, ANTXR2, Anthrax toxin receptor 2	
	(4)	P58335-3, ANTXR2, Anthrax toxin receptor 2	
79		P04083, ANXA1, Annexin A1	14/0/0
80		P50995-1,2, ANXA11, Annexin A11	3/0/0
81	(1)	P07355-1,2, ANXA2, Annexin A2	5/0/0
	(2)	A6NMY6, ANXA2P2, Putative annexin A2-like protein	
82		P12429, ANXA3, Annexin A3	3/0/0
83	(1)	P09525-1, ANXA4, Annexin A4	1/0/2
	(2)	P09525-2, ANXA4, Annexin A4	
84		P08758, ANXA5, Annexin A5	7/0/2
85		P08133-1,2, ANXA6, Annexin A6	4/0/0
86		P20073-1,2, ANXA7, Annexin A7	1/0/0
87	(1)	Q10567-3,4, AP1B1, AP-1 complex subunit beta-1	1/0/6
	(2)	Q10567-1,2, AP1B1, AP-1 complex subunit beta-1	
88		O43747-1,2, AP1G1, AP-1 complex subunit gamma-1	1/0/0
89	(1)	O95782-1,2, AP2A1, AP-2 complex subunit alpha-1	3/0/0
	(2)	O94973-1-3, AP2A2, AP-2 complex subunit alpha-2	
90	(1)	P63010-1,2, AP2B1, AP-2 complex subunit beta	1/0/6
	(2)	P63010-3, AP2B1, AP-2 complex subunit beta	
91		Q96CW1-1,2, AP2M1, AP-2 complex subunit mu	4/0/0
92		P02743, APCS, Serum amyloid P-component	9/0/0
93		Q9BZZ5-1-6, API5, Apoptosis inhibitor 5	2/0/0
94		P51693-1,2, APLP1, Amyloid-like protein 1	1/0/1
95	(1)	Q06481-1,3,6, APLP2, Amyloid-like protein 2	2/0/0
	(2)	Q06481-2,4,5, APLP2, Amyloid-like protein 2	
96	(1)	Q9HDC9-1, APMAP, Adipocyte plasma membrane-associated protein	2/0/0
	(2)	Q9HDC9-2, APMAP, Adipocyte plasma membrane-associated protein	

97		P02647, APOA1, Apolipoprotein A-I	34/0/1
98		P02652, APOA2, Apolipoprotein A-II	6/0/0
99		P06727, APOA4, Apolipoprotein A-IV	38/0/2
100		Q6Q788, APOA5, Apolipoprotein A-V	6/0/0
101		P04114, APOB, Apolipoprotein B-100	271/0/1
102		Q0VD83-1,3,4, APOBR, Apolipoprotein B receptor	4/0/0
103		P02654, APOC1, Apolipoprotein C-I	5/0/0
104		P02655, APOC2, Apolipoprotein C-II	3/0/0
105		P02656, APOC3, Apolipoprotein C-III	7/0/0
106		P55056, APOC4, Apolipoprotein C-IV	8/0/0
107		P05090, APOD, Apolipoprotein D	10/0/0
108		P02649, APOE, Apolipoprotein E	28/0/0
109		Q13790, APOF, Apolipoprotein F	5/0/0
110		P02749, APOH, Beta-2-glycoprotein 1	13/0/0
111		O14791-1-3, APOL1, Apolipoprotein L1	20/0/0
112		Q9BQE5, APOL2, Apolipoprotein L2	2/0/0
113		O95445-1, APOM, Apolipoprotein M	2/0/10
114		O95445-2, APOM, Apolipoprotein M	2/0/10
			0/2/1
115	(1)	P05067-1,3-9,11, APP, Amyloid beta A4 protein	
	(2)	P05067-10, APP, Amyloid beta A4 protein	
	(3)	P05067-2, APP, Amyloid beta A4 protein	
116		P07741-1,2, APRT, Adenine phosphoribosyltransferase	8/0/0
117		P48444-1,2, ARCN1, Coatomer subunit delta	9/0/0
118		P61204-1,2, ARF3, ADP-ribosylation factor 3<br>P84077, ARF1, ADP-ribosylation factor 1	1/0/1
119		P84085, ARF5, ADP-ribosylation factor 5	1/0/1
120		Q8N6H7-1,2, ARFGAP2, ADP-ribosylation factor GTPase-activating protein 2	3/0/0
			0/5/0
121	(1)	P05089-1, ARG1, Arginase-1	
	(2)	P05089-2, ARG1, Arginase-1	
	(3)	P05089-3, ARG1, Arginase-1	
122		Q07960, ARHGAP1, Rho GTPase-activating protein 1	3/0/0
123		Q8N392-1,2, ARHGAP18, Rho GTPase-activating protein 18	2/0/0

124	(1)	P52565-1, ARHGDIA, Rho GDP-dissociation inhibitor 1	3/0/0
	(2)	P52565-2, ARHGDIA, Rho GDP-dissociation inhibitor 1	
125		P52566, ARHGDIB, Rho GDP-dissociation inhibitor 2	4/0/0
126		P36405, ARL3, ADP-ribosylation factor-like protein 3	2/0/0
127		O15144, ARPC2, Actin-related protein 2/3 complex subunit 2	6/0/0
128		P59998-1-3, ARPC4, Actin-related protein 2/3 complex subunit 4	2/0/0
129		Q13510-1-3, ASAH1, Acid ceramidase	3/0/0
130		P07307-1-3, ASGR2, Asialoglycoprotein receptor 2	3/0/0
131		Q12797-1,10, ASPH, Aspartyl/asparaginyl beta-hydroxylase	9/0/0
132	(1)	Q9NT62-1, ATG3, Ubiquitin-like-conjugating enzyme ATG3	1/0/0
	(2)	Q9NT62-2, ATG3, Ubiquitin-like-conjugating enzyme ATG3	
133		P31939-1,2, ATIC, Bifunctional purine biosynthesis protein PURH [Cleaved into: Bifunctional purine biosynthesis protein PURH, N-terminally processed] [Includes: Phosphoribosylaminoimidazolecarboxamide formyltransferase	6/0/0
134		Q6DD88, ATL3, Atlastin-3	3/0/0
135	(1)	P16615-1,4, ATP2A2, Sarcoplasmic/endoplasmic reticulum calcium ATPase 2	1/2/0
	(2)	O14983-1-3, ATP2A1, Sarcoplasmic/endoplasmic reticulum calcium ATPase 1	
	(3)	P16615-2,3,5, ATP2A2, Sarcoplasmic/endoplasmic reticulum calcium ATPase 2	
136	(1)	P25705-1,2, ATP5A1, ATP synthase subunit alpha, mitochondrial	1/0/1
	(2)	P25705-3, ATP5A1, ATP synthase subunit alpha, mitochondrial	
137		P06576, ATP5B, ATP synthase subunit beta, mitochondrial	10/0/0
138		Q15904, ATP6AP1, V-type proton ATPase subunit S1	5/0/0
139		O75787-1,2, ATP6AP2, Renin receptor	10/0/0
140		P38606-1,2, ATP6V1A, V-type proton ATPase catalytic subunit A	4/0/0
141	(1)	P21281, ATP6V1B2, V-type proton ATPase subunit B, brain isoform	1/0/1
	(2)	P15313, ATP6V1B1, V-type proton ATPase subunit B, kidney isoform	
142	(1)	P36543-1,3, ATP6V1E1, V-type proton ATPase subunit E 1	0/1/0
	(2)	Q96A05, ATP6V1E2, V-type proton ATPase subunit E 2	
	(3)	P36543-2, ATP6V1E1, V-type proton ATPase subunit E 1	
143		Q9UBB4-1,2, ATXN10, Ataxin-10	2/0/0

144 P25311, AZGP1, Zinc-alpha-2-glycoprotein — 11/0/0

145 P61769, B2M, Beta-2-microglobulin [Cleaved into: Beta-2-microglobulin form pI 5.3] — 5/0/0

146 (1) O94766-1, B3GAT3, Galactosylgalactosylxylosylprotein 3-beta-glucuronosyltransferase 3 — 2/0/0

(2) O94766-2, B3GAT3, Galactosylgalactosylxylosylprotein 3-beta-glucuronosyltransferase 3

147 Q9NY97-1,2, B3GNT2, N-acetyllactosaminide beta-1,3-N-acetylglucosaminyltransferase 2 — 1/0/0

148 Q6UX72, B3GNT9, UDP-GlcNAc:betaGal beta-1,3-N-acetylglucosaminyltransferase 9 — 2/0/0

149 P15291-1,2, B4GALT1, Beta-1,4-galactosyltransferase 1 — 12/0/0

150 O60513, B4GALT4, Beta-1,4-galactosyltransferase 4 — 9/0/0

151 O43505, B4GAT1, Beta-1,4-glucuronyltransferase 1 — 6/0/0

152 Q07812-1,2,4,8, BAX, Apoptosis regulator BAX — 2/0/0

153 P51572-1,2, BCAP31, B-cell receptor-associated protein 31 — 3/0/0

154 P06276, BCHE, Cholinesterase — 2/0/0

155 Q86U42-1,2, PABPN1, Polyadenylate-binding protein 2 — 3/0/0

Q92843-2, BCL2L2, Bcl-2-like protein 2

156 (1) Q9BUT1-1, BDH2, 3-hydroxybutyrate dehydrogenase type 2 — 1/0/0

(2) Q9BUT1-2, BDH2, 3-hydroxybutyrate dehydrogenase type 2

157 P21810, BGN, Biglycan — 6/0/1

158 Q13867, BLMH, Bleomycin hydrolase — 9/0/0

159 P53004, BLVRA, Biliverdin reductase A — 3/0/0

160 P30043, BLVRB, Flavin reductase — 5/0/0

161 (1) P13497-1, BMP1, Bone morphogenetic protein 1 — 3/6/7

(2) P13497-2,5,6, BMP1, Bone morphogenetic protein 1

(3) P13497-3, BMP1, Bone morphogenetic protein 1

(4) P13497-4, BMP1, Bone morphogenetic protein 1

162 P12645, BMP3, Bone morphogenetic protein 3 — 4/0/0

163 Q9H3K6-1,2, BOLA2, BolA-like protein 2 — 1/0/0

164 (1) Q8TDL5-1, BPIFB1, BPI fold-containing family B member 1 — 5/0/1

(2) Q8TDL5-2, BPIFB1, BPI fold-containing family B member 1

165		Q12830-1,2,4, BPTF, Nucleosome-remodeling factor subunit BPTF	7/0/1
166		P43251-1-4, BTD, Biotinidase	4/0/0
167	(1)	P20290-1, BTF3, Transcription factor BTF3	1/0/0
	(2)	P20290-2, BTF3, Transcription factor BTF3	
168		Q7L1Q6-1-4, BZW1, Basic leucine zipper and W2 domain-containing protein 1	4/0/0
169		Q2M2W7-1, C17orf58, UPF0450 protein C17orf58	2/0/0
170		P02745, C1QA, Complement C1q subcomponent subunit A	3/0/0
171		P02746, C1QB, Complement C1q subcomponent subunit B	7/0/0
172		P02747, C1QC, Complement C1q subcomponent subunit C	5/0/0
173		Q9BXJ3, C1QTNF4, Complement C1q tumor necrosis factor-related protein 4	4/0/0
174		Q9BXJ0, C1QTNF5, Complement C1q tumor necrosis factor-related protein 5	3/0/0
175		P00736, C1R, Complement C1r subcomponent	31/0/2
176		Q9NZP8, C1RL, Complement C1r subcomponent-like protein	9/0/2
177		P09871, C1S, Complement C1s subcomponent	26/0/1
178	(1)	P06681-1, C2, Complement C2	2/23/0
	(2)	P06681-2, C2, Complement C2	
	(3)	P06681-3, C2, Complement C2	
179		Q9H1Z8, C2orf40, Augurin	2/0/0
180		P01024, C3, Complement C3	130/0/0
181	(1)	P0C0L4-1, C4A, Complement C4-A	0/0/83
	(2)	P0C0L4-2, C4A, Complement C4-A	
182		P0C0L5, C4B, Complement C4-B	5/0/83
183		P04003, C4BPA, C4b-binding protein alpha chain	32/0/0
184		P20851-1,2, C4BPB, C4b-binding protein beta chain	12/0/0
185		P01031, C5, Complement C5	72/0/1
186		P13671, C6, Complement component C6	45/0/0
187		P10643, C7, Complement component C7	42/0/0
188		P07357, C8A, Complement component C8 alpha chain	23/0/0
189		P07358, C8B, Complement component C8 beta chain	29/0/0
190		P07360, C8G, Complement component C8 gamma chain	13/0/0

191	(1)	P02748, C9, Complement component C9 [Cleaved into: Complement component C9a; Complement component C9b]	30/0/1
	(2)	Q96BY6-1-3, DOCK10, Dedicator of cytokinesis protein 10	
192		Q9Y376, CAB39, Calcium-binding protein 39	2/0/0
193	(1)	Q01668-2, CACNA1D, Voltage-dependent L-type calcium channel subunit alpha-1D	1/0/0
	(2)	Q01668-1,3,4, CACNA1D, Voltage-dependent L-type calcium channel subunit alpha-1D	
194		Q9HB71-1,2, CACYBP, Calcyclin-binding protein	1/0/1
195		P27708, CAD, CAD protein [Includes: Glutamine-dependent carbamoyl-phosphate synthase	2/0/0
196	(1)	Q05682-1, CALD1, Caldesmon	1/0/1
	(2)	Q05682-2-6, CALD1, Caldesmon	
197		P62158, CALM1, Calmodulin	8/0/2
198		P27482, CALML3, Calmodulin-like protein 3	6/0/2
199		Q9NZT1, CALML5, Calmodulin-like protein 5	5/0/0
200		P27797, CALR, Calreticulin	20/0/0
201	(1)	O43852-1,3, CALU, Calumenin	0/1/5
	(2)	O43852-10, CALU, Calumenin	
	(3)	O43852-11,13,14, CALU, Calumenin	
	(4)	O43852-5, CALU, Calumenin	
	(5)	O43852-6, CALU, Calumenin	
202	(1)	O43852-2,4, CALU, Calumenin	0/1/5
	(2)	O43852-12, CALU, Calumenin	
	(3)	O43852-15, CALU, Calumenin	
	(4)	O43852-7, CALU, Calumenin	
	(5)	O43852-8, CALU, Calumenin	
	(6)	O43852-9, CALU, Calumenin	
203		Q13557-1,3-6,8-12, CAMK2D, Calcium/calmodulin-dependent protein kinase type II subunit delta	1/0/1
204		P49913, CAMP, Cathelicidin antimicrobial peptide	3/0/0
205	(1)	Q86VP6-1, CAND1, Cullin-associated NEDD8-dissociated protein 1	2/3/0

(2) Q86VP6-2, CAND1, Cullin-associated NEDD8-dissociated protein 1

(3) Q86VP6-3, CAND1, Cullin-associated NEDD8-dissociated protein 1

206 (1) P27824-1,2, CANX, Calnexin — 3/0/1

(2) P27824-3, CANX, Calnexin

207 Q01518-1,2, CAP1, Adenylyl cyclase-associated protein 1 — 16/0/0

208 P40121-1,2, CAPG, Macrophage-capping protein — 5/0/0

209 P07384, CAPN1, Calpain-1 catalytic subunit — 6/0/1

210 (1) P17655-1, CAPN2, Calpain-2 catalytic subunit — 2/0/1

(2) P17655-2, CAPN2, Calpain-2 catalytic subunit

211 P04632, CAPNS1, Calpain small subunit 1 — 6/0/0

212 P52907, CAPZA1, F-actin-capping protein subunit alpha-1 — 6/0/2

213 (1) P47755-1, CAPZA2, F-actin-capping protein subunit alpha-2 — 1/0/2

(2) P47755-2, CAPZA2, F-actin-capping protein subunit alpha-2

214 (1) P47756-2, CAPZB, F-actin-capping protein subunit beta — 1/0/0

(2) P47756-1, CAPZB, F-actin-capping protein subunit beta

215 P31944, CASP14, Caspase-14 — 11/0/0

216 (1) P20810-5,6,10, CAST, Calpastatin — 0/6/0

(2) P20810-7,9, CAST, Calpastatin

(3) P20810-3, CAST, Calpastatin

(4) P20810-4, CAST, Calpastatin

(5) P20810-1,2,8, CAST, Calpastatin

217 P04040, CAT, Catalase — 2/0/0

218 Q03135-1,2, CAV1, Caveolin-1 — 2/0/0

219 (1) P16152-1, CBR1, Carbonyl reductase [NADPH] 1 — 9/0/0

(2) P16152-2, CBR1, Carbonyl reductase [NADPH] 1

220 Q13185, CBX3, Chromobox protein homolog 3 — 1/0/0

221 Q8N163-1,2, CCAR2, Cell cycle and apoptosis regulator protein 2 — 3/0/0

222 (1) Q8IYE0-1, CCDC146, Coiled-coil domain-containing protein 146 — 3/0/2

(2) Q8IYE0-2, CCDC146, Coiled-coil domain-containing protein 146

223		Q2TAC2-1,2, CCDC57, Coiled-coil domain-containing protein 57	1/0/1
224		Q76M96-1,2, CCDC80, Coiled-coil domain-containing protein 80	23/0/1
225		I3L3R5, CCER2, Coiled-coil domain-containing glutamate-rich protein 2	1/0/0
226	(1)	Q16627-1, CCL14, C-C motif chemokine 14	1/0/0
	(2)	Q16627-2, CCL14, C-C motif chemokine 14	
227		P55774, CCL18, C-C motif chemokine 18	2/0/0
228	(1)	P78371-1, CCT2, T-complex protein 1 subunit beta	1/0/0
	(2)	P78371-2, CCT2, T-complex protein 1 subunit beta	
229		P49368-1,2, CCT3, T-complex protein 1 subunit gamma	10/0/0
230		P50991-1,2, CCT4, T-complex protein 1 subunit delta	6/0/0
231	(1)	P48643-1, CCT5, T-complex protein 1 subunit epsilon	2/0/0
	(2)	P48643-2, CCT5, T-complex protein 1 subunit epsilon	
232	(1)	P40227-1,2, CCT6A, T-complex protein 1 subunit zeta	6/0/0
	(2)	Q92526-1-3, CCT6B, T-complex protein 1 subunit zeta-2	
233	(1)	Q99832-1, CCT7, T-complex protein 1 subunit eta	1/7/0
	(2)	Q99832-2, CCT7, T-complex protein 1 subunit eta	
	(3)	Q99832-3, CCT7, T-complex protein 1 subunit eta	
	(4)	Q99832-4, CCT7, T-complex protein 1 subunit eta	
234	(1)	P50990-1,2, CCT8, T-complex protein 1 subunit theta	4/0/0
	(2)	P50990-3, CCT8, T-complex protein 1 subunit theta	
235	(1)	Q6YHK3-1, CD109, CD109 antigen	0/12/0
	(2)	Q6YHK3-2, CD109, CD109 antigen	
	(3)	Q6YHK3-3, CD109, CD109 antigen	
	(4)	Q6YHK3-4, CD109, CD109 antigen	
236		P08571, CD14, Monocyte differentiation antigen CD14	15/0/0
237	(1)	Q86VB7-1-3, CD163, Scavenger receptor cysteine-rich type 1 protein M130	1/0/0
	(2)	Q86VB7-4, CD163, Scavenger receptor cysteine-rich type 1 protein M130	
238		Q9HCU0-1, CD248, Endosialin	10/0/0
239		O43866, CD5L, CD5 antigen-like	19/0/0

240		P04233-1,2, CD74, HLA class II histocompatibility antigen gamma chain	2/0/0
241		P60033, CD81, CD81 antigen	2/0/0
242		P01732-1-3, CD8A, T-cell surface glycoprotein CD8 alpha chain	1/0/0
243		P48960-1-3, CD97, CD97 antigen	6/0/0
244		P14209-1,3, CD99, CD99 antigen	2/0/0
245		Q16543, CDC37, Hsp90 co-chaperone Cdc37	4/0/0
			1/0/1
246	(1)	P60953-2, CDC42, Cell division control protein 42 homolog	
	(2)	P60953-1, CDC42, Cell division control protein 42 homolog	
247		P12830-1,2, CDH1, Cadherin-1	3/0/0
			1/0/0
248	(1)	P19022-1, CDH2, Cadherin-2	
	(2)	P19022-2, CDH2, Cadherin-2	
249		Q15517, CDSN, Corneodesmosin	1/0/0
250	(1)	O95319-1-5, CELF2, CUGBP Elav-like family member 2	1/0/0
	(2)	Q92879-1-6, CELF1, CUGBP Elav-like family member 1	
251		Q9HCU4, CELSR2, Cadherin EGF LAG seven-pass G-type receptor 2	3/0/0
252		P49454, CENPF, Centromere protein F	1/0/1
253		Q9BS16, CENPK, Centromere protein K	3/0/0
254		Q5VT06, CEP350, Centrosome-associated protein 350	2/0/1
			5/0/0
255	(1)	P11597-1, CETP, Cholesteryl ester transfer protein	
	(2)	P11597-2, CETP, Cholesteryl ester transfer protein	
			13/0/0
256	(1)	P00751-1, CFB, Complement factor B	
	(2)	P00751-2, CFB, Complement factor B	
257		P00746, CFD, Complement factor D	17/0/0
			31/0/9
258	(1)	P08603-1, CFH, Complement factor H	
	(2)	P08603-2, CFH, Complement factor H	
259		Q03591, CFHR1, Complement factor H-related protein 1	1/0/12
			1/0/5
260	(1)	P36980-1, CFHR2, Complement factor H-related protein 2	
	(2)	P36980-2, CFHR2, Complement factor H-related protein 2	
261		Q02985-1,2, CFHR3, Complement factor H-related protein 3	3/0/6
			4/0/5
262	(1)	Q92496-1,2, CFHR4, Complement factor H-related protein 4	
	(2)	Q92496-3, CFHR4, Complement factor H-related protein 4	

19/0/2 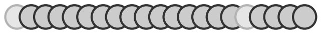

263 Q9BXR6, CFHR5, Complement factor H-related protein 5

22/0/0 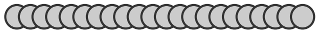

264 P05156, CFI, Complement factor I

6/1/1 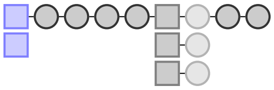

265 (1) P23528, CFL1, Cofilin-1
(2) Q9Y281-1, CFL2, Cofilin-2
(3) Q9Y281-3, CFL2, Cofilin-2

13/0/1 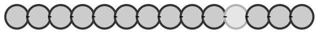

266 P27918, CFP, Properdin

7/0/0 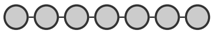

267 Q99674-1,4,5, CGREF1, Cell growth regulator with EF hand domain protein 1

1/0/1 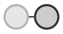

268 Q3L8U1-1-3, CHD9, Chromodomain-helicase-DNA-binding protein 9

30/0/0 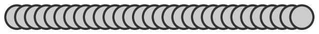

269 P10645, CHGA, Chromogranin-A

21/0/1 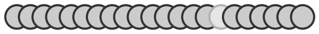

270 P05060, CHGB, Secretogranin-1

2/0/0 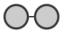

271 Q96FZ7, CHMP6, Charged multivesicular body protein 6

8/1/0 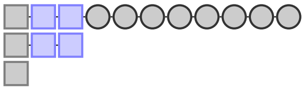

272 (1) Q9H2X0-1,5, CHRD, Chordin
(2) Q9H2X0-4, CHRD, Chordin
(3) Q9H2X0-2,3, CHRD, Chordin

1/20/0 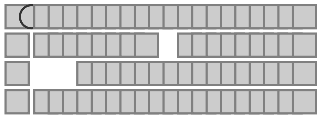

273 (1) Q9BU40-2,4, CHRDL1, Chordin-like protein 1
(2) Q9BU40-1,6, CHRDL1, Chordin-like protein 1
(3) Q9BU40-3, CHRDL1, Chordin-like protein 1
(4) Q9BU40-5, CHRDL1, Chordin-like protein 1

1/0/0 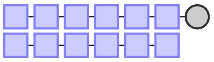

274 (1) Q6WN34-1, CHRDL2, Chordin-like protein 2
(2) Q6WN34-2, CHRDL2, Chordin-like protein 2

1/0/0 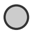

275 Q9NRB3, CHST12, Carbohydrate sulfotransferase 12

3/0/0 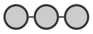

276 Q8NCH0, CHST14, Carbohydrate sulfotransferase 14

2/0/0 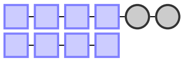

277 (1) Q7LFX5-1, CHST15, Carbohydrate sulfotransferase 15
(2) Q7LFX5-2, CHST15, Carbohydrate sulfotransferase 15

50/0/1 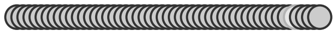

278 O75339, CILP, Cartilage intermediate layer protein 1

3/0/1 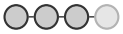

279 Q8IUL8, CILP2, Cartilage intermediate layer protein 2

1/0/0 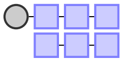

280 (1) Q14011-1, CIRBP, Cold-inducible RNA-binding protein
(2) Q14011-3, CIRBP, Cold-inducible RNA-binding protein

4/0/1 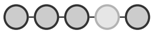

281 Q07065, CKAP4, Cytoskeleton-associated protein 4

8/0/2 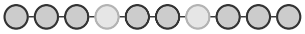

282 P12277, CKB, Creatine kinase B-type

2/0/2 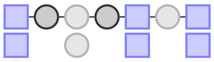

283 (1) P12532-1,2, CKMT1A, Creatine kinase U-type, mitochondrial

	(2)	P17540, CKMT2, Creatine kinase S-type, mitochondrial	
284		Q05315, CLC, Galectin-10	3/0/0
285		Q9Y240, CLEC11A, C-type lectin domain family 11 member A	14/0/0
286		P05452, CLEC3B, Tetranectin	7/0/0
287		O00299, CLIC1, Chloride intracellular channel protein 1	8/0/1
288		O95833, CLIC3, Chloride intracellular channel protein 3	4/0/0
289		Q9Y696, CLIC4, Chloride intracellular channel protein 4	5/0/1
290		O94985-1, CLSTN1, Calsyntenin-1	1/0/15
291		O94985-2, CLSTN1, Calsyntenin-1	1/0/15
292		Q9BQT9-1,2, CLSTN3, Calsyntenin-3	5/0/0
293		P09496-1-4, CLTA, Clathrin light chain A	2/0/0
			1/0/0
294	(1)	P09497-2, CLTB, Clathrin light chain B	
	(2)	P09497-1, CLTB, Clathrin light chain B	
295		Q00610-1,2, CLTC, Clathrin heavy chain 1	22/0/7
			1/0/7
296	(1)	P53675-1, CLTCL1, Clathrin heavy chain 2	
	(2)	P53675-2, CLTCL1, Clathrin heavy chain 2	
			1/16/0
297	(1)	P10909-4, CLU, Clusterin	
	(2)	P10909-3, CLU, Clusterin	
	(3)	P10909-1,2,5, CLU, Clusterin	
298		Q96KN2, CNDP1, Beta-Ala-His dipeptidase	6/0/0
			3/0/1
299	(1)	Q99439-1, CNN2, Calponin-2	
	(2)	Q99439-2, CNN2, Calponin-2	
300	(1)	Q15417-1,3, CNN3, Calponin-3	1/2/1
	(2)	P51911-1,2, CNN1, Calponin-1	
	(3)	Q15417-2, CNN3, Calponin-3	
301		Q9Y2B0-1, CNPY2, Protein canopy homolog 2	1/0/0
			0/27/1
302	(1)	Q99715-1, COL12A1, Collagen alpha-1(XII) chain	
	(2)	Q99715-2, COL12A1, Collagen alpha-1(XII) chain	
	(3)	Q99715-4, COL12A1, Collagen alpha-1(XII) chain	
			5/0/1
303	(1)	Q05707-1,2, COL14A1, Collagen alpha-1(XIV) chain	
	(2)	Q05707-3, COL14A1, Collagen alpha-1(XIV) chain	
304		P39059, COL15A1, Collagen alpha-1(XV) chain [Cleaved into: Restin	5/0/0

305		Q07092-1,2, COL16A1, Collagen alpha-1(XVI) chain	3/0/0
306		P39060-1-3, COL18A1, Collagen alpha-1(XVIII) chain [Cleaved into: Endostatin]	13/0/0
307		P02452, COL1A1, Collagen alpha-1(I) chain	23/0/2
308		P08123, COL1A2, Collagen alpha-2(I) chain	22/0/0
309		P02458-2, COL2A1, Collagen alpha-1(II) chain	1/0/1
310	(1)	P02461-1, COL3A1, Collagen alpha-1(III) chain	4/0/3
	(2)	P02461-2, COL3A1, Collagen alpha-1(III) chain	
311		P02462-1,2, COL4A1, Collagen alpha-1(IV) chain [Cleaved into: Arresten]	1/0/0
312		Q9Y5P4-1-3, COL4A3BP, Collagen type IV alpha-3-binding protein	2/0/0
313		P20908, COL5A1, Collagen alpha-1(V) chain	11/0/0
314		P05997, COL5A2, Collagen alpha-2(V) chain	13/0/2
315		P12109, COL6A1, Collagen alpha-1(VI) chain	30/0/0
316	(1)	P12110-1, COL6A2, Collagen alpha-2(VI) chain	7/0/17
	(2)	P12110-3, COL6A2, Collagen alpha-2(VI) chain	
317		P12110-2, COL6A2, Collagen alpha-2(VI) chain	1/0/17
318	(1)	P12111-1, COL6A3, Collagen alpha-3(VI) chain	4/14/104
	(2)	P12111-2, COL6A3, Collagen alpha-3(VI) chain	
	(3)	P12111-3, COL6A3, Collagen alpha-3(VI) chain	
	(4)	P12111-5, COL6A3, Collagen alpha-3(VI) chain	
319		P12111-4, COL6A3, Collagen alpha-3(VI) chain	2/0/104
320		A6NMZ7-1,2, COL6A6, Collagen alpha-6(VI) chain	3/0/0
321		P27658, COL8A1, Collagen alpha-1(VIII) chain	2/0/1
322		Q9Y6Z7, COLEC10, Collectin-10	1/0/0
323		Q9BWP8-1-10, COLEC11, Collectin-11	3/0/0
324		Q8NBJ5, COLGALT1, Procollagen galactosyltransferase 1	1/0/0
325	(1)	P49747-1, COMP, Cartilage oligomeric matrix protein	1/0/2
	(2)	P49747-2, COMP, Cartilage oligomeric matrix protein	
326		P53621-1,2, COPA, Coatomer subunit alpha	8/0/0
327		P53618, COPB1, Coatomer subunit beta	11/0/0
328		P35606-1,2, COPB2, Coatomer subunit beta’	2/0/0

329	(1)	O14579-1,3, COPE, Coatomer subunit epsilon	1/0/0
	(2)	O14579-2, COPE, Coatomer subunit epsilon	
330		Q9Y678, COPG1, Coatomer subunit gamma-1	7/0/2
331		Q9UBF2-1,2, COPG2, Coatomer subunit gamma-2	2/0/2
332		P61201-1,2, COPS2, COP9 signalosome complex subunit 2	1/0/0
333		P31146, CORO1A, Coronin-1A	6/0/1
334		Q9BR76, CORO1B, Coronin-1B	1/0/1
335		Q9ULV4-1-3, CORO1C, Coronin-1C	4/0/1
336		Q14019, COTL1, Coactosin-like protein	3/0/0
337		P00450, CP, Ceruloplasmin	34/0/0
338	(1)	Q96IY4-1, CPB2, Carboxypeptidase B2	5/0/0
	(2)	Q96IY4-2, CPB2, Carboxypeptidase B2	
339		P16870-1,2, CPE, Carboxypeptidase E	24/0/0
340		P15169, CPN1, Carboxypeptidase N catalytic chain	12/0/0
341		P22792, CPN2, Carboxypeptidase N subunit 2	14/0/0
342		Q9Y646, CPQ, Carboxypeptidase Q	8/0/0
343		Q16630-1-3, CPSF6, Cleavage and polyadenylation specificity factor subunit 6	1/0/0
344		Q9H3G5, CPVL, Probable serine carboxypeptidase CPVL	2/0/0
345		Q96SM3, CPXM1, Probable carboxypeptidase X1	8/0/0
346		Q8N436, CPXM2, Inactive carboxypeptidase-like protein X2	9/0/1
347		Q66K79-1-3, CPZ, Carboxypeptidase Z	4/0/0
348	(1)	P29373, CRABP2, Cellular retinoic acid-binding protein 2	4/0/0
	(2)	P29762, CRABP1, Cellular retinoic acid-binding protein 1	
349		Q9UGL9, CRCT1, Cysteine-rich C-terminal protein 1	1/0/0
350		P24387, CRHBP, Corticotropin-releasing factor-binding protein	3/0/0
351		P50238, CRIP1, Cysteine-rich protein 1	2/0/0
352	(1)	P02741-1, CRP, C-reactive protein [Cleaved into: C-reactive protein(1-205)]	3/0/0
	(2)	P02741-2, CRP, C-reactive protein [Cleaved into: C-reactive protein(1-205)]	
353	(1)	Q9NQ79-1,2, CRTAC1, Cartilage acidic protein 1	1/0/0
	(2)	Q9NQ79-3, CRTAC1, Cartilage acidic protein 1	
354		P02511, CRYAB, Alpha-crystallin B chain	5/0/0

355	(1)	P55060-1,3, CSE1L, Exportin-2	2/2/0
	(2)	P55060-2, CSE1L, Exportin-2	
	(3)	P55060-4, CSE1L, Exportin-2	
356		Q6UVK1, CSPG4, Chondroitin sulfate proteoglycan 4	10/0/0
357		P01036, CST4, Cystatin-S	1/0/0
		P09228, CST2, Cystatin-SA	
358		P01034, CST3, Cystatin-C	6/0/0
359		Q15828, CST6, Cystatin-M	7/0/0
360		P01040, CSTA, Cystatin-A	3/0/0
361		P04080, CSTB, Cystatin-B	4/0/0
362		Q01459, CTBS, Di-N-acetylchitobiase	1/0/0
363		P29279-1,2, CTGF, Connective tissue growth factor	4/0/0
364	(1)	Q96CG8-1,3, CTHRC1, Collagen triple helix repeat-containing protein 1	1/0/0
	(2)	Q96CG8-2, CTHRC1, Collagen triple helix repeat-containing protein 1	
365	(1)	P35221-1-3, CTNNA1, Catenin alpha-1	4/0/0
	(2)	P26232-1-6, CTNNA2, Catenin alpha-2	
366		P35222-1, CTNNB1, Catenin beta-1	3/0/3
367		O60716-1-32, CTNND1, Catenin delta-1	2/0/0
368		P10619-1,2, CTSA, Lysosomal protective protein	6/0/0
369		P07858, CTSB, Cathepsin B	5/0/0
370		P07339, CTSD, Cathepsin D	11/0/0
371		Q9UBX1, CTSF, Cathepsin F	13/0/0
372		P08311, CTSG, Cathepsin G	2/0/0
373		P09668, CTSH, Pro-cathepsin H [Cleaved into: Cathepsin H mini chain; Cathepsin H	2/0/0
374		P43235, CTSK, Cathepsin K	3/0/0
375		Q9UBR2, CTSZ, Cathepsin Z	2/0/0
376	(1)	Q14247-1,3, CTTN, Src substrate cortactin	1/0/0
	(2)	Q14247-2, CTTN, Src substrate cortactin	
377		Q13618-1-3, CUL3, Cullin-3	3/0/0
378		O60888-1-3, CUTA, Protein CutA	2/0/0
379		P48061-1-6, CXCL12, Stromal cell-derived factor 1	3/0/0

380 Q9H7Y0-1, CXorf36, Deleted in autism-related protein 1 — 4/0/0

381 P00167-1-3, CYB5A, Cytochrome b5 — 1/0/0

382 P00387-1-3, CYB5R3, NADH-cytochrome b5 reductase 3 — 2/0/0

383 — 1/1/2
(1) Q7L576-1, CYFIP1, Cytoplasmic FMR1-interacting protein 1
(2) Q7L576-2, CYFIP1, Cytoplasmic FMR1-interacting protein 1
(3) Q7L576-3, CYFIP1, Cytoplasmic FMR1-interacting protein 1

384 Q96F07-1,2, CYFIP2, Cytoplasmic FMR1-interacting protein 2 — 3/0/2

385 Q9NRR1, CYTL1, Cytokine-like protein 1 — 3/0/0

386 Q14118, DAG1, Dystroglycan — 5/0/0

387 P14868-1,2, DARS, Aspartate--tRNA ligase, cytoplasmic — 5/0/0

388 P07108-1-5, DBI, Acyl-CoA-binding protein — 2/0/0

389 — 1/2/0
(1) Q9UJU6-1,5, DBNL, Drebrin-like protein
(2) Q9UJU6-6, DBNL, Drebrin-like protein
(3) Q9UJU6-2-4, DBNL, Drebrin-like protein

390 — 2/0/0
(1) P81605-1, DCD, Dermcidin
(2) P81605-2, DCD, Dermcidin

391 Q96JQ0, DCHS1, Protocadherin-16 — 2/0/0

392 — 3/6/1
(1) P07585-1, DCN, Decorin
(2) P07585-2, DCN, Decorin
(3) P07585-3, DCN, Decorin
(4) P07585-4, DCN, Decorin
(5) P07585-5, DCN, Decorin

393 Q96C86, DCPS, m7GpppX diphosphatase — 1/0/0

394 — 0/0/1
(1) Q14203-1,3,4,6, DCTN1, Dynactin subunit 1
(2) Q14203-2,5, DCTN1, Dynactin subunit 1

395 O75935-1,2, DCTN3, Dynactin subunit 3 — 1/0/0

396 — 1/0/0
(1) Q16531-1, DDB1, DNA damage-binding protein 1
(2) Q16531-2, DDB1, DNA damage-binding protein 1

397 P39656-1,3, DDOST, Dolichyl-diphosphooligosaccharide--protein glycosyltransferase 48 kDa subunit — 2/0/0

0/4/1 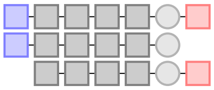

398 (1) Q92499-1, DDX1, ATP-dependent RNA helicase DDX1

(2) Q92499-2, DDX1, ATP-dependent RNA helicase DDX1

(3) Q92499-3, DDX1, ATP-dependent RNA helicase DDX1

399 Q92841-1-4, DDX17, Probable ATP-dependent RNA helicase DDX17 — 1/0/3 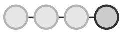

400 (1) Q9NUU7-1, DDX19A, ATP-dependent RNA helicase DDX19A — 1/2/0 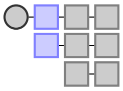

(2) Q9UMR2-1,2,4, DDX19B, ATP-dependent RNA helicase DDX19B

(3) Q9NUU7-2, DDX19A, ATP-dependent RNA helicase DDX19A

· Q9UMR2-3, DDX19B, ATP-dependent RNA helicase DDX19B

401 (1) Q13838-1,2, DDX39B, Spliceosome RNA helicase DDX39B — 3/2/0 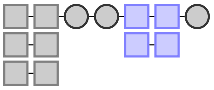

(2) O00148-1, DDX39A, ATP-dependent RNA helicase DDX39A

(3) O00148-2,3, DDX39A, ATP-dependent RNA helicase DDX39A

402 (1) O00571-1,2, DDX3X, ATP-dependent RNA helicase DDX3X — 3/0/1 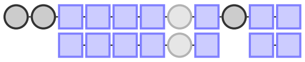

(2) O15523-1,2, DDX3Y, ATP-dependent RNA helicase DDX3Y

2/0/3 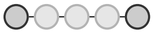

403 P17844-1,2, DDX5, Probable ATP-dependent RNA helicase DDX5

2/0/3 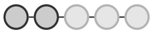

404 P59665, DEFA1, Neutrophil defensin 1

3/0/3 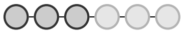

405 P59666, DEFA3, Neutrophil defensin 3

3/0/0 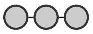

406 P12838, DEFA4, Neutrophil defensin 4

3/0/0 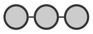

407 Q01523, DEFA5, Defensin-5

1/0/0 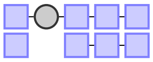

408 (1) P35659-1, DEK, Protein DEK

(2) P35659-2, DEK, Protein DEK

11/0/6 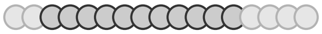

409 P17661, DES, Desmin

5/1/0 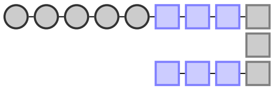

410 (1) O60443-1, DFNA5, Non-syndromic hearing impairment protein 5

(2) O60443-2, DFNA5, Non-syndromic hearing impairment protein 5

(3) O60443-3, DFNA5, Non-syndromic hearing impairment protein 5

10/0/0 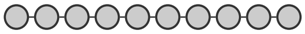

411 Q08211-1, DHX9, ATP-dependent RNA helicase A

4/0/0 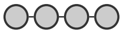

412 O60610-1-3, DIAPH1, Protein diaphanous homolog 1

6/0/0 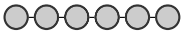

413 O60879-1,2, DIAPH2, Protein diaphanous homolog 2

8/0/0 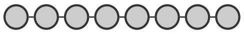

414 Q9UBP4, DKK3, Dickkopf-related protein 3

415	(1)	P09622-1,2, DLD, Dihydrolipoyl dehydrogenase, mitochondrial	1/0/0
	(2)	P09622-3, DLD, Dihydrolipoyl dehydrogenase, mitochondrial	
416		Q6E0U4-1-7,16, DMKN, Dermokine	6/0/0
417		Q13316-1,2, DMP1, Dentin matrix acidic phosphoprotein 1	3/0/0
418		Q6ZR08-1, DNAH12, Dynein heavy chain 12, axonemal	7/0/0
419		O60884, DNAJA2, DnaJ homolog subfamily A member 2	5/0/0
420		P25685-1, DNAJB1, DnaJ homolog subfamily B member 1	4/0/0
421		Q9UBS4, DNAJB11, DnaJ homolog subfamily B member 11	6/0/0
422		Q9UBS3, DNAJB9, DnaJ homolog subfamily B member 9	2/0/0
423		Q13217, DNAJC3, DnaJ homolog subfamily C member 3	18/0/1
424	(1)	P50570-1-5, DNM2, Dynamin-2	5/0/0
	(2)	Q05193-1-3,5, DNM1, Dynamin-1	
425	(1)	O43598-1, DNPH1, 2'-deoxynucleoside 5'-phosphate N-hydrolase 1	1/0/0
	(2)	O43598-2, DNPH1, 2'-deoxynucleoside 5'-phosphate N-hydrolase 1	
426		Q5JSL3, DOCK11, Dedicator of cytokinesis protein 11	1/0/1
427		P27487, DPP4, Dipeptidyl peptidase 4	2/0/0
428		Q07507, DPT, Dermatopontin	9/0/0
429	(1)	Q16555-1,2, DPYSL2, Dihydropyrimidinase-related protein 2	4/0/2
	(2)	Q14194-1,2, CRMP1, Dihydropyrimidinase-related protein 1	
430	(1)	Q14195-2, DPYSL3, Dihydropyrimidinase-related protein 3	1/0/2
	(2)	Q14195-1, DPYSL3, Dihydropyrimidinase-related protein 3	
431		Q9BPU6, DPYSL5, Dihydropyrimidinase-related protein 5	2/0/0
432	(1)	Q08554-1, DSC1, Desmocollin-1	1/0/0
	(2)	Q08554-2, DSC1, Desmocollin-1	
433		Q02487-1,2, DSC2, Desmocollin-2	2/0/1
434	(1)	Q14574-1, DSC3, Desmocollin-3	1/0/1
	(2)	Q14574-2, DSC3, Desmocollin-3	
435	(1)	Q02413-1, DSG1, Desmoglein-1	10/0/2
	(2)	Q02413-2, DSG1, Desmoglein-1	
436		Q86SJ6-1,2, DSG4, Desmoglein-4	2/0/2
437	(1)	P15924-1, DSP, Desmoplakin	34/85/0

	(2)	P15924-2, DSP, Desmoplakin	
	(3)	P15924-3, DSP, Desmoplakin	
438		Q03001-7,8, DST, Dystonin	0/0/2
439		P60981-1,2, DSTN, Destrin	4/0/1
440		O95147, DUSP14, Dual specificity protein phosphatase 14	3/0/0
441		Q14204, DYNC1H1, Cytoplasmic dynein 1 heavy chain 1	38/0/0
442		O43237-1, DYNC1LI2, Cytoplasmic dynein 1 light intermediate chain 2	3/0/0
443		P63167, DYNLL1, Dynein light chain 1, cytoplasmic	2/0/0
			9/6/0
444	(1)	Q16610-1,4, ECM1, Extracellular matrix protein 1	
	(2)	Q16610-2, ECM1, Extracellular matrix protein 1	
	(3)	Q16610-3, ECM1, Extracellular matrix protein 1	
445		O94769-1, ECM2, Extracellular matrix protein 2	2/0/23
446		O94769-2, ECM2, Extracellular matrix protein 2	1/0/23
447	(1)	P68104-1, EEF1A1, Elongation factor 1-alpha 1	1/7/0
	·	Q5VTE0, EEF1A1P5, Putative elongation factor 1-alpha-like 3	
	(2)	P68104-2, EEF1A1, Elongation factor 1-alpha 1	
	(3)	Q05639, EEF1A2, Elongation factor 1-alpha 2	
448		P24534, EEF1B2, Elongation factor 1-beta	5/0/1
			1/0/1
449	(1)	P29692-1-3, EEF1D, Elongation factor 1-delta	
	(2)	P29692-4, EEF1D, Elongation factor 1-delta	
450		P26641-1,2, EEF1G, Elongation factor 1-gamma	13/0/0
451		P13639, EEF2, Elongation factor 2	30/0/0
452		Q12805-1-4, EFEMP1, EGF-containing fibulin-like extracellular matrix protein 1	4/0/23
453		Q12805-5, EFEMP1, EGF-containing fibulin-like extracellular matrix protein 1	2/0/23
454		O95967, EFEMP2, EGF-containing fibulin-like extracellular matrix protein 2	17/0/0
455	(1)	Q9H4M9, EHD1, EH domain-containing protein 1	5/1/1
	(2)	Q9NZN3-1, EHD3, EH domain-containing protein 3	
	(3)	Q9NZN3-2, EHD3, EH domain-containing protein 3	
			3/0/1
456	(1)	Q9NZN4-1, EHD2, EH domain-containing protein 2	
	(2)	Q9NZN4-2, EHD2, EH domain-containing protein 2	
457		O14602, EIF1AY, Eukaryotic translation initiation factor 1A, Y-chromosomal	2/0/0
		P47813, EIF1AX, Eukaryotic translation initiation factor 1A, X-chromosomal	

458 P05198, EIF2S1, Eukaryotic translation initiation factor 2 subunit 1 — 5/0/0 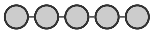

459 P20042, EIF2S2, Eukaryotic translation initiation factor 2 subunit 2 — 2/0/0 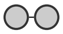

460 (1) P41091, EIF2S3, Eukaryotic translation initiation factor 2 subunit 3 — 2/0/0 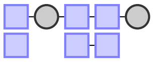

(2) Q2VIR3-1,2, EIF2S3L, Putative eukaryotic translation initiation factor 2 subunit 3-like protein

461 Q14152-1,2, EIF3A, Eukaryotic translation initiation factor 3 subunit A — 7/0/0 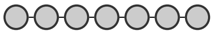

462 P55884-1,2, EIF3B, Eukaryotic translation initiation factor 3 subunit B — 6/0/0 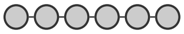

463 B5ME19, EIF3CL, Eukaryotic translation initiation factor 3 subunit C-like protein — 4/0/0 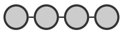

Q99613-1,2, EIF3C, Eukaryotic translation initiation factor 3 subunit C

464 P60228, EIF3E, Eukaryotic translation initiation factor 3 subunit E — 5/0/0 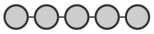

465 O15372, EIF3H, Eukaryotic translation initiation factor 3 subunit H — 4/0/0 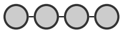

466 Q13347, EIF3I, Eukaryotic translation initiation factor 3 subunit I — 2/0/0 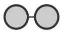

0/3/0 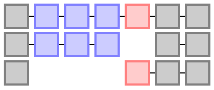

467 (1) O75822-1, EIF3J, Eukaryotic translation initiation factor 3 subunit J

(2) O75822-2, EIF3J, Eukaryotic translation initiation factor 3 subunit J

(3) O75822-3, EIF3J, Eukaryotic translation initiation factor 3 subunit J

468 Q9UBQ5-1,2, EIF3K, Eukaryotic translation initiation factor 3 subunit K — 3/0/0 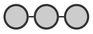

1/0/5 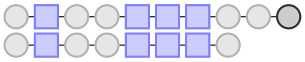

469 (1) P60842-1, EIF4A1, Eukaryotic initiation factor 4A-I

(2) P60842-2, EIF4A1, Eukaryotic initiation factor 4A-I

470 Q14240-1,2, EIF4A2, Eukaryotic initiation factor 4A-II — 1/0/5 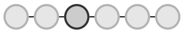

471 P38919, EIF4A3, Eukaryotic initiation factor 4A-III — 3/0/2 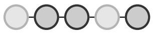

472 P23588-1,2, EIF4B, Eukaryotic translation initiation factor 4B — 3/0/0 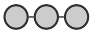

473 P06730-1-3, EIF4E, Eukaryotic translation initiation factor 4E — 2/0/0 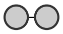

0/8/1 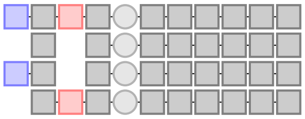

474 (1) Q04637-8, EIF4G1, Eukaryotic translation initiation factor 4 gamma 1

(2) Q04637-4-6, EIF4G1, Eukaryotic translation initiation factor 4 gamma 1

	(3)	Q04637-1,3,9, EIF4G1, Eukaryotic translation initiation factor 4 gamma 1	
	(4)	Q04637-7, EIF4G1, Eukaryotic translation initiation factor 4 gamma 1	
475		O43432-1,3,4, EIF4G3, Eukaryotic translation initiation factor 4 gamma 3	4/0/1
476		Q15056-1,2, EIF4H, Eukaryotic translation initiation factor 4H	4/0/0
477	(1)	P63241-1,2, EIF5A, Eukaryotic translation initiation factor 5A-1	1/1/0
	(2)	Q6IS14, EIF5AL1, Eukaryotic translation initiation factor 5A-1-like	
	(3)	Q9GZV4, EIF5A2, Eukaryotic translation initiation factor 5A-2	
478		P56537-1, EIF6, Eukaryotic translation initiation factor 6	2/0/0
479		P08246, ELANE, Neutrophil elastase	2/0/0
480		Q15717-1,2, ELAVL1, ELAV-like protein 1	1/0/0
481		P15502-1,2,4,5, ELN, Elastin	2/0/0
482	(1)	Q9Y6C2-1, EMILIN1, EMILIN-1	9/0/0
	(2)	Q9Y6C2-2, EMILIN1, EMILIN-1	
483		Q9BXX0, EMILIN2, EMILIN-2	14/0/0
484	(1)	P06733-1, ENO1, Alpha-enolase	7/5/0
	(2)	P06733-2, ENO1, Alpha-enolase	
	(3)	P09104-1,2, ENO2, Gamma-enolase	
	(4)	P13929-1-3, ENO3, Beta-enolase	
485		P58107, EPPK1, Epiplakin	20/0/4
486		P07814, EPRS, Bifunctional glutamate/proline--tRNA ligase	5/0/1
487		Q8TE68-1,2,4, EPS8L1, Epidermal growth factor receptor kinase substrate 8-like protein 1	3/0/0
488		Q9H6S3-1,3, EPS8L2, Epidermal growth factor receptor kinase substrate 8-like protein 2	4/0/0
489		P84090, ERH, Enhancer of rudimentary homolog	2/0/0
490		O94905-1, ERLIN2, Erlin-2	2/0/0
491		Q9BS26, ERP44, Endoplasmic reticulum resident protein 44	10/0/0
492		P10768, ESD, S-formylglutathione hydrolase	1/0/0
493		P62495-1,2, ETF1, Eukaryotic peptide chain release factor subunit 1	3/0/0
494		Q92817, EVPL, Envoplakin	15/0/0
495		Q01844-1-3,5,6, EWSR1, RNA-binding protein EWS	1/0/0
496	(1)	P15311, EZR, Ezrin	18/0/7

(2) P35241-4, RDX, Radixin

497 P00742, F10, Coagulation factor X — 18/0/1

498 (1) P03951-1, F11, Coagulation factor XI — 3/0/1
(2) P03951-2, F11, Coagulation factor XI

499 P00748, F12, Coagulation factor XII — 20/0/0

500 P00488, F13A1, Coagulation factor XIII A chain — 25/0/0

501 P05160, F13B, Coagulation factor XIII B chain — 18/0/0

502 P00734, F2, Prothrombin — 39/0/0

503 P12259, F5, Coagulation factor V — 34/0/0

504 P08709-1,2, F7, Coagulation factor VII — 9/0/0

505 (1) P00740-1, F9, Coagulation factor IX — 2/0/1
(2) P00740-2, F9, Coagulation factor IX

506 P07148, FABP1, Fatty acid-binding protein, liver — 2/0/1

507 P05413, FABP3, Fatty acid-binding protein, heart — 5/0/0

508 P15090, FABP4, Fatty acid-binding protein, adipocyte — 2/0/0

509 (1) Q01469, FABP5, Fatty acid-binding protein, epidermal — 5/0/0
(2) A8MUU1, FABP5P3, Putative fatty acid-binding protein 5-like protein 3

510 Q0Z7S8, FABP9, Fatty acid-binding protein 9 — 4/0/0

511 (1) Q8IWE2-1, FAM114A1, Protein NOXP20 — 1/0/0
(2) Q8IWE2-2, FAM114A1, Protein NOXP20

512 Q96TA1-1,2, FAM129B, Niban-like protein 1 — 7/0/0

513 Q6UWH4-1,2, FAM198B, Protein FAM198B — 1/0/1

514 (1) Q8IXL6-1, FAM20C, Extracellular serine/threonine protein kinase FAM20C — 3/0/0
(2) Q8IXL6-2, FAM20C, Extracellular serine/threonine protein kinase FAM20C

515 Q9NUQ9-1, FAM49B, Protein FAM49B — 4/0/0

516 Q9Y285-1,2, FARSA, Phenylalanine--tRNA ligase alpha subunit — 3/0/0

517 (1) Q9NSD9-1, FARSB, Phenylalanine--tRNA ligase beta subunit — 2/0/1
(2) Q9NSD9-2, FARSB, Phenylalanine--tRNA ligase beta subunit

518 P49327, FASN, Fatty acid synthase — 7/0/0

519	(1)	P23142-1, FBLN1, Fibulin-1	10/0/21
	(2)	P23142-2, FBLN1, Fibulin-1	
520		P23142-3, FBLN1, Fibulin-1	1/0/21
521		P23142-4, FBLN1, Fibulin-1	6/0/21
522	(1)	P98095-2, FBLN2, Fibulin-2	1/0/0
	(2)	P98095-1, FBLN2, Fibulin-2	
523		Q9UBX5, FBLN5, Fibulin-5	17/0/0
524	(1)	Q53RD9-1,2, FBLN7, Fibulin-7	0/3/0
	(2)	Q53RD9-3, FBLN7, Fibulin-7	
	(3)	Q53RD9-4, FBLN7, Fibulin-7	
525		P35555, FBN1, Fibrillin-1 [Cleaved into: Asprosin]	84/0/5
526	(1)	P35556-1, FBN2, Fibrillin-2 [Cleaved into: Fibrillin-2 C-terminal peptide]	11/0/6
	(2)	P35556-2, FBN2, Fibrillin-2 [Cleaved into: Fibrillin-2 C-terminal peptide]	
527		Q9UK22, FBXO2, F-box only protein 2	2/0/0
528		Q9Y6R7, FCGBP, IgGFc-binding protein	19/0/0
529		O00602, FCN1, Ficolin-1	4/0/1
530	(1)	Q15485-1, FCN2, Ficolin-2	1/0/1
	(2)	Q15485-2, FCN2, Ficolin-2	
531		O75636-1, FCN3, Ficolin-3	2/0/8
532		O75636-2, FCN3, Ficolin-3	0/0/9
533		Q96RD9-1,3, FCRL5, Fc receptor-like protein 5	1/0/0
534		Q96AC1-1-3, FERMT2, Fermitin family homolog 2	5/0/0
535	(1)	Q9UGM5-1, FETUB, Fetuin-B	2/0/0
	(2)	Q9UGM5-2, FETUB, Fetuin-B	
536	(1)	P02671-1, FGA, Fibrinogen alpha chain [Cleaved into: Fibrinopeptide A; Fibrinogen alpha chain]	5/0/1
	(2)	P02671-2, FGA, Fibrinogen alpha chain [Cleaved into: Fibrinopeptide A; Fibrinogen alpha chain]	
537		P02675, FGB, Fibrinogen beta chain [Cleaved into: Fibrinopeptide B; Fibrinogen beta chain]	42/0/0
538		P02679-1,2, FGG, Fibrinogen gamma chain	33/0/0
539		Q14314, FGL2, Fibroleukin	2/0/1

540 Q8TAL6, FIBIN, Fin bud initiation factor homolog

2/0/0 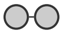

541 P62942, FKBP1A, Peptidyl-prolyl cis-trans isomerase FKBP1A

2/0/0 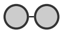

542 P20930, FLG, Filaggrin

38/0/0 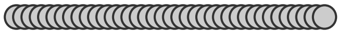

543 Q5D862, FLG2, Filaggrin-2

12/0/0 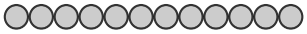

544 Q13045-1-3, FLII, Protein flightless-1 homolog

5/0/0 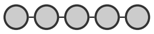

545 P21333-1,2, FLNA, Filamin-A

59/0/10 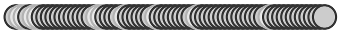

546 (1) O75369-1-3,6,8,9, FLNB, Filamin-B
(2) O75369-7, FLNB, Filamin-B
(3) O75369-4,5, FLNB, Filamin-B

0/7/6 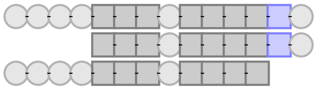

547 Q14315-1,2, FLNC, Filamin-C

39/0/9 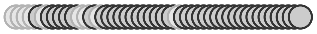

548 Q06828, FMOD, Fibromodulin

10/0/0 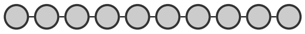

549 (1) P02751-1,3, FN1, Fibronectin
(2) P02751-8,14, FN1, Fibronectin
(3) P02751-7,15, FN1, Fibronectin
(4) P02751-2, FN1, Fibronectin
(5) P02751-4, FN1, Fibronectin
(6) P02751-5, FN1, Fibronectin
(7) P02751-6, FN1, Fibronectin
(8) P02751-9, FN1, Fibronectin
(9) P02751-10, FN1, Fibronectin
(10) P02751-11, FN1, Fibronectin
(11) P02751-12, FN1, Fibronectin
(12) P02751-13, FN1, Fibronectin
(13) P02751-16, FN1, Fibronectin
(14) P02751-17, FN1, Fibronectin

0/92/0 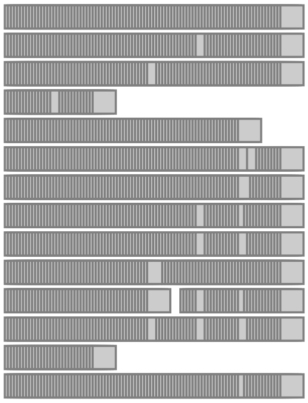

550 Q4ZHG4-1,2, FNDC1, Fibronectin type III domain-containing protein 1

3/0/0 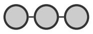

551 Q6PIV2-1, FOXR1, Forkhead box protein R1

2/0/1 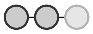

552 (1) Q70Z53-3, FRA10AC1, Protein FRA10AC1
(2) Q70Z53-1,2,4,5, FRA10AC1, Protein FRA10AC1

1/0/0 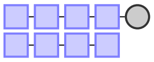

553 (1) Q86XX4-2, FRAS1, Extracellular matrix protein FRAS1 — 1/0/0
(2) Q86XX4-1,5, FRAS1, Extracellular matrix protein FRAS1

554 Q92765, FRZB, Secreted frizzled-related protein 3 — 2/0/0

555 Q16658, FSCN1, Fascin — 6/0/0

556 P19883-1,2, FST, Follistatin — 12/0/0

557 (1) Q12841-1, FSTL1, Follistatin-related protein 1 — 2/0/0
(2) Q12841-2, FSTL1, Follistatin-related protein 1

558 O95633-1,2, FSTL3, Follistatin-related protein 3 — 4/0/0

559 P02792, FTL, Ferritin light chain — 3/0/0

560 Q96AE4-1,2, FUBP1, Far upstream element-binding protein 1 — 4/0/3

561 Q96I24-1, FUBP3, Far upstream element-binding protein 3 — 1/0/1

562 Q9BTY2-1, FUCA2, Plasma alpha-L-fucosidase — 3/0/0

563 P35637-1,2, FUS, RNA-binding protein FUS — 7/0/0

564 Q13283-1, G3BP1, Ras GTPase-activating protein-binding protein 1 — 2/0/0

565 P11413-1-3, G6PD, Glucose-6-phosphate 1-dehydrogenase — 7/0/0

566 Q14376-1,2, GALE, UDP-glucose 4-epimerase — 2/0/0

567 P51570-1,2, GALK1, Galactokinase — 3/0/0

568 Q10471-1,2, GALNT2, Polypeptide N-acetylgalactosaminyltransferase 2 — 1/0/0

569 Q14697-1, GANAB, Neutral alpha-glucosidase AB — 1/0/11

570 Q14697-2, GANAB, Neutral alpha-glucosidase AB — 1/0/11

571 Q14697-3, GANAB, Neutral alpha-glucosidase AB — 1/0/1

572 (1) P04406-1, GAPDH, Glyceraldehyde-3-phosphate dehydrogenase — 2/0/0
(2) P04406-2, GAPDH, Glyceraldehyde-3-phosphate dehydrogenase

573 P41250, GARS, Glycine--tRNA ligase — 6/0/0

574 P54826, GAS1, Growth arrest-specific protein 1 — 9/0/0

575 (1) Q14393-1, GAS6, Growth arrest-specific protein 6 — 1/16/0
(2) Q14393-2, GAS6, Growth arrest-specific protein 6
(3) Q14393-3, GAS6, Growth arrest-specific protein 6
(4) Q14393-4, GAS6, Growth arrest-specific protein 6

(5) Q14393-5, GAS6, Growth arrest-specific protein 6

3/0/0

576 (1) P04062-1,2,4,5, GBA, Glucosylceramidase
(2) P04062-3, GBA, Glucosylceramidase

577 P32455, GBP1, Guanylate-binding protein 1 — 2/0/1

578 P32456, GBP2, Guanylate-binding protein 2 — 3/0/1

9/0/0

579 (1) P02774-1,3, GC, Vitamin D-binding protein
(2) P02774-2, GC, Vitamin D-binding protein

580 P01275, GCG, Glucagon [Cleaved into: Glicentin; Glicentin-related polypeptide — 5/0/0

581 Q92616, GCN1, eIF-2-alpha kinase activator GCN1 — 2/0/0

582 Q99988, GDF15, Growth/differentiation factor 15 — 2/0/0

583 Q7Z4P5, GDF7, Growth/differentiation factor 7 — 1/0/0

584 P31150, GDI1, Rab GDP dissociation inhibitor alpha — 2/0/2

0/0/2

585 (1) P50395-1, GDI2, Rab GDP dissociation inhibitor beta
(2) P50395-2, GDI2, Rab GDP dissociation inhibitor beta

586 P14136-1-3, GFAP, Glial fibrillary acidic protein — 0/0/4

1/0/0

587 (1) O75223-1, GGCT, Gamma-glutamylcyclotransferase
(2) O75223-3, GGCT, Gamma-glutamylcyclotransferase

588 Q92820, GGH, Gamma-glutamyl hydrolase — 2/0/0

589 Q9UBU3-1-5, GHRL, Appetite-regulating hormone — 1/0/0

590 O14908-1,2, GIPC1, PDZ domain-containing protein GIPC1 — 1/0/0

591 Q9NS71, GKN1, Gastrokine-1 — 7/0/0

592 P06280, GLA, Alpha-galactosidase A — 9/0/0

593 Q4G1C9-1,5,6, GLIPR1L2, GLIPR1-like protein 2 — 4/0/0

594 Q9NZD2, GLTP, Glycolipid transfer protein — 1/0/0

595 P15104, GLUL, Glutamine synthetase — 4/0/0

596 P17900, GM2A, Ganglioside GM2 activator — 7/0/0

597 Q9Y5P6-1,2, GMPPB, Mannose-1-phosphate guanyltransferase beta — 3/0/0

1/0/0

598 (1) P04899-6, GNAI2, Guanine nucleotide-binding protein G(i) subunit alpha-2
(2) P04899-1-5, GNAI2, Guanine nucleotide-binding protein G(i) subunit alpha-2

599 P62873-1,2, GNB1, Guanine nucleotide-binding protein G(I)/G(S)/G(T) subunit beta-1 — 2/0/1

600	(1)	P62879-1, GNB2, Guanine nucleotide-binding protein G(I)/G(S)/G(T) subunit beta-2	1/0/1
	·	Q9HAV0, GNB4, Guanine nucleotide-binding protein subunit beta-4	
	(2)	P62879-2, GNB2, Guanine nucleotide-binding protein G(I)/G(S)/G(T) subunit beta-2	
601		Q9UBI6, GNG12, Guanine nucleotide-binding protein G(I)/G(S)/G(O) subunit gamma-12	2/0/0
602		P36915-1,2, GNL1, Guanine nucleotide-binding protein-like 1	1/0/0
603		Q9UJJ9, GNPTG, N-acetylglucosamine-1-phosphotransferase subunit gamma	4/0/0
604		Q13439-1,3-5, GOLGA4, Golgin subfamily A member 4	1/0/1
605		Q8N9W4-1, GOLGA6L2, Golgin subfamily A member 6-like protein 2	6/0/0
606		H0YM25, GOLGA6L22, Golgin subfamily A member 6-like protein 22	1/0/8
607	(1)	A8MZA4, GOLGA6L6, Golgin subfamily A member 6-like protein 6	5/0/8
	(2)	Q8N7Z2, GOLGA6L1, Golgin subfamily A member 6-like protein 1	
608		Q14789-1-4, GOLGB1, Golgin subfamily B member 1	1/0/1
609		O00461, GOLIM4, Golgi integral membrane protein 4	9/0/0
610		Q8NBJ4-1,2, GOLM1, Golgi membrane protein 1	6/0/0
611		Q9H8Y8-1-3, GORASP2, Golgi reassembly-stacking protein 2	2/0/0
612		P00505-1, GOT2, Aspartate aminotransferase, mitochondrial	1/0/0
613		P40197, GP5, Platelet glycoprotein V	1/0/0
614		Q9Y625, GPC6, Glypican-6 [Cleaved into: Secreted glypican-6]	3/0/0
615		P06744-1,2, GPI, Glucose-6-phosphate isomerase	6/0/0
616		P80108-1, GPLD1, Phosphatidylinositol-glycan-specific phospholipase D	14/0/0
617		P22352, GPX3, Glutathione peroxidase 3	9/0/0
618		Q9UBQ7-1, GRHPR, Glyoxylate reductase/hydroxypyruvate reductase	3/0/0
619		Q9ULK0-1, GRID1, Glutamate receptor ionotropic, delta-1	0/0/2
620	(1)	P28799-1,3, GRN, Granulins	2/0/0
	(2)	P28799-2, GRN, Granulins	
621		Q96QA5, GSDMA, Gasdermin-A	7/0/0
622		P57764, GSDMD, Gasdermin-D	3/0/0

623 (1) P06396-1, GSN, Gelsolin — 2/0/43
(2) P06396-3,4, GSN, Gelsolin

624 P06396-2, GSN, Gelsolin — 1/0/43

625 P09488-1, GSTM1, Glutathione S-transferase Mu 1 — 0/0/3
Q03013-1, GSTM4, Glutathione S-transferase Mu 4

626 P28161-1, GSTM2, Glutathione S-transferase Mu 2 — 0/0/3

627 P21266, GSTM3, Glutathione S-transferase Mu 3 — 9/0/1

628 (1) P46439, GSTM5, Glutathione S-transferase Mu 5 — 1/0/3
(2) P09488-2, GSTM1, Glutathione S-transferase Mu 1
· Q03013-2, GSTM4, Glutathione S-transferase Mu 4
(3) P28161-2, GSTM2, Glutathione S-transferase Mu 2

629 (1) P78417-1, GSTO1, Glutathione S-transferase omega-1 — 0/1/0
(2) P78417-2, GSTO1, Glutathione S-transferase omega-1
(3) P78417-3, GSTO1, Glutathione S-transferase omega-1

630 P09211, GSTP1, Glutathione S-transferase P — 7/0/0

631 Q02747, GUCA2A, Guanylin — 2/0/0

632 Q92522, H1FX, Histone H1x — 2/0/0

633 (1) P0C0S8, HIST1H2AG, Histone H2A type 1 — 0/1/3
· Q16777, HIST2H2AC, Histone H2A type 2-C
· Q6FI13, HIST2H2AA3, Histone H2A type 2-A
· Q93077, HIST1H2AC, Histone H2A type 1-C
· Q96KK5, HIST1H2AH, Histone H2A type 1-H
· Q99878, HIST1H2AJ, Histone H2A type 1-J
· Q9BTM1-1, H2AFJ, Histone H2A.J
(2) P04908, HIST1H2AB, Histone H2A type 1-B/E
· P20671, HIST1H2AD, Histone H2A type 1-D
· Q7L7L0, HIST3H2A, Histone H2A type 3
(3) Q8IUE6, HIST2H2AB, Histone H2A type 2-B
(4) Q96QV6, HIST1H2AA, Histone H2A type 1-A
· Q9BTM1-2, H2AFJ, Histone H2A.J

634 (1) P0C0S5, H2AFZ, Histone H2A.Z — 0/0/2
· Q71UI9-1,2, H2AFV, Histone H2A.V
(2) Q71UI9-3,4, H2AFV, Histone H2A.V

635 P16104, H2AFX, Histone H2AX — 3/0/3

636 P84243, H3F3A, Histone H3.3 — 1/0/7

637 Q14520-1,2, HABP2, Hyaluronan-binding protein 2 — 19/0/0

638 Q6Y1H2, HACD2, Very-long-chain — 1/0/0

639 P40939-1, HADHA, Trifunctional enzyme subunit alpha, mitochondrial — 3/0/0

640		P42357-1-3, HAL, Histidine ammonia-lyase	2/0/0
641		P12081-1-4, HARS, Histidine--tRNA ligase, cytoplasmic	2/0/0
642		P69905, HBA1;, Hemoglobin subunit alpha	5/0/0
643	(1)	P68871, HBB, Hemoglobin subunit beta	4/0/0
	(2)	P02042, HBD, Hemoglobin subunit delta	
644		P51858-1-3, HDGF, Hepatoma-derived growth factor	8/0/0
645		Q00341-1,2, HDLBP, Vigilin	4/0/0
646		Q9NRV9, HEBP1, Heme-binding protein 1	1/0/0
647		O95714, HERC2, E3 ubiquitin-protein ligase HERC2	4/0/0
648	(1)	P06865-1, HEXA, Beta-hexosaminidase subunit alpha	6/0/0
	(2)	P06865-2, HEXA, Beta-hexosaminidase subunit alpha	
649		P07686, HEXB, Beta-hexosaminidase subunit beta	4/0/0
650		P49773, HINT1, Histidine triad nucleotide-binding protein 1	2/0/0
651		P16401, HIST1H1B, Histone H1.5	5/0/4
652		P16403, HIST1H1C, Histone H1.2	4/0/8
653		P16402, HIST1H1D, Histone H1.3	4/0/8
654	(1)	P10412, HIST1H1E, Histone H1.4	4/0/9
	(2)	P22492, HIST1H1T, Histone H1t	
	(3)	Q02539, HIST1H1A, Histone H1.1	
655	(1)	P06899, HIST1H2BJ, Histone H2B type 1-J	0/0/11
	·	P23527, HIST1H2BO, Histone H2B type 1-O	
	·	P33778, HIST1H2BB, Histone H2B type 1-B	
	·	Q16778, HIST2H2BE, Histone H2B type 2-E	
	(2)	Q6DN03, HIST2H2BC, Putative histone H2B type 2-C	
	·	Q6DRA6, HIST2H2BD, Putative histone H2B type 2-D	
656	(1)	O60814, HIST1H2BK, Histone H2B type 1-K	0/0/12
	·	P58876, HIST1H2BD, Histone H2B type 1-D	
	·	P62807, HIST1H2BC, Histone H2B type 1-C/E/F/G/I	
	·	Q5QNW6-1,2, HIST2H2BF, Histone H2B type 2-F	
	·	Q93079, HIST1H2BH, Histone H2B type 1-H	
	·	Q99877, HIST1H2BN, Histone H2B type 1-N	
	(2)	Q99879, HIST1H2BM, Histone H2B type 1-M	
	(3)	P57053, H2BFS, Histone H2B type F-S	
657	(1)	Q99880, HIST1H2BL, Histone H2B type 1-L	0/0/12
	(2)	Q96A08, HIST1H2BA, Histone H2B type 1-A	
658	(1)	P68431, HIST1H3A, Histone H3.1	1/0/7
	·	Q16695, HIST3H3, Histone H3.1t	
	·	Q71DI3, HIST2H3A;, Histone H3.2	
	(2)	Q6NXT2, H3F3C, Histone H3.3C	

659 P62805, HIST1H4A, Histone H4 — 6/0/0

660 Q8N257, HIST3H2BB, Histone H2B type 3-B — 0/0/10

661 P01891, HLA-A, HLA class I histocompatibility antigen, A-68 alpha chain — 0/0/6

662 (1) P04439, HLA-A, HLA class I histocompatibility antigen, A-3 alpha chain — 1/0/6

(2) P13747, HLA-E, HLA class I histocompatibility antigen, alpha chain E

(3) P13746-1,2, HLA-A, HLA class I histocompatibility antigen, A-11 alpha chain

(4) Q09160, HLA-A, HLA class I histocompatibility antigen, A-80 alpha chain

(5) P30443, HLA-A, HLA class I histocompatibility antigen, A-1 alpha chain

· P30455, HLA-A, HLA class I histocompatibility antigen, A-36 alpha chain

(6) P17693, HLA-G, HLA class I histocompatibility antigen, alpha chain G

663 P05534, HLA-A, HLA class I histocompatibility antigen, A-24 alpha chain — 0/0/4

P30447, HLA-A, HLA class I histocompatibility antigen, A-23 alpha chain

664 (1) P10316, HLA-A, HLA class I histocompatibility antigen, A-69 alpha chain — 0/0/7

(2) P01892, HLA-A, HLA class I histocompatibility antigen, A-2 alpha chain

665 P16188, HLA-A, HLA class I histocompatibility antigen, A-30 alpha chain — 0/0/6

666 (1) P30453, HLA-A, HLA class I histocompatibility antigen, A-34 alpha chain

(2) P18462, HLA-A, HLA class I histocompatibility antigen, A-25 alpha chain — 0/0/7

· P30450, HLA-A, HLA class I histocompatibility antigen, A-26 alpha chain

(3) P16190, HLA-A, HLA class I histocompatibility antigen, A-33 alpha chain

(4) P10314, HLA-A, HLA class I histocompatibility antigen, A-32 alpha chain

· P16189, HLA-A, HLA class I histocompatibility antigen, A-31 alpha chain

· P30459, HLA-A, HLA class I histocompatibility antigen, A-74 alpha chain

(5) P30456, HLA-A, HLA class I histocompatibility antigen, A-43 alpha chain

(6) P30457, HLA-A, HLA class I histocompatibility antigen, A-66 alpha chain

667 P30512, HLA-A, HLA class I histocompatibility antigen, A-29 alpha chain — 0/0/6

668 (1) P01889, HLA-B, HLA class I histocompatibility antigen, B-7 alpha chain — 0/0/8

	(2)	P03989, HLA-B, HLA class I histocompatibility antigen, B-27 alpha chain	
669		P18463, HLA-B, HLA class I histocompatibility antigen, B-37 alpha chain	0/0/6
670	(1)	P30460, HLA-B, HLA class I histocompatibility antigen, B-8 alpha chain	0/0/9
	(2)	P30498, HLA-B, HLA class I histocompatibility antigen, B-78 alpha chain	
671		P30462, HLA-B, HLA class I histocompatibility antigen, B-14 alpha chain	0/0/7
672		P30464, HLA-B, HLA class I histocompatibility antigen, B-15 alpha chain	0/0/8
673		P30466, HLA-B, HLA class I histocompatibility antigen, B-18 alpha chain	0/0/6
674	(1)	P30475, HLA-B, HLA class I histocompatibility antigen, B-39 alpha chain	0/0/8
	(2)	Q95365, HLA-B, HLA class I histocompatibility antigen, B-38 alpha chain	
675	(1)	P30479, HLA-B, HLA class I histocompatibility antigen, B-41 alpha chain	0/0/9
	(2)	P30481, HLA-B, HLA class I histocompatibility antigen, B-44 alpha chain	
676	(1)	P30480, HLA-B, HLA class I histocompatibility antigen, B-42 alpha chain	0/0/9
	(2)	Q29940, HLA-B, HLA class I histocompatibility antigen, B-59 alpha chain	
	(3)	P30495, HLA-B, HLA class I histocompatibility antigen, B-56 alpha chain	
	·	Q29718, HLA-B, HLA class I histocompatibility antigen, B-82 alpha chain	
	(4)	P30493, HLA-B, HLA class I histocompatibility antigen, B-55 alpha chain	
	(5)	P18464, HLA-B, HLA class I histocompatibility antigen, B-51 alpha chain	
	(6)	P18465, HLA-B, HLA class I histocompatibility antigen, B-57 alpha chain	
677		P30483, HLA-B, HLA class I histocompatibility antigen, B-45 alpha chain	0/0/5
		P30487, HLA-B, HLA class I histocompatibility antigen, B-49 alpha chain	
		P30488, HLA-B, HLA class I histocompatibility antigen, B-50 alpha chain	
678		P30484, HLA-B, HLA class I histocompatibility antigen, B-46 alpha chain	0/0/8
679		P30486, HLA-B, HLA class I histocompatibility antigen, B-48 alpha chain	0/0/7
680		P30490, HLA-B, HLA class I histocompatibility antigen, B-52 alpha chain	0/0/6
681	(1)	P30491, HLA-B, HLA class I histocompatibility antigen, B-53 alpha chain	0/0/5

· P30685, HLA-B, HLA class I histocompatibility antigen, B-35 alpha chain

(2) P10319, HLA-B, HLA class I histocompatibility antigen, B-58 alpha chain

682 P30492, HLA-B, HLA class I histocompatibility antigen, B-54 alpha chain — 0/0/6

683 (1) Q04826, HLA-B, HLA class I histocompatibility antigen, B-40 alpha chain — 0/0/8

(2) P30461, HLA-B, HLA class I histocompatibility antigen, B-13 alpha chain

· P30485, HLA-B, HLA class I histocompatibility antigen, B-47 alpha chain

684 Q29836, HLA-B, HLA class I histocompatibility antigen, B-67 alpha chain — 0/0/7

685 Q31610, HLA-B, HLA class I histocompatibility antigen, B-81 alpha chain — 0/0/7

686 Q31612, HLA-B, HLA class I histocompatibility antigen, B-73 alpha chain — 1/0/8

687 P04222, HLA-C, HLA class I histocompatibility antigen, Cw-3 alpha chain — 0/0/8

688 P10321, HLA-C, HLA class I histocompatibility antigen, Cw-7 alpha chain — 1/0/10

689 P30499, HLA-C, HLA class I histocompatibility antigen, Cw-1 alpha chain — 0/0/7

690 P30501, HLA-C, HLA class I histocompatibility antigen, Cw-2 alpha chain — 1/0/8

691 (1) P30508, HLA-C, HLA class I histocompatibility antigen, Cw-12 alpha chain — 0/0/10

· Q29963, HLA-C, HLA class I histocompatibility antigen, Cw-6 alpha chain

(2) Q29865, HLA-C, HLA class I histocompatibility antigen, Cw-18 alpha chain

(3) P30504, HLA-C, HLA class I histocompatibility antigen, Cw-4 alpha chain

692 P30510, HLA-C, HLA class I histocompatibility antigen, Cw-14 alpha chain — 0/0/8

693 (1) Q07000, HLA-C, HLA class I histocompatibility antigen, Cw-15 alpha chain — 0/0/8

(2) Q9TNN7, HLA-C, HLA class I histocompatibility antigen, Cw-5 alpha chain

(3) P30505, HLA-C, HLA class I histocompatibility antigen, Cw-8 alpha chain

694 Q29960-1,2, HLA-C, HLA class I histocompatibility antigen, Cw-16 alpha chain — 0/0/7

695 Q95604, HLA-C, HLA class I histocompatibility antigen, Cw-17 alpha chain — 0/0/8

696 P01893, HLA-H, Putative HLA class I histocompatibility antigen, alpha chain H — 0/0/4

697 Q96RW7-1,2, HMCN1, Hemicentin-1

24/0/1 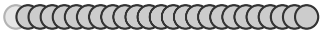

3/9/1 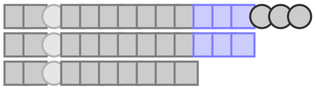

698 (1) Q8NDA2-1, HMCN2, Hemicentin-2

(2) Q8NDA2-2, HMCN2, Hemicentin-2

(3) Q8NDA2-4, HMCN2, Hemicentin-2

1/0/0 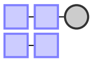

699 (1) P17096-1,2, HMGA1, High mobility group protein HMG-I/HMG-Y

(2) P17096-3, HMGA1, High mobility group protein HMG-I/HMG-Y

700 (1) P09429, HMGB1, High mobility group protein B1

0/0/1 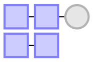

(2) B2RPK0, HMGB1P1, Putative high mobility group protein B1-like 1

701 O00479, HMGN4, High mobility group nucleosome-binding domain-containing protein 4

2/0/0 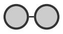

702 P50135-1, HNMT, Histamine N-methyltransferase

5/0/0 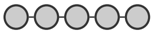

703 Q13151, HNRNPA0, Heterogeneous nuclear ribonucleoprotein A0

3/0/0 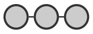

704 P09651-1-3, HNRNPA1, Heterogeneous nuclear ribonucleoprotein A1

1/0/3 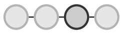

Q32P51, HNRNPA1L2, Heterogeneous nuclear ribonucleoprotein A1-like 2

705 P22626-1,2, HNRNPA2B1, Heterogeneous nuclear ribonucleoproteins A2/B1

9/0/1 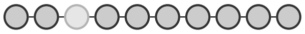

2/0/2 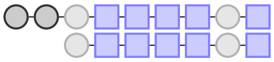

706 (1) P51991-1, HNRNPA3, Heterogeneous nuclear ribonucleoprotein A3

(2) P51991-2, HNRNPA3, Heterogeneous nuclear ribonucleoprotein A3

707 Q99729-1-4, HNRNPAB, Heterogeneous nuclear ribonucleoprotein A/B

3/0/2 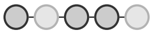

0/8/5 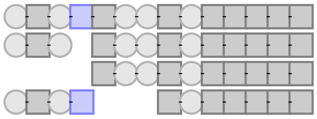

708 (1) P07910-2, HNRNPC, Heterogeneous nuclear ribonucleoproteins C1/C2

(2) P07910-1, HNRNPC, Heterogeneous nuclear ribonucleoproteins C1/C2

(3) P07910-3, HNRNPC, Heterogeneous nuclear ribonucleoproteins C1/C2

(4) P07910-4, HNRNPC, Heterogeneous nuclear ribonucleoproteins C1/C2

709 B2RXH8, HNRNPCL2, Heterogeneous nuclear ribonucleoprotein C-like 2

1/0/5 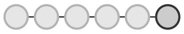

O60812, HNRNPCL1, Heterogeneous nuclear ribonucleoprotein C-like 1

710 B7ZW38, HNRNPCL3, Heterogeneous nuclear ribonucleoprotein C-like 3

1/0/5 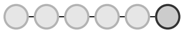

P0DMR1, HNRNPCL4, Heterogeneous nuclear ribonucleoprotein C-like 4

1/0/3 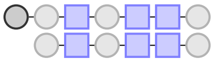

711 (1) Q14103-1,3, HNRNPD, Heterogeneous nuclear ribonucleoprotein D0

(2) Q14103-2,4, HNRNPD, Heterogeneous nuclear ribonucleoprotein D0

1/0/2 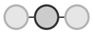

712 O14979-1-3, HNRNPDL, Heterogeneous nuclear ribonucleoprotein D-like

3/0/3 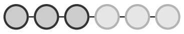

713 P52597, HNRNPF, Heterogeneous nuclear ribonucleoprotein F

1/0/4 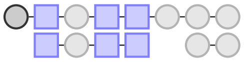

714 (1) P31943, HNRNPH1, Heterogeneous nuclear ribonucleoprotein H

(2) P55795, HNRNPH2, Heterogeneous nuclear ribonucleoprotein H2

0/0/1 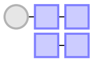

715 (1) P31942-1,2, HNRNPH3, Heterogeneous nuclear ribonucleoprotein H3

(2) P31942-3-6, HNRNPH3, Heterogeneous nuclear ribonucleoprotein H3

1/20/0 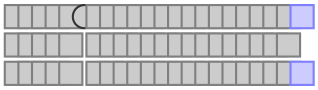

716 (1) P61978-3, HNRNPK, Heterogeneous nuclear ribonucleoprotein K

(2) P61978-1, HNRNPK, Heterogeneous nuclear ribonucleoprotein K

(3) P61978-2, HNRNPK, Heterogeneous nuclear ribonucleoprotein K

1/0/0 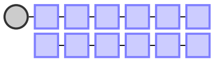

717 (1) P14866-1, HNRNPL, Heterogeneous nuclear ribonucleoprotein L

(2) P14866-2, HNRNPL, Heterogeneous nuclear ribonucleoprotein L

11/0/0 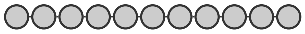

718 P52272-1,2, HNRNPM, Heterogeneous nuclear ribonucleoprotein M

4/0/6 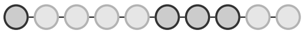

719 O43390-1-4, HNRNPR, Heterogeneous nuclear ribonucleoprotein R

1/0/14 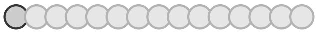

720 Q00839-1, HNRNPU, Heterogeneous nuclear ribonucleoprotein U

1/0/14 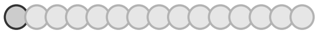

721 Q00839-2, HNRNPU, Heterogeneous nuclear ribonucleoprotein U

2/0/0 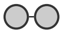

722 Q9BUJ2-1-4, HNRNPUL1, Heterogeneous nuclear ribonucleoprotein U-like protein 1

8/0/0 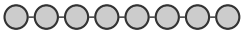

723 Q1KMD3, HNRNPUL2, Heterogeneous nuclear ribonucleoprotein U-like protein 2

1/0/14 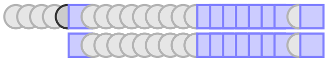

724 (1) P00738-1, HP, Haptoglobin

(2) P00738-2, HP, Haptoglobin

2/0/0 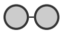

725 Q5SSJ5-1,2, HP1BP3, Heterochromatin protein 1-binding protein 3

726		P00739-1,2, HPR, Haptoglobin-related protein	9/0/14
727		P02790, HPX, Hemopexin	16/0/0
728		P23327, HRC, Sarcoplasmic reticulum histidine-rich calcium-binding protein	5/0/0
729		P04196, HRG, Histidine-rich glycoprotein	24/0/0
730		Q86YZ3, HRNR, Hornerin	5/0/0
731	(1)	P51659-2, HSD17B4, Peroxisomal multifunctional enzyme type 2	1/0/0
	(2)	P51659-1,3, HSD17B4, Peroxisomal multifunctional enzyme type 2	
732	(1)	P07900-1,2, HSP90AA1, Heat shock protein HSP 90-alpha	10/1/13
	(2)	Q58FF6, HSP90AB4P, Putative heat shock protein HSP 90-beta 4	
	(3)	Q58FG0, HSP90AA5P, Putative heat shock protein HSP 90-alpha A5	
	(4)	Q58FG1, HSP90AA4P, Putative heat shock protein HSP 90-alpha A4	
	(5)	Q14568, HSP90AA2P, Heat shock protein HSP 90-alpha A2	
	(6)	Q12931-1,2, TRAP1, Heat shock protein 75 kDa, mitochondrial	
733	(1)	P08238, HSP90AB1, Heat shock protein HSP 90-beta	10/0/20
	(2)	Q58FF8, HSP90AB2P, Putative heat shock protein HSP 90-beta 2	
734		Q58FF7, HSP90AB3P, Putative heat shock protein HSP 90-beta-3	1/0/12
735	(1)	P14625, HSP90B1, Endoplasmin	23/0/1
	(2)	Q58FF3, HSP90B2P, Putative endoplasmin-like protein	
736		P48723, HSPA13, Heat shock 70 kDa protein 13	2/0/0
737	(1)	P0DMV8-1, HSPA1A, Heat shock 70 kDa protein 1A	2/3/12
	·	P0DMV9, HSPA1B, Heat shock 70 kDa protein 1B	
	(2)	P48741, HSPA7, Putative heat shock 70 kDa protein 7	
	(3)	P17066, HSPA6, Heat shock 70 kDa protein 6	
	(4)	P0DMV8-2, HSPA1A, Heat shock 70 kDa protein 1A	
738		P34931, HSPA1L, Heat shock 70 kDa protein 1-like	0/0/13
739		P54652, HSPA2, Heat shock-related 70 kDa protein 2	7/0/10
740		P34932-1, HSPA4, Heat shock 70 kDa protein 4	5/0/0
741		P11021, HSPA5, 78 kDa glucose-regulated protein	29/0/3
742	(1)	P11142-1, HSPA8, Heat shock cognate 71 kDa protein	6/0/10
	(2)	P11142-2, HSPA8, Heat shock cognate 71 kDa protein	
743		P38646, HSPA9, Stress-70 protein, mitochondrial	8/0/1
744		P04792, HSPB1, Heat shock protein beta-1	9/0/0

745		O14558, HSPB6, Heat shock protein beta-6	3/0/0
746		P10809-1, HSPD1, 60 kDa heat shock protein, mitochondrial	5/0/0
747		P98160, HSPG2, Basement membrane-specific heparan sulfate proteoglycan core protein	81/0/0
748		Q92743, HTRA1, Serine protease HTRA1	18/0/1
749		P83110-1,2, HTRA3, Serine protease HTRA3	3/0/0
750		Q7Z6Z7-1-3, HUWE1, E3 ubiquitin-protein ligase HUWE1	3/0/0
751	(1)	Q12794-1-3, HYAL1, Hyaluronidase-1	4/0/0
	(2)	Q12794-7, HYAL1, Hyaluronidase-1	
752	(1)	Q9Y4L1-1, HYOU1, Hypoxia up-regulated protein 1	5/0/0
	(2)	Q9Y4L1-2, HYOU1, Hypoxia up-regulated protein 1	
753	(1)	P14735-1, IDE, Insulin-degrading enzyme	7/0/0
	(2)	P14735-2, IDE, Insulin-degrading enzyme	
754		P13284, IFI30, Gamma-interferon-inducible lysosomal thiol reductase	4/0/0
755		Q96RY7-1, IFT140, Intraflagellar transport protein 140 homolog	4/0/0
756	(1)	P05019-1, IGF1, Insulin-like growth factor I	2/3/0
	(2)	P05019-2, IGF1, Insulin-like growth factor I	
	(3)	P05019-3, IGF1, Insulin-like growth factor I	
	(4)	P05019-4, IGF1, Insulin-like growth factor I	
757		P08069, IGF1R, Insulin-like growth factor 1 receptor	6/0/0
758		P01344-1-3, IGF2, Insulin-like growth factor II	5/0/0
759		P11717, IGF2R, Cation-independent mannose-6-phosphate receptor	10/0/0
760		P35858-1,2, IGFALS, Insulin-like growth factor-binding protein complex acid labile subunit	15/0/0
761		P08833, IGFBP1, Insulin-like growth factor-binding protein 1	8/0/0
762		P18065, IGFBP2, Insulin-like growth factor-binding protein 2	10/0/0
763		P17936-1,2, IGFBP3, Insulin-like growth factor-binding protein 3	17/0/1
764	(1)	P22692-1, IGFBP4, Insulin-like growth factor-binding protein 4	1/0/1
	(2)	P22692-2, IGFBP4, Insulin-like growth factor-binding protein 4	

765		P24593, IGFBP5, Insulin-like growth factor-binding protein 5	14/0/0
766		P24592, IGFBP6, Insulin-like growth factor-binding protein 6	7/0/0
767		Q16270-1,2, IGFBP7, Insulin-like growth factor-binding protein 7	11/0/0
768		P01876, IGHA1, Ig alpha-1 chain C region	5/0/8
769		P01877, IGHA2, Ig alpha-2 chain C region	1/0/8
770		P01880-1,2, IGHD, Ig delta chain C region	9/0/0
771		P01857, IGHG1, Ig gamma-1 chain C region	7/0/9
772		P01859, IGHG2, Ig gamma-2 chain C region	4/0/10
773		P01860, IGHG3, Ig gamma-3 chain C region	8/0/12
774		P01861, IGHG4, Ig gamma-4 chain C region	4/0/7
775		P01871-1,2, IGHM, Ig mu chain C region	9/0/12
776		A0A0C4DH31, NA, NA	1/0/1
		P23083, IGHV1-2, Immunoglobulin heavy variable 1-2	
777		P01742, IGHV1-69, Immunoglobulin heavy variable 1-69	2/0/1
778		P01766, IGHV3-13, Immunoglobulin heavy variable 3-13	1/0/0
779	(1)	P01768, IGHV3-30, Immunoglobulin heavy variable 3-30	0/0/1
	(2)	P01764, IGHV3-23, Immunoglobulin heavy variable 3-23	
780		P01772, IGHV3-33, Immunoglobulin heavy variable 3-33	0/0/2
781	(1)	P01780, IGHV3-7, Immunoglobulin heavy variable 3-7	2/0/1
	(2)	A0A0B4J1V1, NA, NA	
	·	P01762, IGHV3-11, Immunoglobulin heavy variable 3-11	
	·	P01763, IGHV3-48, Immunoglobulin heavy variable 3-48	
	·	P01767, IGHV3-53, Immunoglobulin heavy variable 3-53	
782		P01782, IGHV3-9, Immunoglobulin heavy variable 3-9	1/0/0
783	(1)	P01824, IGHV4-39, Immunoglobulin heavy variable 4-39	1/0/0
	(2)	P01825, IGHV4-59, Immunoglobulin heavy variable 4-59	
	·	P06331, IGHV4-34, Immunoglobulin heavy variable 4-34	
784		P01834, IGKC, Ig kappa chain C region	6/0/0
785		P01599, IGKV1-17, Immunoglobulin kappa variable 1-17	2/0/1
786		P01593, IGKV1D-33, Immunoglobulin kappa variable 1D-33	1/0/0
		P01594, IGKV1-33, Immunoglobulin kappa variable 1-33	
787		A0A0C4DH73, NA, NA	1/0/1
		P01597, IGKV1-39, Immunoglobulin kappa variable 1-39	
		P01611, IGKV1D-12, Immunoglobulin kappa variable 1D-12	
		P04432, IGKV1D-39, Immunoglobulin kappa variable 1D-39	
788		P01602, IGKV1-5, Immunoglobulin kappa variable 1-5	1/0/1

789		P06310, IGKV2-30, Immunoglobulin kappa variable 2-30	1/0/1
790	(1)	P01614, IGKV2D-40, Immunoglobulin kappa variable 2D-40	1/0/1
	(2)	A0A075B6P5, NA, NA	
	·	P01615, IGKV2D-28, Immunoglobulin kappa variable 2D-28	
	(3)	A0A075B6S6, NA, NA	
791		P04433, IGKV3-11, Immunoglobulin kappa variable 3-11	1/0/2
792		P01624, IGKV3-15, Immunoglobulin kappa variable 3-15	2/0/2
793		P01619, IGKV3-20, Immunoglobulin kappa variable 3-20	1/0/3
794		P06312, IGKV4-1, Immunoglobulin kappa variable 4-1	5/0/0
795	(1)	P0CG05, IGLC2, Ig lambda-2 chain C regions	2/0/4
	(2)	P0CG06, IGLC3, Ig lambda-3 chain C regions	
796		P0CF74, IGLC6, Ig lambda-6 chain C region	0/0/4
797		A0M8Q6, IGLC7, Ig lambda-7 chain C region	1/0/4
798		P15814-1, IGLL1, Immunoglobulin lambda-like polypeptide 1	1/0/1
799	(1)	B9A064-1, IGLL5, Immunoglobulin lambda-like polypeptide 5	1/0/4
	(2)	P0CG04, IGLC1, Ig lambda-1 chain C regions	
800		P01703, IGLV1-40, Immunoglobulin lambda variable 1-40	1/0/0
801	(1)	P01700, IGLV1-47, Immunoglobulin lambda variable 1-47	1/0/0
	(2)	P01699, IGLV1-44, Immunoglobulin lambda variable 1-44	
802		P01705, IGLV2-23, Immunoglobulin lambda variable 2-23	2/0/0
803		P01714, IGLV3-19, Immunoglobulin lambda variable 3-19	2/0/0
804		P80748, IGLV3-21, Immunoglobulin lambda variable 3-21	2/0/0
805		P01717, IGLV3-25, Immunoglobulin lambda variable 3-25	1/0/1
806		P01718, IGLV3-27, Immunoglobulin lambda variable 3-27	1/0/1
807		P04211, IGLV7-43, Immunoglobulin lambda variable 7-43	3/0/0
808		Q969P0-1,3, IGSF8, Immunoglobulin superfamily member 8	1/0/0
809		Q70UQ0-4, IKBIP, Inhibitor of nuclear factor kappa-B kinase-interacting protein	2/0/0
810		Q9NZH8-1,2, IL36G, Interleukin-36 gamma	3/0/0
811	(1)	Q12906-4, ILF3, Interleukin enhancer-binding factor 3	1/0/0
	(2)	Q12906-1-3,5-7, ILF3, Interleukin enhancer-binding factor 3	
812		Q13418-1-3, ILK, Integrin-linked protein kinase	1/0/0
813		Q9NX62, IMPAD1, Inositol monophosphatase 3	7/0/0
814		P08476, INHBA, Inhibin beta A chain	4/0/0

815 P09529, INHBB, Inhibin beta B chain — 5/0/0

816 P55103, INHBC, Inhibin beta C chain — 8/0/0

817 P58166, INHBE, Inhibin beta E chain — 3/0/0

818 (1) O00410-1,3, IPO5, Importin-5 — 3/0/0

(2) O00410-2, IPO5, Importin-5

819 O95373, IPO7, Importin-7 — 1/0/0

820 P46940, IQGAP1, Ras GTPase-activating-like protein IQGAP1 — 28/0/0

821 O14498, ISLR, Immunoglobulin superfamily containing leucine-rich repeat protein — 7/0/0

822 P11215-1,2, ITGAM, Integrin alpha-M — 3/0/0

823 (1) P05556-1, ITGB1, Integrin beta-1 — 1/0/0

(2) P05556-2-5, ITGB1, Integrin beta-1

824 P05107, ITGB2, Integrin beta-2 — 3/0/0

825 P26012-1,2, ITGB8, Integrin beta-8 — 5/0/0

826 (1) O95965-1, ITGBL1, Integrin beta-like protein 1 — 1/14/0

(2) O95965-2, ITGBL1, Integrin beta-like protein 1

(3) O95965-3, ITGBL1, Integrin beta-like protein 1

827 (1) P19827-1, ITIH1, Inter-alpha-trypsin inhibitor heavy chain H1 — 10/10/1

(2) P19827-2, ITIH1, Inter-alpha-trypsin inhibitor heavy chain H1

(3) P19827-3, ITIH1, Inter-alpha-trypsin inhibitor heavy chain H1

828 P19823, ITIH2, Inter-alpha-trypsin inhibitor heavy chain H2 — 32/0/0

829 Q06033-1,2, ITIH3, Inter-alpha-trypsin inhibitor heavy chain H3 — 35/0/0

830 Q14624-1, ITIH4, Inter-alpha-trypsin inhibitor heavy chain H4 — 4/0/35

831 (1) Q14624-2,3, ITIH4, Inter-alpha-trypsin inhibitor heavy chain H4 — 0/0/35

(2) Q14624-4, ITIH4, Inter-alpha-trypsin inhibitor heavy chain H4

832 (1) Q86UX2-1, ITIH5, Inter-alpha-trypsin inhibitor heavy chain H5 — 0/8/0

	(2)	Q86UX2-2, ITIH5, Inter-alpha-trypsin inhibitor heavy chain H5	
	(3)	Q86UX2-3, ITIH5, Inter-alpha-trypsin inhibitor heavy chain H5	
	(4)	Q86UX2-4, ITIH5, Inter-alpha-trypsin inhibitor heavy chain H5	
833	(1)	Q8WWA0, ITLN1, Intelectin-1	9/0/0
	(2)	Q8WWU7-1,2, ITLN2, Intelectin-2	
834		O43736-1,2, ITM2A, Integral membrane protein 2A	2/0/0
835		Q9Y287-1,2, ITM2B, Integral membrane protein 2B	2/0/0
836		Q9BY32-1, ITPA, Inosine triphosphate pyrophosphatase	1/0/0
837		P07476, IVL, Involucrin	13/0/0
838		P01591, JCHAIN, Immunoglobulin J chain	5/0/0
839		P14923, JUP, Junction plakoglobin	19/0/3
840		Q96I82-1,2, KAZALD1, Kazal-type serine protease inhibitor domain-containing protein 1	1/0/0
841	(1)	Q96CX2, KCTD12, BTB/POZ domain-containing protein KCTD12	9/0/0
	(2)	Q6ZWB6, KCTD8, BTB/POZ domain-containing protein KCTD8	
842		Q9Y4C1, KDM3A, Lysine-specific demethylase 3A	4/0/0
843	(1)	Q07666-1,2, KHDRBS1, KH domain-containing, RNA-binding, signal transduction-associated protein 1	1/0/0
	(2)	Q07666-3, KHDRBS1, KH domain-containing, RNA-binding, signal transduction-associated protein 1	
844		Q92945, KHSRP, Far upstream element-binding protein 2	2/0/3
845		Q8IZA0-1,2, KIAA0319L, Dyslexia-associated protein KIAA0319-like protein	2/0/0
846		O94854-1,2, KIAA0754, Uncharacterized protein KIAA0754	2/0/0
847		Q9NS87-1,2,4, KIF15, Kinesin-like protein KIF15	1/0/1
848		P33176, KIF5B, Kinesin-1 heavy chain	5/0/3
849		O60282-1, KIF5C, Kinesin heavy chain isoform 5C	1/0/3
850		Q07866-1-10, KLC1, Kinesin light chain 1	3/0/0
851		O43240, KLK10, Kallikrein-10	4/0/0
852	(1)	Q92876-1, KLK6, Kallikrein-6	2/0/0
	(2)	Q92876-2, KLK6, Kallikrein-6	
853		P49862-1,2, KLK7, Kallikrein-7	2/0/0
854		P03952, KLKB1, Plasma kallikrein	35/0/1

855		Q03164-1-3, KMT2A, Histone-lysine N-methyltransferase 2A	7/0/0
856		P01042-1, KNG1, Kininogen-1	10/0/24
857	(1)	P01042-2, KNG1, Kininogen-1	0/0/24
	(2)	P01042-3, KNG1, Kininogen-1	
858	(1)	Q14974-1, KPNB1, Importin subunit beta-1	4/0/0
	(2)	Q14974-2, KPNB1, Importin subunit beta-1	
859		Q5T749, KPRP, Keratinocyte proline-rich protein	10/0/0
860		P04264, KRT1, Keratin, type II cytoskeletal 1	39/0/9
861		P13645, KRT10, Keratin, type I cytoskeletal 10	27/0/10
862	(1)	P13646-1, KRT13, Keratin, type I cytoskeletal 13	1/0/13
	(2)	P13646-2, KRT13, Keratin, type I cytoskeletal 13	
	(3)	P13646-3, KRT13, Keratin, type I cytoskeletal 13	
863		P02533, KRT14, Keratin, type I cytoskeletal 14	9/0/27
864	(1)	P19012-1, KRT15, Keratin, type I cytoskeletal 15	1/0/13
	(2)	P19012-2, KRT15, Keratin, type I cytoskeletal 15	
865	(1)	P08779, KRT16, Keratin, type I cytoskeletal 16	22/0/18
	(2)	Q8N1A0-1,2, KRT222, Keratin-like protein KRT222	
866		Q04695, KRT17, Keratin, type I cytoskeletal 17	15/0/20
867		P05783, KRT18, Keratin, type I cytoskeletal 18	24/0/3
868	(1)	P08727, KRT19, Keratin, type I cytoskeletal 19	21/0/14
	(2)	Q99456, KRT12, Keratin, type I cytoskeletal 12	
869		P35908, KRT2, Keratin, type II cytoskeletal 2 epidermal	24/0/10
870		P35900, KRT20, Keratin, type I cytoskeletal 20	0/0/6
871	(1)	Q9C075-1, KRT23, Keratin, type I cytoskeletal 23	1/0/3
	(2)	Q9C075-2, KRT23, Keratin, type I cytoskeletal 23	
872		Q2M2I5, KRT24, Keratin, type I cytoskeletal 24	1/0/4
873	(1)	Q7Z3Z0, KRT25, Keratin, type I cytoskeletal 25	3/0/5
	(2)	Q7Z3Y9, KRT26, Keratin, type I cytoskeletal 26	
874		Q7Z3Y8, KRT27, Keratin, type I cytoskeletal 27	1/0/7
875		Q7Z3Y7, KRT28, Keratin, type I cytoskeletal 28	2/0/8
876		P12035, KRT3, Keratin, type II cytoskeletal 3	0/0/10
877		Q15323, KRT31, Keratin, type I cuticular Ha1	2/0/16
878		Q14532, KRT32, Keratin, type I cuticular Ha2	2/0/7

879		O76009, KRT33A, Keratin, type I cuticular Ha3-I	1/0/15
880		Q14525, KRT33B, Keratin, type I cuticular Ha3-II	1/0/10
881		O76011, KRT34, Keratin, type I cuticular Ha4	1/0/10
882		Q92764, KRT35, Keratin, type I cuticular Ha5	8/0/11
883		O76013-1,2, KRT36, Keratin, type I cuticular Ha6	3/0/9
884		O76014, KRT37, Keratin, type I cuticular Ha7	0/0/4
885		O76015, KRT38, Keratin, type I cuticular Ha8	1/0/4
886		Q6A163, KRT39, Keratin, type I cytoskeletal 39	1/0/1
887		P19013, KRT4, Keratin, type II cytoskeletal 4	0/0/8
888		Q6A162, KRT40, Keratin, type I cytoskeletal 40	1/0/4
889		P13647, KRT5, Keratin, type II cytoskeletal 5	21/0/22
890		P02538, KRT6A, Keratin, type II cytoskeletal 6A	2/0/46
891		P04259, KRT6B, Keratin, type II cytoskeletal 6B	1/0/45
892		P48668, KRT6C, Keratin, type II cytoskeletal 6C	0/0/46
893		P08729, KRT7, Keratin, type II cytoskeletal 7	23/0/10
894	(1)	Q3SY84, KRT71, Keratin, type II cytoskeletal 71	5/0/6
	(2)	Q86Y46-2, KRT73, Keratin, type II cytoskeletal 73	
895		Q14CN4-1-3, KRT72, Keratin, type II cytoskeletal 72	0/0/6
896		Q7RTS7, KRT74, Keratin, type II cytoskeletal 74	0/0/8
		Q86Y46-1, KRT73, Keratin, type II cytoskeletal 73	
897		O95678, KRT75, Keratin, type II cytoskeletal 75	0/0/19
898		Q01546, KRT76, Keratin, type II cytoskeletal 2 oral	0/0/11
899		Q7Z794, KRT77, Keratin, type II cytoskeletal 1b	1/0/5
900	(1)	Q8N1N4-1, KRT78, Keratin, type II cytoskeletal 78	4/0/4
	(2)	Q8N1N4-2, KRT78, Keratin, type II cytoskeletal 78	
901		Q5XKE5, KRT79, Keratin, type II cytoskeletal 79	1/0/9
902		P05787-1,2, KRT8, Keratin, type II cytoskeletal 8	25/0/13
903	(1)	Q6KB66-1,2, KRT80, Keratin, type II cytoskeletal 80	2/0/1
	(2)	Q6KB66-3, KRT80, Keratin, type II cytoskeletal 80	
904		Q9NSB4, KRT82, Keratin, type II cuticular Hb2	2/0/6
905	(1)	P78385, KRT83, Keratin, type II cuticular Hb3	0/0/29
	(2)	A6NCN2, KRT87P, Putative keratin-87 protein	
906		Q9NSB2, KRT84, Keratin, type II cuticular Hb4	1/0/4

907		P78386, KRT85, Keratin, type II cuticular Hb5	5/0/23
908	(1)	O43790, KRT86, Keratin, type II cuticular Hb6	0/0/27
	(2)	Q14533, KRT81, Keratin, type II cuticular Hb1	
909		P35527, KRT9, Keratin, type I cytoskeletal 9	28/0/2
910		Q8IUC1, KRTAP11-1, Keratin-associated protein 11-1	1/0/0
911		Q86UP2-1-4, KTN1, Kinectin	2/0/0
912		P24043, LAMA2, Laminin subunit alpha-2	28/0/0
913		Q16363-1,2, LAMA4, Laminin subunit alpha-4	35/0/0
914		O15230-1, LAMA5, Laminin subunit alpha-5	10/0/0
915		P07942, LAMB1, Laminin subunit beta-1	32/0/2
916		P55268, LAMB2, Laminin subunit beta-2	28/0/0
917		Q13751, LAMB3, Laminin subunit beta-3	3/0/1
918		P11047, LAMC1, Laminin subunit gamma-1	42/0/0
919		Q13753-1,2, LAMC2, Laminin subunit gamma-2	7/0/0
920		P11279-1, LAMP1, Lysosome-associated membrane glycoprotein 1	2/0/0
921		P28838-1,2, LAP3, Cytosol aminopeptidase	3/0/0
922	(1)	Q14847-1, LASP1, LIM and SH3 domain protein 1	0/1/0
	(2)	Q14847-2, LASP1, LIM and SH3 domain protein 1	
	(3)	Q14847-3, LASP1, LIM and SH3 domain protein 1	
923		P18428, LBP, Lipopolysaccharide-binding protein	14/0/1
924	(1)	P04180, LCAT, Phosphatidylcholine-sterol acyltransferase	9/0/0
	(2)	Q8NCC3-1, PLA2G15, Group XV phospholipase A2	
925		P31025, LCN1, Lipocalin-1	2/0/0
		Q5VSP4, LCN1P1, Putative lipocalin 1-like protein 1	
926	(1)	P13796-1, LCP1, Plastin-2	18/0/15
	(2)	Q14651, PLS1, Plastin-1	
927		P13796-2, LCP1, Plastin-2	1/0/8
928	(1)	P00338-1,3, LDHA, L-lactate dehydrogenase A chain	0/6/0
	(2)	P07195, LDHB, L-lactate dehydrogenase B chain	
	(3)	P07864, LDHC, L-lactate dehydrogenase C chain	
	·	Q6ZMR3, LDHAL6A, L-lactate dehydrogenase A-like 6A	
	(4)	P00338-2,5, LDHA, L-lactate dehydrogenase A chain	
	(5)	P00338-4, LDHA, L-lactate dehydrogenase A chain	
929	(1)	P01130-1,5, LDLR, Low-density lipoprotein receptor	0/3/0
	(2)	P01130-2,3, LDLR, Low-density lipoprotein receptor	

	(3)	P01130-4, LDLR, Low-density lipoprotein receptor	
930		O14960, LECT2, Leukocyte cell-derived chemotaxin-2	6/0/0
931		P41159, LEP, Leptin	6/0/0
932		Q8NES3-1-4, LFNG, Beta-1,3-N-acetylglucosaminyltransferase lunatic fringe	4/0/0
933		P09382, LGALS1, Galectin-1	8/0/0
934		P17931, LGALS3, Galectin-3	9/0/0
935		Q08380, LGALS3BP, Galectin-3-binding protein	18/0/0
936		P47929, LGALS7, Galectin-7	9/0/0
937		Q8N0V4, LGI2, Leucine-rich repeat LGI family member 2	3/0/0
938		O75019-1,2, LILRA1, Leukocyte immunoglobulin-like receptor subfamily A member 1	1/0/1
939		P11150, LIPC, Hepatic triacylglycerol lipase	3/0/0
940		Q9Y5X9-1,2, LIPG, Endothelial lipase	1/0/1
941		P49257, LMAN1, Protein ERGIC-53	6/0/1
942		Q12907, LMAN2, Vesicular integral-membrane protein VIP36	2/0/0
943		Q68DH5, LMBRD2, LMBR1 domain-containing protein 2	3/0/0
944	(1)	P02545-1-3,6, LMNA, Prelamin-A/C [Cleaved into: Lamin-A/C	9/0/20
	(2)	P02545-4, LMNA, Prelamin-A/C [Cleaved into: Lamin-A/C	
945		P02545-5, LMNA, Prelamin-A/C [Cleaved into: Lamin-A/C	1/0/20
946		P20700, LMNB1, Lamin-B1	9/0/2
947		Q03252, LMNB2, Lamin-B2	1/0/3
948		P28300, LOX, Protein-lysine 6-oxidase	10/0/1
949		Q08397, LOXL1, Lysyl oxidase homolog 1	18/0/1
950		Q9Y4K0, LOXL2, Lysyl oxidase homolog 2	13/0/1
951	(1)	P58215-1, LOXL3, Lysyl oxidase homolog 3	1/0/1
	(2)	P58215-3, LOXL3, Lysyl oxidase homolog 3	
952		Q96JB6, LOXL4, Lysyl oxidase homolog 4	1/0/1
953		P08519, LPA, Apolipoprotein(a)	29/0/0
954		Q9UBY5, LPAR3, Lysophosphatidic acid receptor 3	5/0/0
955		P06858, LPL, Lipoprotein lipase	10/0/1
956		Q93052, LPP, Lipoma-preferred partner	1/0/1

957 P02750, LRG1, Leucine-rich alpha-2-glycoprotein — 4/0/0

958 Q07954-1, LRP1, Prolow-density lipoprotein receptor-related protein 1 — 44/0/2

959 Q9NZR2, LRP1B, Low-density lipoprotein receptor-related protein 1B — 26/0/2

960 (1) Q14114-1,3, LRP8, Low-density lipoprotein receptor-related protein 8 — 2/0/1
(2) Q14114-2,4, LRP8, Low-density lipoprotein receptor-related protein 8

961 Q8TF66-1,2, LRRC15, Leucine-rich repeat-containing protein 15 — 1/0/0

962 (1) Q8N6Y2-1, LRRC17, Leucine-rich repeat-containing protein 17 — 1/0/0
(2) Q8N6Y2-2, LRRC17, Leucine-rich repeat-containing protein 17

963 Q8N9N7, LRRC57, Leucine-rich repeat-containing protein 57 — 2/0/0

964 Q9Y608-1, LRRFIP2, Leucine-rich repeat flightless-interacting protein 2 — 2/0/0

965 P33241-1,3, LSP1, Lymphocyte-specific protein 1 — 1/0/0

966 (1) Q86X29-1,4, LSR, Lipolysis-stimulated lipoprotein receptor — 1/0/7
(2) Q86X29-2, LSR, Lipolysis-stimulated lipoprotein receptor
(3) Q86X29-5,6, LSR, Lipolysis-stimulated lipoprotein receptor

967 Q86X29-3, LSR, Lipolysis-stimulated lipoprotein receptor — 1/0/7

968 (1) Q14766-1,4, LTBP1, Latent-transforming growth factor beta-binding protein 1 — 2/0/30
(2) Q14766-2,5, LTBP1, Latent-transforming growth factor beta-binding protein 1

969 Q14766-3, LTBP1, Latent-transforming growth factor beta-binding protein 1 — 1/0/30

970 Q14767, LTBP2, Latent-transforming growth factor beta-binding protein 2 — 33/0/1

971 (1) Q9NS15-1, LTBP3, Latent-transforming growth factor beta-binding protein 3 — 1/0/1
(2) Q9NS15-2, LTBP3, Latent-transforming growth factor beta-binding protein 3

972 (1) Q8N2S1-2, LTBP4, Latent-transforming growth factor beta-binding protein 4 — 2/9/1
(2) Q8N2S1-1,3, LTBP4, Latent-transforming growth factor beta-binding protein 4
(3) Q8N2S1-4, LTBP4, Latent-transforming growth factor beta-binding protein 4

973	(1)	P02788-1, LTF, Lactotransferrin	2/0/0
	(2)	P02788-2, LTF, Lactotransferrin	
974		P51884, LUM, Lumican	14/0/0
975		O60449-2,3, LY75, Lymphocyte antigen 75	4/0/0
976		Q9Y6Y9-1,2, LY96, Lymphocyte antigen 96	1/0/0
977		P61626, LYZ, Lysozyme C	13/0/0
978	(1)	Q9UPN3-1, MACF1, Microtubule-actin cross-linking factor 1, isoforms 1/2/3/5	0/0/8
	(2)	Q9UPN3-2-4, MACF1, Microtubule-actin cross-linking factor 1, isoforms 1/2/3/5	
979		Q9UPN3-5, MACF1, Microtubule-actin cross-linking factor 1, isoforms 1/2/3/5	2/0/8
980		Q7Z304-1, MAMDC2, MAM domain-containing protein 2	2/0/0
981	(1)	P33908-1, MAN1A1, Mannosyl-oligosaccharide 1,2-alpha-mannosidase IA	10/0/0
	(2)	P33908-2, MAN1A1, Mannosyl-oligosaccharide 1,2-alpha-mannosidase IA	
982		Q16706, MAN2A1, Alpha-mannosidase 2	2/0/0
983		P46821, MAP1B, Microtubule-associated protein 1B	4/0/0
984		P29966, MARCKS, Myristoylated alanine-rich C-kinase substrate	3/0/0
985	(1)	P48740-1, MASP1, Mannan-binding lectin serine protease 1	5/0/11
	(2)	P48740-3, MASP1, Mannan-binding lectin serine protease 1	
986		P48740-2,4, MASP1, Mannan-binding lectin serine protease 1	5/0/11
987		O00187-1, MASP2, Mannan-binding lectin serine protease 2	2/0/6
988		O00187-2, MASP2, Mannan-binding lectin serine protease 2	1/0/6
989		P31153-1,2, MAT2A, S-adenosylmethionine synthase isoform type-2	2/0/0
990	(1)	O00339-2, MATN2, Matrilin-2	1/16/0
	(2)	O00339-1, MATN2, Matrilin-2	
	(3)	O00339-3, MATN2, Matrilin-2	
	(4)	O00339-4, MATN2, Matrilin-2	
991	(1)	P43243-1, MATR3, Matrin-3	4/0/0
	(2)	P43243-2, MATR3, Matrin-3	
992		MatrixProt_SaCV_Frankf, NA, NA	2/0/0

993		P02144, MB, Myoglobin	7/0/1
994		Q8NI22-1-3, MCFD2, Multiple coagulation factor deficiency protein 2	1/0/0
995		P40925-1-3, MDH1, Malate dehydrogenase, cytoplasmic	4/0/0
996	(1)	P21741-1, MDK, Midkine	2/0/0
	(2)	P21741-2, MDK, Midkine	
997		P48163-1, ME1, NADP-dependent malic enzyme	4/0/0
998		O60244, MED14, Mediator of RNA polymerase II transcription subunit 14	2/0/0
999		O75095-1,2, MEGF6, Multiple epidermal growth factor-like domains protein 6	7/0/0
1000		Q9UJH8, METRN, Meteorin	4/0/0
1001	(1)	Q641Q3-1, METRNL, Meteorin-like protein	4/0/0
	(2)	Q641Q3-2, METRNL, Meteorin-like protein	
1002		P55001-1-3, MFAP2, Microfibrillar-associated protein 2	3/0/0
1003		P55083-1,2, MFAP4, Microfibril-associated glycoprotein 4	7/0/0
1004		Q13361-1,2, MFAP5, Microfibrillar-associated protein 5	5/0/1
1005		P26572, MGAT1, Alpha-1,3-mannosyl-glycoprotein 2-beta-N-acetylglucosaminyltransferase	12/0/0
1006		Q9UQ53-1-3, MGAT4B, Alpha-1,3-mannosyl-glycoprotein 4-beta-N-acetylglucosaminyltransferase B	4/0/0
1007	(1)	Q09328, MGAT5, Alpha-1,6-mannosylglycoprotein 6-beta-N-acetylglucosaminyltransferase A	7/0/0
	(2)	Q96EE4, CCDC126, Coiled-coil domain-containing protein 126	
1008		P08493-1,2, MGP, Matrix Gla protein	5/0/0
1009	(1)	Q5JRA6-1,2, MIA3, Melanoma inhibitory activity protein 3	2/0/0
	(2)	Q5JRA6-3, MIA3, Melanoma inhibitory activity protein 3	
1010	(1)	Q9UNW1-1, MINPP1, Multiple inositol polyphosphate phosphatase 1	0/2/0
	(2)	Q9UNW1-2,3, MINPP1, Multiple inositol polyphosphate phosphatase 1	
	(3)	Q9UNW1-4, MINPP1, Multiple inositol polyphosphate phosphatase 1	
1011		P50281, MMP14, Matrix metalloproteinase-14	4/0/0
1012	(1)	Q99542-1, MMP19, Matrix metalloproteinase-19	2/0/0
	(2)	Q99542-3, MMP19, Matrix metalloproteinase-19	

	(3)	Q99542-5, MMP19, Matrix metalloproteinase-19	
1013	(1)	P08253-1, MMP2, 72 kDa type IV collagenase	2/19/0
	(2)	P08253-2, MMP2, 72 kDa type IV collagenase	
	(3)	P08253-3, MMP2, 72 kDa type IV collagenase	
1014	(1)	Q9H239-1, MMP28, Matrix metalloproteinase-28	1/0/0
	(2)	Q9H239-2, MMP28, Matrix metalloproteinase-28	
1015		Q9H8L6, MMRN2, Multimerin-2	6/0/0
1016		Q86VD1-1,2, MORC1, MORC family CW-type zinc finger protein 1	2/0/0
1017	(1)	P05164-1-3, MPO, Myeloperoxidase	5/0/0
	(2)	P11678, EPX, Eosinophil peroxidase	
1018		P25325-1,2, MPST, 3-mercaptopyruvate sulfurtransferase	4/0/0
1019		Q9UBG0, MRC2, C-type mannose receptor 2	3/0/0
1020	(1)	Q13421-2-4, MSLN, Mesothelin	1/0/0
	(2)	Q13421-1, MSLN, Mesothelin	
1021	(1)	P26038, MSN, Moesin	11/0/7
	(2)	P35241-1,5, RDX, Radixin	
1022	(1)	P26927, MST1, Hepatocyte growth factor-like protein	9/0/0
	(2)	Q2TV78-1,2, MST1L, Putative macrophage stimulating 1-like protein	
1023		P04731, MT1A, Metallothionein-1A	4/0/0
1024		P11586, MTHFD1, C-1-tetrahydrofolate synthase, cytoplasmic	9/0/0
1025		Q14764, MVP, Major vault protein	6/0/0
1026		Q9NR99, MXRA5, Matrix-remodeling-associated protein 5	41/0/0
1027	(1)	P84157-1, MXRA7, Matrix-remodeling-associated protein 7	2/0/2
	(2)	P84157-3, MXRA7, Matrix-remodeling-associated protein 7	
1028		P84157-2, MXRA7, Matrix-remodeling-associated protein 7	1/0/2
1029		O75592-1,2, MYCBP2, E3 ubiquitin-protein ligase MYCBP2	8/0/0
1030	(1)	P35580-4,5, MYH10, Myosin-10	1/0/22
	(2)	P35580-1-3, MYH10, Myosin-10	
1031	(1)	P35749-1-4, MYH11, Myosin-11	0/0/20
	(2)	P13533, MYH6, Myosin-6	
1032	(1)	Q9UKX3, MYH13, Myosin-13	0/0/2
	(2)	P11055, MYH3, Myosin-3	
	·	P12882, MYH1, Myosin-1	
	·	P13535, MYH8, Myosin-8	

	·	Q9UKX2-1, MYH2, Myosin-2	
	·	Q9Y623, MYH4, Myosin-4	
	(3)	A7E2Y1-1, MYH7B, Myosin-7B	
1033	(1)	Q7Z406-1,2,6, MYH14, Myosin-14	0/1/10
	(2)	Q7Z406-4, MYH14, Myosin-14	
	(3)	Q7Z406-5, MYH14, Myosin-14	
1034		P12883, MYH7, Myosin-7	1/0/1
1035	(1)	P35579-1, MYH9, Myosin-9	22/0/21
	(2)	P35579-2, MYH9, Myosin-9	
1036	(1)	P05976-1, MYL1, Myosin light chain 1/3, skeletal muscle isoform	2/0/1
	(2)	P05976-2, MYL1, Myosin light chain 1/3, skeletal muscle isoform	
1037		Q9BUA6, MYL10, Myosin regulatory light chain 10	1/0/2
1038		O14950, MYL12B, Myosin regulatory light chain 12B	2/0/7
		P19105, MYL12A, Myosin regulatory light chain 12A	
1039	(1)	P10916, MYL2, Myosin regulatory light chain 2, ventricular/cardiac muscle isoform	7/0/2
	(2)	Q02045-1, MYL5, Myosin light chain 5	
1040		P08590, MYL3, Myosin light chain 3	10/0/1
1041	(1)	P60660-1,2, MYL6, Myosin light polypeptide 6	4/0/1
	(2)	P14649, MYL6B, Myosin light chain 6B	
1042	(1)	P24844-1, MYL9, Myosin regulatory light polypeptide 9	0/0/7
	(2)	P24844-2, MYL9, Myosin regulatory light polypeptide 9	
1043	(1)	Q15746-1-6, MYLK, Myosin light chain kinase, smooth muscle	2/4/0
	(2)	Q15746-8,10, MYLK, Myosin light chain kinase, smooth muscle	
	(3)	Q15746-7,9,11, MYLK, Myosin light chain kinase, smooth muscle	
1044		Q96A32, MYLPF, Myosin regulatory light chain 2, skeletal muscle isoform	6/0/0
1045		Q92614-1-5, MYO18A, Unconventional myosin-XVIIIa	5/0/1
1046		O00159-1-3, MYO1C, Unconventional myosin-Ic	4/0/0
1047		Q99972, MYOC, Myocilin	16/0/0
1048		E9PAV3-1,2, NACA, Nascent polypeptide-associated complex subunit alpha, muscle-specific form	3/0/0
		Q13765-1, NACA, Nascent polypeptide-associated complex subunit alpha	

1049	(1)	Q9UJ70-2, NAGK, N-acetyl-D-glucosamine kinase	1/0/0
	(2)	Q9UJ70-1, NAGK, N-acetyl-D-glucosamine kinase	
1050		P54802, NAGLU, Alpha-N-acetylglucosaminidase	1/0/0
1051		P43490, NAMPT, Nicotinamide phosphoribosyltransferase	5/0/0
1052		Q9NR45, NANS, Sialic acid synthase	5/0/0
1053		P55209-1-3, NAP1L1, Nucleosome assembly protein 1-like 1	1/0/1
1054		Q99733-1,2, NAP1L4, Nucleosome assembly protein 1-like 4	1/0/1
1055		P54920, NAPA, Alpha-soluble NSF attachment protein	6/0/0
1056	(1)	O43776-1, NARS, Asparagine--tRNA ligase, cytoplasmic	2/0/0
	(2)	O43776-2, NARS, Asparagine--tRNA ligase, cytoplasmic	
1057		P41271-1,2, NBL1, Neuroblastoma suppressor of tumorigenicity 1	3/0/0
1058	(1)	P61601, NCALD, Neurocalcin-delta	2/0/0
	(2)	P37235, HPCAL1, Hippocalcin-like protein 1	
	·	P84074, HPCA, Neuron-specific calcium-binding protein hippocalcin	
1059		P13591-1-6, NCAM1, Neural cell adhesion molecule 1	3/0/0
1060		Q6ZVX7, NCCRP1, F-box only protein 50	4/0/0
1061		A6NI72, NCF1B, Putative neutrophil cytosol factor 1B	2/0/0
		A8MVU1, NCF1C, Putative neutrophil cytosol factor 1C	
		P14598-1, NCF1, Neutrophil cytosol factor 1	
1062		P19338, NCL, Nucleolin	24/0/0
1063		Q9HCD5, NCOA5, Nuclear receptor coactivator 5	6/0/0
1064	(1)	Q92597-1, NDRG1, Protein NDRG1	1/0/0
	(2)	Q92597-2,3, NDRG1, Protein NDRG1	
1065		Q9UN36-1,3, NDRG2, Protein NDRG2	2/0/0
1066	(1)	Q9NVZ3-1,2,4, NECAP2, Adaptin ear-binding coat-associated protein 2	1/0/0
	(2)	Q9NVZ3-3, NECAP2, Adaptin ear-binding coat-associated protein 2	
1067		Q92692-1,2, NECTIN2, Nectin-2	2/0/0
1068		P12036-1,2, NEFH, Neurofilament heavy polypeptide	2/0/3
1069		P48681, NES, Nestin	3/0/1
1070		P19838-1-3, NFKB1, Nuclear factor NF-kappa-B p105 subunit	2/0/0
1071	(1)	P14543-1, NID1, Nidogen-1	1/0/0

(2) P14543-2, NID1, Nidogen-1

1/0/1

1072 (1) Q14112-1, NID2, Nidogen-2
(2) Q14112-2, NID2, Nidogen-2

0/1/0

1073 (1) P22392-1,2, NME2, Nucleoside diphosphate kinase B
(2) O60361, NME2P1, Putative nucleoside diphosphate kinase
(3) P15531-1,2, NME1, Nucleoside diphosphate kinase A

1/0/0

1074 P40261, NNMT, Nicotinamide N-methyltransferase

2/0/0

1075 O60936-1-3, NOL3, Nucleolar protein 3

1/1/2

1076 (1) Q15233-1, NONO, Non-POU domain-containing octamer-binding protein
(2) Q8WXF1-1,2, PSPC1, Paraspeckle component 1
(3) Q15233-2, NONO, Non-POU domain-containing octamer-binding protein

8/0/0

1077 Q04721, NOTCH2, Neurogenic locus notch homolog protein 2

10/0/0

1078 Q9UM47, NOTCH3, Neurogenic locus notch homolog protein 3

4/0/0

1079 P61916-1,2, NPC2, Epididymal secretory protein E1

2/0/0

1080 P55786-1, NPEPPS, Puromycin-sensitive aminopeptidase

0/9/0

1081 (1) P06748-1, NPM1, Nucleophosmin
(2) P06748-2, NPM1, Nucleophosmin
(3) P06748-3, NPM1, Nucleophosmin

3/0/0

1082 P01160, NPPA, Natriuretic peptides A

2/0/0

1083 P15559-1-3, NQO1, NAD(P)H dehydrogenase [quinone] 1

2/0/0

1084 (1) O60462-1-5, NRP2, Neuropilin-2
(2) O60462-6, NRP2, Neuropilin-2

1/0/0

1085 (1) P46459-1, NSF, Vesicle-fusing ATPase
(2) P46459-2, NSF, Vesicle-fusing ATPase

2/0/0

1086 Q9UNZ2-1,4,5, NSFL1C, NSFL1 cofactor p47

3/0/0

1087 Q9H0P0-1-4, NT5C3A, Cytosolic 5'-nucleotidase 3A

30/0/2

1088 Q02818, NUCB1, Nucleobindin-1

12/0/2

1089 P80303-1,2, NUCB2, Nucleobindin-2

6/0/0

1090 (1) Q9H1E3-1, NUCKS1, Nuclear ubiquitous casein and cyclin-dependent kinase substrate 1
(2) Q9H1E3-2, NUCKS1, Nuclear ubiquitous casein and cyclin-dependent kinase substrate 1

5/0/1

1091 Q9Y266, NUDC, Nuclear migration protein nudC

1092		O95989, NUDT3, Diphosphoinositol polyphosphate phosphohydrolase 1	1/0/0
1093		Q9UKK9, NUDT5, ADP-sugar pyrophosphatase	2/0/0
1094		Q14980-1-4, NUMA1, Nuclear mitotic apparatus protein 1	8/0/1
1095		P61970, NUTF2, Nuclear transport factor 2	2/0/0
1096	(1)	O00292-1, NA, NA	0/3/0
	(2)	O00292-2, NA, NA	
	(3)	O75610, LEFTY1, Left-right determination factor 1	
1097		Q86UD1, OAF, Out at first protein homolog	7/0/0
1098		P20774, OGN, Mimecan	13/0/0
1099	(1)	Q8WWZ8-1, OIT3, Oncoprotein-induced transcript 3 protein	2/0/0
	(2)	Q8WWZ8-2, OIT3, Oncoprotein-induced transcript 3 protein	
1100		Q9NTK5-1,2, OLA1, Obg-like ATPase 1	4/0/0
1101		Q6UWY5, OLFML1, Olfactomedin-like protein 1	10/0/0
1102		Q68BL7-1-3, OLFML2A, Olfactomedin-like protein 2A	1/0/0
1103		Q68BL8-1, OLFML2B, Olfactomedin-like protein 2B	2/0/0
1104	(1)	Q9NRN5-1, OLFML3, Olfactomedin-like protein 3	0/5/0
	(2)	Q9NRN5-2, OLFML3, Olfactomedin-like protein 3	
	(3)	Q9NRN5-3, OLFML3, Olfactomedin-like protein 3	
1105		Q99983, OMD, Osteomodulin	4/0/0
1106		Q96CV9-1-3, OPTN, Optineurin	0/0/2
1107		P02763, ORM1, Alpha-1-acid glycoprotein 1	4/0/4
1108		P19652, ORM2, Alpha-1-acid glycoprotein 2	5/0/4
1109		P22059, OSBP, Oxysterol-binding protein 1	3/0/0
1110		Q12889, OVGP1, Oviduct-specific glycoprotein	12/0/0
1111		P04220, NA, Ig mu heavy chain disease protein	1/0/12
1112		Q32P28-1,3,4, P3H1, Prolyl 3-hydroxylase 1	4/0/1
1113		P13674-1-3, P4HA1, Prolyl 4-hydroxylase subunit alpha-1	1/0/0
1114		P07237, P4HB, Protein disulfide-isomerase	26/0/1
1115		Q9UQ80-1,2, PA2G4, Proliferation-associated protein 2G4	4/0/1
1116	(1)	P11940-1, PABPC1, Polyadenylate-binding protein 1	0/9/0
	(2)	Q4VXU2-1,2, PABPC1L, Polyadenylate-binding protein 1-like	
	(3)	Q9H361, PABPC3, Polyadenylate-binding protein 3	
	(4)	P11940-2, PABPC1, Polyadenylate-binding protein 1	

1117		P68402-1, PAFAH1B2, Platelet-activating factor acetylhydrolase IB subunit beta	2/0/0
1118		Q9H074-1-3, PAIP1, Polyadenylate-binding protein-interacting protein 1	2/0/0
1119		Q13177, PAK2, Serine/threonine-protein kinase PAK 2	3/0/0
1120	(1)	P19021-1,3,5, PAM, Peptidyl-glycine alpha-amidating monooxygenase	0/13/0
	(2)	P19021-4,6, PAM, Peptidyl-glycine alpha-amidating monooxygenase	
	(3)	P19021-2, PAM, Peptidyl-glycine alpha-amidating monooxygenase	
1121	(1)	Q6UXH9-1, PAMR1, Inactive serine protease PAMR1	0/0/15
	(2)	Q6UXH9-3, PAMR1, Inactive serine protease PAMR1	
1122		Q6UXH9-2, PAMR1, Inactive serine protease PAMR1	1/0/15
1123	(1)	O95428-1,5,6, PAPLN, Papilin	0/9/0
	(2)	O95428-2, PAPLN, Papilin	
	(3)	O95428-3, PAPLN, Papilin	
	(4)	O95428-4, PAPLN, Papilin	
1124		O95340-1,2, PAPSS2, Bifunctional 3'-phosphoadenosine 5'-phosphosulfate synthase 2	3/0/0
1125		Q99497, PARK7, Protein deglycase DJ-1	2/0/0
1126		Q9NVD7-1, PARVA, Alpha-parvin	4/0/0
1127	(1)	Q96AQ6-1,2, PBXIP1, Pre-B-cell leukemia transcription factor-interacting protein 1	1/0/0
	(2)	Q96AQ6-3, PBXIP1, Pre-B-cell leukemia transcription factor-interacting protein 1	
1128	(1)	Q15365, PCBP1, Poly(rC)-binding protein 1	6/0/5
	(2)	P57721-1-5, PCBP3, Poly(rC)-binding protein 3	
1129		Q15366-1-8, PCBP2, Poly(rC)-binding protein 2	1/0/5
1130		Q9Y5F9-1,2, PCDHGB6, Protocadherin gamma-B6	2/0/0
1131		P22061-1,2, PCMT1, Protein-L-isoaspartate(D-aspartate) O-methyltransferase	6/0/0
1132		O95613-1, PCNT, Pericentrin	3/0/0
1133		Q15113, PCOLCE, Procollagen C-endopeptidase enhancer 1	22/0/0
1134		Q9UKZ9, PCOLCE2, Procollagen C-endopeptidase enhancer 2	12/0/0
1135		Q9UHG2, PCSK1N, ProSAAS	5/0/0

16/0/16 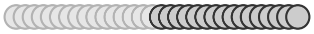

1136 Q92824-1, PCSK5, Proprotein convertase subtilisin/kexin type 5

1/0/16 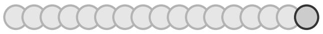

1137 Q92824-2, PCSK5, Proprotein convertase subtilisin/kexin type 5

9/0/0 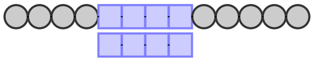

1138 (1) Q8NBP7-1, PCSK9, Proprotein convertase subtilisin/kexin type 9

(2) Q8NBP7-2, PCSK9, Proprotein convertase subtilisin/kexin type 9

2/0/0 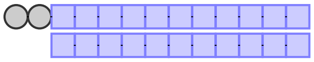

1139 (1) Q9UHG3-1, PCYOX1, Prenylcysteine oxidase 1

(2) Q9UHG3-2, PCYOX1, Prenylcysteine oxidase 1

2/0/0 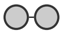

1140 Q53EL6-1,2, PDCD4, Programmed cell death protein 4

13/0/0 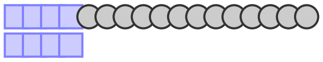

1141 (1) Q8WUM4-1,2, PDCD6IP, Programmed cell death 6-interacting protein

(2) Q8WUM4-3, PDCD6IP, Programmed cell death 6-interacting protein

2/0/0 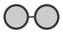

1142 P01127-1,2, PDGFB, Platelet-derived growth factor subunit B

0/5/0 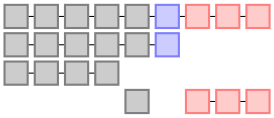

1143 (1) Q9NRA1-1, PDGFC, Platelet-derived growth factor C

(2) Q9NRA1-2, PDGFC, Platelet-derived growth factor C

(3) Q9NRA1-3, PDGFC, Platelet-derived growth factor C

(4) Q9NRA1-4, PDGFC, Platelet-derived growth factor C

3/0/0 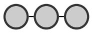

1144 Q9GZP0-1,2, PDGFD, Platelet-derived growth factor D

8/0/0 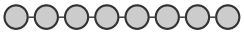

1145 Q15198, PDGFRL, Platelet-derived growth factor receptor-like protein

9/0/0 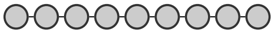

1146 P30101, PDIA3, Protein disulfide-isomerase A3

15/0/0 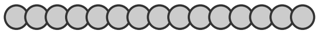

1147 P13667, PDIA4, Protein disulfide-isomerase A4

11/0/0 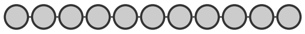

1148 Q15084-1-5, PDIA6, Protein disulfide-isomerase A6

5/0/0 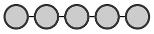

1149 Q96HC4-1,4,6,7, PDLIM5, PDZ and LIM domain protein 5

3/0/0 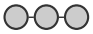

1150 Q15121-1,2, PEA15, Astrocytic phosphoprotein PEA-15

13/0/0 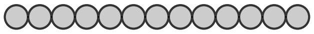

1151 P01210, PENK, Proenkephalin-A [Cleaved into: Synenkephalin; Met-enkephalin

4/0/1 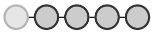

1152 P17858-1,2, PFKL, ATP-dependent 6-phosphofructokinase, liver type

1/0/1 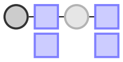

1153 (1) P08237-1,3, PFKM, ATP-dependent 6-phosphofructokinase, muscle type

(2) P08237-2, PFKM, ATP-dependent 6-phosphofructokinase, muscle type

7/0/0 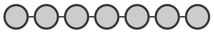

1154 P07737, PFN1, Profilin-1

1155		P35080-1,2, PFN2, Profilin-2	1/0/0
1156		P0DJD7, PGA4, Pepsin A-4	2/0/0
		P0DJD8, PGA3, Pepsin A-3	
		P0DJD9, PGA5, Pepsin A-5	
1157	(1)	P18669, PGAM1, Phosphoglycerate mutase 1	0/2/0
	(2)	Q8N0Y7, PGAM4, Probable phosphoglycerate mutase 4	
	(3)	P15259, PGAM2, Phosphoglycerate mutase 2	
1158		P52209-1,2, PGD, 6-phosphogluconate dehydrogenase, decarboxylating	10/0/0
1159	(1)	P00558-1,2, PGK1, Phosphoglycerate kinase 1	8/0/0
	(2)	P07205, PGK2, Phosphoglycerate kinase 2	
1160		O95336, PGLS, 6-phosphogluconolactonase	5/0/0
1161		Q96PD5-1,2, PGLYRP2, N-acetylmuramoyl-L-alanine amidase	11/0/0
1162	(1)	P36871-1, PGM1, Phosphoglucomutase-1	2/6/0
	(2)	P36871-2, PGM1, Phosphoglucomutase-1	
	(3)	P36871-3, PGM1, Phosphoglucomutase-1	
1163	(1)	Q96G03-1, PGM2, Phosphoglucomutase-2	2/0/0
	(2)	Q96G03-2, PGM2, Phosphoglucomutase-2	
1164		O95394-1,3,4, PGM3, Phosphoacetylglucosamine mutase	5/0/0
1165		O00264-1,2, PGRMC1, Membrane-associated progesterone receptor component 1	3/0/0
1166		P35232-1, PHB, Prohibitin	2/0/0
1167		Q99623-1, PHB2, Prohibitin-2	3/0/0
1168		O43175, PHGDH, D-3-phosphoglycerate dehydrogenase	11/0/0
1169		Q9NRX4-1,2, PHPT1, 14 kDa phosphohistidine phosphatase	1/0/0
1170		P19957, PI3, Elafin	6/0/0
1171		P01833, PIGR, Polymeric immunoglobulin receptor	5/0/0
1172		Q13526, PIN1, Peptidyl-prolyl cis-trans isomerase NIMA-interacting 1	1/0/0
1173		Q00169, PITPNA, Phosphatidylinositol transfer protein alpha isoform	5/0/0
1174		Q504Y2, PKDCC, Extracellular tyrosine-protein kinase PKDCC	6/0/0
1175	(1)	P14618-1, PKM, Pyruvate kinase PKM	0/27/0
	(2)	P30613-1,2, PKLR, Pyruvate kinase PKLR	
	(3)	P14618-2, PKM, Pyruvate kinase PKM	
	(4)	P14618-3, PKM, Pyruvate kinase PKM	
1176		Q13835-1,2, PKP1, Plakophilin-1	12/0/0

1177 Q9Y446-1,2, PKP3, Plakophilin-3

2/0/0 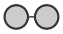

0/2/0 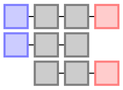

1178 (1) Q53H76-1,3, PLA1A, Phospholipase A1 member A
(2) Q53H76-2, PLA1A, Phospholipase A1 member A
(3) Q53H76-4, PLA1A, Phospholipase A1 member A

4/0/0 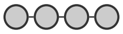

1179 Q9BZM1, PLA2G12A, Group XIIA secretory phospholipase A2

12/0/0 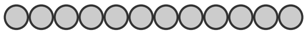

1180 P14555, PLA2G2A, Phospholipase A2, membrane associated

0/1/13 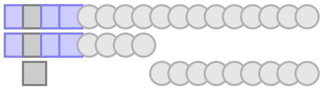

1181 (1) P00750-1, PLAT, Tissue-type plasminogen activator
(2) P00750-2, PLAT, Tissue-type plasminogen activator
(3) P00750-4, PLAT, Tissue-type plasminogen activator

1/0/13 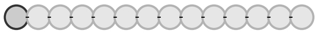

1182 P00750-3, PLAT, Tissue-type plasminogen activator

2/0/0 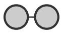

1183 P51178-1,2, PLCD1, 1-phosphatidylinositol 4,5-bisphosphate phosphodiesterase delta-1

2/0/0 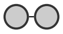

1184 Q96BZ4, PLD4, Phospholipase D4

1/102/6 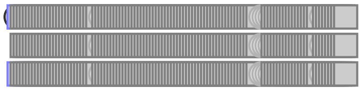

1185 (1) Q15149-5, PLEC, Plectin
(2) Q15149-3, PLEC, Plectin
(3) Q15149-1,2,4,6-9, PLEC, Plectin

48/2/2 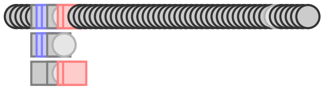

1186 (1) P00747, PLG, Plasminogen
(2) Q02325, PLGLB1, Plasminogen-like protein B
(3) Q15195, PLGLA, Plasminogen-like protein A

3/0/0 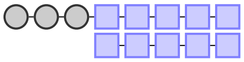

1187 (1) O60664-1,3,4, PLIN3, Perilipin-3
(2) O60664-2, PLIN3, Perilipin-3

6/0/0 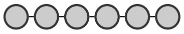

1188 Q96Q06-1,2, PLIN4, Perilipin-4

5/0/0 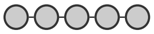

1189 Q02809-1,2, PLOD1, Procollagen-lysine,2-oxoglutarate 5-dioxygenase 1

10/0/0 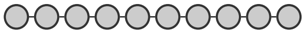

1190 O60568, PLOD3, Procollagen-lysine,2-oxoglutarate 5-dioxygenase 3

1/0/7 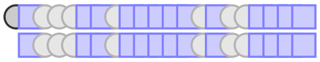

1191 (1) P13797-1,2, PLS3, Plastin-3
(2) P13797-3, PLS3, Plastin-3

0/13/0 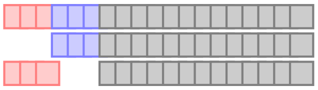

1192 (1) P55058-1, PLTP, Phospholipid transfer protein
(2) P55058-4, PLTP, Phospholipid transfer protein
(3) P55058-2,3, PLTP, Phospholipid transfer protein

2/0/0 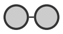

1193 Q6UX71-1,2, PLXDC2, Plexin domain-containing protein 2

3/0/0 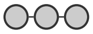

1194 O15031, PLXNB2, Plexin-B2

1195		Q15126, PMVK, Phosphomevalonate kinase	2/0/0
1196		P00491, PNP, Purine nucleoside phosphorylase	2/0/0
1197		Q9NP80-1-3, PNPLA8, Calcium-independent phospholipase A2-gamma	1/0/1
1198	(1)	Q7Z5L7-1-3, PODN, Podocan	4/0/0
	(2)	Q7Z5L7-4, PODN, Podocan	
1199	(1)	Q8WVV4-1,2, POF1B, Protein POF1B	8/0/0
	(2)	Q8WVV4-3, POF1B, Protein POF1B	
1200		Q7Z3K3-1-3,5-7, POGZ, Pogo transposable element with ZNF domain	3/0/0
1201		Q8WZA1-1,2, POMGNT1, Protein O-linked-mannose beta-1,2-N-acetylglucosaminyltransferase 1	6/0/0
1202	(1)	P27169, PON1, Serum paraoxonase/arylesterase 1	11/0/2
	(2)	Q15165-1-3, PON2, Serum paraoxonase/arylesterase 2	
1203		Q15166, PON3, Serum paraoxonase/lactonase 3	6/0/2
1204		Q15063-1, POSTN, Periostin	3/0/45
1205		Q15063-2, POSTN, Periostin	0/0/44
1206		Q15063-3, POSTN, Periostin	0/0/44
1207		Q15063-4, POSTN, Periostin	0/0/42
1208		Q15063-5, POSTN, Periostin	0/0/46
1209		Q15063-6, POSTN, Periostin	0/0/41
1210		Q15063-7, POSTN, Periostin	0/0/39
1211		Q6S8J3-1, POTEE, POTE ankyrin domain family member E	3/0/11
1212		A5A3E0, POTEF, POTE ankyrin domain family member F	2/0/10
1213		Q6S545-1,2, POTEH, POTE ankyrin domain family member H	4/0/3
1214		P0CG38, POTEI, POTE ankyrin domain family member I	0/0/10
		P0CG39, POTEJ, POTE ankyrin domain family member J	
1215		Q15181, PPA1, Inorganic pyrophosphatase	3/0/0
1216	(1)	P62937-1, PPIA, Peptidyl-prolyl cis-trans isomerase A	1/4/1
	(2)	P62937-2, PPIA, Peptidyl-prolyl cis-trans isomerase A	
	(3)	A0A075B759, NA, NA	
	·	A0A0B4J2A2, NA, NA	
	·	F5H284, PPIAL4D, Peptidyl-prolyl cis-trans isomerase A-like 4D	
	·	P0DN26, PPIAL4F, Peptidyl-prolyl cis-trans isomerase A-like 4F	

· P0DN37, PPIAL4G, Peptidyl-prolyl cis-trans isomerase A-like 4G

(4) Q9Y536, PPIAL4A, Peptidyl-prolyl cis-trans isomerase A-like 4A

1217 P23284, PPIB, Peptidyl-prolyl cis-trans isomerase B — 5/0/1

1218 O60437, PPL, Periplakin — 42/0/2

1219 P49593-1, PPM1F, Protein phosphatase 1F — 3/0/0

1220 P36873-1,2, PPP1CC, Serine/threonine-protein phosphatase PP1-gamma catalytic subunit — 3/0/0

P62136-1-3, PPP1CA, Serine/threonine-protein phosphatase PP1-alpha catalytic subunit

P62140, PPP1CB, Serine/threonine-protein phosphatase PP1-beta catalytic subunit

0/2/0

1221 (1) Q15435-1, PPP1R7, Protein phosphatase 1 regulatory subunit 7

(2) Q15435-2, PPP1R7, Protein phosphatase 1 regulatory subunit 7

(3) Q15435-3, PPP1R7, Protein phosphatase 1 regulatory subunit 7

(4) Q15435-4,5, PPP1R7, Protein phosphatase 1 regulatory subunit 7

1222 P67775-1, PPP2CA, Serine/threonine-protein phosphatase 2A catalytic subunit alpha isoform — 0/0/4

1223 P67775-2, PPP2CA, Serine/threonine-protein phosphatase 2A catalytic subunit alpha isoform — 1/0/3

1224 P62714, PPP2CB, Serine/threonine-protein phosphatase 2A catalytic subunit beta isoform — 1/0/3

1225 P30153, PPP2R1A, Serine/threonine-protein phosphatase 2A 65 kDa regulatory subunit A alpha isoform — 10/0/0

1226 Q08209-1-3, PPP3CA, Serine/threonine-protein phosphatase 2B catalytic subunit alpha isoform — 2/0/0

0/1/4

1227 (1) Q96NZ9-1, PRAP1, Proline-rich acidic protein 1

(2) Q96NZ9-2, PRAP1, Proline-rich acidic protein 1

(3) Q96NZ9-4, PRAP1, Proline-rich acidic protein 1

1228 Q96NZ9-3, PRAP1, Proline-rich acidic protein 1 — 1/0/4

1229 Q9NQV6-6, PRDM10, PR domain zinc finger protein 10 — 5/0/1

1230 Q06830, PRDX1, Peroxiredoxin-1 — 6/0/1

1231 P32119-1, PRDX2, Peroxiredoxin-2 — 6/0/1

1232 P30048-1,2, PRDX3, Thioredoxin-dependent peroxide reductase, mitochondrial — 2/0/0

1/0/0

1233 (1) P30044-1-3, PRDX5, Peroxiredoxin-5, mitochondrial

(2) P30044-4, PRDX5, Peroxiredoxin-5, mitochondrial

1234 P30041, PRDX6, Peroxiredoxin-6 — 11/0/0

1235 P51888, PRELP, Prolargin — 7/0/0

1236 P48147, PREP, Prolyl endopeptidase — 2/0/0

1237 P13727-1,2, PRG2, Bone marrow proteoglycan — 2/0/0

1238 (1) Q92954-3, PRG4, Proteoglycan 4 — 0/0/49
(2) Q92954-4, PRG4, Proteoglycan 4

1239 (1) Q92954-6, PRG4, Proteoglycan 4 — 1/3/49
(2) Q92954-1, PRG4, Proteoglycan 4
(3) Q92954-2, PRG4, Proteoglycan 4
(4) Q92954-5, PRG4, Proteoglycan 4

1240 Q13131-1,2, PRKAA1, 5'-AMP-activated protein kinase catalytic subunit alpha-1 — 2/0/0

1241 (1) P10644-1, PRKAR1A, cAMP-dependent protein kinase type I-alpha regulatory subunit — 2/0/0
(2) P10644-2, PRKAR1A, cAMP-dependent protein kinase type I-alpha regulatory subunit

1242 (1) P13861-1, PRKAR2A, cAMP-dependent protein kinase type II-alpha regulatory subunit — 1/0/0
(2) P13861-2, PRKAR2A, cAMP-dependent protein kinase type II-alpha regulatory subunit

1243 P14314-1,2, PRKCSH, Glucosidase 2 subunit beta — 11/0/0

1244 P78527-1,2, PRKDC, DNA-dependent protein kinase catalytic subunit — 5/0/0

1245 Q99873-1-4, PRMT1, Protein arginine N-methyltransferase 1 — 4/0/0

1246 P04156-1,2, PRNP, Major prion protein — 4/0/0

1247 P04070-1,2, PROC, Vitamin K-dependent protein C — 12/0/0

1248 P07225, PROS1, Vitamin K-dependent protein S — 27/0/1

1249 P22891-1,2, PROZ, Vitamin K-dependent protein Z — 8/0/0

1250 Q9UMS4, PRPF19, Pre-mRNA-processing factor 19 — 2/0/0

1251 P41219-1,2, PRPH, Peripherin — 0/0/4

1252 (1) P60891-1,2, PRPS1, Ribose-phosphate pyrophosphokinase 1 — 1/0/0
(2) P11908-1,2, PRPS2, Ribose-phosphate pyrophosphokinase 2

1253 O60256-1-4, PRPSAP2, Phosphoribosyl pyrophosphate synthase-associated protein 2 — 2/0/0

1254 P07477, PRSS1, Trypsin-1 — 1/0/1

1255 (1) P07478, PRSS2, Trypsin-2 — 2/0/2
(2) P35030-1-5, PRSS3, Trypsin-3

1256 O95084-1, PRSS23, Serine protease 23 — 2/0/2

1257 O95084-2, PRSS23, Serine protease 23 — 1/0/2

1258 Q8NHM4, PRSS3P2, Putative trypsin-6 — 1/0/2

1259 P07602-1-3, PSAP, Prosaposin — 19/0/0

1260 P25786-1,2, PSMA1, Proteasome subunit alpha type-1 — 3/0/0

1261 P25787, PSMA2, Proteasome subunit alpha type-2 — 2/0/0

1262 P25788-1,2, PSMA3, Proteasome subunit alpha type-3 — 4/0/0

1263 (1) P25789-1, PSMA4, Proteasome subunit alpha type-4 — 1/0/0
(2) P25789-2, PSMA4, Proteasome subunit alpha type-4

1264 P60900-1,2, PSMA6, Proteasome subunit alpha type-6 — 2/0/0

1265 (1) O14818-1, PSMA7, Proteasome subunit alpha type-7 — 1/0/0
(2) O14818-2, PSMA7, Proteasome subunit alpha type-7

1266 P20618, PSMB1, Proteasome subunit beta type-1 — 2/0/0

1267 P49721, PSMB2, Proteasome subunit beta type-2 — 1/0/1

1268 (1) Q99436-1, PSMB7, Proteasome subunit beta type-7 — 1/0/0
(2) Q99436-2, PSMB7, Proteasome subunit beta type-7

1269 (1) P62191-1, PSMC1, 26S protease regulatory subunit 4 — 1/0/1
(2) P62191-2, PSMC1, 26S protease regulatory subunit 4

1270 (1) P35998-1, PSMC2, 26S protease regulatory subunit 7 — 1/0/0
(2) P35998-2, PSMC2, 26S protease regulatory subunit 7

1271 P62195-1,2, PSMC5, 26S protease regulatory subunit 8 — 2/0/1

1272 Q99460-1,2, PSMD1, 26S proteasome non-ATPase regulatory subunit 1 — 5/0/0

1273 (1) O00232-1, PSMD12, 26S proteasome non-ATPase regulatory subunit 12 — 2/0/0
(2) O00232-2, PSMD12, 26S proteasome non-ATPase regulatory subunit 12

1274 Q9UNM6-1,2, PSMD13, 26S proteasome non-ATPase regulatory subunit 13 — 3/0/0

1275 (1) Q13200-1, PSMD2, 26S proteasome non-ATPase regulatory subunit 2 — 1/4/0
(2) Q13200-2, PSMD2, 26S proteasome non-ATPase regulatory subunit 2

(3) Q13200-3, PSMD2, 26S proteasome non-ATPase regulatory subunit 2

1276 O43242-1, PSMD3, 26S proteasome non-ATPase regulatory subunit 3 — 6/0/0

1277 Q16401-1,2, PSMD5, 26S proteasome non-ATPase regulatory subunit 5 — 1/0/0

1278 (1) Q15008-1, PSMD6, 26S proteasome non-ATPase regulatory subunit 6 — 1/0/2

(2) Q15008-3,4, PSMD6, 26S proteasome non-ATPase regulatory subunit 6

1279 Q15008-2, PSMD6, 26S proteasome non-ATPase regulatory subunit 6 — 1/0/2

1280 Q06323-1-3, PSME1, Proteasome activator complex subunit 1 — 5/0/0

1281 Q9UL46, PSME2, Proteasome activator complex subunit 2 — 10/0/0

1282 (1) P26599-1-3, PTBP1, Polypyrimidine tract-binding protein 1 — 2/1/0

(2) O95758-1-6, PTBP3, Polypyrimidine tract-binding protein 3

(3) O95758-7, PTBP3, Polypyrimidine tract-binding protein 3

1283 P41222, PTGDS, Prostaglandin-H2 D-isomerase — 5/0/0

1284 Q15185-1-4, PTGES3, Prostaglandin E synthase 3 — 2/0/0

1285 Q9P2B2, PTGFRN, Prostaglandin F2 receptor negative regulator — 1/0/0

1286 Q16647, PTGIS, Prostacyclin synthase — 2/0/0

1287 Q14914-1,2, PTGR1, Prostaglandin reductase 1 — 2/0/0

1288 P06454-1,2, PTMA, Prothymosin alpha [Cleaved into: Prothymosin alpha, N-terminally processed; Thymosin alpha-1] — 2/0/0

1289 P20962, PTMS, Parathymosin — 4/0/0

1290 P21246, PTN, Pleiotrophin — 2/0/0

1291 Q06124-2,3, PTPN11, Tyrosine-protein phosphatase non-receptor type 11 — 4/0/0

1292 P10586-1,2, PTPRF, Receptor-type tyrosine-protein phosphatase F — 3/0/0

1293 (1) Q6NZI2-1, PTRF, Polymerase I and transcript release factor — 5/4/0

(2) Q6NZI2-2, PTRF, Polymerase I and transcript release factor

(3) Q6NZI2-3, PTRF, Polymerase I and transcript release factor

1294 P26022, PTX3, Pentraxin-related protein PTX3 — 2/0/0

1295 Q00577, PURA, Transcriptional activator protein Pur-alpha — 2/0/0

1296 (1) Q92626-1, PXDN, Peroxidasin homolog — 14/0/1

(2) Q92626-2, PXDN, Peroxidasin homolog

1297		Q9ULZ3-1-3, PYCARD, Apoptosis-associated speck-like protein containing a CARD	3/0/0
1298	(1)	P20742-1, PZP, Pregnancy zone protein	7/0/9
	(2)	P20742-2, PZP, Pregnancy zone protein	
1299	(1)	Q16769-1, QPCT, Glutaminyl-peptide cyclotransferase	1/0/0
	(2)	Q16769-2, QPCT, Glutaminyl-peptide cyclotransferase	
1300	(1)	O00391-1, QSOX1, Sulfhydryl oxidase 1	3/0/0
	(2)	O00391-2, QSOX1, Sulfhydryl oxidase 1	
1301		Q9H974-1-4, QTRT2, Queuine tRNA-ribosyltransferase accessory subunit 2	3/0/0
1302		P61026, RAB10, Ras-related protein Rab-10	4/0/1
1303	(1)	Q15907-1, RAB11B, Ras-related protein Rab-11B	1/0/0
	(2)	P62491-1,2, RAB11A, Ras-related protein Rab-11A	
	·	Q15907-2, RAB11B, Ras-related protein Rab-11B	
1304		P61106, RAB14, Ras-related protein Rab-14	3/0/0
1305	(1)	P62820-1, RAB1A, Ras-related protein Rab-1A	0/1/5
	(2)	P62820-2, RAB1A, Ras-related protein Rab-1A	
	(3)	P62820-3, RAB1A, Ras-related protein Rab-1A	
1306	(1)	Q9H0U4, RAB1B, Ras-related protein Rab-1B	0/0/5
	(2)	Q92928, RAB1C, Putative Ras-related protein Rab-1C	
1307	(1)	P61019-1,2, RAB2A, Ras-related protein Rab-2A	2/0/0
	(2)	Q8WUD1-1,2, RAB2B, Ras-related protein Rab-2B	
1308		Q15286-1,2, RAB35, Ras-related protein Rab-35	0/0/2
1309		P20339-1,2, RAB5A, Ras-related protein Rab-5A	1/0/0
1310		P51148-1,2, RAB5C, Ras-related protein Rab-5C	2/0/0
1311		P51149, RAB7A, Ras-related protein Rab-7a	5/0/0
1312	(1)	Q92930, RAB8B, Ras-related protein Rab-8B	1/1/2
	(2)	P61006-1,2, RAB8A, Ras-related protein Rab-8A	
	(3)	P51153, RAB13, Ras-related protein Rab-13	
1313		Q15276-1,2, RABEP1, Rab GTPase-binding effector protein 1	5/0/1
1314	(1)	P63000-1,2, RAC1, Ras-related C3 botulinum toxin substrate 1	1/2/1
	(2)	P15153, RAC2, Ras-related C3 botulinum toxin substrate 2	
	(3)	P60763, RAC3, Ras-related C3 botulinum toxin substrate 3	
1315		P63244, RACK1, Receptor of activated protein C kinase 1	12/0/0
1316	(1)	P54727-1, RAD23B, UV excision repair protein RAD23 homolog B	2/0/0

(2) P54725-1-3, RAD23A, UV excision repair protein RAD23 homolog A

1317 Q9UKM9-1,2, RALY, RNA-binding protein Raly 3/0/0

1318 P62826, RAN, GTP-binding nuclear protein Ran 4/0/0

1319 (1) P61224-3, RAP1B, Ras-related protein Rap-1b 1/1/0

(2) P62834, RAP1A, Ras-related protein Rap-1A

(3) A6NIZ1, NA, Ras-related protein Rap-1b-like protein

· P61224-1,2, RAP1B, Ras-related protein Rap-1b

1320 P49788-1,2, RARRES1, Retinoic acid receptor responder protein 1 7/0/0

1321 Q99969, RARRES2, Retinoic acid receptor responder protein 2 8/0/0

1322 P54136-1,2, RARS, Arginine--tRNA ligase, cytoplasmic 3/0/0

1323 (1) P38159-1,2, RBMX, RNA-binding motif protein, X chromosome 3/0/0

(2) Q96E39, RBMXL1, RNA binding motif protein, X-linked-like-1

1324 P02753, RBP4, Retinol-binding protein 4 10/0/0

1325 Q15293-1,2, RCN1, Reticulocalbin-1 3/0/0

1326 Q96D15, RCN3, Reticulocalbin-3 3/0/0

1327 P61586, RHOA, Transforming protein RhoA 2/0/4

1328 P08134, RHOC, Rho-related GTP-binding protein RhoC 1/0/4

1329 (1) Q9H4E5-1,2, RHOJ, Rho-related GTP-binding protein RhoJ 2/0/1

(2) P17081, RHOQ, Rho-related GTP-binding protein RhoQ

· P84095, RHOG, Rho-related GTP-binding protein RhoG

1330 Q9NPQ8-1-4, RIC8A, Synembryn-A 2/0/1

1331 P07998, RNASE1, Ribonuclease pancreatic 6/0/0

1332 P34096, RNASE4, Ribonuclease 4 12/0/1

1333 Q9P0P0, RNF181, E3 ubiquitin-protein ligase RNF181 1/0/0

1334 P13489, RNH1, Ribonuclease inhibitor 12/0/0

1335 P15927-1-3, RPA2, Replication protein A 32 kDa subunit 2/0/0

1336 P27635, RPL10, 60S ribosomal protein L10 2/0/0

1337 P62906, RPL10A, 60S ribosomal protein L10a 5/0/0

1338 (1) P30050-1, RPL12, 60S ribosomal protein L12 5/0/0

(2) P30050-2, RPL12, 60S ribosomal protein L12

1339 (1) P26373-1, RPL13, 60S ribosomal protein L13 1/0/1

(2) P26373-2, RPL13, 60S ribosomal protein L13

1340		P50914, RPL14, 60S ribosomal protein L14	8/0/0
1341	(1)	P61313-1, RPL15, 60S ribosomal protein L15	2/0/0
	(2)	P61313-2, RPL15, 60S ribosomal protein L15	
1342		P18621-1-3, RPL17, 60S ribosomal protein L17	2/0/1
1343		Q07020-1,2, RPL18, 60S ribosomal protein L18	2/0/0
1344		P84098, RPL19, 60S ribosomal protein L19	4/0/0
1345		P46778, RPL21, 60S ribosomal protein L21	2/0/0
1346		P35268, RPL22, 60S ribosomal protein L22	3/0/0
1347		P62750, RPL23A, 60S ribosomal protein L23a	4/0/0
1348		P83731, RPL24, 60S ribosomal protein L24	4/0/0
1349		P61254, RPL26, 60S ribosomal protein L26	1/0/0
		Q9UNX3, RPL26L1, 60S ribosomal protein L26-like 1	
1350		P61353, RPL27, 60S ribosomal protein L27	5/0/0
1351	(1)	P46779-1-3, RPL28, 60S ribosomal protein L28	1/0/0
	(2)	P46779-4,5, RPL28, 60S ribosomal protein L28	
1352		P47914, RPL29, 60S ribosomal protein L29	3/0/0
1353		P39023, RPL3, 60S ribosomal protein L3	7/0/0
1354		P62888, RPL30, 60S ribosomal protein L30	4/0/0
1355	(1)	P62899-1, RPL31, 60S ribosomal protein L31	1/0/0
	(2)	P62899-2,3, RPL31, 60S ribosomal protein L31	
1356		P49207, RPL34, 60S ribosomal protein L34	3/0/0
1357		P42766, RPL35, 60S ribosomal protein L35	2/0/0
1358		P18077, RPL35A, 60S ribosomal protein L35a	4/0/0
1359		Q9Y3U8, RPL36, 60S ribosomal protein L36	1/0/0
1360		A6NKH3, RPL37AP8, Putative 60S ribosomal protein L37a-like protein	1/0/0
		P61513, RPL37A, 60S ribosomal protein L37a	
1361		P36578, RPL4, 60S ribosomal protein L4	5/0/0
1362		P46777, RPL5, 60S ribosomal protein L5	8/0/0
1363		Q02878, RPL6, 60S ribosomal protein L6	6/0/0
1364		P18124, RPL7, 60S ribosomal protein L7	4/0/0
1365		P62424, RPL7A, 60S ribosomal protein L7a	4/0/0
1366		P62917, RPL8, 60S ribosomal protein L8	7/0/0

1367		P32969, RPL9, 60S ribosomal protein L9	6/0/0
1368	(1)	P05388-1, RPLP0, 60S acidic ribosomal protein P0	0/7/0
	(2)	P05388-2, RPLP0, 60S acidic ribosomal protein P0	
	(3)	Q8NHW5, RPLP0P6, 60S acidic ribosomal protein P0-like	
1369		P05387, RPLP2, 60S acidic ribosomal protein P2	2/0/0
1370		P04843, RPN1, Dolichyl-diphosphooligosaccharide--protein glycosyltransferase subunit 1	4/0/0
1371	(1)	P46783, RPS10, 40S ribosomal protein S10	4/0/0
	(2)	Q9NQ39, RPS10P5, Putative 40S ribosomal protein S10-like	
1372		P62280, RPS11, 40S ribosomal protein S11	2/0/0
1373		P25398, RPS12, 40S ribosomal protein S12	3/0/0
1374		P62277, RPS13, 40S ribosomal protein S13	4/0/0
1375		P62263, RPS14, 40S ribosomal protein S14	4/0/0
1376		P62244, RPS15A, 40S ribosomal protein S15a	2/0/0
1377		P62249, RPS16, 40S ribosomal protein S16	5/0/0
1378		P62269, RPS18, 40S ribosomal protein S18	4/0/0
1379		P39019, RPS19, 40S ribosomal protein S19	5/0/0
1380		P15880, RPS2, 40S ribosomal protein S2	8/0/0
1381		P60866-1,2, RPS20, 40S ribosomal protein S20	3/0/0
1382		P63220, RPS21, 40S ribosomal protein S21	2/0/0
1383		P62266, RPS23, 40S ribosomal protein S23	3/0/0
1384		P62847-1-4, RPS24, 40S ribosomal protein S24	3/0/0
1385	(1)	P62979, RPS27A, Ubiquitin-40S ribosomal protein S27a	4/0/0
	(2)	P0CG47, UBB, Polyubiquitin-B [Cleaved into: Ubiquitin]	
	·	P0CG48, UBC, Polyubiquitin-C [Cleaved into: Ubiquitin]	
	·	P62987, UBA52, Ubiquitin-60S ribosomal protein L40	
1386		P62857, RPS28, 40S ribosomal protein S28	2/0/0
1387		P23396-1,2, RPS3, 40S ribosomal protein S3	7/0/0
1388		P61247, RPS3A, 40S ribosomal protein S3a	4/0/0
1389	(1)	P62701, RPS4X, 40S ribosomal protein S4, X isoform	4/0/0
	(2)	P22090, RPS4Y1, 40S ribosomal protein S4, Y isoform 1	
	·	Q8TD47, RPS4Y2, 40S ribosomal protein S4, Y isoform 2	
1390		P46782, RPS5, 40S ribosomal protein S5 [Cleaved into: 40S ribosomal protein S5, N-terminally processed]	4/0/0
1391		P62753, RPS6, 40S ribosomal protein S6	6/0/0
1392		P51812, RPS6KA3, Ribosomal protein S6 kinase alpha-3	9/0/0

1393 P62081, RPS7, 40S ribosomal protein S7 3/0/0

1394 P62241, RPS8, 40S ribosomal protein S8 2/0/0

1395 P46781, RPS9, 40S ribosomal protein S9 7/0/0

1396 P08865, RPSA, 40S ribosomal protein SA 6/0/0

1397 (1) Q9P2E9-1,2, RRBP1, Ribosome-binding protein 1 1/0/0
(2) Q9P2E9-3, RRBP1, Ribosome-binding protein 1

1398 Q15404-1, RSU1, Ras suppressor protein 1 1/0/0

1399 Q9Y3I0, RTCB, tRNA-splicing ligase RtcB homolog 3/0/0

1400 O95197-1-7, RTN3, Reticulon-3 1/0/0
Q16799-1-3, RTN1, Reticulon-1

1401 P60903, S100A10, Protein S100-A10 5/0/0

1402 P31949, S100A11, Protein S100-A11 5/0/0

1403 Q9HCY8, S100A14, Protein S100-A14 2/0/0

1404 Q96FQ6, S100A16, Protein S100-A16 2/0/0

1405 P29034, S100A2, Protein S100-A2 4/0/0

1406 P26447, S100A4, Protein S100-A4 3/0/0

1407 P06703, S100A6, Protein S100-A6 4/0/0

1408 (1) P31151, S100A7, Protein S100-A7 3/0/0
(2) Q86SG5, S100A7A, Protein S100-A7A

1409 P05109, S100A8, Protein S100-A8 10/0/0

1410 P06702, S100A9, Protein S100-A9 7/0/0

1411 (1) P0DJI8, SAA1, Serum amyloid A-1 protein 3/0/7
(2) P0DJI9-2, SAA2, Serum amyloid A-2 protein

1412 P0DJI9-1, SAA2, Serum amyloid A-2 protein 2/0/7

1413 P35542, SAA4, Serum amyloid A-4 protein 7/0/0

1414 (1) Q9Y3Z3-1,3,4, SAMHD1, Deoxynucleoside triphosphate triphosphohydrolase SAMHD1 1/0/0
(2) Q9Y3Z3-2, SAMHD1, Deoxynucleoside triphosphate triphosphohydrolase SAMHD1

1415 P49591, SARS, Serine--tRNA ligase, cytoplasmic 7/0/0

1416 Q15020-1,4, SART3, Squamous cell carcinoma antigen recognized by T-cells 3 1/0/1

1417 (1) Q6UWP8-1, SBSN, Suprabasin 7/0/0
(2) Q6UWP8-2, SBSN, Suprabasin

1418 Q96GP6-1,2, SCARF2, Scavenger receptor class F member 2 5/0/0

1419		P13521, SCG2, Secretogranin-2	21/0/0
1420		Q8WXD2-1,2, SCG3, Secretogranin-3	5/0/0
1421		P05408-1, SCG5, Neuroendocrine protein 7B2	1/0/1
1422		P05408-2, SCG5, Neuroendocrine protein 7B2	1/0/1
1423		P11684, SCGB1A1, Uteroglobin	3/0/0
1424		Q12765-1-3, SCRN1, Secernin-1	3/0/0
1425		O00560-1-3, SDCBP, Syntenin-1	3/0/0
			0/12/0
1426	(1)	Q9BRK5-1, SDF4, 45 kDa calcium-binding protein	
	(2)	Q9BRK5-2, SDF4, 45 kDa calcium-binding protein	
	(3)	Q9BRK5-5, SDF4, 45 kDa calcium-binding protein	
	(4)	Q9BRK5-6, SDF4, 45 kDa calcium-binding protein	
	(5)	Q9BRK5-3,4, SDF4, 45 kDa calcium-binding protein	
1427		O95810, SDPR, Serum deprivation-response protein	7/0/0
1428		Q8N3Y7-1,2, SDR16C5, Epidermal retinol dehydrogenase 2	1/0/0
1429		Q8NEX9, SDR9C7, Short-chain dehydrogenase/reductase family 9C member 7	2/0/0
1430	(1)	Q15436-1, SEC23A, Protein transport protein Sec23A	0/0/0
	(2)	Q15437, SEC23B, Protein transport protein Sec23B	
	(3)	Q15436-2, SEC23A, Protein transport protein Sec23A	
			1/0/1
1431	(1)	Q9Y6Y8-1, SEC23IP, SEC23-interacting protein	
	(2)	Q9Y6Y8-2, SEC23IP, SEC23-interacting protein	
1432		P53992-1, SEC24C, Protein transport protein Sec24C	4/0/0
			2/0/0
1433	(1)	O94979-1-4,6,8-10, SEC31A, Protein transport protein Sec31A	
	(2)	O94979-7, SEC31A, Protein transport protein Sec31A	
1434		Q9UBV2-1,2, SEL1L, Protein sel-1 homolog 1	4/0/0
1435		Q13214-1,2, SEMA3B, Semaphorin-3B	6/0/1
			4/0/1
1436	(1)	Q99985-1, SEMA3C, Semaphorin-3C	
	(2)	Q99985-2, SEMA3C, Semaphorin-3C	
1437		Q13275-1,2, SEMA3F, Semaphorin-3F	6/0/0
1438		Q9NPR2-1,2, SEMA4B, Semaphorin-4B	3/0/0
1439		P49908, SEPP1, Selenoprotein P	10/0/0
			1/0/0
1440	(1)	Q15019-1,2, SEPT2, Septin-2	

	(2)	Q15019-3, SEPT2, Septin-2	
1441		Q16181-1,2, SEPT7, Septin-7	7/0/0
1442		Q9UHD8-1,2,5,7, SEPT9, Septin-9	3/0/0
1443		Q8NC51-1-4, SERBP1, Plasminogen activator inhibitor 1 RNA-binding protein	2/0/0
1444	(1)	P01009-1, SERPINA1, Alpha-1-antitrypsin	4/18/0
	(2)	P01009-2, SERPINA1, Alpha-1-antitrypsin	
	(3)	P01009-3, SERPINA1, Alpha-1-antitrypsin	
1445		Q9UK55, SERPINA10, Protein Z-dependent protease inhibitor	17/0/0
1446		Q86U17, SERPINA11, Serpin A11	3/0/0
1447	(1)	P01011-1, SERPINA3, Alpha-1-antichymotrypsin	8/0/0
	(2)	P01011-2, SERPINA3, Alpha-1-antichymotrypsin	
1448		P29622, SERPINA4, Kallistatin	20/0/0
1449		P05154, SERPINA5, Plasma serine protease inhibitor	12/0/0
1450		P08185, SERPINA6, Corticosteroid-binding globulin	5/0/0
1451		P05543, SERPINA7, Thyroxine-binding globulin	11/0/0
1452		P30740-1,2, SERPINB1, Leukocyte elastase inhibitor	2/0/0
1453		Q96P63-1,2, SERPINB12, Serpin B12	2/0/0
1454		P05120, SERPINB2, Plasminogen activator inhibitor 2	3/0/0
1455	(1)	P29508-1, SERPINB3, Serpin B3	1/0/9
	(2)	P29508-2, SERPINB3, Serpin B3	
1456		P48594, SERPINB4, Serpin B4	7/0/9
1457		P36952-1, SERPINB5, Serpin B5	4/0/0
1458		P01008, SERPINC1, Antithrombin-III	28/0/0
1459		P05546, SERPIND1, Heparin cofactor 2	27/0/0
1460	(1)	P05121-1, SERPINE1, Plasminogen activator inhibitor 1	1/0/0
	(2)	P05121-2, SERPINE1, Plasminogen activator inhibitor 1	
1461		P07093-1-3, SERPINE2, Glia-derived nexin	4/0/0
1462		P36955, SERPINF1, Pigment epithelium-derived factor	18/0/0
1463	(1)	P08697-1, SERPINF2, Alpha-2-antiplasmin	3/0/0
	(2)	P08697-2, SERPINF2, Alpha-2-antiplasmin	
1464	(1)	P05155-1,3, SERPING1, Plasma protease C1 inhibitor	1/0/0

	(2)	P05155-2, SERPING1, Plasma protease C1 inhibitor	
1465		P50454, SERPINH1, Serpin H1	5/0/0
1466	(1)	Q01105-1-4, SET, Protein SET	1/0/0
	(2)	P0DME0, SETSIP, Protein SETSIP	
1467	(1)	P31947-1, SFN, 14-3-3 protein sigma	1/0/4
	(2)	P31947-2, SFN, 14-3-3 protein sigma	
1468	(1)	P23246-1, SFPQ, Splicing factor, proline- and glutamine-rich	1/0/2
	(2)	P23246-2, SFPQ, Splicing factor, proline- and glutamine-rich	
1469		Q8N474, SFRP1, Secreted frizzled-related protein 1	5/0/0
1470		Q96HF1, SFRP2, Secreted frizzled-related protein 2	12/0/0
1471		Q6FHJ7, SFRP4, Secreted frizzled-related protein 4	4/0/0
1472		O43765, SGTA, Small glutamine-rich tetratricopeptide repeat-containing protein alpha	2/0/0
1473		Q9H299, SH3BGRL3, SH3 domain-binding glutamic acid-rich-like protein 3	3/0/0
1474		Q99961-1-3, SH3GL1, Endophilin-A2	2/0/0
1475		Q9Y371-1,3, SH3GLB1, Endophilin-B1	3/0/0
1476	(1)	P04278-1, SHBG, Sex hormone-binding globulin	0/13/0
	(2)	P04278-2,3, SHBG, Sex hormone-binding globulin	
	(3)	P04278-4, SHBG, Sex hormone-binding globulin	
	(4)	P04278-5, SHBG, Sex hormone-binding globulin	
1477		Q9H173, SIL1, Nucleotide exchange factor SIL1	5/0/0
1478		Q15477, SKIV2L, Helicase SKI2W	1/0/1
1479		P42285, SKIV2L2, Superkiller viralicidic activity 2-like 2	2/0/1
1480		P63208-1,2, SKP1, S-phase kinase-associated protein 1	3/0/1
1481		Q00325-1,2, SLC25A3, Phosphate carrier protein, mitochondrial	2/0/0
1482	(1)	P05141, SLC25A5, ADP/ATP translocase 2	2/3/0
	(2)	P12235, SLC25A4, ADP/ATP translocase 1	
	·	P12236, SLC25A6, ADP/ATP translocase 3	
	(3)	Q9H0C2, SLC25A31, ADP/ATP translocase 4	
1483	(1)	O14745-1, SLC9A3R1, Na(+)/H(+) exchange regulatory cofactor NHE-RF1	3/0/0
	(2)	O14745-2, SLC9A3R1, Na(+)/H(+) exchange regulatory cofactor NHE-RF1	
1484		Q9BQI6-2, SLF1, SMC5-SMC6 complex localization factor protein 1	2/0/0

1485		O75094-1,2,4, SLIT3, Slit homolog 3 protein	7/0/0
1486		Q9H2G2-1,2, SLK, STE20-like serine/threonine-protein kinase	9/0/1
1487		P03973, SLPI, Antileukoproteinase	15/0/0
1488		P55000, SLURP1, Secreted Ly-6/uPAR-related protein 1	2/0/0
1489		Q5SXM2, SNAPC4, snRNA-activating protein complex subunit 4	0/0/2
1490		P37840-1,2, SNCA, Alpha-synuclein	1/0/0
1491		Q7KZF4, SND1, Staphylococcal nuclease domain-containing protein 1	11/0/0
1492		Q8TER0-1,3-5, SNED1, Sushi, nidogen and EGF-like domain-containing protein 1	8/0/0
1493		P08621-1-3, SNRNP70, U1 small nuclear ribonucleoprotein 70 kDa	3/0/0
1494	(1)	P62316-1, SNRPD2, Small nuclear ribonucleoprotein Sm D2	1/0/0
	(2)	P62316-2, SNRPD2, Small nuclear ribonucleoprotein Sm D2	
1495		P62318-1,2, SNRPD3, Small nuclear ribonucleoprotein Sm D3	1/0/0
1496		Q13596-1-3, SNX1, Sorting nexin-1	4/0/0
1497		O60749-1,2, SNX2, Sorting nexin-2	2/0/0
1498		O60493-1,2,4, SNX3, Sorting nexin-3	2/0/0
1499		Q9Y5X3-1, SNX5, Sorting nexin-5	2/0/1
1500		Q9UNH7-1,2, SNX6, Sorting nexin-6	6/0/0
1501		P08294, SOD3, Extracellular superoxide dismutase [Cu-Zn]	8/0/0
1502		P23497-1, SP100, Nuclear autoantigen Sp-100	3/0/1
1503		P09486, SPARC, SPARC	13/0/0
1504	(1)	Q14515-1, SPARCL1, SPARC-like protein 1	5/0/0
	(2)	Q14515-2, SPARCL1, SPARC-like protein 1	
1505		Q5M775-1-5, SPECC1, Cytospin-B	1/0/1
1506		Q92563-1, SPOCK2, Testican-2	1/0/0
1507		Q9HCB6, SPON1, Spondin-1	21/0/0
1508		P10451-1-5, SPP1, Osteopontin	5/0/0
1509		Q13103, SPP2, Secreted phosphoprotein 24	8/0/0
1510		P35270, SPR, Sepiapterin reductase	3/0/0
1511		P22528, SPRR1B, Cornifin-B<br>P35321, SPRR1A, Cornifin-A	5/0/0

1512 (1) P22532, SPRR2D, Small proline-rich protein 2D — 1/0/3
(2) P35325, SPRR2B, Small proline-rich protein 2B

1513 (1) P22531, SPRR2E, Small proline-rich protein 2E — 1/0/3
(2) Q9BYE4, SPRR2G, Small proline-rich protein 2G
(3) P35326, SPRR2A, Small proline-rich protein 2A
(4) Q96RM1, SPRR2F, Small proline-rich protein 2F

1514 Q13813-1-3, SPTAN1, Spectrin alpha chain, non-erythrocytic 1 — 17/0/0

1515 Q01082-1-3, SPTBN1, Spectrin beta chain, non-erythrocytic 1 — 14/0/3

1516 (1) O15020-1, SPTBN2, Spectrin beta chain, non-erythrocytic 2 — 1/0/3
(2) O15020-2, SPTBN2, Spectrin beta chain, non-erythrocytic 2

1517 P30626-1-3, SRI, Sorcin — 5/0/0

1518 (1) P78539-1, SRPX, Sushi repeat-containing protein SRPX — 0/4/14
(2) P78539-2,5, SRPX, Sushi repeat-containing protein SRPX
(3) P78539-4, SRPX, Sushi repeat-containing protein SRPX

1519 P78539-3, SRPX, Sushi repeat-containing protein SRPX — 1/0/14

1520 Q8IYB3-1,2, SRRM1, Serine/arginine repetitive matrix protein 1 — 1/0/1

1521 (1) Q07955-1, SRSF1, Serine/arginine-rich splicing factor 1 — 1/0/0
(2) Q07955-2,3, SRSF1, Serine/arginine-rich splicing factor 1

1522 O75494-1-6, SRSF10, Serine/arginine-rich splicing factor 10 — 1/0/0

1523 (1) P84103-1, SRSF3, Serine/arginine-rich splicing factor 3 — 2/0/1
(2) P84103-2, SRSF3, Serine/arginine-rich splicing factor 3

1524 Q13247-1,3, SRSF6, Serine/arginine-rich splicing factor 6 — 2/0/0

1525 Q16629-1-4, SRSF7, Serine/arginine-rich splicing factor 7 — 5/0/1

1526 A1L4H1-1,2, SSC5D, Soluble scavenger receptor cysteine-rich domain-containing protein SSC5D — 3/0/0

1527 (1) P50502, ST13, Hsc70-interacting protein — 1/5/0
(2) Q8NFI4, ST13P5, Putative protein FAM10A5
(3) Q8IZP2, ST13P4, Putative protein FAM10A4

1528 Q11201, ST3GAL1, CMP-N-acetylneuraminate-beta-galactosamide-alpha-2,3-sialyltransferase 1 — 5/0/0

1529 P15907-1, ST6GAL1, Beta-galactoside alpha-2,6-sialyltransferase 1 — 3/0/0

1530 (1) Q9NY15-1, STAB1, Stabilin-1 — 8/0/0
(2) Q9NY15-2, STAB1, Stabilin-1

1531 P40763-1-3, STAT3, Signal transducer and activator of transcription 3

5/0/0 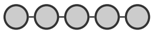

1532 P42229-1,2, STAT5A, Signal transducer and activator of transcription 5A

P51692, STAT5B, Signal transducer and activator of transcription 5B

2/0/0 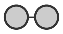

1533 O76061, STC2, Stanniocalcin-2

1/0/0 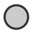

1534 P31948-1-3, STIP1, Stress-induced-phosphoprotein 1

2/0/0 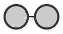

1535 Q9Y6E0-1,2, STK24, Serine/threonine-protein kinase 24

2/0/0 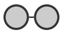

1536 Q9Y3F4-1,2, STRAP, Serine-threonine kinase receptor-associated protein

3/0/0 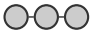

1537 P53999, SUB1, Activated RNA polymerase II transcriptional coactivator p15

6/0/0 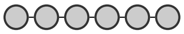

1538 Q9Y2Z0-1,2, SUGT1, Protein SGT1 homolog

2/0/0 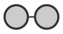

1539 Q8IWU6, SULF1, Extracellular sulfatase Sulf-1

6/0/0 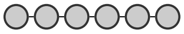

1540 Q8IWU5-1,2, SULF2, Extracellular sulfatase Sulf-2

4/0/0 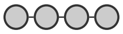

1541 (1) P50225-1, SULT1A1, Sulfotransferase 1A1

(2) P50225-2, SULT1A1, Sulfotransferase 1A1

(3) P50226, SULT1A2, Sulfotransferase 1A2

1/0/4 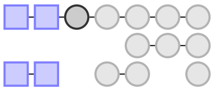

1542 (1) P0DMM9-1,3, SULT1A3, Sulfotransferase 1A3

· P0DMN0, SULT1A4, Sulfotransferase 1A4

(2) P0DMM9-2, SULT1A3, Sulfotransferase 1A3

0/0/4 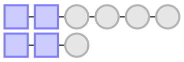

1543 O60279, SUSD5, Sushi domain-containing protein 5

7/0/0 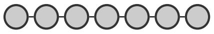

1544 (1) Q4LDE5-1, SVEP1, Sushi, von Willebrand factor type A, EGF and pentraxin domain-containing protein 1

(2) Q4LDE5-2, SVEP1, Sushi, von Willebrand factor type A, EGF and pentraxin domain-containing protein 1

(3) Q4LDE5-3, SVEP1, Sushi, von Willebrand factor type A, EGF and pentraxin domain-containing protein 1

(4) Q4LDE5-4, SVEP1, Sushi, von Willebrand factor type A, EGF and pentraxin domain-containing protein 1

7/28/0 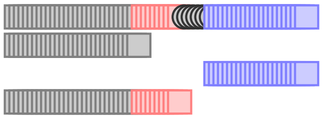

1545 Q9UH65, SWAP70, Switch-associated protein 70

2/0/0 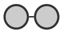

1546 (1) O60506-1, SYNCRIP, Heterogeneous nuclear ribonucleoprotein Q

(2) O60506-2, SYNCRIP, Heterogeneous nuclear ribonucleoprotein Q

(3) O60506-3, SYNCRIP, Heterogeneous nuclear ribonucleoprotein Q

(4) O60506-4, SYNCRIP, Heterogeneous nuclear ribonucleoprotein Q

0/7/6 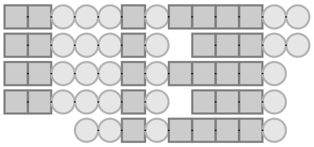

(5) O60506-5, SYNCRIP, Heterogeneous nuclear ribonucleoprotein Q

1547 Q8NF91-1,4, SYNE1, Nesprin-1 — 4/0/0

1548 Q9UHF0-1-3, TAC3, Tachykinin-3 — 2/0/0

1549 Q92804-1,2, TAF15, TATA-binding protein-associated factor 2N — 3/0/0

1550 Q01995, TAGLN, Transgelin — 8/0/1

1551 P37802-1,2, TAGLN2, Transgelin-2 — 10/0/1

1552 Q9UI15, TAGLN3, Transgelin-3 — 0/0/2

1553 P37837, TALDO1, Transaldolase — 6/0/0

1554 — 3/0/0
(1) Q13148-1, TARDBP, TAR DNA-binding protein 43
(2) Q13148-4, TARDBP, TAR DNA-binding protein 43

1555 Q99426-1,2, TBCB, Tubulin-folding cofactor B — 2/0/0

1556 Q15370-1,2, TCEB2, Transcription elongation factor B polypeptide 2 — 2/0/0

1557 Q07283, TCHH, Trichohyalin — 3/0/1

1558 — 3/0/0
(1) P20062-1, TCN2, Transcobalamin-2
(2) P20062-2, TCN2, Transcobalamin-2

1559 P17987, TCP1, T-complex protein 1 subunit alpha — 6/0/0

1560 P02787, TF, Serotransferrin — 38/0/0

1561 Q92734-1-4, TFG, Protein TFG — 3/0/0

1562 P10646-1, TFPI, Tissue factor pathway inhibitor — 1/0/0

1563 P48307-1,2, TFPI2, Tissue factor pathway inhibitor 2 — 4/0/0

1564 P01137, TGFB1, Transforming growth factor beta-1 — 2/0/0

1565 Q15582, TGFBI, Transforming growth factor-beta-induced protein ig-h3 — 25/0/0

1566 Q03167-1,2, TGFBR3, Transforming growth factor beta receptor type 3 — 14/0/0

1567 — 5/0/0
(1) P22735-1, TGM1, Protein-glutamine gamma-glutamyltransferase K
(2) P22735-2, TGM1, Protein-glutamine gamma-glutamyltransferase K

1568 — 3/0/1
(1) P21980-1, TGM2, Protein-glutamine gamma-glutamyltransferase 2
(2) P21980-2, TGM2, Protein-glutamine gamma-glutamyltransferase 2

1569 Q08188, TGM3, Protein-glutamine gamma-glutamyltransferase E — 11/0/0

1570		P49221, TGM4, Protein-glutamine gamma-glutamyltransferase 4	4/0/1
1571	(1)	P07996-1, THBS1, Thrombospondin-1	5/0/2
	(2)	P07996-2, THBS1, Thrombospondin-1	
1572		P35442, THBS2, Thrombospondin-2	26/0/2
1573	(1)	P49746-1, THBS3, Thrombospondin-3	2/0/1
	(2)	P49746-2, THBS3, Thrombospondin-3	
1574		P35443, THBS4, Thrombospondin-4	23/0/3
1575	(1)	Q6ZMP0-1, THSD4, Thrombospondin type-1 domain-containing protein 4	1/0/0
	(2)	Q6ZMP0-4, THSD4, Thrombospondin type-1 domain-containing protein 4	
1576	(1)	P35590-1, TIE1, Tyrosine-protein kinase receptor Tie-1	5/4/0
	(2)	P35590-2, TIE1, Tyrosine-protein kinase receptor Tie-1	
	(3)	P35590-3, TIE1, Tyrosine-protein kinase receptor Tie-1	
1577		P01033, TIMP1, Metalloproteinase inhibitor 1	5/0/0
1578		P16035, TIMP2, Metalloproteinase inhibitor 2	9/0/0
1579		P35625, TIMP3, Metalloproteinase inhibitor 3	3/0/0
1580		Q99727, TIMP4, Metalloproteinase inhibitor 4	3/0/0
1581	(1)	Q9GZM7-1, TINAGL1, Tubulointerstitial nephritis antigen-like	1/0/0
	(2)	Q9GZM7-3, TINAGL1, Tubulointerstitial nephritis antigen-like	
1582		O75663-1, TIPRL, TIP41-like protein	2/0/0
1583		Q07157-1,2, TJP1, Tight junction protein ZO-1	3/0/0
1584		Q9UDY2-1-7, TJP2, Tight junction protein ZO-2	3/0/0
1585	(1)	Q3LXA3-1, TKFC, Triokinase/FMN cyclase	1/0/0
	(2)	Q3LXA3-2, TKFC, Triokinase/FMN cyclase	
1586		P29401-1,2, TKT, Transketolase	4/0/0
1587	(1)	O43897-1, TLL1, Tolloid-like protein 1	4/0/3
	(2)	O43897-2, TLL1, Tolloid-like protein 1	
1588		Q9Y6L7, TLL2, Tolloid-like protein 2	1/0/6
1589		Q9Y490, TLN1, Talin-1	50/0/11
1590		Q9Y4G6, TLN2, Talin-2	3/0/11

1591		Q9NYL9, TMOD3, Tropomodulin-3	2/0/0
1592	(1)	P24821-1,4, TNC, Tenascin	2/0/0
	(2)	P24821-2,3,5,6, TNC, Tenascin	
1593		P98066, TNFAIP6, Tumor necrosis factor-inducible gene 6 protein	5/0/0
1594		O95379-1-4, TNFAIP8, Tumor necrosis factor alpha-induced protein 8	2/0/0
1595		Q9C0C2-1,2, TNKS1BP1, 182 kDa tankyrase-1-binding protein	3/0/0
1596		P13805-1-3, TNNT1, Troponin T, slow skeletal muscle	5/0/0
1597		Q92973-1-3, TNPO1, Transportin-1	5/0/1
1598	(1)	O14787-2, TNPO2, Transportin-2	0/0/1
	(2)	O14787-1, TNPO2, Transportin-2	
1599	(1)	P22105-1,3, TNXB, Tenascin-X	1/9/0
	(2)	P22105-2, TNXB, Tenascin-X	
	(3)	P22105-4, TNXB, Tenascin-X	
	(4)	Q16473, TNXA, Putative tenascin-XA	
1600		O14656-1, TOR1A, Torsin-1A	1/0/0
1601		O43399-1-7, TPD52L2, Tumor protein D54	2/0/0
1602	(1)	P60174-1,3, TPI1, Triosephosphate isomerase	1/0/0
	(2)	P60174-4, TPI1, Triosephosphate isomerase	
1603		P09493-1, TPM1, Tropomyosin alpha-1 chain	0/0/21
1604		P09493-4,10, TPM1, Tropomyosin alpha-1 chain	0/0/21
1605	(1)	P09493-3, TPM1, Tropomyosin alpha-1 chain	0/0/21
	(2)	P09493-2, TPM1, Tropomyosin alpha-1 chain	
1606		P09493-6, TPM1, Tropomyosin alpha-1 chain	0/0/20
1607		P09493-7, TPM1, Tropomyosin alpha-1 chain	0/0/20
1608		P09493-8, TPM1, Tropomyosin alpha-1 chain	0/0/20
1609	(1)	P09493-9, TPM1, Tropomyosin alpha-1 chain	0/0/21
	(2)	P09493-5, TPM1, Tropomyosin alpha-1 chain	
1610		P07951-1, TPM2, Tropomyosin beta chain	3/0/18
1611	(1)	P07951-2, TPM2, Tropomyosin beta chain	0/0/17
	(2)	P07951-3, TPM2, Tropomyosin beta chain	
1612		P06753-1, TPM3, Tropomyosin alpha-3 chain	1/0/17

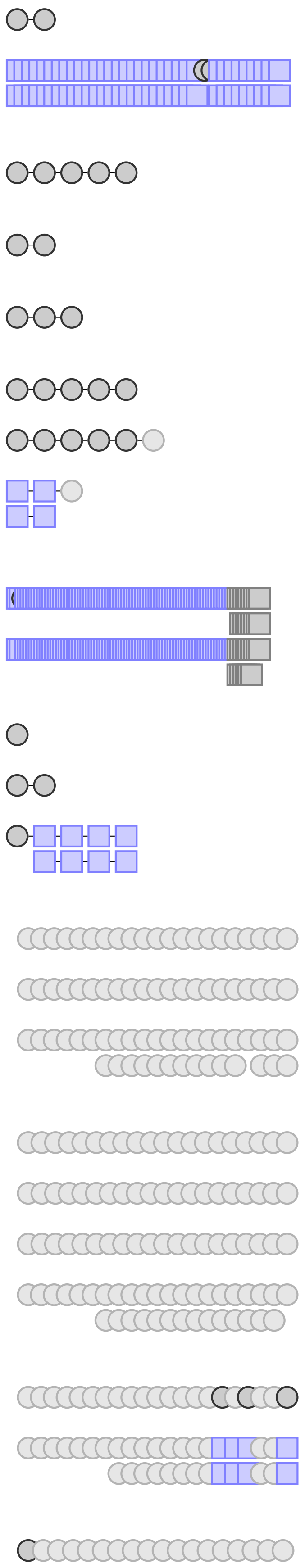

1613	(1)	P06753-2, TPM3, Tropomyosin alpha-3 chain	0/0/18
	(2)	P06753-3,6, TPM3, Tropomyosin alpha-3 chain	
1614	(1)	P06753-5, TPM3, Tropomyosin alpha-3 chain	0/0/22
	(2)	P06753-4, TPM3, Tropomyosin alpha-3 chain	
	(3)	P06753-7, TPM3, Tropomyosin alpha-3 chain	
1615		P67936-1, TPM4, Tropomyosin alpha-4 chain	4/0/15
1616		P67936-2, TPM4, Tropomyosin alpha-4 chain	0/0/17
1617		O14773-1,2, TPP1, Tripeptidyl-peptidase 1	4/0/0
1618		P20231, TPSB2, Tryptase beta-2	2/0/0
		Q15661-1, TPSAB1, Tryptase alpha/beta-1	
1619	(1)	P13693-1, TPT1, Translationally-controlled tumor protein	1/0/0
	(2)	P13693-2, TPT1, Translationally-controlled tumor protein	
1620		Q14134-1,2, TRIM29, Tripartite motif-containing protein 29	12/0/1
1621		Q15654-1, TRIP6, Thyroid receptor-interacting protein 6	2/0/1
1622		Q8WUA8, TSKU, Tsukushin	10/0/0
1623		Q13630, TSTA3, GDP-L-fucose synthase	3/0/0
1624		Q14166, TTLL12, Tubulin--tyrosine ligase-like protein 12	2/0/0
1625	(1)	Q8WZ42-12, TTN, Titin	4/26/6
	(2)	Q8WZ42-1,8, TTN, Titin	
	(3)	Q8WZ42-13, TTN, Titin	
	(4)	Q8WZ42-2, TTN, Titin	
	(5)	Q8WZ42-5, TTN, Titin	
	(6)	Q8WZ42-3,9,10, TTN, Titin	
	(7)	Q8WZ42-4,7,11, TTN, Titin	
1626		Q8WZ42-6, TTN, Titin	2/0/4
1627		P02766, TTR, Transthyretin	11/0/0
1628	(1)	Q71U36-1,2, TUBA1A, Tubulin alpha-1A chain	0/0/18
	(2)	Q9BQE3, TUBA1C, Tubulin alpha-1C chain	
	(3)	Q6PEY2, TUBA3E, Tubulin alpha-3E chain	
1629	(1)	P68363-1, TUBA1B, Tubulin alpha-1B chain	0/0/18
	(2)	P68363-2, TUBA1B, Tubulin alpha-1B chain	
1630	(1)	Q13748-1, TUBA3C, Tubulin alpha-3C/D chain	0/0/15

	(2)	Q13748-2, TUBA3C, Tubulin alpha-3C/D chain	
1631		P68366-1,2, TUBA4A, Tubulin alpha-4A chain	2/0/14
1632	(1)	Q9NY65-1, TUBA8, Tubulin alpha-8 chain	1/0/10
	(2)	Q9NY65-2, TUBA8, Tubulin alpha-8 chain	
1633		A6NHL2-1,2, TUBAL3, Tubulin alpha chain-like 3	1/0/3
1634	(1)	P07437, TUBB, Tubulin beta chain	2/0/15
	(2)	A6NNZ2, NA, Tubulin beta-8 chain-like protein LOC260334	
1635		Q9H4B7, TUBB1, Tubulin beta-1 chain	1/0/3
1636	(1)	Q13885, TUBB2A, Tubulin beta-2A chain	1/0/14
	(2)	Q9BVA1, TUBB2B, Tubulin beta-2B chain	
1637		Q13509-1, TUBB3, Tubulin beta-3 chain	0/0/10
1638		P04350, TUBB4A, Tubulin beta-4A chain	0/0/14
1639	(1)	P68371, TUBB4B, Tubulin beta-4B chain	1/0/16
	(2)	Q13509-2, TUBB3, Tubulin beta-3 chain	
	(3)	Q3ZCM7, TUBB8, Tubulin beta-8 chain	
1640		Q9BUF5, TUBB6, Tubulin beta-6 chain	2/0/7
1641		Q12792-2-4, TWF1, Twinfilin-1	2/0/1
1642		Q6IBS0, TWF2, Twinfilin-2	2/0/1
1643	(1)	Q9GZX9-1, TWSG1, Twisted gastrulation protein homolog 1	4/0/0
	(2)	Q9GZX9-2, TWSG1, Twisted gastrulation protein homolog 1	
1644		P10599-1,2, TXN, Thioredoxin	2/0/0
1645		Q8NBS9-1,2, TXNDC5, Thioredoxin domain-containing protein 5	4/0/1
1646		O43396, TXNL1, Thioredoxin-like protein 1	5/0/0
1647	(1)	P19971-1, TYMP, Thymidine phosphorylase	1/0/0
	(2)	P19971-2, TYMP, Thymidine phosphorylase	
1648		P22314-1,2, UBA1, Ubiquitin-like modifier-activating enzyme 1	21/0/1
1649		Q9GZZ9-1, UBA5, Ubiquitin-like modifier-activating enzyme 5	3/0/0
1650		A0AVT1-1,2, UBA6, Ubiquitin-like modifier-activating enzyme 6	1/0/0
1651		P61077-1-3, UBE2D3, Ubiquitin-conjugating enzyme E2 D3	2/0/0
		P62837-1,2, UBE2D2, Ubiquitin-conjugating enzyme E2 D2	
1652	(1)	Q969M7-3, UBE2F, NEDD8-conjugating enzyme UBE2F	1/0/0

(2) Q969M7-1,2,4-6, UBE2F, NEDD8-conjugating enzyme UBE2F

1653 P61086-1,3, UBE2K, Ubiquitin-conjugating enzyme E2 K — 4/0/0

1654 P68036-1, UBE2L3, Ubiquitin-conjugating enzyme E2 L3 — 1/0/1

1655 P61081, UBE2M, NEDD8-conjugating enzyme Ubc12 — 1/0/0

1656 P61088, UBE2N, Ubiquitin-conjugating enzyme E2 N — 3/0/0
Q5JXB2, UBE2NL, Putative ubiquitin-conjugating enzyme E2 N-like

1657 (1) Q15819, UBE2V2, Ubiquitin-conjugating enzyme E2 variant 2 — 1/0/0
(2) Q13404-1,2,4,7,8, UBE2V1, Ubiquitin-conjugating enzyme E2 variant 1

1658 Q5T4S7-1-4, UBR4, E3 ubiquitin-protein ligase UBR4 — 6/0/1

1/8/0

1659 (1) O60701-1, UGDH, UDP-glucose 6-dehydrogenase
(2) O60701-2, UGDH, UDP-glucose 6-dehydrogenase
(3) O60701-3, UGDH, UDP-glucose 6-dehydrogenase

1660 Q9NYU2-1,2, UGGT1, UDP-glucose:glycoprotein glucosyltransferase 1 — 4/0/0

1/0/0

1661 (1) Q16851-2, UGP2, UTP--glucose-1-phosphate uridylyltransferase
(2) Q16851-1, UGP2, UTP--glucose-1-phosphate uridylyltransferase

1662 Q9H3U1-1,2, UNC45A, Protein unc-45 homolog A — 3/0/0

1663 Q8N2C7-1,2,4,7, UNC80, Protein unc-80 homolog — 2/0/0

1664 Q92900-1,2, UPF1, Regulator of nonsense transcripts 1 — 2/0/0

1665 P07919, UQCRH, Cytochrome b-c1 complex subunit 6, mitochondrial — 2/0/0

1666 O94763-1, URI1, Unconventional prefoldin RPB5 interactor 1 — 0/0/2

1667 O60763-1,2, USO1, General vesicular transport factor p115 — 7/0/0

1668 P45974-1,2, USP5, Ubiquitin carboxyl-terminal hydrolase 5 — 5/0/0

1669 Q93008-1,3, USP9X, Probable ubiquitin carboxyl-terminal hydrolase FAF-X — 3/0/6

1670 O00507-1,2, USP9Y, Probable ubiquitin carboxyl-terminal hydrolase FAF-Y — 3/0/6

1671 Q9NQZ2, UTP3, Something about silencing protein 10 — 2/0/0

2/5/0

1672 (1) Q99536-1, VAT1, Synaptic vesicle membrane protein VAT-1 homolog
(2) Q99536-2, VAT1, Synaptic vesicle membrane protein VAT-1 homolog

(3) Q99536-3, VAT1, Synaptic vesicle membrane protein VAT-1 homolog

1673 Q9HCJ6, VAT1L, Synaptic vesicle membrane protein VAT-1 homolog-like — 1/0/0

1674 (1) P19320-1,3, VCAM1, Vascular cell adhesion protein 1 — 1/0/1

(2) P19320-2, VCAM1, Vascular cell adhesion protein 1

1675 P13611-1-5, VCAN, Versican core protein — 3/0/0

1676 (1) P18206-1,2, VCL, Vinculin — 37/0/0

(2) P18206-3, VCL, Vinculin

1677 P55072, VCP, Transitional endoplasmic reticulum ATPase — 18/0/0

1678 P21796, VDAC1, Voltage-dependent anion-selective channel protein 1 — 2/0/0

1679 P49767, VEGFC, Vascular endothelial growth factor C — 17/0/0

1680 O43915, VEGFD, Vascular endothelial growth factor D — 2/0/0

1681 O15240, VGF, Neurosecretory protein VGF [Cleaved into: Neuroendocrine regulatory peptide-1 — 11/0/0

1682 P08670, VIM, Vimentin — 23/0/9

1683 P98155-1,2, VLDLR, Very low-density lipoprotein receptor — 9/0/0

1684 Q96QK1, VPS35, Vacuolar protein sorting-associated protein 35 — 10/0/0

1685 (1) Q9UN37, VPS4A, Vacuolar protein sorting-associated protein 4A — 1/1/0

(2) Q6PIW4-1,2, FIGNL1, Fidgetin-like protein 1

(3) O75351, VPS4B, Vacuolar protein sorting-associated protein 4B

1686 P04004, VTN, Vitronectin — 17/0/0

1687 (1) Q6PCB0-1, VWA1, von Willebrand factor A domain-containing protein 1 — 2/0/0

(2) Q6PCB0-2, VWA1, von Willebrand factor A domain-containing protein 1

1688 (1) P04275-1, VWF, von Willebrand factor — 62/0/0

(2) P04275-2, VWF, von Willebrand factor

1689 P23381-1,2, WARS, Tryptophan--tRNA ligase, cytoplasmic — 4/0/0

1690 (1) O75083-1, WDR1, WD repeat-containing protein 1 — 2/0/0

(2) O75083-3, WDR1, WD repeat-containing protein 1

1691 Q8WWY7, WFDC12, WAP four-disulfide core domain protein 12 — 3/0/0

1692 (1) O76076-1, WISP2, WNT1-inducible-signaling pathway protein 2 — 6/0/1

	(2)	O76076-2, WISP2, WNT1-inducible-signaling pathway protein 2	
1693		Q5T750, XP32, Skin-specific protein 32	1/0/0
1694	(1)	Q9NQW7-3, XPNPEP1, Xaa-Pro aminopeptidase 1	1/0/0
	(2)	Q9NQW7-4, XPNPEP1, Xaa-Pro aminopeptidase 1	
1695		O14980, XPO1, Exportin-1	6/0/0
1696		P13010, XRCC5, X-ray repair cross-complementing protein 5	10/0/0
1697		P12956-1,2, XRCC6, X-ray repair cross-complementing protein 6	18/0/0
1698	(1)	Q8NBI6-1, XXYLT1, Xyloside xylosyltransferase 1	3/0/2
	(2)	Q8NBI6-2, XXYLT1, Xyloside xylosyltransferase 1	
1699		Q8NBI6-3, XXYLT1, Xyloside xylosyltransferase 1	1/0/2
1700		P16989-1-3, YBX3, Y-box-binding protein 3	1/0/0
1701	(1)	O15498-1, YKT6, Synaptobrevin homolog YKT6	1/0/0
	(2)	O15498-2, YKT6, Synaptobrevin homolog YKT6	
1702		Q5VVQ6-1,2, YOD1, Ubiquitin thioesterase OTU1	1/0/0
1703		P31946-1,2, YWHAB, 14-3-3 protein beta/alpha	6/0/9
1704	(1)	P62258-1, YWHAE, 14-3-3 protein epsilon	2/0/3
	(2)	P62258-2, YWHAE, 14-3-3 protein epsilon	
1705		P61981, YWHAG, 14-3-3 protein gamma	10/0/6
1706		Q04917, YWHAH, 14-3-3 protein eta	8/0/5
1707		P27348, YWHAQ, 14-3-3 protein theta	11/0/7
1708	(1)	P63104-1, YWHAZ, 14-3-3 protein zeta/delta	9/0/7
	(2)	P63104-2, YWHAZ, 14-3-3 protein zeta/delta	
1709		Q15916, ZBTB6, Zinc finger and BTB domain-containing protein 6	2/0/0
1710		O15231-1,3-8, ZNF185, Zinc finger protein 185	2/0/0
1711		Q5VUA4-1, ZNF318, Zinc finger protein 318	2/0/2
1712		Q96JM2-1,3, ZNF462, Zinc finger protein 462	8/0/0
1713		Q6ZNG1, ZNF600, Zinc finger protein 600	2/0/1
1714		Q03923-1,2, ZNF85, Zinc finger protein 85	4/0/1
1715	(1)	Q15942-1, ZYX, Zyxin	1/0/0
	(2)	Q15942-2, ZYX, Zyxin	

2/0/0 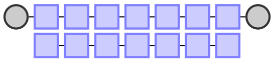

1716 (1) O43149-1, ZZEF1, Zinc finger ZZ-type and EF-hand domain-containing protein 1

(2) O43149-2, ZZEF1, Zinc finger ZZ-type and EF-hand domain-containing protein 1

Isobaric Tag Quantification Report
P7123_Run 4 *

isobar R package v1.18.0

February 6, 2017

Channel	Class
126	*P8-treat*
127	*P8-ctrl*
128	*P9-treat*
129	*P9-ctrl*
130	*P10-treat*
131	*P10-ctrl*

Column Descriptions

protein	Protein name, accession code, and description.
group	Protein group size, only displayed if it is not '1/1'. First number: Number of group reporters; second number: group size. See Protein Group Details for details.
peptides	Number of peptides for protein.
spectra	Number of idenfitied spectra for protein.
quant	Number of spectra with quantitative information for *ch2* versus *ch1*.
ratio	Ratio of protein in channel *ch2* relative to *ch1*.
*	Significance of protein ratio in sample.

1 Protein Relative Quantitation

Number of quantified proteins: P10-treat/P10-ctrl: 1477; P8-treat/P8-ctrl: 1477; P9-treat/P9-ctrl: 1467

1.1 Quantified Proteins

#	protein	group	peptides	spectra	ch1	ch2	quant	ratio
					127	126	100	**0.82**
**1**	136429		6	117	129	128	107	**4.42**
					131	130	115	**0.61**
					127	126	96	**0.92**
**2**	162648		48	337	129	128	100	**27.13**
					131	130	102	**1.70**
					127	126	2	**0.84**
**3**	A0A0B4J1V0		2	2	129	128	2	**1.17**
					131	130	2	**1.06**

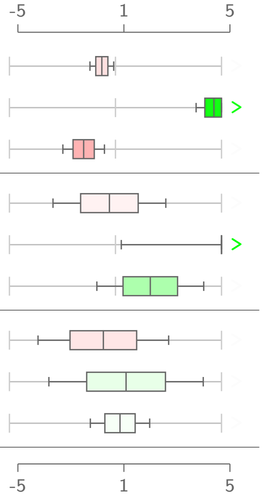

*This report was generated using the isobar R package version 1.18.0 [built using R 3.3.2; ; 2017-02-05 14:44:42 UTC; unix]. If you use it in published work, please cite 'Breitwieser FP *et al.*: General statistical modeling of data from protein relative expression isobaric tags, *Journal of Proteome Research* 2011' and 'Breitwieser FP and Colinge J: isobarPTM: A software tool for the quantitative analysis of post-translationally modified proteins,*Journal of Proteomics* 2013'

#	protein	group	peptides	spectra	ch1	ch2	quant	ratio
4	A0A0C4DH25		3	10	127	126	1	**1.03**
					129	128	1	**0.66**
					131	130	1	**0.98**
5	A0A0C4DH33		2	4	127	126	3	**1.23**
					129	128	2	**0.59**
					131	130	3	**1.14**
6	A1BG P04217$_1$: Alpha-1B-glycoprotein	1/2	13	21	127	126	21	**1.21**
					129	128	21	**1.02**
					131	130	20	**1.20**
7	A2M P01023: Alpha-2-macroglobulin		44	106	127	126	82	**0.84**
					129	128	83	**0.95**
					131	130	86	**1.07**
8	A4GALT Q9NPC4: Lactosylceramide 4-alpha-galactosyltransferase		3	3	127	126	3	**0.86**
					129	128	3	**1.74**
					131	130	3	**1.22**
9	AARS P49588$_{1,2}$: Alanine--tRNA ligase, cytoplasmic	2/2	12	13	127	126	10	**1.07**
					129	128	13	**1.23**
					131	130	13	**1.12**
10	ABHD14A Q9BUJ0: Protein ABHD14A		2	2	127	126	2	**0.78**
					129	128	2	**2.61**
					131	130	2	**0.97**
11	ABHD14B Q96IU4$_1$: Protein ABHD14B		2	2	127	126	2	**0.65**
					129	128	2	**1.27**
					131	130	2	**0.90**
12	ABI3BP Q7Z7G0$_1$: Target of Nesh-SH3		4	4	127	126	4	**0.64**
					129	128	4	**3.27**
					131	130	4	**0.71**
13	ACADVL P49748$_{1-3}$: Very long-chain specific acyl-CoA dehydrogenase, mitochondrial	3/3	2	2	127	126	2	**1.43**
					129	128	2	**2.44**
					131	130	2	**1.27**
14	ACO2 Q99798: Aconitate hydratase, mitochondrial		2	3	127	126	3	**0.65**
					129	128	3	**0.96**
					131	130	3	**0.85**
15	ACTB P60709, ACTG1 P63261: Actin, cytoplasmic 1, Actin, cytoplasmic 2	2/3	19	245	127	126	73	**0.86**
					129	128	70	**1.18**
					131	130	72	**1.03**

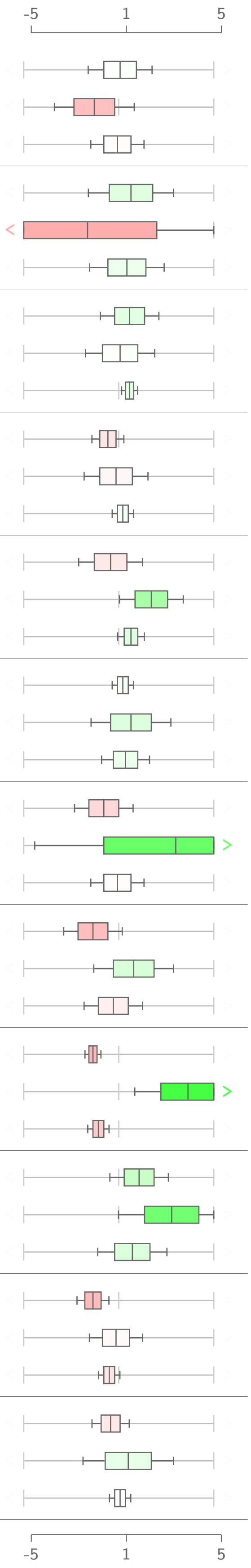

#	protein	group	peptides	spectra	ch1	ch2	quant	ratio
16	ACTC1 P68032: Actin, alpha cardiac muscle 1	1/5	16	201	127	126	38	**0.86**
					129	128	38	**0.95**
					131	130	39	**1.07**
17	ACTN1 P12814$_{2-4}$: Alpha-actinin-1	3/4	20	25	127	126	9	**1.36**
					129	128	8	**0.73**
					131	130	10	**1.13**
19	ACTN3 Q08043: Alpha-actinin-3		4	4	127	126	1	**0.94**
					129	128	1	**1.94**
					131	130	1	**0.92**
20	ACTN4 O43707$_1$: Alpha-actinin-4	1/3	29	43	127	126	27	**1.87**
					129	128	25	**0.51**
					131	130	23	**1.41**
21	ACTR1A P61163: Alpha-centractin		5	17	127	126	5	**1.94**
					129	128	5	**0.62**
					131	130	4	**1.05**
22	ACTR2 P61160$_1$: Actin-related protein 2	1/2	3	14	127	126	2	**0.78**
					129	128	2	**1.93**
					131	130	2	**1.00**
23	ACTR3 P61158: Actin-related protein 3		3	4	127	126	4	**0.79**
					129	128	3	**2.09**
					131	130	4	**0.96**
24	ADAM10 O14672$_1$: Disintegrin and metalloproteinase domain-containing protein 10		10	27	127	126	25	**1.63**
					129	128	21	**1.51**
					131	130	22	**1.08**
25	ADAM15 Q13444$_{1-10,12}$: Disintegrin and metalloproteinase domain-containing protein 15	11/13	3	4	127	126	4	**1.31**
					129	128	3	**0.54**
					131	130	4	**1.02**
26	ADAMDEC1 O15204$_{1,2}$: ADAM DEC1	2/2	6	7	127	126	7	**0.52**
					129	128	5	**1.63**
					131	130	7	**1.16**
27	ADAMTS1 Q9UHI8: A disintegrin and metalloproteinase with thrombospondin motifs 1		4	8	127	126	6	**2.04**
					129	128	4	**1.40**
					131	130	4	**1.06**
28	ADAMTS13 Q76LX8$_{1-3}$: A disintegrin and metalloproteinase with thrombospondin motifs 13	3/3	2	2	127	126	2	**1.04**
					129	128	2	**1.13**
					131	130	2	**1.09**

#	protein	group	peptides	spectra	ch1	ch2	quant	ratio
**29**	ADAMTS15 Q8TE58: A disintegrin and metalloproteinase with thrombospondin motifs 15		2	3	127	126	3	**1.44**
					129	128	3	**2.38**
					131	130	3	**1.05**
**30**	ADAMTS2 O95450$_{1}$: A disintegrin and metalloproteinase with thrombospondin motifs 2	1/2	7	11	127	126	9	**1.07**
					129	128	10	**1.65**
					131	130	7	**1.12**
**31**	ADAMTS5 Q9UNA0: A disintegrin and metalloproteinase with thrombospondin motifs 5		7	8	127	126	8	**1.49**
					129	128	8	**1.18**
					131	130	8	**1.12**
**32**	ADAMTS8 Q9UP79: A disintegrin and metalloproteinase with thrombospondin motifs 8		2	2	127	126	2	**1.59**
					129	128	2	**1.44**
					131	130	2	**1.22**
**33**	ADAMTSL2 Q86TH1: ADAMTS-like protein 2		4	4	127	126	3	**1.73**
					129	128	3	**1.47**
					131	130	3	**1.35**
**34**	ADAMTSL3 P82987$_{1}$: ADAMTS-like protein 3	1/2	9	13	127	126	10	**1.38**
					129	128	11	**0.82**
					131	130	12	**1.07**
**35**	ADAMTSL4 Q6UY14$_{1,3}$: ADAMTS-like protein 4	2/3	18	20	127	126	18	**1.48**
					129	128	17	**1.38**
					131	130	19	**1.28**
**36**	ADAMTSL5 Q6ZMM2$_{1,2}$: ADAMTS-like protein 5	2/2	1	1	127	126	1	**1.14**
					129	128	1	**1.21**
					131	130	1	**0.92**
**37**	ADD1 P35611$_{1-6}$: Alpha-adducin	6/6	1	1	127	126	1	**1.36**
					129	128	1	**1.29**
					131	130	1	**1.26**
**38**	ADGB Q8N7X0$_{1}$: Androglobin		3	4	127	126	4	**1.65**
					129	128	3	**0.95**
					131	130	3	**0.94**
**39**	ADH1B P00325$_{1}$: Alcohol dehydrogenase 1B	1/4	7	7	127	126	7	**1.20**
					129	128	7	**1.11**
					131	130	6	**1.01**
**40**	ADH5 P11766: Alcohol dehydrogenase class-3		9	12	127	126	11	**2.09**
					129	128	12	**0.77**
					131	130	11	**1.16**

#	protein	group	peptides	spectra	ch1	ch2	quant	ratio
41	ADIPOQ Q15848: Adiponectin		4	8	127	126	8	**1.22**
					129	128	7	**1.23**
					131	130	8	**1.58**
42	AEBP1 Q8IUX7$_1$: Adipocyte enhancer-binding protein 1	1/2	38	85	127	126	79	**1.36**
					129	128	75	**1.24**
					131	130	81	**1.10**
43	AFM P43652: Afamin		32	78	127	126	72	**1.39**
					129	128	76	**0.85**
					131	130	76	**1.11**
44	AGRN O00468$_{3,6}$: Agrin [Cleaved into: Agrin N-terminal 110 kDa subunit; Agrin C-terminal 110 . . .	2/7	31	32	127	126	29	**0.49**
					129	128	26	**2.15**
					131	130	27	**0.84**
45	AGT P01019: Angiotensinogen		12	26	127	126	25	**0.25**
					129	128	21	**2.75**
					131	130	24	**1.00**
46	AHCY P23526$_{1,2}$: Adenosylhomocysteinase	2/2	2	2	127	126	2	**1.25**
					129	128	2	**0.75**
					131	130	2	**1.02**
47	AHCYL1 O43865$_1$: Adenosylhomocysteinase 2		2	2	127	126	2	**1.21**
					129	128	2	**0.93**
					131	130	2	**1.21**
48	AHNAK Q09666$_1$: Neuroblast differentiation-associated protein AHNAK	1/2	35	35	127	126	33	**1.76**
					129	128	33	**0.78**
					131	130	35	**1.66**
49	AHSA1 O95433$_1$: Activator of 90 kDa heat shock protein ATPase homolog 1	1/2	4	4	127	126	4	**0.79**
					129	128	4	**1.46**
					131	130	4	**1.01**
50	AHSG P02765: Alpha-2-HS-glycoprotein		13	385	127	126	365	**1.04**
					129	128	337	**1.59**
					131	130	361	**1.12**
51	AIFM1 O95831$_{1-3,5}$: Apoptosis-inducing factor 1, mitochondrial	4/4	1	1	127	126	1	**1.52**
					129	128	1	**1.05**
					131	130	1	**2.38**
52	AK1 P00568: Adenylate kinase isoenzyme 1		3	3	127	126	3	**0.94**
					129	128	3	**1.22**
					131	130	3	**1.05**

#	protein	group	peptides	spectra	ch1	ch2	quant	ratio
53	AKAP12 Q02952$_{1-3}$: A-kinase anchor protein 12	3/3	3	3	127	126	3	**2.21**
					129	128	3	**0.99**
					131	130	3	**1.08**
54	AKR7A2 O43488: Aflatoxin B1 aldehyde reductase member 2		3	4	127	126	4	**1.19**
					129	128	4	**1.00**
					131	130	4	**1.04**
55	ALB P02768$_1$: Serum albumin	1/3	65	3732	127	126	3363	**1.34**
					129	128	3041	**1.02**
					131	130	3229	**0.96**
56	ALDH1A1 P00352: Retinal dehydrogenase 1		5	5	127	126	2	**0.73**
					129	128	2	**1.43**
					131	130	2	**1.03**
57	ALDH1A2 O94788$_1$: Retinal dehydrogenase 2	1/4	10	13	127	126	9	**1.95**
					129	128	9	**0.71**
					131	130	10	**1.12**
58	ALDH1A3 P47895: Aldehyde dehydrogenase family 1 member A3	1/2	10	10	127	126	7	**1.67**
					129	128	7	**0.70**
					131	130	7	**1.18**
59	ALDH2 P05091$_1$: Aldehyde dehydrogenase, mitochondrial	1/2	4	4	127	126	1	**0.56**
					129	128	1	**2.12**
					131	130	1	**0.81**
60	ALDH7A1 P49419$_{1,2,4}$: Alpha-aminoadipic semialdehyde dehydrogenase	3/3	2	2	127	126	2	**1.03**
					129	128	2	**1.14**
					131	130	2	**1.14**
61	ALDH9A1 P49189$_1$: 4-trimethylaminobutyraldehyde dehydrogenase	1/2	6	8	127	126	7	**1.96**
					129	128	8	**0.86**
					131	130	8	**1.10**
62	ALDOA P04075$_{1,2}$: Fructose-bisphosphate aldolase A	2/3	8	11	127	126	9	**0.73**
					129	128	11	**1.77**
					131	130	10	**1.14**
63	ALOX15 P16050$_{1,2}$: Arachidonate 15-lipoxygenase	2/2	5	6	127	126	6	**1.09**
					129	128	5	**0.72**
					131	130	6	**0.89**
64	AMBP P02760: Protein AMBP [Cleaved into: Alpha-1-microglobulin		14	218	127	126	202	**0.48**
					129	128	192	**2.36**
					131	130	214	**0.96**

#	protein	group	peptides	spectra	ch1	ch2	quant	ratio
65	ANG P03950: Angiogenin		13	117	127	126	115	**1.52**
					129	128	115	**3.53**
					131	130	103	**0.97**
66	ANGPT2 $O15123_{1-3}$: Angiopoietin-2	3/3	2	3	127	126	3	**0.84**
					129	128	2	**2.22**
					131	130	3	**0.95**
67	ANGPTL1 O95841: Angiopoietin-related protein 1		3	4	127	126	4	**1.30**
					129	128	4	**0.97**
					131	130	4	**1.07**
68	ANGPTL2 $Q9UKU9_{1}$: Angiopoietin-related protein 2	1/2	11	16	127	126	13	**0.98**
					129	128	16	**0.88**
					131	130	14	**1.11**
69	ANGPTL3 Q9Y5C1: Angiopoietin-related protein 3		4	4	127	126	4	**1.08**
					129	128	4	**0.55**
					131	130	3	**0.94**
70	ANGPTL4 $Q9BY76_{1}$: Angiopoietin-related protein 4	1/3	12	27	127	126	27	**1.25**
					129	128	22	**1.38**
					131	130	25	**1.21**
71	ANK3 $Q12955_{3}$: Ankyrin-3		4	9	127	126	5	**0.47**
					129	128	5	**2.38**
					131	130	5	**0.86**
72	ANTXR1 $Q9H6X2_{1,5}$: Anthrax toxin receptor 1	2/6	13	21	127	126	18	**0.42**
					129	128	19	**2.20**
					131	130	19	**0.91**
73	ANTXR2 $P58335_{1,2}$: Anthrax toxin receptor 2	2/3	12	13	127	126	1	**0.84**
					129	128	1	**0.35**
					131	130	1	**0.92**
74	ANTXR2 $P58335_{4}$: Anthrax toxin receptor 2		12	13	127	126	1	**0.33**
					129	128	1	**1.97**
					131	130	1	**1.00**
75	ANXA1 P04083: Annexin A1		13	20	127	126	18	**1.27**
					129	128	18	**1.01**
					131	130	19	**1.22**
76	ANXA11 $P50995_{1,2}$: Annexin A11	2/2	3	4	127	126	4	**1.01**
					129	128	4	**2.02**
					131	130	4	**1.01**

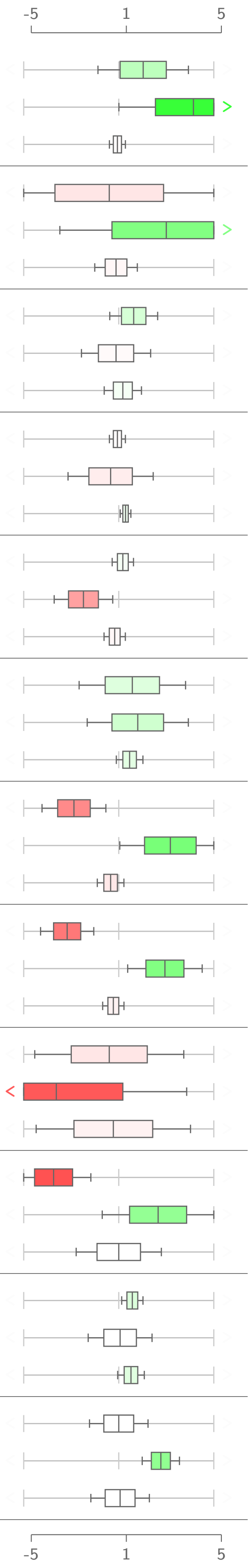

#	protein	group	peptides	spectra	ch1	ch2	quant	ratio
					127	126	31	**1.62**
**77**	ANXA2 P07355$_{1,2}$: Annexin A2	2/3	16	32	129	128	25	**0.72**
					131	130	30	**1.89**
					127	126	5	**1.14**
**78**	ANXA4 P09525$_{1}$: Annexin A4	1/2	6	7	129	128	5	**1.44**
					131	130	5	**1.37**
					127	126	4	**1.50**
**79**	ANXA5 P08758: Annexin A5		5	6	129	128	3	**0.81**
					131	130	4	**1.22**
					127	126	2	**2.34**
**80**	ANXA6 P08133$_{1,2}$: Annexin A6	2/2	2	2	129	128	2	**1.64**
					131	130	2	**1.09**
					127	126	4	**1.14**
**81**	ANXA7 P20073$_{1,2}$: Annexin A7	2/2	3	4	129	128	4	**1.68**
					131	130	4	**1.08**
					127	126	2	**3.13**
**82**	ANXA9 O76027: Annexin A9		1	2	129	128	2	**0.41**
					131	130	2	**1.30**
					127	126	7	**1.48**
**83**	AOX1 Q06278: Aldehyde oxidase		8	8	129	128	7	**1.53**
					131	130	8	**0.91**
					127	126	2	**0.54**
**84**	AP1B1 Q10567$_{1-4}$: AP-1 complex subunit beta-1	4/4	7	9	129	128	2	**1.09**
					131	130	2	**1.11**
					127	126	1	**0.87**
**85**	AP1G1 O43747$_{1,2}$: AP-1 complex subunit gamma-1	2/2	1	1	129	128	1	**1.29**
					131	130	1	**1.25**
					127	126	3	**0.89**
**86**	AP1M1 Q9BXS5$_{1,2}$: AP-1 complex subunit mu-1	2/2	2	3	129	128	3	**1.21**
					131	130	3	**1.01**
					127	126	3	**0.87**
**87**	AP2A1 O95782$_{1,2}$: AP-2 complex subunit alpha-1	2/5	5	5	129	128	5	**1.27**
					131	130	5	**0.99**
					127	126	1	**0.68**
**88**	AP2B1 P63010$_{1,2}$: AP-2 complex subunit beta	2/3	7	9	129	128	1	**1.25**
					131	130	1	**0.97**

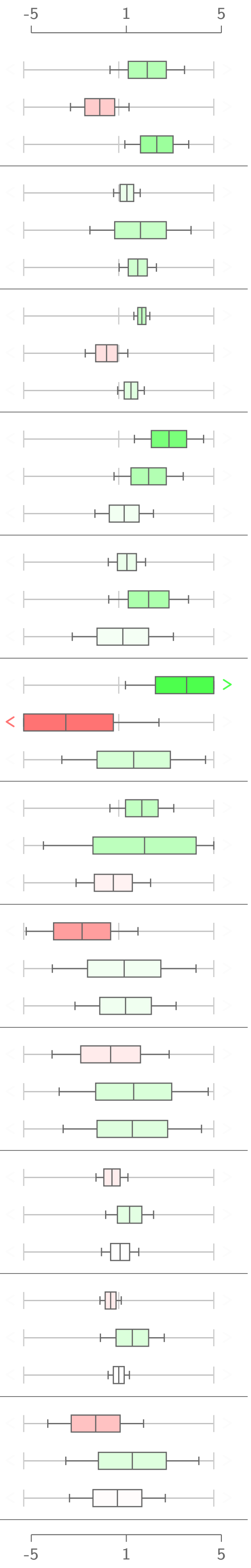

#	protein	group	peptides	spectra	ch1	ch2	quant	ratio
**89**	AP2M1 Q96CW1$_{1,2}$: AP-2 complex subunit mu	2/2	2	2	127	126	2	**1.00**
					129	128	2	**1.21**
					131	130	2	**1.19**
**90**	APCS P02743: Serum amyloid P-component		9	184	127	126	174	**1.32**
					129	128	174	**1.19**
					131	130	171	**1.01**
**91**	APEH P13798: Acylamino-acid-releasing enzyme		4	5	127	126	4	**1.91**
					129	128	4	**0.55**
					131	130	5	**1.18**
**92**	APLP2 Q06481$_{1,3,6}$: Amyloid-like protein 2	3/6	5	27	127	126	26	**1.03**
					129	128	20	**0.20**
					131	130	21	**1.09**
**93**	APMAP Q9HDC9$_{1}$: Adipocyte plasma membrane-associated protein	1/2	7	7	127	126	6	**0.73**
					129	128	5	**0.93**
					131	130	7	**0.99**
**94**	APOA1 P02647: Apolipoprotein A-I		35	2097	127	126	1969	**2.00**
					129	128	1958	**1.52**
					131	130	1879	**1.02**
**95**	APOA2 P02652: Apolipoprotein A-II		5	343	127	126	324	**1.69**
					129	128	298	**1.86**
					131	130	326	**0.99**
**96**	APOA4 P06727: Apolipoprotein A-IV		39	1659	127	126	1508	**2.13**
					129	128	1408	**0.85**
					131	130	1452	**1.12**
**97**	APOA5 Q6Q788: Apolipoprotein A-V		4	4	127	126	4	**0.78**
					129	128	3	**0.52**
					131	130	4	**0.87**
**98**	APOB P04114: Apolipoprotein B-100		270	1402	127	126	1339	**0.19**
					129	128	1199	**0.81**
					131	130	1370	**0.69**
**99**	APOBR Q0VD83$_{1,3,4}$: Apolipoprotein B receptor	3/3	5	5	127	126	5	**0.77**
					129	128	3	**1.45**
					131	130	4	**1.05**
**100**	APOC1 P02654: Apolipoprotein C-I		5	74	127	126	73	**1.84**
					129	128	72	**0.40**
					131	130	70	**0.95**

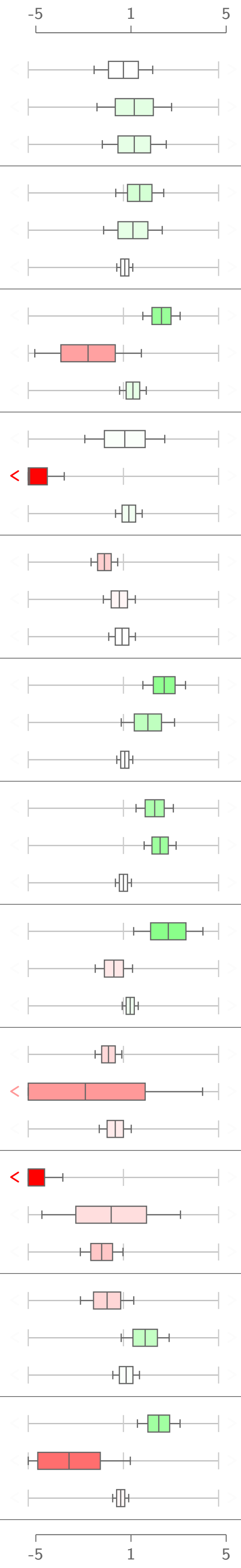

#	protein	group	peptides	spectra	ch1	ch2	quant	ratio
101	APOC2 P02655: Apolipoprotein C-II		4	24	127	126	18	**0.69**
					129	128	22	**0.44**
					131	130	22	**1.25**
102	APOC3 P02656: Apolipoprotein C-III		8	386	127	126	348	**1.08**
					129	128	339	**1.47**
					131	130	351	**1.25**
103	APOC4 P55056: Apolipoprotein C-IV		5	7	127	126	7	**0.65**
					129	128	7	**1.08**
					131	130	5	**0.76**
104	APOD P05090: Apolipoprotein D		8	245	127	126	239	**1.43**
					129	128	234	**0.71**
					131	130	208	**1.13**
105	APOE P02649: Apolipoprotein E		28	277	127	126	253	**1.04**
					129	128	254	**0.87**
					131	130	261	**0.96**
106	APOF Q13790: Apolipoprotein F		8	13	127	126	12	**0.85**
					129	128	12	**1.90**
					131	130	9	**1.03**
107	APOH P02749: Beta-2-glycoprotein 1		13	34	127	126	31	**1.53**
					129	128	32	**1.00**
					131	130	28	**1.28**
108	APOL1 $O14791_{1,2}$: Apolipoprotein L1	2/3	19	77	127	126	73	**0.35**
					129	128	71	**0.55**
					131	130	75	**0.74**
109	APOL2 Q9BQE5: Apolipoprotein L2		4	4	127	126	4	**0.87**
					129	128	4	**0.41**
					131	130	4	**0.77**
110	APOM $O95445_{1}$: Apolipoprotein M	1/2	10	31	127	126	25	**0.62**
					129	128	25	**1.02**
					131	130	26	**0.89**
111	APP $P05067_{1,7-9,11}$: Amyloid beta A4 protein	5/11	8	11	127	126	11	**1.48**
					129	128	8	**1.61**
					131	130	10	**1.12**
112	APRT $P07741_{1,2}$: Adenine phosphoribosyltransferase	2/2	4	5	127	126	5	**1.66**
					129	128	4	**0.63**
					131	130	5	**1.06**

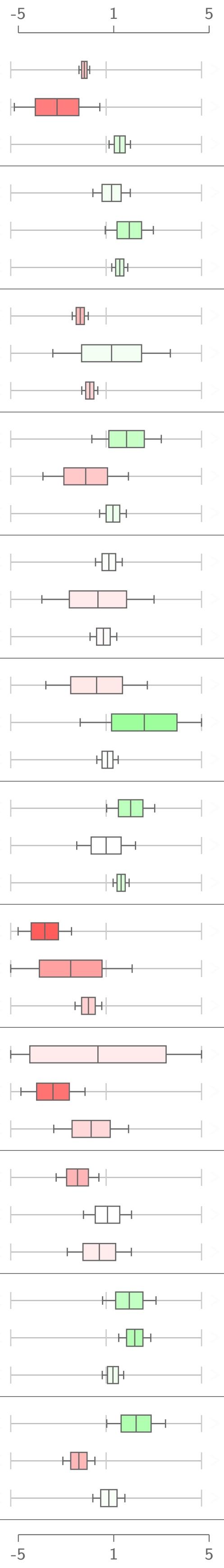

#	protein	group	peptides	spectra	ch1	ch2	quant	ratio
**113**	ARF1 P84077, ARF3 $P61204_{1,2}$: ADP-ribosylation factor 1, ADP-ribosylation factor 3	3/3	3	4	127	126	1	**0.93**
					129	128	1	**1.04**
					131	130	1	**0.80**
**114**	ARF4 P18085: ADP-ribosylation factor 4		3	4	127	126	3	**1.47**
					129	128	3	**1.08**
					131	130	3	**1.15**
**115**	ARF5 P84085: ADP-ribosylation factor 5		3	4	127	126	1	**1.76**
					129	128	1	**0.65**
					131	130	1	**1.20**
**116**	ARHGAP1 Q07960: Rho GTPase-activating protein 1		5	5	127	126	5	**0.75**
					129	128	3	**1.31**
					131	130	4	**0.99**
**117**	ARHGAP18 $Q8N392_{1}$: Rho GTPase-activating protein 18	1/2	2	2	127	126	2	**0.95**
					129	128	2	**1.28**
					131	130	2	**1.10**
**118**	ARHGAP35 Q9NRY4: Rho GTPase-activating protein 35		4	4	127	126	2	**0.95**
					129	128	2	**1.04**
					131	130	2	**0.90**
**119**	ARHGDIA $P52565_{1}$: Rho GDP-dissociation inhibitor 1	1/2	6	9	127	126	8	**0.55**
					129	128	6	**1.47**
					131	130	8	**1.15**
**120**	ARHGDIB P52566: Rho GDP-dissociation inhibitor 2		2	3	127	126	3	**0.60**
					129	128	3	**2.12**
					131	130	3	**1.19**
**121**	ARL3 P36405: ADP-ribosylation factor-like protein 3		2	2	127	126	2	**1.33**
					129	128	2	**1.05**
					131	130	2	**0.96**
**122**	ARPC1B O15143: Actin-related protein 2/3 complex subunit 1B		2	2	127	126	2	**0.91**
					129	128	2	**1.31**
					131	130	2	**1.16**
**123**	ARPC2 O15144: Actin-related protein 2/3 complex subunit 2		2	2	127	126	2	**0.93**
					129	128	2	**1.45**
					131	130	2	**1.14**
**124**	ASGR2 $P07307_{1-3}$: Asialoglycoprotein receptor 2	3/3	5	6	127	126	4	**1.15**
					129	128	6	**1.15**
					131	130	5	**1.15**

#	protein	group	peptides	spectra	ch1	ch2	quant	ratio
**125**	ASMTL O95671$_{1-3}$: N-acetylserotonin O-methyltransferase-like protein	3/3	2	2	127	126	2	**1.14**
					129	128	1	**1.97**
					131	130	2	**1.38**
**126**	ASPH Q12797$_{1,10}$: Aspartyl/asparaginyl beta-hydroxylase	2/2	10	11	127	126	11	**1.15**
					129	128	7	**1.18**
					131	130	9	**1.05**
**127**	ASPN Q9BXN1: Asporin		1	1	127	126	1	**0.91**
					129	128	1	**1.49**
					131	130	1	**1.03**
**128**	ATG3 Q9NT62$_{1,2}$: Ubiquitin-like-conjugating enzyme ATG3	2/2	1	3	127	126	3	**1.07**
					129	128	3	**0.87**
					131	130	3	**0.91**
**129**	ATIC P31939$_{1,2}$: Bifunctional purine biosynthesis protein PURH [Cleaved into: Bifunctional pu ...	2/2	5	6	127	126	6	**1.23**
					129	128	6	**0.99**
					131	130	6	**0.90**
**130**	ATL3 Q6DD88: Atlastin-3		3	3	127	126	3	**1.06**
					129	128	3	**1.17**
					131	130	3	**1.43**
**131**	ATP2A1 O14983$_{1}$: Sarcoplasmic/endoplasmic reticulum calcium ATPase 1		3	4	127	126	3	**0.92**
					129	128	3	**1.26**
					131	130	3	**0.91**
**132**	ATP2A2 P16615$_{1-5}$: Sarcoplasmic/endoplasmic reticulum calcium ATPase 2	5/5	2	2	127	126	1	**1.14**
					129	128	1	**1.27**
					131	130	1	**1.19**
**133**	ATP5A1 P25705$_{1,3}$: ATP synthase subunit alpha, mitochondrial	2/3	14	16	127	126	15	**1.94**
					129	128	13	**0.45**
					131	130	16	**1.46**
**134**	ATP5B P06576: ATP synthase subunit beta, mitochondrial		5	6	127	126	6	**1.81**
					129	128	6	**0.63**
					131	130	6	**1.44**
**135**	ATP5C1 P36542$_{1,2}$: ATP synthase subunit gamma, mitochondrial	2/2	4	7	127	126	3	**1.82**
					129	128	3	**0.71**
					131	130	3	**1.66**
**136**	ATP5I P56385: ATP synthase subunit e, mitochondrial		2	2	127	126	1	**2.83**
					129	128	1	**0.50**
					131	130	1	**2.17**

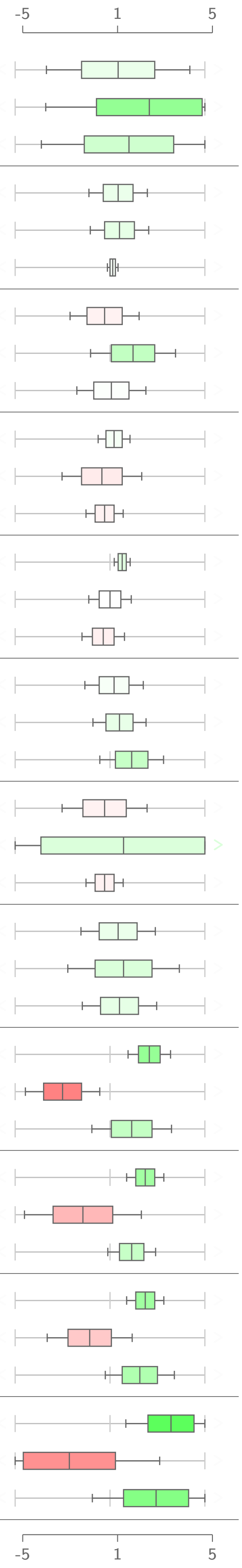

#	protein	group	peptides	spectra	ch1	ch2	quant	ratio
**137**	ATP6AP1 Q15904: V-type proton ATPase subunit S1		5	5	127	126	4	**1.07**
					129	128	5	**1.51**
					131	130	5	**1.14**
**138**	ATP6AP2 O75787$_{1,2}$: Renin receptor	2/2	8	8	127	126	6	**1.23**
					129	128	8	**0.58**
					131	130	7	**0.93**
**139**	ATP6V1B2 P21281: V-type proton ATPase subunit B, brain isoform	1/2	4	5	127	126	3	**1.26**
					129	128	3	**1.56**
					131	130	3	**0.99**
**140**	ATP6V1E1 P36543$_{1,3}$: V-type proton ATPase subunit E 1	2/3	3	4	127	126	4	**1.07**
					129	128	4	**0.89**
					131	130	4	**1.14**
**141**	AXL P30530$_{1,2}$: Tyrosine-protein kinase receptor UFO	2/2	2	2	127	126	2	**1.61**
					129	128	2	**1.39**
					131	130	2	**1.07**
**142**	AZGP1 P25311: Zinc-alpha-2-glycoprotein		9	10	127	126	9	**1.42**
					129	128	9	**1.40**
					131	130	9	**0.65**
**143**	B2M P61769: Beta-2-microglobulin [Cleaved into: Beta-2-microglobulin form pI 5.3]		5	29	127	126	29	**1.31**
					129	128	28	**2.81**
					131	130	27	**1.29**
**144**	B3GAT3 O94766$_{1}$: Galactosylgalactosylxylosylprotein 3-beta-glucuronosyltransferase 3	1/2	3	3	127	126	3	**0.68**
					129	128	3	**1.87**
					131	130	3	**0.91**
**145**	B3GNT2 Q9NY97$_{1,2}$: N-acetyllactosaminide beta-1,3-N-acetylglucosaminyltransferase 2	2/2	2	3	127	126	3	**0.54**
					129	128	3	**1.66**
					131	130	3	**0.94**
**146**	B3GNT7 Q8NFL0: UDP-GlcNAc:betaGal beta-1,3-N-acetylglucosaminyltransferase 7		2	2	127	126	2	**0.80**
					129	128	2	**1.50**
					131	130	2	**0.86**
**147**	B3GNT9 Q6UX72: UDP-GlcNAc:betaGal beta-1,3-N-acetylglucosaminyltransferase 9		2	2	127	126	2	**0.95**
					129	128	2	**1.91**
					131	130	2	**0.86**
**148**	B4GALT1 P15291$_{1,2}$: Beta-1,4-galactosyltransferase 1	2/2	11	21	127	126	21	**0.57**
					129	128	20	**2.59**
					131	130	21	**0.83**

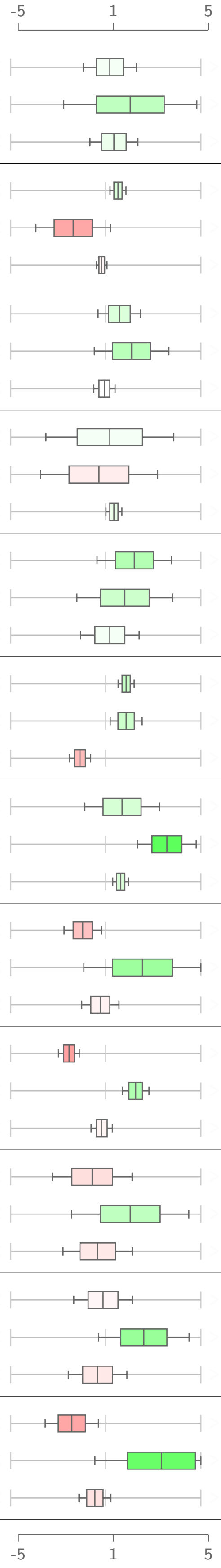

#	protein	group	peptides	spectra	ch1	ch2	quant	ratio
**149**	B4GALT4 O60513: Beta-1,4-galactosyltransferase 4		7	8	127	126	7	**0.64**
					129	128	8	**2.28**
					131	130	7	**0.90**
**150**	B4GAT1 O43505: Beta-1,4-glucuronyltransferase 1		6	8	127	126	6	**1.00**
					129	128	7	**1.90**
					131	130	7	**0.94**
**151**	BAMBI Q13145: BMP and activin membrane-bound inhibitor homolog		4	15	127	126	11	**0.98**
					129	128	4	**2.02**
					131	130	10	**0.94**
**152**	BANF1 O75531: Barrier-to-autointegration factor		2	2	127	126	2	**4.49**
					129	128	2	**0.66**
					131	130	2	**1.08**
**153**	BCAP31 P51572$_{1,2}$: B-cell receptor-associated protein 31	2/2	2	2	127	126	1	**1.39**
					129	128	1	**1.01**
					131	130	1	**1.69**
**154**	BCHE P06276: Cholinesterase		5	14	127	126	10	**0.80**
					129	128	7	**1.36**
					131	130	9	**0.92**
**155**	BDH2 Q9BUT1$_1$: 3-hydroxybutyrate dehydrogenase type 2	1/3	4	4	127	126	4	**1.95**
					129	128	4	**0.48**
					131	130	4	**1.33**
**156**	BGN P21810: Biglycan		10	19	127	126	11	**0.76**
					129	128	14	**2.53**
					131	130	14	**0.76**
**157**	BHLHB9 Q6PI77: Protein BHLHb9		3	11	127	126	3	**1.13**
					129	128	3	**0.98**
					131	130	4	**0.99**
**158**	BLVRB P30043: Flavin reductase		5	6	127	126	5	**0.91**
					129	128	6	**1.31**
					131	130	6	**1.11**
**159**	BMP1 P13497$_1$: Bone morphogenetic protein 1	1/6	17	22	127	126	15	**1.60**
					129	128	16	**0.60**
					131	130	16	**1.17**
**160**	BMP3 P12645: Bone morphogenetic protein 3		5	8	127	126	8	**1.63**
					129	128	8	**1.28**
					131	130	8	**0.97**

#	protein	group	peptides	spectra	ch1	ch2	quant	ratio
**161**	BPIFB1 Q8TDL5$_1$: BPI fold-containing family B member 1	1/2	7	8	127	126	6	**0.58**
					129	128	3	**0.24**
					131	130	4	**0.69**
**162**	BTD P43251$_{1-4}$: Biotinidase	4/4	3	4	127	126	4	**1.40**
					129	128	4	**0.63**
					131	130	4	**0.96**
**163**	BZW1 Q7L1Q6$_{1,3,4}$: Basic leucine zipper and W2 domain-containing protein 1	3/4	5	8	127	126	8	**1.44**
					129	128	7	**0.58**
					131	130	8	**1.20**
**164**	C1QA P02745: Complement C1q subcomponent subunit A		7	22	127	126	19	**1.15**
					129	128	20	**0.58**
					131	130	17	**1.23**
**165**	C1QB P02746: Complement C1q subcomponent subunit B		8	51	127	126	51	**1.07**
					129	128	47	**0.92**
					131	130	49	**1.10**
**166**	C1QBP Q07021: Complement component 1 Q subcomponent-binding protein, mitochondrial		1	1	127	126	1	**2.34**
					129	128	1	**0.68**
					131	130	1	**1.91**
**167**	C1QC P02747: Complement C1q subcomponent subunit C		5	16	127	126	14	**1.14**
					129	128	15	**1.00**
					131	130	13	**1.18**
**168**	C1QTNF4 Q9BXJ3: Complement C1q tumor necrosis factor-related protein 4		2	2	127	126	2	**1.28**
					129	128	2	**1.32**
					131	130	2	**1.04**
**169**	C1R P00736: Complement C1r subcomponent		32	181	127	126	170	**1.46**
					129	128	163	**0.86**
					131	130	169	**1.22**
**170**	C1RL Q9NZP8: Complement C1r subcomponent-like protein		12	25	127	126	15	**0.37**
					129	128	17	**2.15**
					131	130	14	**1.09**
**171**	C1S P09871: Complement C1s subcomponent		25	120	127	126	98	**1.64**
					129	128	90	**0.64**
					131	130	97	**1.12**
**172**	C2 P06681$_1$: Complement C2	1/3	32	58	127	126	58	**0.57**
					129	128	56	**1.47**
					131	130	55	**1.00**

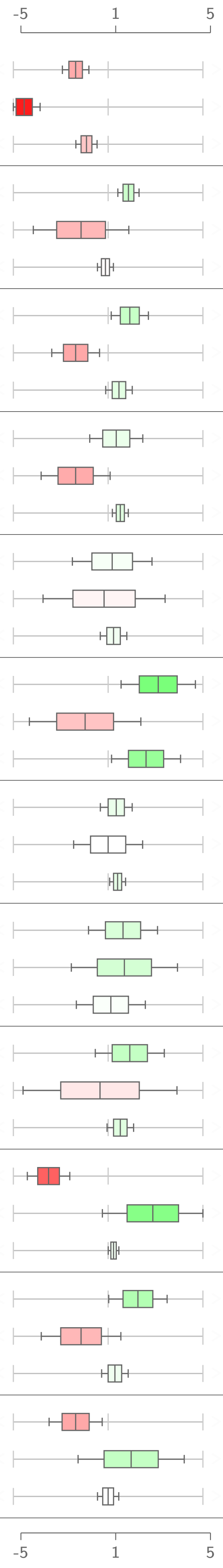

#	protein	group	peptides	spectra	ch1	ch2	quant	ratio
**173**	C2orf40 Q9H1Z8: Augurin		4	8	127	126	7	**1.17**
					129	128	6	**1.78**
					131	130	5	**1.35**
**174**	C3 P01024: Complement C3		137	3316	127	126	3133	**0.58**
					129	128	2802	**1.23**
					131	130	3021	**0.98**
**175**	C4A P0C0L4[1]: Complement C4-A	1/2	92	2178	127	126	16	**0.86**
					129	128	16	**0.80**
					131	130	13	**1.01**
**176**	C4B P0C0L5: Complement C4-B		93	2281	127	126	109	**0.86**
					129	128	116	**0.77**
					131	130	113	**0.96**
**177**	C4BPA P04003: C4b-binding protein alpha chain		33	565	127	126	522	**0.82**
					129	128	540	**5.22**
					131	130	534	**1.16**
**178**	C4BPB P20851[1,2]: C4b-binding protein beta chain	2/2	12	53	127	126	37	**0.82**
					129	128	43	**4.33**
					131	130	48	**1.17**
**179**	C5 P01031: Complement C5		74	223	127	126	200	**0.49**
					129	128	184	**0.89**
					131	130	207	**0.83**
**180**	C5orf47 Q569G3: Uncharacterized protein C5orf47		2	2	127	126	2	**0.81**
					129	128	2	**3.48**
					131	130	2	**0.94**
**181**	C6 P13671: Complement component C6		40	80	127	126	61	**0.73**
					129	128	58	**0.86**
					131	130	61	**1.01**
**182**	C7 P10643: Complement component C7		43	128	127	126	114	**0.54**
					129	128	112	**1.28**
					131	130	112	**0.96**
**183**	C8A P07357: Complement component C8 alpha chain		22	85	127	126	75	**0.56**
					129	128	71	**1.45**
					131	130	71	**1.00**
**184**	C8B P07358: Complement component C8 beta chain		29	91	127	126	88	**0.37**
					129	128	87	**2.02**
					131	130	86	**0.90**

#	protein	group	peptides	spectra	ch1	ch2	quant	ratio
185	C8G P07360: Complement component C8 gamma chain		11	43	127	126	41	**0.55**
					129	128	40	**1.19**
					131	130	43	**1.10**
186	C9 P02748: Complement component C9 [Cleaved into: Complement component C9a; Complement . . .		31	708	127	126	586	**0.30**
					129	128	563	**3.00**
					131	130	580	**0.89**
187	C9orf64 Q5T6V5: UPF0553 protein C9orf64		3	4	127	126	4	**0.76**
					129	128	4	**1.06**
					131	130	4	**0.80**
188	CACNA1B $Q00975_{2}$: Voltage-dependent N-type calcium channel subunit alpha-1B	1/2	4	6	127	126	5	**1.00**
					129	128	3	**0.78**
					131	130	5	**0.89**
189	CACNA1D $Q01668_{2}$: Voltage-dependent L-type calcium channel subunit alpha-1D	1/4	5	78	127	126	51	**0.57**
					129	128	30	**0.95**
					131	130	44	**0.92**
190	CALD1 $Q05682_{1-6}$: Caldesmon	6/6	4	5	127	126	4	**1.16**
					129	128	4	**0.90**
					131	130	4	**1.50**
191	CALM1 P62158: Calmodulin		8	29	127	126	22	**1.71**
					129	128	18	**0.75**
					131	130	22	**1.23**
192	CALML3 P27482: Calmodulin-like protein 3		6	11	127	126	4	**0.73**
					129	128	5	**3.60**
					131	130	5	**0.75**
193	CALR P27797: Calreticulin		20	45	127	126	37	**1.73**
					129	128	37	**0.80**
					131	130	39	**1.19**
194	CALU $O43852_{1,3,6,10}$: Calumenin	4/12	4	5	127	126	4	**2.20**
					129	128	4	**0.84**
					131	130	5	**1.17**
195	CAMK2B $Q13554_{3-5}$, CAMK2D $Q13557_{1,3-6,8-12}$, CAMK2G $Q13555_{1-4,7-10}$: Calcium/calmodulin-dependent protein kinase type II subunit beta, Calcium/calmodulin-dependent protein kinase type II subunit delta,	21/21	2	2	127	126	1	**1.28**
					129	128	1	**1.34**
					131	130	1	**1.02**
196	CAMP P49913: Cathelicidin antimicrobial peptide		3	3	127	126	3	**0.43**
					129	128	3	**1.05**
					131	130	3	**1.10**

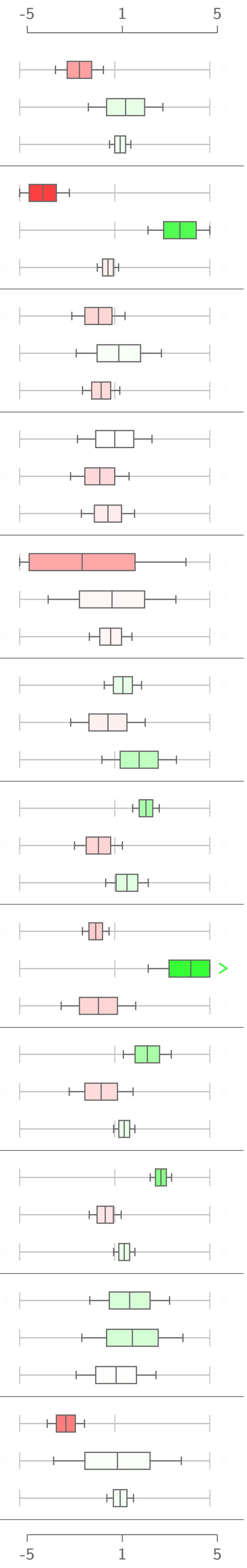

#	protein	group	peptides	spectra	ch1	ch2	quant	ratio
**197**	CAND1 Q86VP6$_1$: Cullin-associated NEDD8-dissociated protein 1	1/3	18	23	127	126	21	**0.87**
					129	128	20	**1.48**
					131	130	22	**1.03**
**198**	CANX P27824$_{1,2}$: Calnexin	2/3	10	13	127	126	12	**1.64**
					129	128	10	**0.91**
					131	130	12	**1.42**
**199**	CAP1 Q01518$_{1,2}$: Adenylyl cyclase-associated protein 1	2/2	14	18	127	126	16	**1.27**
					129	128	17	**1.05**
					131	130	17	**0.98**
**200**	CAP2 P40123$_1$: Adenylyl cyclase-associated protein 2		2	2	127	126	1	**1.16**
					129	128	1	**0.93**
					131	130	1	**0.97**
**201**	CAPG P40121$_{1,2}$: Macrophage-capping protein	2/2	6	9	127	126	7	**0.59**
					129	128	9	**1.66**
					131	130	9	**0.93**
**202**	CAPN1 P07384: Calpain-1 catalytic subunit		5	5	127	126	4	**0.97**
					129	128	4	**1.51**
					131	130	3	**0.94**
**203**	CAPN2 P17655$_1$: Calpain-2 catalytic subunit	1/2	9	9	127	126	8	**1.12**
					129	128	8	**0.87**
					131	130	7	**1.12**
**204**	CAPNS1 P04632: Calpain small subunit 1		6	7	127	126	7	**1.34**
					129	128	7	**0.85**
					131	130	7	**1.13**
**205**	CAPZA1 P52907: F-actin-capping protein subunit alpha-1		5	5	127	126	4	**1.26**
					129	128	4	**0.88**
					131	130	3	**0.97**
**206**	CAPZA2 P47755$_1$: F-actin-capping protein subunit alpha-2	1/2	3	3	127	126	2	**1.15**
					129	128	2	**0.81**
					131	130	2	**1.08**
**207**	CAPZB P47756$_2$: F-actin-capping protein subunit beta	1/2	3	3	127	126	3	**1.26**
					129	128	3	**0.66**
					131	130	3	**0.96**
**208**	CASP14 P31944: Caspase-14		4	4	127	126	4	**0.94**
					129	128	4	**3.51**
					131	130	4	**0.40**

#	protein	group	peptides	spectra	ch1	ch2	quant	ratio
**209**	CAST P20810$_{5,6,10}$: Calpastatin	3/10	9	12	127	126	12	**1.71**
					129	128	10	**0.56**
					131	130	11	**1.31**
**210**	CAV1 Q03135$_{1,2}$: Caveolin-1	2/2	2	2	127	126	2	**1.12**
					129	128	2	**2.64**
					131	130	2	**2.46**
**211**	CBR1 P16152$_{1}$: Carbonyl reductase [NADPH] 1		6	8	127	126	6	**0.52**
					129	128	8	**0.96**
					131	130	7	**0.96**
**212**	CBX3 Q13185: Chromobox protein homolog 3		2	2	127	126	2	**2.17**
					129	128	2	**0.76**
					131	130	2	**1.45**
**213**	CCAR2 Q8N163$_{1,2}$: Cell cycle and apoptosis regulator protein 2	2/2	4	4	127	126	3	**1.50**
					129	128	3	**1.08**
					131	130	4	**1.07**
**214**	CCDC80 Q76M96$_{1,2}$: Coiled-coil domain-containing protein 80	2/2	26	43	127	126	39	**0.92**
					129	128	34	**1.28**
					131	130	42	**1.35**
**215**	CCER2 I3L3R5: Coiled-coil domain-containing glutamate-rich protein 2		3	3	127	126	3	**1.41**
					129	128	3	**1.91**
					131	130	3	**1.02**
**216**	CCL14 Q16627$_{1}$: C-C motif chemokine 14	1/2	5	5	127	126	5	**2.03**
					129	128	5	**1.81**
					131	130	3	**0.99**
**217**	CCL16 O15467: C-C motif chemokine 16		3	3	127	126	3	**1.00**
					129	128	3	**1.73**
					131	130	3	**0.86**
**218**	CCL18 P55774: C-C motif chemokine 18		3	6	127	126	5	**3.35**
					129	128	6	**1.62**
					131	130	6	**0.77**
**219**	CCT2 P78371$_{1}$: T-complex protein 1 subunit beta	1/2	7	8	127	126	7	**1.18**
					129	128	8	**1.13**
					131	130	7	**1.06**
**220**	CCT3 P49368$_{1}$: T-complex protein 1 subunit gamma	1/2	3	4	127	126	3	**1.07**
					129	128	3	**1.03**
					131	130	4	**1.08**

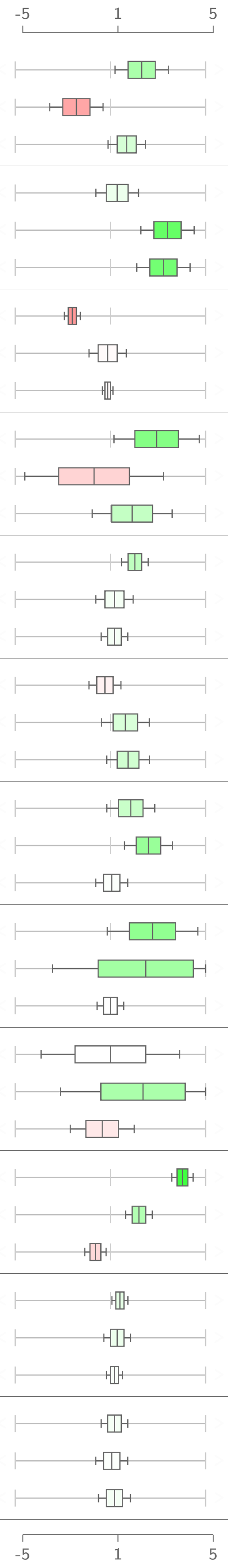

#	protein	group	peptides	spectra	ch1	ch2	quant	ratio
**221**	CCT4 P50991$_{1,2}$: T-complex protein 1 subunit delta	2/2	6	6	127	126	6	**1.05**
					129	128	6	**1.02**
					131	130	4	**1.07**
**222**	CCT5 P48643$_{1,2}$: T-complex protein 1 subunit epsilon	2/2	6	7	127	126	7	**1.17**
					129	128	6	**0.93**
					131	130	7	**1.04**
**223**	CCT6A P40227$_{1}$: T-complex protein 1 subunit zeta	1/5	6	6	127	126	6	**1.17**
					129	128	4	**0.92**
					131	130	6	**1.01**
**224**	CCT8 P50990$_{1,2}$: T-complex protein 1 subunit theta	2/3	5	5	127	126	4	**1.11**
					129	128	4	**1.07**
					131	130	5	**1.07**
**225**	CD109 Q6YHK3$_{1,2}$: CD109 antigen	2/4	12	12	127	126	11	**0.93**
					129	128	11	**0.98**
					131	130	12	**0.99**
**226**	CD14 P08571: Monocyte differentiation antigen CD14		17	157	127	126	156	**0.52**
					129	128	155	**2.72**
					131	130	150	**1.00**
**227**	CD151 P48509: CD151 antigen		3	3	127	126	3	**0.64**
					129	128	3	**3.79**
					131	130	3	**1.18**
**228**	CD163 Q86VB7$_{1-3}$: Scavenger receptor cysteine-rich type 1 protein M130	3/4	21	26	127	126	24	**1.54**
					129	128	26	**1.09**
					131	130	24	**1.14**
**229**	CD163L1 Q9NR16$_{1,2,4}$: Scavenger receptor cysteine-rich type 1 protein M160	3/3	2	2	127	126	2	**1.57**
					129	128	2	**1.07**
					131	130	2	**1.28**
**230**	CD248 Q9HCU0$_{1}$: Endosialin		4	6	127	126	4	**1.37**
					129	128	5	**0.97**
					131	130	6	**1.01**
**231**	CD302 Q8IX05$_{1}$, LY75 O60449$_{2,3}$: CD302 antigen, Lymphocyte antigen 75	3/3	3	5	127	126	4	**1.08**
					129	128	4	**1.23**
					131	130	4	**1.14**
**232**	CD5L O43866: CD5 antigen-like		19	34	127	126	31	**1.21**
					129	128	30	**0.74**
					131	130	32	**1.00**

#	protein	group	peptides	spectra	ch1	ch2	quant	ratio
**233**	CD74 P04233$_{1,2}$: HLA class II histocompatibility antigen gamma chain	2/2	2	4	127	126	3	**1.36**
					129	128	3	**2.27**
					131	130	3	**0.97**
**234**	CD81 P60033: CD81 antigen		2	3	127	126	3	**0.35**
					129	128	3	**6.56**
					131	130	3	**1.01**
**235**	CD8A P01732$_{1-3}$: T-cell surface glycoprotein CD8 alpha chain	3/3	1	1	127	126	1	**0.41**
					129	128	1	**1.89**
					131	130	1	**0.70**
**236**	CD9 P21926: CD9 antigen		3	3	127	126	3	**0.37**
					129	128	3	**6.31**
					131	130	3	**1.10**
**237**	CD97 P48960$_{1-3}$: CD97 antigen	3/3	3	4	127	126	4	**1.20**
					129	128	4	**1.11**
					131	130	4	**1.15**
**238**	CD99 P14209$_{1,3}$: CD99 antigen	2/2	1	1	127	126	1	**0.89**
					129	128	1	**1.06**
					131	130	1	**0.93**
**239**	CDC37 Q16543: Hsp90 co-chaperone Cdc37		3	6	127	126	5	**1.76**
					129	128	4	**0.79**
					131	130	6	**1.25**
**240**	CDC42 P60953$_{1,2}$: Cell division control protein 42 homolog	2/6	3	3	127	126	2	**1.07**
					129	128	2	**0.96**
					131	130	2	**1.20**
**241**	CDH1 P12830$_{1,2}$: Cadherin-1	2/2	3	6	127	126	5	**1.80**
					129	128	6	**1.47**
					131	130	6	**1.04**
**242**	CDH2 P19022$_{1}$: Cadherin-2	1/2	7	12	127	126	11	**1.27**
					129	128	11	**1.88**
					131	130	12	**1.14**
**243**	CELF2 O95319$_{1-5}$: CUGBP Elav-like family member 2	5/5	2	2	127	126	2	**1.82**
					129	128	2	**0.76**
					131	130	2	**1.31**
**244**	CENPK Q9BS16: Centromere protein K		3	17	127	126	9	**0.93**
					129	128	10	**0.77**
					131	130	8	**0.87**

#	protein	group	peptides	spectra	ch1	ch2	quant	ratio
**245**	CEP350 Q5VT06: Centrosome-associated protein 350		3	11	127	126	2	**1.37**
					129	128	2	**0.60**
					131	130	2	**1.07**
**246**	CETP $P11597_1$: Cholesteryl ester transfer protein	1/2	15	48	127	126	46	**0.53**
					129	128	43	**2.39**
					131	130	46	**0.85**
**247**	CFB $P00751_1$: Complement factor B	1/2	29	56	127	126	56	**0.66**
					129	128	54	**1.05**
					131	130	55	**1.11**
**248**	CFD P00746: Complement factor D		17	105	127	126	100	**0.75**
					129	128	105	**1.42**
					131	130	102	**1.01**
**249**	CFH $P08603_1$: Complement factor H	1/2	67	612	127	126	445	**1.43**
					129	128	412	**0.54**
					131	130	437	**1.04**
**250**	CFHR1 Q03591: Complement factor H-related protein 1		13	192	127	126	12	**1.53**
					129	128	10	**0.97**
					131	130	10	**1.06**
**251**	CFHR2 $P36980_1$: Complement factor H-related protein 2	1/2	13	55	127	126	13	**1.13**
					129	128	13	**1.30**
					131	130	8	**1.05**
**252**	CFHR3 $Q02985_{1,2}$: Complement factor H-related protein 3	2/2	8	51	127	126	8	**1.18**
					129	128	8	**1.34**
					131	130	7	**1.10**
**253**	CFHR4 $Q92496_{1,2}$: Complement factor H-related protein 4	2/3	9	20	127	126	4	**1.12**
					129	128	3	**0.84**
					131	130	4	**1.12**
**254**	CFHR5 Q9BXR6: Complement factor H-related protein 5		23	73	127	126	38	**1.17**
					129	128	35	**0.68**
					131	130	38	**1.11**
**255**	CFI P05156: Complement factor I		21	60	127	126	52	**1.24**
					129	128	48	**1.00**
					131	130	51	**1.24**
**256**	CFL1 P23528: Cofilin-1	1/3	7	8	127	126	6	**1.50**
					129	128	7	**1.37**
					131	130	7	**1.02**

#	protein	group	peptides	spectra	ch1	ch2	quant	ratio
**257**	CFP P27918: Properdin		16	22	127	126	18	**0.77**
					129	128	20	**0.41**
					131	130	19	**1.05**
**258**	CGREF1 Q99674$_{1,4,5}$: Cell growth regulator with EF hand domain protein 1	3/3	10	12	127	126	11	**1.31**
					129	128	10	**1.25**
					131	130	11	**1.04**
**259**	CHGA P10645: Chromogranin-A		29	108	127	126	101	**1.15**
					129	128	101	**1.35**
					131	130	107	**1.17**
**260**	CHGB P05060: Secretogranin-1		27	65	127	126	50	**1.58**
					129	128	51	**1.81**
					131	130	52	**1.17**
**261**	CHMP2A O43633: Charged multivesicular body protein 2a		2	2	127	126	1	**1.70**
					129	128	1	**1.21**
					131	130	2	**1.12**
**262**	CHRD Q9H2X0$_{1,5}$: Chordin	2/3	16	22	127	126	21	**1.19**
					129	128	20	**2.21**
					131	130	22	**1.07**
**263**	CHRDL1 Q9BU40$_{2,4}$: Chordin-like protein 1	2/6	21	52	127	126	51	**1.08**
					129	128	50	**1.08**
					131	130	48	**1.14**
**264**	CHRDL2 Q6WN34$_{1,2}$: Chordin-like protein 2	2/2	4	5	127	126	5	**1.19**
					129	128	4	**1.03**
					131	130	4	**1.37**
**265**	CHST11 Q9NPF2$_{1,2}$: Carbohydrate sulfotransferase 11	2/2	2	2	127	126	1	**1.38**
					129	128	1	**2.10**
					131	130	1	**1.30**
**266**	CHST12 Q9NRB3: Carbohydrate sulfotransferase 12		2	2	127	126	1	**1.37**
					129	128	1	**1.78**
					131	130	1	**1.02**
**267**	CHST14 Q8NCH0: Carbohydrate sulfotransferase 14		6	9	127	126	9	**0.80**
					129	128	9	**2.43**
					131	130	7	**0.75**
**268**	CHST15 Q7LFX5$_{1}$: Carbohydrate sulfotransferase 15	1/2	3	3	127	126	3	**0.62**
					129	128	3	**2.08**
					131	130	3	**0.97**

#	protein	group	peptides	spectra	ch1	ch2	quant	ratio
**269**	CILP O75339: Cartilage intermediate layer protein 1		52	106	127	126	99	**1.76**
					129	128	92	**0.76**
					131	130	98	**1.06**
**270**	CILP2 Q8IUL8: Cartilage intermediate layer protein 2		2	3	127	126	1	**1.61**
					129	128	1	**1.70**
					131	130	1	**0.93**
**271**	CKAP4 Q07065: Cytoskeleton-associated protein 4		9	11	127	126	9	**1.44**
					129	128	9	**0.72**
					131	130	9	**1.59**
**272**	CKB P12277: Creatine kinase B-type		7	7	127	126	7	**1.22**
					129	128	7	**1.09**
					131	130	6	**1.83**
**273**	CLC Q05315: Galectin-10		4	5	127	126	5	**1.25**
					129	128	4	**1.06**
					131	130	4	**0.98**
**274**	CLEC11A Q9Y240: C-type lectin domain family 11 member A		14	26	127	126	25	**1.40**
					129	128	22	**1.02**
					131	130	24	**1.03**
**275**	CLEC3B P05452: Tetranectin		8	11	127	126	11	**1.21**
					129	128	8	**2.99**
					131	130	9	**1.19**
**276**	CLGN O14967$_1$: Calmegin		2	2	127	126	1	**0.75**
					129	128	1	**0.89**
					131	130	1	**0.94**
**277**	CLIC1 O00299: Chloride intracellular channel protein 1		10	13	127	126	12	**1.48**
					129	128	11	**0.90**
					131	130	12	**1.08**
**278**	CLIC4 Q9Y696: Chloride intracellular channel protein 4		5	6	127	126	4	**0.86**
					129	128	4	**1.78**
					131	130	4	**1.34**
**279**	CLIC5 Q9NZA1$_1$: Chloride intracellular channel protein 5	1/3	3	6	127	126	5	**1.10**
					129	128	3	**0.70**
					131	130	4	**1.01**
**280**	CLSTN1 O94985$_1$: Calsyntenin-1		17	21	127	126	1	**1.41**
					129	128	1	**1.13**
					131	130	1	**1.02**

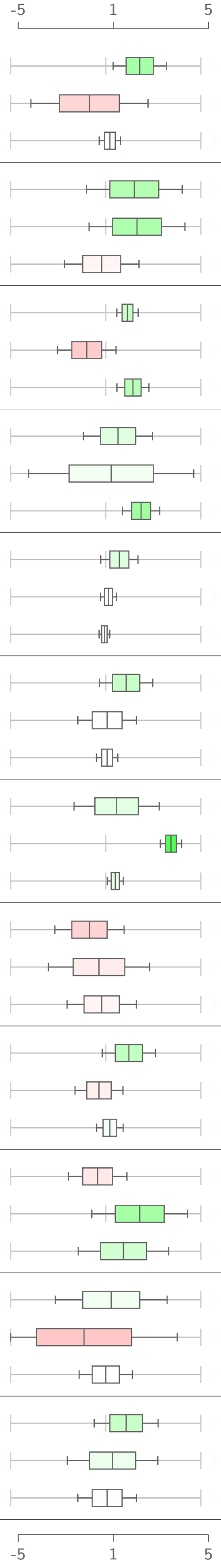

#	protein	group	peptides	spectra	ch1	ch2	quant	ratio
**281**	CLSTN1 O94985$_2$: Calsyntenin-1		17	21	127	126	1	**2.11**
					129	128	1	**0.43**
					131	130	1	**1.13**
**282**	CLSTN3 Q9BQT9$_{1,2}$: Calsyntenin-3	2/2	3	3	127	126	3	**0.90**
					129	128	3	**2.25**
					131	130	3	**1.17**
**283**	CLTB P09497$_{1,2}$: Clathrin light chain B	2/2	2	2	127	126	2	**2.26**
					129	128	2	**0.76**
					131	130	2	**1.33**
**284**	CLTC Q00610$_{1,2}$: Clathrin heavy chain 1	2/4	22	26	127	126	22	**0.88**
					129	128	24	**1.38**
					131	130	22	**1.20**
**285**	CLU P10909$_4$: Clusterin	1/5	26	608	127	126	562	**1.26**
					129	128	574	**0.59**
					131	130	580	**1.08**
**286**	CNDP1 Q96KN2: Beta-Ala-His dipeptidase		8	10	127	126	10	**1.18**
					129	128	10	**0.65**
					131	130	9	**0.80**
**287**	CNN1 P51911$_{1,2}$: Calponin-1	2/2	3	3	127	126	2	**1.23**
					129	128	2	**0.98**
					131	130	2	**1.03**
**288**	CNN3 Q15417$_{1,3}$: Calponin-3	2/3	8	10	127	126	8	**2.32**
					129	128	9	**0.64**
					131	130	9	**1.39**
**289**	COL12A1 Q99715$_1$: Collagen alpha-1(XII) chain	1/3	61	90	127	126	86	**0.68**
					129	128	86	**1.82**
					131	130	83	**1.04**
**290**	COL14A1 Q05707$_{1,2}$: Collagen alpha-1(XIV) chain	2/3	50	82	127	126	75	**0.82**
					129	128	77	**1.20**
					131	130	80	**1.06**
**291**	COL15A1 P39059: Collagen alpha-1(XV) chain [Cleaved into: Restin		5	5	127	126	5	**0.74**
					129	128	5	**0.92**
					131	130	5	**1.00**
**292**	COL16A1 Q07092$_{1,2}$: Collagen alpha-1(XVI) chain	2/2	3	5	127	126	5	**0.62**
					129	128	3	**2.22**
					131	130	4	**0.98**

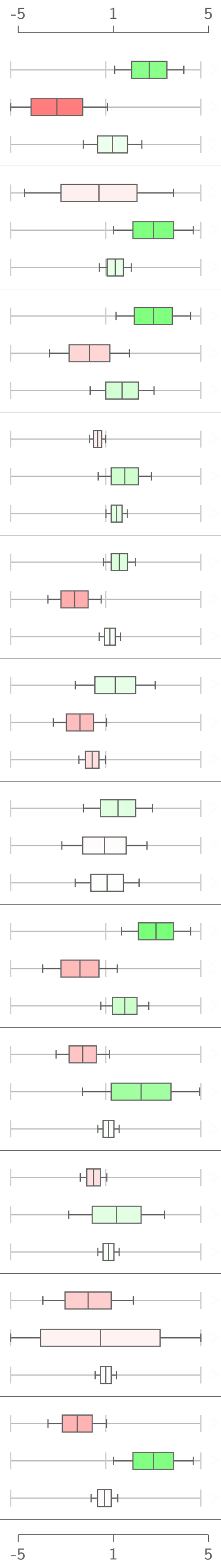

#	protein	group	peptides	spectra	ch1	ch2	quant	ratio
**293**	COL18A1 P39060$_{1-3}$: Collagen alpha-1(XVIII) chain [Cleaved into: Endostatin]	3/3	13	27	127	126	27	**0.96**
					129	128	25	**1.26**
					131	130	24	**1.10**
**294**	COL1A1 P02452: Collagen alpha-1(I) chain		24	70	127	126	58	**1.78**
					129	128	56	**2.22**
					131	130	58	**1.19**
**295**	COL1A2 P08123: Collagen alpha-2(I) chain		22	31	127	126	28	**1.25**
					129	128	30	**2.32**
					131	130	29	**1.06**
**296**	COL2A1 P02458$_2$: Collagen alpha-1(II) chain		3	10	127	126	1	**0.70**
					129	128	1	**0.48**
					131	130	1	**1.00**
**297**	COL3A1 P02461$_1$: Collagen alpha-1(III) chain	1/2	15	36	127	126	30	**0.92**
					129	128	28	**1.55**
					131	130	30	**1.18**
**298**	COL4A2 P08572: Collagen alpha-2(IV) chain [Cleaved into: Canstatin]		2	2	127	126	2	**0.91**
					129	128	2	**1.37**
					131	130	2	**1.26**
**299**	COL4A3BP Q9Y5P4$_{1-3}$: Collagen type IV alpha-3-binding protein	3/3	1	1	127	126	1	**1.22**
					129	128	1	**1.14**
					131	130	1	**1.21**
**300**	COL5A1 P20908: Collagen alpha-1(V) chain		10	19	127	126	19	**1.50**
					129	128	19	**0.94**
					131	130	19	**1.08**
**301**	COL5A2 P05997: Collagen alpha-2(V) chain		14	20	127	126	16	**1.66**
					129	128	17	**1.17**
					131	130	17	**1.08**
**302**	COL6A1 P12109: Collagen alpha-1(VI) chain		27	56	127	126	51	**0.40**
					129	128	53	**2.06**
					131	130	51	**0.91**
**303**	COL6A2 P12110$_1$: Collagen alpha-2(VI) chain	1/3	23	175	127	126	143	**0.84**
					129	128	123	**1.49**
					131	130	157	**0.93**
**304**	COL6A3 P12111$_1$: Collagen alpha-3(VI) chain	1/4	119	261	127	126	17	**0.55**
					129	128	17	**0.93**
					131	130	17	**0.83**

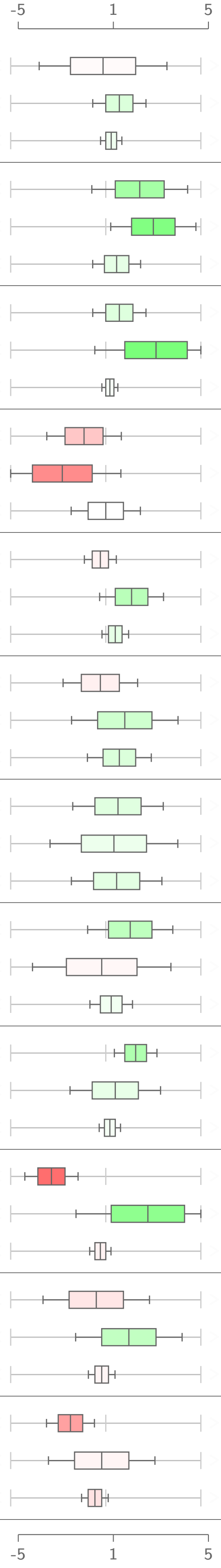

#	protein	group	peptides	spectra	ch1	ch2	quant	ratio
**305**	COL6A3 P12111$_4$: Collagen alpha-3(VI) chain		108	250	127	126	6	**0.63**
					129	128	6	**0.58**
					131	130	5	**0.70**
**306**	COL6A6 A6NMZ7$_1$: Collagen alpha-6(VI) chain		3	5	127	126	5	**0.61**
					129	128	3	**3.06**
					131	130	5	**1.06**
**307**	COL7A1 Q02388$_{1,2}$: Collagen alpha-1(VII) chain	2/2	3	3	127	126	2	**0.91**
					129	128	2	**2.78**
					131	130	2	**1.09**
**308**	COL8A1 P27658: Collagen alpha-1(VIII) chain		2	2	127	126	1	**1.41**
					129	128	1	**3.90**
					131	130	1	**1.61**
**309**	COLEC10 Q9Y6Z7: Collectin-10		3	3	127	126	3	**1.06**
					129	128	3	**1.40**
					131	130	3	**1.11**
**310**	COLGALT1 Q8NBJ5: Procollagen galactosyltransferase 1		1	1	127	126	1	**0.77**
					129	128	1	**1.05**
					131	130	1	**0.51**
**311**	COMP P49747$_1$: Cartilage oligomeric matrix protein	1/2	28	59	127	126	48	**1.54**
					129	128	49	**0.84**
					131	130	50	**1.05**
**312**	COPA P53621$_{1,2}$: Coatomer subunit alpha	2/2	4	4	127	126	4	**1.16**
					129	128	4	**1.07**
					131	130	4	**1.32**
**313**	COPB1 P53618: Coatomer subunit beta		10	11	127	126	9	**1.04**
					129	128	11	**1.54**
					131	130	10	**1.15**
**314**	COPB2 P35606$_{1,2}$: Coatomer subunit beta'	2/2	3	3	127	126	3	**1.39**
					129	128	3	**0.93**
					131	130	3	**1.29**
**315**	COPE O14579$_{1,3}$: Coatomer subunit epsilon	2/3	2	2	127	126	2	**2.33**
					129	128	2	**0.40**
					131	130	2	**1.29**
**316**	COPG1 Q9Y678: Coatomer subunit gamma-1		7	7	127	126	7	**1.10**
					129	128	7	**1.22**
					131	130	6	**1.15**

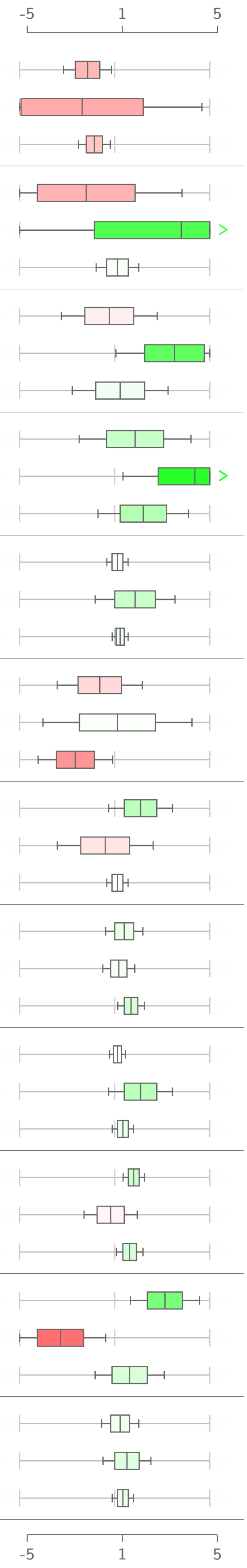

#	protein	group	peptides	spectra	ch1	ch2	quant	ratio
317	COPS4 Q9BT78$_{1,2}$: COP9 signalosome complex subunit 4	2/2	1	1	127	126	1	**1.52**
					129	128	1	**0.66**
					131	130	1	**1.08**
318	COPZ1 P61923$_{1,4,5}$: Coatomer subunit zeta-1	3/3	1	1	127	126	1	**1.18**
					129	128	1	**0.80**
					131	130	1	**1.00**
319	CORO1A P31146: Coronin-1A	1/6	7	8	127	126	5	**0.80**
					129	128	6	**1.47**
					131	130	6	**1.04**
320	CORO1B Q9BR76: Coronin-1B		4	5	127	126	4	**0.99**
					129	128	4	**0.56**
					131	130	4	**0.91**
321	CORO1C Q9ULV4$_{1-3}$: Coronin-1C	3/3	6	7	127	126	5	**0.94**
					129	128	4	**1.53**
					131	130	3	**1.09**
322	CORO7 P57737$_{3}$: Coronin-7		3	3	127	126	3	**0.90**
					129	128	3	**0.24**
					131	130	3	**0.68**
323	COTL1 Q14019: Coactosin-like protein		2	3	127	126	3	**0.63**
					129	128	3	**2.63**
					131	130	3	**1.04**
324	CP P00450: Ceruloplasmin		39	101	127	126	93	**1.53**
					129	128	95	**0.96**
					131	130	95	**1.03**
325	CPA4 Q9UI42$_{1,2}$: Carboxypeptidase A4	2/2	1	1	127	126	1	**0.96**
					129	128	1	**4.33**
					131	130	1	**0.98**
326	CPB2 Q96IY4$_{1}$: Carboxypeptidase B2	1/2	11	19	127	126	19	**0.77**
					129	128	17	**1.45**
					131	130	19	**0.88**
327	CPE P16870$_{1,2}$: Carboxypeptidase E	2/2	27	98	127	126	82	**0.72**
					129	128	92	**2.11**
					131	130	93	**0.78**
328	CPN1 P15169: Carboxypeptidase N catalytic chain		15	28	127	126	27	**1.25**
					129	128	28	**0.78**
					131	130	27	**0.97**

#	protein	group	peptides	spectra	ch1	ch2	quant	ratio
**329**	CPN2 P22792: Carboxypeptidase N subunit 2		14	36	127	126	34	**0.45**
					129	128	31	**1.52**
					131	130	33	**1.00**
**330**	CPNE1 Q99829: Copine-1		2	2	127	126	2	**0.90**
					129	128	2	**1.49**
					131	130	2	**1.05**
**331**	CPQ Q9Y646: Carboxypeptidase Q		6	6	127	126	4	**0.82**
					129	128	6	**1.52**
					131	130	6	**0.93**
**332**	CPSF1 Q10570: Cleavage and polyadenylation specificity factor subunit 1		2	3	127	126		
					129	128		
					131	130	1	**0.64**
**333**	CPXM1 Q96SM3: Probable carboxypeptidase X1		11	12	127	126	11	**1.42**
					129	128	11	**0.94**
					131	130	11	**1.08**
**334**	CPXM2 Q8N436: Inactive carboxypeptidase-like protein X2		16	21	127	126	20	**1.47**
					129	128	18	**0.94**
					131	130	19	**1.41**
**335**	CPZ Q66K79$_{1,2}$: Carboxypeptidase Z	2/3	7	8	127	126	7	**1.18**
					129	128	6	**0.99**
					131	130	8	**1.29**
**336**	CRABP2 P29373: Cellular retinoic acid-binding protein 2		6	8	127	126	8	**0.68**
					129	128	8	**1.72**
					131	130	7	**1.03**
**337**	CRHBP P24387: Corticotropin-releasing factor-binding protein		5	6	127	126	6	**0.90**
					129	128	6	**1.44**
					131	130	6	**1.03**
**338**	CRIP1 P50238: Cysteine-rich protein 1		2	2	127	126	1	**1.66**
					129	128	1	**5.55**
					131	130	1	**0.95**
**339**	CRIP2 P52943$_1$: Cysteine-rich protein 2	1/2	3	3	127	126	2	**1.24**
					129	128	2	**1.32**
					131	130	2	**1.09**
**341**	CRP P02741$_1$: C-reactive protein [Cleaved into: C-reactive protein(1-205)]	1/2	8	35	127	126	34	**0.79**
					129	128	35	**1.45**
					131	130	33	**1.11**

-5 1 5

#	protein	group	peptides	spectra	ch1	ch2	quant	ratio
**342**	CRTAC1 Q9NQ79$_1$: Cartilage acidic protein 1	1/3	6	7	127	126	6	**0.81**
					129	128	6	**0.87**
					131	130	6	**0.98**
**343**	CRYAB P02511: Alpha-crystallin B chain		2	2	127	126	2	**2.22**
					129	128	2	**0.97**
					131	130	2	**1.24**
**344**	CRYL1 Q9Y2S2$_{1,2}$: Lambda-crystallin homolog	2/2	2	2	127	126	2	**1.21**
					129	128	2	**1.07**
					131	130	2	**0.99**
**345**	CSDE1 O75534$_{1-4}$: Cold shock domain-containing protein E1	4/4	1	1	127	126	1	**1.97**
					129	128	1	**0.96**
					131	130	1	**1.02**
**346**	CSE1L P55060$_{1,3}$: Exportin-2	2/4	7	7	127	126	6	**1.07**
					129	128	5	**1.32**
					131	130	7	**1.01**
**347**	CSGALNACT1 Q8TDX6$_1$: Chondroitin sulfate N-acetylgalactosaminyltransferase 1		2	2	127	126	1	**0.57**
					129	128	1	**0.72**
					131	130	1	**0.60**
**348**	CSPG4 Q6UVK1: Chondroitin sulfate proteoglycan 4		11	11	127	126	11	**0.70**
					129	128	10	**1.89**
					131	130	11	**1.15**
**349**	CST3 P01034: Cystatin-C		6	32	127	126	27	**0.94**
					129	128	29	**2.00**
					131	130	28	**1.05**
**350**	CST6 Q15828: Cystatin-M		8	16	127	126	15	**0.92**
					129	128	11	**1.53**
					131	130	14	**1.23**
**351**	CSTB P04080: Cystatin-B		2	2	127	126	2	**0.65**
					129	128	2	**4.46**
					131	130	2	**1.02**
**352**	CTGF P29279$_{1,2}$: Connective tissue growth factor	2/2	5	7	127	126	4	**2.00**
					129	128	4	**2.01**
					131	130	5	**1.27**
**353**	CTNNA1 P35221$_{1,2}$: Catenin alpha-1	2/9	11	11	127	126	11	**1.04**
					129	128	10	**1.33**
					131	130	11	**1.05**

#	protein	group	peptides	spectra	ch1	ch2	quant	ratio
**354**	CTSA P10619$_{1,2}$: Lysosomal protective protein	2/2	3	3	127	126	3	**0.90**
					129	128	3	**1.55**
					131	130	3	**0.90**
**355**	CTSB P07858: Cathepsin B		4	10	127	126	10	**1.28**
					129	128	10	**1.48**
					131	130	9	**0.90**
**356**	CTSC P53634$_{1}$: Dipeptidyl peptidase 1		2	3	127	126	3	**1.34**
					129	128	3	**2.47**
					131	130	3	**1.06**
**357**	CTSD P07339: Cathepsin D		7	9	127	126	9	**1.42**
					129	128	8	**1.38**
					131	130	9	**0.89**
**358**	CTSF Q9UBX1: Cathepsin F		11	12	127	126	11	**0.93**
					129	128	11	**1.19**
					131	130	11	**0.84**
**359**	CTSH P09668: Pro-cathepsin H [Cleaved into: Cathepsin H mini chain; Cathepsin H		2	3	127	126	3	**1.42**
					129	128	3	**0.60**
					131	130	3	**1.05**
**360**	CTSK P43235: Cathepsin K		3	3	127	126	3	**1.23**
					129	128	3	**2.03**
					131	130	3	**0.96**
**361**	CTSL P07711: Cathepsin L1		1	1	127	126	1	**1.72**
					129	128	1	**1.48**
					131	130	1	**1.45**
**362**	CTSS P25774$_{1,2}$: Cathepsin S	2/2	1	1	127	126	1	**1.35**
					129	128	1	**1.67**
					131	130	1	**0.95**
**363**	CTTN Q14247$_{1,3}$: Src substrate cortactin	2/2	2	2	127	126	2	**1.44**
					129	128	2	**1.46**
					131	130	2	**1.18**
**364**	CUL3 Q13618$_{1-3}$: Cullin-3	3/3	2	2	127	126	2	**1.02**
					129	128	2	**2.12**
					131	130	2	**1.08**
**365**	CXCL12 P48061$_{1-6}$: Stromal cell-derived factor 1	6/6	5	13	127	126	8	**2.51**
					129	128	12	**1.22**
					131	130	11	**0.94**

#	protein	group	peptides	spectra	ch1	ch2	quant	ratio
**366**	CYFIP1 Q7L576$_1$: Cytoplasmic FMR1-interacting protein 1	1/3	3	3	127	126	1	**0.63**
					129	128	1	**1.20**
					131	130	1	**0.79**
**367**	CYFIP2 Q96F07$_{1,2}$: Cytoplasmic FMR1-interacting protein 2	2/2	4	4	127	126	2	**0.33**
					129	128	2	**2.42**
					131	130	2	**1.07**
**368**	CYR61 O00622: Protein CYR61	1/3	2	3	127	126	2	**1.98**
					129	128	2	**1.24**
					131	130	2	**1.26**
**369**	CYTL1 Q9NRR1: Cytokine-like protein 1		4	4	127	126	4	**1.22**
					129	128	3	**1.66**
					131	130	4	**0.99**
**370**	DAG1 Q14118: Dystroglycan		3	3	127	126	3	**1.29**
					129	128	3	**1.10**
					131	130	3	**1.20**
**371**	DARS P14868$_{1,2}$: Aspartate--tRNA ligase, cytoplasmic	2/2	3	3	127	126	3	**1.42**
					129	128	3	**0.74**
					131	130	3	**1.12**
**372**	DBNL Q9UJU6$_{1-6}$: Drebrin-like protein	6/6	2	3	127	126	3	**1.97**
					129	128	3	**0.87**
					131	130	3	**1.23**
**373**	DCD P81605$_1$: Dermcidin	1/2	6	10	127	126	10	**0.68**
					129	128	10	**2.36**
					131	130	10	**0.62**
**374**	DCN P07585$_1$: Decorin	1/5	14	38	127	126	30	**0.63**
					129	128	33	**3.71**
					131	130	31	**1.03**
**375**	DCTN1 Q14203$_{1,3,4,6}$: Dynactin subunit 1	4/6	8	9	127	126	7	**1.12**
					129	128	7	**1.49**
					131	130	7	**1.07**
**376**	DDB1 Q16531$_{1,2}$: DNA damage-binding protein 1	2/2	2	3	127	126	3	**2.69**
					129	128	3	**0.38**
					131	130	3	**1.69**
**377**	DDOST P39656$_{1,3}$: Dolichyl-diphosphooligosaccharide--protein glycosyltransferase 48 kDa subunit	2/2	3	3	127	126	3	**1.61**
					129	128	3	**0.71**
					131	130	3	**1.47**

#	protein	group	peptides	spectra	ch1	ch2	quant	ratio
**378**	DDX1 Q92499$_{1,2}$: ATP-dependent RNA helicase DDX1	2/3	6	13	127	126	3	**1.53**
					129	128	4	**1.42**
					131	130	5	**1.08**
**379**	DDX17 Q92841$_{1-4}$: Probable ATP-dependent RNA helicase DDX17	4/4	3	3	127	126	1	**1.57**
					129	128	1	**0.83**
					131	130	1	**1.35**
**380**	DDX19A Q9NUU7$_{1,2}$, DDX19B Q9UMR2$_{1-4}$: ATP-dependent RNA helicase DDX19A, ATP-dependent RNA helicase DDX19B	6/6	2	2	127	126	2	**1.17**
					129	128	2	**0.82**
					131	130	2	**1.10**
**381**	DDX39A O00148$_{1}$: ATP-dependent RNA helicase DDX39A	1/3	8	10	127	126	4	**0.77**
					129	128	3	**0.56**
					131	130	3	**0.80**
**382**	DDX39B Q13838$_{1,2}$: Spliceosome RNA helicase DDX39B	2/2	8	9	127	126	4	**1.07**
					129	128	4	**0.67**
					131	130	4	**1.11**
**383**	DDX5 P17844$_{1,2}$: Probable ATP-dependent RNA helicase DDX5	2/2	3	3	127	126	1	**1.19**
					129	128	1	**1.66**
					131	130	1	**1.22**
**384**	DDX6 P26196: Probable ATP-dependent RNA helicase DDX6		1	1	127	126	1	**0.86**
					129	128	1	**1.25**
					131	130	1	**1.06**
**385**	DEFA1 P59665: Neutrophil defensin 1		5	10	127	126	6	**1.98**
					129	128	5	**0.78**
					131	130	6	**1.21**
**386**	DEFA3 P59666: Neutrophil defensin 3		6	9	127	126	5	**0.68**
					129	128	4	**1.69**
					131	130	5	**1.09**
**387**	DEFA4 P12838: Neutrophil defensin 4		2	3	127	126	3	**1.82**
					129	128	3	**1.33**
					131	130	3	**1.03**
**388**	DEFA5 Q01523: Defensin-5		3	5	127	126	5	**2.64**
					129	128	5	**2.20**
					131	130	5	**1.18**
**389**	DEFA6 Q01524: Defensin-6		1	1	127	126	1	**2.57**
					129	128	1	**1.43**
					131	130	1	**1.15**

#	protein	group	peptides	spectra	ch1	ch2	quant	ratio
**390**	DEK P35659$_1$: Protein DEK		4	7	127	126	3	**1.90**
					129	128	2	**1.39**
					131	130	3	**1.46**
**391**	DES P17661: Desmin		16	49	127	126	12	**2.05**
					129	128	12	**0.77**
					131	130	12	**1.15**
**392**	DFNA5 O60443$_1$: Non-syndromic hearing impairment protein 5	1/3	8	8	127	126	8	**0.80**
					129	128	8	**1.12**
					131	130	6	**1.05**
**393**	DHX15 O43143: Pre-mRNA-splicing factor ATP-dependent RNA helicase DHX15		1	1	127	126	1	**1.73**
					129	128	1	**0.97**
					131	130	1	**1.50**
**394**	DHX9 Q08211$_1$: ATP-dependent RNA helicase A		5	5	127	126	5	**1.48**
					129	128	4	**0.91**
					131	130	5	**1.33**
**395**	DIAPH1 O60610$_{1-3}$: Protein diaphanous homolog 1	3/3	3	3	127	126	2	**0.92**
					129	128	2	**2.22**
					131	130	2	**1.00**
**396**	DKK3 Q9UBP4: Dickkopf-related protein 3		10	32	127	126	26	**1.42**
					129	128	23	**1.18**
					131	130	24	**1.10**
**397**	DLST P36957$_{1,2}$: Dihydrolipoyllysine-residue succinyltransferase component of 2-oxoglutarate ...	2/2	2	2	127	126	2	**1.77**
					129	128	2	**0.90**
					131	130	2	**1.89**
**398**	DMKN Q6E0U4$_5$: Dermokine	1/8	7	8	127	126	7	**1.68**
					129	128	7	**2.82**
					131	130	7	**1.19**
**399**	DMP1 Q13316$_{1,2}$: Dentin matrix acidic phosphoprotein 1	2/2	3	3	127	126	3	**0.88**
					129	128	3	**2.26**
					131	130	3	**1.22**
**400**	DNAH7 Q8WXX0$_1$: Dynein heavy chain 7, axonemal		7	18	127	126	15	**1.04**
					129	128	13	**0.75**
					131	130	15	**0.93**
**401**	DNAJA2 O60884: DnaJ homolog subfamily A member 2		4	5	127	126	5	**1.59**
					129	128	5	**0.67**
					131	130	4	**1.15**

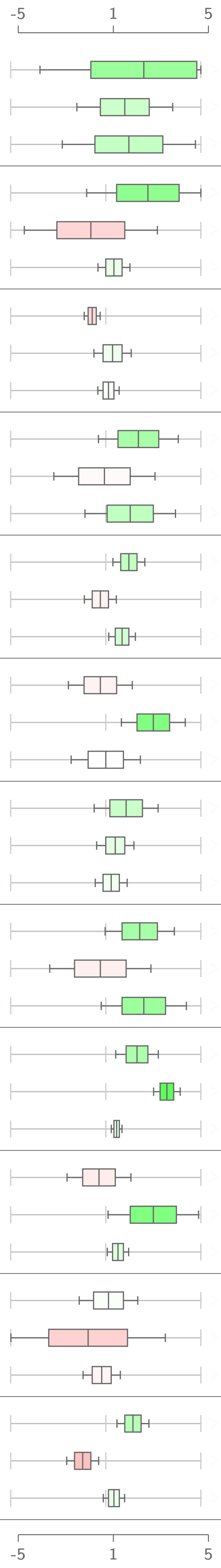

#	protein	group	peptides	spectra	ch1	ch2	quant	ratio
**402**	DNAJB1 P25685$_1$: DnaJ homolog subfamily B member 1		3	3	127	126	3	**2.12**
					129	128	3	**1.10**
					131	130	3	**1.38**
**403**	DNAJB11 Q9UBS4: DnaJ homolog subfamily B member 11		4	4	127	126	4	**1.30**
					129	128	4	**0.75**
					131	130	3	**1.23**
**404**	DNAJC3 Q13217: DnaJ homolog subfamily C member 3		17	24	127	126	21	**1.38**
					129	128	20	**1.26**
					131	130	22	**1.08**
**405**	DNM2 P50570$_{1-5}$: Dynamin-2	5/5	3	3	127	126	3	**1.18**
					129	128	3	**1.61**
					131	130	3	**1.04**
**406**	DNPH1 O43598$_{1,2}$: 2'-deoxynucleoside 5'-phosphate N-hydrolase 1	2/2	1	1	127	126	1	**1.41**
					129	128	1	**1.31**
					131	130	1	**1.36**
**407**	DOCK10 Q96BY6$_{1,3}$: Dedicator of cytokinesis protein 10	2/3	4	55	127	126	2	**0.63**
					129	128	2	**1.05**
					131	130	2	**1.00**
**408**	DPP4 P27487: Dipeptidyl peptidase 4		3	3	127	126	3	**0.98**
					129	128	2	**0.46**
					131	130	3	**1.62**
**409**	DPT Q07507: Dermatopontin		10	183	127	126	171	**1.29**
					129	128	135	**1.88**
					131	130	172	**1.18**
**410**	DPYSL2 Q16555$_{1,2}$: Dihydropyrimidinase-related protein 2	2/4	5	7	127	126	3	**1.77**
					129	128	3	**0.69**
					131	130	3	**1.09**
**411**	DPYSL3 Q14195$_2$: Dihydropyrimidinase-related protein 3	1/2	9	11	127	126	6	**1.63**
					129	128	7	**1.00**
					131	130	6	**1.16**
**412**	DSC1 Q08554$_1$: Desmocollin-1	1/2	4	8	127	126	4	**0.62**
					129	128	2	**3.38**
					131	130	4	**1.20**
**413**	DSC2 Q02487$_{1,2}$: Desmocollin-2	2/2	2	2	127	126	1	**0.76**
					129	128	1	**3.36**
					131	130	1	**1.00**

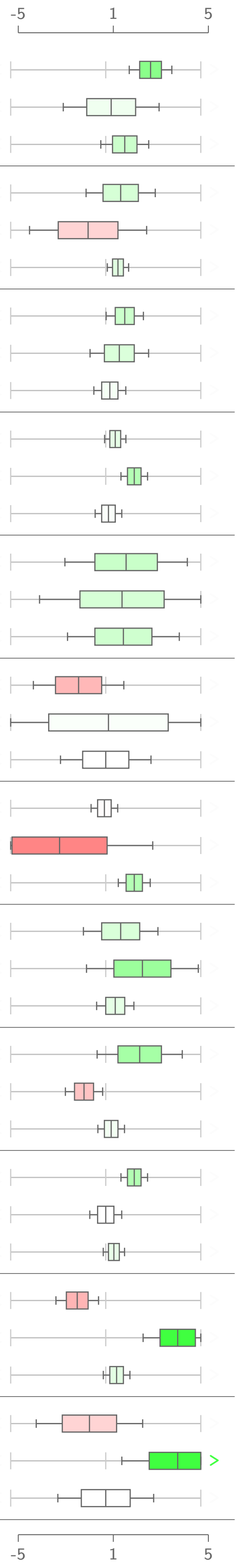

#	protein	group	peptides	spectra	ch1	ch2	quant	ratio
414	DSC3 Q14574$_{1,2}$: Desmocollin-3	2/2	6	6	127	126	5	**1.73**
					129	128	4	**2.40**
					131	130	5	**0.99**
415	DSG1 Q02413$_{1}$: Desmoglein-1	1/4	10	10	127	126	8	**0.70**
					129	128	9	**2.40**
					131	130	9	**0.96**
416	DSG2 Q14126: Desmoglein-2		1	1	127	126	1	**0.74**
					129	128	1	**1.94**
					131	130	1	**1.49**
417	DSP P15924$_{1}$: Desmoplakin	1/3	29	34	127	126	33	**0.70**
					129	128	31	**2.65**
					131	130	34	**1.07**
418	DST Q03001$_{3}$: Dystonin		6	21	127	126	16	**1.49**
					129	128	18	**0.97**
					131	130	18	**1.04**
420	DSTN P60981$_{1,2}$: Destrin	2/2	5	5	127	126	4	**1.10**
					129	128	4	**0.94**
					131	130	4	**1.09**
421	DYNC1H1 Q14204: Cytoplasmic dynein 1 heavy chain 1		20	23	127	126	20	**0.85**
					129	128	22	**1.98**
					131	130	21	**1.00**
422	DYNC1I2 Q13409$_{1-3,5-7}$: Cytoplasmic dynein 1 intermediate chain 2	6/6	1	1	127	126	1	**2.46**
					129	128	1	**0.74**
					131	130	1	**1.51**
423	DYNC1LI2 O43237$_{1,2}$: Cytoplasmic dynein 1 light intermediate chain 2	2/2	3	4	127	126	4	**1.22**
					129	128	3	**1.11**
					131	130	3	**1.02**
424	DYNC2H1 Q8NCM8$_{1,2}$: Cytoplasmic dynein 2 heavy chain 1	2/2	2	2	127	126	1	**1.79**
					129	128	1	**0.78**
					131	130	1	**1.15**
425	ECH1 Q13011: Delta(3,5)-Delta(2,4)-dienoyl-CoA isomerase, mitochondrial		2	2	127	126	2	**1.33**
					129	128	2	**1.67**
					131	130	2	**1.53**
426	ECM1 Q16610$_{1,4}$: Extracellular matrix protein 1	2/4	23	33	127	126	32	**0.99**
					129	128	28	**0.39**
					131	130	30	**1.07**

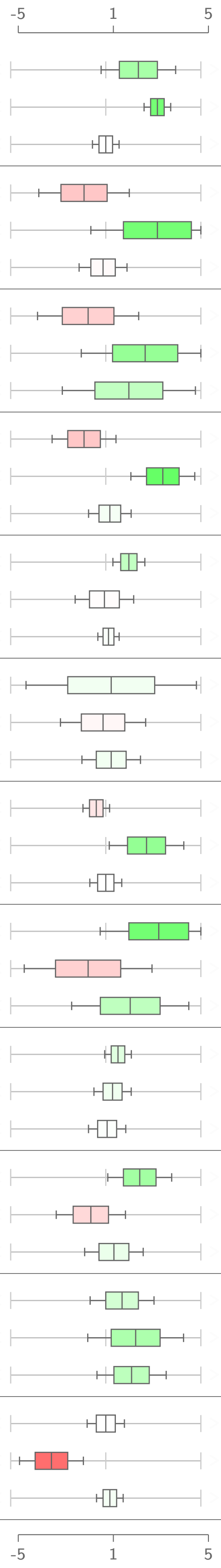

#	protein	group	peptides	spectra	ch1	ch2	quant	ratio
**427**	ECM2 O94769$_1$: Extracellular matrix protein 2	1/2	23	45	127	126	39	**0.98**
					129	128	42	**1.31**
					131	130	44	**0.93**
**428**	EEF1A1 P68104$_1$, EEF1A1P5 Q5VTE0: Elongation factor 1-alpha 1, Putative elongation factor 1-alpha-like 3	2/4	15	27	127	126	27	**1.55**
					129	128	25	**0.53**
					131	130	27	**1.25**
**429**	EEF1B2 P24534: Elongation factor 1-beta		5	7	127	126	4	**2.20**
					129	128	4	**0.61**
					131	130	5	**1.29**
**430**	EEF1D P29692$_{1-3}$: Elongation factor 1-delta	3/4	4	6	127	126	4	**1.45**
					129	128	4	**0.61**
					131	130	4	**1.29**
**431**	EEF1G P26641$_{1,2}$: Elongation factor 1-gamma	2/2	11	14	127	126	14	**1.47**
					129	128	13	**0.70**
					131	130	14	**1.25**
**432**	EEF2 P13639: Elongation factor 2		24	32	127	126	29	**0.88**
					129	128	26	**1.28**
					131	130	32	**1.08**
**433**	EFEMP1 Q12805$_{1-4}$: EGF-containing fibulin-like extracellular matrix protein 1	4/4	26	608	127	126	222	**0.79**
					129	128	150	**0.79**
					131	130	223	**0.85**
**434**	EFEMP1 Q12805$_5$: EGF-containing fibulin-like extracellular matrix protein 1		24	359	127	126	9	**0.66**
					129	128	8	**1.01**
					131	130	8	**0.88**
**435**	EFEMP2 O95967: EGF-containing fibulin-like extracellular matrix protein 2		18	135	127	126	103	**1.03**
					129	128	77	**0.96**
					131	130	105	**0.96**
**436**	EHD1 Q9H4M9: EH domain-containing protein 1	1/3	11	14	127	126	11	**1.33**
					129	128	12	**0.65**
					131	130	12	**1.14**
**437**	EHD2 Q9NZN4$_1$: EH domain-containing protein 2	1/2	10	12	127	126	8	**2.78**
					129	128	7	**0.53**
					131	130	8	**1.32**
**438**	EHD4 Q9H223: EH domain-containing protein 4		3	3	127	126	3	**1.08**
					129	128	2	**0.81**
					131	130	2	**0.87**

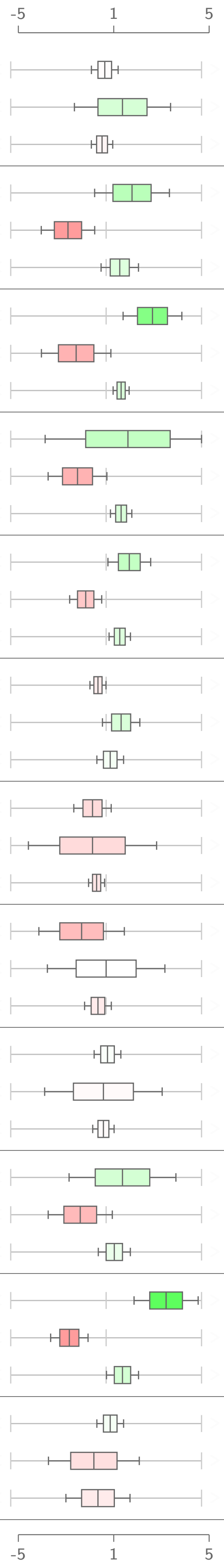

#	protein	group	peptides	spectra	ch1	ch2	quant	ratio
**439**	EIF1AX P47813, EIF1AY O14602: Eukaryotic translation initiation factor 1A, X-chromosomal, Eukaryotic translation initiation factor 1A, Y-chromosomal	2/2	2	2	127	126	2	**2.80**
					129	128	2	**0.80**
					131	130	2	**1.39**
**440**	EIF2S1 P05198: Eukaryotic translation initiation factor 2 subunit 1		2	2	127	126	2	**1.31**
					129	128	2	**0.69**
					131	130	2	**1.29**
**441**	EIF3A Q14152$_{1,2}$: Eukaryotic translation initiation factor 3 subunit A	2/2	3	6	127	126	6	**0.89**
					129	128	6	**1.87**
					131	130	5	**1.12**
**442**	EIF3B P55884$_{1,2}$: Eukaryotic translation initiation factor 3 subunit B	2/2	2	5	127	126	3	**2.19**
					129	128	4	**0.84**
					131	130	4	**1.06**
**443**	EIF3C Q99613$_{1,2}$: Eukaryotic translation initiation factor 3 subunit C	2/3	4	4	127	126	4	**1.06**
					129	128	3	**1.73**
					131	130	3	**1.11**
**444**	EIF3F O00303: Eukaryotic translation initiation factor 3 subunit F		2	2	127	126	2	**1.31**
					129	128	2	**1.49**
					131	130	2	**1.32**
**445**	EIF3H O15372: Eukaryotic translation initiation factor 3 subunit H		3	4	127	126	3	**1.08**
					129	128	4	**0.75**
					131	130	4	**1.06**
**446**	EIF3I Q13347: Eukaryotic translation initiation factor 3 subunit I		2	3	127	126	3	**2.05**
					129	128	3	**0.57**
					131	130	3	**1.25**
**447**	EIF3J O75822$_{1,2}$: Eukaryotic translation initiation factor 3 subunit J	2/3	3	3	127	126	3	**2.14**
					129	128	3	**1.11**
					131	130	3	**1.17**
**448**	EIF4A1 P60842$_{1}$: Eukaryotic initiation factor 4A-I	1/4	7	8	127	126	6	**1.23**
					129	128	6	**0.65**
					131	130	6	**1.14**
**449**	EIF4A3 P38919: Eukaryotic initiation factor 4A-III		3	3	127	126	2	**1.53**
					129	128	2	**0.71**
					131	130	2	**1.09**
**450**	EIF4G1 Q04637$_{1,3-6,9}$: Eukaryotic translation initiation factor 4 gamma 1	6/8	9	9	127	126	9	**1.16**
					129	128	8	**1.50**
					131	130	9	**1.14**

#	protein	group	peptides	spectra	ch1	ch2	quant	ratio
451	EIF4H Q15056$_{1,2}$: Eukaryotic translation initiation factor 4H	2/2	4	4	127	126	4	**1.79**
					129	128	4	**0.83**
					131	130	4	**1.33**
452	EIF5A P63241$_{1,2}$: Eukaryotic translation initiation factor 5A-1	2/2	3	4	127	126	4	**2.06**
					129	128	4	**1.13**
					131	130	4	**1.30**
453	ELANE P08246: Neutrophil elastase		2	3	127	126	3	**0.58**
					129	128	3	**2.29**
					131	130	3	**0.92**
454	EMILIN1 Q9Y6C2$_{1}$: EMILIN-1	1/2	14	17	127	126	16	**1.17**
					129	128	13	**2.29**
					131	130	16	**1.14**
455	EMILIN2 Q9BXX0: EMILIN-2		17	22	127	126	21	**1.05**
					129	128	21	**0.98**
					131	130	19	**1.18**
456	ENG P17813$_{1,2}$: Endoglin	2/2	1	1	127	126	1	**0.73**
					129	128	1	**2.24**
					131	130	1	**1.02**
457	ENO1 P06733$_{1}$: Alpha-enolase	1/2	16	21	127	126	19	**0.80**
					129	128	17	**2.13**
					131	130	21	**0.85**
458	EPPK1 P58107: Epiplakin		13	18	127	126	8	**1.31**
					129	128	7	**1.24**
					131	130	8	**1.12**
459	ERH P84090: Enhancer of rudimentary homolog		2	2	127	126	2	**3.03**
					129	128	2	**1.01**
					131	130	2	**1.28**
460	ERLIN2 O94905$_{1}$: Erlin-2		2	2	127	126	2	**2.70**
					129	128	2	**0.57**
					131	130	2	**1.82**
461	ERP44 Q9BS26: Endoplasmic reticulum resident protein 44		9	13	127	126	12	**1.39**
					129	128	12	**1.24**
					131	130	11	**0.98**
462	ETFB P38117$_{1,2}$: Electron transfer flavoprotein subunit beta	2/2	1	1	127	126	1	**2.00**
					129	128	1	**0.83**
					131	130	1	**1.55**

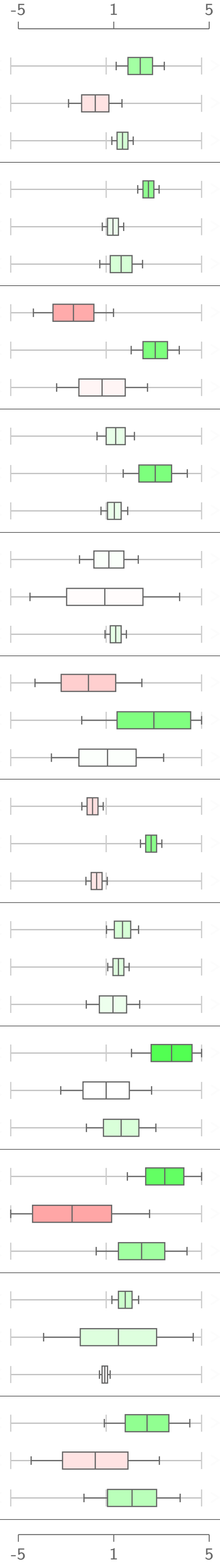

#	protein	group	peptides	spectra	ch1	ch2	quant	ratio
**463**	EWSR1 Q01844$_{1-3,5,6}$: RNA-binding protein EWS	5/5	2	3	127	126	3	**1.90**
					129	128	3	**0.65**
					131	130	3	**1.74**
**464**	EZR P15311: Ezrin		21	27	127	126	21	**0.68**
					129	128	20	**3.87**
					131	130	21	**1.47**
**465**	F10 P00742: Coagulation factor X		20	46	127	126	39	**0.95**
					129	128	37	**0.74**
					131	130	38	**0.92**
**466**	F11 P03951$_1$: Coagulation factor XI	1/2	19	28	127	126	28	**1.09**
					129	128	28	**0.77**
					131	130	25	**1.10**
**467**	F11R Q9Y624$_1$: Junctional adhesion molecule A		3	3	127	126	3	**1.42**
					129	128	3	**1.40**
					131	130	3	**1.09**
**468**	F12 P00748: Coagulation factor XII		22	50	127	126	47	**1.00**
					129	128	48	**1.29**
					131	130	48	**1.10**
**469**	F13A1 P00488: Coagulation factor XIII A chain		24	52	127	126	47	**0.97**
					129	128	47	**0.34**
					131	130	49	**1.08**
**470**	F13B P05160: Coagulation factor XIII B chain		15	20	127	126	18	**1.46**
					129	128	19	**0.72**
					131	130	19	**1.14**
**471**	F2 P00734: Prothrombin		39	751	127	126	691	**0.90**
					129	128	713	**0.74**
					131	130	711	**0.93**
**472**	F5 P12259: Coagulation factor V		24	27	127	126	23	**0.93**
					129	128	27	**0.50**
					131	130	27	**0.82**
**473**	F7 P08709$_{1,2}$: Coagulation factor VII	2/2	9	14	127	126	13	**0.58**
					129	128	13	**0.98**
					131	130	12	**0.88**
**474**	F8 P00451$_1$: Coagulation factor VIII		2	2	127	126	2	**0.97**
					129	128	2	**0.84**
					131	130	2	**1.07**

#	protein	group	peptides	spectra	ch1	ch2	quant	ratio
475	F9 P00740$_{1}$: Coagulation factor IX	1/2	19	41	127	126	31	**0.81**
					129	128	33	**0.76**
					131	130	33	**0.95**
476	FABP3 P05413: Fatty acid-binding protein, heart		4	6	127	126	6	**0.74**
					129	128	6	**3.26**
					131	130	6	**1.28**
477	FABP5 Q01469: Fatty acid-binding protein, epidermal	1/2	3	3	127	126	3	**1.21**
					129	128	3	**3.31**
					131	130	3	**0.88**
478	FABP9 Q0Z7S8: Fatty acid-binding protein 9		2	3	127	126	3	**0.84**
					129	128	3	**4.87**
					131	130	3	**0.85**
479	FAM114A1 Q8IWE2$_{1,2}$: Protein NOXP20	2/2	4	4	127	126	3	**1.18**
					129	128	4	**0.97**
					131	130	3	**1.14**
480	FAM20A Q96MK3: Pseudokinase FAM20A		3	3	127	126	2	**0.50**
					129	128	2	**2.01**
					131	130	2	**1.00**
481	FAM3C Q92520: Protein FAM3C		2	2	127	126	2	**1.30**
					129	128	2	**1.80**
					131	130	2	**1.10**
482	FAM49B Q9NUQ9$_{1}$: Protein FAM49B		5	5	127	126	5	**0.38**
					129	128	5	**3.03**
					131	130	3	**0.82**
483	FAP Q12884$_{1}$: Prolyl endopeptidase FAP		2	4	127	126	1	**1.17**
					129	128	1	**1.43**
					131	130	1	**1.07**
484	FARSA Q9Y285$_{1,2}$: Phenylalanine--tRNA ligase alpha subunit	2/2	3	3	127	126	3	**1.65**
					129	128	3	**0.72**
					131	130	3	**1.21**
485	FARSB Q9NSD9$_{1}$: Phenylalanine--tRNA ligase beta subunit	1/2	5	5	127	126	3	**1.70**
					129	128	4	**0.83**
					131	130	3	**1.01**
486	FAT1 Q14517: Protocadherin Fat 1		2	2	127	126	2	**0.34**
					129	128	2	**0.92**
					131	130	2	**1.16**

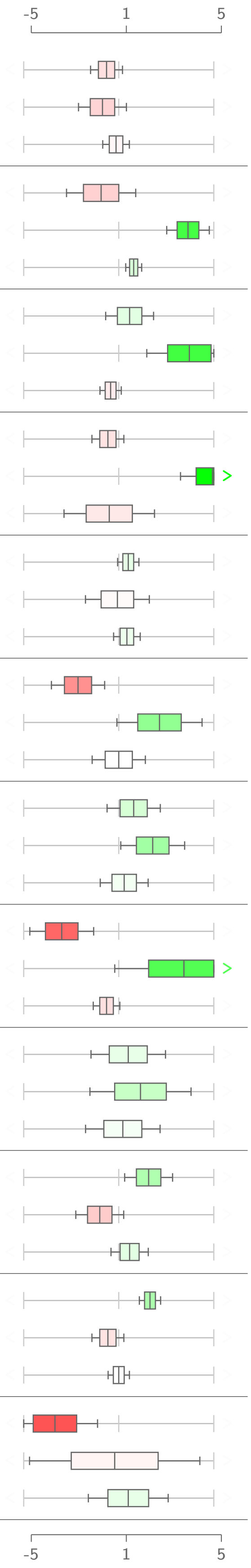

#	protein	group	peptides	spectra	ch1	ch2	quant	ratio
487	FBL P22087: rRNA 2’-O-methyltransferase fibrillarin		1	1	127	126	1	**1.89**
					129	128	1	**0.85**
					131	130	1	**2.19**
488	FBLN1 $P23142_1$: Fibulin-1	1/2	33	786	127	126	134	**0.79**
					129	128	117	**0.68**
					131	130	133	**0.93**
489	FBLN1 $P23142_3$: Fibulin-1		24	646	127	126	2	**1.19**
					129	128	2	**0.65**
					131	130	2	**0.83**
490	FBLN1 $P23142_4$: Fibulin-1		30	676	127	126	26	**1.19**
					129	128	22	**0.91**
					131	130	22	**1.12**
491	FBLN2 $P98095_2$: Fibulin-2	1/2	34	70	127	126	63	**0.82**
					129	128	63	**1.42**
					131	130	64	**1.01**
492	FBLN5 Q9UBX5: Fibulin-5		16	265	127	126	236	**1.15**
					129	128	197	**1.22**
					131	130	249	**1.14**
493	FBLN7 $Q53RD9_1$: Fibulin-7	1/4	6	8	127	126	8	**0.89**
					129	128	8	**1.17**
					131	130	8	**0.96**
494	FBN1 P35555: Fibrillin-1 [Cleaved into: Asprosin]		91	211	127	126	162	**1.77**
					129	128	147	**1.43**
					131	130	161	**1.39**
495	FBN2 $P35556_1$: Fibrillin-2 [Cleaved into: Fibrillin-2 C-terminal peptide]	1/2	20	47	127	126	20	**1.33**
					129	128	15	**0.86**
					131	130	18	**1.14**
496	FBXO2 Q9UK22: F-box only protein 2		4	6	127	126	4	**1.12**
					129	128	4	**0.33**
					131	130	4	**1.18**
497	FCGBP Q9Y6R7: IgGFc-binding protein		22	31	127	126	29	**1.10**
					129	128	31	**0.87**
					131	130	29	**0.89**
498	FCN1 O00602: Ficolin-1		3	5	127	126	3	**1.33**
					129	128	3	**0.59**
					131	130	3	**1.33**

#	protein	group	peptides	spectra	ch1	ch2	quant	ratio
**499**	FCN2 Q15485$_1$: Ficolin-2	1/2	5	7	127	126	5	**1.01**
					129	128	4	**0.56**
					131	130	5	**1.07**
**500**	FCN3 O75636$_1$: Ficolin-3		9	15	127	126	2	**1.09**
					129	128	2	**0.24**
					131	130	2	**1.11**
**502**	FERMT3 Q86UX7$_{1,2}$: Fermitin family homolog 3	2/2	7	7	127	126	5	**0.64**
					129	128	7	**1.07**
					131	130	6	**0.86**
**503**	FETUB Q9UGM5$_1$: Fetuin-B	1/2	16	36	127	126	31	**0.34**
					129	128	32	**2.67**
					131	130	35	**0.88**
**504**	FGA P02671$_1$: Fibrinogen alpha chain [Cleaved into: Fibrinopeptide A; Fibrinogen alpha chain]	1/2	68	4143	127	126	3959	**1.44**
					129	128	3863	**0.33**
					131	130	3964	**1.03**
**505**	FGB P02675: Fibrinogen beta chain [Cleaved into: Fibrinopeptide B; Fibrinogen beta chain]		46	3555	127	126	3361	**1.20**
					129	128	3195	**0.25**
					131	130	3340	**0.92**
**506**	FGG P02679$_{1,2}$: Fibrinogen gamma chain	2/2	34	2426	127	126	2232	**1.19**
					129	128	2135	**0.26**
					131	130	2246	**0.92**
**507**	FIBIN Q8TAL6: Fin bud initiation factor homolog		2	2	127	126	2	**1.17**
					129	128	2	**0.89**
					131	130	2	**1.09**
**508**	FIS1 Q9Y3D6: Mitochondrial fission 1 protein		3	3	127	126	3	**1.83**
					129	128	3	**0.43**
					131	130	3	**1.03**
**509**	FKBP1A P62942: Peptidyl-prolyl cis-trans isomerase FKBP1A		1	1	127	126	1	**0.81**
					129	128	1	**2.74**
					131	130	1	**1.08**
**510**	FLG P20930: Filaggrin		4	5	127	126	3	**0.54**
					129	128	5	**3.71**
					131	130	4	**1.01**
**511**	FLG2 Q5D862: Filaggrin-2		2	3	127	126	3	**0.63**
					129	128	3	**2.09**
					131	130	3	**0.55**

#	protein	group	peptides	spectra	ch1	ch2	quant	ratio
**512**	FLII Q13045$_{1-3}$: Protein flightless-1 homolog	3/3	4	4	127	126	4	**0.92**
					129	128	4	**1.52**
					131	130	4	**1.17**
**513**	FLNA P21333$_{1,2}$: Filamin-A	2/2	71	92	127	126	66	**0.97**
					129	128	71	**1.50**
					131	130	70	**1.11**
**514**	FLNB O75369$_{1-3,6,8,9}$: Filamin-B	6/9	15	15	127	126	9	**0.86**
					129	128	8	**1.73**
					131	130	9	**1.03**
**515**	FLNC Q14315$_{1,2}$: Filamin-C	2/2	44	52	127	126	39	**0.92**
					129	128	40	**1.88**
					131	130	42	**1.10**
**516**	FLT4 P35916$_{1-3}$: Vascular endothelial growth factor receptor 3	3/3	1	1	127	126	1	**1.02**
					129	128	1	**1.30**
					131	130	1	**0.72**
**517**	FMOD Q06828: Fibromodulin		11	24	127	126	15	**1.22**
					129	128	17	**1.13**
					131	130	19	**1.17**
**518**	FN1 P02751$_{1,3,17}$: Fibronectin	3/17	89	1011	127	126	939	**1.02**
					129	128	861	**0.90**
					131	130	935	**1.05**
**519**	FN3KRP Q9HA64: Ketosamine-3-kinase		1	1	127	126	1	**1.51**
					129	128	1	**1.06**
					131	130	1	**0.97**
**520**	FNDC1 Q4ZHG4$_{1,2}$: Fibronectin type III domain-containing protein 1	2/2	7	7	127	126	7	**1.13**
					129	128	7	**1.99**
					131	130	7	**1.02**
**521**	FOXR1 Q6PIV2$_{1}$: Forkhead box protein R1		2	9	127	126	2	**0.56**
					129	128	2	**1.30**
					131	130	2	**0.97**
**522**	FRA10AC1 Q70Z53$_{1-5}$: Protein FRA10AC1	5/5	3	26	127	126	22	**0.90**
					129	128	17	**1.20**
					131	130	21	**0.99**
**523**	FRAS1 Q86XX4$_{2}$: Extracellular matrix protein FRAS1	1/5	13	21	127	126	15	**0.74**
					129	128	14	**1.28**
					131	130	14	**0.99**

#	protein	group	peptides	spectra	ch1	ch2	quant	ratio
**525**	FRZB Q92765: Secreted frizzled-related protein 3		3	4	127	126	4	**0.94**
					129	128	4	**1.39**
					131	130	4	**1.22**
**526**	FSCN1 Q16658: Fascin		2	2	127	126	2	**0.77**
					129	128	2	**1.33**
					131	130	2	**0.92**
**527**	FST P19883$_{1,2}$: Follistatin	2/2	11	13	127	126	13	**1.68**
					129	128	11	**1.56**
					131	130	11	**1.24**
**528**	FSTL1 Q12841$_{1}$: Follistatin-related protein 1	1/2	17	45	127	126	42	**1.68**
					129	128	34	**1.63**
					131	130	38	**1.31**
**529**	FSTL3 O95633$_{1,2}$: Follistatin-related protein 3	2/2	3	4	127	126	3	**1.83**
					129	128	4	**1.10**
					131	130	4	**1.16**
**530**	FTH1 P02794: Ferritin heavy chain		2	2	127	126	2	**2.44**
					129	128	2	**0.95**
					131	130	2	**1.17**
**531**	FUBP1 Q96AE4$_{1,2}$: Far upstream element-binding protein 1	2/2	4	4	127	126	3	**1.26**
					129	128	3	**0.71**
					131	130	3	**1.11**
**532**	FUS P35637$_{1,2}$: RNA-binding protein FUS	2/2	6	10	127	126	5	**2.03**
					129	128	5	**0.65**
					131	130	4	**1.63**
**533**	FXR1 P51114$_{1-3}$: Fragile X mental retardation syndrome-related protein 1	3/3	3	3	127	126	3	**2.11**
					129	128	3	**0.58**
					131	130	3	**1.15**
**534**	G3BP1 Q13283$_{1}$: Ras GTPase-activating protein-binding protein 1		1	1	127	126	1	**3.00**
					129	128	1	**0.39**
					131	130	1	**1.38**
**535**	G6PD P11413$_{1-3}$: Glucose-6-phosphate 1-dehydrogenase	3/3	6	6	127	126	6	**1.16**
					129	128	6	**0.97**
					131	130	6	**1.10**
**536**	GAL3ST4 Q96RP7$_{1,2}$: Galactose-3-O-sulfotransferase 4	2/2	1	1	127	126	1	**0.91**
					129	128	1	**1.47**
					131	130	1	**0.78**

#	protein	group	peptides	spectra	ch1	ch2	quant	ratio
**537**	GALK1 P51570$_{1,2}$: Galactokinase	2/2	3	3	127	126	3	**1.55**
					129	128	3	**0.71**
					131	130	3	**1.07**
**538**	GALNT2 Q10471$_{1}$: Polypeptide N-acetylgalactosaminyltransferase 2	1/2	2	2	127	126	2	**0.97**
					129	128	2	**1.71**
					131	130	2	**1.11**
**539**	GANAB Q14697$_{1}$: Neutral alpha-glucosidase AB		23	31	127	126	2	**1.96**
					129	128	2	**0.59**
					131	130	2	**1.20**
**540**	GANAB Q14697$_{2}$: Neutral alpha-glucosidase AB		22	30	127	126	1	**1.37**
					129	128	1	**0.72**
					131	130	1	**1.57**
**542**	GAPDH P04406$_{1}$: Glyceraldehyde-3-phosphate dehydrogenase	1/2	17	63	127	126	63	**2.24**
					129	128	53	**0.59**
					131	130	62	**0.95**
**543**	GAS1 P54826: Growth arrest-specific protein 1		10	21	127	126	18	**1.37**
					129	128	18	**2.16**
					131	130	19	**0.99**
**544**	GAS6 Q14393$_{1-3}$: Growth arrest-specific protein 6	3/5	15	23	127	126	20	**0.87**
					129	128	22	**1.27**
					131	130	21	**0.89**
**545**	GBA P04062$_{1,2,4,5}$: Glucosylceramidase	4/5	2	2	127	126	2	**0.52**
					129	128	2	**1.07**
					131	130	2	**1.05**
**546**	GC P02774$_{1,3}$: Vitamin D-binding protein	2/3	32	301	127	126	261	**1.62**
					129	128	234	**0.71**
					131	130	275	**1.18**
**547**	GCG P01275: Glucagon [Cleaved into: Glicentin; Glicentin-related polypeptide		6	9	127	126	9	**1.98**
					129	128	8	**0.85**
					131	130	8	**0.80**
**548**	GDF15 Q99988: Growth/differentiation factor 15		6	7	127	126	6	**1.53**
					129	128	7	**1.54**
					131	130	7	**0.97**
**549**	GDF7 Q7Z4P5: Growth/differentiation factor 7		2	2	127	126	2	**0.78**
					129	128	2	**1.40**
					131	130	2	**1.05**

#	protein	group	peptides	spectra	ch1	ch2	quant	ratio
**550**	GFAP P14136$_{1-3}$: Glial fibrillary acidic protein	3/3	3	12	127	126	1	**0.55**
					129	128		
					131	130	1	**0.76**
**551**	GFPT1 Q06210$_{1,2}$: Glutamine--fructose-6-phosphate aminotransferase [isomerizing] 1	2/2	2	2	127	126	2	**0.92**
					129	128	2	**0.92**
					131	130	2	**1.30**
**552**	GFPT2 O94808: Glutamine--fructose-6-phosphate aminotransferase [isomerizing] 2		3	3	127	126	3	**0.73**
					129	128	3	**0.85**
					131	130	3	**1.00**
**553**	GGCT O75223$_{1,3}$: Gamma-glutamylcyclotransferase	2/2	1	1	127	126	1	**0.57**
					129	128	1	**4.29**
					131	130	1	**0.57**
**554**	GHRL Q9UBU3$_{1-5}$: Appetite-regulating hormone	5/5	2	2	127	126	2	**1.30**
					129	128	2	**1.24**
					131	130	2	**1.67**
**555**	GIP P09681: Gastric inhibitory polypeptide		2	3	127	126	3	**1.50**
					129	128	3	**1.21**
					131	130	3	**1.03**
**556**	GIPC1 O14908$_{1,2}$: PDZ domain-containing protein GIPC1	2/2	1	2	127	126	2	**1.55**
					129	128	2	**2.14**
					131	130	2	**1.19**
**557**	GKN1 Q9NS71: Gastrokine-1		6	13	127	126	13	**1.01**
					129	128	13	**2.80**
					131	130	9	**1.27**
**558**	GKN2 Q86XP6: Gastrokine-2		1	1	127	126	1	**1.22**
					129	128	1	**0.82**
					131	130	1	**0.92**
**559**	GLA P06280: Alpha-galactosidase A		4	8	127	126	7	**0.96**
					129	128	6	**2.14**
					131	130	7	**1.09**
**560**	GLB1 P16278$_{1,3}$: Beta-galactosidase	2/3	3	3	127	126	3	**1.12**
					129	128	3	**1.21**
					131	130	3	**0.93**
**561**	GLG1 Q92896$_{1-3}$: Golgi apparatus protein 1	3/3	5	6	127	126	6	**1.18**
					129	128	6	**0.92**
					131	130	5	**1.16**

#	protein	group	peptides	spectra	ch1	ch2	quant	ratio
**562**	GM2A P17900: Ganglioside GM2 activator		6	9	127	126	7	**0.85**
					129	128	8	**1.93**
					131	130	9	**1.02**
**563**	GMPPA $Q96IJ6_{1,2}$: Mannose-1-phosphate guanyltransferase alpha	2/2	3	3	127	126	3	**1.46**
					129	128	3	**0.71**
					131	130	3	**1.10**
**564**	GMPPB $Q9Y5P6_{1,2}$: Mannose-1-phosphate guanyltransferase beta	2/2	2	2	127	126	2	**1.46**
					129	128	2	**0.70**
					131	130	2	**1.10**
**565**	GNAI2 $P04899_{1-6}$: Guanine nucleotide-binding protein G(i) subunit alpha-2	6/6	1	1	127	126	1	**0.72**
					129	128	1	**2.72**
					131	130	1	**0.93**
**566**	GNB1 $P62873_{1,2}$: Guanine nucleotide-binding protein G(I)/G(S)/G(T) subunit beta-1	2/4	4	6	127	126	2	**1.10**
					129	128	2	**1.12**
					131	130	2	**1.29**
**567**	GNB2 $P62879_{1}$: Guanine nucleotide-binding protein G(I)/G(S)/G(T) subunit beta-2	1/3	5	7	127	126	3	**1.15**
					129	128	3	**1.56**
					131	130	3	**1.14**
**568**	GNPTG Q9UJJ9: N-acetylglucosamine-1-phosphotransferase subunit gamma		4	4	127	126	4	**0.94**
					129	128	4	**1.23**
					131	130	4	**1.13**
**569**	GOLGA6L22 H0YM25: Golgin subfamily A member 6-like protein 22		7	46	127	126	28	**1.17**
					129	128	16	**0.69**
					131	130	26	**1.09**
**570**	GOLGB1 $Q14789_{1-4}$: Golgin subfamily B member 1	4/4	2	2	127	126	1	**0.65**
					129	128	1	**0.53**
					131	130	1	**0.93**
**571**	GOLIM4 O00461: Golgi integral membrane protein 4		10	12	127	126	10	**1.45**
					129	128	10	**1.91**
					131	130	11	**1.18**
**572**	GOLM1 $Q8NBJ4_{1,2}$: Golgi membrane protein 1	2/2	14	15	127	126	13	**1.42**
					129	128	12	**1.74**
					131	130	12	**1.49**
**573**	GPLD1 $P80108_{1}$: Phosphatidylinositol-glycan-specific phospholipase D		15	18	127	126	17	**1.37**
					129	128	18	**0.60**
					131	130	16	**0.96**

#	protein	group	peptides	spectra	ch1	ch2	quant	ratio
**574**	GPS1 Q13098$_{4-7}$: COP9 signalosome complex subunit 1	4/4	2	2	127	126	2	**1.34**
					129	128	2	**1.05**
					131	130	2	**1.26**
**575**	GPX3 P22352: Glutathione peroxidase 3		10	21	127	126	21	**1.33**
					129	128	18	**1.15**
					131	130	18	**1.12**
**576**	GREM1 O60565$_{1,2}$: Gremlin-1	2/2	1	1	127	126	1	**0.98**
					129	128	1	**2.34**
					131	130	1	**3.38**
**577**	GRID1 Q9ULK0$_{1}$: Glutamate receptor ionotropic, delta-1		3	20	127	126	1	**1.76**
					129	128	1	**1.53**
					131	130	1	**1.05**
**578**	GRN P28799$_{1}$: Granulins	1/3	7	9	127	126	9	**1.23**
					129	128	9	**1.35**
					131	130	8	**1.13**
**579**	GSDMD P57764: Gasdermin-D		2	3	127	126	3	**1.17**
					129	128	3	**1.00**
					131	130	3	**1.16**
**580**	GSN P06396$_{1}$: Gelsolin	1/3	45	1065	127	126	55	**0.35**
					129	128	59	**3.32**
					131	130	54	**0.80**
**581**	GSN P06396$_{2}$: Gelsolin		43	1008	127	126	2	**0.50**
					129	128	2	**1.25**
					131	130	2	**0.95**
**584**	GSTM3 P21266: Glutathione S-transferase Mu 3		7	7	127	126	6	**1.62**
					129	128	4	**0.80**
					131	130	6	**1.12**
**586**	GSTO1 P78417$_{1,2}$: Glutathione S-transferase omega-1	2/3	3	3	127	126	3	**0.84**
					129	128	3	**1.55**
					131	130	3	**1.08**
**587**	GSTP1 P09211: Glutathione S-transferase P		7	8	127	126	8	**0.68**
					129	128	7	**1.43**
					131	130	8	**1.13**
**588**	GUCA2A Q02747: Guanylin		2	2	127	126	2	**1.99**
					129	128	2	**1.67**
					131	130	2	**1.36**

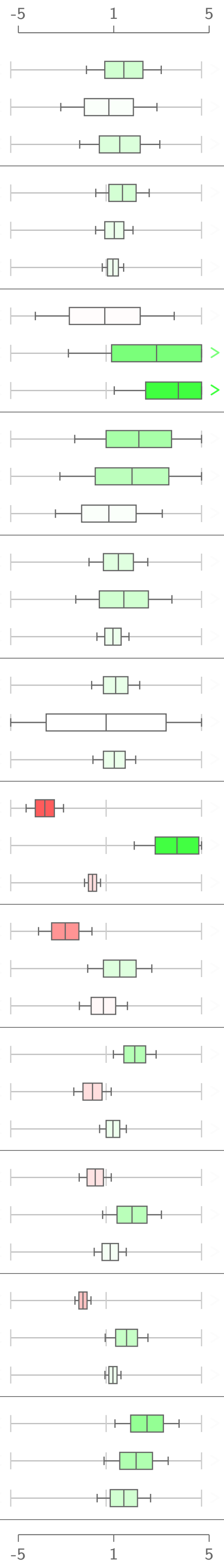

#	protein	group	peptides	spectra	ch1	ch2	quant	ratio
589	H1F0 P07305$_{1,2}$: Histone H1.0	2/2	3	3	127	126	3	**2.48**
					129	128	3	**0.66**
					131	130	3	**1.31**
590	H1FX Q92522: Histone H1x		4	5	127	126	5	**2.13**
					129	128	4	**1.08**
					131	130	5	**1.53**
591	H2AFJ Q9BTM1$_{1}$: Histone H2A.J		5	15	127	126	1	**2.16**
					129	128	1	**1.63**
					131	130	1	**1.31**
592	H2AFV Q71UI9$_{1,2}$, H2AFZ P0C0S5: Histone H2A.V, Histone H2A.Z	3/5	3	5	127	126	1	**2.86**
					129	128	1	**0.62**
					131	130	1	**1.85**
593	H2AFX P16104: Histone H2AX	1/6	9	16	127	126	6	**2.67**
					129	128	5	**0.88**
					131	130	6	**1.59**
594	H3F3A P84243: Histone H3.3		8	23	127	126	1	**3.50**
					129	128	1	**0.48**
					131	130	1	**1.84**
595	HABP2 Q14520$_{1,2}$: Hyaluronan-binding protein 2	2/2	16	30	127	126	30	**0.73**
					129	128	28	**0.95**
					131	130	29	**1.06**
596	HACD2 Q6Y1H2: Very-long-chain		1	1	127	126	1	**1.25**
					129	128	1	**1.15**
					131	130	1	**1.91**
597	HADHA P40939$_{1}$: Trifunctional enzyme subunit alpha, mitochondrial		5	5	127	126	5	**1.34**
					129	128	5	**0.77**
					131	130	5	**1.57**
598	HADHB P55084$_{1}$: Trifunctional enzyme subunit beta, mitochondrial		2	2	127	126	2	**2.01**
					129	128	2	**1.20**
					131	130	2	**2.24**
599	HAMP P81172: Hepcidin		2	11	127	126	7	**0.79**
					129	128	8	**1.32**
					131	130	8	**1.01**
600	HBB P68871: Hemoglobin subunit beta	1/2	5	6	127	126	5	**1.17**
					129	128	5	**0.31**
					131	130	5	**0.71**

#	protein	group	peptides	spectra	ch1	ch2	quant	ratio
**601**	HCLS1 P14317$_1$: Hematopoietic lineage cell-specific protein		2	2	127	126	2	**1.04**
					129	128	2	**1.62**
					131	130	2	**1.00**
**602**	HDGF P51858$_{1-3}$: Hepatoma-derived growth factor	3/3	8	10	127	126	10	**2.08**
					129	128	8	**0.60**
					131	130	9	**1.37**
**603**	HDLBP Q00341$_{1,2}$: Vigilin	2/2	10	11	127	126	11	**1.59**
					129	128	11	**0.99**
					131	130	11	**1.18**
**604**	HEBP1 Q9NRV9: Heme-binding protein 1		2	3	127	126	3	**0.81**
					129	128	3	**1.19**
					131	130	3	**1.07**
**605**	HERC2 O95714: E3 ubiquitin-protein ligase HERC2		4	25	127	126	19	**0.88**
					129	128	11	**0.70**
					131	130	17	**0.89**
**606**	HEXA P06865$_1$: Beta-hexosaminidase subunit alpha	1/2	4	5	127	126	4	**1.34**
					129	128	5	**0.95**
					131	130	5	**0.93**
**607**	HEXB P07686: Beta-hexosaminidase subunit beta		4	5	127	126	5	**1.56**
					129	128	5	**1.35**
					131	130	5	**0.98**
**608**	HGS O14964$_{1,2}$: Hepatocyte growth factor-regulated tyrosine kinase substrate	2/2	3	3	127	126	3	**1.20**
					129	128	3	**1.39**
					131	130	3	**0.97**
**609**	HINT1 P49773: Histidine triad nucleotide-binding protein 1		3	3	127	126	3	**1.05**
					129	128	3	**1.18**
					131	130	3	**1.11**
**610**	HIST1H1B P16401: Histone H1.5		12	24	127	126	7	**1.98**
					129	128	8	**0.74**
					131	130	5	**1.82**
**611**	HIST1H1C P16403: Histone H1.2		15	30	127	126	9	**1.84**
					129	128	9	**0.84**
					131	130	9	**1.64**
**612**	HIST1H1D P16402: Histone H1.3		11	21	127	126	3	**1.50**
					129	128	3	**0.84**
					131	130	3	**1.18**

#	protein	group	peptides	spectra	ch1	ch2	quant	ratio
613	HIST1H1E P10412: Histone H1.4	1/3	17	31	127	126	8	**2.53**
					129	128	8	**1.07**
					131	130	8	**1.98**
614	HIST1H2AC Q93077, HIST1H2AG P0C0S8, HIST1H2AH Q96KK5, . . . : Histone H2A type 1-C, Histone H2A type 1, . . .	5/5	5	16	127	126	2	**2.40**
					129	128	2	**0.46**
					131	130	2	**1.81**
618	HIST1H3A P68431, HIST2H3A; Q71DI3, HIST3H3 Q16695: Histone H3.1, Histone H3.2, . . .	3/4	8	23	127	126	1	**2.10**
					129	128	1	**0.79**
					131	130	1	**1.72**
619	HIST1H4A P62805: Histone H4		7	15	127	126	11	**2.25**
					129	128	14	**0.92**
					131	130	13	**1.71**
620	HIST2H2AB Q8IUE6: Histone H2A type 2-B		3	9	127	126	1	**3.29**
					129	128	1	**0.89**
					131	130	1	**1.32**
621	HIST2H2AC Q16777: Histone H2A type 2-C		5	16	127	126	2	**2.74**
					129	128	2	**0.57**
					131	130	2	**1.52**
623	HLA-A P05534: HLA class I histocompatibility antigen, A-24 alpha chain		8	9	127	126	1	**0.71**
					129	128	1	**0.82**
					131	130	1	**0.99**
625	HLA-A P10316: HLA class I histocompatibility antigen, A-69 alpha chain	1/2	5	8	127	126	1	**0.61**
					129	128	1	**1.71**
					131	130	1	**1.15**
631	HLA-A P30450: HLA class I histocompatibility antigen, A-26 alpha chain	1/7	10	16	127	126	1	**1.41**
					129	128	1	**0.87**
					131	130	1	**1.14**
634	HLA-B P10319: HLA class I histocompatibility antigen, B-58 alpha chain	1/2	9	14	127	126	1	**0.81**
					129	128	1	**3.05**
					131	130	1	**0.99**
635	HLA-B P18463: HLA class I histocompatibility antigen, B-37 alpha chain	1/7	7	11	127	126	1	**0.72**
					129	128		
					131	130		
642	HLA-C P30501: HLA class I histocompatibility antigen, Cw-2 alpha chain	1/10	9	14	127	126	2	**0.78**
					129	128	2	**1.40**
					131	130	2	**0.84**

#	protein	group	peptides	spectra	ch1	ch2	quant	ratio
**643**	HLA-C P30508: HLA class I histocompatibility antigen, Cw-12 alpha chain	1/6	10	14	127	126	1	**0.73**
					129	128	1	**2.84**
					131	130	1	**1.06**
**644**	HLA-DRA P01903: HLA class II histocompatibility antigen, DR alpha chain	1/2	2	2	127	126	2	**0.84**
					129	128	2	**0.78**
					131	130	2	**0.91**
**645**	HLA-DRB1 P04229, HLA-DRB1 Q29974, HLA-DRB1 Q30134, . . . : HLA class II histocompatibility antigen, DRB1-1 beta chain, HLA class II histocompatibility antigen, DRB1-16 beta chain, . . .	4/16	3	3	127	126	3	**0.87**
					129	128	3	**1.06**
					131	130	3	**0.89**
**646**	HMCN1 Q96RW7$_{1,2}$: Hemicentin-1	2/2	36	46	127	126	41	**0.43**
					129	128	37	**2.51**
					131	130	39	**0.75**
**647**	HMCN2 Q8NDA2$_1$: Hemicentin-2	1/3	26	27	127	126	21	**0.56**
					129	128	24	**1.79**
					131	130	23	**0.97**
**648**	HMGB1 P09429: High mobility group protein B1		3	3	127	126	1	**3.11**
					129	128	1	**0.76**
					131	130	1	**1.65**
**649**	HMGN2 P05204: Non-histone chromosomal protein HMG-17		4	4	127	126	3	**2.07**
					129	128	3	**1.24**
					131	130	3	**1.96**
**650**	HMGN4 O00479: High mobility group nucleosome-binding domain-containing protein 4		3	4	127	126	3	**2.14**
					129	128	3	**1.32**
					131	130	3	**2.95**
**651**	HNMT P50135$_1$: Histamine N-methyltransferase		4	5	127	126	4	**0.60**
					129	128	4	**1.81**
					131	130	3	**0.91**
**652**	HNRNPA0 Q13151: Heterogeneous nuclear ribonucleoprotein A0		2	2	127	126	1	**1.68**
					129	128	1	**3.53**
					131	130	1	**1.02**
**653**	HNRNPA1 P09651$_{1-3}$: Heterogeneous nuclear ribonucleoprotein A1	3/4	7	10	127	126	3	**2.33**
					129	128	3	**0.41**
					131	130	3	**1.64**
**654**	HNRNPA2B1 P22626$_1$: Heterogeneous nuclear ribonucleoproteins A2/B1	1/2	10	12	127	126	8	**2.52**
					129	128	9	**0.49**
					131	130	9	**1.83**

#	protein	group	peptides	spectra	ch1	ch2	quant	ratio
**655**	HNRNPA3 P51991$_1$: Heterogeneous nuclear ribonucleoprotein A3	1/2	8	13	127	126	7	**2.67**
					129	128	8	**0.75**
					131	130	8	**1.59**
**656**	HNRNPAB Q99729$_{1-4}$: Heterogeneous nuclear ribonucleoprotein A/B	4/4	5	5	127	126	2	**2.83**
					129	128	2	**0.48**
					131	130	2	**1.69**
**657**	HNRNPC P07910$_2$: Heterogeneous nuclear ribonucleoproteins C1/C2	1/4	10	12	127	126	8	**1.83**
					129	128	7	**1.09**
					131	130	8	**1.45**
**658**	HNRNPCL1 O60812, HNRNPCL2 B2RXH8: Heterogeneous nuclear ribonucleoprotein C-like 1, Heterogeneous nuclear ribonucleoprotein C-like 2	2/4	5	8	127	126	3	**1.02**
					129	128	4	**1.36**
					131	130	4	**0.98**
**659**	HNRNPD Q14103$_{1,3}$: Heterogeneous nuclear ribonucleoprotein D0	2/2	6	10	127	126	6	**1.15**
					129	128	6	**1.49**
					131	130	6	**1.07**
**660**	HNRNPD Q14103$_{2,4}$: Heterogeneous nuclear ribonucleoprotein D0	2/2	5	5	127	126	1	**1.28**
					129	128	1	**0.86**
					131	130	1	**1.77**
**661**	HNRNPDL O14979$_{1-3}$: Heterogeneous nuclear ribonucleoprotein D-like	3/3	4	4	127	126	3	**3.05**
					129	128	3	**0.52**
					131	130	3	**1.57**
**662**	HNRNPF P52597: Heterogeneous nuclear ribonucleoprotein F		7	10	127	126	5	**2.71**
					129	128	4	**0.42**
					131	130	5	**1.51**
**663**	HNRNPH1 P31943: Heterogeneous nuclear ribonucleoprotein H	1/2	7	10	127	126	4	**2.40**
					129	128	3	**0.34**
					131	130	4	**1.56**
**664**	HNRNPH3 P31942$_{1,2}$: Heterogeneous nuclear ribonucleoprotein H3	2/4	5	5	127	126	4	**2.16**
					129	128	4	**0.62**
					131	130	4	**1.44**
**665**	HNRNPK P61978$_{2,3}$: Heterogeneous nuclear ribonucleoprotein K	2/3	15	33	127	126	29	**2.99**
					129	128	31	**0.59**
					131	130	30	**1.47**
**666**	HNRNPL P14866$_1$: Heterogeneous nuclear ribonucleoprotein L	1/2	8	15	127	126	15	**2.03**
					129	128	15	**0.88**
					131	130	14	**1.37**

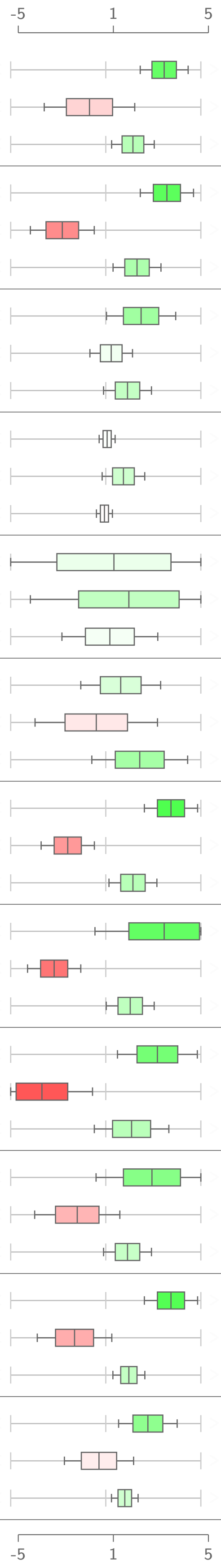

#	protein	group	peptides	spectra	ch1	ch2	quant	ratio
**667**	HNRNPM P52272$_{1,2}$: Heterogeneous nuclear ribonucleoprotein M	2/2	3	3	127	126	3	**1.44**
					129	128	3	**0.62**
					131	130	3	**1.35**
**668**	HNRNPR O43390$_{1,2,4}$: Heterogeneous nuclear ribonucleoprotein R	3/4	7	8	127	126	1	**2.56**
					129	128	1	**0.47**
					131	130	1	**2.07**
**669**	HNRNPU Q00839$_{1}$: Heterogeneous nuclear ribonucleoprotein U		11	12	127	126	2	**2.25**
					129	128	2	**0.83**
					131	130	2	**2.21**
**670**	HNRNPU Q00839$_{2}$: Heterogeneous nuclear ribonucleoprotein U		10	12	127	126	2	**2.35**
					129	128	2	**0.50**
					131	130	2	**2.38**
**671**	HNRNPUL1 Q9BUJ2$_{1-4}$: Heterogeneous nuclear ribonucleoprotein U-like protein 1	4/4	3	3	127	126	2	**2.09**
					129	128	2	**1.69**
					131	130	2	**1.78**
**672**	HNRNPUL2 Q1KMD3: Heterogeneous nuclear ribonucleoprotein U-like protein 2		14	30	127	126	25	**2.09**
					129	128	21	**0.96**
					131	130	23	**1.35**
**673**	HP P00738$_{1}$: Haptoglobin	1/2	25	72	127	126	31	**0.80**
					129	128	32	**0.66**
					131	130	32	**1.19**
**674**	HP1BP3 Q5SSJ5$_{1,2}$: Heterochromatin protein 1-binding protein 3	2/3	6	7	127	126	6	**2.22**
					129	128	4	**0.66**
					131	130	6	**1.68**
**675**	HPR P00739$_{1,2}$: Haptoglobin-related protein	2/2	22	75	127	126	35	**0.53**
					129	128	32	**0.89**
					131	130	31	**0.94**
**676**	HPX P02790: Hemopexin		12	24	127	126	22	**1.39**
					129	128	23	**0.59**
					131	130	20	**1.17**
**677**	HRC P23327: Sarcoplasmic reticulum histidine-rich calcium-binding protein		8	13	127	126	12	**1.02**
					129	128	11	**1.87**
					131	130	11	**0.97**
**678**	HRG P04196: Histidine-rich glycoprotein		23	280	127	126	244	**0.86**
					129	128	270	**6.29**
					131	130	266	**1.32**

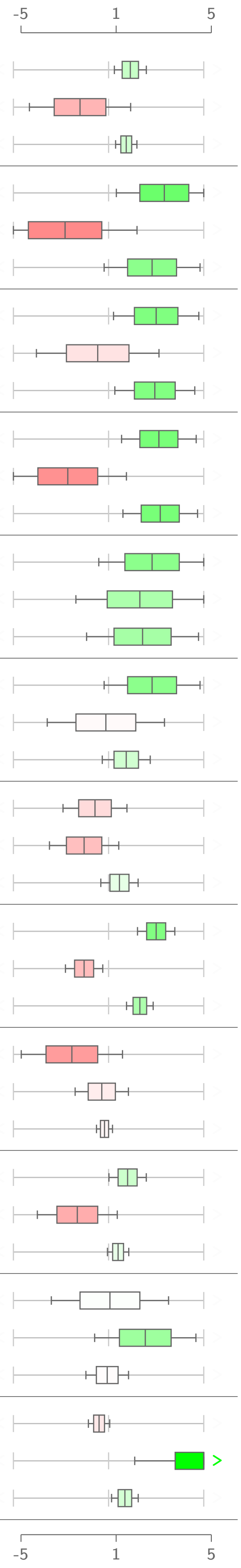

#	protein	group	peptides	spectra	ch1	ch2	quant	ratio
679	HRNR Q86YZ3: Hornerin		3	4	127	126	4	**0.48**
					129	128	4	**5.72**
					131	130	4	**1.22**
680	HS6ST1 O60243$_{1,2}$: Heparan-sulfate 6-O-sulfotransferase 1	2/2	1	1	127	126	1	**0.65**
					129	128	1	**3.56**
					131	130	1	**1.05**
681	HSD17B10 Q99714$_{1,2}$: 3-hydroxyacyl-CoA dehydrogenase type-2	2/2	1	1	127	126	1	**1.42**
					129	128	1	**1.14**
					131	130	1	**1.51**
682	HSP90AA1 P07900$_{1,2}$: Heat shock protein HSP 90-alpha	2/6	34	86	127	126	29	**1.19**
					129	128	26	**0.83**
					131	130	27	**1.12**
683	HSP90AA5P Q58FG0: Putative heat shock protein HSP 90-alpha A5		7	9	127	126	2	**1.21**
					129	128	2	**0.26**
					131	130	2	**0.70**
684	HSP90AB1 P08238: Heat shock protein HSP 90-beta	1/2	32	80	127	126	21	**1.18**
					129	128	20	**0.74**
					131	130	21	**1.14**
685	HSP90AB2P Q58FF8: Putative heat shock protein HSP 90-beta 2		9	13	127	126	1	**0.19**
					129	128	1	**0.24**
					131	130	1	**1.65**
686	HSP90AB4P Q58FF6: Putative heat shock protein HSP 90-beta 4		6	30	127	126	1	**1.32**
					129	128	1	**0.68**
					131	130	1	**1.18**
687	HSP90B1 P14625: Endoplasmin	1/2	37	70	127	126	63	**0.83**
					129	128	64	**1.33**
					131	130	63	**1.10**
688	HSPA1A P0DMV8$_{1}$, HSPA1B P0DMV9: Heat shock 70 kDa protein 1A, Heat shock 70 kDa protein 1B	2/4	21	39	127	126	11	**1.59**
					129	128	12	**0.69**
					131	130	12	**0.97**
689	HSPA1L P34931: Heat shock 70 kDa protein 1-like		12	25	127	126	1	**2.19**
					129	128	1	**0.45**
					131	130	1	**1.08**
690	HSPA2 P54652: Heat shock-related 70 kDa protein 2		18	28	127	126	9	**1.67**
					129	128	9	**0.62**
					131	130	9	**1.13**

#	protein	group	peptides	spectra	ch1	ch2	quant	ratio
**691**	HSPA5 P11021: 78 kDa glucose-regulated protein		28	46	127	126	40	**1.48**
					129	128	39	**0.78**
					131	130	37	**1.15**
**693**	HSPA8 $P11142_1$: Heat shock cognate 71 kDa protein	1/2	22	35	127	126	16	**1.75**
					129	128	15	**0.66**
					131	130	16	**1.18**
**694**	HSPA9 P38646: Stress-70 protein, mitochondrial		8	8	127	126	6	**1.44**
					129	128	5	**1.27**
					131	130	7	**1.41**
**695**	HSPB1 P04792: Heat shock protein beta-1		4	7	127	126	7	**2.11**
					129	128	5	**0.94**
					131	130	7	**1.21**
**696**	HSPB6 O14558: Heat shock protein beta-6		3	4	127	126	4	**1.04**
					129	128	3	**1.38**
					131	130	4	**1.25**
**697**	HSPD1 $P10809_1$: 60 kDa heat shock protein, mitochondrial	1/2	7	8	127	126	6	**1.63**
					129	128	8	**1.00**
					131	130	7	**1.35**
**698**	HSPE1 P61604: 10 kDa heat shock protein, mitochondrial		1	1	127	126	1	**1.59**
					129	128	1	**1.43**
					131	130	1	**2.88**
**699**	HSPG2 P98160: Basement membrane-specific heparan sulfate proteoglycan core protein		93	135	127	126	130	**0.70**
					129	128	132	**1.70**
					131	130	131	**1.11**
**700**	HTRA1 Q92743: Serine protease HTRA1		16	53	127	126	44	**1.33**
					129	128	45	**0.61**
					131	130	44	**0.99**
**701**	HUWE1 $Q7Z6Z7_{1-3}$: E3 ubiquitin-protein ligase HUWE1	3/3	6	7	127	126	7	**0.73**
					129	128	6	**2.32**
					131	130	7	**0.77**
**702**	HYAL1 $Q12794_{1,3,5}$: Hyaluronidase-1	3/3	2	2	127	126	2	**0.51**
					129	128	2	**0.98**
					131	130	2	**1.00**
**703**	HYOU1 $Q9Y4L1_1$: Hypoxia up-regulated protein 1	1/2	18	21	127	126	16	**1.05**
					129	128	15	**0.79**
					131	130	20	**1.07**

#	protein	group	peptides	spectra	ch1	ch2	quant	ratio
704	IFI30 P13284: Gamma-interferon-inducible lysosomal thiol reductase		5	29	127	126	27	**1.41**
					129	128	20	**0.64**
					131	130	24	**0.82**
705	IGF1 $P05019_1$: Insulin-like growth factor I	1/4	3	4	127	126	4	**1.35**
					129	128	4	**3.44**
					131	130	3	**2.27**
706	IGF2 $P01344_{1-3}$: Insulin-like growth factor II	3/3	7	9	127	126	9	**1.76**
					129	128	9	**2.09**
					131	130	8	**0.94**
707	IGF2R P11717: Cation-independent mannose-6-phosphate receptor		6	6	127	126	6	**0.95**
					129	128	4	**1.26**
					131	130	6	**1.11**
708	IGFALS $P35858_{1,2}$: Insulin-like growth factor-binding protein complex acid labile subunit	2/2	16	21	127	126	21	**1.02**
					129	128	19	**1.00**
					131	130	20	**1.08**
709	IGFBP1 P08833: Insulin-like growth factor-binding protein 1		8	16	127	126	14	**0.97**
					129	128	16	**1.59**
					131	130	14	**0.93**
710	IGFBP2 P18065: Insulin-like growth factor-binding protein 2		16	37	127	126	36	**1.22**
					129	128	35	**3.32**
					131	130	35	**1.13**
711	IGFBP3 $P17936_{1,2}$: Insulin-like growth factor-binding protein 3	2/2	19	40	127	126	36	**1.44**
					129	128	36	**1.93**
					131	130	34	**1.06**
712	IGFBP4 $P22692_1$: Insulin-like growth factor-binding protein 4	1/2	10	19	127	126	17	**1.50**
					129	128	14	**3.75**
					131	130	17	**1.19**
713	IGFBP5 P24593: Insulin-like growth factor-binding protein 5		12	35	127	126	32	**1.72**
					129	128	35	**3.19**
					131	130	34	**1.00**
714	IGFBP6 P24592: Insulin-like growth factor-binding protein 6		7	12	127	126	11	**1.52**
					129	128	11	**2.40**
					131	130	12	**0.98**
715	IGFBP7 $Q16270_1$: Insulin-like growth factor-binding protein 7	1/2	12	18	127	126	18	**1.23**
					129	128	15	**1.23**
					131	130	15	**1.19**

#	protein	group	peptides	spectra	ch1	ch2	quant	ratio
**716**	IGHA1 P01876: Ig alpha-1 chain C region		14	114	127	126	40	**0.90**
					129	128	34	**0.81**
					131	130	35	**1.09**
**717**	IGHA2 P01877: Ig alpha-2 chain C region		9	76	127	126	3	**1.25**
					129	128	3	**1.16**
					131	130	3	**1.14**
**718**	IGHG1 P01857: Ig gamma-1 chain C region		15	309	127	126	127	**0.92**
					129	128	122	**0.65**
					131	130	130	**1.04**
**719**	IGHG2 P01859: Ig gamma-2 chain C region		11	125	127	126	8	**1.02**
					129	128	8	**0.53**
					131	130	7	**1.09**
**720**	IGHG3 P01860: Ig gamma-3 chain C region		14	194	127	126	30	**0.73**
					129	128	28	**0.57**
					131	130	27	**1.09**
**721**	IGHG4 P01861: Ig gamma-4 chain C region		10	107	127	126	7	**0.68**
					129	128	7	**0.73**
					131	130	6	**1.12**
**722**	IGHM $P01871_1$: Ig mu chain C region	1/2	19	57	127	126	25	**1.04**
					129	128	25	**0.46**
					131	130	26	**1.14**
**723**	IGHV1-2 P23083, A0A0C4DH31: Immunoglobulin heavy variable 1-2	2/2	2	7	127	126	4	**0.93**
					129	128	5	**0.64**
					131	130	6	**1.03**
**725**	IGHV1-69 P01742: Immunoglobulin heavy variable 1-69		4	11	127	126	6	**0.82**
					129	128	5	**0.75**
					131	130	6	**1.09**
**726**	IGHV3-13 P01766: Immunoglobulin heavy variable 3-13		1	1	127	126	1	**0.96**
					129	128	1	**0.50**
					131	130	1	**1.16**
**727**	IGHV3-30 P01768: Immunoglobulin heavy variable 3-30	1/2	2	5	127	126	2	**0.89**
					129	128	2	**0.36**
					131	130	2	**0.94**
**729**	IGHV3-7 P01780: Immunoglobulin heavy variable 3-7	1/5	2	4	127	126	1	**0.92**
					129	128	1	**0.44**
					131	130	1	**1.04**

-5 1 5

#	protein	group	peptides	spectra	ch1	ch2	quant	ratio
**730**	IGHV4-39 P01824: Immunoglobulin heavy variable 4-39	1/3	4	11	127	126	10	**0.81**
					129	128	11	**0.42**
					131	130	10	**0.99**
**731**	IGKC P01834: Ig kappa chain C region		6	106	127	126	94	**0.81**
					129	128	69	**0.77**
					131	130	99	**1.09**
**732**	IGKV1-17 P01599: Immunoglobulin kappa variable 1-17	1/7	3	9	127	126	2	**1.09**
					129	128	2	**0.76**
					131	130	2	**1.05**
**733**	IGKV1-5 P01602: Immunoglobulin kappa variable 1-5		2	8	127	126	1	**0.79**
					129	128	1	**0.76**
					131	130	1	**1.00**
**734**	IGKV2-30 P06310: Immunoglobulin kappa variable 2-30		2	7	127	126	3	**1.08**
					129	128	3	**1.02**
					131	130	3	**1.13**
**735**	IGKV2D-40 P01614: Immunoglobulin kappa variable 2D-40	1/4	3	11	127	126	6	**1.13**
					129	128	7	**1.74**
					131	130	6	**0.97**
**736**	IGKV3-11 P04433: Immunoglobulin kappa variable 3-11		3	15	127	126	3	**1.25**
					129	128	3	**0.78**
					131	130	3	**1.02**
**737**	IGKV3-15 P01624: Immunoglobulin kappa variable 3-15		4	16	127	126	4	**1.07**
					129	128	3	**1.09**
					131	130	3	**1.07**
**738**	IGKV3-20 P01619: Immunoglobulin kappa variable 3-20		4	18	127	126	2	**0.85**
					129	128	2	**1.02**
					131	130	2	**1.10**
**739**	IGKV4-1 P06312: Immunoglobulin kappa variable 4-1		4	17	127	126	15	**0.85**
					129	128	13	**1.09**
					131	130	17	**1.14**
**740**	IGLC2 P0CG05: Ig lambda-2 chain C regions	1/3	6	217	127	126	54	**0.93**
					129	128	48	**0.83**
					131	130	54	**1.06**
**741**	IGLC7 A0M8Q6: Ig lambda-7 chain C region		4	111	127	126	2	**1.18**
					129	128	2	**0.96**
					131	130	2	**1.16**

#	protein	group	peptides	spectra	ch1	ch2	quant	ratio
**742**	IGLL5 B9A064$_1$: Immunoglobulin lambda-like polypeptide 5	1/2	7	151	127	126	18	**0.90**
					129	128	20	**0.66**
					131	130	20	**1.08**
**743**	IGLV1-47 P01700: Immunoglobulin lambda variable 1-47	1/2	2	2	127	126	2	**0.96**
					129	128	2	**1.20**
					131	130	2	**1.28**
**744**	IGLV2-11 P01706, IGLV2-8 P01709: Immunoglobulin lambda variable 2-11, Immunoglobulin lambda variable 2-8	2/2	1	3	127	126	3	**0.85**
					129	128	3	**0.61**
					131	130	3	**0.99**
**745**	IGLV2-14 P01704: Immunoglobulin lambda variable 2-14		1	2	127	126	2	**0.58**
					129	128	2	**0.47**
					131	130	2	**1.10**
**746**	IGLV2-23 P01705: Immunoglobulin lambda variable 2-23		1	4	127	126	4	**0.96**
					129	128	4	**0.87**
					131	130	3	**1.05**
**747**	IGLV3-19 P01714: Immunoglobulin lambda variable 3-19		2	3	127	126	3	**1.04**
					129	128	3	**0.95**
					131	130	3	**0.98**
**748**	IGLV3-21 P80748: Immunoglobulin lambda variable 3-21		2	3	127	126	3	**1.25**
					129	128	3	**1.08**
					131	130	3	**1.09**
**749**	IGLV3-25 P01717: Immunoglobulin lambda variable 3-25		2	4	127	126	2	**1.30**
					129	128	2	**0.98**
					131	130	2	**1.21**
**750**	IGLV3-27 P01718: Immunoglobulin lambda variable 3-27		2	3	127	126	1	**1.10**
					129	128	1	**0.67**
					131	130	1	**1.03**
**751**	IGLV7-43 P04211: Immunoglobulin lambda variable 7-43		2	2	127	126	2	**0.81**
					129	128	2	**0.87**
					131	130	2	**0.96**
**752**	IL18 Q14116$_{1,2}$: Interleukin-18	2/2	2	2	127	126	2	**1.43**
					129	128	2	**0.58**
					131	130	2	**1.14**
**753**	ILF3 Q12906$_{1-7}$: Interleukin enhancer-binding factor 3	7/7	6	7	127	126	7	**0.97**
					129	128	7	**0.99**
					131	130	6	**1.11**

#	protein	group	peptides	spectra	ch1	ch2	quant	ratio
**754**	ILK Q13418$_{1-3}$: Integrin-linked protein kinase	3/3	2	2	127	126	2	**0.89**
					129	128	2	**1.11**
					131	130	2	**0.89**
**755**	IMMT Q16891$_{1-4}$: MICOS complex subunit MIC60	4/4	3	3	127	126	2	**2.04**
					129	128	3	**0.64**
					131	130	3	**1.51**
**756**	IMPAD1 Q9NX62: Inositol monophosphatase 3		5	5	127	126	4	**0.71**
					129	128	4	**1.01**
					131	130	5	**0.92**
**757**	IMUP Q9GZP8$_{1,2}$: Immortalization up-regulated protein	2/2	1	1	127	126	1	**2.04**
					129	128	1	**0.80**
					131	130	1	**1.54**
**758**	INF2 Q27J81$_{1,2}$: Inverted formin-2	2/2	3	3	127	126	3	**1.29**
					129	128	3	**1.44**
					131	130	3	**1.31**
**759**	INHBB P09529: Inhibin beta B chain		5	6	127	126	6	**0.99**
					129	128	6	**1.08**
					131	130	6	**0.89**
**760**	INHBC P55103: Inhibin beta C chain		10	20	127	126	18	**0.95**
					129	128	16	**1.02**
					131	130	17	**0.89**
**761**	INHBE P58166: Inhibin beta E chain		4	7	127	126	7	**1.31**
					129	128	7	**1.65**
					131	130	6	**0.90**
**762**	INSL3 P51460$_{1}$: Insulin-like 3		3	3	127	126	3	**2.07**
					129	128	3	**3.83**
					131	130	3	**0.99**
**763**	IPO5 O00410$_{1,3}$: Importin-5	2/2	2	2	127	126	2	**1.11**
					129	128	2	**1.40**
					131	130	2	**1.19**
**764**	IQGAP1 P46940: Ras GTPase-activating-like protein IQGAP1		22	28	127	126	27	**1.02**
					129	128	25	**0.94**
					131	130	26	**1.09**
**765**	ISLR O14498: Immunoglobulin superfamily containing leucine-rich repeat protein		7	10	127	126	9	**0.56**
					129	128	10	**1.63**
					131	130	10	**0.96**

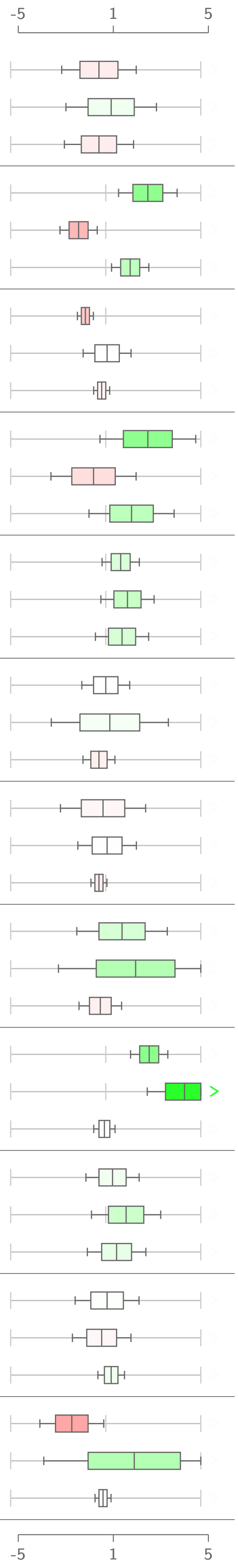

#	protein	group	peptides	spectra	ch1	ch2	quant	ratio
**766**	ITGA11 Q9UKX5$_{1,2}$: Integrin alpha-11	2/2	3	5	127	126	5	**0.75**
					129	128	4	**2.01**
					131	130	5	**0.88**
**767**	ITGA7 Q13683$_{1,3,7,9,10}$: Integrin alpha-7 [Cleaved into: Integrin alpha-7 heavy chain; Integrin alpha ...	5/5	2	2	127	126	2	**1.09**
					129	128	2	**0.90**
					131	130	2	**1.16**
**768**	ITGAM P11215$_{1,2}$: Integrin alpha-M	2/2	4	4	127	126	3	**0.52**
					129	128	3	**0.89**
					131	130	3	**0.95**
**769**	ITGB1 P05556$_{1}$: Integrin beta-1	1/5	11	13	127	126	11	**0.51**
					129	128	12	**1.28**
					131	130	12	**1.05**
**770**	ITGB2 P05107: Integrin beta-2		4	4	127	126	3	**0.49**
					129	128	4	**0.80**
					131	130	4	**0.96**
**771**	ITGBL1 O95965$_{1}$: Integrin beta-like protein 1	1/3	17	30	127	126	20	**1.57**
					129	128	18	**1.52**
					131	130	22	**1.07**
**772**	ITIH1 P19827$_{1}$: Inter-alpha-trypsin inhibitor heavy chain H1	1/3	24	120	127	126	116	**0.29**
					129	128	116	**3.17**
					131	130	116	**0.76**
**773**	ITIH2 P19823: Inter-alpha-trypsin inhibitor heavy chain H2		30	325	127	126	306	**0.37**
					129	128	311	**2.01**
					131	130	308	**0.75**
**774**	ITIH3 Q06033$_{1,2}$: Inter-alpha-trypsin inhibitor heavy chain H3	2/2	39	113	127	126	111	**0.35**
					129	128	109	**1.63**
					131	130	108	**0.83**
**775**	ITIH4 Q14624$_{1}$: Inter-alpha-trypsin inhibitor heavy chain H4		36	297	127	126	11	**0.62**
					129	128	11	**0.76**
					131	130	11	**1.02**
**776**	ITIH4 Q14624$_{2,3}$: Inter-alpha-trypsin inhibitor heavy chain H4	2/3	33	290	127	126	4	**0.25**
					129	128	3	**1.36**
					131	130	4	**0.94**
**777**	ITIH5 Q86UX2$_{1}$: Inter-alpha-trypsin inhibitor heavy chain H5	1/4	15	20	127	126	19	**0.69**
					129	128	18	**1.20**
					131	130	18	**0.95**

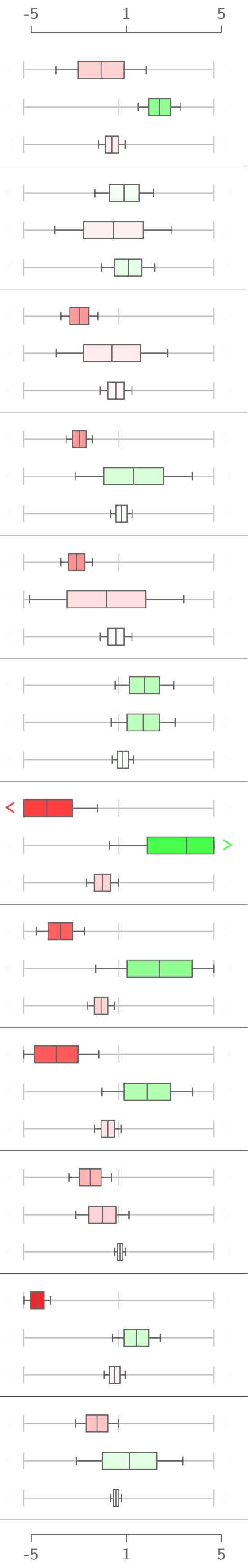

#	protein	group	peptides	spectra	ch1	ch2	quant	ratio
778	ITLN1 Q8WWA0: Intelectin-1		16	246	127	126	138	**1.99**
					129	128	123	**0.67**
					131	130	132	**0.88**
779	ITLN2 Q8WWU7$_{1,2}$: Intelectin-2	2/2	8	107	127	126	1	**1.53**
					129	128		
					131	130	1	**0.53**
780	ITM2A O43736$_{1,2}$: Integral membrane protein 2A	2/2	1	2	127	126	2	**1.74**
					129	128	2	**1.18**
					131	130	2	**1.20**
781	ITM2B Q9Y287$_{1}$: Integral membrane protein 2B		2	2	127	126	2	**1.46**
					129	128	2	**1.62**
					131	130	2	**1.06**
782	JCHAIN P01591: Immunoglobulin J chain		6	11	127	126	8	**1.10**
					129	128	8	**1.11**
					131	130	10	**1.05**
783	JUP P14923: Junction plakoglobin		4	5	127	126	5	**0.58**
					129	128	3	**2.49**
					131	130	4	**1.13**
784	KCTD12 Q96CX2: BTB/POZ domain-containing protein KCTD12	1/2	7	9	127	126	8	**0.50**
					129	128	7	**0.83**
					131	130	8	**0.91**
785	KCTD16 Q68DU8: BTB/POZ domain-containing protein KCTD16		4	20	127	126	16	**1.13**
					129	128	9	**0.52**
					131	130	18	**1.01**
786	KHDRBS1 Q07666$_{1,2}$: KH domain-containing, RNA-binding, signal transduction-associated protein 1	2/3	5	6	127	126	6	**2.01**
					129	128	3	**0.83**
					131	130	6	**1.49**
787	KHSRP Q92945: Far upstream element-binding protein 2		4	5	127	126	4	**2.49**
					129	128	4	**0.82**
					131	130	4	**1.47**
788	KIAA0319L Q8IZA0$_{1,2}$: Dyslexia-associated protein KIAA0319-like protein	2/2	4	4	127	126	4	**0.99**
					129	128	4	**1.99**
					131	130	3	**0.92**
789	KIF5B P33176: Kinesin-1 heavy chain	1/3	8	8	127	126	7	**1.33**
					129	128	8	**1.18**
					131	130	8	**1.12**

-5 1 5

#	protein	group	peptides	spectra	ch1	ch2	quant	ratio
791	KLK10 O43240: Kallikrein-10		2	2	127	126	2	**1.08**
					129	128	2	**1.71**
					131	130	2	**0.82**
792	KLKB1 P03952: Plasma kallikrein	1/2	32	78	127	126	74	**1.06**
					129	128	75	**0.71**
					131	130	75	**1.06**
793	KMT2C Q8NEZ4$_{1,3}$: Histone-lysine N-methyltransferase 2C	2/2	14	47	127	126	19	**1.86**
					129	128	16	**1.24**
					131	130	18	**1.04**
794	KNG1 P01042$_1$: Kininogen-1		41	303	127	126	30	**1.22**
					129	128	32	**2.18**
					131	130	31	**1.09**
795	KNG1 P01042$_2$: Kininogen-1	1/2	32	328	127	126	53	**0.64**
					129	128	51	**1.70**
					131	130	56	**1.09**
796	KPNB1 Q14974$_1$: Importin subunit beta-1	1/2	8	8	127	126	8	**1.27**
					129	128	7	**0.95**
					131	130	7	**1.13**
797	KPRP Q5T749: Keratinocyte proline-rich protein		6	6	127	126	6	**0.67**
					129	128	5	**3.36**
					131	130	6	**1.10**
798	KRT1 P04264: Keratin, type II cytoskeletal 1		46	161	127	126	134	**0.56**
					129	128	128	**3.69**
					131	130	128	**0.90**
799	KRT10 P13645: Keratin, type I cytoskeletal 10		36	135	127	126	68	**0.55**
					129	128	60	**4.09**
					131	130	65	**0.78**
800	KRT13 P13646$_{1,3}$: Keratin, type I cytoskeletal 13	2/5	22	56	127	126	15	**0.30**
					129	128	15	**2.47**
					131	130	15	**0.15**
801	KRT14 P02533: Keratin, type I cytoskeletal 14		29	95	127	126	8	**0.53**
					129	128	7	**4.43**
					131	130	8	**0.87**
802	KRT15 P19012$_1$: Keratin, type I cytoskeletal 15	1/2	14	65	127	126	2	**1.17**
					129	128	2	**1.27**
					131	130	2	**1.01**

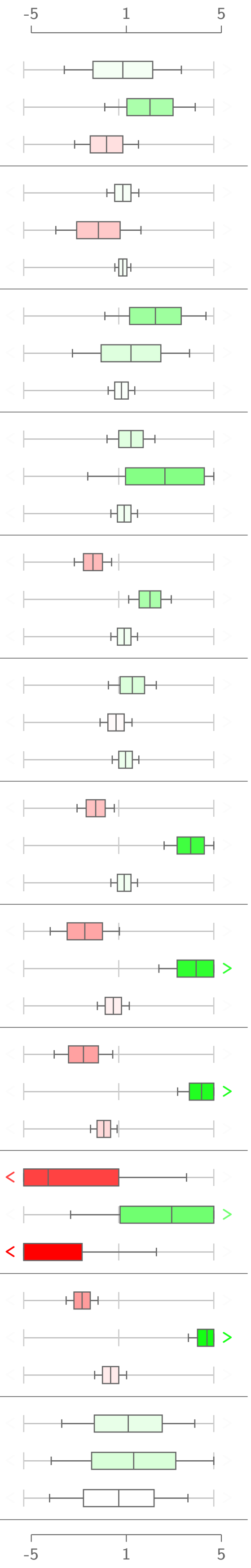

#	protein	group	peptides	spectra	ch1	ch2	quant	ratio
**803**	KRT16 P08779: Keratin, type I cytoskeletal 16		24	72	127	126	13	**0.53**
					129	128	11	**3.05**
					131	130	13	**1.14**
**804**	KRT17 Q04695: Keratin, type I cytoskeletal 17		23	75	127	126	7	**0.59**
					129	128	6	**2.28**
					131	130	6	**0.86**
**805**	KRT18 P05783: Keratin, type I cytoskeletal 18		33	129	127	126	107	**2.01**
					129	128	106	**0.28**
					131	130	108	**1.71**
**806**	KRT19 P08727: Keratin, type I cytoskeletal 19	1/4	37	207	127	126	122	**2.05**
					129	128	110	**0.20**
					131	130	117	**1.76**
**807**	KRT2 P35908: Keratin, type II cytoskeletal 2 epidermal		35	84	127	126	45	**0.51**
					129	128	45	**3.83**
					131	130	44	**1.06**
**809**	KRT24 Q2M2I5: Keratin, type I cytoskeletal 24		5	27	127	126	2	**1.05**
					129	128	2	**1.10**
					131	130	2	**1.00**
**810**	KRT25 Q7Z3Z0: Keratin, type I cytoskeletal 25	1/2	10	23	127	126	4	**0.82**
					129	128	5	**3.89**
					131	130	5	**0.69**
**811**	KRT27 Q7Z3Y8: Keratin, type I cytoskeletal 27		9	34	127	126	1	**0.63**
					129	128	1	**2.93**
					131	130	1	**0.69**
**815**	KRT36 O76013$_{1,2}$: Keratin, type I cuticular Ha6	2/2	2	7	127	126	1	**0.10**
					129	128	1	**1.37**
					131	130	1	**1.26**
**816**	KRT4 P19013: Keratin, type II cytoskeletal 4		9	23	127	126	3	**0.13**
					129	128	3	**0.55**
					131	130	3	**0.08**
**817**	KRT5 P13647: Keratin, type II cytoskeletal 5		36	92	127	126	39	**0.85**
					129	128	34	**1.27**
					131	130	37	**1.14**
**818**	KRT6A P02538: Keratin, type II cytoskeletal 6A	1/4	33	73	127	126	1	**0.33**
					129	128	1	**2.32**
					131	130	1	**0.75**

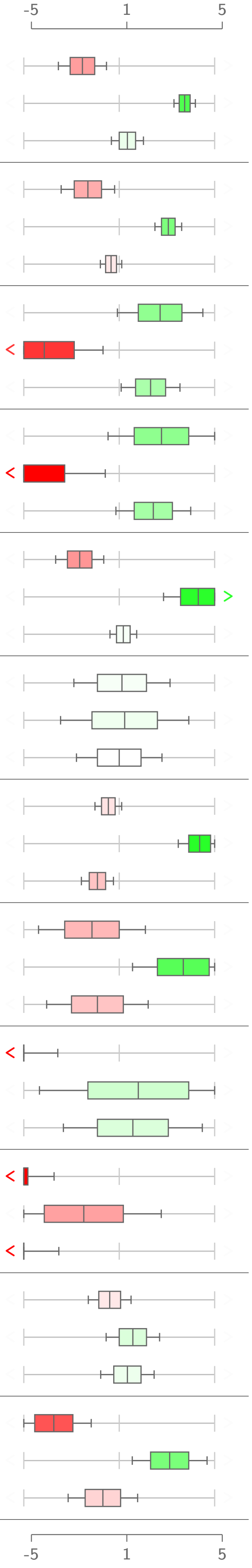

#	protein	group	peptides	spectra	ch1	ch2	quant	ratio
821	KRT7 P08729: Keratin, type II cytoskeletal 7		32	116	127	126	53	**2.05**
					129	128	54	**0.22**
					131	130	59	**2.09**
822	KRT71 Q3SY84: Keratin, type II cytoskeletal 71	1/2	9	12	127	126	4	**0.71**
					129	128	3	**2.60**
					131	130	4	**0.92**
826	KRT77 Q7Z794: Keratin, type II cytoskeletal 1b		7	18	127	126	2	**0.54**
					129	128	2	**1.99**
					131	130	2	**0.38**
827	KRT78 Q8N1N4$_1$: Keratin, type II cytoskeletal 78	1/2	7	21	127	126	4	**0.46**
					129	128	4	**3.90**
					131	130	4	**0.68**
828	KRT79 Q5XKE5: Keratin, type II cytoskeletal 79		13	31	127	126	2	**1.33**
					129	128	2	**0.39**
					131	130	2	**0.94**
829	KRT8 P05787$_{1,2}$: Keratin, type II cytoskeletal 8	2/3	42	188	127	126	117	**2.15**
					129	128	113	**0.18**
					131	130	113	**1.71**
830	KRT80 Q6KB66$_{1-3}$: Keratin, type II cytoskeletal 80	3/3	1	1	127	126	1	**0.39**
					129	128	1	**1.22**
					131	130	1	**0.93**
831	KRT81 Q14533, KRT83 P78385, KRT86 O43790: Keratin, type II cuticular Hb1, Keratin, type II cuticular Hb3, . . .	3/5	3	8	127	126	2	**0.27**
					129	128	2	**1.15**
					131	130	2	**1.18**
832	KRT9 P35527: Keratin, type I cytoskeletal 9		29	116	127	126	90	**0.54**
					129	128	84	**4.00**
					131	130	88	**1.23**
833	LACRT Q9GZZ8: Extracellular glycoprotein lacritin		2	2	127	126	2	**0.84**
					129	128	2	**1.90**
					131	130	2	**0.13**
834	LAMA2 P24043: Laminin subunit alpha-2		40	62	127	126	52	**1.41**
					129	128	50	**1.22**
					131	130	48	**1.39**
835	LAMA4 Q16363$_{1,2}$: Laminin subunit alpha-4	2/2	32	44	127	126	41	**0.78**
					129	128	41	**1.68**
					131	130	42	**1.15**

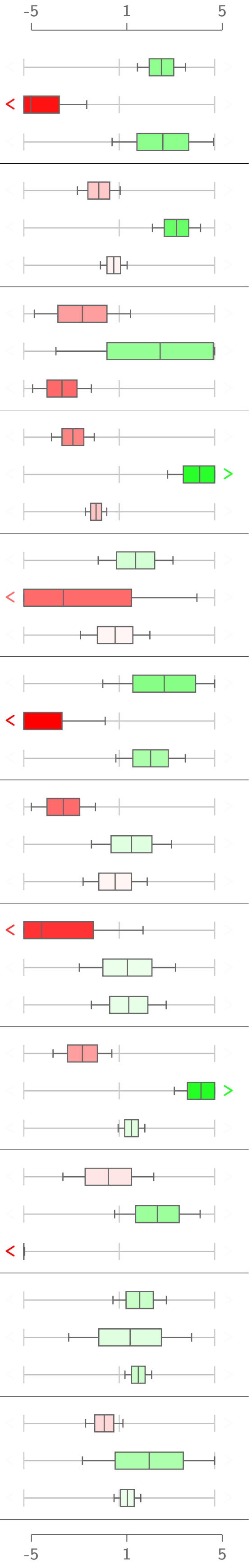

#	protein	group	peptides	spectra	ch1	ch2	quant	ratio
**836**	LAMA5 O15230$_1$: Laminin subunit alpha-5	1/2	12	15	127	126	12	**0.98**
					129	128	10	**1.63**
					131	130	13	**1.12**
**837**	LAMB1 P07942: Laminin subunit beta-1		43	53	127	126	48	**1.01**
					129	128	47	**1.36**
					131	130	46	**1.29**
**838**	LAMB2 P55268: Laminin subunit beta-2		33	41	127	126	33	**1.02**
					129	128	35	**1.38**
					131	130	36	**1.23**
**839**	LAMC1 P11047: Laminin subunit gamma-1		50	79	127	126	72	**0.98**
					129	128	66	**1.33**
					131	130	69	**1.30**
**840**	LAP3 P28838$_{1,2}$: Cytosol aminopeptidase	2/2	1	1	127	126	1	**1.01**
					129	128	1	**1.21**
					131	130	1	**0.92**
**841**	LARS Q9P2J5$_{1,2}$: Leucine--tRNA ligase, cytoplasmic	2/2	4	4	127	126	4	**1.31**
					129	128	3	**0.90**
					131	130	4	**1.12**
**842**	LBP P18428: Lipopolysaccharide-binding protein		15	233	127	126	210	**0.48**
					129	128	221	**2.04**
					131	130	221	**0.86**
**843**	LCAT P04180: Phosphatidylcholine-sterol acyltransferase	1/2	11	48	127	126	47	**0.62**
					129	128	42	**1.20**
					131	130	44	**0.88**
**844**	LCN1 P31025: Lipocalin-1	1/2	10	17	127	126	16	**0.78**
					129	128	17	**2.05**
					131	130	15	**0.12**
**845**	LCP1 P13796$_1$: Plastin-2	1/3	30	71	127	126	54	**0.22**
					129	128	53	**4.14**
					131	130	53	**0.81**
**846**	LDHA P00338$_{1-5}$: L-lactate dehydrogenase A chain	5/8	2	2	127	126	2	**0.73**
					129	128	2	**1.55**
					131	130	2	**1.03**
**847**	LDLR P01130$_{1,5}$: Low-density lipoprotein receptor	2/5	6	18	127	126	8	**1.21**
					129	128	5	**1.44**
					131	130	12	**1.30**

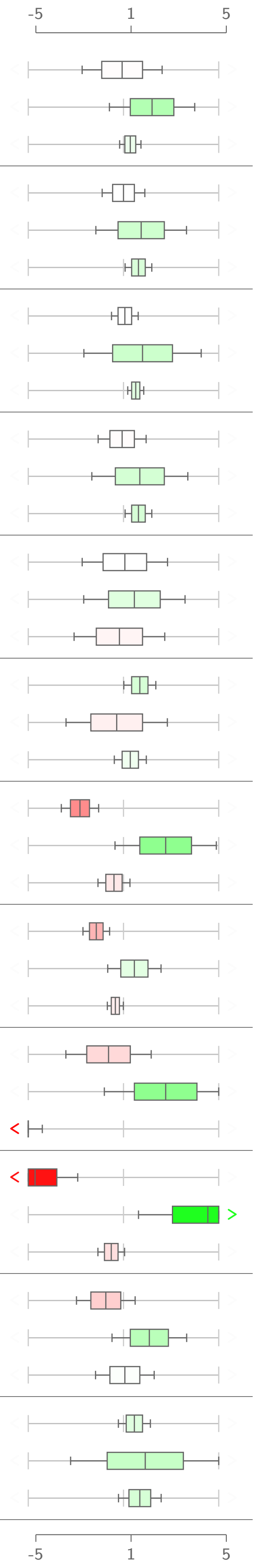

#	protein	group	peptides	spectra	ch1	ch2	quant	ratio
**848**	LECT2 O14960: Leukocyte cell-derived chemotaxin-2		5	9	127	126	8	**2.30**
					129	128	9	**2.47**
					131	130	8	**0.95**
**849**	LEFTY1 O75610, LEFTY2 O00292$_1$: Left-right determination factor 1, Left-right determination factor 2	2/3	5	7	127	126	6	**1.25**
					129	128	7	**0.87**
					131	130	6	**1.02**
**850**	LEP P41159: Leptin		4	6	127	126	5	**1.56**
					129	128	6	**2.52**
					131	130	5	**1.06**
**851**	LFNG Q8NES3$_{1,3}$: Beta-1,3-N-acetylglucosaminyltransferase lunatic fringe	2/4	5	7	127	126	7	**0.80**
					129	128	7	**1.10**
					131	130	5	**0.97**
**852**	LGALS1 P09382: Galectin-1		8	26	127	126	26	**1.69**
					129	128	26	**1.23**
					131	130	23	**1.00**
**853**	LGALS3 P17931: Galectin-3		7	10	127	126	10	**0.96**
					129	128	10	**1.18**
					131	130	9	**1.06**
**854**	LGALS3BP Q08380: Galectin-3-binding protein		18	43	127	126	42	**1.28**
					129	128	37	**1.06**
					131	130	43	**1.16**
**855**	LGALS4 P56470: Galectin-4		2	2	127	126	2	**0.46**
					129	128	2	**1.47**
					131	130	2	**1.03**
**856**	LGALS7 P47929: Galectin-7		4	5	127	126	5	**0.85**
					129	128	4	**2.79**
					131	130	4	**0.75**
**857**	LGI2 Q8N0V4: Leucine-rich repeat LGI family member 2		3	3	127	126	3	**0.91**
					129	128	3	**0.78**
					131	130	3	**1.43**
**858**	LGMN Q99538$_{1-3}$: Legumain	3/3	4	6	127	126	6	**1.33**
					129	128	5	**1.46**
					131	130	6	**0.90**
**859**	LIPA P38571$_{1,2}$: Lysosomal acid lipase/cholesteryl ester hydrolase	2/2	2	2	127	126	2	**0.59**
					129	128	2	**1.72**
					131	130	2	**0.91**

#	protein	group	peptides	spectra	ch1	ch2	quant	ratio
**860**	LMAN1 P49257: Protein ERGIC-53		8	9	127	126	7	**1.70**
					129	128	8	**0.67**
					131	130	7	**1.27**
**861**	LMAN2 Q12907: Vesicular integral-membrane protein VIP36		3	3	127	126	3	**0.83**
					129	128	3	**1.26**
					131	130	3	**1.01**
**862**	LMNA P02545$_{1-3,6}$: Prelamin-A/C [Cleaved into: Lamin-A/C	4/6	31	50	127	126	43	**1.62**
					129	128	42	**0.90**
					131	130	45	**1.56**
**863**	LMNB1 P20700: Lamin-B1		8	9	127	126	4	**2.24**
					129	128	4	**1.13**
					131	130	4	**1.20**
**864**	LMNB2 Q03252: Lamin-B2		8	10	127	126	2	**1.88**
					129	128	2	**0.98**
					131	130	2	**1.26**
**865**	LOX P28300: Protein-lysine 6-oxidase		12	24	127	126	21	**1.23**
					129	128	20	**1.59**
					131	130	20	**1.06**
**866**	LOXL1 Q08397: Lysyl oxidase homolog 1		16	36	127	126	31	**1.25**
					129	128	30	**1.10**
					131	130	32	**1.17**
**867**	LOXL2 Q9Y4K0: Lysyl oxidase homolog 2		12	16	127	126	12	**1.26**
					129	128	11	**1.15**
					131	130	11	**1.02**
**868**	LOXL3 P58215$_1$: Lysyl oxidase homolog 3	1/2	5	8	127	126	4	**1.36**
					129	128	5	**1.04**
					131	130	4	**1.07**
**869**	LPA P08519: Apolipoprotein(a)		17	27	127	126	27	**0.15**
					129	128	27	**3.25**
					131	130	25	**1.00**
**870**	LPAR3 Q9UBY5: Lysophosphatidic acid receptor 3		4	130	127	126	97	**1.03**
					129	128	43	**0.45**
					131	130	97	**0.84**
**871**	LPL P06858: Lipoprotein lipase		6	6	127	126	5	**0.97**
					129	128	6	**1.28**
					131	130	5	**0.99**

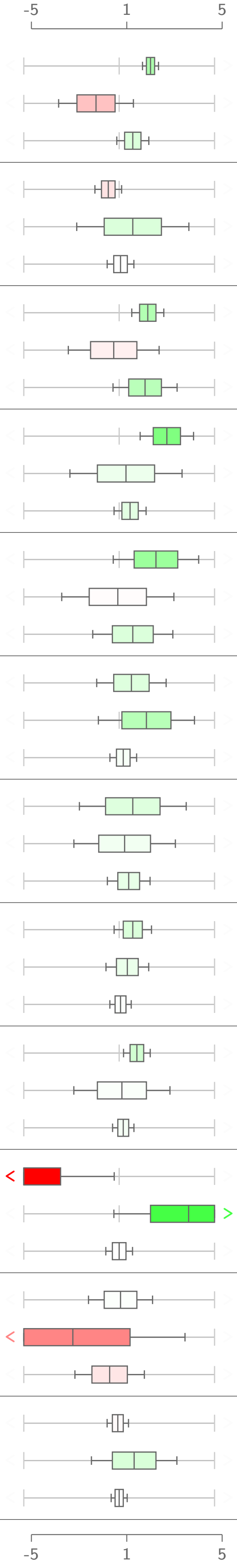

#	protein	group	peptides	spectra	ch1	ch2	quant	ratio
**872**	LRG1 P02750: Leucine-rich alpha-2-glycoprotein		7	8	127	126	6	**1.04**
					129	128	5	**1.27**
					131	130	6	**1.28**
**873**	LRP1 Q07954$_1$: Prolow-density lipoprotein receptor-related protein 1		59	132	127	126	95	**1.22**
					129	128	76	**1.04**
					131	130	82	**1.14**
**874**	LRP1B Q9NZR2: Low-density lipoprotein receptor-related protein 1B		24	106	127	126	30	**0.99**
					129	128	23	**1.15**
					131	130	33	**1.01**
**875**	LRP8 Q14114$_{1,3}$: Low-density lipoprotein receptor-related protein 8	2/4	8	39	127	126	14	**0.70**
					129	128	10	**1.05**
					131	130	22	**0.92**
**876**	LRRC57 Q8N9N7: Leucine-rich repeat-containing protein 57		2	2	127	126	2	**0.71**
					129	128	2	**2.28**
					131	130	2	**1.03**
**877**	LRRFIP1 Q32MZ4$_{1-3}$: Leucine-rich repeat flightless-interacting protein 1	3/3	2	2	127	126	2	**1.28**
					129	128	2	**1.18**
					131	130	2	**1.17**
**878**	LRRN4 Q8WUT4: Leucine-rich repeat neuronal protein 4		3	3	127	126	3	**0.49**
					129	128	3	**3.70**
					131	130	3	**1.13**
**879**	LSP1 P33241$_{1,3}$: Lymphocyte-specific protein 1	2/2	1	1	127	126	1	**1.07**
					129	128	1	**1.62**
					131	130	1	**0.96**
**880**	LSR Q86X29$_{1,4}$: Lipolysis-stimulated lipoprotein receptor	2/4	7	17	127	126	1	**1.47**
					129	128	1	**2.96**
					131	130	1	**1.05**
**881**	LSR Q86X29$_3$: Lipolysis-stimulated lipoprotein receptor	1/2	7	18	127	126	2	**1.07**
					129	128	2	**1.80**
					131	130	2	**1.01**
**882**	LTBP1 Q14766$_{1,4}$: Latent-transforming growth factor beta-binding protein 1	2/4	37	62	127	126	8	**1.61**
					129	128	7	**1.09**
					131	130	8	**1.11**
**883**	LTBP1 Q14766$_3$: Latent-transforming growth factor beta-binding protein 1		32	54	127	126	1	**1.43**
					129	128	1	**1.67**
					131	130	1	**1.05**

#	protein	group	peptides	spectra	ch1	ch2	quant	ratio
**884**	LTBP2 Q14767: Latent-transforming growth factor beta-binding protein 2		38	64	127	126	53	**1.44**
					129	128	52	**1.76**
					131	130	57	**1.23**
**885**	LTBP3 Q9NS15$_1$: Latent-transforming growth factor beta-binding protein 3	1/2	12	13	127	126	10	**1.19**
					129	128	11	**1.10**
					131	130	10	**1.15**
**886**	LTBP4 Q8N2S1$_2$: Latent-transforming growth factor beta-binding protein 4	1/4	43	73	127	126	68	**1.23**
					129	128	59	**1.49**
					131	130	69	**1.24**
**887**	LTF P02788$_1$: Lactotransferrin	1/2	33	49	127	126	48	**1.09**
					129	128	48	**1.44**
					131	130	45	**0.78**
**888**	LUM P51884: Lumican		14	259	127	126	254	**1.57**
					129	128	220	**1.26**
					131	130	216	**1.21**
**889**	LY96 Q9Y6Y9$_{1,2}$: Lymphocyte antigen 96	2/2	1	1	127	126	1	**0.67**
					129	128	1	**0.91**
					131	130	1	**1.26**
**890**	LYPLA1 O75608$_{1,2}$: Acyl-protein thioesterase 1	2/2	3	5	127	126	4	**0.90**
					129	128	2	**1.23**
					131	130	3	**0.98**
**891**	LYZ P61626: Lysozyme C		13	396	127	126	388	**1.73**
					129	128	391	**2.88**
					131	130	354	**1.01**
**892**	M6PR P20645: Cation-dependent mannose-6-phosphate receptor		2	3	127	126	3	**2.09**
					129	128	3	**0.55**
					131	130	3	**1.47**
**893**	MACF1 Q9UPN3$_1$: Microtubule-actin cross-linking factor 1, isoforms 1/2/3/5	1/5	9	10	127	126	7	**0.99**
					129	128	6	**0.88**
					131	130	7	**0.98**
**894**	MAN1A1 P33908$_1$: Mannosyl-oligosaccharide 1,2-alpha-mannosidase IA	1/2	15	21	127	126	18	**0.54**
					129	128	18	**1.75**
					131	130	19	**0.80**
**895**	MAN1C1 Q9NR34: Mannosyl-oligosaccharide 1,2-alpha-mannosidase IC		2	2	127	126	2	**0.72**
					129	128	2	**0.66**
					131	130	2	**0.90**

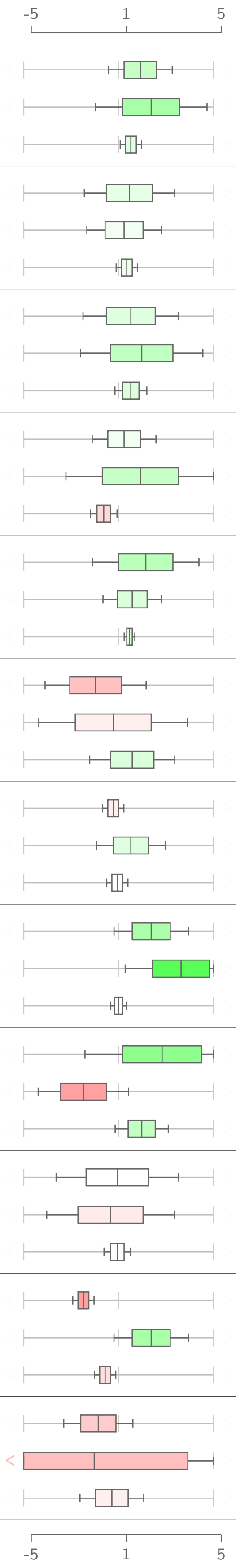

#	protein	group	peptides	spectra	ch1	ch2	quant	ratio
**896**	MAP7D3 Q8IWC1$_{1-4}$: MAP7 domain-containing protein 3	4/4	2	2	127	126	1	**1.10**
					129	128	1	**0.24**
					131	130	1	**0.89**
**897**	MAPK10 P53779$_{1-4}$, MAPK8 P45983$_{1-5}$, MAPK9 P45984$_{1-5}$: Mitogen-activated protein kinase 10, Mitogen-activated protein kinase 8, ...	14/14	1	1	127	126	1	**0.96**
					129	128	1	**0.73**
					131	130	1	**1.05**
**898**	MAPRE1 Q15691: Microtubule-associated protein RP/EB family member 1		3	3	127	126	3	**2.04**
					129	128	3	**0.92**
					131	130	3	**1.22**
**899**	MARCO Q9UEW3$_{1,2}$: Macrophage receptor MARCO	2/2	6	8	127	126	8	**0.73**
					129	128	8	**0.63**
					131	130	7	**1.05**
**900**	MASP1 P48740$_{1}$: Mannan-binding lectin serine protease 1	1/2	14	34	127	126	6	**1.15**
					129	128	6	**0.72**
					131	130	5	**1.07**
**901**	MASP1 P48740$_{2,4}$: Mannan-binding lectin serine protease 1	2/2	13	32	127	126	4	**1.21**
					129	128	4	**0.64**
					131	130	3	**1.07**
**902**	MASP2 O00187$_{1}$: Mannan-binding lectin serine protease 2		9	14	127	126	3	**1.04**
					129	128	3	**0.74**
					131	130	3	**0.97**
**903**	MASP2 O00187$_{2}$: Mannan-binding lectin serine protease 2		7	12	127	126	1	**1.57**
					129	128	1	**0.95**
					131	130	1	**0.85**
**904**	MAT1A Q00266, MAT2A P31153$_{1,2}$: S-adenosylmethionine synthase isoform type-1, S-adenosylmethionine synthase isoform type-2	3/3	2	2	127	126	2	**1.75**
					129	128	2	**0.67**
					131	130	2	**1.77**
**905**	MATN2 O00339$_{2}$: Matrilin-2	1/4	12	13	127	126	11	**0.67**
					129	128	12	**1.39**
					131	130	12	**0.99**
**906**	MATR3 P43243$_{1}$: Matrin-3	1/2	11	11	127	126	9	**1.94**
					129	128	11	**0.84**
					131	130	10	**1.38**
**907**	MB P02144: Myoglobin		7	8	127	126	8	**1.40**
					129	128	7	**1.99**
					131	130	8	**0.92**

#	protein	group	peptides	spectra	ch1	ch2	quant	ratio
**908**	MBL2 P11226: Mannose-binding protein C		1	2	127	126	2	**0.84**
					129	128	2	**0.53**
					131	130	2	**0.91**
**909**	MDK P21741$_{1}$: Midkine		1	1	127	126	1	**1.96**
					129	128	1	**4.97**
					131	130	1	**1.94**
**910**	MDN1 Q9NU22: Midasin		8	16	127	126	12	**1.33**
					129	128	11	**1.09**
					131	130	8	**1.04**
**911**	MEGF6 O75095$_{1}$: Multiple epidermal growth factor-like domains protein 6	1/2	12	18	127	126	16	**1.43**
					129	128	15	**1.52**
					131	130	16	**1.09**
**912**	METRN Q9UJH8: Meteorin		3	3	127	126	2	**0.66**
					129	128	2	**1.40**
					131	130	2	**1.08**
**913**	METRNL Q641Q3$_{1}$: Meteorin-like protein	1/2	4	4	127	126	3	**0.96**
					129	128	3	**1.29**
					131	130	3	**1.11**
**914**	MFAP2 P55001$_{1-3}$: Microfibrillar-associated protein 2	3/3	2	3	127	126	3	**1.19**
					129	128	3	**1.24**
					131	130	3	**0.91**
**915**	MFAP4 P55083$_{1,2}$: Microfibril-associated glycoprotein 4	2/2	7	90	127	126	89	**1.62**
					129	128	79	**0.83**
					131	130	89	**1.26**
**916**	MFAP5 Q13361$_{1,2}$: Microfibrillar-associated protein 5	2/2	6	11	127	126	9	**1.11**
					129	128	9	**1.14**
					131	130	9	**1.09**
**917**	MFGE8 Q08431$_{1-3}$: Lactadherin	3/3	2	2	127	126	2	**0.99**
					129	128	2	**0.75**
					131	130	2	**1.01**
**918**	MGAT1 P26572: Alpha-1,3-mannosyl-glycoprotein 2-beta-N-acetylglucosaminyltransferase		15	20	127	126	19	**0.52**
					129	128	20	**1.99**
					131	130	19	**0.78**
**919**	MGAT4B Q9UQ53$_{1-3}$: Alpha-1,3-mannosyl-glycoprotein 4-beta-N-acetylglucosaminyltransferase B	3/3	5	7	127	126	4	**0.85**
					129	128	5	**1.15**
					131	130	3	**0.87**

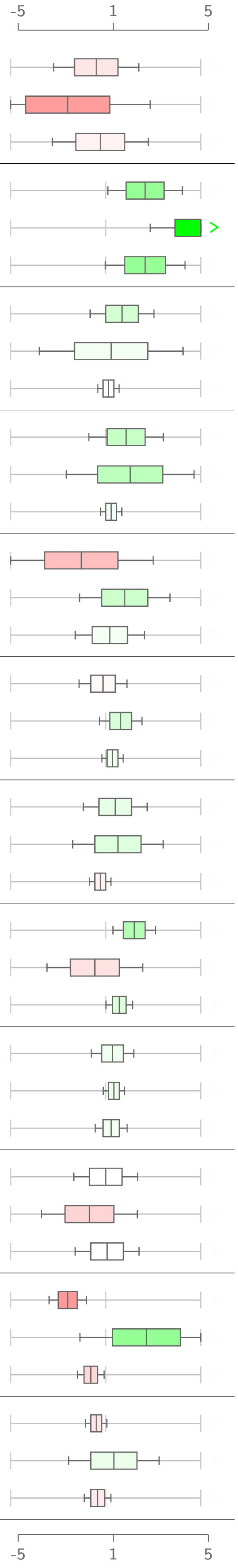

#	protein	group	peptides	spectra	ch1	ch2	quant	ratio
920	MGAT5 Q09328: Alpha-1,6-mannosylglycoprotein 6-beta-N-acetylglucosaminyltransferase A		6	6	127	126	6	**0.69**
					129	128	6	**2.06**
					131	130	5	**1.07**
921	MGP P08493$_{1,2}$: Matrix Gla protein	2/2	5	175	127	126	162	**1.85**
					129	128	159	**1.49**
					131	130	157	**1.46**
922	MIA3 Q5JRA6$_{1-3}$: Melanoma inhibitory activity protein 3	3/3	5	6	127	126	4	**1.32**
					129	128	5	**1.49**
					131	130	4	**1.13**
923	MINPP1 Q9UNW1$_1$: Multiple inositol polyphosphate phosphatase 1	1/4	8	9	127	126	7	**0.52**
					129	128	7	**1.55**
					131	130	7	**0.98**
924	MLEC Q14165: Malectin		2	2	127	126	2	**1.32**
					129	128	2	**1.21**
					131	130	2	**1.24**
925	MMP14 P50281: Matrix metalloproteinase-14		4	5	127	126	5	**1.83**
					129	128	4	**1.48**
					131	130	4	**0.83**
926	MMP19 Q99542$_1$: Matrix metalloproteinase-19	1/2	4	4	127	126	3	**0.84**
					129	128	4	**1.35**
					131	130	4	**1.17**
927	MMP2 P08253$_1$: 72 kDa type IV collagenase	1/3	24	35	127	126	32	**1.01**
					129	128	31	**1.37**
					131	130	33	**1.04**
928	MMP28 Q9H239$_1$: Matrix metalloproteinase-28		2	2	127	126	2	**1.87**
					129	128	2	**1.79**
					131	130	2	**1.02**
929	MMRN1 Q13201$_1$: Multimerin-1	1/2	5	5	127	126	5	**0.83**
					129	128	5	**2.33**
					131	130	5	**0.87**
930	MMRN2 Q9H8L6: Multimerin-2		3	3	127	126	3	**1.27**
					129	128	3	**0.99**
					131	130	3	**1.08**
931	MPO P05164$_{1-3}$: Myeloperoxidase	3/3	6	7	127	126	5	**1.43**
					129	128	6	**1.92**
					131	130	7	**1.05**

#	protein	group	peptides	spectra	ch1	ch2	quant	ratio
**932**	MPST P25325$_{1,2}$: 3-mercaptopyruvate sulfurtransferase	2/2	3	3	127	126	3	**1.31**
					129	128	3	**0.99**
					131	130	3	**1.19**
**933**	MRC1 P22897$_{1}$: Macrophage mannose receptor 1	1/2	3	4	127	126	3	**1.30**
					129	128	3	**1.84**
					131	130	3	**1.08**
**934**	MRC2 Q9UBG0: C-type mannose receptor 2		3	5	127	126	5	**1.13**
					129	128	4	**1.62**
					131	130	5	**1.00**
**935**	MSLN Q13421$_{2-4}$: Mesothelin	3/4	19	39	127	126	38	**1.20**
					129	128	38	**1.53**
					131	130	38	**0.94**
**936**	MSN P26038: Moesin		16	19	127	126	10	**0.68**
					129	128	11	**3.41**
					131	130	10	**1.28**
**937**	MSR1 P21757$_{1,3}$: Macrophage scavenger receptor types I and II	2/2	5	10	127	126	5	**1.30**
					129	128	5	**0.86**
					131	130	6	**1.00**
**938**	MST1 P26927: Hepatocyte growth factor-like protein	1/3	5	5	127	126	5	**1.14**
					129	128	5	**0.91**
					131	130	4	**0.90**
**939**	MT1A P04731: Metallothionein-1A		5	18	127	126	6	**0.45**
					129	128	4	**0.73**
					131	130	7	**0.90**
**940**	MTHFD1 P11586: C-1-tetrahydrofolate synthase, cytoplasmic		5	7	127	126	7	**0.96**
					129	128	7	**1.01**
					131	130	7	**0.95**
**941**	MVP Q14764: Major vault protein		7	8	127	126	7	**1.26**
					129	128	8	**1.02**
					131	130	8	**1.17**
**942**	MXRA5 Q9NR99: Matrix-remodeling-associated protein 5		47	70	127	126	62	**0.75**
					129	128	62	**1.72**
					131	130	65	**0.91**
**943**	MXRA7 P84157$_{1}$: Matrix-remodeling-associated protein 7	1/2	3	3	127	126		
					129	128		
					131	130	1	**0.91**

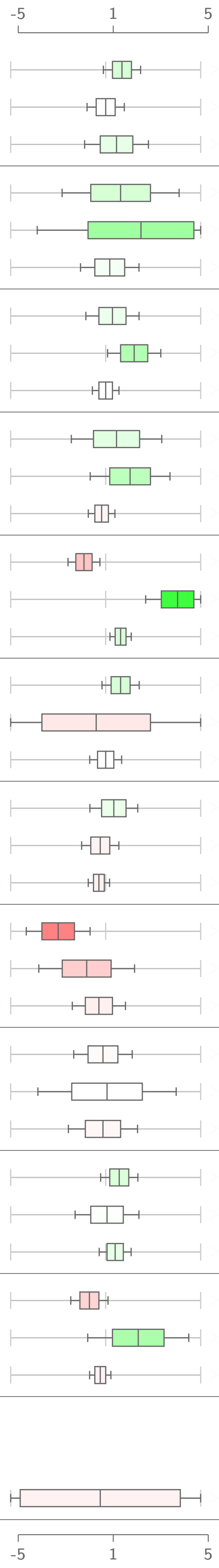

#	protein	group	peptides	spectra	ch1	ch2	quant	ratio
**944**	MXRA7 P84157$_2$: Matrix-remodeling-associated protein 7		3	3	127	126	1	**3.07**
					129	128	1	**2.38**
					131	130	1	**1.20**
**945**	MYCBP2 O75592$_{1,2}$: E3 ubiquitin-protein ligase MYCBP2	2/2	3	16	127	126	16	**0.60**
					129	128	6	**1.57**
					131	130	16	**0.90**
**946**	MYH10 P35580$_{1-5}$: Myosin-10	5/5	44	50	127	126	30	**1.03**
					129	128	28	**2.37**
					131	130	29	**1.27**
**947**	MYH11 P35749$_{1-4}$: Myosin-11	4/4	20	26	127	126	4	**0.85**
					129	128	3	**2.35**
					131	130	3	**1.19**
**948**	MYH14 Q7Z406$_{1,2,4,6}$: Myosin-14	4/5	9	10	127	126	1	**0.51**
					129	128	1	**1.24**
					131	130	1	**1.04**
**949**	MYH9 P35579$_1$: Myosin-9	1/2	77	118	127	126	85	**0.91**
					129	128	85	**3.12**
					131	130	88	**1.14**
**950**	MYL1 P05976$_1$: Myosin light chain 1/3, skeletal muscle isoform	1/2	6	8	127	126	7	**1.35**
					129	128	6	**1.64**
					131	130	7	**0.93**
**951**	MYL12A P19105, MYL12B O14950: Myosin regulatory light chain 12A, Myosin regulatory light chain 12B	2/2	7	12	127	126	4	**2.07**
					129	128	5	**0.90**
					131	130	4	**1.32**
**952**	MYL2 P10916: Myosin regulatory light chain 2, ventricular/cardiac muscle isoform	1/3	9	19	127	126	18	**1.45**
					129	128	17	**1.50**
					131	130	17	**0.98**
**953**	MYL3 P08590: Myosin light chain 3		7	7	127	126	6	**1.42**
					129	128	5	**1.77**
					131	130	5	**0.96**
**954**	MYL6 P60660$_{1,2}$: Myosin light polypeptide 6	2/3	8	14	127	126	11	**1.89**
					129	128	11	**1.01**
					131	130	12	**1.40**
**955**	MYL9 P24844$_{1,2}$: Myosin regulatory light polypeptide 9	2/2	5	10	127	126	3	**1.91**
					129	128	3	**1.48**
					131	130	3	**1.56**

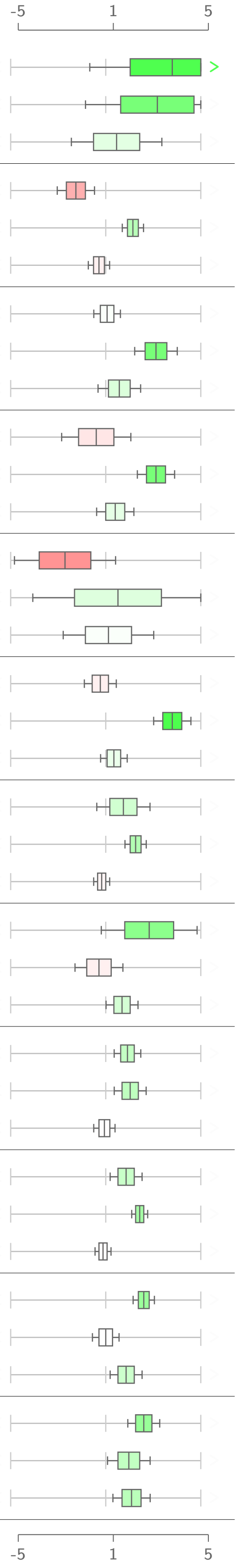

#	protein	group	peptides	spectra	ch1	ch2	quant	ratio
**956**	MYLK Q15746$_{1-7,9,11}$: Myosin light chain kinase, smooth muscle	9/11	3	7	127	126	6	**1.75**
					129	128	4	**0.99**
					131	130	7	**1.55**
**957**	MYLPF Q96A32: Myosin regulatory light chain 2, skeletal muscle isoform		7	8	127	126	8	**1.83**
					129	128	7	**1.43**
					131	130	8	**0.97**
**958**	MYO1C O00159$_{1-3}$: Unconventional myosin-Ic	3/3	2	2	127	126	2	**1.27**
					129	128	2	**1.52**
					131	130	2	**1.13**
**959**	MYO1H Q8N1T3$_{1,3}$: Unconventional myosin-Ih	2/2	2	3	127	126	2	**1.01**
					129	128	2	**0.42**
					131	130	2	**1.24**
**960**	MYOC Q99972: Myocilin		16	20	127	126	20	**0.93**
					129	128	18	**1.40**
					131	130	20	**1.12**
**961**	NAGLU P54802: Alpha-N-acetylglucosaminidase		1	1	127	126	1	**0.84**
					129	128		
					131	130	1	**1.16**
**962**	NAMPT P43490: Nicotinamide phosphoribosyltransferase		1	1	127	126	1	**0.76**
					129	128	1	**1.32**
					131	130	1	**0.74**
**963**	NAP1L1 P55209$_{1-3}$, NAP1L4 Q99733$_{1,2}$: Nucleosome assembly protein 1-like 1, Nucleosome assembly protein 1-like 4	5/5	1	1	127	126	1	**1.88**
					129	128	1	**0.67**
					131	130	1	**1.21**
**964**	NAPA P54920: Alpha-soluble NSF attachment protein	1/4	5	5	127	126	4	**1.43**
					129	128	4	**1.05**
					131	130	5	**1.07**
**965**	NARS O43776$_{1,2}$: Asparagine--tRNA ligase, cytoplasmic	2/2	2	2	127	126	2	**2.33**
					129	128	2	**0.89**
					131	130	2	**1.21**
**966**	NBL1 P41271$_{1,2}$: Neuroblastoma suppressor of tumorigenicity 1	2/2	3	8	127	126	8	**2.52**
					129	128	7	**1.34**
					131	130	7	**1.06**
**967**	NCAM1 P13591$_{1-6}$: Neural cell adhesion molecule 1	6/6	1	1	127	126	1	**1.27**
					129	128	1	**0.90**
					131	130	1	**1.10**

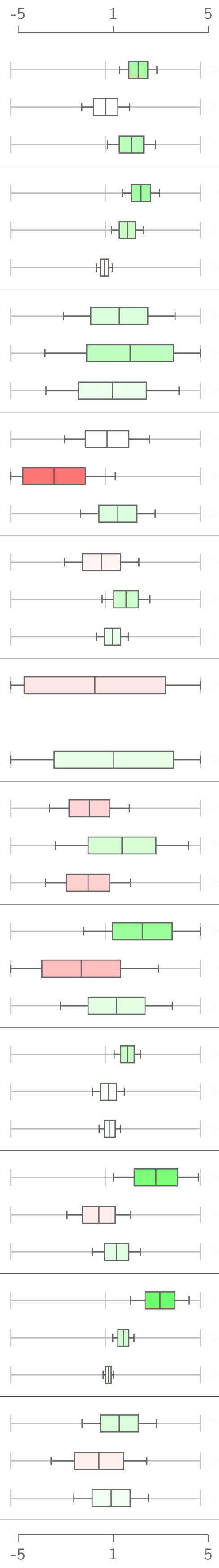

#	protein	group	peptides	spectra	ch1	ch2	quant	ratio
968	NCF1 P14598$_1$, NCF1B A6NI72, NCF1C A8MVU1: Neutrophil cytosol factor 1, Putative neutrophil cytosol factor 1B, . . .	3/3	2	2	127	126	2	**0.50**
					129	128	2	**1.55**
					131	130	2	**0.98**
969	NCL P19338: Nucleolin		19	24	127	126	24	**1.89**
					129	128	24	**0.88**
					131	130	22	**1.41**
970	NDRG1 Q92597$_1$: Protein NDRG1	1/3	3	4	127	126	4	**1.49**
					129	128	4	**1.11**
					131	130	4	**1.29**
971	NECAP2 Q9NVZ3$_{1,2,4}$: Adaptin ear-binding coat-associated protein 2	3/4	5	7	127	126	6	**0.80**
					129	128	6	**2.24**
					131	130	6	**0.96**
972	NECTIN2 Q92692$_2$: Nectin-2	1/2	5	6	127	126	5	**0.57**
					129	128	5	**2.64**
					131	130	5	**1.02**
974	NEFL P07196: Neurofilament light polypeptide		4	7	127	126	3	**0.30**
					129	128	3	**3.78**
					131	130	3	**0.71**
975	NES P48681: Nestin		4	6	127	126	4	**1.00**
					129	128	3	**1.67**
					131	130	3	**0.99**
976	NID1 P14543$_1$: Nidogen-1	1/2	23	36	127	126	33	**1.28**
					129	128	34	**1.00**
					131	130	34	**1.18**
977	NID2 Q14112$_1$: Nidogen-2	1/2	26	32	127	126	26	**1.20**
					129	128	23	**1.20**
					131	130	28	**1.06**
978	NNMT P40261: Nicotinamide N-methyltransferase		3	3	127	126	3	**0.95**
					129	128	2	**1.37**
					131	130	3	**1.17**
979	NOMO1 Q15155, NOMO2 Q5JPE7$_{1-3}$, NOMO3 P69849: Nodal modulator 1, Nodal modulator 2, . . .	5/5	2	2	127	126	2	**1.17**
					129	128	2	**1.28**
					131	130	2	**1.76**
980	NONO Q15233$_1$: Non-POU domain-containing octamer-binding protein	1/4	7	8	127	126	6	**1.87**
					129	128	5	**0.56**
					131	130	6	**1.40**

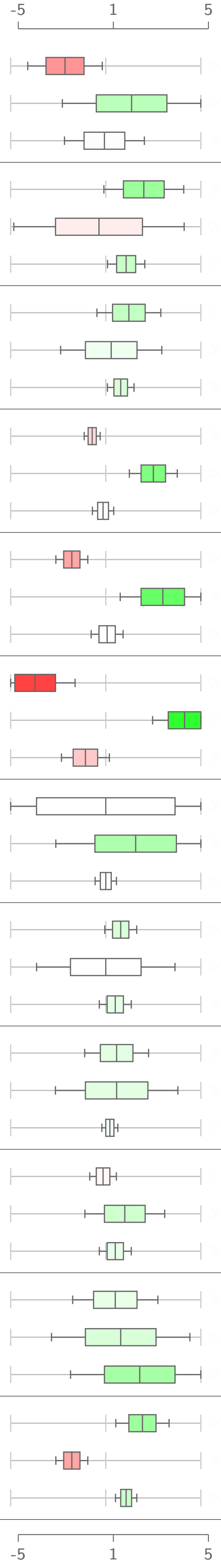

#	protein	group	peptides	spectra	ch1	ch2	quant	ratio
981	NOTCH3 Q9UM47: Neurogenic locus notch homolog protein 3		10	14	127	126	13	**0.95**
					129	128	11	**1.27**
					131	130	11	**1.13**
982	NPC2 P61916$_{1,2}$: Epididymal secretory protein E1	2/2	3	4	127	126	3	**1.23**
					129	128	4	**2.02**
					131	130	3	**1.11**
983	NPM1 P06748$_{1}$: Nucleophosmin	1/3	10	17	127	126	16	**2.53**
					129	128	16	**0.54**
					131	130	16	**1.87**
984	NPPA P01160: Natriuretic peptides A		3	4	127	126	4	**2.15**
					129	128	4	**1.44**
					131	130	4	**1.04**
985	NQO1 P15559$_{1-3}$: NAD(P)H dehydrogenase [quinone] 1	3/3	6	8	127	126	7	**2.16**
					129	128	8	**0.56**
					131	130	7	**1.19**
986	NRP2 O60462$_{1-3}$: Neuropilin-2	3/6	13	15	127	126	13	**1.42**
					129	128	15	**1.00**
					131	130	14	**1.09**
987	NSFL1C Q9UNZ2$_{1,4,5}$: NSFL1 cofactor p47	3/3	2	3	127	126	3	**1.77**
					129	128	3	**0.76**
					131	130	3	**1.19**
988	NT5C2 P49902$_{1}$: Cytosolic purine 5'-nucleotidase	1/2	3	8	127	126	8	**0.76**
					129	128	8	**1.59**
					131	130	7	**0.88**
989	NUCB1 Q02818: Nucleobindin-1		31	96	127	126	90	**1.71**
					129	128	87	**1.43**
					131	130	91	**1.25**
990	NUCB2 P80303$_{1,2}$: Nucleobindin-2	2/2	16	24	127	126	20	**1.96**
					129	128	18	**0.90**
					131	130	18	**1.03**
991	NUCKS1 Q9H1E3$_{1}$: Nuclear ubiquitous casein and cyclin-dependent kinase substrate 1		7	9	127	126	3	**1.60**
					129	128	3	**1.13**
					131	130	3	**0.88**
992	NUCKS1 Q9H1E3$_{2}$: Nuclear ubiquitous casein and cyclin-dependent kinase substrate 1		4	6	127	126	1	**0.89**
					129	128	1	**0.50**
					131	130	1	**0.99**

#	protein	group	peptides	spectra	ch1	ch2	quant	ratio
993	NUMA1 Q14980$_{1-4}$: Nuclear mitotic apparatus protein 1	4/5	10	101	127	126	6	1.44
					129	128	7	1.28
					131	130	6	1.08
994	NUP54 Q7Z3B4$_{1,3}$: Nucleoporin p54	2/2	3	3	127	126	2	1.81
					129	128	2	0.88
					131	130	2	1.16
995	NXN Q6DKJ4$_{1,3}$: Nucleoredoxin	2/2	1	2	127	126	2	1.22
					129	128	2	0.67
					131	130	2	1.24
996	OAF Q86UD1: Out at first protein homolog		8	23	127	126	20	0.99
					129	128	22	0.78
					131	130	23	1.15
997	OGN P20774: Mimecan		18	130	127	126	122	0.88
					129	128	101	1.43
					131	130	114	0.95
998	OIT3 Q8WWZ8$_{1}$: Oncoprotein-induced transcript 3 protein		2	2	127	126	2	1.21
					129	128	2	0.88
					131	130	2	1.01
999	OLFML1 Q6UWY5: Olfactomedin-like protein 1		14	24	127	126	22	1.67
					129	128	21	0.64
					131	130	23	1.20
1000	OLFML3 Q9NRN5$_{1}$: Olfactomedin-like protein 3	1/3	19	59	127	126	51	1.28
					129	128	53	1.02
					131	130	56	1.05
1001	OMD Q99983: Osteomodulin		4	5	127	126	4	1.36
					129	128	5	1.00
					131	130	4	1.02
1003	ORM1 P02763: Alpha-1-acid glycoprotein 1		9	13	127	126	6	0.96
					129	128	4	0.86
					131	130	6	0.92
1004	ORM2 P19652: Alpha-1-acid glycoprotein 2		9	12	127	126	3	0.61
					129	128	5	0.75
					131	130	5	0.91
1005	OXSR1 O95747: Serine/threonine-protein kinase OSR1		2	2	127	126	2	1.48
					129	128	2	1.18
					131	130	2	1.12

#	protein	group	peptides	spectra	ch1	ch2	quant	ratio
**1006**	P04220: Ig mu heavy chain disease protein		12	36	127	126	5	**0.92**
					129	128	4	**0.45**
					131	130	5	**1.17**
**1007**	P3H1 Q32P28$_{1,3,4}$: Prolyl 3-hydroxylase 1	3/3	3	3	127	126	3	**1.37**
					129	128	3	**1.21**
					131	130	3	**1.17**
**1008**	P4HA1 P13674$_{1-3}$: Prolyl 4-hydroxylase subunit alpha-1	3/3	2	2	127	126	2	**1.54**
					129	128	2	**1.49**
					131	130	2	**1.08**
**1009**	P4HB P07237: Protein disulfide-isomerase		27	38	127	126	33	**1.71**
					129	128	35	**0.67**
					131	130	32	**1.14**
**1010**	PABPC1 P11940$_{1}$: Polyadenylate-binding protein 1	1/6	6	10	127	126	9	**1.33**
					129	128	10	**0.70**
					131	130	9	**1.14**
**1011**	PACSIN2 Q9UNF0$_{1}$: Protein kinase C and casein kinase substrate in neurons protein 2	1/2	5	22	127	126	8	**1.62**
					129	128	4	**0.81**
					131	130	7	**1.24**
**1012**	PAFAH1B3 Q15102: Platelet-activating factor acetylhydrolase IB subunit gamma		1	1	127	126	1	**0.37**
					129	128	1	**0.94**
					131	130	1	**0.88**
**1013**	PAK2 Q13177: Serine/threonine-protein kinase PAK 2		2	2	127	126	2	**0.67**
					129	128	2	**1.10**
					131	130	2	**0.98**
**1014**	PAM P19021$_{1,3,5}$: Peptidyl-glycine alpha-amidating monooxygenase	3/6	24	32	127	126	29	**1.16**
					129	128	27	**1.19**
					131	130	27	**1.10**
**1016**	PAMR1 Q6UXH9$_{2}$: Inactive serine protease PAMR1		16	22	127	126	2	**1.68**
					129	128	2	**0.92**
					131	130	2	**1.00**
**1018**	PAPLN O95428$_{1,5,6}$: Papilin	3/6	26	41	127	126	41	**1.34**
					129	128	36	**1.44**
					131	130	40	**1.12**
**1019**	PAPSS2 O95340$_{1,2}$: Bifunctional 3'-phosphoadenosine 5'-phosphosulfate synthase 2	2/2	2	2	127	126	2	**1.50**
					129	128	2	**1.48**
					131	130	2	**1.16**

#	protein	group	peptides	spectra	ch1	ch2	quant	ratio
**1020**	PARVA Q9NVD7$_1$: Alpha-parvin	1/5	5	5	127	126	5	**1.21**
					129	128	5	**0.84**
					131	130	3	**1.03**
**1021**	PBXIP1 Q96AQ6$_{1,2}$: Pre-B-cell leukemia transcription factor-interacting protein 1	2/3	4	5	127	126	5	**1.47**
					129	128	4	**1.43**
					131	130	3	**1.16**
**1022**	PCBP1 Q15365: Poly(rC)-binding protein 1	1/6	8	11	127	126	5	**1.73**
					129	128	5	**0.89**
					131	130	4	**1.27**
**1023**	PCBP2 Q15366$_{1-8}$: Poly(rC)-binding protein 2	8/8	6	9	127	126	3	**1.67**
					129	128	3	**0.75**
					131	130	3	**1.23**
**1024**	PCMT1 P22061$_{1,2}$: Protein-L-isoaspartate(D-aspartate) O-methyltransferase	2/2	8	8	127	126	8	**0.74**
					129	128	7	**1.62**
					131	130	7	**0.96**
**1025**	PCOLCE Q15113: Procollagen C-endopeptidase enhancer 1		23	125	127	126	122	**1.47**
					129	128	117	**1.13**
					131	130	119	**0.96**
**1026**	PCOLCE2 Q9UKZ9: Procollagen C-endopeptidase enhancer 2		10	15	127	126	15	**1.02**
					129	128	14	**0.59**
					131	130	15	**0.93**
**1027**	PCSK1N Q9UHG2: ProSAAS		5	11	127	126	9	**2.24**
					129	128	11	**1.94**
					131	130	11	**0.96**
**1028**	PCSK5 Q92824$_1$: Proprotein convertase subtilisin/kexin type 5		25	120	127	126	55	**0.88**
					129	128	37	**0.94**
					131	130	56	**1.09**
**1029**	PCSK5 Q92824$_2$: Proprotein convertase subtilisin/kexin type 5		17	21	127	126	1	**1.42**
					129	128	1	**1.29**
					131	130	1	**1.51**
**1030**	PCSK7 Q16549: Proprotein convertase subtilisin/kexin type 7		1	1	127	126	1	**1.23**
					129	128	1	**0.92**
					131	130	1	**0.97**
**1031**	PCSK9 Q8NBP7$_1$: Proprotein convertase subtilisin/kexin type 9	1/2	11	18	127	126	16	**0.91**
					129	128	17	**1.18**
					131	130	17	**1.22**

#	protein	group	peptides	spectra	ch1	ch2	quant	ratio
**1032**	PCYOX1 Q9UHG3$_1$: Prenylcysteine oxidase 1	1/2	10	14	127	126	14	**0.71**
					129	128	14	**0.57**
					131	130	14	**0.90**
**1033**	PDCD4 Q53EL6$_{1,2}$: Programmed cell death protein 4	2/2	3	3	127	126	3	**1.17**
					129	128	3	**1.09**
					131	130	3	**1.02**
**1034**	PDCD6IP Q8WUM4$_{1,2}$: Programmed cell death 6-interacting protein	2/3	14	16	127	126	14	**0.93**
					129	128	14	**1.91**
					131	130	14	**1.05**
**1035**	PDGFB P01127$_{1,2}$: Platelet-derived growth factor subunit B	2/2	2	3	127	126	3	**2.16**
					129	128	3	**1.81**
					131	130	3	**1.22**
**1036**	PDGFC Q9NRA1$_1$: Platelet-derived growth factor C	1/4	9	13	127	126	9	**1.20**
					129	128	9	**1.17**
					131	130	7	**0.83**
**1037**	PDGFD Q9GZP0$_{1,2}$: Platelet-derived growth factor D	2/2	5	5	127	126	5	**1.24**
					129	128	5	**1.18**
					131	130	5	**1.21**
**1038**	PDGFRL Q15198: Platelet-derived growth factor receptor-like protein		8	8	127	126	7	**1.09**
					129	128	6	**0.69**
					131	130	8	**1.02**
**1039**	PDIA3 P30101: Protein disulfide-isomerase A3		9	9	127	126	8	**1.37**
					129	128	8	**0.75**
					131	130	9	**1.47**
**1040**	PDIA4 P13667: Protein disulfide-isomerase A4		13	17	127	126	15	**1.85**
					129	128	16	**0.74**
					131	130	16	**1.31**
**1041**	PDIA6 Q15084$_5$: Protein disulfide-isomerase A6	1/5	13	15	127	126	15	**1.17**
					129	128	15	**1.16**
					131	130	12	**1.05**
**1042**	PDLIM5 Q96HC4$_1$: PDZ and LIM domain protein 5	1/6	7	7	127	126	7	**1.84**
					129	128	7	**0.85**
					131	130	6	**1.19**
**1043**	PDLIM7 Q9NR12$_{1,2}$: PDZ and LIM domain protein 7	2/2	2	2	127	126	2	**1.49**
					129	128	2	**1.68**
					131	130	2	**1.08**

#	protein	group	peptides	spectra	ch1	ch2	quant	ratio
**1044**	PEA15 Q15121$_{1,2}$: Astrocytic phosphoprotein PEA-15	2/2	4	4	127	126	4	**1.83**
					129	128	4	**1.05**
					131	130	4	**1.17**
**1045**	PEBP4 Q96S96: Phosphatidylethanolamine-binding protein 4		1	1	127	126	1	**1.36**
					129	128	1	**1.30**
					131	130	1	**1.12**
**1046**	PENK P01210: Proenkephalin-A [Cleaved into: Synenkephalin; Met-enkephalin		8	14	127	126	13	**1.79**
					129	128	14	**1.14**
					131	130	12	**1.13**
**1047**	PFKL P17858$_{1,2}$: ATP-dependent 6-phosphofructokinase, liver type	2/3	4	4	127	126	2	**1.16**
					129	128	2	**1.25**
					131	130	2	**0.92**
**1048**	PFKM P08237$_{1-3}$: ATP-dependent 6-phosphofructokinase, muscle type	3/3	2	2	127	126	2	**1.35**
					129	128	2	**0.81**
					131	130	2	**1.02**
**1049**	PFKP Q01813$_{2}$: ATP-dependent 6-phosphofructokinase, platelet type		3	3	127	126	1	**0.80**
					129	128		
					131	130		
**1050**	PFN1 P07737: Profilin-1		7	13	127	126	13	**0.50**
					129	128	13	**2.58**
					131	130	11	**0.87**
**1051**	PGD P52209$_{1,2}$: 6-phosphogluconate dehydrogenase, decarboxylating	2/2	9	10	127	126	10	**0.60**
					129	128	10	**2.02**
					131	130	9	**0.91**
**1052**	PGK1 P00558$_{1,2}$: Phosphoglycerate kinase 1	2/2	3	3	127	126	1	**0.46**
					129	128	1	**2.77**
					131	130	1	**0.81**
**1053**	PGK2 P07205: Phosphoglycerate kinase 2		3	5	127	126	3	**0.80**
					129	128	3	**2.03**
					131	130	3	**0.99**
**1054**	PGLS O95336: 6-phosphogluconolactonase		2	2	127	126	2	**0.57**
					129	128	2	**1.98**
					131	130	2	**0.95**
**1055**	PGLYRP2 Q96PD5$_{1,2}$: N-acetylmuramoyl-L-alanine amidase	2/2	9	37	127	126	36	**0.49**
					129	128	37	**1.92**
					131	130	36	**0.97**

#	protein	group	peptides	spectra	ch1	ch2	quant	ratio
1056	PGM1 P36871$_{1}$: Phosphoglucomutase-1	1/3	5	5	127	126	5	**0.65**
					129	128	3	**1.80**
					131	130	4	**0.68**
1057	PGM2 Q96G03$_{1}$: Phosphoglucomutase-2		3	3	127	126	3	**0.42**
					129	128	3	**2.46**
					131	130	3	**1.15**
1058	PGM3 O95394$_{1,3,4}$: Phosphoacetylglucosamine mutase	3/3	2	2	127	126	2	**0.76**
					129	128	2	**1.56**
					131	130	2	**1.02**
1059	PGRMC1 O00264$_{1,2}$: Membrane-associated progesterone receptor component 1	2/2	4	4	127	126	4	**1.51**
					129	128	4	**0.81**
					131	130	4	**1.29**
1060	PHB2 Q99623$_{1}$: Prohibitin-2	1/2	3	3	127	126	3	**1.89**
					129	128	3	**0.63**
					131	130	3	**1.57**
1061	PHGDH O43175: D-3-phosphoglycerate dehydrogenase		13	22	127	126	17	**1.75**
					129	128	22	**0.62**
					131	130	22	**1.17**
1062	PHPT1 Q9NRX4$_{1,2}$: 14 kDa phosphohistidine phosphatase	2/2	3	3	127	126	3	**1.90**
					129	128	3	**1.35**
					131	130	3	**1.07**
1063	PICALM Q13492$_{1-5}$: Phosphatidylinositol-binding clathrin assembly protein	5/5	3	3	127	126	3	**1.06**
					129	128	3	**1.74**
					131	130	3	**1.00**
1064	PIGR P01833: Polymeric immunoglobulin receptor		13	15	127	126	14	**0.83**
					129	128	15	**1.13**
					131	130	15	**0.74**
1065	PIP P12273: Prolactin-inducible protein		2	2	127	126	1	**0.66**
					129	128	1	**2.78**
					131	130	1	**0.14** <
1066	PITPNA Q00169: Phosphatidylinositol transfer protein alpha isoform		5	7	127	126	7	**0.51**
					129	128	7	**2.18**
					131	130	7	**0.90**
1067	PKDCC Q504Y2: Extracellular tyrosine-protein kinase PKDCC		5	5	127	126	4	**0.87**
					129	128	5	**1.88**
					131	130	4	**1.17**

#	protein	group	peptides	spectra	ch1	ch2	quant	ratio
**1068**	PKM P14618$_1$: Pyruvate kinase PKM	1/5	30	52	127	126	49	**1.31**
					129	128	47	**0.65**
					131	130	49	**0.96**
**1069**	PLA2G12A Q9BZM1: Group XIIA secretory phospholipase A2		4	15	127	126	11	**0.47**
					129	128	7	**0.90**
					131	130	11	**0.79**
**1070**	PLA2G2A P14555: Phospholipase A2, membrane associated		12	41	127	126	29	**1.37**
					129	128	33	**2.40**
					131	130	32	**2.10**
**1071**	PLA2G7 Q13093: Platelet-activating factor acetyl-hydrolase		2	3	127	126	3	**0.40**
					129	128	3	**1.39**
					131	130	3	**0.94**
**1072**	PLAT P00750$_1$: Tissue-type plasminogen activator	1/3	16	24	127	126	9	**1.15**
					129	128	9	**0.80**
					131	130	5	**1.67**
**1073**	PLAT P00750$_4$: Tissue-type plasminogen activator		11	16	127	126	1	**0.94**
					129	128		
					131	130	1	**1.09**
**1074**	PLEC Q15149$_5$: Plectin	1/9	142	172	127	126	155	**1.18**
					129	128	155	**1.40**
					131	130	156	**1.31**
**1075**	PLG P00747: Plasminogen	1/2	51	520	127	126	426	**0.90**
					129	128	420	**0.50**
					131	130	449	**0.94**
**1076**	PLGLB1 Q02325: Plasminogen-like protein B		5	42	127	126	2	**1.20**
					129	128	1	**0.51**
					131	130	2	**1.16**
**1077**	PLIN3 O60664$_{1,3}$: Perilipin-3	2/4	7	7	127	126	6	**1.00**
					129	128	6	**0.80**
					131	130	7	**1.03**
**1078**	PLIN4 Q96Q06$_{1,2}$: Perilipin-4	2/2	8	8	127	126	6	**0.84**
					129	128	6	**3.00**
					131	130	6	**0.98**
**1079**	PLOD1 Q02809$_{1,2}$: Procollagen-lysine,2-oxoglutarate 5-dioxygenase 1	2/2	8	11	127	126	10	**1.09**
					129	128	9	**2.64**
					131	130	9	**1.11**

#	protein	group	peptides	spectra	ch1	ch2	quant	ratio
**1080**	PLOD3 O60568: Procollagen-lysine,2-oxoglutarate 5-dioxygenase 3		6	7	127	126	6	**1.48**
					129	128	7	**1.36**
					131	130	7	**1.17**
**1081**	PLP2 $Q04941_1$: Proteolipid protein 2		1	1	127	126	1	**0.82**
					129	128	1	**2.24**
					131	130	1	**1.40**
**1082**	PLS3 $P13797_{1-3}$: Plastin-3	3/3	12	23	127	126	7	**0.46**
					129	128	8	**2.25**
					131	130	7	**1.05**
**1083**	PLTP $P55058_1$: Phospholipid transfer protein	1/4	20	378	127	126	350	**0.61**
					129	128	367	**1.98**
					131	130	349	**0.90**
**1084**	PLXDC2 $Q6UX71_{1,2}$: Plexin domain-containing protein 2	2/2	3	4	127	126	3	**0.59**
					129	128	3	**3.01**
					131	130	3	**1.00**
**1085**	PLXNB2 O15031: Plexin-B2		3	4	127	126	4	**0.86**
					129	128	4	**0.84**
					131	130	4	**1.20**
**1086**	PODN $Q7Z5L7_{1-3}$: Podocan	3/4	19	24	127	126	23	**1.55**
					129	128	23	**0.67**
					131	130	21	**1.03**
**1087**	POMGNT1 $Q8WZA1_{1,2}$: Protein O-linked-mannose beta-1,2-N-acetylglucosaminyltransferase 1	2/2	6	6	127	126	6	**0.41**
					129	128	5	**1.83**
					131	130	5	**0.83**
**1088**	PON1 P27169: Serum paraoxonase/arylesterase 1		13	105	127	126	78	**0.87**
					129	128	83	**0.78**
					131	130	91	**0.90**
**1089**	PON3 Q15166: Serum paraoxonase/lactonase 3		4	12	127	126	2	**0.61**
					129	128	2	**0.93**
					131	130	2	**0.90**
**1090**	POSTN $Q15063_1$: Periostin		50	208	127	126	3	**0.76**
					129	128	3	**2.33**
					131	130	3	**0.95**
**1097**	POTEA $Q6S8J7_{1,2}$: POTE ankyrin domain family member A	2/2	3	17	127	126	5	**0.96**
					129	128	5	**1.16**
					131	130	6	**0.92**

#	protein	group	peptides	spectra	ch1	ch2	quant	ratio
1098	POTEE Q6S8J3$_1$: POTE ankyrin domain family member E		15	177	127	126	25	**1.02**
					129	128	15	**0.92**
					131	130	24	**0.93**
1099	POTEF A5A3E0: POTE ankyrin domain family member F		13	139	127	126	5	**1.27**
					129	128	4	**1.07**
					131	130	5	**0.73**
1100	POTEH Q6S545$_{1,2}$: POTE ankyrin domain family member H	2/2	6	69	127	126	8	**0.87**
					129	128	7	**0.86**
					131	130	9	**1.03**
1101	POTEI P0CG38, POTEJ P0CG39: POTE ankyrin domain family member I, POTE ankyrin domain family member J	2/2	13	120	127	126	1	**1.00**
					129	128	1	**0.80**
					131	130	1	**1.02**
1102	POTEKP Q9BYX7: Putative beta-actin-like protein 3		6	47	127	126	1	**0.39**
					129	128	1	**0.45**
					131	130	1	**1.16**
1103	PPA1 Q15181: Inorganic pyrophosphatase		1	1	127	126	1	**0.94**
					129	128	1	**0.18**
					131	130	1	**1.24**
1104	PPBP P02775: Platelet basic protein		2	2	127	126	2	**1.62**
					129	128	2	**2.89**
					131	130	2	**1.16**
1105	PPIA P62937$_1$: Peptidyl-prolyl cis-trans isomerase A	1/8	7	8	127	126	8	**1.45**
					129	128	8	**1.61**
					131	130	7	**1.02**
1106	PPIB P23284: Peptidyl-prolyl cis-trans isomerase B		5	5	127	126	5	**1.20**
					129	128	5	**1.32**
					131	130	5	**1.53**
1107	PPL O60437: Periplakin		18	23	127	126	19	**1.57**
					129	128	20	**1.07**
					131	130	21	**1.38**
1108	PPP1CA P62136$_{1-3}$, PPP1CB P62140, PPP1CC P36873$_{1,2}$: Serine/threonine-protein phosphatase PP1-alpha catalytic subunit, Serine/threonine-protein phosphatase PP1-beta catalytic subunit, . . .	6/6	3	5	127	126	3	**1.29**
					129	128	5	**0.66**
					131	130	3	**1.14**
1109	PPP1R7 Q15435$_{1-5}$: Protein phosphatase 1 regulatory subunit 7	5/5	3	3	127	126	3	**1.42**
					129	128	3	**0.55**
					131	130	3	**1.10**

#	protein	group	peptides	spectra	ch1	ch2	quant	ratio
**1110**	PPP2CA P67775$_1$: Serine/threonine-protein phosphatase 2A catalytic subunit alpha isoform	1/3	3	3	127	126	3	**1.43**
					129	128	3	**0.80**
					131	130	3	**1.21**
**1111**	PPP2R1A P30153: Serine/threonine-protein phosphatase 2A 65 kDa regulatory subunit A alpha is ...		11	12	127	126	11	**1.40**
					129	128	11	**0.81**
					131	130	10	**1.14**
**1112**	PPP2R2A P63151$_{1,2}$: Serine/threonine-protein phosphatase 2A 55 kDa regulatory subunit B alpha is ...	2/2	3	4	127	126	4	**1.37**
					129	128	4	**0.82**
					131	130	4	**0.94**
**1113**	PPP3CA Q08209$_{1-3,5}$, PPP3CB P16298$_{1-4}$, PPP3CC P48454$_{1-3}$: Serine/threonine-protein phosphatase 2B catalytic subunit alpha isoform, Serine/threonine-protein phosphatase 2B catalytic subunit beta isoform, ...	11/11	1	1	127	126	1	**0.64**
					129	128	1	**3.31**
					131	130	1	**1.00**
**1114**	PPP4R3B Q5MIZ7$_{1-3,5}$: Serine/threonine-protein phosphatase 4 regulatory subunit 3B	4/4	2	53	127	126	8	**0.69**
					129	128	4	**0.87**
					131	130	9	**0.93**
**1115**	PRAP1 Q96NZ9$_1$: Proline-rich acidic protein 1	1/3	5	18	127	126	3	**1.43**
					129	128	3	**2.12**
					131	130	3	**0.89**
**1116**	PRAP1 Q96NZ9$_3$: Proline-rich acidic protein 1		5	18	127	126	3	**0.85**
					129	128	3	**1.15**
					131	130	3	**0.88**
**1117**	PRB3 Q04118: Basic salivary proline-rich protein 3		1	1	127	126	1	**0.68**
					129	128	1	**3.65**
					131	130	1	**0.10**
**1118**	PRDM15 P57071$_{1,3}$: PR domain zinc finger protein 15	2/3	6	11	127	126	10	**0.76**
					129	128	10	**1.65**
					131	130	10	**0.82**
**1119**	PRDX1 Q06830: Peroxiredoxin-1		4	5	127	126	5	**1.00**
					129	128	3	**1.55**
					131	130	5	**1.00**
**1120**	PRDX2 P32119$_1$: Peroxiredoxin-2	1/2	3	4	127	126	3	**0.97**
					129	128	4	**1.71**
					131	130	4	**1.10**
**1121**	PRDX5 P30044$_{1-4}$: Peroxiredoxin-5, mitochondrial	4/4	1	1	127	126	1	**1.39**
					129	128	1	**0.60**
					131	130	1	**1.16**

#	protein	group	peptides	spectra	ch1	ch2	quant	ratio
**1122**	PRDX6 P30041: Peroxiredoxin-6		11	25	127	126	25	**1.70**
					129	128	24	**0.75**
					131	130	20	**0.97**
**1123**	PRELP P51888: Prolargin		11	19	127	126	19	**0.92**
					129	128	18	**1.05**
					131	130	18	**1.03**
**1124**	PRG3 Q9Y2Y8: Proteoglycan 3		2	2	127	126	2	**0.93**
					129	128	2	**1.33**
					131	130	2	**0.98**
**1125**	PRG4 Q92954$_3$: Proteoglycan 4	1/2	49	354	127	126	4	**0.51**
					129	128	4	**14.53**
					131	130	4	**0.75**
**1126**	PRG4 Q92954$_6$: Proteoglycan 4	1/4	52	356	127	126	7	**0.58**
					129	128	9	**4.85**
					131	130	9	**0.82**
**1127**	PRKAR2A P13861$_1$: cAMP-dependent protein kinase type II-alpha regulatory subunit	1/2	6	6	127	126	6	**1.43**
					129	128	6	**0.84**
					131	130	4	**1.14**
**1128**	PRKCDBP Q969G5: Protein kinase C delta-binding protein		3	4	127	126	4	**2.28**
					129	128	4	**0.66**
					131	130	4	**1.25**
**1129**	PRKCSH P14314$_{1,2}$: Glucosidase 2 subunit beta	2/2	9	14	127	126	14	**2.06**
					129	128	12	**0.73**
					131	130	14	**1.23**
**1130**	PRKDC P78527$_{1,2}$: DNA-dependent protein kinase catalytic subunit	2/2	2	2	127	126	2	**0.97**
					129	128	2	**1.62**
					131	130	2	**1.13**
**1131**	PRL P01236: Prolactin		3	3	127	126	3	**0.49**
					129	128	3	**0.48**
					131	130	3	**0.90**
**1132**	PRNP P04156$_{1,2}$: Major prion protein	2/2	3	4	127	126	4	**1.68**
					129	128	3	**3.44**
					131	130	4	**1.16**
**1133**	PROC P04070$_{1,2}$: Vitamin K-dependent protein C	2/2	12	18	127	126	14	**1.55**
					129	128	16	**0.75**
					131	130	15	**0.96**

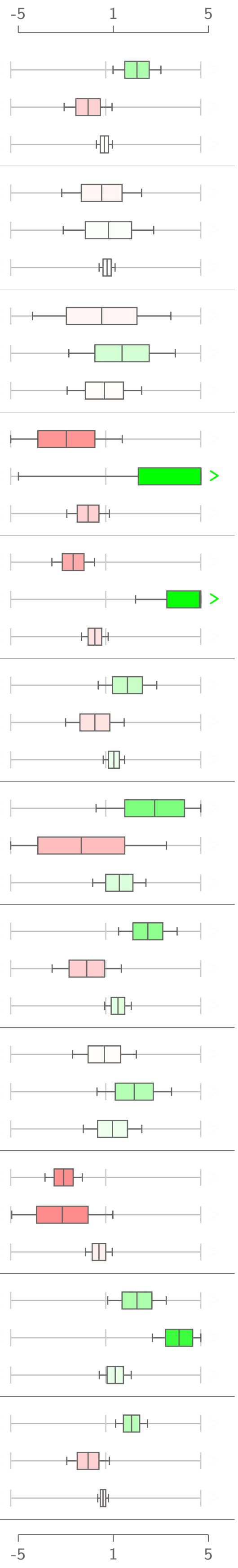

#	protein	group	peptides	spectra	ch1	ch2	quant	ratio
1134	PROS1 P07225: Vitamin K-dependent protein S		29	92	127	126	77	0.72
					129	128	81	0.62
					131	130	81	1.10
1136	PRPS1 P60891$_1$, PRPS2 P11908$_{1,2}$: Ribose-phosphate pyrophosphokinase 1, Ribose-phosphate pyrophosphokinase 2	3/3	3	3	127	126	3	1.26
					129	128	3	1.06
					131	130	3	1.03
1137	PRRC2B Q5JSZ5$_1$: Protein PRRC2B		5	42	127	126	40	1.48
					129	128	26	1.06
					131	130	40	1.04
1138	PRSS1 P07477: Trypsin-1		3	29	127	126	2	1.25
					129	128	2	0.72
					131	130	2	3.16
1139	PRSS2 P07478: Trypsin-2	1/6	6	38	127	126	9	1.14
					129	128	9	1.94
					131	130	8	1.98
1140	PRSS23 O95084$_1$: Serine protease 23		2	2	127	126	2	1.27
					129	128	2	1.95
					131	130	2	1.24
1141	PRSS3P2 Q8NHM4: Putative trypsin-6		3	30	127	126	1	1.32
					129	128	1	3.32
					131	130	1	1.65
1142	PSAP P07602$_{1-3}$: Prosaposin	3/3	20	57	127	126	55	1.75
					129	128	54	1.86
					131	130	54	1.00
1143	PSAPL1 Q6NUJ1: Proactivator polypeptide-like 1 [Cleaved into: Saposin A-like; Saposin B-Val . . .		3	3	127	126	3	1.02
					129	128	3	3.85
					131	130	3	0.75
1144	PSMA1 P25786$_{1,2}$: Proteasome subunit alpha type-1	2/2	2	2	127	126	2	1.65
					129	128	2	0.63
					131	130	2	1.19
1145	PSMA3 P25788$_{1,2}$: Proteasome subunit alpha type-3	2/2	2	2	127	126	2	1.76
					129	128	2	0.77
					131	130	2	1.38
1146	PSMA6 P60900$_1$: Proteasome subunit alpha type-6	1/2	3	3	127	126	3	2.33
					129	128	3	0.68
					131	130	3	1.26

#	protein	group	peptides	spectra	ch1	ch2	quant	ratio
**1147**	PSMB1 P20618: Proteasome subunit beta type-1		2	2	127	126	2	**1.03**
					129	128	2	**1.11**
					131	130	2	**1.13**
**1148**	PSMB6 P28072: Proteasome subunit beta type-6		2	2	127	126	2	**1.35**
					129	128	2	**1.37**
					131	130	2	**1.07**
**1149**	PSMB7 $Q99436_1$: Proteasome subunit beta type-7	1/2	2	2	127	126	2	**1.93**
					129	128	2	**1.00**
					131	130	2	**1.30**
**1150**	PSMC1 $P62191_1$: 26S protease regulatory subunit 4	1/2	4	4	127	126	3	**1.25**
					129	128	3	**0.97**
					131	130	3	**1.07**
**1151**	PSMC5 $P62195_{1,2}$: 26S protease regulatory subunit 8	2/2	2	2	127	126	1	**1.06**
					129	128	1	**0.71**
					131	130	1	**0.97**
**1152**	PSMD1 $Q99460_{1,2}$: 26S proteasome non-ATPase regulatory subunit 1	2/2	4	4	127	126	4	**0.78**
					129	128	4	**1.42**
					131	130	3	**0.98**
**1153**	PSMD2 $Q13200_{1-3}$: 26S proteasome non-ATPase regulatory subunit 2	3/3	2	2	127	126	1	**1.09**
					129	128	1	**1.11**
					131	130	1	**1.06**
**1154**	PSME1 $Q06323_{1-3}$: Proteasome activator complex subunit 1	3/3	7	8	127	126	8	**1.11**
					129	128	7	**0.93**
					131	130	8	**0.88**
**1155**	PSME2 Q9UL46: Proteasome activator complex subunit 2		10	17	127	126	13	**0.97**
					129	128	13	**1.24**
					131	130	14	**0.89**
**1156**	PSPN O60542: Persephin		1	1	127	126	1	**1.12**
					129	128	1	**1.38**
					131	130	1	**0.89**
**1157**	PTBP1 $P26599_{1-3}$: Polypyrimidine tract-binding protein 1	3/16	8	10	127	126	10	**1.75**
					129	128	10	**0.66**
					131	130	9	**1.19**
**1158**	PTGDS P41222: Prostaglandin-H2 D-isomerase		5	9	127	126	9	**1.57**
					129	128	9	**1.20**
					131	130	9	**1.15**

#	protein	group	peptides	spectra	ch1	ch2	quant	ratio
**1159**	PTGIS Q16647: Prostacyclin synthase		2	2	127	126	2	**0.92**
					129	128	2	**1.70**
					131	130	2	**1.20**
**1160**	PTGR1 $Q14914_{1,2}$: Prostaglandin reductase 1	2/2	2	2	127	126	2	**0.72**
					129	128	2	**1.71**
					131	130	2	**0.97**
**1161**	PTMA $P06454_{1,2}$: Prothymosin alpha [Cleaved into: Prothymosin alpha, N-terminally processed; . . .	2/2	3	5	127	126	4	**2.27**
					129	128	4	**0.55**
					131	130	5	**1.39**
**1162**	PTMS P20962: Parathymosin		4	10	127	126	9	**2.67**
					129	128	8	**0.41**
					131	130	9	**1.82**
**1163**	PTN P21246: Pleiotrophin		2	2	127	126	2	**1.62**
					129	128	2	**1.40**
					131	130	2	**1.75**
**1164**	PTPN23 Q9H3S7: Tyrosine-protein phosphatase non-receptor type 23		4	5	127	126	4	**0.82**
					129	128	5	**1.06**
					131	130	3	**0.88**
**1165**	PTPRF $P10586_{1,2}$: Receptor-type tyrosine-protein phosphatase F	2/2	4	4	127	126	4	**0.66**
					129	128	4	**0.79**
					131	130	4	**0.99**
**1166**	PTPRS $Q13332_{1-4,6}$: Receptor-type tyrosine-protein phosphatase S	5/7	3	3	127	126	3	**0.94**
					129	128	3	**2.51**
					131	130	3	**0.87**
**1167**	PTRF $Q6NZI2_{1}$: Polymerase I and transcript release factor	1/3	14	23	127	126	21	**2.35**
					129	128	19	**0.60**
					131	130	20	**1.96**
**1168**	PUF60 $Q9UHX1_{1-6}$: Poly(U)-binding-splicing factor PUF60	6/6	2	3	127	126	3	**1.31**
					129	128	3	**1.21**
					131	130	3	**1.22**
**1169**	PURA Q00577: Transcriptional activator protein Pur-alpha		3	3	127	126	3	**2.09**
					129	128	3	**0.69**
					131	130	3	**1.31**
**1170**	PXDN $Q92626_{1}$: Peroxidasin homolog	1/2	36	46	127	126	41	**0.96**
					129	128	43	**1.45**
					131	130	43	**1.08**

#	protein	group	peptides	spectra	ch1	ch2	quant	ratio
1171	PZP P20742$_1$: Pregnancy zone protein	1/2	32	49	127	126	31	**0.51**
					129	128	19	**0.54**
					131	130	33	**0.94**
1172	QPCT Q16769$_1$: Glutaminyl-peptide cyclotransferase	1/2	4	5	127	126	5	**1.36**
					129	128	5	**1.13**
					131	130	4	**1.05**
1173	QSOX1 O00391$_1$: Sulfhydryl oxidase 1	1/2	38	119	127	126	111	**0.52**
					129	128	110	**2.09**
					131	130	115	**0.83**
1174	RAB10 P61026: Ras-related protein Rab-10		2	3	127	126	3	**0.97**
					129	128	1	**2.41**
					131	130	1	**1.15**
1175	RAB11B Q15907$_1$: Ras-related protein Rab-11B	1/4	7	7	127	126	6	**1.34**
					129	128	7	**0.59**
					131	130	7	**0.89**
1176	RAB14 P61106: Ras-related protein Rab-14		2	3	127	126	3	**1.36**
					129	128	3	**0.90**
					131	130	3	**1.00**
1177	RAB1A P62820$_1$: Ras-related protein Rab-1A	1/2	5	5	127	126	3	**1.18**
					129	128	3	**1.23**
					131	130	3	**0.95**
1178	RAB1B Q9H0U4: Ras-related protein Rab-1B	1/2	6	7	127	126	4	**1.20**
					129	128	5	**1.18**
					131	130	4	**1.00**
1179	RAB2A P61019$_{1,2}$: Ras-related protein Rab-2A	2/2	2	2	127	126	2	**0.86**
					129	128	2	**1.30**
					131	130	2	**1.02**
1180	RAB7A P51149: Ras-related protein Rab-7a		3	3	127	126	3	**1.49**
					129	128	3	**1.07**
					131	130	3	**1.03**
1181	RABEP1 Q15276$_{1,2}$: Rab GTPase-binding effector protein 1	2/2	2	4	127	126	2	**1.27**
					129	128	2	**0.12**
					131	130	2	**0.97**
1182	RAC1 P63000$_{1,2}$: Ras-related C3 botulinum toxin substrate 1	2/4	6	6	127	126	5	**0.81**
					129	128	5	**1.84**
					131	130	4	**1.13**

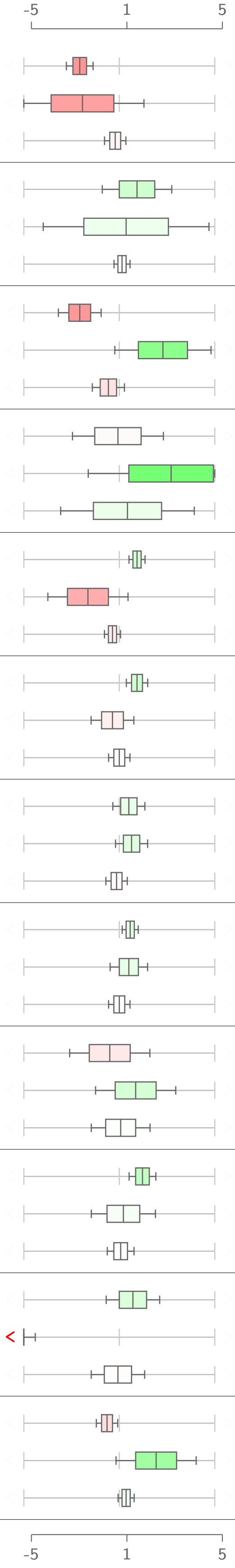

#	protein	group	peptides	spectra	ch1	ch2	quant	ratio
**1183**	RACK1 P63244: Receptor of activated protein C kinase 1		7	8	127	126	7	**2.01**
					129	128	8	**0.62**
					131	130	7	**1.07**
**1184**	RAD23A $P54725_{1-3}$: UV excision repair protein RAD23 homolog A	3/3	2	2	127	126	1	**4.36**
					129	128		
					131	130	1	**1.54**
**1185**	RAD23B $P54727_{1}$: UV excision repair protein RAD23 homolog B		2	2	127	126	1	**1.47**
					129	128	1	**1.02**
					131	130	1	**1.31**
**1186**	RAN P62826: GTP-binding nuclear protein Ran		5	6	127	126	6	**1.61**
					129	128	6	**0.67**
					131	130	6	**1.08**
**1187**	RAP1B $P61224_{3}$: Ras-related protein Rap-1b	1/6	6	8	127	126	7	**0.98**
					129	128	7	**1.30**
					131	130	6	**1.11**
**1188**	RARRES1 $P49788_{1,2}$: Retinoic acid receptor responder protein 1	2/2	9	16	127	126	16	**0.60**
					129	128	16	**1.43**
					131	130	15	**1.22**
**1189**	RARRES2 Q99969: Retinoic acid receptor responder protein 2		9	86	127	126	83	**1.05**
					129	128	85	**1.30**
					131	130	78	**1.10**
**1190**	RARS $P54136_{1,2}$: Arginine--tRNA ligase, cytoplasmic	2/2	3	3	127	126	3	**0.79**
					129	128	3	**1.86**
					131	130	3	**1.07**
**1191**	RBBP4 $Q09028_{1,3}$: Histone-binding protein RBBP4	2/4	3	4	127	126	3	**1.63**
					129	128	3	**0.51**
					131	130	3	**1.21**
**1192**	RBBP7 $Q16576_{1}$: Histone-binding protein RBBP7		2	4	127	126	3	**2.30**
					129	128	2	**0.73**
					131	130	3	**1.45**
**1193**	RBM6 $P78332_{1}$: RNA-binding protein 6	1/2	4	9	127	126	7	**0.81**
					129	128	6	**0.98**
					131	130	7	**1.01**
**1194**	RBM8A $Q9Y5S9_{1,2}$: RNA-binding protein 8A	2/2	1	1	127	126	1	**2.57**
					129	128	1	**0.71**
					131	130	1	**0.93**

#	protein	group	peptides	spectra	ch1	ch2	quant	ratio
**1195**	RBMX P38159$_{1,2}$: RNA-binding motif protein, X chromosome	2/3	8	11	127	126	8	**1.77**
					129	128	8	**0.95**
					131	130	9	**2.08**
**1196**	RBP4 P02753: Retinol-binding protein 4		10	110	127	126	108	**1.64**
					129	128	102	**1.33**
					131	130	102	**1.21**
**1197**	RCN1 Q15293$_{1,2}$: Reticulocalbin-1	2/2	3	3	127	126	3	**1.87**
					129	128	3	**0.83**
					131	130	3	**1.05**
**1198**	RCN3 Q96D15: Reticulocalbin-3		4	5	127	126	5	**1.40**
					129	128	5	**0.95**
					131	130	5	**1.19**
**1199**	RDX P35241$_{1}$: Radixin	1/3	9	11	127	126	2	**0.91**
					129	128	2	**1.14**
					131	130	2	**0.90**
**1200**	RECK O95980: Reversion-inducing cysteine-rich protein with Kazal motifs		1	1	127	126	1	**0.75**
					129	128	1	**2.94**
					131	130	1	**0.99**
**1201**	RELA Q04206$_{1-4}$: Transcription factor p65	4/4	2	2	127	126	2	**1.48**
					129	128	2	**1.70**
					131	130	2	**1.39**
**1202**	RHOA P61586, RHOC P08134: Transforming protein RhoA, Rho-related GTP-binding protein RhoC	2/2	2	2	127	126	2	**1.08**
					129	128	2	**1.07**
					131	130	2	**1.42**
**1203**	RIC8A Q9NPQ8$_{1-4}$: Synembryn-A	4/4	2	3	127	126	1	**0.94**
					129	128	1	**0.91**
					131	130	1	**0.93**
**1204**	RLF Q13129: Zinc finger protein Rlf		7	12	127	126	8	**1.97**
					129	128	9	**1.15**
					131	130	10	**0.90**
**1205**	RNASE1 P07998: Ribonuclease pancreatic		7	36	127	126	35	**1.53**
					129	128	36	**3.39**
					131	130	36	**1.14**
**1206**	RNASE3 P12724: Eosinophil cationic protein		2	2	127	126	2	**1.69**
					129	128	2	**2.39**
					131	130	2	**1.08**

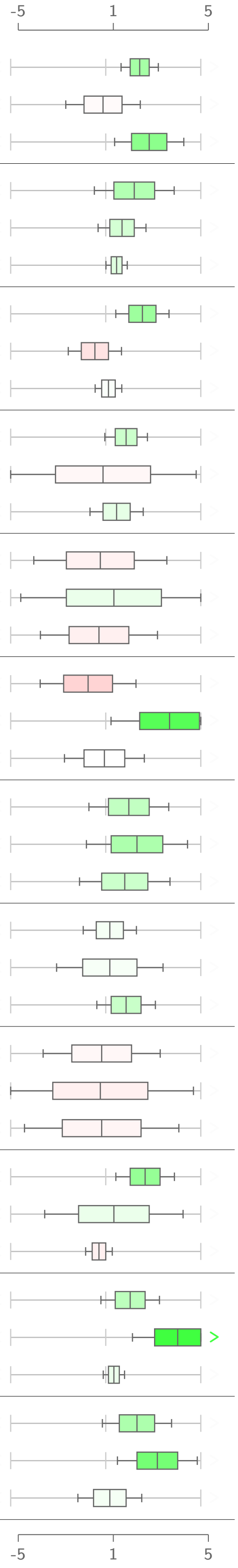

#	protein	group	peptides	spectra	ch1	ch2	quant	ratio
1207	RNASE4 P34096: Ribonuclease 4		13	168	127	126	154	**1.31**
					129	128	150	**2.60**
					131	130	151	**1.00**
1208	RNF181 Q9P0P0: E3 ubiquitin-protein ligase RNF181		1	1	127	126	1	**1.58**
					129	128	1	**1.80**
					131	130	1	**0.87**
1209	RNH1 P13489: Ribonuclease inhibitor		12	13	127	126	10	**0.98**
					129	128	12	**2.02**
					131	130	11	**1.06**
1210	RPA2 $P15927_3$: Replication protein A 32 kDa subunit	1/3	3	4	127	126	4	**1.73**
					129	128	4	**0.81**
					131	130	4	**1.25**
1211	RPL10 P27635: 60S ribosomal protein L10		3	3	127	126	3	**1.15**
					129	128	3	**1.85**
					131	130	3	**1.87**
1212	RPL10A P62906: 60S ribosomal protein L10a		2	2	127	126	2	**2.44**
					129	128	2	**0.59**
					131	130	2	**1.69**
1213	RPL12 $P30050_1$: 60S ribosomal protein L12	1/2	5	5	127	126	5	**1.76**
					129	128	4	**0.65**
					131	130	5	**1.45**
1214	RPL13 $P26373_{1,2}$: 60S ribosomal protein L13	2/2	4	4	127	126	3	**2.00**
					129	128	3	**0.99**
					131	130	3	**2.12**
1215	RPL14 P50914: 60S ribosomal protein L14		2	2	127	126	2	**1.29**
					129	128	2	**1.83**
					131	130	2	**1.93**
1216	RPL17 $P18621_{1-3}$: 60S ribosomal protein L17	3/3	2	2	127	126	1	**1.65**
					129	128	1	**1.30**
					131	130	1	**2.07**
1217	RPL18 $Q07020_{1,2}$: 60S ribosomal protein L18	2/2	1	1	127	126	1	**1.60**
					129	128	1	**0.90**
					131	130	1	**1.56**
1218	RPL18A Q02543: 60S ribosomal protein L18a		1	2	127	126	2	**0.87**
					129	128	2	**0.91**
					131	130	2	**1.11**

#	protein	group	peptides	spectra	ch1	ch2	quant	ratio
**1219**	RPL19 P84098: 60S ribosomal protein L19		5	5	127	126	5	**1.49**
					129	128	5	**1.86**
					131	130	4	**1.80**
**1220**	RPL22 P35268: 60S ribosomal protein L22		2	3	127	126	3	**2.86**
					129	128	3	**0.68**
					131	130	3	**1.74**
**1221**	RPL23A P62750: 60S ribosomal protein L23a		3	4	127	126	4	**1.28**
					129	128	4	**1.17**
					131	130	4	**1.64**
**1222**	RPL24 P83731: 60S ribosomal protein L24		2	3	127	126	3	**1.63**
					129	128	3	**1.48**
					131	130	3	**2.02**
**1223**	RPL26 P61254: 60S ribosomal protein L26		3	3	127	126	3	**2.27**
					129	128	3	**0.68**
					131	130	3	**3.11**
**1224**	RPL27 P61353: 60S ribosomal protein L27		2	2	127	126	2	**1.41**
					129	128	2	**1.67**
					131	130	2	**1.08**
**1225**	RPL28 P46779$_{1-5}$: 60S ribosomal protein L28	5/5	2	2	127	126	2	**1.71**
					129	128	2	**0.84**
					131	130	2	**1.91**
**1226**	RPL3 P39023: 60S ribosomal protein L3		2	2	127	126	2	**1.35**
					129	128	2	**1.35**
					131	130	2	**1.50**
**1227**	RPL30 P62888: 60S ribosomal protein L30		3	3	127	126	3	**1.86**
					129	128	3	**0.79**
					131	130	3	**1.91**
**1228**	RPL31 P62899$_{1-3}$: 60S ribosomal protein L31	3/3	3	3	127	126	3	**1.09**
					129	128	3	**0.78**
					131	130	3	**1.39**
**1229**	RPL34 P49207: 60S ribosomal protein L34		2	2	127	126	2	**2.13**
					129	128	2	**1.72**
					131	130	2	**2.27**
**1230**	RPL35A P18077: 60S ribosomal protein L35a		3	3	127	126	3	**1.59**
					129	128	3	**0.79**
					131	130	3	**1.74**

#	protein	group	peptides	spectra	ch1	ch2	quant	ratio
1231	RPL37 P61927: 60S ribosomal protein L37		2	3	127	126	3	**1.52**
					129	128	3	**1.86**
					131	130	3	**1.76**
1232	RPL5 P46777: 60S ribosomal protein L5		7	9	127	126	5	**1.53**
					129	128	5	**0.95**
					131	130	6	**1.80**
1233	RPL6 Q02878: 60S ribosomal protein L6		2	2	127	126	2	**1.42**
					129	128	2	**0.91**
					131	130	2	**1.61**
1234	RPL7 P18124: 60S ribosomal protein L7		4	4	127	126	3	**1.46**
					129	128	3	**0.89**
					131	130	4	**1.11**
1235	RPL7A P62424: 60S ribosomal protein L7a		5	5	127	126	5	**1.85**
					129	128	4	**0.87**
					131	130	5	**1.48**
1236	RPL8 P62917: 60S ribosomal protein L8		6	6	127	126	5	**1.51**
					129	128	5	**1.07**
					131	130	5	**2.03**
1237	RPLP0 P05388$_{1,2}$: 60S acidic ribosomal protein P0	2/3	7	10	127	126	9	**1.57**
					129	128	9	**0.71**
					131	130	8	**1.36**
1238	RPLP2 P05387: 60S acidic ribosomal protein P2		3	4	127	126	3	**4.04**
					129	128	3	**0.34**
					131	130	4	**1.58**
1239	RPN1 P04843: Dolichyl-diphosphooligosaccharide--protein glycosyltransferase subunit 1		8	9	127	126	8	**1.58**
					129	128	9	**0.84**
					131	130	8	**1.39**
1240	RPS10 P46783: 40S ribosomal protein S10	1/2	4	4	127	126	4	**1.46**
					129	128	3	**1.00**
					131	130	4	**1.49**
1241	RPS12 P25398: 40S ribosomal protein S12		3	3	127	126	3	**2.21**
					129	128	3	**0.69**
					131	130	3	**1.46**
1242	RPS14 P62263: 40S ribosomal protein S14		4	4	127	126	3	**1.74**
					129	128	3	**0.93**
					131	130	4	**1.71**

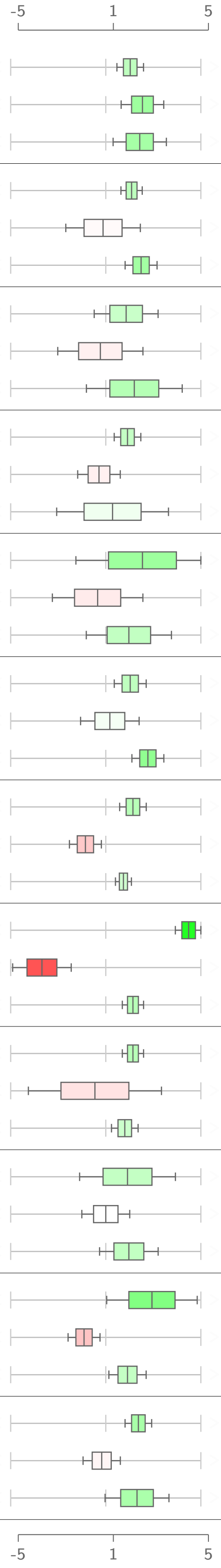

#	protein	group	peptides	spectra	ch1	ch2	quant	ratio
1243	RPS18 P62269: 40S ribosomal protein S18		3	3	127	126	3	**1.59**
					129	128	3	**0.73**
					131	130	3	**1.93**
1244	RPS19 P39019: 40S ribosomal protein S19		3	4	127	126	3	**2.92**
					129	128	3	**1.14**
					131	130	4	**2.14**
1245	RPS2 P15880: 40S ribosomal protein S2		1	1	127	126	1	**1.26**
					129	128	1	**0.84**
					131	130	1	**1.96**
1246	RPS20 P60866$_{1,2}$: 40S ribosomal protein S20	2/2	3	5	127	126	5	**2.18**
					129	128	4	**0.67**
					131	130	5	**1.76**
1247	RPS25 P62851: 40S ribosomal protein S25		2	2	127	126	2	**1.37**
					129	128	2	**0.89**
					131	130	2	**1.99**
1248	RPS27A P62979: Ubiquitin-40S ribosomal protein S27a	1/4	9	13	127	126	12	**1.38**
					129	128	9	**1.72**
					131	130	11	**1.29**
1249	RPS28 P62857: 40S ribosomal protein S28		2	3	127	126	3	**1.16**
					129	128	3	**0.43**
					131	130	3	**0.98**
1250	RPS3 P23396$_{1,2}$: 40S ribosomal protein S3	2/2	7	7	127	126	5	**1.60**
					129	128	7	**0.64**
					131	130	6	**1.21**
1251	RPS3A P61247: 40S ribosomal protein S3a		2	2	127	126	2	**1.80**
					129	128	2	**0.90**
					131	130	2	**1.61**
1252	RPS4X P62701: 40S ribosomal protein S4, X isoform		2	2	127	126	2	**0.92**
					129	128	2	**1.28**
					131	130	2	**1.27**
1253	RPS5 P46782: 40S ribosomal protein S5 [Cleaved into: 40S ribosomal protein S5, N-terminal . . .		3	3	127	126	3	**1.91**
					129	128	3	**0.66**
					131	130	3	**1.42**
1254	RPS6 P62753: 40S ribosomal protein S6		5	6	127	126	5	**1.43**
					129	128	6	**1.23**
					131	130	6	**1.65**

#	protein	group	peptides	spectra	ch1	ch2	quant	ratio
**1255**	RPS6KA3 P51812: Ribosomal protein S6 kinase alpha-3		4	4	127	126	3	**1.19**
					129	128	4	**1.03**
					131	130	4	**1.18**
**1256**	RPS7 P62081: 40S ribosomal protein S7		3	3	127	126	3	**2.64**
					129	128	3	**0.56**
					131	130	3	**1.88**
**1257**	RPS9 P46781: 40S ribosomal protein S9		3	3	127	126	3	**1.80**
					129	128	3	**1.02**
					131	130	3	**2.11**
**1258**	RPSA P08865: 40S ribosomal protein SA		12	15	127	126	13	**2.18**
					129	128	13	**0.38**
					131	130	14	**1.03**
**1259**	RRBP1 Q9P2E9$_{1,2}$: Ribosome-binding protein 1	2/3	9	10	127	126	10	**1.38**
					129	128	7	**1.09**
					131	130	10	**1.59**
**1260**	RTN1 Q16799$_{1-3}$, RTN3 O95197$_{1-7}$: Reticulon-1, Reticulon-3	10/10	1	1	127	126	1	**1.04**
					129	128	1	**1.43**
					131	130	1	**1.29**
**1261**	RTN4 Q9NQC3$_{1,2,5}$: Reticulon-4	3/3	2	2	127	126	2	**0.98**
					129	128	2	**1.26**
					131	130	2	**1.21**
**1262**	RUVBL2 Q9Y230$_{1}$: RuvB-like 2		1	2	127	126	2	**1.58**
					129	128	2	**0.69**
					131	130	2	**1.18**
**1263**	S100A10 P60903: Protein S100-A10		6	14	127	126	11	**1.71**
					129	128	12	**0.94**
					131	130	12	**1.36**
**1264**	S100A11 P31949: Protein S100-A11		3	4	127	126	4	**0.96**
					129	128	3	**2.16**
					131	130	4	**1.00**
**1265**	S100A6 P06703: Protein S100-A6		4	7	127	126	7	**1.39**
					129	128	6	**1.00**
					131	130	7	**1.11**
**1266**	S100A7 P31151: Protein S100-A7	1/2	2	2	127	126	2	**0.84**
					129	128	2	**4.32**
					131	130	2	**0.36**

#	protein	group	peptides	spectra	ch1	ch2	quant	ratio
**1267**	S100A8 P05109: Protein S100-A8		7	12	127	126	12	**0.82**
					129	128	11	**2.77**
					131	130	12	**0.61**
**1268**	S100A9 P06702: Protein S100-A9		2	3	127	126	3	**0.78**
					129	128	3	**4.29**
					131	130	3	**0.42**
**1269**	SAA1 P0DJI8: Serum amyloid A-1 protein	1/2	10	106	127	126	29	**1.19**
					129	128	29	**1.26**
					131	130	26	**0.87**
**1270**	SAA2 P0DJI9$_1$: Serum amyloid A-2 protein		9	81	127	126	4	**1.00**
					129	128	3	**1.18**
					131	130	3	**0.78**
**1271**	SAA4 P35542: Serum amyloid A-4 protein		7	121	127	126	118	**0.53**
					129	128	112	**1.97**
					131	130	115	**0.90**
**1272**	SAE1 Q9UBE0$_{1-3}$: SUMO-activating enzyme subunit 1	3/3	3	3	127	126	3	**1.91**
					129	128	3	**1.07**
					131	130	3	**1.20**
**1273**	SARS P49591: Serine--tRNA ligase, cytoplasmic		7	8	127	126	8	**1.19**
					129	128	8	**0.56**
					131	130	7	**1.07**
**1274**	SBSN Q6UWP8$_1$: Suprabasin	1/2	10	11	127	126	10	**1.14**
					129	128	11	**3.89**
					131	130	11	**1.06**
**1275**	SCG2 P13521: Secretogranin-2		25	37	127	126	37	**1.53**
					129	128	36	**1.53**
					131	130	30	**1.08**
**1276**	SCG3 Q8WXD2$_1$: Secretogranin-3	1/2	7	7	127	126	6	**1.86**
					129	128	7	**1.62**
					131	130	7	**1.15**
**1277**	SCG5 P05408$_2$: Neuroendocrine protein 7B2	1/2	3	7	127	126	7	**1.59**
					129	128	7	**1.92**
					131	130	6	**1.05**
**1278**	SCGB1A1 P11684: Uteroglobin		3	5	127	126	5	**1.61**
					129	128	5	**1.31**
					131	130	3	**1.06**

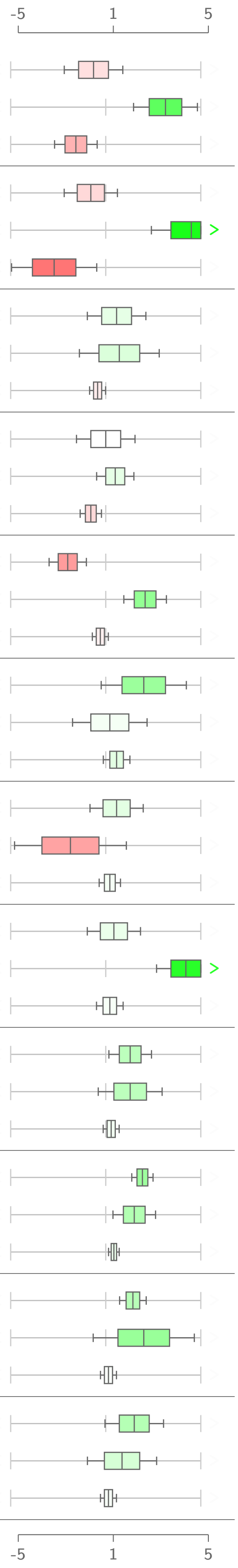

#	protein	group	peptides	spectra	ch1	ch2	quant	ratio
**1279**	SCPEP1 Q9HB40$_1$: Retinoid-inducible serine carboxypeptidase		2	2	127	126	2	**1.02**
					129	128	2	**1.77**
					131	130	2	**1.09**
**1280**	SCRN1 Q12765$_{1-3}$: Secernin-1	3/3	2	2	127	126	2	**2.42**
					129	128	2	**0.56**
					131	130	2	**1.08**
**1281**	SCUBE2 Q9NQ36$_{1,3}$: Signal peptide, CUB and EGF-like domain-containing protein 2	2/3	5	5	127	126	5	**1.00**
					129	128	4	**1.33**
					131	130	3	**0.94**
**1282**	SDF4 Q9BRK5$_1$: 45 kDa calcium-binding protein	1/6	13	24	127	126	24	**1.63**
					129	128	20	**1.13**
					131	130	22	**1.05**
**1283**	SDPR O95810: Serum deprivation-response protein		11	13	127	126	13	**1.94**
					129	128	13	**0.41**
					131	130	13	**1.51**
**1284**	SEC13 P55735$_{1-3}$: Protein SEC13 homolog	3/3	2	2	127	126	2	**2.15**
					129	128	2	**0.50**
					131	130	2	**1.34**
**1285**	SEC23A Q15436$_1$: Protein transport protein Sec23A	1/3	3	3	127	126	3	**1.04**
					129	128	3	**0.89**
					131	130	3	**1.16**
**1286**	SEC23IP Q9Y6Y8$_{1,2}$: SEC23-interacting protein	2/2	4	4	127	126	3	**1.27**
					129	128	3	**0.95**
					131	130	3	**1.01**
**1287**	SEC24C P53992$_1$: Protein transport protein Sec24C	1/2	4	4	127	126	4	**0.90**
					129	128	4	**1.24**
					131	130	4	**1.21**
**1288**	SEMA3B Q13214$_{1,2}$: Semaphorin-3B	2/2	6	6	127	126	6	**0.97**
					129	128	5	**1.19**
					131	130	5	**0.95**
**1289**	SEMA3C Q99985$_1$: Semaphorin-3C	1/2	6	7	127	126	6	**1.02**
					129	128	5	**1.43**
					131	130	7	**1.05**
**1290**	SEMA3F Q13275$_{1,2}$: Semaphorin-3F	2/2	4	4	127	126	4	**1.19**
					129	128	4	**0.79**
					131	130	4	**0.95**

#	protein	group	peptides	spectra	ch1	ch2	quant	ratio
1291	SEMA3G Q9NS98: Semaphorin-3G		5	5	127	126	4	**1.11**
					129	128	5	**0.70**
					131	130	4	**0.87**
1292	SEPP1 P49908: Selenoprotein P		7	19	127	126	18	**1.43**
					129	128	16	**1.15**
					131	130	15	**1.06**
1293	SEPT2 Q15019$_{1,2}$: Septin-2	2/3	5	5	127	126	5	**1.72**
					129	128	4	**0.50**
					131	130	3	**1.18**
1294	SEPT7 Q16181$_{1,2}$: Septin-7	2/2	10	65	127	126	52	**0.85**
					129	128	33	**1.23**
					131	130	50	**1.04**
1295	SERPINA1 P01009$_1$: Alpha-1-antitrypsin	1/3	27	229	127	126	221	**1.04**
					129	128	217	**0.63**
					131	130	217	**1.17**
1296	SERPINA10 Q9UK55: Protein Z-dependent protease inhibitor		14	41	127	126	41	**0.54**
					129	128	37	**1.26**
					131	130	39	**0.89**
1297	SERPINA3 P01011$_1$: Alpha-1-antichymotrypsin	1/2	16	35	127	126	35	**0.74**
					129	128	33	**0.94**
					131	130	34	**1.34**
1298	SERPINA4 P29622: Kallistatin		17	40	127	126	37	**0.33**
					129	128	40	**1.48**
					131	130	40	**0.76**
1299	SERPINA5 P05154: Plasma serine protease inhibitor		11	18	127	126	16	**0.67**
					129	128	17	**0.80**
					131	130	17	**1.02**
1300	SERPINA6 P08185: Corticosteroid-binding globulin		5	6	127	126	6	**0.46**
					129	128	6	**2.41**
					131	130	6	**1.31**
1301	SERPINA7 P05543: Thyroxine-binding globulin		10	12	127	126	12	**0.36**
					129	128	12	**2.55**
					131	130	12	**0.87**
1302	SERPINC1 P01008: Antithrombin-III		28	119	127	126	109	**0.80**
					129	128	106	**0.77**
					131	130	118	**1.12**

#	protein	group	peptides	spectra	ch1	ch2	quant	ratio
**1303**	SERPIND1 P05546: Heparin cofactor 2		27	270	127	126	269	**0.33**
					129	128	253	**1.55**
					131	130	260	**0.75**
**1304**	SERPINE1 $P05121_1$: Plasminogen activator inhibitor 1	1/2	7	10	127	126	10	**1.02**
					129	128	10	**0.55**
					131	130	9	**0.95**
**1305**	SERPINE2 $P07093_{1-3}$: Glia-derived nexin	3/3	8	9	127	126	8	**0.73**
					129	128	9	**1.34**
					131	130	6	**1.04**
**1306**	SERPINF1 P36955: Pigment epithelium-derived factor		20	53	127	126	48	**1.52**
					129	128	52	**0.54**
					131	130	49	**1.11**
**1307**	SERPINF2 $P08697_1$: Alpha-2-antiplasmin	1/2	19	66	127	126	66	**0.59**
					129	128	64	**1.70**
					131	130	65	**0.89**
**1308**	SERPING1 $P05155_{1-3}$: Plasma protease C1 inhibitor	3/3	11	23	127	126	23	**0.46**
					129	128	21	**1.40**
					131	130	23	**1.12**
**1309**	SERPINH1 P50454: Serpin H1		5	5	127	126	5	**1.12**
					129	128	5	**0.76**
					131	130	5	**1.29**
**1310**	SET $Q01105_{1-4}$, SETSIP P0DME0: Protein SET, Protein SETSIP	5/5	2	2	127	126	1	**3.96**
					129	128	1	**0.63**
					131	130	1	**1.37**
**1311**	SF3A1 $Q15459_{1,2}$: Splicing factor 3A subunit 1	2/2	5	7	127	126	5	**1.15**
					129	128	4	**2.33**
					131	130	5	**1.10**
**1312**	SFN $P31947_{1,2}$: 14-3-3 protein sigma	2/2	8	13	127	126	3	**0.56**
					129	128	4	**2.85**
					131	130	4	**0.80**
**1313**	SFPQ $P23246_{1,2}$: Splicing factor, proline- and glutamine-rich	2/2	11	15	127	126	12	**1.74**
					129	128	11	**0.97**
					131	130	10	**1.42**
**1314**	SFRP1 Q8N474: Secreted frizzled-related protein 1		4	7	127	126	6	**1.79**
					129	128	6	**1.43**
					131	130	6	**1.49**

#	protein	group	peptides	spectra	ch1	ch2	quant	ratio
**1315**	SFRP2 Q96HF1: Secreted frizzled-related protein 2		11	46	127	126	42	**1.65**
					129	128	36	**1.44**
					131	130	41	**0.88**
**1316**	SFRP4 Q6FHJ7: Secreted frizzled-related protein 4		6	8	127	126	8	**1.48**
					129	128	7	**1.24**
					131	130	6	**1.25**
**1317**	SH3BGRL3 Q9H299: SH3 domain-binding glutamic acid-rich-like protein 3		2	3	127	126	3	**1.25**
					129	128	3	**2.16**
					131	130	3	**1.25**
**1318**	SHBG P04278$_1$: Sex hormone-binding globulin	1/5	13	24	127	126	24	**0.34**
					129	128	24	**2.25**
					131	130	24	**0.80**
**1319**	SKIV2L2 P42285: Superkiller viralicidic activity 2-like 2	1/2	3	3	127	126	3	**0.54**
					129	128	3	**1.60**
					131	130	3	**0.74**
**1320**	SKP1 P63208$_1$: S-phase kinase-associated protein 1	1/2	8	12	127	126	10	**2.11**
					129	128	9	**0.74**
					131	130	10	**1.23**
**1321**	SLC25A3 Q00325$_{1,2}$: Phosphate carrier protein, mitochondrial	2/2	2	2	127	126	2	**1.17**
					129	128	2	**1.13**
					131	130	2	**1.71**
**1322**	SLC25A5 P05141, SLC25A6 P12236: ADP/ATP translocase 2, ADP/ATP translocase 3	2/3	4	4	127	126	4	**1.79**
					129	128	4	**1.01**
					131	130	4	**1.31**
**1323**	SLC28A3 Q9HAS3$_1$: Solute carrier family 28 member 3		3	29	127	126	12	**0.63**
					129	128	5	**1.22**
					131	130	10	**0.85**
**1324**	SLC9A3R1 O14745$_1$: Na(+)/H(+) exchange regulatory cofactor NHE-RF1	1/2	6	7	127	126	7	**0.60**
					129	128	6	**3.17**
					131	130	5	**1.20**
**1325**	SLIRP Q9GZT3$_{1,2}$: SRA stem-loop-interacting RNA-binding protein, mitochondrial	2/2	2	2	127	126	1	**1.42**
					129	128	1	**1.26**
					131	130	1	**1.26**
**1326**	SLIT3 O75094$_{1,2,4}$: Slit homolog 3 protein	3/3	6	11	127	126	10	**1.00**
					129	128	9	**1.12**
					131	130	10	**0.99**

#	protein	group	peptides	spectra	ch1	ch2	quant	ratio
**1327**	SLK Q9H2G2$_{1,2}$: STE20-like serine/threonine-protein kinase	2/2	5	5	127	126	3	**1.03**
					129	128	5	**1.80**
					131	130	3	**1.09**
**1328**	SLPI P03973: Antileukoproteinase		14	109	127	126	92	**1.72**
					129	128	88	**5.69**
					131	130	89	**1.10**
**1329**	SLURP1 P55000: Secreted Ly-6/uPAR-related protein 1		2	3	127	126	3	**2.07**
					129	128	3	**2.30**
					131	130	3	**1.13**
**1330**	SMS P52788$_{1}$: Spermine synthase		3	3	127	126	3	**1.67**
					129	128	3	**0.69**
					131	130	3	**1.13**
**1332**	SND1 Q7KZF4: Staphylococcal nuclease domain-containing protein 1		11	11	127	126	9	**1.44**
					129	128	7	**1.05**
					131	130	10	**1.27**
**1333**	SNED1 Q8TER0$_{1,3-5}$: Sushi, nidogen and EGF-like domain-containing protein 1	4/4	9	15	127	126	15	**0.94**
					129	128	14	**1.54**
					131	130	15	**1.01**
**1334**	SNRNP70 P08621$_{1,2}$: U1 small nuclear ribonucleoprotein 70 kDa	2/3	3	3	127	126	3	**1.67**
					129	128	3	**1.22**
					131	130	3	**1.67**
**1335**	SNRPF P62306: Small nuclear ribonucleoprotein F		1	1	127	126	1	**1.93**
					129	128	1	**0.66**
					131	130	1	**1.37**
**1336**	SNX1 Q13596$_{1-3}$: Sorting nexin-1	3/3	4	4	127	126	3	**1.82**
					129	128	2	**0.57**
					131	130	3	**1.01**
**1337**	SNX2 O60749$_{1,2}$: Sorting nexin-2	2/2	4	4	127	126	4	**1.42**
					129	128	3	**0.55**
					131	130	3	**0.96**
**1338**	SNX5 Q9Y5X3$_{1}$: Sorting nexin-5		2	2	127	126	1	**2.09**
					129	128	1	**0.85**
					131	130	1	**1.47**
**1339**	SNX6 Q9UNH7$_{1,2}$: Sorting nexin-6	2/2	5	7	127	126	6	**1.68**
					129	128	7	**1.17**
					131	130	7	**1.12**

#	protein	group	peptides	spectra	ch1	ch2	quant	ratio
**1340**	SNX9 Q9Y5X1: Sorting nexin-9		2	2	127	126	2	**1.46**
					129	128	2	**1.50**
					131	130	2	**1.24**
**1341**	SOD3 P08294: Extracellular superoxide dismutase [Cu-Zn]		10	83	127	126	80	**1.33**
					129	128	74	**1.03**
					131	130	78	**1.19**
**1342**	SORBS2 O94875$_{11}$: Sorbin and SH3 domain-containing protein 2		2	2	127	126	2	**3.26**
					129	128	2	**0.49**
					131	130	2	**1.83**
**1343**	SOS1 Q07889$_{1}$: Son of sevenless homolog 1		2	2	127	126	2	**1.30**
					129	128	2	**0.19**
					131	130	2	**0.93**
**1344**	SP100 P23497$_{1}$: Nuclear autoantigen Sp-100		5	12	127	126	3	**1.11**
					129	128	2	**1.55**
					131	130	4	**0.94**
**1345**	SPARC P09486: SPARC		17	47	127	126	46	**2.20**
					129	128	43	**1.11**
					131	130	42	**0.91**
**1346**	SPARCL1 Q14515$_{1}$: SPARC-like protein 1	1/2	37	111	127	126	103	**1.71**
					129	128	99	**1.59**
					131	130	100	**1.17**
**1347**	SPINK5 Q9NQ38$_{3}$: Serine protease inhibitor Kazal-type 5	1/3	7	49	127	126	43	**1.36**
					129	128	35	**1.23**
					131	130	40	**0.92**
**1348**	SPON1 Q9HCB6: Spondin-1		23	35	127	126	32	**1.49**
					129	128	26	**0.92**
					131	130	30	**1.22**
**1349**	SPP1 P10451$_{1-5}$: Osteopontin	5/5	4	6	127	126	6	**1.10**
					129	128	6	**1.14**
					131	130	6	**1.29**
**1350**	SPP2 Q13103: Secreted phosphoprotein 24		9	12	127	126	9	**1.06**
					129	128	8	**1.27**
					131	130	11	**1.17**
**1351**	SPR P35270: Sepiapterin reductase		3	4	127	126	4	**1.15**
					129	128	4	**1.38**
					131	130	3	**1.06**

#	protein	group	peptides	spectra	ch1	ch2	quant	ratio
**1352**	SPTAN1 Q13813$_{1-3}$: Spectrin alpha chain, non-erythrocytic 1	3/3	19	31	127	126	29	**1.66**
					129	128	25	**0.80**
					131	130	26	**1.33**
**1353**	SPTBN1 Q01082$_{1-3}$: Spectrin beta chain, non-erythrocytic 1	3/3	23	30	127	126	24	**1.20**
					129	128	22	**1.51**
					131	130	26	**1.16**
**1354**	SPTBN2 O15020$_{1,2}$: Spectrin beta chain, non-erythrocytic 2	2/2	5	18	127	126	13	**0.98**
					129	128	11	**1.16**
					131	130	14	**0.84**
**1355**	SPTBN4 Q9H254$_{1,2,4}$: Spectrin beta chain, non-erythrocytic 4	3/3	3	4	127	126	2	**0.52**
					129	128		
					131	130	1	**1.01**
**1356**	SRI P30626$_{1-3}$: Sorcin	3/3	2	3	127	126	3	**1.37**
					129	128	3	**0.85**
					131	130	3	**1.06**
**1357**	SRPX P78539$_{1}$: Sushi repeat-containing protein SRPX	1/4	15	30	127	126	8	**0.56**
					129	128	7	**3.54**
					131	130	8	**0.98**
**1358**	SRPX P78539$_{3}$: Sushi repeat-containing protein SRPX		14	23	127	126	2	**0.57**
					129	128	2	**2.34**
					131	130	2	**0.75**
**1359**	SRSF1 Q07955$_{1-3}$: Serine/arginine-rich splicing factor 1	3/3	8	8	127	126	8	**2.37**
					129	128	7	**0.42**
					131	130	8	**1.87**
**1360**	SRSF10 O75494$_{1-6}$: Serine/arginine-rich splicing factor 10	6/6	1	1	127	126	1	**2.37**
					129	128	1	**0.68**
					131	130	1	**2.06**
**1361**	SRSF2 Q01130$_{1}$: Serine/arginine-rich splicing factor 2		4	5	127	126	2	**1.16**
					129	128	2	**2.15**
					131	130	2	**1.66**
**1362**	SRSF3 P84103$_{1,2}$: Serine/arginine-rich splicing factor 3	2/2	3	3	127	126	3	**3.03**
					129	128	3	**0.58**
					131	130	3	**1.97**
**1363**	SRSF4 Q08170: Serine/arginine-rich splicing factor 4	1/4	3	4	127	126	3	**1.80**
					129	128	3	**0.82**
					131	130	3	**1.64**

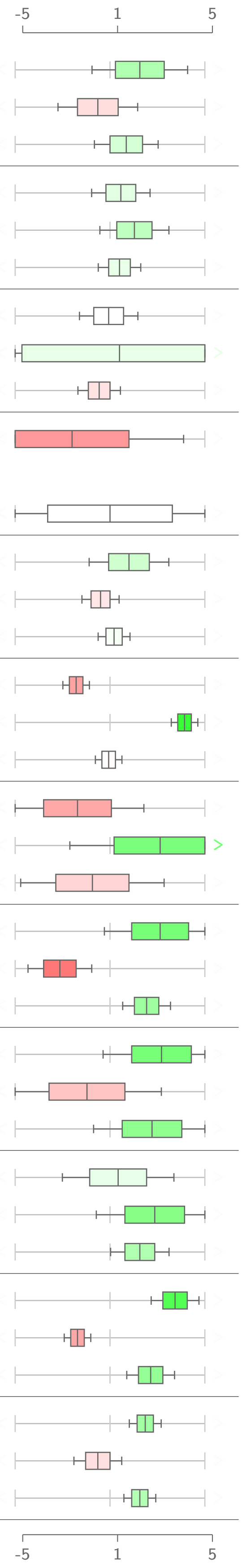

#	protein	group	peptides	spectra	ch1	ch2	quant	ratio
1364	SRSF5 Q13243$_3$: Serine/arginine-rich splicing factor 5	1/2	4	4	127	126	3	**1.11**
					129	128	3	**0.89**
					131	130	3	**1.11**
1365	SSC5D A1L4H1$_{1,2}$: Soluble scavenger receptor cysteine-rich domain-containing protein SSC5D	2/2	3	3	127	126	3	**1.21**
					129	128	3	**1.97**
					131	130	3	**1.24**
1366	ST13 P50502: Hsc70-interacting protein	1/3	10	14	127	126	14	**1.78**
					129	128	13	**0.80**
					131	130	14	**1.16**
1367	ST3GAL1 Q11201: CMP-N-acetylneuraminate-beta-galactosamide-alpha-2,3-sialyltransferase 1		1	1	127	126	1	**0.66**
					129	128	1	**1.02**
					131	130	1	**1.10**
1368	ST3GAL5 Q9UNP4$_{1-3}$: Lactosylceramide alpha-2,3-sialyltransferase	3/3	1	2	127	126	2	**1.61**
					129	128	2	**0.52**
					131	130	2	**1.18**
1369	ST6GAL1 P15907$_1$: Beta-galactoside alpha-2,6-sialyltransferase 1		2	2	127	126	2	**0.85**
					129	128	2	**1.45**
					131	130	2	**1.08**
1370	STAB1 Q9NY15$_1$: Stabilin-1	1/2	30	43	127	126	35	**0.75**
					129	128	32	**1.22**
					131	130	35	**0.99**
1371	STARD13 Q9Y3M8$_4$: StAR-related lipid transfer protein 13		3	5	127	126	3	**0.48**
					129	128	3	**1.81**
					131	130	3	**0.90**
1372	STAT3 P40763$_{1-3}$: Signal transducer and activator of transcription 3	3/3	4	4	127	126	3	**0.86**
					129	128	4	**1.08**
					131	130	4	**1.02**
1373	STC2 O76061: Stanniocalcin-2		1	1	127	126	1	**1.54**
					129	128	1	**1.20**
					131	130	1	**1.03**
1374	STIP1 P31948$_{1-3}$: Stress-induced-phosphoprotein 1	3/3	2	2	127	126	2	**2.86**
					129	128	2	**0.54**
					131	130	2	**2.40**
1375	SUB1 P53999: Activated RNA polymerase II transcriptional coactivator p15		3	3	127	126	3	**2.31**
					129	128	3	**0.83**
					131	130	3	**1.65**

#	protein	group	peptides	spectra	ch1	ch2	quant	ratio
**1376**	SULF1 Q8IWU6: Extracellular sulfatase Sulf-1		9	9	127	126	8	**1.15**
					129	128	8	**1.91**
					131	130	8	**1.58**
**1377**	SULT1A2 P50226: Sulfotransferase 1A2	1/3	5	6	127	126	1	**1.36**
					129	128	1	**1.45**
					131	130	1	**1.00**
**1378**	SULT1A3 P0DMM9$_{1,3}$, SULT1A4 P0DMN0: Sulfotransferase 1A3, Sulfotransferase 1A4	3/4	8	9	127	126	4	**0.78**
					129	128	4	**0.86**
					131	130	4	**1.00**
**1379**	SUSD5 O60279: Sushi domain-containing protein 5		5	10	127	126	8	**1.37**
					129	128	7	**1.38**
					131	130	9	**1.01**
**1380**	SVEP1 Q4LDE5$_{1}$: Sushi, von Willebrand factor type A, EGF and pentraxin domain-containing pro ...	1/4	88	140	127	126	134	**0.93**
					129	128	130	**1.51**
					131	130	130	**1.31**
**1381**	SWAP70 Q9UH65: Switch-associated protein 70		2	3	127	126	3	**0.94**
					129	128	3	**1.53**
					131	130	3	**1.21**
**1382**	SYNCRIP O60506$_{1,3}$: Heterogeneous nuclear ribonucleoprotein Q	2/5	10	13	127	126	6	**1.64**
					129	128	5	**0.58**
					131	130	6	**1.45**
**1383**	TAF15 Q92804$_{1,2}$: TATA-binding protein-associated factor 2N	2/2	7	8	127	126	4	**2.90**
					129	128	4	**0.58**
					131	130	5	**1.89**
**1384**	TAGLN Q01995: Transgelin		11	17	127	126	13	**0.99**
					129	128	15	**1.28**
					131	130	15	**1.33**
**1385**	TAGLN2 P37802$_{1,2}$: Transgelin-2	2/2	12	21	127	126	21	**1.18**
					129	128	21	**0.99**
					131	130	21	**1.01**
**1386**	TBCB Q99426$_{1,2}$: Tubulin-folding cofactor B	2/2	3	3	127	126	3	**0.93**
					129	128	3	**0.89**
					131	130	3	**1.24**
**1387**	TCHH Q07283: Trichohyalin		13	15	127	126	12	**0.79**
					129	128	11	**3.64**
					131	130	12	**0.85**

#	protein	group	peptides	spectra	ch1	ch2	quant	ratio
**1388**	TCN2 P20062$_1$: Transcobalamin-2	1/2	10	14	127	126	14	**0.87**
					129	128	11	**0.48**
					131	130	14	**1.02**
**1389**	TCP1 P17987: T-complex protein 1 subunit alpha		3	4	127	126	4	**1.14**
					129	128	4	**0.82**
					131	130	3	**1.13**
**1390**	TDRD1 Q9BXT4$_{1-4}$: Tudor domain-containing protein 1	4/4	2	7	127	126	5	**0.88**
					129	128	3	**0.46**
					131	130	4	**1.17**
**1391**	TDRD15 B5MCY1: Tudor domain-containing protein 15		5	63	127	126	36	**1.46**
					129	128	21	**1.24**
					131	130	37	**0.98**
**1392**	TF P02787: Serotransferrin		38	71	127	126	69	**1.47**
					129	128	64	**0.69**
					131	130	68	**1.13**
**1393**	TFG Q92734$_{1-4}$: Protein TFG	4/4	3	3	127	126	2	**2.13**
					129	128	2	**0.63**
					131	130	2	**1.87**
**1394**	TFPI P10646$_1$: Tissue factor pathway inhibitor		4	5	127	126	2	**0.94**
					129	128	2	**1.21**
					131	130	3	**1.21**
**1395**	TFPI2 P48307$_{1,2}$: Tissue factor pathway inhibitor 2	2/2	2	2	127	126	2	**1.55**
					129	128	2	**1.56**
					131	130	2	**1.37**
**1396**	TGFB2 P61812$_{1,2}$: Transforming growth factor beta-2	2/2	3	3	127	126	3	**0.53**
					129	128	3	**2.39**
					131	130	3	**0.89**
**1397**	TGFBI Q15582: Transforming growth factor-beta-induced protein ig-h3		30	49	127	126	49	**0.69**
					129	128	46	**1.44**
					131	130	44	**1.06**
**1398**	TGFBR3 Q03167$_{1,2}$: Transforming growth factor beta receptor type 3	2/2	13	20	127	126	19	**0.50**
					129	128	20	**1.71**
					131	130	19	**0.95**
**1399**	TGM1 P22735$_1$: Protein-glutamine gamma-glutamyltransferase K	1/2	6	6	127	126	6	**0.59**
					129	128	5	**3.09**
					131	130	5	**1.09**

#	protein	group	peptides	spectra	ch1	ch2	quant	ratio
**1400**	TGM2 P21980$_1$: Protein-glutamine gamma-glutamyltransferase 2	1/2	11	16	127	126	11	**1.54**
					129	128	12	**0.63**
					131	130	10	**1.11**
**1401**	TGM3 Q08188: Protein-glutamine gamma-glutamyltransferase E		2	2	127	126	2	**0.58**
					129	128	2	**4.17**
					131	130	2	**0.61**
**1402**	TGM4 P49221: Protein-glutamine gamma-glutamyltransferase 4		2	11	127	126	4	**1.15**
					129	128	3	**1.50**
					131	130	6	**1.00**
**1403**	TGOLN2 O43493$_{1-6}$: Trans-Golgi network integral membrane protein 2	6/6	2	2	127	126	2	**1.73**
					129	128	1	**1.04**
					131	130	2	**1.31**
**1404**	THBS1 P07996$_1$: Thrombospondin-1	1/2	41	84	127	126	62	**0.98**
					129	128	57	**1.19**
					131	130	63	**1.14**
**1405**	THBS2 P35442: Thrombospondin-2		23	41	127	126	37	**1.52**
					129	128	36	**1.12**
					131	130	35	**1.12**
**1406**	THBS3 P49746$_1$: Thrombospondin-3	1/2	11	15	127	126	13	**0.65**
					129	128	11	**0.98**
					131	130	12	**1.06**
**1407**	THBS4 P35443: Thrombospondin-4		21	53	127	126	44	**0.65**
					129	128	39	**1.23**
					131	130	45	**0.82**
**1408**	THSD4 Q6ZMP0$_{1,4}$: Thrombospondin type-1 domain-containing protein 4	2/2	8	14	127	126	14	**0.94**
					129	128	10	**1.06**
					131	130	10	**1.06**
**1409**	TIE1 P35590$_1$: Tyrosine-protein kinase receptor Tie-1		4	4	127	126	4	**0.47**
					129	128	3	**1.68**
					131	130	3	**0.85**
**1410**	TIMP1 P01033: Metalloproteinase inhibitor 1		5	10	127	126	7	**1.18**
					129	128	10	**1.28**
					131	130	10	**1.61**
**1411**	TIMP2 P16035: Metalloproteinase inhibitor 2		10	27	127	126	21	**1.31**
					129	128	22	**2.44**
					131	130	19	**1.05**

#	protein	group	peptides	spectra	ch1	ch2	quant	ratio
**1412**	TIMP4 Q99727: Metalloproteinase inhibitor 4		2	2	127	126	2	**1.93**
					129	128	2	**1.82**
					131	130	2	**1.41**
**1413**	TINAGL1 Q9GZM7$_1$: Tubulointerstitial nephritis antigen-like	1/2	6	15	127	126	11	**0.96**
					129	128	9	**2.24**
					131	130	12	**1.04**
**1414**	TJP1 Q07157$_{1,2}$: Tight junction protein ZO-1	2/2	5	6	127	126	6	**1.41**
					129	128	5	**1.04**
					131	130	6	**1.15**
**1415**	TKFC Q3LXA3$_{1,2}$: Triokinase/FMN cyclase	2/2	1	1	127	126	1	**0.31**
					129	128	1	**2.56**
					131	130	1	**1.18**
**1416**	TLL1 O43897$_1$: Tolloid-like protein 1	1/2	7	10	127	126	6	**0.88**
					129	128	5	**1.03**
					131	130	6	**1.05**
**1417**	TLL2 Q9Y6L7: Tolloid-like protein 2		6	6	127	126	1	**1.22**
					129	128	1	**0.98**
					131	130	1	**1.02**
**1418**	TLN1 Q9Y490: Talin-1		54	71	127	126	50	**0.93**
					129	128	46	**2.23**
					131	130	47	**1.03**
**1419**	TLN2 Q9Y4G6: Talin-2		18	20	127	126	5	**0.87**
					129	128	5	**1.23**
					131	130	5	**0.96**
**1420**	TMED10 P49755: Transmembrane emp24 domain-containing protein 10		4	4	127	126	3	**1.54**
					129	128	3	**0.93**
					131	130	4	**1.48**
**1421**	TMED9 Q9BVK6: Transmembrane emp24 domain-containing protein 9		3	3	127	126	3	**2.30**
					129	128	3	**0.83**
					131	130	3	**1.34**
**1422**	TMEM43 Q9BTV4: Transmembrane protein 43		2	2	127	126	2	**1.61**
					129	128	2	**0.99**
					131	130	2	**1.59**
**1423**	TMEM59 Q9BXS4: Transmembrane protein 59		1	1	127	126	1	**1.01**
					129	128	1	**1.42**
					131	130	1	**1.04**

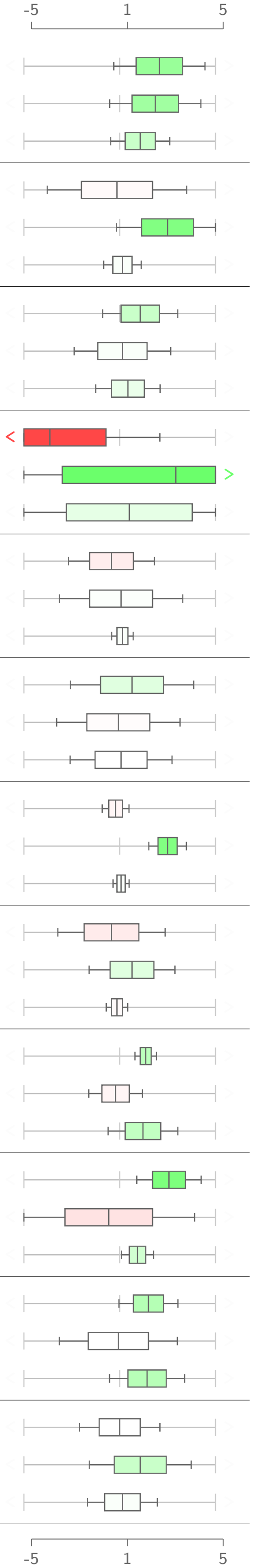

#	protein	group	peptides	spectra	ch1	ch2	quant	ratio
**1424**	TMOD3 Q9NYL9: Tropomodulin-3		2	2	127	126	2	**2.15**
					129	128	2	**0.67**
					131	130	2	**1.29**
**1425**	TNC $P24821_{1,4}$: Tenascin	2/6	40	60	127	126	49	**1.07**
					129	128	45	**1.06**
					131	130	51	**1.13**
**1426**	TNFRSF11B O00300: Tumor necrosis factor receptor superfamily member 11B		4	4	127	126	4	**1.43**
					129	128	3	**1.10**
					131	130	4	**1.17**
**1427**	TNFSF12 $O43508_{2}$, TNFSF13 $O75888_{1,3}$: Tumor necrosis factor ligand superfamily member 12, Tumor necrosis factor ligand superfamily member 13	3/6	3	3	127	126	2	**1.40**
					129	128	2	**0.42**
					131	130	2	**1.06**
**1428**	TNKS1BP1 $Q9C0C2_{1}$: 182 kDa tankyrase-1-binding protein	1/2	7	7	127	126	5	**2.09**
					129	128	6	**0.85**
					131	130	6	**1.20**
**1429**	TNNT1 $P13805_{1-3}$: Troponin T, slow skeletal muscle	3/3	4	11	127	126	10	**1.57**
					129	128	10	**1.73**
					131	130	10	**1.07**
**1430**	TNNT2 $P45379_{3,4}$: Troponin T, cardiac muscle	2/2	2	2	127	126	1	**1.13**
					129	128		
					131	130	1	**1.27**
**1431**	TNPO1 $Q92973_{1-3}$: Transportin-1	3/3	2	2	127	126	2	**1.36**
					129	128	2	**0.98**
					131	130	2	**1.19**
**1432**	TNXB $P22105_{1,3}$: Tenascin-X	2/5	97	149	127	126	136	**1.34**
					129	128	138	**1.44**
					131	130	135	**1.19**
**1433**	TOR1AIP1 $Q5JTV8_{1,3}$: Torsin-1A-interacting protein 1	2/2	3	3	127	126	3	**1.80**
					129	128	3	**0.91**
					131	130	3	**1.30**
**1434**	TOR1B O14657: Torsin-1B		1	1	127	126	1	**0.74**
					129	128	1	**1.42**
					131	130	1	**0.96**
**1438**	TPM1 $P09493_{5}$: Tropomyosin alpha-1 chain		13	20	127	126	1	**1.92**
					129	128	1	**0.80**
					131	130	1	**0.93**

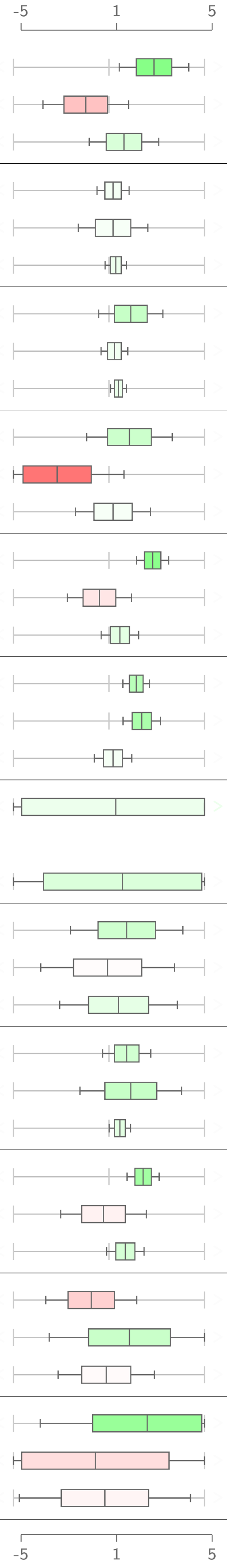

#	protein	group	peptides	spectra	ch1	ch2	quant	ratio
**1443**	TPM2 P07951$_1$: Tropomyosin beta chain		21	37	127	126	3	**1.73**
					129	128	3	**2.67**
					131	130	3	**0.78**
**1444**	TPM2 P07951$_2$: Tropomyosin beta chain	1/2	21	39	127	126	5	**2.28**
					129	128	6	**1.41**
					131	130	4	**1.20**
**1445**	TPM3 P06753$_1$: Tropomyosin alpha-3 chain		18	34	127	126	1	**1.85**
					129	128	1	**0.67**
					131	130	1	**1.14**
**1446**	TPM3 P06753$_2$: Tropomyosin alpha-3 chain	1/3	22	37	127	126	6	**1.50**
					129	128	6	**1.51**
					131	130	6	**1.19**
**1448**	TPM4 P67936$_1$: Tropomyosin alpha-4 chain		21	36	127	126	6	**2.04**
					129	128	7	**1.44**
					131	130	6	**1.18**
**1450**	TPP1 O14773$_{1,2}$: Tripeptidyl-peptidase 1	2/2	2	2	127	126	2	**1.43**
					129	128	2	**1.97**
					131	130	2	**0.96**
**1451**	TRIM28 Q13263$_1$: Transcription intermediary factor 1-beta	1/2	3	3	127	126	3	**2.00**
					129	128	3	**0.71**
					131	130	3	**1.42**
**1452**	TRIM3 O75382$_4$: Tripartite motif-containing protein 3	1/4	5	29	127	126	3	**0.89**
					129	128	3	**1.22**
					131	130	3	**1.17**
**1453**	TRIP6 Q15654$_1$: Thyroid receptor-interacting protein 6		1	1	127	126	1	**1.10**
					129	128	1	**0.75**
					131	130	1	**0.99**
**1454**	TROVE2 P10155$_{1-5}$: 60 kDa SS-A/Ro ribonucleoprotein	5/5	2	3	127	126	3	**1.72**
					129	128	3	**0.76**
					131	130	3	**1.39**
**1455**	TSKU Q8WUA8: Tsukushin		10	14	127	126	14	**0.77**
					129	128	14	**1.02**
					131	130	14	**0.86**
**1456**	TSTA3 Q13630: GDP-L-fucose synthase		2	21	127	126	19	**1.20**
					129	128	20	**1.43**
					131	130	21	**1.03**

#	protein	group	peptides	spectra	ch1	ch2	quant	ratio
**1457**	TTC39A Q5SRH9$_{1,4-6}$: Tetratricopeptide repeat protein 39A	4/4	2	9	127	126	1	**1.08**
					129	128		
					131	130	1	**0.59**
**1458**	TTN Q8WZ42$_{12}$: Titin	1/12	28	46	127	126	25	**0.93**
					129	128	17	**0.67**
					131	130	28	**0.87**
**1459**	TTN Q8WZ42$_{6}$: Titin		6	6	127	126	3	**1.03**
					129	128	3	**1.54**
					131	130	3	**0.89**
**1460**	TTR P02766: Transthyretin		11	112	127	126	109	**1.53**
					129	128	101	**1.19**
					131	130	109	**0.84**
**1465**	TUBA4A P68366$_{1,2}$: Tubulin alpha-4A chain	2/4	16	42	127	126	1	**1.40**
					129	128	1	**0.89**
					131	130	1	**1.19**
**1466**	TUBA8 Q9NY65$_{1,2}$: Tubulin alpha-8 chain	2/2	12	28	127	126	1	**1.59**
					129	128	1	**0.58**
					131	130	1	**1.13**
**1467**	TUBB P07437: Tubulin beta chain	1/4	17	47	127	126	4	**1.24**
					129	128	4	**0.62**
					131	130	3	**1.37**
**1468**	TUBB1 Q9H4B7: Tubulin beta-1 chain		5	9	127	126	1	**0.78**
					129	128	1	**0.69**
					131	130	1	**0.99**
**1470**	TUBB4A P04350: Tubulin beta-4A chain	1/2	14	37	127	126	1	**1.49**
					129	128	1	**0.72**
					131	130	1	**1.11**
**1471**	TUBB4B P68371: Tubulin beta-4B chain	1/3	17	47	127	126	1	**1.89**
					129	128	1	**0.34**
					131	130	1	**1.34**
**1472**	TUFM P49411: Elongation factor Tu, mitochondrial		2	2	127	126	2	**1.07**
					129	128	2	**1.13**
					131	130	2	**1.84**
**1473**	TWF2 Q6IBS0: Twinfilin-2		2	2	127	126	2	**0.76**
					129	128	2	**1.90**
					131	130	2	**0.90**

#	protein	group	peptides	spectra	ch1	ch2	quant	ratio
**1474**	TWSG1 Q9GZX9$_1$: Twisted gastrulation protein homolog 1	1/2	5	6	127	126	2	**1.78**
					129	128	2	**1.70**
					131	130	3	**1.13**
**1475**	TXN P10599$_{1,2}$: Thioredoxin	2/2	2	2	127	126	2	**0.72**
					129	128	2	**2.09**
					131	130	2	**0.88**
**1476**	TXNDC5 Q8NBS9$_1$: Thioredoxin domain-containing protein 5	1/2	8	9	127	126	6	**1.03**
					129	128	6	**1.04**
					131	130	6	**1.06**
**1477**	TXNL1 O43396: Thioredoxin-like protein 1		1	1	127	126	1	**1.01**
					129	128	1	**0.69**
					131	130	1	**0.99**
**1478**	TYMP P19971$_1$: Thymidine phosphorylase	1/2	5	7	127	126	5	**0.67**
					129	128	5	**1.47**
					131	130	6	**0.85**
**1479**	UBA1 P22314$_{1,2}$: Ubiquitin-like modifier-activating enzyme 1	2/2	18	21	127	126	18	**1.18**
					129	128	18	**0.99**
					131	130	20	**1.08**
**1480**	UBA2 Q9UBT2$_{1,2}$: SUMO-activating enzyme subunit 2	2/2	3	3	127	126	3	**2.08**
					129	128	3	**0.88**
					131	130	3	**1.21**
**1481**	UBE2K P61086$_{1,3}$: Ubiquitin-conjugating enzyme E2 K	2/2	3	4	127	126	3	**0.58**
					129	128	4	**2.11**
					131	130	3	**0.93**
**1482**	UBE2M P61081: NEDD8-conjugating enzyme Ubc12		2	2	127	126	2	**1.07**
					129	128	2	**1.06**
					131	130	2	**0.95**
**1483**	UBE2N P61088, UBE2NL Q5JXB2: Ubiquitin-conjugating enzyme E2 N, Putative ubiquitin-conjugating enzyme E2 N-like	2/2	3	4	127	126	3	**0.88**
					129	128	4	**1.58**
					131	130	4	**1.15**
**1484**	UCHL3 P15374: Ubiquitin carboxyl-terminal hydrolase isozyme L3		2	3	127	126	3	**1.61**
					129	128	3	**1.04**
					131	130	3	**1.10**
**1485**	UGDH O60701$_1$: UDP-glucose 6-dehydrogenase	1/3	10	11	127	126	10	**1.36**
					129	128	10	**0.95**
					131	130	10	**0.97**

#	protein	group	peptides	spectra	ch1	ch2	quant	ratio
**1486**	UGGT1 Q9NYU2$_{1,2}$: UDP-glucose:glycoprotein glucosyltransferase 1	2/2	1	1	127	126	1	**0.94**
					129	128	1	**0.87**
					131	130	1	**1.02**
**1487**	UGP2 Q16851$_{2}$: UTP--glucose-1-phosphate uridylyltransferase	1/2	11	13	127	126	13	**0.75**
					129	128	13	**0.99**
					131	130	13	**0.98**
**1488**	UQCRC2 P22695: Cytochrome b-c1 complex subunit 2, mitochondrial		3	5	127	126	5	**0.35**
					129	128	5	**4.58**
					131	130	3	**0.68**
**1489**	UQCRH P07919: Cytochrome b-c1 complex subunit 6, mitochondrial		2	2	127	126	2	**1.87**
					129	128	2	**1.32**
					131	130	2	**1.23**
**1490**	USO1 O60763$_{1,2}$: General vesicular transport factor p115	2/2	11	12	127	126	12	**1.09**
					129	128	10	**1.87**
					131	130	10	**1.11**
**1491**	USP14 P54578$_{1-3}$: Ubiquitin carboxyl-terminal hydrolase 14	3/3	1	3	127	126	3	**1.03**
					129	128	3	**1.05**
					131	130	3	**1.17**
**1492**	USP5 P45974$_{1,2}$: Ubiquitin carboxyl-terminal hydrolase 5	2/2	4	4	127	126	3	**1.78**
					129	128	4	**0.79**
					131	130	4	**1.26**
**1493**	USP9X Q93008$_{1,3}$: Probable ubiquitin carboxyl-terminal hydrolase FAF-X	2/2	8	42	127	126	9	**1.26**
					129	128	5	**0.70**
					131	130	6	**1.06**
**1494**	USP9Y O00507$_{1,2}$: Probable ubiquitin carboxyl-terminal hydrolase FAF-Y	2/2	9	40	127	126	2	**0.96**
					129	128	1	**0.54**
					131	130	1	**0.97**
**1495**	VARS P26640$_{1}$: Valine--tRNA ligase		3	3	127	126	3	**1.02**
					129	128	3	**1.46**
					131	130	3	**0.97**
**1496**	VASP P50552: Vasodilator-stimulated phosphoprotein		1	1	127	126	1	**0.79**
					129	128	1	**1.96**
					131	130	1	**1.04**
**1497**	VAT1 Q99536$_{1}$: Synaptic vesicle membrane protein VAT-1 homolog	1/3	7	7	127	126	6	**0.54**
					129	128	6	**1.82**
					131	130	7	**1.03**

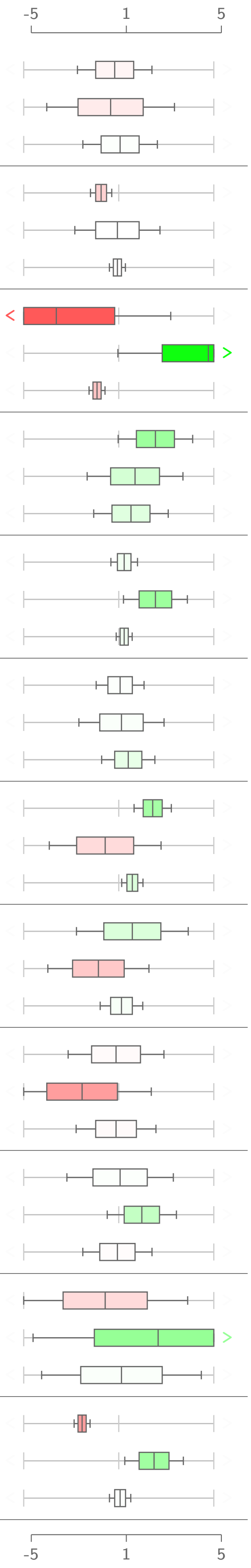

#	protein	group	peptides	spectra	ch1	ch2	quant	ratio
**1498**	VCAM1 P19320$_{1,3}$: Vascular cell adhesion protein 1	2/3	15	21	127	126	18	**0.75**
					129	128	18	**1.65**
					131	130	20	**1.16**
**1499**	VCL P18206$_{1,2}$: Vinculin	2/3	35	42	127	126	38	**0.40**
					129	128	42	**3.01**
					131	130	38	**1.11**
**1500**	VCP P55072: Transitional endoplasmic reticulum ATPase		14	19	127	126	18	**2.02**
					129	128	16	**0.51**
					131	130	16	**1.25**
**1501**	VDAC1 P21796: Voltage-dependent anion-selective channel protein 1		2	2	127	126	2	**1.99**
					129	128	2	**0.67**
					131	130	2	**1.79**
**1502**	VEGFC P49767: Vascular endothelial growth factor C		17	34	127	126	34	**1.49**
					129	128	31	**1.78**
					131	130	34	**1.06**
**1503**	VGF O15240: Neurosecretory protein VGF [Cleaved into: Neuroendocrine regulatory peptide-1		13	20	127	126	19	**1.20**
					129	128	19	**2.08**
					131	130	19	**1.04**
**1504**	VIM P08670: Vimentin		36	128	127	126	78	**2.64**
					129	128	75	**0.55**
					131	130	73	**1.11**
**1505**	VLDLR P98155$_{1,2}$: Very low-density lipoprotein receptor	2/2	7	13	127	126	9	**1.21**
					129	128	7	**0.91**
					131	130	9	**1.01**
**1506**	VMO1 Q7Z5L0$_{1}$: Vitelline membrane outer layer protein 1 homolog		1	1	127	126	1	**1.62**
					129	128	1	**1.58**
					131	130	1	**1.02**
**1507**	VPS35 Q96QK1: Vacuolar protein sorting-associated protein 35		7	8	127	126	7	**1.06**
					129	128	7	**1.18**
					131	130	8	**0.98**
**1508**	VPS4B O75351: Vacuolar protein sorting-associated protein 4B		2	2	127	126	2	**1.49**
					129	128	1	**0.92**
					131	130	1	**1.27**
**1509**	VSIG4 Q9Y279$_{1-3}$: V-set and immunoglobulin domain-containing protein 4	3/3	1	1	127	126	1	**0.65**
					129	128	1	**0.62**
					131	130	1	**1.20**

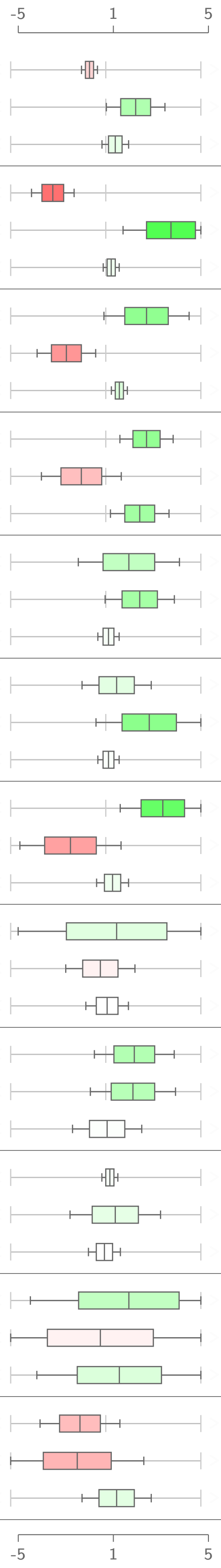

#	protein	group	peptides	spectra	ch1	ch2	quant	ratio
1510	VTN P04004: Vitronectin		19	1191	127	126	1141	**1.40**
					129	128	1013	**0.72**
					131	130	1112	**1.12**
1511	VWA1 Q6PCB0$_1$: von Willebrand factor A domain-containing protein 1	1/2	5	5	127	126	5	**0.88**
					129	128	5	**1.49**
					131	130	4	**1.07**
1512	VWF P04275$_1$: von Willebrand factor	1/2	76	113	127	126	108	**0.27**
					129	128	81	**0.74**
					131	130	107	**0.94**
1513	WARS P23381$_{1,2}$: Tryptophan--tRNA ligase, cytoplasmic	2/2	5	5	127	126	5	**0.72**
					129	128	5	**1.80**
					131	130	5	**0.95**
1514	WDFY1 Q8IWB7: WD repeat and FYVE domain-containing protein 1		2	2	127	126	1	**1.15**
					129	128	1	**0.94**
					131	130	1	**1.23**
1515	WDR1 O75083$_{1,3}$: WD repeat-containing protein 1	2/2	3	3	127	126	3	**1.44**
					129	128	3	**0.90**
					131	130	3	**1.11**
1516	WFDC1 Q9HC57: WAP four-disulfide core domain protein 1		2	5	127	126	5	**0.60**
					129	128	5	**4.91**
					131	130	5	**0.90**
1517	WISP2 O76076$_1$: WNT1-inducible-signaling pathway protein 2	1/2	11	84	127	126	77	**1.65**
					129	128	69	**1.52**
					131	130	76	**0.92**
1518	WNK1 Q9H4A3$_7$: Serine/threonine-protein kinase WNK1	1/6	3	4	127	126	4	**0.61**
					129	128	3	**2.16**
					131	130	4	**0.93**
1519	WNT5A P41221$_{1,2}$: Protein Wnt-5a	2/2	7	31	127	126	28	**0.94**
					129	128	24	**0.67**
					131	130	28	**0.98**
1520	WNT5B Q9H1J7: Protein Wnt-5b		3	7	127	126	6	**1.16**
					129	128	4	**0.68**
					131	130	3	**0.90**
1521	XPO1 O14980: Exportin-1		3	4	127	126	4	**0.94**
					129	128	4	**1.64**
					131	130	3	**1.08**

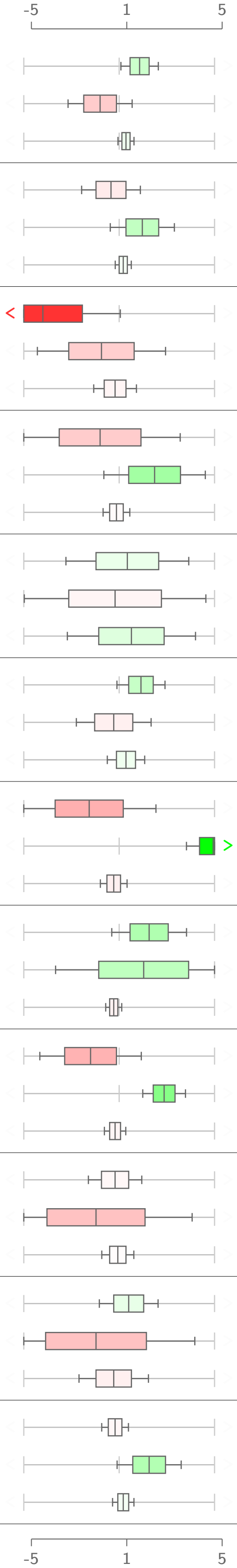

#	protein	group	peptides	spectra	ch1	ch2	quant	ratio
**1522**	XRCC5 P13010: X-ray repair cross-complementing protein 5		7	9	127	126	8	**1.97**
					129	128	8	**0.60**
					131	130	9	**1.50**
**1523**	XRCC6 P12956$_{1,2}$: X-ray repair cross-complementing protein 6	2/2	10	10	127	126	10	**1.35**
					129	128	9	**0.94**
					131	130	10	**1.48**
**1524**	XXYLT1 Q8NBI6$_{1}$: Xyloside xylosyltransferase 1		3	3	127	126	3	**0.57**
					129	128	3	**1.30**
					131	130	3	**0.89**
**1525**	YARS P54577: Tyrosine--tRNA ligase, cytoplasmic		3	4	127	126	3	**1.26**
					129	128	4	**0.92**
					131	130	3	**1.06**
**1526**	YKT6 O15498$_{1}$: Synaptobrevin homolog YKT6	1/2	6	8	127	126	7	**0.73**
					129	128	7	**2.16**
					131	130	8	**0.90**
**1527**	YWHAB P31946$_{1,2}$: 14-3-3 protein beta/alpha	2/2	14	28	127	126	5	**2.13**
					129	128	4	**0.77**
					131	130	5	**1.32**
**1528**	YWHAE P62258$_{1}$: 14-3-3 protein epsilon	1/2	12	28	127	126	18	**2.29**
					129	128	17	**0.68**
					131	130	17	**1.26**
**1529**	YWHAG P61981: 14-3-3 protein gamma		15	30	127	126	15	**1.52**
					129	128	11	**0.69**
					131	130	15	**1.14**
**1530**	YWHAH Q04917: 14-3-3 protein eta		13	24	127	126	14	**2.10**
					129	128	10	**0.62**
					131	130	14	**1.26**
**1531**	YWHAQ P27348: 14-3-3 protein theta		16	35	127	126	17	**2.01**
					129	128	16	**0.71**
					131	130	18	**1.16**
**1532**	YWHAZ P63104$_{1}$: 14-3-3 protein zeta/delta	1/2	18	44	127	126	21	**2.20**
					129	128	22	**0.84**
					131	130	24	**1.32**
**1533**	ZBTB6 Q15916: Zinc finger and BTB domain-containing protein 6		3	18	127	126	11	**1.02**
					129	128	7	**0.91**
					131	130	11	**1.18**

#	protein	group	peptides	spectra	ch1	ch2	quant	ratio
**1534**	ZC3H6 P61129: Zinc finger CCCH domain-containing protein 6		3	94	127	126	2	**0.71**
					129	128	1	**1.46**
					131	130	2	**0.96**
**1535**	ZFAT Q9P243$_{1,2,4}$: Zinc finger protein ZFAT	3/3	9	37	127	126	28	**1.33**
					129	128	23	**1.16**
					131	130	28	**1.08**
**1536**	ZFYVE26 Q68DK2$_{1,2}$: Zinc finger FYVE domain-containing protein 26	2/2	3	16	127	126	9	**1.30**
					129	128	4	**0.73**
					131	130	7	**1.10**
**1537**	ZNF236 Q9UL36$_{1}$: Zinc finger protein 236	1/2	5	23	127	126	19	**0.76**
					129	128	15	**1.80**
					131	130	20	**1.04**
**1538**	ZNF318 Q5VUA4$_{1}$: Zinc finger protein 318		2	2	127	126	2	**3.02**
					129	128	2	**0.63**
					131	130	2	**1.10**
**1539**	ZNF418 Q8TF45: Zinc finger protein 418		6	14	127	126	8	**0.91**
					129	128	4	**0.94**
					131	130	8	**0.99**
**1540**	ZNF428 Q96B54: Zinc finger protein 428		1	1	127	126	1	**2.73**
					129	128	1	**1.88**
					131	130	1	**1.22**
**1541**	ZNF462 Q96JM2$_{1,3}$: Zinc finger protein 462	2/2	7	23	127	126	21	**1.25**
					129	128	17	**0.50**
					131	130	19	**0.89**
**1542**	ZNF512 Q96ME7$_{1-3}$: Zinc finger protein 512	3/3	3	14	127	126	4	**0.99**
					129	128	2	**1.09**
					131	130	5	**0.83**
**1543**	ZNF565 Q8N9K5$_{1,2}$: Zinc finger protein 565	2/2	4	23	127	126	21	**0.72**
					129	128	17	**1.41**
					131	130	19	**0.98**
**1544**	ZYX Q15942$_{1,2}$: Zyxin	2/2	2	2	127	126	2	**1.57**
					129	128	2	**1.47**
					131	130	2	**1.16**
**1545**	ZZEF1 O43149$_{1,2}$: Zinc finger ZZ-type and EF-hand domain-containing protein 1	2/2	6	57	127	126	16	**1.06**
					129	128	9	**0.85**
					131	130	23	**1.02**

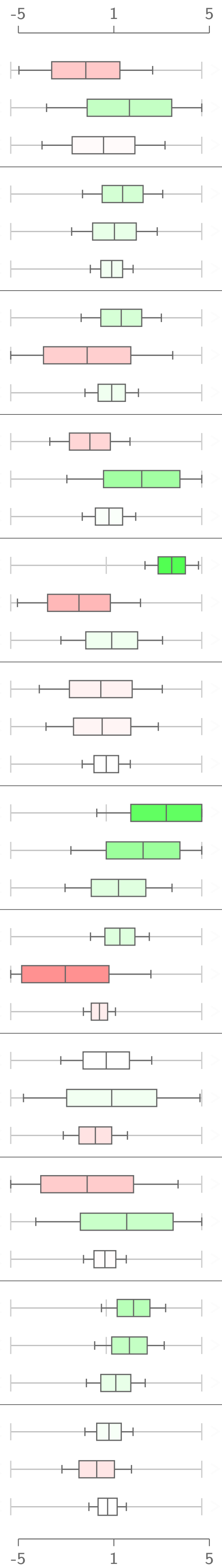

1.2 Not Quantified Proteins

Number of identified but not quantified proteins: P10-treat/P10-ctrl: 68; P8-treat/P8-ctrl: 68; P9-treat/P9-ctrl: 78

#	protein	group	peptides	spectra
724	IGHV1-46 P01743, A0A0C4DH29: Immunoglobulin heavy variable 1-46	2/2	2	6
616	HIST1H2BC P62807, HIST1H2BD P58876, HIST1H2BH Q93079, ...: Histone H2B type 1-C/E/F/G/I, Histone H2B type 1-D, ...	7/9	12	41
501	FCN3 O75636$_{2}$: Ficolin-3		8	14
824	KRT75 O95678: Keratin, type II cytoskeletal 75		16	57
728	IGHV3-33 P01772: Immunoglobulin heavy variable 3-33		2	6
633	HLA-B P01889, HLA-B P30460, HLA-B P30480: HLA class I histocompatibility antigen, B-7 alpha chain, HLA class I histocompatibility antigen, B-8 alpha chain, ...	3/5	8	13
641	HLA-C P04222: HLA class I histocompatibility antigen, Cw-3 alpha chain		7	14
819	KRT6B P04259: Keratin, type II cytoskeletal 6B		32	70
1447	TPM3 P06753$_{5}$: Tropomyosin alpha-3 chain	1/3	21	38
615	HIST1H2BB P33778, HIST1H2BJ P06899, HIST1H2BO P23527, ...: Histone H2B type 1-B, Histone H2B type 1-J, ...	4/6	12	39
582	GSTM1 P09488$_{1,2}$: Glutathione S-transferase Mu 1	2/5	4	4
1435	TPM1 P09493$_{1}$: Tropomyosin alpha-1 chain		20	30
1436	TPM1 P09493$_{4,10}$: Tropomyosin alpha-1 chain	2/2	19	30
1437	TPM1 P09493$_{3}$: Tropomyosin alpha-1 chain	1/2	19	30
1439	TPM1 P09493$_{6}$: Tropomyosin alpha-1 chain		18	29
1440	TPM1 P09493$_{7}$: Tropomyosin alpha-1 chain		18	29
1441	TPM1 P09493$_{8}$: Tropomyosin alpha-1 chain		17	29
1442	TPM1 P09493$_{9}$: Tropomyosin alpha-1 chain		20	30
624	HLA-A P10314, HLA-A P30459: HLA class I histocompatibility antigen, A-32 alpha chain, HLA class I histocompatibility antigen, A-74 alpha chain	2/2	6	8
813	KRT3 P12035: Keratin, type II cytoskeletal 3		11	33
973	NEFH P12036$_{1,2}$: Neurofilament heavy polypeptide	2/2	3	9
626	HLA-A P13746$_{1,2}$: HLA class I histocompatibility antigen, A-11 alpha chain	2/3	6	7
627	HLA-A P16188: HLA class I histocompatibility antigen, A-30 alpha chain		6	8
628	HLA-A P16190: HLA class I histocompatibility antigen, A-33 alpha chain		6	10
692	HSPA6 P17066: Heat shock 70 kDa protein 6		6	12
583	GSTM2 P28161$_{1}$: Glutathione S-transferase Mu 2	1/2	3	3
629	HLA-A P30443, HLA-A P30455: HLA class I histocompatibility antigen, A-1 alpha chain, HLA class I histocompatibility antigen, A-36 alpha chain	2/2	6	7
630	HLA-A P30447: HLA class I histocompatibility antigen, A-23 alpha chain		8	10
636	HLA-B P30464, HLA-B P30484: HLA class I histocompatibility antigen, B-15 alpha chain, HLA class I histocompatibility antigen, B-46 alpha chain	2/10	10	19
637	HLA-B P30479: HLA class I histocompatibility antigen, B-41 alpha chain	1/5	9	16
638	HLA-B P30487, HLA-B P30488: HLA class I histocompatibility antigen, B-49 alpha chain, HLA class I histocompatibility antigen, B-50 alpha chain	2/3	8	13
639	HLA-B P30491, HLA-B P30685: HLA class I histocompatibility antigen, B-53 alpha chain, HLA class I histocompatibility antigen, B-35 alpha chain	2/2	9	16
632	HLA-A P30512: HLA class I histocompatibility antigen, A-29 alpha chain	1/2	6	8
18	ACTN2 P35609$_{2}$: Alpha-actinin-2	1/2	6	7
808	KRT20 P35900: Keratin, type I cytoskeletal 20		5	17
1135	PRPH P41219$_{1,2}$: Peripherin	2/2	5	29
585	GSTM5 P46439: Glutathione S-transferase Mu 5		3	3
820	KRT6C P48668: Keratin, type II cytoskeletal 6C		32	72

#	protein	group	peptides	spectra
1449	TPM4 P67936$_2$: Tropomyosin alpha-4 chain		18	34
1462	TUBA1B P68363$_1$: Tubulin alpha-1B chain	1/2	18	48
825	KRT76 Q01546: Keratin, type II cytoskeletal 2 oral		12	40
419	DST Q03001$_{7,8}$: Dystonin	2/2	2	4
1469	TUBB3 Q13509$_1$: Tubulin beta-3 chain		11	24
1464	TUBA3C Q13748$_1$: Tubulin alpha-3C/D chain	1/3	16	34
541	GANAB Q14697$_3$: Neutral alpha-glucosidase AB		2	6
1091	POSTN Q15063$_2$: Periostin		45	160
1092	POSTN Q15063$_3$: Periostin		46	205
1093	POSTN Q15063$_4$: Periostin		43	155
1094	POSTN Q15063$_5$: Periostin		48	210
1095	POSTN Q15063$_6$: Periostin		42	148
1096	POSTN Q15063$_7$: Periostin		40	143
790	KIF7 Q2M1P5: Kinesin-like protein KIF7		2	2
640	HLA-B Q31612: HLA class I histocompatibility antigen, B-73 alpha chain		5	10
1331	SNAPC4 Q5SXM2: snRNA-activating protein complex subunit 4		2	4
524	FRMPD1 Q5SYB0$_1$: FERM and PDZ domain-containing protein 1		2	4
340	CROCC Q5TZA2$_{1,2}$: Rootletin	2/2	2	4
1015	PAMR1 Q6UXH9$_1$: Inactive serine protease PAMR1		16	22
1017	PAMR1 Q6UXH9$_3$: Inactive serine protease PAMR1		15	22
1461	TUBA1A Q71U36$_{1,2}$: Tubulin alpha-1A chain	2/2	18	49
812	KRT28 Q7Z3Y7: Keratin, type I cytoskeletal 28		8	39
823	KRT73 Q86Y46$_1$: Keratin, type II cytoskeletal 73	1/2	7	13
622	HIST3H2BB Q8N257: Histone H2B type 3-B		11	34
814	KRT35 Q92764: Keratin, type I cuticular Ha5		3	16
1002	OPTN Q96CV9$_{1-3}$: Optineurin	3/3	2	27
617	HIST1H2BL Q99880: Histone H2B type 1-L	1/2	12	39
1463	TUBA1C Q9BQE3: Tubulin alpha-1C chain		17	48

2 Protein Group Details

Protein groups are created by grouping according to mass spectrometry evidence:

1. Proteins which are detected with the exact same set of peptides are clustered - they are indistinguishable based on available MS data. Very often these are splice variants. In the following text, proteins are clustered proteins (cluster size one if they have specific peptides).
2. Proteins, which have unique peptides (*i. e.* peptides not present in other detected proteins) are promoted to *reporters*.
3. Remaining proteins are grouped to those reporters which have a superset of the peptides they have. These proteins are termed *group members*

Peptides are characterized based on their specificity to one or more proteins, and one or more protein groups:

reporter specific rs — Number of peptides specific to reporter.
group specific gs — Number of peptides specific to the group (including rs).
unspecific us — Number of peptides shared with any protein.

1	136429, NA, NA	6/0/0
2	162648, NA, NA	42/0/6
3	A0A0B4J1V0, NA, NA	2/0/0

4		A0A0C4DH25, NA, NA	1/0/2
5		A0A0C4DH33, NA, NA	1/0/1
6	(1)	P04217-1, A1BG, Alpha-1B-glycoprotein	3/0/0
	(2)	P04217-2, A1BG, Alpha-1B-glycoprotein	
7		P01023, A2M, Alpha-2-macroglobulin	37/0/7
8		Q9NPC4, A4GALT, Lactosylceramide 4-alpha-galactosyltransferase	3/0/0
9		P49588-1,2, AARS, Alanine--tRNA ligase, cytoplasmic	12/0/0
10		Q9BUJ0, ABHD14A, Protein ABHD14A	2/0/0
11		Q96IU4-1, ABHD14B, Protein ABHD14B	2/0/0
12		Q7Z7G0-1, ABI3BP, Target of Nesh-SH3	4/0/0
13		P49748-1-3, ACADVL, Very long-chain specific acyl-CoA dehydrogenase, mitochondrial	2/0/0
14		Q99798, ACO2, Aconitate hydratase, mitochondrial	2/0/0
15	(1)	P60709, ACTB, Actin, cytoplasmic 1	5/0/13
	·	P63261, ACTG1, Actin, cytoplasmic 2	
	(2)	Q562R1, ACTBL2, Beta-actin-like protein 2	
16	(1)	P68032, ACTC1, Actin, alpha cardiac muscle 1	0/5/11
	(2)	P62736, ACTA2, Actin, aortic smooth muscle	
	(3)	P68133, ACTA1, Actin, alpha skeletal muscle	
	(4)	P63267-1, ACTG2, Actin, gamma-enteric smooth muscle	
	(5)	P63267-2, ACTG2, Actin, gamma-enteric smooth muscle	
17	(1)	P12814-2-4, ACTN1, Alpha-actinin-1	1/0/10
	(2)	P12814-1, ACTN1, Alpha-actinin-1	
18	(1)	P35609-2, ACTN2, Alpha-actinin-2	0/0/6
	(2)	P35609-1, ACTN2, Alpha-actinin-2	
19		Q08043, ACTN3, Alpha-actinin-3	1/0/3
20	(1)	O43707-1, ACTN4, Alpha-actinin-4	2/8/11
	(2)	O43707-2, ACTN4, Alpha-actinin-4	
	(3)	O43707-3, ACTN4, Alpha-actinin-4	
21		P61163, ACTR1A, Alpha-centractin	4/0/1
22	(1)	P61160-1, ACTR2, Actin-related protein 2	1/0/1
	(2)	P61160-2, ACTR2, Actin-related protein 2	
23		P61158, ACTR3, Actin-related protein 3	3/0/0
24		O14672-1, ADAM10, Disintegrin and metalloproteinase domain-containing protein 10	10/0/0

25	(1)	Q13444-1-10,12, ADAM15, Disintegrin and metalloproteinase domain-containing protein 15	0/0/0
	(2)	Q13444-11, ADAM15, Disintegrin and metalloproteinase domain-containing protein 15	
	(3)	Q13444-13, ADAM15, Disintegrin and metalloproteinase domain-containing protein 15	
26		O15204-1,2, ADAMDEC1, ADAM DEC1	6/0/0
27		Q9UHI8, ADAMTS1, A disintegrin and metalloproteinase with thrombospondin motifs 1	4/0/0
28		Q76LX8-1-3, ADAMTS13, A disintegrin and metalloproteinase with thrombospondin motifs 13	2/0/0
29		Q8TE58, ADAMTS15, A disintegrin and metalloproteinase with thrombospondin motifs 15	2/0/0
30	(1)	O95450-1, ADAMTS2, A disintegrin and metalloproteinase with thrombospondin motifs 2	2/0/0
	(2)	O95450-2, ADAMTS2, A disintegrin and metalloproteinase with thrombospondin motifs 2	
31		Q9UNA0, ADAMTS5, A disintegrin and metalloproteinase with thrombospondin motifs 5	7/0/0
32		Q9UP79, ADAMTS8, A disintegrin and metalloproteinase with thrombospondin motifs 8	2/0/0
33		Q86TH1, ADAMTSL2, ADAMTS-like protein 2	4/0/0
34	(1)	P82987-1, ADAMTSL3, ADAMTS-like protein 3	2/0/0
	(2)	P82987-2, ADAMTSL3, ADAMTS-like protein 3	
35	(1)	Q6UY14-1,3, ADAMTSL4, ADAMTS-like protein 4	6/0/0
	(2)	Q6UY14-2, ADAMTSL4, ADAMTS-like protein 4	
36		Q6ZMM2-1,2, ADAMTSL5, ADAMTS-like protein 5	1/0/0
37		P35611-1-6, ADD1, Alpha-adducin	1/0/0
38		Q8N7X0-1, ADGB, Androglobin	3/0/0
39	(1)	P00325-1, ADH1B, Alcohol dehydrogenase 1B	1/5/0
	(2)	P00326, ADH1C, Alcohol dehydrogenase 1C	
	(3)	P00325-2, ADH1B, Alcohol dehydrogenase 1B	
	·	P07327, ADH1A, Alcohol dehydrogenase 1A	
40		P11766, ADH5, Alcohol dehydrogenase class-3	9/0/0
41		Q15848, ADIPOQ, Adiponectin	4/0/0
42	(1)	Q8IUX7-1, AEBP1, Adipocyte enhancer-binding protein 1	18/0/1
	(2)	Q8IUX7-2, AEBP1, Adipocyte enhancer-binding protein 1	
43		P43652, AFM, Afamin	32/0/0

1/29/0

44 (1) O00468-3,6, AGRN, Agrin [Cleaved into: Agrin N-terminal 110 kDa subunit; Agrin C-terminal 110 kDa subunit; Agrin C-terminal 90 kDa fragment

(2) O00468-2, AGRN, Agrin [Cleaved into: Agrin N-terminal 110 kDa subunit; Agrin C-terminal 110 kDa subunit; Agrin C-terminal 90 kDa fragment

(3) O00468-1,4,5,7, AGRN, Agrin [Cleaved into: Agrin N-terminal 110 kDa subunit; Agrin C-terminal 110 kDa subunit; Agrin C-terminal 90 kDa fragment

12/0/0

45 P01019, AGT, Angiotensinogen

2/0/0

46 P23526-1,2, AHCY, Adenosylhomocysteinase

2/0/0

47 O43865-1, AHCYL1, Adenosylhomocysteinase 2

33/0/0

48 (1) Q09666-1, AHNAK, Neuroblast differentiation-associated protein AHNAK

(2) Q09666-2, AHNAK, Neuroblast differentiation-associated protein AHNAK

1/0/0

49 (1) O95433-1, AHSA1, Activator of 90 kDa heat shock protein ATPase homolog 1

(2) O95433-2, AHSA1, Activator of 90 kDa heat shock protein ATPase homolog 1

13/0/0

50 P02765, AHSG, Alpha-2-HS-glycoprotein

1/0/0

51 O95831-1-3,5, AIFM1, Apoptosis-inducing factor 1, mitochondrial

3/0/0

52 P00568, AK1, Adenylate kinase isoenzyme 1

3/0/0

53 Q02952-1-3, AKAP12, A-kinase anchor protein 12

3/0/0

54 O43488, AKR7A2, Aflatoxin B1 aldehyde reductase member 2

8/26/6

55 (1) P02768-1, ALB, Serum albumin

(2) P02768-2, ALB, Serum albumin

(3) P02768-3, ALB, Serum albumin

2/0/3

56 P00352, ALDH1A1, Retinal dehydrogenase 1

0/6/3

57 (1) O94788-1, ALDH1A2, Retinal dehydrogenase 2

(2) O94788-2, ALDH1A2, Retinal dehydrogenase 2

(3) O94788-3, ALDH1A2, Retinal dehydrogenase 2

(4) O94788-4, ALDH1A2, Retinal dehydrogenase 2

7/0/3

58 (1) P47895, ALDH1A3, Aldehyde dehydrogenase family 1 member A3

(2) P30837, ALDH1B1, Aldehyde dehydrogenase X, mitochondrial

59	(1)	P05091-1, ALDH2, Aldehyde dehydrogenase, mitochondrial	0/0/3
	(2)	P05091-2, ALDH2, Aldehyde dehydrogenase, mitochondrial	
60		P49419-1,2,4, ALDH7A1, Alpha-aminoadipic semialdehyde dehydrogenase	2/0/0
61	(1)	P49189-1, ALDH9A1, 4-trimethylaminobutyraldehyde dehydrogenase	2/0/0
	(2)	P49189-2, ALDH9A1, 4-trimethylaminobutyraldehyde dehydrogenase	
62	(1)	P04075-1,2, ALDOA, Fructose-bisphosphate aldolase A	6/0/0
	(2)	P09972, ALDOC, Fructose-bisphosphate aldolase C	
63		P16050-1,2, ALOX15, Arachidonate 15-lipoxygenase	5/0/0
64		P02760, AMBP, Protein AMBP [Cleaved into: Alpha-1-microglobulin	14/0/0
65		P03950, ANG, Angiogenin	13/0/0
66		O15123-1-3, ANGPT2, Angiopoietin-2	2/0/0
67		O95841, ANGPTL1, Angiopoietin-related protein 1	3/0/0
68	(1)	Q9UKU9-1, ANGPTL2, Angiopoietin-related protein 2	7/0/0
	(2)	Q9UKU9-2, ANGPTL2, Angiopoietin-related protein 2	
69		Q9Y5C1, ANGPTL3, Angiopoietin-related protein 3	4/0/0
70	(1)	Q9BY76-1, ANGPTL4, Angiopoietin-related protein 4	0/5/0
	(2)	Q9BY76-2, ANGPTL4, Angiopoietin-related protein 4	
	(3)	Q9BY76-3, ANGPTL4, Angiopoietin-related protein 4	
71		Q12955-3, ANK3, Ankyrin-3	4/0/0
72	(1)	Q9H6X2-1,5, ANTXR1, Anthrax toxin receptor 1	6/0/1
	(2)	Q9H6X2-2-4,6, ANTXR1, Anthrax toxin receptor 1	
73	(1)	P58335-1,2, ANTXR2, Anthrax toxin receptor 2	1/0/11
	(2)	P58335-3, ANTXR2, Anthrax toxin receptor 2	
74		P58335-4, ANTXR2, Anthrax toxin receptor 2	1/0/11
75		P04083, ANXA1, Annexin A1	13/0/0
76		P50995-1,2, ANXA11, Annexin A11	3/0/0
77	(1)	P07355-1,2, ANXA2, Annexin A2	3/0/0
	(2)	A6NMY6, ANXA2P2, Putative annexin A2-like protein	
78	(1)	P09525-1, ANXA4, Annexin A4	1/0/1
	(2)	P09525-2, ANXA4, Annexin A4	
79		P08758, ANXA5, Annexin A5	4/0/1

80		P08133-1,2, ANXA6, Annexin A6	2/0/0
81		P20073-1,2, ANXA7, Annexin A7	3/0/0
82		O76027, ANXA9, Annexin A9	1/0/0
83		Q06278, AOX1, Aldehyde oxidase	8/0/0
84		Q10567-1-4, AP1B1, AP-1 complex subunit beta-1	2/0/5
85		O43747-1,2, AP1G1, AP-1 complex subunit gamma-1	1/0/0
86		Q9BXS5-1,2, AP1M1, AP-1 complex subunit mu-1	2/0/0
87	(1)	O95782-1,2, AP2A1, AP-2 complex subunit alpha-1	2/0/0
	(2)	O94973-1-3, AP2A2, AP-2 complex subunit alpha-2	0/0/5
88	(1)	P63010-1,2, AP2B1, AP-2 complex subunit beta	
	(2)	P63010-3, AP2B1, AP-2 complex subunit beta	
89		Q96CW1-1,2, AP2M1, AP-2 complex subunit mu	2/0/0
90		P02743, APCS, Serum amyloid P-component	9/0/0
91		P13798, APEH, Acylamino-acid-releasing enzyme	4/0/0
92	(1)	Q06481-1,3,6, APLP2, Amyloid-like protein 2	1/0/0
	(2)	Q06481-2,4,5, APLP2, Amyloid-like protein 2	
93	(1)	Q9HDC9-1, APMAP, Adipocyte plasma membrane-associated protein	1/0/0
	(2)	Q9HDC9-2, APMAP, Adipocyte plasma membrane-associated protein	
94		P02647, APOA1, Apolipoprotein A-I	35/0/0
95		P02652, APOA2, Apolipoprotein A-II	5/0/0
96		P06727, APOA4, Apolipoprotein A-IV	37/0/2
97		Q6Q788, APOA5, Apolipoprotein A-V	4/0/0
98		P04114, APOB, Apolipoprotein B-100	268/0/2
99		Q0VD83-1,3,4, APOBR, Apolipoprotein B receptor	5/0/0
100		P02654, APOC1, Apolipoprotein C-I	5/0/0
101		P02655, APOC2, Apolipoprotein C-II	4/0/0
102		P02656, APOC3, Apolipoprotein C-III	8/0/0
103		P55056, APOC4, Apolipoprotein C-IV	5/0/0
104		P05090, APOD, Apolipoprotein D	8/0/0
105		P02649, APOE, Apolipoprotein E	28/0/0
106		Q13790, APOF, Apolipoprotein F	8/0/0

107 P02749, APOH, Beta-2-glycoprotein 1 — 13/0/0

108 (1) O14791-1,2, APOL1, Apolipoprotein L1 — 1/0/0
(2) O14791-3, APOL1, Apolipoprotein L1

109 Q9BQE5, APOL2, Apolipoprotein L2 — 4/0/0

110 (1) O95445-1, APOM, Apolipoprotein M — 1/0/0
(2) O95445-2, APOM, Apolipoprotein M

111 (1) P05067-1,7-9,11, APP, Amyloid beta A4 protein — 1/7/0
(2) P05067-2, APP, Amyloid beta A4 protein
(3) P05067-10, APP, Amyloid beta A4 protein
(4) P05067-3-6, APP, Amyloid beta A4 protein

112 P07741-1,2, APRT, Adenine phosphoribosyltransferase — 4/0/0

113 P61204-1,2, ARF3, ADP-ribosylation factor 3 — 1/0/2
P84077, ARF1, ADP-ribosylation factor 1

114 P18085, ARF4, ADP-ribosylation factor 4 — 2/0/1

115 P84085, ARF5, ADP-ribosylation factor 5 — 1/0/2

116 Q07960, ARHGAP1, Rho GTPase-activating protein 1 — 5/0/0

117 (1) Q8N392-1, ARHGAP18, Rho GTPase-activating protein 18 — 1/0/0
(2) Q8N392-2, ARHGAP18, Rho GTPase-activating protein 18

118 Q9NRY4, ARHGAP35, Rho GTPase-activating protein 35 — 3/0/1

119 (1) P52565-1, ARHGDIA, Rho GDP-dissociation inhibitor 1 — 2/0/0
(2) P52565-2, ARHGDIA, Rho GDP-dissociation inhibitor 1

120 P52566, ARHGDIB, Rho GDP-dissociation inhibitor 2 — 2/0/0

121 P36405, ARL3, ADP-ribosylation factor-like protein 3 — 2/0/0

122 O15143, ARPC1B, Actin-related protein 2/3 complex subunit 1B — 2/0/0

123 O15144, ARPC2, Actin-related protein 2/3 complex subunit 2 — 2/0/0

124 P07307-1-3, ASGR2, Asialoglycoprotein receptor 2 — 5/0/0

125 O95671-1-3, ASMTL, N-acetylserotonin O-methyltransferase-like protein — 2/0/0

126 Q12797-1,10, ASPH, Aspartyl/asparaginyl beta-hydroxylase — 10/0/0

127 Q9BXN1, ASPN, Asporin — 1/0/0

128 Q9NT62-1,2, ATG3, Ubiquitin-like-conjugating enzyme ATG3 — 1/0/0

129		P31939-1,2, ATIC, Bifunctional purine biosynthesis protein PURH [Cleaved into: Bifunctional purine biosynthesis protein PURH, N-terminally processed] [Includes: Phosphoribosylaminoimidazolecarboxamide formyltransferase	5/0/0
130		Q6DD88, ATL3, Atlastin-3	3/0/0
131		O14983-1, ATP2A1, Sarcoplasmic/endoplasmic reticulum calcium ATPase 1	2/0/1
132		P16615-1-5, ATP2A2, Sarcoplasmic/endoplasmic reticulum calcium ATPase 2	1/0/1
133	(1)	P25705-1,3, ATP5A1, ATP synthase subunit alpha, mitochondrial	1/0/0
	(2)	P25705-2, ATP5A1, ATP synthase subunit alpha, mitochondrial	
134		P06576, ATP5B, ATP synthase subunit beta, mitochondrial	5/0/0
135		P36542-1,2, ATP5C1, ATP synthase subunit gamma, mitochondrial	3/0/1
136		P56385, ATP5I, ATP synthase subunit e, mitochondrial	1/0/1
137		Q15904, ATP6AP1, V-type proton ATPase subunit S1	5/0/0
138		O75787-1,2, ATP6AP2, Renin receptor	8/0/0
139	(1)	P21281, ATP6V1B2, V-type proton ATPase subunit B, brain isoform	1/0/1
	(2)	P15313, ATP6V1B1, V-type proton ATPase subunit B, kidney isoform	
140	(1)	P36543-1,3, ATP6V1E1, V-type proton ATPase subunit E 1	1/0/0
	(2)	Q96A05, ATP6V1E2, V-type proton ATPase subunit E 2	
141		P30530-1,2, AXL, Tyrosine-protein kinase receptor UFO	2/0/0
142		P25311, AZGP1, Zinc-alpha-2-glycoprotein	9/0/0
143		P61769, B2M, Beta-2-microglobulin [Cleaved into: Beta-2-microglobulin form pI 5.3]	5/0/0
144	(1)	O94766-1, B3GAT3, Galactosylgalactosylxylosylprotein 3-beta-glucuronosyltransferase 3	1/0/0
	(2)	O94766-2, B3GAT3, Galactosylgalactosylxylosylprotein 3-beta-glucuronosyltransferase 3	
145		Q9NY97-1,2, B3GNT2, N-acetyllactosaminide beta-1,3-N-acetylglucosaminyltransferase 2	2/0/0
146		Q8NFL0, B3GNT7, UDP-GlcNAc:betaGal beta-1,3-N-acetylglucosaminyltransferase 7	2/0/0
147		Q6UX72, B3GNT9, UDP-GlcNAc:betaGal beta-1,3-N-acetylglucosaminyltransferase 9	2/0/0
148		P15291-1,2, B4GALT1, Beta-1,4-galactosyltransferase 1	11/0/0
149		O60513, B4GALT4, Beta-1,4-galactosyltransferase 4	7/0/0
150		O43505, B4GAT1, Beta-1,4-glucuronyltransferase 1	6/0/0

151		Q13145, BAMBI, BMP and activin membrane-bound inhibitor homolog	4/0/0
152		O75531, BANF1, Barrier-to-autointegration factor	2/0/0
153		P51572-1,2, BCAP31, B-cell receptor-associated protein 31	1/0/1
154		P06276, BCHE, Cholinesterase	5/0/0
155	(1)	Q9BUT1-1, BDH2, 3-hydroxybutyrate dehydrogenase type 2	0/0/0
	(2)	Q9BUT1-2, BDH2, 3-hydroxybutyrate dehydrogenase type 2	
	(3)	Q9BUT1-3, BDH2, 3-hydroxybutyrate dehydrogenase type 2	
156		P21810, BGN, Biglycan	9/0/1
157		Q6PI77, BHLHB9, Protein BHLHb9	2/0/1
158		P30043, BLVRB, Flavin reductase	5/0/0
159	(1)	P13497-1, BMP1, Bone morphogenetic protein 1	3/6/6
	(2)	P13497-2,5,6, BMP1, Bone morphogenetic protein 1	
	(3)	P13497-3, BMP1, Bone morphogenetic protein 1	
	(4)	P13497-4, BMP1, Bone morphogenetic protein 1	
160		P12645, BMP3, Bone morphogenetic protein 3	5/0/0
161	(1)	Q8TDL5-1, BPIFB1, BPI fold-containing family B member 1	4/0/1
	(2)	Q8TDL5-2, BPIFB1, BPI fold-containing family B member 1	
162		P43251-1-4, BTD, Biotinidase	3/0/0
163	(1)	Q7L1Q6-1,3,4, BZW1, Basic leucine zipper and W2 domain-containing protein 1	1/0/0
	(2)	Q7L1Q6-2, BZW1, Basic leucine zipper and W2 domain-containing protein 1	
164		P02745, C1QA, Complement C1q subcomponent subunit A	7/0/0
165		P02746, C1QB, Complement C1q subcomponent subunit B	8/0/0
166		Q07021, C1QBP, Complement component 1 Q subcomponent-binding protein, mitochondrial	1/0/0
167		P02747, C1QC, Complement C1q subcomponent subunit C	5/0/0
168		Q9BXJ3, C1QTNF4, Complement C1q tumor necrosis factor-related protein 4	2/0/0
169		P00736, C1R, Complement C1r subcomponent	30/0/2
170		Q9NZP8, C1RL, Complement C1r subcomponent-like protein	10/0/2
171		P09871, C1S, Complement C1s subcomponent	24/0/1

172	(1)	P06681-1, C2, Complement C2	2/27/0
	(2)	P06681-2, C2, Complement C2	
	(3)	P06681-3, C2, Complement C2	
173		Q9H1Z8, C2orf40, Augurin	4/0/0
174		P01024, C3, Complement C3	137/0/0
175	(1)	P0C0L4-1, C4A, Complement C4-A	0/0/88
	(2)	P0C0L4-2, C4A, Complement C4-A	
176		P0C0L5, C4B, Complement C4-B	5/0/88
177		P04003, C4BPA, C4b-binding protein alpha chain	33/0/0
178		P20851-1,2, C4BPB, C4b-binding protein beta chain	12/0/0
179		P01031, C5, Complement C5	74/0/0
180		Q569G3, C5orf47, Uncharacterized protein C5orf47	2/0/0
181		P13671, C6, Complement component C6	39/0/1
182		P10643, C7, Complement component C7	42/0/1
183		P07357, C8A, Complement component C8 alpha chain	22/0/0
184		P07358, C8B, Complement component C8 beta chain	29/0/0
185		P07360, C8G, Complement component C8 gamma chain	11/0/0
186		P02748, C9, Complement component C9 [Cleaved into: Complement component C9a; Complement component C9b]	28/0/3
187		Q5T6V5, C9orf64, UPF0553 protein C9orf64	3/0/0
188	(1)	Q00975-2, CACNA1B, Voltage-dependent N-type calcium channel subunit alpha-1B	1/0/0
	(2)	Q00975-1, CACNA1B, Voltage-dependent N-type calcium channel subunit alpha-1B	
189	(1)	Q01668-2, CACNA1D, Voltage-dependent L-type calcium channel subunit alpha-1D	1/0/0
	(2)	Q01668-1,3,4, CACNA1D, Voltage-dependent L-type calcium channel subunit alpha-1D	
190		Q05682-1-6, CALD1, Caldesmon	3/0/1
191		P62158, CALM1, Calmodulin	6/0/2
192		P27482, CALML3, Calmodulin-like protein 3	4/0/2
193		P27797, CALR, Calreticulin	20/0/0
194	(1)	O43852-1,3,6,10, CALU, Calumenin	0/1/0

	(2)	O43852-5,11,13,14, CALU, Calumenin	
	(3)	O43852-8, CALU, Calumenin	
	(4)	O43852-2,4,15, CALU, Calumenin	
195		Q13554-3-5, CAMK2B, Calcium/calmodulin-dependent protein kinase type II subunit beta<br>Q13555-1-4,7-10, CAMK2G, Calcium/calmodulin-dependent protein kinase type II subunit gamma<br>Q13557-1,3-6,8-12, CAMK2D, Calcium/calmodulin-dependent protein kinase type II subunit delta	1/0/1
196		P49913, CAMP, Cathelicidin antimicrobial peptide	3/0/0
197	(1)	Q86VP6-1, CAND1, Cullin-associated NEDD8-dissociated protein 1	2/4/0
	(2)	Q86VP6-2, CAND1, Cullin-associated NEDD8-dissociated protein 1	
	(3)	Q86VP6-3, CAND1, Cullin-associated NEDD8-dissociated protein 1	
198	(1)	P27824-1,2, CANX, Calnexin	3/0/1
	(2)	P27824-3, CANX, Calnexin	
199		Q01518-1,2, CAP1, Adenylyl cyclase-associated protein 1	13/0/1
200		P40123-1, CAP2, Adenylyl cyclase-associated protein 2	1/0/1
201		P40121-1,2, CAPG, Macrophage-capping protein	6/0/0
202		P07384, CAPN1, Calpain-1 catalytic subunit	4/0/1
203	(1)	P17655-1, CAPN2, Calpain-2 catalytic subunit	3/0/1
	(2)	P17655-2, CAPN2, Calpain-2 catalytic subunit	
204		P04632, CAPNS1, Calpain small subunit 1	6/0/0
205		P52907, CAPZA1, F-actin-capping protein subunit alpha-1	4/0/1
206	(1)	P47755-1, CAPZA2, F-actin-capping protein subunit alpha-2	0/0/1
	(2)	P47755-2, CAPZA2, F-actin-capping protein subunit alpha-2	
207	(1)	P47756-2, CAPZB, F-actin-capping protein subunit beta	1/0/0
	(2)	P47756-1, CAPZB, F-actin-capping protein subunit beta	
208		P31944, CASP14, Caspase-14	4/0/0
209	(1)	P20810-5,6,10, CAST, Calpastatin	0/6/0
	(2)	P20810-7,9, CAST, Calpastatin	
	(3)	P20810-1,2,8, CAST, Calpastatin	
	(4)	P20810-3, CAST, Calpastatin	
	(5)	P20810-4, CAST, Calpastatin	

210 Q03135-1,2, CAV1, Caveolin-1 — 2/0/0 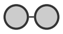

211 P16152-1, CBR1, Carbonyl reductase [NADPH] 1 — 6/0/0 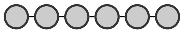

212 Q13185, CBX3, Chromobox protein homolog 3 — 2/0/0 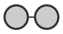

213 Q8N163-1,2, CCAR2, Cell cycle and apoptosis regulator protein 2 — 4/0/0 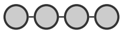

214 Q76M96-1,2, CCDC80, Coiled-coil domain-containing protein 80 — 26/0/0 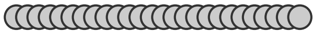

215 I3L3R5, CCER2, Coiled-coil domain-containing glutamate-rich protein 2 — 3/0/0 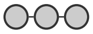

216 — 2/0/0 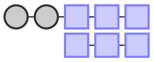
(1) Q16627-1, CCL14, C-C motif chemokine 14
(2) Q16627-2, CCL14, C-C motif chemokine 14

217 O15467, CCL16, C-C motif chemokine 16 — 3/0/0 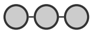

218 P55774, CCL18, C-C motif chemokine 18 — 3/0/0 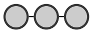

219 — 1/0/0 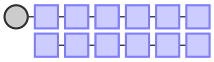
(1) P78371-1, CCT2, T-complex protein 1 subunit beta
(2) P78371-2, CCT2, T-complex protein 1 subunit beta

220 — 1/0/0 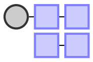
(1) P49368-1, CCT3, T-complex protein 1 subunit gamma
(2) P49368-2, CCT3, T-complex protein 1 subunit gamma

221 P50991-1,2, CCT4, T-complex protein 1 subunit delta — 6/0/0 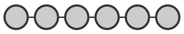

222 P48643-1,2, CCT5, T-complex protein 1 subunit epsilon — 6/0/0 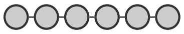

223 — 1/3/0 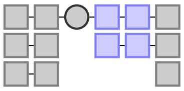
(1) P40227-1, CCT6A, T-complex protein 1 subunit zeta
(2) P40227-2, CCT6A, T-complex protein 1 subunit zeta
(3) Q92526-1-3, CCT6B, T-complex protein 1 subunit zeta-2

224 — 1/0/0 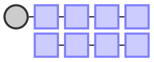
(1) P50990-1,2, CCT8, T-complex protein 1 subunit theta
(2) P50990-3, CCT8, T-complex protein 1 subunit theta

225 — 1/5/0 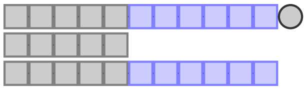
(1) Q6YHK3-1,2, CD109, CD109 antigen
(2) Q6YHK3-3, CD109, CD109 antigen
(3) Q6YHK3-4, CD109, CD109 antigen

226 P08571, CD14, Monocyte differentiation antigen CD14 — 17/0/0 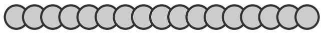

227 P48509, CD151, CD151 antigen — 3/0/0 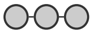

228 — 1/0/0 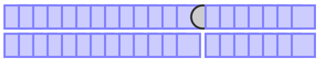
(1) Q86VB7-1-3, CD163, Scavenger receptor cysteine-rich type 1 protein M130
(2) Q86VB7-4, CD163, Scavenger receptor cysteine-rich type 1 protein M130

229 Q9NR16-1,2,4, CD163L1, Scavenger receptor cysteine-rich type 1 protein M160 — 2/0/0 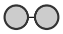

230 Q9HCU0-1, CD248, Endosialin — 4/0/0 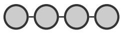

231		O60449-2,3, LY75, Lymphocyte antigen 75	3/0/0
		Q8IX05-1, CD302, CD302 antigen	
232		O43866, CD5L, CD5 antigen-like	18/0/1
233		P04233-1,2, CD74, HLA class II histocompatibility antigen gamma chain	2/0/0
234		P60033, CD81, CD81 antigen	2/0/0
235		P01732-1-3, CD8A, T-cell surface glycoprotein CD8 alpha chain	1/0/0
236		P21926, CD9, CD9 antigen	3/0/0
237		P48960-1-3, CD97, CD97 antigen	3/0/0
238		P14209-1,3, CD99, CD99 antigen	1/0/0
239		Q16543, CDC37, Hsp90 co-chaperone Cdc37	3/0/0
240	(1)	P60953-1,2, CDC42, Cell division control protein 42 homolog	
	(2)	P17081, RHOQ, Rho-related GTP-binding protein RhoQ	2/0/1
	·	P84095, RHOG, Rho-related GTP-binding protein RhoG	
	·	Q9H4E5-1,2, RHOJ, Rho-related GTP-binding protein RhoJ	
241		P12830-1,2, CDH1, Cadherin-1	3/0/0
242	(1)	P19022-1, CDH2, Cadherin-2	1/0/0
	(2)	P19022-2, CDH2, Cadherin-2	
243		O95319-1-5, CELF2, CUGBP Elav-like family member 2	2/0/0
244		Q9BS16, CENPK, Centromere protein K	3/0/0
245		Q5VT06, CEP350, Centrosome-associated protein 350	1/0/2
246	(1)	P11597-1, CETP, Cholesteryl ester transfer protein	5/0/0
	(2)	P11597-2, CETP, Cholesteryl ester transfer protein	
247	(1)	P00751-1, CFB, Complement factor B	12/0/0
	(2)	P00751-2, CFB, Complement factor B	
248		P00746, CFD, Complement factor D	17/0/0
249	(1)	P08603-1, CFH, Complement factor H	30/0/8
	(2)	P08603-2, CFH, Complement factor H	
250		Q03591, CFHR1, Complement factor H-related protein 1	1/0/12
251	(1)	P36980-1, CFHR2, Complement factor H-related protein 2	1/0/5
	(2)	P36980-2, CFHR2, Complement factor H-related protein 2	
252		Q02985-1,2, CFHR3, Complement factor H-related protein 3	3/0/5
253	(1)	Q92496-1,2, CFHR4, Complement factor H-related protein 4	3/0/5
	(2)	Q92496-3, CFHR4, Complement factor H-related protein 4	

254		Q9BXR6, CFHR5, Complement factor H-related protein 5	21/0/2
255		P05156, CFI, Complement factor I	21/0/0
256	(1)	P23528, CFL1, Cofilin-1	5/0/1
	(2)	Q9Y281-1,3, CFL2, Cofilin-2	
257		P27918, CFP, Properdin	15/0/1
258		Q99674-1,4,5, CGREF1, Cell growth regulator with EF hand domain protein 1	10/0/0
259		P10645, CHGA, Chromogranin-A	29/0/0
260		P05060, CHGB, Secretogranin-1	26/0/1
261		O43633, CHMP2A, Charged multivesicular body protein 2a	2/0/0
262	(1)	Q9H2X0-1,5, CHRD, Chordin	10/0/0
	(2)	Q9H2X0-4, CHRD, Chordin	
263	(1)	Q9BU40-2,4, CHRDL1, Chordin-like protein 1	1/20/0
	(2)	Q9BU40-3, CHRDL1, Chordin-like protein 1	
	(3)	Q9BU40-5, CHRDL1, Chordin-like protein 1	
	(4)	Q9BU40-1,6, CHRDL1, Chordin-like protein 1	
264		Q6WN34-1,2, CHRDL2, Chordin-like protein 2	4/0/0
265		Q9NPF2-1,2, CHST11, Carbohydrate sulfotransferase 11	1/0/1
266		Q9NRB3, CHST12, Carbohydrate sulfotransferase 12	1/0/1
267		Q8NCH0, CHST14, Carbohydrate sulfotransferase 14	6/0/0
268	(1)	Q7LFX5-1, CHST15, Carbohydrate sulfotransferase 15	1/0/0
	(2)	Q7LFX5-2, CHST15, Carbohydrate sulfotransferase 15	
269		O75339, CILP, Cartilage intermediate layer protein 1	51/0/1
270		Q8IUL8, CILP2, Cartilage intermediate layer protein 2	1/0/1
271		Q07065, CKAP4, Cytoskeleton-associated protein 4	8/0/1
272		P12277, CKB, Creatine kinase B-type	7/0/0
273		Q05315, CLC, Galectin-10	4/0/0
274		Q9Y240, CLEC11A, C-type lectin domain family 11 member A	14/0/0
275		P05452, CLEC3B, Tetranectin	8/0/0
276		O14967-1, CLGN, Calmegin	1/0/1
277		O00299, CLIC1, Chloride intracellular channel protein 1	9/0/1
278		Q9Y696, CLIC4, Chloride intracellular channel protein 4	3/0/2

279 (1) Q9NZA1-1, CLIC5, Chloride intracellular channel protein 5 — 1/0/1
(2) Q9NZA1-2,3, CLIC5, Chloride intracellular channel protein 5

280 O94985-1, CLSTN1, Calsyntenin-1 — 1/0/16

281 O94985-2, CLSTN1, Calsyntenin-1 — 1/0/16

282 Q9BQT9-1,2, CLSTN3, Calsyntenin-3 — 3/0/0

283 P09497-1,2, CLTB, Clathrin light chain B — 2/0/0

284 (1) Q00610-1,2, CLTC, Clathrin heavy chain 1 — 15/0/0
(2) P53675-1,2, CLTCL1, Clathrin heavy chain 2

285 (1) P10909-4, CLU, Clusterin — 1/15/0
(2) P10909-1,2,5, CLU, Clusterin
(3) P10909-3, CLU, Clusterin

286 Q96KN2, CNDP1, Beta-Ala-His dipeptidase — 8/0/0

287 P51911-1,2, CNN1, Calponin-1 — 2/0/1

288 (1) Q15417-1,3, CNN3, Calponin-3 — 2/0/1
(2) Q15417-2, CNN3, Calponin-3

289 (1) Q99715-1, COL12A1, Collagen alpha-1(XII) chain — 0/31/1
(2) Q99715-2, COL12A1, Collagen alpha-1(XII) chain
(3) Q99715-4, COL12A1, Collagen alpha-1(XII) chain

290 (1) Q05707-1,2, COL14A1, Collagen alpha-1(XIV) chain — 7/0/0
(2) Q05707-3, COL14A1, Collagen alpha-1(XIV) chain

291 P39059, COL15A1, Collagen alpha-1(XV) chain [Cleaved into: Restin — 5/0/0

292 Q07092-1,2, COL16A1, Collagen alpha-1(XVI) chain — 3/0/0

293 P39060-1-3, COL18A1, Collagen alpha-1(XVIII) chain [Cleaved into: Endostatin] — 13/0/0

294 P02452, COL1A1, Collagen alpha-1(I) chain — 21/0/3

295 P08123, COL1A2, Collagen alpha-2(I) chain — 22/0/0

296 P02458-2, COL2A1, Collagen alpha-1(II) chain — 1/0/2

297 (1) P02461-1, COL3A1, Collagen alpha-1(III) chain — 3/0/3
(2) P02461-2, COL3A1, Collagen alpha-1(III) chain

298 P08572, COL4A2, Collagen alpha-2(IV) chain [Cleaved into: Canstatin] — 2/0/0

299 Q9Y5P4-1-3, COL4A3BP, Collagen type IV alpha-3-binding protein — 1/0/0

300		P20908, COL5A1, Collagen alpha-1(V) chain	10/0/0
301		P05997, COL5A2, Collagen alpha-2(V) chain	12/0/2
302		P12109, COL6A1, Collagen alpha-1(VI) chain	27/0/0
303	(1)	P12110-1, COL6A2, Collagen alpha-2(VI) chain	7/0/1
	(2)	P12110-2,3, COL6A2, Collagen alpha-2(VI) chain	
			3/10/106
304	(1)	P12111-1, COL6A3, Collagen alpha-3(VI) chain	
	(2)	P12111-2, COL6A3, Collagen alpha-3(VI) chain	
	(3)	P12111-3, COL6A3, Collagen alpha-3(VI) chain	
	(4)	P12111-5, COL6A3, Collagen alpha-3(VI) chain	
305		P12111-4, COL6A3, Collagen alpha-3(VI) chain	2/0/106
306		A6NMZ7-1, COL6A6, Collagen alpha-6(VI) chain	3/0/0
307		Q02388-1,2, COL7A1, Collagen alpha-1(VII) chain	2/0/1
308		P27658, COL8A1, Collagen alpha-1(VIII) chain	1/0/1
309		Q9Y6Z7, COLEC10, Collectin-10	3/0/0
310		Q8NBJ5, COLGALT1, Procollagen galactosyltransferase 1	1/0/0
311	(1)	P49747-1, COMP, Cartilage oligomeric matrix protein	2/0/2
	(2)	P49747-2, COMP, Cartilage oligomeric matrix protein	
312		P53621-1,2, COPA, Coatomer subunit alpha	4/0/0
313		P53618, COPB1, Coatomer subunit beta	10/0/0
314		P35606-1,2, COPB2, Coatomer subunit beta’	3/0/0
315	(1)	O14579-1,3, COPE, Coatomer subunit epsilon	1/0/0
	(2)	O14579-2, COPE, Coatomer subunit epsilon	
316		Q9Y678, COPG1, Coatomer subunit gamma-1	7/0/0
317		Q9BT78-1,2, COPS4, COP9 signalosome complex subunit 4	1/0/0
318		P61923-1,4,5, COPZ1, Coatomer subunit zeta-1	1/0/0
319	(1)	P31146, CORO1A, Coronin-1A	4/0/2
	(2)	Q6QEF8-1-5, CORO6, Coronin-6	
320		Q9BR76, CORO1B, Coronin-1B	3/0/1
321		Q9ULV4-1-3, CORO1C, Coronin-1C	4/0/2
322		P57737-3, CORO7, Coronin-7	3/0/0
323		Q14019, COTL1, Coactosin-like protein	2/0/0
324		P00450, CP, Ceruloplasmin	39/0/0

325 Q9UI42-1,2, CPA4, Carboxypeptidase A4 — 1/0/0

326 (1) Q96IY4-1, CPB2, Carboxypeptidase B2 — 5/0/0
(2) Q96IY4-2, CPB2, Carboxypeptidase B2

327 P16870-1,2, CPE, Carboxypeptidase E — 27/0/0

328 P15169, CPN1, Carboxypeptidase N catalytic chain — 15/0/0

329 P22792, CPN2, Carboxypeptidase N subunit 2 — 14/0/0

330 Q99829, CPNE1, Copine-1 — 2/0/0

331 Q9Y646, CPQ, Carboxypeptidase Q — 6/0/0

332 Q10570, CPSF1, Cleavage and polyadenylation specificity factor subunit 1 — 1/0/1

333 Q96SM3, CPXM1, Probable carboxypeptidase X1 — 11/0/0

334 Q8N436, CPXM2, Inactive carboxypeptidase-like protein X2 — 15/0/1

335 (1) Q66K79-1,2, CPZ, Carboxypeptidase Z — 1/0/0
(2) Q66K79-3, CPZ, Carboxypeptidase Z

336 P29373, CRABP2, Cellular retinoic acid-binding protein 2 — 6/0/0

337 P24387, CRHBP, Corticotropin-releasing factor-binding protein — 5/0/0

338 P50238, CRIP1, Cysteine-rich protein 1 — 1/0/1

339 (1) P52943-1, CRIP2, Cysteine-rich protein 2 — 1/0/1
(2) P52943-2, CRIP2, Cysteine-rich protein 2

340 Q5TZA2-1,2, CROCC, Rootletin — 0/0/2

341 (1) P02741-1, CRP, C-reactive protein [Cleaved into: C-reactive protein(1-205)] — 3/0/0
(2) P02741-2, CRP, C-reactive protein [Cleaved into: C-reactive protein(1-205)]

342 (1) Q9NQ79-1, CRTAC1, Cartilage acidic protein 1 — 1/0/0
(2) Q9NQ79-2,3, CRTAC1, Cartilage acidic protein 1

343 P02511, CRYAB, Alpha-crystallin B chain — 2/0/0

344 Q9Y2S2-1,2, CRYL1, Lambda-crystallin homolog — 2/0/0

345 O75534-1-4, CSDE1, Cold shock domain-containing protein E1 — 1/0/0

346 (1) P55060-1,3, CSE1L, Exportin-2 — 1/4/0
(2) P55060-2, CSE1L, Exportin-2
(3) P55060-4, CSE1L, Exportin-2

347		Q8TDX6-1, CSGALNACT1, Chondroitin sulfate N-acetylgalactosaminyltransferase 1	1/0/1
348		Q6UVK1, CSPG4, Chondroitin sulfate proteoglycan 4	11/0/0
349		P01034, CST3, Cystatin-C	6/0/0
350		Q15828, CST6, Cystatin-M	8/0/0
351		P04080, CSTB, Cystatin-B	2/0/0
352		P29279-1,2, CTGF, Connective tissue growth factor	5/0/0
353	(1)	P35221-1,2, CTNNA1, Catenin alpha-1	0/6/0
	(2)	P26232-1-3,5, CTNNA2, Catenin alpha-2	
	(3)	P26232-4,6, CTNNA2, Catenin alpha-2	
	(4)	P35221-3, CTNNA1, Catenin alpha-1	
354		P10619-1,2, CTSA, Lysosomal protective protein	3/0/0
355		P07858, CTSB, Cathepsin B	4/0/0
356		P53634-1, CTSC, Dipeptidyl peptidase 1	2/0/0
357		P07339, CTSD, Cathepsin D	7/0/0
358		Q9UBX1, CTSF, Cathepsin F	11/0/0
359		P09668, CTSH, Pro-cathepsin H [Cleaved into: Cathepsin H mini chain; Cathepsin H	2/0/0
360		P43235, CTSK, Cathepsin K	3/0/0
361		P07711, CTSL, Cathepsin L1	1/0/0
362		P25774-1,2, CTSS, Cathepsin S	1/0/0
363		Q14247-1,3, CTTN, Src substrate cortactin	2/0/0
364		Q13618-1-3, CUL3, Cullin-3	2/0/0
365		P48061-1-6, CXCL12, Stromal cell-derived factor 1	5/0/0
366	(1)	Q7L576-1, CYFIP1, Cytoplasmic FMR1-interacting protein 1	0/0/2
	(2)	Q7L576-2,3, CYFIP1, Cytoplasmic FMR1-interacting protein 1	
367		Q96F07-1,2, CYFIP2, Cytoplasmic FMR1-interacting protein 2	2/0/2
368	(1)	O00622, CYR61, Protein CYR61	1/0/0
	(2)	O95389-1,2, WISP3, WNT1-inducible-signaling pathway protein 3	
369		Q9NRR1, CYTL1, Cytokine-like protein 1	4/0/0
370		Q14118, DAG1, Dystroglycan	3/0/0
371		P14868-1,2, DARS, Aspartate--tRNA ligase, cytoplasmic	3/0/0
372		Q9UJU6-1-6, DBNL, Drebrin-like protein	2/0/0

373	(1)	P81605-1, DCD, Dermcidin	1/0/0
	(2)	P81605-2, DCD, Dermcidin	
374	(1)	P07585-1, DCN, Decorin	3/5/1
	(2)	P07585-2, DCN, Decorin	
	(3)	P07585-3, DCN, Decorin	
	(4)	P07585-4, DCN, Decorin	
	(5)	P07585-5, DCN, Decorin	
375	(1)	Q14203-1,3,4,6, DCTN1, Dynactin subunit 1	1/0/1
	(2)	Q14203-2,5, DCTN1, Dynactin subunit 1	
376		Q16531-1,2, DDB1, DNA damage-binding protein 1	2/0/0
377		P39656-1,3, DDOST, Dolichyl-diphosphooligosaccharide--protein glycosyltransferase 48 kDa subunit	3/0/0
378	(1)	Q92499-1,2, DDX1, ATP-dependent RNA helicase DDX1	4/0/1
	(2)	Q92499-3, DDX1, ATP-dependent RNA helicase DDX1	
379		Q92841-1-4, DDX17, Probable ATP-dependent RNA helicase DDX17	1/0/2
380		Q9NUU7-1,2, DDX19A, ATP-dependent RNA helicase DDX19A<br>Q9UMR2-1-4, DDX19B, ATP-dependent RNA helicase DDX19B	2/0/0
381	(1)	O00148-1, DDX39A, ATP-dependent RNA helicase DDX39A	0/1/6
	(2)	O00148-2, DDX39A, ATP-dependent RNA helicase DDX39A	
	(3)	O00148-3, DDX39A, ATP-dependent RNA helicase DDX39A	
382		Q13838-1,2, DDX39B, Spliceosome RNA helicase DDX39B	3/0/5
383		P17844-1,2, DDX5, Probable ATP-dependent RNA helicase DDX5	1/0/2
384		P26196, DDX6, Probable ATP-dependent RNA helicase DDX6	1/0/0
385		P59665, DEFA1, Neutrophil defensin 1	2/0/3
386		P59666, DEFA3, Neutrophil defensin 3	3/0/3
387		P12838, DEFA4, Neutrophil defensin 4	2/0/0
388		Q01523, DEFA5, Defensin-5	3/0/0
389		Q01524, DEFA6, Defensin-6	1/0/0
390		P35659-1, DEK, Protein DEK	4/0/0
391		P17661, DES, Desmin	9/0/7

392 (1) O60443-1, DFNA5, Non-syndromic hearing impairment protein 5 — 4/1/0
(2) O60443-2, DFNA5, Non-syndromic hearing impairment protein 5
(3) O60443-3, DFNA5, Non-syndromic hearing impairment protein 5

393 O43143, DHX15, Pre-mRNA-splicing factor ATP-dependent RNA helicase DHX15 — 1/0/0

394 Q08211-1, DHX9, ATP-dependent RNA helicase A — 5/0/0

395 O60610-1-3, DIAPH1, Protein diaphanous homolog 1 — 2/0/1

396 Q9UBP4, DKK3, Dickkopf-related protein 3 — 10/0/0

397 P36957-1,2, DLST, Dihydrolipoyllysine-residue succinyltransferase component of 2-oxoglutarate dehydrogenase complex, mitochondrial — 2/0/0

398 (1) Q6E0U4-5, DMKN, Dermokine — 1/0/0
(2) Q6E0U4-1-4,6,7,16, DMKN, Dermokine

399 Q13316-1,2, DMP1, Dentin matrix acidic phosphoprotein 1 — 3/0/0

400 Q8WXX0-1, DNAH7, Dynein heavy chain 7, axonemal — 6/0/1

401 O60884, DNAJA2, DnaJ homolog subfamily A member 2 — 4/0/0

402 P25685-1, DNAJB1, DnaJ homolog subfamily B member 1 — 3/0/0

403 Q9UBS4, DNAJB11, DnaJ homolog subfamily B member 11 — 4/0/0

404 Q13217, DNAJC3, DnaJ homolog subfamily C member 3 — 16/0/1

405 P50570-1-5, DNM2, Dynamin-2 — 3/0/0

406 O43598-1,2, DNPH1, 2'-deoxynucleoside 5'-phosphate N-hydrolase 1 — 1/0/0

407 (1) Q96BY6-1,3, DOCK10, Dedicator of cytokinesis protein 10 — 1/0/2
(2) Q96BY6-2, DOCK10, Dedicator of cytokinesis protein 10

408 P27487, DPP4, Dipeptidyl peptidase 4 — 3/0/0

409 Q07507, DPT, Dermatopontin — 10/0/0

410 (1) Q16555-1,2, DPYSL2, Dihydropyrimidinase-related protein 2 — 3/0/2
(2) Q14194-1,2, CRMP1, Dihydropyrimidinase-related protein 1

411 (1) Q14195-2, DPYSL3, Dihydropyrimidinase-related protein 3 — 1/0/2
(2) Q14195-1, DPYSL3, Dihydropyrimidinase-related protein 3

412 (1) Q08554-1, DSC1, Desmocollin-1 — 1/0/0
(2) Q08554-2, DSC1, Desmocollin-1

413 Q02487-1,2, DSC2, Desmocollin-2 — 1/0/1

		Protein	Counts
414		Q14574-1,2, DSC3, Desmocollin-3	5/0/1
415	(1)	Q02413-1, DSG1, Desmoglein-1	8/0/0
	(2)	P32926, DSG3, Desmoglein-3	
	·	Q86SJ6-1,2, DSG4, Desmoglein-4	
416		Q14126, DSG2, Desmoglein-2	1/0/0
417	(1)	P15924-1, DSP, Desmoplakin	6/22/0
	(2)	P15924-2, DSP, Desmoplakin	
	(3)	P15924-3, DSP, Desmoplakin	
418		Q03001-3, DST, Dystonin	4/0/2
419		Q03001-7,8, DST, Dystonin	0/0/2
420		P60981-1,2, DSTN, Destrin	4/0/1
421		Q14204, DYNC1H1, Cytoplasmic dynein 1 heavy chain 1	20/0/0
422		Q13409-1-3,5-7, DYNC1I2, Cytoplasmic dynein 1 intermediate chain 2	1/0/0
423		O43237-1,2, DYNC1LI2, Cytoplasmic dynein 1 light intermediate chain 2	3/0/0
424		Q8NCM8-1,2, DYNC2H1, Cytoplasmic dynein 2 heavy chain 1	1/0/1
425		Q13011, ECH1, Delta(3,5)-Delta(2,4)-dienoyl-CoA isomerase, mitochondrial	2/0/0
426	(1)	Q16610-1,4, ECM1, Extracellular matrix protein 1	8/6/0
	(2)	Q16610-2, ECM1, Extracellular matrix protein 1	
	(3)	Q16610-3, ECM1, Extracellular matrix protein 1	
427	(1)	O94769-1, ECM2, Extracellular matrix protein 2	2/0/0
	(2)	O94769-2, ECM2, Extracellular matrix protein 2	
428	(1)	P68104-1, EEF1A1, Elongation factor 1-alpha 1	0/5/0
	·	Q5VTE0, EEF1A1P5, Putative elongation factor 1-alpha-like 3	
	(2)	P68104-2, EEF1A1, Elongation factor 1-alpha 1	
	(3)	Q05639, EEF1A2, Elongation factor 1-alpha 2	
429		P24534, EEF1B2, Elongation factor 1-beta	4/0/1
430	(1)	P29692-1-3, EEF1D, Elongation factor 1-delta	1/0/1
	(2)	P29692-4, EEF1D, Elongation factor 1-delta	
431		P26641-1,2, EEF1G, Elongation factor 1-gamma	11/0/0
432		P13639, EEF2, Elongation factor 2	24/0/0
433		Q12805-1-4, EFEMP1, EGF-containing fibulin-like extracellular matrix protein 1	4/0/22

434		Q12805-5, EFEMP1, EGF-containing fibulin-like extracellular matrix protein 1	2/0/22
435		O95967, EFEMP2, EGF-containing fibulin-like extracellular matrix protein 2	18/0/0
436	(1)	Q9H4M9, EHD1, EH domain-containing protein 1	7/1/1
	(2)	Q9NZN3-1, EHD3, EH domain-containing protein 3	
	(3)	Q9NZN3-2, EHD3, EH domain-containing protein 3	
			3/0/1
437	(1)	Q9NZN4-1, EHD2, EH domain-containing protein 2	
	(2)	Q9NZN4-2, EHD2, EH domain-containing protein 2	
438		Q9H223, EHD4, EH domain-containing protein 4	3/0/0
439		O14602, EIF1AY, Eukaryotic translation initiation factor 1A, Y-chromosomal	2/0/0
		P47813, EIF1AX, Eukaryotic translation initiation factor 1A, X-chromosomal	
440		P05198, EIF2S1, Eukaryotic translation initiation factor 2 subunit 1	2/0/0
441		Q14152-1,2, EIF3A, Eukaryotic translation initiation factor 3 subunit A	3/0/0
442		P55884-1,2, EIF3B, Eukaryotic translation initiation factor 3 subunit B	2/0/0
443	(1)	Q99613-1,2, EIF3C, Eukaryotic translation initiation factor 3 subunit C	1/0/0
	(2)	B5ME19, EIF3CL, Eukaryotic translation initiation factor 3 subunit C-like protein	
444		O00303, EIF3F, Eukaryotic translation initiation factor 3 subunit F	2/0/0
445		O15372, EIF3H, Eukaryotic translation initiation factor 3 subunit H	3/0/0
446		Q13347, EIF3I, Eukaryotic translation initiation factor 3 subunit I	2/0/0
			2/0/0
447	(1)	O75822-1,2, EIF3J, Eukaryotic translation initiation factor 3 subunit J	
	(2)	O75822-3, EIF3J, Eukaryotic translation initiation factor 3 subunit J	
448	(1)	P60842-1, EIF4A1, Eukaryotic initiation factor 4A-I	0/2/1
	(2)	Q14240-1,2, EIF4A2, Eukaryotic initiation factor 4A-II	
	(3)	P60842-2, EIF4A1, Eukaryotic initiation factor 4A-I	
449		P38919, EIF4A3, Eukaryotic initiation factor 4A-III	2/0/1
			1/0/0
450	(1)	Q04637-1,3-6,9, EIF4G1, Eukaryotic translation initiation factor 4 gamma 1	
	(2)	Q04637-7,8, EIF4G1, Eukaryotic translation initiation factor 4 gamma 1	
451		Q15056-1,2, EIF4H, Eukaryotic translation initiation factor 4H	4/0/0

452		P63241-1,2, EIF5A, Eukaryotic translation initiation factor 5A-1	3/0/0
453		P08246, ELANE, Neutrophil elastase	2/0/0
454	(1)	Q9Y6C2-1, EMILIN1, EMILIN-1	8/0/0
	(2)	Q9Y6C2-2, EMILIN1, EMILIN-1	
455		Q9BXX0, EMILIN2, EMILIN-2	17/0/0
456		P17813-1,2, ENG, Endoglin	1/0/0
457	(1)	P06733-1, ENO1, Alpha-enolase	4/0/0
	(2)	P06733-2, ENO1, Alpha-enolase	
458		P58107, EPPK1, Epiplakin	9/0/4
459		P84090, ERH, Enhancer of rudimentary homolog	2/0/0
460		O94905-1, ERLIN2, Erlin-2	2/0/0
461		Q9BS26, ERP44, Endoplasmic reticulum resident protein 44	9/0/0
462		P38117-1,2, ETFB, Electron transfer flavoprotein subunit beta	1/0/0
463		Q01844-1-3,5,6, EWSR1, RNA-binding protein EWS	2/0/0
464		P15311, EZR, Ezrin	15/0/6
465		P00742, F10, Coagulation factor X	19/0/1
466	(1)	P03951-1, F11, Coagulation factor XI	2/0/0
	(2)	P03951-2, F11, Coagulation factor XI	
467		Q9Y624-1, F11R, Junctional adhesion molecule A	3/0/0
468		P00748, F12, Coagulation factor XII	22/0/0
469		P00488, F13A1, Coagulation factor XIII A chain	24/0/0
470		P05160, F13B, Coagulation factor XIII B chain	15/0/0
471		P00734, F2, Prothrombin	39/0/0
472		P12259, F5, Coagulation factor V	24/0/0
473		P08709-1,2, F7, Coagulation factor VII	9/0/0
474		P00451-1, F8, Coagulation factor VIII	2/0/0
475	(1)	P00740-1, F9, Coagulation factor IX	1/0/1
	(2)	P00740-2, F9, Coagulation factor IX	
476		P05413, FABP3, Fatty acid-binding protein, heart	4/0/0
477	(1)	Q01469, FABP5, Fatty acid-binding protein, epidermal	1/0/0
	(2)	A8MUU1, FABP5P3, Putative fatty acid-binding protein 5-like protein 3	

478		Q0Z7S8, FABP9, Fatty acid-binding protein 9	2/0/0
479		Q8IWE2-1,2, FAM114A1, Protein NOXP20	4/0/0
480		Q96MK3, FAM20A, Pseudokinase FAM20A	3/0/0
481		Q92520, FAM3C, Protein FAM3C	2/0/0
482		Q9NUQ9-1, FAM49B, Protein FAM49B	5/0/0
483		Q12884-1, FAP, Prolyl endopeptidase FAP	1/0/1
484		Q9Y285-1,2, FARSA, Phenylalanine--tRNA ligase alpha subunit	3/0/0
485	(1)	Q9NSD9-1, FARSB, Phenylalanine--tRNA ligase beta subunit	1/0/1
	(2)	Q9NSD9-2, FARSB, Phenylalanine--tRNA ligase beta subunit	
486		Q14517, FAT1, Protocadherin Fat 1	2/0/0
487		P22087, FBL, rRNA 2'-O-methyltransferase fibrillarin	1/0/0
488	(1)	P23142-1, FBLN1, Fibulin-1	10/0/23
	(2)	P23142-2, FBLN1, Fibulin-1	
489		P23142-3, FBLN1, Fibulin-1	1/0/23
490		P23142-4, FBLN1, Fibulin-1	6/0/24
491	(1)	P98095-2, FBLN2, Fibulin-2	1/0/0
	(2)	P98095-1, FBLN2, Fibulin-2	
492		Q9UBX5, FBLN5, Fibulin-5	16/0/0
493	(1)	Q53RD9-1, FBLN7, Fibulin-7	0/4/0
	(2)	Q53RD9-2,4, FBLN7, Fibulin-7	
	(3)	Q53RD9-3, FBLN7, Fibulin-7	
494		P35555, FBN1, Fibrillin-1 [Cleaved into: Asprosin]	86/0/5
495	(1)	P35556-1, FBN2, Fibrillin-2 [Cleaved into: Fibrillin-2 C-terminal peptide]	8/0/6
	(2)	P35556-2, FBN2, Fibrillin-2 [Cleaved into: Fibrillin-2 C-terminal peptide]	
496		Q9UK22, FBXO2, F-box only protein 2	4/0/0
497		Q9Y6R7, FCGBP, IgGFc-binding protein	22/0/0
498		O00602, FCN1, Ficolin-1	2/0/1
499	(1)	Q15485-1, FCN2, Ficolin-2	1/0/1
	(2)	Q15485-2, FCN2, Ficolin-2	

500		O75636-1, FCN3, Ficolin-3	2/0/7
501		O75636-2, FCN3, Ficolin-3	0/0/8
502		Q86UX7-1,2, FERMT3, Fermitin family homolog 3	7/0/0
503	(1)	Q9UGM5-1, FETUB, Fetuin-B	3/0/0
	(2)	Q9UGM5-2, FETUB, Fetuin-B	
504	(1)	P02671-1, FGA, Fibrinogen alpha chain [Cleaved into: Fibrinopeptide A; Fibrinogen alpha chain]	5/0/0
	(2)	P02671-2, FGA, Fibrinogen alpha chain [Cleaved into: Fibrinopeptide A; Fibrinogen alpha chain]	
505		P02675, FGB, Fibrinogen beta chain [Cleaved into: Fibrinopeptide B; Fibrinogen beta chain]	46/0/0
506		P02679-1,2, FGG, Fibrinogen gamma chain	34/0/0
507		Q8TAL6, FIBIN, Fin bud initiation factor homolog	2/0/0
508		Q9Y3D6, FIS1, Mitochondrial fission 1 protein	3/0/0
509		P62942, FKBP1A, Peptidyl-prolyl cis-trans isomerase FKBP1A	1/0/0
510		P20930, FLG, Filaggrin	4/0/0
511		Q5D862, FLG2, Filaggrin-2	2/0/0
512		Q13045-1-3, FLII, Protein flightless-1 homolog	4/0/0
513		P21333-1,2, FLNA, Filamin-A	61/0/10
514	(1)	O75369-1-3,6,8,9, FLNB, Filamin-B	0/7/6
	(2)	O75369-7, FLNB, Filamin-B	
	(3)	O75369-4,5, FLNB, Filamin-B	
515		Q14315-1,2, FLNC, Filamin-C	35/0/9
516		P35916-1-3, FLT4, Vascular endothelial growth factor receptor 3	1/0/0
517		Q06828, FMOD, Fibromodulin	11/0/0
518	(1)	P02751-1,3,17, FN1, Fibronectin	0/89/0
	(2)	P02751-2, FN1, Fibronectin	
	(3)	P02751-4, FN1, Fibronectin	

(4) P02751-5, FN1, Fibronectin
(5) P02751-6, FN1, Fibronectin
(6) P02751-8,9,14, FN1, Fibronectin
(7) P02751-7,15, FN1, Fibronectin
(8) P02751-10, FN1, Fibronectin
(9) P02751-11, FN1, Fibronectin
(10) P02751-12, FN1, Fibronectin
(11) P02751-13, FN1, Fibronectin
(12) P02751-16, FN1, Fibronectin

519 Q9HA64, FN3KRP, Ketosamine-3-kinase — 1/0/0

520 Q4ZHG4-1,2, FNDC1, Fibronectin type III domain-containing protein 1 — 7/0/0

521 Q6PIV2-1, FOXR1, Forkhead box protein R1 — 1/0/1

522 Q70Z53-1-5, FRA10AC1, Protein FRA10AC1 — 3/0/0

523 (1) Q86XX4-2, FRAS1, Extracellular matrix protein FRAS1 — 1/11/0
(2) Q86XX4-1, FRAS1, Extracellular matrix protein FRAS1
(3) Q86XX4-4, FRAS1, Extracellular matrix protein FRAS1
(4) Q86XX4-5, FRAS1, Extracellular matrix protein FRAS1
(5) Q86XX4-6, FRAS1, Extracellular matrix protein FRAS1

524 Q5SYB0-1, FRMPD1, FERM and PDZ domain-containing protein 1 — 0/0/2

525 Q92765, FRZB, Secreted frizzled-related protein 3 — 3/0/0

526 Q16658, FSCN1, Fascin — 2/0/0

527 P19883-1,2, FST, Follistatin — 11/0/0

528 (1) Q12841-1, FSTL1, Follistatin-related protein 1 — 2/0/0
(2) Q12841-2, FSTL1, Follistatin-related protein 1

529 O95633-1,2, FSTL3, Follistatin-related protein 3 — 3/0/0

530 P02794, FTH1, Ferritin heavy chain — 2/0/0

531 Q96AE4-1,2, FUBP1, Far upstream element-binding protein 1 — 3/0/1

532 P35637-1,2, FUS, RNA-binding protein FUS — 4/0/2

533 P51114-1-3, FXR1, Fragile X mental retardation syndrome-related protein 1 — 3/0/0

534 Q13283-1, G3BP1, Ras GTPase-activating protein-binding protein 1 — 1/0/0

535 P11413-1-3, G6PD, Glucose-6-phosphate 1-dehydrogenase — 6/0/0

536 Q96RP7-1,2, GAL3ST4, Galactose-3-O-sulfotransferase 4 — 1/0/0

537 P51570-1,2, GALK1, Galactokinase 3/0/0

538 (1) Q10471-1, GALNT2, Polypeptide N-acetylgalactosaminyltransferase 2 1/0/0
(2) Q10471-2, GALNT2, Polypeptide N-acetylgalactosaminyltransferase 2

539 Q14697-1, GANAB, Neutral alpha-glucosidase AB 2/0/21

540 Q14697-2, GANAB, Neutral alpha-glucosidase AB 1/0/21

541 Q14697-3, GANAB, Neutral alpha-glucosidase AB 1/0/1

542 (1) P04406-1, GAPDH, Glyceraldehyde-3-phosphate dehydrogenase 2/0/0
(2) P04406-2, GAPDH, Glyceraldehyde-3-phosphate dehydrogenase

543 P54826, GAS1, Growth arrest-specific protein 1 10/0/0

544 (1) Q14393-1-3, GAS6, Growth arrest-specific protein 6 4/10/0
(2) Q14393-4, GAS6, Growth arrest-specific protein 6
(3) Q14393-5, GAS6, Growth arrest-specific protein 6

545 (1) P04062-1,2,4,5, GBA, Glucosylceramidase 1/0/0
(2) P04062-3, GBA, Glucosylceramidase

546 (1) P02774-1,3, GC, Vitamin D-binding protein 10/0/0
(2) P02774-2, GC, Vitamin D-binding protein

547 P01275, GCG, Glucagon [Cleaved into: Glicentin; Glicentin-related polypeptide 6/0/0

548 Q99988, GDF15, Growth/differentiation factor 15 6/0/0

549 Q7Z4P5, GDF7, Growth/differentiation factor 7 2/0/0

550 P14136-1-3, GFAP, Glial fibrillary acidic protein 1/0/2

551 Q06210-1,2, GFPT1, Glutamine--fructose-6-phosphate aminotransferase [isomerizing] 1 2/0/0

552 O94808, GFPT2, Glutamine--fructose-6-phosphate aminotransferase [isomerizing] 2 3/0/0

553 O75223-1,3, GGCT, Gamma-glutamylcyclotransferase 1/0/0

554 Q9UBU3-1-5, GHRL, Appetite-regulating hormone 2/0/0

555 P09681, GIP, Gastric inhibitory polypeptide 2/0/0

556 O14908-1,2, GIPC1, PDZ domain-containing protein GIPC1 1/0/0

557 Q9NS71, GKN1, Gastrokine-1 6/0/0

558 Q86XP6, GKN2, Gastrokine-2 1/0/0

559 P06280, GLA, Alpha-galactosidase A — 4/0/0

560 (1) P16278-1,3, GLB1, Beta-galactosidase — 1/0/0
(2) P16278-2, GLB1, Beta-galactosidase

561 Q92896-1-3, GLG1, Golgi apparatus protein 1 — 5/0/0

562 P17900, GM2A, Ganglioside GM2 activator — 6/0/0

563 Q96IJ6-1,2, GMPPA, Mannose-1-phosphate guanyltransferase alpha — 3/0/0

564 Q9Y5P6-1,2, GMPPB, Mannose-1-phosphate guanyltransferase beta — 2/0/0

565 P04899-1-6, GNAI2, Guanine nucleotide-binding protein G(i) subunit alpha-2 — 1/0/0

566 (1) P62873-1,2, GNB1, Guanine nucleotide-binding protein G(I)/G(S)/G(T) subunit beta-1 — 1/0/2
(2) P16520-1,2, GNB3, Guanine nucleotide-binding protein G(I)/G(S)/G(T) subunit beta-3

567 (1) P62879-1, GNB2, Guanine nucleotide-binding protein G(I)/G(S)/G(T) subunit beta-2 — 0/1/2
(2) P62879-2, GNB2, Guanine nucleotide-binding protein G(I)/G(S)/G(T) subunit beta-2
(3) Q9HAV0, GNB4, Guanine nucleotide-binding protein subunit beta-4

568 Q9UJJ9, GNPTG, N-acetylglucosamine-1-phosphotransferase subunit gamma — 4/0/0

569 H0YM25, GOLGA6L22, Golgin subfamily A member 6-like protein 22 — 7/0/0

570 Q14789-1-4, GOLGB1, Golgin subfamily B member 1 — 1/0/1

571 O00461, GOLIM4, Golgi integral membrane protein 4 — 10/0/0

572 Q8NBJ4-1,2, GOLM1, Golgi membrane protein 1 — 14/0/0

573 P80108-1, GPLD1, Phosphatidylinositol-glycan-specific phospholipase D — 15/0/0

574 Q13098-4-7, GPS1, COP9 signalosome complex subunit 1 — 2/0/0

575 P22352, GPX3, Glutathione peroxidase 3 — 10/0/0

576 O60565-1,2, GREM1, Gremlin-1 — 1/0/0

577 Q9ULK0-1, GRID1, Glutamate receptor ionotropic, delta-1 — 1/0/2

578 (1) P28799-1, GRN, Granulins — 0/4/0
(2) P28799-2, GRN, Granulins
(3) P28799-3, GRN, Granulins

579 P57764, GSDMD, Gasdermin-D — 2/0/0

580 (1) P06396-1, GSN, Gelsolin — 3/0/42

	(2)	P06396-3,4, GSN, Gelsolin	
581		P06396-2, GSN, Gelsolin	1/0/42
582	(1)	P09488-1,2, GSTM1, Glutathione S-transferase Mu 1	0/0/4
	(2)	Q03013-1,2, GSTM4, Glutathione S-transferase Mu 4	
	(3)	Q03013-3, GSTM4, Glutathione S-transferase Mu 4	
583	(1)	P28161-1, GSTM2, Glutathione S-transferase Mu 2	0/0/3
	(2)	P28161-2, GSTM2, Glutathione S-transferase Mu 2	
584		P21266, GSTM3, Glutathione S-transferase Mu 3	6/0/1
585		P46439, GSTM5, Glutathione S-transferase Mu 5	0/0/3
586	(1)	P78417-1,2, GSTO1, Glutathione S-transferase omega-1	1/0/0
	(2)	P78417-3, GSTO1, Glutathione S-transferase omega-1	
587		P09211, GSTP1, Glutathione S-transferase P	7/0/0
588		Q02747, GUCA2A, Guanylin	2/0/0
589		P07305-1,2, H1F0, Histone H1.0	3/0/0
590		Q92522, H1FX, Histone H1x	4/0/0
591		Q9BTM1-1, H2AFJ, Histone H2A.J	1/0/4
592	(1)	P0C0S5, H2AFZ, Histone H2A.Z	0/0/2
	·	Q71UI9-1,2, H2AFV, Histone H2A.V	
	(2)	Q71UI9-3,4, H2AFV, Histone H2A.V	
593	(1)	P16104, H2AFX, Histone H2AX	6/0/3
	(2)	P04908, HIST1H2AB, Histone H2A type 1-B/E	
	·	P20671, HIST1H2AD, Histone H2A type 1-D	
	·	Q7L7L0, HIST3H2A, Histone H2A type 3	
	·	Q96QV6, HIST1H2AA, Histone H2A type 1-A	
	·	Q9BTM1-2, H2AFJ, Histone H2A.J	
594		P84243, H3F3A, Histone H3.3	1/0/7
595		Q14520-1,2, HABP2, Hyaluronan-binding protein 2	16/0/0
596		Q6Y1H2, HACD2, Very-long-chain	1/0/0
597		P40939-1, HADHA, Trifunctional enzyme subunit alpha, mitochondrial	5/0/0
598		P55084-1, HADHB, Trifunctional enzyme subunit beta, mitochondrial	2/0/0
599		P81172, HAMP, Hepcidin	2/0/0
600	(1)	P68871, HBB, Hemoglobin subunit beta	2/0/0
	(2)	P02042, HBD, Hemoglobin subunit delta	
601		P14317-1, HCLS1, Hematopoietic lineage cell-specific protein	2/0/0
602		P51858-1-3, HDGF, Hepatoma-derived growth factor	8/0/0

603		Q00341-1,2, HDLBP, Vigilin	10/0/0
604		Q9NRV9, HEBP1, Heme-binding protein 1	2/0/0
605		O95714, HERC2, E3 ubiquitin-protein ligase HERC2	4/0/0
606	(1)	P06865-1, HEXA, Beta-hexosaminidase subunit alpha	2/0/0
	(2)	P06865-2, HEXA, Beta-hexosaminidase subunit alpha	
607		P07686, HEXB, Beta-hexosaminidase subunit beta	4/0/0
608		O14964-1,2, HGS, Hepatocyte growth factor-regulated tyrosine kinase substrate	3/0/0
609		P49773, HINT1, Histidine triad nucleotide-binding protein 1	3/0/0
610		P16401, HIST1H1B, Histone H1.5	7/0/5
611		P16403, HIST1H1C, Histone H1.2	5/0/10
612		P16402, HIST1H1D, Histone H1.3	2/0/9
613	(1)	P10412, HIST1H1E, Histone H1.4	6/0/11
	(2)	P22492, HIST1H1T, Histone H1t	
	(3)	Q02539, HIST1H1A, Histone H1.1	
614		P0C0S8, HIST1H2AG, Histone H2A type 1	
		Q6FI13, HIST2H2AA3, Histone H2A type 2-A	1/0/4
		Q93077, HIST1H2AC, Histone H2A type 1-C	
		Q96KK5, HIST1H2AH, Histone H2A type 1-H	
		Q99878, HIST1H2AJ, Histone H2A type 1-J	
615	(1)	P06899, HIST1H2BJ, Histone H2B type 1-J	
	·	P23527, HIST1H2BO, Histone H2B type 1-O	
	·	P33778, HIST1H2BB, Histone H2B type 1-B	0/0/12
	·	Q16778, HIST2H2BE, Histone H2B type 2-E	
	(2)	Q6DN03, HIST2H2BC, Putative histone H2B type 2-C	
	·	Q6DRA6, HIST2H2BD, Putative histone H2B type 2-D	
616	(1)	O60814, HIST1H2BK, Histone H2B type 1-K	
	·	P58876, HIST1H2BD, Histone H2B type 1-D	
	·	P62807, HIST1H2BC, Histone H2B type 1-C/E/F/G/I	0/0/12
	·	Q5QNW6-1,2, HIST2H2BF, Histone H2B type 2-F	
	·	Q93079, HIST1H2BH, Histone H2B type 1-H	
	·	Q99877, HIST1H2BN, Histone H2B type 1-N	
	(2)	Q99879, HIST1H2BM, Histone H2B type 1-M	
	(3)	P57053, H2BFS, Histone H2B type F-S	
617	(1)	Q99880, HIST1H2BL, Histone H2B type 1-L	0/0/12
	(2)	Q96A08, HIST1H2BA, Histone H2B type 1-A	
618	(1)	P68431, HIST1H3A, Histone H3.1	
	·	Q16695, HIST3H3, Histone H3.1t	1/0/7
	·	Q71DI3, HIST2H3A;, Histone H3.2	
	(2)	Q6NXT2, H3F3C, Histone H3.3C	
619		P62805, HIST1H4A, Histone H4	7/0/0

620		Q8IUE6, HIST2H2AB, Histone H2A type 2-B	1/0/2
621		Q16777, HIST2H2AC, Histone H2A type 2-C	1/0/4
622		Q8N257, HIST3H2BB, Histone H2B type 3-B	0/0/11
623		P05534, HLA-A, HLA class I histocompatibility antigen, A-24 alpha chain	1/0/7
624		P10314, HLA-A, HLA class I histocompatibility antigen, A-32 alpha chain	0/0/6
		P30459, HLA-A, HLA class I histocompatibility antigen, A-74 alpha chain	
625	(1)	P10316, HLA-A, HLA class I histocompatibility antigen, A-69 alpha chain	0/0/4
	(2)	P01892, HLA-A, HLA class I histocompatibility antigen, A-2 alpha chain	
626	(1)	P13746-1,2, HLA-A, HLA class I histocompatibility antigen, A-11 alpha chain	0/0/6
	(2)	P04439, HLA-A, HLA class I histocompatibility antigen, A-3 alpha chain	
627		P16188, HLA-A, HLA class I histocompatibility antigen, A-30 alpha chain	0/0/6
628		P16190, HLA-A, HLA class I histocompatibility antigen, A-33 alpha chain	0/0/6
629		P30443, HLA-A, HLA class I histocompatibility antigen, A-1 alpha chain	0/0/6
		P30455, HLA-A, HLA class I histocompatibility antigen, A-36 alpha chain	
630		P30447, HLA-A, HLA class I histocompatibility antigen, A-23 alpha chain	0/0/8
631	(1)	P30450, HLA-A, HLA class I histocompatibility antigen, A-26 alpha chain	0/1/9
	(2)	P30453, HLA-A, HLA class I histocompatibility antigen, A-34 alpha chain	
	(3)	P30456, HLA-A, HLA class I histocompatibility antigen, A-43 alpha chain	
	(4)	P30457, HLA-A, HLA class I histocompatibility antigen, A-66 alpha chain	
	(5)	Q09160, HLA-A, HLA class I histocompatibility antigen, A-80 alpha chain	
	(6)	P18462, HLA-A, HLA class I histocompatibility antigen, A-25 alpha chain	
	(7)	P01891, HLA-A, HLA class I histocompatibility antigen, A-68 alpha chain	
632	(1)	P30512, HLA-A, HLA class I histocompatibility antigen, A-29 alpha chain	0/0/6
	(2)	P16189, HLA-A, HLA class I histocompatibility antigen, A-31 alpha chain	
633	(1)	P01889, HLA-B, HLA class I histocompatibility antigen, B-7 alpha chain	
	·	P30460, HLA-B, HLA class I histocompatibility antigen, B-8 alpha chain	0/0/8

· P30480, HLA-B, HLA class I histocompatibility antigen, B-42 alpha chain

(2) P30486, HLA-B, HLA class I histocompatibility antigen, B-48 alpha chain

· Q31610, HLA-B, HLA class I histocompatibility antigen, B-81 alpha chain

634 (1) P10319, HLA-B, HLA class I histocompatibility antigen, B-58 alpha chain — 0/0/8

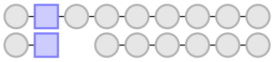

(2) P18465, HLA-B, HLA class I histocompatibility antigen, B-57 alpha chain

635 (1) P18463, HLA-B, HLA class I histocompatibility antigen, B-37 alpha chain

(2) P30492, HLA-B, HLA class I histocompatibility antigen, B-54 alpha chain — 1/0/6

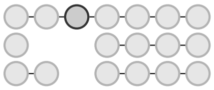

(3) P03989, HLA-B, HLA class I histocompatibility antigen, B-27 alpha chain

· P30462, HLA-B, HLA class I histocompatibility antigen, B-14 alpha chain

· P30493, HLA-B, HLA class I histocompatibility antigen, B-55 alpha chain

· Q29718, HLA-B, HLA class I histocompatibility antigen, B-82 alpha chain

· Q29940, HLA-B, HLA class I histocompatibility antigen, B-59 alpha chain

636 (1) P30464, HLA-B, HLA class I histocompatibility antigen, B-15 alpha chain

· P30484, HLA-B, HLA class I histocompatibility antigen, B-46 alpha chain

(2) P30466, HLA-B, HLA class I histocompatibility antigen, B-18 alpha chain — 0/0/10

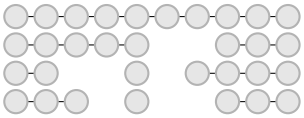

(3) P18464, HLA-B, HLA class I histocompatibility antigen, B-51 alpha chain

· P30490, HLA-B, HLA class I histocompatibility antigen, B-52 alpha chain

· P30495, HLA-B, HLA class I histocompatibility antigen, B-56 alpha chain

· P30498, HLA-B, HLA class I histocompatibility antigen, B-78 alpha chain

(4) P30475, HLA-B, HLA class I histocompatibility antigen, B-39 alpha chain

· Q29836, HLA-B, HLA class I histocompatibility antigen, B-67 alpha chain

· Q95365, HLA-B, HLA class I histocompatibility antigen, B-38 alpha chain

637 (1) P30479, HLA-B, HLA class I histocompatibility antigen, B-41 alpha chain — 0/0/9

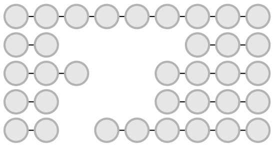

(2) P30461, HLA-B, HLA class I histocompatibility antigen, B-13 alpha chain

(3) P30481, HLA-B, HLA class I histocompatibility antigen, B-44 alpha chain

(4) P30485, HLA-B, HLA class I histocompatibility antigen, B-47 alpha chain

(5) Q04826, HLA-B, HLA class I histocompatibility antigen, B-40 alpha chain

638 (1) P30487, HLA-B, HLA class I histocompatibility antigen, B-49 alpha chain
· P30488, HLA-B, HLA class I histocompatibility antigen, B-50 alpha chain
(2) P30483, HLA-B, HLA class I histocompatibility antigen, B-45 alpha chain

0/0/8 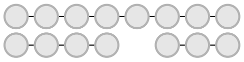

639 P30491, HLA-B, HLA class I histocompatibility antigen, B-53 alpha chain
P30685, HLA-B, HLA class I histocompatibility antigen, B-35 alpha chain

0/0/9 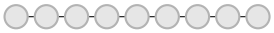

640 Q31612, HLA-B, HLA class I histocompatibility antigen, B-73 alpha chain

0/0/5 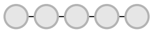

641 P04222, HLA-C, HLA class I histocompatibility antigen, Cw-3 alpha chain

0/0/7 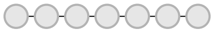

642 (1) P30501, HLA-C, HLA class I histocompatibility antigen, Cw-2 alpha chain
(2) P30499, HLA-C, HLA class I histocompatibility antigen, Cw-1 alpha chain
(3) P30505, HLA-C, HLA class I histocompatibility antigen, Cw-8 alpha chain
(4) P30510, HLA-C, HLA class I histocompatibility antigen, Cw-14 alpha chain
(5) Q29960-1,2, HLA-C, HLA class I histocompatibility antigen, Cw-16 alpha chain
(6) P17693, HLA-G, HLA class I histocompatibility antigen, alpha chain G
(7) P01893, HLA-H, Putative HLA class I histocompatibility antigen, alpha chain H
(8) Q9TNN7, HLA-C, HLA class I histocompatibility antigen, Cw-5 alpha chain
(9) Q07000, HLA-C, HLA class I histocompatibility antigen, Cw-15 alpha chain

1/0/8 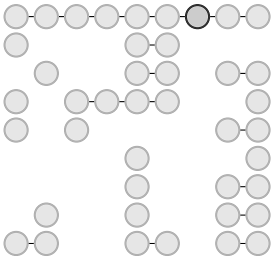

643 (1) P30508, HLA-C, HLA class I histocompatibility antigen, Cw-12 alpha chain
(2) Q95604, HLA-C, HLA class I histocompatibility antigen, Cw-17 alpha chain
(3) P10321, HLA-C, HLA class I histocompatibility antigen, Cw-7 alpha chain
(4) P30504, HLA-C, HLA class I histocompatibility antigen, Cw-4 alpha chain
· Q29865, HLA-C, HLA class I histocompatibility antigen, Cw-18 alpha chain
(5) Q29963, HLA-C, HLA class I histocompatibility antigen, Cw-6 alpha chain

0/1/9 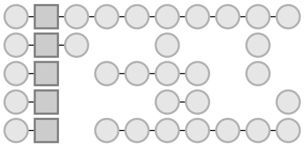

644 (1) P01903, HLA-DRA, HLA class II histocompatibility antigen, DR alpha chain
(2) P01906, HLA-DQA2, HLA class II histocompatibility antigen, DQ alpha 2 chain

1/0/0 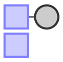

645 (1) P04229, HLA-DRB1, HLA class II histocompatibility antigen, DRB1-1 beta chain
· Q29974, HLA-DRB1, HLA class II histocompatibility antigen, DRB1-16 beta chain

0/2/0 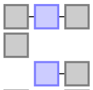

	·	Q30134, HLA-DRB1, HLA class II histocompatibility antigen, DRB1-8 beta chain	
	·	Q9GIY3, HLA-DRB1, HLA class II histocompatibility antigen, DRB1-14 beta chain	
	(2)	Q30154, HLA-DRB5, HLA class II histocompatibility antigen, DR beta 5 chain	
	(3)	P01911, HLA-DRB1, HLA class II histocompatibility antigen, DRB1-15 beta chain	
	·	P01912, HLA-DRB1, HLA class II histocompatibility antigen, DRB1-3 chain	
	·	P13762, HLA-DRB4, HLA class II histocompatibility antigen, DR beta 4 chain	
	·	P20039, HLA-DRB1, HLA class II histocompatibility antigen, DRB1-11 beta chain	
	·	Q30167, HLA-DRB1, HLA class II histocompatibility antigen, DRB1-10 beta chain	
	·	Q5Y7A7, HLA-DRB1, HLA class II histocompatibility antigen, DRB1-13 beta chain	
	·	Q95IE3, HLA-DRB1, HLA class II histocompatibility antigen, DRB1-12 beta chain	
	(4)	P13760, HLA-DRB1, HLA class II histocompatibility antigen, DRB1-4 beta chain	
	·	P13761, HLA-DRB1, HLA class II histocompatibility antigen, DRB1-7 beta chain	
	·	P79483, HLA-DRB3, HLA class II histocompatibility antigen, DR beta 3 chain	
	·	Q9TQE0, HLA-DRB1, HLA class II histocompatibility antigen, DRB1-9 beta chain	
646		Q96RW7-1,2, HMCN1, Hemicentin-1	36/0/0
647	(1)	Q8NDA2-1, HMCN2, Hemicentin-2	4/16/0
	(2)	Q8NDA2-2, HMCN2, Hemicentin-2	
	(3)	Q8NDA2-4, HMCN2, Hemicentin-2	
648		P09429, HMGB1, High mobility group protein B1	1/0/2
649		P05204, HMGN2, Non-histone chromosomal protein HMG-17	3/0/1
650		O00479, HMGN4, High mobility group nucleosome-binding domain-containing protein 4	2/0/1
651		P50135-1, HNMT, Histamine N-methyltransferase	4/0/0
652		Q13151, HNRNPA0, Heterogeneous nuclear ribonucleoprotein A0	1/0/1
653	(1)	P09651-1-3, HNRNPA1, Heterogeneous nuclear ribonucleoprotein A1	1/0/4
	(2)	Q32P51, HNRNPA1L2, Heterogeneous nuclear ribonucleoprotein A1-like 2	
654	(1)	P22626-1, HNRNPA2B1, Heterogeneous nuclear ribonucleoproteins A2/B1	1/0/2
	(2)	P22626-2, HNRNPA2B1, Heterogeneous nuclear ribonucleoproteins A2/B1	

2/0/3 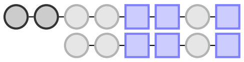

655 (1) P51991-1, HNRNPA3, Heterogeneous nuclear ribonucleoprotein A3

(2) P51991-2, HNRNPA3, Heterogeneous nuclear ribonucleoprotein A3

2/0/3 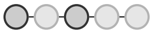

656 Q99729-1-4, HNRNPAB, Heterogeneous nuclear ribonucleoprotein A/B

0/6/3 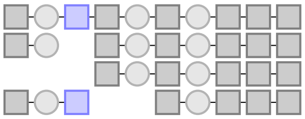

657 (1) P07910-2, HNRNPC, Heterogeneous nuclear ribonucleoproteins C1/C2

(2) P07910-1, HNRNPC, Heterogeneous nuclear ribonucleoproteins C1/C2

(3) P07910-3, HNRNPC, Heterogeneous nuclear ribonucleoproteins C1/C2

(4) P07910-4, HNRNPC, Heterogeneous nuclear ribonucleoproteins C1/C2

658 (1) B2RXH8, HNRNPCL2, Heterogeneous nuclear ribonucleoprotein C-like 2

1/0/3 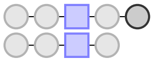

· O60812, HNRNPCL1, Heterogeneous nuclear ribonucleoprotein C-like 1

(2) B7ZW38, HNRNPCL3, Heterogeneous nuclear ribonucleoprotein C-like 3

· P0DMR1, HNRNPCL4, Heterogeneous nuclear ribonucleoprotein C-like 4

2/0/4 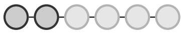

659 Q14103-1,3, HNRNPD, Heterogeneous nuclear ribonucleoprotein D0

1/0/4 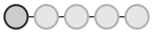

660 Q14103-2,4, HNRNPD, Heterogeneous nuclear ribonucleoprotein D0

3/0/1 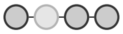

661 O14979-1-3, HNRNPDL, Heterogeneous nuclear ribonucleoprotein D-like

5/0/2 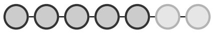

662 P52597, HNRNPF, Heterogeneous nuclear ribonucleoprotein F

3/0/3 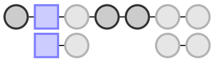

663 (1) P31943, HNRNPH1, Heterogeneous nuclear ribonucleoprotein H

(2) P55795, HNRNPH2, Heterogeneous nuclear ribonucleoprotein H2

1/2/1 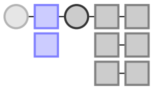

664 (1) P31942-1,2, HNRNPH3, Heterogeneous nuclear ribonucleoprotein H3

(2) P31942-3, HNRNPH3, Heterogeneous nuclear ribonucleoprotein H3

(3) P31942-4, HNRNPH3, Heterogeneous nuclear ribonucleoprotein H3

1/0/0 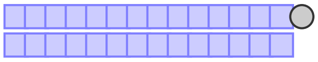

665 (1) P61978-2,3, HNRNPK, Heterogeneous nuclear ribonucleoprotein K

(2) P61978-1, HNRNPK, Heterogeneous nuclear ribonucleoprotein K

666	(1)	P14866-1, HNRNPL, Heterogeneous nuclear ribonucleoprotein L	3/0/0
	(2)	P14866-2, HNRNPL, Heterogeneous nuclear ribonucleoprotein L	
667		P52272-1,2, HNRNPM, Heterogeneous nuclear ribonucleoprotein M	3/0/0
668	(1)	O43390-1,2,4, HNRNPR, Heterogeneous nuclear ribonucleoprotein R	0/0/6
	(2)	O43390-3, HNRNPR, Heterogeneous nuclear ribonucleoprotein R	
669		Q00839-1, HNRNPU, Heterogeneous nuclear ribonucleoprotein U	2/0/9
670		Q00839-2, HNRNPU, Heterogeneous nuclear ribonucleoprotein U	1/0/9
671		Q9BUJ2-1-4, HNRNPUL1, Heterogeneous nuclear ribonucleoprotein U-like protein 1	2/0/1
672		Q1KMD3, HNRNPUL2, Heterogeneous nuclear ribonucleoprotein U-like protein 2	14/0/0
673	(1)	P00738-1, HP, Haptoglobin	1/0/13
	(2)	P00738-2, HP, Haptoglobin	
674	(1)	Q5SSJ5-1,2, HP1BP3, Heterochromatin protein 1-binding protein 3	1/0/0
	(2)	Q5SSJ5-3, HP1BP3, Heterochromatin protein 1-binding protein 3	
675		P00739-1,2, HPR, Haptoglobin-related protein	9/0/13
676		P02790, HPX, Hemopexin	12/0/0
677		P23327, HRC, Sarcoplasmic reticulum histidine-rich calcium-binding protein	8/0/0
678		P04196, HRG, Histidine-rich glycoprotein	23/0/0
679		Q86YZ3, HRNR, Hornerin	3/0/0
680		O60243-1,2, HS6ST1, Heparan-sulfate 6-O-sulfotransferase 1	1/0/0
681		Q99714-1,2, HSD17B10, 3-hydroxyacyl-CoA dehydrogenase type-2	1/0/0
682	(1)	P07900-1,2, HSP90AA1, Heat shock protein HSP 90-alpha	13/1/17
	(2)	Q58FG1, HSP90AA4P, Putative heat shock protein HSP 90-alpha A4	
	(3)	Q14568, HSP90AA2P, Heat shock protein HSP 90-alpha A2	
	(4)	Q12931-1,2, TRAP1, Heat shock protein 75 kDa, mitochondrial	
683		Q58FG0, HSP90AA5P, Putative heat shock protein HSP 90-alpha A5	1/0/6
684	(1)	P08238, HSP90AB1, Heat shock protein HSP 90-beta	8/0/19

	(2)	Q58FF7, HSP90AB3P, Putative heat shock protein HSP 90-beta-3	
685		Q58FF8, HSP90AB2P, Putative heat shock protein HSP 90-beta 2	1/0/8
686		Q58FF6, HSP90AB4P, Putative heat shock protein HSP 90-beta 4	1/0/5
687	(1)	P14625, HSP90B1, Endoplasmin	26/0/1
	(2)	Q58FF3, HSP90B2P, Putative endoplasmin-like protein	
688	(1)	P0DMV8-1, HSPA1A, Heat shock 70 kDa protein 1A	2/0/13
	·	P0DMV9, HSPA1B, Heat shock 70 kDa protein 1B	
	(2)	P0DMV8-2, HSPA1A, Heat shock 70 kDa protein 1A	
	(3)	P48741, HSPA7, Putative heat shock 70 kDa protein 7	
689		P34931, HSPA1L, Heat shock 70 kDa protein 1-like	1/0/11
690		P54652, HSPA2, Heat shock-related 70 kDa protein 2	8/0/10
691		P11021, HSPA5, 78 kDa glucose-regulated protein	25/0/3
692		P17066, HSPA6, Heat shock 70 kDa protein 6	0/0/6
693	(1)	P11142-1, HSPA8, Heat shock cognate 71 kDa protein	3/0/10
	(2)	P11142-2, HSPA8, Heat shock cognate 71 kDa protein	
694		P38646, HSPA9, Stress-70 protein, mitochondrial	7/0/1
695		P04792, HSPB1, Heat shock protein beta-1	4/0/0
696		O14558, HSPB6, Heat shock protein beta-6	3/0/0
697	(1)	P10809-1, HSPD1, 60 kDa heat shock protein, mitochondrial	5/0/0
	(2)	P10809-2, HSPD1, 60 kDa heat shock protein, mitochondrial	
698		P61604, HSPE1, 10 kDa heat shock protein, mitochondrial	1/0/0
699		P98160, HSPG2, Basement membrane-specific heparan sulfate proteoglycan core protein	93/0/0
700		Q92743, HTRA1, Serine protease HTRA1	15/0/1
701		Q7Z6Z7-1-3, HUWE1, E3 ubiquitin-protein ligase HUWE1	6/0/0
702		Q12794-1,3,5, HYAL1, Hyaluronidase-1	2/0/0
703	(1)	Q9Y4L1-1, HYOU1, Hypoxia up-regulated protein 1	4/0/0
	(2)	Q9Y4L1-2, HYOU1, Hypoxia up-regulated protein 1	
704		P13284, IFI30, Gamma-interferon-inducible lysosomal thiol reductase	5/0/0
705	(1)	P05019-1, IGF1, Insulin-like growth factor I	2/0/0
	(2)	P05019-2-4, IGF1, Insulin-like growth factor I	
706		P01344-1-3, IGF2, Insulin-like growth factor II	7/0/0

707		P11717, IGF2R, Cation-independent mannose-6-phosphate receptor	6/0/0
708		P35858-1,2, IGFALS, Insulin-like growth factor-binding protein complex acid labile subunit	16/0/0
709		P08833, IGFBP1, Insulin-like growth factor-binding protein 1	8/0/0
710		P18065, IGFBP2, Insulin-like growth factor-binding protein 2	16/0/0
711		P17936-1,2, IGFBP3, Insulin-like growth factor-binding protein 3	18/0/1
712	(1)	P22692-1, IGFBP4, Insulin-like growth factor-binding protein 4	1/0/1
	(2)	P22692-2, IGFBP4, Insulin-like growth factor-binding protein 4	
713		P24593, IGFBP5, Insulin-like growth factor-binding protein 5	12/0/0
714		P24592, IGFBP6, Insulin-like growth factor-binding protein 6	7/0/0
715	(1)	Q16270-1, IGFBP7, Insulin-like growth factor-binding protein 7	1/0/0
	(2)	Q16270-2, IGFBP7, Insulin-like growth factor-binding protein 7	
716		P01876, IGHA1, Ig alpha-1 chain C region	6/0/8
717		P01877, IGHA2, Ig alpha-2 chain C region	1/0/8
718		P01857, IGHG1, Ig gamma-1 chain C region	7/0/8
719		P01859, IGHG2, Ig gamma-2 chain C region	3/0/8
720		P01860, IGHG3, Ig gamma-3 chain C region	4/0/10
721		P01861, IGHG4, Ig gamma-4 chain C region	4/0/6
722	(1)	P01871-1, IGHM, Ig mu chain C region	0/0/11
	(2)	P01871-2, IGHM, Ig mu chain C region	
723		A0A0C4DH31, NA, NA<br>P23083, IGHV1-2, Immunoglobulin heavy variable 1-2	1/0/1
724		A0A0C4DH29, NA, NA<br>P01743, IGHV1-46, Immunoglobulin heavy variable 1-46	0/0/2
725		P01742, IGHV1-69, Immunoglobulin heavy variable 1-69	3/0/1
726		P01766, IGHV3-13, Immunoglobulin heavy variable 3-13	1/0/0
727	(1)	P01768, IGHV3-30, Immunoglobulin heavy variable 3-30	0/0/1
	(2)	P01764, IGHV3-23, Immunoglobulin heavy variable 3-23	
728		P01772, IGHV3-33, Immunoglobulin heavy variable 3-33	0/0/2
729	(1)	P01780, IGHV3-7, Immunoglobulin heavy variable 3-7	1/0/1

	(2)	A0A0B4J1V1, NA, NA	
	·	P01762, IGHV3-11, Immunoglobulin heavy variable 3-11	
	·	P01763, IGHV3-48, Immunoglobulin heavy variable 3-48	
	·	P01767, IGHV3-53, Immunoglobulin heavy variable 3-53	
730	(1)	P01824, IGHV4-39, Immunoglobulin heavy variable 4-39	1/0/0
	(2)	P01825, IGHV4-59, Immunoglobulin heavy variable 4-59	
	·	P06331, IGHV4-34, Immunoglobulin heavy variable 4-34	
731		P01834, IGKC, Ig kappa chain C region	6/0/0
732	(1)	P01599, IGKV1-17, Immunoglobulin kappa variable 1-17	2/0/1
	(2)	A0A0C4DH73, NA, NA	
	·	P01597, IGKV1-39, Immunoglobulin kappa variable 1-39	
	·	P01601, IGKV1D-16, Immunoglobulin kappa variable 1D-16	
	·	P01611, IGKV1D-12, Immunoglobulin kappa variable 1D-12	
	·	P04430, IGKV1-16, Immunoglobulin kappa variable 1-16	
	·	P04432, IGKV1D-39, Immunoglobulin kappa variable 1D-39	
733		P01602, IGKV1-5, Immunoglobulin kappa variable 1-5	1/0/1
734		P06310, IGKV2-30, Immunoglobulin kappa variable 2-30	1/0/1
735	(1)	P01614, IGKV2D-40, Immunoglobulin kappa variable 2D-40	1/0/1
	(2)	A0A075B6P5, NA, NA	
	·	P01615, IGKV2D-28, Immunoglobulin kappa variable 2D-28	
	(3)	A0A075B6S6, NA, NA	
736		P04433, IGKV3-11, Immunoglobulin kappa variable 3-11	1/0/2
737		P01624, IGKV3-15, Immunoglobulin kappa variable 3-15	2/0/2
738		P01619, IGKV3-20, Immunoglobulin kappa variable 3-20	1/0/3
739		P06312, IGKV4-1, Immunoglobulin kappa variable 4-1	4/0/0
740	(1)	P0CG05, IGLC2, Ig lambda-2 chain C regions	1/0/4
	(2)	P0CF74, IGLC6, Ig lambda-6 chain C region	
	(3)	P0CG06, IGLC3, Ig lambda-3 chain C regions	
741		A0M8Q6, IGLC7, Ig lambda-7 chain C region	1/0/3
742	(1)	B9A064-1, IGLL5, Immunoglobulin lambda-like polypeptide 5	1/0/4
	(2)	P0CG04, IGLC1, Ig lambda-1 chain C regions	
743	(1)	P01700, IGLV1-47, Immunoglobulin lambda variable 1-47	1/0/0
	(2)	P01699, IGLV1-44, Immunoglobulin lambda variable 1-44	
744		P01706, IGLV2-11, Immunoglobulin lambda variable 2-11	1/0/0
		P01709, IGLV2-8, Immunoglobulin lambda variable 2-8	
745		P01704, IGLV2-14, Immunoglobulin lambda variable 2-14	1/0/0
746		P01705, IGLV2-23, Immunoglobulin lambda variable 2-23	1/0/0
747		P01714, IGLV3-19, Immunoglobulin lambda variable 3-19	2/0/0
748		P80748, IGLV3-21, Immunoglobulin lambda variable 3-21	2/0/0
749		P01717, IGLV3-25, Immunoglobulin lambda variable 3-25	1/0/1

750		P01718, IGLV3-27, Immunoglobulin lambda variable 3-27	1/0/1
751		P04211, IGLV7-43, Immunoglobulin lambda variable 7-43	2/0/0
752		Q14116-1,2, IL18, Interleukin-18	2/0/0
753		Q12906-1-7, ILF3, Interleukin enhancer-binding factor 3	6/0/0
754		Q13418-1-3, ILK, Integrin-linked protein kinase	2/0/0
755		Q16891-1-4, IMMT, MICOS complex subunit MIC60	3/0/0
756		Q9NX62, IMPAD1, Inositol monophosphatase 3	5/0/0
757		Q9GZP8-1,2, IMUP, Immortalization up-regulated protein	1/0/0
758		Q27J81-1,2, INF2, Inverted formin-2	3/0/0
759		P09529, INHBB, Inhibin beta B chain	5/0/0
760		P55103, INHBC, Inhibin beta C chain	10/0/0
761		P58166, INHBE, Inhibin beta E chain	4/0/0
762		P51460-1, INSL3, Insulin-like 3	3/0/0
763		O00410-1,3, IPO5, Importin-5	2/0/0
764		P46940, IQGAP1, Ras GTPase-activating-like protein IQGAP1	22/0/0
765		O14498, ISLR, Immunoglobulin superfamily containing leucine-rich repeat protein	7/0/0
766		Q9UKX5-1,2, ITGA11, Integrin alpha-11	3/0/0
767		Q13683-1,3,7,9,10, ITGA7, Integrin alpha-7 [Cleaved into: Integrin alpha-7 heavy chain; Integrin alpha-7 light chain; Integrin alpha-7 70 kDa form]	2/0/0
768		P11215-1,2, ITGAM, Integrin alpha-M	3/0/1
769	(1)	P05556-1, ITGB1, Integrin beta-1	1/0/0
	(2)	P05556-2-5, ITGB1, Integrin beta-1	
770		P05107, ITGB2, Integrin beta-2	4/0/0
771	(1)	O95965-1, ITGBL1, Integrin beta-like protein 1	3/12/0
	(2)	O95965-2, ITGBL1, Integrin beta-like protein 1	
	(3)	O95965-3, ITGBL1, Integrin beta-like protein 1	
772	(1)	P19827-1, ITIH1, Inter-alpha-trypsin inhibitor heavy chain H1	7/11/1
	(2)	P19827-2, ITIH1, Inter-alpha-trypsin inhibitor heavy chain H1	
	(3)	P19827-3, ITIH1, Inter-alpha-trypsin inhibitor heavy chain H1	

773		P19823, ITIH2, Inter-alpha-trypsin inhibitor heavy chain H2	30/0/0
774		Q06033-1,2, ITIH3, Inter-alpha-trypsin inhibitor heavy chain H3	39/0/0
775		Q14624-1, ITIH4, Inter-alpha-trypsin inhibitor heavy chain H4	4/0/32
776	(1)	Q14624-2,3, ITIH4, Inter-alpha-trypsin inhibitor heavy chain H4	0/0/32
	(2)	Q14624-4, ITIH4, Inter-alpha-trypsin inhibitor heavy chain H4	
777	(1)	Q86UX2-1, ITIH5, Inter-alpha-trypsin inhibitor heavy chain H5	0/8/0
	(2)	Q86UX2-2, ITIH5, Inter-alpha-trypsin inhibitor heavy chain H5	
	(3)	Q86UX2-3, ITIH5, Inter-alpha-trypsin inhibitor heavy chain H5	
	(4)	Q86UX2-4, ITIH5, Inter-alpha-trypsin inhibitor heavy chain H5	
778		Q8WWA0, ITLN1, Intelectin-1	9/0/7
779		Q8WWU7-1,2, ITLN2, Intelectin-2	1/0/7
780		O43736-1,2, ITM2A, Integral membrane protein 2A	1/0/0
781		Q9Y287-1, ITM2B, Integral membrane protein 2B	2/0/0
782		P01591, JCHAIN, Immunoglobulin J chain	6/0/0
783		P14923, JUP, Junction plakoglobin	4/0/0
784	(1)	Q96CX2, KCTD12, BTB/POZ domain-containing protein KCTD12	5/0/1
	(2)	Q6ZWB6, KCTD8, BTB/POZ domain-containing protein KCTD8	
785		Q68DU8, KCTD16, BTB/POZ domain-containing protein KCTD16	3/0/1
786	(1)	Q07666-1,2, KHDRBS1, KH domain-containing, RNA-binding, signal transduction-associated protein 1	3/0/0
	(2)	Q5VWX1, KHDRBS2, KH domain-containing, RNA-binding, signal transduction-associated protein 2	
787		Q92945, KHSRP, Far upstream element-binding protein 2	3/0/1
788		Q8IZA0-1,2, KIAA0319L, Dyslexia-associated protein KIAA0319-like protein	4/0/0
789	(1)	P33176, KIF5B, Kinesin-1 heavy chain	5/0/0
	(2)	O60282-1,2, KIF5C, Kinesin heavy chain isoform 5C	
790		Q2M1P5, KIF7, Kinesin-like protein KIF7	0/0/2
791		O43240, KLK10, Kallikrein-10	2/0/0

792	(1)	P03952, KLKB1, Plasma kallikrein	31/0/0
	(2)	P20718-1, GZMH, Granzyme H	
793		Q8NEZ4-1,3, KMT2C, Histone-lysine N-methyltransferase 2C	14/0/0
794		P01042-1, KNG1, Kininogen-1	12/0/29
795	(1)	P01042-2, KNG1, Kininogen-1	0/0/29
	(2)	P01042-3, KNG1, Kininogen-1	
796	(1)	Q14974-1, KPNB1, Importin subunit beta-1	5/0/0
	(2)	Q14974-2, KPNB1, Importin subunit beta-1	
797		Q5T749, KPRP, Keratinocyte proline-rich protein	6/0/0
798		P04264, KRT1, Keratin, type II cytoskeletal 1	38/0/8
799		P13645, KRT10, Keratin, type I cytoskeletal 10	27/0/9
800	(1)	P13646-1,3, KRT13, Keratin, type I cytoskeletal 13	2/0/13
	(2)	Q14525, KRT33B, Keratin, type I cuticular Ha3-II	
	·	Q15323, KRT31, Keratin, type I cuticular Ha1	
	(3)	P13646-2, KRT13, Keratin, type I cytoskeletal 13	
801		P02533, KRT14, Keratin, type I cytoskeletal 14	5/0/24
802	(1)	P19012-1, KRT15, Keratin, type I cytoskeletal 15	0/0/13
	(2)	P19012-2, KRT15, Keratin, type I cytoskeletal 15	
803		P08779, KRT16, Keratin, type I cytoskeletal 16	8/0/16
804		Q04695, KRT17, Keratin, type I cytoskeletal 17	6/0/17
805		P05783, KRT18, Keratin, type I cytoskeletal 18	30/0/3
806	(1)	P08727, KRT19, Keratin, type I cytoskeletal 19	21/0/14
	(2)	Q99456, KRT12, Keratin, type I cytoskeletal 12	
	(3)	Q8N1A0-1,2, KRT222, Keratin-like protein KRT222	
807		P35908, KRT2, Keratin, type II cytoskeletal 2 epidermal	23/0/12
808		P35900, KRT20, Keratin, type I cytoskeletal 20	0/0/5
809		Q2M2I5, KRT24, Keratin, type I cytoskeletal 24	1/0/4
810	(1)	Q7Z3Z0, KRT25, Keratin, type I cytoskeletal 25	4/0/6
	(2)	Q7Z3Y9, KRT26, Keratin, type I cytoskeletal 26	
811		Q7Z3Y8, KRT27, Keratin, type I cytoskeletal 27	1/0/8
812		Q7Z3Y7, KRT28, Keratin, type I cytoskeletal 28	0/0/8
813		P12035, KRT3, Keratin, type II cytoskeletal 3	0/0/11
814		Q92764, KRT35, Keratin, type I cuticular Ha5	1/0/2
815		O76013-1,2, KRT36, Keratin, type I cuticular Ha6	1/0/1

816		P19013, KRT4, Keratin, type II cytoskeletal 4	2/0/7
817		P13647, KRT5, Keratin, type II cytoskeletal 5	18/0/18
818	(1)	P02538, KRT6A, Keratin, type II cytoskeletal 6A	1/0/32
	(2)	Q14CN4-1-3, KRT72, Keratin, type II cytoskeletal 72	
819		P04259, KRT6B, Keratin, type II cytoskeletal 6B	0/0/32
820		P48668, KRT6C, Keratin, type II cytoskeletal 6C	0/0/32
821		P08729, KRT7, Keratin, type II cytoskeletal 7	22/0/10
822	(1)	Q3SY84, KRT71, Keratin, type II cytoskeletal 71	4/0/5
	(2)	Q86Y46-2, KRT73, Keratin, type II cytoskeletal 73	
823	(1)	Q86Y46-1, KRT73, Keratin, type II cytoskeletal 73	0/0/7
	(2)	Q7RTS7, KRT74, Keratin, type II cytoskeletal 74	
824		O95678, KRT75, Keratin, type II cytoskeletal 75	0/0/16
825		Q01546, KRT76, Keratin, type II cytoskeletal 2 oral	0/0/12
826		Q7Z794, KRT77, Keratin, type II cytoskeletal 1b	2/0/5
827	(1)	Q8N1N4-1, KRT78, Keratin, type II cytoskeletal 78	1/0/3
	(2)	Q8N1N4-2, KRT78, Keratin, type II cytoskeletal 78	
828		Q5XKE5, KRT79, Keratin, type II cytoskeletal 79	2/0/11
829	(1)	P05787-1,2, KRT8, Keratin, type II cytoskeletal 8	29/0/13
	(2)	Q9NSB2, KRT84, Keratin, type II cuticular Hb4	
830		Q6KB66-1-3, KRT80, Keratin, type II cytoskeletal 80	1/0/0
831	(1)	O43790, KRT86, Keratin, type II cuticular Hb6	0/0/1
	·	P78385, KRT83, Keratin, type II cuticular Hb3	
	·	Q14533, KRT81, Keratin, type II cuticular Hb1	
	(2)	P78386, KRT85, Keratin, type II cuticular Hb5	
	(3)	125116, NA, NA	
832		P35527, KRT9, Keratin, type I cytoskeletal 9	27/0/2
833		Q9GZZ8, LACRT, Extracellular glycoprotein lacritin	2/0/0
834		P24043, LAMA2, Laminin subunit alpha-2	40/0/0
835		Q16363-1,2, LAMA4, Laminin subunit alpha-4	32/0/0
836	(1)	O15230-1, LAMA5, Laminin subunit alpha-5	9/0/0
	(2)	O15230-2, LAMA5, Laminin subunit alpha-5	
837		P07942, LAMB1, Laminin subunit beta-1	41/0/2
838		P55268, LAMB2, Laminin subunit beta-2	33/0/0
839		P11047, LAMC1, Laminin subunit gamma-1	50/0/0
840		P28838-1,2, LAP3, Cytosol aminopeptidase	1/0/0

841		Q9P2J5-1,2, LARS, Leucine--tRNA ligase, cytoplasmic	4/0/0
842		P18428, LBP, Lipopolysaccharide-binding protein	14/0/1
843	(1)	P04180, LCAT, Phosphatidylcholine-sterol acyltransferase	9/0/0
	(2)	Q8NCC3-1, PLA2G15, Group XV phospholipase A2	
844	(1)	P31025, LCN1, Lipocalin-1	5/0/0
	(2)	Q5VSP4, LCN1P1, Putative lipocalin 1-like protein 1	
845	(1)	P13796-1, LCP1, Plastin-2	17/0/6
	(2)	P13796-2, LCP1, Plastin-2	
	(3)	Q14651, PLS1, Plastin-1	
846	(1)	P00338-1-5, LDHA, L-lactate dehydrogenase A chain	
	(2)	P07195, LDHB, L-lactate dehydrogenase B chain	1/0/0
	·	P07864, LDHC, L-lactate dehydrogenase C chain	
	·	Q6ZMR3, LDHAL6A, L-lactate dehydrogenase A-like 6A	
			0/3/0
847	(1)	P01130-1,5, LDLR, Low-density lipoprotein receptor	
	(2)	P01130-2,3, LDLR, Low-density lipoprotein receptor	
	(3)	P01130-4, LDLR, Low-density lipoprotein receptor	
848		O14960, LECT2, Leukocyte cell-derived chemotaxin-2	5/0/0
849	(1)	O00292-1, LEFTY2, Left-right determination factor 2	2/0/0
	·	O75610, LEFTY1, Left-right determination factor 1	
	(2)	O00292-2, LEFTY2, Left-right determination factor 2	
850		P41159, LEP, Leptin	4/0/0
			1/0/0
851	(1)	Q8NES3-1,3, LFNG, Beta-1,3-N-acetylglucosaminyltransferase lunatic fringe	
	(2)	Q8NES3-2,4, LFNG, Beta-1,3-N-acetylglucosaminyltransferase lunatic fringe	
852		P09382, LGALS1, Galectin-1	8/0/0
853		P17931, LGALS3, Galectin-3	7/0/0
854		Q08380, LGALS3BP, Galectin-3-binding protein	18/0/0
855		P56470, LGALS4, Galectin-4	2/0/0
856		P47929, LGALS7, Galectin-7	4/0/0
857		Q8N0V4, LGI2, Leucine-rich repeat LGI family member 2	3/0/0
858		Q99538-1-3, LGMN, Legumain	4/0/0
859		P38571-1,2, LIPA, Lysosomal acid lipase/cholesteryl ester hydrolase	2/0/0
860		P49257, LMAN1, Protein ERGIC-53	7/0/1
861		Q12907, LMAN2, Vesicular integral-membrane protein VIP36	3/0/0

862	(1)	P02545-1-3,6, LMNA, Prelamin-A/C [Cleaved into: Lamin-A/C	9/0/3
	(2)	P02545-4,5, LMNA, Prelamin-A/C [Cleaved into: Lamin-A/C	
863		P20700, LMNB1, Lamin-B1	4/0/4
864		Q03252, LMNB2, Lamin-B2	2/0/6
865		P28300, LOX, Protein-lysine 6-oxidase	11/0/1
866		Q08397, LOXL1, Lysyl oxidase homolog 1	15/0/1
867		Q9Y4K0, LOXL2, Lysyl oxidase homolog 2	11/0/1
868	(1)	P58215-1, LOXL3, Lysyl oxidase homolog 3	1/0/1
	(2)	P58215-3, LOXL3, Lysyl oxidase homolog 3	
869		P08519, LPA, Apolipoprotein(a)	17/0/0
870		Q9UBY5, LPAR3, Lysophosphatidic acid receptor 3	4/0/0
871		P06858, LPL, Lipoprotein lipase	6/0/0
872		P02750, LRG1, Leucine-rich alpha-2-glycoprotein	6/0/1
873		Q07954-1, LRP1, Prolow-density lipoprotein receptor-related protein 1	57/0/2
874		Q9NZR2, LRP1B, Low-density lipoprotein receptor-related protein 1B	22/0/2
875	(1)	Q14114-1,3, LRP8, Low-density lipoprotein receptor-related protein 8	2/0/1
	(2)	Q14114-2,4, LRP8, Low-density lipoprotein receptor-related protein 8	
876		Q8N9N7, LRRC57, Leucine-rich repeat-containing protein 57	2/0/0
877		Q32MZ4-1-3, LRRFIP1, Leucine-rich repeat flightless-interacting protein 1	2/0/0
878		Q8WUT4, LRRN4, Leucine-rich repeat neuronal protein 4	3/0/0
879		P33241-1,3, LSP1, Lymphocyte-specific protein 1	1/0/0
880	(1)	Q86X29-1,4, LSR, Lipolysis-stimulated lipoprotein receptor	0/0/6
	(2)	Q86X29-5,6, LSR, Lipolysis-stimulated lipoprotein receptor	
881	(1)	Q86X29-3, LSR, Lipolysis-stimulated lipoprotein receptor	1/0/6
	(2)	Q86X29-2, LSR, Lipolysis-stimulated lipoprotein receptor	
882	(1)	Q14766-1,4, LTBP1, Latent-transforming growth factor beta-binding protein 1	1/0/31
	(2)	Q14766-2,5, LTBP1, Latent-transforming growth factor beta-binding protein 1	

883 Q14766-3, LTBP1, Latent-transforming growth factor beta-binding protein 1 — 1/0/31

884 Q14767, LTBP2, Latent-transforming growth factor beta-binding protein 2 — 37/0/1

885 (1) Q9NS15-1, LTBP3, Latent-transforming growth factor beta-binding protein 3 — 1/0/1
(2) Q9NS15-2, LTBP3, Latent-transforming growth factor beta-binding protein 3

886 (1) Q8N2S1-2, LTBP4, Latent-transforming growth factor beta-binding protein 4 — 2/14/1
(2) Q8N2S1-4, LTBP4, Latent-transforming growth factor beta-binding protein 4
(3) Q8N2S1-1,3, LTBP4, Latent-transforming growth factor beta-binding protein 4

887 (1) P02788-1, LTF, Lactotransferrin — 1/0/0
(2) P02788-2, LTF, Lactotransferrin

888 P51884, LUM, Lumican — 14/0/0

889 Q9Y6Y9-1,2, LY96, Lymphocyte antigen 96 — 1/0/0

890 O75608-1,2, LYPLA1, Acyl-protein thioesterase 1 — 3/0/0

891 P61626, LYZ, Lysozyme C — 13/0/0

892 P20645, M6PR, Cation-dependent mannose-6-phosphate receptor — 2/0/0

893 (1) Q9UPN3-1, MACF1, Microtubule-actin cross-linking factor 1, isoforms 1/2/3/5 — 0/1/2
(2) Q9UPN3-2-4, MACF1, Microtubule-actin cross-linking factor 1, isoforms 1/2/3/5
(3) Q9UPN3-5, MACF1, Microtubule-actin cross-linking factor 1, isoforms 1/2/3/5

894 (1) P33908-1, MAN1A1, Mannosyl-oligosaccharide 1,2-alpha-mannosidase IA — 5/0/0
(2) P33908-2, MAN1A1, Mannosyl-oligosaccharide 1,2-alpha-mannosidase IA

895 Q9NR34, MAN1C1, Mannosyl-oligosaccharide 1,2-alpha-mannosidase IC — 2/0/0

896 Q8IWC1-1-4, MAP7D3, MAP7 domain-containing protein 3 — 1/0/1

897 P45983-1-5, MAPK8, Mitogen-activated protein kinase 8 — 1/0/0
P45984-1-5, MAPK9, Mitogen-activated protein kinase 9
P53779-1-4, MAPK10, Mitogen-activated protein kinase 10

898 Q15691, MAPRE1, Microtubule-associated protein RP/EB family member 1 — 3/0/0

899		Q9UEW3-1,2, MARCO, Macrophage receptor MARCO	6/0/0
900	(1)	P48740-1, MASP1, Mannan-binding lectin serine protease 1	5/0/9
	(2)	P48740-3, MASP1, Mannan-binding lectin serine protease 1	
901		P48740-2,4, MASP1, Mannan-binding lectin serine protease 1	4/0/9
902		O00187-1, MASP2, Mannan-binding lectin serine protease 2	3/0/6
903		O00187-2, MASP2, Mannan-binding lectin serine protease 2	1/0/6
904		P31153-1,2, MAT2A, S-adenosylmethionine synthase isoform type-2	2/0/0
		Q00266, MAT1A, S-adenosylmethionine synthase isoform type-1	
905	(1)	O00339-2, MATN2, Matrilin-2	1/11/0
	(2)	O00339-1, MATN2, Matrilin-2	
	(3)	O00339-3, MATN2, Matrilin-2	
	(4)	O00339-4, MATN2, Matrilin-2	
906	(1)	P43243-1, MATR3, Matrin-3	5/0/0
	(2)	P43243-2, MATR3, Matrin-3	
907		P02144, MB, Myoglobin	7/0/0
908		P11226, MBL2, Mannose-binding protein C	1/0/0
909		P21741-1, MDK, Midkine	1/0/0
910		Q9NU22, MDN1, Midasin	8/0/0
911	(1)	O75095-1, MEGF6, Multiple epidermal growth factor-like domains protein 6	1/0/0
	(2)	O75095-2, MEGF6, Multiple epidermal growth factor-like domains protein 6	
912		Q9UJH8, METRN, Meteorin	2/0/1
913	(1)	Q641Q3-1, METRNL, Meteorin-like protein	1/0/1
	(2)	Q641Q3-2, METRNL, Meteorin-like protein	
914		P55001-1-3, MFAP2, Microfibrillar-associated protein 2	2/0/0
915		P55083-1,2, MFAP4, Microfibril-associated glycoprotein 4	7/0/0
916		Q13361-1,2, MFAP5, Microfibrillar-associated protein 5	5/0/1
917		Q08431-1-3, MFGE8, Lactadherin	2/0/0
918		P26572, MGAT1, Alpha-1,3-mannosyl-glycoprotein 2-beta-N-acetylglucosaminyltransferase	15/0/0
919		Q9UQ53-1-3, MGAT4B, Alpha-1,3-mannosyl-glycoprotein 4-beta-N-acetylglucosaminyltransferase B	5/0/0

920 Q09328, MGAT5, Alpha-1,6-mannosylglycoprotein 6-beta-N-acetylglucosaminyltransferase A — 6/0/0

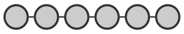

921 P08493-1,2, MGP, Matrix Gla protein — 5/0/0

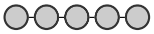

922 Q5JRA6-1-3, MIA3, Melanoma inhibitory activity protein 3 — 5/0/0

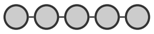

0/2/0

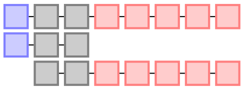

923 (1) Q9UNW1-1, MINPP1, Multiple inositol polyphosphate phosphatase 1
(2) Q9UNW1-2,3, MINPP1, Multiple inositol polyphosphate phosphatase 1
(3) Q9UNW1-4, MINPP1, Multiple inositol polyphosphate phosphatase 1

924 Q14165, MLEC, Malectin — 2/0/0

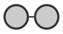

925 P50281, MMP14, Matrix metalloproteinase-14 — 4/0/0

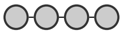

2/0/0

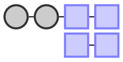

926 (1) Q99542-1, MMP19, Matrix metalloproteinase-19
(2) Q99542-3, MMP19, Matrix metalloproteinase-19

1/22/0

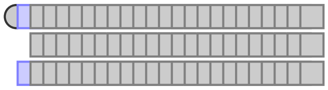

927 (1) P08253-1, MMP2, 72 kDa type IV collagenase
(2) P08253-2, MMP2, 72 kDa type IV collagenase
(3) P08253-3, MMP2, 72 kDa type IV collagenase

928 Q9H239-1, MMP28, Matrix metalloproteinase-28 — 2/0/0

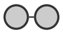

3/0/0

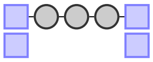

929 (1) Q13201-1, MMRN1, Multimerin-1
(2) Q13201-2, MMRN1, Multimerin-1

930 Q9H8L6, MMRN2, Multimerin-2 — 3/0/0

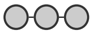

931 P05164-1-3, MPO, Myeloperoxidase — 6/0/0

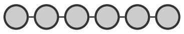

932 P25325-1,2, MPST, 3-mercaptopyruvate sulfurtransferase — 3/0/0

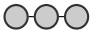

1/0/0

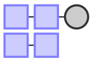

933 (1) P22897-1, MRC1, Macrophage mannose receptor 1
(2) P22897-2, MRC1, Macrophage mannose receptor 1

934 Q9UBG0, MRC2, C-type mannose receptor 2 — 3/0/0

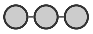

1/0/0

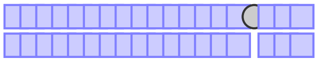

935 (1) Q13421-2-4, MSLN, Mesothelin
(2) Q13421-1, MSLN, Mesothelin

936 P26038, MSN, Moesin — 10/0/6

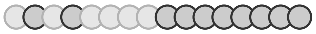

937 P21757-1,3, MSR1, Macrophage scavenger receptor types I and II — 5/0/0

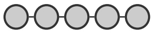

938 (1) P26927, MST1, Hepatocyte growth factor-like protein — 2/1/0
(2) Q2TV78-1, MST1L, Putative macrophage stimulating 1-like protein
(3) Q2TV78-2, MST1L, Putative macrophage stimulating 1-like protein

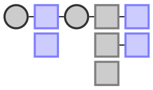

939		P04731, MT1A, Metallothionein-1A	5/0/0
940		P11586, MTHFD1, C-1-tetrahydrofolate synthase, cytoplasmic	5/0/0
941		Q14764, MVP, Major vault protein	7/0/0
942		Q9NR99, MXRA5, Matrix-remodeling-associated protein 5	47/0/0
943	(1)	P84157-1, MXRA7, Matrix-remodeling-associated protein 7	1/0/2
	(2)	P84157-3, MXRA7, Matrix-remodeling-associated protein 7	
944		P84157-2, MXRA7, Matrix-remodeling-associated protein 7	1/0/2
945		O75592-1,2, MYCBP2, E3 ubiquitin-protein ligase MYCBP2	3/0/0
946		P35580-1-5, MYH10, Myosin-10	27/0/17
947		P35749-1-4, MYH11, Myosin-11	4/0/16
948	(1)	Q7Z406-1,2,4,6, MYH14, Myosin-14	1/0/8
	(2)	Q7Z406-5, MYH14, Myosin-14	
949	(1)	P35579-1, MYH9, Myosin-9	21/0/19
	(2)	P35579-2, MYH9, Myosin-9	
950	(1)	P05976-1, MYL1, Myosin light chain 1/3, skeletal muscle isoform	1/0/1
	(2)	P05976-2, MYL1, Myosin light chain 1/3, skeletal muscle isoform	
951		O14950, MYL12B, Myosin regulatory light chain 12B<br>P19105, MYL12A, Myosin regulatory light chain 12A	3/0/4
952	(1)	P10916, MYL2, Myosin regulatory light chain 2, ventricular/cardiac muscle isoform	6/0/1
	(2)	Q02045-1, MYL5, Myosin light chain 5	
	(3)	Q9BUA6, MYL10, Myosin regulatory light chain 10	
953		P08590, MYL3, Myosin light chain 3	6/0/1
954	(1)	P60660-1,2, MYL6, Myosin light polypeptide 6	4/0/1
	(2)	P14649, MYL6B, Myosin light chain 6B	
955		P24844-1,2, MYL9, Myosin regulatory light polypeptide 9	1/0/4
956	(1)	Q15746-1-7,9,11, MYLK, Myosin light chain kinase, smooth muscle	1/0/0
	(2)	Q15746-8,10, MYLK, Myosin light chain kinase, smooth muscle	
957		Q96A32, MYLPF, Myosin regulatory light chain 2, skeletal muscle isoform	7/0/0
958		O00159-1-3, MYO1C, Unconventional myosin-Ic	2/0/0
959		Q8N1T3-1,3, MYO1H, Unconventional myosin-Ih	2/0/0

960		Q99972, MYOC, Myocilin	16/0/0
961		P54802, NAGLU, Alpha-N-acetylglucosaminidase	1/0/0
962		P43490, NAMPT, Nicotinamide phosphoribosyltransferase	1/0/0
963		P55209-1-3, NAP1L1, Nucleosome assembly protein 1-like 1<br>Q99733-1,2, NAP1L4, Nucleosome assembly protein 1-like 4	1/0/0
964	(1)	P54920, NAPA, Alpha-soluble NSF attachment protein	4/0/0
	(2)	Q9H115-1-3, NAPB, Beta-soluble NSF attachment protein	
965		O43776-1,2, NARS, Asparagine--tRNA ligase, cytoplasmic	2/0/0
966		P41271-1,2, NBL1, Neuroblastoma suppressor of tumorigenicity 1	3/0/0
967		P13591-1-6, NCAM1, Neural cell adhesion molecule 1	1/0/0
968		A6NI72, NCF1B, Putative neutrophil cytosol factor 1B<br>A8MVU1, NCF1C, Putative neutrophil cytosol factor 1C<br>P14598-1, NCF1, Neutrophil cytosol factor 1	2/0/0
969		P19338, NCL, Nucleolin	19/0/0
970	(1)	Q92597-1, NDRG1, Protein NDRG1	1/0/0
	(2)	Q92597-2,3, NDRG1, Protein NDRG1	
971	(1)	Q9NVZ3-1,2,4, NECAP2, Adaptin ear-binding coat-associated protein 2	1/0/0
	(2)	Q9NVZ3-3, NECAP2, Adaptin ear-binding coat-associated protein 2	
972	(1)	Q92692-2, NECTIN2, Nectin-2	2/0/0
	(2)	Q92692-1, NECTIN2, Nectin-2	
973		P12036-1,2, NEFH, Neurofilament heavy polypeptide	0/0/3
974		P07196, NEFL, Neurofilament light polypeptide	3/0/1
975		P48681, NES, Nestin	4/0/0
976	(1)	P14543-1, NID1, Nidogen-1	1/0/1
	(2)	P14543-2, NID1, Nidogen-1	
977	(1)	Q14112-1, NID2, Nidogen-2	1/0/2
	(2)	Q14112-2, NID2, Nidogen-2	
978		P40261, NNMT, Nicotinamide N-methyltransferase	3/0/0
979		P69849, NOMO3, Nodal modulator 3<br>Q15155, NOMO1, Nodal modulator 1<br>Q5JPE7-1-3, NOMO2, Nodal modulator 2	2/0/0
980	(1)	Q15233-1, NONO, Non-POU domain-containing octamer-binding protein	1/1/2
	(2)	Q15233-2, NONO, Non-POU domain-containing octamer-binding protein	

	(3)	Q8WXF1-1,2, PSPC1, Paraspeckle component 1	
981		Q9UM47, NOTCH3, Neurogenic locus notch homolog protein 3	10/0/0
982		P61916-1,2, NPC2, Epididymal secretory protein E1	3/0/0
983	(1)	P06748-1, NPM1, Nucleophosmin	0/7/0
	(2)	P06748-2, NPM1, Nucleophosmin	
	(3)	P06748-3, NPM1, Nucleophosmin	
984		P01160, NPPA, Natriuretic peptides A	3/0/0
985		P15559-1-3, NQO1, NAD(P)H dehydrogenase [quinone] 1	6/0/0
986	(1)	O60462-1-3, NRP2, Neuropilin-2	1/7/0
	(2)	O60462-6, NRP2, Neuropilin-2	
	(3)	O60462-4,5, NRP2, Neuropilin-2	
987		Q9UNZ2-1,4,5, NSFL1C, NSFL1 cofactor p47	2/0/0
988	(1)	P49902-1, NT5C2, Cytosolic purine 5'-nucleotidase	1/0/0
	(2)	P49902-2, NT5C2, Cytosolic purine 5'-nucleotidase	
989		Q02818, NUCB1, Nucleobindin-1	30/0/1
990		P80303-1,2, NUCB2, Nucleobindin-2	15/0/1
991		Q9H1E3-1, NUCKS1, Nuclear ubiquitous casein and cyclin-dependent kinase substrate 1	4/0/3
992		Q9H1E3-2, NUCKS1, Nuclear ubiquitous casein and cyclin-dependent kinase substrate 1	1/0/3
993	(1)	Q14980-1-4, NUMA1, Nuclear mitotic apparatus protein 1	8/0/1
	(2)	Q14980-5, NUMA1, Nuclear mitotic apparatus protein 1	
994		Q7Z3B4-1,3, NUP54, Nucleoporin p54	2/0/1
995		Q6DKJ4-1,3, NXN, Nucleoredoxin	1/0/0
996		Q86UD1, OAF, Out at first protein homolog	8/0/0
997		P20774, OGN, Mimecan	18/0/0
998		Q8WWZ8-1, OIT3, Oncoprotein-induced transcript 3 protein	2/0/0
999		Q6UWY5, OLFML1, Olfactomedin-like protein 1	14/0/0
1000	(1)	Q9NRN5-1, OLFML3, Olfactomedin-like protein 3	0/6/0
	(2)	Q9NRN5-2, OLFML3, Olfactomedin-like protein 3	
	(3)	Q9NRN5-3, OLFML3, Olfactomedin-like protein 3	
1001		Q99983, OMD, Osteomodulin	4/0/0

1002 Q96CV9-1-3, OPTN, Optineurin — 0/0/2

1003 P02763, ORM1, Alpha-1-acid glycoprotein 1 — 4/0/5

1004 P19652, ORM2, Alpha-1-acid glycoprotein 2 — 4/0/5

1005 O95747, OXSR1, Serine/threonine-protein kinase OSR1 — 2/0/0

1006 P04220, NA, Ig mu heavy chain disease protein — 1/0/11

1007 Q32P28-1,3,4, P3H1, Prolyl 3-hydroxylase 1 — 3/0/0

1008 P13674-1-3, P4HA1, Prolyl 4-hydroxylase subunit alpha-1 — 2/0/0

1009 P07237, P4HB, Protein disulfide-isomerase — 26/0/1

1010 — 1/5/0
(1) P11940-1, PABPC1, Polyadenylate-binding protein 1
(2) Q9H361, PABPC3, Polyadenylate-binding protein 3
(3) Q13310-1-3, PABPC4, Polyadenylate-binding protein 4
(4) P11940-2, PABPC1, Polyadenylate-binding protein 1

1011 — 1/0/0
(1) Q9UNF0-1, PACSIN2, Protein kinase C and casein kinase substrate in neurons protein 2
(2) Q9UNF0-2, PACSIN2, Protein kinase C and casein kinase substrate in neurons protein 2

1012 Q15102, PAFAH1B3, Platelet-activating factor acetylhydrolase IB subunit gamma — 1/0/0

1013 Q13177, PAK2, Serine/threonine-protein kinase PAK 2 — 2/0/0

1014 — 0/15/1
(1) P19021-1,3,5, PAM, Peptidyl-glycine alpha-amidating monooxygenase
(2) P19021-4,6, PAM, Peptidyl-glycine alpha-amidating monooxygenase
(3) P19021-2, PAM, Peptidyl-glycine alpha-amidating monooxygenase

1015 Q6UXH9-1, PAMR1, Inactive serine protease PAMR1 — 0/0/16

1016 Q6UXH9-2, PAMR1, Inactive serine protease PAMR1 — 1/0/15

1017 Q6UXH9-3, PAMR1, Inactive serine protease PAMR1 — 1/0/14

1018 — 0/10/0
(1) O95428-1,5,6, PAPLN, Papilin
(2) O95428-2, PAPLN, Papilin
(3) O95428-3, PAPLN, Papilin
(4) O95428-4, PAPLN, Papilin

1019 O95340-1,2, PAPSS2, Bifunctional 3'-phosphoadenosine 5'-phosphosulfate synthase 2 — 2/0/0

1020 — 2/0/0
(1) Q9NVD7-1, PARVA, Alpha-parvin
(2) Q9NVD7-2, PARVA, Alpha-parvin
(3) Q9HBI1-1-3, PARVB, Beta-parvin

1021 (1) Q96AQ6-1,2, PBXIP1, Pre-B-cell leukemia transcription factor-interacting protein 1 — 2/0/0
(2) Q96AQ6-3, PBXIP1, Pre-B-cell leukemia transcription factor-interacting protein 1

1022 (1) Q15365, PCBP1, Poly(rC)-binding protein 1 — 4/0/4
(2) P57721-1-5, PCBP3, Poly(rC)-binding protein 3

1023 Q15366-1-8, PCBP2, Poly(rC)-binding protein 2 — 2/0/4

1024 P22061-1,2, PCMT1, Protein-L-isoaspartate(D-aspartate) O-methyltransferase — 8/0/0

1025 Q15113, PCOLCE, Procollagen C-endopeptidase enhancer 1 — 23/0/0

1026 Q9UKZ9, PCOLCE2, Procollagen C-endopeptidase enhancer 2 — 10/0/0

1027 Q9UHG2, PCSK1N, ProSAAS — 5/0/0

1028 Q92824-1, PCSK5, Proprotein convertase subtilisin/kexin type 5 — 9/0/16

1029 Q92824-2, PCSK5, Proprotein convertase subtilisin/kexin type 5 — 1/0/16

1030 Q16549, PCSK7, Proprotein convertase subtilisin/kexin type 7 — 1/0/0

1031 (1) Q8NBP7-1, PCSK9, Proprotein convertase subtilisin/kexin type 9 — 7/0/0
(2) Q8NBP7-2, PCSK9, Proprotein convertase subtilisin/kexin type 9

1032 (1) Q9UHG3-1, PCYOX1, Prenylcysteine oxidase 1 — 2/0/0
(2) Q9UHG3-2, PCYOX1, Prenylcysteine oxidase 1

1033 Q53EL6-1,2, PDCD4, Programmed cell death protein 4 — 3/0/0

1034 (1) Q8WUM4-1,2, PDCD6IP, Programmed cell death 6-interacting protein — 12/0/1
(2) Q8WUM4-3, PDCD6IP, Programmed cell death 6-interacting protein

1035 P01127-1,2, PDGFB, Platelet-derived growth factor subunit B — 2/0/0

1036 (1) Q9NRA1-1, PDGFC, Platelet-derived growth factor C — 2/5/0
(2) Q9NRA1-2, PDGFC, Platelet-derived growth factor C
(3) Q9NRA1-3, PDGFC, Platelet-derived growth factor C
(4) Q9NRA1-4, PDGFC, Platelet-derived growth factor C

1037 Q9GZP0-1,2, PDGFD, Platelet-derived growth factor D — 5/0/0

1038 Q15198, PDGFRL, Platelet-derived growth factor receptor-like protein — 8/0/0

1039		P30101, PDIA3, Protein disulfide-isomerase A3	9/0/0
1040		P13667, PDIA4, Protein disulfide-isomerase A4	13/0/0
1041	(1)	Q15084-5, PDIA6, Protein disulfide-isomerase A6	1/0/0
	(2)	Q15084-1-4, PDIA6, Protein disulfide-isomerase A6	
1042	(1)	Q96HC4-1, PDLIM5, PDZ and LIM domain protein 5	1/5/0
	(2)	Q96HC4-2,3, PDLIM5, PDZ and LIM domain protein 5	
	(3)	Q96HC4-4,6, PDLIM5, PDZ and LIM domain protein 5	
	(4)	Q96HC4-7, PDLIM5, PDZ and LIM domain protein 5	
1043		Q9NR12-1,2, PDLIM7, PDZ and LIM domain protein 7	2/0/0
1044		Q15121-1,2, PEA15, Astrocytic phosphoprotein PEA-15	4/0/0
1045		Q96S96, PEBP4, Phosphatidylethanolamine-binding protein 4	1/0/0
1046		P01210, PENK, Proenkephalin-A [Cleaved into: Synenkephalin; Met-enkephalin	8/0/0
1047	(1)	P17858-1,2, PFKL, ATP-dependent 6-phosphofructokinase, liver type	2/0/2
	(2)	Q01813-1, PFKP, ATP-dependent 6-phosphofructokinase, platelet type	
1048		P08237-1-3, PFKM, ATP-dependent 6-phosphofructokinase, muscle type	2/0/0
1049		Q01813-2, PFKP, ATP-dependent 6-phosphofructokinase, platelet type	1/0/2
1050		P07737, PFN1, Profilin-1	7/0/0
1051		P52209-1,2, PGD, 6-phosphogluconate dehydrogenase, decarboxylating	9/0/0
1052		P00558-1,2, PGK1, Phosphoglycerate kinase 1	1/0/2
1053		P07205, PGK2, Phosphoglycerate kinase 2	1/0/2
1054		O95336, PGLS, 6-phosphogluconolactonase	2/0/0
1055		Q96PD5-1,2, PGLYRP2, N-acetylmuramoyl-L-alanine amidase	9/0/0
1056	(1)	P36871-1, PGM1, Phosphoglucomutase-1	2/0/0
	(2)	P36871-2,3, PGM1, Phosphoglucomutase-1	
1057		Q96G03-1, PGM2, Phosphoglucomutase-2	3/0/0
1058		O95394-1,3,4, PGM3, Phosphoacetylglucosamine mutase	2/0/0
1059		O00264-1,2, PGRMC1, Membrane-associated progesterone receptor component 1	4/0/0
1060	(1)	Q99623-1, PHB2, Prohibitin-2	1/0/0

(2) Q99623-2, PHB2, Prohibitin-2

1061 O43175, PHGDH, D-3-phosphoglycerate dehydrogenase 13/0/0

1062 Q9NRX4-1,2, PHPT1, 14 kDa phosphohistidine phosphatase 3/0/0

1063 Q13492-1-5, PICALM, Phosphatidylinositol-binding clathrin assembly protein 3/0/0

1064 P01833, PIGR, Polymeric immunoglobulin receptor 13/0/0

1065 P12273, PIP, Prolactin-inducible protein 1/0/1

1066 Q00169, PITPNA, Phosphatidylinositol transfer protein alpha isoform 5/0/0

1067 Q504Y2, PKDCC, Extracellular tyrosine-protein kinase PKDCC 5/0/0

1068 (1) P14618-1, PKM, Pyruvate kinase PKM 0/25/0

(2) P14618-2, PKM, Pyruvate kinase PKM

(3) P14618-3, PKM, Pyruvate kinase PKM

(4) P30613-1,2, PKLR, Pyruvate kinase PKLR

1069 Q9BZM1, PLA2G12A, Group XIIA secretory phospholipase A2 4/0/0

1070 P14555, PLA2G2A, Phospholipase A2, membrane associated 12/0/0

1071 Q13093, PLA2G7, Platelet-activating factor acetylhydrolase 2/0/0

1072 (1) P00750-1, PLAT, Tissue-type plasminogen activator 0/3/10

(2) P00750-2, PLAT, Tissue-type plasminogen activator

(3) P00750-3, PLAT, Tissue-type plasminogen activator

1073 P00750-4, PLAT, Tissue-type plasminogen activator 1/0/10

1074 (1) Q15149-5, PLEC, Plectin 1/133/7

(2) Q15149-3, PLEC, Plectin

(3) Q15149-1,2,4,6-9, PLEC, Plectin

1075 (1) P00747, PLG, Plasminogen 44/0/5

(2) Q15195, PLGLA, Plasminogen-like protein A

1076 Q02325, PLGLB1, Plasminogen-like protein B 1/0/4

1077 (1) O60664-1,3, PLIN3, Perilipin-3 1/5/0

(2) O60664-2, PLIN3, Perilipin-3

(3) O60664-4, PLIN3, Perilipin-3

1078 Q96Q06-1,2, PLIN4, Perilipin-4 8/0/0

1079 Q02809-1,2, PLOD1, Procollagen-lysine,2-oxoglutarate 5-dioxygenase 1 8/0/0

1080 O60568, PLOD3, Procollagen-lysine,2-oxoglutarate 5-dioxygenase 3 6/0/0

1081		Q04941-1, PLP2, Proteolipid protein 2	1/0/0
1082		P13797-1-3, PLS3, Plastin-3	6/0/6
1083	(1)	P55058-1, PLTP, Phospholipid transfer protein	0/14/0
	(2)	P55058-2,3, PLTP, Phospholipid transfer protein	
	(3)	P55058-4, PLTP, Phospholipid transfer protein	
1084		Q6UX71-1,2, PLXDC2, Plexin domain-containing protein 2	3/0/0
1085		O15031, PLXNB2, Plexin-B2	3/0/0
1086	(1)	Q7Z5L7-1-3, PODN, Podocan	3/0/0
	(2)	Q7Z5L7-4, PODN, Podocan	
1087		Q8WZA1-1,2, POMGNT1, Protein O-linked-mannose beta-1,2-N-acetylglucosaminyltransferase 1	6/0/0
1088		P27169, PON1, Serum paraoxonase/arylesterase 1	11/0/2
1089		Q15166, PON3, Serum paraoxonase/lactonase 3	2/0/2
1090		Q15063-1, POSTN, Periostin	3/0/47
1091		Q15063-2, POSTN, Periostin	0/0/45
1092		Q15063-3, POSTN, Periostin	0/0/46
1093		Q15063-4, POSTN, Periostin	0/0/43
1094		Q15063-5, POSTN, Periostin	0/0/48
1095		Q15063-6, POSTN, Periostin	0/0/42
1096		Q15063-7, POSTN, Periostin	0/0/40
1097		Q6S8J7-1,2, POTEA, POTE ankyrin domain family member A	3/0/0
1098		Q6S8J3-1, POTEE, POTE ankyrin domain family member E	3/0/12
1099		A5A3E0, POTEF, POTE ankyrin domain family member F	2/0/11
1100		Q6S545-1,2, POTEH, POTE ankyrin domain family member H	3/0/3
1101		P0CG38, POTEI, POTE ankyrin domain family member I	1/0/12
		P0CG39, POTEJ, POTE ankyrin domain family member J	
1102		Q9BYX7, POTEKP, Putative beta-actin-like protein 3	1/0/5
1103		Q15181, PPA1, Inorganic pyrophosphatase	1/0/0
1104		P02775, PPBP, Platelet basic protein	2/0/0
1105	(1)	P62937-1, PPIA, Peptidyl-prolyl cis-trans isomerase A	2/4/0
	(2)	P62937-2, PPIA, Peptidyl-prolyl cis-trans isomerase A	
	(3)	Q9Y536, PPIAL4A, Peptidyl-prolyl cis-trans isomerase A-like 4A	

(4) A0A075B759, NA, NA
· A0A0B4J2A2, NA, NA
· F5H284, PPIAL4D, Peptidyl-prolyl cis-trans isomerase A-like 4D
· P0DN26, PPIAL4F, Peptidyl-prolyl cis-trans isomerase A-like 4F
· P0DN37, PPIAL4G, Peptidyl-prolyl cis-trans isomerase A-like 4G

1106 P23284, PPIB, Peptidyl-prolyl cis-trans isomerase B — 5/0/0

1107 O60437, PPL, Periplakin — 17/0/1

1108 P36873-1,2, PPP1CC, Serine/threonine-protein phosphatase PP1-gamma catalytic subunit — 3/0/0
P62136-1-3, PPP1CA, Serine/threonine-protein phosphatase PP1-alpha catalytic subunit
P62140, PPP1CB, Serine/threonine-protein phosphatase PP1-beta catalytic subunit

1109 Q15435-1-5, PPP1R7, Protein phosphatase 1 regulatory subunit 7 — 3/0/0

1110 (1) P67775-1, PPP2CA, Serine/threonine-protein phosphatase 2A catalytic subunit alpha isoform — 0/1/0
(2) P62714, PPP2CB, Serine/threonine-protein phosphatase 2A catalytic subunit beta isoform
(3) P67775-2, PPP2CA, Serine/threonine-protein phosphatase 2A catalytic subunit alpha isoform

1111 P30153, PPP2R1A, Serine/threonine-protein phosphatase 2A 65 kDa regulatory subunit A alpha isoform — 11/0/0

1112 P63151-1,2, PPP2R2A, Serine/threonine-protein phosphatase 2A 55 kDa regulatory subunit B alpha isoform — 3/0/0

1113 P16298-1-4, PPP3CB, Serine/threonine-protein phosphatase 2B catalytic subunit beta isoform — 1/0/0
P48454-1-3, PPP3CC, Serine/threonine-protein phosphatase 2B catalytic subunit gamma isoform
Q08209-1-3,5, PPP3CA, Serine/threonine-protein phosphatase 2B catalytic subunit alpha isoform

1114 Q5MIZ7-1-3,5, PPP4R3B, Serine/threonine-protein phosphatase 4 regulatory subunit 3B — 2/0/0

1115 (1) Q96NZ9-1, PRAP1, Proline-rich acidic protein 1 — 0/1/4
(2) Q96NZ9-2, PRAP1, Proline-rich acidic protein 1
(3) Q96NZ9-4, PRAP1, Proline-rich acidic protein 1

1116 Q96NZ9-3, PRAP1, Proline-rich acidic protein 1 — 1/0/4

1117 Q04118, PRB3, Basic salivary proline-rich protein 3 — 1/0/0

1118 (1) P57071-1,3, PRDM15, PR domain zinc finger protein 15 — 1/0/0
(2) P57071-2, PRDM15, PR domain zinc finger protein 15

1119 Q06830, PRDX1, Peroxiredoxin-1 — 4/0/0

2/0/0

1120 (1) P32119-1, PRDX2, Peroxiredoxin-2

(2) P32119-2, PRDX2, Peroxiredoxin-2

1/0/0

1121 P30044-1-4, PRDX5, Peroxiredoxin-5, mitochondrial

11/0/0

1122 P30041, PRDX6, Peroxiredoxin-6

11/0/0

1123 P51888, PRELP, Prolargin

2/0/0

1124 Q9Y2Y8, PRG3, Proteoglycan 3

0/0/47

1125 (1) Q92954-3, PRG4, Proteoglycan 4

(2) Q92954-4, PRG4, Proteoglycan 4

2/3/47

1126 (1) Q92954-6, PRG4, Proteoglycan 4

(2) Q92954-1, PRG4, Proteoglycan 4

(3) Q92954-2, PRG4, Proteoglycan 4

(4) Q92954-5, PRG4, Proteoglycan 4

1/0/0

1127 (1) P13861-1, PRKAR2A, cAMP-dependent protein kinase type II-alpha regulatory subunit

(2) P13861-2, PRKAR2A, cAMP-dependent protein kinase type II-alpha regulatory subunit

3/0/0

1128 Q969G5, PRKCDBP, Protein kinase C delta-binding protein

9/0/0

1129 P14314-1,2, PRKCSH, Glucosidase 2 subunit beta

2/0/0

1130 P78527-1,2, PRKDC, DNA-dependent protein kinase catalytic subunit

3/0/0

1131 P01236, PRL, Prolactin

3/0/0

1132 P04156-1,2, PRNP, Major prion protein

12/0/0

1133 P04070-1,2, PROC, Vitamin K-dependent protein C

28/0/1

1134 P07225, PROS1, Vitamin K-dependent protein S

0/0/5

1135 P41219-1,2, PRPH, Peripherin

1136 P11908-1,2, PRPS2, Ribose-phosphate pyrophosphokinase 2

3/0/0

P60891-1, PRPS1, Ribose-phosphate pyrophosphokinase 1

5/0/0

1137 Q5JSZ5-1, PRRC2B, Protein PRRC2B

2/0/1

1138 P07477, PRSS1, Trypsin-1

1139 (1) P07478, PRSS2, Trypsin-2

3/0/2

(2) P35030-1-5, PRSS3, Trypsin-3

2/0/0

1140 O95084-1, PRSS23, Serine protease 23

1/0/2

1141 Q8NHM4, PRSS3P2, Putative trypsin-6

20/0/0

1142 P07602-1-3, PSAP, Prosaposin

1143		Q6NUJ1, PSAPL1, Proactivator polypeptide-like 1 [Cleaved into: Saposin A-like; Saposin B-Val-like; Saposin B-like; Saposin C-like; Saposin D-like]	3/0/0
1144		P25786-1,2, PSMA1, Proteasome subunit alpha type-1	2/0/0
1145		P25788-1,2, PSMA3, Proteasome subunit alpha type-3	2/0/0
1146	(1)	P60900-1, PSMA6, Proteasome subunit alpha type-6	1/0/0
	(2)	P60900-2, PSMA6, Proteasome subunit alpha type-6	
1147		P20618, PSMB1, Proteasome subunit beta type-1	2/0/0
1148		P28072, PSMB6, Proteasome subunit beta type-6	2/0/0
1149	(1)	Q99436-1, PSMB7, Proteasome subunit beta type-7	1/0/0
	(2)	Q99436-2, PSMB7, Proteasome subunit beta type-7	
1150	(1)	P62191-1, PSMC1, 26S protease regulatory subunit 4	1/0/1
	(2)	P62191-2, PSMC1, 26S protease regulatory subunit 4	
1151		P62195-1,2, PSMC5, 26S protease regulatory subunit 8	1/0/1
1152		Q99460-1,2, PSMD1, 26S proteasome non-ATPase regulatory subunit 1	4/0/0
1153		Q13200-1-3, PSMD2, 26S proteasome non-ATPase regulatory subunit 2	1/0/1
1154		Q06323-1-3, PSME1, Proteasome activator complex subunit 1	7/0/0
1155		Q9UL46, PSME2, Proteasome activator complex subunit 2	10/0/0
1156		O60542, PSPN, Persephin	1/0/0
1157	(1)	P26599-1-3, PTBP1, Polypyrimidine tract-binding protein 1	5/3/0
	(2)	O95758-1-6, PTBP3, Polypyrimidine tract-binding protein 3	
	(3)	O95758-7, PTBP3, Polypyrimidine tract-binding protein 3	
	(4)	Q9UKA9-1-6, PTBP2, Polypyrimidine tract-binding protein 2	
1158		P41222, PTGDS, Prostaglandin-H2 D-isomerase	5/0/0
1159		Q16647, PTGIS, Prostacyclin synthase	2/0/0
1160		Q14914-1,2, PTGR1, Prostaglandin reductase 1	2/0/0
1161		P06454-1,2, PTMA, Prothymosin alpha [Cleaved into: Prothymosin alpha, N-terminally processed; Thymosin alpha-1]	3/0/0
1162		P20962, PTMS, Parathymosin	4/0/0
1163		P21246, PTN, Pleiotrophin	2/0/0
1164		Q9H3S7, PTPN23, Tyrosine-protein phosphatase non-receptor type 23	4/0/0
1165		P10586-1,2, PTPRF, Receptor-type tyrosine-protein phosphatase F	4/0/0

1/0/0

1166 (1) Q13332-1-4,6, PTPRS, Receptor-type tyrosine-protein phosphatase S

(2) Q13332-5,7, PTPRS, Receptor-type tyrosine-protein phosphatase S

6/6/0

1167 (1) Q6NZI2-1, PTRF, Polymerase I and transcript release factor

(2) Q6NZI2-2, PTRF, Polymerase I and transcript release factor

(3) Q6NZI2-3, PTRF, Polymerase I and transcript release factor

1168 Q9UHX1-1-6, PUF60, Poly(U)-binding-splicing factor PUF60 — 2/0/0

1169 Q00577, PURA, Transcriptional activator protein Pur-alpha — 3/0/0

19/0/1

1170 (1) Q92626-1, PXDN, Peroxidasin homolog

(2) Q92626-2, PXDN, Peroxidasin homolog

7/0/8

1171 (1) P20742-1, PZP, Pregnancy zone protein

(2) P20742-2, PZP, Pregnancy zone protein

2/0/0

1172 (1) Q16769-1, QPCT, Glutaminyl-peptide cyclotransferase

(2) Q16769-2, QPCT, Glutaminyl-peptide cyclotransferase

1/0/0

1173 (1) O00391-1, QSOX1, Sulfhydryl oxidase 1

(2) O00391-2, QSOX1, Sulfhydryl oxidase 1

1174 P61026, RAB10, Ras-related protein Rab-10 — 2/0/0

1175 (1) Q15907-1, RAB11B, Ras-related protein Rab-11B — 1/0/0

(2) P62491-1,2, RAB11A, Ras-related protein Rab-11A

· Q15907-2, RAB11B, Ras-related protein Rab-11B

1176 P61106, RAB14, Ras-related protein Rab-14 — 2/0/0

0/0/2

1177 (1) P62820-1, RAB1A, Ras-related protein Rab-1A

(2) P62820-2, RAB1A, Ras-related protein Rab-1A

1178 (1) Q9H0U4, RAB1B, Ras-related protein Rab-1B — 1/0/2

(2) Q92928, RAB1C, Putative Ras-related protein Rab-1C

1179 P61019-1,2, RAB2A, Ras-related protein Rab-2A — 2/0/0

1180 P51149, RAB7A, Ras-related protein Rab-7a — 3/0/0

1181 Q15276-1,2, RABEP1, Rab GTPase-binding effector protein 1 — 1/0/1

1182 (1) P63000-1,2, RAC1, Ras-related C3 botulinum toxin substrate 1 — 2/2/1

(2) P15153, RAC2, Ras-related C3 botulinum toxin substrate 2

(3) P60763, RAC3, Ras-related C3 botulinum toxin substrate 3

1183 P63244, RACK1, Receptor of activated protein C kinase 1 — 7/0/0

1184		P54725-1-3, RAD23A, UV excision repair protein RAD23 homolog A	1/0/1
1185		P54727-1, RAD23B, UV excision repair protein RAD23 homolog B	1/0/1
1186		P62826, RAN, GTP-binding nuclear protein Ran	5/0/0
1187	(1)	P61224-3, RAP1B, Ras-related protein Rap-1b	1/5/0
	(2)	A6NIZ1, NA, Ras-related protein Rap-1b-like protein	
	·	P61224-1,2, RAP1B, Ras-related protein Rap-1b	
	(3)	P61224-4, RAP1B, Ras-related protein Rap-1b	
	(4)	P62834, RAP1A, Ras-related protein Rap-1A	
1188		P49788-1,2, RARRES1, Retinoic acid receptor responder protein 1	9/0/0
1189		Q99969, RARRES2, Retinoic acid receptor responder protein 2	9/0/0
1190		P54136-1,2, RARS, Arginine--tRNA ligase, cytoplasmic	3/0/0
1191	(1)	Q09028-1,3, RBBP4, Histone-binding protein RBBP4	1/0/1
	(2)	Q09028-2,4, RBBP4, Histone-binding protein RBBP4	
1192		Q16576-1, RBBP7, Histone-binding protein RBBP7	1/0/1
1193	(1)	P78332-1, RBM6, RNA-binding protein 6	2/0/0
	(2)	P78332-3, RBM6, RNA-binding protein 6	
1194		Q9Y5S9-1,2, RBM8A, RNA-binding protein 8A	1/0/0
1195	(1)	P38159-1,2, RBMX, RNA-binding motif protein, X chromosome	1/0/1
	(2)	Q96E39, RBMXL1, RNA binding motif protein, X-linked-like-1	
1196		P02753, RBP4, Retinol-binding protein 4	10/0/0
1197		Q15293-1,2, RCN1, Reticulocalbin-1	3/0/0
1198		Q96D15, RCN3, Reticulocalbin-3	4/0/0
1199	(1)	P35241-1, RDX, Radixin	0/1/7
	(2)	P35241-4, RDX, Radixin	
	(3)	P35241-5, RDX, Radixin	
1200		O95980, RECK, Reversion-inducing cysteine-rich protein with Kazal motifs	1/0/0
1201		Q04206-1-4, RELA, Transcription factor p65	2/0/0
1202		P08134, RHOC, Rho-related GTP-binding protein RhoC	2/0/0
		P61586, RHOA, Transforming protein RhoA	
1203		Q9NPQ8-1-4, RIC8A, Synembryn-A	1/0/1
1204		Q13129, RLF, Zinc finger protein Rlf	7/0/0

1205		P07998, RNASE1, Ribonuclease pancreatic	7/0/0
1206		P12724, RNASE3, Eosinophil cationic protein	2/0/0
1207		P34096, RNASE4, Ribonuclease 4	12/0/1
1208		Q9P0P0, RNF181, E3 ubiquitin-protein ligase RNF181	1/0/0
1209		P13489, RNH1, Ribonuclease inhibitor	11/0/1
1210	(1)	P15927-3, RPA2, Replication protein A 32 kDa subunit	1/0/0
	(2)	P15927-1,2, RPA2, Replication protein A 32 kDa subunit	
1211		P27635, RPL10, 60S ribosomal protein L10	3/0/0
1212		P62906, RPL10A, 60S ribosomal protein L10a	2/0/0
1213	(1)	P30050-1, RPL12, 60S ribosomal protein L12	3/0/0
	(2)	P30050-2, RPL12, 60S ribosomal protein L12	
1214		P26373-1,2, RPL13, 60S ribosomal protein L13	3/0/1
1215		P50914, RPL14, 60S ribosomal protein L14	2/0/0
1216		P18621-1-3, RPL17, 60S ribosomal protein L17	1/0/1
1217		Q07020-1,2, RPL18, 60S ribosomal protein L18	1/0/0
1218		Q02543, RPL18A, 60S ribosomal protein L18a	1/0/0
1219		P84098, RPL19, 60S ribosomal protein L19	5/0/0
1220		P35268, RPL22, 60S ribosomal protein L22	2/0/0
1221		P62750, RPL23A, 60S ribosomal protein L23a	3/0/0
1222		P83731, RPL24, 60S ribosomal protein L24	2/0/0
1223		P61254, RPL26, 60S ribosomal protein L26	3/0/0
1224		P61353, RPL27, 60S ribosomal protein L27	2/0/0
1225		P46779-1-5, RPL28, 60S ribosomal protein L28	2/0/0
1226		P39023, RPL3, 60S ribosomal protein L3	2/0/0
1227		P62888, RPL30, 60S ribosomal protein L30	3/0/0
1228		P62899-1-3, RPL31, 60S ribosomal protein L31	3/0/0
1229		P49207, RPL34, 60S ribosomal protein L34	2/0/0
1230		P18077, RPL35A, 60S ribosomal protein L35a	3/0/0
1231		P61927, RPL37, 60S ribosomal protein L37	2/0/0
1232		P46777, RPL5, 60S ribosomal protein L5	7/0/0
1233		Q02878, RPL6, 60S ribosomal protein L6	2/0/0
1234		P18124, RPL7, 60S ribosomal protein L7	4/0/0

1235		P62424, RPL7A, 60S ribosomal protein L7a	5/0/0
1236		P62917, RPL8, 60S ribosomal protein L8	6/0/0
1237	(1)	P05388-1,2, RPLP0, 60S acidic ribosomal protein P0	1/0/0
	(2)	Q8NHW5, RPLP0P6, 60S acidic ribosomal protein P0-like	
1238		P05387, RPLP2, 60S acidic ribosomal protein P2	3/0/0
1239		P04843, RPN1, Dolichyl-diphosphooligosaccharide--protein glycosyltransferase subunit 1	8/0/0
1240	(1)	P46783, RPS10, 40S ribosomal protein S10	2/0/0
	(2)	Q9NQ39, RPS10P5, Putative 40S ribosomal protein S10-like	
1241		P25398, RPS12, 40S ribosomal protein S12	3/0/0
1242		P62263, RPS14, 40S ribosomal protein S14	4/0/0
1243		P62269, RPS18, 40S ribosomal protein S18	3/0/0
1244		P39019, RPS19, 40S ribosomal protein S19	3/0/0
1245		P15880, RPS2, 40S ribosomal protein S2	1/0/0
1246		P60866-1,2, RPS20, 40S ribosomal protein S20	3/0/0
1247		P62851, RPS25, 40S ribosomal protein S25	2/0/0
1248	(1)	P62979, RPS27A, Ubiquitin-40S ribosomal protein S27a	
	(2)	P0CG47, UBB, Polyubiquitin-B [Cleaved into: Ubiquitin]	3/0/0
	·	P0CG48, UBC, Polyubiquitin-C [Cleaved into: Ubiquitin]	
	·	P62987, UBA52, Ubiquitin-60S ribosomal protein L40	
1249		P62857, RPS28, 40S ribosomal protein S28	2/0/0
1250		P23396-1,2, RPS3, 40S ribosomal protein S3	7/0/0
1251		P61247, RPS3A, 40S ribosomal protein S3a	2/0/0
1252		P62701, RPS4X, 40S ribosomal protein S4, X isoform	2/0/0
1253		P46782, RPS5, 40S ribosomal protein S5 [Cleaved into: 40S ribosomal protein S5, N-terminally processed]	3/0/0
1254		P62753, RPS6, 40S ribosomal protein S6	5/0/0
1255		P51812, RPS6KA3, Ribosomal protein S6 kinase alpha-3	4/0/0
1256		P62081, RPS7, 40S ribosomal protein S7	3/0/0
1257		P46781, RPS9, 40S ribosomal protein S9	3/0/0
1258		P08865, RPSA, 40S ribosomal protein SA	11/0/1
1259	(1)	Q9P2E9-1,2, RRBP1, Ribosome-binding protein 1	4/0/0
	(2)	Q9P2E9-3, RRBP1, Ribosome-binding protein 1	
1260		O95197-1-7, RTN3, Reticulon-3	1/0/0
		Q16799-1-3, RTN1, Reticulon-1	
1261		Q9NQC3-1,2,5, RTN4, Reticulon-4	2/0/0

1262		Q9Y230-1, RUVBL2, RuvB-like 2	1/0/0
1263		P60903, S100A10, Protein S100-A10	6/0/0
1264		P31949, S100A11, Protein S100-A11	3/0/0
1265		P06703, S100A6, Protein S100-A6	4/0/0
1266	(1)	P31151, S100A7, Protein S100-A7	1/0/0
	(2)	Q86SG5, S100A7A, Protein S100-A7A	
1267		P05109, S100A8, Protein S100-A8	7/0/0
1268		P06702, S100A9, Protein S100-A9	2/0/0
1269	(1)	P0DJI8, SAA1, Serum amyloid A-1 protein	3/0/7
	(2)	P0DJI9-2, SAA2, Serum amyloid A-2 protein	
1270		P0DJI9-1, SAA2, Serum amyloid A-2 protein	2/0/7
1271		P35542, SAA4, Serum amyloid A-4 protein	7/0/0
1272		Q9UBE0-1-3, SAE1, SUMO-activating enzyme subunit 1	3/0/0
1273		P49591, SARS, Serine--tRNA ligase, cytoplasmic	7/0/0
1274	(1)	Q6UWP8-1, SBSN, Suprabasin	3/0/0
	(2)	Q6UWP8-2, SBSN, Suprabasin	
1275		P13521, SCG2, Secretogranin-2	25/0/0
1276	(1)	Q8WXD2-1, SCG3, Secretogranin-3	3/0/0
	(2)	Q8WXD2-2, SCG3, Secretogranin-3	
1277	(1)	P05408-2, SCG5, Neuroendocrine protein 7B2	1/0/0
	(2)	P05408-1, SCG5, Neuroendocrine protein 7B2	
1278		P11684, SCGB1A1, Uteroglobin	3/0/0
1279		Q9HB40-1, SCPEP1, Retinoid-inducible serine carboxypeptidase	2/0/0
1280		Q12765-1-3, SCRN1, Secernin-1	2/0/0
1281	(1)	Q9NQ36-1,3, SCUBE2, Signal peptide, CUB and EGF-like domain-containing protein 2	2/0/0
	(2)	Q9NQ36-2, SCUBE2, Signal peptide, CUB and EGF-like domain-containing protein 2	
1282	(1)	Q9BRK5-1, SDF4, 45 kDa calcium-binding protein	0/13/0
	(2)	Q9BRK5-2, SDF4, 45 kDa calcium-binding protein	
	(3)	Q9BRK5-5, SDF4, 45 kDa calcium-binding protein	
	(4)	Q9BRK5-6, SDF4, 45 kDa calcium-binding protein	
	(5)	Q9BRK5-3,4, SDF4, 45 kDa calcium-binding protein	

1283 O95810, SDPR, Serum deprivation-response protein — 11/0/0

1284 P55735-1-3, SEC13, Protein SEC13 homolog — 2/0/0

1285 (1) Q15436-1, SEC23A, Protein transport protein Sec23A — 0/0/0
(2) Q15436-2, SEC23A, Protein transport protein Sec23A
(3) Q15437, SEC23B, Protein transport protein Sec23B

1286 Q9Y6Y8-1,2, SEC23IP, SEC23-interacting protein — 3/0/1

1287 (1) P53992-1, SEC24C, Protein transport protein Sec24C — 2/0/0
(2) P53992-2, SEC24C, Protein transport protein Sec24C

1288 Q13214-1,2, SEMA3B, Semaphorin-3B — 6/0/0

1289 (1) Q99985-1, SEMA3C, Semaphorin-3C — 5/0/0
(2) Q99985-2, SEMA3C, Semaphorin-3C

1290 Q13275-1,2, SEMA3F, Semaphorin-3F — 4/0/0

1291 Q9NS98, SEMA3G, Semaphorin-3G — 5/0/0

1292 P49908, SEPP1, Selenoprotein P — 7/0/0

1293 (1) Q15019-1,2, SEPT2, Septin-2 — 1/0/0
(2) Q15019-3, SEPT2, Septin-2

1294 Q16181-1,2, SEPT7, Septin-7 — 10/0/0

1295 (1) P01009-1, SERPINA1, Alpha-1-antitrypsin — 4/19/0
(2) P01009-2, SERPINA1, Alpha-1-antitrypsin
(3) P01009-3, SERPINA1, Alpha-1-antitrypsin

1296 Q9UK55, SERPINA10, Protein Z-dependent protease inhibitor — 14/0/0

1297 (1) P01011-1, SERPINA3, Alpha-1-antichymotrypsin — 9/0/0
(2) P01011-2, SERPINA3, Alpha-1-antichymotrypsin

1298 P29622, SERPINA4, Kallistatin — 17/0/0

1299 P05154, SERPINA5, Plasma serine protease inhibitor — 11/0/0

1300 P08185, SERPINA6, Corticosteroid-binding globulin — 5/0/0

1301 P05543, SERPINA7, Thyroxine-binding globulin — 10/0/0

1302 P01008, SERPINC1, Antithrombin-III — 28/0/0

1303 P05546, SERPIND1, Heparin cofactor 2 — 27/0/0

1304 (1) P05121-1, SERPINE1, Plasminogen activator inhibitor 1 — 1/0/0
(2) P05121-2, SERPINE1, Plasminogen activator inhibitor 1

1305 P07093-1-3, SERPINE2, Glia-derived nexin — 8/0/0

1306		P36955, SERPINF1, Pigment epithelium-derived factor	20/0/0
1307	(1)	P08697-1, SERPINF2, Alpha-2-antiplasmin	3/0/0
	(2)	P08697-2, SERPINF2, Alpha-2-antiplasmin	
1308		P05155-1-3, SERPING1, Plasma protease C1 inhibitor	11/0/0
1309		P50454, SERPINH1, Serpin H1	5/0/0
1310		P0DME0, SETSIP, Protein SETSIP	1/0/1
		Q01105-1-4, SET, Protein SET	
1311		Q15459-1,2, SF3A1, Splicing factor 3A subunit 1	5/0/0
1312		P31947-1,2, SFN, 14-3-3 protein sigma	4/0/4
1313		P23246-1,2, SFPQ, Splicing factor, proline- and glutamine-rich	9/0/2
1314		Q8N474, SFRP1, Secreted frizzled-related protein 1	3/0/1
1315		Q96HF1, SFRP2, Secreted frizzled-related protein 2	11/0/0
1316		Q6FHJ7, SFRP4, Secreted frizzled-related protein 4	6/0/0
1317		Q9H299, SH3BGRL3, SH3 domain-binding glutamic acid-rich-like protein 3	2/0/0
1318	(1)	P04278-1, SHBG, Sex hormone-binding globulin	0/8/0
	(2)	P04278-2-4, SHBG, Sex hormone-binding globulin	
	(3)	P04278-5, SHBG, Sex hormone-binding globulin	
1319	(1)	P42285, SKIV2L2, Superkiller viralicidic activity 2-like 2	2/0/0
	(2)	Q15477, SKIV2L, Helicase SKI2W	
1320	(1)	P63208-1, SKP1, S-phase kinase-associated protein 1	3/0/1
	(2)	P63208-2, SKP1, S-phase kinase-associated protein 1	
1321		Q00325-1,2, SLC25A3, Phosphate carrier protein, mitochondrial	2/0/0
1322	(1)	P05141, SLC25A5, ADP/ATP translocase 2	1/0/0
	·	P12236, SLC25A6, ADP/ATP translocase 3	
	(2)	P12235, SLC25A4, ADP/ATP translocase 1	
1323		Q9HAS3-1, SLC28A3, Solute carrier family 28 member 3	3/0/0
1324	(1)	O14745-1, SLC9A3R1, Na(+)/H(+) exchange regulatory cofactor NHE-RF1	4/0/0
	(2)	O14745-2, SLC9A3R1, Na(+)/H(+) exchange regulatory cofactor NHE-RF1	
1325		Q9GZT3-1,2, SLIRP, SRA stem-loop-interacting RNA-binding protein, mitochondrial	1/0/1
1326		O75094-1,2,4, SLIT3, Slit homolog 3 protein	6/0/0

1327		Q9H2G2-1,2, SLK, STE20-like serine/threonine-protein kinase	5/0/0
1328		P03973, SLPI, Antileukoproteinase	14/0/0
1329		P55000, SLURP1, Secreted Ly-6/uPAR-related protein 1	2/0/0
1330		P52788-1, SMS, Spermine synthase	3/0/0
1331		Q5SXM2, SNAPC4, snRNA-activating protein complex subunit 4	0/0/2
1332		Q7KZF4, SND1, Staphylococcal nuclease domain-containing protein 1	11/0/0
1333		Q8TER0-1,3-5, SNED1, Sushi, nidogen and EGF-like domain-containing protein 1	9/0/0
1334	(1)	P08621-1,2, SNRNP70, U1 small nuclear ribonucleoprotein 70 kDa	1/0/0
	(2)	P08621-3, SNRNP70, U1 small nuclear ribonucleoprotein 70 kDa	
1335		P62306, SNRPF, Small nuclear ribonucleoprotein F	1/0/0
1336		Q13596-1-3, SNX1, Sorting nexin-1	3/0/1
1337		O60749-1,2, SNX2, Sorting nexin-2	4/0/0
1338		Q9Y5X3-1, SNX5, Sorting nexin-5	1/0/1
1339		Q9UNH7-1,2, SNX6, Sorting nexin-6	5/0/0
1340		Q9Y5X1, SNX9, Sorting nexin-9	2/0/0
1341		P08294, SOD3, Extracellular superoxide dismutase [Cu-Zn]	10/0/0
1342		O94875-11, SORBS2, Sorbin and SH3 domain-containing protein 2	2/0/0
1343		Q07889-1, SOS1, Son of sevenless homolog 1	2/0/0
1344		P23497-1, SP100, Nuclear autoantigen Sp-100	3/0/2
1345		P09486, SPARC, SPARC	16/0/1
1346	(1)	Q14515-1, SPARCL1, SPARC-like protein 1	5/0/1
	(2)	Q14515-2, SPARCL1, SPARC-like protein 1	
1347	(1)	Q9NQ38-3, SPINK5, Serine protease inhibitor Kazal-type 5	1/5/0
	(2)	Q9NQ38-1, SPINK5, Serine protease inhibitor Kazal-type 5	
	(3)	Q9NQ38-2, SPINK5, Serine protease inhibitor Kazal-type 5	
1348		Q9HCB6, SPON1, Spondin-1	23/0/0
1349		P10451-1-5, SPP1, Osteopontin	4/0/0
1350		Q13103, SPP2, Secreted phosphoprotein 24	9/0/0
1351		P35270, SPR, Sepiapterin reductase	3/0/0

1352		Q13813-1-3, SPTAN1, Spectrin alpha chain, non-erythrocytic 1	19/0/0
1353		Q01082-1-3, SPTBN1, Spectrin beta chain, non-erythrocytic 1	21/0/2
1354		O15020-1,2, SPTBN2, Spectrin beta chain, non-erythrocytic 2	3/0/2
1355		Q9H254-1,2,4, SPTBN4, Spectrin beta chain, non-erythrocytic 4	1/0/2
1356		P30626-1-3, SRI, Sorcin	2/0/0
1357	(1)	P78539-1, SRPX, Sushi repeat-containing protein SRPX	0/2/13
	(2)	P78539-2,5, SRPX, Sushi repeat-containing protein SRPX	
	(3)	P78539-4, SRPX, Sushi repeat-containing protein SRPX	
1358		P78539-3, SRPX, Sushi repeat-containing protein SRPX	1/0/13
1359		Q07955-1-3, SRSF1, Serine/arginine-rich splicing factor 1	8/0/0
1360		O75494-1-6, SRSF10, Serine/arginine-rich splicing factor 10	1/0/0
1361		Q01130-1, SRSF2, Serine/arginine-rich splicing factor 2	2/0/2
1362		P84103-1,2, SRSF3, Serine/arginine-rich splicing factor 3	3/0/0
1363	(1)	Q08170, SRSF4, Serine/arginine-rich splicing factor 4	1/1/1
	(2)	Q13247-2, SRSF6, Serine/arginine-rich splicing factor 6	
	(3)	Q13247-1,3, SRSF6, Serine/arginine-rich splicing factor 6	
1364	(1)	Q13243-3, SRSF5, Serine/arginine-rich splicing factor 5	2/0/1
	(2)	Q13243-1, SRSF5, Serine/arginine-rich splicing factor 5	
1365		A1L4H1-1,2, SSC5D, Soluble scavenger receptor cysteine-rich domain-containing protein SSC5D	3/0/0
1366	(1)	P50502, ST13, Hsc70-interacting protein	2/1/0
	(2)	Q8NFI4, ST13P5, Putative protein FAM10A5	
	(3)	Q8IZP2, ST13P4, Putative protein FAM10A4	
1367		Q11201, ST3GAL1, CMP-N-acetylneuraminate-beta-galactosamide-alpha-2,3-sialyltransferase 1	1/0/0
1368		Q9UNP4-1-3, ST3GAL5, Lactosylceramide alpha-2,3-sialyltransferase	1/0/0
1369		P15907-1, ST6GAL1, Beta-galactoside alpha-2,6-sialyltransferase 1	2/0/0
1370	(1)	Q9NY15-1, STAB1, Stabilin-1	13/0/0
	(2)	Q9NY15-2, STAB1, Stabilin-1	
1371		Q9Y3M8-4, STARD13, StAR-related lipid transfer protein 13	2/0/1
1372		P40763-1-3, STAT3, Signal transducer and activator of transcription 3	4/0/0

1373		O76061, STC2, Stanniocalcin-2	1/0/0
1374		P31948-1-3, STIP1, Stress-induced-phosphoprotein 1	2/0/0
1375		P53999, SUB1, Activated RNA polymerase II transcriptional coactivator p15	3/0/0
1376		Q8IWU6, SULF1, Extracellular sulfatase Sulf-1	9/0/0
1377	(1)	P50226, SULT1A2, Sulfotransferase 1A2	1/0/4
	(2)	P50225-1, SULT1A1, Sulfotransferase 1A1	
	(3)	P50225-2, SULT1A1, Sulfotransferase 1A1	
1378	(1)	P0DMM9-1,3, SULT1A3, Sulfotransferase 1A3	0/0/4
	·	P0DMN0, SULT1A4, Sulfotransferase 1A4	
	(2)	P0DMM9-2, SULT1A3, Sulfotransferase 1A3	
1379		O60279, SUSD5, Sushi domain-containing protein 5	5/0/0
1380	(1)	Q4LDE5-1, SVEP1, Sushi, von Willebrand factor type A, EGF and pentraxin domain-containing protein 1	11/36/0
	(2)	Q4LDE5-2, SVEP1, Sushi, von Willebrand factor type A, EGF and pentraxin domain-containing protein 1	
	(3)	Q4LDE5-3, SVEP1, Sushi, von Willebrand factor type A, EGF and pentraxin domain-containing protein 1	
	(4)	Q4LDE5-4, SVEP1, Sushi, von Willebrand factor type A, EGF and pentraxin domain-containing protein 1	
1381		Q9UH65, SWAP70, Switch-associated protein 70	2/0/0
1382	(1)	O60506-1,3, SYNCRIP, Heterogeneous nuclear ribonucleoprotein Q	0/0/6
	(2)	O60506-2,4, SYNCRIP, Heterogeneous nuclear ribonucleoprotein Q	
	(3)	O60506-5, SYNCRIP, Heterogeneous nuclear ribonucleoprotein Q	
1383		Q92804-1,2, TAF15, TATA-binding protein-associated factor 2N	5/0/2
1384		Q01995, TAGLN, Transgelin	11/0/0
1385		P37802-1,2, TAGLN2, Transgelin-2	12/0/0
1386		Q99426-1,2, TBCB, Tubulin-folding cofactor B	3/0/0
1387		Q07283, TCHH, Trichohyalin	12/0/1
1388	(1)	P20062-1, TCN2, Transcobalamin-2	3/0/0
	(2)	P20062-2, TCN2, Transcobalamin-2	
1389		P17987, TCP1, T-complex protein 1 subunit alpha	3/0/0
1390		Q9BXT4-1-4, TDRD1, Tudor domain-containing protein 1	2/0/0
1391		B5MCY1, TDRD15, Tudor domain-containing protein 15	5/0/0

1392		P02787, TF, Serotransferrin	38/0/0
1393		Q92734-1-4, TFG, Protein TFG	2/0/1
1394		P10646-1, TFPI, Tissue factor pathway inhibitor	4/0/0
1395		P48307-1,2, TFPI2, Tissue factor pathway inhibitor 2	2/0/0
1396		P61812-1,2, TGFB2, Transforming growth factor beta-2	3/0/0
1397		Q15582, TGFBI, Transforming growth factor-beta-induced protein ig-h3	30/0/0
1398		Q03167-1,2, TGFBR3, Transforming growth factor beta receptor type 3	13/0/0
1399	(1)	P22735-1, TGM1, Protein-glutamine gamma-glutamyltransferase K	4/0/0
	(2)	P22735-2, TGM1, Protein-glutamine gamma-glutamyltransferase K	
1400	(1)	P21980-1, TGM2, Protein-glutamine gamma-glutamyltransferase 2	4/0/1
	(2)	P21980-2, TGM2, Protein-glutamine gamma-glutamyltransferase 2	
1401		Q08188, TGM3, Protein-glutamine gamma-glutamyltransferase E	2/0/0
1402		P49221, TGM4, Protein-glutamine gamma-glutamyltransferase 4	1/0/1
1403		O43493-1-6, TGOLN2, Trans-Golgi network integral membrane protein 2	2/0/0
1404	(1)	P07996-1, THBS1, Thrombospondin-1	7/0/3
	(2)	P07996-2, THBS1, Thrombospondin-1	
1405		P35442, THBS2, Thrombospondin-2	21/0/2
1406	(1)	P49746-1, THBS3, Thrombospondin-3	2/0/1
	(2)	P49746-2, THBS3, Thrombospondin-3	
1407		P35443, THBS4, Thrombospondin-4	18/0/3
1408		Q6ZMP0-1,4, THSD4, Thrombospondin type-1 domain-containing protein 4	8/0/0
1409		P35590-1, TIE1, Tyrosine-protein kinase receptor Tie-1	4/0/0
1410		P01033, TIMP1, Metalloproteinase inhibitor 1	5/0/0
1411		P16035, TIMP2, Metalloproteinase inhibitor 2	10/0/0
1412		Q99727, TIMP4, Metalloproteinase inhibitor 4	2/0/0
1413	(1)	Q9GZM7-1, TINAGL1, Tubulointerstitial nephritis antigen-like	2/0/0

	(2)	Q9GZM7-3, TINAGL1, Tubulointerstitial nephritis antigen-like	
1414		Q07157-1,2, TJP1, Tight junction protein ZO-1	5/0/0
1415		Q3LXA3-1,2, TKFC, Triokinase/FMN cyclase	1/0/0
1416	(1)	O43897-1, TLL1, Tolloid-like protein 1	4/0/2
	(2)	O43897-2, TLL1, Tolloid-like protein 1	
1417		Q9Y6L7, TLL2, Tolloid-like protein 2	1/0/5
1418		Q9Y490, TLN1, Talin-1	40/0/14
1419		Q9Y4G6, TLN2, Talin-2	5/0/13
1420		P49755, TMED10, Transmembrane emp24 domain-containing protein 10	4/0/0
1421		Q9BVK6, TMED9, Transmembrane emp24 domain-containing protein 9	3/0/0
1422		Q9BTV4, TMEM43, Transmembrane protein 43	2/0/0
1423		Q9BXS4, TMEM59, Transmembrane protein 59	1/0/0
1424		Q9NYL9, TMOD3, Tropomodulin-3	2/0/0
1425	(1)	P24821-1,4, TNC, Tenascin	1/38/0
	(2)	P24821-6, TNC, Tenascin	
	(3)	P24821-2,3,5, TNC, Tenascin	
1426		O00300, TNFRSF11B, Tumor necrosis factor receptor superfamily member 11B	4/0/0
1427	(1)	O43508-2, TNFSF12, Tumor necrosis factor ligand superfamily member 12	0/1/1
	·	O75888-1,3, TNFSF13, Tumor necrosis factor ligand superfamily member 13	
	(2)	O75888-4,5, TNFSF13, Tumor necrosis factor ligand superfamily member 13	
	(3)	O75888-2, TNFSF13, Tumor necrosis factor ligand superfamily member 13	
1428	(1)	Q9C0C2-1, TNKS1BP1, 182 kDa tankyrase-1-binding protein	1/0/0
	(2)	Q9C0C2-2, TNKS1BP1, 182 kDa tankyrase-1-binding protein	
1429		P13805-1-3, TNNT1, Troponin T, slow skeletal muscle	3/0/1
1430		P45379-3,4, TNNT2, Troponin T, cardiac muscle	1/0/1
1431		Q92973-1-3, TNPO1, Transportin-1	2/0/0
1432	(1)	P22105-1,3, TNXB, Tenascin-X	1/11/0
	(2)	P22105-2, TNXB, Tenascin-X	
	(3)	P22105-4, TNXB, Tenascin-X	
	(4)	Q16473, TNXA, Putative tenascin-XA	

1433		Q5JTV8-1,3, TOR1AIP1, Torsin-1A-interacting protein 1	3/0/0
1434		O14657, TOR1B, Torsin-1B	1/0/0
1435		P09493-1, TPM1, Tropomyosin alpha-1 chain	0/0/20
1436		P09493-4,10, TPM1, Tropomyosin alpha-1 chain	0/0/19
1437	(1)	P09493-3, TPM1, Tropomyosin alpha-1 chain	0/0/19
	(2)	P09493-2, TPM1, Tropomyosin alpha-1 chain	
1438		P09493-5, TPM1, Tropomyosin alpha-1 chain	1/0/12
1439		P09493-6, TPM1, Tropomyosin alpha-1 chain	0/0/18
1440		P09493-7, TPM1, Tropomyosin alpha-1 chain	0/0/18
1441		P09493-8, TPM1, Tropomyosin alpha-1 chain	0/0/17
1442		P09493-9, TPM1, Tropomyosin alpha-1 chain	0/0/20
1443		P07951-1, TPM2, Tropomyosin beta chain	3/0/18
1444	(1)	P07951-2, TPM2, Tropomyosin beta chain	0/0/17
	(2)	P07951-3, TPM2, Tropomyosin beta chain	
1445		P06753-1, TPM3, Tropomyosin alpha-3 chain	1/0/17
1446	(1)	P06753-2, TPM3, Tropomyosin alpha-3 chain	0/0/18
	(2)	P06753-3,6, TPM3, Tropomyosin alpha-3 chain	
1447	(1)	P06753-5, TPM3, Tropomyosin alpha-3 chain	0/0/21
	(2)	P06753-4, TPM3, Tropomyosin alpha-3 chain	
	(3)	P06753-7, TPM3, Tropomyosin alpha-3 chain	
1448		P67936-1, TPM4, Tropomyosin alpha-4 chain	6/0/15
1449		P67936-2, TPM4, Tropomyosin alpha-4 chain	0/0/18
1450		O14773-1,2, TPP1, Tripeptidyl-peptidase 1	2/0/0
1451	(1)	Q13263-1, TRIM28, Transcription intermediary factor 1-beta	1/0/0
	(2)	Q13263-2, TRIM28, Transcription intermediary factor 1-beta	
1452	(1)	O75382-4, TRIM3, Tripartite motif-containing protein 3	3/0/0
	(2)	O75382-1-3, TRIM3, Tripartite motif-containing protein 3	
1453		Q15654-1, TRIP6, Thyroid receptor-interacting protein 6	1/0/0
1454		P10155-1-5, TROVE2, 60 kDa SS-A/Ro ribonucleoprotein	2/0/0
1455		Q8WUA8, TSKU, Tsukushin	10/0/0
1456		Q13630, TSTA3, GDP-L-fucose synthase	2/0/0

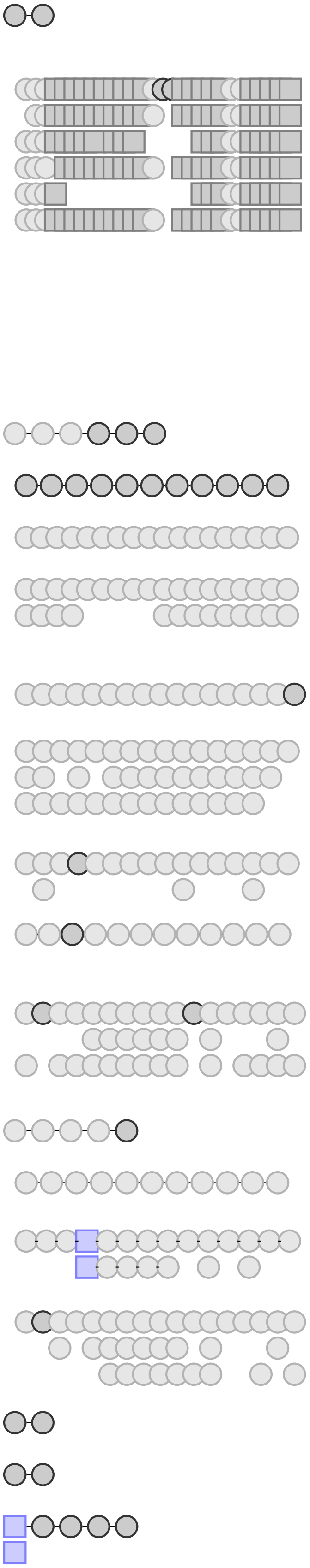

1457		Q5SRH9-1,4-6, TTC39A, Tetratricopeptide repeat protein 39A	2/0/0
			2/20/6
1458	(1)	Q8WZ42-12, TTN, Titin	
	(2)	Q8WZ42-2, TTN, Titin	
	(3)	Q8WZ42-5, TTN, Titin	
	(4)	Q8WZ42-4,7,11, TTN, Titin	
	(5)	Q8WZ42-3,9,10, TTN, Titin	
	(6)	Q8WZ42-1,8,13, TTN, Titin	
1459		Q8WZ42-6, TTN, Titin	3/0/3
1460		P02766, TTR, Transthyretin	11/0/0
1461		Q71U36-1,2, TUBA1A, Tubulin alpha-1A chain	0/0/18
			0/0/18
1462	(1)	P68363-1, TUBA1B, Tubulin alpha-1B chain	
	(2)	P68363-2, TUBA1B, Tubulin alpha-1B chain	
1463		Q9BQE3, TUBA1C, Tubulin alpha-1C chain	1/0/16
1464	(1)	Q13748-1, TUBA3C, Tubulin alpha-3C/D chain	0/0/16
	(2)	Q6PEY2, TUBA3E, Tubulin alpha-3E chain	
	(3)	Q13748-2, TUBA3C, Tubulin alpha-3C/D chain	
1465	(1)	P68366-1,2, TUBA4A, Tubulin alpha-4A chain	1/0/15
	(2)	A6NHL2-1,2, TUBAL3, Tubulin alpha chain-like 3	
1466		Q9NY65-1,2, TUBA8, Tubulin alpha-8 chain	1/0/11
1467	(1)	P07437, TUBB, Tubulin beta chain	2/0/15
	(2)	A6NNZ2, NA, Tubulin beta-8 chain-like protein LOC260334	
	(3)	Q13885, TUBB2A, Tubulin beta-2A chain	
	·	Q9BVA1, TUBB2B, Tubulin beta-2B chain	
1468		Q9H4B7, TUBB1, Tubulin beta-1 chain	1/0/4
1469		Q13509-1, TUBB3, Tubulin beta-3 chain	0/0/11
1470	(1)	P04350, TUBB4A, Tubulin beta-4A chain	0/0/13
	(2)	Q9BUF5, TUBB6, Tubulin beta-6 chain	
1471	(1)	P68371, TUBB4B, Tubulin beta-4B chain	1/0/16
	(2)	Q3ZCM7, TUBB8, Tubulin beta-8 chain	
	(3)	Q13509-2, TUBB3, Tubulin beta-3 chain	
1472		P49411, TUFM, Elongation factor Tu, mitochondrial	2/0/0
1473		Q6IBS0, TWF2, Twinfilin-2	2/0/0
			4/0/0
1474	(1)	Q9GZX9-1, TWSG1, Twisted gastrulation protein homolog 1	
	(2)	Q9GZX9-2, TWSG1, Twisted gastrulation protein homolog 1	

1475		P10599-1,2, TXN, Thioredoxin	2/0/0
1476	(1)	Q8NBS9-1, TXNDC5, Thioredoxin domain-containing protein 5	1/0/1
	(2)	Q8NBS9-2, TXNDC5, Thioredoxin domain-containing protein 5	
1477		O43396, TXNL1, Thioredoxin-like protein 1	1/0/0
1478	(1)	P19971-1, TYMP, Thymidine phosphorylase	1/0/0
	(2)	P19971-2, TYMP, Thymidine phosphorylase	
1479		P22314-1,2, UBA1, Ubiquitin-like modifier-activating enzyme 1	18/0/0
1480		Q9UBT2-1,2, UBA2, SUMO-activating enzyme subunit 2	3/0/0
1481		P61086-1,3, UBE2K, Ubiquitin-conjugating enzyme E2 K	3/0/0
1482		P61081, UBE2M, NEDD8-conjugating enzyme Ubc12	2/0/0
1483		P61088, UBE2N, Ubiquitin-conjugating enzyme E2 N	3/0/0
		Q5JXB2, UBE2NL, Putative ubiquitin-conjugating enzyme E2 N-like	
1484		P15374, UCHL3, Ubiquitin carboxyl-terminal hydrolase isozyme L3	2/0/0
1485	(1)	O60701-1, UGDH, UDP-glucose 6-dehydrogenase	1/7/0
	(2)	O60701-2, UGDH, UDP-glucose 6-dehydrogenase	
	(3)	O60701-3, UGDH, UDP-glucose 6-dehydrogenase	
1486		Q9NYU2-1,2, UGGT1, UDP-glucose:glycoprotein glucosyltransferase 1	1/0/0
1487	(1)	Q16851-2, UGP2, UTP--glucose-1-phosphate uridylyltransferase	1/0/0
	(2)	Q16851-1, UGP2, UTP--glucose-1-phosphate uridylyltransferase	
1488		P22695, UQCRC2, Cytochrome b-c1 complex subunit 2, mitochondrial	3/0/0
1489		P07919, UQCRH, Cytochrome b-c1 complex subunit 6, mitochondrial	2/0/0
1490		O60763-1,2, USO1, General vesicular transport factor p115	11/0/0
1491		P54578-1-3, USP14, Ubiquitin carboxyl-terminal hydrolase 14	1/0/0
1492		P45974-1,2, USP5, Ubiquitin carboxyl-terminal hydrolase 5	4/0/0
1493		Q93008-1,3, USP9X, Probable ubiquitin carboxyl-terminal hydrolase FAF-X	2/0/6
1494		O00507-1,2, USP9Y, Probable ubiquitin carboxyl-terminal hydrolase FAF-Y	3/0/6

1495		P26640-1, VARS, Valine--tRNA ligase	3/0/0
1496		P50552, VASP, Vasodilator-stimulated phosphoprotein	1/0/0
1497	(1)	Q99536-1, VAT1, Synaptic vesicle membrane protein VAT-1 homolog	1/3/0
	(2)	Q99536-2, VAT1, Synaptic vesicle membrane protein VAT-1 homolog	
	(3)	Q99536-3, VAT1, Synaptic vesicle membrane protein VAT-1 homolog	
1498	(1)	P19320-1,3, VCAM1, Vascular cell adhesion protein 1	1/0/0
	(2)	P19320-2, VCAM1, Vascular cell adhesion protein 1	
1499	(1)	P18206-1,2, VCL, Vinculin	29/0/0
	(2)	P18206-3, VCL, Vinculin	
1500		P55072, VCP, Transitional endoplasmic reticulum ATPase	13/0/1
1501		P21796, VDAC1, Voltage-dependent anion-selective channel protein 1	2/0/0
1502		P49767, VEGFC, Vascular endothelial growth factor C	17/0/0
1503		O15240, VGF, Neurosecretory protein VGF [Cleaved into: Neuroendocrine regulatory peptide-1	13/0/0
1504		P08670, VIM, Vimentin	26/0/10
1505		P98155-1,2, VLDLR, Very low-density lipoprotein receptor	7/0/0
1506		Q7Z5L0-1, VMO1, Vitelline membrane outer layer protein 1 homolog	1/0/0
1507		Q96QK1, VPS35, Vacuolar protein sorting-associated protein 35	7/0/0
1508		O75351, VPS4B, Vacuolar protein sorting-associated protein 4B	2/0/0
1509		Q9Y279-1-3, VSIG4, V-set and immunoglobulin domain-containing protein 4	1/0/0
1510		P04004, VTN, Vitronectin	19/0/0
1511	(1)	Q6PCB0-1, VWA1, von Willebrand factor A domain-containing protein 1	3/0/0
	(2)	Q6PCB0-2, VWA1, von Willebrand factor A domain-containing protein 1	
1512	(1)	P04275-1, VWF, von Willebrand factor	72/0/0
	(2)	P04275-2, VWF, von Willebrand factor	
1513		P23381-1,2, WARS, Tryptophan--tRNA ligase, cytoplasmic	5/0/0
1514		Q8IWB7, WDFY1, WD repeat and FYVE domain-containing protein 1	2/0/0

1515		O75083-1,3, WDR1, WD repeat-containing protein 1	3/0/0
1516		Q9HC57, WFDC1, WAP four-disulfide core domain protein 1	2/0/0
1517	(1)	O76076-1, WISP2, WNT1-inducible-signaling pathway protein 2	7/0/1
	(2)	O76076-2, WISP2, WNT1-inducible-signaling pathway protein 2	
1518	(1)	Q9H4A3-7, WNK1, Serine/threonine-protein kinase WNK1	2/0/0
	(2)	Q9H4A3-1,2,4-6, WNK1, Serine/threonine-protein kinase WNK1	
1519		P41221-1,2, WNT5A, Protein Wnt-5a	6/0/1
1520		Q9H1J7, WNT5B, Protein Wnt-5b	2/0/1
1521		O14980, XPO1, Exportin-1	3/0/0
1522		P13010, XRCC5, X-ray repair cross-complementing protein 5	7/0/0
1523		P12956-1,2, XRCC6, X-ray repair cross-complementing protein 6	10/0/0
1524		Q8NBI6-1, XXYLT1, Xyloside xylosyltransferase 1	3/0/0
1525		P54577, YARS, Tyrosine--tRNA ligase, cytoplasmic	3/0/0
1526	(1)	O15498-1, YKT6, Synaptobrevin homolog YKT6	1/0/0
	(2)	O15498-2, YKT6, Synaptobrevin homolog YKT6	
1527		P31946-1,2, YWHAB, 14-3-3 protein beta/alpha	5/0/9
1528	(1)	P62258-1, YWHAE, 14-3-3 protein epsilon	3/0/3
	(2)	P62258-2, YWHAE, 14-3-3 protein epsilon	
1529		P61981, YWHAG, 14-3-3 protein gamma	9/0/6
1530		Q04917, YWHAH, 14-3-3 protein eta	8/0/5
1531		P27348, YWHAQ, 14-3-3 protein theta	9/0/7
1532	(1)	P63104-1, YWHAZ, 14-3-3 protein zeta/delta	8/0/7
	(2)	P63104-2, YWHAZ, 14-3-3 protein zeta/delta	
1533		Q15916, ZBTB6, Zinc finger and BTB domain-containing protein 6	3/0/0
1534		P61129, ZC3H6, Zinc finger CCCH domain-containing protein 6	2/0/1
1535		Q9P243-1,2,4, ZFAT, Zinc finger protein ZFAT	9/0/0
1536		Q68DK2-1,2, ZFYVE26, Zinc finger FYVE domain-containing protein 26	2/0/1

1537	(1)	Q9UL36-1, ZNF236, Zinc finger protein 236	1/0/0
	(2)	Q9UL36-2, ZNF236, Zinc finger protein 236	
1538		Q5VUA4-1, ZNF318, Zinc finger protein 318	2/0/0
1539		Q8TF45, ZNF418, Zinc finger protein 418	6/0/0
1540		Q96B54, ZNF428, Zinc finger protein 428	1/0/0
1541		Q96JM2-1,3, ZNF462, Zinc finger protein 462	7/0/0
1542		Q96ME7-1-3, ZNF512, Zinc finger protein 512	3/0/0
1543		Q8N9K5-1,2, ZNF565, Zinc finger protein 565	4/0/0
1544		Q15942-1,2, ZYX, Zyxin	2/0/0
1545		O43149-1,2, ZZEF1, Zinc finger ZZ-type and EF-hand domain-containing protein 1	6/0/0

Isobaric Tag Quantification Report
P7131_Run 5 *

isobar R package v1.18.0

February 7, 2017

Channel	Class
126	*P11-treat*
127	*P11-ctrl*
128	*P12-treat*
129	*P12-ctrl*
130	*P13-treat*
131	*P13-ctrl*

Column Descriptions

protein	Protein name, accession code, and description.
group	Protein group size, only displayed if it is not '1/1'. First number: Number of group reporters; second number: group size. See Protein Group Details for details.
peptides	Number of peptides for protein.
spectra	Number of idenfitied spectra for protein.
quant	Number of spectra with quantitative information for *ch2* versus *ch1*.
ratio	Ratio of protein in channel *ch2* relative to *ch1*.
*	Significance of protein ratio in sample.

1 Protein Relative Quantitation

Number of quantified proteins: P11-treat/P11-ctrl: 1612; P12-treat/P12-ctrl: 1582; P13-treat/P13-ctrl: 1624

1.1 Quantified Proteins

#	protein	group	peptides	spectra	ch1	ch2	quant	ratio
					127	126	112	**12.37**
**1**	136429		6	126	129	128	112	**0.02**
					131	130	110	**3.98**
					127	126	73	**28.18**
**2**	162648		41	258	129	128	60	**0.11**
					131	130	78	**1.68**
					127	126	3	**0.26**
**3**	A0A0A0MS14		3	3	129	128	3	**0.40**
					131	130	3	**1.61**

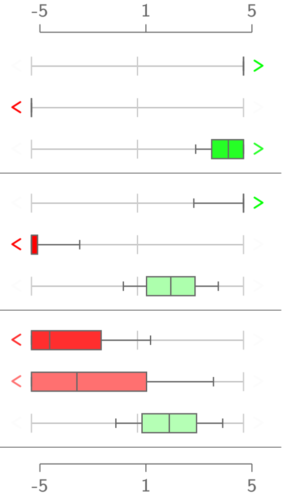

*This report was generated using the `isobar` R package version 1.18.0 [built using R 3.3.2; ; 2017-02-05 14:44:42 UTC; unix]. If you use it in published work, please cite 'Breitwieser FP *et al.*: General statistical modeling of data from protein relative expression isobaric tags, *Journal of Proteome Research* 2011' and 'Breitwieser FP and Colinge J: isobarPTM: A software tool for the quantitative analysis of post-translationally modified proteins,*Journal of Proteomics* 2013'

#	protein	group	peptides	spectra	ch1	ch2	quant	ratio
4	A0A0B4J1V0		2	2	127	126	2	**0.41**
					129	128	2	**5.59**
					131	130	2	**1.65**
5	A0A0C4DH25		3	8	127	126	1	**0.36**
					129	128	1	**9.83**
					131	130	1	**1.81**
6	A0A0C4DH29	1/2	2	8	127	126	1	**0.21**
					129	128		
					131	130	1	**0.54**
7	A1BG P04217$_1$: Alpha-1B-glycoprotein	1/2	13	19	127	126	19	**0.69**
					129	128	15	**0.47**
					131	130	18	**1.60**
8	A2M P01023: Alpha-2-macroglobulin		38	93	127	126	73	**0.45**
					129	128	67	**1.46**
					131	130	67	**0.90**
9	AARS P49588$_1$: Alanine--tRNA ligase, cytoplasmic	1/2	19	27	127	126	22	**0.87**
					129	128	21	**1.39**
					131	130	26	**0.43**
10	ABCE1 P61221: ATP-binding cassette sub-family E member 1		2	2	127	126	2	**0.97**
					129	128	2	**1.78**
					131	130	2	**0.99**
11	ABHD14A Q9BUJ0: Protein ABHD14A		3	3	127	126	3	**1.46**
					129	128	3	**0.40**
					131	130	3	**1.17**
12	ABHD14B Q96IU4$_1$: Protein ABHD14B	1/2	3	3	127	126	3	**1.12**
					129	128	2	**1.27**
					131	130	3	**0.19**
13	ABI1 Q8IZP0$_{1-12}$, ABI2 Q9NYB9$_{1,2,4}$: Abl interactor 1, Abl interactor 2	15/16	2	2	127	126	2	**0.88**
					129	128	1	**0.56**
					131	130	2	**0.81**
14	ACADVL P49748$_{1-3}$: Very long-chain specific acyl-CoA dehydrogenase, mitochondrial	3/3	6	7	127	126	5	**2.14**
					129	128	5	**1.07**
					131	130	6	**0.58**
15	ACBD3 Q9H3P7: Golgi resident protein GCP60		2	3	127	126	2	**0.13**
					129	128	2	**2.19**
					131	130	2	**1.84**

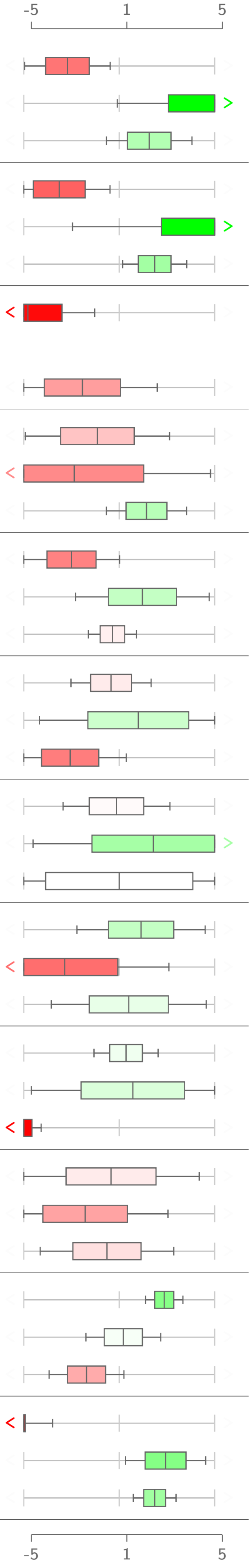

#	protein	group	peptides	spectra	ch1	ch2	quant	ratio
16	ACLY P53396$_{1-3}$: ATP-citrate synthase	3/3	5	5	127	126	5	**1.98**
					129	128	5	**0.47**
					131	130	5	**0.60**
17	ACOT9 Q9Y305$_{1-4}$: Acyl-coenzyme A thioesterase 9, mitochondrial	4/4	2	2	127	126	2	**2.54**
					129	128	2	**0.72**
					131	130	2	**0.56**
18	ACTB P60709, ACTG1 P63261: Actin, cytoplasmic 1, Actin, cytoplasmic 2	2/2	19	394	127	126	69	**0.62**
					129	128	62	**1.48**
					131	130	95	**0.48**
19	ACTBL2 Q562R1: Beta-actin-like protein 2		7	194	127	126		
					129	128	1	**7.70**
					131	130	1	**1.29**
20	ACTC1 P68032: Actin, alpha cardiac muscle 1	1/5	17	315	127	126	43	**0.48**
					129	128	38	**1.31**
					131	130	54	**0.46**
21	ACTN1 P12814$_{1-4}$: Alpha-actinin-1	4/4	26	44	127	126	18	**0.98**
					129	128	16	**0.93**
					131	130	19	**1.25**
22	ACTN2 P35609$_2$: Alpha-actinin-2	1/2	12	14	127	126	3	**2.27**
					129	128	3	**0.45**
					131	130	3	**1.63**
23	ACTN3 Q08043: Alpha-actinin-3		5	5	127	126	1	**0.81**
					129	128	1	**1.27**
					131	130	1	**1.30**
24	ACTN4 O43707$_1$: Alpha-actinin-4	1/3	33	55	127	126	28	**0.79**
					129	128	22	**1.58**
					131	130	30	**1.18**
25	ACTR1A P61163: Alpha-centractin		5	42	127	126	5	**5.10**
					129	128	3	**0.11**
					131	130	6	**3.97**
26	ACTR1B P42025: Beta-centractin		2	2	127	126	1	**2.07**
					129	128	1	**0.35**
					131	130	1	**0.75**
27	ACTR2 P61160$_1$: Actin-related protein 2	1/2	8	44	127	126	10	**1.44**
					129	128	8	**0.80**
					131	130	10	**0.60**

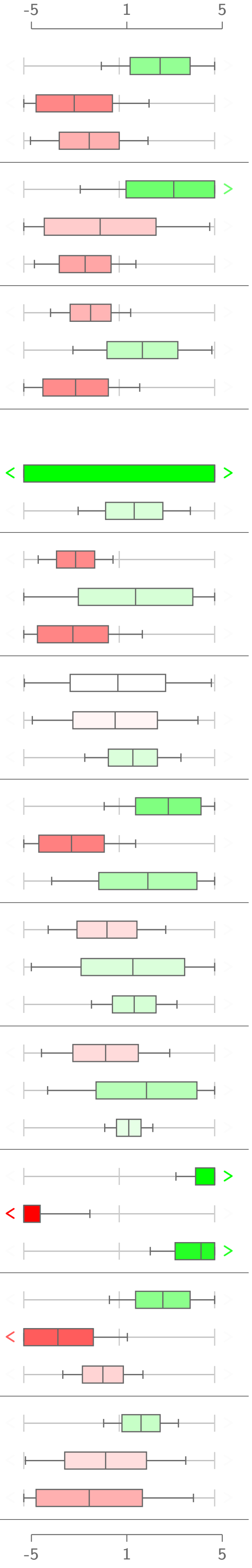

#	protein	group	peptides	spectra	ch1	ch2	quant	ratio
28	ACTR3 P61158: Actin-related protein 3		4	4	127	126	3	**1.32**
					129	128	2	**0.87**
					131	130	4	**0.52**
29	ADAM10 O14672$_1$: Disintegrin and metalloproteinase domain-containing protein 10		10	42	127	126	15	**0.32**
					129	128	15	**0.68**
					131	130	32	**2.23**
30	ADAM15 Q13444$_{1-10,12}$: Disintegrin and metalloproteinase domain-containing protein 15	11/13	4	4	127	126	4	**0.93**
					129	128	3	**0.59**
					131	130	4	**1.07**
31	ADAMDEC1 O15204$_{1,2}$: ADAM DEC1	2/2	5	6	127	126	6	**1.73**
					129	128	6	**0.30**
					131	130	5	**0.92**
32	ADAMTS1 Q9UHI8: A disintegrin and metalloproteinase with thrombospondin motifs 1		2	2	127	126	2	**0.14**
					129	128	2	**1.97**
					131	130	2	**3.11**
33	ADAMTS13 Q76LX8$_{1-3}$: A disintegrin and metalloproteinase with thrombospondin motifs 13	3/4	3	3	127	126	3	**0.52**
					129	128	2	**0.84**
					131	130	3	**1.45**
34	ADAMTS2 O95450$_1$: A disintegrin and metalloproteinase with thrombospondin motifs 2	1/2	4	6	127	126	3	**0.54**
					129	128	2	**0.44**
					131	130	4	**1.70**
35	ADAMTS5 Q9UNA0: A disintegrin and metalloproteinase with thrombospondin motifs 5		4	4	127	126	4	**0.46**
					129	128	3	**0.86**
					131	130	4	**2.15**
36	ADAMTS8 Q9UP79: A disintegrin and metalloproteinase with thrombospondin motifs 8		2	2	127	126	2	**1.00**
					129	128	2	**0.98**
					131	130	2	**3.18**
37	ADAMTSL2 Q86TH1: ADAMTS-like protein 2		2	3	127	126	3	**0.31**
					129	128	3	**0.97**
					131	130	3	**2.07**
38	ADAMTSL3 P82987$_1$: ADAMTS-like protein 3	1/2	6	16	127	126	11	**3.22**
					129	128	7	**0.12**
					131	130	15	**3.45**
39	ADAMTSL4 Q6UY14$_{1,3}$: ADAMTS-like protein 4	2/3	12	12	127	126	10	**1.18**
					129	128	10	**0.48**
					131	130	11	**1.39**

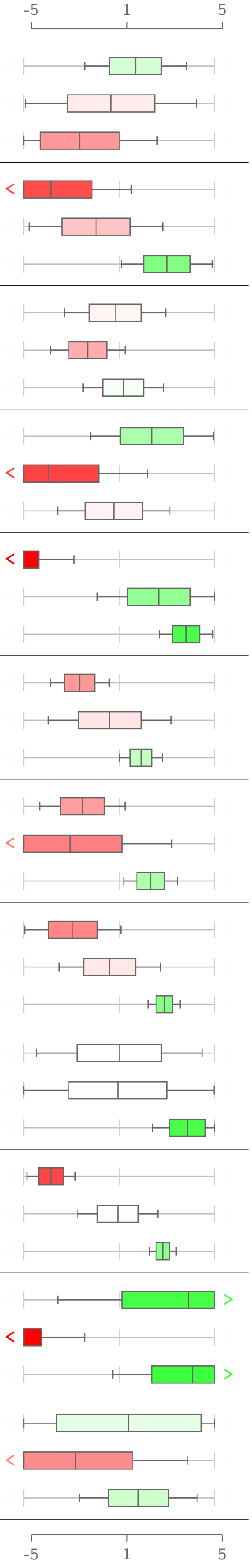

#	protein	group	peptides	spectra	ch1	ch2	quant	ratio
**40**	ADH1B P00325$_{1}$: Alcohol dehydrogenase 1B	1/4	11	15	127	126	13	**1.08**
					129	128	11	**0.50**
					131	130	15	**0.23**
**41**	ADH5 P11766: Alcohol dehydrogenase class-3		12	17	127	126	17	**0.88**
					129	128	14	**2.60**
					131	130	16	**0.43**
**42**	ADIPOQ Q15848: Adiponectin		4	10	127	126	10	**1.23**
					129	128	9	**1.44**
					131	130	9	**6.17**
**43**	ADK P55263$_{1-4}$: Adenosine kinase	4/4	2	2	127	126	2	**2.34**
					129	128	2	**0.47**
					131	130	2	**0.26**
**44**	ADRM1 Q16186: Proteasomal ubiquitin receptor ADRM1		2	2	127	126		
					129	128	1	**0.12**
					131	130	2	**0.54**
**45**	AEBP1 Q8IUX7$_{1}$: Adipocyte enhancer-binding protein 1	1/2	33	70	127	126	64	**0.54**
					129	128	66	**0.66**
					131	130	67	**2.41**
**46**	AFM P43652: Afamin		32	67	127	126	58	**0.44**
					129	128	53	**1.76**
					131	130	62	**2.07**
**47**	AGR3 Q8TD06: Anterior gradient protein 3		3	3	127	126	3	**3.86**
					129	128	2	**0.47**
					131	130	3	**0.72**
**48**	AGRN O00468$_{3,6}$: Agrin [Cleaved into: Agrin N-terminal 110 kDa subunit; Agrin C-terminal 110 . . .	2/7	21	21	127	126	19	**1.38**
					129	128	19	**0.34**
					131	130	19	**0.55**
**49**	AGT P01019: Angiotensinogen		11	35	127	126	27	**0.70**
					129	128	17	**1.19**
					131	130	27	**0.28**
**50**	AHCY P23526$_{1,2}$: Adenosylhomocysteinase	2/2	6	9	127	126	9	**0.94**
					129	128	5	**1.53**
					131	130	9	**0.67**
**51**	AHCYL1 O43865$_{1,2}$: Adenosylhomocysteinase 2	2/6	7	7	127	126	5	**0.94**
					129	128	5	**0.60**
					131	130	6	**0.91**

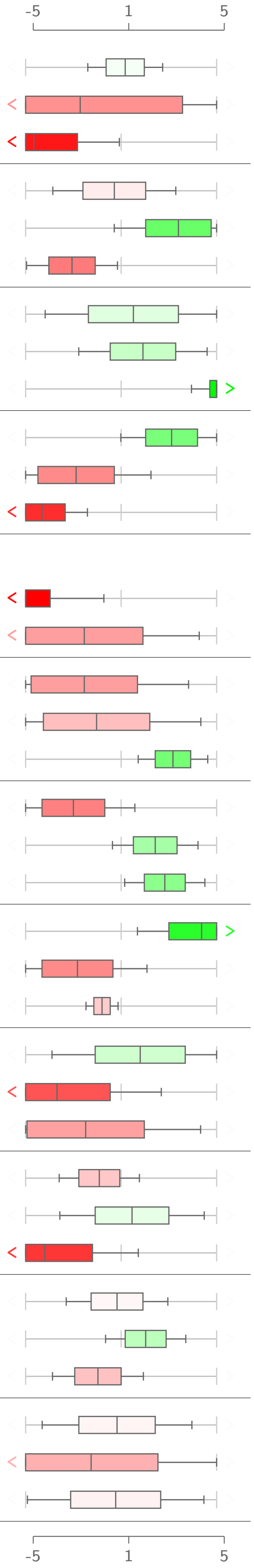

#	protein	group	peptides	spectra	ch1	ch2	quant	ratio
52	AHNAK Q09666$_1$: Neuroblast differentiation-associated protein AHNAK	1/2	20	24	127	126	22	**2.18**
					129	128	19	**0.31**
					131	130	21	**1.01**
53	AHSA1 O95433$_1$: Activator of 90 kDa heat shock protein ATPase homolog 1		3	4	127	126	4	**2.77**
					129	128	4	**0.25**
					131	130	4	**0.27**
54	AHSG P02765: Alpha-2-HS-glycoprotein		12	301	127	126	271	**0.49**
					129	128	258	**1.70**
					131	130	288	**0.96**
55	AIP O00170: AH receptor-interacting protein		3	4	127	126	4	**1.08**
					129	128	3	**0.56**
					131	130	4	**1.65**
56	AK1 P00568: Adenylate kinase isoenzyme 1		4	5	127	126	4	**1.25**
					129	128	5	**0.77**
					131	130	5	**0.32**
57	AK3 Q9UIJ7$_{1,2}$: GTP:AMP phosphotransferase AK3, mitochondrial	2/3	4	4	127	126	4	**2.78**
					129	128	3	**0.92**
					131	130	4	**0.54**
58	AKR7A2 O43488: Aflatoxin B1 aldehyde reductase member 2		6	9	127	126	7	**0.58**
					129	128	6	**1.76**
					131	130	8	**0.64**
59	AKT1 P31749$_1$: RAC-alpha serine/threonine-protein kinase	1/2	5	5	127	126	5	**1.17**
					129	128	3	**2.01**
					131	130	3	**0.44**
60	ALB P02768$_1$: Serum albumin	1/3	61	2625	127	126	2063	**0.52**
					129	128	1681	**2.13**
					131	130	2282	**1.48**
61	ALDH1A1 P00352: Retinal dehydrogenase 1		2	2	127	126	1	**1.27**
					129	128	1	**5.70**
					131	130	1	**1.40**
62	ALDH1A2 O94788$_1$: Retinal dehydrogenase 2	1/4	10	12	127	126	9	**0.88**
					129	128	7	**0.84**
					131	130	9	**0.65**
63	ALDH1A3 P47895: Aldehyde dehydrogenase family 1 member A3		12	13	127	126	10	**0.76**
					129	128	10	**0.57**
					131	130	11	**0.57**

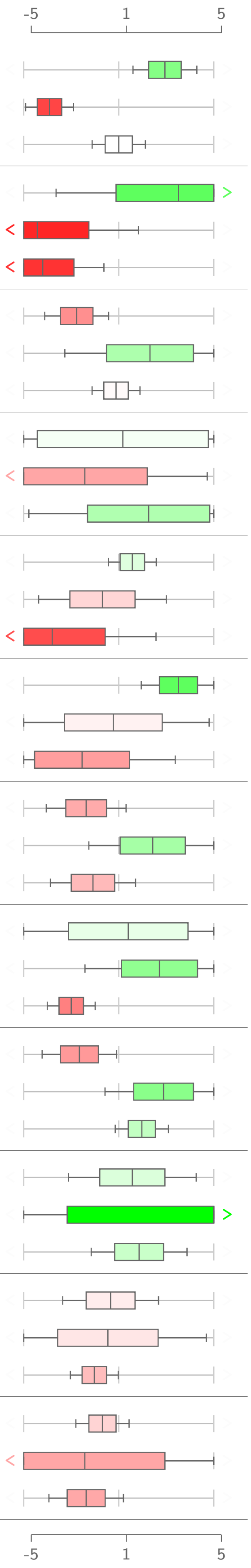

#	protein	group	peptides	spectra	ch1	ch2	quant	ratio
64	ALDH1B1 P30837: Aldehyde dehydrogenase X, mitochondrial		3	3	127	126	1	**1.99**
					129	128	1	**1.38**
					131	130	1	**0.47**
65	ALDH2 P05091$_{1,2}$: Aldehyde dehydrogenase, mitochondrial	2/2	3	4	127	126	3	**0.69**
					129	128	3	**1.84**
					131	130	3	**0.68**
66	ALDH7A1 P49419$_{1,2}$: Alpha-aminoadipic semialdehyde dehydrogenase	2/3	7	7	127	126	6	**2.07**
					129	128	6	**0.45**
					131	130	7	**0.43**
67	ALDH9A1 P49189$_{1}$: 4-trimethylaminobutyraldehyde dehydrogenase	1/2	11	16	127	126	12	**1.18**
					129	128	11	**1.22**
					131	130	10	**0.66**
68	ALDOA P04075$_{1,2}$: Fructose-bisphosphate aldolase A	2/3	7	7	127	126	6	**1.25**
					129	128	7	**1.01**
					131	130	7	**0.57**
69	ALMS1 Q8TCU4$_{1-3}$: Alstrom syndrome protein 1	3/3	5	10	127	126	6	**0.66**
					129	128	6	**1.16**
					131	130	6	**3.55**
70	ALOX15 P16050$_{1}$: Arachidonate 15-lipoxygenase	1/2	15	22	127	126	21	**1.02**
					129	128	16	**2.83**
					131	130	20	**0.25**
71	ALYREF Q86V81: THO complex subunit 4		2	2	127	126	2	**0.90**
					129	128	2	**0.96**
					131	130	2	**1.67**
72	AMBP P02760: Protein AMBP [Cleaved into: Alpha-1-microglobulin		14	199	127	126	126	**0.85**
					129	128	65	**1.41**
					131	130	132	**0.77**
73	ANG P03950: Angiogenin		13	116	127	126	105	**0.69**
					129	128	104	**0.24**
					131	130	112	**1.97**
74	ANGPTL2 Q9UKU9$_{1}$: Angiopoietin-related protein 2	1/2	7	11	127	126	11	**0.33**
					129	128	8	**1.16**
					131	130	11	**1.49**
75	ANGPTL3 Q9Y5C1: Angiopoietin-related protein 3		2	2	127	126	2	**0.44**
					129	128	2	**4.07**
					131	130	2	**1.13**

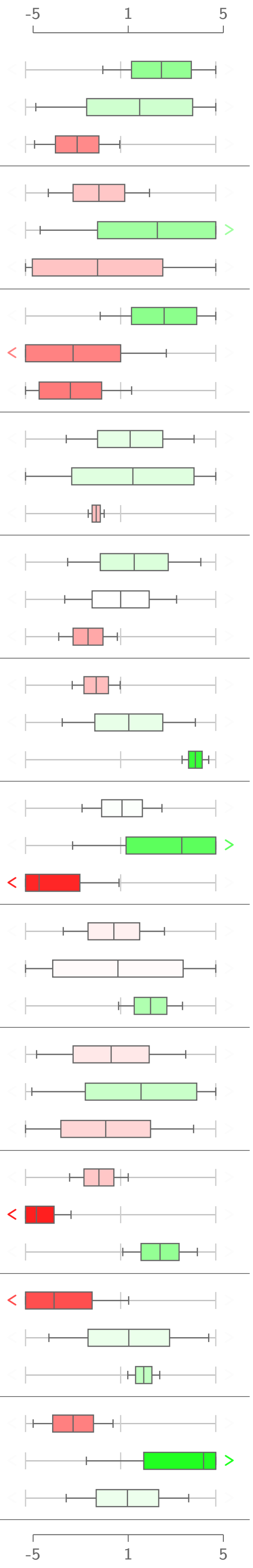

#	protein	group	peptides	spectra	ch1	ch2	quant	ratio
**76**	ANGPTL4 Q9BY76$_{1}$: Angiopoietin-related protein 4	1/3	8	14	127	126	12	**0.61**
					129	128	13	**0.57**
					131	130	14	**1.84**
**77**	ANGPTL6 Q8NI99: Angiopoietin-related protein 6		3	3	127	126	3	**0.52**
					129	128	3	**1.21**
					131	130	3	**1.93**
**78**	ANK2 Q01484$_{4}$: Ankyrin-2		3	4	127	126	3	**0.99**
					129	128	2	**1.59**
					131	130	3	**0.31** <
**79**	ANKFY1 Q9P2R3$_{1,2,4}$: Rabankyrin-5	3/3	4	4	127	126	4	**2.26**
					129	128	3	**0.88**
					131	130	4	**0.55**
**80**	ANTXR1 Q9H6X2$_{1,5}$: Anthrax toxin receptor 1	2/6	9	12	127	126	11	**1.03**
					129	128	10	**1.27**
					131	130	11	**0.30** <
**81**	ANTXR2 P58335$_{4}$: Anthrax toxin receptor 2	1/4	15	20	127	126	16	**1.18**
					129	128	10	**1.38**
					131	130	19	**0.23** <
**82**	ANXA1 P04083: Annexin A1		12	21	127	126	17	**1.39**
					129	128	16	**0.67**
					131	130	18	**0.39**
**83**	ANXA11 P50995$_{1,2}$: Annexin A11	2/2	7	8	127	126	7	**1.73**
					129	128	5	**0.44** <
					131	130	8	**0.97**
**84**	ANXA2 P07355$_{1,2}$: Annexin A2	2/3	17	27	127	126	25	**2.12**
					129	128	24	**0.36**
					131	130	26	**0.49**
**85**	ANXA3 P12429: Annexin A3		6	6	127	126	6	**1.48**
					129	128	3	**0.75** <
					131	130	4	**0.62**
**86**	ANXA4 P09525$_{1,2}$: Annexin A4	2/2	5	6	127	126	5	**3.35**
					129	128	5	**0.34** <
					131	130	5	**0.37**
**87**	ANXA5 P08758: Annexin A5		10	10	127	126	9	**2.64**
					129	128	8	**0.26** <
					131	130	9	**0.51**

#	protein	group	peptides	spectra	ch1	ch2	quant	ratio
**88**	ANXA7 P20073$_{1,2}$: Annexin A7	2/2	3	4	127	126	4	**2.06**
					129	128	3	**0.47**
					131	130	4	**0.61**
**89**	ANXA9 O76027: Annexin A9		4	5	127	126	5	**1.30**
					129	128	4	**0.53**
					131	130	5	**0.85**
**90**	AOX1 Q06278: Aldehyde oxidase		2	2	127	126	2	**1.02**
					129	128	2	**2.18**
					131	130	2	**0.64**
**91**	AP1B1 Q10567$_{1-4}$: AP-1 complex subunit beta-1	4/4	14	18	127	126	4	**1.07**
					129	128	3	**1.11**
					131	130	3	**0.70**
**92**	AP1G1 O43747$_{1,2}$: AP-1 complex subunit gamma-1	2/2	8	9	127	126	9	**2.83**
					129	128	9	**0.14**
					131	130	9	**0.32**
**93**	AP1M1 Q9BXS5$_{1,2}$: AP-1 complex subunit mu-1	2/2	5	6	127	126	5	**1.56**
					129	128	4	**1.35**
					131	130	6	**0.26**
**94**	AP2A1 O95782$_{1,2}$: AP-2 complex subunit alpha-1	2/2	16	19	127	126	9	**1.20**
					129	128	6	**1.35**
					131	130	9	**0.53**
**95**	AP2A2 O94973$_{1-3}$: AP-2 complex subunit alpha-2	3/3	11	12	127	126	2	**1.31**
					129	128	2	**1.00**
					131	130	2	**0.54**
**96**	AP2B1 P63010$_{1,2}$: AP-2 complex subunit beta	2/3	16	20	127	126	6	**1.17**
					129	128	5	**2.38**
					131	130	6	**0.40**
**97**	AP2M1 Q96CW1$_{1,2}$: AP-2 complex subunit mu	2/2	5	5	127	126	4	**1.06**
					129	128	5	**0.65**
					131	130	5	**0.44**
**98**	AP3B1 O00203$_{1,3}$: AP-3 complex subunit beta-1	2/2	5	5	127	126	3	**1.53**
					129	128	4	**0.38**
					131	130	4	**1.64**
**99**	AP3B2 Q13367$_{1,3,4}$: AP-3 complex subunit beta-2	3/3	2	2	127	126	1	**0.72**
					129	128	1	**1.54**
					131	130	1	**3.25**

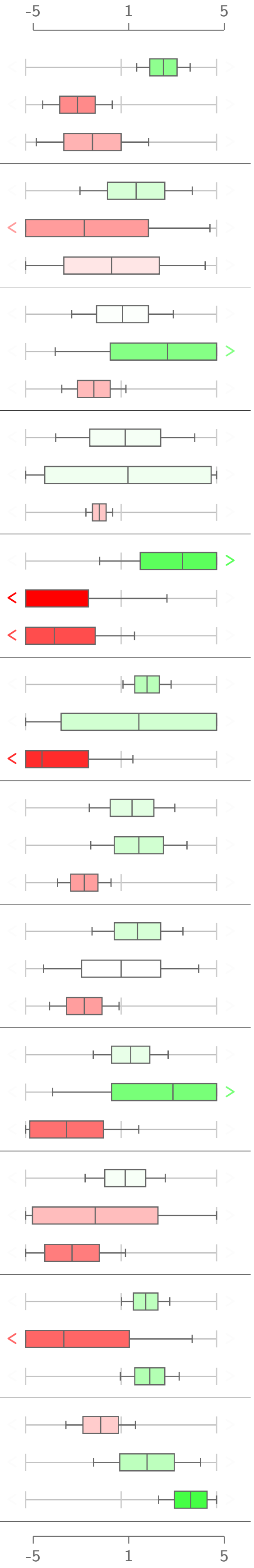

#	protein	group	peptides	spectra	ch1	ch2	quant	ratio
**100**	AP3D1 O14617$_5$: AP-3 complex subunit delta-1	1/5	6	7	127	126	6	**1.13**
					129	128	5	**0.80**
					131	130	7	**1.29**
**102**	APCS P02743: Serum amyloid P-component		9	280	127	126	241	**0.22**
					129	128	232	**1.32**
					131	130	274	**1.64**
**103**	APEH P13798: Acylamino-acid-releasing enzyme		5	7	127	126	6	**1.03**
					129	128	5	**0.99**
					131	130	7	**2.45**
**104**	API5 Q9BZZ5$_{1,2,4-6}$: Apoptosis inhibitor 5	5/6	4	4	127	126	3	**1.12**
					129	128	3	**2.37**
					131	130	4	**0.42**
**105**	APLP2 Q06481$_{1,3,6}$: Amyloid-like protein 2	3/6	3	4	127	126	4	**1.09**
					129	128	2	**3.50**
					131	130	4	**2.69**
**106**	APMAP Q9HDC9$_1$: Adipocyte plasma membrane-associated protein	1/2	9	13	127	126	11	**0.52**
					129	128	10	**0.98**
					131	130	13	**0.91**
**107**	APOA1 P02647: Apolipoprotein A-I		38	1963	127	126	1567	**0.32**
					129	128	1416	**1.98**
					131	130	1829	**4.37**
**108**	APOA2 P02652: Apolipoprotein A-II		5	276	127	126	233	**0.49**
					129	128	237	**2.00**
					131	130	265	**4.13**
**109**	APOA4 P06727: Apolipoprotein A-IV		37	1549	127	126	1269	**0.26**
					129	128	1250	**0.97**
					131	130	1369	**2.44**
**110**	APOB P04114: Apolipoprotein B-100		271	2555	127	126	2186	**1.23**
					129	128	1359	**0.66**
					131	130	2331	**0.25**
**111**	APOBR Q0VD83$_{1,3,4}$: Apolipoprotein B receptor	3/3	4	4	127	126	3	**1.18**
					129	128	3	**1.07**
					131	130	3	**2.07**
**112**	APOC1 P02654: Apolipoprotein C-I		6	96	127	126	91	**0.84**
					129	128	84	**0.46**
					131	130	90	**0.15**

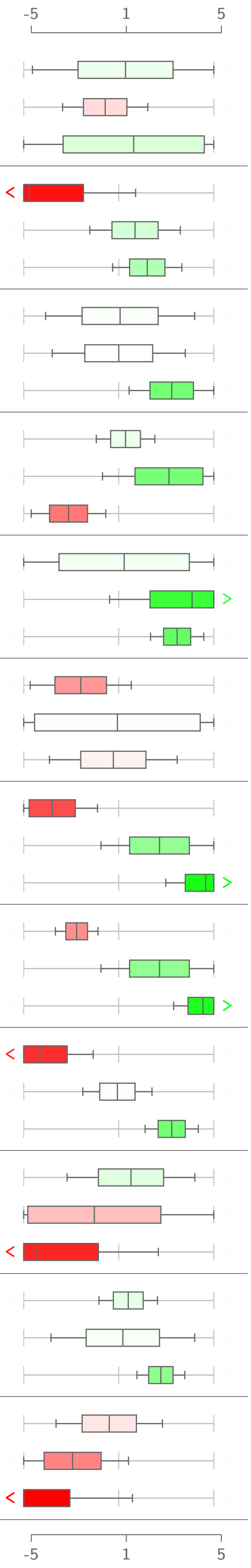

#	protein	group	peptides	spectra	ch1	ch2	quant	ratio
**113**	APOC2 P02655: Apolipoprotein C-II		4	33	127	126	32	**0.79**
					129	128	25	**2.00**
					131	130	28	**0.67**
**114**	APOC3 P02656: Apolipoprotein C-III		7	329	127	126	274	**0.42**
					129	128	239	**1.33**
					131	130	296	**0.95**
**115**	APOC4 P55056: Apolipoprotein C-IV		6	10	127	126	10	**1.46**
					129	128	10	**1.04**
					131	130	9	**0.29**
**116**	APOD P05090: Apolipoprotein D		8	255	127	126	225	**0.35**
					129	128	208	**1.12**
					131	130	251	**3.12**
**117**	APOE P02649: Apolipoprotein E		27	264	127	126	232	**0.95**
					129	128	199	**1.08**
					131	130	243	**1.13**
**118**	APOF Q13790: Apolipoprotein F		6	10	127	126	10	**1.21**
					129	128	7	**0.26**
					131	130	10	**1.20**
**119**	APOH P02749: Beta-2-glycoprotein 1		12	29	127	126	27	**1.00**
					129	128	24	**1.14**
					131	130	27	**2.57**
**120**	APOL1 O14791$_{1-3}$: Apolipoprotein L1	3/3	19	139	127	126	124	**3.04**
					129	128	93	**0.54**
					131	130	117	**0.25**
**121**	APOL2 Q9BQE5: Apolipoprotein L2		8	9	127	126	9	**2.36**
					129	128	7	**0.53**
					131	130	7	**0.40**
**122**	APOM O95445$_1$: Apolipoprotein M		9	44	127	126		
					129	128		
					131	130	1	**0.59**
**123**	APOM O95445$_2$: Apolipoprotein M		10	48	127	126	5	**1.41**
					129	128	4	**1.47**
					131	130	5	**1.50**
**124**	APP P05067$_{1,3-9,11}$: Amyloid beta A4 protein	9/11	7	11	127	126	11	**0.42**
					129	128	11	**0.52**
					131	130	11	**2.57**

#	protein	group	peptides	spectra	ch1	ch2	quant	ratio
**125**	APPL1 Q9UKG1: DCC-interacting protein 13-alpha		2	2	127	126	1	**23.99**
					129	128	1	**0.10**
					131	130	1	**0.30**
**126**	APRT P07741$_{1,2}$: Adenine phosphoribosyltransferase	2/2	8	12	127	126	11	**0.73**
					129	128	12	**0.48**
					131	130	11	**0.65**
**127**	ARCN1 P48444$_1$: Coatomer subunit delta	1/2	12	13	127	126	12	**1.35**
					129	128	9	**0.85**
					131	130	12	**0.46**
**128**	ARF1 P84077, ARF3 P61204$_1$: ADP-ribosylation factor 1, ADP-ribosylation factor 3	2/3	6	10	127	126	4	**5.02**
					129	128	3	**0.20**
					131	130	4	**0.58**
**129**	ARF4 P18085: ADP-ribosylation factor 4		6	7	127	126	4	**1.56**
					129	128	3	**1.18**
					131	130	4	**0.32**
**130**	ARF5 P84085: ADP-ribosylation factor 5		5	7	127	126	2	**2.53**
					129	128	2	**0.40**
					131	130	2	**0.19**
**131**	ARHGAP1 Q07960: Rho GTPase-activating protein 1		7	10	127	126	9	**1.40**
					129	128	6	**0.63**
					131	130	10	**0.40**
**132**	ARHGAP17 Q68EM7$_{1,2,5,6}$: Rho GTPase-activating protein 17	4/4	3	6	127	126	6	**1.55**
					129	128	5	**1.18**
					131	130	6	**0.30**
**133**	ARHGAP18 Q8N392$_1$: Rho GTPase-activating protein 18	1/2	10	11	127	126	10	**1.62**
					129	128	7	**1.43**
					131	130	11	**0.46**
**134**	ARHGAP35 Q9NRY4: Rho GTPase-activating protein 35		4	7	127	126	5	**2.51**
					129	128	5	**0.31**
					131	130	3	**0.32**
**135**	ARHGDIA P52565$_1$: Rho GDP-dissociation inhibitor 1	1/2	6	12	127	126	9	**1.25**
					129	128	9	**1.40**
					131	130	11	**0.32**
**136**	ARHGDIB P52566: Rho GDP-dissociation inhibitor 2		3	3	127	126	2	**0.96**
					129	128	2	**0.83**
					131	130	3	**0.96**

#	protein	group	peptides	spectra	ch1	ch2	quant	ratio
**137**	ARHGEF7 Q14155$_{1,5}$: Rho guanine nucleotide exchange factor 7	2/2	2	3	127	126	3	**0.69**
					129	128	3	**5.83**
					131	130	3	**0.30**
**138**	ARL8A Q96BM9: ADP-ribosylation factor-like protein 8A		1	1	127	126	1	**1.27**
					129	128	1	**0.99**
					131	130	1	**0.29**
**139**	ARPC1B O15143: Actin-related protein 2/3 complex subunit 1B		2	2	127	126	2	**0.99**
					129	128	2	**0.79**
					131	130	2	**0.41**
**140**	ASGR2 P07307$_{1-3}$: Asialoglycoprotein receptor 2	3/3	3	4	127	126	3	**0.52**
					129	128	3	**2.07**
					131	130	4	**2.30**
**141**	ASMTL O95671$_{1-3}$: N-acetylserotonin O-methyltransferase-like protein	3/3	2	2	127	126	2	**3.19**
					129	128	1	**0.43**
					131	130	2	**0.36**
**142**	ASPH Q12797$_{1,10}$: Aspartyl/asparaginyl beta-hydroxylase	2/2	5	6	127	126	3	**0.58**
					129	128	4	**1.87**
					131	130	5	**1.27**
**143**	ATG3 Q9NT62$_{1,2}$: Ubiquitin-like-conjugating enzyme ATG3	2/2	4	6	127	126	4	**2.15**
					129	128	5	**1.16**
					131	130	6	**1.24**
**144**	ATIC P31939$_{1,2}$: Bifunctional purine biosynthesis protein PURH [Cleaved into: Bifunctional pu ...	2/2	11	16	127	126	14	**1.47**
					129	128	13	**0.80**
					131	130	16	**0.44**
**145**	ATL3 Q6DD88: Atlastin-3		5	6	127	126	6	**1.19**
					129	128	5	**1.24**
					131	130	5	**0.55**
**146**	ATP1A1 P05023$_{1-4}$: Sodium/potassium-transporting ATPase subunit alpha-1	4/6	6	7	127	126	3	**1.93**
					129	128	3	**0.29**
					131	130	3	**0.50**
**147**	ATP1A3 P13637$_{1-3}$: Sodium/potassium-transporting ATPase subunit alpha-3	3/3	4	5	127	126	1	**0.85**
					129	128	1	**2.58**
					131	130	1	**1.64**
**148**	ATP2A2 P16615$_{1,4}$: Sarcoplasmic/endoplasmic reticulum calcium ATPase 2	2/5	3	3	127	126	3	**1.69**
					129	128	3	**0.81**
					131	130	3	**0.71**

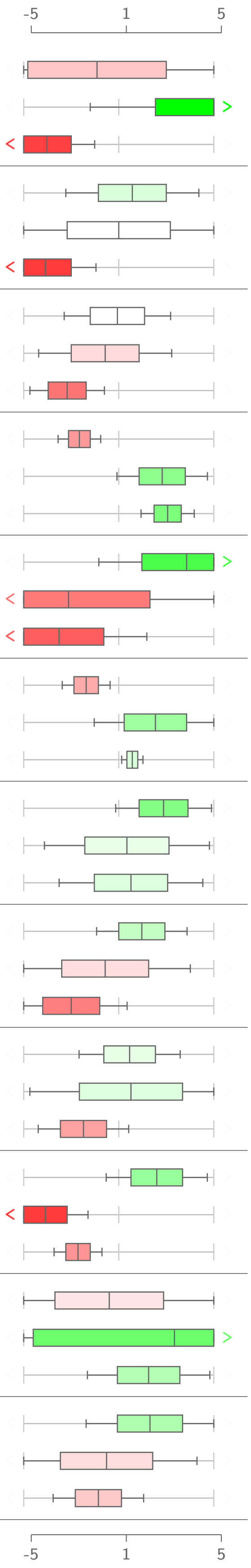

#	protein	group	peptides	spectra	ch1	ch2	quant	ratio
**149**	ATP5A1 P25705$_1$: ATP synthase subunit alpha, mitochondrial	1/3	19	25	127	126	24	**0.94**
					129	128	21	**2.32**
					131	130	22	**0.44**
**150**	ATP5B P06576: ATP synthase subunit beta, mitochondrial		8	10	127	126	9	**1.16**
					129	128	5	**2.42**
					131	130	9	**0.68**
**151**	ATP5C1 P36542$_{1,2}$: ATP synthase subunit gamma, mitochondrial	2/2	4	6	127	126	3	**1.90**
					129	128	3	**2.01**
					131	130	3	**0.52**
**152**	ATP5D P30049: ATP synthase subunit delta, mitochondrial		2	2	127	126	2	**1.95**
					129	128	2	**1.56**
					131	130	2	**1.27**
**153**	ATP5O P48047: ATP synthase subunit O, mitochondrial		2	2	127	126	2	**1.76**
					129	128	2	**2.15**
					131	130	2	**0.63**
**154**	ATP6AP1 Q15904: V-type proton ATPase subunit S1		2	2	127	126	2	**0.68**
					129	128	2	**0.46**
					131	130	2	**1.79**
**155**	ATP6AP2 O75787$_{1,2}$: Renin receptor	2/2	6	7	127	126	7	**0.86**
					129	128	7	**1.69**
					131	130	6	**0.41**
**156**	ATP6V1B2 P21281: V-type proton ATPase subunit B, brain isoform	1/2	5	5	127	126	3	**1.24**
					129	128	4	**0.65**
					131	130	4	**0.67**
**157**	ATP6V1E1 P36543$_{1,3}$: V-type proton ATPase subunit E 1	2/4	4	4	127	126	4	**0.81**
					129	128	4	**1.13**
					131	130	4	**1.19**
**158**	ATXN10 Q9UBB4$_1$: Ataxin-10	1/2	5	5	127	126	4	**1.12**
					129	128	3	**1.48**
					131	130	4	**0.38**
**159**	AXL P30530$_{1,2}$: Tyrosine-protein kinase receptor UFO	2/2	2	2	127	126	2	**0.34**
					129	128	2	**0.90**
					131	130	2	**1.70**
**160**	AZGP1 P25311: Zinc-alpha-2-glycoprotein		6	9	127	126	7	**1.93**
					129	128	9	**0.45**
					131	130	9	**1.32**

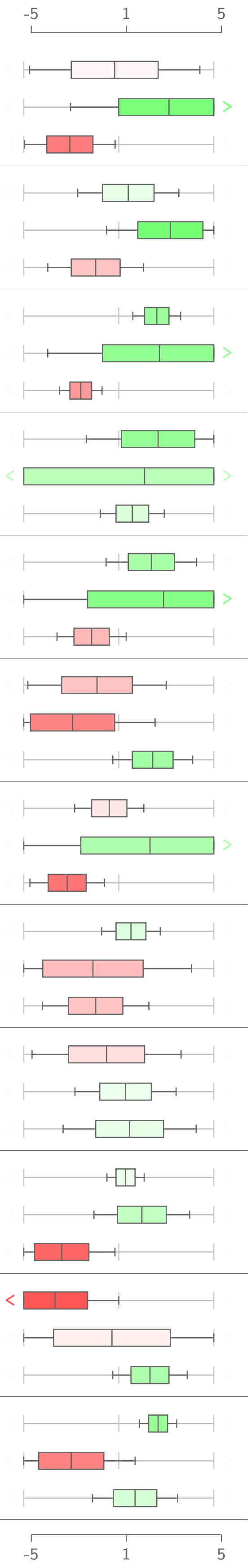

#	protein	group	peptides	spectra	ch1	ch2	quant	ratio
161	B2M P61769: Beta-2-microglobulin [Cleaved into: Beta-2-microglobulin form pI 5.3]		5	24	127	126	24	**1.50**
					129	128	23	**0.17**
					131	130	22	**1.47**
162	B3GAT3 O94766$_{1}$: Galactosylgalactosylxylosylprotein 3-beta-glucuronosyltransferase 3		2	2	127	126	1	**2.99**
					129	128	1	**0.03**
					131	130	2	**0.11**
163	B4GALT1 P15291$_{1,2}$: Beta-1,4-galactosyltransferase 1	2/2	8	15	127	126	15	**0.94**
					129	128	14	**0.92**
					131	130	13	**0.29**
164	B4GALT4 O60513: Beta-1,4-galactosyltransferase 4		7	8	127	126	7	**0.93**
					129	128	6	**0.07**
					131	130	8	**0.29**
165	B4GAT1 O43505: Beta-1,4-glucuronyltransferase 1		4	5	127	126	5	**0.63**
					129	128	3	**1.34**
					131	130	3	**0.84**
166	BAX Q07812$_{1,2,5,8}$: Apoptosis regulator BAX	4/6	2	3	127	126	3	**1.56**
					129	128	2	**0.41**
					131	130	3	**0.45**
167	BCAP31 P51572$_{2}$: B-cell receptor-associated protein 31	1/2	5	7	127	126	6	**2.28**
					129	128	5	**0.79**
					131	130	6	**0.57**
168	BCHE P06276: Cholinesterase		6	10	127	126	6	**0.60**
					129	128	4	**2.37**
					131	130	10	**1.10**
169	BCL2L2 Q92843$_{2}$, PABPN1 Q86U42$_{1,2}$: Bcl-2-like protein 2, Polyadenylate-binding protein 2	3/3	3	3	127	126	3	**0.87**
					129	128	2	**2.60**
					131	130	3	**1.82**
170	BDH2 Q9BUT1$_{1}$: 3-hydroxybutyrate dehydrogenase type 2	1/3	4	6	127	126	6	**1.34**
					129	128	3	**0.56**
					131	130	6	**0.30**
171	BGN P21810: Biglycan		6	8	127	126	4	**0.95**
					129	128	5	**0.31**
					131	130	4	**0.80**
172	BLVRA P53004: Biliverdin reductase A		4	6	127	126	6	**0.88**
					129	128	5	**2.68**
					131	130	6	**0.15**

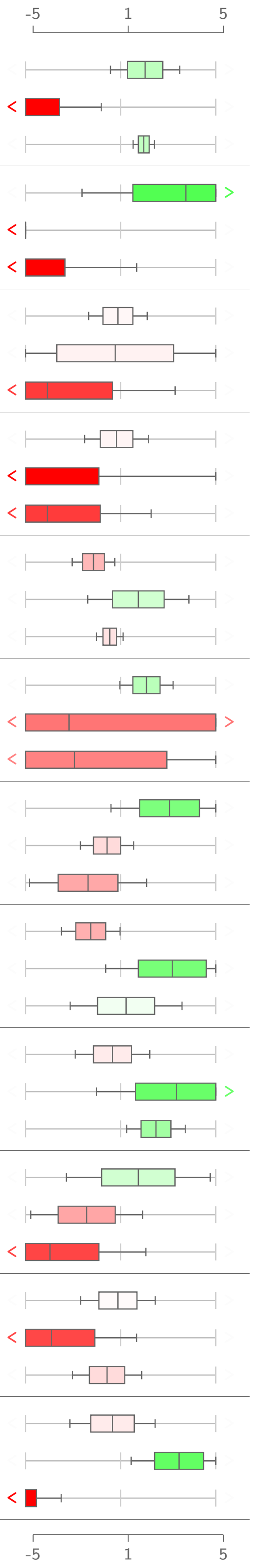

#	protein	group	peptides	spectra	ch1	ch2	quant	ratio
**173**	BLVRB P30043: Flavin reductase		5	5	127	126	4	**1.07**
					129	128	4	**0.66**
					131	130	5	**0.67**
**174**	BMP1 P13497$_1$: Bone morphogenetic protein 1	1/7	12	13	127	126	12	**1.07**
					129	128	11	**1.36**
					131	130	12	**3.28**
**175**	BMP3 P12645: Bone morphogenetic protein 3		3	3	127	126	3	**0.69**
					129	128	2	**0.34**
					131	130	3	**0.54**
**176**	BPIFA1 Q9NP55$_{1,2}$: BPI fold-containing family A member 1	2/2	3	3	127	126	2	**0.77**
					129	128	1	**1.18**
					131	130	2	**0.52**
**177**	BPIFB1 Q8TDL5$_1$: BPI fold-containing family B member 1	1/2	8	10	127	126	9	**2.12**
					129	128	5	**1.13**
					131	130	5	**0.19**
**178**	BSG P35613$_{1,2}$: Basigin	2/2	3	3	127	126	3	**2.23**
					129	128	3	**1.10**
					131	130	3	**0.71**
**179**	BTD P43251$_{1-4}$: Biotinidase	4/4	3	3	127	126	3	**0.57**
					129	128	2	**0.62**
					131	130	3	**1.95**
**180**	BTF3 P20290$_{1,2}$, BTF3L4 Q96K17$_{1,3}$: Transcription factor BTF3, Transcription factor BTF3 homolog 4	4/4	3	3	127	126	3	**1.40**
					129	128	3	**0.77**
					131	130	3	**1.30**
**181**	BZW1 Q7L1Q6$_{1-4}$: Basic leucine zipper and W2 domain-containing protein 1	4/4	7	8	127	126	8	**1.09**
					129	128	7	**0.46**
					131	130	8	**0.51**
**182**	C1QA P02745: Complement C1q subcomponent subunit A		2	17	127	126	17	**0.24**
					129	128	16	**2.05**
					131	130	14	**3.33**
**183**	C1QB P02746: Complement C1q subcomponent subunit B		8	47	127	126	41	**0.33**
					129	128	37	**1.26**
					131	130	46	**2.00**
**184**	C1QC P02747: Complement C1q subcomponent subunit C		5	17	127	126	17	**0.58**
					129	128	16	**1.63**
					131	130	16	**2.62**

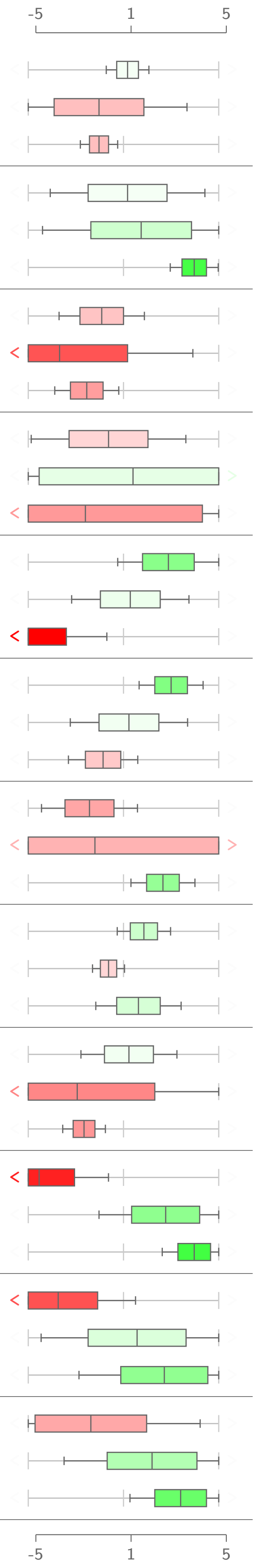

#	protein	group	peptides	spectra	ch1	ch2	quant	ratio
					127	126	1	**0.20**
**185**	C1QTNF5 Q9BXJ0: Complement C1q tumor necrosis factor-related protein 5		1	1	129	128	1	**2.10**
					131	130	1	**1.91**
					127	126	87	**0.43**
**186**	C1R P00736: Complement C1r subcomponent		31	105	129	128	73	**1.20**
					131	130	95	**2.14**
					127	126	21	**0.84**
**187**	C1RL Q9NZP8: Complement C1r subcomponent-like protein		13	29	129	128	15	**0.49**
					131	130	22	**0.30**
					127	126	78	**0.29**
**188**	C1S P09871: Complement C1s subcomponent		25	92	129	128	65	**1.75**
					131	130	84	**3.07**
					127	126	37	**0.62**
**189**	C2 $P06681_1$: Complement C2	1/3	28	40	129	128	29	**0.91**
					131	130	40	**0.41**
					127	126	3	**0.48**
**190**	C2orf40 Q9H1Z8: Augurin		2	3	129	128	3	**0.22**
					131	130	3	**2.50**
					127	126	2208	**0.58**
**191**	C3 P01024: Complement C3		118	2688	129	128	1707	**1.94**
					131	130	2509	**0.39**
					127	126	9	**0.27**
**192**	C4A $P0C0L4_1$: Complement C4-A	1/2	81	1611	129	128	7	**0.54**
					131	130	10	**0.95**
					127	126	99	**0.35**
**193**	C4B P0C0L5: Complement C4-B		83	1707	129	128	80	**1.84**
					131	130	99	**1.05**
					127	126	670	**1.03**
**194**	C4BPA P04003: C4b-binding protein alpha chain		31	759	129	128	644	**0.26**
					131	130	750	**3.18**
					127	126	49	**1.19**
**195**	C4BPB $P20851_{1,2}$: C4b-binding protein beta chain	2/2	12	69	129	128	42	**0.17**
					131	130	60	**2.90**
					127	126	217	**0.65**
**196**	C5 P01031: Complement C5		74	263	129	128	167	**1.30**
					131	130	233	**0.61**

#	protein	group	peptides	spectra	ch1	ch2	quant	ratio
**197**	C6 P13671: Complement component C6		44	102	127	126	74	**0.69**
					129	128	51	**1.60**
					131	130	84	**2.28**
**198**	C7 P10643: Complement component C7		41	118	127	126	98	**0.59**
					129	128	70	**0.84**
					131	130	103	**1.23**
**199**	C8A P07357: Complement component C8 alpha chain		20	76	127	126	65	**0.57**
					129	128	54	**1.35**
					131	130	64	**0.96**
**200**	C8B P07358: Complement component C8 beta chain		28	147	127	126	113	**0.57**
					129	128	64	**1.14**
					131	130	141	**0.32**
**201**	C8G P07360: Complement component C8 gamma chain		11	40	127	126	39	**0.77**
					129	128	26	**1.05**
					131	130	39	**0.36**
**202**	C9 P02748: Complement component C9 [Cleaved into: Complement component C9a; Complement . . .	1/4	31	1004	127	126	814	**0.81**
					129	128	393	**0.45**
					131	130	845	**0.20**
**203**	CACNA1D Q01668$_{1-4}$: Voltage-dependent L-type calcium channel subunit alpha-1D	4/4	5	111	127	126	35	**2.30**
					129	128	22	**0.21**
					131	130	63	**1.95**
**204**	CACYBP Q9HB71$_{1,2}$: Calcyclin-binding protein	2/2	2	112	127	126	2	**1.14**
					129	128	2	**0.76**
					131	130	2	**0.34**
**205**	CAD P27708: CAD protein [Includes: Glutamine-dependent carbamoyl-phosphate synthase	1/4	6	6	127	126	4	**1.42**
					129	128	5	**0.90**
					131	130	6	**0.69**
**206**	CALM1 P62158: Calmodulin	1/2	7	24	127	126	21	**0.34**
					129	128	22	**1.90**
					131	130	22	**2.16**
**207**	CALR P27797: Calreticulin		18	53	127	126	42	**0.40**
					129	128	32	**1.41**
					131	130	50	**2.92**
**209**	CAMK2D Q13557$_{4,5}$: Calcium/calmodulin-dependent protein kinase type II subunit delta	2/2	8	11	127	126		
					129	128		
					131	130	1	**1.41**

#	protein	group	peptides	spectra	ch1	ch2	quant	ratio
**210**	CAMP P49913: Cathelicidin antimicrobial peptide		4	4	127	126	4	**2.52**
					129	128	4	**0.58**
					131	130	4	**1.00**
**211**	CAND1 Q86VP6$_1$: Cullin-associated NEDD8-dissociated protein 1	1/3	34	52	127	126	47	**1.21**
					129	128	40	**0.96**
					131	130	47	**0.34**
**212**	CANX P27824$_{1,2}$: Calnexin	2/3	13	19	127	126	17	**0.83**
					129	128	16	**1.39**
					131	130	18	**1.07**
**213**	CAP1 Q01518$_{1,2}$: Adenylyl cyclase-associated protein 1	2/2	14	18	127	126	17	**1.79**
					129	128	13	**1.25**
					131	130	17	**0.28**
**214**	CAP2 P40123$_1$: Adenylyl cyclase-associated protein 2	1/2	5	5	127	126	4	**1.63**
					129	128	4	**0.24**
					131	130	3	**0.22**
**215**	CAPG P40121$_{1,2}$: Macrophage-capping protein	2/2	6	10	127	126	7	**0.92**
					129	128	5	**1.23**
					131	130	7	**0.21**
**216**	CAPN1 P07384: Calpain-1 catalytic subunit		11	14	127	126	12	**1.41**
					129	128	10	**0.61**
					131	130	10	**0.30**
**217**	CAPN2 P17655$_1$: Calpain-2 catalytic subunit	1/2	13	18	127	126	15	**1.14**
					129	128	13	**0.87**
					131	130	14	**0.72**
**218**	CAPNS1 P04632: Calpain small subunit 1		5	8	127	126	7	**1.22**
					129	128	5	**0.84**
					131	130	8	**0.47**
**219**	CAPZA1 P52907: F-actin-capping protein subunit alpha-1		5	5	127	126	2	**2.68**
					129	128	3	**1.79**
					131	130	4	**0.65**
**220**	CAPZA2 P47755$_1$: F-actin-capping protein subunit alpha-2	1/2	3	3	127	126	2	**0.86**
					129	128	2	**0.92**
					131	130	2	**1.10**
**221**	CAPZB P47756$_{1,2}$: F-actin-capping protein subunit beta	2/2	6	8	127	126	8	**1.22**
					129	128	6	**1.53**
					131	130	8	**0.82**

#	protein	group	peptides	spectra	ch1	ch2	quant	ratio
**222**	CARNS1 A5YM72$_{3-5}$: Carnosine synthase 1	3/3	3	3	127	126	2	**0.55**
					129	128	2	**2.57**
					131	130	2	**0.94**
**223**	CAST P20810$_{5-7,9,10}$: Calpastatin	5/10	7	8	127	126	7	**1.62**
					129	128	7	**0.61**
					131	130	8	**0.56**
**224**	CAV1 Q03135$_{1}$: Caveolin-1	1/2	4	10	127	126	9	**3.77**
					129	128	10	**0.14**
					131	130	9	**0.33**
**225**	CAV2 P51636$_{1}$: Caveolin-2	1/3	3	3	127	126	3	**2.83**
					129	128	3	**0.20**
					131	130	3	**0.57**
**226**	CBR1 P16152$_{1}$: Carbonyl reductase [NADPH] 1	1/3	13	18	127	126	17	**0.90**
					129	128	13	**0.55**
					131	130	17	**0.26**
**227**	CBX3 Q13185: Chromobox protein homolog 3		1	1	127	126	1	**0.81**
					129	128	1	**2.60**
					131	130	1	**1.76**
**228**	CCAR1 Q8IX12$_{1,2}$: Cell division cycle and apoptosis regulator protein 1	2/2	4	4	127	126	2	**1.59**
					129	128	2	**0.21**
					131	130	3	**0.77**
**229**	CCAR2 Q8N163$_{1,2}$: Cell cycle and apoptosis regulator protein 2	2/2	9	9	127	126	9	**1.56**
					129	128	6	**0.72**
					131	130	7	**0.65**
**230**	CCDC102B Q68D86$_{1,2}$: Coiled-coil domain-containing protein 102B	2/2	2	2	127	126	1	**0.95**
					129	128	1	**0.64**
					131	130	1	**29.67**
**231**	CCDC80 Q76M96$_{1,2}$: Coiled-coil domain-containing protein 80	2/2	23	40	127	126	32	**0.70**
					129	128	28	**0.50**
					131	130	37	**0.90**
**232**	CCER2 I3L3R5: Coiled-coil domain-containing glutamate-rich protein 2		2	2	127	126	1	**0.66**
					129	128	1	**0.38**
					131	130	1	**1.73**
**233**	CCL14 Q16627$_{1,2}$: C-C motif chemokine 14	2/2	3	3	127	126	3	**0.55**
					129	128	3	**0.15**
					131	130	3	**0.62**

#	protein	group	peptides	spectra	ch1	ch2	quant	ratio
**234**	CCL18 P55774: C-C motif chemokine 18		2	4	127	126	3	**0.92**
					129	128	4	**0.24**
					131	130	4	**0.45**
**235**	CCT2 P78371$_{1}$: T-complex protein 1 subunit beta	1/2	12	15	127	126	12	**1.05**
					129	128	11	**1.75**
					131	130	15	**0.63**
**236**	CCT3 P49368$_{1}$: T-complex protein 1 subunit gamma	1/2	15	19	127	126	16	**1.11**
					129	128	14	**1.32**
					131	130	19	**0.44**
**237**	CCT4 P50991$_{1,2}$: T-complex protein 1 subunit delta	2/2	9	9	127	126	9	**1.30**
					129	128	8	**0.62**
					131	130	9	**0.44**
**238**	CCT5 P48643$_{1}$: T-complex protein 1 subunit epsilon	1/2	12	17	127	126	16	**1.68**
					129	128	12	**1.34**
					131	130	15	**0.58**
**239**	CCT6A P40227$_{1,2}$: T-complex protein 1 subunit zeta	2/5	14	15	127	126	15	**1.03**
					129	128	10	**1.74**
					131	130	15	**0.51**
**240**	CCT7 Q99832$_{1}$: T-complex protein 1 subunit eta	1/3	12	14	127	126	1	**0.67**
					129	128	1	**14.74**
					131	130	1	**1.33**
**241**	CCT7 Q99832$_{3}$: T-complex protein 1 subunit eta		12	14	127	126	1	**1.08**
					129	128	1	**0.14**
					131	130	1	**4.23**
**242**	CCT8 P50990$_{1,2}$: T-complex protein 1 subunit theta	2/3	11	12	127	126	7	**0.99**
					129	128	8	**0.45**
					131	130	10	**0.54**
**243**	CD109 Q6YHK3$_{1,2}$: CD109 antigen	2/4	7	8	127	126	8	**0.49**
					129	128	7	**1.54**
					131	130	7	**0.72**
**244**	CD14 P08571: Monocyte differentiation antigen CD14		17	121	127	126	109	**1.09**
					129	128	77	**0.66**
					131	130	107	**0.51**
**245**	CD151 P48509: CD151 antigen		2	2	127	126	2	**1.25**
					129	128	2	**0.19**
					131	130	2	**0.43**

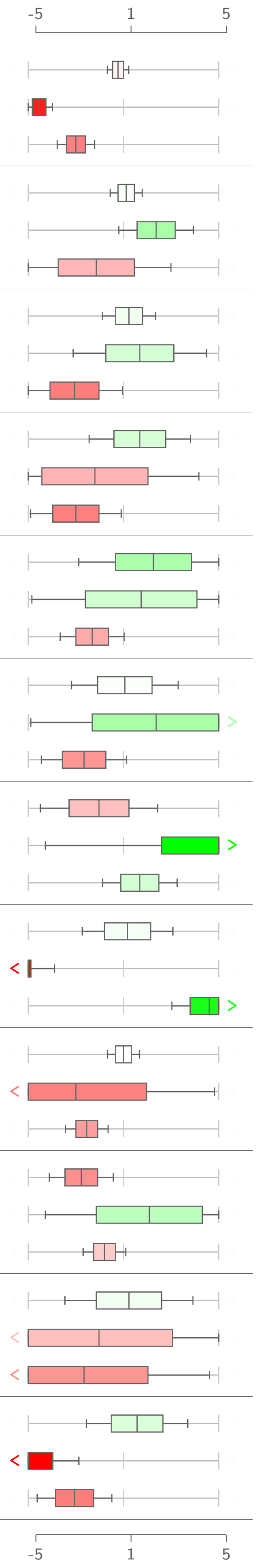

#	protein	group	peptides	spectra	ch1	ch2	quant	ratio
**246**	CD163 Q86VB7$_{1-4}$: Scavenger receptor cysteine-rich type 1 protein M130	4/4	10	12	127	126	11	**0.86**
					129	128	10	**1.05**
					131	130	12	**1.25**
**247**	CD248 Q9HCU0$_1$: Endosialin		7	8	127	126	6	**0.50**
					129	128	6	**2.11**
					131	130	8	**2.91**
**248**	CD5L O43866: CD5 antigen-like		19	47	127	126	41	**0.50**
					129	128	37	**1.89**
					131	130	44	**2.58**
**249**	CD81 P60033: CD81 antigen		3	4	127	126	4	**2.70**
					129	128	3	**0.15**
					131	130	3	**0.12**
**250**	CD8A P01732$_{1-3}$: T-cell surface glycoprotein CD8 alpha chain	3/3	1	1	127	126	1	**1.05**
					129	128	1	**0.51**
					131	130	1	**0.58**
**251**	CD9 P21926: CD9 antigen		3	4	127	126	3	**1.59**
					129	128	3	**0.21**
					131	130	3	**0.35**
**252**	CD99 P14209$_{1,3}$: CD99 antigen	2/2	1	1	127	126	1	**0.61**
					129	128	1	**0.79**
					131	130	1	**1.01**
**253**	CDC37 Q16543: Hsp90 co-chaperone Cdc37		3	3	127	126	3	**1.43**
					129	128	3	**0.55**
					131	130	3	**1.34**
**254**	CDC42 P60953$_2$: Cell division control protein 42 homolog	1/2	5	12	127	126	10	**1.17**
					129	128	10	**0.70**
					131	130	10	**0.38**
**255**	CDH1 P12830$_{1,2}$: Cadherin-1	2/2	3	8	127	126	7	**0.47**
					129	128	6	**0.58**
					131	130	8	**1.94**
**256**	CDH2 P19022$_{1,2}$: Cadherin-2	2/2	4	4	127	126	3	**1.17**
					129	128	3	**0.31**
					131	130	4	**1.05**
**257**	CDK5RAP3 Q96JB5$_{1,4}$: CDK5 regulatory subunit-associated protein 3	2/2	2	2	127	126	2	**0.58**
					129	128	2	**1.77**
					131	130	2	**0.57**

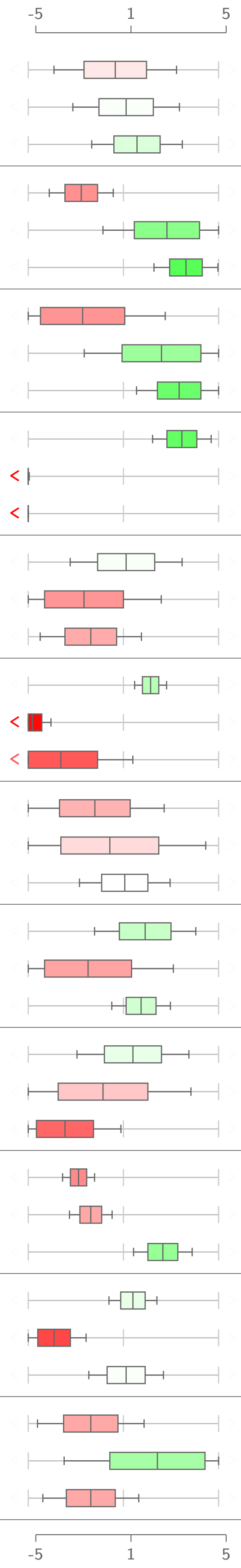

#	protein	group	peptides	spectra	ch1	ch2	quant	ratio
**258**	CELF2 O95319$_{1-5}$: CUGBP Elav-like family member 2	5/11	6	8	127	126	7	**1.27**
					129	128	4	**1.25**
					131	130	7	**0.55**
**260**	CEP350 Q5VT06: Centrosome-associated protein 350		5	33	127	126	4	**0.85**
					129	128	4	**0.76**
					131	130	4	**2.46**
**261**	CETP P11597$_1$: Cholesteryl ester transfer protein	1/2	15	50	127	126	47	**1.56**
					129	128	37	**0.66**
					131	130	46	**0.49**
**262**	CFB P00751$_1$: Complement factor B	1/2	24	36	127	126	32	**0.57**
					129	128	29	**1.87**
					131	130	36	**0.59**
**263**	CFD P00746: Complement factor D		16	97	127	126	80	**0.84**
					129	128	63	**0.59**
					131	130	94	**0.62**
**264**	CFH P08603$_1$: Complement factor H	1/2	65	673	127	126	428	**0.28**
					129	128	379	**1.62**
					131	130	509	**2.45**
**265**	CFHR1 Q03591: Complement factor H-related protein 1		12	196	127	126	15	**0.23**
					129	128	10	**3.43**
					131	130	15	**4.37**
**266**	CFHR2 P36980$_1$: Complement factor H-related protein 2	1/2	10	56	127	126	7	**0.59**
					129	128	8	**0.40**
					131	130	9	**1.73**
**267**	CFHR3 Q02985$_{1,2}$: Complement factor H-related protein 3	2/2	9	66	127	126	12	**0.28**
					129	128	7	**1.18**
					131	130	11	**2.09**
**268**	CFHR4 Q92496$_{1,2}$: Complement factor H-related protein 4	2/3	9	36	127	126	6	**0.36**
					129	128	8	**1.85**
					131	130	8	**2.85**
**269**	CFHR5 Q9BXR6: Complement factor H-related protein 5		23	75	127	126	43	**0.44**
					129	128	34	**2.36**
					131	130	43	**2.79**
**270**	CFI P05156: Complement factor I		18	40	127	126	29	**0.69**
					129	128	15	**0.93**
					131	130	31	**1.58**

#	protein	group	peptides	spectra	ch1	ch2	quant	ratio
271	CFL1 P23528: Cofilin-1	1/3	9	10	127	126	9	**1.15**
					129	128	5	**0.68**
					131	130	9	**1.29**
272	CFP P27918: Properdin		15	27	127	126	21	**0.59**
					129	128	18	**1.56**
					131	130	22	**3.29**
273	CGREF1 Q99674$_{1,4,5}$: Cell growth regulator with EF hand domain protein 1	3/3	5	8	127	126	7	**0.64**
					129	128	8	**0.63**
					131	130	8	**2.80**
274	CHCHD3 Q9NX63: MICOS complex subunit MIC19		4	4	127	126	4	**3.08**
					129	128	4	**0.70**
					131	130	4	**0.57**
275	CHD3 Q12873$_{1-3}$: Chromodomain-helicase-DNA-binding protein 3	3/3	5	7	127	126	1	**0.73**
					129	128	1	**0.15**
					131	130	5	**1.60**
276	CHD4 Q14839$_{1,2}$: Chromodomain-helicase-DNA-binding protein 4	2/3	9	16	127	126	3	**1.60**
					129	128	2	**0.29**
					131	130	8	**1.48**
277	CHGA P10645: Chromogranin-A		27	276	127	126	224	**0.35**
					129	128	167	**0.21**
					131	130	268	**3.50**
278	CHGB P05060: Secretogranin-1		29	79	127	126	52	**0.29**
					129	128	39	**0.47**
					131	130	70	**2.29**
279	CHMP4A Q9BY43$_{1,2}$: Charged multivesicular body protein 4a	2/2	2	16	127	126	1	**0.53**
					129	128	1	**0.42**
					131	130	1	**1.92**
280	CHMP5 Q9NZZ3$_{1,2}$: Charged multivesicular body protein 5	2/2	2	3	127	126	3	**1.59**
					129	128	3	**0.35**
					131	130	3	**1.09**
281	CHRD Q9H2X0$_{1,5}$: Chordin	2/5	6	6	127	126	6	**0.64**
					129	128	6	**0.29**
					131	130	6	**1.03**
282	CHRDL1 Q9BU40$_{2,4}$: Chordin-like protein 1	2/6	17	33	127	126	30	**0.54**
					129	128	28	**0.96**
					131	130	29	**0.91**

-5 1 5

#	protein	group	peptides	spectra	ch1	ch2	quant	ratio
**283**	CHRDL2 Q6WN34$_1$: Chordin-like protein 2	1/2	4	4	127	126	4	**0.46**
					129	128	3	**1.12**
					131	130	4	**1.04**
**284**	CHST14 Q8NCH0: Carbohydrate sulfotransferase 14		4	6	127	126	5	**1.52**
					129	128	5	**0.40**
					131	130	6	**0.37**
**285**	CHST15 Q7LFX5$_1$: Carbohydrate sulfotransferase 15		3	3	127	126	3	**1.42**
					129	128	3	**0.40**
					131	130	3	**0.27**
**286**	CILP O75339: Cartilage intermediate layer protein 1		43	71	127	126	60	**0.37**
					129	128	57	**0.86**
					131	130	70	**2.06**
**287**	CIRBP Q14011$_{1,3}$: Cold-inducible RNA-binding protein	2/2	2	2	127	126	2	**0.95**
					129	128	2	**0.61**
					131	130	2	**1.28**
**288**	CKAP4 Q07065: Cytoskeleton-associated protein 4		15	17	127	126	14	**1.72**
					129	128	15	**0.57**
					131	130	15	**0.57**
**289**	CKB P12277: Creatine kinase B-type		4	4	127	126	4	**0.51**
					129	128	3	**0.90**
					131	130	4	**1.82**
**290**	CLC Q05315: Galectin-10		4	4	127	126	4	**1.42**
					129	128	4	**2.30**
					131	130	4	**0.83**
**291**	CLDN1 O95832: Claudin-1		1	1	127	126	1	**2.17**
					129	128	1	**0.41**
					131	130	1	**0.22**
**292**	CLEC11A Q9Y240: C-type lectin domain family 11 member A		13	22	127	126	20	**0.45**
					129	128	19	**1.64**
					131	130	20	**2.39**
**293**	CLEC3B P05452: Tetranectin		5	8	127	126	8	**3.56**
					129	128	5	**0.32**
					131	130	8	**1.74**
**294**	CLIC1 O00299: Chloride intracellular channel protein 1		8	13	127	126	10	**0.71**
					129	128	11	**0.82**
					131	130	10	**1.20**

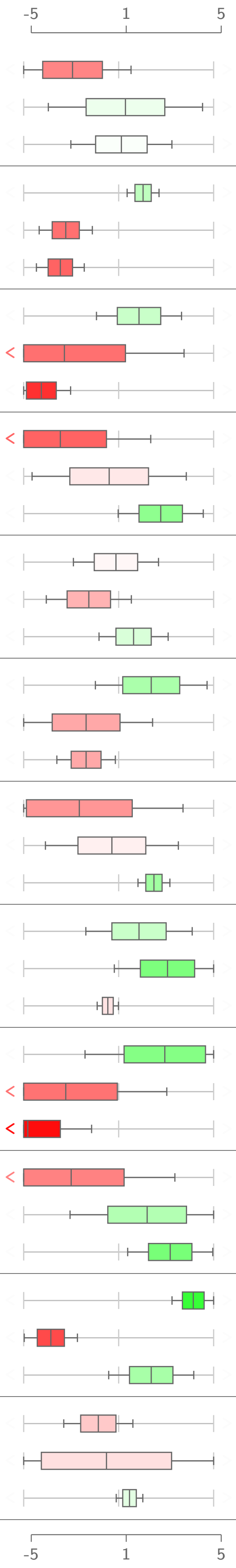

#	protein	group	peptides	spectra	ch1	ch2	quant	ratio
295	CLIC3 O95833: Chloride intracellular channel protein 3		4	4	127	126	4	**3.95**
					129	128	4	**1.01**
					131	130	4	**0.30**
296	CLIC5 Q9NZA1$_{1-3}$: Chloride intracellular channel protein 5	3/3	2	2	127	126	1	**0.39**
					129	128	1	**3.82**
					131	130	1	**0.20**
297	CLSTN1 O94985$_1$: Calsyntenin-1	1/2	9	10	127	126	7	**0.33**
					129	128	9	**1.41**
					131	130	8	**1.24**
298	CLTC Q00610$_{1,2}$: Clathrin heavy chain 1	2/4	47	74	127	126	68	**1.12**
					129	128	54	**0.61**
					131	130	67	**0.45**
299	CLU P10909$_4$: Clusterin	1/5	26	630	127	126	570	**0.23**
					129	128	475	**1.85**
					131	130	597	**1.76**
300	CMAS Q8NFW8$_1$: N-acylneuraminate cytidylyltransferase	1/2	2	2	127	126	2	**1.43**
					129	128	2	**0.41**
					131	130	2	**0.35**
301	CNDP1 Q96KN2: Beta-Ala-His dipeptidase		10	11	127	126	11	**0.44**
					129	128	9	**2.43**
					131	130	11	**1.68**
302	CNN2 Q99439$_1$: Calponin-2		2	2	127	126	1	**0.70**
					129	128	1	**0.44**
					131	130	1	**0.66**
303	CNN3 Q15417$_{1,3}$: Calponin-3	2/3	7	8	127	126	7	**1.09**
					129	128	3	**0.70**
					131	130	6	**0.37**
304	CNOT1 A5YKK6$_{1-3}$: CCR4-NOT transcription complex subunit 1	3/3	5	6	127	126	3	**1.89**
					129	128	5	**0.74**
					131	130	5	**1.77**
305	CNPY2 Q9Y2B0$_1$: Protein canopy homolog 2		2	2	127	126	2	**0.50**
					129	128	2	**0.36**
					131	130	2	**2.09**
306	COASY Q13057$_{1,2}$: Bifunctional coenzyme A synthase	2/2	3	3	127	126	2	**4.45**
					129	128	2	**0.41**
					131	130	2	**0.57**

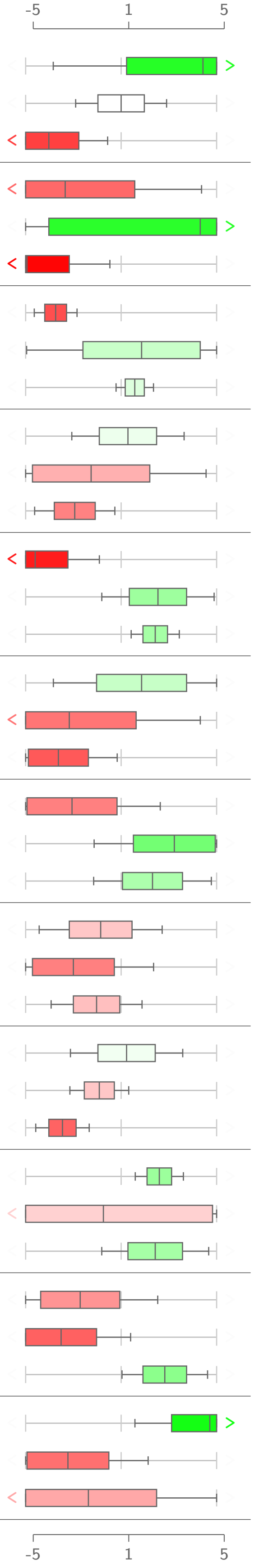

#	protein	group	peptides	spectra	ch1	ch2	quant	ratio
**307**	COL12A1 Q99715$_{1,4}$: Collagen alpha-1(XII) chain	2/3	38	44	127	126	39	**0.73**
					129	128	38	**0.57**
					131	130	40	**0.56**
**308**	COL14A1 Q05707$_{1,2}$: Collagen alpha-1(XIV) chain	2/3	34	49	127	126	45	**0.36**
					129	128	43	**1.19**
					131	130	45	**0.87**
**309**	COL15A1 P39059: Collagen alpha-1(XV) chain [Cleaved into: Restin		3	4	127	126	4	**1.63**
					129	128	4	**0.32**
					131	130	4	**0.50**
**310**	COL16A1 Q07092$_{1,2}$: Collagen alpha-1(XVI) chain	2/2	1	2	127	126	2	**0.76**
					129	128	2	**2.07**
					131	130	2	**0.34**
**311**	COL18A1 P39060$_{1-3}$: Collagen alpha-1(XVIII) chain [Cleaved into: Endostatin]	3/3	11	18	127	126	14	**0.43**
					129	128	17	**0.55**
					131	130	16	**1.01**
**312**	COL1A1 P02452: Collagen alpha-1(I) chain	1/2	13	49	127	126	30	**0.62**
					129	128	47	**1.12**
					131	130	46	**2.71**
**313**	COL1A2 P08123: Collagen alpha-2(I) chain		14	18	127	126	15	**0.44**
					129	128	16	**0.45**
					131	130	17	**2.16**
**314**	COL3A1 P02461$_{1}$: Collagen alpha-1(III) chain	1/2	11	16	127	126	14	**0.79**
					129	128	14	**0.88**
					131	130	13	**0.87**
**315**	COL4A3BP Q9Y5P4$_{1-3}$: Collagen type IV alpha-3-binding protein	3/3	2	2	127	126	1	**0.58**
					129	128	1	**7.22**
					131	130	2	**0.91**
**316**	COL5A1 P20908: Collagen alpha-1(V) chain		10	12	127	126	12	**0.58**
					129	128	12	**1.76**
					131	130	12	**1.68**
**317**	COL5A2 P05997: Collagen alpha-2(V) chain		10	13	127	126	11	**0.67**
					129	128	7	**3.27**
					131	130	12	**2.22**
**318**	COL6A1 P12109: Collagen alpha-1(VI) chain		23	44	127	126	39	**0.86**
					129	128	30	**0.52**
					131	130	39	**0.29**

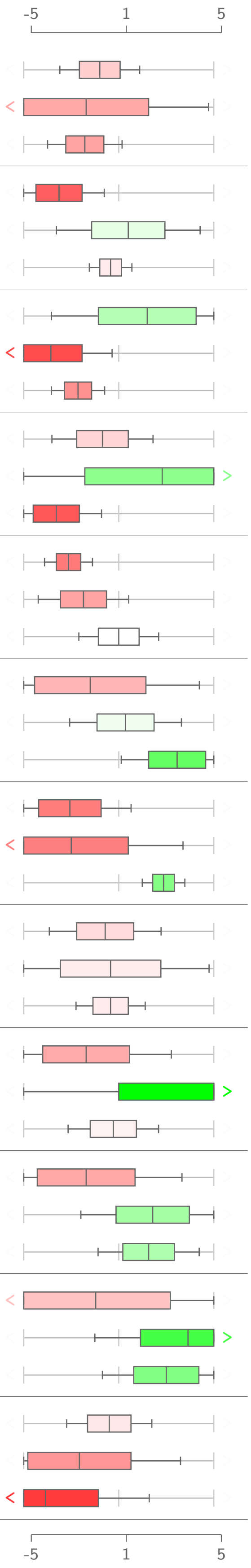

#	protein	group	peptides	spectra	ch1	ch2	quant	ratio
**319**	COL6A2 P12110$_1$: Collagen alpha-2(VI) chain	1/3	18	150	127	126	56	**0.86**
					129	128	31	**0.53**
					131	130	111	**1.42**
**320**	COL6A3 P12111$_1$: Collagen alpha-3(VI) chain	1/4	103	244	127	126	14	**0.98**
					129	128	8	**2.30**
					131	130	15	**0.33**
**321**	COL6A3 P12111$_4$: Collagen alpha-3(VI) chain		92	237	127	126	8	**1.08**
					129	128	1	**1.76**
					131	130	6	**0.06**
**322**	COL7A1 Q02388$_{1,2}$: Collagen alpha-1(VII) chain	2/2	3	3	127	126	2	**1.08**
					129	128	2	**1.42**
					131	130	2	**1.20**
**323**	COLEC10 Q9Y6Z7: Collectin-10		3	3	127	126	3	**0.74**
					129	128	3	**0.45**
					131	130	3	**1.85**
**324**	COLEC11 Q9BWP8$_{1-10}$: Collectin-11	10/10	2	2	127	126	2	**0.52**
					129	128	1	**2.59**
					131	130	2	**2.86**
**325**	COMP P49747$_{1,2}$: Cartilage oligomeric matrix protein	2/2	18	26	127	126	20	**1.86**
					129	128	14	**2.57**
					131	130	19	**1.60**
**326**	COPA P53621$_1$: Coatomer subunit alpha	1/2	18	21	127	126	18	**1.12**
					129	128	17	**1.11**
					131	130	20	**0.69**
**327**	COPB1 P53618: Coatomer subunit beta		22	32	127	126	27	**1.17**
					129	128	21	**0.41**
					131	130	29	**0.42**
**328**	COPB2 P35606$_{1,2}$: Coatomer subunit beta’	2/2	13	15	127	126	14	**1.18**
					129	128	11	**2.81**
					131	130	13	**1.08**
**329**	COPE O14579$_{1,3}$: Coatomer subunit epsilon	2/2	2	2	127	126	2	**1.51**
					129	128	1	**7.52**
					131	130	2	**0.88**
**330**	COPG1 Q9Y678: Coatomer subunit gamma-1		13	16	127	126	9	**1.01**
					129	128	12	**1.30**
					131	130	14	**0.55**

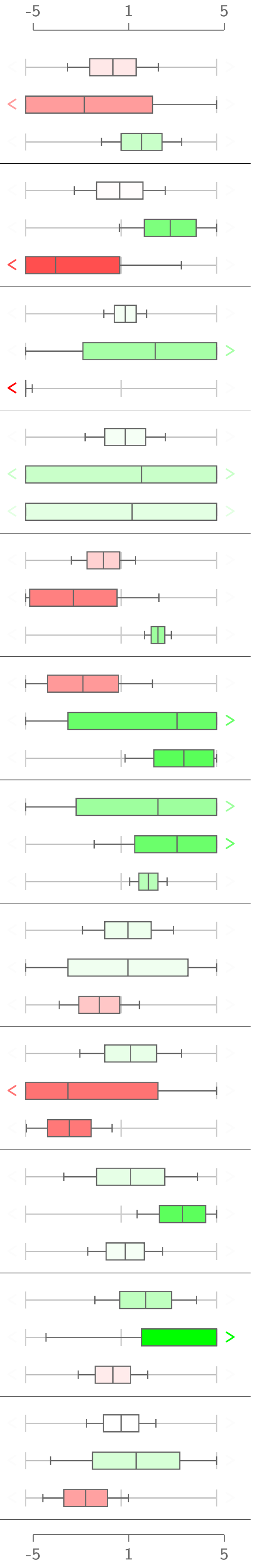

#	protein	group	peptides	spectra	ch1	ch2	quant	ratio
**331**	COPG2 Q9UBF2$_1$: Coatomer subunit gamma-2	1/2	5	5	127	126	3	**1.49**
					129	128	3	**0.84**
					131	130	3	**0.55**
**332**	COPS2 P61201$_{1,2}$: COP9 signalosome complex subunit 2	2/2	2	2	127	126	2	**2.56**
					129	128		
					131	130	2	**0.33**
**333**	CORO1A P31146: Coronin-1A	1/6	6	7	127	126	4	**0.68**
					129	128	5	**2.70**
					131	130	6	**0.77**
**334**	CORO1B Q9BR76: Coronin-1B		2	2	127	126		
					129	128		
					131	130	1	**0.21**
**335**	CORO1C Q9ULV4$_{1-3}$: Coronin-1C	3/3	11	13	127	126	9	**0.88**
					129	128	8	**1.42**
					131	130	10	**0.31**
**336**	CP P00450: Ceruloplasmin		37	88	127	126	78	**0.41**
					129	128	69	**2.31**
					131	130	85	**2.25**
**337**	CPB2 Q96IY4$_1$: Carboxypeptidase B2	1/2	9	18	127	126	18	**0.71**
					129	128	18	**0.74**
					131	130	18	**0.69**
**338**	CPE P16870$_{1,2}$: Carboxypeptidase E	2/2	23	82	127	126	75	**0.56**
					129	128	67	**1.68**
					131	130	74	**0.61**
**339**	CPN1 P15169: Carboxypeptidase N catalytic chain		12	22	127	126	20	**0.49**
					129	128	15	**1.50**
					131	130	21	**2.19**
**340**	CPN2 P22792: Carboxypeptidase N subunit 2		12	48	127	126	34	**0.62**
					129	128	29	**1.47**
					131	130	44	**0.52**
**341**	CPNE1 Q99829: Copine-1		4	4	127	126	4	**1.77**
					129	128	3	**0.36**
					131	130	4	**0.92**
**342**	CPQ Q9Y646: Carboxypeptidase Q		3	3	127	126	3	**0.84**
					129	128	3	**0.56**
					131	130	3	**0.74**

#	protein	group	peptides	spectra	ch1	ch2	quant	ratio
**343**	CPSF1 Q10570: Cleavage and polyadenylation specificity factor subunit 1		3	4	127	126	1	**1.24**
					129	128	1	**2.64**
					131	130	1	**0.43**
**344**	CPXM1 Q96SM3: Probable carboxypeptidase X1		4	6	127	126	6	**0.57**
					129	128	6	**1.08**
					131	130	5	**1.55**
**345**	CPXM2 Q8N436: Inactive carboxypeptidase-like protein X2		8	9	127	126	8	**0.55**
					129	128	6	**1.02**
					131	130	8	**1.79**
**346**	CPZ $Q66K79_{1,2}$: Carboxypeptidase Z	2/3	6	7	127	126	5	**0.98**
					129	128	4	**0.84**
					131	130	6	**1.50**
**347**	CRABP2 P29373: Cellular retinoic acid-binding protein 2	1/2	6	6	127	126	5	**1.14**
					129	128	6	**0.21**
					131	130	6	**0.41**
**348**	CRHBP P24387: Corticotropin-releasing factor-binding protein		3	5	127	126	3	**0.71**
					129	128	4	**0.37**
					131	130	3	**0.97**
**349**	CRIP2 $P52943_{1}$: Cysteine-rich protein 2		2	2	127	126	2	**1.14**
					129	128	2	**0.25**
					131	130	2	**0.95**
**350**	CRP $P02741_{1}$: C-reactive protein [Cleaved into: C-reactive protein(1-205)]	1/2	8	37	127	126	34	**0.15**
					129	128	28	**0.31**
					131	130	33	**1.32**
**351**	CRTAC1 $Q9NQ79_{1-3}$: Cartilage acidic protein 1	3/3	3	4	127	126	4	**0.92**
					129	128	4	**1.00**
					131	130	4	**1.17**
**352**	CRYAB P02511: Alpha-crystallin B chain		4	4	127	126	4	**1.36**
					129	128	3	**1.00**
					131	130	4	**1.19**
**353**	CRYL1 $Q9Y2S2_{1,2}$: Lambda-crystallin homolog	2/2	1	2	127	126	2	**1.52**
					129	128	2	**0.57**
					131	130	2	**1.21**
**354**	CSE1L $P55060_{1,3}$: Exportin-2	2/4	11	14	127	126	13	**1.33**
					129	128	12	**0.99**
					131	130	12	**0.44**

#	protein	group	peptides	spectra	ch1	ch2	quant	ratio
**355**	CSPG4 Q6UVK1: Chondroitin sulfate proteoglycan 4		3	3	127	126	2	**0.74**
					129	128	2	**2.92**
					131	130	3	**1.06**
**356**	CST3 P01034: Cystatin-C		6	31	127	126	29	**1.33**
					129	128	28	**0.39**
					131	130	31	**0.63**
**357**	CST6 Q15828: Cystatin-M		5	8	127	126	7	**1.07**
					129	128	7	**0.25**
					131	130	8	**0.64**
**358**	CTBP1 $Q13363_{1,2}$: C-terminal-binding protein 1	2/4	3	3	127	126	3	**1.13**
					129	128	3	**1.52**
					131	130	3	**0.73**
**359**	CTGF $P29279_{1,2}$: Connective tissue growth factor	2/2	6	8	127	126	5	**0.39**
					129	128	5	**0.28**
					131	130	5	**1.54**
**360**	CTNNA1 $P35221_{1,2}$: Catenin alpha-1	2/5	14	14	127	126	6	**1.61**
					129	128	4	**0.81**
					131	130	5	**0.59**
**361**	CTNNA2 $P26232_{1,2,5}$: Catenin alpha-2	3/4	10	10	127	126	2	**1.40**
					129	128	1	**0.31**
					131	130	1	**1.13**
**362**	CTNNB1 $P35222_{1}$: Catenin beta-1		10	15	127	126	9	**1.82**
					129	128	6	**0.90**
					131	130	12	**1.36**
**363**	CTSB P07858: Cathepsin B		3	10	127	126	10	**0.27**
					129	128	10	**0.51**
					131	130	10	**1.76**
**364**	CTSD P07339: Cathepsin D		6	8	127	126	8	**0.80**
					129	128	7	**0.77**
					131	130	8	**0.78**
**365**	CTSF Q9UBX1: Cathepsin F		6	6	127	126	5	**0.47**
					129	128	4	**0.99**
					131	130	6	**0.56**
**366**	CTTN $Q14247_{1,3}$: Src substrate cortactin	2/2	2	3	127	126	3	**0.48**
					129	128	3	**0.91**
					131	130	3	**1.31**

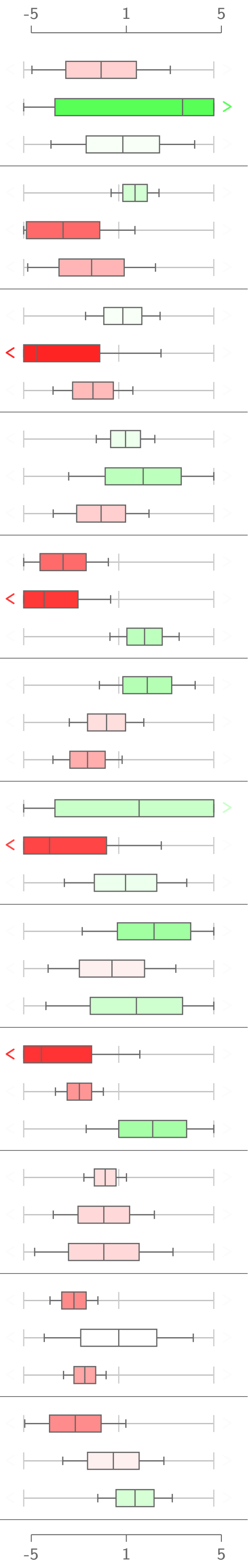

#	protein	group	peptides	spectra	ch1	ch2	quant	ratio
**367**	CUL1 Q13616: Cullin-1		2	2	127	126	2	**1.96**
					129	128	2	**0.58**
					131	130	2	**0.40**
**368**	CUL3 Q13618$_{1,2}$: Cullin-3	2/3	7	7	127	126	7	**1.35**
					129	128	7	**0.35**
					131	130	6	**0.30**
**369**	CUL4B Q13620$_{2}$: Cullin-4B	1/3	4	4	127	126	4	**1.09**
					129	128	3	**0.52**
					131	130	3	**1.49**
**370**	CUL5 Q93034: Cullin-5		3	4	127	126	4	**1.60**
					129	128	4	**0.61**
					131	130	4	**0.34**
**371**	CXCL10 P02778: C-X-C motif chemokine 10		2	3	127	126	3	**1.17**
					129	128	3	**0.33**
					131	130	3	**1.19**
**372**	CXCL12 P48061$_{1-6}$: Stromal cell-derived factor 1	6/6	4	10	127	126	9	**1.02**
					129	128	9	**0.23**
					131	130	9	**0.43**
**373**	CXCL5 P42830, CXCL6 P80162: C-X-C motif chemokine 5, C-X-C motif chemokine 6	2/2	1	1	127	126	1	**1.75**
					129	128	1	**0.42**
					131	130	1	**0.62**
**374**	CYFIP1 Q7L576$_{1}$: Cytoplasmic FMR1-interacting protein 1	1/3	12	12	127	126	4	**1.62**
					129	128	3	**0.66**
					131	130	4	**0.45**
**375**	CYFIP2 Q96F07$_{1,2}$: Cytoplasmic FMR1-interacting protein 2	2/2	9	9	127	126	1	**1.12**
					129	128		
					131	130	1	**0.26**
**376**	DAG1 Q14118: Dystroglycan		4	7	127	126	7	**0.58**
					129	128	7	**1.68**
					131	130	6	**0.84**
**377**	DARS P14868$_{1,2}$: Aspartate--tRNA ligase, cytoplasmic	2/2	7	7	127	126	6	**0.87**
					129	128	6	**1.24**
					131	130	7	**0.55**
**378**	DBNL Q9UJU6$_{1,5}$: Drebrin-like protein	2/2	3	3	127	126	3	**0.82**
					129	128	3	**2.35**
					131	130	3	**1.66**

#	protein	group	peptides	spectra	ch1	ch2	quant	ratio
**379**	DCD P81605$_1$: Dermcidin	1/2	6	9	127	126	9	**10.22**
					129	128	9	**0.05**
					131	130	9	**2.31**
**380**	DCN P07585$_1$: Decorin	1/5	11	28	127	126	25	**1.02**
					129	128	23	**0.23**
					131	130	25	**0.44**
**381**	DCTN1 Q14203$_{1,3,4,6}$: Dynactin subunit 1	4/6	11	12	127	126	9	**1.26**
					129	128	9	**0.63**
					131	130	10	**0.54**
**382**	DCTN2 Q13561$_{1-3}$: Dynactin subunit 2	3/3	4	4	127	126	3	**0.86**
					129	128	3	**1.33**
					131	130	3	**1.38**
**383**	DDB1 Q16531$_1$: DNA damage-binding protein 1	1/2	5	5	127	126	5	**0.91**
					129	128	4	**1.26**
					131	130	4	**1.86**
**384**	DDOST P39656$_{1,3}$: Dolichyl-diphosphooligosaccharide--protein glycosyltransferase 48 kDa subunit	2/2	2	2	127	126	2	**2.32**
					129	128	2	**0.18**
					131	130	2	**0.52**
**385**	DDX1 Q92499$_1$: ATP-dependent RNA helicase DDX1	1/3	12	24	127	126	9	**1.05**
					129	128	9	**1.71**
					131	130	10	**0.37**
**386**	DDX17 Q92841$_{1,3,4}$: Probable ATP-dependent RNA helicase DDX17	3/4	8	10	127	126	3	**0.98**
					129	128	2	**2.45**
					131	130	3	**0.58**
**387**	DDX19A Q9NUU7$_1$: ATP-dependent RNA helicase DDX19A	1/6	8	8	127	126	8	**1.23**
					129	128	8	**0.86**
					131	130	7	**0.47**
**388**	DDX39A O00148$_1$: ATP-dependent RNA helicase DDX39A	1/3	10	12	127	126	2	**2.28**
					129	128	2	**0.38**
					131	130	2	**1.06**
**389**	DDX39B Q13838$_{1,2}$: Spliceosome RNA helicase DDX39B	2/2	13	18	127	126	8	**1.22**
					129	128	6	**0.92**
					131	130	8	**0.39**
**390**	DDX3X O00571$_{1,2}$: ATP-dependent RNA helicase DDX3X	2/4	5	5	127	126	3	**1.00**
					129	128	4	**1.17**
					131	130	3	**0.59**

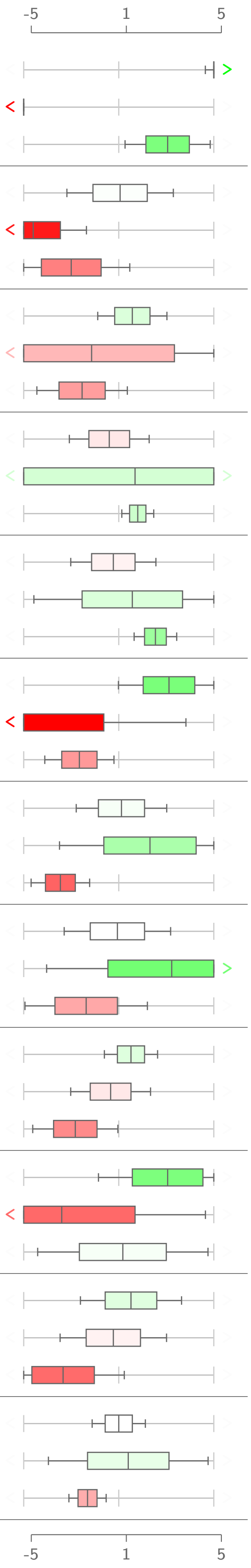

#	protein	group	peptides	spectra	ch1	ch2	quant	ratio
**391**	DDX42 Q86XP3$_{1,2}$: ATP-dependent RNA helicase DDX42	2/2	2	3	127	126	3	**1.38**
					129	128	2	**0.44**
					131	130	3	**0.34**
**392**	DDX5 P17844$_{1,2}$: Probable ATP-dependent RNA helicase DDX5	2/2	11	14	127	126	7	**1.03**
					129	128	6	**1.55**
					131	130	6	**0.45**
**393**	DDX6 P26196: Probable ATP-dependent RNA helicase DDX6		5	6	127	126	5	**1.61**
					129	128	5	**0.78**
					131	130	6	**0.91**
**394**	DEFA1 P59665: Neutrophil defensin 1		3	8	127	126	4	**1.50**
					129	128	4	**0.31**
					131	130	4	**0.94**
**395**	DEFA3 P59666: Neutrophil defensin 3		5	16	127	126	8	**0.48**
					129	128	5	**0.39**
					131	130	12	**1.11**
**396**	DEFA4 P12838: Neutrophil defensin 4		2	3	127	126	3	**0.80**
					129	128	3	**0.23**
					131	130	3	**2.04**
**397**	DEFA5 Q01523: Defensin-5		3	3	127	126	3	**0.42**
					129	128	3	**0.24**
					131	130	3	**1.85**
**398**	DEFA6 Q01524: Defensin-6		1	1	127	126	1	**0.23**
					129	128	1	**0.36**
					131	130	1	**2.34**
**399**	DEK P35659$_{1}$: Protein DEK		2	2	127	126	2	**1.10**
					129	128	2	**1.74**
					131	130	2	**1.76**
**400**	DES P17661: Desmin		25	78	127	126	22	**1.07**
					129	128	20	**0.56**
					131	130	26	**1.04**
**401**	DFNA5 O60443$_{1}$: Non-syndromic hearing impairment protein 5	1/2	10	14	127	126	11	**0.90**
					129	128	8	**0.54**
					131	130	14	**0.19**
**402**	DHX15 O43143: Pre-mRNA-splicing factor ATP-dependent RNA helicase DHX15		8	10	127	126	9	**1.60**
					129	128	9	**0.22**
					131	130	9	**0.63**

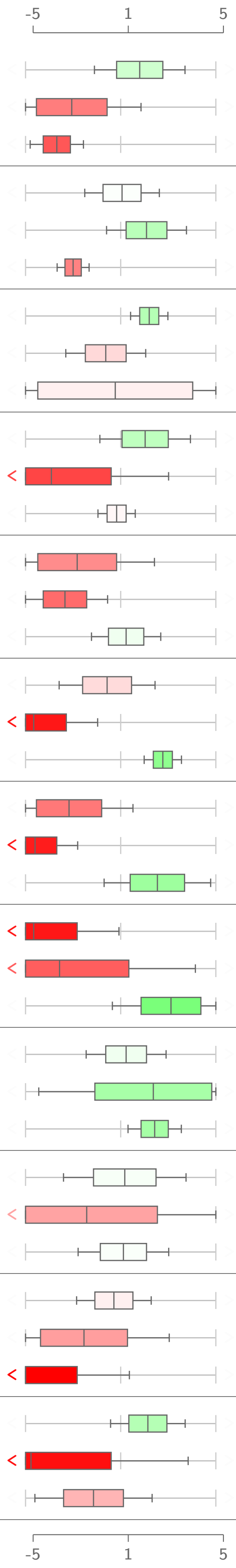

#	protein	group	peptides	spectra	ch1	ch2	quant	ratio
					127	126	14	**1.56**
**403**	DHX9 Q08211$_1$: ATP-dependent RNA helicase A		14	16	129	128	14	**0.57**
					131	130	16	**0.59**
					127	126	6	**1.62**
**404**	DIAPH1 O60610$_{1-3}$: Protein diaphanous homolog 1	3/3	7	8	129	128	6	**0.65**
					131	130	8	**0.40**
					127	126	14	**0.48**
**405**	DKK3 Q9UBP4: Dickkopf-related protein 3		8	28	129	128	12	**1.23**
					131	130	22	**4.12**
					127	126	2	**1.48**
**406**	DLG1 Q12959$_{1-9}$: Disks large homolog 1	9/9	3	3	129	128	2	**0.56**
					131	130	3	**1.17**
					127	126	2	**1.06**
**407**	DLST P36957$_{1,2}$: Dihydrolipoyllysine-residue succinyltransferase component of 2-oxoglutarate ...	2/2	2	2	129	128	2	**0.81**
					131	130	2	**1.03**
					127	126	5	**1.62**
**408**	DMKN Q6E0U4$_{1-7,16}$: Dermokine	8/8	5	6	129	128	6	**0.45**
					131	130	6	**2.11**
					127	126	2	**1.78**
**409**	DMXL2 Q8TDJ6$_{1,3}$: DmX-like protein 2	2/2	2	9	129	128	1	**0.75**
					131	130	2	**2.12**
					127	126	3	**0.85**
**410**	DNAJA2 O60884: DnaJ homolog subfamily A member 2		3	3	129	128	3	**0.89**
					131	130	3	**1.22**
					127	126	2	**2.50**
**411**	DNAJB1 P25685$_1$: DnaJ homolog subfamily B member 1		2	3	129	128	3	**0.97**
					131	130	3	**0.38**
					127	126	3	**0.65**
**412**	DNAJB11 Q9UBS4: DnaJ homolog subfamily B member 11		3	4	129	128	4	**0.76**
					131	130	4	**1.44**
					127	126	3	**0.76**
**413**	DNAJB9 Q9UBS3: DnaJ homolog subfamily B member 9		2	3	129	128	1	**0.47**
					131	130	3	**1.64**
					127	126	9	**0.69**
**414**	DNAJC3 Q13217: DnaJ homolog subfamily C member 3		12	13	129	128	10	**0.56**
					131	130	12	**0.91**

#	protein	group	peptides	spectra	ch1	ch2	quant	ratio
**415**	DNM1L O00429$_{1-6,8}$: Dynamin-1-like protein	7/7	4	4	127	126	4	**4.39**
					129	128	4	**0.21**
					131	130	4	**0.63**
**416**	DNM2 P50570$_{1-5}$: Dynamin-2	5/14	9	10	127	126	9	**1.71**
					129	128	8	**0.76**
					131	130	10	**0.43**
**417**	DNPH1 O43598$_{1,2}$: 2'-deoxynucleoside 5'-phosphate N-hydrolase 1	2/2	2	2	127	126	2	**0.57**
					129	128	2	**1.77**
					131	130	2	**0.90**
**418**	DOCK11 Q5JSL3: Dedicator of cytokinesis protein 11		9	10	127	126	8	**1.44**
					129	128	7	**0.63**
					131	130	9	**2.27**
**419**	DPP4 P27487: Dipeptidyl peptidase 4		7	7	127	126	6	**1.15**
					129	128	2	**0.47**
					131	130	7	**0.41**
**420**	DPT Q07507: Dermatopontin		8	98	127	126	49	**0.40**
					129	128	42	**0.70**
					131	130	80	**2.86**
**421**	DPYSL2 Q16555$_{1,2}$: Dihydropyrimidinase-related protein 2	2/2	6	7	127	126	5	**1.54**
					129	128	3	**0.45**
					131	130	4	**0.80**
**422**	DPYSL3 Q14195$_{1,2}$: Dihydropyrimidinase-related protein 3	2/2	7	9	127	126	7	**0.83**
					129	128	5	**0.94**
					131	130	5	**0.69**
**423**	DSC1 Q08554$_1$: Desmocollin-1	1/2	3	9	127	126	5	**6.12**
					129	128	3	**0.08**
					131	130	5	**2.27**
**424**	DSG1 Q02413$_1$: Desmoglein-1		6	8	127	126	5	**5.62**
					129	128	5	**0.13**
					131	130	5	**1.92**
**425**	DSG3 P32926: Desmoglein-3		3	5	127	126	2	**1.15**
					129	128	2	**0.70**
					131	130	3	**1.66**
**426**	DSP P15924$_1$: Desmoplakin	1/3	32	40	127	126	34	**5.97**
					129	128	34	**0.13**
					131	130	34	**0.95**

#	protein	group	peptides	spectra	ch1	ch2	quant	ratio
427	DST Q03001$_3$: Dystonin		5	26	127	126	16	**0.40**
					129	128	10	**1.38**
					131	130	23	**3.45**
428	DST Q03001$_7$: Dystonin	1/2	8	9	127	126	4	**0.91**
					129	128	3	**0.77**
					131	130	3	**2.50**
429	DSTN P60981$_{1,2}$: Destrin	2/2	6	7	127	126	5	**0.75**
					129	128	5	**0.59**
					131	130	6	**1.38**
430	DUSP23 Q9BVJ7: Dual specificity protein phosphatase 23		1	1	127	126	1	**1.11**
					129	128	1	**0.93**
					131	130	1	**0.65**
431	DVL2 O14641: Segment polarity protein dishevelled homolog DVL-2		2	3	127	126	2	**1.48**
					129	128	3	**0.23**
					131	130	3	**0.89**
432	DYNC1H1 Q14204: Cytoplasmic dynein 1 heavy chain 1		71	96	127	126	83	**1.86**
					129	128	71	**0.61**
					131	130	93	**0.47**
433	DYNC1I2 Q13409$_{1-3,5-7}$: Cytoplasmic dynein 1 intermediate chain 2	6/6	5	5	127	126	4	**0.48**
					129	128	4	**1.72**
					131	130	5	**1.10**
434	DYNC1LI2 O43237$_{1,2}$: Cytoplasmic dynein 1 light intermediate chain 2	2/2	5	6	127	126	6	**2.37**
					129	128	4	**0.70**
					131	130	6	**0.69**
435	DYNC2H1 Q8NCM8$_{1,2}$: Cytoplasmic dynein 2 heavy chain 1	2/2	2	2	127	126	1	**2.43**
					129	128	1	**0.71**
					131	130	1	**1.68**
436	ECHDC1 Q9NTX5$_{1,2,6}$: Ethylmalonyl-CoA decarboxylase	3/4	2	2	127	126	2	**2.18**
					129	128	2	**0.97**
					131	130	2	**0.58**
437	ECM1 Q16610$_{1,4}$: Extracellular matrix protein 1	2/4	22	42	127	126	38	**0.74**
					129	128	30	**1.30**
					131	130	41	**3.47**
438	ECM2 O94769$_1$: Extracellular matrix protein 2	1/2	18	35	127	126	29	**0.59**
					129	128	29	**1.03**
					131	130	34	**1.12**

#	protein	group	peptides	spectra	ch1	ch2	quant	ratio
439	ECM29 Q5VYK3: Proteasome-associated protein ECM29 homolog		4	4	127	126	4	**1.50**
					129	128	3	**1.14**
					131	130	4	**0.23**
440	EEF1A1 P68104$_1$, EEF1A1P5 Q5VTE0: Elongation factor 1-alpha 1, Putative elongation factor 1-alpha-like 3	2/3	17	49	127	126	19	**1.03**
					129	128	21	**1.28**
					131	130	22	**0.37**
441	EEF1A2 Q05639: Elongation factor 1-alpha 2		12	29	127	126	3	**2.68**
					129	128	3	**0.49**
					131	130	3	**0.36**
442	EEF1B2 P24534: Elongation factor 1-beta		5	7	127	126	6	**0.44**
					129	128	5	**1.96**
					131	130	6	**1.51**
443	EEF1D P29692$_{1-3}$: Elongation factor 1-delta	3/4	9	11	127	126	10	**0.77**
					129	128	7	**1.25**
					131	130	9	**1.74**
444	EEF1G P26641$_{1,2}$: Elongation factor 1-gamma	2/2	13	17	127	126	16	**1.01**
					129	128	17	**1.39**
					131	130	16	**0.51**
445	EEF2 P13639: Elongation factor 2		38	70	127	126	61	**1.54**
					129	128	46	**0.84**
					131	130	62	**0.38**
446	EFEMP1 Q12805$_{1-4}$: EGF-containing fibulin-like extracellular matrix protein 1	4/4	24	528	127	126	85	**0.61**
					129	128	61	**0.39**
					131	130	141	**0.65**
447	EFEMP1 Q12805$_5$: EGF-containing fibulin-like extracellular matrix protein 1		22	342	127	126	5	**0.89**
					129	128	4	**0.36**
					131	130	5	**0.66**
448	EFEMP2 O95967: EGF-containing fibulin-like extracellular matrix protein 2		16	83	127	126	61	**0.83**
					129	128	57	**0.74**
					131	130	76	**1.18**
449	EGF P01133$_{1-3}$: Pro-epidermal growth factor	3/3	2	2	127	126	2	**0.84**
					129	128		
					131	130	2	**0.20**
450	EHD1 Q9H4M9: EH domain-containing protein 1	1/2	18	29	127	126	17	**1.00**
					129	128	15	**1.25**
					131	130	19	**0.30**

#	protein	group	peptides	spectra	ch1	ch2	quant	ratio
**451**	EHD2 Q9NZN4$_1$: EH domain-containing protein 2	1/2	17	28	127	126	18	**0.93**
					129	128	14	**1.10**
					131	130	21	**0.44**
**453**	EHD4 Q9H223: EH domain-containing protein 4		4	6	127	126	2	**1.71**
					129	128	2	**1.48**
					131	130	2	**0.31**
**454**	EIF1AX P47813, EIF1AY O14602: Eukaryotic translation initiation factor 1A, X-chromosomal, Eukaryotic translation initiation factor 1A, Y-chromosomal	2/2	2	2	127	126	2	**0.87**
					129	128	2	**0.71**
					131	130	2	**2.46**
**455**	EIF2S1 P05198: Eukaryotic translation initiation factor 2 subunit 1		2	2	127	126	2	**0.57**
					129	128	1	**3.76**
					131	130	2	**0.70**
**456**	EIF2S2 P20042: Eukaryotic translation initiation factor 2 subunit 2		3	3	127	126	3	**0.86**
					129	128	3	**1.44**
					131	130	3	**0.65**
**457**	EIF2S3 P41091, EIF2S3L Q2VIR3$_1$: Eukaryotic translation initiation factor 2 subunit 3, Putative eukaryotic translation initiation factor 2 subunit 3-like protein	2/3	6	6	127	126	6	**1.74**
					129	128	5	**0.44**
					131	130	5	**0.43**
**458**	EIF3A Q14152$_{1,2}$: Eukaryotic translation initiation factor 3 subunit A	2/2	18	19	127	126	18	**1.95**
					129	128	17	**0.61**
					131	130	17	**0.60**
**459**	EIF3B P55884$_{1,2}$: Eukaryotic translation initiation factor 3 subunit B	2/2	6	6	127	126	3	**2.18**
					129	128	5	**0.29**
					131	130	6	**1.26**
**460**	EIF3CL B5ME19: Eukaryotic translation initiation factor 3 subunit C-like protein	1/3	12	12	127	126	11	**1.70**
					129	128	11	**0.72**
					131	130	11	**0.46**
**461**	EIF3E P60228: Eukaryotic translation initiation factor 3 subunit E		6	7	127	126	7	**0.92**
					129	128	3	**0.51**
					131	130	6	**0.65**
**462**	EIF3F O00303: Eukaryotic translation initiation factor 3 subunit F		4	4	127	126	4	**1.65**
					129	128	4	**0.47**
					131	130	4	**0.59**
**463**	EIF3H O15372: Eukaryotic translation initiation factor 3 subunit H		10	10	127	126	7	**0.98**
					129	128	7	**0.86**
					131	130	8	**0.79**

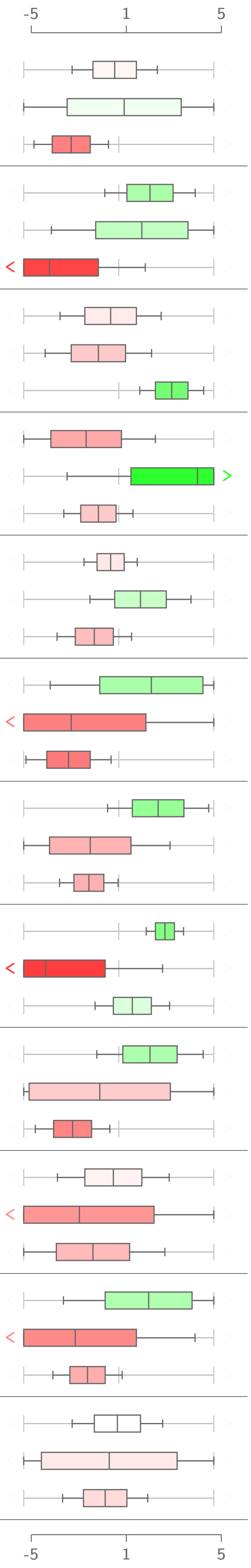

#	protein	group	peptides	spectra	ch1	ch2	quant	ratio
**464**	EIF3I Q13347: Eukaryotic translation initiation factor 3 subunit I		4	4	127	126	4	**1.01**
					129	128	4	**5.06**
					131	130	3	**1.23**
**465**	EIF3J O75822$_1$: Eukaryotic translation initiation factor 3 subunit J	1/3	6	15	127	126	4	**0.55**
					129	128	8	**0.75**
					131	130	12	**2.24**
**466**	EIF3L Q9Y262$_1$: Eukaryotic translation initiation factor 3 subunit L	1/2	8	8	127	126	8	**1.85**
					129	128	7	**0.68**
					131	130	7	**0.78**
**467**	EIF3M Q7L2H7$_{1,2}$: Eukaryotic translation initiation factor 3 subunit M	2/2	3	4	127	126	3	**1.87**
					129	128	3	**0.50**
					131	130	3	**0.42**
**468**	EIF4A1 P60842$_1$: Eukaryotic initiation factor 4A-I	1/2	15	25	127	126	14	**0.94**
					129	128	11	**0.64**
					131	130	14	**0.41**
**469**	EIF4A2 Q14240$_{1,2}$: Eukaryotic initiation factor 4A-II	2/2	10	13	127	126	2	**0.88**
					129	128	2	**5.04**
					131	130	2	**0.12**
**470**	EIF4A3 P38919: Eukaryotic initiation factor 4A-III		8	8	127	126	5	**2.06**
					129	128	4	**0.90**
					131	130	6	**0.45**
**471**	EIF4G1 Q04637$_{1,3-6,9}$: Eukaryotic translation initiation factor 4 gamma 1	6/8	16	20	127	126	17	**1.57**
					129	128	16	**0.30**
					131	130	20	**0.48**
**472**	EIF4G2 P78344$_{1,2}$: Eukaryotic translation initiation factor 4 gamma 2	2/2	2	2	127	126	2	**3.40**
					129	128	1	**0.59**
					131	130	1	**0.17**
**473**	EIF4H Q15056$_{1,2}$: Eukaryotic translation initiation factor 4H	2/2	2	2	127	126	2	**1.76**
					129	128	2	**0.45**
					131	130	2	**1.04**
**474**	EIF5A P63241$_{1,2}$: Eukaryotic translation initiation factor 5A-1	2/4	3	4	127	126	4	**1.17**
					129	128	3	**0.40**
					131	130	4	**1.42**
**475**	EIF5B O60841: Eukaryotic translation factor 5B		4	4	127	126	1	**5.33**
					129	128	2	**0.14**
					131	130	2	**0.92**

#	protein	group	peptides	spectra	ch1	ch2	quant	ratio
**476**	ELAVL1 Q15717$_{1,2}$: ELAV-like protein 1	2/2	2	2	127	126	2	**0.92**
					129	128	1	**1.91**
					131	130	2	**0.46**
**477**	EMILIN1 Q9Y6C2$_{1}$: EMILIN-1	1/2	6	8	127	126	7	**0.65**
					129	128	8	**0.19**
					131	130	7	**1.60**
**478**	EMILIN2 Q9BXX0: EMILIN-2		5	6	127	126	6	**1.14**
					129	128	6	**1.12**
					131	130	6	**1.67**
**479**	EML4 Q9HC35$_{1,2}$: Echinoderm microtubule-associated protein-like 4	2/2	2	2	127	126	1	**0.51**
					129	128	1	**2.08**
					131	130	1	**0.76**
**480**	ENG P17813$_{1,2}$: Endoglin	2/2	1	1	127	126	1	**1.39**
					129	128	1	**0.83**
					131	130	1	**1.13**
**481**	ENO1 P06733$_{1}$: Alpha-enolase	1/7	21	40	127	126	38	**0.98**
					129	128	28	**2.67**
					131	130	38	**0.24**
**482**	EPHX1 P07099: Epoxide hydrolase 1		2	3	127	126	1	**1.34**
					129	128	1	**0.79**
					131	130	3	**0.39**
**483**	EPHX2 P34913$_{1}$: Bifunctional epoxide hydrolase 2 [Includes: Cytosolic epoxide hydrolase 2		2	2	127	126	2	**1.20**
					129	128	2	**1.78**
					131	130	2	**0.15**
**484**	EPPK1 P58107: Epiplakin		16	23	127	126	13	**1.18**
					129	128	6	**0.62**
					131	130	11	**0.60**
**485**	EPS8L1 Q8TE68$_{1,2}$: Epidermal growth factor receptor kinase substrate 8-like protein 1	2/2	2	2	127	126	2	**3.43**
					129	128	2	**0.26**
					131	130	2	**0.23**
**486**	EPX P11678: Eosinophil peroxidase		16	20	127	126	13	**1.90**
					129	128	14	**2.19**
					131	130	12	**1.68**
**488**	ERLIN2 O94905$_{1}$: Erlin-2		2	2	127	126	1	**1.83**
					129	128	1	**1.37**
					131	130	1	**0.81**

#	protein	group	peptides	spectra	ch1	ch2	quant	ratio
**489**	ERP44 Q9BS26: Endoplasmic reticulum resident protein 44		8	9	127	126	9	**1.09**
					129	128	9	**0.93**
					131	130	9	**1.33**
**490**	ETF1 P62495$_1$: Eukaryotic peptide chain release factor subunit 1	1/2	3	3	127	126	3	**10.56**
					129	128	3	**2.20**
					131	130	3	**0.50**
**491**	ETFB P38117$_{1,2}$: Electron transfer flavoprotein subunit beta	2/2	2	3	127	126	3	**2.17**
					129	128	3	**0.89**
					131	130	3	**0.51**
**492**	EWSR1 Q01844$_{1-3,5,6}$: RNA-binding protein EWS	5/5	1	1	127	126	1	**0.46**
					129	128	1	**0.83**
					131	130	1	**1.58**
**493**	EXOC3 O60645$_{1-3}$: Exocyst complex component 3	3/3	2	2	127	126	2	**1.95**
					129	128	2	**0.89**
					131	130	2	**0.47**
**494**	EXOC8 Q8IYI6: Exocyst complex component 8		2	2	127	126	2	**3.12**
					129	128	2	**0.23**
					131	130	2	**0.39**
**495**	EXT2 Q93063$_{1-3}$: Exostosin-2	3/3	2	3	127	126	3	**1.94**
					129	128	3	**0.16**
					131	130	3	**1.08**
**496**	EZR P15311: Ezrin	1/2	22	28	127	126	21	**1.63**
					129	128	23	**0.60**
					131	130	23	**0.40**
**497**	F10 P00742: Coagulation factor X		19	83	127	126	63	**0.43**
					129	128	42	**1.81**
					131	130	67	**1.76**
**498**	F11 P03951$_1$: Coagulation factor XI	1/2	15	25	127	126	25	**0.77**
					129	128	18	**1.41**
					131	130	24	**1.78**
**499**	F12 P00748: Coagulation factor XII		19	43	127	126	43	**0.64**
					129	128	34	**0.41**
					131	130	42	**1.37**
**500**	F13A1 P00488: Coagulation factor XIII A chain		22	59	127	126	56	**0.65**
					129	128	49	**0.96**
					131	130	59	**2.82**

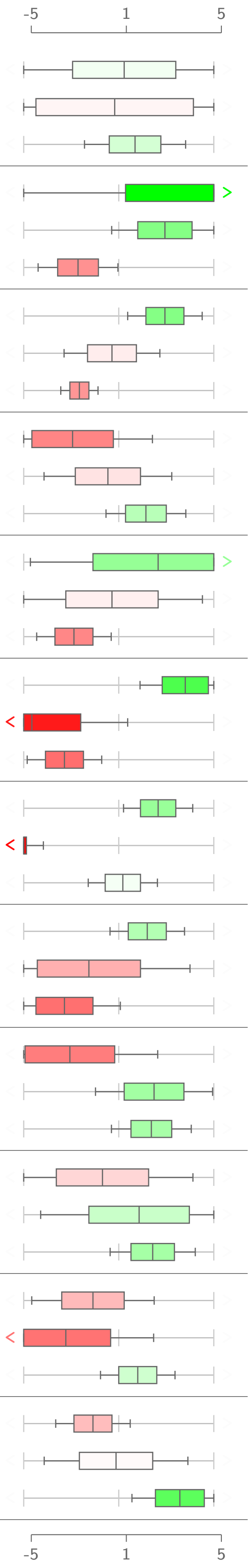

#	protein	group	peptides	spectra	ch1	ch2	quant	ratio
**501**	F13B P05160: Coagulation factor XIII B chain		10	13	127	126	12	**0.37**
					129	128	9	**1.41**
					131	130	11	**3.06**
**502**	F2 P00734: Prothrombin		37	813	127	126	670	**0.31**
					129	128	559	**1.98**
					131	130	767	**1.39**
**503**	F5 P12259: Coagulation factor V		29	42	127	126	36	**0.51**
					129	128	33	**1.45**
					131	130	41	**2.47**
**504**	F7 P08709$_{1,2}$: Coagulation factor VII	2/2	7	10	127	126	8	**0.58**
					129	128	9	**1.72**
					131	130	10	**0.48**
**505**	F9 P00740$_{1}$: Coagulation factor IX	1/2	21	52	127	126	40	**0.41**
					129	128	39	**0.96**
					131	130	43	**1.09**
**506**	FABP3 P05413: Fatty acid-binding protein, heart		3	3	127	126	3	**1.31**
					129	128	3	**0.43**
					131	130	3	**0.39**
**507**	FABP4 P15090: Fatty acid-binding protein, adipocyte		2	3	127	126	3	**0.81**
					129	128	3	**0.14**
					131	130	3	**0.32**
**508**	FAF1 Q9UNN5$_{1,2}$: FAS-associated factor 1	2/2	2	2	127	126	2	**0.77**
					129	128		
					131	130	2	**1.69**
**509**	FAM114A1 Q8IWE2$_{1,2}$: Protein NOXP20	2/2	6	6	127	126	4	**1.16**
					129	128	5	**0.82**
					131	130	6	**0.41**
**510**	FAM120A Q9NZB2$_{1,2,4-6}$: Constitutive coactivator of PPAR-gamma-like protein 1	5/5	2	2	127	126	2	**1.46**
					129	128	2	**0.68**
					131	130	2	**0.62**
**511**	FAM129A Q9BZQ8: Protein Niban		2	2	127	126	1	**0.89**
					129	128	1	**4.52**
					131	130	1	**0.51**
**512**	FAM129B Q96TA1$_{2}$: Niban-like protein 1	1/2	11	23	127	126	19	**1.17**
					129	128	17	**0.62**
					131	130	21	**0.83**

#	protein	group	peptides	spectra	ch1	ch2	quant	ratio
**513**	FAM160B1 Q5W0V3$_{1,2}$: Protein FAM160B1	2/2	2	2	127	126	2	**1.39**
					129	128	2	**1.76**
					131	130	2	**0.76**
**514**	FAM184B Q9ULE4: Protein FAM184B		5	51	127	126	23	**1.55**
					129	128	26	**0.29**
					131	130	40	**3.65**
**515**	FAM49B Q9NUQ9$_1$: Protein FAM49B		5	6	127	126	5	**1.41**
					129	128	4	**0.41**
					131	130	5	**0.33**
**516**	FAM84B Q96KN1: Protein FAM84B		2	2	127	126	2	**1.21**
					129	128	2	**0.92**
					131	130	2	**0.51**
**517**	FARSA Q9Y285$_{1,2}$: Phenylalanine--tRNA ligase alpha subunit	2/2	4	5	127	126	4	**1.72**
					129	128	4	**0.90**
					131	130	3	**0.46**
**518**	FARSB Q9NSD9$_1$: Phenylalanine--tRNA ligase beta subunit	1/2	12	13	127	126	10	**1.51**
					129	128	12	**0.59**
					131	130	11	**0.26**
**519**	FASN P49327: Fatty acid synthase		6	6	127	126	6	**0.81**
					129	128	6	**2.01**
					131	130	6	**0.66**
**520**	FBLN1 P23142$_1$: Fibulin-1	1/2	29	702	127	126	96	**0.44**
					129	128	82	**1.55**
					131	130	91	**0.80**
**521**	FBLN1 P23142$_3$: Fibulin-1		21	594	127	126	1	**4.22**
					129	128	1	**0.30**
					131	130	1	**3.68**
**522**	FBLN1 P23142$_4$: Fibulin-1		24	652	127	126	52	**0.37**
					129	128	43	**0.86**
					131	130	55	**1.92**
**523**	FBLN2 P98095$_{1,2}$: Fibulin-2	2/2	28	49	127	126	43	**0.69**
					129	128	40	**0.57**
					131	130	42	**0.87**
**524**	FBLN5 Q9UBX5: Fibulin-5		15	185	127	126	121	**0.32**
					129	128	109	**1.40**
					131	130	170	**1.88**

#	protein	group	peptides	spectra	ch1	ch2	quant	ratio
525	FBLN7 Q53RD9$_1$: Fibulin-7	1/4	11	14	127	126	12	**0.97**
					129	128	12	**0.82**
					131	130	14	**0.83**
526	FBN1 P35555: Fibrillin-1 [Cleaved into: Asprosin]		63	127	127	126	64	**0.44**
					129	128	55	**0.65**
					131	130	78	**2.51**
527	FBN2 P35556$_1$: Fibrillin-2 [Cleaved into: Fibrillin-2 C-terminal peptide]	1/2	19	68	127	126	21	**1.96**
					129	128	8	**0.91**
					131	130	40	**2.03**
528	FBXO2 Q9UK22: F-box only protein 2		2	2	127	126	2	**0.17**
					129	128		
					131	130	2	**1.61**
529	FCGBP Q9Y6R7: IgGFc-binding protein		12	14	127	126	12	**0.51**
					129	128	11	**0.54**
					131	130	12	**3.33**
530	FCN1 O00602: Ficolin-1		3	6	127	126	1	**0.50**
					129	128	1	**1.82**
					131	130	2	**3.76**
531	FCN2 Q15485$_1$: Ficolin-2	1/2	7	13	127	126	8	**0.15**
					129	128	9	**0.84**
					131	130	9	**3.26**
532	FCN3 O75636$_1$: Ficolin-3		10	25	127	126	3	**0.97**
					129	128	3	**1.60**
					131	130	3	**8.53**
534	FERMT2 Q96AC1$_{1,3}$: Fermitin family homolog 2	2/3	8	12	127	126	9	**1.25**
					129	128	9	**0.55**
					131	130	9	**0.29**
535	FERMT3 Q86UX7$_{1,2}$: Fermitin family homolog 3	2/2	5	6	127	126	3	**0.75**
					129	128	2	**1.00**
					131	130	4	**0.42**
536	FETUB Q9UGM5$_1$: Fetuin-B	1/2	15	33	127	126	28	**1.06**
					129	128	25	**0.78**
					131	130	30	**0.32**
537	FGA P02671$_1$: Fibrinogen alpha chain [Cleaved into: Fibrinopeptide A; Fibrinogen alpha chain]	1/2	54	2709	127	126	2392	**2.83**
					129	128	1738	**1.90**
					131	130	2497	**2.58**

#	protein	group	peptides	spectra	ch1	ch2	quant	ratio
**538**	FGB P02675: Fibrinogen beta chain [Cleaved into: Fibrinopeptide B; Fibrinogen beta chain]		39	2643	127	126	2406	**2.46**
					129	128	1506	**1.83**
					131	130	2520	**2.88**
**539**	FGD6 Q6ZV73$_{1,2}$: FYVE, RhoGEF and PH domain-containing protein 6	2/2	4	4	127	126	3	**2.52**
					129	128	3	**0.88**
					131	130	3	**0.62**
**540**	FGG P02679$_{1,2}$: Fibrinogen gamma chain	2/2	28	1614	127	126	1473	**2.69**
					129	128	1108	**2.07**
					131	130	1545	**2.92**
**541**	FILIP1L Q4L180$_{1-3,7}$: Filamin A-interacting protein 1-like	4/4	2	2	127	126	1	**1.49**
					129	128	1	**2.14**
					131	130	1	**0.47**
**542**	FIS1 Q9Y3D6: Mitochondrial fission 1 protein		2	2	127	126	2	**2.46**
					129	128	2	**0.87**
					131	130	2	**0.29**
**543**	FKBP15 Q5T1M5$_{1,2}$: FK506-binding protein 15	2/2	2	4	127	126	4	**2.16**
					129	128	4	**1.69**
					131	130	4	**0.61**
**544**	FKBP1A P62942: Peptidyl-prolyl cis-trans isomerase FKBP1A		1	2	127	126	2	**1.98**
					129	128	2	**0.60**
					131	130	2	**0.90**
**545**	FLG P20930: Filaggrin		3	3	127	126	3	**14.83**
					129	128	3	**0.05**
					131	130	3	**3.28**
**546**	FLG2 Q5D862: Filaggrin-2		2	2	127	126	2	**11.97**
					129	128	2	**0.10**
					131	130	2	**3.46**
**547**	FLII Q13045$_{1-3}$: Protein flightless-1 homolog	3/3	5	6	127	126	5	**0.93**
					129	128	5	**0.67**
					131	130	6	**0.75**
**548**	FLNA P21333$_{1,2}$: Filamin-A	2/2	88	147	127	126	106	**1.34**
					129	128	101	**0.50**
					131	130	122	**0.37**
**549**	FLNB O75369$_{1-3,6,8,9}$: Filamin-B	6/9	25	30	127	126	19	**1.31**
					129	128	18	**0.58**
					131	130	17	**0.36**

#	protein	group	peptides	spectra	ch1	ch2	quant	ratio
550	FLNC Q14315$_{1,2}$: Filamin-C	2/2	75	102	127	126	77	**1.28**
					129	128	76	**0.47**
					131	130	79	**0.53**
551	FMOD Q06828: Fibromodulin		7	28	127	126	14	**0.70**
					129	128	13	**0.90**
					131	130	15	**2.33**
552	FN1 P02751$_{1,3,17}$: Fibronectin	3/17	87	983	127	126	802	**0.46**
					129	128	669	**1.95**
					131	130	942	**3.04**
553	FN3KRP Q9HA64: Ketosamine-3-kinase		4	4	127	126	4	**1.19**
					129	128	4	**0.91**
					131	130	4	**0.41**
554	FRA10AC1 Q70Z53$_{1-5}$: Protein FRA10AC1	5/5	4	23	127	126	16	**0.49**
					129	128	8	**2.32**
					131	130	17	**0.55**
555	FRZB Q92765: Secreted frizzled-related protein 3		1	2	127	126	2	**0.69**
					129	128	2	**0.49**
					131	130	2	**2.32**
556	FST P19883$_{1,2}$: Follistatin	2/2	7	8	127	126	7	**0.37**
					129	128	6	**0.49**
					131	130	8	**2.89**
557	FSTL1 Q12841$_{1}$: Follistatin-related protein 1	1/2	15	22	127	126	16	**0.29**
					129	128	17	**1.22**
					131	130	18	**2.06**
558	FSTL3 O95633$_{1,2}$: Follistatin-related protein 3	2/2	2	2	127	126	2	**0.31**
					129	128	2	**0.62**
					131	130	2	**2.11**
559	FTH1 P02794: Ferritin heavy chain		2	2	127	126	2	**0.91**
					129	128	2	**0.51**
					131	130	2	**1.76**
560	FTL P02792: Ferritin light chain		4	4	127	126	3	**0.68**
					129	128	3	**1.76**
					131	130	4	**1.52**
561	FUBP1 Q96AE4$_{1,2}$: Far upstream element-binding protein 1	2/2	6	6	127	126	3	**0.84**
					129	128	3	**1.01**
					131	130	3	**1.29**

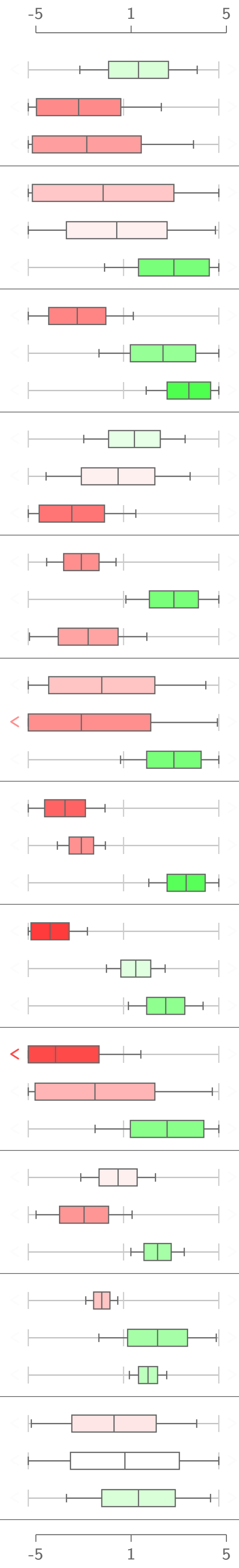

#	protein	group	peptides	spectra	ch1	ch2	quant	ratio
**562**	FUBP3 Q96I24$_1$: Far upstream element-binding protein 3		3	3	127	126	2	**2.51**
					129	128	2	**0.39**
					131	130	2	**1.68**
**563**	FUS P35637$_{1,2}$: RNA-binding protein FUS	2/2	5	10	127	126	6	**0.85**
					129	128	7	**0.27**
					131	130	6	**1.33**
**564**	FXR1 P51114$_{1-3}$: Fragile X mental retardation syndrome-related protein 1	3/3	4	4	127	126	4	**2.11**
					129	128	4	**0.80**
					131	130	3	**0.57**
**565**	FYCO1 Q9BQS8$_{1,4}$: FYVE and coiled-coil domain-containing protein 1	2/2	5	11	127	126	3	**2.91**
					129	128	2	**0.52**
					131	130	5	**0.53**
**566**	G3BP1 Q13283$_1$: Ras GTPase-activating protein-binding protein 1		2	3	127	126	3	**0.78**
					129	128	3	**1.88**
					131	130	3	**0.72**
**567**	G6PD P11413$_{1-3}$: Glucose-6-phosphate 1-dehydrogenase	3/3	5	5	127	126	5	**0.76**
					129	128	3	**1.16**
					131	130	4	**0.88**
**568**	GAK O14976$_{1,2}$: Cyclin-G-associated kinase	2/2	2	2	127	126	2	**0.80**
					129	128	2	**0.83**
					131	130	2	**1.77**
**569**	GALE Q14376$_{1,2}$: UDP-glucose 4-epimerase	2/2	3	3	127	126	2	**0.98**
					129	128	2	**0.84**
					131	130	3	**0.59**
**570**	GALK1 P51570$_{1,2}$: Galactokinase	2/2	3	3	127	126	3	**0.93**
					129	128	3	**1.27**
					131	130	3	**0.83**
**571**	GANAB Q14697$_1$: Neutral alpha-glucosidase AB		16	23	127	126	1	**0.50**
					129	128	1	**4.12**
					131	130	1	**2.12**
**572**	GANAB Q14697$_2$: Neutral alpha-glucosidase AB		16	23	127	126	1	**0.32**
					129	128	1	**2.81**
					131	130	1	**1.38**
**573**	GAPDH P04406$_1$: Glyceraldehyde-3-phosphate dehydrogenase	1/2	16	77	127	126	72	**1.56**
					129	128	65	**1.22**
					131	130	74	**0.46**

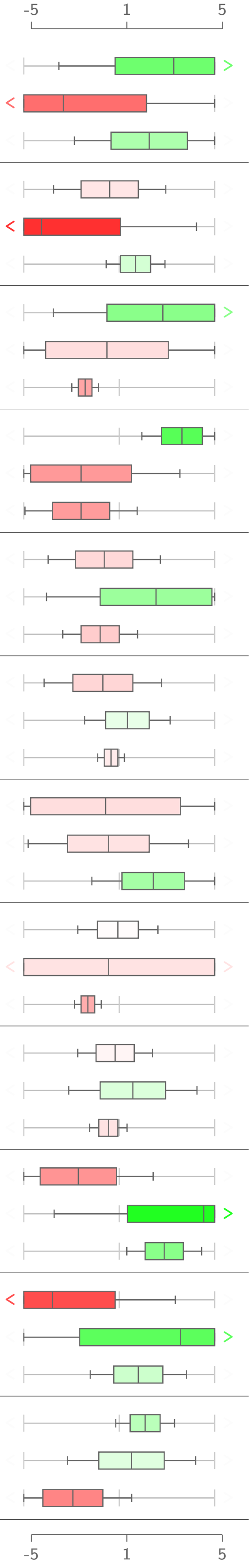

#	protein	group	peptides	spectra	ch1	ch2	quant	ratio
**574**	GARS P41250: Glycine--tRNA ligase		4	7	127	126	3	**1.60**
					129	128	3	**0.20**
					131	130	3	**0.63**
**575**	GART P22102$_1$: Trifunctional purine biosynthetic protein adenosine-3 [Includes: Phospho-ribo . . .	1/2	6	6	127	126	3	**1.61**
					129	128	5	**0.77**
					131	130	6	**0.39**
**576**	GAS1 P54826: Growth arrest-specific protein 1		11	22	127	126	18	**0.47**
					129	128	14	**2.01**
					131	130	21	**3.00**
**577**	GAS6 Q14393$_{1-3}$: Growth arrest-specific protein 6	3/5	14	20	127	126	17	**0.50**
					129	128	16	**0.44**
					131	130	17	**0.82**
**578**	GBA P04062$_{1,2,4,5}$: Glucosylceramidase	4/4	2	2	127	126	2	**2.12**
					129	128	2	**0.37**
					131	130	2	**1.60**
**579**	GBP1 P32455: Guanylate-binding protein 1	1/3	5	5	127	126	3	**1.65**
					129	128	3	**1.09**
					131	130	4	**0.18**
**580**	GBP2 P32456: Guanylate-binding protein 2		3	3	127	126	2	**1.64**
					129	128	2	**3.64**
					131	130	2	**0.08**
**581**	GC P02774$_{1,3}$: Vitamin D-binding protein	2/3	30	276	127	126	210	**0.37**
					129	128	178	**1.76**
					131	130	244	**2.02**
**582**	GCA P28676: Grancalcin		2	2	127	126	2	**0.49**
					129	128	2	**3.50**
					131	130	2	**0.84**
**583**	GCG P01275: Glucagon [Cleaved into: Glicentin; Glicentin-related polypeptide		4	5	127	126	5	**0.50**
					129	128	5	**0.24**
					131	130	5	**1.94**
**584**	GCN1 Q92616: eIF-2-alpha kinase activator GCN1		10	11	127	126	8	**2.35**
					129	128	10	**0.34**
					131	130	11	**0.46**
**585**	GDF15 Q99988: Growth/differentiation factor 15		2	3	127	126	3	**0.95**
					129	128	3	**0.64**
					131	130	3	**0.93**

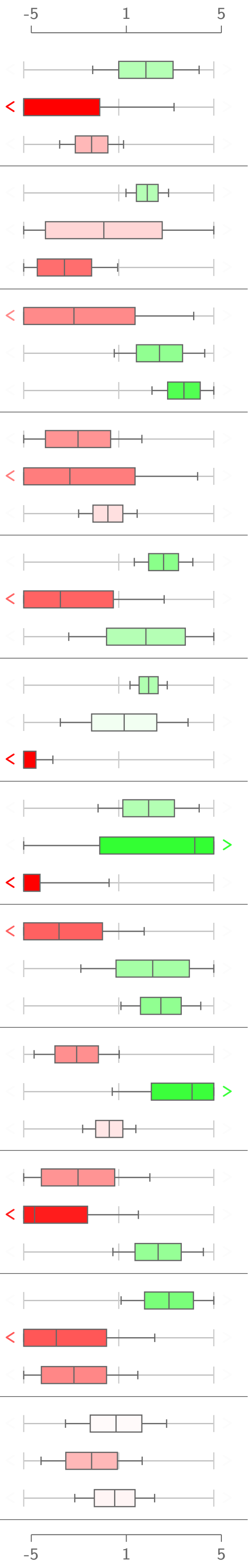

#	protein	group	peptides	spectra	ch1	ch2	quant	ratio
**586**	GFAP P14136$_2$: Glial fibrillary acidic protein	1/3	7	22	127	126	4	**0.83**
					129	128	1	**0.38**
					131	130	6	**1.78**
**587**	GFPT1 Q06210$_{1,2}$: Glutamine--fructose-6-phosphate aminotransferase [isomerizing] 1	2/2	5	7	127	126	7	**1.54**
					129	128	6	**0.30**
					131	130	6	**0.34**
**588**	GFPT2 O94808: Glutamine--fructose-6-phosphate aminotransferase [isomerizing] 2		4	5	127	126	4	**0.80**
					129	128	4	**1.79**
					131	130	5	**0.87**
**589**	GHRL Q9UBU3$_{1-4}$: Appetite-regulating hormone	4/5	3	6	127	126	4	**0.59**
					129	128	2	**0.61**
					131	130	6	**2.63**
**591**	GIP P09681: Gastric inhibitory polypeptide		2	3	127	126	3	**1.18**
					129	128	3	**0.46**
					131	130	3	**0.85**
**592**	GIPC1 O14908$_{1,2}$: PDZ domain-containing protein GIPC1	2/2	2	4	127	126	3	**0.84**
					129	128	3	**0.41**
					131	130	4	**1.30**
**593**	GKN1 Q9NS71: Gastrokine-1		8	23	127	126	23	**1.24**
					129	128	20	**0.43**
					131	130	17	**0.78**
**594**	GKN2 Q86XP6: Gastrokine-2		1	1	127	126	1	**1.35**
					129	128		
					131	130	1	**0.22**
**595**	GLG1 Q92896$_{1-3}$: Golgi apparatus protein 1	3/3	5	7	127	126	6	**4.52**
					129	128	5	**0.33**
					131	130	7	**0.55**
**596**	GLIPR1L2 Q4G1C9$_{1,5,6}$: GLIPR1-like protein 2	3/3	4	21	127	126	5	**2.63**
					129	128	2	**0.98**
					131	130	6	**2.38**
**597**	GM2A P17900: Ganglioside GM2 activator		6	12	127	126	11	**0.68**
					129	128	10	**0.97**
					131	130	11	**0.31**
**598**	GMPPA Q96IJ6$_{1,2}$: Mannose-1-phosphate guanyltransferase alpha	2/2	4	4	127	126	4	**1.84**
					129	128	3	**0.16**
					131	130	4	**0.59**

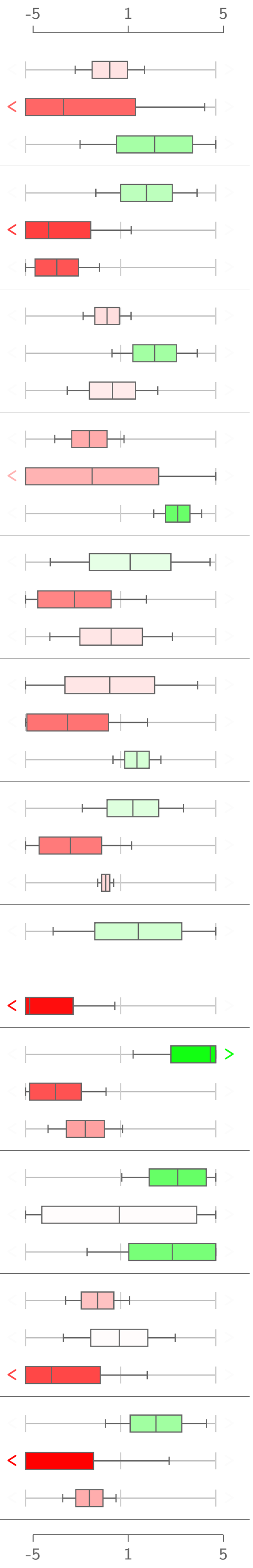

#	protein	group	peptides	spectra	ch1	ch2	quant	ratio
**599**	GMPPB Q9Y5P6$_{1,2}$: Mannose-1-phosphate guanyltransferase beta	2/2	5	5	127	126	4	**1.06**
					129	128	5	**0.47**
					131	130	4	**0.41**
**600**	GMPR P36959: GMP reductase 1		1	1	127	126	1	**0.89**
					129	128	1	**3.76**
					131	130	1	**0.46**
**601**	GNAI2 P04899$_{1-6}$: Guanine nucleotide-binding protein G(i) subunit alpha-2	6/6	2	2	127	126	2	**5.33**
					129	128	2	**0.03**
					131	130	2	**0.88**
**602**	GNAS P63092$_{1-4}$, GNAS Q5JWF2$_{1,2}$: Guanine nucleotide-binding protein G(s) subunit alpha isoforms short, Guanine nucleotide-binding protein G(s) subunit alpha isoforms XLas	6/6	1	1	127	126	1	**1.11**
					129	128	1	**1.65**
					131	130	1	**0.38**
**603**	GNB1 P62873$_{1,2}$: Guanine nucleotide-binding protein G(I)/G(S)/G(T) subunit beta-1	2/4	5	5	127	126	2	**5.76**
					129	128	1	**1.35**
					131	130	2	**1.14**
**604**	GNB2 P62879$_{1}$: Guanine nucleotide-binding protein G(I)/G(S)/G(T) subunit beta-2	1/3	6	6	127	126	3	**1.03**
					129	128	2	**0.17**
					131	130	3	**1.14**
**605**	GNG12 Q9UBI6: Guanine nucleotide-binding protein G(I)/G(S)/G(O) subunit gamma-12		2	2	127	126	2	**1.96**
					129	128	2	**0.32**
					131	130	2	**0.50**
**606**	GNL1 P36915$_{1}$: Guanine nucleotide-binding protein-like 1	1/2	4	4	127	126	2	**0.88**
					129	128	2	**3.44**
					131	130	2	**0.69**
**607**	GNPTG Q9UJJ9: N-acetylglucosamine-1-phosphotransferase subunit gamma		4	4	127	126	4	**0.33**
					129	128	4	**2.54**
					131	130	3	**0.87**
**608**	GOLGA6L2 Q8N9W4$_{1}$: Golgin subfamily A member 6-like protein 2		7	25	127	126	6	**1.52**
					129	128	3	**0.16**
					131	130	12	**1.30**
**609**	GOLGB1 Q14789$_{1-4}$: Golgin subfamily B member 1	4/4	2	2	127	126	1	**0.73**
					129	128	1	**4.06**
					131	130	1	**0.81**
**610**	GOLIM4 O00461: Golgi integral membrane protein 4		10	12	127	126	8	**0.55**
					129	128	8	**0.18**
					131	130	11	**2.75**

#	protein	group	peptides	spectra	ch1	ch2	quant	ratio
**611**	GOLM1 Q8NBJ4$_{1,2}$: Golgi membrane protein 1	2/2	8	9	127	126	8	**0.79**
					129	128	8	**0.81**
					131	130	8	**1.20**
**612**	GPC6 Q9Y625: Glypican-6 [Cleaved into: Secreted glypican-6]		3	13	127	126	3	**1.19**
					129	128	4	**0.61**
					131	130	10	**1.59**
**613**	GPLD1 P80108$_{1}$: Phosphatidylinositol-glycan-specific phospholipase D		11	13	127	126	13	**0.39**
					129	128	8	**1.97**
					131	130	11	**2.67**
**614**	GPS1 Q13098$_{4,5,7}$: COP9 signalosome complex subunit 1	3/4	10	10	127	126	9	**1.63**
					129	128	8	**0.34**
					131	130	10	**0.53**
**615**	GPX1 P07203$_{1}$: Glutathione peroxidase 1		1	1	127	126	1	**0.70**
					129	128	1	**6.20**
					131	130	1	**0.97**
**616**	GPX3 P22352: Glutathione peroxidase 3		9	18	127	126	18	**0.39**
					129	128	17	**1.21**
					131	130	18	**1.49**
**617**	GRHPR Q9UBQ7$_{1}$: Glyoxylate reductase/hydroxypyruvate reductase		5	5	127	126	5	**1.08**
					129	128	5	**1.35**
					131	130	3	**0.59**
**619**	GRN P28799$_{1}$: Granulins	1/3	4	4	127	126	4	**1.73**
					129	128	4	**0.65**
					131	130	4	**2.03**
**620**	GSDMD P57764: Gasdermin-D		3	4	127	126	3	**1.01**
					129	128	3	**0.96**
					131	130	3	**0.62**
**621**	GSN P06396$_{1}$: Gelsolin		40	1095	127	126	35	**1.55**
					129	128	31	**0.85**
					131	130	37	**0.29**
**622**	GSN P06396$_{2}$: Gelsolin	1/3	41	1053	127	126	3	**0.89**
					129	128	2	**2.31**
					131	130	3	**0.15**
**623**	GSPT1 P15170$_{1-3}$: Eukaryotic peptide chain release factor GTP-binding subunit ERF3A	3/3	3	3	127	126	2	**1.27**
					129	128	2	**1.10**
					131	130	3	**0.90**

#	protein	group	peptides	spectra	ch1	ch2	quant	ratio
**624**	GSTK1 Q9Y2Q3$_{1,4}$: Glutathione S-transferase kappa 1	2/2	2	2	127	126	2	**2.84**
					129	128	2	**0.68**
					131	130	2	**0.62**
**625**	GSTM3 P21266: Glutathione S-transferase Mu 3		11	18	127	126	16	**1.00**
					129	128	13	**0.63**
					131	130	17	**0.42**
**626**	GSTO1 P78417$_1$: Glutathione S-transferase omega-1		3	4	127	126	4	**0.86**
					129	128	4	**0.13**
					131	130	4	**0.52**
**627**	GSTP1 P09211: Glutathione S-transferase P		6	10	127	126	10	**0.86**
					129	128	3	**1.17**
					131	130	8	**0.27**
**628**	GYG1 P46976$_{1,2}$: Glycogenin-1	2/3	3	3	127	126	3	**1.09**
					129	128	3	**0.91**
					131	130	3	**0.17**
**629**	H1F0 P07305$_{1,2}$: Histone H1.0	2/2	7	9	127	126	7	**1.74**
					129	128	7	**0.69**
					131	130	7	**1.11**
**630**	H1FX Q92522: Histone H1x		6	7	127	126	7	**2.00**
					129	128	6	**0.66**
					131	130	7	**1.80**
**631**	H2AFV Q71UI9$_1$, H2AFZ P0C0S5: Histone H2A.V, Histone H2A.Z	2/5	4	14	127	126	3	**0.98**
					129	128	3	**1.79**
					131	130	3	**1.22**
**632**	H2AFX P16104: Histone H2AX		9	59	127	126	7	**2.27**
					129	128	4	**0.47**
					131	130	7	**1.80**
**633**	H2AFY O75367$_{1-3}$: Core histone macro-H2A.1	3/3	3	5	127	126	5	**1.65**
					129	128	4	**0.80**
					131	130	5	**0.80**
**634**	H3F3A P84243: Histone H3.3		9	49	127	126	1	**3.51**
					129	128	1	**0.86**
					131	130	1	**1.67**
**635**	H6PD O95479: GDH/6PGL endoplasmic bifunctional protein [Includes: Glucose 1-dehydrogenase		2	2	127	126	2	**0.68**
					129	128		
					131	130	2	**0.68**

#	protein	group	peptides	spectra	ch1	ch2	quant	ratio
**636**	HABP2 Q14520$_{1,2}$: Hyaluronan-binding protein 2	2/2	19	50	127	126	46	**0.33**
					129	128	44	**1.85**
					131	130	47	**1.59**
**637**	HADHA P40939$_{1}$: Trifunctional enzyme subunit alpha, mitochondrial		8	11	127	126	11	**1.94**
					129	128	9	**0.58**
					131	130	9	**0.70**
**638**	HADHB P55084$_{1}$: Trifunctional enzyme subunit beta, mitochondrial	1/2	3	3	127	126	3	**2.98**
					129	128	3	**2.44**
					131	130	3	**1.01**
**639**	HBA1; P69905: Hemoglobin subunit alpha		4	9	127	126	9	**12.25**
					129	128	6	**1.41**
					131	130	9	**1.15**
**640**	HBB P68871: Hemoglobin subunit beta	1/2	7	15	127	126	15	**7.51**
					129	128	13	**0.93**
					131	130	13	**1.00**
**641**	HCLS1 P14317$_{1}$: Hematopoietic lineage cell-specific protein		2	2	127	126	2	**1.18**
					129	128	2	**0.94**
					131	130	2	**1.45**
**642**	HDGF P51858$_{1}$: Hepatoma-derived growth factor	1/3	4	6	127	126	5	**0.67**
					129	128	6	**0.77**
					131	130	6	**1.56**
**643**	HDLBP Q00341$_{1}$: Vigilin	1/2	20	21	127	126	18	**1.53**
					129	128	18	**0.50**
					131	130	21	**0.51**
**644**	HEBP1 Q9NRV9: Heme-binding protein 1		3	3	127	126	3	**7.80**
					129	128	3	**0.26**
					131	130	3	**0.59**
**645**	HERC2 O95714: E3 ubiquitin-protein ligase HERC2		3	20	127	126	4	**0.42**
					129	128	3	**1.48**
					131	130	16	**2.70**
**646**	HEXA P06865$_{1}$: Beta-hexosaminidase subunit alpha		2	2	127	126	2	**0.35**
					129	128	2	**2.94**
					131	130	2	**1.83**
**647**	HEXB P07686: Beta-hexosaminidase subunit beta		2	2	127	126	2	**0.60**
					129	128	2	**0.70**
					131	130	2	**1.12**

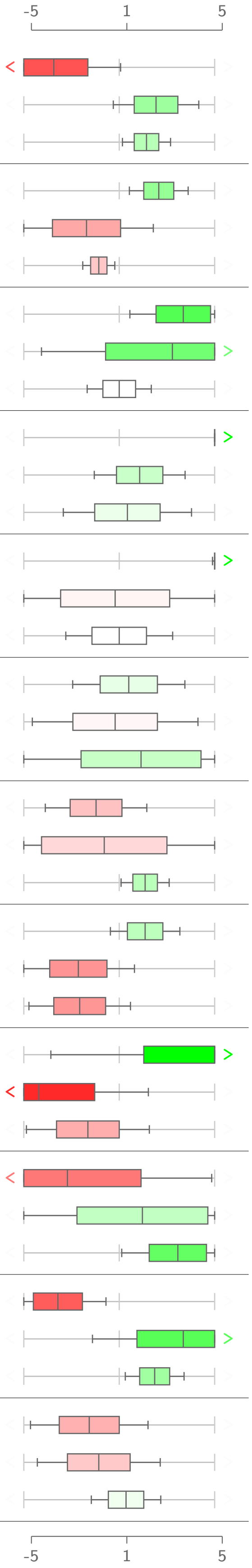

#	protein	group	peptides	spectra	ch1	ch2	quant	ratio
**648**	HGS O14964$_{1,2}$: Hepatocyte growth factor-regulated tyrosine kinase substrate	2/2	2	2	127	126	2	**1.51**
					129	128	2	**0.30**
					131	130	2	**0.84**
**649**	HINT1 P49773: Histidine triad nucleotide-binding protein 1		2	2	127	126	2	**0.68**
					129	128	2	**2.18**
					131	130	2	**0.82**
**650**	HIP1 O00291$_{1,4}$: Huntingtin-interacting protein 1	2/3	3	3	127	126	2	**1.35**
					129	128	1	**2.04**
					131	130	2	**0.41**
**651**	HIP1R O75146$_{1}$: Huntingtin-interacting protein 1-related protein		3	3	127	126	1	**0.21**
					129	128	1	**2.77**
					131	130	1	**2.08**
**652**	HIST1H1B P16401: Histone H1.5		13	26	127	126	9	**1.88**
					129	128	8	**1.19**
					131	130	8	**2.56**
**653**	HIST1H1C P16403: Histone H1.2	1/3	16	35	127	126	8	**1.55**
					129	128	6	**1.20**
					131	130	7	**2.06**
**654**	HIST1H1D P16402: Histone H1.3		10	28	127	126	1	**2.43**
					129	128	1	**0.75**
					131	130	1	**2.26**
**655**	HIST1H1E P10412: Histone H1.4		15	37	127	126	7	**1.93**
					129	128	7	**0.74**
					131	130	5	**1.55**
**656**	HIST1H2AB P04908, HIST1H2AD P20671, HIST3H2A Q7L7L0: Histone H2A type 1-B/E, Histone H2A type 1-D, ...	3/3	6	56	127	126	1	**3.20**
					129	128	1	**0.46**
					131	130	1	**2.60**
**661**	HIST1H3A P68431, HIST2H3A; Q71DI3, HIST3H3 Q16695: Histone H3.1, Histone H3.2, ...	3/4	9	49	127	126	1	**1.70**
					129	128	1	**13.70**
					131	130	1	**3.65**
**662**	HIST1H4A P62805: Histone H4		7	28	127	126	27	**1.97**
					129	128	21	**0.65**
					131	130	28	**1.61**
**663**	HIST2H2AB Q8IUE6: Histone H2A type 2-B		5	57	127	126	1	**1.01**
					129	128	1	**0.50**
					131	130	1	**1.59**

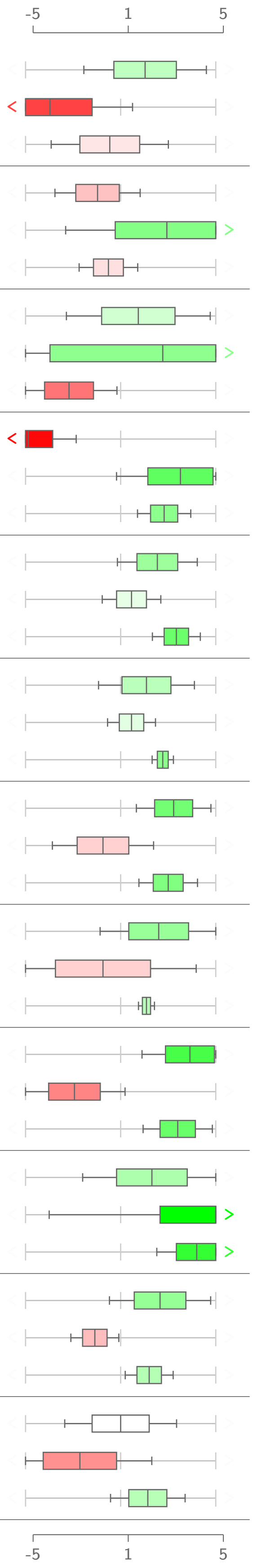

#	protein	group	peptides	spectra	ch1	ch2	quant	ratio
**664**	HIST2H2AC Q16777: Histone H2A type 2-C	1/4	7	64	127	126	2	**3.32**
					129	128	2	**0.61**
					131	130	2	**1.98**
**666**	HLA-A P10316: HLA class I histocompatibility antigen, A-69 alpha chain	1/2	5	6	127	126	3	**0.65**
					129	128	3	**0.84**
					131	130	3	**1.66**
**667**	HLA-A P16190: HLA class I histocompatibility antigen, A-33 alpha chain	1/6	5	5	127	126	1	**12.58**
					129	128		
					131	130	1	**1.05**
**670**	HLA-B P10319, HLA-B P18465: HLA class I histocompatibility antigen, B-58 alpha chain, HLA class I histocompatibility antigen, B-57 alpha chain	2/14	6	7	127	126	1	**0.77**
					129	128	1	**0.53**
					131	130	1	**1.21**
**672**	HLA-C Q07000: HLA class I histocompatibility antigen, Cw-15 alpha chain		3	3	127	126	1	**1.32**
					129	128		
					131	130	1	**1.59**
**673**	HLA-C Q9TNN7: HLA class I histocompatibility antigen, Cw-5 alpha chain		2	2	127	126	1	**2.21**
					129	128	1	**0.70**
					131	130	1	**1.01**
**674**	HLA-DRB1 P01911, HLA-DRB1 P01912, HLA-DRB1 P04229, . . . : HLA class II histocompatibility antigen, DRB1-15 beta chain, HLA class II histocompatibility antigen, DRB1-3 chain, . . .	11/11	2	3	127	126	3	**1.53**
					129	128	3	**1.68**
					131	130	3	**1.02**
**675**	HMCN1 Q96RW7$_{1,2}$: Hemicentin-1	2/2	14	23	127	126	10	**1.17**
					129	128	8	**0.46**
					131	130	13	**0.92**
**676**	HMCN2 Q8NDA2$_{1}$: Hemicentin-2	1/3	6	6	127	126	4	**1.79**
					129	128	6	**0.12**
					131	130	5	**0.70**
**677**	HMGA1 P17096$_{1,2}$: High mobility group protein HMG-I/HMG-Y	2/2	3	4	127	126	3	**1.31**
					129	128	4	**0.46**
					131	130	4	**1.95**
**678**	HMGB2 P26583: High mobility group protein B2		4	4	127	126	1	**1.01**
					129	128	1	**1.65**
					131	130	1	**2.41**
**679**	HNMT P50135$_{1}$: Histamine N-methyltransferase		5	7	127	126	4	**1.26**
					129	128	3	**1.16**
					131	130	5	**0.45**

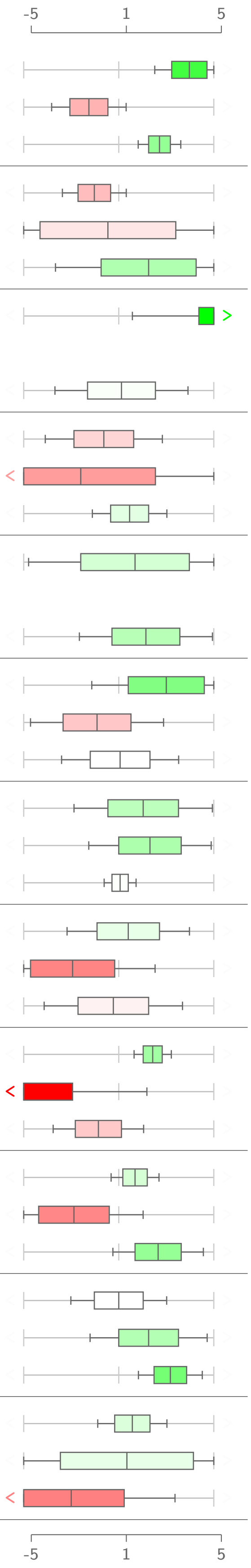

#	protein	group	peptides	spectra	ch1	ch2	quant	ratio
**680**	HNRNPA0 Q13151: Heterogeneous nuclear ribonucleoprotein A0		3	4	127	126	2	**1.31**
					129	128	2	**0.51**
					131	130	2	**0.86**
**681**	HNRNPA1 $P09651_{1-3}$, HNRNPA1L2 Q32P51: Heterogeneous nuclear ribonucleoprotein A1, Heterogeneous nuclear ribonucleoprotein A1-like 2	4/4	4	7	127	126	1	**0.56**
					129	128	1	**3.43**
					131	130	1	**0.98**
**682**	HNRNPA2B1 $P22626_{1,2}$: Heterogeneous nuclear ribonucleoproteins A2/B1	2/2	9	11	127	126	8	**1.29**
					129	128	7	**1.21**
					131	130	7	**1.21**
**683**	HNRNPA3 $P51991_{1}$: Heterogeneous nuclear ribonucleoprotein A3	1/2	8	16	127	126	10	**0.91**
					129	128	10	**1.25**
					131	130	11	**1.64**
**684**	HNRNPAB $Q99729_{1-4}$: Heterogeneous nuclear ribonucleoprotein A/B	4/4	4	4	127	126	3	**1.69**
					129	128	3	**5.28**
					131	130	3	**1.64**
**685**	HNRNPC $P07910_{1,2}$: Heterogeneous nuclear ribonucleoproteins C1/C2	2/4	12	18	127	126	8	**0.68**
					129	128	8	**0.73**
					131	130	7	**2.06**
**686**	HNRNPCL1 O60812, HNRNPCL2 B2RXH8: Heterogeneous nuclear ribonucleoprotein C-like 1, Heterogeneous nuclear ribonucleoprotein C-like 2	2/2	8	12	127	126	2	**0.45**
					129	128	2	**3.09**
					131	130	2	**1.32**
**687**	HNRNPCL3 B7ZW38, HNRNPCL4 P0DMR1: Heterogeneous nuclear ribonucleoprotein C-like 3, Heterogeneous nuclear ribonucleoprotein C-like 4	2/2	8	11	127	126	1	**0.40**
					129	128	1	**4.14**
					131	130	1	**5.62**
**688**	HNRNPD $Q14103_{1,3}$: Heterogeneous nuclear ribonucleoprotein D0	2/2	4	6	127	126	2	**1.47**
					129	128	2	**4.20**
					131	130	2	**2.10**
**689**	HNRNPD $Q14103_{2,4}$: Heterogeneous nuclear ribonucleoprotein D0	2/2	4	5	127	126	1	**1.89**
					129	128	1	**1.46**
					131	130	1	**1.33**
**690**	HNRNPDL $O14979_{2,3}$: Heterogeneous nuclear ribonucleoprotein D-like	2/3	8	9	127	126	8	**0.90**
					129	128	6	**2.64**
					131	130	7	**1.87**
**691**	HNRNPF P52597: Heterogeneous nuclear ribonucleoprotein F		7	11	127	126	4	**0.85**
					129	128	5	**1.45**
					131	130	5	**1.81**

#	protein	group	peptides	spectra	ch1	ch2	quant	ratio
**692**	HNRNPH1 P31943: Heterogeneous nuclear ribonucleoprotein H		10	14	127	126	4	**1.10**
					129	128	2	**3.40**
					131	130	4	**1.77**
**693**	HNRNPH2 P55795: Heterogeneous nuclear ribonucleoprotein H2		9	12	127	126	1	**1.06**
					129	128	1	**2.43**
					131	130	2	**1.36**
**695**	HNRNPH3 P31942$_4$: Heterogeneous nuclear ribonucleoprotein H3	1/2	4	4	127	126	2	**1.04**
					129	128	1	**1.10**
					131	130	2	**1.47**
**696**	HNRNPK P61978$_{2,3}$: Heterogeneous nuclear ribonucleoprotein K	2/3	20	40	127	126	31	**0.71**
					129	128	28	**1.09**
					131	130	38	**0.66**
**697**	HNRNPL P14866$_1$: Heterogeneous nuclear ribonucleoprotein L	1/2	14	18	127	126	15	**1.09**
					129	128	12	**1.33**
					131	130	17	**0.66**
**698**	HNRNPLL Q8WVV9$_{1,4,5}$: Heterogeneous nuclear ribonucleoprotein L-like	3/3	3	5	127	126	3	**1.77**
					129	128	2	**1.31**
					131	130	3	**2.30**
**699**	HNRNPM P52272$_{1,2}$: Heterogeneous nuclear ribonucleoprotein M	2/2	13	14	127	126	13	**1.38**
					129	128	13	**0.93**
					131	130	13	**0.81**
**700**	HNRNPR O43390$_{1,2,4}$: Heterogeneous nuclear ribonucleoprotein R	3/4	9	12	127	126	2	**0.80**
					129	128	2	**4.69**
					131	130	2	**1.05**
**701**	HNRNPU Q00839$_1$: Heterogeneous nuclear ribonucleoprotein U		16	23	127	126	2	**1.12**
					129	128	1	**1.36**
					131	130	2	**0.92**
**702**	HNRNPU Q00839$_2$: Heterogeneous nuclear ribonucleoprotein U		15	22	127	126	1	**1.07**
					129	128	1	**5.94**
					131	130	1	**1.47**
**703**	HNRNPUL1 Q9BUJ2$_{1-4}$: Heterogeneous nuclear ribonucleoprotein U-like protein 1	4/5	6	7	127	126	4	**0.98**
					129	128	5	**0.80**
					131	130	5	**0.99**
**704**	HNRNPUL2 Q1KMD3: Heterogeneous nuclear ribonucleoprotein U-like protein 2		16	28	127	126	22	**1.18**
					129	128	19	**1.52**
					131	130	23	**1.51**

#	protein	group	peptides	spectra	ch1	ch2	quant	ratio
**705**	HP P00738$_1$: Haptoglobin	1/2	23	73	127	126	21	**0.91**
					129	128	22	**1.27**
					131	130	22	**1.21**
**706**	HP1BP3 Q5SSJ5$_{1,2}$: Heterochromatin protein 1-binding protein 3	2/3	7	9	127	126	7	**1.21**
					129	128	7	**0.62**
					131	130	9	**1.47**
**707**	HPCA P84074, HPCAL1 P37235, NCALD P61601: Neuron-specific calcium-binding protein hippocalcin, Hippocalcin-like protein 1, ...	3/3	3	3	127	126	2	**1.50**
					129	128	3	**0.15**
					131	130	3	**0.69**
**708**	HPR P00739$_{1,2}$: Haptoglobin-related protein	2/2	22	90	127	126	39	**0.47**
					129	128	30	**2.25**
					131	130	40	**0.58**
**709**	HPX P02790: Hemopexin		10	24	127	126	20	**0.74**
					129	128	23	**0.52**
					131	130	24	**1.84**
**710**	HRC P23327: Sarcoplasmic reticulum histidine-rich calcium-binding protein		4	11	127	126	1	**0.86**
					129	128		
					131	130	3	**2.73**
**711**	HRG P04196: Histidine-rich glycoprotein		21	315	127	126	251	**0.57**
					129	128	257	**0.46**
					131	130	307	**3.30**
**712**	HRNR Q86YZ3: Hornerin		3	4	127	126	3	**13.29**
					129	128	3	**0.13**
					131	130	4	**2.50**
**713**	HS6ST1 O60243$_1$: Heparan-sulfate 6-O-sulfotransferase 1		2	3	127	126	3	**0.43**
					129	128	3	**2.61**
					131	130	3	**0.98**
**714**	HSD17B10 Q99714$_{1,2}$: 3-hydroxyacyl-CoA dehydrogenase type-2	2/2	1	2	127	126	2	**1.85**
					129	128	1	**0.34**
					131	130	2	**1.06**
**715**	HSP90AA1 P07900$_{1,2}$: Heat shock protein HSP 90-alpha	2/7	36	121	127	126	34	**0.80**
					129	128	28	**1.00**
					131	130	37	**0.68**
**716**	HSP90AA5P Q58FG0: Putative heat shock protein HSP 90-alpha A5		8	19	127	126	2	**3.63**
					129	128	1	**1.04**
					131	130	2	**3.15**

#	protein	group	peptides	spectra	ch1	ch2	quant	ratio
717	HSP90AB1 P08238: Heat shock protein HSP 90-beta	1/2	37	114	127	126	19	**0.79**
					129	128	13	**1.21**
					131	130	17	**0.49**
718	HSP90AB3P Q58FF7: Putative heat shock protein HSP 90-beta-3		18	70	127	126	1	**0.64**
					129	128		
					131	130	2	**2.86**
719	HSP90B1 P14625: Endoplasmin	1/2	37	83	127	126	67	**0.75**
					129	128	44	**1.22**
					131	130	77	**0.91**
720	HSPA12A O43301: Heat shock 70 kDa protein 12A		2	2	127	126	2	**1.29**
					129	128	2	**0.36**
					131	130	2	**0.17**
721	HSPA1A P0DMV8$_{1,2}$, HSPA1B P0DMV9: Heat shock 70 kDa protein 1A, Heat shock 70 kDa protein 1B	3/4	21	39	127	126	7	**1.33**
					129	128	6	**2.19**
					131	130	7	**0.44**
723	HSPA2 P54652: Heat shock-related 70 kDa protein 2		24	44	127	126	17	**1.50**
					129	128	14	**0.74**
					131	130	16	**0.38**
724	HSPA5 P11021: 78 kDa glucose-regulated protein		26	47	127	126	33	**0.64**
					129	128	26	**2.39**
					131	130	36	**1.41**
726	HSPA8 P11142$_1$: Heat shock cognate 71 kDa protein	1/2	24	50	127	126	24	**0.97**
					129	128	20	**2.22**
					131	130	23	**0.82**
727	HSPA9 P38646: Stress-70 protein, mitochondrial		7	8	127	126	7	**2.55**
					129	128	6	**0.83**
					131	130	5	**0.76**
728	HSPB1 P04792: Heat shock protein beta-1		7	9	127	126	9	**0.58**
					129	128	9	**1.04**
					131	130	9	**1.31**
729	HSPB6 O14558: Heat shock protein beta-6		2	2	127	126	2	**0.69**
					129	128	2	**0.45**
					131	130	2	**1.01**
730	HSPD1 P10809$_1$: 60 kDa heat shock protein, mitochondrial		9	10	127	126	9	**1.44**
					129	128	8	**1.53**
					131	130	9	**1.17**

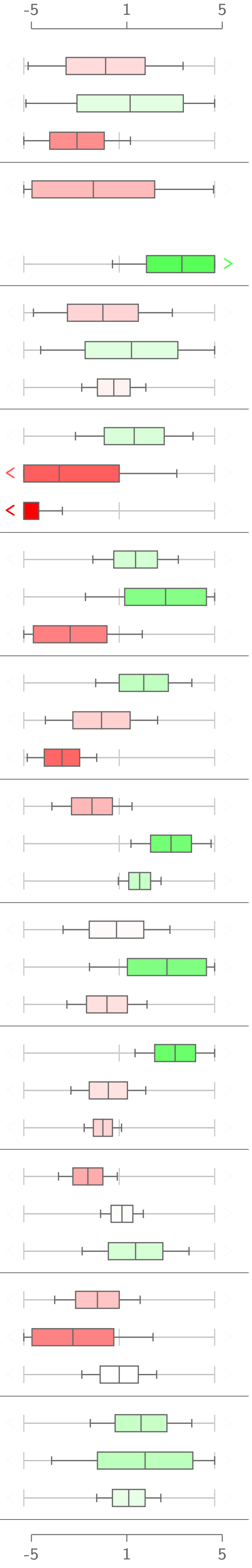

#	protein	group	peptides	spectra	ch1	ch2	quant	ratio
**731**	HSPG2 P98160: Basement membrane-specific heparan sulfate proteoglycan core protein		66	84	127	126	69	**0.99**
					129	128	60	**0.63**
					131	130	74	**0.87**
**732**	HTRA1 Q92743: Serine protease HTRA1		15	49	127	126	41	**0.59**
					129	128	31	**1.01**
					131	130	37	**0.62**
**733**	HUWE1 $Q7Z6Z7_{1,2}$: E3 ubiquitin-protein ligase HUWE1	2/3	21	25	127	126	20	**1.89**
					129	128	20	**0.82**
					131	130	23	**0.48**
**734**	HYDIN $Q4G0P3_{1}$: Hydrocephalus-inducing protein homolog		5	13	127	126	11	**0.50**
					129	128	8	**4.45**
					131	130	12	**0.42**
**735**	HYOU1 $Q9Y4L1_{1}$: Hypoxia up-regulated protein 1	1/2	13	14	127	126	14	**0.91**
					129	128	13	**1.71**
					131	130	14	**0.87**
**736**	IARS P41252: Isoleucine--tRNA ligase, cytoplasmic		3	3	127	126	2	**1.47**
					129	128	2	**1.32**
					131	130	2	**0.57**
**737**	IFI16 $Q16666_{1-3,6}$: Gamma-interferon-inducible protein 16	4/4	2	2	127	126	1	**1.06**
					129	128		
					131	130	1	**0.50**
**738**	IFI30 P13284: Gamma-interferon-inducible lysosomal thiol reductase		4	16	127	126	12	**1.25**
					129	128	9	**0.69**
					131	130	16	**1.80**
**739**	IGF1 $P05019_{1}$: Insulin-like growth factor I		3	5	127	126	5	**1.01**
					129	128	4	**0.24**
					131	130	4	**1.96**
**740**	IGF2 $P01344_{1-3}$: Insulin-like growth factor II	3/3	5	7	127	126	5	**0.86**
					129	128	6	**0.28**
					131	130	7	**2.05**
**741**	IGFALS $P35858_{1,2}$: Insulin-like growth factor-binding protein complex acid labile subunit	2/2	7	8	127	126	7	**0.48**
					129	128	6	**2.18**
					131	130	7	**0.85**
**742**	IGFBP1 P08833: Insulin-like growth factor-binding protein 1		10	20	127	126	18	**1.26**
					129	128	13	**0.63**
					131	130	19	**3.23**

#	protein	group	peptides	spectra	ch1	ch2	quant	ratio
**743**	IGFBP2 P18065: Insulin-like growth factor-binding protein 2		12	18	127	126	17	**1.07**
					129	128	16	**0.37**
					131	130	18	**1.48**
**744**	IGFBP3 P17936$_{1,2}$: Insulin-like growth factor-binding protein 3	2/2	14	33	127	126	28	**0.49**
					129	128	29	**0.42**
					131	130	32	**2.53**
**745**	IGFBP4 P22692$_{1}$: Insulin-like growth factor-binding protein 4	1/2	10	16	127	126	16	**0.67**
					129	128	14	**0.21**
					131	130	16	**1.40**
**746**	IGFBP5 P24593: Insulin-like growth factor-binding protein 5		12	24	127	126	18	**0.68**
					129	128	18	**0.12**
					131	130	21	**1.61**
**747**	IGFBP6 P24592: Insulin-like growth factor-binding protein 6		3	5	127	126	5	**0.72**
					129	128	5	**0.32**
					131	130	5	**1.31**
**748**	IGFBP7 Q16270$_{1,2}$: Insulin-like growth factor-binding protein 7	2/2	7	7	127	126	6	**0.31**
					129	128	7	**0.80**
					131	130	7	**1.44**
**749**	IGHA1 P01876: Ig alpha-1 chain C region		13	115	127	126	30	**0.30**
					129	128	21	**2.45**
					131	130	34	**1.67**
**750**	IGHA2 P01877: Ig alpha-2 chain C region		10	78	127	126	2	**0.79**
					129	128	2	**0.30**
					131	130	2	**2.41**
**751**	IGHD P01880$_{1,2}$: Ig delta chain C region	2/2	7	8	127	126	8	**1.22**
					129	128	6	**2.37**
					131	130	7	**0.59**
**752**	IGHG1 P01857: Ig gamma-1 chain C region		15	326	127	126	89	**0.30**
					129	128	79	**1.53**
					131	130	123	**1.05**
**753**	IGHG2 P01859: Ig gamma-2 chain C region		10	129	127	126	8	**0.36**
					129	128	8	**2.51**
					131	130	12	**1.23**
**754**	IGHG3 P01860: Ig gamma-3 chain C region		15	257	127	126	54	**0.68**
					129	128	47	**1.59**
					131	130	67	**1.12**

#	protein	group	peptides	spectra	ch1	ch2	quant	ratio
**755**	IGHG4 P01861: Ig gamma-4 chain C region		11	113	127	126	10	**0.78**
					129	128	9	**2.15**
					131	130	10	**0.59**
**756**	IGHM P01871$_{1,2}$: Ig mu chain C region	2/2	19	123	127	126	48	**0.51**
					129	128	36	**1.70**
					131	130	56	**3.26**
**757**	IGHV1-2 P23083, A0A0C4DH31: Immunoglobulin heavy variable 1-2	2/2	1	5	127	126	5	**1.15**
					129	128	3	**0.97**
					131	130	4	**1.98**
**758**	IGHV1-69 P01742: Immunoglobulin heavy variable 1-69		2	8	127	126	1	**0.26**
					129	128		
					131	130	1	**0.46**
**759**	IGHV3-30 P01768: Immunoglobulin heavy variable 3-30	1/2	2	8	127	126	2	**0.52**
					129	128	1	**0.60**
					131	130	2	**2.07**
**761**	IGHV3-7 P01780: Immunoglobulin heavy variable 3-7	1/5	3	8	127	126	2	**0.37**
					129	128	2	**3.45**
					131	130	2	**2.63**
**762**	IGHV3-9 P01782: Immunoglobulin heavy variable 3-9		1	2	127	126	2	**0.42**
					129	128	1	**0.80**
					131	130	2	**1.14**
**763**	IGHV4-39 P01824: Immunoglobulin heavy variable 4-39	1/3	4	10	127	126	10	**0.23**
					129	128	7	**3.09**
					131	130	10	**0.91**
**764**	IGKC P01834: Ig kappa chain C region		5	104	127	126	55	**0.41**
					129	128	30	**1.61**
					131	130	90	**1.33**
**765**	IGKV1-17 P01599: Immunoglobulin kappa variable 1-17	1/7	3	9	127	126	2	**0.32**
					129	128	2	**0.62**
					131	130	2	**1.89**
**766**	IGKV1D-33 P01593, IGKV1-33 P01594: Immunoglobulin kappa variable 1D-33, Immunoglobulin kappa variable 1-33	2/2	1	1	127	126	1	**0.37**
					129	128	1	**34.83**
					131	130	1	**2.01**
**767**	IGKV1-5 P01602: Immunoglobulin kappa variable 1-5		2	9	127	126	2	**0.38**
					129	128	2	**1.80**
					131	130	2	**1.12**

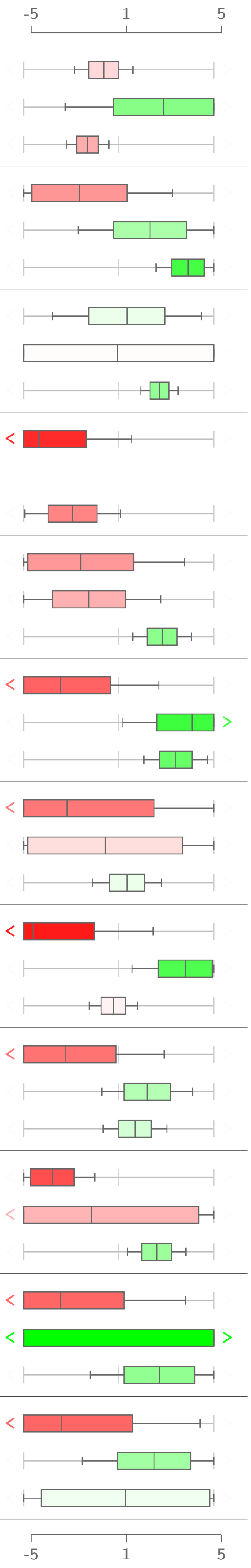

#	protein	group	peptides	spectra	ch1	ch2	quant	ratio
768	IGKV2-30 P06310: Immunoglobulin kappa variable 2-30		2	19	127	126	2	**0.55**
					129	128	2	**1.64**
					131	130	2	**1.70**
769	IGKV2D-28 P01615, IGKV2D-40 P01614, A0A075B6P5: Immunoglobulin kappa variable 2D-28, Immunoglobulin kappa variable 2D-40	3/4	2	29	127	126	12	**0.68**
					129	128	11	**0.51**
					131	130	10	**1.30**
770	IGKV3-11 P04433: Immunoglobulin kappa variable 3-11		3	18	127	126	3	**0.36**
					129	128	3	**2.83**
					131	130	3	**1.67**
771	IGKV3-15 P01624: Immunoglobulin kappa variable 3-15		4	19	127	126	3	**0.43**
					129	128	4	**1.57**
					131	130	4	**1.12**
772	IGKV3-20 P01619: Immunoglobulin kappa variable 3-20		4	20	127	126	2	**0.77**
					129	128	2	**0.26**
					131	130	2	**1.56**
773	IGKV4-1 P06312: Immunoglobulin kappa variable 4-1		3	7	127	126	7	**0.55**
					129	128	5	**1.88**
					131	130	6	**2.25**
774	IGLC2 P0CG05: Ig lambda-2 chain C regions	1/2	6	253	127	126	76	**0.36**
					129	128	60	**1.99**
					131	130	73	**1.67**
776	IGLC7 A0M8Q6: Ig lambda-7 chain C region		6	104	127	126	3	**0.40**
					129	128	3	**0.86**
					131	130	5	**1.31**
777	IGLL5 B9A064$_1$: Immunoglobulin lambda-like polypeptide 5	1/2	8	167	127	126	26	**0.28**
					129	128	24	**1.66**
					131	130	28	**1.17**
778	IGLV1-47 P01700: Immunoglobulin lambda variable 1-47	1/2	2	3	127	126	3	**0.35**
					129	128	3	**0.82**
					131	130	3	**1.13**
779	IGLV1-51 P01701: Immunoglobulin lambda variable 1-51		2	5	127	126	5	**0.45**
					129	128	4	**1.37**
					131	130	5	**1.62**
780	IGLV2-14 P01704: Immunoglobulin lambda variable 2-14		1	3	127	126	3	**0.87**
					129	128	3	**1.36**
					131	130	3	**1.20**

#	protein	group	peptides	spectra	ch1	ch2	quant	ratio
**781**	IGLV3-21 P80748: Immunoglobulin lambda variable 3-21		2	4	127	126	3	**0.39**
					129	128	2	**5.24**
					131	130	4	**1.97**
**782**	IGLV3-25 P01717: Immunoglobulin lambda variable 3-25		2	4	127	126	2	**0.27**
					129	128	2	**2.70**
					131	130	2	**3.83**
**783**	IGLV3-27 P01718: Immunoglobulin lambda variable 3-27		2	3	127	126	1	**0.28**
					129	128	1	**11.06**
					131	130	1	**1.97**
**784**	IGLV7-43 P04211: Immunoglobulin lambda variable 7-43		2	3	127	126	3	**0.42**
					129	128	3	**0.91**
					131	130	3	**1.18**
**785**	IL18 Q14116$_{1,2}$: Interleukin-18	2/2	2	2	127	126	2	**0.93**
					129	128	2	**3.39**
					131	130	2	**1.43**
**786**	ILF2 Q12905: Interleukin enhancer-binding factor 2		4	4	127	126	3	**0.94**
					129	128	4	**0.22**
					131	130	4	**0.34**
**787**	ILF3 Q12906$_{4}$: Interleukin enhancer-binding factor 3	1/9	11	15	127	126	15	**1.14**
					129	128	11	**0.99**
					131	130	15	**0.45**
**788**	IMMT Q16891$_{1,2,4}$: MICOS complex subunit MIC60	3/4	6	6	127	126	6	**1.34**
					129	128	5	**0.80**
					131	130	6	**0.59**
**789**	IMPAD1 Q9NX62: Inositol monophosphatase 3		5	5	127	126	4	**1.05**
					129	128	5	**0.60**
					131	130	5	**0.29**
**790**	IMPDH2 P12268: Inosine-5'-monophosphate dehydrogenase 2		2	2	127	126	2	**1.00**
					129	128	2	**2.23**
					131	130	2	**1.16**
**791**	IMUP Q9GZP8$_{1}$: Immortalization up-regulated protein	1/2	2	2	127	126	1	**1.13**
					129	128	2	**0.65**
					131	130	2	**2.43**
**792**	INCENP Q9NQS7$_{1,2}$: Inner centromere protein	2/2	2	4	127	126	3	**0.75**
					129	128	3	**2.28**
					131	130	3	**0.95**

#	protein	group	peptides	spectra	ch1	ch2	quant	ratio
793	INF2 Q27J81$_{1,2}$: Inverted formin-2	2/2	4	5	127	126	4	**1.94**
					129	128	5	**0.48**
					131	130	5	**0.70**
794	INHBB P09529: Inhibin beta B chain		5	7	127	126	6	**1.35**
					129	128	4	**0.40**
					131	130	6	**0.40**
795	INHBC P55103: Inhibin beta C chain		8	19	127	126	14	**0.86**
					129	128	12	**0.91**
					131	130	17	**1.33**
796	INHBE P58166: Inhibin beta E chain		5	9	127	126	5	**0.99**
					129	128	4	**0.70**
					131	130	9	**1.01**
797	IPO4 Q8TEX9$_{1,2}$: Importin-4	2/2	3	3	127	126	3	**1.52**
					129	128	3	**0.48**
					131	130	3	**0.86**
798	IPO5 O00410$_{1,3}$: Importin-5	2/3	10	12	127	126	6	**1.11**
					129	128	7	**1.22**
					131	130	9	**0.38**
799	IPO7 O95373: Importin-7		3	3	127	126	3	**1.01**
					129	128	2	**8.28**
					131	130	3	**0.67**
800	IQGAP1 P46940: Ras GTPase-activating-like protein IQGAP1		40	56	127	126	51	**1.00**
					129	128	51	**0.91**
					131	130	55	**0.53**
801	ISLR O14498: Immunoglobulin superfamily containing leucine-rich repeat protein		7	13	127	126	12	**0.45**
					129	128	12	**0.74**
					131	130	13	**0.48**
802	ITGA2 P17301: Integrin alpha-2		4	7	127	126	2	**3.02**
					129	128	2	**0.15**
					131	130	3	**0.33**
803	ITGB1 P05556$_{1}$: Integrin beta-1	1/5	11	15	127	126	13	**0.92**
					129	128	12	**0.64**
					131	130	15	**0.56**
804	ITGBL1 O95965$_{1}$: Integrin beta-like protein 1	1/3	15	42	127	126	19	**0.38**
					129	128	11	**0.74**
					131	130	26	**2.13**

#	protein	group	peptides	spectra	ch1	ch2	quant	ratio
805	ITIH1 P19827$_1$: Inter-alpha-trypsin inhibitor heavy chain H1	1/3	23	166	127	126	146	**1.26**
					129	128	105	**0.50**
					131	130	163	**0.40**
806	ITIH2 P19823: Inter-alpha-trypsin inhibitor heavy chain H2		30	526	127	126	459	**1.10**
					129	128	311	**0.57**
					131	130	517	**0.38**
807	ITIH3 Q06033$_{1,2}$: Inter-alpha-trypsin inhibitor heavy chain H3	2/2	39	201	127	126	189	**1.16**
					129	128	117	**0.71**
					131	130	193	**0.52**
808	ITIH4 Q14624$_1$: Inter-alpha-trypsin inhibitor heavy chain H4		35	361	127	126	18	**0.44**
					129	128	13	**1.76**
					131	130	15	**1.16**
809	ITIH4 Q14624$_{2,3}$: Inter-alpha-trypsin inhibitor heavy chain H4	2/3	32	347	127	126	3	**0.60**
					129	128	1	**1.04**
					131	130	4	**0.08**
810	ITIH5 Q86UX2$_1$: Inter-alpha-trypsin inhibitor heavy chain H5	1/3	11	17	127	126	17	**1.10**
					129	128	16	**0.46**
					131	130	16	**0.42**
811	ITLN1 Q8WWA0: Intelectin-1	1/3	15	157	127	126	140	**0.49**
					129	128	123	**1.68**
					131	130	155	**4.74**
812	ITM2A O43736$_{1,2}$: Integral membrane protein 2A	2/2	1	1	127	126	1	**0.50**
					129	128	1	**0.76**
					131	130	1	**3.37**
813	IVD P26440$_1$: Isovaleryl-CoA dehydrogenase, mitochondrial	1/2	3	3	127	126	3	**1.39**
					129	128	2	**0.85**
					131	130	3	**0.68**
814	JCHAIN P01591: Immunoglobulin J chain		5	15	127	126	11	**0.23**
					129	128	11	**2.04**
					131	130	15	**2.35**
815	JUP P14923: Junction plakoglobin		6	8	127	126	7	**6.20**
					129	128	5	**0.14**
					131	130	6	**0.88**
816	KARS Q15046$_1$: Lysine--tRNA ligase	1/2	4	4	127	126	4	**1.56**
					129	128	3	**0.42**
					131	130	4	**0.57**

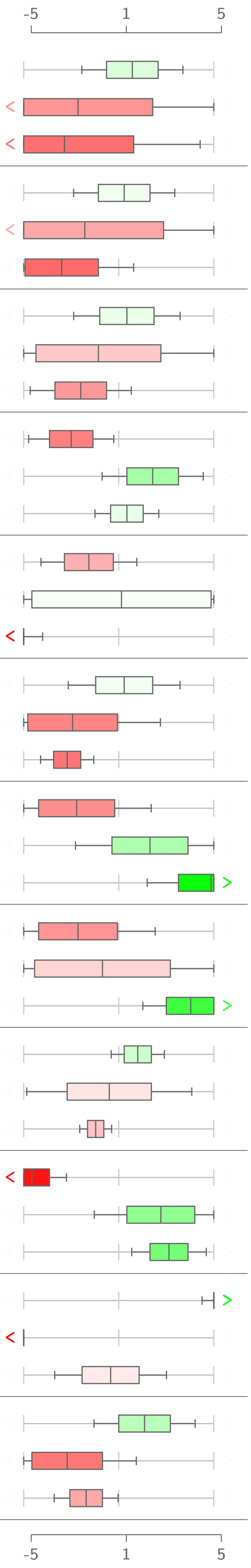

#	protein	group	peptides	spectra	ch1	ch2	quant	ratio
**817**	KAZALD1 Q96I82$_{1,2}$: Kazal-type serine protease inhibitor domain-containing protein 1	2/2	2	2	127	126	2	**0.82**
					129	128	2	**1.13**
					131	130	2	**0.75**
**818**	KCTD12 Q96CX2: BTB/POZ domain-containing protein KCTD12		9	11	127	126	6	**1.01**
					129	128	4	**0.69**
					131	130	10	**0.19**
**819**	KCTD16 Q68DU8: BTB/POZ domain-containing protein KCTD16		4	16	127	126	3	**0.87**
					129	128	3	**0.25**
					131	130	10	**1.54**
**820**	KHDRBS1 Q07666$_{1,2}$: KH domain-containing, RNA-binding, signal transduction-associated protein 1	2/3	7	10	127	126	10	**1.16**
					129	128	10	**1.62**
					131	130	9	**0.76**
**821**	KHSRP Q92945: Far upstream element-binding protein 2		7	8	127	126	5	**0.42**
					129	128	5	**2.27**
					131	130	6	**1.80**
**822**	KIAA0196 Q12768: WASH complex subunit strumpellin		3	3	127	126	2	**1.38**
					129	128	1	**0.46**
					131	130	2	**0.50**
**823**	KIAA0319L Q8IZA0$_{1,2}$: Dyslexia-associated protein KIAA0319-like protein	2/2	4	4	127	126	4	**0.53**
					129	128	3	**0.54**
					131	130	4	**1.14**
**824**	KIAA0355 O15063: Uncharacterized protein KIAA0355		4	12	127	126	4	**1.24**
					129	128	2	**0.22**
					131	130	9	**1.98**
**825**	KIF15 Q9NS87$_{1,2,4}$: Kinesin-like protein KIF15	3/3	2	3	127	126	2	**0.37**
					129	128	1	**0.77**
					131	130	2	**2.22**
**826**	KIF5A Q12840: Kinesin heavy chain isoform 5A		5	5	127	126	1	**2.18**
					129	128		
					131	130	1	**2.55**
**827**	KIF5B P33176: Kinesin-1 heavy chain		16	16	127	126	7	**1.53**
					129	128	10	**0.66**
					131	130	10	**0.61**
**828**	KIF5C O60282$_{1}$: Kinesin heavy chain isoform 5C	1/2	8	8	127	126	1	**0.82**
					129	128	1	**0.62**
					131	130	2	**1.44**

#	protein	group	peptides	spectra	ch1	ch2	quant	ratio
**830**	KLKB1 P03952: Plasma kallikrein	1/2	32	70	127	126	67	**0.55**
					129	128	56	**1.19**
					131	130	65	**1.03**
**831**	KMT2A $Q03164_{1-3}$: Histone-lysine N-methyltransferase 2A	3/3	7	33	127	126	15	**1.80**
					129	128	17	**0.28**
					131	130	24	**1.95**
**832**	KNG1 $P01042_1$: Kininogen-1		32	207	127	126	13	**0.68**
					129	128	12	**1.10**
					131	130	13	**2.01**
**833**	KNG1 $P01042_2$: Kininogen-1	1/2	28	212	127	126	17	**0.82**
					129	128	15	**0.12**
					131	130	17	**0.56**
**834**	KPNA1 P52294: Importin subunit alpha-5		2	2	127	126	1	**1.46**
					129	128	1	**1.32**
					131	130	1	**0.87**
**835**	KPNA4 O00629: Importin subunit alpha-3	1/2	4	6	127	126	6	**0.94**
					129	128	6	**0.46**
					131	130	4	**0.62**
**836**	KPNA6 O60684: Importin subunit alpha-7		2	2	127	126	1	**0.94**
					129	128	1	**1.60**
					131	130	1	**0.60**
**837**	KPNB1 $Q14974_1$: Importin subunit beta-1	1/2	12	17	127	126	14	**0.90**
					129	128	10	**1.34**
					131	130	15	**0.75**
**838**	KPRP Q5T749: Keratinocyte proline-rich protein		6	8	127	126	4	**10.99**
					129	128	6	**0.15**
					131	130	5	**1.71**
**839**	KRT1 P04264: Keratin, type II cytoskeletal 1	1/4	40	164	127	126	106	**12.84**
					129	128	108	**0.08**
					131	130	112	**3.29**
**840**	KRT10 P13645: Keratin, type I cytoskeletal 10	1/5	30	149	127	126	66	**12.91**
					129	128	71	**0.06**
					131	130	74	**3.00**
**841**	KRT13 $P13646_1$: Keratin, type I cytoskeletal 13	1/3	11	43	127	126	1	**0.42**
					129	128	1	**0.66**
					131	130	2	**0.65**

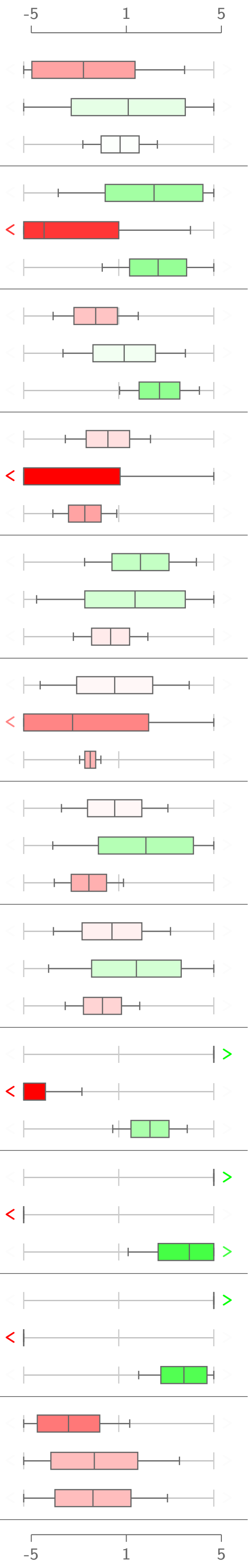

#	protein	group	peptides	spectra	ch1	ch2	quant	ratio
**842**	KRT14 P02533: Keratin, type I cytoskeletal 14		26	115	127	126	3	**11.24**
					129	128	4	**0.11**
					131	130	4	**2.11**
**843**	KRT15 P19012$_1$: Keratin, type I cytoskeletal 15	1/2	12	76	127	126	1	**0.64**
					129	128		
					131	130	4	**1.61**
**844**	KRT16 P08779: Keratin, type I cytoskeletal 16		27	98	127	126	22	**4.94**
					129	128	21	**0.09**
					131	130	20	**3.15**
**845**	KRT17 Q04695: Keratin, type I cytoskeletal 17		22	93	127	126	6	**6.68**
					129	128	5	**0.03**
					131	130	5	**2.49**
**846**	KRT18 P05783: Keratin, type I cytoskeletal 18		33	204	127	126	149	**1.28**
					129	128	130	**1.30**
					131	130	150	**1.18**
**847**	KRT19 P08727: Keratin, type I cytoskeletal 19	1/4	37	290	127	126	152	**1.02**
					129	128	119	**1.24**
					131	130	167	**0.89**
**848**	KRT2 P35908: Keratin, type II cytoskeletal 2 epidermal		35	100	127	126	45	**9.73**
					129	128	46	**0.06**
					131	130	43	**2.56**
**850**	KRT24 Q2M2I5: Keratin, type I cytoskeletal 24		5	30	127	126	3	**2.46**
					129	128	3	**0.31**
					131	130	3	**2.55**
**852**	KRT35 Q92764: Keratin, type I cuticular Ha5		3	33	127	126		
					129	128		
					131	130	2	**2.60**
**854**	KRT5 P13647: Keratin, type II cytoskeletal 5		37	132	127	126	48	**3.28**
					129	128	48	**0.14**
					131	130	53	**0.97**
**855**	KRT6A P02538: Keratin, type II cytoskeletal 6A		32	96	127	126	1	**5.12**
					129	128	1	**0.09**
					131	130	1	**2.82**
**858**	KRT7 P08729: Keratin, type II cytoskeletal 7		36	235	127	126	125	**1.20**
					129	128	77	**0.51**
					131	130	132	**0.65**

#	protein	group	peptides	spectra	ch1	ch2	quant	ratio
**859**	KRT72 Q14CN4$_{1-3}$: Keratin, type II cytoskeletal 72	3/3	6	22	127	126	1	**0.83**
					129	128	1	**18.46**
					131	130	1	**0.86**
**863**	KRT77 Q7Z794: Keratin, type II cytoskeletal 1b		6	32	127	126	1	**13.14**
					129	128	1	**0.10**
					131	130	1	**2.54**
**864**	KRT78 Q8N1N4$_{1,2}$: Keratin, type II cytoskeletal 78	2/2	7	24	127	126	4	**9.06**
					129	128	4	**0.08**
					131	130	4	**2.09**
**865**	KRT79 Q5XKE5: Keratin, type II cytoskeletal 79		9	43	127	126	1	**2.14**
					129	128	1	**1.84**
					131	130	1	**3.13**
**866**	KRT8 P05787$_{1,2}$: Keratin, type II cytoskeletal 8	2/2	42	293	127	126	179	**1.19**
					129	128	132	**1.62**
					131	130	184	**1.11**
**867**	KRT81 Q14533: Keratin, type II cuticular Hb1	1/6	3	9	127	126	2	**3.77**
					129	128	1	**0.06**
					131	130	2	**1.24**
**868**	KRT84 Q9NSB2: Keratin, type II cuticular Hb4		5	43	127	126	2	**1.24**
					129	128	1	**1.37**
					131	130	2	**0.90**
**869**	KRT9 P35527: Keratin, type I cytoskeletal 9		28	108	127	126	68	**13.92**
					129	128	72	**0.10**
					131	130	77	**3.47**
**870**	LAMA2 P24043: Laminin subunit alpha-2		18	24	127	126	16	**0.94**
					129	128	11	**0.29**
					131	130	13	**3.25**
**871**	LAMA4 Q16363$_{1,2}$: Laminin subunit alpha-4	2/2	16	20	127	126	18	**0.68**
					129	128	16	**0.69**
					131	130	17	**1.28**
**872**	LAMA5 O15230$_{1}$: Laminin subunit alpha-5		6	7	127	126	2	**8.13**
					129	128	1	**0.49**
					131	130	3	**1.55**
**873**	LAMB1 P07942: Laminin subunit beta-1		24	30	127	126	26	**0.76**
					129	128	23	**0.62**
					131	130	26	**1.60**

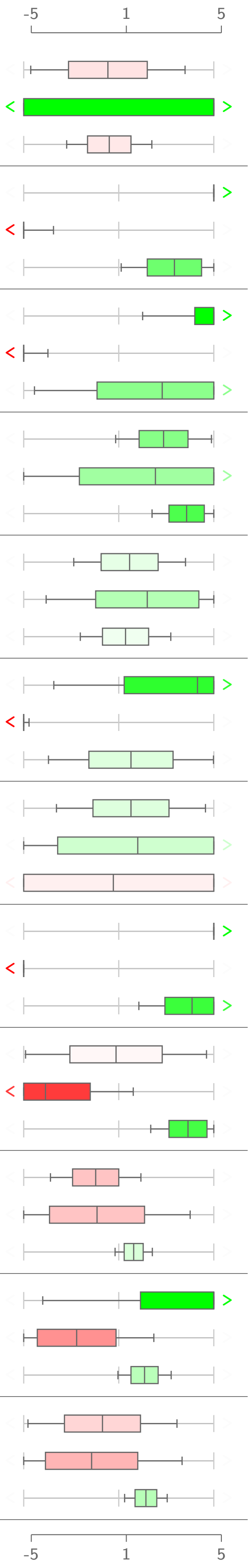

#	protein	group	peptides	spectra	ch1	ch2	quant	ratio
**874**	LAMB2 P55268: Laminin subunit beta-2		11	13	127	126	9	**0.49**
					129	128	7	**0.97**
					131	130	12	**1.52**
**875**	LAMC1 P11047: Laminin subunit gamma-1		34	42	127	126	36	**0.67**
					129	128	25	**0.83**
					131	130	33	**1.72**
**876**	LARS Q9P2J5$_{1,2}$: Leucine--tRNA ligase, cytoplasmic	2/2	4	9	127	126	7	**2.04**
					129	128	3	**0.77**
					131	130	5	**1.71**
**877**	LBP P18428: Lipopolysaccharide-binding protein		14	225	127	126	210	**1.36**
					129	128	167	**0.45**
					131	130	200	**0.35**
**878**	LCAT P04180: Phosphatidylcholine-sterol acyltransferase		10	43	127	126	28	**0.55**
					129	128	22	**2.21**
					131	130	39	**0.99**
**879**	LCN1 P31025: Lipocalin-1	1/2	3	3	127	126	3	**12.06**
					129	128	3	**0.19**
					131	130	3	**0.75**
**880**	LCN2 P80188$_{1,2}$: Neutrophil gelatinase-associated lipocalin	2/2	4	4	127	126	4	**1.12**
					129	128	3	**0.64**
					131	130	4	**0.48**
**881**	LCP1 P13796$_{1}$: Plastin-2	1/2	31	101	127	126	67	**0.96**
					129	128	41	**1.47**
					131	130	60	**0.39**
**882**	LDHA P00338$_{1,3}$: L-lactate dehydrogenase A chain	2/8	4	4	127	126	4	**2.32**
					129	128	4	**0.18**
					131	130	4	**0.68**
**883**	LDLR P01130$_{1,5}$: Low-density lipoprotein receptor	2/5	7	17	127	126	5	**1.70**
					129	128	4	**0.47**
					131	130	8	**2.87**
**884**	LECT2 O14960: Leukocyte cell-derived chemotaxin-2		3	4	127	126	4	**0.71**
					129	128	4	**0.28**
					131	130	4	**2.94**
**885**	LEFTY1 O75610, LEFTY2 O00292$_{1}$: Left-right determination factor 1, Left-right determination factor 2	2/2	3	3	127	126	3	**3.15**
					129	128	2	**0.88**
					131	130	3	**4.02**

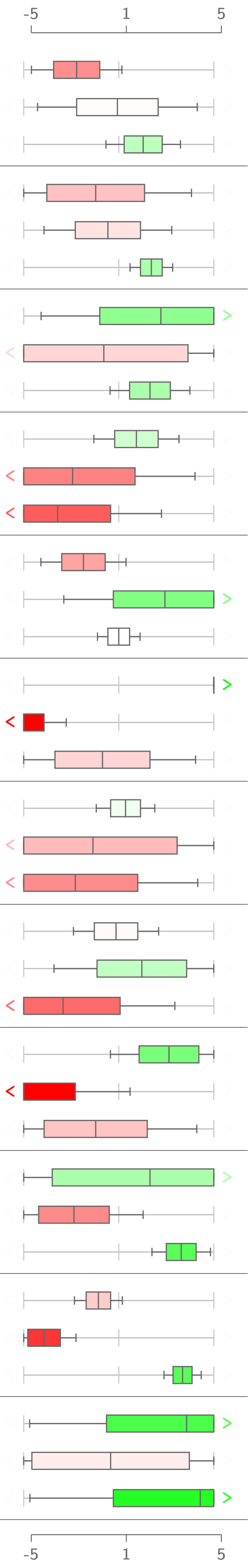

#	protein	group	peptides	spectra	ch1	ch2	quant	ratio
886	LEP P41159: Leptin		2	4	127	126	4	**0.87**
					129	128	2	**0.01**
					131	130	4	**1.75**
887	LFNG $Q8NES3_{1-4}$: Beta-1,3-N-acetylglucosaminyltransferase lunatic fringe	4/4	3	3	127	126	2	**1.05**
					129	128	2	**0.64**
					131	130	3	**0.81**
888	LGALS1 P09382: Galectin-1		8	22	127	126	21	**0.81**
					129	128	20	**1.10**
					131	130	21	**1.32**
889	LGALS3 P17931: Galectin-3		6	10	127	126	8	**0.96**
					129	128	9	**0.44**
					131	130	10	**0.79**
890	LGALS3BP Q08380: Galectin-3-binding protein		18	44	127	126	42	**0.29**
					129	128	34	**0.85**
					131	130	43	**3.59**
891	LGALS4 P56470: Galectin-4		2	3	127	126	3	**1.19**
					129	128	3	**0.46**
					131	130	3	**0.44**
892	LMAN1 P49257: Protein ERGIC-53		9	11	127	126	9	**0.92**
					129	128	8	**0.54**
					131	130	7	**0.83**
893	LMAN2 Q12907: Vesicular integral-membrane protein VIP36		3	3	127	126	3	**1.31**
					129	128	3	**1.06**
					131	130	3	**0.55**
894	LMNA $P02545_{1,2,6}$: Prelamin-A/C [Cleaved into: Lamin-A/C	3/4	29	48	127	126	11	**2.88**
					129	128	11	**0.51**
					131	130	10	**1.09**
895	LMNA $P02545_{4}$: Prelamin-A/C [Cleaved into: Lamin-A/C	1/2	23	38	127	126		
					129	128		
					131	130	1	**0.14**
896	LMNB1 P20700: Lamin-B1		11	12	127	126	4	**1.54**
					129	128	4	**1.66**
					131	130	7	**1.86**
897	LMNB2 Q03252: Lamin-B2		8	8	127	126	2	**2.05**
					129	128	2	**0.49**
					131	130	2	**1.22**

#	protein	group	peptides	spectra	ch1	ch2	quant	ratio
**898**	LOX P28300: Protein-lysine 6-oxidase		10	15	127	126	13	**0.93**
					129	128	13	**0.71**
					131	130	13	**1.85**
**899**	LOXL1 Q08397: Lysyl oxidase homolog 1		13	19	127	126	14	**0.66**
					129	128	16	**0.98**
					131	130	17	**1.92**
**900**	LOXL2 Q9Y4K0: Lysyl oxidase homolog 2		7	8	127	126	6	**0.40**
					129	128	6	**1.80**
					131	130	5	**1.80**
**901**	LOXL3 P58215$_1$: Lysyl oxidase homolog 3		3	4	127	126	2	**1.72**
					129	128	2	**1.01**
					131	130	2	**1.37**
**902**	LPA P08519: Apolipoprotein(a)		36	151	127	126	128	**1.92**
					129	128	78	**0.34**
					131	130	116	**0.48**
**903**	LPAR3 Q9UBY5: Lysophosphatidic acid receptor 3		5	133	127	126	44	**3.10**
					129	128	9	**0.40**
					131	130	97	**3.37**
**904**	LPL P06858: Lipoprotein lipase		8	8	127	126	8	**0.64**
					129	128	4	**1.26**
					131	130	8	**0.85**
**905**	LPP Q93052: Lipoma-preferred partner		2	2	127	126	2	**0.42**
					129	128	2	**0.73**
					131	130	2	**1.62**
**906**	LRBA P50851$_{1,2}$: Lipopolysaccharide-responsive and beige-like anchor protein	2/2	8	8	127	126	7	**1.52**
					129	128	5	**1.16**
					131	130	8	**0.92**
**907**	LRG1 P02750: Leucine-rich alpha-2-glycoprotein		5	5	127	126	4	**0.44**
					129	128	4	**1.77**
					131	130	5	**1.50**
**908**	LRP1 Q07954$_1$: Prolow-density lipoprotein receptor-related protein 1		50	107	127	126	56	**0.60**
					129	128	42	**1.03**
					131	130	81	**2.02**
**909**	LRP1B Q9NZR2: Low-density lipoprotein receptor-related protein 1B		21	97	127	126	16	**1.46**
					129	128	10	**0.32**
					131	130	22	**2.44**

#	protein	group	peptides	spectra	ch1	ch2	quant	ratio
**910**	LRP8 Q14114$_{1,3}$: Low-density lipoprotein receptor-related protein 8	2/3	11	30	127	126	2	**2.32**
					129	128		
					131	130	7	**1.70**
**911**	LRP8 Q14114$_{2}$: Low-density lipoprotein receptor-related protein 8		10	23	127	126		
					129	128		
					131	130	1	**2.86**
**912**	LRRC47 Q8N1G4: Leucine-rich repeat-containing protein 47		3	10	127	126	7	**0.94**
					129	128	6	**0.32**
					131	130	7	**1.43**
**913**	LRRC57 Q8N9N7: Leucine-rich repeat-containing protein 57		4	5	127	126	5	**2.66**
					129	128	3	**0.65**
					131	130	5	**0.32**
**914**	LRRFIP1 Q32MZ4$_{1-3}$: Leucine-rich repeat flightless-interacting protein 1	3/3	2	2	127	126	2	**0.59**
					129	128	2	**0.78**
					131	130	2	**1.94**
**915**	LRRN4 Q8WUT4: Leucine-rich repeat neuronal protein 4		7	8	127	126	6	**1.47**
					129	128	7	**0.79**
					131	130	8	**0.63**
**916**	LSP1 P33241$_{1,3}$: Lymphocyte-specific protein 1	2/2	1	1	127	126		
					129	128	1	**1.75**
					131	130	1	**3.22**
**917**	LSR Q86X29$_{1,4}$: Lipolysis-stimulated lipoprotein receptor	2/6	5	20	127	126	12	**1.37**
					129	128	6	**0.71**
					131	130	18	**1.93**
**918**	LTBP1 Q14766$_{1,4}$: Latent-transforming growth factor beta-binding protein 1	2/4	26	45	127	126	4	**0.56**
					129	128	6	**2.13**
					131	130	5	**1.23**
**919**	LTBP1 Q14766$_{3}$: Latent-transforming growth factor beta-binding protein 1		23	37	127	126	1	**0.24**
					129	128	1	**1.57**
					131	130	1	**2.04**
**920**	LTBP2 Q14767: Latent-transforming growth factor beta-binding protein 2		27	36	127	126	28	**0.73**
					129	128	24	**0.23**
					131	130	30	**1.56**
**921**	LTBP3 Q9NS15$_{1,2}$: Latent-transforming growth factor beta-binding protein 3	2/2	7	7	127	126	3	**0.51**
					129	128	4	**1.54**
					131	130	4	**1.00**

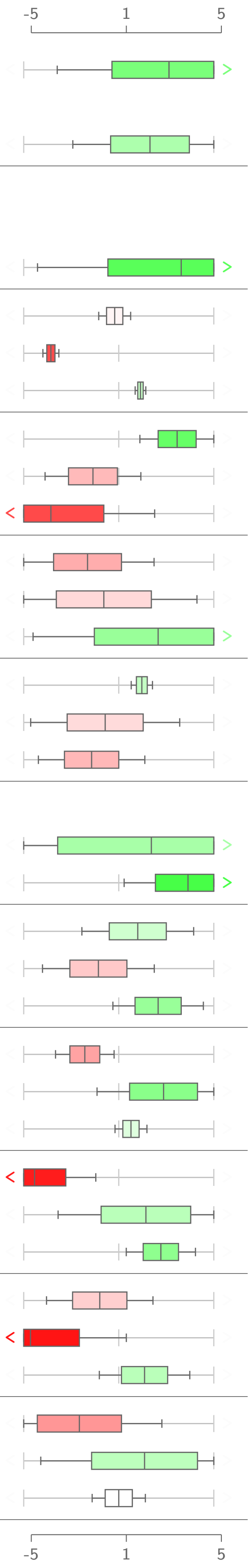

#	protein	group	peptides	spectra	ch1	ch2	quant	ratio
**922**	LTBP4 Q8N2S1$_2$: Latent-transforming growth factor beta-binding protein 4	1/4	31	40	127	126	31	**0.63**
					129	128	32	**0.47**
					131	130	36	**2.31**
**923**	LTF P02788$_{1,2}$: Lactotransferrin	2/2	21	24	127	126	24	**1.45**
					129	128	14	**1.45**
					131	130	24	**1.69**
**924**	LUM P51884: Lumican		14	117	127	126	106	**0.36**
					129	128	96	**1.57**
					131	130	108	**1.23**
**925**	LXN Q9BS40: Latexin		3	3	127	126	3	**1.61**
					129	128	2	**0.41**
					131	130	2	**0.59**
**926**	LYPLA1 O75608$_{1,2}$: Acyl-protein thioesterase 1	2/2	4	5	127	126	3	**1.06**
					129	128	3	**2.40**
					131	130	4	**0.56**
**927**	LYPLA2 O95372: Acyl-protein thioesterase 2		4	4	127	126	4	**0.97**
					129	128	4	**1.08**
					131	130	4	**0.32**
**928**	LYZ P61626: Lysozyme C		12	294	127	126	272	**0.56**
					129	128	258	**0.26**
					131	130	276	**1.05**
**929**	M6PR P20645: Cation-dependent mannose-6-phosphate receptor		2	3	127	126	3	**0.60**
					129	128	3	**2.94**
					131	130	3	**0.95**
**930**	MACF1 Q9UPN3$_1$: Microtubule-actin cross-linking factor 1, isoforms 1/2/3/5	1/5	11	12	127	126	8	**1.67**
					129	128	5	**0.76**
					131	130	10	**1.01**
**931**	MAGED2 Q9UNF1$_{1,2}$: Melanoma-associated antigen D2	2/2	2	2	127	126	2	**1.40**
					129	128	2	**0.34**
					131	130	2	**0.29**
**932**	MAGOH P61326$_1$, MAGOHB Q96A72: Protein mago nashi homolog, Protein mago nashi homolog 2	2/2	2	3	127	126	3	**1.09**
					129	128	3	**0.30**
					131	130	3	**0.89**
**933**	MAMDC2 Q7Z304$_1$: MAM domain-containing protein 2		1	1	127	126	1	**1.24**
					129	128	1	**0.39**
					131	130	1	**1.28**

#	protein	group	peptides	spectra	ch1	ch2	quant	ratio
**934**	MAN1A1 P33908$_1$: Mannosyl-oligosaccharide 1,2-alpha-mannosidase IA	1/2	15	23	127	126	22	**1.12**
					129	128	17	**1.02**
					131	130	22	**0.32**
**935**	MAP1B P46821: Microtubule-associated protein 1B		6	8	127	126	6	**1.62**
					129	128	5	**0.09**
					131	130	4	**1.49**
**936**	MAP1S Q66K74$_{1,2}$: Microtubule-associated protein 1S	2/2	5	5	127	126	5	**2.19**
					129	128	5	**0.46**
					131	130	4	**0.62**
**937**	MAP2K2 P36507: Dual specificity mitogen-activated protein kinase kinase 2		3	3	127	126	3	**1.19**
					129	128	3	**1.44**
					131	130	3	**0.19**
**938**	MAP2K3 P46734$_{1-3}$: Dual specificity mitogen-activated protein kinase kinase 3	3/3	3	3	127	126	3	**0.81**
					129	128	3	**0.78**
					131	130	3	**0.65**
**939**	MAPK1 P28482$_{1,2}$: Mitogen-activated protein kinase 1	2/2	2	2	127	126	1	**1.68**
					129	128	1	**46.87**
					131	130	1	**0.23**
**940**	MAPK3 P27361$_1$: Mitogen-activated protein kinase 3	1/3	5	5	127	126	4	**1.50**
					129	128	3	**0.83**
					131	130	4	**0.71**
**941**	MAPRE1 Q15691: Microtubule-associated protein RP/EB family member 1		4	4	127	126	4	**0.72**
					129	128	3	**0.58**
					131	130	4	**0.99**
**942**	MARCKS P29966: Myristoylated alanine-rich C-kinase substrate		4	4	127	126	3	**0.60**
					129	128	4	**0.57**
					131	130	3	**1.09**
**943**	MARCO Q9UEW3$_{1,2}$: Macrophage receptor MARCO	2/2	2	2	127	126	2	**0.52**
					129	128	2	**1.06**
					131	130	2	**0.83**
**944**	MARS P56192$_1$: Methionine--tRNA ligase, cytoplasmic	1/2	8	11	127	126	10	**1.62**
					129	128	10	**0.63**
					131	130	10	**0.71**
**945**	MASP1 P48740$_1$: Mannan-binding lectin serine protease 1	1/2	14	29	127	126	6	**0.21**
					129	128	4	**3.81**
					131	130	6	**4.95**

#	protein	group	peptides	spectra	ch1	ch2	quant	ratio
946	MASP1 P48740$_{2,4}$: Mannan-binding lectin serine protease 1	2/2	14	27	127	126	4	**0.34**
					129	128	2	**1.43**
					131	130	4	**3.55**
947	MASP2 O00187$_{1}$: Mannan-binding lectin serine protease 2	1/2	9	15	127	126	15	**0.54**
					129	128	15	**1.33**
					131	130	15	**2.96**
948	MAT1A Q00266, MAT2A P31153$_{1,2}$: S-adenosylmethionine synthase isoform type-1, S-adenosylmethionine synthase isoform type-2	3/3	2	3	127	126	3	**1.20**
					129	128	3	**0.77**
					131	130	3	**0.59**
949	MATN2 O00339$_{1-3}$: Matrilin-2	3/4	6	7	127	126	6	**1.03**
					129	128	6	**0.65**
					131	130	7	**0.32**
950	MATR3 P43243$_{1}$: Matrin-3	1/2	18	22	127	126	21	**1.76**
					129	128	21	**0.81**
					131	130	21	**0.66**
951	MB P02144: Myoglobin		5	7	127	126	7	**1.71**
					129	128	6	**0.15**
					131	130	7	**0.64**
952	MCM3 P25205$_{1,2}$: DNA replication licensing factor MCM3	2/2	6	13	127	126	12	**1.31**
					129	128	10	**0.72**
					131	130	13	**1.87**
953	MDH2 P40926$_{1}$: Malate dehydrogenase, mitochondrial		2	2	127	126	2	**1.94**
					129	128	2	**0.72**
					131	130	2	**0.65**
954	MECP2 P51608$_{1,2}$: Methyl-CpG-binding protein 2	2/2	1	1	127	126	1	**1.37**
					129	128	1	**9.05**
					131	130	1	**1.58**
955	MEGF6 O75095$_{1,2}$: Multiple epidermal growth factor-like domains protein 6	2/2	7	7	127	126	4	**0.93**
					129	128	4	**0.09**
					131	130	7	**2.30**
956	MENT Q9BUN1$_{1,2}$: Protein MENT	2/2	1	1	127	126	1	**1.64**
					129	128		
					131	130	1	**0.29**
957	METTL7A Q9H8H3: Methyltransferase-like protein 7A		5	5	127	126	5	**1.26**
					129	128	3	**0.53**
					131	130	3	**0.46**

#	protein	group	peptides	spectra	ch1	ch2	quant	ratio
**958**	MFAP4 P55083$_{1,2}$: Microfibril-associated glycoprotein 4	2/2	7	58	127	126	48	**0.15**
					129	128	55	**1.16**
					131	130	50	**2.99**
**959**	MFAP5 Q13361$_{1}$: Microfibrillar-associated protein 5	1/2	5	11	127	126	11	**0.54**
					129	128	8	**0.83**
					131	130	9	**1.36**
**960**	MFGE8 Q08431$_{1,2}$: Lactadherin	2/3	3	3	127	126	3	**0.92**
					129	128	3	**0.84**
					131	130	3	**0.80**
**961**	MGARP Q8TDB4: Protein MGARP		2	2	127	126	2	**2.76**
					129	128	1	**1.51**
					131	130	2	**0.70**
**962**	MGAT1 P26572: Alpha-1,3-mannosyl-glycoprotein 2-beta-N-acetylglucosaminyltransferase		12	17	127	126	15	**1.08**
					129	128	13	**1.12**
					131	130	17	**0.52**
**963**	MGAT5 Q09328: Alpha-1,6-mannosylglycoprotein 6-beta-N-acetylglucosaminyltransferase A		2	2	127	126	2	**0.82**
					129	128	2	**1.61**
					131	130	2	**0.54**
**964**	MGP P08493$_{1,2}$: Matrix Gla protein	2/2	5	94	127	126	89	**0.32**
					129	128	86	**0.34**
					131	130	84	**1.21**
**965**	MINPP1 Q9UNW1$_{1}$: Multiple inositol polyphosphate phosphatase 1	1/4	9	14	127	126	13	**0.57**
					129	128	7	**1.43**
					131	130	10	**0.39**
**966**	MLEC Q14165: Malectin		5	6	127	126	6	**1.54**
					129	128	5	**0.30**
					131	130	5	**0.91**
**967**	MMP14 P50281: Matrix metalloproteinase-14		3	3	127	126	3	**0.60**
					129	128	3	**0.50**
					131	130	3	**0.89**
**968**	MMP2 P08253$_{1}$: 72 kDa type IV collagenase	1/3	15	18	127	126	16	**0.33**
					129	128	15	**1.29**
					131	130	16	**0.77**
**969**	MMRN2 Q9H8L6: Multimerin-2		4	4	127	126	3	**0.44**
					129	128	2	**0.85**
					131	130	4	**1.88**

#	protein	group	peptides	spectra	ch1	ch2	quant	ratio
**970**	MNDA P41218: Myeloid cell nuclear differentiation antigen		2	3	127	126	3	**1.39**
					129	128	3	**4.47**
					131	130	3	**1.53**
**971**	MOB1A Q9H8S9$_1$, MOB1B Q7L9L4$_{1,2}$: MOB kinase activator 1A, MOB kinase activator 1B	3/3	2	2	127	126	2	**1.70**
					129	128	2	**0.98**
					131	130	2	**0.30**
**972**	MPO P05164$_{1-3}$: Myeloperoxidase	3/3	7	9	127	126	4	**1.01**
					129	128	2	**3.92**
					131	130	4	**1.43**
**973**	MPST P25325$_{1,2}$: 3-mercaptopyruvate sulfurtransferase	2/2	2	2	127	126	2	**0.51**
					129	128	2	**1.58**
					131	130	2	**0.59**
**974**	MRC2 Q9UBG0: C-type mannose receptor 2		3	3	127	126	2	**0.87**
					129	128	2	**0.58**
					131	130	3	**0.77**
**975**	MSLN Q13421$_{2-4}$: Mesothelin	3/4	18	35	127	126	32	**0.59**
					129	128	32	**0.34**
					131	130	33	**0.77**
**976**	MSN P26038: Moesin	1/3	15	16	127	126	10	**1.02**
					129	128	10	**1.08**
					131	130	11	**0.65**
**977**	MST1 P26927: Hepatocyte growth factor-like protein	1/3	9	10	127	126	9	**0.43**
					129	128	8	**2.81**
					131	130	8	**1.36**
**978**	MT1A P04731: Metallothionein-1A		4	23	127	126	4	**0.72**
					129	128	1	**0.63**
					131	130	6	**1.74**
**979**	MTAP Q13126$_{1-7}$: S-methyl-5'-thioadenosine phosphorylase	7/7	2	2	127	126	2	**0.75**
					129	128	2	**2.99**
					131	130	2	**0.35**
**980**	MT-CO2 P00403: Cytochrome c oxidase subunit 2		2	3	127	126	3	**14.37**
					129	128	3	**0.16**
					131	130	3	**0.73**
**981**	MTHFD1 P11586: C-1-tetrahydrofolate synthase, cytoplasmic		13	16	127	126	15	**1.74**
					129	128	15	**0.45**
					131	130	14	**0.66**

#	protein	group	peptides	spectra	ch1	ch2	quant	ratio
982	MVP Q14764: Major vault protein		17	21	127	126	19	1.16
					129	128	15	0.52
					131	130	18	0.81
983	MXRA5 Q9NR99: Matrix-remodeling-associated protein 5		30	78	127	126	64	0.85
					129	128	55	0.76
					131	130	74	2.26
984	MXRA7 $P84157_1$: Matrix-remodeling-associated protein 7	1/2	3	4	127	126		
					129	128		
					131	130	2	0.90
985	MXRA7 $P84157_2$: Matrix-remodeling-associated protein 7		3	3	127	126	1	0.39
					129	128	1	0.43
					131	130	1	3.06
986	MYH10 $P35580_{4,5}$: Myosin-10	2/5	80	125	127	126	66	2.01
					129	128	54	0.37
					131	130	73	0.36
987	MYH11 $P35749_{1-4}$: Myosin-11	4/4	25	44	127	126	1	2.81
					129	128	1	0.77
					131	130	1	0.29
988	MYH14 $Q7Z406_{1,2,6}$: Myosin-14	3/5	15	20	127	126	3	2.96
					129	128	3	0.28
					131	130	4	0.50
989	MYH1 P12882, MYH4 Q9Y623: Myosin-1, Myosin-4	2/2	2	3	127	126	2	0.86
					129	128	2	2.91
					131	130	2	1.36
991	MYH9 $P35579_1$: Myosin-9	1/2	97	185	127	126	127	2.94
					129	128	110	0.40
					131	130	125	0.36
992	MYL1 $P05976_1$: Myosin light chain 1/3, skeletal muscle isoform	1/2	7	10	127	126	8	0.35
					129	128	8	0.37
					131	130	8	1.63
993	MYL12A P19105, MYL12B O14950: Myosin regulatory light chain 12A, Myosin regulatory light chain 12B	2/2	7	15	127	126	4	0.52
					129	128	4	0.57
					131	130	6	1.28
994	MYL2 P10916: Myosin regulatory light chain 2, ventricular/cardiac muscle isoform		7	14	127	126	11	0.39
					129	128	11	0.65
					131	130	14	1.81

#	protein	group	peptides	spectra	ch1	ch2	quant	ratio
**995**	MYL3 P08590: Myosin light chain 3		4	4	127	126	3	**0.41**
					129	128	3	**0.42**
					131	130	3	**1.65**
**996**	MYL6 $P60660_{1,2}$: Myosin light polypeptide 6	2/2	8	17	127	126	9	**0.84**
					129	128	9	**0.97**
					131	130	9	**1.14**
**997**	MYL6B P14649: Myosin light chain 6B		4	7	127	126	1	**0.44**
					129	128		
					131	130	1	**1.13**
**998**	MYL9 $P24844_{1,2}$: Myosin regulatory light polypeptide 9	2/2	6	11	127	126	2	**0.32**
					129	128	2	**0.36**
					131	130	2	**1.63**
**999**	MYLK $Q15746_{1-6}$: Myosin light chain kinase, smooth muscle	6/11	5	21	127	126	9	**0.43**
					129	128	5	**0.66**
					131	130	12	**3.21**
**1000**	MYLPF Q96A32: Myosin regulatory light chain 2, skeletal muscle isoform		5	6	127	126	5	**0.37**
					129	128	4	**0.41**
					131	130	6	**1.62**
**1001**	MYO10 $Q9HD67_{1,3}$: Unconventional myosin-X	2/2	2	2	127	126	1	**0.79**
					129	128	1	**5.56**
					131	130	1	**0.52**
**1002**	MYO18A $Q92614_{1-5}$: Unconventional myosin-XVIIIa	5/5	2	3	127	126	3	**2.49**
					129	128	3	**0.36**
					131	130	3	**0.79**
**1003**	MYO1C $O00159_{1,3}$: Unconventional myosin-Ic	2/3	14	17	127	126	15	**1.11**
					129	128	13	**0.50**
					131	130	16	**0.57**
**1004**	MYOC Q99972: Myocilin		17	22	127	126	20	**0.71**
					129	128	18	**0.39**
					131	130	22	**1.18**
**1005**	N4BP2 $Q86UW6_{2}$: NEDD4-binding protein 2	1/2	5	6	127	126	6	**0.66**
					129	128	4	**0.89**
					131	130	5	**2.01**
**1006**	NAMPT P43490: Nicotinamide phosphoribosyltransferase		6	7	127	126	7	**1.26**
					129	128	6	**0.84**
					131	130	6	**0.31**

#	protein	group	peptides	spectra	ch1	ch2	quant	ratio
**1007**	NANS Q9NR45: Sialic acid synthase		1	1	127	126	1	**1.18**
					129	128	1	**2.14**
					131	130	1	**0.42**
**1008**	NAP1L1 $P55209_{1-3}$: Nucleosome assembly protein 1-like 1	3/3	2	2	127	126	1	**0.41**
					129	128	1	**2.39**
					131	130	1	**1.75**
**1009**	NAP1L4 $Q99733_{1,2}$: Nucleosome assembly protein 1-like 4	2/2	3	3	127	126	2	**1.02**
					129	128	2	**0.81**
					131	130	2	**2.05**
**1010**	NAPA P54920: Alpha-soluble NSF attachment protein		3	4	127	126	3	**0.59**
					129	128	3	**1.61**
					131	130	4	**0.83**
**1011**	NAPRT $Q6XQN6_{1-3}$: Nicotinate phosphoribosyltransferase	3/3	1	1	127	126	1	**0.87**
					129	128	1	**0.96**
					131	130	1	**0.41**
**1012**	NBL1 $P41271_{1,2}$: Neuroblastoma suppressor of tumorigenicity 1	2/2	3	5	127	126	4	**0.23**
					129	128	4	**0.43**
					131	130	3	**2.22**
**1013**	NCBP1 Q09161: Nuclear cap-binding protein subunit 1		2	3	127	126	1	**1.38**
					129	128	1	**0.71**
					131	130	3	**0.39**
**1014**	NCF1 $P14598_{1}$, NCF1B A6NI72, NCF1C A8MVU1: Neutrophil cytosol factor 1, Putative neutrophil cytosol factor 1B, ...	3/3	1	1	127	126	1	**0.98**
					129	128	1	**0.65**
					131	130	1	**0.41**
**1015**	NCKAP1 $Q9Y2A7_{1,2}$: Nck-associated protein 1	2/2	7	7	127	126	6	**1.88**
					129	128	6	**0.27**
					131	130	7	**0.63**
**1016**	NCL P19338: Nucleolin		23	25	127	126	21	**0.91**
					129	128	21	**2.77**
					131	130	22	**1.41**
**1017**	NDRG1 $Q92597_{1}$: Protein NDRG1	1/3	7	10	127	126	7	**1.28**
					129	128	4	**1.57**
					131	130	9	**0.56**
**1018**	NDUFS1 $P28331_{1-5}$: NADH-ubiquinone oxidoreductase 75 kDa subunit, mitochondrial	5/5	3	4	127	126	3	**0.73**
					129	128	2	**1.04**
					131	130	4	**0.40**

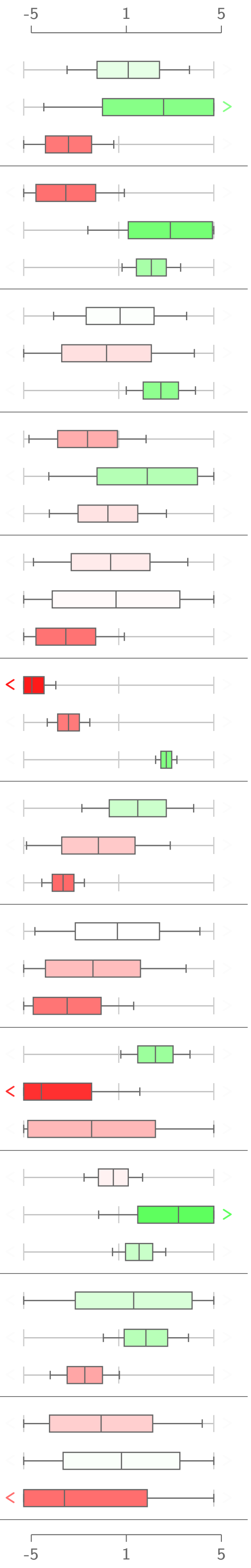

#	protein	group	peptides	spectra	ch1	ch2	quant	ratio
**1019**	NDUFS3 O75489$_{1}$: NADH dehydrogenase [ubiquinone] iron-sulfur protein 3, mitochondrial		2	2	127	126	2	**2.41**
					129	128	2	**0.72**
					131	130	2	**0.47**
**1020**	NDUFV1 P49821$_{1,2}$: NADH dehydrogenase [ubiquinone] flavoprotein 1, mitochondrial	2/2	2	2	127	126	2	**1.98**
					129	128	2	**0.65**
					131	130	2	**0.67**
**1021**	NECAP2 Q9NVZ3$_{1,2,4}$: Adaptin ear-binding coat-associated protein 2	3/4	4	5	127	126	5	**2.24**
					129	128	4	**0.26**
					131	130	4	**0.21**
**1022**	NECTIN2 Q92692$_{1,2}$: Nectin-2	2/2	3	3	127	126	3	**0.90**
					129	128	3	**0.90**
					131	130	3	**0.21**
**1023**	NEDD8 Q15843: NEDD8		2	2	127	126	2	**1.14**
					129	128	2	**0.05**
					131	130	2	**1.47**
**1025**	NEFL P07196: Neurofilament light polypeptide		3	7	127	126	3	**1.57**
					129	128	2	**0.10**
					131	130	3	**0.57**
**1026**	NEU1 Q99519: Sialidase-1		1	1	127	126	1	**0.54**
					129	128	1	**1.94**
					131	130	1	**1.45**
**1027**	NFKB1 P19838$_{1,2}$: Nuclear factor NF-kappa-B p105 subunit	2/3	4	5	127	126	4	**0.85**
					129	128	4	**1.93**
					131	130	5	**0.61**
**1028**	NHLRC2 Q8NBF2$_{1,2}$: NHL repeat-containing protein 2	2/2	1	2	127	126	2	**1.82**
					129	128	2	**0.77**
					131	130	2	**0.64**
**1029**	NID1 P14543$_{1}$: Nidogen-1	1/2	14	17	127	126	15	**0.35**
					129	128	12	**1.49**
					131	130	17	**2.41**
**1030**	NID2 Q14112$_{1,2}$: Nidogen-2	2/2	16	20	127	126	12	**0.87**
					129	128	11	**0.90**
					131	130	15	**1.84**
**1031**	NNMT P40261: Nicotinamide N-methyltransferase		3	4	127	126	3	**1.33**
					129	128	4	**1.17**
					131	130	4	**0.28**

#	protein	group	peptides	spectra	ch1	ch2	quant	ratio
**1032**	NOMO1 Q15155, NOMO2 Q5JPE7$_{1-3}$, NOMO3 P69849: Nodal modulator 1, Nodal modulator 2, . . .	5/5	3	6	127	126	5	**0.81**
					129	128	6	**2.04**
					131	130	6	**1.05**
**1033**	NONO Q15233$_1$: Non-POU domain-containing octamer-binding protein	1/4	12	15	127	126	11	**1.02**
					129	128	11	**2.85**
					131	130	13	**0.80**
**1034**	NOP58 Q9Y2X3: Nucleolar protein 58		2	2	127	126	2	**1.71**
					129	128	2	**0.86**
					131	130	2	**0.71**
**1035**	NOTCH3 Q9UM47: Neurogenic locus notch homolog protein 3		9	11	127	126	7	**0.72**
					129	128	4	**0.94**
					131	130	6	**1.01**
**1036**	NPC2 P61916$_{1,2}$: Epididymal secretory protein E1	2/2	2	2	127	126	2	**1.26**
					129	128	2	**0.29**
					131	130	2	**0.88**
**1037**	NPM1 P06748$_1$: Nucleophosmin	1/3	11	23	127	126	23	**0.78**
					129	128	22	**2.18**
					131	130	22	**1.82**
**1038**	NPPA P01160: Natriuretic peptides A		3	3	127	126	3	**0.38**
					129	128	3	**0.39**
					131	130	3	**1.15**
**1039**	NQO1 P15559$_{1-3}$: NAD(P)H dehydrogenase [quinone] 1	3/3	8	10	127	126	8	**1.54**
					129	128	7	**1.20**
					131	130	8	**0.32**
**1040**	NRP2 O60462$_{1-3}$: Neuropilin-2	3/6	6	7	127	126	3	**0.30**
					129	128	4	**1.06**
					131	130	6	**2.23**
**1041**	NSF P46459$_{1,2}$: Vesicle-fusing ATPase	2/2	7	10	127	126	9	**1.61**
					129	128	8	**0.73**
					131	130	10	**0.46**
**1042**	NUBP2 Q9Y5Y2: Cytosolic Fe-S cluster assembly factor NUBP2		1	1	127	126	1	**1.35**
					129	128		
					131	130	1	**0.42**
**1043**	NUCB1 Q02818: Nucleobindin-1		27	58	127	126	55	**0.39**
					129	128	49	**1.55**
					131	130	54	**2.32**

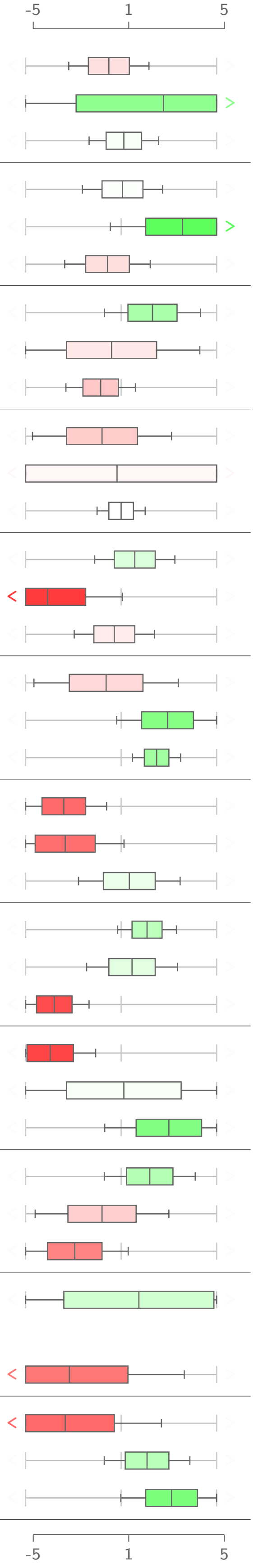

#	protein	group	peptides	spectra	ch1	ch2	quant	ratio
**1044**	NUCB2 P80303$_{1,2}$: Nucleobindin-2	2/2	14	20	127	126	16	**0.65**
					129	128	14	**1.29**
					131	130	19	**2.17**
**1045**	NUCKS1 Q9H1E3$_1$: Nuclear ubiquitous casein and cyclin-dependent kinase substrate 1	1/2	6	8	127	126	4	**0.93**
					129	128	5	**1.73**
					131	130	4	**1.87**
**1046**	NUDCD1 Q96RS6$_{1-3}$: NudC domain-containing protein 1	3/3	4	4	127	126	4	**2.94**
					129	128	3	**1.06**
					131	130	4	**0.58**
**1047**	NUDCD3 Q8IVD9: NudC domain-containing protein 3		2	2	127	126	1	**2.07**
					129	128	1	**0.54**
					131	130	1	**0.28**
**1048**	NUDT21 O43809: Cleavage and polyadenylation specificity factor subunit 5		2	2	127	126	2	**2.02**
					129	128	2	**1.64**
					131	130	2	**0.15**
**1049**	NUMA1 Q14980$_{1-4}$: Nuclear mitotic apparatus protein 1	4/5	27	126	127	126	28	**2.15**
					129	128	21	**0.53**
					131	130	26	**0.47**
**1050**	NUP214 P35658$_{1-5}$: Nuclear pore complex protein Nup214	5/5	2	2	127	126	2	**0.64**
					129	128	2	**1.91**
					131	130	2	**1.50**
**1051**	NUP54 Q7Z3B4$_{1,3}$: Nucleoporin p54	2/2	3	3	127	126	2	**2.76**
					129	128	2	**0.67**
					131	130	2	**0.52**
**1052**	NUP88 Q99567: Nuclear pore complex protein Nup88		2	2	127	126	2	**2.09**
					129	128	1	**1.52**
					131	130	2	**0.71**
**1053**	OAF Q86UD1: Out at first protein homolog		8	18	127	126	17	**0.61**
					129	128	15	**0.91**
					131	130	18	**0.90**
**1054**	OGDH Q02218$_{1-3}$, OGDHL Q9ULD0$_{1-3}$: 2-oxoglutarate dehydrogenase, mitochondrial, 2-oxoglutarate dehydrogenase-like, mitochondrial	6/6	1	1	127	126	1	**1.45**
					129	128	1	**7.69**
					131	130	1	**0.65**
**1055**	OGN P20774: Mimecan		15	88	127	126	69	**0.34**
					129	128	75	**1.96**
					131	130	88	**0.62**

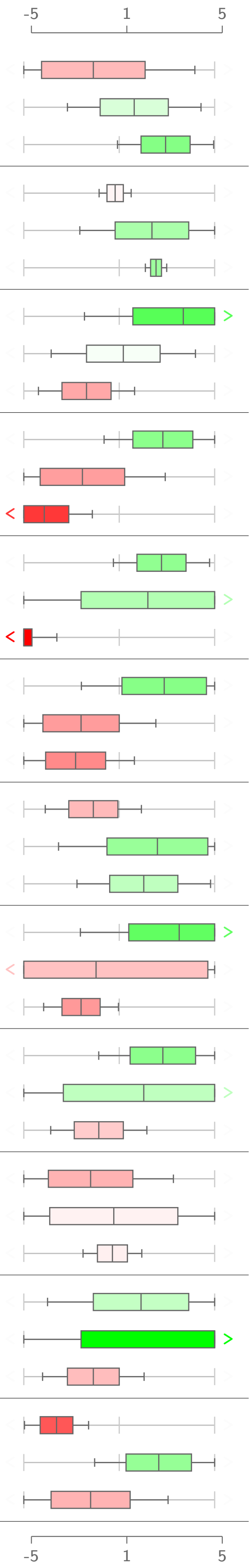

#	protein	group	peptides	spectra	ch1	ch2	quant	ratio
1056	OLA1 Q9NTK5$_1$: Obg-like ATPase 1	1/2	4	4	127	126	3	**1.34**
					129	128	4	**0.60**
					131	130	4	**0.13**
1057	OLFML1 Q6UWY5: Olfactomedin-like protein 1		11	17	127	126	14	**0.28**
					129	128	9	**1.54**
					131	130	15	**1.89**
1058	OLFML3 Q9NRN5$_1$: Olfactomedin-like protein 3	1/3	17	43	127	126	36	**0.34**
					129	128	34	**1.38**
					131	130	40	**1.51**
1059	OMD Q99983: Osteomodulin		3	4	127	126	4	**0.35**
					129	128	4	**1.37**
					131	130	4	**2.26**
1060	OPLAH O14841: 5-oxoprolinase		3	4	127	126	3	**1.73**
					129	128	3	**2.22**
					131	130	3	**1.10**
1062	ORM1 P02763: Alpha-1-acid glycoprotein 1		8	17	127	126	7	**1.16**
					129	128	6	**1.18**
					131	130	7	**1.01**
1063	ORM2 P19652: Alpha-1-acid glycoprotein 2		6	13	127	126	3	**0.52**
					129	128	3	**1.30**
					131	130	4	**0.82**
1064	OSBP P22059: Oxysterol-binding protein 1		3	3	127	126	3	**1.47**
					129	128	2	**0.35**
					131	130	3	**0.56**
1065	OTUB1 Q96FW1$_1$: Ubiquitin thioesterase OTUB1		4	4	127	126	4	**0.87**
					129	128	3	**0.66**
					131	130	4	**0.90**
1066	OXSR1 O95747: Serine/threonine-protein kinase OSR1		6	6	127	126	6	**2.34**
					129	128	4	**0.26**
					131	130	6	**0.83**
1067	P04220: Ig mu heavy chain disease protein		14	79	127	126	15	**0.44**
					129	128	8	**1.56**
					131	130	12	**3.49**
1068	P3H1 Q32P28$_{1,3,4}$: Prolyl 3-hydroxylase 1	3/3	3	4	127	126	4	**1.13**
					129	128	3	**1.38**
					131	130	3	**1.94**

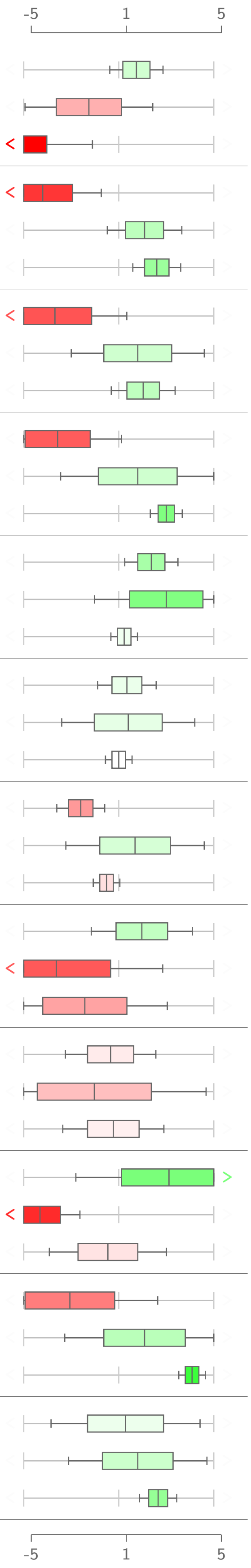

#	protein	group	peptides	spectra	ch1	ch2	quant	ratio
**1069**	P4HA1 P13674$_{1-3}$: Prolyl 4-hydroxylase subunit alpha-1	3/3	2	2	127	126	2	**0.26**
					129	128	2	**1.11**
					131	130	2	**0.90**
**1070**	P4HA2 O15460$_{1,2}$: Prolyl 4-hydroxylase subunit alpha-2	2/2	2	2	127	126	2	**1.14**
					129	128	2	**0.70**
					131	130	2	**1.26**
**1071**	P4HB P07237: Protein disulfide-isomerase		30	42	127	126	38	**0.49**
					129	128	32	**2.75**
					131	130	37	**2.20**
**1072**	PABPC1 P11940$_{1}$: Polyadenylate-binding protein 1	1/6	12	14	127	126	13	**1.59**
					129	128	10	**1.00**
					131	130	12	**0.60**
**1073**	PACSIN2 Q9UNF0$_{1,2}$: Protein kinase C and casein kinase substrate in neurons protein 2	2/2	6	10	127	126	3	**0.57**
					129	128	4	**3.83**
					131	130	8	**1.39**
**1074**	PAFAH1B3 Q15102: Platelet-activating factor acetylhydrolase IB subunit gamma		3	3	127	126	3	**1.90**
					129	128	3	**0.28**
					131	130	3	**0.53**
**1075**	PAICS P22234$_{1,2}$: Multifunctional protein ADE2 [Includes: Phosphoribosylaminoimidazole-succino . . .	2/2	4	4	127	126	4	**0.98**
					129	128	3	**0.61**
					131	130	3	**0.48**
**1076**	PAK2 Q13177: Serine/threonine-protein kinase PAK 2		2	2	127	126	2	**0.83**
					129	128	1	**1.28**
					131	130	2	**0.49**
**1077**	PAM P19021$_{1,3,5}$: Peptidyl-glycine alpha-amidating monooxygenase	3/6	18	27	127	126	21	**0.43**
					129	128	20	**0.34**
					131	130	23	**0.79**
**1079**	PAMR1 Q6UXH9$_{2}$: Inactive serine protease PAMR1		10	19	127	126	1	**0.34**
					129	128	1	**2.69**
					131	130	1	**1.27**
**1081**	PAPLN O95428$_{1,5,6}$: Papilin	3/6	21	27	127	126	24	**0.80**
					129	128	21	**0.64**
					131	130	27	**1.44**
**1082**	PAPSS2 O95340$_{1,2}$: Bifunctional 3'-phosphoadenosine 5'-phosphosulfate synthase 2	2/3	5	5	127	126	4	**0.87**
					129	128	4	**0.55**
					131	130	5	**0.55**

#	protein	group	peptides	spectra	ch1	ch2	quant	ratio
**1083**	PARK2 O60260$_{1,2,4,6-8}$: E3 ubiquitin-protein ligase parkin	6/6	4	59	127	126	2	**0.58**
					129	128	1	**1.90**
					131	130	5	**1.64**
**1084**	PARP2 Q9UGN5$_{1,2}$: Poly [ADP-ribose] polymerase 2	2/2	6	15	127	126	4	**0.72**
					129	128	1	**0.27**
					131	130	7	**2.40**
**1085**	PARVA Q9NVD7$_{1}$: Alpha-parvin	1/5	7	10	127	126	9	**1.77**
					129	128	9	**0.55**
					131	130	10	**0.35**
**1086**	PBXIP1 Q96AQ6$_{1,2}$: Pre-B-cell leukemia transcription factor-interacting protein 1	2/2	2	3	127	126	3	**1.76**
					129	128	3	**0.19**
					131	130	3	**1.72**
**1087**	PCBP1 Q15365: Poly(rC)-binding protein 1	1/6	9	14	127	126	3	**1.56**
					129	128	4	**1.47**
					131	130	5	**0.59**
**1088**	PCBP2 Q15366$_{1-8}$: Poly(rC)-binding protein 2	8/8	8	14	127	126	4	**1.19**
					129	128	5	**0.64**
					131	130	5	**0.65**
**1089**	PCGF5 Q86SE9$_{1}$: Polycomb group RING finger protein 5		2	8	127	126	2	**1.50**
					129	128		
					131	130	6	**2.63**
**1090**	PCMT1 P22061$_{1,2}$: Protein-L-isoaspartate(D-aspartate) O-methyltransferase	2/2	5	8	127	126	6	**1.24**
					129	128	8	**1.51**
					131	130	8	**0.40**
**1091**	PCNT O95613$_{1,2}$: Pericentrin	2/2	3	5	127	126	3	**5.45**
					129	128	4	**0.26**
					131	130	4	**2.36**
**1092**	PCOLCE Q15113: Procollagen C-endopeptidase enhancer 1		22	107	127	126	96	**0.40**
					129	128	91	**1.47**
					131	130	100	**1.92**
**1093**	PCOLCE2 Q9UKZ9: Procollagen C-endopeptidase enhancer 2		11	21	127	126	20	**0.73**
					129	128	19	**0.95**
					131	130	18	**0.79**
**1094**	PCSK1N Q9UHG2: ProSAAS		5	12	127	126	10	**0.42**
					129	128	10	**0.49**
					131	130	12	**2.11**

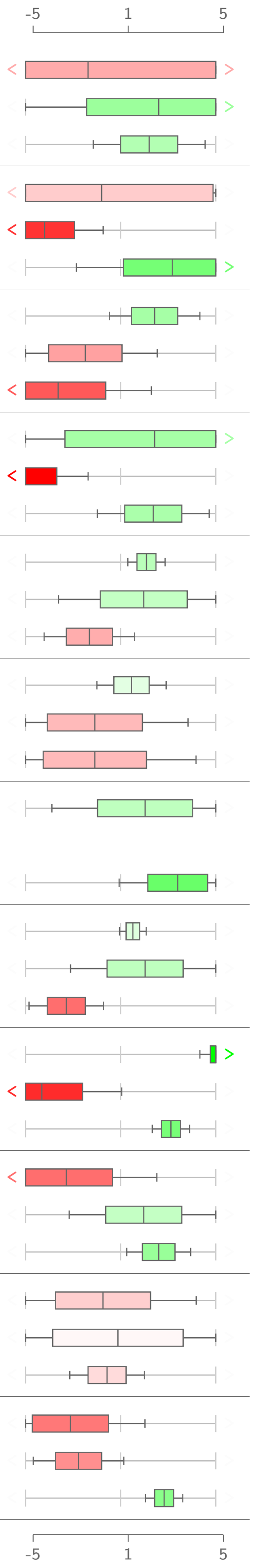

#	protein	group	peptides	spectra	ch1	ch2	quant	ratio
**1095**	PCSK5 Q92824$_1$: Proprotein convertase subtilisin/kexin type 5	1/2	19	115	127	126	30	**1.02**
					129	128	21	**0.60**
					131	130	65	**1.71**
**1096**	PCSK9 Q8NBP7$_1$: Proprotein convertase subtilisin/kexin type 9	1/2	13	22	127	126	19	**0.42**
					129	128	15	**0.81**
					131	130	22	**1.65**
**1097**	PCYOX1 Q9UHG3$_1$: Prenylcysteine oxidase 1	1/2	11	15	127	126	13	**0.76**
					129	128	12	**2.16**
					131	130	15	**0.71**
**1098**	PDCD4 Q53EL6$_{1,2}$: Programmed cell death protein 4	2/2	4	4	127	126	4	**1.78**
					129	128	4	**0.45**
					131	130	4	**0.59**
**1099**	PDCD6IP Q8WUM4$_{1,2}$: Programmed cell death 6-interacting protein	2/3	20	25	127	126	21	**1.23**
					129	128	20	**0.68**
					131	130	21	**0.27**
**1100**	PDGFB P01127$_{1,2}$: Platelet-derived growth factor subunit B	2/2	3	4	127	126	3	**1.88**
					129	128	3	**0.06**
					131	130	3	**2.42**
**1101**	PDGFC Q9NRA1$_1$: Platelet-derived growth factor C	1/3	5	8	127	126	5	**0.58**
					129	128	3	**0.84**
					131	130	6	**1.68**
**1102**	PDGFRL Q15198: Platelet-derived growth factor receptor-like protein		8	8	127	126	6	**0.37**
					129	128	4	**3.11**
					131	130	6	**1.13**
**1103**	PDIA3 P30101: Protein disulfide-isomerase A3		11	12	127	126	11	**1.17**
					129	128	9	**1.28**
					131	130	10	**1.19**
**1104**	PDIA4 P13667: Protein disulfide-isomerase A4		17	20	127	126	16	**0.78**
					129	128	14	**2.20**
					131	130	15	**2.50**
**1105**	PDIA6 Q15084$_5$: Protein disulfide-isomerase A6	1/5	16	23	127	126	22	**1.20**
					129	128	17	**1.76**
					131	130	23	**0.52**
**1106**	PDLIM5 Q96HC4$_{1,4,6,7}$: PDZ and LIM domain protein 5	4/6	5	5	127	126	5	**0.89**
					129	128	4	**1.17**
					131	130	5	**0.68**

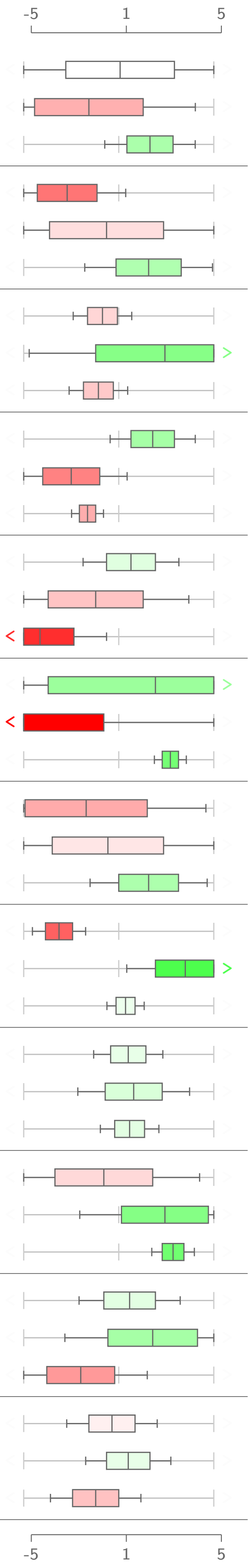

#	protein	group	peptides	spectra	ch1	ch2	quant	ratio
**1107**	PENK P01210: Proenkephalin-A [Cleaved into: Synenkephalin; Met-enkephalin		8	9	127	126	8	**0.38**
					129	128	8	**0.85**
					131	130	9	**1.60**
**1108**	PFKL P17858$_{1,2}$: ATP-dependent 6-phosphofructokinase, liver type	2/4	7	7	127	126	5	**1.31**
					129	128	6	**0.57**
					131	130	6	**0.75**
**1109**	PFKM P08237$_{1,3}$: ATP-dependent 6-phosphofructokinase, muscle type	2/3	8	11	127	126	10	**1.01**
					129	128	8	**0.42**
					131	130	10	**0.72**
**1110**	PFN1 P07737: Profilin-1		7	15	127	126	15	**1.37**
					129	128	8	**0.52**
					131	130	14	**0.35**
**1111**	PFN2 P35080$_{1,2}$: Profilin-2	2/2	2	3	127	126	3	**0.50**
					129	128	2	**0.78**
					131	130	3	**0.81**
**1112**	PGA3 P0DJD8, PGA4 P0DJD7, PGA5 P0DJD9: Pepsin A-3, Pepsin A-4, . . .	3/3	3	4	127	126	3	**0.96**
					129	128	3	**1.28**
					131	130	3	**1.53**
**1113**	PGAM1 P18669: Phosphoglycerate mutase 1	1/2	4	5	127	126	4	**1.41**
					129	128	5	**0.81**
					131	130	5	**0.17**
**1114**	PGD P52209$_{1,2}$: 6-phosphogluconate dehydrogenase, decarboxylating	2/2	8	10	127	126	7	**1.18**
					129	128	9	**0.55**
					131	130	9	**0.17**
**1115**	PGK1 P00558$_{1,2}$, PGK2 P07205: Phosphoglycerate kinase 1, Phosphoglycerate kinase 2	3/3	2	2	127	126	2	**1.49**
					129	128	2	**0.34**
					131	130	2	**0.51**
**1116**	PGLS O95336: 6-phosphogluconolactonase		3	3	127	126	3	**0.96**
					129	128	3	**0.61**
					131	130	3	**0.27**
**1117**	PGLYRP2 Q96PD5$_{1,2}$: N-acetylmuramoyl-L-alanine amidase	2/2	9	45	127	126	43	**0.97**
					129	128	31	**0.74**
					131	130	42	**0.34**
**1118**	PGM1 P36871$_{1}$: Phosphoglucomutase-1	1/3	10	11	127	126	11	**1.64**
					129	128	10	**0.69**
					131	130	9	**0.26**

#	protein	group	peptides	spectra	ch1	ch2	quant	ratio
**1119**	PGM2 Q96G03$_1$: Phosphoglucomutase-2		9	11	127	126	11	**1.44**
					129	128	8	**0.88**
					131	130	9	**0.23**
**1120**	PGM3 O95394$_{1,3,4}$: Phosphoacetylglucosamine mutase	3/3	3	3	127	126	3	**1.04**
					129	128	3	**2.59**
					131	130	3	**0.38**
**1121**	PGRMC1 O00264$_{1,2}$: Membrane-associated progesterone receptor component 1	2/2	2	3	127	126	3	**0.65**
					129	128	3	**1.11**
					131	130	3	**0.76**
**1122**	PHB P35232$_1$: Prohibitin	1/2	7	7	127	126	7	**0.67**
					129	128	3	**2.36**
					131	130	7	**0.50**
**1123**	PHB2 Q99623$_{1,2}$: Prohibitin-2	2/2	5	5	127	126	5	**2.31**
					129	128	4	**0.45**
					131	130	5	**0.53**
**1124**	PHGDH O43175: D-3-phosphoglycerate dehydrogenase		18	38	127	126	37	**1.09**
					129	128	31	**1.12**
					131	130	38	**0.42**
**1125**	PICALM Q13492$_{1-5}$: Phosphatidylinositol-binding clathrin assembly protein	5/5	3	3	127	126	2	**2.04**
					129	128	3	**0.60**
					131	130	3	**0.34**
**1126**	PIGR P01833: Polymeric immunoglobulin receptor		15	18	127	126	18	**10.39**
					129	128	16	**0.50**
					131	130	15	**2.52**
**1127**	PIK3CG P48736: Phosphatidylinositol 4,5-bisphosphate 3-kinase catalytic subunit gamma isoform		2	7	127	126	1	**1.45**
					129	128	1	**0.72**
					131	130	1	**0.67**
**1128**	PITPNA Q00169: Phosphatidylinositol transfer protein alpha isoform		10	16	127	126	10	**1.05**
					129	128	9	**0.75**
					131	130	14	**0.37**
**1129**	PITPNB P48739$_{1-3}$: Phosphatidylinositol transfer protein beta isoform	3/3	2	3	127	126	1	**0.81**
					129	128	1	**0.12**
					131	130	1	**0.14**
**1130**	PKDCC Q504Y2: Extracellular tyrosine-protein kinase PKDCC		3	3	127	126	3	**2.08**
					129	128	3	**0.37**
					131	130	3	**0.69**

#	protein	group	peptides	spectra	ch1	ch2	quant	ratio
**1131**	PKM P14618$_1$: Pyruvate kinase PKM	1/5	31	63	127	126	57	**0.91**
					129	128	47	**1.66**
					131	130	62	**0.42**
**1132**	PKN1 Q16512$_{1,2}$, PKN2 Q16513$_{1-3}$: Serine/threonine-protein kinase N1, Serine/threonine-protein kinase N2	5/7	5	5	127	126	5	**1.59**
					129	128	5	**0.67**
					131	130	5	**0.54**
**1133**	PLA2G12A Q9BZM1: Group XIIA secretory phospholipase A2		3	42	127	126	19	**1.34**
					129	128	12	**0.39**
					131	130	28	**1.08**
**1134**	PLA2G2A P14555: Phospholipase A2, membrane associated		12	38	127	126	29	**1.17**
					129	128	24	**0.34**
					131	130	30	**2.30**
**1135**	PLA2G4A P47712: Cytosolic phospholipase A2		5	6	127	126	6	**0.97**
					129	128	3	**0.70**
					131	130	6	**0.76**
**1136**	PLAT P00750$_1$: Tissue-type plasminogen activator	1/4	13	16	127	126	14	**0.49**
					129	128	14	**1.71**
					131	130	16	**2.93**
**1137**	PLCD1 P51178$_{1,2}$: 1-phosphatidylinositol 4,5-bisphosphate phosphodiesterase delta-1	2/2	3	3	127	126	2	**1.82**
					129	128	2	**0.06**
					131	130	2	**1.31**
**1138**	PLEC Q15149$_1$: Plectin	1/9	180	233	127	126	206	**1.71**
					129	128	185	**0.73**
					131	130	215	**0.51**
**1139**	PLG P00747: Plasminogen	1/2	51	678	127	126	492	**0.58**
					129	128	373	**1.69**
					131	130	581	**1.61**
**1140**	PLGLB1 Q02325: Plasminogen-like protein B		5	44	127	126	1	**6.25**
					129	128		
					131	130	1	**2.29**
**1141**	PLIN3 O60664$_{1,3}$: Perilipin-3	2/4	10	10	127	126	10	**1.38**
					129	128	9	**0.80**
					131	130	9	**0.67**
**1142**	PLIN4 Q96Q06$_{1,2}$: Perilipin-4	2/2	2	2	127	126	2	**1.27**
					129	128	2	**0.38**
					131	130	2	**0.32**

#	protein	group	peptides	spectra	ch1	ch2	quant	ratio
1143	PLOD1 Q02809$_{1,2}$: Procollagen-lysine,2-oxoglutarate 5-dioxygenase 1	2/2	4	4	127	126	3	**0.73**
					129	128	4	**1.24**
					131	130	4	**2.04**
1144	PLOD3 O60568: Procollagen-lysine,2-oxoglutarate 5-dioxygenase 3		8	8	127	126	7	**0.50**
					129	128	7	**1.29**
					131	130	6	**2.48**
1145	PLS1 Q14651: Plastin-1		5	9	127	126	1	**1.06**
					129	128	1	**2.06**
					131	130	1	**1.44**
1146	PLS3 P13797$_{1}$: Plastin-3	1/3	23	48	127	126	21	**1.43**
					129	128	21	**0.47**
					131	130	19	**0.32**
1147	PLTP P55058$_{1}$: Phospholipid transfer protein	1/4	17	231	127	126	202	**1.12**
					129	128	173	**0.70**
					131	130	225	**0.36**
1148	PLXDC2 Q6UX71$_{1,2}$: Plexin domain-containing protein 2	2/2	4	5	127	126	5	**1.12**
					129	128	5	**0.51**
					131	130	5	**1.11**
1149	PML P29590$_{1-5,8,9,11-13}$: Protein PML	10/10	2	2	127	126	2	**1.59**
					129	128	2	**0.43**
					131	130	2	**0.56**
1150	PMVK Q15126: Phosphomevalonate kinase		3	3	127	126	3	**1.41**
					129	128	3	**0.56**
					131	130	3	**0.52**
1151	PODN Q7Z5L7$_{1-3}$: Podocan	3/4	16	22	127	126	19	**0.86**
					129	128	17	**0.75**
					131	130	22	**0.80**
1152	PODXL O00592$_{1,2}$: Podocalyxin	2/2	2	2	127	126	2	**3.26**
					129	128	2	**0.16**
					131	130	2	**0.15**
1153	POGZ Q7Z3K3$_{1-3,5-7}$: Pogo transposable element with ZNF domain	6/6	4	26	127	126	10	**1.18**
					129	128	4	**1.11**
					131	130	16	**3.31**
1154	POMGNT1 Q8WZA1$_{1,2}$: Protein O-linked-mannose beta-1,2-N-acetylglucosaminyltransferase 1	2/2	3	3	127	126	3	**1.40**
					129	128	2	**0.56**
					131	130	3	**0.77**

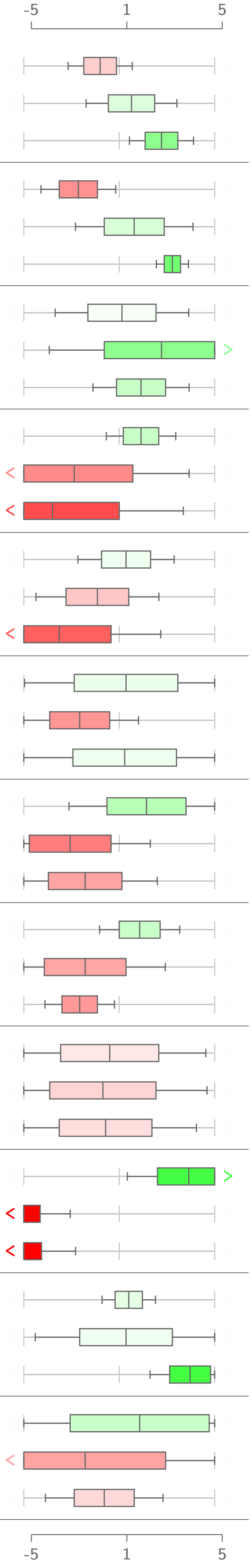

#	protein	group	peptides	spectra	ch1	ch2	quant	ratio
**1155**	PON1 P27169: Serum paraoxonase/arylesterase 1	1/4	14	125	127	126	99	**0.39**
					129	128	86	**2.42**
					131	130	109	**1.80**
**1156**	PON3 Q15166: Serum paraoxonase/lactonase 3		7	20	127	126	4	**0.61**
					129	128	5	**1.38**
					131	130	6	**1.07**
**1157**	POSTN $Q15063_1$: Periostin		35	111	127	126	2	**0.96**
					129	128	2	**0.80**
					131	130	2	**0.19**
**1164**	POTEE $Q6S8J3_1$: POTE ankyrin domain family member E	1/4	19	215	127	126	19	**1.41**
					129	128	9	**0.40**
					131	130	34	**2.96**
**1165**	POTEF A5A3E0: POTE ankyrin domain family member F		14	152	127	126	1	**1.26**
					129	128		
					131	130	9	**1.84**
**1166**	POTEH $Q6S545_{1,2}$: POTE ankyrin domain family member H	2/2	8	69	127	126	4	**0.95**
					129	128	2	**0.47**
					131	130	11	**2.59**
**1168**	PPA1 Q15181: Inorganic pyrophosphatase		3	4	127	126	4	**0.82**
					129	128	4	**1.96**
					131	130	4	**0.31**
**1169**	PPCS $Q9HAB8_1$: Phosphopantothenate--cysteine ligase	1/2	3	3	127	126	3	**3.90**
					129	128	1	**5.37**
					131	130	3	**0.13**
**1170**	PPIA $P62937_1$: Peptidyl-prolyl cis-trans isomerase A	1/7	5	5	127	126	4	**1.05**
					129	128	5	**1.26**
					131	130	5	**1.32**
**1171**	PPIB P23284: Peptidyl-prolyl cis-trans isomerase B		5	5	127	126	5	**1.20**
					129	128	3	**0.88**
					131	130	5	**0.87**
**1172**	PPL O60437: Periplakin		10	12	127	126	9	**1.84**
					129	128	7	**0.82**
					131	130	9	**0.88**
**1173**	PPME1 $Q9Y570_{1,4}$: Protein phosphatase methylesterase 1	2/2	3	3	127	126	3	**1.47**
					129	128	3	**0.83**
					131	130	3	**0.38**

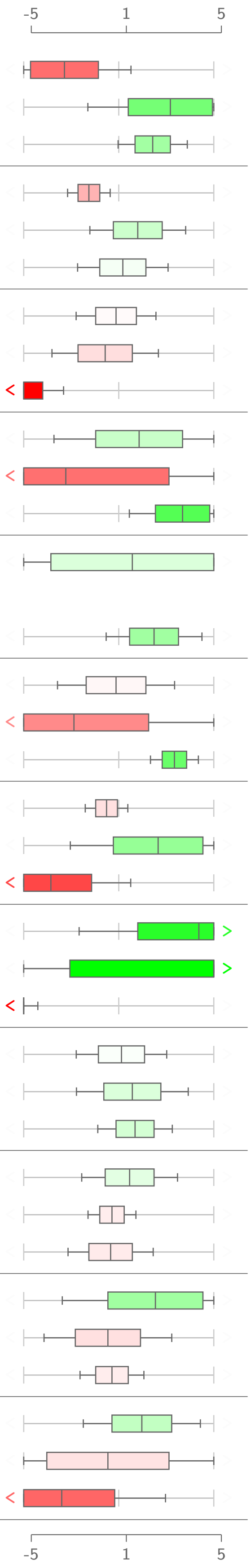

#	protein	group	peptides	spectra	ch1	ch2	quant	ratio
**1174**	PPP1CB P62140: Serine/threonine-protein phosphatase PP1-beta catalytic subunit	1/6	4	4	127	126	4	**0.57**
					129	128	4	**1.46**
					131	130	3	**1.22**
**1175**	PPP1R7 Q15435$_{1,2}$: Protein phosphatase 1 regulatory subunit 7	2/5	4	4	127	126	4	**0.47**
					129	128	4	**1.42**
					131	130	4	**1.08**
**1176**	PPP2CA P67775$_{1,2}$, PPP2CB P62714: Serine/threonine-protein phosphatase 2A catalytic subunit alpha isoform, Serine/threonine-protein phosphatase 2A catalytic subunit beta isoform	3/3	1	2	127	126	2	**0.66**
					129	128	2	**0.60**
					131	130	2	**1.94**
**1177**	PPP2R1A P30153: Serine/threonine-protein phosphatase 2A 65 kDa regulatory subunit A alpha is . . .	1/6	19	25	127	126	22	**0.81**
					129	128	23	**0.26**
					131	130	21	**0.61**
**1178**	PPP2R2A P63151$_{1,2}$: Serine/threonine-protein phosphatase 2A 55 kDa regulatory subunit B alpha is . . .	2/2	4	5	127	126	5	**1.14**
					129	128	4	**0.92**
					131	130	4	**1.03**
**1179**	PPP2R4 Q15257$_{1-4}$: Serine/threonine-protein phosphatase 2A activator	4/4	2	2	127	126	2	**1.10**
					129	128	2	**1.11**
					131	130	2	**0.65**
**1180**	PPP2R5D Q14738$_{1,2}$: Serine/threonine-protein phosphatase 2A 56 kDa regulatory subunit delta isoform	2/2	2	2	127	126	2	**1.10**
					129	128	1	**1.69**
					131	130	2	**0.15**
**1181**	PPP3CA Q08209$_{1-3,5}$, PPP3CB P16298$_{1-4}$, PPP3CC P48454$_{1-3}$: Serine/threonine-protein phosphatase 2B catalytic subunit alpha isoform, Serine/threonine-protein phosphatase 2B catalytic subunit beta isoform, . . .	11/11	1	1	127	126	1	**0.89**
					129	128	1	**1.58**
					131	130	1	**1.29**
**1182**	PPP4R3B Q5MIZ7$_{1-3}$: Serine/threonine-protein phosphatase 4 regulatory subunit 3B	3/4	3	8	127	126	1	**0.69**
					129	128	1	**2.15**
					131	130	3	**1.13**
**1183**	PPP6C O00743$_{1-3}$: Serine/threonine-protein phosphatase 6 catalytic subunit	3/3	5	5	127	126	3	**1.01**
					129	128	4	**0.62**
					131	130	5	**1.47**
**1184**	PRAP1 Q96NZ9$_{1}$: Proline-rich acidic protein 1		2	2	127	126	2	**0.54**
					129	128	2	**0.19**
					131	130	2	**2.01**
**1185**	PRDM10 Q9NQV6$_{6}$: PR domain zinc finger protein 10		5	9	127	126	2	**1.80**
					129	128	1	**0.29**
					131	130	5	**0.75**

#	protein	group	peptides	spectra	ch1	ch2	quant	ratio
1186	PRDX1 Q06830: Peroxiredoxin-1		7	9	127	126	8	**1.54**
					129	128	7	**0.83**
					131	130	7	**0.20**
1187	PRDX2 P32119$_1$: Peroxiredoxin-2	1/2	6	7	127	126	5	**1.68**
					129	128	6	**0.60**
					131	130	5	**0.34**
1188	PRDX3 P30048$_{1,2}$: Thioredoxin-dependent peroxide reductase, mitochondrial	2/2	2	2	127	126	2	**0.96**
					129	128	2	**4.71**
					131	130	2	**0.76**
1189	PRDX5 P30044$_{1-3}$: Peroxiredoxin-5, mitochondrial	3/4	2	2	127	126	2	**1.09**
					129	128	2	**1.50**
					131	130	2	**0.55**
1190	PRDX6 P30041: Peroxiredoxin-6		11	38	127	126	36	**2.11**
					129	128	28	**0.90**
					131	130	33	**0.31**
1191	PRELP P51888: Prolargin		9	12	127	126	12	**0.55**
					129	128	11	**0.69**
					131	130	12	**0.57**
1192	PRG2 P13727$_{1,2}$: Bone marrow proteoglycan	2/2	2	4	127	126	4	**1.20**
					129	128	3	**1.54**
					131	130	4	**1.08**
1193	PRG3 Q9Y2Y8: Proteoglycan 3		2	2	127	126	1	**0.68**
					129	128	1	**2.53**
					131	130	2	**2.07**
1194	PRG4 Q92954$_3$: Proteoglycan 4	1/2	51	304	127	126	4	**3.64**
					129	128	4	**0.35**
					131	130	6	**2.23**
1195	PRG4 Q92954$_6$: Proteoglycan 4	1/4	53	300	127	126	6	**1.97**
					129	128	6	**0.43**
					131	130	6	**0.49**
1196	PRKACA P17612$_1$: cAMP-dependent protein kinase catalytic subunit alpha		3	3	127	126	3	**1.12**
					129	128	3	**2.38**
					131	130	3	**0.23**
1197	PRKAR1A P10644$_1$: cAMP-dependent protein kinase type I-alpha regulatory subunit		2	2	127	126	2	**1.04**
					129	128	2	**0.85**
					131	130	2	**0.16**

#	protein	group	peptides	spectra	ch1	ch2	quant	ratio
1198	PRKAR2A P13861$_1$: cAMP-dependent protein kinase type II-alpha regulatory subunit	1/2	10	11	127	126	11	**0.82**
					129	128	9	**0.37**
					131	130	10	**0.65**
1199	PRKCD Q05655$_2$: Protein kinase C delta type		3	9	127	126	3	**1.00**
					129	128		
					131	130	5	**0.48**
1200	PRKCDBP Q969G5: Protein kinase C delta-binding protein		3	5	127	126	4	**1.28**
					129	128	4	**1.35**
					131	130	5	**0.64**
1201	PRKCSH P14314$_{1,2}$: Glucosidase 2 subunit beta	2/2	11	17	127	126	17	**0.42**
					129	128	10	**1.60**
					131	130	16	**2.31**
1202	PRKDC P78527$_{1,2}$: DNA-dependent protein kinase catalytic subunit	2/2	18	20	127	126	15	**1.50**
					129	128	16	**0.56**
					131	130	19	**0.56**
1203	PRMT1 Q99873$_{1-4}$: Protein arginine N-methyltransferase 1	4/4	2	2	127	126	2	**0.64**
					129	128	2	**2.48**
					131	130	2	**1.51**
1204	PRNP P04156$_{1,2}$: Major prion protein	2/2	3	3	127	126	3	**0.54**
					129	128	3	**0.07**
					131	130	3	**1.17**
1205	PROC P04070$_{1,2}$: Vitamin K-dependent protein C	2/2	11	19	127	126	19	**0.44**
					129	128	13	**1.89**
					131	130	17	**2.82**
1206	PROS1 P07225: Vitamin K-dependent protein S		27	119	127	126	94	**0.48**
					129	128	82	**1.90**
					131	130	108	**1.47**
1208	PRPS1 P60891$_1$, PRPS2 P11908$_{1,2}$: Ribose-phosphate pyrophosphokinase 1, Ribose-phosphate pyrophosphokinase 2	3/4	3	3	127	126	3	**1.02**
					129	128	3	**0.63**
					131	130	3	**0.41**
1209	PRSS1 P07477: Trypsin-1		3	4	127	126	2	**0.31**
					129	128	2	**0.39**
					131	130	2	**1.33**
1210	PRSS2 P07478: Trypsin-2	1/6	5	6	127	126	6	**0.71**
					129	128	6	**0.49**
					131	130	5	**3.00**

#	protein	group	peptides	spectra	ch1	ch2	quant	ratio
1211	PRSS23 O95084$_1$: Serine protease 23		3	3	127	126	3	**1.44**
					129	128	3	**0.40**
					131	130	3	**2.29**
1212	PSAP P07602$_{1-3}$: Prosaposin	3/3	15	35	127	126	33	**0.36**
					129	128	35	**0.40**
					131	130	34	**1.90**
1213	PSMA1 P25786$_{1,2}$: Proteasome subunit alpha type-1	2/2	3	3	127	126	2	**0.79**
					129	128	2	**0.54**
					131	130	3	**1.61**
1214	PSMA3 P25788$_{1,2}$: Proteasome subunit alpha type-3	2/2	2	3	127	126	3	**0.86**
					129	128	3	**3.14**
					131	130	3	**2.36**
1215	PSMA4 P25789$_{1,2}$: Proteasome subunit alpha type-4	2/2	2	2	127	126	2	**0.66**
					129	128		
					131	130	2	**1.59**
1216	PSMA6 P60900$_{1,2}$: Proteasome subunit alpha type-6	2/2	2	2	127	126	2	**0.75**
					129	128	2	**2.45**
					131	130	2	**2.84**
1217	PSMA7 O14818$_1$: Proteasome subunit alpha type-7	1/2	4	4	127	126	3	**1.32**
					129	128	3	**0.80**
					131	130	4	**1.67**
1218	PSMB10 P40306: Proteasome subunit beta type-10		2	2	127	126	2	**0.67**
					129	128	2	**1.34**
					131	130	2	**2.34**
1219	PSMB6 P28072: Proteasome subunit beta type-6		2	2	127	126	2	**1.09**
					129	128	2	**2.57**
					131	130	2	**2.18**
1220	PSMC1 P62191$_{1,2}$: 26S protease regulatory subunit 4	2/2	2	3	127	126	1	**1.28**
					129	128		
					131	130	1	**0.69**
1221	PSMC2 P35998$_1$: 26S protease regulatory subunit 7	1/2	3	3	127	126	3	**0.91**
					129	128	2	**1.21**
					131	130	3	**1.07**
1222	PSMC3 P17980: 26S protease regulatory subunit 6A		4	5	127	126	5	**0.80**
					129	128	4	**2.05**
					131	130	5	**0.93**

#	protein	group	peptides	spectra	ch1	ch2	quant	ratio
1223	PSMC4 P43686$_{1}$: 26S protease regulatory subunit 6B	1/2	6	7	127	126	5	**1.20**
					129	128	5	**1.46**
					131	130	5	**0.66**
1224	PSMC5 P62195$_{1,2}$: 26S protease regulatory subunit 8	2/2	6	8	127	126	6	**0.96**
					129	128	5	**1.30**
					131	130	6	**0.54**
1225	PSMC6 P62333: 26S protease regulatory subunit 10B		5	5	127	126	4	**1.15**
					129	128	5	**0.87**
					131	130	5	**0.65**
1226	PSMD1 Q99460$_{1,2}$: 26S proteasome non-ATPase regulatory subunit 1	2/2	10	12	127	126	12	**1.17**
					129	128	8	**1.19**
					131	130	11	**0.51**
1227	PSMD11 O00231$_{1,2}$: 26S proteasome non-ATPase regulatory subunit 11	2/2	5	6	127	126	3	**1.13**
					129	128	3	**0.51**
					131	130	5	**0.50**
1228	PSMD12 O00232$_{1,2}$: 26S proteasome non-ATPase regulatory subunit 12	2/2	2	2	127	126	2	**7.27**
					129	128	2	**0.37**
					131	130	2	**0.71**
1229	PSMD13 Q9UNM6$_{1,2}$: 26S proteasome non-ATPase regulatory subunit 13	2/2	3	3	127	126	3	**1.40**
					129	128	2	**2.41**
					131	130	3	**0.37**
1230	PSMD2 Q13200$_{1}$: 26S proteasome non-ATPase regulatory subunit 2	1/3	9	10	127	126	9	**1.02**
					129	128	6	**1.68**
					131	130	10	**0.75**
1231	PSMD3 O43242$_{1}$: 26S proteasome non-ATPase regulatory subunit 3	1/2	8	11	127	126	7	**1.02**
					129	128	8	**1.16**
					131	130	9	**0.86**
1232	PSMD4 P55036$_{1,2}$: 26S proteasome non-ATPase regulatory subunit 4	2/3	3	3	127	126	2	**1.34**
					129	128	2	**1.67**
					131	130	2	**1.34**
1233	PSMD5 Q16401$_{1}$: 26S proteasome non-ATPase regulatory subunit 5	1/2	5	6	127	126	5	**1.70**
					129	128	6	**0.18**
					131	130	6	**0.41**
1234	PSMD6 Q15008$_{1}$: 26S proteasome non-ATPase regulatory subunit 6	1/3	5	5	127	126	1	**2.72**
					129	128		
					131	130	1	**3.07**

#	protein	group	peptides	spectra	ch1	ch2	quant	ratio
**1235**	PSMD6 Q15008$_4$: 26S proteasome non-ATPase regulatory subunit 6		5	5	127	126	1	**1.06**
					129	128	1	**1.77**
					131	130	1	**2.56**
**1236**	PSME1 Q06323$_1$: Proteasome activator complex subunit 1	1/3	10	12	127	126	11	**2.03**
					129	128	9	**0.54**
					131	130	12	**0.17**
**1237**	PSME2 Q9UL46: Proteasome activator complex subunit 2		8	13	127	126	13	**1.61**
					129	128	10	**0.16**
					131	130	12	**0.39**
**1238**	PSMF1 Q92530: Proteasome inhibitor PI31 subunit		1	1	127	126	1	**0.93**
					129	128	1	**1.88**
					131	130	1	**0.50**
**1239**	PTBP1 P26599$_{1-3}$: Polypyrimidine tract-binding protein 1	3/10	7	10	127	126	10	**2.30**
					129	128	8	**0.81**
					131	130	10	**0.30**
**1240**	PTGDS P41222: Prostaglandin-H2 D-isomerase		4	10	127	126	10	**0.53**
					129	128	10	**0.81**
					131	130	8	**2.09**
**1241**	PTGES3 Q15185$_{1-4}$: Prostaglandin E synthase 3	4/4	2	2	127	126	2	**0.58**
					129	128	2	**1.65**
					131	130	2	**1.83**
**1242**	PTGFRN Q9P2B2: Prostaglandin F2 receptor negative regulator		4	5	127	126	4	**1.58**
					129	128	4	**0.73**
					131	130	3	**0.45**
**1243**	PTGIS Q16647: Prostacyclin synthase		4	5	127	126	5	**1.92**
					129	128	5	**0.60**
					131	130	5	**0.82**
**1244**	PTGR1 Q14914$_1$: Prostaglandin reductase 1	1/2	3	4	127	126	3	**1.36**
					129	128	3	**0.68**
					131	130	3	**0.23**
**1245**	PTMA P06454$_{1,2}$: Prothymosin alpha [Cleaved into: Prothymosin alpha, N-terminally processed; . . .	2/2	3	3	127	126	2	**0.86**
					129	128	2	**2.04**
					131	130	3	**2.63**
**1246**	PTMS P20962: Parathymosin		4	8	127	126	8	**0.76**
					129	128	6	**2.42**
					131	130	6	**1.77**

#	protein	group	peptides	spectra	ch1	ch2	quant	ratio
1247	PTN P21246: Pleiotrophin		2	2	127	126	2	**1.55**
					129	128	2	**0.19**
					131	130	2	**2.21**
1248	PTPN23 Q9H3S7: Tyrosine-protein phosphatase non-receptor type 23		7	11	127	126	11	**0.65**
					129	128	11	**1.07**
					131	130	10	**0.96**
1249	PTPN6 $P29350_{1-4}$: Tyrosine-protein phosphatase non-receptor type 6	4/4	3	3	127	126	3	**1.35**
					129	128	3	**0.58**
					131	130	3	**0.52**
1250	PTRF $Q6NZI2_{1}$: Polymerase I and transcript release factor	1/3	11	24	127	126	24	**1.12**
					129	128	23	**0.67**
					131	130	24	**1.79**
1251	PXDN $Q92626_{1}$: Peroxidasin homolog	1/2	27	30	127	126	26	**0.60**
					129	128	24	**0.64**
					131	130	26	**1.07**
1252	PYGB P11216: Glycogen phosphorylase, brain form		6	6	127	126	5	**1.19**
					129	128	6	**0.76**
					131	130	6	**0.43**
1253	PZP $P20742_{1}$: Pregnancy zone protein	1/2	27	42	127	126	27	**0.42**
					129	128	20	**0.82**
					131	130	27	**0.87**
1254	QARS $P47897_{1,2}$: Glutamine--tRNA ligase	2/2	5	6	127	126	5	**1.32**
					129	128	3	**0.53**
					131	130	6	**0.44**
1255	QPCT $Q16769_{1}$: Glutaminyl-peptide cyclotransferase	1/2	4	5	127	126	4	**0.49**
					129	128	5	**0.97**
					131	130	5	**2.38**
1256	QSOX1 $O00391_{1}$: Sulfhydryl oxidase 1	1/2	37	116	127	126	101	**0.81**
					129	128	87	**1.02**
					131	130	114	**0.40**
1257	RAB10 P61026: Ras-related protein Rab-10		3	9	127	126	4	**7.11**
					129	128	2	**0.19**
					131	130	3	**0.16**
1258	RAB11B $Q15907_{1}$: Ras-related protein Rab-11B	1/4	9	10	127	126	6	**1.41**
					129	128	8	**1.17**
					131	130	9	**0.19**

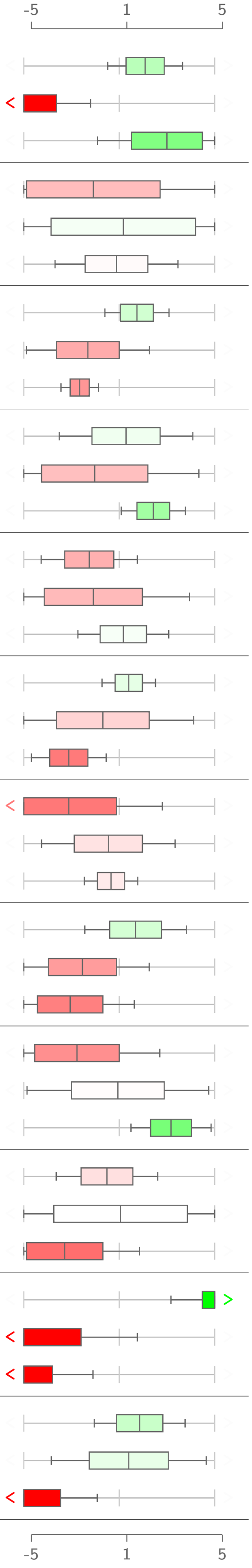

#	protein	group	peptides	spectra	ch1	ch2	quant	ratio
**1260**	RAB14 P61106: Ras-related protein Rab-14		3	4	127	126	3	**1.53**
					129	128	2	**0.66**
					131	130	4	**0.53**
**1261**	RAB1A P62820$_1$: Ras-related protein Rab-1A	1/3	8	11	127	126	4	**2.17**
					129	128	3	**0.61**
					131	130	6	**0.17**
**1262**	RAB1B Q9H0U4: Ras-related protein Rab-1B	1/2	6	7	127	126	2	**1.14**
					129	128	2	**0.63**
					131	130	2	**0.17**
**1263**	RAB2A P61019$_{1,2}$: Ras-related protein Rab-2A	2/2	3	4	127	126	4	**1.62**
					129	128	3	**0.73**
					131	130	4	**0.40**
**1264**	RAB35 Q15286$_1$: Ras-related protein Rab-35	1/2	4	7	127	126	2	**1.81**
					129	128	2	**0.46**
					131	130	2	**0.51**
**1266**	RAB3GAP2 Q9H2M9$_1$: Rab3 GTPase-activating protein non-catalytic subunit		4	4	127	126	4	**1.57**
					129	128	2	**0.95**
					131	130	3	**0.38**
**1267**	RAB5C P51148$_{1,2}$: Ras-related protein Rab-5C	2/6	4	4	127	126	3	**1.24**
					129	128	3	**0.57**
					131	130	4	**0.43**
**1268**	RAB7A P51149: Ras-related protein Rab-7a		4	4	127	126	4	**1.24**
					129	128	3	**1.36**
					131	130	4	**0.76**
**1269**	RABEP1 Q15276$_{1,2}$: Rab GTPase-binding effector protein 1	2/2	5	39	127	126	14	**0.96**
					129	128	11	**1.01**
					131	130	24	**2.44**
**1270**	RABGAP1 Q9Y3P9$_1$: Rab GTPase-activating protein 1		3	3	127	126	3	**2.66**
					129	128	3	**0.49**
					131	130	3	**0.67**
**1271**	RAC1 P63000$_{1,2}$: Ras-related C3 botulinum toxin substrate 1	2/8	6	9	127	126	6	**2.59**
					129	128	6	**0.90**
					131	130	6	**0.34**
**1272**	RACK1 P63244: Receptor of activated protein C kinase 1		12	12	127	126	12	**0.94**
					129	128	10	**1.86**
					131	130	10	**0.60**

#	protein	group	peptides	spectra	ch1	ch2	quant	ratio
1273	RAD23A P54725$_{1-3}$: UV excision repair protein RAD23 homolog A	3/3	2	2	127	126		
					129	128		
					131	130	1	**2.59**
1274	RAD23B P54727$_{1}$: UV excision repair protein RAD23 homolog B		2	2	127	126	1	**0.64**
					129	128	1	**1.79**
					131	130	1	**1.33**
1275	RALGAPB Q86X10$_{1,3}$: Ral GTPase-activating protein subunit beta	2/3	3	3	127	126	3	**2.01**
					129	128	3	**0.69**
					131	130	3	**0.44**
1276	RALY Q9UKM9$_{1,2}$: RNA-binding protein Raly	2/2	2	2	127	126	2	**0.69**
					129	128	2	**2.99**
					131	130	2	**1.70**
1277	RAN P62826: GTP-binding nuclear protein Ran		8	9	127	126	9	**0.80**
					129	128	9	**1.50**
					131	130	8	**0.58**
1278	RANBP2 P49792: E3 SUMO-protein ligase RanBP2		6	8	127	126	2	**0.43**
					129	128	4	**0.40**
					131	130	6	**0.96**
1280	RAP1B P61224$_{1-3}$, A6NIZ1: Ras-related protein Rap-1b, Ras-related protein Rap-1b-like protein	4/6	4	8	127	126	5	**1.70**
					129	128	5	**0.58**
					131	130	5	**0.50**
1281	RAP1GDS1 P52306$_{1-6}$: Rap1 GTPase-GDP dissociation stimulator 1	6/6	2	2	127	126	1	**0.72**
					129	128	1	**2.70**
					131	130	1	**0.31**
1282	RARRES1 P49788$_{1,2}$: Retinoic acid receptor responder protein 1	2/2	8	13	127	126	13	**0.47**
					129	128	13	**0.30**
					131	130	12	**0.73**
1283	RARRES2 Q99969: Retinoic acid receptor responder protein 2		8	74	127	126	60	**0.66**
					129	128	51	**0.57**
					131	130	66	**0.64**
1284	RARS P54136$_{1}$: Arginine--tRNA ligase, cytoplasmic	1/2	12	12	127	126	12	**1.15**
					129	128	9	**0.96**
					131	130	9	**0.28**
1285	RBBP4 Q09028$_{1,3}$: Histone-binding protein RBBP4	2/4	4	5	127	126	1	**0.90**
					129	128		
					131	130	1	**2.21**

-5 1 5

#	protein	group	peptides	spectra	ch1	ch2	quant	ratio
**1286**	RBBP7 Q16576$_{1,2}$: Histone-binding protein RBBP7	2/2	4	5	127	126	1	**0.68**
					129	128	1	**4.62**
					131	130	1	**1.41**
**1287**	RBM39 Q14498$_{1-3}$: RNA-binding protein 39	3/3	4	5	127	126	3	**2.55**
					129	128	2	**1.01**
					131	130	5	**0.87**
**1288**	RBMX P38159$_{1,2}$: RNA-binding motif protein, X chromosome	2/3	7	9	127	126	7	**1.98**
					129	128	7	**0.52**
					131	130	9	**1.36**
**1289**	RBP4 P02753: Retinol-binding protein 4		8	50	127	126	45	**0.53**
					129	128	46	**1.25**
					131	130	49	**1.78**
**1290**	RCC1 P18754$_{1,2}$: Regulator of chromosome condensation	2/2	2	2	127	126	2	**1.09**
					129	128	2	**2.66**
					131	130	2	**0.54**
**1291**	RECK O95980: Reversion-inducing cysteine-rich protein with Kazal motifs		2	2	127	126	2	**1.14**
					129	128	2	**0.54**
					131	130	2	**0.35**
**1292**	RECQL P46063: ATP-dependent DNA helicase Q1		4	24	127	126	7	**1.87**
					129	128	3	**0.55**
					131	130	9	**0.55**
**1293**	REEP5 Q00765$_{1}$: Receptor expression-enhancing protein 5		2	3	127	126	3	**0.97**
					129	128	3	**0.75**
					131	130	3	**0.43**
**1294**	REG3A Q06141: Regenerating islet-derived protein 3-alpha		4	4	127	126	3	**0.73**
					129	128	3	**0.27**
					131	130	4	**0.90**
**1295**	RELA Q04206$_{1-4}$: Transcription factor p65	4/4	6	6	127	126	6	**1.50**
					129	128	6	**0.28**
					131	130	5	**0.91**
**1296**	RHOA P61586: Transforming protein RhoA		5	6	127	126	2	**1.73**
					129	128	2	**0.51**
					131	130	2	**0.23**
**1297**	RHOC P08134: Rho-related GTP-binding protein RhoC		4	5	127	126	1	**2.38**
					129	128	1	**0.54**
					131	130	1	**0.10**

#	protein	group	peptides	spectra	ch1	ch2	quant	ratio
**1298**	RIC8A Q9NPQ8$_{1-4}$: Synembryn-A	4/4	2	2	127	126	1	**0.88**
					129	128	1	**1.18**
					131	130	2	**0.70**
**1299**	RNASE1 P07998: Ribonuclease pancreatic		8	26	127	126	21	**0.61**
					129	128	21	**0.18**
					131	130	24	**2.89**
**1300**	RNASE3 P12724: Eosinophil cationic protein		6	11	127	126	10	**1.69**
					129	128	10	**1.17**
					131	130	11	**1.53**
**1301**	RNASE4 P34096: Ribonuclease 4		12	164	127	126	131	**0.81**
					129	128	138	**0.25**
					131	130	154	**2.04**
**1302**	RNH1 P13489: Ribonuclease inhibitor		11	14	127	126	13	**1.02**
					129	128	11	**1.54**
					131	130	13	**0.35**
**1303**	ROCK2 O75116: Rho-associated protein kinase 2		2	2	127	126	2	**0.75**
					129	128	2	**0.98**
					131	130	2	**0.56**
**1304**	RPA1 P27694: Replication protein A 70 kDa DNA-binding subunit		5	16	127	126	3	**2.00**
					129	128	4	**0.61**
					131	130	7	**0.47**
**1305**	RPA2 P15927$_{1-3}$: Replication protein A 32 kDa subunit	3/3	3	4	127	126	3	**0.92**
					129	128	3	**1.27**
					131	130	3	**0.42**
**1306**	RPL10 P27635: 60S ribosomal protein L10		5	5	127	126	4	**1.81**
					129	128	5	**0.17**
					131	130	3	**0.82**
**1307**	RPL10A P62906: 60S ribosomal protein L10a		2	2	127	126	2	**0.48**
					129	128	2	**1.08**
					131	130	2	**1.01**
**1308**	RPL12 P30050$_{1}$: 60S ribosomal protein L12	1/2	7	8	127	126	8	**1.71**
					129	128	5	**0.55**
					131	130	8	**0.62**
**1309**	RPL13 P26373$_{1}$: 60S ribosomal protein L13	1/2	4	4	127	126	2	**1.47**
					129	128	2	**0.53**
					131	130	2	**1.03**

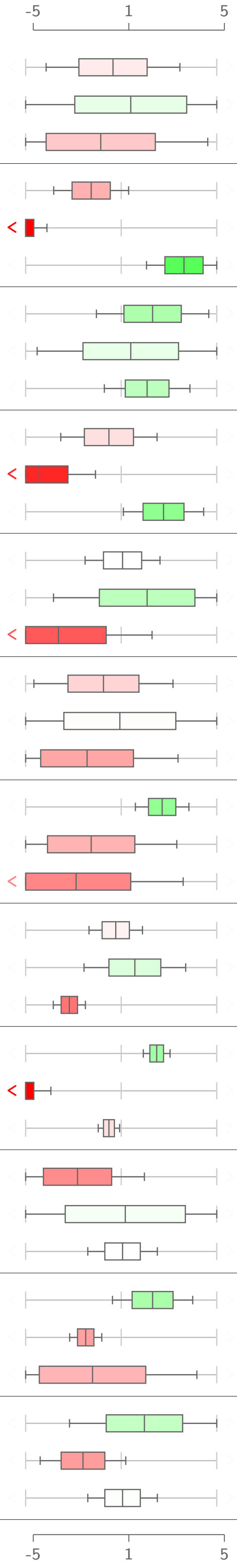

#	protein	group	peptides	spectra	ch1	ch2	quant	ratio
**1310**	RPL13A P40429: 60S ribosomal protein L13a		3	3	127	126	3	**1.53**
					129	128	3	**0.56**
					131	130	3	**0.70**
**1311**	RPL14 P50914: 60S ribosomal protein L14		8	9	127	126	8	**1.42**
					129	128	8	**0.46**
					131	130	8	**0.92**
**1312**	RPL15 $P61313_1$: 60S ribosomal protein L15		4	4	127	126	3	**2.49**
					129	128	4	**0.23**
					131	130	3	**1.03**
**1313**	RPL17 $P18621_{1,3}$: 60S ribosomal protein L17	2/3	4	5	127	126	4	**1.36**
					129	128	4	**0.29**
					131	130	4	**1.14**
**1314**	RPL18 $Q07020_{1,2}$: 60S ribosomal protein L18	2/2	3	3	127	126	3	**0.94**
					129	128	2	**0.50**
					131	130	3	**1.05**
**1315**	RPL18A Q02543: 60S ribosomal protein L18a		2	3	127	126	3	**0.83**
					129	128	2	**0.28**
					131	130	3	**1.32**
**1316**	RPL19 P84098: 60S ribosomal protein L19		5	7	127	126	6	**2.17**
					129	128	6	**0.14**
					131	130	6	**1.08**
**1317**	RPL22 P35268: 60S ribosomal protein L22		5	7	127	126	5	**0.79**
					129	128	5	**0.68**
					131	130	5	**1.83**
**1318**	RPL23 P62829: 60S ribosomal protein L23		2	3	127	126	3	**1.00**
					129	128	3	**0.58**
					131	130	3	**1.03**
**1319**	RPL23A P62750: 60S ribosomal protein L23a		5	7	127	126	7	**2.82**
					129	128	6	**0.23**
					131	130	7	**1.36**
**1320**	RPL24 P83731: 60S ribosomal protein L24		2	3	127	126	3	**4.41**
					129	128	3	**0.22**
					131	130	3	**1.63**
**1321**	RPL26 P61254: 60S ribosomal protein L26	1/2	5	5	127	126	4	**1.18**
					129	128	5	**0.63**
					131	130	4	**1.64**

#	protein	group	peptides	spectra	ch1	ch2	quant	ratio
1322	RPL27 P61353: 60S ribosomal protein L27		5	9	127	126	8	1.35
					129	128	8	0.55
					131	130	7	1.16
1323	RPL28 $P46779_{1-3}$: 60S ribosomal protein L28	3/5	4	6	127	126	6	2.10
					129	128	6	0.41
					131	130	6	0.82
1324	RPL3 P39023: 60S ribosomal protein L3		5	6	127	126	4	1.52
					129	128	5	0.25
					131	130	5	0.78
1325	RPL30 P62888: 60S ribosomal protein L30		5	7	127	126	5	1.19
					129	128	7	0.41
					131	130	6	0.93
1326	RPL31 $P62899_{1-3}$: 60S ribosomal protein L31	3/3	4	6	127	126	5	1.91
					129	128	5	0.57
					131	130	6	0.75
1327	RPL32 P62910: 60S ribosomal protein L32		2	2	127	126	2	2.08
					129	128	2	0.38
					131	130	2	0.80
1328	RPL34 P49207: 60S ribosomal protein L34		2	4	127	126	4	1.97
					129	128	4	0.32
					131	130	4	1.22
1329	RPL35 P42766: 60S ribosomal protein L35		4	4	127	126	3	2.15
					129	128	3	0.24
					131	130	3	1.71
1330	RPL35A P18077: 60S ribosomal protein L35a		7	9	127	126	8	1.57
					129	128	8	0.78
					131	130	8	0.92
1331	RPL36A P83881, RPL36AL Q969Q0: 60S ribosomal protein L36a, 60S ribosomal protein L36a-like	2/2	2	2	127	126	2	2.94
					129	128	2	0.22
					131	130	2	1.10
1332	RPL37 P61927: 60S ribosomal protein L37		4	4	127	126	4	1.88
					129	128	3	0.21
					131	130	3	1.31
1333	RPL37A P61513, RPL37AP8 A6NKH3: 60S ribosomal protein L37a, Putative 60S ribosomal protein L37a-like protein	2/2	1	1	127	126	1	1.36
					129	128	1	0.54
					131	130	1	1.01

#	protein	group	peptides	spectra	ch1	ch2	quant	ratio
**1334**	RPL38 P63173: 60S ribosomal protein L38		2	2	127	126	2	**1.74**
					129	128	2	**0.30**
					131	130	2	**1.43**
**1335**	RPL4 P36578: 60S ribosomal protein L4		3	3	127	126	3	**2.40**
					129	128	3	**0.18**
					131	130	3	**0.95**
**1336**	RPL5 P46777: 60S ribosomal protein L5		9	15	127	126	7	**1.18**
					129	128	7	**0.74**
					131	130	12	**0.82**
**1337**	RPL6 Q02878: 60S ribosomal protein L6		8	8	127	126	8	**1.74**
					129	128	8	**0.39**
					131	130	8	**0.83**
**1338**	RPL7 P18124: 60S ribosomal protein L7		4	4	127	126	3	**1.99**
					129	128	4	**0.48**
					131	130	3	**0.60**
**1339**	RPL7A P62424: 60S ribosomal protein L7a		6	11	127	126	8	**1.41**
					129	128	10	**0.24**
					131	130	11	**0.88**
**1340**	RPL8 P62917: 60S ribosomal protein L8		7	7	127	126	5	**1.78**
					129	128	6	**0.34**
					131	130	7	**0.93**
**1341**	RPL9 P32969: 60S ribosomal protein L9		4	4	127	126	4	**1.03**
					129	128	4	**0.82**
					131	130	4	**0.82**
**1342**	RPLP0 P05388$_1$: 60S acidic ribosomal protein P0	1/3	10	20	127	126	18	**1.46**
					129	128	16	**1.02**
					131	130	19	**0.37**
**1343**	RPLP2 P05387: 60S acidic ribosomal protein P2		3	4	127	126	3	**0.38**
					129	128	3	**2.30**
					131	130	4	**2.44**
**1344**	RPN1 P04843: Dolichyl-diphosphooligosaccharide--protein glycosyltransferase subunit 1		11	12	127	126	10	**1.88**
					129	128	11	**0.86**
					131	130	11	**0.62**
**1345**	RPS10 P46783: 40S ribosomal protein S10	1/2	8	10	127	126	8	**1.80**
					129	128	8	**0.34**
					131	130	9	**0.75**

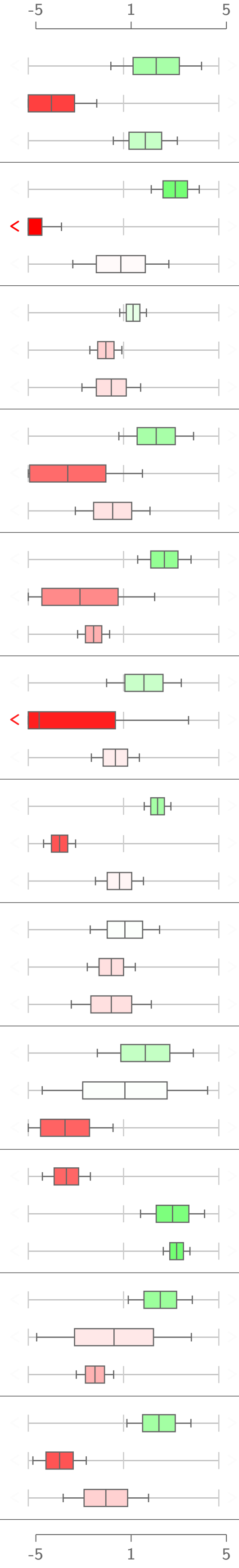

#	protein	group	peptides	spectra	ch1	ch2	quant	ratio
**1346**	RPS11 P62280: 40S ribosomal protein S11		4	4	127	126	4	**1.40**
					129	128	3	**0.65**
					131	130	4	**0.69**
**1347**	RPS13 P62277: 40S ribosomal protein S13		3	3	127	126	3	**1.48**
					129	128	3	**0.52**
					131	130	3	**0.84**
**1348**	RPS14 P62263: 40S ribosomal protein S14		4	5	127	126	5	**1.34**
					129	128	5	**0.80**
					131	130	5	**0.90**
**1349**	RPS15A P62244: 40S ribosomal protein S15a		2	2	127	126	2	**1.83**
					129	128	2	**0.41**
					131	130	2	**0.63**
**1350**	RPS16 P62249: 40S ribosomal protein S16		6	6	127	126	6	**1.59**
					129	128	6	**0.42**
					131	130	6	**0.62**
**1351**	RPS18 P62269: 40S ribosomal protein S18		2	3	127	126	3	**1.72**
					129	128	2	**0.91**
					131	130	3	**0.59**
**1352**	RPS19 P39019: 40S ribosomal protein S19		5	6	127	126	4	**1.74**
					129	128	6	**0.28**
					131	130	5	**1.15**
**1353**	RPS2 P15880: 40S ribosomal protein S2		8	8	127	126	8	**1.31**
					129	128	7	**0.61**
					131	130	7	**0.78**
**1354**	RPS20 $P60866_{1,2}$: 40S ribosomal protein S20	2/2	4	7	127	126	7	**1.89**
					129	128	7	**0.64**
					131	130	7	**0.71**
**1355**	RPS23 P62266: 40S ribosomal protein S23		3	4	127	126	4	**1.47**
					129	128	3	**0.49**
					131	130	4	**0.81**
**1356**	RPS24 $P62847_{1-4}$: 40S ribosomal protein S24	4/4	2	2	127	126	2	**3.20**
					129	128	2	**0.46**
					131	130	2	**1.53**
**1357**	RPS27A P62979: Ubiquitin-40S ribosomal protein S27a	1/4	10	17	127	126	14	**2.75**
					129	128	15	**0.20**
					131	130	15	**1.07**

#	protein	group	peptides	spectra	ch1	ch2	quant	ratio
**1358**	RPS3 P23396$_{1,2}$: 40S ribosomal protein S3	2/2	7	9	127	126	8	**1.34**
					129	128	8	**1.03**
					131	130	6	**0.66**
**1359**	RPS3A P61247: 40S ribosomal protein S3a		2	2	127	126	2	**1.30**
					129	128	2	**0.44**
					131	130	2	**0.92**
**1360**	RPS4X P62701: 40S ribosomal protein S4, X isoform		6	7	127	126	3	**1.62**
					129	128	3	**0.70**
					131	130	3	**0.76**
**1361**	RPS4Y2 Q8TD47: 40S ribosomal protein S4, Y isoform 2	1/2	4	5	127	126	1	**1.55**
					129	128	1	**0.48**
					131	130	1	**0.83**
**1362**	RPS5 P46782: 40S ribosomal protein S5 [Cleaved into: 40S ribosomal protein S5, N-terminal ...		4	6	127	126	6	**3.03**
					129	128	5	**0.43**
					131	130	6	**0.53**
**1363**	RPS6 P62753: 40S ribosomal protein S6		5	6	127	126	5	**1.91**
					129	128	6	**0.21**
					131	130	6	**1.37**
**1364**	RPS6KA3 P51812: Ribosomal protein S6 kinase alpha-3	1/8	11	11	127	126	10	**1.28**
					129	128	7	**1.42**
					131	130	10	**0.31**
**1365**	RPS7 P62081: 40S ribosomal protein S7		2	2	127	126	2	**1.67**
					129	128	2	**0.92**
					131	130	2	**0.99**
**1366**	RPS8 P62241: 40S ribosomal protein S8		4	4	127	126	3	**2.36**
					129	128	3	**0.12**
					131	130	4	**0.98**
**1367**	RPS9 P46781: 40S ribosomal protein S9		8	9	127	126	8	**1.63**
					129	128	9	**0.58**
					131	130	9	**0.87**
**1368**	RPSA P08865: 40S ribosomal protein SA		12	20	127	126	17	**0.70**
					129	128	15	**0.83**
					131	130	19	**0.74**
**1369**	RRBP1 Q9P2E9$_{1,2}$: Ribosome-binding protein 1	2/3	5	7	127	126	6	**0.78**
					129	128	5	**0.30**
					131	130	6	**1.52**

#	protein	group	peptides	spectra	ch1	ch2	quant	ratio
**1370**	RTCB Q9Y3I0: tRNA-splicing ligase RtcB homolog		4	4	127	126	4	**0.69**
					129	128	3	**3.17**
					131	130	4	**0.75**
**1371**	RTN1 $Q16799_{1,2}$: Reticulon-1	2/2	2	2	127	126	1	**1.64**
					129	128		
					131	130	1	**0.73**
**1372**	RTN3 $O95197_{1-5,7}$: Reticulon-3	6/6	2	2	127	126	1	**1.57**
					129	128	1	**1.39**
					131	130	1	**0.35**
**1373**	RTN4 $Q9NQC3_{1,2,5}$: Reticulon-4	3/6	2	3	127	126	3	**1.35**
					129	128	3	**1.03**
					131	130	3	**0.55**
**1374**	RUVBL1 $Q9Y265_{1}$: RuvB-like 1	1/2	2	2	127	126	2	**1.12**
					129	128	2	**4.50**
					131	130	2	**0.70**
**1375**	RUVBL2 $Q9Y230_{1}$: RuvB-like 2		3	4	127	126	4	**1.66**
					129	128	4	**0.20**
					131	130	4	**0.91**
**1376**	RYR1 $P21817_{1-3}$: Ryanodine receptor 1	3/3	2	4	127	126	4	**0.85**
					129	128	3	**1.08**
					131	130	4	**0.15**
**1377**	S100A10 P60903: Protein S100-A10		6	15	127	126	11	**1.31**
					129	128	9	**0.64**
					131	130	9	**1.07**
**1378**	S100A11 P31949: Protein S100-A11		2	2	127	126	2	**1.75**
					129	128	2	**0.55**
					131	130	2	**0.76**
**1379**	S100A6 P06703: Protein S100-A6		4	8	127	126	7	**1.34**
					129	128	8	**0.70**
					131	130	8	**0.34**
**1380**	S100A8 P05109: Protein S100-A8		4	4	127	126	4	**3.87**
					129	128	4	**0.80**
					131	130	4	**1.16**
**1381**	S100A9 P06702: Protein S100-A9		1	1	127	126	1	**4.96**
					129	128	1	**0.40**
					131	130	1	**0.92**

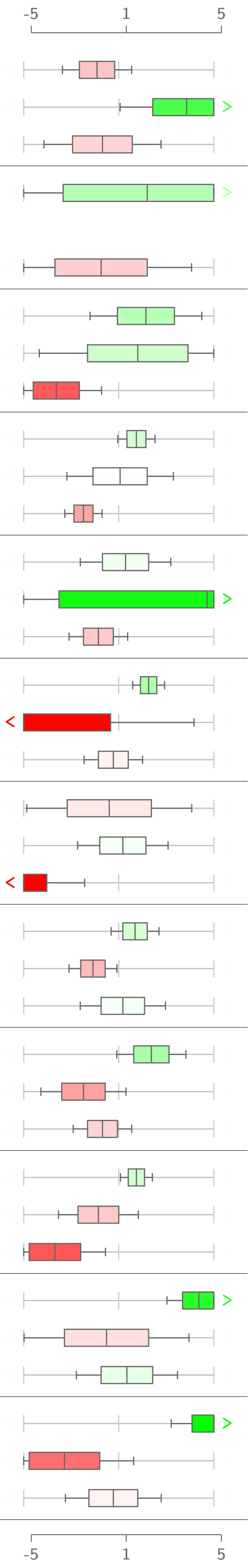

#	protein	group	peptides	spectra	ch1	ch2	quant	ratio
**1382**	SAA1 P0DJI8: Serum amyloid A-1 protein	1/2	9	51	127	126	10	**0.44**
					129	128	8	**0.46**
					131	130	11	**1.31**
**1383**	SAA2 P0DJI9$_1$: Serum amyloid A-2 protein		9	46	127	126	6	**0.95**
					129	128	6	**0.42**
					131	130	6	**1.07**
**1384**	SAA4 P35542: Serum amyloid A-4 protein		6	193	127	126	182	**0.86**
					129	128	179	**0.58**
					131	130	184	**0.68**
**1385**	SAE1 Q9UBE0$_{1-3}$: SUMO-activating enzyme subunit 1	3/3	2	2	127	126	2	**1.75**
					129	128	2	**0.58**
					131	130	2	**0.50**
**1386**	SAFB Q15424$_{1,3,4}$: Scaffold attachment factor B1	3/4	6	6	127	126	4	**0.95**
					129	128	5	**0.62**
					131	130	5	**1.71**
**1387**	SAMHD1 Q9Y3Z3$_{1-3}$: Deoxynucleoside triphosphate triphosphohydrolase SAMHD1	3/4	3	3	127	126	3	**0.66**
					129	128	2	**8.12**
					131	130	3	**0.96**
**1388**	SAR1B Q9Y6B6: GTP-binding protein SAR1b	1/3	3	3	127	126	2	**1.55**
					129	128	2	**0.23**
					131	130	3	**0.53**
**1389**	SARS P49591: Serine--tRNA ligase, cytoplasmic		8	9	127	126	8	**1.03**
					129	128	6	**0.63**
					131	130	8	**0.85**
**1390**	SART3 Q15020$_{1,4}$: Squamous cell carcinoma antigen recognized by T-cells 3	2/2	6	102	127	126	4	**1.42**
					129	128	4	**1.26**
					131	130	3	**0.71**
**1391**	SBSN Q6UWP8$_1$: Suprabasin		4	4	127	126	4	**2.22**
					129	128	4	**0.17**
					131	130	4	**1.35**
**1392**	SCFD1 Q8WVM8$_1$: Sec1 family domain-containing protein 1	1/3	4	4	127	126	4	**1.48**
					129	128	3	**0.54**
					131	130	4	**0.26**
**1393**	SCG2 P13521: Secretogranin-2		22	33	127	126	31	**0.39**
					129	128	31	**0.36**
					131	130	31	**2.39**

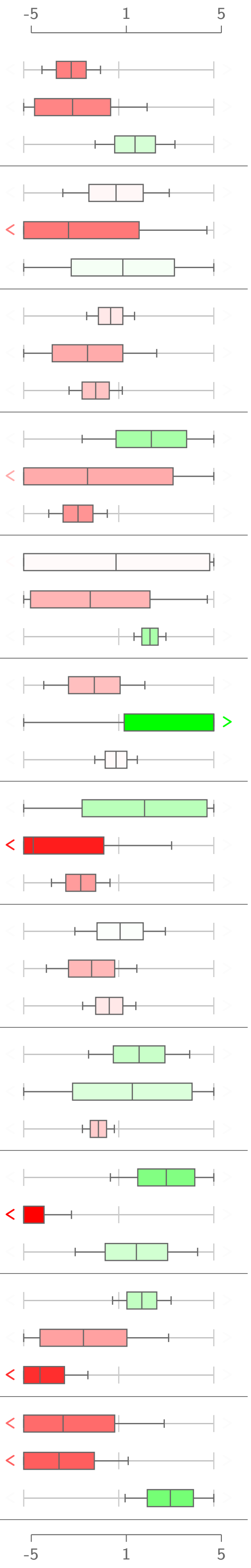

#	protein	group	peptides	spectra	ch1	ch2	quant	ratio
**1394**	SCG3 Q8WXD2$_1$: Secretogranin-3	1/2	5	6	127	126	5	**0.34**
					129	128	6	**0.33**
					131	130	5	**3.03**
**1395**	SCGB1A1 P11684: Uteroglobin		2	2	127	126	2	**0.39**
					129	128	2	**0.75**
					131	130	2	**0.95**
**1396**	SCRN1 Q12765$_{1-3}$: Secernin-1	3/3	1	1	127	126	1	**0.65**
					129	128	1	**0.92**
					131	130	1	**2.08**
**1397**	SCUBE2 Q9NQ36$_{1-3}$: Signal peptide, CUB and EGF-like domain-containing protein 2	3/3	5	5	127	126	4	**1.32**
					129	128	4	**0.24**
					131	130	4	**0.70**
**1398**	SDF4 Q9BRK5$_1$: 45 kDa calcium-binding protein	1/6	8	15	127	126	15	**0.39**
					129	128	14	**1.59**
					131	130	14	**2.77**
**1399**	SDPR O95810: Serum deprivation-response protein		8	10	127	126	8	**0.68**
					129	128	9	**3.29**
					131	130	9	**2.39**
**1400**	SEC22B O75396: Vesicle-trafficking protein SEC22b		2	2	127	126	2	**1.42**
					129	128	2	**1.32**
					131	130	2	**0.55**
**1401**	SEC23A Q15436$_1$: Protein transport protein Sec23A	1/3	8	8	127	126	7	**0.50**
					129	128	5	**1.28**
					131	130	7	**0.42**
**1402**	SEC23IP Q9Y6Y8$_{1,2}$: SEC23-interacting protein	2/2	6	6	127	126	5	**1.28**
					129	128	4	**1.46**
					131	130	4	**0.52**
**1403**	SEC24C P53992$_1$: Protein transport protein Sec24C	1/2	6	6	127	126	5	**1.05**
					129	128	4	**0.39**
					131	130	6	**0.86**
**1404**	SEC31A O94979$_{1-4,6,8-10}$: Protein transport protein Sec31A	8/10	10	11	127	126	9	**1.43**
					129	128	10	**1.07**
					131	130	10	**0.35**
**1405**	SEMA3B Q13214$_{1,2}$: Semaphorin-3B	2/2	3	3	127	126	3	**0.82**
					129	128	3	**0.66**
					131	130	3	**0.73**

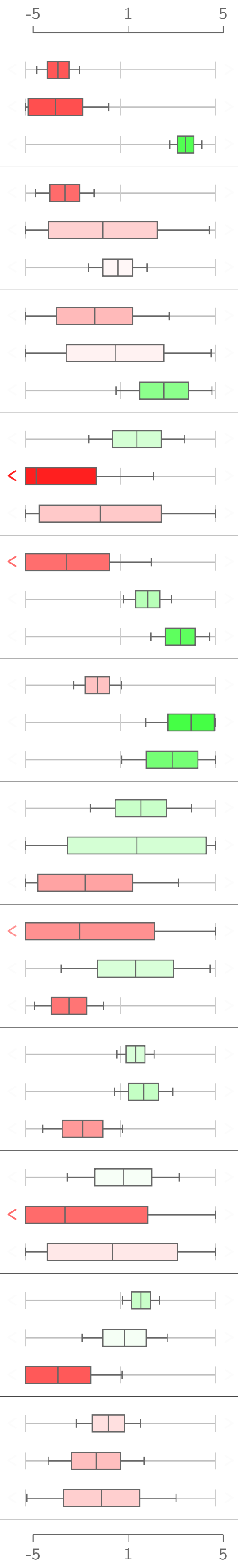

#	protein	group	peptides	spectra	ch1	ch2	quant	ratio
**1406**	SEPP1 P49908: Selenoprotein P		7	20	127	126	17	**0.36**
					129	128	10	**2.78**
					131	130	18	**1.42**
**1407**	SEPT2 Q15019$_{1,2}$: Septin-2	2/3	5	5	127	126	4	**1.16**
					129	128	5	**0.63**
					131	130	4	**0.39**
**1408**	SEPT7 Q16181$_{1,2}$: Septin-7	2/2	8	111	127	126	23	**1.55**
					129	128	19	**0.46**
					131	130	46	**0.55**
**1409**	SEPT9 Q9UHD8$_{1,2,5,7}$: Septin-9	4/8	3	3	127	126	3	**2.36**
					129	128	3	**0.34**
					131	130	3	**0.22**
**1410**	SERBP1 Q8NC51$_{1-4}$: Plasminogen activator inhibitor 1 RNA-binding protein	4/4	2	2	127	126	2	**0.74**
					129	128	2	**1.14**
					131	130	2	**0.87**
**1411**	SERPINA1 P01009$_{1}$: Alpha-1-antitrypsin	1/3	24	202	127	126	166	**0.52**
					129	128	133	**1.40**
					131	130	199	**0.86**
**1412**	SERPINA10 Q9UK55: Protein Z-dependent protease inhibitor		14	32	127	126	30	**0.89**
					129	128	19	**0.97**
					131	130	31	**0.30**
**1413**	SERPINA3 P01011$_{1}$: Alpha-1-antichymotrypsin	1/2	17	48	127	126	40	**0.47**
					129	128	32	**1.72**
					131	130	45	**0.40**
**1414**	SERPINA4 P29622: Kallistatin		19	50	127	126	45	**0.89**
					129	128	28	**1.19**
					131	130	44	**0.46**
**1415**	SERPINA5 P05154: Plasma serine protease inhibitor		11	21	127	126	21	**0.75**
					129	128	18	**1.02**
					131	130	19	**0.66**
**1416**	SERPINA6 P08185: Corticosteroid-binding globulin		6	7	127	126	6	**0.50**
					129	128	5	**0.95**
					131	130	6	**0.36**
**1417**	SERPINA7 P05543: Thyroxine-binding globulin		9	11	127	126	11	**0.96**
					129	128	8	**1.37**
					131	130	11	**0.22**

#	protein	group	peptides	spectra	ch1	ch2	quant	ratio
**1418**	SERPINB2 P05120: Plasminogen activator inhibitor 2		3	5	127	126	5	**1.19**
					129	128	2	**0.77**
					131	130	4	**0.05**
**1419**	SERPINC1 P01008: Antithrombin-III		27	157	127	126	144	**0.46**
					129	128	116	**1.42**
					131	130	151	**1.51**
**1420**	SERPIND1 P05546: Heparin cofactor 2		27	469	127	126	418	**0.90**
					129	128	245	**0.48**
					131	130	396	**0.32**
**1421**	SERPINE1 $P05121_1$: Plasminogen activator inhibitor 1	1/2	8	10	127	126	9	**0.93**
					129	128	10	**0.60**
					131	130	9	**1.33**
**1422**	SERPINE2 $P07093_{1-3}$: Glia-derived nexin	3/3	3	4	127	126	4	**0.89**
					129	128	4	**0.97**
					131	130	4	**0.68**
**1423**	SERPINF1 P36955: Pigment epithelium-derived factor		16	39	127	126	37	**0.47**
					129	128	36	**1.21**
					131	130	37	**0.99**
**1424**	SERPINF2 $P08697_1$: Alpha-2-antiplasmin	1/2	17	83	127	126	72	**0.71**
					129	128	61	**1.01**
					131	130	70	**0.42**
**1425**	SERPING1 $P05155_{1-3}$: Plasma protease C1 inhibitor	3/3	12	19	127	126	19	**0.78**
					129	128	17	**1.00**
					131	130	19	**0.69**
**1426**	SERPINH1 P50454: Serpin H1		6	6	127	126	6	**1.30**
					129	128	6	**0.83**
					131	130	6	**0.54**
**1427**	SET $Q01105_2$: Protein SET	1/5	4	5	127	126	5	**0.52**
					129	128	4	**0.48**
					131	130	5	**2.43**
**1428**	SF3A1 $Q15459_{1,2}$: Splicing factor 3A subunit 1	2/2	5	7	127	126	4	**3.34**
					129	128	3	**0.49**
					131	130	4	**0.80**
**1429**	SF3B1 $O75533_1$: Splicing factor 3B subunit 1		8	12	127	126	12	**0.58**
					129	128	10	**1.68**
					131	130	12	**1.15**

#	protein	group	peptides	spectra	ch1	ch2	quant	ratio
**1430**	SF3B2 Q13435: Splicing factor 3B subunit 2		2	2	127	126	2	**2.58**
					129	128	2	**0.67**
					131	130	2	**1.43**
**1431**	SF3B3 Q15393$_1$: Splicing factor 3B subunit 3	1/2	3	3	127	126	3	**1.50**
					129	128	3	**1.25**
					131	130	3	**1.61**
**1432**	SFN P31947$_{1,2}$: 14-3-3 protein sigma	2/2	5	16	127	126	1	**1.80**
					129	128	1	**0.62**
					131	130	1	**2.94**
**1433**	SFPQ P23246$_1$: Splicing factor, proline- and glutamine-rich	1/2	11	18	127	126	12	**0.67**
					129	128	10	**1.72**
					131	130	14	**1.48**
**1434**	SFRP1 Q8N474: Secreted frizzled-related protein 1		4	6	127	126	5	**0.30**
					129	128	4	**0.47**
					131	130	5	**2.14**
**1435**	SFRP2 Q96HF1: Secreted frizzled-related protein 2		10	26	127	126	22	**0.37**
					129	128	21	**1.04**
					131	130	21	**1.32**
**1436**	SFTPB P07988: Pulmonary surfactant-associated protein B		5	7	127	126	6	**1.03**
					129	128	5	**0.44**
					131	130	6	**0.46**
**1437**	SGTA O43765: Small glutamine-rich tetratricopeptide repeat-containing protein alpha		2	2	127	126	2	**0.56**
					129	128	2	**2.00**
					131	130	2	**2.23**
**1438**	SH3BGRL3 Q9H299: SH3 domain-binding glutamic acid-rich-like protein 3		3	4	127	126	3	**0.78**
					129	128	3	**0.63**
					131	130	3	**1.44**
**1439**	SH3GLB1 Q9Y371$_{1-3}$: Endophilin-B1	3/3	3	4	127	126	4	**1.61**
					129	128	4	**0.40**
					131	130	4	**0.56**
**1440**	SH3GLB2 Q9NR46$_{1,2}$: Endophilin-B2	2/2	3	3	127	126	3	**1.41**
					129	128	3	**0.95**
					131	130	3	**0.67**
**1441**	SHBG P04278$_1$: Sex hormone-binding globulin	1/5	13	62	127	126	53	**0.85**
					129	128	36	**0.41**
					131	130	54	**0.43**

#	protein	group	peptides	spectra	ch1	ch2	quant	ratio
1442	SHROOM3 Q8TF72$_1$: Protein Shroom3		1	1	127	126	1	**8.08**
					129	128	1	**0.11**
					131	130	1	**2.86**
1443	SIL1 Q9H173: Nucleotide exchange factor SIL1		3	3	127	126	3	**0.99**
					129	128	3	**0.47**
					131	130	3	**0.77**
1444	SKIV2L2 P42285: Superkiller viralicidic activity 2-like 2	1/2	4	4	127	126	4	**1.63**
					129	128	3	**0.84**
					131	130	4	**0.40**
1445	SKP1 P63208$_1$: S-phase kinase-associated protein 1	1/2	5	5	127	126	4	**0.48**
					129	128	4	**1.43**
					131	130	3	**1.46**
1446	SLC25A3 Q00325$_{1,2}$: Phosphate carrier protein, mitochondrial	2/2	3	4	127	126	4	**3.02**
					129	128	3	**0.23**
					131	130	3	**0.31**
1447	SLC25A5 P05141: ADP/ATP translocase 2	1/3	6	7	127	126	7	**1.15**
					129	128	6	**1.56**
					131	130	7	**1.20**
1448	SLC3A2 P08195$_{1-4}$: 4F2 cell-surface antigen heavy chain	4/4	1	1	127	126	1	**0.88**
					129	128	1	**1.15**
					131	130	1	**0.20**
1449	SLC9A3R1 O14745$_1$: Na(+)/H(+) exchange regulatory cofactor NHE-RF1		5	5	127	126	4	**0.66**
					129	128	2	**2.07**
					131	130	5	**2.55**
1450	SLK Q9H2G2$_{1,2}$: STE20-like serine/threonine-protein kinase	2/2	10	10	127	126	8	**1.49**
					129	128	8	**0.66**
					131	130	8	**0.52**
1451	SLPI P03973: Antileukoproteinase		15	89	127	126	72	**0.91**
					129	128	71	**0.11**
					131	130	78	**1.73**
1452	SMARCC2 Q8TAQ2$_{1-3}$: SWI/SNF complex subunit SMARCC2	3/3	6	7	127	126	5	**2.76**
					129	128	5	**0.14**
					131	130	4	**1.50**
1453	SMS P52788$_1$: Spermine synthase	1/2	3	3	127	126	3	**2.23**
					129	128	3	**0.14**
					131	130	3	**1.37**

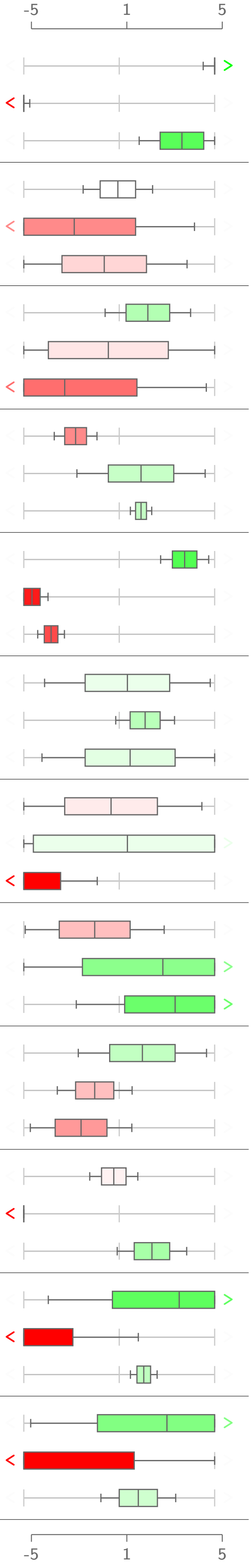

#	protein	group	peptides	spectra	ch1	ch2	quant	ratio
**1454**	SMYD5 Q6GMV2: SET and MYND domain-containing protein 5		2	2	127	126	2	**0.73**
					129	128	2	**1.07**
					131	130	2	**1.74**
**1456**	SND1 Q7KZF4: Staphylococcal nuclease domain-containing protein 1		14	19	127	126	18	**1.51**
					129	128	14	**0.32**
					131	130	19	**0.32**
**1457**	SNED1 Q8TER0$_{1,3-5}$: Sushi, nidogen and EGF-like domain-containing protein 1	4/4	6	7	127	126	6	**1.26**
					129	128	6	**0.31**
					131	130	7	**0.90**
**1458**	SNRNP200 O75643$_{1}$: U5 small nuclear ribonucleoprotein 200 kDa helicase		8	9	127	126	9	**1.48**
					129	128	9	**0.60**
					131	130	8	**0.81**
**1459**	SNRNP70 P08621$_{1,2}$: U1 small nuclear ribonucleoprotein 70 kDa	2/3	5	5	127	126	3	**1.59**
					129	128	5	**0.47**
					131	130	4	**2.22**
**1460**	SNRPA P09012: U1 small nuclear ribonucleoprotein A		2	2	127	126	2	**2.54**
					129	128	2	**1.55**
					131	130	2	**0.39**
**1461**	SNRPD2 P62316$_{1}$: Small nuclear ribonucleoprotein Sm D2		1	1	127	126	1	**2.54**
					129	128	1	**0.35**
					131	130	1	**1.44**
**1462**	SNRPF P62306: Small nuclear ribonucleoprotein F		1	1	127	126	1	**0.92**
					129	128		
					131	130	1	**1.64**
**1463**	SNX1 Q13596$_{1-3}$: Sorting nexin-1	3/3	3	6	127	126	6	**1.21**
					129	128	4	**0.82**
					131	130	6	**0.75**
**1464**	SNX12 Q9UMY4$_{1,2}$: Sorting nexin-12	2/3	3	3	127	126	2	**0.81**
					129	128	2	**0.89**
					131	130	2	**0.84**
**1465**	SNX2 O60749$_{1,2}$: Sorting nexin-2	2/2	2	2	127	126	2	**0.79**
					129	128	2	**3.57**
					131	130	2	**0.55**
**1466**	SNX3 O60493$_{1}$: Sorting nexin-3	1/3	3	3	127	126	2	**2.63**
					129	128	2	**0.97**
					131	130	2	**1.23**

#	protein	group	peptides	spectra	ch1	ch2	quant	ratio
**1467**	SNX5 Q9Y5X3$_1$: Sorting nexin-5		4	4	127	126	3	**1.97**
					129	128	3	**1.13**
					131	130	3	**1.26**
**1468**	SNX6 Q9UNH7$_{1,2}$: Sorting nexin-6	2/2	8	10	127	126	9	**0.91**
					129	128	9	**0.91**
					131	130	10	**0.92**
**1469**	SOD3 P08294: Extracellular superoxide dismutase [Cu-Zn]		9	43	127	126	40	**1.11**
					129	128	38	**0.62**
					131	130	39	**2.23**
**1470**	SP100 P23497$_1$: Nuclear autoantigen Sp-100		6	17	127	126	1	**0.85**
					129	128	1	**3.58**
					131	130	1	**0.68**
**1471**	SPARC P09486: SPARC		14	37	127	126	33	**0.41**
					129	128	30	**1.95**
					131	130	36	**2.07**
**1472**	SPARCL1 Q14515$_1$: SPARC-like protein 1	1/2	32	83	127	126	65	**0.32**
					129	128	64	**1.35**
					131	130	80	**3.12**
**1473**	SPCS2 Q15005: Signal peptidase complex subunit 2		2	2	127	126	2	**1.90**
					129	128	2	**0.99**
					131	130	2	**0.53**
**1474**	SPCS3 P61009: Signal peptidase complex subunit 3		1	1	127	126	1	**2.80**
					129	128	1	**0.89**
					131	130	1	**0.93**
**1475**	SPEF2 Q9C093$_{1,2}$: Sperm flagellar protein 2	2/2	3	5	127	126	2	**0.88**
					129	128	2	**5.03**
					131	130	2	**0.86**
**1476**	SPINK5 Q9NQ38$_{1-3}$: Serine protease inhibitor Kazal-type 5	3/3	8	69	127	126	31	**1.99**
					129	128	9	**0.62**
					131	130	56	**2.77**
**1477**	SPON1 Q9HCB6: Spondin-1		17	22	127	126	20	**0.52**
					129	128	21	**0.55**
					131	130	20	**1.62**
**1478**	SPP2 Q13103: Secreted phosphoprotein 24		9	12	127	126	9	**0.60**
					129	128	8	**1.20**
					131	130	11	**2.04**

#	protein	group	peptides	spectra	ch1	ch2	quant	ratio
1479	SPR P35270: Sepiapterin reductase		3	4	127	126	4	**1.48**
					129	128	3	**0.27**
					131	130	4	**0.31**
1480	SPTAN1 Q13813$_{1,3}$: Spectrin alpha chain, non-erythrocytic 1	2/3	47	75	127	126	57	**1.63**
					129	128	36	**0.63**
					131	130	70	**0.77**
1481	SPTBN1 Q01082$_{1}$: Spectrin beta chain, non-erythrocytic 1	1/2	47	61	127	126	1	**0.50**
					129	128	1	**3.34**
					131	130	1	**5.78**
1482	SPTBN1 Q01082$_{3}$: Spectrin beta chain, non-erythrocytic 1		47	61	127	126	1	**4.27**
					129	128	1	**0.50**
					131	130	1	**0.23**
1483	SPTBN2 O15020$_{1}$: Spectrin beta chain, non-erythrocytic 2	1/2	9	21	127	126	11	**2.01**
					129	128	6	**1.20**
					131	130	15	**2.12**
1485	SQRDL Q9Y6N5: Sulfide:quinone oxidoreductase, mitochondrial		4	5	127	126	5	**1.86**
					129	128	3	**1.60**
					131	130	5	**0.77**
1486	SRGAP2 O75044: SLIT-ROBO Rho GTPase-activating protein 2		4	4	127	126	2	**2.66**
					129	128	2	**0.32**
					131	130	4	**0.55**
1487	SRI P30626$_{1-3}$: Sorcin	3/3	3	4	127	126	4	**0.57**
					129	128	4	**0.78**
					131	130	4	**1.58**
1488	SRM P19623: Spermidine synthase		2	2	127	126	2	**1.00**
					129	128	2	**2.55**
					131	130	2	**0.74**
1489	SRPX P78539$_{1,4}$: Sushi repeat-containing protein SRPX	2/5	8	13	127	126	11	**1.15**
					129	128	13	**0.41**
					131	130	13	**0.42**
1490	SRRM1 Q8IYB3$_{1,2}$: Serine/arginine repetitive matrix protein 1	2/2	2	4	127	126	3	**0.46**
					129	128	3	**3.26**
					131	130	3	**1.34**
1491	SRRT Q9BXP5$_{1-4}$: Serrate RNA effector molecule homolog	4/5	4	4	127	126	4	**1.18**
					129	128	3	**1.20**
					131	130	4	**1.99**

#	protein	group	peptides	spectra	ch1	ch2	quant	ratio
**1492**	SRSF1 Q07955$_1$: Serine/arginine-rich splicing factor 1	1/3	11	17	127	126	15	**0.99**
					129	128	16	**0.91**
					131	130	17	**1.88**
**1493**	SRSF10 O75494$_{1-6}$: Serine/arginine-rich splicing factor 10	6/6	1	1	127	126	1	**2.30**
					129	128	1	**2.32**
					131	130	1	**1.15**
**1494**	SRSF3 P84103$_1$: Serine/arginine-rich splicing factor 3	1/2	5	39	127	126	34	**2.37**
					129	128	26	**1.52**
					131	130	30	**3.60**
**1495**	SRSF4 Q08170, SRSF6 Q13247$_{1,3}$: Serine/arginine-rich splicing factor 4, Serine/arginine-rich splicing factor 6	3/4	2	3	127	126	3	**1.13**
					129	128	3	**1.63**
					131	130	3	**2.34**
**1496**	SRSF9 Q13242: Serine/arginine-rich splicing factor 9		2	2	127	126	2	**0.54**
					129	128	2	**1.50**
					131	130	2	**1.85**
**1497**	SSH3 Q8TE77$_{1,4}$: Protein phosphatase Slingshot homolog 3	2/2	2	2	127	126	1	**2.15**
					129	128	1	**0.82**
					131	130	2	**0.56**
**1498**	ST13 P50502: Hsc70-interacting protein	1/2	6	9	127	126	1	**0.32**
					129	128	1	**1.55**
					131	130	1	**1.65**
**1499**	ST13P5 Q8NFI4: Putative protein FAM10A5		6	9	127	126	1	**0.45**
					129	128	1	**0.24**
					131	130	1	**2.74**
**1500**	STAB1 Q9NY15$_1$: Stabilin-1	1/2	23	34	127	126	23	**0.93**
					129	128	17	**0.22**
					131	130	29	**0.77**
**1501**	STAT3 P40763$_{1-3}$: Signal transducer and activator of transcription 3	3/3	9	9	127	126	9	**1.21**
					129	128	7	**1.14**
					131	130	6	**0.20**
**1502**	STAT5A P42229$_{1,2}$, STAT5B P51692: Signal transducer and activator of transcription 5A, Signal transducer and activator of transcription 5B	3/3	4	4	127	126	3	**1.56**
					129	128	2	**1.19**
					131	130	3	**0.20**
**1503**	STAT6 P42226$_{1,3}$: Signal transducer and activator of transcription 6	2/3	3	3	127	126	3	**1.49**
					129	128	3	**0.93**
					131	130	3	**0.31**

#	protein	group	peptides	spectra	ch1	ch2	quant	ratio
**1504**	STIP1 P31948$_{1-3}$: Stress-induced-phosphoprotein 1	3/3	3	3	127	126	3	**0.71**
					129	128	3	**0.46**
					131	130	3	**1.66**
**1505**	STK26 Q9P289$_{1,3}$: Serine/threonine-protein kinase 26	2/3	2	2	127	126	2	**0.54**
					129	128	2	**2.33**
					131	130	2	**0.99**
**1506**	STOML2 Q9UJZ1$_{1,2}$: Stomatin-like protein 2, mitochondrial	2/2	3	5	127	126	5	**0.55**
					129	128	5	**0.73**
					131	130	5	**3.32**
**1507**	STRAP Q9Y3F4$_{1,2}$: Serine-threonine kinase receptor-associated protein	2/2	2	2	127	126	2	**0.58**
					129	128	1	**2.40**
					131	130	2	**2.01**
**1508**	STXBP2 Q15833$_{1-3}$: Syntaxin-binding protein 2	3/3	4	4	127	126	4	**2.14**
					129	128	4	**0.90**
					131	130	4	**0.31**
**1509**	SUB1 P53999: Activated RNA polymerase II transcriptional coactivator p15		3	3	127	126	3	**1.00**
					129	128	3	**1.82**
					131	130	3	**1.68**
**1510**	SULT1A1 P50225$_{1}$: Sulfotransferase 1A1	1/3	9	13	127	126	4	**0.86**
					129	128	3	**1.29**
					131	130	5	**0.22**
**1511**	SULT1A3 P0DMM9$_{1,3}$, SULT1A4 P0DMN0: Sulfotransferase 1A3, Sulfotransferase 1A4	3/4	9	15	127	126	7	**1.41**
					129	128	7	**1.32**
					131	130	7	**0.59**
**1512**	SUN2 Q9UH99$_{1-3}$: SUN domain-containing protein 2	3/3	2	2	127	126	2	**0.76**
					129	128	2	**1.82**
					131	130	2	**1.27**
**1513**	SUPT6H Q7KZ85$_{1}$: Transcription elongation factor SPT6	1/2	6	19	127	126	3	**0.49**
					129	128	3	**2.09**
					131	130	4	**0.99**
**1514**	SUSD5 O60279: Sushi domain-containing protein 5		4	8	127	126	7	**0.67**
					129	128	8	**0.58**
					131	130	8	**1.88**
**1515**	SVEP1 Q4LDE5$_{1}$: Sushi, von Willebrand factor type A, EGF and pentraxin domain-containing pro ...	1/4	52	78	127	126	66	**0.58**
					129	128	58	**0.74**
					131	130	75	**0.86**

#	protein	group	peptides	spectra	ch1	ch2	quant	ratio
**1516**	SWAP70 Q9UH65: Switch-associated protein 70		2	2	127	126	2	**2.29**
					129	128	2	**0.56**
					131	130	2	**0.68**
**1517**	SYNCRIP O60506$_{3}$: Heterogeneous nuclear ribonucleoprotein Q	1/5	11	15	127	126	4	**0.58**
					129	128	3	**3.35**
					131	130	5	**1.51**
**1518**	TAF15 Q92804$_{1,2}$: TATA-binding protein-associated factor 2N	2/2	5	6	127	126	3	**1.04**
					129	128	3	**1.51**
					131	130	3	**1.55**
**1519**	TAGLN Q01995: Transgelin		10	14	127	126	14	**1.07**
					129	128	12	**0.43**
					131	130	14	**0.34**
**1520**	TAGLN2 P37802$_{1,2}$: Transgelin-2	2/2	11	18	127	126	17	**0.71**
					129	128	14	**0.84**
					131	130	18	**0.67**
**1521**	TARDBP Q13148$_{1}$: TAR DNA-binding protein 43		3	5	127	126	2	**1.26**
					129	128	2	**0.37**
					131	130	5	**1.22**
**1522**	TBC1D2B Q9UPU7$_{1-3}$: TBC1 domain family member 2B	3/3	1	1	127	126	1	**2.55**
					129	128	1	**1.81**
					131	130	1	**0.78**
**1523**	TBCB Q99426$_{1,2}$: Tubulin-folding cofactor B	2/2	2	2	127	126	2	**2.47**
					129	128	2	**0.17**
					131	130	2	**0.91**
**1524**	TBCD Q9BTW9$_{1,4}$: Tubulin-specific chaperone D	2/2	1	1	127	126	1	**2.60**
					129	128	1	**0.30**
					131	130	1	**0.68**
**1525**	TCN2 P20062$_{1}$: Transcobalamin-2	1/2	7	10	127	126	10	**1.10**
					129	128	6	**0.77**
					131	130	9	**0.39**
**1526**	TCP1 P17987: T-complex protein 1 subunit alpha		8	13	127	126	13	**1.25**
					129	128	11	**0.85**
					131	130	13	**0.59**
**1527**	TF P02787: Serotransferrin		32	53	127	126	45	**0.78**
					129	128	36	**2.00**
					131	130	49	**1.21**

#	protein	group	peptides	spectra	ch1	ch2	quant	ratio
**1528**	TFF1 P04155: Trefoil factor 1		1	1	127	126	1	**1.02**
					129	128	1	**0.33**
					131	130	1	**1.62**
**1529**	TFG Q92734$_{1-4}$: Protein TFG	4/4	5	5	127	126	4	**0.49**
					129	128	3	**2.67**
					131	130	3	**1.21**
**1530**	TFPI2 P48307$_{1,2}$: Tissue factor pathway inhibitor 2	2/2	2	2	127	126	2	**0.60**
					129	128	2	**0.71**
					131	130	2	**1.98**
**1531**	TGFB2 P61812$_{1,2}$: Transforming growth factor beta-2	2/2	2	3	127	126	3	**0.62**
					129	128	2	**1.42**
					131	130	3	**0.46**
**1532**	TGFBI Q15582: Transforming growth factor-beta-induced protein ig-h3		28	49	127	126	43	**0.71**
					129	128	39	**2.48**
					131	130	47	**0.51**
**1533**	TGFBR3 Q03167$_{1,2}$: Transforming growth factor beta receptor type 3	2/2	9	15	127	126	15	**1.12**
					129	128	11	**1.54**
					131	130	15	**0.48**
**1534**	TGM1 P22735$_{1}$: Protein-glutamine gamma-glutamyltransferase K	1/2	9	9	127	126	8	**3.89**
					129	128	8	**0.32**
					131	130	8	**0.54**
**1535**	TGM2 P21980$_{1}$: Protein-glutamine gamma-glutamyltransferase 2	1/2	14	22	127	126	21	**1.27**
					129	128	19	**1.33**
					131	130	22	**1.31**
**1536**	TGM3 Q08188: Protein-glutamine gamma-glutamyltransferase E		3	3	127	126	3	**8.52**
					129	128	3	**0.09**
					131	130	3	**1.46**
**1537**	THAP4 Q8WY91$_{1}$: THAP domain-containing protein 4		2	2	127	126	2	**0.75**
					129	128	1	**0.72**
					131	130	1	**0.84**
**1538**	THBS1 P07996$_{1}$: Thrombospondin-1	1/2	29	54	127	126	41	**0.48**
					129	128	29	**1.81**
					131	130	48	**0.78**
**1539**	THBS2 P35442: Thrombospondin-2		25	35	127	126	28	**0.24**
					129	128	25	**0.78**
					131	130	34	**1.88**

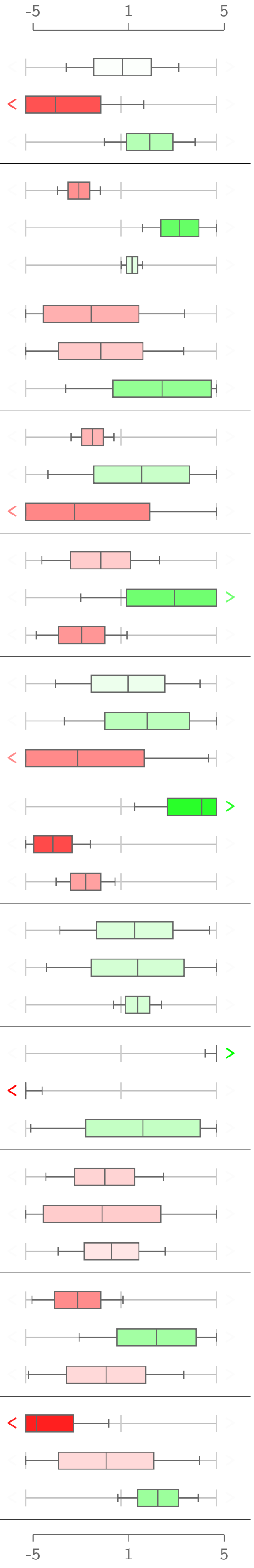

#	protein	group	peptides	spectra	ch1	ch2	quant	ratio
**1540**	THBS3 P49746$_1$: Thrombospondin-3	1/2	8	9	127	126	9	**1.07**
					129	128	8	**1.23**
					131	130	8	**0.78**
**1541**	THBS4 P35443: Thrombospondin-4		21	90	127	126	61	**1.82**
					129	128	35	**0.89**
					131	130	77	**0.82**
**1542**	THRAP3 Q9Y2W1: Thyroid hormone receptor-associated protein 3		2	2	127	126	1	**1.29**
					129	128	1	**1.15**
					131	130	1	**1.55**
**1543**	THSD4 Q6ZMP0$_1$: Thrombospondin type-1 domain-containing protein 4	1/2	8	17	127	126	11	**1.00**
					129	128	7	**0.68**
					131	130	16	**1.62**
**1544**	TIAL1 Q01085$_{1,2}$: Nucleolysin TIAR	2/2	3	5	127	126	3	**1.51**
					129	128	4	**0.63**
					131	130	4	**0.67**
**1545**	TIMP1 P01033: Metalloproteinase inhibitor 1		3	3	127	126	3	**0.42**
					129	128	3	**1.28**
					131	130	3	**2.12**
**1546**	TIMP2 P16035: Metalloproteinase inhibitor 2		8	20	127	126	17	**0.70**
					129	128	13	**0.43**
					131	130	18	**1.23**
**1547**	TIMP3 P35625: Metalloproteinase inhibitor 3		4	4	127	126	3	**0.77**
					129	128	3	**0.61**
					131	130	4	**1.11**
**1548**	TIMP4 Q99727: Metalloproteinase inhibitor 4		2	2	127	126	2	**0.60**
					129	128	1	**7.41**
					131	130	2	**2.79**
**1549**	TINAGL1 Q9GZM7$_1$: Tubulointerstitial nephritis antigen-like	1/2	4	8	127	126	4	**0.46**
					129	128	6	**0.36**
					131	130	6	**2.35**
**1550**	TIPRL O75663$_1$: TIP41-like protein	1/2	3	4	127	126	3	**1.31**
					129	128	4	**1.09**
					131	130	4	**0.29**
**1551**	TJP1 Q07157$_{1,2}$: Tight junction protein ZO-1	2/2	2	2	127	126	2	**1.14**
					129	128	2	**0.57**
					131	130	2	**0.74**

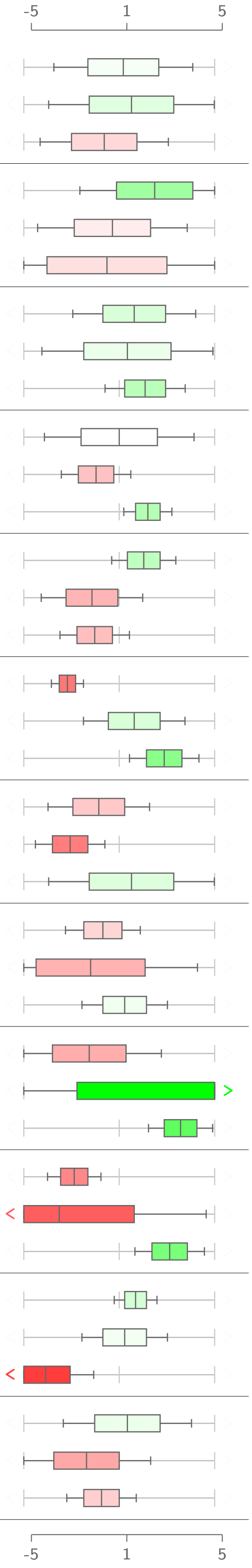

#	protein	group	peptides	spectra	ch1	ch2	quant	ratio
**1552**	TKFC Q3LXA3$_{1,2}$: Triokinase/FMN cyclase	2/2	3	4	127	126	2	**1.29**
					129	128		
					131	130	3	**0.42**
**1553**	TLN1 Q9Y490: Talin-1		87	120	127	126	88	**1.79**
					129	128	87	**0.57**
					131	130	97	**0.32**
**1554**	TLN2 Q9Y4G6: Talin-2		23	27	127	126	8	**1.51**
					129	128	6	**0.15**
					131	130	8	**0.60**
**1555**	TMED10 P49755: Transmembrane emp24 domain-containing protein 10		6	6	127	126	6	**3.60**
					129	128	4	**1.32**
					131	130	6	**0.37**
**1556**	TMED2 Q15363: Transmembrane emp24 domain-containing protein 2		2	2	127	126	2	**1.07**
					129	128	2	**1.26**
					131	130	2	**0.53**
**1557**	TMED9 Q9BVK6: Transmembrane emp24 domain-containing protein 9		4	6	127	126	6	**4.10**
					129	128	4	**0.43**
					131	130	6	**0.52**
**1558**	TMEM43 Q9BTV4: Transmembrane protein 43		3	3	127	126	3	**2.83**
					129	128	3	**1.01**
					131	130	3	**0.56**
**1559**	TMOD3 Q9NYL9: Tropomodulin-3		3	3	127	126	3	**0.62**
					129	128	3	**1.43**
					131	130	3	**1.41**
**1560**	TMSB4X P62328: Thymosin beta-4		2	2	127	126	2	**1.96**
					129	128	2	**0.49**
					131	130	2	**0.42**
**1561**	TNC P24821$_{1,4}$: Tenascin	2/6	23	34	127	126	27	**0.65**
					129	128	22	**2.21**
					131	130	32	**1.38**
**1562**	TNFAIP2 Q03169: Tumor necrosis factor alpha-induced protein 2		2	2	127	126	2	**2.45**
					129	128	1	**0.31**
					131	130	2	**0.30**
**1563**	TNFRSF11B O00300: Tumor necrosis factor receptor superfamily member 11B		4	4	127	126	4	**1.72**
					129	128	4	**0.64**
					131	130	4	**1.34**

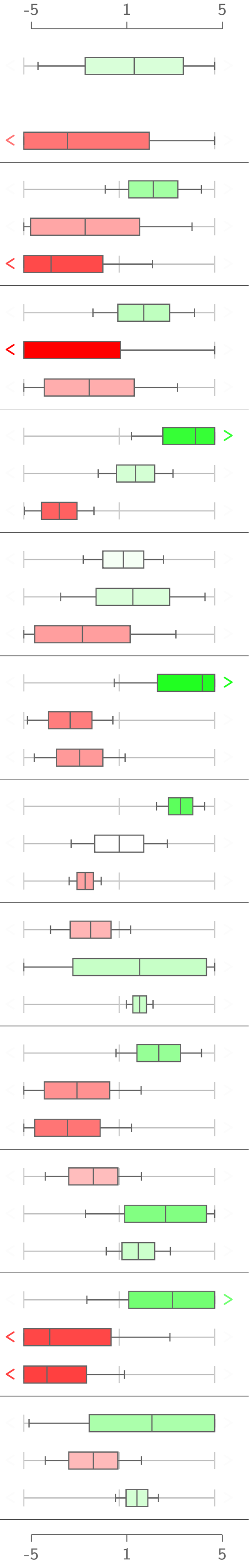

#	protein	group	peptides	spectra	ch1	ch2	quant	ratio
**1564**	TNFRSF6B O95407: Tumor necrosis factor receptor superfamily member 6B		1	1	127	126	1	**1.76**
					129	128	1	**0.09**
					131	130	1	**1.22**
**1565**	TNFSF12 O43508$_{2}$, TNFSF13 O75888$_{1-3}$: Tumor necrosis factor ligand superfamily member 12, Tumor necrosis factor ligand superfamily member 13	4/6	2	3	127	126	1	**0.71**
					129	128	1	**0.42**
					131	130	1	**1.10**
**1566**	TNKS1BP1 Q9C0C2$_{1}$: 182 kDa tankyrase-1-binding protein	1/2	8	9	127	126	7	**1.29**
					129	128	8	**0.25**
					131	130	8	**0.91**
**1567**	TNNT1 P13805$_{1-3}$: Troponin T, slow skeletal muscle	3/3	3	3	127	126	2	**0.88**
					129	128	2	**0.08**
					131	130	2	**3.44**
**1568**	TNPO1 Q92973$_{1,2}$: Transportin-1	2/3	9	10	127	126	5	**0.69**
					129	128	2	**1.71**
					131	130	3	**0.77**
**1569**	TNPO2 O14787$_{2}$: Transportin-2	1/2	6	6	127	126	1	**2.45**
					129	128	1	**1.06**
					131	130	1	**0.28**
**1570**	TNXB P22105$_{1,3,4}$: Tenascin-X	3/5	56	77	127	126	72	**1.00**
					129	128	61	**0.95**
					131	130	72	**1.72**
**1571**	TOR1AIP1 Q5JTV8$_{1,3}$: Torsin-1A-interacting protein 1	2/2	3	3	127	126	3	**2.06**
					129	128	3	**0.50**
					131	130	3	**1.19**
**1573**	TPM1 P09493$_{5}$: Tropomyosin alpha-1 chain		15	25	127	126	1	**2.16**
					129	128	2	**0.23**
					131	130	2	**1.75**
**1577**	TPM2 P07951$_{1}$: Tropomyosin beta chain		17	30	127	126	1	**0.22**
					129	128	1	**0.10**
					131	130	1	**3.40**
**1578**	TPM2 P07951$_{2}$: Tropomyosin beta chain	1/2	20	32	127	126	7	**0.67**
					129	128	6	**0.40**
					131	130	6	**2.25**
**1580**	TPM3 P06753$_{2}$: Tropomyosin alpha-3 chain	1/3	19	33	127	126	2	**4.08**
					129	128	2	**0.11**
					131	130	2	**2.43**

#	protein	group	peptides	spectra	ch1	ch2	quant	ratio
**1582**	TPM4 P67936$_1$: Tropomyosin alpha-4 chain		19	36	127	126	7	**0.89**
					129	128	6	**0.17**
					131	130	6	**2.89**
**1584**	TPP1 O14773$_{1,2}$: Tripeptidyl-peptidase 1	2/2	3	3	127	126	3	**1.13**
					129	128	3	**0.85**
					131	130	3	**1.37**
**1585**	TRAPPC3 O43617$_1$: Trafficking protein particle complex subunit 3	1/2	4	4	127	126	3	**1.10**
					129	128	3	**0.65**
					131	130	3	**0.66**
**1586**	TRIM28 Q13263$_1$: Transcription intermediary factor 1-beta	1/2	7	7	127	126	7	**1.05**
					129	128	7	**1.14**
					131	130	7	**0.65**
**1587**	TRIM42 Q8IWZ5$_1$: Tripartite motif-containing protein 42		3	80	127	126	1	**2.18**
					129	128		
					131	130	10	**1.36**
**1588**	TRIP11 Q15643: Thyroid receptor-interacting protein 11		2	2	127	126	1	**1.12**
					129	128	1	**0.08**
					131	130	1	**0.43**
**1589**	TRIP12 Q14669$_{1-4}$: E3 ubiquitin-protein ligase TRIP12	4/4	3	3	127	126	2	**2.80**
					129	128	2	**0.55**
					131	130	2	**0.40**
**1590**	TROVE2 P10155$_{1,3-5}$: 60 kDa SS-A/Ro ribonucleoprotein	4/5	4	4	127	126	4	**0.59**
					129	128	3	**1.84**
					131	130	3	**1.19**
**1591**	TSKU Q8WUA8: Tsukushin		10	22	127	126	18	**0.98**
					129	128	16	**0.88**
					131	130	20	**0.54**
**1592**	TST Q16762: Thiosulfate sulfurtransferase		2	2	127	126	2	**5.28**
					129	128	2	**0.47**
					131	130	2	**0.74**
**1593**	TSTA3 Q13630: GDP-L-fucose synthase		2	22	127	126	14	**0.92**
					129	128	9	**0.26**
					131	130	21	**0.86**
**1594**	TTN Q8WZ42$_{12}$: Titin	1/9	27	34	127	126	18	**1.55**
					129	128	12	**0.44**
					131	130	19	**0.66**

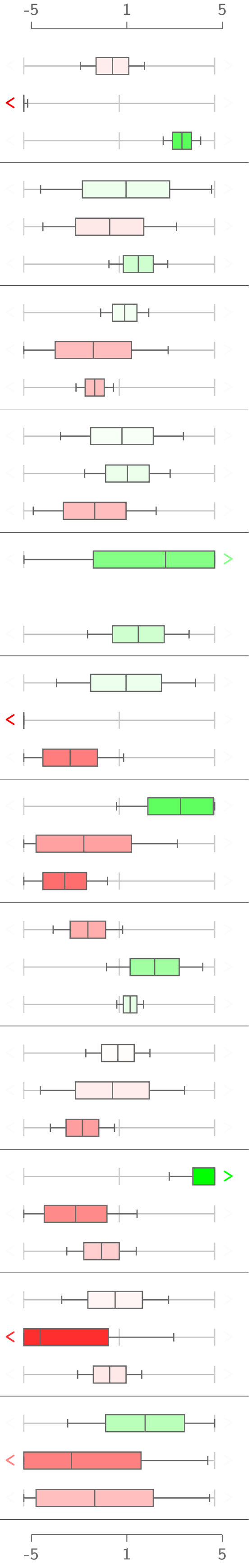

#	protein	group	peptides	spectra	ch1	ch2	quant	ratio
**1595**	TTR P02766: Transthyretin		7	101	127	126	97	**0.87**
					129	128	92	**0.44**
					131	130	97	**1.44**
**1599**	TUBA3E Q6PEY2: Tubulin alpha-3E chain		11	34	127	126	1	**2.50**
					129	128		
					131	130	1	**0.80**
**1600**	TUBA4A $P68366_{1,2}$: Tubulin alpha-4A chain	2/2	14	63	127	126	1	**0.85**
					129	128	1	**2.95**
					131	130	1	**1.10**
**1601**	TUBAL3 $A6NHL2_{1,2}$: Tubulin alpha chain-like 3	2/2	5	28	127	126	2	**1.19**
					129	128	2	**1.48**
					131	130	3	**0.98**
**1602**	TUBB P07437: Tubulin beta chain	1/3	16	67	127	126	4	**0.43**
					129	128	4	**1.72**
					131	130	5	**0.85**
**1603**	TUBB2A Q13885, TUBB2B Q9BVA1: Tubulin beta-2A chain, Tubulin beta-2B chain	2/2	14	60	127	126	1	**1.16**
					129	128	1	**0.45**
					131	130	1	**1.26**
**1606**	TUBB4B P68371: Tubulin beta-4B chain	1/3	16	67	127	126	1	**0.43**
					129	128	1	**25.88**
					131	130	1	**1.27**
**1607**	TUBB6 Q9BUF5: Tubulin beta-6 chain		8	21	127	126	2	**1.58**
					129	128	1	**2.35**
					131	130	2	**1.36**
**1608**	TUFM P49411: Elongation factor Tu, mitochondrial		9	10	127	126	9	**1.91**
					129	128	8	**0.69**
					131	130	7	**0.56**
**1609**	TWF1 $Q12792_{2-4}$: Twinfilin-1	3/3	4	4	127	126	3	**1.23**
					129	128	3	**1.37**
					131	130	3	**0.18**
**1610**	TWSG1 $Q9GZX9_{1}$: Twisted gastrulation protein homolog 1	1/2	7	9	127	126	2	**0.57**
					129	128	2	**0.59**
					131	130	3	**1.74**
**1611**	TXNDC5 $Q8NBS9_{1}$: Thioredoxin domain-containing protein 5	1/2	5	7	127	126	5	**0.79**
					129	128	4	**1.25**
					131	130	4	**1.08**

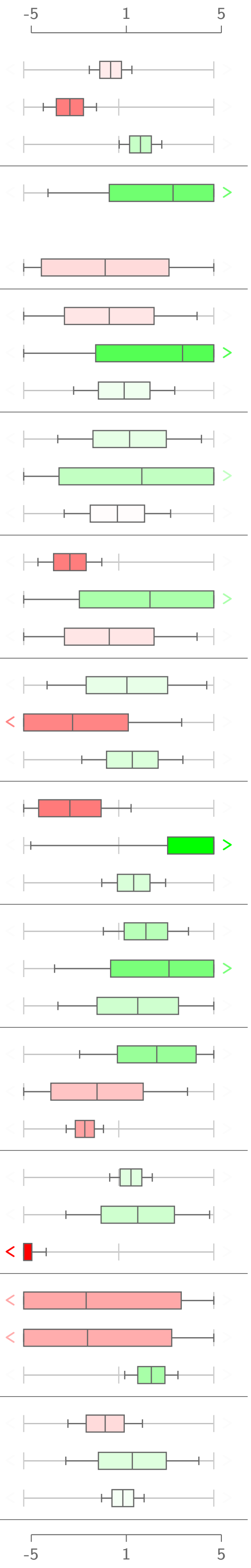

#	protein	group	peptides	spectra	ch1	ch2	quant	ratio
**1612**	TXNL1 O43396: Thioredoxin-like protein 1		2	2	127	126	2	**1.18**
					129	128	2	**0.58**
					131	130	2	**0.84**
**1613**	TYMP P19971$_{1,2}$: Thymidine phosphorylase	2/2	6	6	127	126	6	**0.93**
					129	128	6	**1.84**
					131	130	5	**0.36**
**1614**	U2AF1 Q01081$_{4}$: Splicing factor U2AF 35 kDa subunit	1/4	3	5	127	126	2	**1.54**
					129	128	2	**0.67**
					131	130	5	**2.08**
**1615**	UBA1 P22314$_{1,2}$: Ubiquitin-like modifier-activating enzyme 1	2/2	25	35	127	126	32	**0.92**
					129	128	25	**0.50**
					131	130	32	**0.37**
**1616**	UBA2 Q9UBT2$_{1,2}$: SUMO-activating enzyme subunit 2	2/2	3	3	127	126	3	**1.65**
					129	128	3	**0.27**
					131	130	3	**0.38**
**1617**	UBA3 Q8TBC4$_{1,2}$: NEDD8-activating enzyme E1 catalytic subunit	2/2	2	2	127	126	2	**0.63**
					129	128	2	**2.08**
					131	130	2	**0.64**
**1618**	UBA6 A0AVT1$_{1}$: Ubiquitin-like modifier-activating enzyme 6	1/2	4	4	127	126	4	**0.72**
					129	128	3	**0.93**
					131	130	4	**0.94**
**1619**	UBA7 P41226: Ubiquitin-like modifier-activating enzyme 7		3	3	127	126	3	**1.57**
					129	128	3	**0.18**
					131	130	3	**0.67**
**1620**	UBE2F Q969M7$_{1,2,4-6}$: NEDD8-conjugating enzyme UBE2F	5/6	2	2	127	126	2	**1.20**
					129	128	2	**0.59**
					131	130	2	**0.61**
**1621**	UBE2K P61086$_{1,3}$: Ubiquitin-conjugating enzyme E2 K	2/2	2	3	127	126	3	**2.06**
					129	128	2	**0.37**
					131	130	3	**0.49**
**1622**	UBE2M P61081: NEDD8-conjugating enzyme Ubc12		4	5	127	126	5	**3.64**
					129	128	4	**0.48**
					131	130	4	**0.34**
[truncated: 779,167 more chars]
